# Vaccinia viral A26 protein is a fusion suppressor of mature virus and triggers membrane fusion through conformational change at low pH

**DOI:** 10.1371/journal.ppat.1007826

**Published:** 2019-06-20

**Authors:** Hung-Wei Chang, Cheng-Han Yang, Yu-Chun Luo, Bo-Gang Su, Huei-Yin Cheng, Shu-Yun Tung, Kathleen Joyce D. Carillo, Yi-Ting Liao, Der-Lii M. Tzou, Hao-Ching Wang, Wen Chang

**Affiliations:** 1 Institute of Molecular Biology, Academia Sinica, Taipei, Taiwan; 2 Sustainable Chemical Science and Technology Program, Taiwan International Graduate Program, Academia Sinica, Taipei, Taiwan; 3 Institute of Chemistry, Academia Sinica, Taipei, Taiwan; 4 Department of Applied Chemistry, National Chiao Tung University, Hsinchu, Taiwan; 5 The Ph.D. Program for Translational Medicine, College of Medical Science and Technology, Taipei Medical University and Academia Sinica, Taipei, Taiwan; 6 Graduate Institute of Translational Medicine, College of Medical Science and Technology, Taipei Medical University, Taiwan; 7 Department of Applied Chemistry, National Chia-Yi University, Chia-Yi, Taiwan; University of Florida, UNITED STATES

## Abstract

Vaccinia mature virus requires A26 envelope protein to mediate acid-dependent endocytosis into HeLa cells in which we hypothesized that A26 protein functions as an acid-sensitive membrane fusion suppressor. Here, we provide evidence showing that N-terminal domain (aa1-75) of A26 protein is an acid-sensitive region that regulates membrane fusion. Crystal structure of A26 protein revealed that His48 and His53 are in close contact with Lys47, Arg57, His314 and Arg312, suggesting that at low pH these His-cation pairs could initiate conformational changes through protonation of His48 and His53 and subsequent electrostatic repulsion. All the A26 mutant mature viruses that interrupted His-cation pair interactions of His48 and His 53 indeed have lost virion infectivity. Isolation of revertant viruses revealed that second site mutations caused frame shifts and premature termination of A26 protein such that reverent viruses regained cell entry through plasma membrane fusion. Together, we conclude that viral A26 protein functions as an acid-sensitive fusion suppressor during vaccinia mature virus endocytosis.

## Author summary

Vaccinia virus is a complex large DNA virus with a large number of viral membrane proteins to facilitate cell entry. Although it is well established that vaccinia mature virus uses endocytosis to enter cells, it remains unclear how it triggers membrane fusion in the acidic environment of endosomes. Recently, we hypothesized that A26 protein in vaccinia mature virus functions as an acid-sensitive membrane fusion suppressor, which suggests a novel viral regulation not present in other enveloped viruses. We postulated that conformational changes of A26 protein at low pH result in de-repression of viral fusion complex activity to trigger viral and endosomal membrane fusion. Here, we provide structural, biochemical and biological evidence demonstrating that vaccinia A26 protein does indeed function as an acid-sensitive fusion suppressor protein to regulate vaccinia mature virus membrane fusion during endocytosis. Our data reveal an important and unique “checkpoint” for vaccinia mature virus endocytosis that has not been described for other viruses. Furthermore, by isolating adaptive vaccinia mutants that escaped endocytic blockage, we discovered that mutations within the *A26L* gene serve as an effective strategy for switching the viral infection route from endocytosis to plasma membrane fusion, expanding viral host range.

## Introduction

Virus entry represents the initial stage of infection and is a target for developing new antiviral therapeutics. Poxvirus is a family of enveloped DNA viruses with genomes of ~200 kilobases [1]. Vaccinia virus, an orthopoxvirus, is a model system for investigating poxvirus entry into host cells, producing mature (MV) and extracellular virus (EV) [2–4].

Vaccinia MV attaches to cell surface glycosaminoglycans and extracellular matrix laminin [5–10]. It then clusters at lipid rafts, triggering the integrin β1-CD98-PI3K signaling cascade [11, 12] to induce actin-dependent endocytosis that may [13] or may not involve apoptotic mimicry [14–16]. After internalization, vaccinia MV is trafficked in vesicles inside the cells, with subsequent endosomal acidification triggering viral membrane fusion with the vesicular membrane to release viral cores into the cytoplasm [17–20].

How vaccinia virus triggers fusion with host cells remains unclear. Many enveloped viruses contain a viral fusion protein that induces conformational changes at low pH [21–23]. Conformational change of viral fusion proteins exposes a hydrophobic terminal fusion peptide [24, 25] or internal fusion loop [26, 27] that can be inserted into host membranes. Subsequent conformational changes and oligomerization of viral fusion proteins then triggers fusion of viral and host membranes. Vaccinia MV employs a highly conserved eleven-component fusion protein complex to mediate virus fusion with cells [3, 28], but how it functions remains unknown [4].

**Fig 1:**
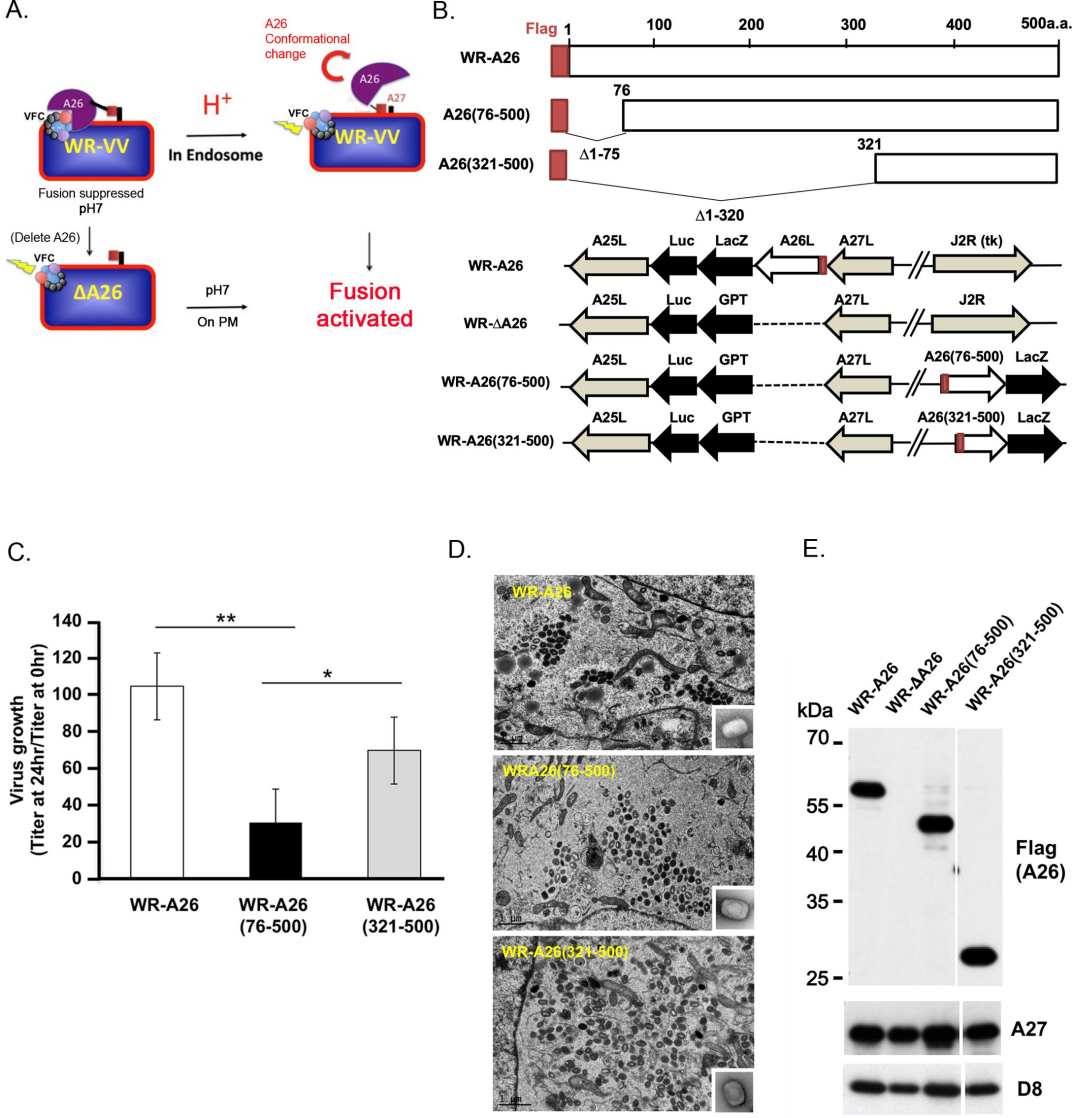
Model of A26 protein function and generation of mutant vaccinia viruses containing N-terminal deletions of A26 protein. **(A)**. Current model of how A26 protein functions as an acid-sensitive fusion suppressor of the WR strain of vaccinia virus MV. Vaccinia MV contains A26 protein that binds to the A16 and G9 components of the viral fusion complex (VFC) to suppress virus membrane fusion activity, which keeps the virus stable at neutral pH. When MV is internalized into endosomes, acidification triggers conformational changes of A26 protein that dissociates itself from the VFC, resulting in activation of the VFC and fusion of the viral and vesicular membranes. However, deletion of the *A26L* gene results in WR-ΔA26 MV particles lacking fusion suppressor activity, so these MV particles readily fuse to the plasma membrane (PM) at neutral pH. **(B).** Schematics of full-length WR-A26 and truncated WR-A26 proteins (aa 76–500 or 321–500), as well as each construct containing flag-tag sequences (red) at their N-termini. Locations of *A26L* mutant genes in the corresponding viral genomes are also shown. WR-A26 and WR-ΔA26 were described previously [32, 33], and the latter was used as the parental virus to generate the WR-A26(76–500) and WR-A26(321–500) recombinant viruses. The *J2R* locus encodes a non-essential viral thymidine kinase (*tk*). We inserted A26(76–500) and A26(321–500) gene constructs into the *tk* locus and then selected blue plaques in agar plates containing X-gal. **(C).** One-step growth of mutant vaccinia viruses in HeLa cells. Cells were infected with each virus at a multiplicity of infection (MOI) of 5 PFU per cell and cells were washed and harvested at 0 and 24 hpi for virus titer determination. The Y-axis represents MV growth, determined by dividing virus titers at 24 hpi with the respective input virus titer at 0 hpi. We performed three experimental repeats for each virus and used the Student *t*-test for statistical analyses. *p <0.05, ** p < 0.01. **(D).** EM images of MV in infected HeLa cells at 24 hpi. Insets in lower right corner represent EM images of CsCl-purified MV. The scale bar represents 1 μm. **(E).** Immunoblots of N-terminal A26-deletion proteins in CsCl-purified MV particles. A27 and D8 are two viral envelope proteins that served as positive controls.

Vaccinia MV exhibits broad infectivity, acting via endocytosis or plasma membrane fusion [9, 29, 30], depending on vaccinia virus strains and cell types [17, 31]. We previously demonstrated that the WR strain of vaccinia MV uses viral A26 protein for the endocytosis pathway, whereas deletion of A26 protein induced the plasma membrane fusion pathway in HeLa cells [32, 33]. However, loss of A26 protein renders viral MV particles resistant to bafilomycin (BFLA) without loss of fusion activity, suggesting that A26 protein is the target of acid regulation, not the viral fusion complex [32]. The A26 protein binds to A16 and G9 proteins of the viral entry fusion complex at neutral pH and, when purified MV was treated with acidic buffer, the A26-A27 protein complex dissociated from MVs at low pH [33], inspiring our model wherein A26 protein is an acid-sensitive fusion suppressor of MV (Fig 1A). In this model, A26 protein binds to viral fusion complex to suppress MV fusion at neutral pH. However, the acidic pH of endosomes triggers conformational changes in A26 protein, which is subsequently released from the viral fusion protein complex, resulting in viral and vesicular membrane fusion. In the absence of A26 protein, viral fusion protein complex becomes fusion-competent at neutral pH, triggering efficient fusion with the plasma membrane, consistent with electron microscopy (EM) data [32, 33]. Here, we provide genetic, biochemical and structural evidence that A26 protein is a fusion suppressor that regulates vaccinia MV membrane fusion through acid-dependent conformational changes.

## Results

### The N-terminal region of A26 protein is important for vaccinia MV infectivity

We generated two N-terminal deletion constructs of the A26 open-reading frame (ORF), in which we removed amino acids (aa) 1–75 or aa 1–320 of A26 protein (Fig 1B). Each deletion construct was fused in-frame with N-terminal flag sequences and inserted into a non-essential thymidine kinase (tk) locus of the WR-ΔA26 virus, which deleted the A26 ORF (Fig 1B). We did not generate any C-terminal deletions of the A26 ORF because this region is required for A26 protein binding to viral A27 protein and subsequent packaging into MV particles [34, 35]. Recombinant viruses expressing WR-A26(76–500) or WR-A26(321–500) were isolated and plaque-purified. A recombinant vaccinia virus expressing full-length flag-tagged A26 protein, named WR-A26, was also included [36]. HeLa cells were infected with individual virus at a multiplicity of infection (MOI) of 5 plaque-forming units (PFU) per cell and harvested at 24 hours post infection (hpi) to determine MV growth (Fig 1C). Control WR-A26 grew approximately 100-fold at 24 hpi. WR-A26(76–500) exhibited significantly reduced MV yield in the same timeframe, whereas WR-A26(321–500) yield was similar to that of control WR-A26 virus. None of these three viruses exhibited defective virus assembly, with each presenting large amounts of MV in cytoplasm of infected cells at 24 hpi (Fig 1D), consistent with previous results demonstrating that A26 protein is not required for MV assembly [10]. We purified MV particles via CsCl gradient purification and found that WR-A26, WR-A26(76–500) and WR-A26(321–500) presented similar morphologies under EM (enlarged MV images in the insets of Fig 1D). Immunoblot analyses of purified MV also revealed comparable A26 protein levels in MV particles of WR-A26, WR-A26(76–500) and WR-A26(321–500) (Fig 1E). We assessed whether the reduced growth of WR-A26(76–500) virus reflects low MV particle infectivity by counting how many virus particles are required to initiate a single infection event in HeLa cells, i.e., to determine the particle-to-PFU ratio (Table 1). For the control WR-A26 MV particles, this ratio is ~43, whereas for WR-A26(76–500) it is ~88, demonstrating that removal of aa 1–75 of A26 protein significantly reduced MV infectivity. Surprisingly, further deletion of A26 protein, aa 1–320, recovered MV infectivity of WR-A26(321–500) to a ratio of ~33, i.e., not statistically different from control WR-A26 MV infectivity (an outcome we explain in the next section).

**Table 1:** MV particle infectivity of WR-A26(76–500), WR-A26(321–500), WR-A26-H2R, WR-A26-H3R and the revertant viruses.

MV Virus	Infectivity (Mean ±SD)
**WR-A26^a^**	**43 (±7)**
**WR-A26(76–500)**	88(±12)*
**WR-A26(321–500)**	**33(±16)**
**WR-A26-H2R**	254(±37)*
**WR-A26-H3R**	**112^§^**
**WR-A26-H2R-Rev1**	**47^§^**
**WR-A26-H3R-Rev1**	**43**^**§**^

^a^WR-A26 was used as a positive control in the experiments.

*Data were analyzed using a two-tailed unpaired student’s *t* test. *, P<0.05.

**^§^**Infectivity was determined once.

### The N-terminal region of A26 protein is essential for acid-sensitivity and the middle region is responsible for fusion suppression

**Fig 2:**
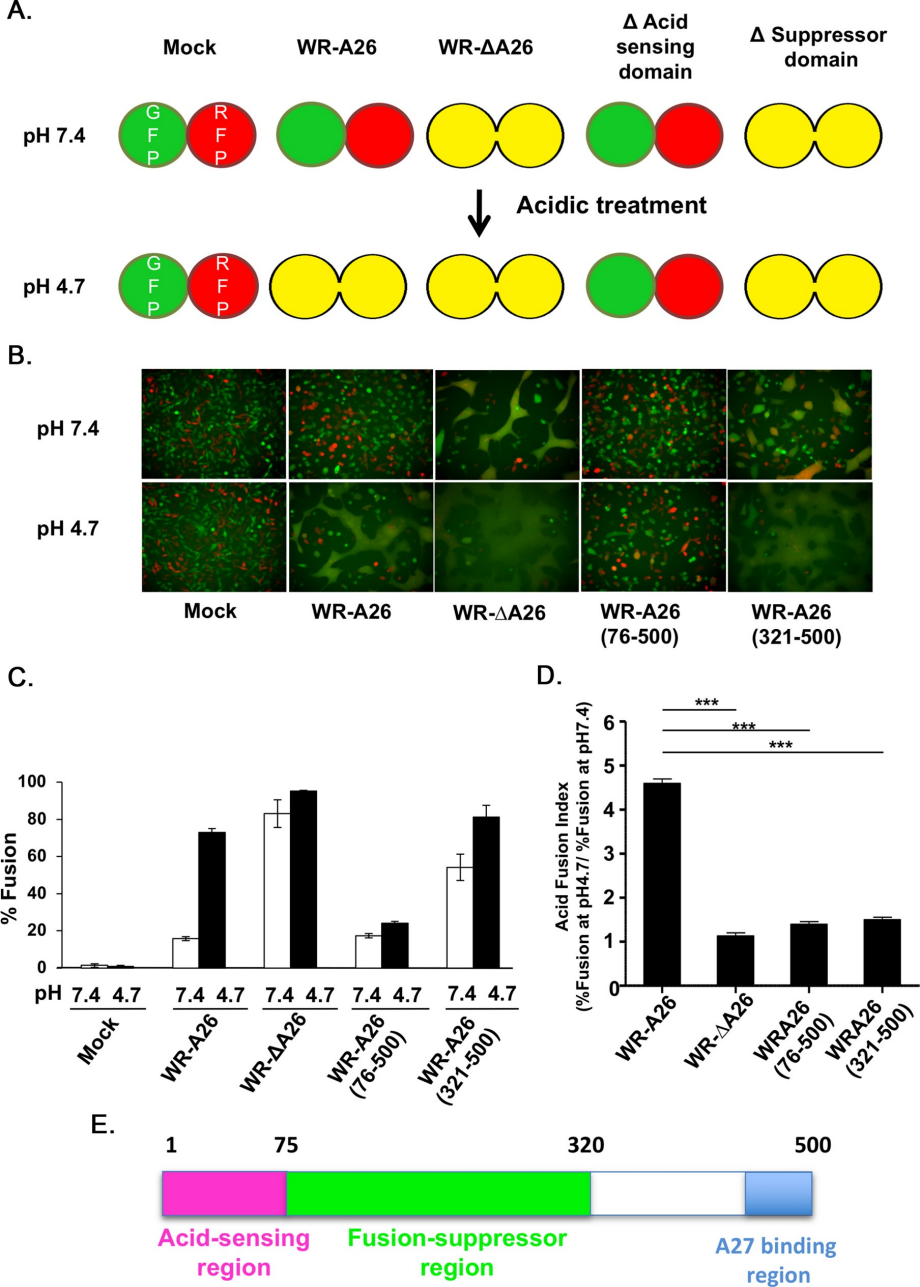
N-terminal region of A26 protein (aa 1–75) is important for endocytic-mediated MV membrane fusion at low pH. **(A).** Schematic presentation of vaccinia MV-dependent cell-cell fusion at neutral and acidic pH. L cells expressing GFP and RFP were mixed at a 1:1 ratio during seeding. No cell fusion occurs at either pH in the absence of infection (Mock). When cells were infected with the endocytic WR-A26 virus, cell-cell fusion only occurred at low pH (which mimics endosomal environments) and no cell-cell fusion occurred at neutral pH (which allows plasma membrane fusion). In contrast, WR-ΔA26 virus does not require acidic environments to activate membrane fusion, so cell-cell fusion occurs at both acidic and neutral pH. Truncation of A26 protein to remove the acid-sensing/acid-sensitive domain (WR-A26(76–500)) renders the protein a constitutive fusion suppressor, so no fusion occurred at either pH. Further truncation of A26 protein to remove the fusion suppressor domain (WR-A26(321–500)) resulted in a virus phenotype like that of WR-ΔA26 in that it fuses at both pH. **(B).** Images of cell-cell fusion induced by various vaccinia MV infections. Single fluorescent cells described in (A) were infected with each virus at an MOI of 50 PFU per cell, washed with neutral or acidic pH buffer for 3 min, and then subjected to live-imaging to monitor cell-cell fusion at 37°C for 2 h. **(C).** Quantification of % cell fusion induced by each virus at neutral or low pH conditions as described in (B). Five images for each virus were recorded and the % fusion was calculated using the image area of GFP^+^RFP^+^ double-fluorescent cells divided by that of single-fluorescent cells. The experiments were repeated three times for each virus and bars represent standard deviation. **(D).** An “Acid Fusion Index” was calculated to represent the acid-dependence of each A26 deletion protein, i.e., the occurrence of A26 protein conformational change. The index for each virus was obtained by dividing the % of cell fusion at low pH (black bar in C) by that recorded for neutral pH (white bar in C). Endocytic WR-A26 virus demonstrated an Acid-Fusion Index of ~4.6, whereas the value for all other viruses was ~1, meaning these latter exhibit limited dependence on acidic environments for cell fusion. The experiments were repeated three times for each virus and the Student *t*-test was used for statistical analyses. *** p <0.001. **(E).** Schematic representation of A26 protein functional regions. The N-terminal region of A26 protein (aa 1–75, in pink) is important for acid sensitivity, whereas the middle region (aa 76–320, in green) is required for the fusion suppressor function. The C-terminal region (in blue) was previously shown to mediate binding to viral A27 protein during MV assembly [34, 35].

Based on our model (Fig 1A), A26 protein contains an acid-sensing or acid-sensitive region responsible for inducing acid-dependent conformational changes, as well as a fusion suppressor region to suppress fusion activity at neutral pH. To establish if these region functions are absent in our WR-A26(76–500) and WR-A26(321–500) protein constructs, we adopted a cell-cell fusion assay to investigate virus-cell membrane fusion (Fig 2A, 2B and 2C). Mock-infected cells did not fuse at either neutral or acidic pH, so GFP- and RFP-expressing cells were well separated. Control WR-A26 virus needs a low pH endocytic environment to initiate fusion, so surface-bound MV did not induce cell-cell fusion at neutral pH. However, brief treatment of these surface-bound MV with low pH buffer created an acidic environment that mimicked endosomes, leading to conformational changes of the A26 protein that activated virus-mediated cell-cell fusion to produce double-fluorescent fused cells. In contrast, WR-ΔA26 MV lacked a fusion suppressor, so cell-cell fusion was triggered by virus infection at both neutral and acidic pH. Interestingly, WR-A26(76–500) did not fuse at either neutral or acidic pH, thus acting like a pH-independent fusion suppressor and indicating that aa 1–75 represents the acid-sensitive region of A26 protein. Finally, WR-A26(321–500) triggered robust membrane fusion at both neutral and acidic pH, i.e., similar to WR-ΔA26, suggesting that the fusion suppressor region is absent in WR-A26(321–500) and that aa 76–320 represents the fusion suppressor region of A26 protein.

Based on the fusion quantification data in Fig 2C, we divided the % fusion at low pH (4.7) by that at neutral pH (7.4) in order to obtain an “acid fusion index” that reflects the acid dependence of each MV construct (Fig 2D). Only endocytic WR-A26 had a high acid fusion index, whereas all other viruses lost their acid dependence with WR-ΔA26 and WR-A26(321–500) fusing well at both pH and WR-A26(76–500) fusing poorly at both pH. Therefore, we conclude that the N-terminal region of aa 1–75 of A26 protein is important for acid-sensing or acid sensitivity and the middle region of aa 76–320 is required for fusion suppression (Fig 2E). This conclusion fits well with the low infectivity of WR-A26(76–500) MV and the normal infectivity of WR-A26(321–500) MV (described in the previous section), since this latter virus exhibited the ability to enter cells via plasma membrane fusion, just like WR-ΔA26 virus.

### A26 protein N-terminal region triggers pH-sensitive conformational changes *in vitro* that are dependent on His48 and His53

**Fig 3:**
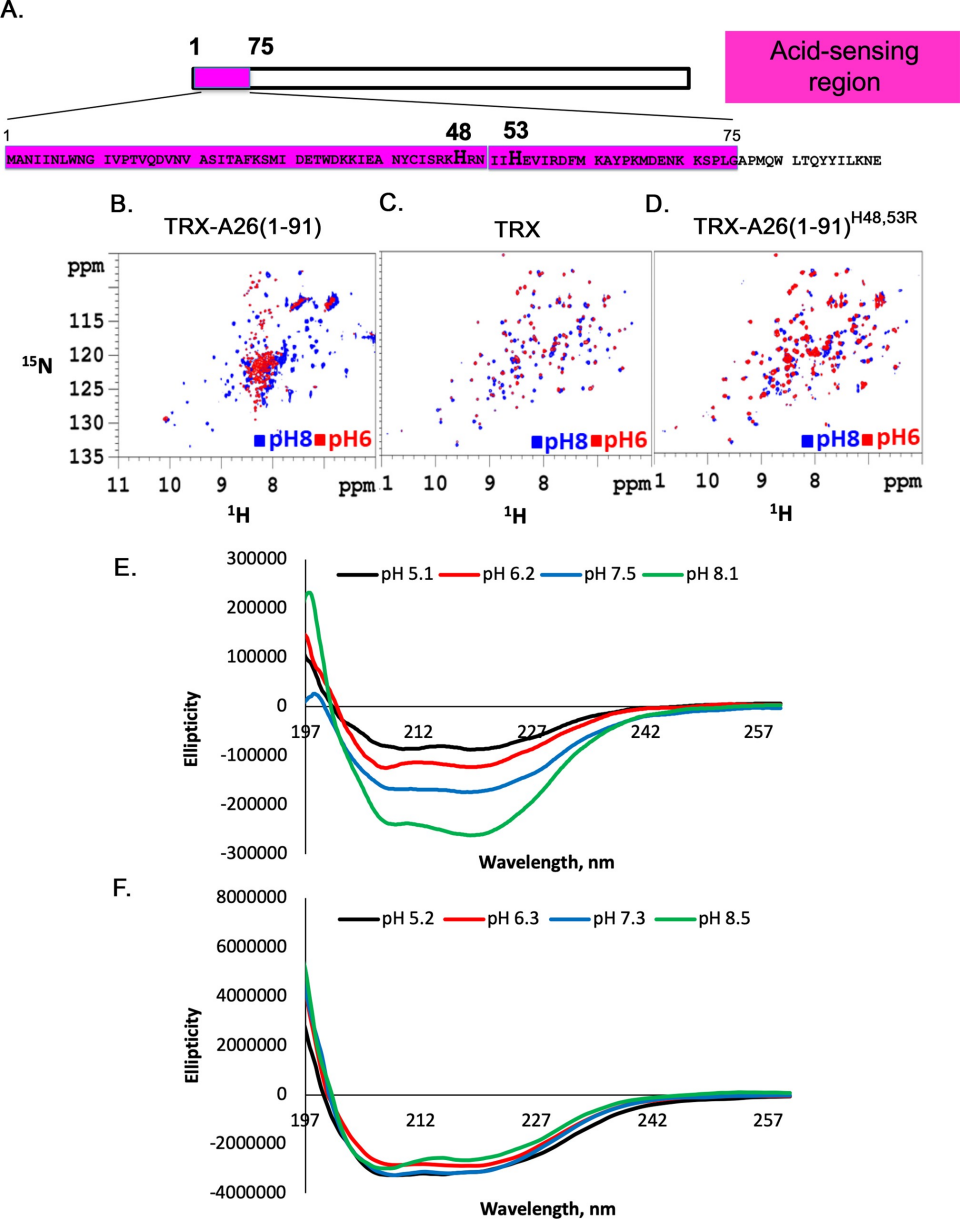
NMR analyses show that His48 and His53 are critical residues for acid-dependent conformational change of an A26 protein N-terminal fragment (aa 1–91) *in vitro*. **(A).** Protein sequence of the N-terminal of vaccinia WR-A26 protein (aa 1–91), showing positions of His48 and His53 within aa1-75 (in pink). **(B-D).** 2D ^1^H/^15^N HSQC NMR spectra of TRX-A26(1–91) fusion protein (in B), TRX (in C) and TRX-A26 (1–91)^H48.53R^ (in D). The 2D HSQC spectra were measured at pH 8 (in blue) and pH 6 (in red), respectively. **(E and F).** CD spectroscopy analyses of recombinant A26(1–91) (in E) and A26(1–91)^H48.53R^ (in F) as a function of pH values ranging from pH 5.1 to 8.5.

The above-described analyses prompted us to investigate the N-terminal aa sequences of A26 protein. Since two-dimensional (2D) ^1^H-^15^N heteronuclear single quantum coherence (HSQC) serves as a reliable measure of secondary structure and conformational change in solution, we then performed 2D HSQC experiment to examine the N-terminus of A26. To achieve better protein solubility and stability critical for NMR study, we fused A26(aa 1–91) coding region with thioredoxin (TRX), and purified the recombinant fusion protein TRX-A26(1–91). In addition, we also purified wild type TRX control protein and a mutant TRX-fused protein TRX-A26(1–91)^H48,53R^, in which His48 and His53 were mutated to Arg. We then applied 2D HSQC experiment to TRX-A26 (1–91), TRX-A26 (1–91)^H48,53R^ and TRX respectively (Fig 3B–3D). Notably, the 2D HSQC spectra of all three proteins—TRX, TRX-A26(1–91) and TRX-A26(1–91)^H48, H53R^—at pH 8 are somewhat similar (blue in Fig 3B–3D), suggesting that the 2D spectral signals are dominated by those of TRX protein. Furthermore, the spectral patterns of control TRX at pH 6 and pH 8 are nearly identical (Fig 3C), demonstrating that TRX is pH-insensitive. However, recombinant TRX-A26(1–91) at pH 6 exhibits a different 2D spectral pattern (red in Fig 3B) from that at pH 8 (blue in Fig 3B). At pH 6, the amide-^1^H signals gave rise to a narrow dispersion within 8–8.5 ppm, indicating a partially unfolded conformation [37]; in contrast, at pH 8, the amide-^1^H signals displayed a much wider distribution 7–10 ppm, indicative of a structured conformation. The data thus suggested that, when fused with TRX, the A26(1–91) fragment induced significant conformational changes in the TRX-A26(1–91) fusion protein at low pH. However, recombinant TRX-A26(1–91)^H48, H53R^ fusion protein exhibited similar 2D spectral patterning at pH 6 and pH 8 (Fig 3D), confirming that H48 and H53 are responsible for pH sensitivity of TRX-A26(1–91) *in vitro*.

To further confirm this conclusion, we then removed the TRX tag from the above-mentioned fusion proteins and performed circular dichroism (CD) spectroscopy to analyze conformational changes of recombinant A26(1–91) protein and A26(1–91)^H48, H53R^ mutant protein at different pH, ranging from 5.1 to 8.5 (Fig 3E and 3F). The CD spectrum of recombinant A26(1–91) protein exhibited α-helical ellipticity at 208 and 222 nm (Fig 3E). The ellipticity decreased as the pH value decreased, suggesting a transition from an α-helix to a random coil. In contrast, the A26(1–91)^H48, H53R^ mutant protein was insensitive to pH alteration from 5.2 to 8.5 (Fig 3F) although the secondary structure of the mutant protein may appear similar to A26(1–91) in α-helix content. Based on these data, we concluded that the N-terminal region (1–91) of A26 protein is sufficient for low pH-dependent conformational changes, and that His48 and His53 are essential for acid sensitivity of A26(1–91) protein *in vitro*.

### His48 and His53 are critical for A26 protein to regulate low pH-dependent membrane fusion

**Fig 4:**
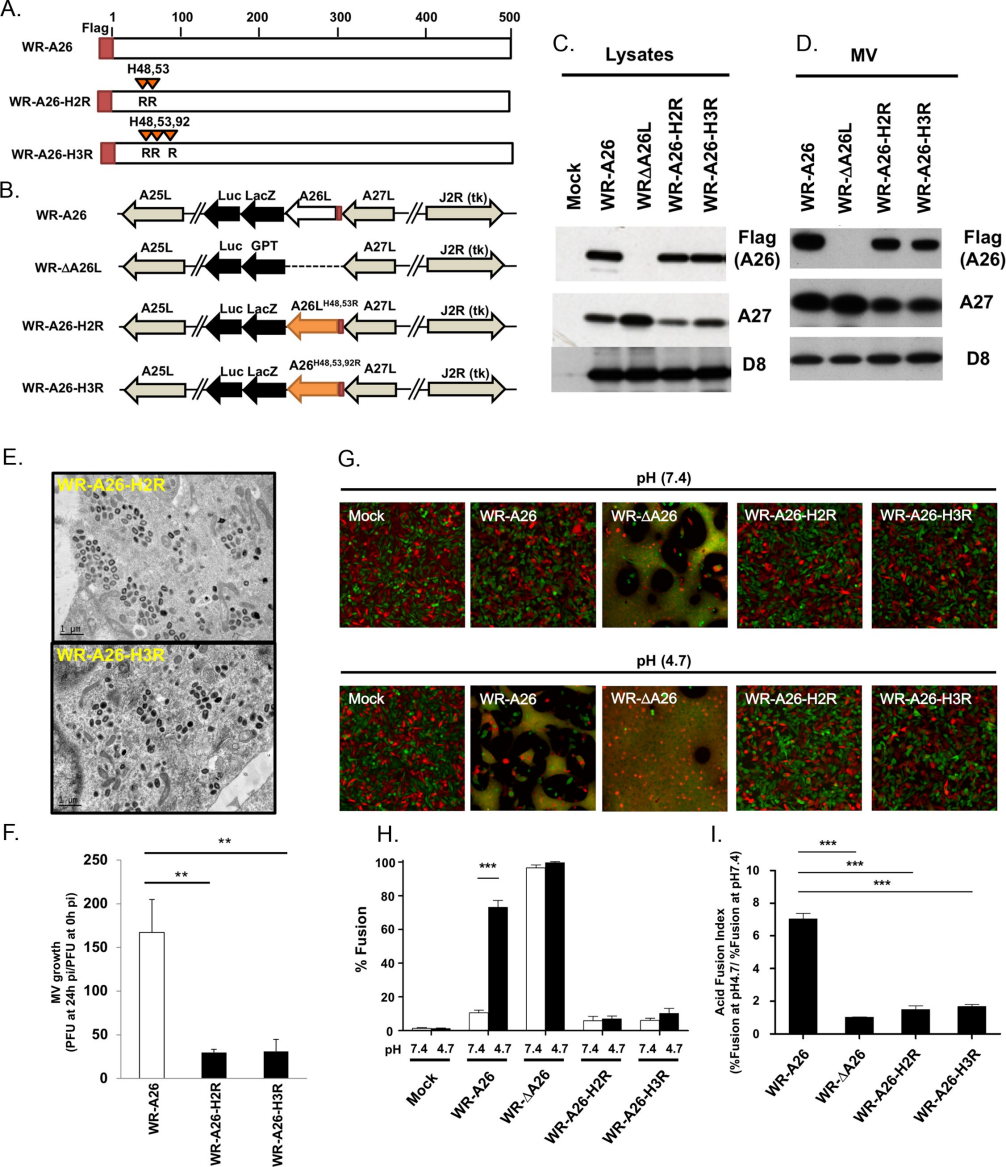
Generation of mutant vaccinia viruses containing His48R and His53R mutations (H2R) of A26 protein. **(A).** Schematic representations of full-length WR-A26, A26 His48R and His53R double-mutant (H2R mutant), and A26 His48R, His53R and His92R triple-mutant (H3R mutant) proteins. Each construct contained flag-tag sequences at the N-terminus. **(B).** Schematics of recombinant viruses showing the WR-A26-H2R and WR-A26-H3R mutant genome arrangements. WR-A26 and WR-ΔA26 were described previously [32, 36], and the latter was used as the parental virus to generate the WR-A26-H2R and WR-A26-H3R recombinant viruses. The A26-H2R and A26-H3R gene cassettes were inserted into the *A26L* native locus and recombinant virus was selected as blue plaques in agar plates containing X-gal. **(C).** Immunoblots of viral A26-H2R and A26-H3R proteins expressed in virus-infected cells at 24 hpi. (**D).** Immunoblots of A26-H2R and A26-H3R proteins in CsCl-purified MV particles. A27 and D8 proteins are two viral envelope proteins that serve as positive controls. **(E).** EM images of abundant MV from the WR-A26-H2R and WR-A26-H3R viruses in infected HeLa cells at 24 hpi. The scale bar represents 1 μm. **(F).** One-step growth of the WR-A26, WR-A26-H2R and WR-A26-H3R viruses in HeLa cells. Cells were infected with each virus at an MOI of 5 PFU per cell and then washed and harvested at 0 and 24 hpi for virus titer determination. The Y-axis represents MV growth, which was determined by dividing virus titers at 24 hpi with the respective input virus titer at 0 hpi. The experiments were repeated three times and the Student *t*-test was used for statistical analysis. ** p < 0.01. **(G).** Images of cell-cell fusion upon infection by respective MV. Single-fluorescent cells as described in Fig 2A were infected with each virus at an MOI of 50 PFU per cell, washed with neutral or acidic pH buffer for 3 min, and then subjected to live-imaging to monitor cell-cell fusion at 37°C for 2 h. **(H).** Quantification of % fusion for images described in (G). Five images for each virus were recorded and the % fusion was calculated using the image area of GFP^+^RFP^+^ double-fluorescent cells divided by that of single-fluorescent cells. The experiments were repeated three times and the bars represent standard deviations. Student *t*-test was used for statistical analyses. *** p <0.001. **(I).** The Acid Fusion Index was calculated by dividing the % of cell fusion at low pH (black bars in H) by that at neutral pH (white bars in H). Endocytic WR-A26 virus demonstrated an Acid Fusion Index of ~7.02, whereas the indexes of all other viruses were much lower, demonstrating their limited dependence on an acid environment for cell fusion. These experiments were repeated three times for each virus and the Student *t*-test was used for statistical analyses. *** p <0.001.

To demonstrate that His48 and His53 of A26 protein are indeed involved in acid-dependent membrane fusion of MV in cells, we created a recombinant vaccinia virus (WR-A26-H2R) that expresses flag-tagged A26-H48, H53R double mutant protein (A26-H2R protein) in the infected cells (Fig 4A and 4B). Unlike wild-type A26 protein, A26-H2R mutant protein should fail to undergo conformational changes in response to low environmental pH. We also generated another recombinant virus A26-H3R that contains an extra H92R mutation in flag-tagged A26 protein, in addition to H48R and H53R (Fig 4A and 4B).

Immunoblot analyses revealed comparable levels of WR-A26, A26-H2R and A26-H3R proteins in the infected cells (Fig 4C) and in purified MV particles (Fig 4D). Although WR-A26-H2R and WR-A26-H3R mutant viruses exhibited normal MV assembly in the infected cells (Fig 4E), MV growth was reduced (Fig 4F) and MV particles presented very low infectivity (Table 1), reminiscent of our observations of the WR-A26(76–500) deletion virus.

In cell-cell fusion assays, WR-A26-H2R and WR-A26-H3R mutant viruses did not trigger cell-cell fusion at either neutral or low pH (Fig 4G, quantified in Fig 4H), similar to WR-A26(76–500) virus, suggesting that the A26-H2R and A26-H3R proteins are constitutive fusion suppressors, as evidenced by their low acid fusion indexes (Fig 4I). Taken together, these mutational studies show that H48 and H53 are required for the functioning of the acid-sensitive region of A26 protein during vaccinia MV-mediated membrane fusion. Additional mutation of His92 did not enhance the mutant virus phenotype.

### His48 and His53 in helix α2 of recombinant A26(1–397) protein form His-cation pairs within the N-terminal region to create pH-dependent electrostatic repulsions

**Fig 5:**
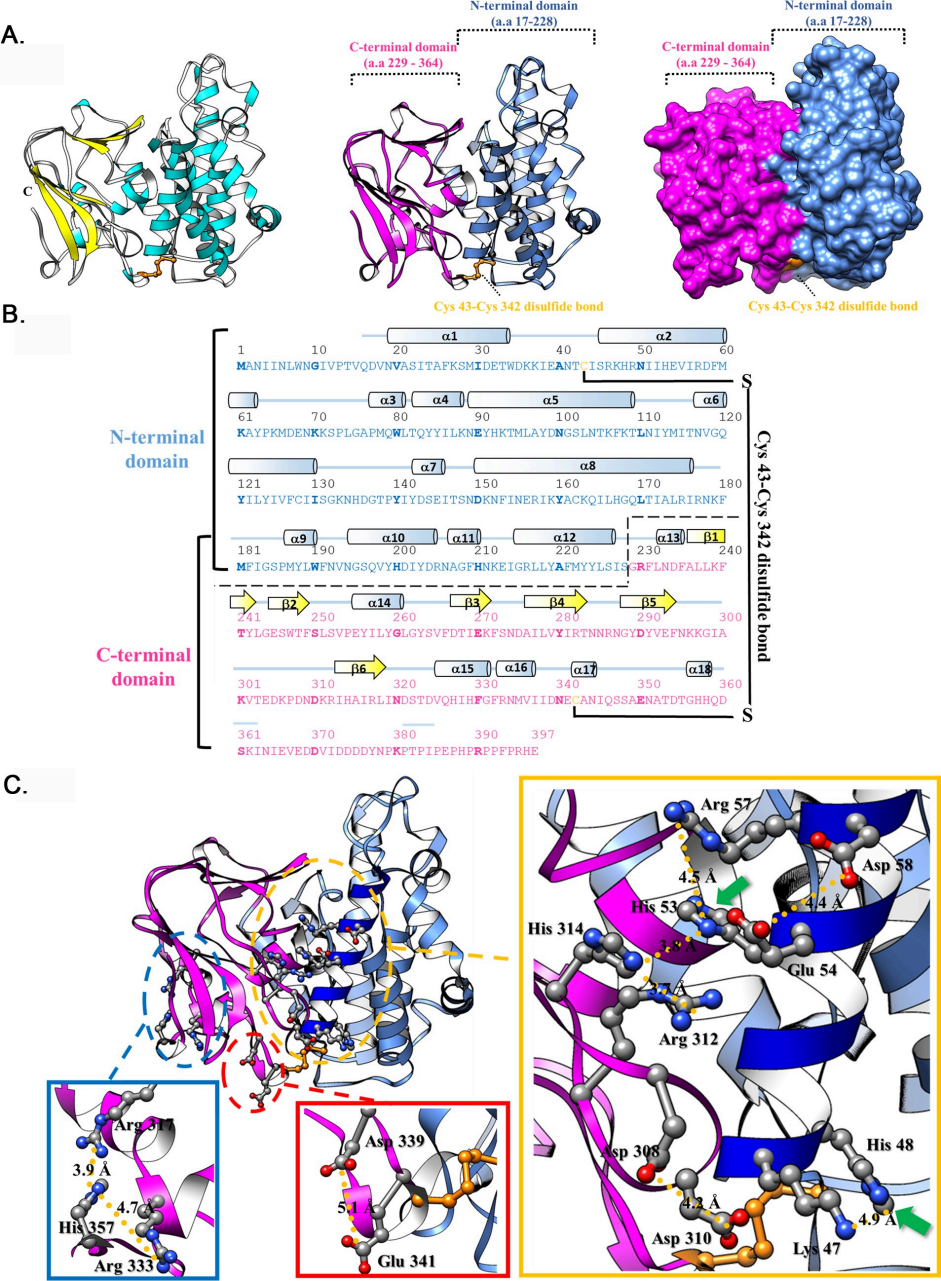
Crystal structure of recombinant A26 protein (aa 1–397). **(A).** A ribbon diagram of the A26^1-397^ structure. The α-helices and β-strands are colored in cyan and yellow, respectively. Two domains, NTD (aa 17–228) and CTD (aa229-364), are shown and highlighted in cornflower blue and magenta, respectively. **(B)**. The secondary structural elements are shown above the amino acid sequences, with cyan cylinders and yellow arrows representing α-helices and β-strands, respectively. **(C).** The His-cation and AniAni pairs in the recombinant A26^1-397^ structure. The amino acids that involve in His-cation and AniAni pairs are shown as ball and stick models. Green arrows show the position of His48 and His53.

To further understand how His48 and His53 mediate A26 protein conformational change, we endeavored to obtain a crystal structure of A26 protein. We generated various A26 gene constructs for protein expression in *E*. *coli* and only A26(1–420) and A26(1–420)-C43C342A were successfully purified. However, we failed to obtain crystals from either proteins, suggesting that A26(1–420) and A26(1–420)-C43C342A still contain some disordered regions. Our limited trypsin digestion identified aa 395–420 as an unstable region so we generated recombinant A26(1–397) protein and purified it through affinity and monoQ ion exchange chromatography before solving its crystal structure (Table 2 and Fig 5). The overall A26(1–397) structure consists of 18 α-helices and 6 β-strands (Fig 5A and 5B), with an N-terminal α-helical domain (NTD; aa 17–228) and a C-terminal β-sheet domain (CTD; aa 229–364). The total solvent accessible surface area (SA) of A26^1-397^ is 14606 Å^2^, and the buried area between these two domains are 4252.3 Å^2^ the interface (the contact areas on NTD and CTD are 2387 and 1865.3 Å^2^, respectively). Additionally, an inter-domain disulfide bond is present between Cys43 and Cys342, consistent with our previous mutational analyses [35]. To address the novel fold of this structure, we used the DALI server (http://ekhidna2.biocenter.helsinki.fi/dali/) to perform a structural homolog analysis. The results showed that the overall structure of A26^1-397^ does not have any significant hit. The most similar protein to A26^1-397^ is the C-terminal MIF4G domain in NOT1 (PDB ID 6H3Z with RMSD above 11.4 Å). Moreover, the A26^1-397^ NTD exhibits only minor similarity to importin protein (PDB ID:3zkv; with RMSD above 6 Å). In comparison, the folding of A26^1-397^ CTD is similar to gamma crystallin S (PDB ID: 1m8u; RMSD = 2.2 Å). However, since sequence identity between the A26^1-397^ CTD and gamma crystallin S is below 15%, it does not suggest a close relationship between these two proteins. Consequently, the A26^1-397^ structure appears to present a novel fold with two distinct domains.

**Table 2:** Data collection and refinement statistics for the crystal structure of A26(1–397) protein.

Data collection	SeMet A26^1-397^
Wavelength (Å)	0.97907
Space group	*P*2_1_
Unit cell *a*, *b*, *c* (Å)	45.38 80.72 53.81
α, β, γ (°)	90.00 113.80 90.00
Resolution (Å)	20–1.18 (1.22–1.18)
Unique reflections	114858 (10993)
Redundancy	3.4 (2.7)
Completeness (%)	99.4(95.2)
I / σ(I)	13.7 (2.2)
R_merge_ (%)	8.3 (41.3)
**Refinement**	
R_work_ (%)	10.7
R_free_ (%)	13.0
Bond r.m.s.d. (Å)	0.013
Angle r.m.s.d. (°)	1.532
Mean B value / no of atom	20.0 / 3544
**Ramachandran plot (%)**	
Most favored (%)	96.72
Allowed (%)	3.28
Outliers (%)	0
**Structure validation by MolProbity** [38]	
MolProbity score	1.10
Clash score	2.68

Many viral fusion proteins exhibit pH-dependent conformational changes that are mainly controlled by electrostatic repulsion [39]. Although A26 protein is a fusion suppressor and not a viral fusion protein, its ability to respond to acidic environments suggests that electrostatic replusion may also contribute to its conformational changes at low pH. In general, two classes of paired amino acids are involved in pH-dependent electrostatic repulsions within a protein, i.e., His-cation repulsion at acidic pH and anion-anion (Ani-Ani) repulsion at neutral pH [39]. For His-cation pairs, the histidine residues are usually close (< 7 Å) to other His or basic residues (Arg or Lys). Therefore, we investigated whether any His-cation or Ani-Ani pairs are present in the A26(1–397) structure. As shown in Fig 5C, most His-cation or Ani-Ani pairs are located around helix α2 that hosts His53 (green arrow in Fig 5C), so upon encountering the acidic endosomal pH, charge repulsion produced by the His-cation pair between His53 and Arg57 destablizes the conformation of helix α2. It is worth noting that His53 is also cation-paired with Arg312 and His314, both of which are located in the CTD of A26(1–397), so electrostatic repulsions of His53 at low pH may also destabilize the interactions between helix α2 and the CTD. Furthermore, His48 (green arrow in Fig 5C) is His-cation-paired with Lys47 in helix α2. We observed that an Asp308-Asp310 pair is also adjacent to this region. In another notable obervation, the predicted pKa of the residues that involve in His-cation or Ani-Ani pairs (S1 Table) are usually low (below 5) and most residues with low pKa are found in the helix α2 region, indicating that these residues prefer proton release. Thus, our crystal structure of A26(1–397) protein strongly supports that His-cation pairs involving both His48 and His53 within the N-terminal region most likely contribute to structural alterations by increasing electrostatic repulsions under acidic conditions.

### His-cation pairs at the N-terminal domain of A26 protein are critical for the acid-dependent electrostatic repulsion that initiates acid-dependent membrane fusion

**Fig 6:**
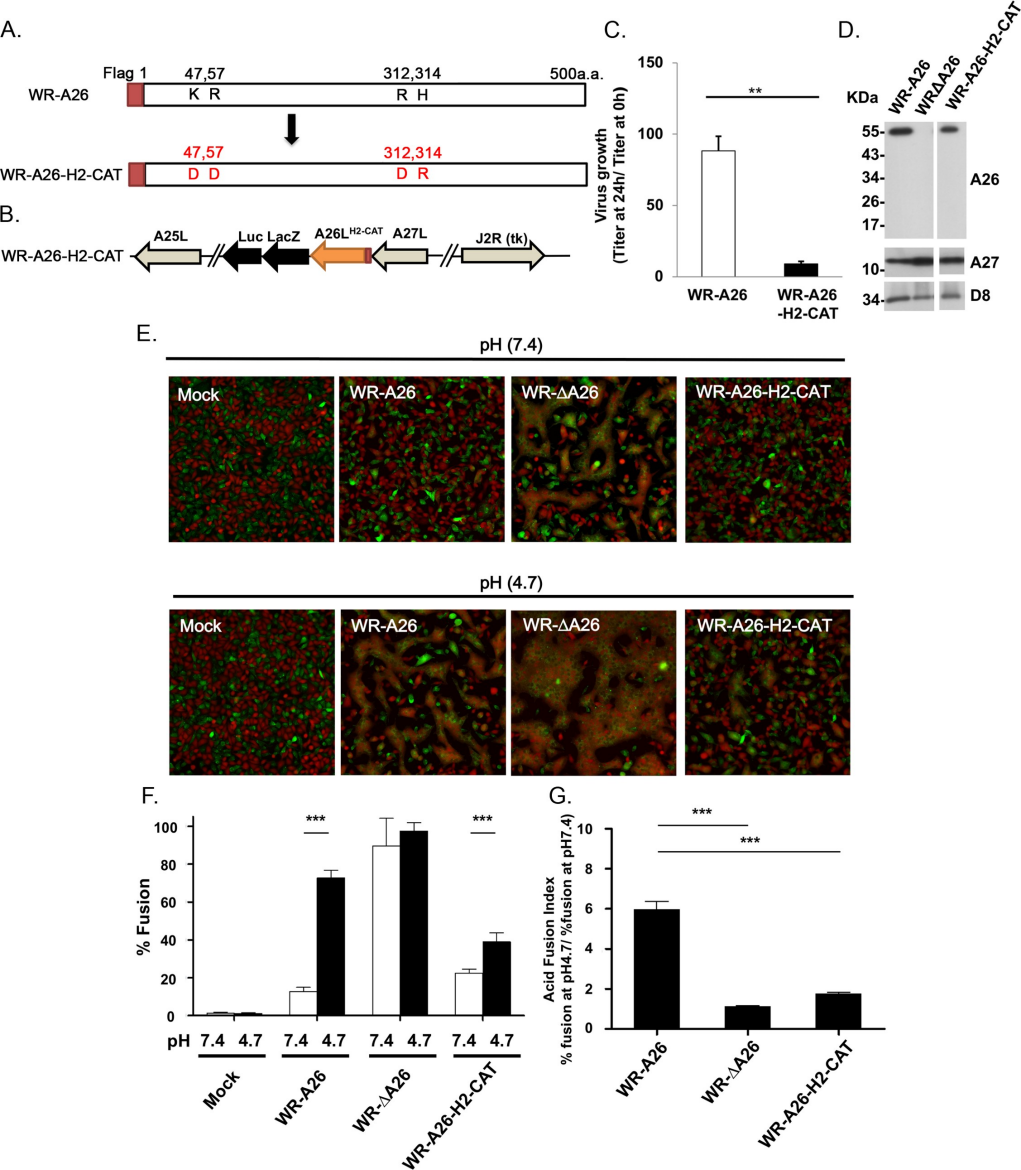
Generation of A26 mutant vaccinia viruses containing K47D, R57D, R312D and H314R mutations in A26 protein (A26-H2-CAT). **(A).** Schematic representation of full-length WR-A26 and mutant A26-H2-CAT proteins. Each construct contains flag-tag sequences at the N-terminus. **(B).** Genome arrangement of WR-A26-H2-CAT recombinant virus. The A26-H2-CAT gene cassette was inserted into the endogenous A26L locus and selected as blue plaques in agar plates containing X-gal. **(C).** One-step growth of the WR-A26 and WR-A26-H2-CAT viruses in HeLa cells. Cells were infected with each virus at an MOI of 5 PFU per cell for 60 min and then cells were washed and harvested at 0 and 24 hpi for virus titer determination. The Y-axis represents MV growth, which was determined by dividing virus titers at 24 hpi with the respective input virus titers at 0 hpi. The experiments were repeated three times and the Student *t*-test was used for statistical analysis. ** p < 0.01. **(D).** Immunoblots of WR-A26, WR-ΔA26 and WR-A26-H2-CAT mutant proteins in CsCl-purified MV particles. A27 and D8 proteins are two viral envelope proteins that served as positive controls. **(E)**. Images of cell-cell fusion induced upon MV infection. Single-fluorescent cells as described in Fig 2A were infected with each virus at an MOI of 50 PFU per cell, washed with neutral or acidic pH buffer for 3 min, and then subjected to live-imaging to monitor cell-cell fusion at 37°C for 2 h. **(F).** Quantification of the % fusion of each virus described in E. Five images for each virus were recorded and the % fusion was calculated based on the ratio of image area of GFP^+^RFP^+^ double-fluorescent cells divided by that of single-fluorescent cells. *** p <0.001. **(G).** The Acid Fusion Index was calculated from data (in F) by dividing the % of cell fusion at low pH (the black bars) by that at neutral pH (the white bars). Endocytic WR-A26 had an Acid Fusion Index of ~5.96, whereas the indexes of other viruses were much lower, demonstrating a significantly reduced dependence on acidic environments for cell fusion. The experiments were repeated three times for each virus and the Student *t*-test was used for statistical analyses. *** p <0.001.

We performed *in vitro* mutagenesis to express an A26 mutant protein (A26-H2-CAT) that contains K47D, R57D, R312D and H314R mutations to reduce cation-mediated repulsion at low pH via His48 and His53 (Fig 6A). As expected, yield of the recombinant WR-A26-H2-CAT virus (Fig 6B) at 24 hpi was significantly reduced (Fig 6C). Purified WR-A26-H2-CAT MV particles contained mutant A26 protein of the correct size (Fig 6D), but exhibited low infectivity with an increased particle-to-PFU ratio of ~147 (Table 3). Importantly, the WR-A26-H2-CAT mutant virus triggered less cell-cell fusion at both neutral and acidic pH (Fig 6E and 6F) and it presented a low acid fusion index (Fig 6G). Thus, we conclude that electrostatic repulsion induced by the N-terminal-protonated His48 and His53 residues and their surrounding basic aa (K47, R57, R312 and H314) is essential for conformational changes of A26 protein at low pH. Interference with these conformational changes will inhibit subsequent membrane fusion of an endocytic vaccinia virus.

**Table 3:** MV particle infectivity of WR-A26, WR-A26-H2-CAT and the revertant virus WR-H2-CAT-Rev1.

MV	Infectivity (Mean ±SD)
**WR-A26**^**a**^	**42.5 (±15.4)**
**WR-A26-H2-CAT**	147(±27)*
**WR-A26-H2-CAT-Rev1**	**33.6(±16.7)**

^a^WR-A26 was used as a positive control in the experiments.

*Data were analyzed using a two-tailed unpaired student’s *t* test. *, P<0.05.

### A26 protein second-site mutations can rescue recombinant vaccinia MV infectivity by activating a suppressor-independent plasma membrane fusion pathway

**Fig 7:**
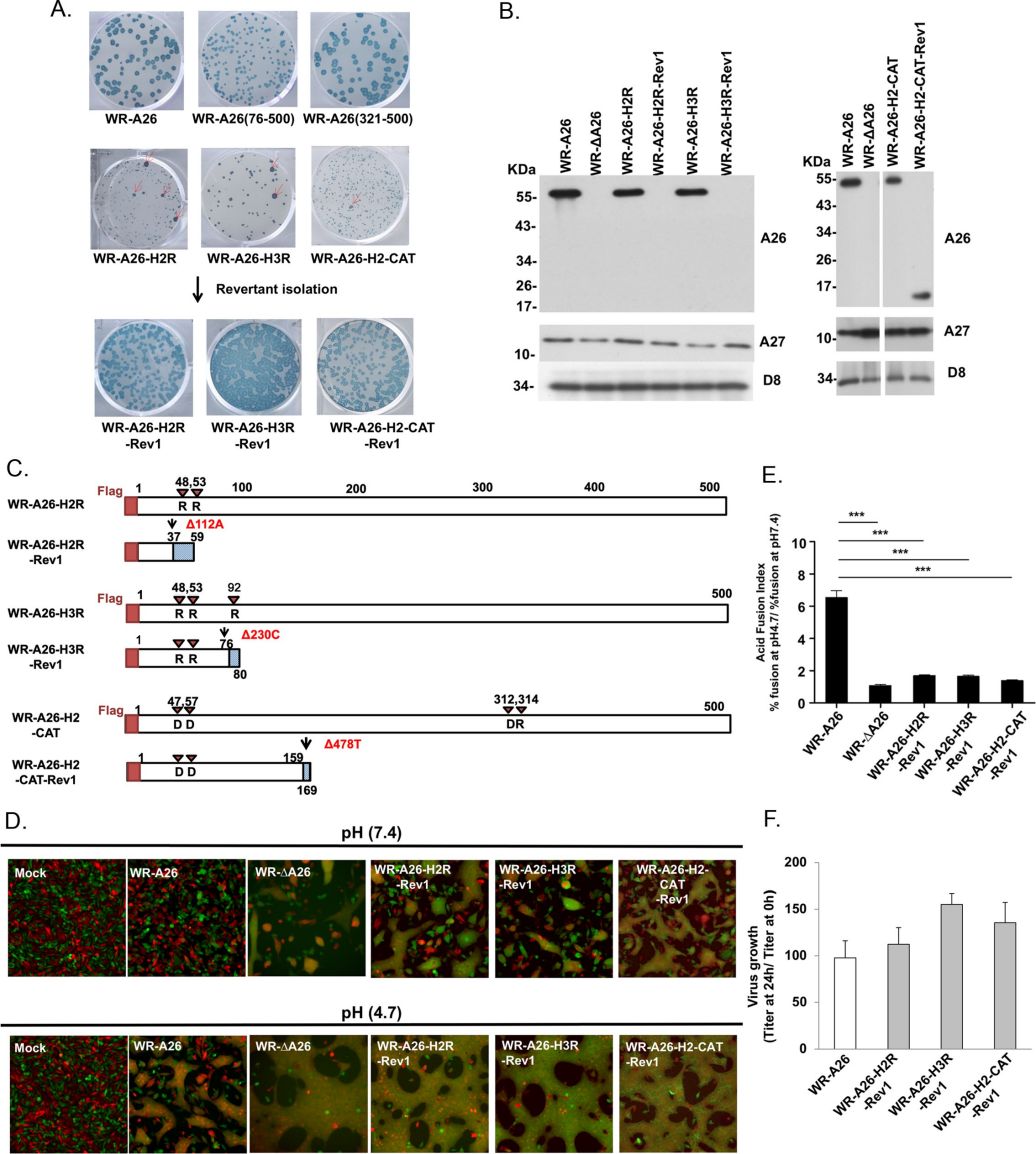
Revertant viruses exhibit a large plaque phenotype and regained MV infectivity by generating intragenic second-site mutations in the *A26L* ORF that caused a frame-shift and premature termination of A26 protein translation. **(A).** Plaque phenotypes of control WR-A26 virus and the small plaque phenotype of various A26 mutant viruses. Low percentages (~2%) of large plaque revertants (Rev) (indicated by red arrows) were isolated from each mutant virus and plaque-purified. **(B).** Immunoblots of A26 protein from infected HeLa cells at 24 hpi. HeLa cells were infected with each virus as indicated and the cell lysates were harvested at 24 hpi for immunoblot analyses. A27 and D8 proteins are two viral envelope proteins that served as positive controls. **(C).** Schematic representation of mutant A26 proteins and their revertant A26 proteins, respectively. Revertant A26 proteins contain a second-site deletion that causes a frame-shift and premature termination of A26 mutant protein. Red arrowheads show the designed H2R, H3R and H2-CAT amino acids mutated in each A26 protein. Each revertant A26 protein is color-coded: N-terminal flag tag (red box); translated A26 aa upstream of the second-site deletion (white box); and aberrant downstream aa due to frame-shift (blue dotted box). Second-site mutations are shown in red text. **(D).** Images of cell-cell fusion induced by MV infections. Single-fluorescent cells as described in Fig 2A were infected with each virus at an MOI of 50 PFU per cell, washed with neutral or acidic pH buffer for 3 min, and then subjected to live-imaging to monitor cell-cell fusion at 37°C for 2 h. **(E).** The Acid Fusion Index was calculated by dividing the % of cell fusion at low pH by that at neutral pH, similar to what is described in Fig 6F. Endocytic WR-A26 virus has an Acid Fusion Index of 6.53, whereas the indexes of the other viruses were much smaller, indicating limited dependence on an acid environment for cell fusion. The experiments were repeated three times for each virus and the Student *t*-test was used for statistical analyses. *** p <0.001. **(F).** One-step growth of WR-A26 and revertant viruses in HeLa cells. Cells were infected with each virus at an MOI of 5 PFU per cell and cells were washed and harvested at 0 and 24 hpi for virus titer determination. The Y-axis represents MV growth, which was determined by dividing virus titers at 24 hpi with the respective input virus titer at 0 hpi. The experiments were repeated three times and there was no significant growth difference among these revertant viruses and control WR-A26 virus (Student *t*-test).

During our experiments on HeLa cells, we noticed that WR-A26(76–500) recombinant virus formed plaques that were slightly smaller than those of control WR-A26 and recombinant WR-A26(321–500) viruses (Fig 7A). Interestingly, WR-A26-H2R, WR-A26-H3R and WR-A26-H2-CAT recombinant virus all formed tiny plaques on HeLa cells. These A26 mutant viruses appeared unstable, generating spontaneous “large plaque” revertants during early virus passaging and propagation (red arrows in Fig 7A). Therefore, we isolated several of the large plaque revertants (Rev) from the WR-A26-H2R, WR-A26-H3R and WR-A26-H2-CAT mutant viruses and analyzed their protein expression in the infected HeLa cells. As shown in Fig 7B, the size of the A26 protein in all these large-plaque revertant viruses was either smaller relative to control or the protein was completely absent in the infected cells. We suspected that this outcome was due to second-site mutations within the A26 ORF so we purified viral genomic DNA from cells infected with the WR-A26-H2R-Rev1, WR-A26-H3R-Rev1 and WR-A26-H2-CAT-Rev1 revertant viruses and subjected it to whole genome sequencing (results summarized in Table 4).

**Table 4:** Whole genome sequencing of A26 revertant viruses derived from WR-A26-H2R, WR-A26-H3R and WR-A26-H2-CAT mutants.

Recombinant virus	In vitro mutagenesis	Second-site mutations in *A26L* ORF in Revertants	Second-site mutations cause a.a. changes of A26 protein
**WR-A26-H2R**	**H48,53R**	**None**	**(500 aa)**
**WR-A26-H2R-Rev1**	**H48,53R**	**Δ112A**	**Frameshift** **(59 aa)**
**WR-A26-H3R**	**H48,53,92R**	**None**	**(500 a.a.)**
WR-A26-H3R-Rev1^*^	**H48,53,92R**	**Δ230C**	**Frameshift** **(80 aa)**
**WR-A26-H2-CAT**	**H314R,R57D,R312D,K47D**	**None**	**(500 aa)**
**WR-A26-H2-CAT** **-Rev1**	**H314R,R57D,R312D,K47D**	**Δ478T**	**Frameshift** **(169aa)**

^*^Also contained a C-to-A mutation in a truncated pseudogene *B3R* ORF.

All revertant viruses retained the designed A26 mutations present in the parental WR-A26-H2R, WR-A26-H3R and WR-A26-H2-CAT mutant strains, but they also all contained an extra intragenic deletion in the A26 ORF that resulted in a frame-shift and premature termination of A26 protein translation (Fig 7C). Our sequencing results are consistent with the immunoblots (Fig 7B), although some small A26 fragments were not detected in the lysates, probably due to rapid degradation. WR-A26-H2R-Rev1 and WR-A26-H2-CAT-Rev1 genomes contained no other gene mutations, whereas WR-A26-H3R-Rev1 contains a C-to-A mutation in a pseudogene B3R ORF (a truncated ortholog of camelpox viral gene 176R). The camelpox 176R encodes a schlafen-like protein in virus-infected cells, but a screening of 16 vaccinia viruses revealed no evidence of B3R expression [40]. We conclude that all three revertant viruses host second-site mutations that only affect A26 protein function. Since the A26 fragments in all of these revertant viruses are much shorter and lack the C-terminal A27-interacting region (Fig 7C), they are unlikely to be packaged into revertant MV particles. Accordingly, we anticipated that these three revertant viruses would exhibit a phenotype similar to that of WR-ΔA26 virus. Indeed, the WR-A26-H2R-Rev1, WR-A26-H3R-Rev1 and WR-A26-H2-CAT-Rev1 viruses mediated clear cell-cell fusion under neutral pH, just like WR-ΔA26 (Fig 7D), and presented acid-independent fusion activity (Fig 7E). Therefore, by mutating A26 protein to eliminate His-cation-mediated repulsion at low pH, we created a constitutive suppressor for viral membrane fusion so mutant MV infectivity diminished significantly. However, second-site mutations in the A26 gene resulted in revertant viruses regaining MV infectivity (Tables 1 and 3) and exhibiting normal virus yields (Fig 7F) through plasma membrane fusion. Successful selection of these revertant viruses provides strong evidence that vaccinia MV can switch between the endocytosis and plasma membrane fusion entry pathways, mediated by A26 protein on MV. Most importantly, we have uncovered the structure of the N-terminal region of A26 protein and provide mechanistic insights demonstrating that electrostatic repulsion of His48 and His53 is critical for controlling acid-dependent conformational change of A26 protein prior to virus-mediated endocytic membrane fusion.

## Discussion

Poxviruses are very large and are known to contain multiple proteins of overlapping or redundant functions. Vaccinia virus contains four envelope proteins for cell attachment [5–8, 10], whereas viral membrane fusion requires a separate fusion protein complex of 11 components that specifically performs membrane fusion (reviewed in [3, 4]). Therefore, vaccinia virus has evolved two separate sets of envelope proteins specialized for cell attachment and membrane fusion, respectively, during cell entry.

Many studies have reported that vaccinia virus entry pathways vary depending on virus strains and cell types [17, 31, 41]. How can virus entry pathways be strain-dependent? Using proteomics and genetic complementation analyses, we previously showed that A26 protein determines MV entry pathways in several cell lines [32]. A26^+^ strains, such as the WR and IHD-J strains, employ endocytosis to enter HeLa cells, whereas A26^-^ strains, such as MVA and Copenhagen, employ a plasma membrane fusion pathway [32, 33]. Viral endocytosis would appear to be an optimal mode of virus entry into cells since no envelope proteins or viral membranes remain on the host cell surface for host B and T cell detection. However, under certain conditions when endocytosis becomes a less optimal route for A26^+^ vaccinia virus to enter cells, deletion of the A26 ORF results in A26^-^ MV progeny that can infect cells through plasma membrane fusion, thereby broadening the host range. Another advantage of having multiple entry pathways is to avoid innate immune sensing and antiviral signaling activation. We recently infected murine bone marrow-derived macrophages (BMDM) with WR or WR-ΔA26 virus and found that the IFNβ-Stat1 signaling pathway was preferentially induced by endocytic WR virus but not by WR-ΔA26 virus [36]. Consequently, WR-ΔA26 exhibited enhanced virulence in mice compared to WR vaccinia virus [36].

Here in this study, we have used genetic, biochemical and structure analyses to provide strong evidence supporting that vaccinia A26 viral protein functions as an acid-sensitive fusion suppressor of MV particles during virus endocytosis. To demonstrate the critical role of the pH-dependent conformational changes, we purposely generated His48R and His53R mutations (A26-H2R) in the N-terminal domain of A26 protein so that the acid-sensing region is rendered pH-independent. We assume that the N-terminal domain in the A26-H2R mutant may structurally mimic the low pH conformation because of constitutive repulsion forces even in neutral pH environments. However, this scenario does not necessarily mean that the conformation of the fusion suppressor domain also changes during assembly into MV particles. Based on the A26 crystal structure, we generated H2-CAT mutations such that the resulting N-terminal domain structure of the A26-H2-CAT protein also becomes pH-independent but, in this case, His-Cation repulsion was replaced by His-Anion attraction. Therefore, we anticipated that the N-terminal domain of A26-H2-CAT mimics the neutral pH conformation, even in acidic environments. Despite different structural mimics being generated in the N-terminal regions of A26-H2 and A26-H2-CAT, both proteins were acid-insensitive and constitutively suppressed fusion. These outcomes demonstrate that pH-dependent conformational changes via His-Cation repulsion, as opposed to a particular acid-stable N-terminal domain structure *per se*, are essential for regulating membrane fusion activation. The A26-H2R and A26-H2R-CAT mutations eliminated the acid-dependent response and these mutant proteins retain fusion-suppressing functions. Taken together, our deletion and mutagenesis data support that His48 and His53 in the N-terminal domain are protonated at low pH, creating electrostatic repulsion with surrounding residues (K47, R57, R312 and H314) that results in conformational changes. Our model is also consistent with the data from the A26(76–500) deletion protein, which behaves as a constitutive fusion suppressor upon deletion of the acid-sensing domain. Finally, although we do not have the crystal structure of the C-terminal region of A26 protein, we have generated other C-terminal His-to-Arg mutant viruses, such as A26^H357R^, A26^H425, 432 439R^, A26^H439, 452, 453R^ and A26^H425, 432, 439, 452, 453R^. All these A26 C-terminal mutant viruses expressed A26 mutant proteins of correct size, formed plaques of normal size, and grew to high titers, suggesting that, in contrast to His48 and His53, these C-terminal His residues have a limited role in A26-mediated MV entry and fusion regulation.

In our A26 crystal structure obtained at neutral pH, His48 and His53 are located in the helix α2, which is strategically sandwiched between N-terminal helix clusters and C-terminal beta strands (Fig 5C). This implies that the helix α2 is important for maintaining protein structure stability at neutral pH. The A26 protein structure revealed that these C-terminal beta sheets are distinct from the N-terminal helix cluster, with a sole intra-molecular cysteine disulfide bond formed between C43 and C342, suggesting that the C-terminal domain may stabilize the helix-rich N-terminal domain or *vice versa*. Currently, we do not have an A26 crystal structure under conditions of low pH nor for A26-H2R mutant protein at neutral pH so we do not know how the N-terminal domain alters A26 protein structure under the acidic condition.

To investigate pH-mediated changes of A26 by using the current results, we employed Discovery Studio [42] to produce an A26^1-397^ model at pH 4.7 [A26^1-397^ model (pH 4.7)]. We first compared the surface electrostatic potential between A26^1-397^ and A26^1-397^ (pH 4.7) model (S1 Fig). Our results show that the NTD of A26^1-397^ is highly positively charged, and the CTD of A26^1-397^ is relatively negatively charged. Furthermore, we identified a positively-charged cavity (dotted green box in S1 Fig, panel A) between two domains. The cavity is formed by the α2 helix region, which we propose plays an important role in the pH-dependent regulation of A26.

Next, we endeavored to address the issue of pH-mediated changes in A26. Since the crystal structure of A26^1-397^ was obtained from a neutral pH, we first used Discovery Studio to produce a model of A26^1-397^ at pH 4.7, as described in materials and methods. We used the final conformation for subsequent analysis (e.g. to calculate surface electrostatic potential and solvent accessibility, etc.). We then compared the structures and surface charges of the A26^1-397^ and the A26^1-397^ (pH 4.7) model. In the A26^1-397^ (pH 4.7) model, the low pH enriches the positive charges and reduces the negative charges on the protein surface (S1 Fig, panel B). We also observed a significant difference in the low pH model in terms of the region comprising the α1 and α2 helices, both of which presented a loosely coiled structure. This outcome may be related to electrostatic repulsions caused by the enrichment of positive charges in this region. Although our low pH model seems to support our hypothesis, it will be necessary to resolve the actual structure of A26^1-397^ at low pH for verification.

The solvent accessibility (SA) of A26^1-397^ and A26^1-397^ (pH 4.7) model were calculated using Discovery Studio [42]. However, the SA difference between overall protein of A26^1-397^ and A26^1-397^ model (pH 4.7) is not much (14606 and 14357 Å^2^, respectively, representing a difference of 1.7%). We also established the SA of each residue for both structures (S2 Table). Using a threshold for individual residues of a 15% difference in SA between structures, we predicted the SA of 27 residues is reduced in the A26^1-397^ (pH 4.7) model, whereas it was increased for another 17 residues. Notably, although the SA of most residues that involve His–Cation and Ani-Ani pairs were not greatly altered, we observed pronounced SA changes adjacent to the α2 helix (S2 Fig), suggesting that this region may undergo a conformational change at low pH. Again, this analysis was based on a model of A26^1-397^ at low pH, it is necessary to resolve the actual structure of A26 at low pH to precisely elucidate the pH-dependent changes in A26.

**Fig 8:**
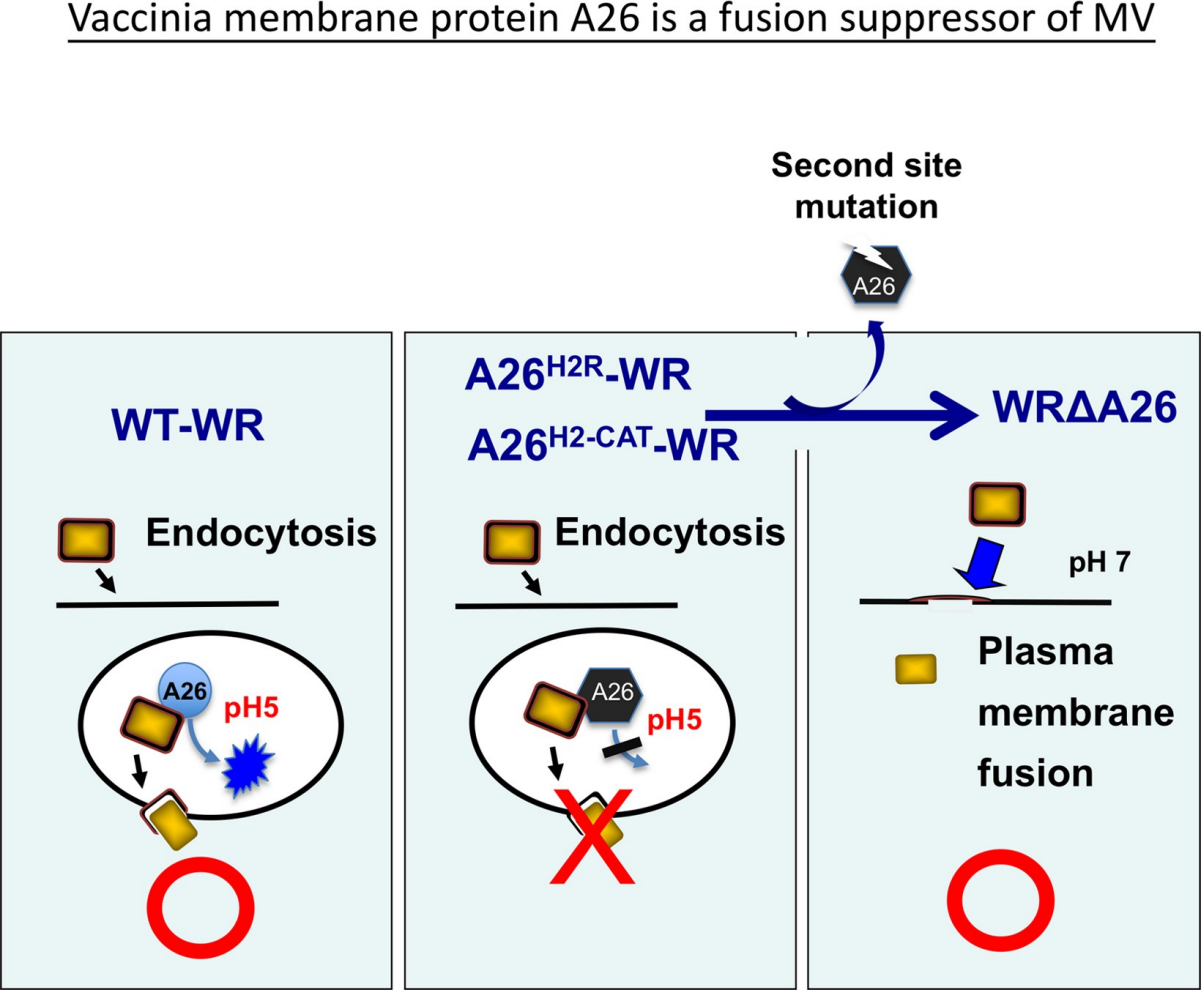
Vaccinia virus membrane protein A26 is a fusion suppressor of MV. Data presented in this study reveal an important role for A26 protein in endocytic entry of the WR strain of vaccinia MV. Low pH in endosomes triggers conformational changes of wild type A26 protein to allow membrane fusion within vesicles. However, when mutant A26 protein, such as A26^H2R^ or A26^H2-CAT^, loses the ability to execute conformational changes at low pH, it becomes a constitutive suppressor that blocks virus membrane fusion, resulting in significant loss of mature virus infectivity. Subsequent generation of the second-site mutations then leads to truncation of A26 protein so the resulting revertant viruses acquire a phenotype similar to that of WRΔA26 virus and initiate virus fusion with the plasma membrane at neutral pH to recover MV infectivity.

It is worth noting that though A26 protein only exists on MV and not on EV, it has been hypothesized that A26 protein in cells can negatively regulate MV egress to Golgi to form EV [43, 44]. Since all of our designed acid-insensitive A26 mutant viruses exhibit small plaque sizes, we rationalized that conformational changes of A26 protein may also control MV to EV egress at late phase in infected cells. Subsequent isolation of revertant viruses from the WR-A26-H2R, WR-A26-H3R and WR-A26-H2-CAT mutant viruses was unexpected. However, these revertant viruses clearly demonstrate how vaccinia mature virus can cope with detrimental mutations of A26 protein and how, through second-site mutations in A26 protein, the revertant mature virus regains host cell entry ability by switching from endocytosis to plasma membrane fusion (Fig 8). Apart from the three revertants reported in Fig 7, we analyzed additional revertants with the large plaque phenotype—Rev2 and 3 from WR-A26-H2R; Rev2, 3 and 4 from WR-A26-H3R; and Rev2, 3, and 4 from WR-A26-H2-CAT (S3 Fig.)—and immunoblots showed that all of these revertants contain a smaller form of A26 protein or no A26 protein at all (S4 Fig). PCR amplification and sequencing of *A26L* genes from these viral genomes revealed additional second-site mutations within the *A26L* ORF (S5 Fig.) that resulted in a frame-shift and premature termination of A26 protein (S6 Fig.), consistent with our immunoblot data and supporting our model shown in Fig 8. As expected, all of these revertant viruses infected cells via plasma membrane fusion, and displayed robust cell-cell fusion at both neutral and acidic pH (S7 Fig).

Previous crystallization experiments on viral fusion proteins under different pH have provided strong evidence to support acid-dependent conformational changes of viral fusion proteins [23, 45–51]. At low pH, Type I, II and III fusion proteins exposed an N-terminal fusion peptide or internal fusion loop for target membrane insertion, followed by fusion protein oligomerization, hemifusion and subsequent complete fusion between viral and host membrane [23, 26, 52, 53]. Although A26 protein is not a viral fusion protein, its acid-dependent conformational changes provide a new paradigm of membrane fusion activation, i.e. activation of viral membrane fusion by “de-repression” of a viral fusion suppressor. In the report by Gershon et al., the N-terminal region of A26 was proposed to interact with the N-terminal of G9 and the C-terminal of ATI based on crosslinking analysis [54]. However, the protein structures of G9, A16 and ATI remain unresolved. Without this structural information, it is difficult to evaluate the interfaces between these proteins and A26^1-397^. We speculate that a conformational change at low pH may affect the G9 and ATI interfaces on A26, resulting in disassociation of A26 from these two proteins, but the actual structures of A26 at low pH, as well as G9, A16 and ATI will be necessary to verify this possibility. A recent crystal structure study of the Gn glycoprotein of Rift Valley Fever Virus (RVFV) also revealed that Gn protein shields the hydrophobic fusion loops of the Gc fusion protein to prevent premature fusion of RVFV [55]. Despite a lack of similarity between the RVFV Gn protein and vaccinia A26 protein, conformational changes of viral regulatory proteins may represent a new mechanism to activate viral fusion proteins for membrane fusion.

## Materials and methods

### Cell culture and viruses

An African green monkey kidney cell line BSC40 (provided by Dr. Sridhar Pennathur), a human cervical adenocarcinoma cell line HeLa (Obtained from American Type Culture Collection (ATCC CCL-2) and murine L (ATCC CRL-2648) cells expressing GFP or RFP were cultured in Dulbecco’s modified Eagle’s medium (DMEM) supplemented with 10% fetal bovine serum (Invitrogen). The wild-type Western Reserve (WR) strain of vaccinia virus, the *A26L* deletion virus (WR-ΔA26), and a revertant WR-Flag-A26 virus (WR-A26) in which an N-terminal flag-tagged *A26L* ORF was reinserted back into the genome of WR-ΔA26 were all described previously [33]. Viruses were propagated in BSC40 cells and purified through a 36% sucrose cushion and a 25–40% sucrose gradient, followed by CsCl gradient centrifugation as previously described [56, 57]. Anti-A27 [5] and anti-D8 [7] antibodies were described previously. Anti-flag monoclonal antibody was purchased from Sigma Inc.

### Generation of recombinant vaccinia viruses expressing mutant A26 proteins

Two *A26L* N-terminal deletion ORFs, A26(aa 76–500) and A26(aa 321–500), were generated by PCR using the WR-*A26L* ORF as template. PCR fragments containing N-terminal flag sequences were individually cloned into pMJ601 plasmid so that each flag-*A26L* deletion ORF was expressed from a synthetic late promoter and flanked by the left and right arm viral tk sequences. In addition, we performed *in vitro* mutagenesis (QuickChange Lightning site-directed mutagenesis kit; Agilent Tech. Inc.) on the pMJ601-flag-*A26L* ORF plasmid to generate His-to-Arg mutations at His48 and His53 (A26-H2R), as well as three histidine residues at His48, His53 and His92 (A26-H3R). We also performed *in vitro* mutagenesis on the pMJ601-flag-*A26L* ORF plasmid to generate an A26 mutant protein (A26-H2-CAT) that contains K47D, R57D, R312D and H314R mutations to reduce cation-mediated repulsion at low pH via His48 and His53. All mutant *A26L* plasmids were sequenced to confirm accuracy. To generate N-terminal A26 deletion viruses and the other A26 mutant viruses, CV-1 cells (A kidney cell line of *Cercopithecus aethiops* purchased from ATCC CCL-70) were infected with WR-ΔA26 at an MOI of 1 PFU/cell, subsequently transfected with each plasmid and cultured for another 3 days. Lysates were subsequently harvested for recombinant virus isolation on BSC40 cells via three rounds of plaque purification in agar containing 150 μg/ml 5-bromo-4-chloro-3-indolyl-β-D-galactopyranoside (X-Gal), as described previously [58].

### Immunoblot analyses

CsCl purified vaccinia mature virions (1μg) were loaded on SDS-PAGE gels for immunoblot analyses as previously described [32]. Alternatively, BSC40 or HeLa cells were infected with various vaccinia viruses at an MOI of 5 PFU per cell for 1 h at 37°C and harvested at 24 hpi. Cell lysates were separated on SDS-PAGE gels and transferred onto nitrocellulose membranes for immunoblot analysis with anti-flag, anti-A27, and anti-D8 antibodies as previously described [32].

### Electron microscopy

EM analyses of virus-infected cells were performed as previously described but with minor modifications [59]. In brief, BSC40 and HeLa cells were infected with each virus at an MOI of 5 PFU per cell and harvested at 24 hpi. After embedding and sectioning, samples in thin sections were stained with 1% uranyl acetate in H_2_O and Reynold’s lead citrate solution. The samples were subsequently examined under a Tecnai G2 Spirit TWIN electron microscope (FEI Company, The Netherlands) and photographed using a CCD camera (4*3 model 832, Orius SC1000B).

### Determination of MV particle infectivity

EM analyses of purified vaccinia MV were performed as previously described [8]. In brief, CsCl-purified vaccinia MV samples were serially diluted, and then half of the samples were stained with 1% Sodium phosphotungstate (PTA) and loaded onto 400-mesh, 10 nm Formvar and 1 nm carbon-coated grids to count MV particle numbers under a Tecnai G2 Spirit TWIN electron microscope (FEI Company, The Netherlands). The other half of the serially-diluted MV samples was used to infect BSC40 cells for plaque assays to determine virus titers. Vaccinia MV infectivity is calculated as the Particle-to-PFU ratio = (Particle number per ml) / (PFU per ml) as previously described [8]. The infectivity assays for each virus were repeated three times and statistical analyses were performed using Student's *t* test in Prism (version 5) software (GraphPad). Statistical significance is represented as *, *P* value <0.05; ** <0.01; and *** <0.001.

### Vaccinia MV-triggered cell-cell fusion assay at neutral and acidic pH

Cell-cell fusion assays induced by vaccinia MV infections were performed as previously described [32]. In brief, L cells expressing EGFP or RFP were mixed at a 1:1 ratio and seeded in 96-well plates. The next day, cells were pretreated with 40 μg/ml cordycepin (Sigma) for 60 min and subsequently infected with each vaccinia virus at an MOI of 50 PFU per cell in triplicate. Cordycepin was present in the medium throughout the experiments. After infection at 37°C for 30 min, cells were treated with PBS at either pH 7.4 or pH 4.7 at 37°C for 3 min, washed with growth medium, further incubated at 37°C, and then photographed at 2 hpi using a Zeiss Axiovert fluorescence microscope. Five images for each virus were recorded and the % fusion was calculated using the image area of GFP^+^RFP^+^ double-fluorescent cells divided by that of single-fluorescent cells.

“Acid Fusion Index” was calculated to represent the acid-dependence of each A26 deletion protein, i.e., the occurrence of A26 protein conformational change. The index for each virus was obtained by dividing the percentage of cell fusion at low pH with that recorded for neutral pH. To quantify fusion activity of each virus as described above the fusion assays were repeated three times and statistical analyses were performed using Student's *t* test in Prism (version 5) software (GraphPad). Statistical significance is represented as *, *P* value <0.05, ** <0.01, and *** <0.001.

### Plasmid constructions and expression of recombinant TRX, TRX-A26 (1–91) and TRX-A26(1–91)^H48, 53R^ proteins for *in vitro* pH titration experiments using NMR and CD spectroscopy

An NdeI-EcoRI DNA fragment containing a thioredoxin (TRX)-hexahistidine-Xa cutting site was synthesized and cloned into the bacterial expression vector pET21a, resulting in pET21a-TRX. Subsequently, an EcoRI-XhoI fragment containing the A26L ORF encoding aa 1–91 was synthesized and cloned into pET21a-TRX, resulting in pET21a-TRX-A26(1–91) that expresses a bacterial fusion protein containing N-terminal TRX fused with A26 (aa 1–91) (Yao-Hong Biotechnology Inc., Taiwan). Next, *in vitro* mutagenesis was performed using pET21a-TRX-A26(1–91) plasmid as template to generate the pET-21a-TRX-A26(1–91)^H48, 53R^ mutant plasmid. To generate a control TRX expression plasmid, we inserted a stop codon immediately before the A26(1–91) sequence in pET-21a-TRX-A26(1–91) to generate a control plasmid that only expresses TRX protein (XL Site-Directed Mutagenesis Kit, Agilent Technologies, Santa Clara, CA).

Each plasmid was transformed into BL21(DE3) and recombinant proteins were expressed via 0.2 mM isopropyl 1-thio-β-d-galactopyranoside (IPTG) induction for 4 h, before harvesting for protein purification by nickel column chromatography as suggested by the manufacturer. All of the recombinant A26 proteins used in our NMR analyses contained the TRX fusion tag. To render recombinant TRX fusion proteins suitable for heteronuclear NMR studies, bacterial cultures were incubated at 37°C in M9 medium supplemented with [^15^N]ammonium chloride (1 g/liter) (Sigma-Aldrich Co., St. Louis, MO) to an absorbance at 600 nm of 0.8, induced for 4 h at 37°C with 0.2 mM IPTG, and harvested for protein purification through nickel-nitrilotriacetic acid affinity column chromatography. The bound recombinant TRX-fusion proteins were eluted with 0.3 M imidazole and dialyzed overnight against 0.1 M MES buffer pH 6.0 at 4°C before use.

For NMR measurement, all ^1^H-^15^N HSQC spectra were recorded at pH 6.0 or 8.0 and at 25°C on a Bruker Avance 600 MHz spectrometer equipped with a 5 mm QXI (^1^H/^13^C/^15^N) *z-*axis gradient probe. For ^15^N-labeled proteins (0.8–1.0 mM), Shigemi NMR tubes (5 mm outer diameter) were used. The pH values were measured at 25°C with a Suntex TS-100 pH meter. Proteins started to aggregate under acidic conditions (pH<6.0). All spectra were processed using Topspin version 3.2 (Bruker, Karlsruhe, Germany).

For CD measurements, the TRX tag was removed from the fusion A26 proteins using ~2 μg Factor Xa (Sigma Aldrich) in 20 mM Tris·HCl, pH 6.5 with 50 mM NaCl and 1 mM CaCl_2_ at 4°C. After cleaving, purification was carried out using Ni-NTA resin to separate the cleaved His-TRX tag from the A26 protein samples. The CD spectra were recorded on a Jasco J-815 spectrometer equipped with a water bath for temperature control. All CD spectra were collected at 25°C using a quartz cuvette with a 1 mm path length and a protein concentration of 15.4 μM. The step size was 0.2 nm with a 1.0 nm bandwidth at a scan speed of 50 nm/min. Each spectrum represents the average of three measurements. All spectra were collected in 20 mM potassium phosphate buffer with background buffer correction.

### Preliminary screening of suitable recombinant A26 expression constructs

Four *A26L* DNA fragments coding for (1) full-length A26 protein, (2) residues 1–420 (A26(1–420)), (3) residues 1–420 with Cys43/Cys342 mutations (A26(1–420)C43C342A), and (4) residues 1–110 (A261-110) were each ligated into pET-16 vector (Novagen). Each construct was transformed into *Escherichia coli* BL21(DE3). After induction with 1 mM IPTG, each recombinant protein was expressed at 16°C for 16 hours. Each soluble A26 protein was purified by immobilized metal-ion chromatography with a Ni-NTA column (GE Healthcare).

### Limited trypsin digestion of A26(1–420)C43C342A

Since we failed to crystallize A26(1–420) and A26(1–420)C43C342A, we used a limited trypsin digestion assay to determine the core structure of A26 protein. Briefly, 500 μg purified A26(1–420)C43C342A was incubated in 30 μl reaction buffer (30 mM Tris pH 8.0, 100 mM NaCl, 1 mM dithiothreitol and 5% glycerol) either alone or in the presence of trypsin at a 1:500 ratio (w/w; trypsin:protein). Digestion was carried out at 4°C for 16 hours. The reaction products were analyzed by 12% Bis-tris SDS-PAGE and stained with coomassie blue. The bands containing A26 fragments were excised from the SDS PAGE gel and then subjected to in-gel digestion with trypsin. The digested peptide mixtures were then subjected to a NanoLC−nanoESI-MS/MS analysis. MS data were analyzed using the MASCOT server (http://www.matrixscience.com/search_form_select.html).

### Protein purification of A26(1–397) and Selenomethionine-labeled A26(1–397)

Based on the above-described digestion analysis, we decided to use A26(1–397) for further crystallization experiments using the (smt3/Ulp) system provided by Dr. C. D. Lima for recombinant protein expression and purification as previously described [60]. The pET-His_10_-SUMO-A26^1-397^ DNA construct (codon-optimized in bacteria) was transformed into the *E*. *coli* BL21(DE3) strain (Novagen), and the cells were cultured in LB broth containing 50·μg/ml Kanamycin until the optical density at 600 nm (OD_600_) reached 0.6–0.8 at 37°C. A final concentration of 0.1 mM isopropyl-®-thiogalactopyranoside (IPTG) was added to induce expression and cultured overnight at 17°C for 20 h until the OD_600_ reached 1.20. Bacterial pellets were harvested by centrifugation at 6,000×*g* for 30 min at 4°C and then disrupted by sonication in lysis buffer [20 mM Tris pH 8.0, 20 mM imidazole, 0.5 M NaCl, 10% (w/v) glycerol, 1 mM PMSF, 1 mg/ml lysozyme, 0.1 mg/ml DNase I, 1 mM benzamidine (Novagen), and EDTA-free protease inhibitor cocktail (Roche)]. SUMO-A26(1–397) protein was loaded onto a Ni-NTA affinity chromatography column (GE Healthcare), washed first with 40 volumes of binding buffer 1 [20 mM Tris pH 8.0, 20 mM imidazole, 0.5 M NaCl, 10% (w/v) glycerol], then with 40 volumes of binding buffer 2 [20 mM Tris pH 8.0, 100 mM imidazole, 0.5 M NaCl, 10% (w/v) glycerol], before elution with a linear gradient of up to 100% (v/v) elution buffer [20 mM Tris pH 8.0, 0.5 M imidazole, 0.5 M NaCl, 10% (w/v) glycerol]. The eluted SUMO-A26(1–397) protein was dialyzed three times against 7.5 liters of buffer [20 mM Tris pH 8.0, 0.3 M NaCl, 10% (w/v) glycerol] and then subjected to Ubiquitin-like-specific protease 1 (Ulp1) treatment to remove the histidine-tagged SUMO fusion protein. The histidine-tagged SUMO fusion protein was cleaved using Ulp1 at a ratio of 1:500 (w/w; Ulp1:protein) that was later removed with a Ni-NTA affinity chromatography column (GE Healthcare). The untagged A26(1–397) proteins were purified using another HiPrep Q FF 16/10 column (GE Healthcare). These untagged A26(1–397) proteins were purified through a HiPrep Q FF 16/10 column (GE Healthcare), the column was washed with 10 volumes of Q binding buffer [20 mM Tris pH 8.0, 1 mM DTT, 50 mM NaCl], and eluted with a linear gradient of up to 100% (v/v) elution buffer [20 mM Tris pH 8.0, 1 mM DTT, 0.5 M NaCl]. The A26(1–397) proteins were stored in a buffer containing 20 mM Tris pH 8.0, 1 mM DTT, and 100 mM NaCl at 4°C. We used a 12% SDS PAGE gel to confirm the purity of A26(1–397) proteins as being above 99%. Selenomethionine-labeled A26(1–397) protein (SeMet A26(1–397)) was labeled using a SelenoMethionine medium complete kit (Molecular Dimensions) and purified according to the same procedures.

### Crystallization, data collection and structure determination of SeMet A26(1–397) recombinant protein

Since A26 shows no sequence homology to any reported protein structure, we produced SeMet A26(1–397) for X-ray analysis. SeMet A26(1–397) was crystallized by the sitting drop method, in which 2 μl of the purified protein (15 mg/ml) was mixed with 2 μl of a reservoir containing 0.2 M sodium acetate trihydrate, 0.1 M Tris pH 8.5, 30% w/v PEG 4000, and equilibrated with 200 μl of the reservoir at 25°C. For X-ray data collection, 15% ethylene glycol was used as a cryoprotectant. A single-wavelength anomalous dispersion (SAD) X-ray diffraction dataset was collected from Taiwan Photon Source (TPS) beamline 05A at the National Synchrotron Radiation Research Center (NSRRC) in Hsinchu, Taiwan. The X-ray data were processed by using HKL2000 [61].

The space group of the SeMet A26(1–397) crystal is P2_1_, with unit cell dimensions of *a* = 45.38 Å, *b* = 80.72 Å, *c* = 53.81 Å and β = 113.8° (Table 2). The initial electron density map of SeMet A26(1–397) was calculated by using the peak dataset collected at wavelength 0.97907 Å and the program Shelix CDE [62]. The program BUCCANEER [63] was then used to produce the initial model. Only one SeMet A26(1–397) monomer was found in each asymmetric unit. We used the programs COOT [64] and Refmac [65] for model refinement. Finally, residues 17–364 of SeMet A26(1–397) were successfully built. In addition, resdiues 382–385 of SeMet A26(1–397) (Thr-Pro-Ile-Pro) were also built as a separate fragment. Data collection and refinement statistics are shown in Table 2. The completeness of 95.2% is actually for the outermost shell and this is now clarified in **Table 2**. We also analyzed our SeMET A26 structure by MolProbity (http://molprobity.biochem.duke.edu/) [38]. The resulting MolProbity score and Clashscore are 1.10 and 2.68, confirming the good quality of this structure. We used the CCP4 package [66], Chimera program [67], Areaimol [68], PROPKA3 [69] and Discovery studio [42] for structural analyses and to generate figures. The corresponding positions of the regions of constructs used in this study are highlighted in S8 Fig. Discovery studio was also used to build a A26^1-397^ model at pH4.7 [A26^1-397^ model (pH4.7)].

Then, the A26 ^1-397^model (pH4.7) was subjected to molecular dynamic simulations following the Standard Dynamics Cascade protocol. The dynamic simulations were performed for a production time of 1,000 ps using default parameters. The final conformation of A26 ^1–397^ model (pH4.7) was then used in subsequent analysis (e.g. calculate Surface electrostatic potential and solvent accessibility, etc).

### Large plaque revertant virus isolation

WR-A26-H2R, WR-A26-H3R and WR-A26-H2-CAT mutant viruses formed tiny plaques of HeLa cells. Revertant (Rev) viruses displaying a large plaque phenotype were spontaneously derived at an initial rate of ~0.1% during early expansion of WR-A26-H2R, WR-A26-H3R and WR-A26-H2-CAT viruses. We independently isolated three revertant viruses (Rev1-3) from WR-A26-H2R, four revertant viruses (Rev1-4) from WR-A26-H3R, and four revertant viruses (Rev1-4) from WR-A26-H2-CAT, respectively. All of the revertant viruses were subsequently purified to 100% purity. Viral genomic DNA was purified from all the revertant viruses but only WR-A26-H2R-Rev1, WR-A26-H3R-Rev1 and WR-A26-H2-CAT-Rev1 viruses were sent for whole genome sequencing. Viral genomic DNA of all other revertant viruses were used in PCR amplification and sequencing to determine the location of the second-site mutations in *A26L* ORF.

### Viral genome sequencing and data analyses

Vaccinia viral genomic DNA was extracted as previously described [70]. All genomic DNA was quantified by Qubit ds DNA BR assays using a Qubit 3.0 fluorometer (Life Technologies, Carlsbad, US-CA). Genomic DNA (2 μg) was sheared to an average length of 550 bp, end-repaired and A-tailed, and then ligated with indexed adapters for PCR-free library preparation [71] based on the manufacturer’s protocols (Illumina TruSeq DNA PCR-Free Library Preparation kit protocol 15036187 Rev.B) (Illumina Inc., San Diego, CA, USA). The quality and size distribution of the genomic libraries was verified using an Agilent DNA High Sensitivity kit (5067–4626) and Agilent 2100 Bioanalyzer. Viral genomic sequencing was performed using an Illumina Miseq 2x300 cycle or NextSeq500 2x150 cycle paired-end run at the Genomics Core facility of the Institute of Molecular Biology, Academia Sinica.

The sequence data were performed using CLC Genomics Workbench 11.0.1 (Qiagen, Aarhus, Denmark) for raw sequencing trimming, sequence mapping, and variant detection. Raw sequencing reads were trimmed by removing adapter sequences, low-quality sequences (Phred quality score of less than Q20) and sequencing fragments of shorter than 30 nucleotides. Sequencing reads were mapped to the human genome (GRCh38, from ftp.ensembl.org/pub/release-82/fasta/homo_sapiens/dna/) with the following parameters: mismatches cost = 2, insertion cost = 3, deletion cost = 3, minimum fraction length = 0.8, minimum fraction similarity = 0.8. All host genome sequences that met the above parameters were removed, as were duplicate reads, before mapping the remaining paired-end reads to the vaccinia virus WR genome (GenBank NC_006998) [72] with much more stringent parameters (mismatches cost = 2, insertion cost = 3, deletion cost = 3, minimum fraction length = 0.9, minimum fraction similarity = 0.9). We used the Basic Variant Detection tool in CLC Genomics Workbench 11.0.1 to call single nucleotide polymorphisms (SNPs) and insertions/deletions (InDels) with customized parameters to identify mutation positions: (1) minimum frequency of 10% and minimum coverage 10 reads; and (2) minimum quality of SNPs/InDels should be larger than Q25 and the neighborhood quality (upstream/downstream five bases) should be larger than Q20.

We also used the paired-end reads after removing host genome sequences to generate mutant and revertant viral genomes by de novo genome assembly but excluding the terminal repeat sequences of vaccinia virus. Using the alignment program MAFFT version 7[73, 74], we aligned all viral genome sequences with the reference WR strain (GenBank NC_006998) to identify the second-site mutations in revertant viruses. The multiple alignments of viral genomic sequences of WR-A26, WR-A26-H2R-Rev1, WR-A26-H3R-Rev1 and WR-H2-CAT-Rev1 are included in Supplemental S1 Appendix.

### Accession number

Coordinates and structure factors of A26 have been deposited in the Protein Data Bank with 6A9S accession number (PDB ID: 6A9S).

## Supporting information

S1 Fig Analysis of the surface electrostatic potential of A26^1-397^. (A) The surface electrostatic potential of A26^1-397^ is presented in four different views. (B) A comparison of the structures and surface charges between A26^1-397^ crystal at neutral pH (left) and our computed A26^1-397^ (pH 4.7) model (right, generated in Discovery Studio [42] and analyzed by the Standard Dynamics Cascade protocol).
(TIF)

S2 Fig Analysis of the solvent accessible surface area (SA) of A26^1-397^. The SA of A26^1-397^ and the A26^1-397^ (pH 4.7) model were calculated using Discovery Studio. The 2 helix of A26^1-397^ is colored in yellow. Residues exhibiting increased or reduced SA (>15%) in the A26^1-397^ (pH 4.7) model are colored in cyan or blue, respectively.
(TIF)

S3 Fig Comparison of plaque sizes among control WR-A26, small-plaque mutant WR-A26-H2R, WR-A26-H3R, and WR-A26-H2-CAT viruses, and their respective large-plaque revertants. (TIF)

S4 Fig Immunoblot analyses of A26 protein in lysates infected with WR-A26, small-plaque mutant WR-A26-H2R, WR-A26-H3R, and WR-A26-H2-CAT viruses, and their respective large-plaque revertants. D8 and A27 proteins were used as lysate controls.
(TIF)

S5 Fig Mutations identified by PCR sequencing of the A26 ORF obtained from revertant viruses derived from the WR-A26-H2R, WR-A26-H3R and WR-A26-H2-CAT mutant viruses. (TIF)

S6 Fig Schematic representations of second-site mutations in the revertant viruses described in S4 Fig. Each revertant A26 protein contains a second site mutation and becomes truncated, with a N-terminal A26 fragment (a.a. number on white box) fused with aberrant aa (a.a. number on dotted light blue box) due to frame-shift and premature termination.
(TIF)

S7 Fig Cell-cell fusion assay mediated by various viruses at neutral (pH 7.4) or low pH (pH 4.7). All the revertant viruses do not require acidic pH to trigger cell-cell fusion, similar to WR-ΔA26 virus.
(TIF)

S8 Fig Structural overview of full-length and various truncated forms of A26 constructs used in this study. (TIF)

S1 Table The predicted pKa of relevant residues of A^261-397^ in this study. (PDF)

S2 Table The A^261-397^ residues with SA changes (>15%). (PDF)

S1 Appendix Multiple viral genome sequence alignments among WR-A26, WR-A26-H2R-Rev1, WR-A26-H3R-Rev1 and WR-H2-CAT-Rev1. (ZIP)
