# Supplementary figures and images for: Vaccinia viral A26 protein is a fusion suppressor of mature virus and triggers membrane fusion through conformational change at low pH
Source: PLoS Pathog. 2019 Jun 20;15(6):e1007826. doi: 10.1371/journal.ppat.1007826 (PMC6605681; doi:10.1371/journal.ppat.1007826)

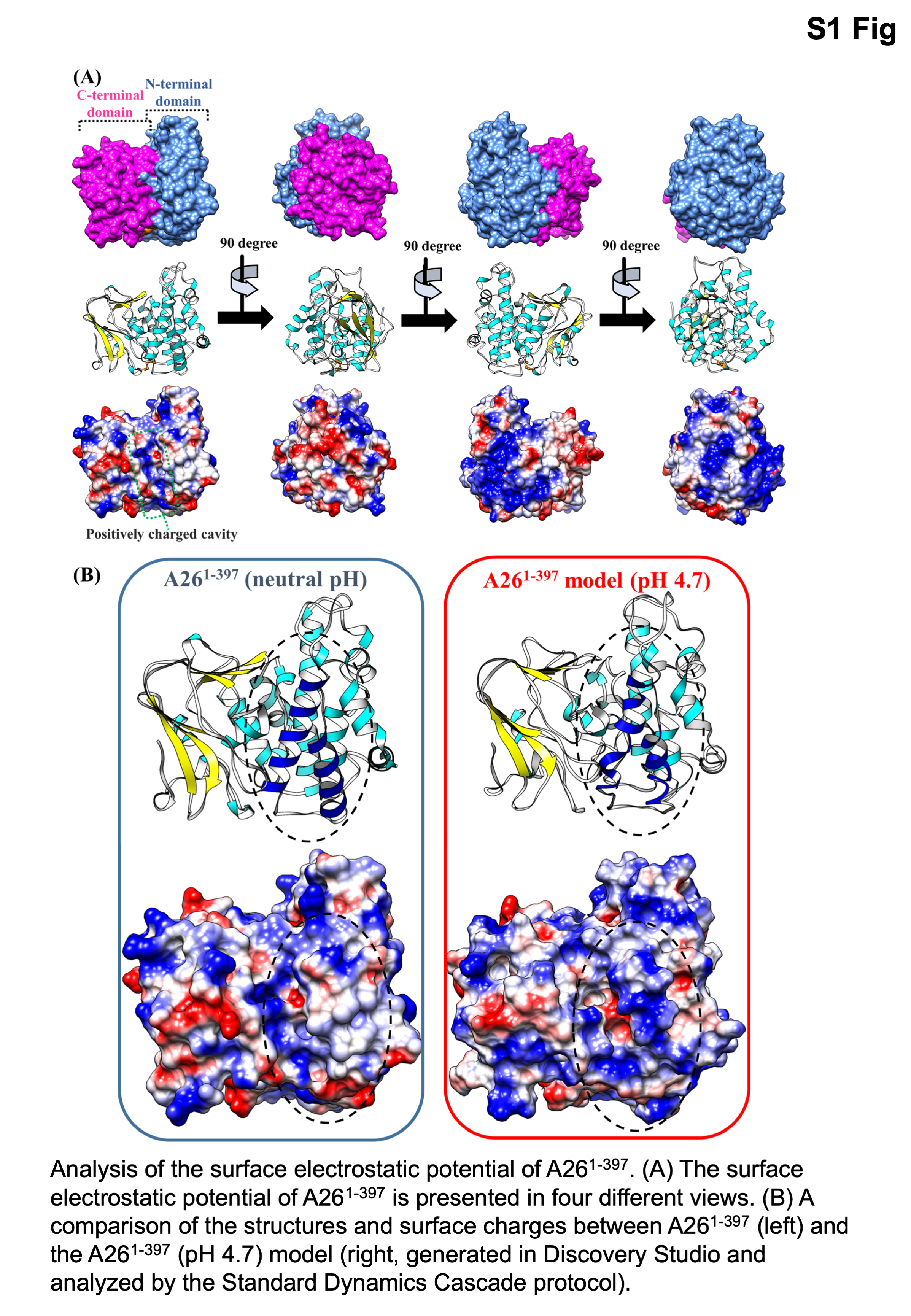

Supplement: S1 Fig — (A) The surface electrostatic potential of A261-397 is presented in four different views. (B) A comparison of the structures and surface charges between A261-397 crystal at neutral pH (left) and our computed A261-397 (pH 4.7) model (right, generated in Discovery Studio [42] and analyzed by the Standard Dynamics Cascade protocol). (TIF) [file ppat.1007826.s001.tif]

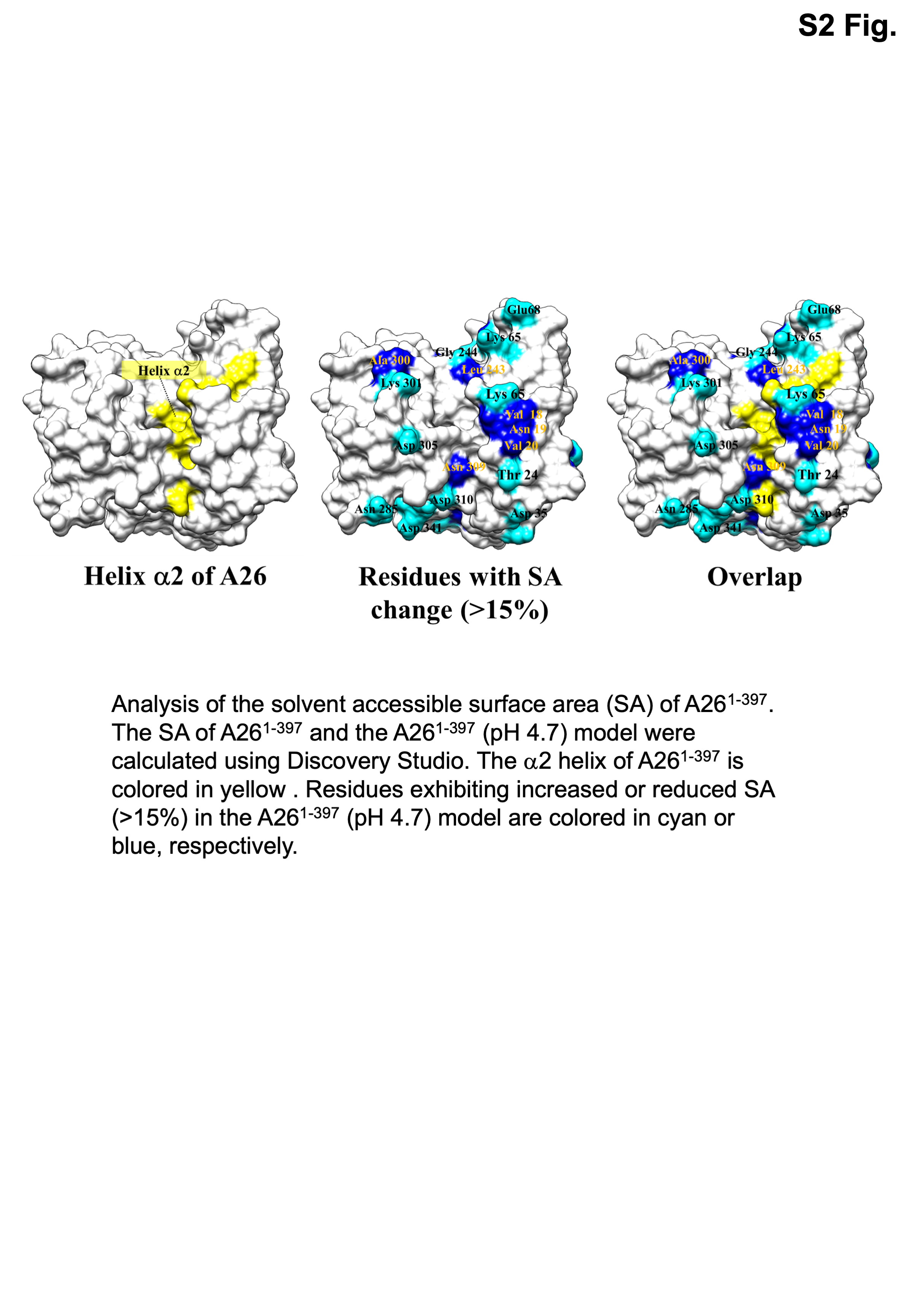

Supplement: S2 Fig — The SA of A261-397 and the A261-397 (pH 4.7) model were calculated using Discovery Studio. The 2 helix of A261-397 is colored in yellow. Residues exhibiting increased or reduced SA (>15%) in the A261-397 (pH 4.7) model are colored in cyan or blue, respectively. (TIF) [file ppat.1007826.s002.tif]

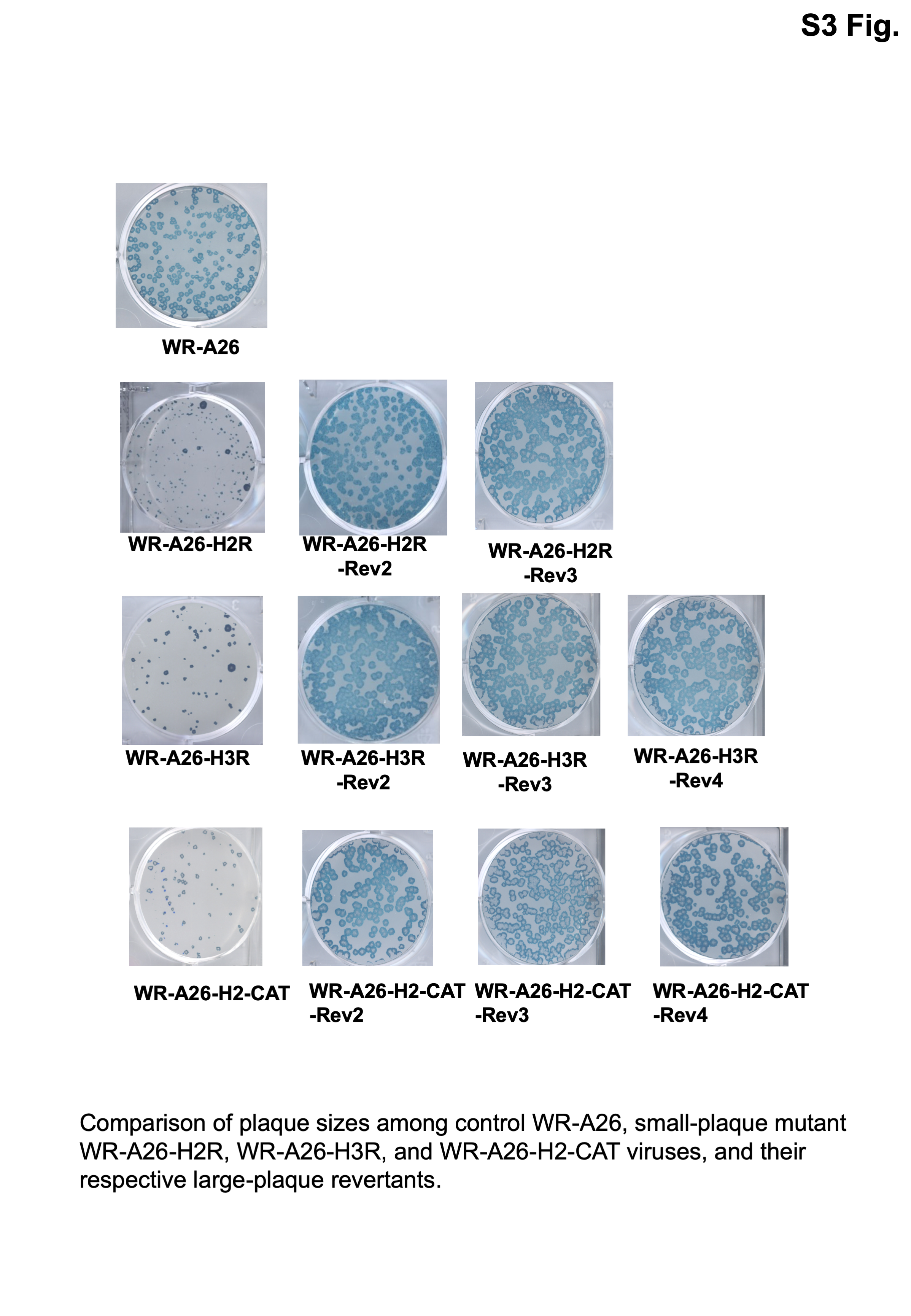

Supplement: S3 Fig — (TIF) [file ppat.1007826.s003.tif]

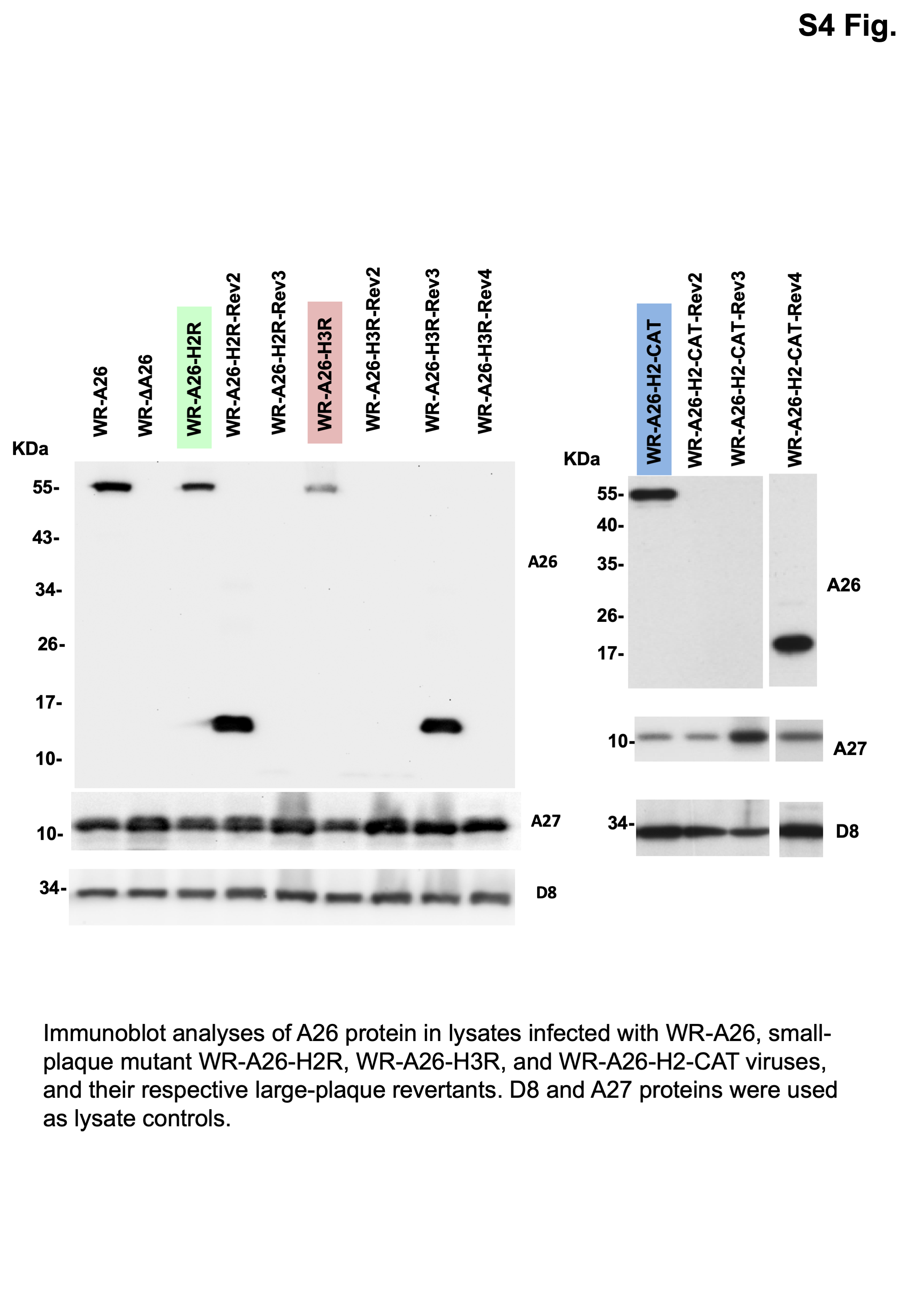

Supplement: S4 Fig — D8 and A27 proteins were used as lysate controls. (TIF) [file ppat.1007826.s004.tif]

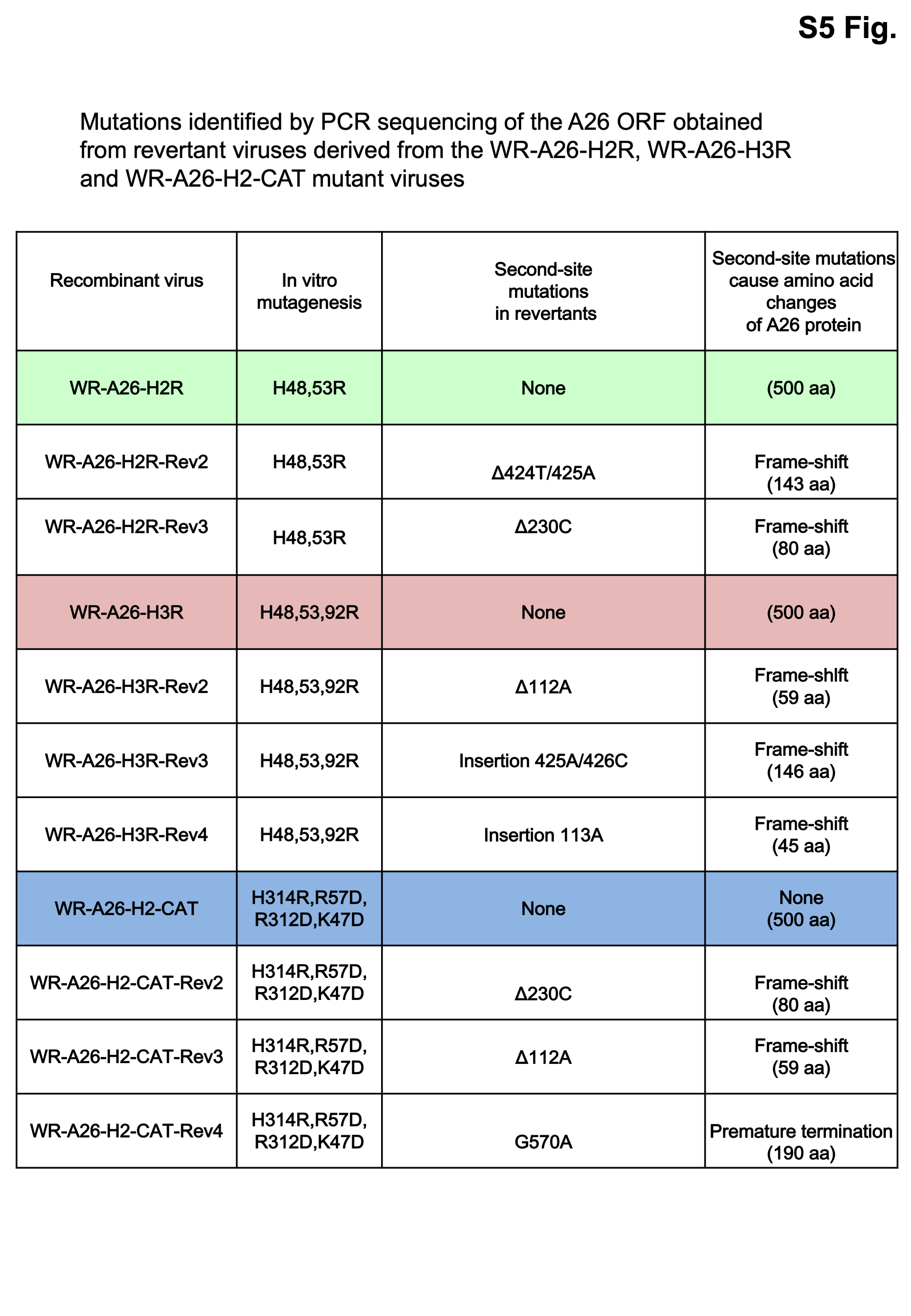

Supplement: S5 Fig — (TIF) [file ppat.1007826.s005.tif]

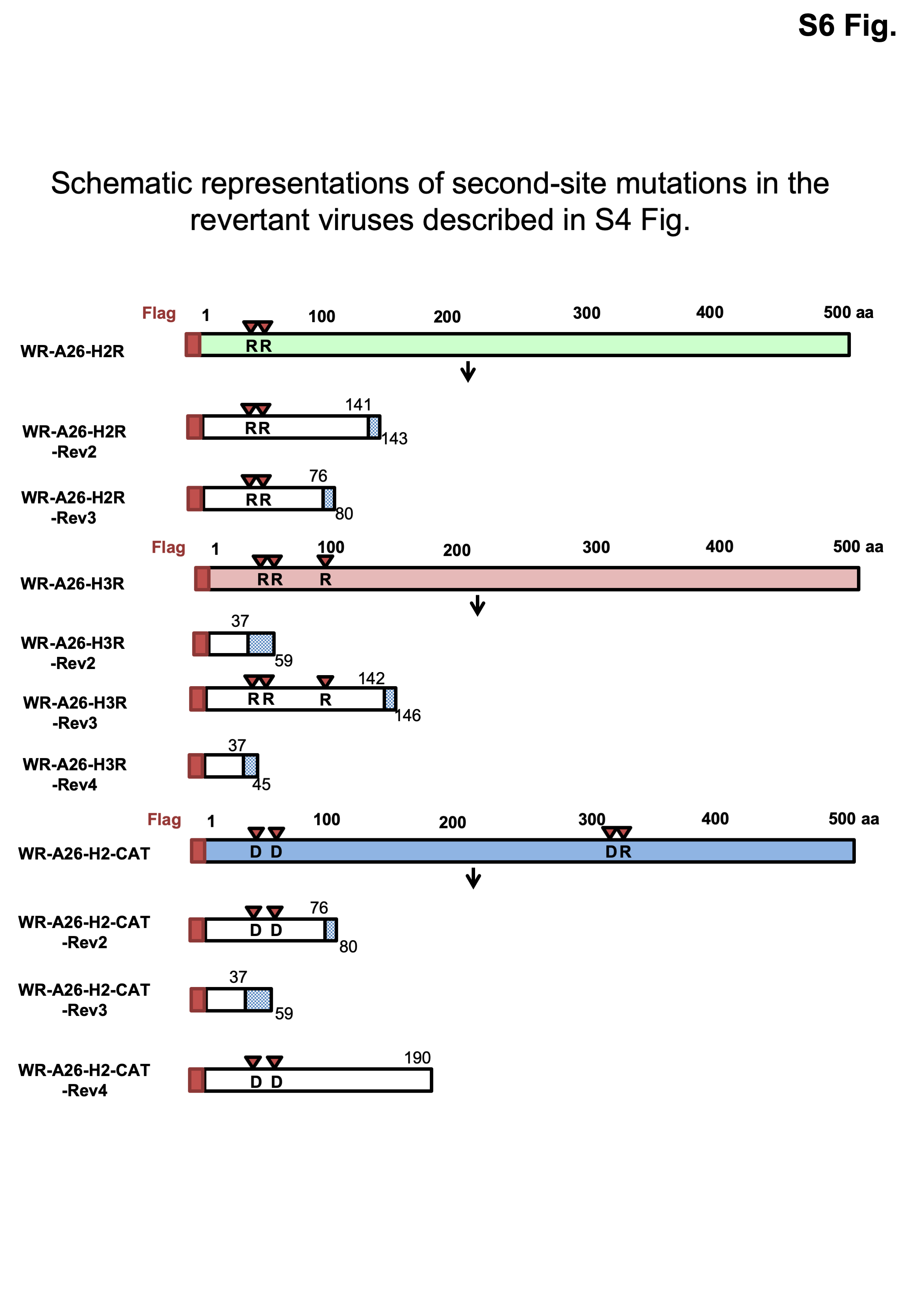

Supplement: S6 Fig — Each revertant A26 protein contains a second site mutation and becomes truncated, with a N-terminal A26 fragment (a.a. number on white box) fused with aberrant aa (a.a. number on dotted light blue box) due to frame-shift and premature termination. (TIF) [file ppat.1007826.s006.tif]

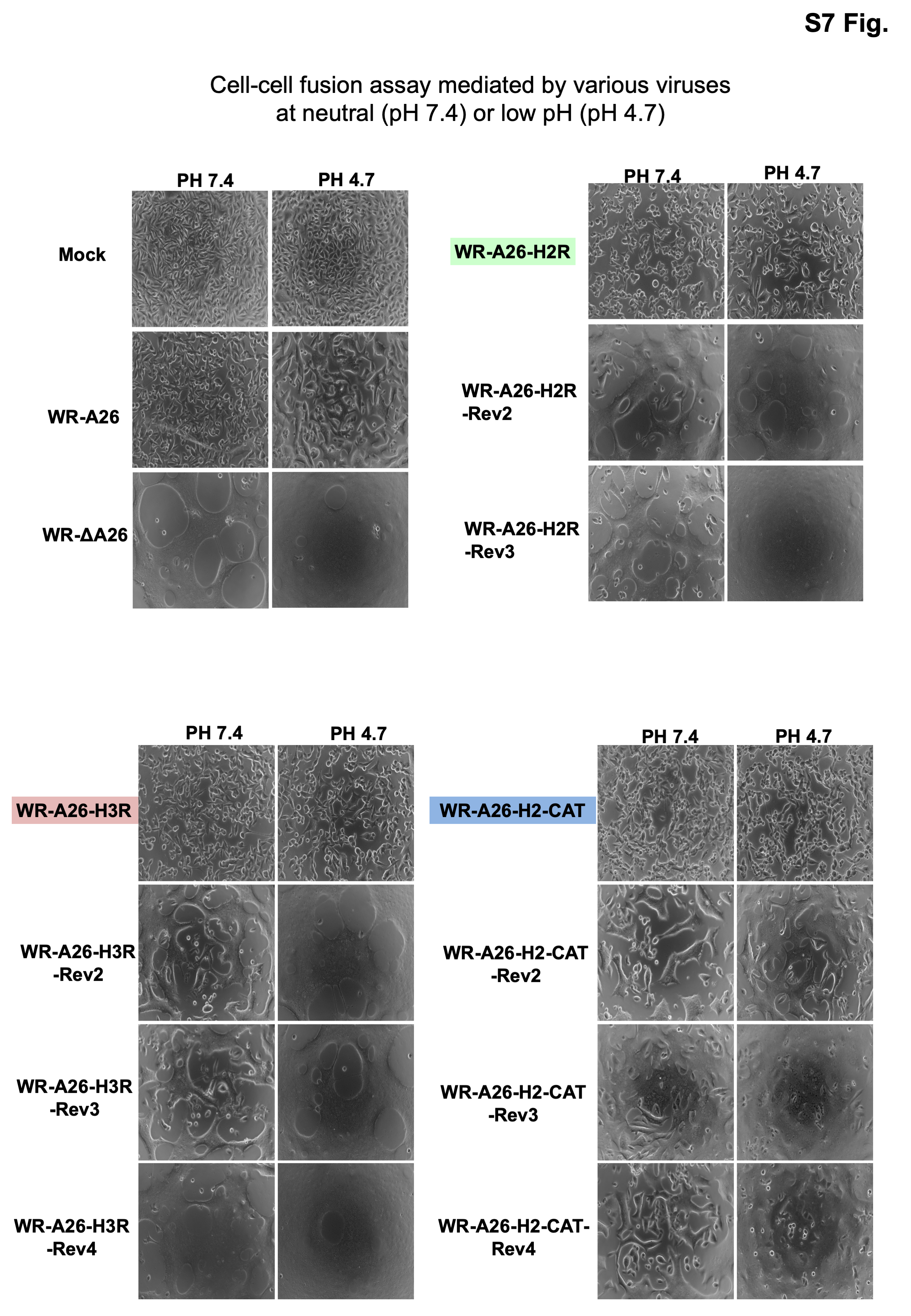

Supplement: S7 Fig — All the revertant viruses do not require acidic pH to trigger cell-cell fusion, similar to WR-ΔA26 virus. (TIF) [file ppat.1007826.s007.tif]

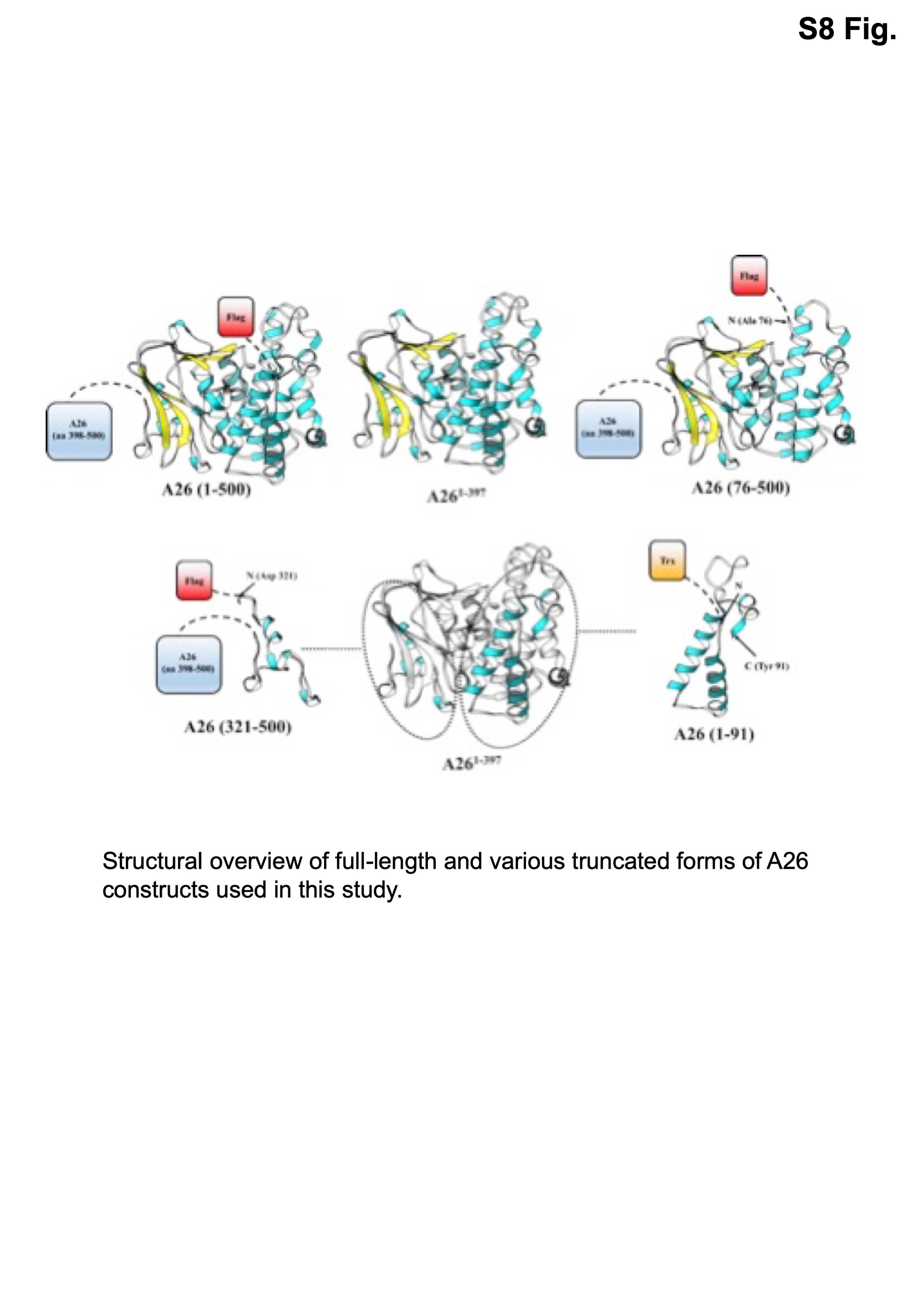

Supplement: S8 Fig — (TIF) [file ppat.1007826.s008.tif]
