# Supplementary material for: Vaccinia viral A26 protein is a fusion suppressor of mature virus and triggers membrane fusion through conformational change at low pH
Source: PLoS Pathog. 2019 Jun 20;15(6):e1007826. doi: 10.1371/journal.ppat.1007826 (PMC6605681; doi:10.1371/journal.ppat.1007826)
Supplement: S1 Table — (PDF) [file ppat.1007826.s009.pdf]

Table S1. The predicted pKa of relevant residues of $A^{261-397}$ in this study.

Residue	Predicted pKa*	His-Cation and Ani-Ani pairs
**The residues on alpha-helix 2 region that involve in His-Cation and Ani-Ani pairs**		
Lys 47	10.86	Lys 47-His 48
His 48	4.9	Lys 47-His 48
His 53	2.15	His 53-Arg 57, His 53-His 314
Glu 54	3.36	Glu 54-Asp 58
Arg 57	14.81	His 53-Arg 57
Asp 58	4.53	Glu 54-Asp 58
Asp 308	2.96	Asp 308-Asp 310
Asp 310	3.97	Asp 308-Asp 310
Arg 312	10.90	Arg 312- His 314
His 314	-0.11	His 53-His 314, Arg 312- His 314
**Other residues that involve in His-Cation and Ani-Ani pairs**		
Asp 339	4.25	Asp 339-Glu 341
Glu 341	4.85	Asp 339-Glu 341
Arg 317	13.27	Arg 317-His 357
Arg 333	13.72	Arg 333-His 357
His 357	6.80	Arg 317-His 357, Arg 333-His 357

*The pKa is predicted by using PROPKA3 (**68**).
