# Supplementary material for: Vaccinia viral A26 protein is a fusion suppressor of mature virus and triggers membrane fusion through conformational change at low pH
Source: PLoS Pathog. 2019 Jun 20;15(6):e1007826. doi: 10.1371/journal.ppat.1007826 (PMC6605681; doi:10.1371/journal.ppat.1007826)
Supplement: S2 Table — (PDF) [file ppat.1007826.s010.pdf]

S2 Table. The A$^{261-397}$ residues with SA changes (>15 %).

**Residue**	**SA (%) A26$^{1-397}$**	**SA (%) A26$^{1-397}$ model (pH 4.7)**	**SA different (%)**
Asp 305	92.138	48.033	44.105
Gly 137	67.382	32.347	35.035
Asn 192	66.556	31.642	34.914
Lys 65	74.864	40.375	34.489
Asn 285	105.433	73.539	31.894
Asp 35	89.412	58.009	31.403
Glu 68	83.112	52.322	30.79
Asp 204	72.723	42.447	30.276
Asp 310	60.528	31.923	28.605
Asp 17	84.876	59.563	25.313
Gly 244	92.713	68.794	23.919
Ser 144	95.497	71.714	23.783
Asn 340	64.027	43.392	20.635
Glu 156	55.263	35.703	19.56
Thr 24	45.785	26.697	19.088
Asp 275	25.977	7.261	18.716
Lys 301	70.143	51.455	18.688
Lys 163	26.047	7.84	18.207
Gly 75	63.211	45.146	18.065
Asn 287	81.554	63.932	17.622
Ile 319	47.628	30.642	16.986
Asp 339	29.799	12.923	16.876
Ser 322	92.753	76.684	16.069
Asn 41	44.288	28.348	15.94
Asp 31	57.626	41.966	15.66
Lys 108	75.714	60.329	15.385
Ile 328	40.84	25.604	15.236
Gly 101	14.595	29.616	-15.021
Val 20	54.019	70.375	-16.356
Asn 104	21.407	38.395	-16.988
Ile 299	68.717	86.088	-17.371
Ala 300	1.476	19.505	-18.029
Ile 227	19.72	38.106	-18.386
Asn 309	29.041	49.372	-20.331

Pro 139	16.412	38.871	-22.459
Thr 353	39.509	62.404	-22.895
Thr 42	14.932	37.864	-22.932
Gly 356	26.835	50.364	-23.529
Cys 43	6.243	29.945	-23.702
Asp 201	33.194	57.916	-24.722
Phe 153	37.958	65.397	-27.439
Ser 246	14.872	43.434	-28.562
Asn 19	29.879	60.561	-30.682
Leu 243	41.719	83.742	-42.023
Val 18	36.343	83.524	-47.181
Leu 74	24.299	97.81	-73.511
