# Supplementary material for: Vaccinia viral A26 protein is a fusion suppressor of mature virus and triggers membrane fusion through conformational change at low pH
Source: PLoS Pathog. 2019 Jun 20;15(6):e1007826. doi: 10.1371/journal.ppat.1007826 (PMC6605681; doi:10.1371/journal.ppat.1007826)
Supplement: S1 Appendix — (ZIP) [file ppat.1007826.s011.zip › S1 Appendix.pdf]

Seq1=WR-A26
Seq2=WR-A26-H2R-Rev1
Seq3=WR-A26-H3R-Rev1
Seq4=WR-A26-H2-CAT-Rev1

```
                  *        20         *        40         *        60         *        80         *       100
Seq1 : attttatttagtgtctagaaaaaaatgtgtgacccatgactgtaggaaactctagagtgtaagaaagatcgatcgctttatagagaccatcagaaagagg :   100
Seq2 : attttatttagtgtctagaaaaaaatgtgtgacccatgactgtaggaaactctagagtgtaagaaagatcgatcgctttatagagaccatcagaaagagg :   100
Seq3 : attttatttagtgtctagaaaaaaatgtgtgacccatgactgtaggaaactctagagtgtaagaaagatcgatcgctttatagagaccatcagaaagagg :   100
Seq4 : attttatttagtgtctagaaaaaaatgtgtgacccatgactgtaggaaactctagagtgtaagaaagatcgatcgctttatagagaccatcagaaagagg :   100

                  *       120         *       140         *       160         *       180         *       200
Seq1 : tttaatattttgtgagaccatcgaagagagaaagagataaaactttttttacgactccatcagaaagaggtttaatattttgtgagaccatcgaagaga :   200
Seq2 : tttaatattttgtgagaccatcgaagagagaaagagataaaactttttttacgactccatcagaaagaggtttaatattttgtgagaccatcgaagaga :   200
Seq3 : tttaatattttgtgagaccatcgaagagagaaagagataaaactttttttacgactccatcagaaagaggtttaatattttgtgagaccatcgaagaga :   200
Seq4 : tttaatattttgtgagaccatcgaagagagaaagagataaaactttttttacgactccatcagaaagaggtttaatattttgtgagaccatcgaagaga :   200

                  *       220         *       240         *       260         *       280         *       300
Seq1 : gaaagagataaaactttttttacgactccatcagaaagaggtttaatattttgtgagaccatcgaaggagaaagagataaaactttttttacgactccatc :   300
Seq2 : gaaagagataaaactttttttacgactccatcagaaagaggtttaatattttgtgagaccatcgaaggagaaagagataaaactttttttacgactccatc :   300
Seq3 : gaaagagataaaactttttttacgactccatcagaaagaggtttaatattttgtgagaccatcgaaggagaaagagataaaactttttttacgactccatc :   300
Seq4 : gaaagagataaaactttttttacgactccatcagaaagaggtttaatattttgtgagaccatcgaaggagaaagagataaaactttttttacgactccatc :   300

                  *       320         *       340         *       360         *       380         *       400
Seq1 : agaaagaggtttaatattttgtgagaccatcgaaggagaaagagataaaactttttttacgactccatcagaaagaggtttaatattttgtgagaccat :   400
Seq2 : agaaagaggtttaatattttgtgagaccatcgaaggagaaagagataaaactttttttacgactccatcagaaagaggtttaatattttgtgagaccat :   400
Seq3 : agaaagaggtttaatattttgtgagaccatcgaaggagaaagagataaaactttttttacgactccatcagaaagaggtttaatattttgtgagaccat :   400
Seq4 : agaaagaggtttaatattttgtgagaccatcgaaggagaaagagataaaactttttttacgactccatcagaaagaggtttaatattttgtgagaccat :   400

                  *       420         *       440         *       460         *       480         *       500
Seq1 : cgaagagagaaagagataaaactttttttacgactccatcagaaagaggtttaatattttgtgagaccatcgaagagagaaagagataaaactttttttac :   500
Seq2 : cgaagagagaaagagataaaactttttttacgactccatcagaaagaggtttaatattttgtgagaccatcgaagagagaaagagataaaactttttttac :   500
Seq3 : cgaagagagaaagagataaaactttttttacgactccatcagaaagaggtttaatattttgtgagaccatcgaagagagaaagagataaaactttttttac :   500
Seq4 : cgaagagagaaagagataaaactttttttacgactccatcagaaagaggtttaatattttgtgagaccatcgaagagagaaagagataaaactttttttac :   500

                  *       520         *       540         *       560         *       580         *       600
Seq1 : gactccatcagaaagaggtttaatattttgtgagaccatcgaaggagaaagagataaaactttttttacgactccatcagaaagaggtttaatattttg :   600
Seq2 : gactccatcagaaagaggtttaatattttgtgagaccatcgaaggagaaagagataaaactttttttacgactccatcagaaagaggtttaatattttg :   600
Seq3 : gactccatcagaaagaggtttaatattttgtgagaccatcgaaggagaaagagataaaactttttttacgactccatcagaaagaggtttaatattttg :   600
Seq4 : gactccatcagaaagaggtttaatattttgtgagaccatcgaaggagaaagagataaaactttttttacgactccatcagaaagaggtttaatattttg :   600

                  *       620         *       640         *       660         *       680         *       700
Seq1 : tgagaccatcgaagagagaaagagataaaactttttttacgactccatcagaaagaggtttaatattttgtgagaccatcgaagagagaaagagataaaa :   700
Seq2 : tgagaccatcgaagagagaaagagataaaactttttttacgactccatcagaaagaggtttaatattttgtgagaccatcgaagagagaaagagataaaa :   700
Seq3 : tgagaccatcgaagagagaaagagataaaactttttttacgactccatcagaaagaggtttaatattttgtgagaccatcgaagagagaaagagataaaa :   700
Seq4 : tgagaccatcgaagagagaaagagataaaactttttttacgactccatcagaaagaggtttaatattttgtgagaccatcgaagagagaaagagataaaa :   700
```

```
                 *        720         *        740         *        760         *        780         *        800
Seq1 : cttttttacgactccatcagaaagaggtttaatatttttgtgagaccatcgaagagagaaagagataaaacttttttacgactccatcagaaagaggttt :    800
Seq2 : cttttttacgactccatcagaaagaggtttaatatttttgtgagaccatcgaagagagaaagagataaaacttttttacgactccatcagaaagaggttt :    800
Seq3 : cttttttacgactccatcagaaagaggtttaatatttttgtgagaccatcgaagagagaaagagataaaacttttttacgactccatcagaaagaggttt :    800
Seq4 : cttttttacgactccatcagaaagaggtttaatatttttgtgagaccatcgaagagagaaagagataaaacttttttacgactccatcagaaagaggttt :    800

                 *        820         *        840         *        860         *        880         *        900
Seq1 : aatatttttgtgagaccatcgaagagagaaagagataaaacttttttacgactccatcagaaagaggtttaatatttttgtgagaccatcgaagagagaa :    900
Seq2 : aatatttttgtgagaccatcgaagagagaaagagataaaacttttttacgactccatcagaaagaggtttaatatttttgtgagaccatcgaagagagaa :    900
Seq3 : aatatttttgtgagaccatcgaagagagaaagagataaaacttttttacgactccatcagaaagaggtttaatatttttgtgagaccatcgaagagagaa :    900
Seq4 : aatatttttgtgagaccatcgaagagagaaagagataaaacttttttacgactccatcagaaagaggtttaatatttttgtgagaccatcgaagagagaa :    900

                 *        920         *        940         *        960         *        980         *       1000
Seq1 : agagataaaacttttttacgactccatcagaaagaggtttaatatttttgtgagaccatcgaagagagaaagagaaagagatagttagtctagatatttt :   1000
Seq2 : agagataaaacttttttacgactccatcagaaagaggtttaatatttttgtgagaccatcgaagagagaaagagaaagagatagttagtctagatatttt :   1000
Seq3 : agagataaaacttttttacgactccatcagaaagaggtttaatatttttgtgagaccatcgaagagagaaagagaaagagatagttagtctagatatttt :   1000
Seq4 : agagataaaacttttttacgactccatcagaaagaggtttaatatttttgtgagaccatcgaagagagaaagagaaagagatagttagtctagatatttt :   1000

                 *       1020         *       1040         *       1060         *       1080         *       1100
Seq1 : tcttagtacaaaagtcaatgttttaaaatatatggacaagaatttgtctgtataaaaacttgtgtgaaattttgtaccaaagaaaaaatgtgagcagtat :   1100
Seq2 : tcttagtacaaaagtcaatgttttaaaatatatggacaagaatttgtctgtataaaaacttgtgtgaaattttgtaccaaagaaaaaatgtgagcagtat :   1100
Seq3 : tcttagtacaaaagtcaatgttttaaaatatatggacaagaatttgtctgtataaaaacttgtgtgaaattttgtaccaaagaaaaaatgtgagcagtat :   1100
Seq4 : tcttagtacaaaagtcaatgttttaaaatatatggacaagaatttgtctgtataaaaacttgtgtgaaattttgtaccaaagaaaaaatgtgagcagtat :   1100

                 *       1120         *       1140         *       1160         *       1180         *       1200
Seq1 : cccctacatggattttactagatcatttatataccaaaaaatattatacgatctacgttttattatatgattttaacgtgtaaattataaacattatttt :   1200
Seq2 : cccctacatggattttactagatcatttatataccaaaaaatattatacgatctacgttttattatatgattttaacgtgtaaattataaacattatttt :   1200
Seq3 : cccctacatggattttactagatcatttatataccaaaaaatattatacgatctacgttttattatatgattttaacgtgtaaattataaacattatttt :   1200
Seq4 : cccctacatggattttactagatcatttatataccaaaaaatattatacgatctacgttttattatatgattttaacgtgtaaattataaacattatttt :   1200

                 *       1220         *       1240         *       1260         *       1280         *       1300
Seq1 : atgatatacaattgtctggtaacctagatgggcataggggatgttgataagctcgacgagtatatgttgttggacgttattgtttaagaaatagttgatg :   1300
Seq2 : atgatatacaattgtctggtaacctagatgggcataggggatgttgataagctcgacgagtatatgttgttggacgttattgtttaagaaatagttgatg :   1300
Seq3 : atgatatacaattgtctggtaacctagatgggcataggggatgttgataagctcgacgagtatatgttgttggacgttattgtttaagaaatagttgatg :   1300
Seq4 : atgatatacaattgtctggtaacctagatgggcataggggatgttgataagctcgacgagtatatgttgttggacgttattgtttaagaaatagttgatg :   1300

                 *       1320         *       1340         *       1360         *       1380         *       1400
Seq1 : catcagaaagagaataaaaaatatttagtgagaccatcgaagagagaaagagataaaacttttttacgactccatcagaaagaggtttaatatttttgt :   1400
Seq2 : catcagaaagagaataaaaaatatttagtgagaccatcgaagagagaaagagataaaacttttttacgactccatcagaaagaggtttaatatttttgt :   1400
Seq3 : catcagaaagagaataaaaaatatttagtgagaccatcgaagagagaaagagataaaacttttttacgactccatcagaaagaggtttaatatttttgt :   1400
Seq4 : catcagaaagagaataaaaaatatttagtgagaccatcgaagagagaaagagataaaacttttttacgactccatcagaaagaggtttaatatttttgt :   1400
```

```
                *      1420         *      1440         *      1460         *      1480         *      1500
Seq1 : gagaccatcgaagagagaaagagataaaacttttttacgactccatcagaaagaggtttaatatttttgtgagaccatcgaagagagaaagagataaaac :   1500
Seq2 : gagaccatcgaagagagaaagagataaaacttttttacgactccatcagaaagaggtttaatatttttgtgagaccatcgaagagagaaagagataaaac :   1500
Seq3 : gagaccatcgaagagagaaagagataaaacttttttacgactccatcagaaagaggtttaatatttttgtgagaccatcgaagagagaaagagataaaac :   1500
Seq4 : gagaccatcgaagagagaaagagataaaacttttttacgactccatcagaaagaggtttaatatttttgtgagaccatcgaagagagaaagagataaaac :   1500

                *      1520         *      1540         *      1560         *      1580         *      1600
Seq1 : ttttttacgactccatcagaaagaggtttaatatttttgtgagaccatcgaagagagaaagagataaaacttttttacgactccatcagaaagaggttta :   1600
Seq2 : ttttttacgactccatcagaaagaggtttaatatttttgtgagaccatcgaagagagaaagagataaaacttttttacgactccatcagaaagaggttta :   1600
Seq3 : ttttttacgactccatcagaaagaggtttaatatttttgtgagaccatcgaagagagaaagagataaaacttttttacgactccatcagaaagaggttta :   1600
Seq4 : ttttttacgactccatcagaaagaggtttaatatttttgtgagaccatcgaagagagaaagagataaaacttttttacgactccatcagaaagaggttta :   1600

                *      1620         *      1640         *      1660         *      1680         *      1700
Seq1 : atatttttgtgagaccatcgaagagagaaagagataaaacttttttacgactccatcagaaagaggtttaatatttttgtgagaccatcgaagagagaaa :   1700
Seq2 : atatttttgtgagaccatcgaagagagaaagagataaaacttttttacgactccatcagaaagaggtttaatatttttgtgagaccatcgaagagagaaa :   1700
Seq3 : atatttttgtgagaccatcgaagagagaaagagataaaacttttttacgactccatcagaaagaggtttaatatttttgtgagaccatcgaagagagaaa :   1700
Seq4 : atatttttgtgagaccatcgaagagagaaagagataaaacttttttacgactccatcagaaagaggtttaatatttttgtgagaccatcgaagagagaaa :   1700

                *      1720         *      1740         *      1760         *      1780         *      1800
Seq1 : gagataaaacttttttacgactccatcagaaagaggtttaatatttttgtgagaccatcgaagagagaaagagataaaacttttttacgactccatcaga :   1800
Seq2 : gagataaaacttttttacgactccatcagaaagaggtttaatatttttgtgagaccatcgaagagagaaagagataaaacttttttacgactccatcaga :   1800
Seq3 : gagataaaacttttttacgactccatcagaaagaggtttaatatttttgtgagaccatcgaagagagaaagagataaaacttttttacgactccatcaga :   1800
Seq4 : gagataaaacttttttacgactccatcagaaagaggtttaatatttttgtgagaccatcgaagagagaaagagataaaacttttttacgactccatcaga :   1800

                *      1820         *      1840         *      1860         *      1880         *      1900
Seq1 : aagaggtttaatatttttgtgagaccatcgaagagagaaagagataaaacttttttacgactccatcagaaagaggtttaatatttttgtgagaccatcg :   1900
Seq2 : aagaggtttaatatttttgtgagaccatcgaagagagaaagagataaaacttttttacgactccatcagaaagaggtttaatatttttgtgagaccatcg :   1900
Seq3 : aagaggtttaatatttttgtgagaccatcgaagagagaaagagataaaacttttttacgactccatcagaaagaggtttaatatttttgtgagaccatcg :   1900
Seq4 : aagaggtttaatatttttgtgagaccatcgaagagagaaagagataaaacttttttacgactccatcagaaagaggtttaatatttttgtgagaccatcg :   1900

                *      1920         *      1940         *      1960         *      1980         *      2000
Seq1 : aagagagaaagagataaaacttttttacgactccatcagaaagaggtttaatatttttgtgagaccatcgaagagagaaagagataaaacttttttacga :   2000
Seq2 : aagagagaaagagataaaacttttttacgactccatcagaaagaggtttaatatttttgtgagaccatcgaagagagaaagagataaaacttttttacga :   2000
Seq3 : aagagagaaagagataaaacttttttacgactccatcagaaagaggtttaatatttttgtgagaccatcgaagagagaaagagataaaacttttttacga :   2000
Seq4 : aagagagaaagagataaaacttttttacgactccatcagaaagaggtttaatatttttgtgagaccatcgaagagagaaagagataaaacttttttacga :   2000

                *      2020         *      2040         *      2060         *      2080         *      2100
Seq1 : ctccatcagaaagaggtttaatatttttgtgagaccatcgaagagagaaagagataaaacttttttacgactccatcagaaagaggtttaatatttttgt :   2100
Seq2 : ctccatcagaaagaggtttaatatttttgtgagaccatcgaagagagaaagagataaaacttttttacgactccatcagaaagaggtttaatatttttgt :   2100
Seq3 : ctccatcagaaagaggtttaatatttttgtgagaccatcgaagagagaaagagataaaacttttttacgactccatcagaaagaggtttaatatttttgt :   2100
Seq4 : ctccatcagaaagaggtttaatatttttgtgagaccatcgaagagagaaagagataaaacttttttacgactccatcagaaagaggtttaatatttttgt :   2100
```

```
                      *        2120         *        2140         *        2160         *        2180         *        2200
Seq1 : gagaccatcgaagagagaaagagataaaacttttttacgactccatcagaaagaggtttaatatttttgtgagaccatcgaagagagaaagagataaaac :   2200
Seq2 : gagaccatcgaagagagaaagagataaaacttttttacgactccatcagaaagaggtttaatatttttgtgagaccatcgaagagagaaagagataaaac :   2200
Seq3 : gagaccatcgaagagagaaagagataaaacttttttacgactccatcagaaagaggtttaatatttttgtgagaccatcgaagagagaaagagataaaac :   2200
Seq4 : gagaccatcgaagagagaaagagataaaacttttttacgactccatcagaaagaggtttaatatttttgtgagaccatcgaagagagaaagagataaaac :   2200

                      *        2220         *        2240         *        2260         *        2280         *        2300
Seq1 : ttttttacgactccatcagaaagaggtttaatatttttgtgagaccatcgaagagagaaagagataaaacttttttacgactccatcagaaagaggttta :   2300
Seq2 : ttttttacgactccatcagaaagaggtttaatatttttgtgagaccatcgaagagagaaagagataaaacttttttacgactccatcagaaagaggttta :   2300
Seq3 : ttttttacgactccatcagaaagaggtttaatatttttgtgagaccatcgaagagagaaagagataaaacttttttacgactccatcagaaagaggttta :   2300
Seq4 : ttttttacgactccatcagaaagaggtttaatatttttgtgagaccatcgaagagagaaagagataaaacttttttacgactccatcagaaagaggttta :   2300

                      *        2320         *        2340         *        2360         *        2380         *        2400
Seq1 : atatttttgtgagaccatcgaagagagaaagagataaaacttttttacgactccatcagaaagaggtttaatatttttgtgagaccatcgaagagagaaa :   2400
Seq2 : atatttttgtgagaccatcgaagagagaaagagataaaacttttttacgactccatcagaaagaggtttaatatttttgtgagaccatcgaagagagaaa :   2400
Seq3 : atatttttgtgagaccatcgaagagagaaagagataaaacttttttacgactccatcagaaagaggtttaatatttttgtgagaccatcgaagagagaaa :   2400
Seq4 : atatttttgtgagaccatcgaagagagaaagagataaaacttttttacgactccatcagaaagaggtttaatatttttgtgagaccatcgaagagagaaa :   2400

                      *        2420         *        2440         *        2460         *        2480         *        2500
Seq1 : gagataaaacttttttacgactccatcagaaagaggtttaatatttttgtgagaccatcgaagagagaaagagataaaacttttttacgactccatcaga :   2500
Seq2 : gagataaaacttttttacgactccatcagaaagaggtttaatatttttgtgagaccatcgaagagagaaagagataaaacttttttacgactccatcaga :   2500
Seq3 : gagataaaacttttttacgactccatcagaaagaggtttaatatttttgtgagaccatcgaagagagaaagagataaaacttttttacgactccatcaga :   2500
Seq4 : gagataaaacttttttacgactccatcagaaagaggtttaatatttttgtgagaccatcgaagagagaaagagataaaacttttttacgactccatcaga :   2500

                      *        2520         *        2540         *        2560         *        2580         *        2600
Seq1 : aagaggtttaatattttagtgagaccatcgaagagagaaagagataaaacttttttacgactccatcagaaagaggtttaatatttttgtgagaccatcg :   2600
Seq2 : aagaggtttaatattttagtgagaccatcgaagagagaaagagataaaacttttttacgactccatcagaaagaggtttaatatttttgtgagaccatcg :   2600
Seq3 : aagaggtttaatatttttgtgagaccatcgaagagagaaagagataaaacttttttacgactccatcagaaagaggtttaatatttttgtgagaccatcg :   2600
Seq4 : aagaggtttaatatttttgtgagaccatcgaagagagaaagagataaaacttttttacgactccatcagaaagaggtttaatatttttgtgagaccatcg :   2600

                      *        2620         *        2640         *        2660         *        2680         *        2700
Seq1 : aagagagaaagagaataaaaatattttatgactccattgaagagagaaagagaaaatgagaatgagaataaaaatattttagtgacaccatcagaaagag :   2700
Seq2 : aagagagaaagagaataaaaatattttatgactccattgaagagagaaagagaaaatgagaatgagaataaaaatattttagtgacaccatcagaaagag :   2700
Seq3 : aagagagaaagagaataaaaatattttatgactccattgaagagagaaagagaaaatgagaatgagaataaaaatattttagtgacaccatcagaaagag :   2700
Seq4 : aagagagaaagagaataaaaatattttatgactccattgaagagagaaagagaaaatgagaatgagaataaaaatattttagtgacaccatcagaaagag :   2700

                      *        2720         *        2740         *        2760         *        2780         *        2800
Seq1 : gtttaatatttttgtgagaccatcgaagagagaaagagaataaaaatattttatgactccattgaagagagaaagagaaaatgagaatgagaataaaaat :   2800
Seq2 : gtttaatatttttgtgagaccatcgaagagagaaagagaataaaaatattttatgactccattgaagagagaaagagaaaatgagaatgagaataaaaat :   2800
Seq3 : gtttaatatttttgtgagaccatcgaagagagaaagagaataaaaatattttatgactccattgaagagagaaagagaaaatgagaatgagaataaaaat :   2800
Seq4 : gtttaatatttttgtgagaccatcgaagagagaaagagaataaaaatattttatgactccattgaagagagaaagagaaaatgagaatgagaataaaaat :   2800
```

```
                 *      2820         *      2840         *      2860         *      2880         *      2900
Seq1 : atttagtgacaccatcagaaagaggtttaatatttttttatgagaccatcaaagagagaaagagaataaaaatatttttgtaaaactttttttatgagac :   2900
Seq2 : atttagtgacaccatcagaaagaggtttaatatttttttatgagaccatcaaagagagaaagagaataaaaatatttttgtaaaactttttttatgagac :   2900
Seq3 : atttagtgacaccatcagaaagaggtttaatatttttttatgagaccatcaaagagagaaagagaataaaaatatttttgtaaaactttttttatgagac :   2900
Seq4 : atttagtgacaccatcagaaagaggtttaatatttttttatgagaccatcaaagagagaaagagaataaaaatatttttgtaaaactttttttatgagac :   2900

                 *      2920         *      2940         *      2960         *      2980         *      3000
Seq1 : catcaaagagagaaagagaataaaaatatttttgtaaaactttttttatgagaccatcaaagagagaaagagaataaaaatatttttgtaaaactttttt :   3000
Seq2 : catcaaagagagaaagagaataaaaatatttttgtaaaactttttttatgagaccatcaaagagagaaagagaataaaaatatttttgtaaaactttttt :   3000
Seq3 : catcaaagagagaaagagaataaaaatatttttgtaaaactttttttatgagaccatcaaagagagaaagagaataaaaatatttttgtaaaactttttt :   3000
Seq4 : catcaaagagagaaagagaataaaaatatttttgtaaaactttttttatgagaccatcaaagagagaaagagaataaaaatatttttgtaaaactttttt :   3000

                 *      3020         *      3040         *      3060         *      3080         *      3100
Seq1 : tatgagaccatcaaagagagaaagagaataaaaatatttttgtaaaactttttttatgagaccatcaaagagagaaagagaataaaaatatttttgtaaa :   3100
Seq2 : tatgagaccatcaaagagagaaagagaataaaaatatttttgtaaaactttttttatgagaccatcaaagagagaaagagaataaaaatatttttgtaaa :   3100
Seq3 : tatgagaccatcaaagagagaaagagaataaaaatatttttgtaaaactttttttatgagaccatcaaagagagaaagagaataaaaatatttttgtaaa :   3100
Seq4 : tatgagaccatcaaagagagaaagagaataaaaatatttttgtaaaactttttttatgagaccatcaaagagagaaagagaataaaaatatttttgtaaa :   3100

                 *      3120         *      3140         *      3160         *      3180         *      3200
Seq1 : actttttttatgagaccatcaaagagagaaagagaataaaaatatttttgtaaaactttttttatgagaccatcaaagagagaaagagaataaaaatatt :   3200
Seq2 : actttttttatgagaccatcaaagagagaaagagaataaaaatatttttgtaaaactttttttatgagaccatcaaagagagaaagagaataaaaatatt :   3200
Seq3 : actttttttatgagaccatcaaagagagaaagagaataaaaatatttttgtaaaactttttttatgagaccatcaaagagagaaagagaataaaaatatt :   3200
Seq4 : actttttttatgagaccatcaaagagagaaagagaataaaaatatttttgtaaaactttttttatgagaccatcaaagagagaaagagaataaaaatatt :   3200

                 *      3220         *      3240         *      3260         *      3280         *      3300
Seq1 : tttgtaaaactttttttatgagaccatcaaagagagaaagagaataaaaatatttttgtaaaactttttttatgagaccatcagaaagaggtttaatatt :   3300
Seq2 : tttgtaaaactttttttatgagaccatcaaagagagaaagagaataaaaatatttttgtaaaactttttttatgagaccatcagaaagaggtttaatatt :   3300
Seq3 : tttgtaaaactttttttatgagaccatcaaagagagaaagagaataaaaatatttttgtaaaactttttttatgagaccatcagaaagaggtttaatatt :   3300
Seq4 : tttgtaaaactttttttatgagaccatcaaagagagaaagagaataaaaatatttttgtaaaactttttttatgagaccatcagaaagaggtttaatatt :   3300

                 *      3320         *      3340         *      3360         *      3380         *      3400
Seq1 : tttgtgataccctgaaaggaaataggaataggaataggaatagtgtcataatcgtatcacactattgagacagaaaaagaagaagtcgcgagaggtaact :   3400
Seq2 : tttgtgataccctgaaaggaaataggaataggaataggaatagtgtcataatcgtatcacactattgagacagaaaaagaagaagtcgcgagaggtaact :   3400
Seq3 : tttgtgataccctgaaaggaaataggaataggaataggaatagtgtcataatcgtatcacactattgagacagaaaaagaagaagtcgcgagaggtaact :   3400
Seq4 : tttgtgataccctgaaaggaaataggaataggaataggaatagtgtcataatcgtatcacactattgagacagaaaaagaagaagtcgcgagaggtaact :   3400

                 *      3420         *      3440         *      3460         *      3480         *      3500
Seq1 : ttttgtgaatgtagttaagaacatttttgttttgcaaaccggaatatagtgtccggtacacttttttaattcgtggtgtgcctgaatcgttcgattaacc :   3500
Seq2 : ttttgtgaatgtagttaagaacatttttgttttgcaaaccggaatatagtgtccggtacacttttttaattcgtggtgtgcctgaatcgttcgattaacc :   3500
Seq3 : ttttgtgaatgtagttaagaacatttttgttttgcaaaccggaatatagtgtccggtacacttttttaattcgtggtgtgcctgaatcgttcgattaacc :   3500
Seq4 : ttttgtgaatgtagttaagaacatttttgttttgcaaaccggaatatagtgtccggtacacttttttaattcgtggtgtgcctgaatcgttcgattaacc :   3500
```

```
                 *        3520          *        3540          *        3560          *        3580          *        3600
Seq1 : ctactcatccaatttcagatgaatagagttatcgattcagacacacgctttgagttttgttgaatcgatgagtgaagtatcatcggttgcaccttcagat :  3600
Seq2 : ctactcatccaatttcagatgaatagagttatcgattcagacacacgctttgagttttgttgaatcgatgagtgaagtatcatcggttgcaccttcagat :  3600
Seq3 : ctactcatccaatttcagatgaatagagttatcgattcagacacacgctttgagttttgttgaatcgatgagtgaagtatcatcggttgcaccttcagat :  3600
Seq4 : ctactcatccaatttcagatgaatagagttatcgattcagacacacgctttgagttttgttgaatcgatgagtgaagtatcatcggttgcaccttcagat :  3600

                 *        3620          *        3640          *        3660          *        3680          *        3700
Seq1 : gccgatccgtcgacatacttaaatccatccttgacctcaagttcagatgattccttgcacatgtctccgatacgaacgctaaactctagattcttgacac :  3700
Seq2 : gccgatccgtcgacatacttaaatccatccttgacctcaagttcagatgattccttgcacatgtctccgatacgaacgctaaactctagattcttgacac :  3700
Seq3 : gccgatccgtcgacatacttaaatccatccttgacctcaagttcagatgattccttgcacatgtctccgatacgaacgctaaactctagattcttgacac :  3700
Seq4 : gccgatccgtcgacatacttaaatccatccttgacctcaagttcagatgattccttgcacatgtctccgatacgaacgctaaactctagattcttgacac :  3700

                 *        3720          *        3740          *        3760          *        3780          *        3800
Seq1 : attttgtatcgacgatcgttgaaccgatgatatcttcgtaactcactttcttatgagagatgttagacccgagtactggatgggtcttgatgtcgctgtc :  3800
Seq2 : attttgtatcgacgatcgttgaaccgatgatatcttcgtaactcactttcttatgagagatgttagacccgagtactggatgggtcttgatgtcgctgtc :  3800
Seq3 : attttgtatcgacgatcgttgaaccgatgatatcttcgtaactcactttcttatgagagatgttagacccgagtactggatgggtcttgatgtcgctgtc :  3800
Seq4 : attttgtatcgacgatcgttgaaccgatgatatcttcgtaactcactttcttatgagagatgttagacccgagtactggatgggtcttgatgtcgctgtc :  3800

                 *        3820          *        3840          *        3860          *        3880          *        3900
Seq1 : tttctcttcttcgctacatctgatgtcgatagacacctcacagtctttgatcatagccagagcttcttcatgagtgatcgcgggagagtccttaccttgt :  3900
Seq2 : tttctcttcttcgctacatctgatgtcgatagacacctcacagtctttgatcatagccagagcttcttcatgagtgatcgcgggagagtccttaccttgt :  3900
Seq3 : tttctcttcttcgctacatctgatgtcgatagacacctcacagtctttgatcatagccagagcttcttcatgagtgatcgcgggagagtccttaccttgt :  3900
Seq4 : tttctcttcttcgctacatctgatgtcgatagacacctcacagtctttgatcatagccagagcttcttcatgagtgatcgcgggagagtccttaccttgt :  3900

                 *        3920          *        3940          *        3960          *        3980          *        4000
Seq1 : cctggggacacgctggacaatctagcattcactgtgtttccatcagcggattctgagatggatttaatctgaggacatttggtgaatccaaagttcattc :  4000
Seq2 : cctggggacacgctggacaatctagcattcactgtgtttccatcagcggattctgagatggatttaatctgaggacatttggtgaatccaaagttcattc :  4000
Seq3 : cctggggacacgctggacaatctagcattcactgtgtttccatcagcggattctgagatggatttaatctgaggacatttggtgaatccaaagttcattc :  4000
Seq4 : cctggggacacgctggacaatctagcattcactgtgtttccatcagcggattctgagatggatttaatctgaggacatttggtgaatccaaagttcattc :  4000

                 *        4020          *        4040          *        4060          *        4080          *        4100
Seq1 : tcagacctccaccgatgatggagtaataagtggtaggaggatctacatcctcgactgatgtggaatcatcttctgattccacctcgggatctggatctga :  4100
Seq2 : tcagacctccaccgatgatggagtaataagtggtaggaggatctacatcctcgactgatgtggaatcatcttctgattccacctcgggatctggatctga :  4100
Seq3 : tcagacctccaccgatgatggagtaataagtggtaggaggatctacatcctcgactgatgtggaatcatcttctgattccacctcgggatctggatctga :  4100
Seq4 : tcagacctccaccgatgatggagtaataagtggtaggaggatctacatcctcgactgatgtggaatcatcttctgattccacctcgggatctggatctga :  4100

                 *        4120          *        4140          *        4160          *        4180          *        4200
Seq1 : ctcggactctgtaatttccgttacggattggcaaatcttatcattggtcggtgtttggtcttgctttgtgactttgataataacatcgattcccatatga :  4200
Seq2 : ctcggactctgtaatttccgttacggattggcaaatcttatcattggtcggtgtttggtcttgctttgtgactttgataataacatcgattcccatatga :  4200
Seq3 : ctcggactctgtaatttccgttacggattggcaaatcttatcattggtcggtgtttggtcttgctttgtgactttgataataacatcgattcccatatga :  4200
Seq4 : ctcggactctgtaatttccgttacggattggcaaatcttatcattggtcggtgtttggtcttgctttgtgactttgataataacatcgattcccatatga :  4200
```

```
                 *       4220          *       4240          *       4260          *       4280          *       4300
Seq1 : tgtttgttttcttcttccgtacacgaggaggaggatgaggatgattgctgaagactggcaggcacatgcatgccaggacgatatattgtttcatgattgc :   4300
Seq2 : tgtttgttttcttcttccgtacacgaggaggaggatgaggatgattgctgaagactggcaggcacatgcatgccaggacgatatattgtttcatgattgc :   4300
Seq3 : tgtttgttttcttcttccgtacacgaggaggaggatgaggatgattgctgaagactggcaggcacatgcatgccaggacgatatattgtttcatgattgc :   4300
Seq4 : tgtttgttttcttcttccgtacacgaggaggaggatgaggatgattgctgaagactggcaggcacatgcatgccaggacgatatattgtttcatgattgc :   4300

                 *       4320          *       4340          *       4360          *       4380          *       4400
Seq1 : tattgattgagtactgttctttatgattctacttccttaccgtgcaataaattagaatatattttctactttacgagaaattaattattgtatttatta :   4400
Seq2 : tattgattgagtactgttctttatgattctacttccttaccgtgcaataaattagaatatattttctactttacgagaaattaattattgtatttatta :   4400
Seq3 : tattgattgagtactgttctttatgattctacttccttaccgtgcaataaattagaatatattttctactttacgagaaattaattattgtatttatta :   4400
Seq4 : tattgattgagtactgttctttatgattctacttccttaccgtgcaataaattagaatatattttctactttacgagaaattaattattgtatttatta :   4400

                 *       4420          *       4440          *       4460          *       4480          *       4500
Seq1 : tttatgggtgaaaaacttactataaaaagcgggtgggtttggaattagtgatcagtttatgtatatcgcaactaccgggcatatggctatcgacatcgag :   4500
Seq2 : tttatgggtgaaaaacttactataaaaagcgggtgggtttggaattagtgatcagtttatgtatatcgcaactaccgggcatatggctatcgacatcgag :   4500
Seq3 : tttatgggtgaaaaacttactataaaaagcgggtgggtttggaattagtgatcagtttatgtatatcgcaactaccgggcatatggctatcgacatcgag :   4500
Seq4 : tttatgggtgaaaaacttactataaaaagcgggtgggtttggaattagtgatcagtttatgtatatcgcaactaccgggcatatggctatcgacatcgag :   4500

                 *       4520          *       4540          *       4560          *       4580          *       4600
Seq1 : aacattacccacatgataagagattgtatcagtttcgtagtcttgagtattggtattactatatagtatatagatgtcgacgctagatagacagtctccg :   4600
Seq2 : aacattacccacatgataagagattgtatcagtttcgtagtcttgagtattggtattactatatagtatatagatgtcgacgctagatagacagtctccg :   4600
Seq3 : aacattacccacatgataagagattgtatcagtttcgtagtcttgagtattggtattactatatagtatatagatgtcgacgctagatagacagtctccg :   4600
Seq4 : aacattacccacatgataagagattgtatcagtttcgtagtcttgagtattggtattactatatagtatatagatgtcgacgctagatagacagtctccg :   4600

                 *       4620          *       4640          *       4660          *       4680          *       4700
Seq1 : aatgcggcatgataccgtcatcattctttgctttcgttaactgtttggaggaaaaatttttgttattgcatttaatctcgaaattcagagtgcacacctt :   4700
Seq2 : aatgcggcatgataccgtcatcattctttgctttcgttaactgtttggaggaaaaatttttgttattgcatttaatctcgaaattcagagtgcacacctt :   4700
Seq3 : aatgcggcatgataccgtcatcattctttgctttcgttaactgtttggaggaaaaatttttgttattgcatttaatctcgaaattcagagtgcacacctt :   4700
Seq4 : aatgcggcatgataccgtcatcattctttgctttcgttaactgtttggaggaaaaatttttgttattgcatttaatctcgaaattcagagtgcacacctt :   4700

                 *       4720          *       4740          *       4760          *       4780          *       4800
Seq1 : tctcctgtaaagaaacctgaagttgctaccttattaaggacggagaagtattcctcacgaaatacgggattacagtctttatgattcatagtaatagtta :   4800
Seq2 : tctcctgtaaagaaacctgaagttgctaccttattaaggacggagaagtattcctcacgaaatacgggattacagtctttatgattcatagtaatagtta :   4800
Seq3 : tctcctgtaaagaaacctgaagttgctaccttattaaggacggagaagtattcctcacgaaatacgggattacagtctttatgattcatagtaatagtta :   4800
Seq4 : tctcctgtaaagaaacctgaagttgctaccttattaaggacggagaagtattcctcacgaaatacgggattacagtctttatgattcatagtaatagtta :   4800

                 *       4820          *       4840          *       4860          *       4880          *       4900
Seq1 : gttccgacgttgagatggattcgctgagaccggtagtggtcgttaactggatacagattaatttccacatcgatatagttaaaggtattactgggtacgg :   4900
Seq2 : gttccgacgttgagatggattcgctgagaccggtagtggtcgttaactggatacagattaatttccacatcgatatagttaaaggtattactgggtacgg :   4900
Seq3 : gttccgacgttgagatggattcgctgagaccggtagtggtcgttaactggatacagattaatttccacatcgatatagttaaaggtattactgggtacgg :   4900
Seq4 : gttccgacgttgagatggattcgctgagaccggtagtggtcgttaactggatacagattaatttccacatcgatatagttaaaggtattactgggtacgg :   4900
```

```
                *       4920          *       4940          *       4960          *       4980          *       5000
Seq1 : gttcgcatttatctgcggaagagacggtgtgagaatatgttccgagaccacacggagaacagatgacgtctccggatactccgtatcctattccacattt :   5000
Seq2 : gttcgcatttatctgcggaagagacggtgtgagaatatgttccgagaccacacggagaacagatgacgtctccggatactccgtatcctattccacattt :   5000
Seq3 : gttcgcatttatctgcggaagagacggtgtgagaatatgttccgagaccacacggagaacagatgacgtctccggatactccgtatcctattccacattt :   5000
Seq4 : gttcgcatttatctgcggaagagacggtgtgagaatatgttccgagaccacacggagaacagatgacgtctccggatactccgtatcctattccacattt :   5000

                *       5020          *       5040          *       5060          *       5080          *       5100
Seq1 : tgtttgggaaacacatgccttgcatccggatgatcctttgagaagacaataatatccgggagagcattcacagattctattgtgagtcgtgttacacggt :   5100
Seq2 : tgtttgggaaacacatgccttgcatccggatgatcctttgagaagacaataatatccgggagagcattcacagattctattgtgagtcgtgttacacggt :   5100
Seq3 : tgtttgggaaacacatgccttgcatccggatgatcctttgagaagacaataatatccgggagagcattcacagattctattgtgagtcgtgttacacggt :   5100
Seq4 : tgtttgggaaacacatgccttgcatccggatgatcctttgagaagacaataatatccgggagagcattcacagattctattgtgagtcgtgttacacggt :   5100

                *       5120          *       5140          *       5160          *       5180          *       5200
Seq1 : cgcgtcttccgttacaacttagacaagcgggtaaatgattattgcgagatgtgaaggtacccgaaccacacggcgtacattgtgtgttagtcttgctatc :   5200
Seq2 : cgcgtcttccgttacaacttagacaagcgggtaaatgattattgcgagatgtgaaggtacccgaaccacacggcgtacattgtgtgttagtcttgctatc :   5200
Seq3 : cgcgtcttccgttacaacttagacaagcgggtaaatgattattgcgagatgtgaaggtacccgaaccacacggcgtacattgtgtgttagtcttgctatc :   5200
Seq4 : cgcgtcttccgttacaacttagacaagcgggtaaatgattattgcgagatgtgaaggtacccgaaccacacggcgtacattgtgtgttagtcttgctatc :   5200

                *       5220          *       5240          *       5260          *       5280          *       5300
Seq1 : gcataatctggaagcgtatgttcccggacacaaattatggcgtttgtattcgttgtctttacactttccatcggatggtgcatgcggtgctatatctctt :   5300
Seq2 : gcataatctggaagcgtatgttcccggacacaaattatggcgtttgtattcgttgtctttacactttccatcggatggtgcatgcggtgctatatctctt :   5300
Seq3 : gcataatctggaagcgtatgttcccggacacaaattatggcgtttgtattcgttgtctttacactttccatcggatggtgcatgcggtgctatatctctt :   5300
Seq4 : gcataatctggaagcgtatgttcccggacacaaattatggcgtttgtattcgttgtctttacactttccatcggatggtgcatgcggtgctatatctctt :   5300

                *       5320          *       5340          *       5360          *       5380          *       5400
Seq1 : ccgtttattattatacatgagagaaacaatatatacgagtataatacggacttcatgatttaataatgtagtaatcgtcgtcttgttcctgtttcctact :   5400
Seq2 : ccgtttattattatacatgagagaaacaatatatacgagtataatacggacttcatgatttaataatgtagtaatcgtcgtcttgttcctgtttcctact :   5400
Seq3 : ccgtttattattatacatgagagaaacaatatatacgagtataatacggacttcatgatttaataatgtagtaatcgtcgtcttgttcctgtttcctact :   5400
Seq4 : ccgtttattattatacatgagagaaacaatatatacgagtataatacggacttcatgatttaataatgtagtaatcgtcgtcttgttcctgtttcctact :   5400

                *       5420          *       5440          *       5460          *       5480          *       5500
Seq1 : tctccaatcatatagatattttctttctatcatggataatatttgtaatggttcttttcgtacaacatactgtttagatgatattgcgcataatttccgg :   5500
Seq2 : tctccaatcatatagatattttctttctatcatggataatatttgtaatggttcttttcgtacaacatactgtttagatgatattgcgcataatttccgg :   5500
Seq3 : tctccaatcatatagatattttctttctatcatggataatatttgtaatggttcttttcgtacaacatactgtttagatgatattgcgcataatttccgg :   5500
Seq4 : tctccaatcatatagatattttctttctatcatggataatatttgtaatggttcttttcgtacaacatactgtttagatgatattgcgcataatttccgg :   5500

                *       5520          *       5540          *       5560          *       5580          *       5600
Seq1 : aggcaaatacgatagtctagattgaccgatggtagactctaatttattgagtgctttgtcgacgagtttacttttacgctccatcgatagatggcactgt :   5600
Seq2 : aggcaaatacgatagtctagattgaccgatggtagactctaatttattgagtgctttgtcgacgagtttacttttacgctccatcgatagatggcactgt :   5600
Seq3 : aggcaaatacgatagtctagattgaccgatggtagactctaatttattgagtgctttgtcgacgagtttacttttacgctccatcgatagatggcactgt :   5600
Seq4 : aggcaaatacgatagtctagattgaccgatggtagactctaatttattgagtgctttgtcgacgagtttacttttacgctccatcgatagatggcactgt :   5600
```

```
                 *      5620          *      5640          *      5660          *      5680          *      5700
Seq1 : tctatgagatcgtcgtacatgggaaatgaaatgtgactgtctgaatgtatggctttaagatagctgtgataccgtatacaggtcggtgtcggagattcga :   5700
Seq2 : tctatgagatcgtcgtacatgggaaatgaaatgtgactgtctgaatgtatggctttaagatagctgtgataccgtatacaggtcggtgtcggagattcga :   5700
Seq3 : tctatgagatcgtcgtacatgggaaatgaaatgtgactgtctgaatgtatggctttaagatagctgtgataccgtatacaggtcggtgtcggagattcga :   5700
Seq4 : tctatgagatcgtcgtacatgggaaatgaaatgtgactgtctgaatgtatggctttaagatagctgtgataccgtatacaggtcggtgtcggagattcga :   5700

                 *      5720          *      5740          *      5760          *      5780          *      5800
Seq1 : atctctttaaggcgacttatgtcacgatgatggaatctatcttatcgaatgatatattttttcataaatacacttttatagtcctcgtttaaacagaattt :   5800
Seq2 : atctctttaaggcgacttatgtcacgatgatggaatctatcttatcgaatgatatattttttcataaatacacttttatagtcctcgtttaaacagaattt :   5800
Seq3 : atctctttaaggcgacttatgtcacgatgatggaatctatcttatcgaatgatatattttttcataaatacacttttatagtcctcgtttaaacagaattt :   5800
Seq4 : atctctttaaggcgacttatgtcacgatgatggaatctatcttatcgaatgatatattttttcataaatacacttttatagtcctcgtttaaacagaattt :   5800

                 *      5820          *      5840          *      5860          *      5880          *      5900
Seq1 : actatgtagttccgcgaatgactcgtcccttaataggcagtaggctattatcttctttacgtagtaatcgtcgtagggagagacatcttgtagaacaacg :   5900
Seq2 : actatgtagttccgcgaatgactcgtcccttaataggcagtaggctattatcttctttacgtagtaatcgtcgtagggagagacatcttgtagaacaacg :   5900
Seq3 : actatgtagttccgcgaatgactcgtcccttaataggcagtaggctattatcttctttacgtagtaatcgtcgtagggagagacatcttgtagaacaacg :   5900
Seq4 : actatgtagttccgcgaatgactcgtcccttaataggcagtaggctattatcttctttacgtagtaatcgtcgtagggagagacatcttgtagaacaacg :   5900

                 *      5920          *      5940          *      5960          *      5980          *      6000
Seq1 : atttaatcataggtagagatactttcagtctgtggtggatgatgtcattcacaacatccgccttgtatatgatgtttctgttttcaaacaccaagtcgaa :   6000
Seq2 : atttaatcataggtagagatactttcagtctgtggtggatgatgtcattcacaacatccgccttgtatatgatgtttctgttttcaaacaccaagtcgaa :   6000
Seq3 : atttaatcataggtagagatactttcagtctgtggtggatgatgtcattcacaacatccgccttgtatatgatgtttctgttttcaaacaccaagtcgaa :   6000
Seq4 : atttaatcataggtagagatactttcagtctgtggtggatgatgtcattcacaacatccgccttgtatatgatgtttctgttttcaaacaccaagtcgaa :   6000

                 *      6020          *      6040          *      6060          *      6080          *      6100
Seq1 : taccgtctttagtcggaaggttgatgtcgtatccgatgtatgaggcaacattgttgttacaattttgaaaggcggtattatagtattcgtctttctgaat :   6100
Seq2 : taccgtctttagtcggaaggttgatgtcgtatccgatgtatgaggcaacattgttgttacaattttgaaaggcggtattatagtattcgtctttctgaat :   6100
Seq3 : taccgtctttagtcggaaggttgatgtcgtatccgatgtatgaggcaacattgttgttacaattttgaaaggcggtattatagtattcgtctttctgaat :   6100
Seq4 : taccgtctttagtcggaaggttgatgtcgtatccgatgtatgaggcaacattgttgttacaattttgaaaggcggtattatagtattcgtctttctgaat :   6100

                 *      6120          *      6140          *      6160          *      6180          *      6200
Seq1 : gtcgaacctatctagtagataccgtagtatattgagagtgtatccttgattatgttttatgaatagataaagtagatgttgtccttcttccttttgttcg :   6200
Seq2 : gtcgaacctatctagtagataccgtagtatattgagagtgtatccttgattatgttttatgaatagataaagtagatgttgtccttcttccttttgttcg :   6200
Seq3 : gtcgaacctatctagtagataccgtagtatattgagagtgtatccttgattatgttttatgaatagataaagtagatgttgtccttcttccttttgttcg :   6200
Seq4 : gtcgaacctatctagtagataccgtagtatattgagagtgtatccttgattatgttttatgaatagataaagtagatgttgtccttcttccttttgttcg :   6200

                 *      6220          *      6240          *      6260          *      6280          *      6300
Seq1 : tgccaattgagtaacattatgagaatatgacctgttgcacaatcgttccatgatgggtgtacaatcaagattattacgtatcctcgtatcggctcctcga :   6300
Seq2 : tgccaattgagtaacattatgagaatatgacctgttgcacaatcgttccatgatgggtgtacaatcaagattattacgtatcctcgtatcggctcctcga :   6300
Seq3 : tgccaattgagtaacattatgagaatatgacctgttgcacaatcgttccatgatgggtgtacaatcaagattattacgtatcctcgtatcggctcctcga :   6300
Seq4 : tgccaattgagtaacattatgagaatatgacctgttgcacaatcgttccatgatgggtgtacaatcaagattattacgtatcctcgtatcggctcctcga :   6300
```

```
                  *      6320         *      6340         *      6360         *      6380         *      6400
Seq1 : gataaaagagcatacaccacacgaggactatgtttggtatactgttgaaggtaagtgtgtaaccgcgttaatgtttgctccataatctattatcgcgtag :   6400
Seq2 : gataaaagagcatacaccacacgaggactatgtttggtatactgttgaaggtaagtgtgtaaccgcgttaatgtttgctccataatctattatcgcgtag :   6400
Seq3 : gataaaagagcatacaccacacgaggactatgtttggtatactgttgaaggtaagtgtgtaaccgcgttaatgtttgctccataatctattatcgcgtag :   6400
Seq4 : gataaaagagcatacaccacacgaggactatgtttggtatactgttgaaggtaagtgtgtaaccgcgttaatgtttgctccataatctattatcgcgtag :   6400

                  *      6420         *      6440         *      6460         *      6480         *      6500
Seq1 : atgaatcgcttctcggctcgcatcttagtgtgacttaacttgtaataattgcttttgtagaacgtggatatgtgtttacagtagtaatgaagagaagtga :   6500
Seq2 : atgaatcgcttctcggctcgcatcttagtgtgacttaacttgtaataattgcttttgtagaacgtggatatgtgtttacagtagtaatgaagagaagtga :   6500
Seq3 : atgaatcgcttctcggctcgcatcttagtgtgacttaacttgtaataattgcttttgtagaacgtggatatgtgtttacagtagtaatgaagagaagtga :   6500
Seq4 : atgaatcgcttctcggctcgcatcttagtgtgacttaacttgtaataattgcttttgtagaacgtggatatgtgtttacagtagtaatgaagagaagtga :   6500

                  *      6520         *      6540         *      6560         *      6580         *      6600
Seq1 : gttcatcctcgtcggatcctttgtacagaacgtaatagtttaagctcccattgaatttatatctaagataacacagcaatagatcggatgatttactaaa :   6600
Seq2 : gttcatcctcgtcggatcctttgtacagaacgtaatagtttaagctcccattgaatttatatctaagataacacagcaatagatcggatgatttactaaa :   6600
Seq3 : gttcatcctcgtcggatcctttgtacagaacgtaatagtttaagctcccattgaatttatatctaagataacacagcaatagatcggatgatttactaaa :   6600
Seq4 : gttcatcctcgtcggatcctttgtacagaacgtaatagtttaagctcccattgaatttatatctaagataacacagcaatagatcggatgatttactaaa :   6600

                  *      6620         *      6640         *      6660         *      6680         *      6700
Seq1 : gtcatcaatggtgtccgttagtatatcaaagatcttgttatcgattgatagtggtgtcctttttcatccttgctatcaaagttacgcatgccgtggtgta :   6700
Seq2 : gtcatcaatggtgtccgttagtatatcaaagatcttgttatcgattgatagtggtgtcctttttcatccttgctatcaaagttacgcatgccgtggtgta :   6700
Seq3 : gtcatcaatggtgtccgttagtatatcaaagatcttgttatcgattgatagtggtgtcctttttcatccttgctatcaaagttacgcatgccgtggtgta :   6700
Seq4 : gtcatcaatggtgtccgttagtatatcaaagatcttgttatcgattgatagtggtgtcctttttcatccttgctatcaaagttacgcatgccgtggtgta :   6700

                  *      6720         *      6740         *      6760         *      6780         *      6800
Seq1 : acaatatctttaatacagatggattaaatcgtgtattcatcgtatagcaatgtaatggagagttacctcgtttattcagatcgcagtgtttaataactag :   6800
Seq2 : acaatatctttaatacagatggattaaatcgtgtattcatcgtatagcaatgtaatggagagttacctcgtttattcagatcgcagtgtttaataactag :   6800
Seq3 : acaatatctttaatacagatggattaaatcgtgtattcatcgtatagcaatgtaatggagagttacctcgtttattcagatcgcagtgtttaataactag :   6800
Seq4 : acaatatctttaatacagatggattaaatcgtgtattcatcgtatagcaatgtaatggagagttacctcgtttattcagatcgcagtgtttaataactag :   6800

                  *      6820         *      6840         *      6860         *      6880         *      6900
Seq1 : cttaaacagatgagacgatgtatccacatcaaagaacgtgaaatacatatgacagacattgttgacagaaacgtgaccttcattcttaccgtcgtccata :   6900
Seq2 : cttaaacagatgagacgatgtatccacatcaaagaacgtgaaatacatatgacagacattgttgacagaaacgtgaccttcattcttaccgtcgtccata :   6900
Seq3 : cttaaacagatgagacgatgtatccacatcaaagaacgtgaaatacatatgacagacattgttgacagaaacgtgaccttcattcttaccgtcgtccata :   6900
Seq4 : cttaaacagatgagacgatgtatccacatcaaagaacgtgaaatacatatgacagacattgttgacagaaacgtgaccttcattcttaccgtcgtccata :   6900

                  *      6920         *      6940         *      6960         *      6980         *      7000
Seq1 : aatacgttaggtatgtaccacatactgtcgcgaacgatgcgtacaatctcgtccatctcataatgatttactttttcataattaaagatgtgaaagaaaa :   7000
Seq2 : aatacgttaggtatgtaccacatactgtcgcgaacgatgcgtacaatctcgtccatctcataatgatttactttttcataattaaagatgtgaaagaaaa :   7000
Seq3 : aatacgttaggtatgtaccacatactgtcgcgaacgatgcgtacaatctcgtccatctcataatgatttactttttcataattaaagatgtgaaagaaaa :   7000
Seq4 : aatacgttaggtatgtaccacatactgtcgcgaacgatgcgtacaatctcgtccatctcataatgatttactttttcataattaaagatgtgaaagaaaa :   7000
```

```
            *      7020          *      7040          *      7060          *      7080          *      7100
Seq1 : acagaacaatatattttttttagtaatgtttatgcgagacatataaaataaactccgtgtttatgatcatttttaacagcaacacattcaatattgtattg :   7100
Seq2 : acagaacaatatattttttttagtaatgtttatgcgagacatataaaataaactccgtgtttatgatcatttttaacagcaacacattcaatattgtattg :   7100
Seq3 : acagaacaatatattttttttagtaatgtttatgcgagacatataaaataaactccgtgtttatgatcatttttaacagcaacacattcaatattgtattg :   7100
Seq4 : acagaacaatatattttttttagtaatgtttatgcgagacatataaaataaactccgtgtttatgatcatttttaacagcaacacattcaatattgtattg :   7100

            *      7120          *      7140          *      7160          *      7180          *      7200
Seq1 : ttatttttatattatttacacaattaacaatatattattagtttatattactgaattaataatataaaattcccaatcttgtcataaacacacactgaga :   7200
Seq2 : ttatttttatattatttacacaattaacaatatattattagtttatattactgaattaataatataaaattcccaatcttgtcataaacacacactgaga :   7200
Seq3 : ttatttttatattatttacacaattaacaatatattattagtttatattactgaattaataatataaaattcccaatcttgtcataaacacacactgaga :   7200
Seq4 : ttatttttatattatttacacaattaacaatatattattagtttatattactgaattaataatataaaattcccaatcttgtcataaacacacactgaga :   7200

            *      7220          *      7240          *      7260          *      7280          *      7300
Seq1 : aacagcataaacacaaaatccatcaaaaatgtcgatgaaatatctgatgttgttgttcgctgctatgataatcagatcattcgccgatagtggtaacgct :   7300
Seq2 : aacagcataaacacaaaatccatcaaaaatgtcgatgaaatatctgatgttgttgttcgctgctatgataatcagatcattcgccgatagtggtaacgct :   7300
Seq3 : aacagcataaacacaaaatccatcaaaaatgtcgatgaaatatctgatgttgttgttcgctgctatgataatcagatcattcgccgatagtggtaacgct :   7300
Seq4 : aacagcataaacacaaaatccatcaaaaatgtcgatgaaatatctgatgttgttgttcgctgctatgataatcagatcattcgccgatagtggtaacgct :   7300

            *      7320          *      7340          *      7360          *      7380          *      7400
Seq1 : atcgaaacgacatcgccagaaattacaaacgctacaacagatattccagctatcagattatgcggtccagagggagatggatattgtttacacggtgact :   7400
Seq2 : atcgaaacgacatcgccagaaattacaaacgctacaacagatattccagctatcagattatgcggtccagagggagatggatattgtttacacggtgact :   7400
Seq3 : atcgaaacgacatcgccagaaattacaaacgctacaacagatattccagctatcagattatgcggtccagagggagatggatattgtttacacggtgact :   7400
Seq4 : atcgaaacgacatcgccagaaattacaaacgctacaacagatattccagctatcagattatgcggtccagagggagatggatattgtttacacggtgact :   7400

            *      7420          *      7440          *      7460          *      7480          *      7500
Seq1 : gtatccacgctagagatattgacggtatgtattgtagatgctctcatggttatacaggcattagatgtcagcatgtagtattagtagactatcaacgttc :   7500
Seq2 : gtatccacgctagagatattgacggtatgtattgtagatgctctcatggttatacaggcattagatgtcagcatgtagtattagtagactatcaacgttc :   7500
Seq3 : gtatccacgctagagatattgacggtatgtattgtagatgctctcatggttatacaggcattagatgtcagcatgtagtattagtagactatcaacgttc :   7500
Seq4 : gtatccacgctagagatattgacggtatgtattgtagatgctctcatggttatacaggcattagatgtcagcatgtagtattagtagactatcaacgttc :   7500

            *      7520          *      7540          *      7560          *      7580          *      7600
Seq1 : agaaaacccaaacactacaacgtcatatatcccatctcccggtattatgcttgtattagtaggcattattattattacgtgttgtctattatctgtttat :   7600
Seq2 : agaaaacccaaacactacaacgtcatatatcccatctcccggtattatgcttgtattagtaggcattattattattacgtgttgtctattatctgtttat :   7600
Seq3 : agaaaacccaaacactacaacgtcatatatcccatctcccggtattatgcttgtattagtaggcattattattattacgtgttgtctattatctgtttat :   7600
Seq4 : agaaaacccaaacactacaacgtcatatatcccatctcccggtattatgcttgtattagtaggcattattattattacgtgttgtctattatctgtttat :   7600

            *      7620          *      7640          *      7660          *      7680          *      7700
Seq1 : aggttcactcgacgaactaaactacctatacaagatatggttgtgccataattttataaatttttttatgagtatttttacaaaaaaaatgtataaagt :   7700
Seq2 : aggttcactcgacgaactaaactacctatacaagatatggttgtgccataattttataaatttttttatgagtatttttacaaaaaaaatgtataaagt :   7700
Seq3 : aggttcactcgacgaactaaactacctatacaagatatggttgtgccataattttataaatttttttatgagtatttttacaaaaaaaatgtataaagt :   7700
Seq4 : aggttcactcgacgaactaaactacctatacaagatatggttgtgccataattttataaatttttttatgagtatttttacaaaaaaaatgtataaagt :   7700
```

```
              *      7720          *      7740          *      7760          *      7780          *      7800
Seq1 : gtatgtcttatgtatatttataaaaatgctaagtatgcgatgtatctatgttatttgtatttatctaaacaatacctctacctctagatattatacaaaa :   7800
Seq2 : gtatgtcttatgtatatttataaaaatgctaagtatgcgatgtatctatgttatttgtatttatctaaacaatacctctacctctagatattatacaaaa :   7800
Seq3 : gtatgtcttatgtatatttataaaaatgctaagtatgcgatgtatctatgttatttgtatttatctaaacaatacctctacctctagatattatacaaaa :   7800
Seq4 : gtatgtcttatgtatatttataaaaatgctaagtatgcgatgtatctatgttatttgtatttatctaaacaatacctctacctctagatattatacaaaa :   7800

              *      7820          *      7840          *      7860          *      7880          *      7900
Seq1 : atttttatttcggcatattaaagtaaaatctagttaccttgaaaatgaatacagtgggtggttccgtatcaccagtaagaacataatagtcgaatacag :   7900
Seq2 : atttttatttcggcatattaaagtaaaatctagttaccttgaaaatgaatacagtgggtggttccgtatcaccagtaagaacataatagtcgaatacag :   7900
Seq3 : atttttatttcggcatattaaagtaaaatctagttaccttgaaaatgaatacagtgggtggttccgtatcaccagtaagaacataatagtcgaatacag :   7900
Seq4 : atttttatttcggcatattaaagtaaaatctagttaccttgaaaatgaatacagtgggtggttccgtatcaccagtaagaacataatagtcgaatacag :   7900

              *      7920          *      7940          *      7960          *      7980          *      8000
Seq1 : tatccgattgagattttgcatacaatactagtctagaaagaaatttgtaatcatcttctgtgacgggagtccatatatctgtatcatcgtctagtttatc :   8000
Seq2 : tatccgattgagattttgcatacaatactagtctagaaagaaatttgtaatcatcttctgtgacgggagtccatatatctgtatcatcgtctagtttatc :   8000
Seq3 : tatccgattgagattttgcatacaatactagtctagaaagaaatttgtaatcatcttctgtgacgggagtccatatatctgtatcatcgtctagtttatc :   8000
Seq4 : tatccgattgagattttgcatacaatactagtctagaaagaaatttgtaatcatcttctgtgacgggagtccatatatctgtatcatcgtctagtttatc :   8000

              *      8020          *      8040          *      8060          *      8080          *      8100
Seq1 : agtgtcccatgctatattcctgttatcatcattagttaatgaaaataactctcgtgcttcagaaaagtcaaatattgtatccatacatacatctccaaaa :   8100
Seq2 : agtgtcccatgctatattcctgttatcatcattagttaatgaaaataactctcgtgcttcagaaaagtcaaatattgtatccatacatacatctccaaaa :   8100
Seq3 : agtgtcccatgctatattcctgttatcatcattagttaatgaaaataactctcgtgcttcagaaaagtcaaatattgtatccatacatacatctccaaaa :   8100
Seq4 : agtgtcccatgctatattcctgttatcatcattagttaatgaaaataactctcgtgcttcagaaaagtcaaatattgtatccatacatacatctccaaaa :   8100

              *      8120          *      8140          *      8160          *      8180          *      8200
Seq1 : ctatcgcttatacgtttatctttaacgatacctatacctagatggttatttactaacagacattttccagatctattgactataactcctatagtttcca :   8200
Seq2 : ctatcgcttatacgtttatctttaacgatacctatacctagatggttatttactaacagacattttccagatctattgactataactcctatagtttcca :   8200
Seq3 : ctatcgcttatacgtttatctttaacgatacctatacctagatggttatttactaacagacattttccagatctattgactataactcctatagtttcca :   8200
Seq4 : ctatcgcttatacgtttatctttaacgatacctatacctagatggttatttactaacagacattttccagatctattgactataactcctatagtttcca :   8200

              *      8220          *      8240          *      8260          *      8280          *      8300
Seq1 : catcaaccaagtaatgatcatctattgttatataacaataacataactcttttccatttttatcagtatgtatatctatatcaacgtcgtcgttgtagtg :   8300
Seq2 : catcaaccaagtaatgatcatctattgttatataacaataacataactcttttccatttttatcagtatgtatatctatatcaacgtcgtcgttgtagtg :   8300
Seq3 : catcaaccaagtaatgatcatctattgttatataacaataacataactcttttccatttttatcagtatgtatatctatatcaacgtcgtcgttgtagtg :   8300
Seq4 : catcaaccaagtaatgatcatctattgttatataacaataacataactcttttccatttttatcagtatgtatatctatatcaacgtcgtcgttgtagtg :   8300

              *      8320          *      8340          *      8360          *      8380          *      8400
Seq1 : aatagtagtcattgatctattatatgaaacggatatgtctagaacggcaattgttttacgtccagttaacactttctttgatttaaagtctagagtcttt :   8400
Seq2 : aatagtagtcattgatctattatatgaaacggatatgtctagaacggcaattgttttacgtccagttaacactttctttgatttaaagtctagagtcttt :   8400
Seq3 : aatagtagtcattgatctattatatgaaacggatatgtctagaacggcaattgttttacgtccagttaacactttctttgatttaaagtctagagtcttt :   8400
Seq4 : aatagtagtcattgatctattatatgaaacggatatgtctagaacggcaattgttttacgtccagttaacactttctttgatttaaagtctagagtcttt :   8400
```

```
                  *      8420         *      8440         *      8460         *      8480         *      8500
Seq1 : gcaaacataatatccttatccgactttatatttcctgtagggtggtataattttattttgcctccacatatcggtgtttccaaatatattactagacaat :   8500
Seq2 : gcaaacataatatccttatccgactttatatttcctgtagggtggtataattttattttgcctccacatatcggtgtttccaaatatattactagacaat :   8500
Seq3 : gcaaacataatatccttatccgactttatatttcctgtagggtggtataattttattttgcctccacatatcggtgtttccaaatatattactagacaat :   8500
Seq4 : gcaaacataatatccttatccgactttatatttcctgtagggtggtataattttattttgcctccacatatcggtgtttccaaatatattactagacaat :   8500

                  *      8520         *      8540         *      8560         *      8580         *      8600
Seq1 : attccatatagttattagttaagggtacccaattagaacacgtacgcttattatcatcatttggatcgtatttcataaaagttattgtactatcgatgtc :   8600
Seq2 : attccatatagttattagttaagggtacccaattagaacacgtacgcttattatcatcatttggatcgtatttcataaaagttattgtactatcgatgtc :   8600
Seq3 : attccatatagttattagttaagggtacccaattagaacacgtacgcttattatcatcatttggatcgtatttcataaaagttattgtactatcgatgtc :   8600
Seq4 : attccatatagttattagttaagggtacccaattagaacacgtacgcttattatcatcatttggatcgtatttcataaaagttattgtactatcgatgtc :   8600

                  *      8620         *      8640         *      8660         *      8680         *      8700
Seq1 : aacacattctacattttttaatcgtctatatagtatttttctgatattttctataatatcagaattgtcttccatcggaagttgtatactatcggaatca :   8700
Seq2 : aacacattctacattttttaatcgtctatatagtatttttctgatattttctataatatcagaattgtcttccatcggaagttgtatactatcggaatca :   8700
Seq3 : aacacattctacattttttaatcgtctatatagtatttttctgatattttctataatatcagaattgtcttccatcggaagttgtatactatcggaatca :   8700
Seq4 : aacacattctacattttttaatcgtctatatagtatttttctgatattttctataatatcagaattgtcttccatcggaagttgtatactatcggaatca :   8700

                  *      8720         *      8740         *      8760         *      8780         *      8800
Seq1 : gttacatgtttaaataattctctgatgtcattccttatacaatcaaattcattattaaacagtttaatagtctgtagacctttatcgtcgtaaatatcca :   8800
Seq2 : gttacatgtttaaataattctctgatgtcattccttatacaatcaaattcattattaaacagtttaatagtctgtagacctttatcgtcgtaaatatcca :   8800
Seq3 : gttacatgtttaaataattctctgatgtcattccttatacaatcaaattcattattaaacagtttaatagtctgtagacctttatcgtcgtaaatatcca :   8800
Seq4 : gttacatgtttaaataattctctgatgtcattccttatacaatcaaattcattattaaacagtttaatagtctgtagacctttatcgtcgtaaatatcca :   8800

                  *      8820         *      8840         *      8860         *      8880         *      8900
Seq1 : ttgtcttattagttacgcttatttttatgtgttttacgttgctttattatattttataagaatgattgtttgacgaatcacgagaactattaagacacat :   8900
Seq2 : ttgtcttattagttacgcttatttttatgtgttttacgttgctttattatattttataagaatgattgtttgacgaatcacgagaactattaagacacat :   8900
Seq3 : ttgtcttattagttacgcttatttttatgtgttttacgttgctttattatattttataagaatgattgtttgacgaatcacgagaactattaagacacat :   8900
Seq4 : ttgtcttattagttacgcttatttttatgtgttttacgttgctttattatattttataagaatgattgtttgacgaatcacgagaactattaagacacat :   8900

                  *      8920         *      8940         *      8960         *      8980         *      9000
Seq1 : tattaggtatatattataaaaaagtttttgattacgatgttataagaggaaagaggacacattaacatcatacatcaattaactacattcttataacatc :   9000
Seq2 : tattaggtatatattataaaaaagtttttgattacgatgttataagaggaaagaggacacattaacatcatacatcaattaactacattcttataacatc :   9000
Seq3 : tattaggtatatattataaaaaagtttttgattacgatgttataagaggaaagaggacacattaacatcatacatcaattaactacattcttataacatc :   9000
Seq4 : tattaggtatatattataaaaaagtttttgattacgatgttataagaggaaagaggacacattaacatcatacatcaattaactacattcttataacatc :   9000

                  *      9020         *      9040         *      9060         *      9080         *      9100
Seq1 : gtaatcaaaagaattgcaattttgatgtataacaactgtcaatgggttatggaattgtatattacatattatacggtatgttggtaacgacaaataccga :   9100
Seq2 : gtaatcaaaagaattgcaattttgatgtataacaactgtcaatgggttatggaattgtatattacatattatacggtatgttggtaacgacaaataccga :   9100
Seq3 : gtaatcaaaagaattgcaattttgatgtataacaactgtcaatgggttatggaattgtatattacatattatacggtatgttggtaacgacaaataccga :   9100
Seq4 : gtaatcaaaagaattgcaattttgatgtataacaactgtcaatgggttatggaattgtatattacatattatacggtatgttggtaacgacaaataccga :   9100
```

```
                 *      9120         *      9140         *      9160         *      9180         *      9200
Seq1 : tcggtaattgtctgccggtgtaatagaattatatatatctatctattacaccggctgagtatgcataataataagttgtggtagtatgatctccatattt :   9200
Seq2 : tcggtaattgtctgccggtgtaatagaattatatatatctatctattacaccggctgagtatgcataataataagttgtggtagtatgatctccatattt :   9200
Seq3 : tcggtaattgtctgccggtgtaatagaattatatatatctatctattacaccggctgagtatgcataataataagttgtggtagtatgatctccatattt :   9200
Seq4 : tcggtaattgtctgccggtgtaatagaattatatatatctatctattacaccggctgagtatgcataataataagttgtggtagtatgatctccatattt :   9200

                 *      9220         *      9240         *      9260         *      9280         *      9300
Seq1 : ataatttaggactttgtattcagtatttttggaatcataaaaaataaaaaaaagttttactaatttaaaatttaaaaagtatttacatttttttcactgt :   9300
Seq2 : ataatttaggactttgtattcagtatttttggaatcataaaaaataaaaaaaagttttactaatttaaaatttaaaaagtatttacatttttttcactgt :   9300
Seq3 : ataatttaggactttgtattcagtatttttggaatcataaaaaataaaaaaaagttttactaatttaaaatttaaaaagtatttacatttttttcactgt :   9300
Seq4 : ataatttaggactttgtattcagtatttttggaatcataaaaaataaaaaaaagttttactaatttaaaatttaaaaagtatttacatttttttcactgt :   9300

                 *      9320         *      9340         *      9360         *      9380         *      9400
Seq1 : ttagtcgcggatatggaattcgatcctgccaaaatcaatacatcatctatagatcatgtaacaatattacaatacatagatgaaccaaatgatataagac :   9400
Seq2 : ttagtcgcggatatggaattcgatcctgccaaaatcaatacatcatctatagatcatgtaacaatattacaatacatagatgaaccaaatgatataagac :   9400
Seq3 : ttagtcgcggatatggaattcgatcctgccaaaatcaatacatcatctatagatcatgtaacaatattacaatacatagatgaaccaaatgatataagac :   9400
Seq4 : ttagtcgcggatatggaattcgatcctgccaaaatcaatacatcatctatagatcatgtaacaatattacaatacatagatgaaccaaatgatataagac :   9400

                 *      9420         *      9440         *      9460         *      9480         *      9500
Seq1 : taacagtatgcattatccgaaatattaataacattacatattatatcaatatcacaaaaataaatacacatttggctaatcaatttcgggcttggaaaaa :   9500
Seq2 : taacagtatgcattatccgaaatattaataacattacatattatatcaatatcacaaaaataaatacacatttggctaatcaatttcgggcttggaaaaa :   9500
Seq3 : taacagtatgcattatccgaaatattaataacattacatattatatcaatatcacaaaaataaatacacatttggctaatcaatttcgggcttggaaaaa :   9500
Seq4 : taacagtatgcattatccgaaatattaataacattacatattatatcaatatcacaaaaataaatacacatttggctaatcaatttcgggcttggaaaaa :   9500

                 *      9520         *      9540         *      9560         *      9580         *      9600
Seq1 : acgtatcgccggaagggactatatgactaacttatctagagatacaggaatacaacaatcaaaacttactgaaactatacgtaactgtcaaaaaaataga :   9600
Seq2 : acgtatcgccggaagggactatatgactaacttatctagagatacaggaatacaacaatcaaaacttactgaaactatacgtaactgtcaaaaaaataga :   9600
Seq3 : acgtatcgccggaagggactatatgactaacttatctagagatacaggaatacaacaatcaaaacttactgaaactatacgtaactgtcaaaaaaataga :   9600
Seq4 : acgtatcgccggaagggactatatgactaacttatctagagatacaggaatacaacaatcaaaacttactgaaactatacgtaactgtcaaaaaaataga :   9600

                 *      9620         *      9640         *      9660         *      9680         *      9700
Seq1 : aacatatatggtctatatatacactacaatttagttattaatgtggttattgattggataaccgatgtgattgttcaatcaatattaagagggttggtaa :   9700
Seq2 : aacatatatggtctatatatacactacaatttagttattaatgtggttattgattggataaccgatgtgattgttcaatcaatattaagagggttggtaa :   9700
Seq3 : aacatatatggtctatatatacactacaatttagttattaatgtggttattgattggataaccgatgtgattgttcaatcaatattaagagggttggtaa :   9700
Seq4 : aacatatatggtctatatatacactacaatttagttattaatgtggttattgattggataaccgatgtgattgttcaatcaatattaagagggttggtaa :   9700

                 *      9720         *      9740         *      9760         *      9780         *      9800
Seq1 : attggtacatagctaataatacctatacacccaataatacaacaaccatttctgagttggatatcatcaaaatactggataaatacgaggacgtgtatag :   9800
Seq2 : attggtacatagctaataatacctatacacccaataatacaacaaccatttctgagttggatatcatcaaaatactggataaatacgaggacgtgtatag :   9800
Seq3 : attggtacatagctaataatacctatacacccaataatacaacaaccatttctgagttggatatcatcaaaatactggataaatacgaggacgtgtatag :   9800
Seq4 : attggtacatagctaataatacctatacacccaataatacaacaaccatttctgagttggatatcatcaaaatactggataaatacgaggacgtgtatag :   9800
```

```
                 *        9820          *        9840          *        9860          *        9880          *        9900
Seq1 : agtaagtaaagaaaaagaatgtggaatttgctatgaagttgtttactcaaaacgatagatactttggtttattggattcgtgtactcatatattttgcat :   9900
Seq2 : agtaagtaaagaaaaagaatgtggaatttgctatgaagttgtttactcaaaacgatagatactttggtttattggattcgtgtactcatatattttgcat :   9900
Seq3 : agtaagtaaagaaaaagaatgtggaatttgctatgaagttgtttactcaaaacgatagatactttggtttattggattcgtgtactcatatattttgcat :   9900
Seq4 : agtaagtaaagaaaaagaatgtggaatttgctatgaagttgtttactcaaaacgatagatactttggtttattggattcgtgtactcatatattttgcat :   9900

                 *        9920          *        9940          *        9960          *        9980          *       10000
Seq1 : aacatgcatcaatatatggcataaaacacgaagagaaaccggtgcgtcggataattgtcctatatgtcgtacccgttttagaaacataacaatgagcaag :  10000
Seq2 : aacatgcatcaatatatggcataaaacacgaagagaaaccggtgcgtcggataattgtcctatatgtcgtacccgttttagaaacataacaatgagcaag :  10000
Seq3 : aacatgcatcaatatatggcataaaacacgaagagaaaccggtgcgtcggataattgtcctatatgtcgtacccgttttagaaacataacaatgagcaag :  10000
Seq4 : aacatgcatcaatatatggcataaaacacgaagagaaaccggtgcgtcggataattgtcctatatgtcgtacccgttttagaaacataacaatgagcaag :  10000

                 *       10020          *       10040          *       10060          *       10080          *       10100
Seq1 : ttctataagctagttaactaataaataaaaagtttaatttgttgacgacgtatgtcgttatttttctcgtatgaaagattaaattcaattcaattcgttg :  10100
Seq2 : ttctataagctagttaactaataaataaaaagtttaatttgttgacgacgtatgtcgttatttttctcgtatgaaagattaaattcaattcaattcgttg :  10100
Seq3 : ttctataagctagttaactaataaataaaaagtttaatttgttgacgacgtatgtcgttatttttctcgtatgaaagattaaattcaattcaattcgttg :  10100
Seq4 : ttctataagctagttaactaataaataaaaagtttaatttgttgacgacgtatgtcgttatttttctcgtatgaaagattaaattcaattcaattcgttg :  10100

                 *       10120          *       10140          *       10160          *       10180          *       10200
Seq1 : tttctaatataatctgccgtattggatggattctcaagacaattgcatttagattatattatcatgaataaaaatagtagcacgcactacttcagccaaa :  10200
Seq2 : tttctaatataatctgccgtattggatggattctcaagacaattgcatttagattatattatcatgaataaaaatagtagcacgcactacttcagccaaa :  10200
Seq3 : tttctaatataatctgccgtattggatggattctcaagacaattgcatttagattatattatcatgaataaaaatagtagcacgcactacttcagccaaa :  10200
Seq4 : tttctaatataatctgccgtattggatggattctcaagacaattgcatttagattatattatcatgaataaaaatagtagcacgcactacttcagccaaa :  10200

                 *       10220          *       10240          *       10260          *       10280          *       10300
Seq1 : tattctttttgaaacgccatctatcgtagtgaggacacaagtgaacctataattatcaaatttattagtatcagtcacatgaaggactttctgtagagt :  10300
Seq2 : tattctttttgaaacgccatctatcgtagtgaggacacaagtgaacctataattatcaaatttattagtatcagtcacatgaaggactttctgtagagt :  10300
Seq3 : tattctttttgaaacgccatctatcgtagtgaggacacaagtgaacctataattatcaaatttattagtatcagtcacatgaaggactttctgtagagt :  10300
Seq4 : tattctttttgaaacgccatctatcgtagtgaggacacaagtgaacctataattatcaaatttattagtatcagtcacatgaaggactttctgtagagt :  10300

                 *       10320          *       10340          *       10360          *       10380          *       10400
Seq1 : gacgattctaccatctatggtactaacggtttcatcctccttgataccctcacccaaatgttctataaatttagcatcctcgtccgatctcatatccttt :  10400
Seq2 : gacgattctaccatctatggtactaacggtttcatcctccttgataccctcacccaaatgttctataaatttagcatcctcgtccgatctcatatccttt :  10400
Seq3 : gacgattctaccatctatggtactaacggtttcatcctccttgataccctcacccaaatgttctataaatttagcatcctcgtccgatctcatatccttt :  10400
Seq4 : gacgattctaccatctatggtactaacggtttcatcctccttgataccctcacccaaatgttctataaatttagcatcctcgtccgatctcatatccttt :  10400

                 *       10420          *       10440          *       10460          *       10480          *       10500
Seq1 : gccaaccaatacatgtagctaaaattaggcataaatttcacacatccagtgcaacgaaattctccagaagatgttacgatgtttaggttaggacatttga :  10500
Seq2 : gccaaccaatacatgtagctaaaattaggcataaatttcacacatccagtgcaacgaaattctccagaagatgttacgatgtttaggttaggacatttga :  10500
Seq3 : gccaaccaatacatgtagctaaaattaggcataaatttcacacatccagtgcaacgaaattctccagaagatgttacgatgtttaggttaggacatttga :  10500
Seq4 : gccaaccaatacatgtagctaaaattaggcataaatttcacacatccagtgcaacgaaattctccagaagatgttacgatgtttaggttaggacatttga :  10500
```

```
              *     10520          *     10540          *     10560          *     10580          *     10600
Seq1 : tttcgtcggcattaacatatgggtgaacacacccatacatgaaagcgatgagaaataggattctcatcttgccaaaatatcactagaaaaaatttattta :  10600
Seq2 : tttcgtcggcattaacatatgggtgaacacacccatacatgaaagcgatgagaaataggattctcatcttgccaaaatatcactagaaaaaatttattta :  10600
Seq3 : tttcgtcggcattaacatatgggtgaacacacccatacatgaaagcgatgagaaataggattctcatcttgccaaaatatcactagaaaaaatttattta :  10600
Seq4 : tttcgtcggcattaacatatgggtgaacacacccatacatgaaagcgatgagaaataggattctcatcttgccaaaatatcactagaaaaaatttattta :  10600

              *     10620          *     10640          *     10660          *     10680          *     10700
Seq1 : tcaattttaaaggtataaaaaatacttattgttgctcgaatattttgtatttgatggtatacggaagattagaaatgtaggtattatcatcaactgattc :  10700
Seq2 : tcaattttaaaggtataaaaaatacttattgttgctcgaatattttgtatttgatggtatacggaagattagaaatgtaggtattatcatcaactgattc :  10700
Seq3 : tcaattttaaaggtataaaaaatacttattgttgctcgaatattttgtatttgatggtatacggaagattagaaatgtaggtattatcatcaactgattc :  10700
Seq4 : tcaattttaaaggtataaaaaatacttattgttgctcgaatattttgtatttgatggtatacggaagattagaaatgtaggtattatcatcaactgattc :  10700

              *     10720          *     10740          *     10760          *     10780          *     10800
Seq1 : tatggttttatgtattctatcatgtttcactattgcgtcggaaataatatcatatgcttccacatatattttattttgttttaactcataatactcacgt :  10800
Seq2 : tatggttttatgtattctatcatgtttcactattgcgtcggaaataatatcatatgcttccacatatattttattttgttttaactcataatactcacgt :  10800
Seq3 : tatggttttatgtattctatcatgtttcactattgcgtcggaaataatatcatatgcttccacatatattttattttgttttaactcataatactcacgt :  10800
Seq4 : tatggttttatgtattctatcatgtttcactattgcgtcggaaataatatcatatgcttccacatatattttattttgttttaactcataatactcacgt :  10800

              *     10820          *     10840          *     10860          *     10880          *     10900
Seq1 : aattctggattattggcatatctatgaataattttagctccatgatcagtaaatattaatgagaacatagtattaccacctaccattattttttttcattt :  10900
Seq2 : aattctggattattggcatatctatgaataattttagctccatgatcagtaaatattaatgagaacatagtattaccacctaccattattttttttcattt :  10900
Seq3 : aattctggattattggcatatctatgaataattttagctccatgatcagtaaatattaatgagaacatagtattaccacctaccattattttttttcattt :  10900
Seq4 : aattctggattattggcatatctatgaataattttagctccatgatcagtaaatattaatgagaacatagtattaccacctaccattattttttttcattt :  10900

              *     10920          *     10940          *     10960          *     10980          *     11000
Seq1 : cgttcaattcttgattgcaaagatctatataatcattatagcgttgacttatggactctggaatcttagacgatgtacagtcatctataatcatggcata :  11000
Seq2 : cgttcaattcttgattgcaaagatctatataatcattatagcgttgacttatggactctggaatcttagacgatgtacagtcatctataatcatggcata :  11000
Seq3 : cgttcaattcttgattgcaaagatctatataatcattatagcgttgacttatggactctggaatcttagacgatgtacagtcatctataatcatggcata :  11000
Seq4 : cgttcaattcttgattgcaaagatctatataatcattatagcgttgacttatggactctggaatcttagacgatgtacagtcatctataatcatggcata :  11000

              *     11020          *     11040          *     11060          *     11080          *     11100
Seq1 : tttaatacattgttttatagcatagtagttatctacgatgttagatatttctctcaatgaatcaatcacacaatctaatgtaggtttatgacataatagc :  11100
Seq2 : tttaatacattgttttatagcatagtagttatctacgatgttagatatttctctcaatgaatcaatcacacaatctaatgtaggtttatgacataatagc :  11100
Seq3 : tttaatacattgttttatagcatagtagttatctacgatgttagatatttctctcaatgaatcaatcacacaatctaatgtaggtttatgacataatagc :  11100
Seq4 : tttaatacattgttttatagcatagtagttatctacgatgttagatatttctctcaatgaatcaatcacacaatctaatgtaggtttatgacataatagc :  11100

              *     11120          *     11140          *     11160          *     11180          *     11200
Seq1 : attttcagcagttcaatgtttctagattcgttgatggcaatggctatacatgtatatccgttatttgatctaatgttgacatctgaaccggattctagca :  11200
Seq2 : attttcagcagttcaatgtttctagattcgttgatggcaatggctatacatgtatatccgttatttgatctaatgttgacatctgaaccggattctagca :  11200
Seq3 : attttcagcagttcaatgtttctagattcgttgatggcaatggctatacatgtatatccgttatttgatctaatgttgacatctgaaccggattctagca :  11200
Seq4 : attttcagcagttcaatgtttctagattcgttgatggcaatggctatacatgtatatccgttatttgatctaatgttgacatctgaaccggattctagca :  11200
```

```
                 *     11220         *     11240         *     11260         *     11280         *     11300
Seq1 : gtaaagatactagagattgtttattatatctaacagccttgtgaagaagtgtttctcctcgtttgtcaatcatgttaatgtctttaagataaggtaggca :  11300
Seq2 : gtaaagatactagagattgtttattatatctaacagccttgtgaagaagtgtttctcctcgtttgtcaatcatgttaatgtctttaagataaggtaggca :  11300
Seq3 : gtaaagatactagagattgtttattatatctaacagccttgtgaagaagtgtttctcctcgtttgtcaatcatgttaatgtctttaagataaggtaggca :  11300
Seq4 : gtaaagatactagagattgtttattatatctaacagccttgtgaagaagtgtttctcctcgtttgtcaatcatgttaatgtctttaagataaggtaggca :  11300

                 *     11320         *     11340         *     11360         *     11380         *     11400
Seq1 : aatgtttatagtactaagaattggcaagcataagacatgtcacaaagacccttttttgtatgtataagtgtaaaaattataacattcatagttggattta :  11400
Seq2 : aatgtttatagtactaagaattggcaagcataagacatgtcacaaagacccttttttgtatgtataagtgtaaaaattataacattcatagttggattta :  11400
Seq3 : aatgtttatagtactaagaattggcaagcataagacatgtcacaaagacccttttttgtatgtataagtgtaaaaattataacattcatagttggattta :  11400
Seq4 : aatgtttatagtactaagaattggcaagcataagacatgtcacaaagacccttttttgtatgtataagtgtaaaaattataacattcatagttggattta :  11400

                 *     11420         *     11440         *     11460         *     11480         *     11500
Seq1 : cataggtgtccaatcgggatctctccatcatcgagataattgatggcatctcccttccttttttagtagatatttcatcgtgtaagaatcaatattaata :  11500
Seq2 : cataggtgtccaatcgggatctctccatcatcgagataattgatggcatctcccttccttttttagtagatatttcatcgtgtaagaatcaatattaata :  11500
Seq3 : cataggtgtccaatcgggatctctccatcatcgagataattgatggcatctcccttccttttttagtagatatttcatcgtgtaagaatcaatattaata :  11500
Seq4 : cataggtgtccaatcgggatctctccatcatcgagataattgatggcatctcccttccttttttagtagatatttcatcgtgtaagaatcaatattaata :  11500

                 *     11520         *     11540         *     11560         *     11580         *     11600
Seq1 : tttctaaagtattcgtgtatagcctctttatttaccacagttccatattccactagaggatatcgccgaatgtcatatactcaattagtatatgttgga :  11600
Seq2 : tttctaaagtattcgtgtatagcctctttatttaccacagttccatattccactagaggatatcgccgaatgtcatatactcaattagtatatgttgga :  11600
Seq3 : tttctaaagtattcgtgtatagcctctttatttaccacagttccatattccactagaggatatcgccgaatgtcatatactcaattagtatatgttgga :  11600
Seq4 : tttctaaagtattcgtgtatagcctctttatttaccacagttccatattccactagaggatatcgccgaatgtcatatactcaattagtatatgttgga :  11600

                 *     11620         *     11640         *     11660         *     11680         *     11700
Seq1 : ggacatccgagttcattgttttcaatatcaaaaagatggtttccttatcatttctccatagtggtacaatactacacattattccgtgcggctttccatt :  11700
Seq2 : ggacatccgagttcattgttttcaatatcaaaaagatggtttccttatcatttctccatagtggtacaatactacacattattccgtgcggctttccatt :  11700
Seq3 : ggacatccgagttcattgttttcaatatcaaaaagatggtttccttatcatttctccatagtggtacaatactacacattattccgtgcggctttccatt :  11700
Seq4 : ggacatccgagttcattgttttcaatatcaaaaagatggtttccttatcatttctccatagtggtacaatactacacattattccgtgcggctttccatt :  11700

                 *     11720         *     11740         *     11760         *     11780         *     11800
Seq1 : ttccaaaaacaatttgaccaaatctaaatctacatctttattgtatctataatcactatttagataatcagccataattactcgagtgcaacatgttaga :  11800
Seq2 : ttccaaaaacaatttgaccaaatctaaatctacatctttattgtatctataatcactatttagataatcagccataattactcgagtgcaacatgttaga :  11800
Seq3 : ttccaaaaacaatttgaccaaatctaaatctacatctttattgtatctataatcactatttagataatcagccataattactcgagtgcaacatgttaga :  11800
Seq4 : ttccaaaaacaatttgaccaaatctaaatctacatctttattgtatctataatcactatttagataatcagccataattactcgagtgcaacatgttaga :  11800

                 *     11820         *     11840         *     11860         *     11880         *     11900
Seq1 : tcgtctatatatgaataagcagtgttatctattcctttcattaacaatttaacgatgtctatatctatatgagatgacttaatataatattgaagagctg :  11900
Seq2 : tcgtctatatatgaataagcagtgttatctattcctttcattaacaatttaacgatgtctatatctatatgagatgacttaatataatattgaagagctg :  11900
Seq3 : tcgtctatatatgaataagcagtgttatctattcctttcattaacaatttaacgatgtctatatctatatgagatgacttaatataatattgaagagctg :  11900
Seq4 : tcgtctatatatgaataagcagtgttatctattcctttcattaacaatttaacgatgtctatatctatatgagatgacttaatataatattgaagagctg :  11900
```

```
                *      11920          *      11940          *      11960          *      11980          *      12000
Seq1 : tacaatagttttatctatagaagacggcttgattccgtgattaattagacatttaacaacttccggacgcacatatgctctcgtatccgactttgaata :  12000
Seq2 : tacaatagttttatctatagaagacggcttgattccgtgattaattagacatttaacaacttccggacgcacatatgctctcgtatccgactttgaata :  12000
Seq3 : tacaatagttttatctatagaagacggcttgattccgtgattaattagacatttaacaacttccggacgcacatatgctctcgtatccgactttgaata :  12000
Seq4 : tacaatagttttatctatagaagacggcttgattccgtgattaattagacatttaacaacttccggacgcacatatgctctcgtatccgactttgaata :  12000

                *      12020          *      12040          *      12060          *      12080          *      12100
Seq1 : cagatgagagatgatatacagatgcaatacggtaccgcaatttcgtagttgataatcatcatacgcgtatcagtactcgtcctcataaagaacactgcag :  12100
Seq2 : cagatgagagatgatatacagatgcaatacggtaccgcaatttcgtagttgataatcatcatacgcgtatcagtactcgtcctcataaagaacactgcag :  12100
Seq3 : cagatgagagatgatatacagatgcaatacggtaccgcaatttcgtagttgataatcatcatacgcgtatcagtactcgtcctcataaagaacactgcag :  12100
Seq4 : cagatgagagatgatatacagatgcaatacggtaccgcaatttcgtagttgataatcatcatacgcgtatcagtactcgtcctcataaagaacactgcag :  12100

                *      12120          *      12140          *      12160          *      12180          *      12200
Seq1 : ccattttctatgaacaaatcaataattttaggaacaggatcattgtcattacataattttctataactgaacgatggttttcacatttaacactcaagtc :  12200
Seq2 : ccattttctatgaacaaatcaataattttaggaacaggatcattgtcattacataattttctataactgaacgatggttttcacatttaacactcaagtc :  12200
Seq3 : ccattttctatgaacaaatcaataattttaggaacaggatcattgtcattacataattttctataactgaacgatggttttcacatttaacactcaagtc :  12200
Seq4 : ccattttctatgaacaaatcaataattttaggaacaggatcattgtcattacataattttctataactgaacgatggttttcacatttaacactcaagtc :  12200

                *      12220          *      12240          *      12260          *      12280          *      12300
Seq1 : aaatccatgttctaccaacacctttatcaagtcaacgtctacattttggatttcatatagctgaatatattaaagtcatttatgttgctaaatccagtg :  12300
Seq2 : aaatccatgttctaccaacacctttatcaagtcaacgtctacattttggatttcatatagctgaatatattaaagtcatttatgttgctaaatccagtg :  12300
Seq3 : aaatccatgttctaccaacacctttatcaagtcaacgtctacattttggatttcatatagctgaatatattaaagtcatttatgttgctaaatccagtg :  12300
Seq4 : aaatccatgttctaccaacacctttatcaagtcaacgtctacattttggatttcatatagctgaatatattaaagtcatttatgttgctaaatccagtg :  12300

                *      12320          *      12340          *      12360          *      12380          *      12400
Seq1 : gcttctagtagagccatcgctatatcctttaactttaacatgtctactatttgtgtattcttctaatggggtagctgtctccaatttttgcgtaatggat :  12400
Seq2 : gcttctagtagagccatcgctatatcctttaactttaacatgtctactatttgtgtattcttctaatggggtagctgtctccaatttttgcgtaatggat :  12400
Seq3 : gcttctagtagagccatcgctatatcctttaactttaacatgtctactatttgtgtattcttctaatggggtagctgtctccaatttttgcgtaatggat :  12400
Seq4 : gcttctagtagagccatcgctatatcctttaactttaacatgtctactatttgtgtattcttctaatggggtagctgtctccaatttttgcgtaatggat :  12400

                *      12420          *      12440          *      12460          *      12480          *      12500
Seq1 : tagtgccactgtctagtagtagtttgacgacctcgacattattacaatgctcattaaaaaggtatgcgtgtaaagcattattcttgaattggttcctggt :  12500
Seq2 : tagtgccactgtctagtagtagtttgacgacctcgacattattacaatgctcattaaaaaggtatgcgtgtaaagcattattcttgaattggttcctggt :  12500
Seq3 : tagtgccactgtctagtagtagtttgacgacctcgacattattacaatgctcattaaaaaggtatgcgtgtaaagcattattcttgaattggttcctggt :  12500
Seq4 : tagtgccactgtctagtagtagtttgacgacctcgacattattacaatgctcattaaaaaggtatgcgtgtaaagcattattcttgaattggttcctggt :  12500

                *      12520          *      12540          *      12560          *      12580          *      12600
Seq1 : atcattaggatctctgtctctcaacatctgtttaagttcatcgagagccacctcctcattttccagatagtcaaacattttgactgaatgagctactgtg :  12600
Seq2 : atcattaggatctctgtctctcaacatctgtttaagttcatcgagagccacctcctcattttccagatagtcaaacattttgactgaatgagctactgtg :  12600
Seq3 : atcattaggatctctgtctctcaacatctgtttaagttcatcgagagccacctcctcattttccagatagtcaaacattttgactgaatgagctactgtg :  12600
Seq4 : atcattaggatctctgtctctcaacatctgtttaagttcatcgagagccacctcctcattttccagatagtcaaacattttgactgaatgagctactgtg :  12600
```

```
                *     12620         *     12640         *     12660         *     12680         *     12700
Seq1 : aactctatacacccacacaactaatgtcattaaatattatttttttgaatgtatttataccatgtcaaaaacttgtacaattattaataaaaataattta :  12700
Seq2 : aactctatacacccacacaactaatgtcattaaatattatttttttgaatgtatttataccatgtcaaaaacttgtacaattattaataaaaataattta :  12700
Seq3 : aactctatacacccacacaactaatgtcattaaatattatttttttgaatgtatttataccatgtcaaaaacttgtacaattattaataaaaataattta :  12700
Seq4 : aactctatacacccacacaactaatgtcattaaatattatttttttgaatgtatttataccatgtcaaaaacttgtacaattattaataaaaataattta :  12700

                *     12720         *     12740         *     12760         *     12780         *     12800
Seq1 : gtgtttaaattttaccagttccagattttacacctccgttaaccccactttttacaccactggacgatcctcctccccacattccaccgccaccagatgt :  12800
Seq2 : gtgtttaaattttaccagttccagattttacacctccgttaaccccactttttacaccactggacgatcctcctccccacattccaccgccaccagatgt :  12800
Seq3 : gtgtttaaattttaccagttccagattttacacctccgttaaccccactttttacaccactggacgatcctcctccccacattccaccgccaccagatgt :  12800
Seq4 : gtgtttaaattttaccagttccagattttacacctccgttaaccccactttttacaccactggacgatcctcctccccacattccaccgccaccagatgt :  12800

                *     12820         *     12840         *     12860         *     12880         *     12900
Seq1 : ataagttttagatcctttattactaccatcatgtccatggataaagacactccacatgccgccactactaccccctttagaagacatattaataagactt :  12900
Seq2 : ataagttttagatcctttattactaccatcatgtccatggataaagacactccacatgccgccactactaccccctttagaagacatattaataagactt :  12900
Seq3 : ataagttttagatcctttattactaccatcatgtccatggataaagacactccacatgccgccactactaccccctttagaagacatattaataagactt :  12900
Seq4 : ataagttttagatcctttattactaccatcatgtccatggataaagacactccacatgccgccactactaccccctttagaagacatattaataagactt :  12900

                *     12920         *     12940         *     12960         *     12980         *     13000
Seq1 : aaggacaagtttaacaataaaattaatcacgagtaccctactaccaacctacactattatatgattatagtttctatttttacagtaccttaactaaagt :  13000
Seq2 : aaggacaagtttaacaataaaattaatcacgagtaccctactaccaacctacactattatatgattatagtttctatttttacagtaccttaactaaagt :  13000
Seq3 : aaggacaagtttaacaataaaattaatcacgagtaccctactaccaacctacactattatatgattatagtttctatttttacagtaccttaactaaagt :  13000
Seq4 : aaggacaagtttaacaataaaattaatcacgagtaccctactaccaacctacactattatatgattatagtttctatttttacagtaccttaactaaagt :  13000

                *     13020         *     13040         *     13060         *     13080         *     13100
Seq1 : ctctagtcacaagagcaatactaccaacctacactattatatgattatagtttctatttttataggaacgcgtacgagaaaatcaaatgtctaatttcta :  13100
Seq2 : ctctagtcacaagagcaatactaccaacctacactattatatgattatagtttctatttttataggaacgcgtacgagaaaatcaaatgtctaatttcta :  13100
Seq3 : ctctagtcacaagagcaatactaccaacctacactattatatgattatagtttctatttttataggaacgcgtacgagaaaatcaaatgtctaatttcta :  13100
Seq4 : ctctagtcacaagagcaatactaccaacctacactattatatgattatagtttctatttttataggaacgcgtacgagaaaatcaaatgtctaatttcta :  13100

                *     13120         *     13140         *     13160         *     13180         *     13200
Seq1 : acggtagtgttgataaacgattatcgtcaatggatacctcctctatcatgtcgtctattttcttactttgttctattaacttattagcattatatattat :  13200
Seq2 : acggtagtgttgataaacgattatcgtcaatggatacctcctctatcatgtcgtctattttcttactttgttctattaacttattagcattatatattat :  13200
Seq3 : acggtagtgttgataaacgattatcgtcaatggatacctcctctatcatgtcgtctattttcttactttgttctattaacttattagcattatatattat :  13200
Seq4 : acggtagtgttgataaacgattatcgtcaatggatacctcctctatcatgtcgtctattttcttactttgttctattaacttattagcattatatattat :  13200

                *     13220         *     13240         *     13260         *     13280         *     13300
Seq1 : ttgattataaaacttatattgcttattagcccaatctgtaaatatcggattattaacatatcgtttctttgtaggtttatttaacatgtacatcactgta :  13300
Seq2 : ttgattataaaacttatattgcttattagcccaatctgtaaatatcggattattaacatatcgtttctttgtaggtttatttaacatgtacatcactgta :  13300
Seq3 : ttgattataaaacttatattgcttattagcccaatctgtaaatatcggattattaacatatcgtttctttgtaggtttatttaacatgtacatcactgta :  13300
Seq4 : ttgattataaaacttatattgcttattagcccaatctgtaaatatcggattattaacatatcgtttctttgtaggtttatttaacatgtacatcactgta :  13300
```

```
                *      13320          *      13340          *      13360          *      13380          *      13400
Seq1 : agcatgtccgtaccatttattttaatttgacgcatatccgcaatttctttttcgcagtcggttataaattctatatatgatggatacatgctacatgtgt :  13400
Seq2 : agcatgtccgtaccatttattttaatttgacgcatatccgcaatttctttttcgcagtcggttataaattctatatatgatggatacatgctacatgtgt :  13400
Seq3 : agcatgtccgtaccatttattttaatttgacgcatatccgcaatttctttttcgcagtcggttataaattctatatatgatggatacatgctacatgtgt :  13400
Seq4 : agcatgtccgtaccatttattttaatttgacgcatatccgcaatttctttttcgcagtcggttataaattctatatatgatggatacatgctacatgtgt :  13400

                *      13420          *      13440          *      13460          *      13480          *      13500
Seq1 : acttataatcgactaatatgaagtacttgatacatattttcagtaacgatttattattaccacctatgaataagtacctgtgatcgtctaggtaatcaac :  13500
Seq2 : acttataatcgactaatatgaagtacttgatacatattttcagtaacgatttattattaccacctatgaataagtacctgtgatcgtctaggtaatcaac :  13500
Seq3 : acttataatcgactaatatgaagtacttgatacatattttcagtaacgatttattattaccacctatgaataagtacctgtgatcgtctaggtaatcaac :  13500
Seq4 : acttataatcgactaatatgaagtacttgatacatattttcagtaacgatttattattaccacctatgaataagtacctgtgatcgtctaggtaatcaac :  13500

                *      13520          *      13540          *      13560          *      13580          *      13600
Seq1 : tgttttcttaatacattcgatggttggtaatttactcagaataatttccaatatcttaatatataattctgctatttctgggatatatttatctgccagt :  13600
Seq2 : tgttttcttaatacattcgatggttggtaatttactcagaataatttccaatatcttaatatataattctgctatttctgggatatatttatctgccagt :  13600
Seq3 : tgttttcttaatacattcgatggttggtaatttactcagaataatttccaatatcttaatatataattctgctatttctgggatatatttatctgccagt :  13600
Seq4 : tgttttcttaatacattcgatggttggtaatttactcagaataatttccaatatcttaatatataattctgctatttctgggatatatttatctgccagt :  13600

                *      13620          *      13640          *      13660          *      13680          *      13700
Seq1 : ataacacaaatagtaatacatgtaaacccatatttgttattatattaatgtctgcgccattatctattaaccattctactaggctgacactatgcgact :  13700
Seq2 : ataacacaaatagtaatacatgtaaacccatatttgttattatattaatgtctgcgccattatctattaaccattctactaggctgacactatgcgact :  13700
Seq3 : ataacacaaatagtaatacatgtaaacccatatttgttattatattaatgtctgcgccattatctattaaccattctactaggctgacactatgcgact :  13700
Seq4 : ataacacaaatagtaatacatgtaaacccatatttgttattatattaatgtctgcgccattatctattaaccattctactaggctgacactatgcgact :  13700

                *      13720          *      13740          *      13760          *      13780          *      13800
Seq1 : taatacaatgataaagtatactacatccatgtttatctatttgtttatatcatcaatatacggcttacaaagttttagtatcgataacacatccaactc :  13800
Seq2 : taatacaatgataaagtatactacatccatgtttatctatttgtttatatcatcaatatacggcttacaaagttttagtatcgataacacatccaactc :  13800
Seq3 : taatacaatgataaagtatactacatccatgtttatctatttgtttatatcatcaatatacggcttacaaagttttagtatcgataacacatccaactc :  13800
Seq4 : taatacaatgataaagtatactacatccatgtttatctatttgtttatatcatcaatatacggcttacaaagttttagtatcgataacacatccaactc :  13800

                *      13820          *      13840          *      13860          *      13880          *      13900
Seq1 : acgcatagagaaggtagggaataatggcataatatttattaggttatcatcattgtcattatctacaactaagtttccatttttaaaatatactcgaca :  13900
Seq2 : acgcatagagaaggtagggaataatggcataatatttattaggttatcatcattgtcattatctacaactaagtttccatttttaaaatatactcgaca :  13900
Seq3 : acgcatagagaaggtagggaataatggcataatatttattaggttatcatcattgtcattatctacaactaagtttccatttttaaaatatactcgaca :  13900
Seq4 : acgcatagagaaggtagggaataatggcataatatttattaggttatcatcattgtcattatctacaactaagtttccatttttaaaatatactcgaca :  13900

                *      13920          *      13940          *      13960          *      13980          *      14000
Seq1 : actttaggatctctattgccaaattttgaaaatatttatttatatgcttaaatctatataatgtagctccttcatcaatcatacatttaataacattga :  14000
Seq2 : actttaggatctctattgccaaattttgaaaatatttatttatatgcttaaatctatataatgtagctccttcatcaatcatacatttaataacattga :  14000
Seq3 : actttaggatctctattgccaaattttgaaaatatttatttatatgcttaaatctatataatgtagctccttcatcaatcatacatttaataacattga :  14000
Seq4 : actttaggatctctattgccaaattttgaaaatatttatttatatgcttaaatctatataatgtagctccttcatcaatcatacatttaataacattga :  14000
```

```
                *     14020         *     14040         *     14060         *     14080         *     14100
Seq1 : tgtatactgtatgataagatacatattctaacaatagatcttgtatagaatctgtatatcttttaagaattgtggatattaggatattattacgtaaact :  14100
Seq2 : tgtatactgtatgataagatacatattctaacaatagatcttgtatagaatctgtatatcttttaagaattgtggatattaggatattattacgtaaact :  14100
Seq3 : tgtatactgtatgataagatacatattctaacaatagatcttgtatagaatctgtatatcttttaagaattgtggatattaggatattattacgtaaact :  14100
Seq4 : tgtatactgtatgataagatacatattctaacaatagatcttgtatagaatctgtatatcttttaagaattgtggatattaggatattattacgtaaact :  14100

                *     14120         *     14140         *     14160         *     14180         *     14200
Seq1 : attacacaattctaaaatataaaacgtatcacggtcgaataatagttgatcaactatataattatcgattttgtgatttttcttcctaaactgtttacgt :  14200
Seq2 : attacacaattctaaaatataaaacgtatcacggtcgaataatagttgatcaactatataattatcgattttgtgatttttcttcctaaactgtttacgt :  14200
Seq3 : attacacaattctaaaatataaaacgtatcacggtcgaataatagttgatcaactatataattatcgattttgtgatttttcttcctaaactgtttacgt :  14200
Seq4 : attacacaattctaaaatataaaacgtatcacggtcgaataatagttgatcaactatataattatcgattttgtgatttttcttcctaaactgtttacgt :  14200

                *     14220         *     14240         *     14260         *     14280         *     14300
Seq1 : aaatagttagatagaatattcattagttcatgaccactatagttactatcgaataacgcgtcaaatatttcccgtttaatatcgcatttgtcaagataat :  14300
Seq2 : aaatagttagatagaatattcattagttcatgaccactatagttactatcgaataacgcgtcaaatatttcccgtttaatatcgcatttgtcaagataat :  14300
Seq3 : aaatagttagatagaatattcattagttcatgaccactatagttactatcgaataacgcgtcaaatatttcccgtttaatatcgcatttgtcaagataat :  14300
Seq4 : aaatagttagatagaatattcattagttcatgaccactatagttactatcgaataacgcgtcaaatatttcccgtttaatatcgcatttgtcaagataat :  14300

                *     14320         *     14340         *     14360         *     14380         *     14400
Seq1 : aatagagtgtggtatgttcacgataagtataataacgcatctcttttttgtgtgaaattaaatagtttatcacgtccaaagatgtagcataaccatcttg :  14400
Seq2 : aatagagtgtggtatgttcacgataagtataataacgcatctcttttttgtgtgaaattaaatagtttatcacgtccaaagatgtagcataaccatcttg :  14400
Seq3 : aatagagtgtggtatgttcacgataagtataataacgcatctcttttttgtgtgaaattaaatagtttatcacgtccaaagatgtagcataaccatcttg :  14400
Seq4 : aatagagtgtggtatgttcacgataagtataataacgcatctcttttttgtgtgaaattaaatagtttatcacgtccaaagatgtagcataaccatcttg :  14400

                *     14420         *     14440         *     14460         *     14480         *     14500
Seq1 : tgacctagtaataatataataatagagaactgttttacccattctatcatcataatcagtggtgtagtcgtaatcgtaatcgtctaattcatcatcccaa :  14500
Seq2 : tgacctagtaataatataataatagagaactgttttacccattctatcatcataatcagtggtgtagtcgtaatcgtaatcgtctaattcatcatcccaa :  14500
Seq3 : tgacctagtaataatataataatagagaactgttttacccattctatcatcataatcagtggtgtagtcgtaatcgtaatcgtctaattcatcatcccaa :  14500
Seq4 : tgacctagtaataatataataatagagaactgttttacccattctatcatcataatcagtggtgtagtcgtaatcgtaatcgtctaattcatcatcccaa :  14500

                *     14520         *     14540         *     14560         *     14580         *     14600
Seq1 : ttataatattcaccagcacgtctaatctgttctattttgatcttgtatccatactgtatgttgctacatgtaggtattcctttatccaataatagtttaa :  14600
Seq2 : ttataatattcaccagcacgtctaatctgttctattttgatcttgtatccatactgtatgttgctacatgtaggtattcctttatccaataatagtttaa :  14600
Seq3 : ttataatattcaccagcacgtctaatctgttctattttgatcttgtatccatactgtatgttgctacatgtaggtattcctttatccaataatagtttaa :  14600
Seq4 : ttataatattcaccagcacgtctaatctgttctattttgatcttgtatccatactgtatgttgctacatgtaggtattcctttatccaataatagtttaa :  14600

                *     14620         *     14640         *     14660         *     14680         *     14700
Seq1 : acacatctacattgggatttgatgttgtagcgtatttctctacaatattaataccatttttgatactatttatttctatacctttcgaaattagtaattt :  14700
Seq2 : acacatctacattgggatttgatgttgtagcgtatttctctacaatattaataccatttttgatactatttatttctatacctttcgaaattagtaattt :  14700
Seq3 : acacatctacattgggatttgatgttgtagcgtatttctctacaatattaataccatttttgatactatttatttctatacctttcgaaattagtaattt :  14700
Seq4 : acacatctacattgggatttgatgttgtagcgtatttctctacaatattaataccatttttgatactatttatttctatacctttcgaaattagtaattt :  14700
```

```
                 *     14720         *     14740         *     14760         *     14780         *     14800
Seq1 : caataagtctatatcgatgttatcagaacatagatattcgaatatatcaaaatcattgatatttttatagtcgactgacgacaataacaaaatcacaaca :  14800
Seq2 : caataagtctatatcgatgttatcagaacatagatattcgaatatatcaaaatcattgatatttttatagtcgactgacgacaataacaaaatcacaaca :  14800
Seq3 : caataagtctatatcgatgttatcagaacatagatattcgaatatatcaaaatcattgatatttttatagtcgactgacgacaataacaaaatcacaaca :  14800
Seq4 : caataagtctatatcgatgttatcagaacatagatattcgaatatatcaaaatcattgatatttttatagtcgactgacgacaataacaaaatcacaaca :  14800

                 *     14820         *     14840         *     14860         *     14880         *     14900
Seq1 : tcgtttttgatattattatttttcttggtaacgtatgcctttaatggagtttcaccatcatactcatataatggatttgcaccactttctatcaatgatt :  14900
Seq2 : tcgtttttgatattattatttttcttggtaacgtatgcctttaatggagtttcaccatcatactcatataatggatttgcaccactttctatcaatgatt :  14900
Seq3 : tcgtttttgatattattatttttcttggtaacgtatgcctttaatggagtttcaccatcatactcatataatggatttgcaccactttctatcaatgatt :  14900
Seq4 : tcgtttttgatattattatttttcttggtaacgtatgcctttaatggagtttcaccatcatactcatataatggatttgcaccactttctatcaatgatt :  14900

                 *     14920         *     14940         *     14960         *     14980         *     15000
Seq1 : gtgcactgctggcatcgatgttaaatgttttacaactatcatagagtatcttatcgttaaccatgattggttgttgatgctatcgcattttttggtttct :  15000
Seq2 : gtgcactgctggcatcgatgttaaatgttttacaactatcatagagtatcttatcgttaaccatgattggttgttgatgctatcgcattttttggtttct :  15000
Seq3 : gtgcactgctggcatcgatgttaaatgttttacaactatcatagagtatcttatcgttaaccatgattggttgttgatgctatcgcattttttggtttct :  15000
Seq4 : gtgcactgctggcatcgatgttaaatgttttacaactatcatagagtatcttatcgttaaccatgattggttgttgatgctatcgcattttttggtttct :  15000

                 *     15020         *     15040         *     15060         *     15080         *     15100
Seq1 : ttcatttcagttatgtatggatttagcacgtttgggaagcatgagctcatatgatttcagtactgtagtgtcagtactattagtttcgatcagatcaatg :  15100
Seq2 : ttcatttcagttatgtatggatttagcacgtttgggaagcatgagctcatatgatttcagtactgtagtgtcagtactattagtttcgatcagatcaatg :  15100
Seq3 : ttcatttcagttatgtatggatttagcacgtttgggaagcatgagctcatatgatttcagtactgtagtgtcagtactattagtttcgatcagatcaatg :  15100
Seq4 : ttcatttcagttatgtatggatttagcacgtttgggaagcatgagctcatatgatttcagtactgtagtgtcagtactattagtttcgatcagatcaatg :  15100

                 *     15120         *     15140         *     15160         *     15180         *     15200
Seq1 : tctagatctatagaatcaaaacacgataggtcagaagataatgaatatctgtacgcttctttttgtactgtaacttctggttttgttagatggttgcatc :  15200
Seq2 : tctagatctatagaatcaaaacacgataggtcagaagataatgaatatctgtacgcttctttttgtactgtaacttctggttttgttagatggttgcatc :  15200
Seq3 : tctagatctatagaatcaaaacacgataggtcagaagataatgaatatctgtacgcttctttttgtactgtaacttctggttttgttagatggttgcatc :  15200
Seq4 : tctagatctatagaatcaaaacacgataggtcagaagataatgaatatctgtacgcttctttttgtactgtaacttctggttttgttagatggttgcatc :  15200

                 *     15220         *     15240         *     15260         *     15280         *     15300
Seq1 : gtgctttaacatcaatggtacaaattttatcctcgctttgtgtatcatattcgtctctagtataaaattctatattcagattatcatgcgatgtgtatac :  15300
Seq2 : gtgctttaacatcaatggtacaaattttatcctcgctttgtgtatcatattcgtctctagtataaaattctatattcagattatcatgcgatgtgtatac :  15300
Seq3 : gtgctttaacatcaatggtacaaattttatcctcgctttgtgtatcatattcgtctctagtataaaattctatattcagattatcatgcgatgtgtatac :  15300
Seq4 : gtgctttaacatcaatggtacaaattttatcctcgctttgtgtatcatattcgtctctagtataaaattctatattcagattatcatgcgatgtgtatac :  15300

                 *     15320         *     15340         *     15360         *     15380         *     15400
Seq1 : gctaacggtatcaataaacggagcacaccatttagtcataacagtaatccaaaattttttaaagtatatcttaacgaaagaagttgtgtcattgtctacg :  15400
Seq2 : gctaacggtatcaataaacggagcacaccatttagtcataacagtaatccaaaattttttaaagtatatcttaacgaaagaagttgtgtcattgtctacg :  15400
Seq3 : gctaacggtatcaataaacggagcacaccatttagtcataacagtaatccaaaattttttaaagtatatcttaacgaaagaagttgtgtcattgtctacg :  15400
Seq4 : gctaacggtatcaataaacggagcacaccatttagtcataacagtaatccaaaattttttaaagtatatcttaacgaaagaagttgtgtcattgtctacg :  15400
```

```
             *     15420         *     15440         *     15460         *     15480         *     15500
Seq1 : gtgtatggtactagatcctcataagtgtatatatctagagtaatgtttaatttattaaatggttgataatatggatcctcatgacaatttccgaagatgg :  15500
Seq2 : gtgtatggtactagatcctcataagtgtatatatctagagtaatgtttaatttattaaatggttgataatatggatcctcatgacaatttccgaagatgg :  15500
Seq3 : gtgtatggtactagatcctcataagtgtatatatctagagtaatgtttaatttattaaatggttgataatatggatcctcatgacaatttccgaagatgg :  15500
Seq4 : gtgtatggtactagatcctcataagtgtatatatctagagtaatgtttaatttattaaatggttgataatatggatcctcatgacaatttccgaagatgg :  15500

             *     15520         *     15540         *     15560         *     15580         *     15600
Seq1 : aaatgagatatagacatgcaataaatctaatcgaagacatggttactccttaaaaaaatacgaataatcaccttggctatttagtaagtgtcatttaaca :  15600
Seq2 : aaatgagatatagacatgcaataaatctaatcgaagacatggttactccttaaaaaaatacgaataatcaccttggctatttagtaagtgtcatttaaca :  15600
Seq3 : aaatgagatatagacatgcaataaatctaatcgaagacatggttactccttaaaaaaatacgaataatcaccttggctatttagtaagtgtcatttaaca :  15600
Seq4 : aaatgagatatagacatgcaataaatctaatcgaagacatggttactccttaaaaaaatacgaataatcaccttggctatttagtaagtgtcatttaaca :  15600

             *     15620         *     15640         *     15660         *     15680         *     15700
Seq1 : ctatactcatattaatccatggactcataatctctatacgggattaacggatgttctatatacggggatgagtagttttcttctttaactttatactttt :  15700
Seq2 : ctatactcatattaatccatggactcataatctctatacgggattaacggatgttctatatacggggatgagtagttttcttctttaactttatactttt :  15700
Seq3 : ctatactcatattaatccatggactcataatctctatacgggattaacggatgttctatatacggggatgagtagttttcttctttaactttatactttt :  15700
Seq4 : ctatactcatattaatccatggactcataatctctatacgggattaacggatgttctatatacggggatgagtagttttcttctttaactttatactttt :  15700

             *     15720         *     15740         *     15760         *     15780         *     15800
Seq1 : tactaatcatatttagactgatgtatgggtaatagtgtttaaagagttcgttctcatcatcagaataaatcaatatctctgtttttttgttatacagatg :  15800
Seq2 : tactaatcatatttagactgatgtatgggtaatagtgtttaaagagttcgttctcatcatcagaataaatcaatatctctgtttttttgttatacagatg :  15800
Seq3 : tactaatcatatttagactgatgtatgggtaatagtgtttaaagagttcgttctcatcatcagaataaatcaatatctctgtttttttgttatacagatg :  15800
Seq4 : tactaatcatatttagactgatgtatgggtaatagtgtttaaagagttcgttctcatcatcagaataaatcaatatctctgtttttttgttatacagatg :  15800

             *     15820         *     15840         *     15860         *     15880         *     15900
Seq1 : tattacagcctcatatattacgtaatagaacgtgtcatctaccttattaactttcaccgcatagttgtttgcaaatacggttaatcctttgacctcgtcg :  15900
Seq2 : tattacagcctcatatattacgtaatagaacgtgtcatctaccttattaactttcaccgcatagttgtttgcaaatacggttaatcctttgacctcgtcg :  15900
Seq3 : tattacagcctcatatattacgtaatagaacgtgtcatctaccttattaactttcaccgcatagttgtttgcaaatacggttaatcctttgacctcgtcg :  15900
Seq4 : tattacagcctcatatattacgtaatagaacgtgtcatctaccttattaactttcaccgcatagttgtttgcaaatacggttaatcctttgacctcgtcg :  15900

             *     15920         *     15940         *     15960         *     15980         *     16000
Seq1 : atttccgaccaatctgggcgtataatgaatctaaactttaatttcttgtaatcattcgaaataatttttagtttgcatccgtagttatcccctttatgta :  16000
Seq2 : atttccgaccaatctgggcgtataatgaatctaaactttaatttcttgtaatcattcgaaataatttttagtttgcatccgtagttatcccctttatgta :  16000
Seq3 : atttccgaccaatctgggcgtataatgaatctaaactttaatttcttgtaatcattcgaaataatttttagtttgcatccgtagttatcccctttatgta :  16000
Seq4 : atttccgaccaatctgggcgtataatgaatctaaactttaatttcttgtaatcattcgaaataatttttagtttgcatccgtagttatcccctttatgta :  16000

             *     16020         *     16040         *     16060         *     16080         *     16100
Seq1 : actgtaaatttctcaacgcgatatctccattaataatgatgtcgaattcgtgctgtatacccatactgaatggatgaactaacgaatatcaacggcgtta :  16100
Seq2 : actgtaaatttctcaacgcgatatctccattaataatgatgtcgaattcgtgctgtatacccatactgaatggatgaactaacgaatatcaacggcgtta :  16100
Seq3 : actgtaaatttctcaacgcgatatctccattaataatgatgtcgaattcgtgctgtatacccatactgaatggatgaactaacgaatatcaacggcgtta :  16100
Seq4 : actgtaaatttctcaacgcgatatctccattaataatgatgtcgaattcgtgctgtatacccatactgaatggatgaactaacgaatatcaacggcgtta :  16100
```

```
              *     16120         *     16140         *     16160         *     16180         *     16200
Seq1 : atagtaatttactttttcatctttacatattgggtactagttttactatcataagtttataaattccacaagctactatggaataagccaaccatcttag :  16200
Seq2 : atagtaatttactttttcatctttacatattgggtactagttttactatcataagtttataaattccacaagctactatggaataagccaaccatcttag :  16200
Seq3 : atagtaatttactttttcatctttacatattgggtactagttttactatcataagtttataaattccacaagctactatggaataagccaaccatcttag :  16200
Seq4 : atagtaatttactttttcatctttacatattgggtactagttttactatcataagtttataaattccacaagctactatggaataagccaaccatcttag :  16200

              *     16220         *     16240         *     16260         *     16280         *     16300
Seq1 : tataccacacatgtcttaaagtttattaattaattacatgttgttttatatatatcgctacgaatttaaagagaaatcagtttaggaagaaaaaaattat :  16300
Seq2 : tataccacacatgtcttaaagtttattaattaattacatgttgttttatatatatcgctacgaatttaaagagaaatcagtttaggaagaaaaaaattat :  16300
Seq3 : tataccacacatgtcttaaagtttattaattaattacatgttgttttatatatatcgctacgaatttaaagagaaatcagtttaggaagaaaaaaattat :  16300
Seq4 : tataccacacatgtcttaaagtttattaattaattacatgttgttttatatatatcgctacgaatttaaagagaaatcagtttaggaagaaaaaaattat :  16300

              *     16320         *     16340         *     16360         *     16380         *     16400
Seq1 : ctatctacatcatcacgtctctgtattctacgatagagtgctactttaagatgagacatatccgtgtcatcaaaaatatactccattaaaatgattattc :  16400
Seq2 : ctatctacatcatcacgtctctgtattctacgatagagtgctactttaagatgagacatatccgtgtcatcaaaaatatactccattaaaatgattattc :  16400
Seq3 : ctatctacatcatcacgtctctgtattctacgatagagtgctactttaagatgagacatatccgtgtcatcaaaaatatactccattaaaatgattattc :  16400
Seq4 : ctatctacatcatcacgtctctgtattctacgatagagtgctactttaagatgagacatatccgtgtcatcaaaaatatactccattaaaatgattattc :  16400

              *     16420         *     16440         *     16460         *     16480         *     16500
Seq1 : cggcagcgaacttgatattggatatatcacaacctttgttaatatctacgacaatagacagcagtcccatggttccataaacagtgagtttatctttctt :  16500
Seq2 : cggcagcgaacttgatattggatatatcacaacctttgttaatatctacgacaatagacagcagtcccatggttccataaacagtgagtttatctttctt :  16500
Seq3 : cggcagcgaacttgatattggatatatcacaacctttgttaatatctacgacaatagacagcagtcccatggttccataaacagtgagtttatctttctt :  16500
Seq4 : cggcagcgaacttgatattggatatatcacaacctttgttaatatctacgacaatagacagcagtcccatggttccataaacagtgagtttatctttctt :  16500

              *     16520         *     16540         *     16560         *     16580         *     16600
Seq1 : tgaagcgatagtttgtagagatcttataaaaccgtcaaacgacatcgcatttatatctttagctaattcatatatgttaccatcgtaatatctaaccgcg :  16600
Seq2 : tgaagcgatagtttgtagagatcttataaaaccgtcaaacgacatcgcatttatatctttagctaattcatatatgttaccatcgtaatatctaaccgcg :  16600
Seq3 : tgaagcgatagtttgtagagatcttataaaaccgtcaaacgacatcgcatttatatctttagctaattcatatatgttaccatcgtaatatctaaccgcg :  16600
Seq4 : tgaagcgatagtttgtagagatcttataaaaccgtcaaacgacatcgcatttatatctttagctaattcatatatgttaccatcgtaatatctaaccgcg :  16600

              *     16620         *     16640         *     16660         *     16680         *     16700
Seq1 : tctatcttaaacgtttccatcgctttaaagacgtttccgatagatggtctcatttcatcagtcatactgagccaacaaatataatcgtgtataacatctt :  16700
Seq2 : tctatcttaaacgtttccatcgctttaaagacgtttccgatagatggtctcatttcatcagtcatactgagccaacaaatataatcgtgtataacatctt :  16700
Seq3 : tctatcttaaacgtttccatcgctttaaagacgtttccgatagatggtctcatttcatcagtcatactgagccaacaaatataatcgtgtataacatctt :  16700
Seq4 : tctatcttaaacgtttccatcgctttaaagacgtttccgatagatggtctcatttcatcagtcatactgagccaacaaatataatcgtgtataacatctt :  16700

              *     16720         *     16740         *     16760         *     16780         *     16800
Seq1 : tgatagaatcagactctaaagaaaacgaatcggctttattatacgcattcatgataaacttaatgaaaaatgtttttcgttgtttaagttggatgaatag :  16800
Seq2 : tgatagaatcagactctaaagaaaacgaatcggctttattatacgcattcatgataaacttaatgaaaaatgtttttcgttgtttaagttggatgaatag :  16800
Seq3 : tgatagaatcagactctaaagaaaacgaatcggctttattatacgcattcatgataaacttaatgaaaaatgtttttcgttgtttaagttggatgaatag :  16800
Seq4 : tgatagaatcagactctaaagaaaacgaatcggctttattatacgcattcatgataaacttaatgaaaaatgtttttcgttgtttaagttggatgaatag :  16800
```

```
                *      16820         *      16840         *      16860         *      16880         *      16900
Seq1 : tatgtcttaataattgttattatttcattaattaatatttagtaacgagtacactctataaaaacgagaatgacataactagttatcaaagtgtctagga :  16900
Seq2 : tatgtcttaataattgttattatttcattaattaatatttagtaacgagtacactctataaaaacgagaatgacataactagttatcaaagtgtctagga :  16900
Seq3 : tatgtcttaataattgttattatttcattaattaatatttagtaacgagtacactctataaaaacgagaatgacataactagttatcaaagtgtctagga :  16900
Seq4 : tatgtcttaataattgttattatttcattaattaatatttagtaacgagtacactctataaaaacgagaatgacataactagttatcaaagtgtctagga :  16900

                *      16920         *      16940         *      16960         *      16980         *      17000
Seq1 : cgcgtaattttcatatggtatagatcctgtaagcattgtctgtattctggagctattttctttatcgcattagtaagttcagaatatgttataaatttaa :  17000
Seq2 : cgcgtaattttcatatggtatagatcctgtaagcattgtctgtattctggagctattttctttatcgcattagtaagttcagaatatgttataaatttaa :  17000
Seq3 : cgcgtaattttcatatggtatagatcctgtaagcattgtctgtattctggagctattttctttatcgcattagtaagttcagaatatgttataaatttaa :  17000
Seq4 : cgcgtaattttcatatggtatagatcctgtaagcattgtctgtattctggagctattttctttatcgcattagtaagttcagaatatgttataaatttaa :  17000

                *      17020         *      17040         *      17060         *      17080         *      17100
Seq1 : atcgaataacgaacatgactttagtaaagtcgtctatattaactcttttattttctagccatcgtaataccatgtttaagatagtatattctctagttac :  17100
Seq2 : atcgaataacgaacatgactttagtaaagtcgtctatattaactcttttattttctagccatcgtaataccatgtttaagatagtatattctctagttac :  17100
Seq3 : atcgaataacgaacatgactttagtaaagtcgtctatattaactcttttattttctagccatcgtaataccatgtttaagatagtatattctctagttac :  17100
Seq4 : atcgaataacgaacatgactttagtaaagtcgtctatattaactcttttattttctagccatcgtaataccatgtttaagatagtatattctctagttac :  17100

                *      17120         *      17140         *      17160         *      17180         *      17200
Seq1 : tacgatctcatcgttgtctagaatatcacatactgaatctacatccaattttagaaattggtctgtgttacatatctcttctatattattgttgatgtat :  17200
Seq2 : tacgatctcatcgttgtctagaatatcacatactgaatctacatccaattttagaaattggtctgtgttacatatctcttctatattattgttgatgtat :  17200
Seq3 : tacgatctcatcgttgtctagaatatcacatactgaatctacatccaattttagaaattggtctgtgttacatatctcttctatattattgttgatgtat :  17200
Seq4 : tacgatctcatcgttgtctagaatatcacatactgaatctacatccaattttagaaattggtctgtgttacatatctcttctatattattgttgatgtat :  17200

                *      17220         *      17240         *      17260         *      17280         *      17300
Seq1 : tgtcgtagaaaactattacgtagaccattttctttataaaacgaatatatagtactccaattatctttaccgatatatttgcacacataatccattctct :  17300
Seq2 : tgtcgtagaaaactattacgtagaccattttctttataaaacgaatatatagtactccaattatctttaccgatatatttgcacacataatccattctct :  17300
Seq3 : tgtcgtagaaaactattacgtagaccattttctttataaaacgaatatatagtactccaattatctttaccgatatatttgcacacataatccattctct :  17300
Seq4 : tgtcgtagaaaactattacgtagaccattttctttataaaacgaatatatagtactccaattatctttaccgatatatttgcacacataatccattctct :  17300

                *      17320         *      17340         *      17360         *      17380         *      17400
Seq1 : caatcactacatctttaagatttcgttgttaagatatttggctaaactatataattctattagatcatcaacagaatcagtatatattttctagatcc :  17400
Seq2 : caatcactacatctttaagatttcgttgttaagatatttggctaaactatataattctattagatcatcaacagaatcagtatatattttctagatcc :  17400
Seq3 : caatcactacatctttaagatttcgttgttaagatatttggctaaactatataattctattagatcatcaacagaatcagtatatattttctagatcc :  17400
Seq4 : caatcactacatctttaagatttcgttgttaagatatttggctaaactatataattctattagatcatcaacagaatcagtatatattttctagatcc :  17400

                *      17420         *      17440         *      17460         *      17480         *      17500
Seq1 : aaagacgaactctttggcgtcctctataatattcccagaaaagatattttcgtgttttagtttatcgagatctgatctgttcatatacgccatgattgta :  17500
Seq2 : aaagacgaactctttggcgtcctctataatattcccagaaaagatattttcgtgttttagtttatcgagatctgatctgttcatatacgccatgattgta :  17500
Seq3 : aaagacgaactctttggcgtcctctataatattcccagaaaagatattttcgtgttttagtttatcgagatctgatctgttcatatacgccatgattgta :  17500
Seq4 : aaagacgaactctttggcgtcctctataatattcccagaaaagatattttcgtgttttagtttatcgagatctgatctgttcatatacgccatgattgta :  17500
```

```
                *     17520         *     17540         *     17560         *     17580         *     17600
Seq1 : cggtacgttatgataaccgcataaaataaaaatccattttcatttttaaccaatactattcataattgagattgatgtaatactttgttactttgaacgt :  17600
Seq2 : cggtacgttatgataaccgcataaaataaaaatccattttcatttttaaccaatactattcataattgagattgatgtaatactttgttactttgaacgt :  17600
Seq3 : cggtacgttatgataaccgcataaaataaaaatccattttcatttttaaccaatactattcataattgagattgatgtaatactttgttactttgaacgt :  17600
Seq4 : cggtacgttatgataaccgcataaaataaaaatccattttcatttttaaccaatactattcataattgagattgatgtaatactttgttactttgaacgt :  17600

                *     17620         *     17640         *     17660         *     17680         *     17700
Seq1 : aaagacagtacacggatccgtatctccaacaagcacgtagtaatcaaatttggtgttgttaaacttcgcaatattcatcaatttagatagaaacttatac :  17700
Seq2 : aaagacagtacacggatccgtatctccaacaagcacgtagtaatcaaatttggtgttgttaaacttcgcaatattcatcaatttagatagaaacttatac :  17700
Seq3 : aaagacagtacacggatccgtatctccaacaagcacgtagtaatcaaatttggtgttgttaaacttcgcaatattcatcaatttagatagaaacttatac :  17700
Seq4 : aaagacagtacacggatccgtatctccaacaagcacgtagtaatcaaatttggtgttgttaaacttcgcaatattcatcaatttagatagaaacttatac :  17700

                *     17720         *     17740         *     17760         *     17780         *     17800
Seq1 : tcatcatctgttttaggaatccatgtattattaccactttccaacttatcattatcccaggctatgtttcgtccatcatcgttgcgcagagtgaataatt :  17800
Seq2 : tcatcatctgttttaggaatccatgtattattaccactttccaacttatcattatcccaggctatgtttcgtccatcatcgttgcgcagagtgaataatt :  17800
Seq3 : tcatcatctgttttaggaatccatgtattattaccactttccaacttatcattatcccaggctatgtttcgtccatcatcgttgcgcagagtgaataatt :  17800
Seq4 : tcatcatctgttttaggaatccatgtattattaccactttccaacttatcattatcccaggctatgtttcgtccatcatcgttgcgcagagtgaataatt :  17800

                *     17820         *     17840         *     17860         *     17880         *     17900
Seq1 : cttttgtattcggtagttcaaatatatgatccatgcatagatcggcaaagctattgtagatgtgatttttcctaaatctaatataaaactcgtttactag :  17900
Seq2 : cttttgtattcggtagttcaaatatatgatccatgcatagatcggcaaagctattgtagatgtgatttttcctaaatctaatataaaactcgtttactag :  17900
Seq3 : cttttgtattcggtagttcaaatatatgatccatgcatagatcggcaaagctattgtagatgtgatttttcctaaatctaatataaaactcgtttactag :  17900
Seq4 : cttttgtattcggtagttcaaatatatgatccatgcatagatcggcaaagctattgtagatgtgatttttcctaaatctaatataaaactcgtttactag :  17900

                *     17920         *     17940         *     17960         *     17980         *     18000
Seq1 : caaacactttcctgatttatcgaccaagacacatatggtttctaaatctatcaagtggtggggatccatagttatgacgcagtaacatagattattacat :  18000
Seq2 : caaacactttcctgatttatcgaccaagacacatatggtttctaaatctatcaagtggtggggatccatagttatgacgcagtaacatagattattacat :  18000
Seq3 : caaacactttcctgatttatcgaccaagacacatatggtttctaaatctatcaagtggtggggatccatagttatgacgcagtaacatagattattacat :  18000
Seq4 : caaacactttcctgatttatcgaccaagacacatatggtttctaaatctatcaagtggtggggatccatagttatgacgcagtaacatagattattacat :  18000

                *     18020         *     18040         *     18060         *     18080         *     18100
Seq1 : tcttgactgtcgctaatatctaaatatttattgttatcgtattggattctgcatatagatggcttgtatgtcaaagatatagaacacataaccaatttat :  18100
Seq2 : tcttgactgtcgctaatatctaaatatttattgttatcgtattggattctgcatatagatggcttgtatgtcaaagatatagaacacataaccaatttat :  18100
Seq3 : tcttgactgtcgctaatatctaaatatttattgttatcgtattggattctgcatatagatggcttgtatgtcaaagatatagaacacataaccaatttat :  18100
Seq4 : tcttgactgtcgctaatatctaaatatttattgttatcgtattggattctgcatatagatggcttgtatgtcaaagatatagaacacataaccaatttat :  18100

                *     18120         *     18140         *     18160         *     18180         *     18200
Seq1 : agtcgcgctttacattctcgaatctaaagttaagagatttagaaaacattatatcctcggatgatgttatcactgtttctggagtaggatatattaaagt :  18200
Seq2 : agtcgcgctttacattctcgaatctaaagttaagagatttagaaaacattatatcctcggatgatgttatcactgtttctggagtaggatatattaaagt :  18200
Seq3 : agtcgcgctttacattctcgaatctaaagttaagagatttagaaaacattatatcctcggatgatgttatcactgtttctggagtaggatatattaaagt :  18200
Seq4 : agtcgcgctttacattctcgaatctaaagttaagagatttagaaaacattatatcctcggatgatgttatcactgtttctggagtaggatatattaaagt :  18200
```

```
               *     18220         *     18240         *     18260         *     18280         *     18300
Seq1 : ctttacagatttcgtccgattcaaataaatcactaaataatatcccacattatcatctgttagagtagtatcattaaatctattatattttatgaaagat :  18300
Seq2 : ctttacagatttcgtccgattcaaataaatcactaaataatatcccacattatcatctgttagagtagtatcattaaatctattatattttatgaaagat :  18300
Seq3 : ctttacagatttcgtccgattcaaataaatcactaaataatatcccacattatcatctgttagagtagtatcattaaatctattatattttatgaaagat :  18300
Seq4 : ctttacagatttcgtccgattcaaataaatcactaaataatatcccacattatcatctgttagagtagtatcattaaatctattatattttatgaaagat :  18300

               *     18320         *     18340         *     18360         *     18380         *     18400
Seq1 : atatcactgctcacctctatatttcgtacattttttaaactgtttgtataatatctctctgatacaatcagatatatctattgtgtcggtagacgataccg :  18400
Seq2 : atatcactgctcacctctatatttcgtacattttttaaactgtttgtataatatctctctgatacaatcagatatatctattgtgtcggtagacgataccg :  18400
Seq3 : atatcactgctcacctctatatttcgtacattttttaaactgtttgtataatatctctctgatacaatcagatatatctattgtgtcggtagacgataccg :  18400
Seq4 : atatcactgctcacctctatatttcgtacattttttaaactgtttgtataatatctctctgatacaatcagatatatctattgtgtcggtagacgataccg :  18400

               *     18420         *     18440         *     18460         *     18480         *     18500
Seq1 : ttacatttgaattaatggtgttccattttacaacttttaacaagttgaccaattcatttctaatagtatcaaactctccatgattaaatattttaatagt :  18500
Seq2 : ttacatttgaattaatggtgttccattttacaacttttaacaagttgaccaattcatttctaatagtatcaaactctccatgattaaatattttaatagt :  18500
Seq3 : ttacatttgaattaatggtgttccattttacaacttttaacaagttgaccaattcatttctaatagtatcaaactctccatgattaaatattttaatagt :  18500
Seq4 : ttacatttgaattaatggtgttccattttacaacttttaacaagttgaccaattcatttctaatagtatcaaactctccatgattaaatattttaatagt :  18500

               *     18520         *     18540         *     18560         *     18580         *     18600
Seq1 : atccattttatatcactacggacacaaagtagctgacataaaccattgtataatttttatgttttatgtttattagcgtacacattttggaagttccggc :  18600
Seq2 : atccattttatatcactacggacacaaagtagctgacataaaccattgtataatttttatgttttatgtttattagcgtacacattttggaagttccggc :  18600
Seq3 : atccattttatatcactacggacacaaagtagctgacataaaccattgtataatttttatgttttatgtttattagcgtacacattttggaagttccggc :  18600
Seq4 : atccattttatatcactacggacacaaagtagctgacataaaccattgtataatttttatgttttatgtttattagcgtacacattttggaagttccggc :  18600

               *     18620         *     18640         *     18660         *     18680         *     18700
Seq1 : ttccatgtatttcctggagagcaagtagatgatgaggaaccagatagtttatatccgtacttgcacttaaagtctacattgtcgttgtatgagtatgatc :  18700
Seq2 : ttccatgtatttcctggagagcaagtagatgatgaggaaccagatagtttatatccgtacttgcacttaaagtctacattgtcgttgtatgagtatgatc :  18700
Seq3 : ttccatgtatttcctggagagcaagtagatgatgaggaaccagatagtttatatccgtacttgcacttaaagtctacattgtcgttgtatgagtatgatc :  18700
Seq4 : ttccatgtatttcctggagagcaagtagatgatgaggaaccagatagtttatatccgtacttgcacttaaagtctacattgtcgttgtatgagtatgatc :  18700

               *     18720         *     18740         *     18760         *     18780         *     18800
Seq1 : ttttaaacccgctagacaagtatccgtttgatattgtaggatgtggacatttaacaatctgacacgtgggtggatcggaccattctcctcctgaacacag :  18800
Seq2 : ttttaaacccgctagacaagtatccgtttgatattgtaggatgtggacatttaacaatctgacacgtgggtggatcggaccattctcctcctgaacacag :  18800
Seq3 : ttttaaacccgctagacaagtatccgtttgatattgtaggatgtggacatttaacaatctgacacgtgggtggatcggaccattctcctcctgaacacag :  18800
Seq4 : ttttaaacccgctagacaagtatccgtttgatattgtaggatgtggacatttaacaatctgacacgtgggtggatcggaccattctcctcctgaacacag :  18800

               *     18820         *     18840         *     18860         *     18880         *     18900
Seq1 : gacaccagagttaccaatcaacgaatatccactattgcaactataagttacaacgctcccatcggtataaaaatcctcgtatccgttatgtcttccgttg :  18900
Seq2 : gacaccagagttaccaatcaacgaatatccactattgcaactataagttacaacgctcccatcggtataaaaatcctcgtatccgttatgtcttccgttg :  18900
Seq3 : gacaccagagttaccaatcaacgaatatccactattgcaactataagttacaacgctcccatcggtataaaaatcctcgtatccgttatgtcttccgttg :  18900
Seq4 : gacaccagagttaccaatcaacgaatatccactattgcaactataagttacaacgctcccatcggtataaaaatcctcgtatccgttatgtcttccgttg :  18900
```

```
                *     19020         *     19040         *     19060         *     19080         *     19100
```

```
                *     18920         *     18940         *     18960         *     18980         *     19000
Seq1 : gatatagatggaggggattggcatttaacagattcacaaataggtgcctcgggattccataccatagatccagtagatcctaattcacaatacgatttag :  19000
Seq2 : gatatagatggaggggattggcatttaacagattcacaaataggtgcctcgggattccataccatagatccagtagatcctaattcacaatacgatttag :  19000
Seq3 : gatatagatggaggggattggcatttaacagattcacaaataggtgcctcgggattccataccatagatccagtagatcctaattcacaatacgatttag :  19000
Seq4 : gatatagatggaggggattggcatttaacagattcacaaataggtgcctcgggattccataccatagatccagtagatcctaattcacaatacgatttag :  19000

                *     19020         *     19040         *     19060         *     19080         *     19100
Seq1 : attcaccgatcaaatgatatccgctattacaagagtacgttatactagagccaaagtctactccaccaatatcaagttggccattatcgatatctcgagg :  19100
Seq2 : attcaccgatcaaatgatatccgctattacaagagtacgttatactagagccaaagtctactccaccaatatcaagttggccattatcgatatctcgagg :  19100
Seq3 : attcaccgatcaaatgatatccgctattacaagagtacgttatactagagccaaagtctactccaccaatatcaagttggccattatcgatatctcgagg :  19100
Seq4 : attcaccgatcaaatgatatccgctattacaagagtacgttatactagagccaaagtctactccaccaatatcaagttggccattatcgatatctcgagg :  19100

                *     19120         *     19140         *     19160         *     19180         *     19200
Seq1 : cgatgggcatctccgtttaatacattgattaaagagtgtccatccagtacctgtacatttagcatatataggtcccattttttgctttctgtatccaggt :  19200
Seq2 : cgatgggcatctccgtttaatacattgattaaagagtgtccatccagtacctgtacatttagcatatataggtcccattttttgctttctgtatccaggt :  19200
Seq3 : cgatgggcatctccgtttaatacattgattaaagagtgtccatccagtacctgtacatttagcatatataggtcccattttttgctttctgtatccaggt :  19200
Seq4 : cgatgggcatctccgtttaatacattgattaaagagtgtccatccagtacctgtacatttagcatatataggtcccattttttgctttctgtatccaggt :  19200

                *     19220         *     19240         *     19260         *     19280         *     19300
Seq1 : agacatagatattctatagtgtctcctatgttgtaattagcattagcatcagtctccacactattcttaaatttcatattaatgggtcgtgacggaatag :  19300
Seq2 : agacatagatattctatagtgtctcctatgttgtaattagcattagcatcagtctccacactattcttaaatttcatattaatgggtcgtgacggaatag :  19300
Seq3 : agacatagatattctatagtgtctcctatgttgtaattagcattagcatcagtctccacactattcttaaatttcatattaatgggtcgtgacggaatag :  19300
Seq4 : agacatagatattctatagtgtctcctatgttgtaattagcattagcatcagtctccacactattcttaaatttcatattaatgggtcgtgacggaatag :  19300

                *     19320         *     19340         *     19360         *     19380         *     19400
Seq1 : tacagcatgatagaacgcatcctattcccaacaatgtcaggaacgtcacgctctccaccttcatatttatttatccgtaaaaatgttatcctggacatcg :  19400
Seq2 : tacagcatgatagaacgcatcctattcccaacaatgtcaggaacgtcacgctctccaccttcatatttatttatccgtaaaaatgttatcctggacatcg :  19400
Seq3 : tacagcatgatagaacgcatcctattcccaacaatgtcaggaacgtcacgctctccaccttcatatttatttatccgtaaaaatgttatcctggacatcg :  19400
Seq4 : tacagcatgatagaacgcatcctattcccaacaatgtcaggaacgtcacgctctccaccttcatatttatttatccgtaaaaatgttatcctggacatcg :  19400

                *     19420         *     19440         *     19460         *     19480         *     19500
Seq1 : tacaaataataaaaagcccatatatgttcgctattgtagaaattgtttttcacagttgctcaaaaacgatggcagtgacttatgagttacgttacacttt :  19500
Seq2 : tacaaataataaaaagcccatatatgttcgctattgtagaaattgtttttcacagttgctcaaaaacgatggcagtgacttatgagttacgttacacttt :  19500
Seq3 : tacaaataataaaaagcccatatatgttcgctattgtagaaattgtttttcacagttgctcaaaaacgatggcagtgacttatgagttacgttacacttt :  19500
Seq4 : tacaaataataaaaagcccatatatgttcgctattgtagaaattgtttttcacagttgctcaaaaacgatggcagtgacttatgagttacgttacacttt :  19500

                *     19520         *     19540         *     19560         *     19580         *     19600
Seq1 : ggagtctcatctttagtaaacatatcataatattcgatattacgagttgacatatcgaacaaattccaagtatttgattttggataatattcgtatttttg :  19600
Seq2 : ggagtctcatctttagtaaacatatcataatattcgatattacgagttgacatatcgaacaaattccaagtatttgattttggataatattcgtatttttg :  19600
Seq3 : ggagtctcatctttagtaaacatatcataatattcgatattacgagttgacatatcgaacaaattccaagtatttgattttggataatattcgtatttttg :  19600
Seq4 : ggagtctcatctttagtaaacatatcataatattcgatattacgagttgacatatcgaacaaattccaagtatttgattttggataatattcgtatttttg :  19600
```

```
                *      19620           *      19640           *      19660           *      19680           *      19700
Seq1 : catctgctataattaagatataatcaccgcaagaacacacgaacatctttcctacatggttaaagtacatgtataattctatccatttgtcttccttaac :  19700
Seq2 : catctgctataattaagatataatcaccgcaagaacacacgaacatctttcctacatggttaaagtacatgtataattctatccatttgtcttccttaac :  19700
Seq3 : catctgctataattaagatataatcaccgcaagaacacacgaacatctttcctacatggttaaagtacatgtataattctatccatttgtcttccttaac :  19700
Seq4 : catctgctataattaagatataatcaccgcaagaacacacgaacatctttcctacatggttaaagtacatgtataattctatccatttgtcttccttaac :  19700

                *      19720           *      19740           *      19760           *      19780           *      19800
Seq1 : tatatatttgtatagataattacgagtctcgtgagtaattccagtaattacatagatgtcgccgtcgtactctacagcataaactatactatgatgtcta :  19800
Seq2 : tatatatttgtatagataattacgagtctcgtgagtaattccagtaattacatagatgtcgccgtcgtactctacagcataaactatactatgatgtcta :  19800
Seq3 : tatatatttgtatagataattacgagtctcgtgagtaattccagtaattacatagatgtcgccgtcgtactctacagcataaactatactatgatgtcta :  19800
Seq4 : tatatatttgtatagataattacgagtctcgtgagtaattccagtaattacatagatgtcgccgtcgtactctacagcataaactatactatgatgtcta :  19800

                *      19820           *      19840           *      19860           *      19880           *      19900
Seq1 : ggcatgggagacttttttatccaacgattttagtgaaacattccacatcgtttaatactacatattttttcatacgtggtataaactccacccattacat :  19900
Seq2 : ggcatgggagacttttttatccaacgattttagtgaaacattccacatcgtttaatactacatattttttcatacgtggtataaactccacccattacat :  19900
Seq3 : ggcatgggagacttttttatccaacgattttagtgaaacattccacatcgtttaatactacatattttttcatacgtggtataaactccacccattacat :  19900
Seq4 : ggcatgggagacttttttatccaacgattttagtgaaacattccacatcgtttaatactacatattttttcatacgtggtataaactccacccattacat :  19900

                *      19920           *      19940           *      19960           *      19980           *      20000
Seq1 : atatatcatcgtttacgaataccgacgcgcctgaatatctaggagtaattaagtttggaagtcttatccatttcgaagtgccgtgtttcaaatattctgc :  20000
Seq2 : atatatcatcgtttacgaataccgacgcgcctgaatatctaggagtaattaagtttggaagtcttatccatttcgaagtgccgtgtttcaaatattctgc :  20000
Seq3 : atatatcatcgtttacgaataccgacgcgcctgaatatctaggagtaattaagtttggaagtcttatccatttcgaagtgccgtgtttcaaatattctgc :  20000
Seq4 : atatatcatcgtttacgaataccgacgcgcctgaatatctaggagtaattaagtttggaagtcttatccatttcgaagtgccgtgtttcaaatattctgc :  20000

                *      20020           *      20040           *      20060           *      20080           *      20100
Seq1 : cacacccgttgaaatagaaaattctaatcctcctattacatataactttccatcgttaacacaagtactaacttctgattttaacgacgacatattagta :  20100
Seq2 : cacacccgttgaaatagaaaattctaatcctcctattacatataactttccatcgttaacacaagtactaacttctgattttaacgacgacatattagta :  20100
Seq3 : cacacccgttgaaatagaaaattctaatcctcctattacatataactttccatcgttaacacaagtactaacttctgattttaacgacgacatattagta :  20100
Seq4 : cacacccgttgaaatagaaaattctaatcctcctattacatataactttccatcgttaacacaagtactaacttctgattttaacgacgacatattagta :  20100

                *      20120           *      20140           *      20160           *      20180           *      20200
Seq1 : accgttttccattttttcgtttcaagatctacccgcgatacggaataaacatgtctattgttaatcatgccgccaataatgtatagacaattatgtaaaa :  20200
Seq2 : accgttttccattttttcgtttcaagatctacccgcgatacggaataaacatgtctattgttaatcatgccgccaataatgtatagacaattatgtaaaa :  20200
Seq3 : accgttttccattttttcgtttcaagatctacccgcgatacggaataaacatgtctattgttaatcatgccgccaataatgtatagacaattatgtaaaa :  20200
Seq4 : accgttttccattttttcgtttcaagatctacccgcgatacggaataaacatgtctattgttaatcatgccgccaataatgtatagacaattatgtaaaa :  20200

                *      20220           *      20240           *      20260           *      20280           *      20300
Seq1 : catttgcattatagaattgtctatctgtattaccgactatcgtccaatattctgttctaggagagtaatgggttattgtggatatataatcagagttttt :  20300
Seq2 : catttgcattatagaattgtctatctgtattaccgactatcgtccaatattctgttctaggagagtaatgggttattgtggatatataatcagagttttt :  20300
Seq3 : catttgcattatagaattgtctatctgtattaccgactatcgtccaatattctgttctaggagagtaatgggttattgtggatatataatcagagttttt :  20300
Seq4 : catttgcattatagaattgtctatctgtattaccgactatcgtccaatattctgttctaggagagtaatgggttattgtggatatataatcagagttttt :  20300
```

```
                  *     20320         *     20340         *     20360         *     20380         *     20400
Seq1 : aatgactactatattatgttttataccatttcgtgtcactggctttgtagatttggatatagttaatcccaacaatgatatagcattgcgcatagtatta :  20400
Seq2 : aatgactactatattatgttttataccatttcgtgtcactggctttgtagatttggatatagttaatcccaacaatgatatagcattgcgcatagtatta :  20400
Seq3 : aatgactactatattatgttttataccatttcgtgtcactggctttgtagatttggatatagttaatcccaacaatgatatagcattgcgcatagtatta :  20400
Seq4 : aatgactactatattatgttttataccatttcgtgtcactggctttgtagatttggatatagttaatcccaacaatgatatagcattgcgcatagtatta :  20400

                  *     20420         *     20440         *     20460         *     20480         *     20500
Seq1 : gtcataaacttgggatgtaaaatgttgatgatatctacatcgtttggatttttatgtatccactttaataatatcatagctgtaacatcctcatgattta :  20500
Seq2 : gtcataaacttgggatgtaaaatgttgatgatatctacatcgtttggatttttatgtatccactttaataatatcatagctgtaacatcctcatgattta :  20500
Seq3 : gtcataaacttgggatgtaaaatgttgatgatatctacatcgtttggatttttatgtatccactttaataatatcatagctgtaacatcctcatgattta :  20500
Seq4 : gtcataaacttgggatgtaaaatgttgatgatatctacatcgtttggatttttatgtatccactttaataatatcatagctgtaacatcctcatgattta :  20500

                  *     20520         *     20540         *     20560         *     20580         *     20600
Seq1 : cgttaacgttttcgtgggataagatagttgtcagttcatcctttgataattttccaaattctggatcggatgtcaccgcagtaatattgttgattatttc :  20600
Seq2 : cgttaacgttttcgtgggataagatagttgtcagttcatcctttgataattttccaaattctggatcggatgtcaccgcagtaatattgttgattatttc :  20600
Seq3 : cgttaacgttttcgtgggataagatagttgtcagttcatcctttgataattttccaaattctggatcggatgtcaccgcagtaatattgttgattatttc :  20600
Seq4 : cgttaacgttttcgtgggataagatagttgtcagttcatcctttgataattttccaaattctggatcggatgtcaccgcagtaatattgttgattatttc :  20600

                  *     20620         *     20640         *     20660         *     20680         *     20700
Seq1 : tgacatcgacgcattatatagttttttaattccatatctttagaaaagttaaacatccttatacaatttgtggaattaatattatgaatcatagtttttt :  20700
Seq2 : tgacatcgacgcattatatagttttttaattccatatctttagaaaagttaaacatccttatacaatttgtggaattaatattatgaatcatagtttttt :  20700
Seq3 : tgacatcgacgcattatatagttttttaattccatatctttagaaaagttaaacatccttatacaatttgtggaattaatattatgaatcatagtttttt :  20700
Seq4 : tgacatcgacgcattatatagttttttaattccatatctttagaaaagttaaacatccttatacaatttgtggaattaatattatgaatcatagtttttt :  20700

                  *     20720         *     20740         *     20760         *     20780         *     20800
Seq1 : acacatagatctactacaggcggaacatcaattattatggcagcaactagtatcatttctacattgtttatggtgatgtttatcttcttccagcgtatat :  20800
Seq2 : acacatagatctactacaggcggaacatcaattattatggcagcaactagtatcatttctacattgtttatggtgatgtttatcttcttccagcgtatat :  20800
Seq3 : acacatagatctactacaggcggaacatcaattattatggcagcaactagtatcatttctacattgtttatggtgatgtttatcttcttccagcgtatat :  20800
Seq4 : acacatagatctactacaggcggaacatcaattattatggcagcaactagtatcatttctacattgtttatggtgatgtttatcttcttccagcgtatat :  20800

                  *     20820         *     20840         *     20860         *     20880         *     20900
Seq1 : agtctaatagcgattcaaacgcgtgatagtttataccattcaatataatcgcttcatcctttagatggtgatcctgaattcgtttaaaaaaattatacgg :  20900
Seq2 : agtctaatagcgattcaaacgcgtgatagtttataccattcaatataatcgcttcatcctttagatggtgatcctgaattcgtttaaaaaaattatacgg :  20900
Seq3 : agtctaatagcgattcaaacgcgtgatagtttataccattcaatataatcgcttcatcctttagatggtgatcctgaattcgtttaaaaaaattatacgg :  20900
Seq4 : agtctaatagcgattcaaacgcgtgatagtttataccattcaatataatcgcttcatcctttagatggtgatcctgaattcgtttaaaaaaattatacgg :  20900

                  *     20920         *     20940         *     20960         *     20980         *     21000
Seq1 : agatgccgtaataatttccttattcacttgtataatttccccattgatagaaaatatcacgctttccattcttgaagtactataagtaattatagtataa :  21000
Seq2 : agatgccgtaataatttccttattcacttgtataatttccccattgatagaaaatatcacgctttccattcttgaagtactataagtaattatagtataa :  21000
Seq3 : agatgccgtaataatttccttattcacttgtataatttccccattgatagaaaatatcacgctttccattcttgaagtactataagtaattatagtataa :  21000
Seq4 : agatgccgtaataatttccttattcacttgtataatttccccattgatagaaaatatcacgctttccattcttgaagtactataagtaattatagtataa :  21000
```

```
              *     21020         *     21040         *     21060         *     21080         *     21100
Seq1 : tgtaaaggtttatatattcaatattttttataaaaaaatcattttgacattaattcctttttaaatttccgtctatcatctatagaaacgtattctatga :  21100
Seq2 : tgtaaaggtttatatattcaatattttttataaaaaaatcattttgacattaattcctttttaaatttccgtctatcatctatagaaacgtattctatga :  21100
Seq3 : tgtaaaggtttatatattcaatattttttataaaaaaatcattttgacattaattcctttttaaatttccgtctatcatctatagaaacgtattctatga :  21100
Seq4 : tgtaaaggtttatatattcaatattttttataaaaaaatcattttgacattaattcctttttaaatttccgtctatcatctatagaaacgtattctatga :  21100

              *     21120         *     21140         *     21160         *     21180         *     21200
Seq1 : atttataaaatgcttttacgtgtcctatcgtaggcgatagaaccgctaaaaagcctatcgaatttctacaaaagaatctgttatatggtatagggagagt :  21200
Seq2 : atttataaaatgcttttacgtgtcctatcgtaggcgatagaaccgctaaaaagcctatcgaatttctacaaaagaatctgttatatggtatagggagagt :  21200
Seq3 : atttataaaatgcttttacgtgtcctatcgtaggcgatagaaccgctaaaaagcctatcgaatttctacaaaagaatctgttatatggtatagggagagt :  21200
Seq4 : atttataaaatgcttttacgtgtcctatcgtaggcgatagaaccgctaaaaagcctatcgaatttctacaaaagaatctgttatatggtatagggagagt :  21200

              *     21220         *     21240         *     21260         *     21280         *     21300
Seq1 : ataaaacattaaatgtccgtacttattaaagtattcagtagccaatcctaactctttcgaatacttattaatggctcttgttctgtacgaatctattttt :  21300
Seq2 : ataaaacattaaatgtccgtacttattaaagtattcagtagccaatcctaactctttcgaatacttattaatggctcttgttctgtacgaatctattttt :  21300
Seq3 : ataaaacattaaatgtccgtacttattaaagtattcagtagccaatcctaactctttcgaatacttattaatggctcttgttctgtacgaatctattttt :  21300
Seq4 : ataaaacattaaatgtccgtacttattaaagtattcagtagccaatcctaactctttcgaatacttattaatggctcttgttctgtacgaatctattttt :  21300

              *     21320         *     21340         *     21360         *     21380         *     21400
Seq1 : ttgaacaacggacctagtggtatatcttgttctatgtatctaaaataatgtctgactagatccgttagtttaatatcctcagtcatcttgtctagaatgg :  21400
Seq2 : ttgaacaacggacctagtggtatatcttgttctatgtatctaaaataatgtctgactagatccgttagtttaatatcctcagtcatcttgtctagaatgg :  21400
Seq3 : ttgaacaacggacctagtggtatatcttgttctatgtatctaaaataatgtctgactagatccgttagtttaatatcctcagtcatcttgtctagaatgg :  21400
Seq4 : ttgaacaacggacctagtggtatatcttgttctatgtatctaaaataatgtctgactagatccgttagtttaatatcctcagtcatcttgtctagaatgg :  21400

              *     21420         *     21440         *     21460         *     21480         *     21500
Seq1 : caaatctaactgcgggtttaggctttagtttagtttttatatctacatctatgtctttatctaacaccaaaaatataatagctaatattttattacaatc :  21500
Seq2 : caaatctaactgcgggtttaggctttagtttagtttttatatctacatctatgtctttatctaacaccaaaaatataatagctaatattttattacaatc :  21500
Seq3 : caaatctaactgcgggtttaggctttagtttagtttttatatctacatctatgtctttatctaacaccaaaaatataatagctaatattttattacaatc :  21500
Seq4 : caaatctaactgcgggtttaggctttagtttagtttttatatctacatctatgtctttatctaacaccaaaaatataatagctaatattttattacaatc :  21500

              *     21520         *     21540         *     21560         *     21580         *     21600
Seq1 : atccggatattcttctacgatctcactaactaatgtttcttttgttatactagtatagtcactatcggacaaataaagaaaatcagatgatcgatgaata :  21600
Seq2 : atccggatattcttctacgatctcactaactaatgtttcttttgttatactagtatagtcactatcggacaaataaagaaaatcagatgatcgatgaata :  21600
Seq3 : atccggatattcttctacgatctcactaactaatgtttcttttgttatactagtatagtcactatcggacaaataaagaaaatcagatgatcgatgaata :  21600
Seq4 : atccggatattcttctacgatctcactaactaatgtttcttttgttatactagtatagtcactatcggacaaataaagaaaatcagatgatcgatgaata :  21600

              *     21620         *     21640         *     21660         *     21680         *     21700
Seq1 : atacatttaaattcatcatctgtaagattttgagatgtctcattaaaatattattagggtcagtactcattatcattcggcagctattacttattttat :  21700
Seq2 : atacatttaaattcatcatctgtaagattttgagatgtctcattaaaatattattagggtcagtactcattatcattcggcagctattacttattttat :  21700
Seq3 : atacatttaaattcatcatctgtaagattttgagatgtctcattaaaatattattagggtcagtactcattatcattcggcagctattacttattttat :  21700
Seq4 : atacatttaaattcatcatctgtaagattttgagatgtctcattaaaatattattagggtcagtactcattatcattcggcagctattacttattttat :  21700
```

```
                 *     21720          *     21740          *     21760          *     21780          *     21800
Seq1 : ttttctgtatttattatttttcaccatatagatcaatcattagatcatcaaaatatgtttcaatcatcctaaagagtatggtgaatgactcttcccatc :  21800
Seq2 : ttttctgtatttattatttttcaccatatagatcaatcattagatcatcaaaatatgtttcaatcatcctaaagagtatggtgaatgactcttcccatc :  21800
Seq3 : ttttctgtatttattatttttcaccatatagatcaatcattagatcatcaaaatatgtttcaatcatcctaaagagtatggtgaatgactcttcccatc :  21800
Seq4 : ttttctgtatttattatttttcaccatatagatcaatcattagatcatcaaaatatgtttcaatcatcctaaagagtatggtgaatgactcttcccatc :  21800

                 *     21820          *     21840          *     21860          *     21880          *     21900
Seq1 : taatttctgaacgttcaccaatgtctctagccactttggcactaatagcgatcattcgcttagcgtcttctatattattaactggttgattcaatctatc :  21900
Seq2 : taatttctgaacgttcaccaatgtctctagccactttggcactaatagcgatcattcgcttagcgtcttctatattattaactggttgattcaatctatc :  21900
Seq3 : taatttctgaacgttcaccaatgtctctagccactttggcactaatagcgatcattcgcttagcgtcttctatattattaactggttgattcaatctatc :  21900
Seq4 : taatttctgaacgttcaccaatgtctctagccactttggcactaatagcgatcattcgcttagcgtcttctatattattaactggttgattcaatctatc :  21900

                 *     21920          *     21940          *     21960          *     21980          *     22000
Seq1 : tagcaatggaccgtcggacagcgtcattctcatgttcttaatcaatgtacatacatcgccgtcatctaccaattcatccaacaacataagcttttaaaa :  22000
Seq2 : tagcaatggaccgtcggacagcgtcattctcatgttcttaatcaatgtacatacatcgccgtcatctaccaattcatccaacaacataagcttttaaaa :  22000
Seq3 : tagcaatggaccgtcggacagcgtcattctcatgttcttaatcaatgtacatacatcgccgtcatctaccaattcatccaacaacataagcttttaaaa :  22000
Seq4 : tagcaatggaccgtcggacagcgtcattctcatgttcttaatcaatgtacatacatcgccgtcatctaccaattcatccaacaacataagcttttaaaa :  22000

                 *     22020          *     22040          *     22060          *     22080          *     22100
Seq1 : tcatcattataataggtttgatcgttgtcatttctccaaagaatatatctaataagtagagtcctcatgcttagtaatttaactattttagttaacaact :  22100
Seq2 : tcatcattataataggtttgatcgttgtcatttctccaaagaatatatctaataagtagagtcctcatgcttagtaatttaactattttagttaacaact :  22100
Seq3 : tcatcattataataggtttgatcgttgtcatttctccaaagaatatatctaataagtagagtcctcatgcttagtaatttaactattttagttaacaact :  22100
Seq4 : tcatcattataataggtttgatcgttgtcatttctccaaagaatatatctaataagtagagtcctcatgcttagtaatttaactattttagttaacaact :  22100

                 *     22120          *     22140          *     22160          *     22180          *     22200
Seq1 : attttttatgttaaatcaattagtacaccgctatgtttaatacttattcatattttagtttttaggattgagaatcaatacaaaaaattaatgcatcatt :  22200
Seq2 : attttttatgttaaatcaattagtacaccgctatgtttaatacttattcatattttagtttttaggattgagaatcaatacaaaaaattaatgcatcatt :  22200
Seq3 : attttttatgttaaatcaattagtacaccgctatgtttaatacttattcatattttagtttttaggattgagaatcaatacaaaaaattaatgcatcatt :  22200
Seq4 : attttttatgttaaatcaattagtacaccgctatgtttaatacttattcatattttagtttttaggattgagaatcaatacaaaaaattaatgcatcatt :  22200

                 *     22220          *     22240          *     22260          *     22280          *     22300
Seq1 : aattttagaaatacttagtttccacgtagttaatgaaacatttgaactcatcgtacaggacgttctcgtacaggacgtaactataaaccggtttatattt :  22300
Seq2 : aattttagaaatacttagtttccacgtagttaatgaaacatttgaactcatcgtacaggacgttctcgtacaggacgtaactataaaccggtttatattt :  22300
Seq3 : aattttagaaatacttagtttccacgtagttaatgaaacatttgaactcatcgtacaggacgttctcgtacaggacgtaactataaaccggtttatattt :  22300
Seq4 : aattttagaaatacttagtttccacgtagttaatgaaacatttgaactcatcgtacaggacgttctcgtacaggacgtaactataaaccggtttatattt :  22300

                 *     22320          *     22340          *     22360          *     22380          *     22400
Seq1 : gttcaagatagatacaaatccgataacttttttacgaattctacgggatccactttaaaagtgtcataccgggttctttttattctttaaacagatca :  22400
Seq2 : gttcaagatagatacaaatccgataacttttttacgaattctacgggatccactttaaaagtgtcataccgggttctttttattctttaaacagatca :  22400
Seq3 : gttcaagatagatacaaatccgataacttttttacgaattctacgggatccactttaaaagtgtcataccgggttctttttattctttaaacagatca :  22400
Seq4 : gttcaagatagatacaaatccgataacttttttacgaattctacgggatccactttaaaagtgtcataccgggttctttttattctttaaacagatca :  22400
```

```
                 *     22420         *     22440         *     22460         *     22480         *     22500
Seq1 : atggtgtgatgttgattaggtcttttacgaatttgatatagaatagcgttcacatatcctccataatggtcaatcgccatttgttcgtatgtcataaatt :  22500
Seq2 : atggtgtgatgttgattaggtcttttacgaatttgatatagaatagcgttcacatatcctccataatggtcaatcgccatttgttcgtatgtcataaatt :  22500
Seq3 : atggtgtgatgttgattaggtcttttacgaatttgatatagaatagcgttcacatatcctccataatggtcaatcgccatttgttcgtatgtcataaatt :  22500
Seq4 : atggtgtgatgttgattaggtcttttacgaatttgatatagaatagcgttcacatatcctccataatggtcaatcgccatttgttcgtatgtcataaatt :  22500

                 *     22520         *     22540         *     22560         *     22580         *     22600
Seq1 : ctttaattatatgacactgtgtattatttagttcatccttgttcatcattaggaatctatccaaaatggcaattatactagaactataggtgcgttgtat :  22600
Seq2 : ctttaattatatgacactgtgtattatttagttcatccttgttcatcattaggaatctatccaaaatggcaattatactagaactataggtgcgttgtat :  22600
Seq3 : ctttaattatatgacactgtgtattatttagttcatccttgttcatcattaggaatctatccaaaatggcaattatactagaactataggtgcgttgtat :  22600
Seq4 : ctttaattatatgacactgtgtattatttagttcatccttgttcatcattaggaatctatccaaaatggcaattatactagaactataggtgcgttgtat :  22600

                 *     22620         *     22640         *     22660         *     22680         *     22700
Seq1 : acacatattgatgtgtctgtttatacaatccatgatatttggatccatgctactaccttcgggtaaaattgtagcatcatataccatttctagtacttta :  22700
Seq2 : acacatattgatgtgtctgtttatacaatccatgatatttggatccatgctactaccttcgggtaaaattgtagcatcatataccatttctagtacttta :  22700
Seq3 : acacatattgatgtgtctgtttatacaatccatgatatttggatccatgctactaccttcgggtaaaattgtagcatcatataccatttctagtacttta :  22700
Seq4 : acacatattgatgtgtctgtttatacaatccatgatatttggatccatgctactaccttcgggtaaaattgtagcatcatataccatttctagtacttta :  22700

                 *     22720         *     22740         *     22760         *     22780         *     22800
Seq1 : ggttcattattatccattgcagaggacgtcatgatcgaatcctaaaaaaatatattatttttatgttattttgttaaaaataatcatcgaatacttcgta :  22800
Seq2 : ggttcattattatccattgcagaggacgtcatgatcgaatcctaaaaaaatatattatttttatgttattttgttaaaaataatcatcgaatacttcgta :  22800
Seq3 : ggttcattattatccattgcagaggacgtcatgatcgaatcctaaaaaaatatattatttttatgttattttgttaaaaataatcatcgaatacttcgta :  22800
Seq4 : ggttcattattatccattgcagaggacgtcatgatcgaatcctaaaaaaatatattatttttatgttattttgttaaaaataatcatcgaatacttcgta :  22800

                 *     22820         *     22840         *     22860         *     22880         *     22900
Seq1 : agatactccttcatgaacataatcagttacaaaacgtttatatgaagtaaagtatctacgatttttacaaaagtccggatgcataagtacaaagtacgcg :  22900
Seq2 : agatactccttcatgaacataatcagttacaaaacgtttatatgaagtaaagtatctacgatttttacaaaagtccggatgcataagtacaaagtacgcg :  22900
Seq3 : agatactccttcatgaacataatcagttacaaaacgtttatatgaagtaaagtatctacgatttttacaaaagtccggatgcataagtacaaagtacgcg :  22900
Seq4 : agatactccttcatgaacataatcagttacaaaacgtttatatgaagtaaagtatctacgatttttacaaaagtccggatgcataagtacaaagtacgcg :  22900

                 *     22920         *     22940         *     22960         *     22980         *     23000
Seq1 : ataaacggaataataatagatttatctagtctatctttttctatagctttcatagttagatacatggtctcagaagtaggattatgtaacatcagcttcg :  23000
Seq2 : ataaacggaataataatagatttatctagtctatctttttctatagctttcatagttagatacatggtctcagaagtaggattatgtaacatcagcttcg :  23000
Seq3 : ataaacggaataataatagatttatctagtctatctttttctatagctttcatagttagatacatggtctcagaagtaggattatgtaacatcagcttcg :  23000
Seq4 : ataaacggaataataatagatttatctagtctatctttttctatagctttcatagttagatacatggtctcagaagtaggattatgtaacatcagcttcg :  23000

                 *     23020         *     23040         *     23060         *     23080         *     23100
Seq1 : ataaaatgactgggttatttagtcttacacattcgctcatacatgtatgaccgttaactacaaagtctacactaaaatgattgaacaatagatagtctac :  23100
Seq2 : ataaaatgactgggttatttagtcttacacattcgctcatacatgtatgaccgttaactacaaagtctacactaaaatgattgaacaatagatagtctac :  23100
Seq3 : ataaaatgactgggttatttagtcttacacattcgctcatacatgtatgaccgttaactacaaagtctacactaaaatgattgaacaatagatagtctac :  23100
Seq4 : ataaaatgactgggttatttagtcttacacattcgctcatacatgtatgaccgttaactacaaagtctacactaaaatgattgaacaatagatagtctac :  23100
```

```
                 *     23120         *     23140         *     23160         *     23180         *     23200
Seq1 : cattgtttcgtattcagatagtacagcgtagtacatagcatcttcacaaattatatcattgtctaatagatatttgacgcatcttatggatcccacttca :  23200
Seq2 : cattgtttcgtattcagatagtacagcgtagtacatagcatcttcacaaattatatcattgtctaatagatatttgacgcatcttatggatcccacttca :  23200
Seq3 : cattgtttcgtattcagatagtacagcgtagtacatagcatcttcacaaattatatcattgtctaatagatatttgacgcatcttatggatcccacttca :  23200
Seq4 : cattgtttcgtattcagatagtacagcgtagtacatagcatcttcacaaattatatcattgtctaatagatatttgacgcatcttatggatcccacttca :  23200

                 *     23220         *     23240         *     23260         *     23280         *     23300
Seq1 : acagccatcttaaaatcggtagaatcatattgctttcctttatcattaataatttctaaaacatcatctctatcataaaagatacaaatattaactgttt :  23300
Seq2 : acagccatcttaaaatcggtagaatcatattgctttcctttatcattaataatttctaaaacatcatctctatcataaaagatacaaatattaactgttt :  23300
Seq3 : acagccatcttaaaatcggtagaatcatattgctttcctttatcattaataatttctaaaacatcatctctatcataaaagatacaaatattaactgttt :  23300
Seq4 : acagccatcttaaaatcggtagaatcatattgctttcctttatcattaataatttctaaaacatcatctctatcataaaagatacaaatattaactgttt :  23300

                 *     23320         *     23340         *     23360         *     23380         *     23400
Seq1 : gatccgtaataacattgctagtcgatagcaatttgttaataagatgcgctgggctcaatgtcttaataagaagtgtaagaggactatctccgaatttgtt :  23400
Seq2 : gatccgtaataacattgctagtcgatagcaatttgttaataagatgcgctgggctcaatgtcttaataagaagtgtaagaggactatctccgaatttgtt :  23400
Seq3 : gatccgtaataacattgctagtcgatagcaatttgttaataagatgcgctgggctcaatgtcttaataagaagtgtaagaggactatctccgaatttgtt :  23400
Seq4 : gatccgtaataacattgctagtcgatagcaatttgttaataagatgcgctgggctcaatgtcttaataagaagtgtaagaggactatctccgaatttgtt :  23400

                 *     23420         *     23440         *     23460         *     23480         *     23500
Seq1 : ttgtttattaacatccgttgatggaagtaaaagatctataatgtctacattcttgactgttttagagcatacaatatggagaggtgtatttccatcatga :  23500
Seq2 : ttgtttattaacatccgttgatggaagtaaaagatctataatgtctacattcttgactgttttagagcatacaatatggagaggtgtatttccatcatga :  23500
Seq3 : ttgtttattaacatccgttgatggaagtaaaagatctataatgtctacattcttgactgttttagagcatacaatatggagaggtgtatttccatcatga :  23500
Seq4 : ttgtttattaacatccgttgatggaagtaaaagatctataatgtctacattcttgactgttttagagcatacaatatggagaggtgtatttccatcatga :  23500

                 *     23520         *     23540         *     23560         *     23580         *     23600
Seq1 : tctggttttgagggactaattcctagtttcatcatccatgagattgtagaagcttttggattgtctgacataagatgtctatgaatatgatttttgccaa :  23600
Seq2 : tctggttttgagggactaattcctagtttcatcatccatgagattgtagaagcttttggattgtctgacataagatgtctatgaatatgatttttgccaa :  23600
Seq3 : tctggttttgagggactaattcctagtttcatcatccatgagattgtagaagcttttggattgtctgacataagatgtctatgaatatgatttttgccaa :  23600
Seq4 : tctggttttgagggactaattcctagtttcatcatccatgagattgtagaagcttttggattgtctgacataagatgtctatgaatatgatttttgccaa :  23600

                 *     23620         *     23640         *     23660         *     23680         *     23700
Seq1 : atttatccactatcctggcttcgaatccgatggacattatttttttaaacactctttctgaaggatctgtacacgccaacaacggaccacatccttcttc :  23700
Seq2 : atttatccactatcctggcttcgaatccgatggacattatttttttaaacactctttctgaaggatctgtacacgccaacaacggaccacatccttcttc :  23700
Seq3 : atttatccactatcctggcttcgaatccgatggacattatttttttaaacactctttctgaaggatctgtacacgccaacaacggaccacatccttcttc :  23700
Seq4 : atttatccactatcctggcttcgaatccgatggacattatttttttaaacactctttctgaaggatctgtacacgccaacaacggaccacatccttcttc :  23700

                 *     23720         *     23740         *     23760         *     23780         *     23800
Seq1 : atcaaccgagttgttaatcttggctccatactgtaccaataaatttattctctctatgacttcatcatctgttcccgagagataatatagaggtgtttta :  23800
Seq2 : atcaaccgagttgttaatcttggctccatactgtaccaataaatttattctctctatgacttcatcatctgttcccgagagataatatagaggtgtttta :  23800
Seq3 : atcaaccgagttgttaatcttggctccatactgtaccaataaatttattctctctatgacttcatcatctgttcccgagagataatatagaggtgtttta :  23800
Seq4 : atcaaccgagttgttaatcttggctccatactgtaccaataaatttattctctctatgacttcatcatctgttcccgagagataatatagaggtgtttta :  23800
```

```
              *     23820          *     23840          *     23860          *     23880          *     23900
Seq1 : ttatgtttatcacacgcgtttggatctgcgccgtgcgtcagcagcatcgcgactattctattattattaattttagaagctatatgcaatggataatttc :  23900
Seq2 : ttatgtttatcacacgcgtttggatctgcgccgtgcgtcagcagcatcgcgactattctattattattaattttagaagctatatgcaatggataatttc :  23900
Seq3 : ttatgtttatcacacgcgtttggatctgcgccgtgcgtcagcagcatcgcgactattctattattattaattttagaagctatatgcaatggataatttc :  23900
Seq4 : ttatgtttatcacacgcgtttggatctgcgccgtgcgtcagcagcatcgcgactattctattattattaattttagaagctatatgcaatggataatttc :  23900

              *     23920          *     23940          *     23960          *     23980          *     24000
Seq1 : catcatcatccgtctcatttggagagtatcctctatgaagaagttcttcgacaaatcgttcatctagtcctttaattccacaatacgcatgtagaatgtg :  24000
Seq2 : catcatcatccgtctcatttggagagtatcctctatgaagaagttcttcgacaaatcgttcatctagtcctttaattccacaatacgcatgtagaatgtg :  24000
Seq3 : catcatcatccgtctcatttggagagtatcctctatgaagaagttcttcgacaaatcgttcatctagtcctttaattccacaatacgcatgtagaatgtg :  24000
Seq4 : catcatcatccgtctcatttggagagtatcctctatgaagaagttcttcgacaaatcgttcatctagtcctttaattccacaatacgcatgtagaatgtg :  24000

              *     24020          *     24040          *     24060          *     24080          *     24100
Seq1 : ataattatttccagaaggttcgatagcttgtagcatattcctaaatacatctaaatttttactattatatttggcataaagagatagataatactcggcc :  24100
Seq2 : ataattatttccagaaggttcgatagcttgtagcatattcctaaatacatctaaatttttactattatatttggcataaagagatagataatactcggcc :  24100
Seq3 : ataattatttccagaaggttcgatagcttgtagcatattcctaaatacatctaaatttttactattatatttggcataaagagatagataatactcggcc :  24100
Seq4 : ataattatttccagaaggttcgatagcttgtagcatattcctaaatacatctaaatttttactattatatttggcataaagagatagataatactcggcc :  24100

              *     24120          *     24140          *     24160          *     24180          *     24200
Seq1 : gacataatgttgtccattgtagtataaaaattaatatttctatttctatttctgtatatttgcaacaatttactctctataacaaatatcataacttagt :  24200
Seq2 : gacataatgttgtccattgtagtataaaaattaatatttctatttctatttctgtatatttgcaacaatttactctctataacaaatatcataacttagt :  24200
Seq3 : gacataatgttgtccattgtagtataaaaattaatatttctatttctatttctgtatatttgcaacaatttactctctataacaaatatcataacttagt :  24200
Seq4 : gacataatgttgtccattgtagtataaaaattaatatttctatttctatttctgtatatttgcaacaatttactctctataacaaatatcataacttagt :  24200

              *     24220          *     24240          *     24260          *     24280          *     24300
Seq1 : tcttttatgtcaagaaggcactggtttagttcatctataaatgtcacgccataactaccacgcatgctatactcagaattatgataaagatatttatcct :  24300
Seq2 : tcttttatgtcaagaaggcactggtttagttcatctataaatgtcacgccataactaccacgcatgctatactcagaattatgataaagatatttatcct :  24300
Seq3 : tcttttatgtcaagaaggcactggtttagttcatctataaatgtcacgccataactaccacgcatgctatactcagaattatgataaagatatttatcct :  24300
Seq4 : tcttttatgtcaagaaggcactggtttagttcatctataaatgtcacgccataactaccacgcatgctatactcagaattatgataaagatatttatcct :  24300

              *     24320          *     24340          *     24360          *     24380          *     24400
Seq1 : tggggtgtaggtaatggggattaatctttgttggatcagtctctaagttaacacatgtcacacatgatccatttatagttatatcacacgatgatgattt :  24400
Seq2 : tggggtgtaggtaatggggattaatctttgttggatcagtctctaagttaacacatgtcacacatgatccatttatagttatatcacacgatgatgattt :  24400
Seq3 : tggggtgtaggtaatggggattaatctttgttggatcagtctctaagttaacacatgtcacacatgatccatttatagttatatcacacgatgatgattt :  24400
Seq4 : tggggtgtaggtaatggggattaatctttgttggatcagtctctaagttaacacatgtcacacatgatccatttatagttatatcacacgatgatgattt :  24400

              *     24420          *     24440          *     24460          *     24480          *     24500
Seq1 : atgaattgattccggaagatcgctatcgtattttgtggttccacaattcatttccatacatgttattgtcacactaatattatgatgaactttatctagc :  24500
Seq2 : atgaattgattccggaagatcgctatcgtattttgtggttccacaattcatttccatacatgttattgtcacactaatattatgatgaactttatctagc :  24500
Seq3 : atgaattgattccggaagatcgctatcgtattttgtggttccacaattcatttccatacatgttattgtcacactaatattatgatgaactttatctagc :  24500
Seq4 : atgaattgattccggaagatcgctatcgtattttgtggttccacaattcatttccatacatgttattgtcacactaatattatgatgaactttatctagc :  24500
```

```
              *     24520          *     24540          *     24560          *     24580          *     24600
Seq1 : cgctgagtggtaaacaacagaacagatagtttattatctttaccaacaccctcagccgctgccacaaatctctgatccgtatccatgatggtcatgttta :  24600
Seq2 : cgctgagtggtaaacaacagaacagatagtttattatctttaccaacaccctcagccgctgccacaaatctctgatccgtatccatgatggtcatgttta :  24600
Seq3 : cgctgagtggtaaacaacagaacagatagtttattatctttaccaacaccctcagccgctgccacaaatctctgatccgtatccatgatggtcatgttta :  24600
Seq4 : cgctgagtggtaaacaacagaacagatagtttattatctttaccaacaccctcagccgctgccacaaatctctgatccgtatccatgatggtcatgttta :  24600

              *     24620          *     24640          *     24660          *     24680          *     24700
Seq1 : tttctagtccgtatccagtcaacactatgttagcatttctgtcgatatagctttcactcatatgacactcaccaataatagtagaattaatgtcgtaatt :  24700
Seq2 : tttctagtccgtatccagtcaacactatgttagcatttctgtcgatatagctttcactcatatgacactcaccaataatagtagaattaatgtcgtaatt :  24700
Seq3 : tttctagtccgtatccagtcaacactatgttagcatttctgtcgatatagctttcactcatatgacactcaccaataatagtagaattaatgtcgtaatt :  24700
Seq4 : tttctagtccgtatccagtcaacactatgttagcatttctgtcgatatagctttcactcatatgacactcaccaataatagtagaattaatgtcgtaatt :  24700

              *     24720          *     24740          *     24760          *     24780          *     24800
Seq1 : tacaccaatagtgagttcggcggcaaagtaccaataccggtaatcttgtcgaggaggacatatagtattcttgtattctaccgaatacccgagagatgcg :  24800
Seq2 : tacaccaatagtgagttcggcggcaaagtaccaataccggtaatcttgtcgaggaggacatatagtattcttgtattctaccgaatacccgagagatgcg :  24800
Seq3 : tacaccaatagtgagttcggcggcaaagtaccaataccggtaatcttgtcgaggaggacatatagtattcttgtattctaccgaatacccgagagatgcg :  24800
Seq4 : tacaccaatagtgagttcggcggcaaagtaccaataccggtaatcttgtcgaggaggacatatagtattcttgtattctaccgaatacccgagagatgcg :  24800

              *     24820          *     24840          *     24860          *     24880          *     24900
Seq1 : atacaaaagagtaagactaatttgtaaaccatcttactcaaaatatgtaacaatagtacgatgcaatgagtaagacaataggaaatctatcttatataca :  24900
Seq2 : atacaaaagagtaagactaatttgtaaaccatcttactcaaaatatgtaacaatagtacgatgcaatgagtaagacaataggaaatctatcttatataca :  24900
Seq3 : atacaaaagagtaagactaatttgtaaaccatcttactcaaaatatgtaacaatagtacgatgcaatgagtaagacaataggaaatctatcttatataca :  24900
Seq4 : atacaaaagagtaagactaatttgtaaaccatcttactcaaaatatgtaacaatagtacgatgcaatgagtaagacaataggaaatctatcttatataca :  24900

              *     24920          *     24940          *     24960          *     24980          *     25000
Seq1 : cataattattctatcaattttaccaattagttagtgtaatgttaacaaaaatgtgggagaatctaattagtttttctttacacaattgacgtacatgagt :  25000
Seq2 : cataattattctatcaattttaccaattagttagtgtaatgttaacaaaaatgtgggagaatctaattagtttttctttacacaattgacgtacatgagt :  25000
Seq3 : cataattattctatcaattttaccaattagttagtgtaatgttaacaaaaatgtgggagaatctaattagtttttctttacacaattgacgtacatgagt :  25000
Seq4 : cataattattctatcaattttaccaattagttagtgtaatgttaacaaaaatgtgggagaatctaattagtttttctttacacaattgacgtacatgagt :  25000

              *     25020          *     25040          *     25060          *     25080          *     25100
Seq1 : ctgagttccttgtttttgctaattatttcatccaatttattattcttgactatatcgagatcttttgtataggagtcagacttgtattcaacatgctttt :  25100
Seq2 : ctgagttccttgtttttgctaattatttcatccaatttattattcttgactatatcgagatcttttgtataggagtcagacttgtattcaacatgctttt :  25100
Seq3 : ctgagttccttgtttttgctaattatttcatccaatttattattcttgactatatcgagatcttttgtataggagtcagacttgtattcaacatgctttt :  25100
Seq4 : ctgagttccttgtttttgctaattatttcatccaatttattattcttgactatatcgagatcttttgtataggagtcagacttgtattcaacatgctttt :  25100

              *     25120          *     25140          *     25160          *     25180          *     25200
Seq1 : ctataatcatttagctatttcggcatcatccaatagtacattttccagattagcagaatagatattaatgtcgtatttgaacagagcctgtaacatctc :  25200
Seq2 : ctataatcatttagctatttcggcatcatccaatagtacattttccagattagcagaatagatattaatgtcgtatttgaacagagcctgtaacatctc :  25200
Seq3 : ctataatcatttagctatttcggcatcatccaatagtacattttccagattagcagaatagatattaatgtcgtatttgaacagagcctgtaacatctc :  25200
Seq4 : ctataatcatttagctatttcggcatcatccaatagtacattttccagattagcagaatagatattaatgtcgtatttgaacagagcctgtaacatctc :  25200
```

```
                 *     25220         *     25240         *     25260         *     25280         *     25300
Seq1 : aatgtctttattatctatagccaatttaatgtccggaatgaagagaagggaattattggtgtttgtcgacgtcatatagtcgagcaagagaatcatcata :  25300
Seq2 : aatgtctttattatctatagccaatttaatgtccggaatgaagagaagggaattattggtgtttgtcgacgtcatatagtcgagcaagagaatcatcata :  25300
Seq3 : aatgtctttattatctatagccaatttaatgtccggaatgaagagaagggaattattggtgtttgtcgacgtcatatagtcgagcaagagaatcatcata :  25300
Seq4 : aatgtctttattatctatagccaatttaatgtccggaatgaagagaagggaattattggtgtttgtcgacgtcatatagtcgagcaagagaatcatcata :  25300

                 *     25320         *     25340         *     25360         *     25380         *     25400
Seq1 : tccacgtgtccatttttttatagtgatgtgaatacaactaaggagaatagccagatcaaaagtagatggtatctctgaaagaaagtaggaaacaatactta :  25400
Seq2 : tccacgtgtccatttttttatagtgatgtgaatacaactaaggagaatagccagatcaaaagtagatggtatctctgaaagaaagtaggaaacaatactta :  25400
Seq3 : tccacgtgtccatttttttatagtgatgtgaatacaactaaggagaatagccagatcaaaagtagatggtatctctgaaagaaagtaggaaacaatactta :  25400
Seq4 : tccacgtgtccatttttttatagtgatgtgaatacaactaaggagaatagccagatcaaaagtagatggtatctctgaaagaaagtaggaaacaatactta :  25400

                 *     25420         *     25440         *     25460         *     25480         *     25500
Seq1 : catcattaagcatgacggcatgataaaatgaagttttccatccagttttcccatagaacatcagtctccaatttttcttaacaaacagttttaccgtttg :  25500
Seq2 : catcattaagcatgacggcatgataaaatgaagttttccatccagttttcccatagaacatcagtctccaatttttcttaacaaacagttttaccgtttg :  25500
Seq3 : catcattaagcatgacggcatgataaaatgaagttttccatccagttttcccatagaacatcagtctccaatttttcttaacaaacagttttaccgtttg :  25500
Seq4 : catcattaagcatgacggcatgataaaatgaagttttccatccagttttcccatagaacatcagtctccaatttttcttaacaaacagttttaccgtttg :  25500

                 *     25520         *     25540         *     25560         *     25580         *     25600
Seq1 : catgttaccactatcaaccgcataatacaatgcggtgtttcccttgtcatcaaattgtgaatcatccagtccactgaatagcaaaatctttactattttg :  25600
Seq2 : catgttaccactatcaaccgcataatacaatgcggtgtttcccttgtcatcaaattgtgaatcatccagtccactgaatagcaaaatctttactattttg :  25600
Seq3 : catgttaccactatcaaccgcataatacaatgcggtgtttcccttgtcatcaaattgtgaatcatccagtccactgaatagcaaaatctttactattttg :  25600
Seq4 : catgttaccactatcaaccgcataatacaatgcggtgtttcccttgtcatcaaattgtgaatcatccagtccactgaatagcaaaatctttactattttg :  25600

                 *     25620         *     25640         *     25660         *     25680         *     25700
Seq1 : gtatcttccaatgtggctgcctgatgtaatggaaattcattctctagaagatttttcaatgctccagcgttcaacaacgtacatactagacgcacgttat :  25700
Seq2 : gtatcttccaatgtggctgcctgatgtaatggaaattcattctctagaagatttttcaatgctccagcgttcaacaacgtacatactagacgcacgttat :  25700
Seq3 : gtatcttccaatgtggctgcctgatgtaatggaaattcattctctagaagatttttcaatgctccagcgttcaacaacgtacatactagacgcacgttat :  25700
Seq4 : gtatcttccaatgtggctgcctgatgtaatggaaattcattctctagaagatttttcaatgctccagcgttcaacaacgtacatactagacgcacgttat :  25700

                 *     25720         *     25740         *     25760         *     25780         *     25800
Seq1 : tatcagctattgcataatacaaggcactatgtccatggacatccgccttaaatgcatctttgctagagagaaagcttttcagctgcttagacttccaagt :  25800
Seq2 : tatcagctattgcataatacaaggcactatgtccatggacatccgccttaaatgcatctttgctagagagaaagcttttcagctgcttagacttccaagt :  25800
Seq3 : tatcagctattgcataatacaaggcactatgtccatggacatccgccttaaatgcatctttgctagagagaaagcttttcagctgcttagacttccaagt :  25800
Seq4 : tatcagctattgcataatacaaggcactatgtccatggacatccgccttaaatgcatctttgctagagagaaagcttttcagctgcttagacttccaagt :  25800

                 *     25820         *     25840         *     25860         *     25880         *     25900
Seq1 : attaattcgtgacagatccatgtctgaaacgagacgctaattagtgtatatttttttcatttttttataatttttgtcatattgcaccagaattaataatatc :  25900
Seq2 : attaattcgtgacagatccatgtctgaaacgagacgctaattagtgtatatttttttcatttttttataatttttgtcatattgcaccagaattaataatatc :  25900
Seq3 : attaattcgtgacagatccatgtctgaaacgagacgctaattagtgtatatttttttcatttttttataatttttgtcatattgcaccagaattaataatatc :  25900
Seq4 : attaattcgtgacagatccatgtctgaaacgagacgctaattagtgtatatttttttcatttttttataatttttgtcatattgcaccagaattaataatatc :  25900
```

```
                   *     25920         *     25940         *     25960         *     25980         *     26000
Seq1 : tctaatagatctgattagtagatacatggctatcgcaaaacaacatatacacatttaataaaaataatatttattaagaaaattcagatttcacgtaccc :  26000
Seq2 : tctaatagatctgattagtagatacatggctatcgcaaaacaacatatacacatttaataaaaataatatttattaagaaaattcagatttcacgtaccc :  26000
Seq3 : tctaatagatctgattagtagatacatggctatcgcaaaacaacatatacacatttaataaaaataatatttattaagaaaattcagatttcacgtaccc :  26000
Seq4 : tctaatagatctgattagtagatacatggctatcgcaaaacaacatatacacatttaataaaaataatatttattaagaaaattcagatttcacgtaccc :  26000

                   *     26020         *     26040         *     26060         *     26080         *     26100
Seq1 : atcaatataaataaaataatgattccttacaccgtacccatattaaggagattccaccttacccataaacaatataaatccagtaatatcatgtctgatg :  26100
Seq2 : atcaatataaataaaataatgattccttacaccgtacccatattaaggagattccaccttacccataaacaatataaatccagtaatatcatgtctgatg :  26100
Seq3 : atcaatataaataaaataatgattccttacaccgtacccatattaaggagattccaccttacccataaacaatataaatccagtaatatcatgtctgatg :  26100
Seq4 : atcaatataaataaaataatgattccttacaccgtacccatattaaggagattccaccttacccataaacaatataaatccagtaatatcatgtctgatg :  26100

                   *     26120         *     26140         *     26160         *     26180         *     26200
Seq1 : atgaacacaaatggtgtattaaattccagttttcaggagatgatctcgccgtagctaccataatagtagatgcctctgctacagttccttgttcgtcga :  26200
Seq2 : atgaacacaaatggtgtattaaattccagttttcaggagatgatctcgccgtagctaccataatagtagatgcctctgctacagttccttgttcgtcga :  26200
Seq3 : atgaacacaaatggtgtattaaattccagttttcaggagatgatctcgccgtagctaccataatagtagatgcctctgctacagttccttgttcgtcga :  26200
Seq4 : atgaacacaaatggtgtattaaattccagttttcaggagatgatctcgccgtagctaccataatagtagatgcctctgctacagttccttgttcgtcga :  26200

                   *     26220         *     26240         *     26260         *     26280         *     26300
Seq1 : catctatctttgcattctgaaacattttataaatatataatgggtccctagtcatatgtttaaacgacgcattatctggattaaacatactaggagccat :  26300
Seq2 : catctatctttgcattctgaaacattttataaatatataatgggtccctagtcatatgtttaaacgacgcattatctggattaaacatactaggagccat :  26300
Seq3 : catctatctttgcattctgaaacattttataaatatataatgggtccctagtcatatgtttaaacgacgcattatctggattaaacatactaggagccat :  26300
Seq4 : catctatctttgcattctgaaacattttataaatatataatgggtccctagtcatatgtttaaacgacgcattatctggattaaacatactaggagccat :  26300

                   *     26320         *     26340         *     26360         *     26380         *     26400
Seq1 : catttcggctatcgacttaatatccctcttattttcgatagaaaatttagggagtttaagattgtacactttattccctaattgaaacgaccaatagtct :  26400
Seq2 : catttcggctatcgacttaatatccctcttattttcgatagaaaatttagggagtttaagattgtacactttattccctaattgaaacgaccaatagtct :  26400
Seq3 : catttcggctatcgacttaatatccctcttattttcgatagaaaatttagggagtttaagattgtacactttattccctaattgaaacgaccaatagtct :  26400
Seq4 : catttcggctatcgacttaatatccctcttattttcgatagaaaatttagggagtttaagattgtacactttattccctaattgaaacgaccaatagtct :  26400

                   *     26420         *     26440         *     26460         *     26480         *     26500
Seq1 : aattttgcagccgtaatagaatctgtgaaatgggtcatattatcacctattgccaggtacatactaatattagcatccttatacggaaggcgtaccatat :  26500
Seq2 : aattttgcagccgtaatagaatctgtgaaatgggtcatattatcacctattgccaggtacatactaatattagcatccttatacggaaggcgtaccatat :  26500
Seq3 : aattttgcagccgtaatagaatctgtgaaatgggtcatattatcacctattgccaggtacatactaatattagcatccttatacggaaggcgtaccatat :  26500
Seq4 : aattttgcagccgtaatagaatctgtgaaatgggtcatattatcacctattgccaggtacatactaatattagcatccttatacggaaggcgtaccatat :  26500

                   *     26520         *     26540         *     26560         *     26580         *     26600
Seq1 : catattcttcgtcatcgattgtgattgtatttccttgcaatttagtaactacgttcatcatgggaaccgttttcgtaccgtacttattagtaaaactagc :  26600
Seq2 : catattcttcgtcatcgattgtgattgtatttccttgcaatttagtaactacgttcatcatgggaaccgttttcgtaccgtacttattagtaaaactagc :  26600
Seq3 : catattcttcgtcatcgattgtgattgtatttccttgcaatttagtaactacgttcatcatgggaaccgttttcgtaccgtacttattagtaaaactagc :  26600
Seq4 : catattcttcgtcatcgattgtgattgtatttccttgcaatttagtaactacgttcatcatgggaaccgttttcgtaccgtacttattagtaaaactagc :  26600
```

```
                *     26620         *     26640         *     26660         *     26680         *     26700
Seq1 : attgcgtgttttagtgatatcaaacggatattgccatatacctttaaaatatatagtattaatgattgcccatagagtattattgtcgagcatattagaa :  26700
Seq2 : attgcgtgttttagtgatatcaaacggatattgccatatacctttaaaatatatagtattaatgattgcccatagagtattattgtcgagcatattagaa :  26700
Seq3 : attgcgtgttttagtgatatcaaacggatattgccatatacctttaaaatatatagtattaatgattgcccatagagtattattgtcgagcatattagaa :  26700
Seq4 : attgcgtgttttagtgatatcaaacggatattgccatatacctttaaaatatatagtattaatgattgcccatagagtattattgtcgagcatattagaa :  26700

                *     26720         *     26740         *     26760         *     26780         *     26800
Seq1 : tctactacattagacataccggatctacgttctactatagaattaattttattaaccgcatctcgtctaaagtttaatctatataggccgaatctatgat :  26800
Seq2 : tctactacattagacataccggatctacgttctactatagaattaattttattaaccgcatctcgtctaaagtttaatctatataggccgaatctatgat :  26800
Seq3 : tctactacattagacataccggatctacgttctactatagaattaattttattaaccgcatctcgtctaaagtttaatctatataggccgaatctatgat :  26800
Seq4 : tctactacattagacataccggatctacgttctactatagaattaattttattaaccgcatctcgtctaaagtttaatctatataggccgaatctatgat :  26800

                *     26820         *     26840         *     26860         *     26880         *     26900
Seq1 : attgttgataatacgacggtttaatgcacacagtattatctacgaaactttgataagttagatcagtgtacgtatatttagatgttttcagcttagctaa :  26900
Seq2 : attgttgataatacgacggtttaatgcacacagtattatctacgaaactttgataagttagatcagtgtacgtatatttagatgttttcagcttagctaa :  26900
Seq3 : attgttgataatacgacggtttaatgcacacagtattatctacgaaactttgataagttagatcagtgtacgtatatttagatgttttcagcttagctaa :  26900
Seq4 : attgttgataatacgacggtttaatgcacacagtattatctacgaaactttgataagttagatcagtgtacgtatatttagatgttttcagcttagctaa :  26900

                *     26920         *     26940         *     26960         *     26980         *     27000
Seq1 : tcctgatattaattctgtaaatgctggacccagatctctttttctcaaatccatagtcttcaataattctattctagtattacctgatgcaggcaatagc :  27000
Seq2 : tcctgatattaattctgtaaatgctggacccagatctctttttctcaaatccatagtcttcaataattctattctagtattacctgatgcaggcaatagc :  27000
Seq3 : tcctgatattaattctgtaaatgctggacccagatctctttttctcaaatccatagtcttcaataattctattctagtattacctgatgcaggcaatagc :  27000
Seq4 : tcctgatattaattctgtaaatgctggacccagatctctttttctcaaatccatagtcttcaataattctattctagtattacctgatgcaggcaatagc :  27000

                *     27020         *     27040         *     27060         *     27080         *     27100
Seq1 : gacataaacatagaaaacgaataaccaaacggtgagaagacaatattatcatcttgaatatttttatacgctactataccggcattggtaaatccttgca :  27100
Seq2 : gacataaacatagaaaacgaataaccaaacggtgagaagacaatattatcatcttgaatatttttatacgctactataccggcattggtaaatccttgca :  27100
Seq3 : gacataaacatagaaaacgaataaccaaacggtgagaagacaatattatcatcttgaatatttttatacgctactataccggcattggtaaatccttgca :  27100
Seq4 : gacataaacatagaaaacgaataaccaaacggtgagaagacaatattatcatcttgaatatttttatacgctactataccggcattggtaaatccttgca :  27100

                *     27120         *     27140         *     27160         *     27180         *     27200
Seq1 : gacgataggtagacactgaacacgttaacgatagtatcaataacgcaatcatgattttatggtattaataattaaccttatttttatgttcggtataaaa :  27200
Seq2 : gacgataggtagacactgaacacgttaacgatagtatcaataacgcaatcatgattttatggtattaataattaaccttatttttatgttcggtataaaa :  27200
Seq3 : gacgataggtagacactgaacacgttaacgatagtatcaataacgcaatcatgattttatggtattaataattaaccttatttttatgttcggtataaaa :  27200
Seq4 : gacgataggtagacactgaacacgttaacgatagtatcaataacgcaatcatgattttatggtattaataattaaccttatttttatgttcggtataaaa :  27200

                *     27220         *     27240         *     27260         *     27280         *     27300
Seq1 : attattgatgtctacacatccttttgtaattgacatctatatatccttttgtataatcaactctaatcactttaacttttacagttttccctaccagttt :  27300
Seq2 : attattgatgtctacacatccttttgtaattgacatctatatatccttttgtataatcaactctaatcactttaacttttacagttttccctaccagttt :  27300
Seq3 : attattgatgtctacacatccttttgtaattgacatctatatatccttttgtataatcaactctaatcactttaacttttacagttttccctaccagttt :  27300
Seq4 : attattgatgtctacacatccttttgtaattgacatctatatatccttttgtataatcaactctaatcactttaacttttacagttttccctaccagttt :  27300
```

```
                *     27320         *     27340         *     27360         *     27380         *     27400
Seq1 : atccctatattcaacatatctatccatatgcatcttaacactctctgccaagatagcttcaaagtgaggatagtcaaaaagataaatatatagagcataa :  27400
Seq2 : atccctatattcaacatatctatccatatgcatcttaacactctctgccaagatagcttcaaagtgaggatagtcaaaaagataaatatatagagcataa :  27400
Seq3 : atccctatattcaacatatctatccatatgcatcttaacactctctgccaagatagcttcaaagtgaggatagtcaaaaagataaatatatagagcataa :  27400
Seq4 : atccctatattcaacatatctatccatatgcatcttaacactctctgccaagatagcttcaaagtgaggatagtcaaaaagataaatatatagagcataa :  27400

                *     27420         *     27440         *     27460         *     27480         *     27500
Seq1 : tccttctcgtatactctgccctttattacatcacccgcattgggcaacgaataacaaaatgcaagcatcttgttaacgggctcgtaaattgggataaaaa :  27500
Seq2 : tccttctcgtatactctgccctttattacatcacccgcattgggcaacgaataacaaaatgcaagcatcttgttaacgggctcgtaaattgggataaaaa :  27500
Seq3 : tccttctcgtatactctgccctttattacatcacccgcattgggcaacgaataacaaaatgcaagcatcttgttaacgggctcgtaaattgggataaaaa :  27500
Seq4 : tccttctcgtatactctgccctttattacatcacccgcattgggcaacgaataacaaaatgcaagcatcttgttaacgggctcgtaaattgggataaaaa :  27500

                *     27520         *     27540         *     27560         *     27580         *     27600
Seq1 : ttatgttttatatctatttattcaagagaatattcaggaatttctttttccggttgtatctcatcgcagtatatatcatttgtacattgtttcatatt :  27600
Seq2 : ttatgttttatatctatttattcaagagaatattcaggaatttctttttccggttgtatctcatcgcagtatatatcatttgtacattgtttcatatt :  27600
Seq3 : ttatgttttatatctatttattcaagagaatattcaggaatttctttttccggttgtatctcatcgcagtatatatcatttgtacattgtttcatatt :  27600
Seq4 : ttatgttttatatctatttattcaagagaatattcaggaatttctttttccggttgtatctcatcgcagtatatatcatttgtacattgtttcatatt :  27600

                *     27620         *     27640         *     27660         *     27680         *     27700
Seq1 : ttttaatagtctacaccttttagtaggactagtatcgtacaattcatagctgtattttgaattccaatcacgcataaaaatatcttccaattgttgacga :  27700
Seq2 : ttttaatagtctacaccttttagtaggactagtatcgtacaattcatagctgtattttgaattccaatcacgcataaaaatatcttccaattgttgacga :  27700
Seq3 : ttttaatagtctacaccttttagtaggactagtatcgtacaattcatagctgtattttgaattccaatcacgcataaaaatatcttccaattgttgacga :  27700
Seq4 : ttttaatagtctacaccttttagtaggactagtatcgtacaattcatagctgtattttgaattccaatcacgcataaaaatatcttccaattgttgacga :  27700

                *     27720         *     27740         *     27760         *     27780         *     27800
Seq1 : agacctaatccatcatccggtgtaatattaatagatgctccacatgtatccgtaaagtaatttcctgtccaatttgaggtacctatatacgccgttttat :  27800
Seq2 : agacctaatccatcatccggtgtaatattaatagatgctccacatgtatccgtaaagtaatttcctgtccaatttgaggtacctatatacgccgttttat :  27800
Seq3 : agacctaatccatcatccggtgtaatattaatagatgctccacatgtatccgtaaagtaatttcctgtccaatttgaggtacctatatacgccgttttat :  27800
Seq4 : agacctaatccatcatccggtgtaatattaatagatgctccacatgtatccgtaaagtaatttcctgtccaatttgaggtacctatatacgccgttttat :  27800

                *     27820         *     27840         *     27860         *     27880         *     27900
Seq1 : cggttaccatatatttggcatggtttaccctagaatacggaatgggaggatcagcatctggtacaataaatagctttacttctatatttatgtttttaga :  27900
Seq2 : cggttaccatatatttggcatggtttaccctagaatacggaatgggaggatcagcatctggtacaataaatagctttacttctatatttatgtttttaga :  27900
Seq3 : cggttaccatatatttggcatggtttaccctagaatacggaatgggaggatcagcatctggtacaataaatagctttacttctatatttatgtttttaga :  27900
Seq4 : cggttaccatatatttggcatggtttaccctagaatacggaatgggaggatcagcatctggtacaataaatagctttacttctatatttatgtttttaga :  27900

                *     27920         *     27940         *     27960         *     27980         *     28000
Seq1 : ttttagcatagcgatagatcttaaaaagtttctcatgataaacgaagatcgttgccagcaactaatcaatagcttaactgacacttgtctgtctatagcg :  28000
Seq2 : ttttagcatagcgatagatcttaaaaagtttctcatgataaacgaagatcgttgccagcaactaatcaatagcttaactgacacttgtctgtctatagcg :  28000
Seq3 : ttttagcatagcgatagatcttaaaaagtttctcatgataaacgaagatcgttgccagcaactaatcaatagcttaactgacacttgtctgtctatagcg :  28000
Seq4 : ttttagcatagcgatagatcttaaaaagtttctcatgataaacgaagatcgttgccagcaactaatcaatagcttaactgacacttgtctgtctatagcg :  28000
```

```
              *     28020         *     28040         *     28060         *     28080         *     28100
Seq1 : gctcttcttaattcatcttctatataaggccaaaacaaaatattgcctgccttcgaataaataatagggataaagttcataacagatacataaacgaatt :  28100
Seq2 : gctcttcttaattcatcttctatataaggccaaaacaaaatattgcctgccttcgaataaataatagggataaagttcataacagatacataaacgaatt :  28100
Seq3 : gctcttcttaattcatcttctatataaggccaaaacaaaatattgcctgccttcgaataaataatagggataaagttcataacagatacataaacgaatt :  28100
Seq4 : gctcttcttaattcatcttctatataaggccaaaacaaaatattgcctgccttcgaataaataatagggataaagttcataacagatacataaacgaatt :  28100

              *     28120         *     28140         *     28160         *     28180         *     28200
Seq1 : tactcgcatttctgatacatgacaataaagcggttaaatcattggttctttccatagtacatagttgttgcggtgcagaagcaataaatacagagtgtgg :  28200
Seq2 : tactcgcatttctgatacatgacaataaagcggttaaatcattggttctttccatagtacatagttgttgcggtgcagaagcaataaatacagagtgtgg :  28200
Seq3 : tactcgcatttctgatacatgacaataaagcggttaaatcattggttctttccatagtacatagttgttgcggtgcagaagcaataaatacagagtgtgg :  28200
Seq4 : tactcgcatttctgatacatgacaataaagcggttaaatcattggttctttccatagtacatagttgttgcggtgcagaagcaataaatacagagtgtgg :  28200

              *     28220         *     28240         *     28260         *     28280         *     28300
Seq1 : aacgccgcttacgttaatactaagaggatgatctgtattataatacgacggataaaagtttttccaattatatggtagattgttaactccaagataccag :  28300
Seq2 : aacgccgcttacgttaatactaagaggatgatctgtattataatacgacggataaaagtttttccaattatatggtagattgttaactccaagataccag :  28300
Seq3 : aacgccgcttacgttaatactaagaggatgatctgtattataatacgacggataaaagtttttccaattatatggtagattgttaactccaagataccag :  28300
Seq4 : aacgccgcttacgttaatactaagaggatgatctgtattataatacgacggataaaagtttttccaattatatggtagattgttaactccaagataccag :  28300

              *     28320         *     28340         *     28360         *     28380         *     28400
Seq1 : tatacctcaaaaatttgagtgagatccgctgccaagttcctattattgaagatcgcaatacccaattctttgacctgagttagtgatctccaatccatgt :  28400
Seq2 : tatacctcaaaaatttgagtgagatccgctgccaagttcctattattgaagatcgcaatacccaattctttgacctgagttagtgatctccaatccatgt :  28400
Seq3 : tatacctcaaaaatttgagtgagatccgctgccaagttcctattattgaagatcgcaatacccaattctttgacctgagttagtgatctccaatccatgt :  28400
Seq4 : tatacctcaaaaatttgagtgagatccgctgccaagttcctattattgaagatcgcaatacccaattctttgacctgagttagtgatctccaatccatgt :  28400

              *     28420         *     28440         *     28460         *     28480         *     28500
Seq1 : tagcgcttcctaaataaatatgtgtattatcagatatccaaaattttgtatgaagaactcctcctaggatatttgtaatatctatgtatcgtacttcaac :  28500
Seq2 : tagcgcttcctaaataaatatgtgtattatcagatatccaaaattttgtatgaagaactcctcctaggatatttgtaatatctatgtatcgtacttcaac :  28500
Seq3 : tagcgcttcctaaataaatatgtgtattatcagatatccaaaattttgtatgaagaactcctcctaggatatttgtaatatctatgtatcgtacttcaac :  28500
Seq4 : tagcgcttcctaaataaatatgtgtattatcagatatccaaaattttgtatgaagaactcctcctaggatatttgtaatatctatgtatcgtacttcaac :  28500

              *     28520         *     28540         *     28560         *     28580         *     28600
Seq1 : tccggccatttgtagtctttcaacatcctttaatggtttgttagatttattgacggctactctaactcgtactcctcttttgggtaattgtacaatcttg :  28600
Seq2 : tccggccatttgtagtctttcaacatcctttaatggtttgttagatttattgacggctactctaactcgtactcctcttttgggtaattgtacaatcttg :  28600
Seq3 : tccggccatttgtagtctttcaacatcctttaatggtttgttagatttattgacggctactctaactcgtactcctcttttgggtaattgtacaatcttg :  28600
Seq4 : tccggccatttgtagtctttcaacatcctttaatggtttgttagatttattgacggctactctaactcgtactcctcttttgggtaattgtacaatcttg :  28600

              *     28620         *     28640         *     28660         *     28680         *     28700
Seq1 : tttaatattatcgtgccgaaattcgtacccacttcatccgataaactccaataaaaagatgatatatctagtgtttttgtggtattggatagaatttccc :  28700
Seq2 : tttaatattatcgtgccgaaattcgtacccacttcatccgataaactccaataaaaagatgatatatctagtgtttttgtggtattggatagaatttccc :  28700
Seq3 : tttaatattatcgtgccgaaattcgtacccacttcatccgataaactccaataaaaagatgatatatctagtgtttttgtggtattggatagaatttccc :  28700
Seq4 : tttaatattatcgtgccgaaattcgtacccacttcatccgataaactccaataaaaagatgatatatctagtgtttttgtggtattggatagaatttccc :  28700
```

```
                 *     28720         *     28740         *     28760         *     28780         *     28800
Seq1 : tccacatgttaaatgtagacaaatatactttatcaaattgcatacctataggaatagtctctgtaatcactgcgattgtattatccggattcattttatt :  28800
Seq2 : tccacatgttaaatgtagacaaatatactttatcaaattgcatacctataggaatagtctctgtaatcactgcgattgtattatccggattcattttatt :  28800
Seq3 : tccacatgttaaatgtagacaaatatactttatcaaattgcatacctataggaatagtctctgtaatcactgcgattgtattatccggattcattttatt :  28800
Seq4 : tccacatgttaaatgtagacaaatatactttatcaaattgcatacctataggaatagtctctgtaatcactgcgattgtattatccggattcattttatt :  28800

                 *     28820         *     28840         *     28860         *     28880         *     28900
Seq1 : tgttaaaagaataatcctatatcacttcactctattaaaaatccaagtttctatttctttcatgactgatttttaacttcatccgtttccttatgaaga :  28900
Seq2 : tgttaaaagaataatcctatatcacttcactctattaaaaatccaagtttctatttctttcatgactgatttttaacttcatccgtttccttatgaaga :  28900
Seq3 : tgttaaaagaataatcctatatcacttcactctattaaaaatccaagtttctatttctttcatgactgatttttaacttcatccgtttccttatgaaga :  28900
Seq4 : tgttaaaagaataatcctatatcacttcactctattaaaaatccaagtttctatttctttcatgactgatttttaacttcatccgtttccttatgaaga :  28900

                 *     28920         *     28940         *     28960         *     28980         *     29000
Seq1 : tgatgtttggcaccttcataaatttttatttctctattacaatttgcatgttgcatgaaataatatgcacctaaaacatcgctaatcttattgtttgttc :  29000
Seq2 : tgatgtttggcaccttcataaatttttatttctctattacaatttgcatgttgcatgaaataatatgcacctaaaacatcgctaatcttattgtttgttc :  29000
Seq3 : tgatgtttggcaccttcataaatttttatttctctattacaatttgcatgttgcatgaaataatatgcacctaaaacatcgctaatcttattgtttgttc :  29000
Seq4 : tgatgtttggcaccttcataaatttttatttctctattacaatttgcatgttgcatgaaataatatgcacctaaaacatcgctaatcttattgtttgttc :  29000

                 *     29020         *     29040         *     29060         *     29080         *     29100
Seq1 : cctggagtatgagagtcggggggtgttaatcttggaaattatttttctaaccttgttggtagccttcaagacctgactagcaaatccagccttaattttt :  29100
Seq2 : cctggagtatgagagtcggggggtgttaatcttggaaattatttttctaaccttgttggtagccttcaagacctgactagcaaatccagccttaattttt :  29100
Seq3 : cctggagtatgagagtcggggggtgttaatcttggaaattatttttctaaccttgttggtagccttcaagacctgactagcaaatccagccttaattttt :  29100
Seq4 : cctggagtatgagagtcggggggtgttaatcttggaaattatttttctaaccttgttggtagccttcaagacctgactagcaaatccagccttaattttt :  29100

                 *     29120         *     29140         *     29160         *     29180         *     29200
Seq1 : tcatgattgattaatgggtcgtattggtatttataaactttatccatatctctagatactgattctggacatagctttccgactggcgcatttagtgtga :  29200
Seq2 : tcatgattgattaatgggtcgtattggtatttataaactttatccatatctctagatactgattctggacatagctttccgactggcgcatttagtgtga :  29200
Seq3 : tcatgattgattaatgggtcgtattggtatttataaactttatccatatctctagatactgattctggacatagctttccgactggcgcatttagtgtga :  29200
Seq4 : tcatgattgattaatgggtcgtattggtatttataaactttatccatatctctagatactgattctggacatagctttccgactggcgcatttagtgtga :  29200

                 *     29220         *     29240         *     29260         *     29280         *     29300
Seq1 : tggttcccataagtttggcagctagcagattcagttttgaaacagcatctgcattaactagaggagacattagaatcattgctgtaaacaagtttggatt :  29300
Seq2 : tggttcccataagtttggcagctagcagattcagttttgaaacagcatctgcattaactagaggagacattagaatcattgctgtaaacaagtttggatt :  29300
Seq3 : tggttcccataagtttggcagctagcagattcagttttgaaacagcatctgcattaactagaggagacattagaatcattgctgtaaacaagtttggatt :  29300
Seq4 : tggttcccataagtttggcagctagcagattcagttttgaaacagcatctgcattaactagaggagacattagaatcattgctgtaaacaagtttggatt :  29300

                 *     29320         *     29340         *     29360         *     29380         *     29400
Seq1 : atcgtaagaggctagctcccatggaatgacccaataagtagatttaatagttaccacgtgctgtaccaaagtcatcaatcatcatttttttcaccattact :  29400
Seq2 : atcgtaagaggctagctcccatggaatgacccaataagtagatttaatagttaccacgtgctgtaccaaagtcatcaatcatcatttttttcaccattact :  29400
Seq3 : atcgtaagaggctagctcccatggaatgacccaataagtagatttaatagttaccacgtgctgtaccaaagtcatcaatcatcatttttttcaccattact :  29400
Seq4 : atcgtaagaggctagctcccatggaatgacccaataagtagatttaatagttaccacgtgctgtaccaaagtcatcaatcatcatttttttcaccattact :  29400
```

```
              *     29420         *     29440         *     29460         *     29480         *     29500
Seq1 : tcttccatgtccaatatgatcatgtgagaatactaaaattcctaacgatgatatgttttcagctagttcgtcataacgtccagaatgtttaccagctcca :  29500
Seq2 : tcttccatgtccaatatgatcatgtgagaatactaaaattcctaacgatgatatgttttcagctagttcgtcataacgtccagaatgtttaccagctcca :  29500
Seq3 : tcttccatgtccaatatgatcatgtgagaatactaaaattcctaacgatgatatgttttcagctagttcgtcataacgtccagaatgtttaccagctcca :  29500
Seq4 : tcttccatgtccaatatgatcatgtgagaatactaaaattcctaacgatgatatgttttcagctagttcgtcataacgtccagaatgtttaccagctcca :  29500

              *     29520         *     29540         *     29560         *     29580         *     29600
Seq1 : tgacttatgaatactaatgccttaggatatgtaataggtttccaatatatgtaatcattgtccagattgaacatacagtttgcactcatgattcacgtta :  29600
Seq2 : tgacttatgaatactaatgccttaggatatgtaataggtttccaatatatgtaatcattgtccagattgaacatacagtttgcactcatgattcacgtta :  29600
Seq3 : tgacttatgaatactaatgccttaggatatgtaataggtttccaatatatgtaatcattgtccagattgaacatacagtttgcactcatgattcacgtta :  29600
Seq4 : tgacttatgaatactaatgccttaggatatgtaataggtttccaatatatgtaatcattgtccagattgaacatacagtttgcactcatgattcacgtta :  29600

              *     29620         *     29640         *     29660         *     29680         *     29700
Seq1 : tataactatcaatattaacagttcgtttgatgatcatattatttttatgtttattgataattgtaaaaacatacaattaaatcaatatagaggaaggag :  29700
Seq2 : tataactatcaatattaacagttcgtttgatgatcatattatttttatgtttattgataattgtaaaaacatacaattaaatcaatatagaggaaggag :  29700
Seq3 : tataactatcaatattaacagttcgtttgatgatcatattatttttatgtttattgataattgtaaaaacatacaattaaatcaatatagaggaaggag :  29700
Seq4 : tataactatcaatattaacagttcgtttgatgatcatattatttttatgtttattgataattgtaaaaacatacaattaaatcaatatagaggaaggag :  29700

              *     29720         *     29740         *     29760         *     29780         *     29800
Seq1 : acggctactgtcttttgtgagatagtcatggcgactaaattagattatgaggatgctgttttttactttgtggatgatgataaaatatgtagtcgcgact :  29800
Seq2 : acggctactgtcttttgtgagatagtcatggcgactaaattagattatgaggatgctgttttttactttgtggatgatgataaaatatgtagtcgcgact :  29800
Seq3 : acggctactgtcttttgtgagatagtcatggcgactaaattagattatgaggatgctgttttttactttgtggatgatgataaaatatgtagtcgcgact :  29800
Seq4 : acggctactgtcttttgtgagatagtcatggcgactaaattagattatgaggatgctgttttttactttgtggatgatgataaaatatgtagtcgcgact :  29800

              *     29820         *     29840         *     29860         *     29880         *     29900
Seq1 : ccatcatcgatctaatagatgaatatattacgtggagaaatcatgttatagtgtttaacaaagatattaccagttgtggaagactgtacaaggaattgat :  29900
Seq2 : ccatcatcgatctaatagatgaatatattacgtggagaaatcatgttatagtgtttaacaaagatattaccagttgtggaagactgtacaaggaattgat :  29900
Seq3 : ccatcatcgatctaatagatgaatatattacgtggagaaatcatgttatagtgtttaacaaagatattaccagttgtggaagactgtacaaggaattgat :  29900
Seq4 : ccatcatcgatctaatagatgaatatattacgtggagaaatcatgttatagtgtttaacaaagatattaccagttgtggaagactgtacaaggaattgat :  29900

              *     29920         *     29940         *     29960         *     29980         *     30000
Seq1 : gaagttcgatgatgtcgctatacggtactatggtattgataaaattaatgagattgtcgaagctatgagcgaaggagaccactacatcaattttacaaaa :  30000
Seq2 : gaagttcgatgatgtcgctatacggtactatggtattgataaaattaatgagattgtcgaagctatgagcgaaggagaccactacatcaattttacaaaa :  30000
Seq3 : gaagttcgatgatgtcgctatacggtactatggtattgataaaattaatgagattgtcgaagctatgagcgaaggagaccactacatcaattttacaaaa :  30000
Seq4 : gaagttcgatgatgtcgctatacggtactatggtattgataaaattaatgagattgtcgaagctatgagcgaaggagaccactacatcaattttacaaaa :  30000

              *     30020         *     30040         *     30060         *     30080         *     30100
Seq1 : gtccatgatcaggaaagtttattcgctaccataggaatatgtgctaaaatcactgaacattggggatacaaaaagatttcagaatctagattccaatcat :  30100
Seq2 : gtccatgatcaggaaagtttattcgctaccataggaatatgtgctaaaatcactgaacattggggatacaaaaagatttcagaatctagattccaatcat :  30100
Seq3 : gtccatgatcaggaaagtttattcgctaccataggaatatgtgctaaaatcactgaacattggggatacaaaaagatttcagaatctagattccaatcat :  30100
Seq4 : gtccatgatcaggaaagtttattcgctaccataggaatatgtgctaaaatcactgaacattggggatacaaaaagatttcagaatctagattccaatcat :  30100
```

```
               *     30120         *     30140         *     30160         *     30180         *     30200
Seq1 : tgggaaacattacagatctgatgaccgacgataatataaacatcttgatactttttctagaaaaaaaattgaattgatgatataggggtcttcataacgc :  30200
Seq2 : tgggaaacattacagatctgatgaccgacgataatataaacatcttgatactttttctagaaaaaaaattgaattgatgatataggggtcttcataacgc :  30200
Seq3 : tgggaaacattacagatctgatgaccgacgataatataaacatcttgatactttttctagaaaaaaaattgaattgatgatataggggtcttcataacgc :  30200
Seq4 : tgggaaacattacagatctgatgaccgacgataatataaacatcttgatactttttctagaaaaaaaattgaattgatgatataggggtcttcataacgc :  30200

               *     30220         *     30240         *     30260         *     30280         *     30300
Seq1 : ataattattacgttagcattctatatccgtgttaaaaaaaattatcctatcatgtatttgagagttttatatgtagcaaacatgatagctgtgatgccaa :  30300
Seq2 : ataattattacgttagcattctatatccgtgttaaaaaaaattatcctatcatgtatttgagagttttatatgtagcaaacatgatagctgtgatgccaa :  30300
Seq3 : ataattattacgttagcattctatatccgtgttaaaaaaaattatcctatcatgtatttgagagttttatatgtagcaaacatgatagctgtgatgccaa :  30300
Seq4 : ataattattacgttagcattctatatccgtgttaaaaaaaattatcctatcatgtatttgagagttttatatgtagcaaacatgatagctgtgatgccaa :  30300

               *     30320         *     30340         *     30360         *     30380         *     30400
Seq1 : taagctttagatattcacgcgtgctagtgttagggatggtattatctggtggtgaaatgtccgttatataatctacaaaataatcatcgcatatagtatg :  30400
Seq2 : taagctttagatattcacgcgtgctagtgttagggatggtattatctggtggtgaaatgtccgttatataatctacaaaataatcatcgcatatagtatg :  30400
Seq3 : taagctttagatattcacgcgtgctagtgttagggatggtattatctggtggtgaaatgtccgttatataatctacaaaataatcatcgcatatagtatg :  30400
Seq4 : taagctttagatattcacgcgtgctagtgttagggatggtattatctggtggtgaaatgtccgttatataatctacaaaataatcatcgcatatagtatg :  30400

               *     30420         *     30440         *     30460         *     30480         *     30500
Seq1 : cgatagtagagtaaacatttttatcgtttctactgggttcatacatcgtctacccaattcggttataaatgaaattgtcgccaatcttacacccaacccc :  30500
Seq2 : cgatagtagagtaaacatttttatcgtttctactgggttcatacatcgtctacccaattcggttataaatgaaattgtcgccaatcttacacccaacccc :  30500
Seq3 : cgatagtagagtaaacatttttatcgtttctactgggttcatacatcgtctacccaattcggttataaatgaaattgtcgccaatcttacacccaacccc :  30500
Seq4 : cgatagtagagtaaacatttttatcgtttctactgggttcatacatcgtctacccaattcggttataaatgaaattgtcgccaatcttacacccaacccc :  30500

               *     30520         *     30540         *     30560         *     30580         *     30600
Seq1 : ttgttatccattagtatagtattaacttcgttatttatgtcataaactgtaaatgattttgtagatgccatatcatacatgatattcatgtccctattat :  30600
Seq2 : ttgttatccattagtatagtattaacttcgttatttatgtcataaactgtaaatgattttgtagatgccatatcatacatgatattcatgtccctattat :  30600
Seq3 : ttgttatccattagtatagtattaacttcgttatttatgtcataaactgtaaatgattttgtagatgccatatcatacatgatattcatgtccctattat :  30600
Seq4 : ttgttatccattagtatagtattaacttcgttatttatgtcataaactgtaaatgattttgtagatgccatatcatacatgatattcatgtccctattat :  30600

               *     30620         *     30640         *     30660         *     30680         *     30700
Seq1 : aatcattactaactttatcacaatatatgttgataatatctatatatgatctagtctttgtgggcaactgtctatacaagtcgtctaaacgttgtttact :  30700
Seq2 : aatcattactaactttatcacaatatatgttgataatatctatatatgatctagtctttgtgggcaactgtctatacaagtcgtctaaacgttgtttact :  30700
Seq3 : aatcattactaactttatcacaatatatgttgataatatctatatatgatctagtctttgtgggcaactgtctatacaagtcgtctaaacgttgtttact :  30700
Seq4 : aatcattactaactttatcacaatatatgttgataatatctatatatgatctagtctttgtgggcaactgtctatacaagtcgtctaaacgttgtttact :  30700

               *     30720         *     30740         *     30760         *     30780         *     30800
Seq1 : catatagtatcgaacagccatcattacatggtcccgttccgttgatagataatcgagtatgttagtggacttgtcaaatctatataccatattttctgga :  30800
Seq2 : catatagtatcgaacagccatcattacatggtcccgttccgttgatagataatcgagtatgttagtggacttgtcaaatctatataccatattttctgga :  30800
Seq3 : catatagtatcgaacagccatcattacatggtcccgttccgttgatagataatcgagtatgttagtggacttgtcaaatctatataccatattttctgga :  30800
Seq4 : catatagtatcgaacagccatcattacatggtcccgttccgttgatagataatcgagtatgttagtggacttgtcaaatctatataccatattttctgga :  30800
```

```
               *     30820         *     30840         *     30860         *     30880         *     30900
Seq1 : agtggatatacatagtcgtgatcaacattattgctagcctcatcttctatatcctgtactataccattatctatatcatctacataatctacgatattat :  30900
Seq2 : agtggatatacatagtcgtgatcaacattattgctagcctcatcttctatatcctgtactataccattatctatatcatctacataatctacgatattat :  30900
Seq3 : agtggatatacatagtcgtgatcaacattattgctagcctcatcttctatatcctgtactataccattatctatatcatctacataatctacgatattat :  30900
Seq4 : agtggatatacatagtcgtgatcaacattattgctagcctcatcttctatatcctgtactataccattatctatatcatctacataatctacgatattat :  30900

               *     30920         *     30940         *     30960         *     30980         *     31000
Seq1 : tacacataaacatcgacaacatactattgtttattatctaagtcctgttgatccaaacccttgatctcctctatttgtactatctagagattgtacttct :  31000
Seq2 : tacacataaacatcgacaacatactattgtttattatctaagtcctgttgatccaaacccttgatctcctctatttgtactatctagagattgtacttct :  31000
Seq3 : tacacataaacatcgacaacatactattgtttattatctaagtcctgttgatccaaacccttgatctcctctatttgtactatctagagattgtacttct :  31000
Seq4 : tacacataaacatcgacaacatactattgtttattatctaagtcctgttgatccaaacccttgatctcctctatttgtactatctagagattgtacttct :  31000

               *     31020         *     31040         *     31060         *     31080         *     31100
Seq1 : tccagttctggataatatatacgttgatagattagctgagctattctatctccagtatttacattaaacgtacattttccattattaataagaatgactc :  31100
Seq2 : tccagttctggataatatatacgttgatagattagctgagctattctatctccagtatttacattaaacgtacattttccattattaataagaatgactc :  31100
Seq3 : tccagttctggataatatatacgttgatagattagctgagctattctatctccagtatttacattaaacgtacattttccattattaataagaatgactc :  31100
Seq4 : tccagttctggataatatatacgttgatagattagctgagctattctatctccagtatttacattaaacgtacattttccattattaataagaatgactc :  31100

               *     31120         *     31140         *     31160         *     31180         *     31200
Seq1 : ctatgtttcccctataatcttcgtctattacaccgcctcctatatcaatgccttttagggacagaccagacctaggagctattctaccatagcagaactt :  31200
Seq2 : ctatgtttcccctataatcttcgtctattacaccgcctcctatatcaatgccttttagggacagaccagacctaggagctattctaccatagcagaactt :  31200
Seq3 : ctatgtttcccctataatcttcgtctattacaccgcctcctatatcaatgccttttagggacagaccagacctaggagctattctaccatagcagaactt :  31200
Seq4 : ctatgtttcccctataatcttcgtctattacaccgcctcctatatcaatgccttttagggacagaccagacctaggagctattctaccatagcagaactt :  31200

               *     31220         *     31240         *     31260         *     31280         *     31300
Seq1 : aggcatggacatactaatatctgtcttaattaactgtcgttctcctggagggatagtataatcgtaagcgctatataaatcatatccggcggcgtaaggt :  31300
Seq2 : aggcatggacatactaatatctgtcttaattaactgtcgttctcctggagggatagtataatcgtaagcgctatataaatcatatccggcggcgtaaggt :  31300
Seq3 : aggcatggacatactaatatctgtcttaattaactgtcgttctcctggagggatagtataatcgtaagcgctatataaatcatatccggcggcgtaaggt :  31300
Seq4 : aggcatggacatactaatatctgtcttaattaactgtcgttctcctggagggatagtataatcgtaagcgctatataaatcatatccggcggcgtaaggt :  31300

               *     31320         *     31340         *     31360         *     31380         *     31400
Seq1 : gattgcctagtaggagatttagctctgttagtttccttaacaaatctaactggtgagttaatattcatgttgaacataaaactaatatttatttcaaaa :  31400
Seq2 : gattgcctagtaggagatttagctctgttagtttccttaacaaatctaactggtgagttaatattcatgttgaacataaaactaatatttatttcaaaa :  31400
Seq3 : gattgcctagtaggagatttagctctgttagtttccttaacaaatctaactggtgagttaatattcatgttgaacataaaactaatatttatttcaaaa :  31400
Seq4 : gattgcctagtaggagatttagctctgttagtttccttaacaaatctaactggtgagttaatattcatgttgaacataaaactaatatttatttcaaaa :  31400

               *     31420         *     31440         *     31460         *     31480         *     31500
Seq1 : ttatttaccatcccatatattccatgaataagtgtgatgattgtacacttctatagtatctatatacgattcacgataaaatcctcctatcaatagcagt :  31500
Seq2 : ttatttaccatcccatatattccatgaataagtgtgatgattgtacacttctatagtatctatatacgattcacgataaaatcctcctatcaatagcagt :  31500
Seq3 : ttatttaccatcccatatattccatgaataagtgtgatgattgtacacttctatagtatctatatacgattcacgataaaatcctcctatcaatagcagt :  31500
Seq4 : ttatttaccatcccatatattccatgaataagtgtgatgattgtacacttctatagtatctatatacgattcacgataaaatcctcctatcaatagcagt :  31500
```

```
                *     31520         *     31540         *     31560         *     31580         *     31600
Seq1 : ttattatccactatgatcaattctggattatccctcggataaataggatcatctatcagagtccatgtattgctggattcacaataaaattccgcatttc :  31600
Seq2 : ttattatccactatgatcaattctggattatccctcggataaataggatcatctatcagagtccatgtattgctggattcacaataaaattccgcatttc :  31600
Seq3 : ttattatccactatgatcaattctggattatccctcggataaataggatcatctatcagagtccatgtattgctggattcacaataaaattccgcatttc :  31600
Seq4 : ttattatccactatgatcaattctggattatccctcggataaataggatcatctatcagagtccatgtattgctggattcacaataaaattccgcatttc :  31600

                *     31620         *     31640         *     31660         *     31680         *     31700
Seq1 : taccaaccaagaataaccttctaccgaacactaacgcgcatgatttataatgaggataataagtggatggtccaaactgccactgatcatgattgggtag :  31700
Seq2 : taccaaccaagaataaccttctaccgaacactaacgcgcatgatttataatgaggataataagtggatggtccaaactgccactgatcatgattgggtag :  31700
Seq3 : taccaaccaagaataaccttctaccgaacactaacgcgcatgatttataatgaggataataagtggatggtccaaactgccactgatcatgattgggtag :  31700
Seq4 : taccaaccaagaataaccttctaccgaacactaacgcgcatgatttataatgaggataataagtggatggtccaaactgccactgatcatgattgggtag :  31700

                *     31720         *     31740         *     31760         *     31780         *     31800
Seq1 : caaatattctgtagttgtatcagtttcagaatgtcctcccattacgtatataacattgtttatggatgccactgctggattacatctaggtttcagaaga :  31800
Seq2 : caaatattctgtagttgtatcagtttcagaatgtcctcccattacgtatataacattgtttatggatgccactgctggattacatctaggtttcagaaga :  31800
Seq3 : caaatattctgtagttgtatcagtttcagaatgtcctcccattacgtatataacattgtttatggatgccactgctggattacatctaggtttcagaaga :  31800
Seq4 : caaatattctgtagttgtatcagtttcagaatgtcctcccattacgtatataacattgtttatggatgccactgctggattacatctaggtttcagaaga :  31800

                *     31820         *     31840         *     31860         *     31880         *     31900
Seq1 : ctcggcatattaacccaagcagcatccccgtggaaccaacgctcaacagatgtgggatttggtagacctcctactacgtataatttattgttagcgggta :  31900
Seq2 : ctcggcatattaacccaagcagcatccccgtggaaccaacgctcaacagatgtgggatttggtagacctcctactacgtataatttattgttagcgggta :  31900
Seq3 : ctcggcatattaacccaagcagcatccccgtggaaccaacgctcaacagatgtgggatttggtagacctcctactacgtataatttattgttagcgggta :  31900
Seq4 : ctcggcatattaacccaagcagcatccccgtggaaccaacgctcaacagatgtgggatttggtagacctcctactacgtataatttattgttagcgggta :  31900

                *     31920         *     31940         *     31960         *     31980         *     32000
Seq1 : tcccgctagcatacagtctggggctattcatcggaggaattggaatccaattgtttgatatataatttaccgctatagcattgttatgtatttcattgtt :  32000
Seq2 : tcccgctagcatacagtctggggctattcatcggaggaattggaatccaattgtttgatatataatttaccgctatagcattgttatgtatttcattgtt :  32000
Seq3 : tcccgctagcatacagtctggggctattcatcggaggaattggaatccaattgtttgatatataatttaccgctatagcattgttatgtatttcattgtt :  32000
Seq4 : tcccgctagcatacagtctggggctattcatcggaggaattggaatccaattgtttgatatataatttaccgctatagcattgttatgtatttcattgtt :  32000

                *     32020         *     32040         *     32060         *     32080         *     32100
Seq1 : catccatccaccgatgagatatactacttctccaacatgagtacttgtacacatatggaatatatctataatttgatccatgttcataggatactctatg :  32100
Seq2 : catccatccaccgatgagatatactacttctccaacatgagtacttgtacacatatggaatatatctataatttgatccatgttcataggatactctatg :  32100
Seq3 : catccatccaccgatgagatatactacttctccaacatgagtacttgtacacatatggaatatatctataatttgatccatgttcataggatactctatg :  32100
Seq4 : catccatccaccgatgagatatactacttctccaacatgagtacttgtacacatatggaatatatctataatttgatccatgttcataggatactctatg :  32100

                *     32120         *     32140         *     32160         *     32180         *     32200
Seq1 : aatggatacttgtatgatttgcgtggttgtttatcacaatgaaatatttggtacagtctagtatccattttacattatttatacctctgggagaaagat :  32200
Seq2 : aatggatacttgtatgatttgcgtggttgtttatcacaatgaaatatttggtacagtctagtatccattttacattatttatacctctgggagaaagat :  32200
Seq3 : aatggatacttgtatgatttgcgtggttgtttatcacaatgaaatatttggtacagtctagtatccattttacattatttatacctctgggagaaagat :  32200
Seq4 : aatggatacttgtatgatttgcgtggttgtttatcacaatgaaatatttggtacagtctagtatccattttacattatttatacctctgggagaaagat :  32200
```

```
                *     32220          *     32240          *     32260          *     32280          *     32300
Seq1 : aatttgacctgattacattttgataaggagtagcagatttcctaatttatttcttcgctttatataccacttaatgacaaaatcaactacataatcctc :  32300
Seq2 : aatttgacctgattacattttgataaggagtagcagatttcctaatttatttcttcgctttatataccacttaatgacaaaatcaactacataatcctc :  32300
Seq3 : aatttgacctgattacattttgataaggagtagcagatttcctaatttatttcttcgctttatataccacttaatgacaaaatcaactacataatcctc :  32300
Seq4 : aatttgacctgattacattttgataaggagtagcagatttcctaatttatttcttcgctttatataccacttaatgacaaaatcaactacataatcctc :  32300

                *     32320          *     32340          *     32360          *     32380          *     32400
Seq1 : atctggaacatttagttcatcgctttctagaataagtttcatagatagataatcaaaattgtctatgatgtcatcttccagttccaaaaagtgtttggca :  32400
Seq2 : atctggaacatttagttcatcgctttctagaataagtttcatagatagataatcaaaattgtctatgatgtcatcttccagttccaaaaagtgtttggca :  32400
Seq3 : atctggaacatttagttcatcgctttctagaataagtttcatagatagataatcaaaattgtctatgatgtcatcttccagttccaaaaagtgtttggca :  32400
Seq4 : atctggaacatttagttcatcgctttctagaataagtttcatagatagataatcaaaattgtctatgatgtcatcttccagttccaaaaagtgtttggca :  32400

                *     32420          *     32440          *     32460          *     32480          *     32500
Seq1 : ataaagttttagtatgacataagagattggatagtccgtattctatacccatcatgtaacactcgacacaatattcctttctaaaatctcgtaagataa :  32500
Seq2 : ataaagttttagtatgacataagagattggatagtccgtattctatacccatcatgtaacactcgacacaatattcctttctaaaatctcgtaagataa :  32500
Seq3 : ataaagttttagtatgacataagagattggatagtccgtattctatacccatcatgtaacactcgacacaatattcctttctaaaatctcgtaagataa :  32500
Seq4 : ataaagttttagtatgacataagagattggatagtccgtattctatacccatcatgtaacactcgacacaatattcctttctaaaatctcgtaagataa :  32500

                *     32520          *     32540          *     32560          *     32580          *     32600
Seq1 : agtttatacaagtgtagatgataaattctacagaggttaatatagaagcacgtaataaattgacgacgttatgactatctatatatacctttccagtata :  32600
Seq2 : agtttatacaagtgtagatgataaattctacagaggttaatatagaagcacgtaataaattgacgacgttatgactatctatatatacctttccagtata :  32600
Seq3 : agtttatacaagtgtagatgataaattctacagaggttaatatagaagcacgtaataaattgacgacgttatgactatctatatatacctttccagtata :  32600
Seq4 : agtttatacaagtgtagatgataaattctacagaggttaatatagaagcacgtaataaattgacgacgttatgactatctatatatacctttccagtata :  32600

                *     32620          *     32640          *     32660          *     32680          *     32700
Seq1 : cgagtaaataactatagaagttaaactgtgaatgtcaaggtctagacaaacccttgtaactggatctttattttcgtgtattttgacgtaaatgtgtg :  32700
Seq2 : cgagtaaataactatagaagttaaactgtgaatgtcaaggtctagacaaacccttgtaactggatctttattttcgtgtattttgacgtaaatgtgtg :  32700
Seq3 : cgagtaaataactatagaagttaaactgtgaatgtcaaggtctagacaaacccttgtaactggatctttattttcgtgtattttgacgtaaatgtgtg :  32700
Seq4 : cgagtaaataactatagaagttaaactgtgaatgtcaaggtctagacaaacccttgtaactggatctttattttcgtgtattttgacgtaaatgtgtg :  32700

                *     32720          *     32740          *     32760          *     32780          *     32800
Seq1 : cgaaagtaaggagataacttttcaatatcgtagaattgactattatattgccacctatagcatcaataattgttttgaatttcttagtcatagacaatg :  32800
Seq2 : cgaaagtaaggagataacttttcaatatcgtagaattgactattatattgccacctatagcatcaataattgttttgaatttcttagtcatagacaatg :  32800
Seq3 : cgaaagtaaggagataacttttcaatatcgtagaattgactattatattgccacctatagcatcaataattgttttgaatttcttagtcatagacaatg :  32800
Seq4 : cgaaagtaaggagataacttttcaatatcgtagaattgactattatattgccacctatagcatcaataattgttttgaatttcttagtcatagacaatg :  32800

                *     32820          *     32840          *     32860          *     32880          *     32900
Seq1 : ctaatatattcttacagtacacagtattaacaaatatcggcatttatgtttctttaaaagtcaacatctaaagaaaaatgattatcttcttgagacataa :  32900
Seq2 : ctaatatattcttacagtacacagtattaacaaatatcggcatttatgtttctttaaaagtcaacatctaaagaaaaatgattatcttcttgagacataa :  32900
Seq3 : ctaatatattcttacagtacacagtattaacaaatatcggcatttatgtttctttaaaagtcaacatctaaagaaaaatgattatcttcttgagacataa :  32900
Seq4 : ctaatatattcttacagtacacagtattaacaaatatcggcatttatgtttctttaaaagtcaacatctaaagaaaaatgattatcttcttgagacataa :  32900
```

```
                 *     32920         *     32940         *     32960         *     32980         *     33000
Seq1 : ctcccattttttggtattcacccacacgtttttcgaaaaaattagtttttccttccaatgatatattttccatgaaatcaaacggattggtaacattata :  33000
Seq2 : ctcccattttttggtattcacccacacgtttttcgaaaaaattagtttttccttccaatgatatattttccatgaaatcaaacggattggtaacattata :  33000
Seq3 : ctcccattttttggtattcacccacacgtttttcgaaaaaattagtttttccttccaatgatatattttccatgaaatcaaacggattggtaacattata :  33000
Seq4 : ctcccattttttggtattcacccacacgtttttcgaaaaaattagtttttccttccaatgatatattttccatgaaatcaaacggattggtaacattata :  33000

                 *     33020         *     33040         *     33060         *     33080         *     33100
Seq1 : aatttttttaaatcccaattcagaaatcaatctatccgcgacgaattctatatatgttttcatcatttcacaattcattcctataagtttaactggaaga :  33100
Seq2 : aatttttttaaatcccaattcagaaatcaatctatccgcgacgaattctatatatgttttcatcatttcacaattcattcctataagtttaactggaaga :  33100
Seq3 : aatttttttaaatcccaattcagaaatcaatctatccgcgacgaattctatatatgttttcatcatttcacaattcattcctataagtttaactggaaga :  33100
Seq4 : aatttttttaaatcccaattcagaaatcaatctatccgcgacgaattctatatatgttttcatcatttcacaattcattcctataagtttaactggaaga :  33100

                 *     33120         *     33140         *     33160         *     33180         *     33200
Seq1 : gccgcagtaagaaattcttgttcaatggataccgcatctgttataatagatctaacggtttcttcactcggtggatgcaataaatgtttaaacatcaaac :  33200
Seq2 : gccgcagtaagaaattcttgttcaatggataccgcatctgttataatagatctaacggtttcttcactcggtggatgcaataaatgtttaaacatcaaac :  33200
Seq3 : gccgcagtaagaaattcttgttcaatggataccgcatctgttataatagatctaacggtttcttcactcggtggatgcaataaatgtttaaacatcaaac :  33200
Seq4 : gccgcagtaagaaattcttgttcaatggataccgcatctgttataatagatctaacggtttcttcactcggtggatgcaataaatgtttaaacatcaaac :  33200

                 *     33220         *     33240         *     33260         *     33280         *     33300
Seq1 : atgcgaaatcgcagtgcagaccctcgtctctactaattagttcgttggaaaacgtgagtccgggcattaggccacgctttttaagccaaaatatggaagc :  33300
Seq2 : atgcgaaatcgcagtgcagaccctcgtctctactaattagttcgttggaaaacgtgagtccgggcattaggccacgctttttaagccaaaatatggaagc :  33300
Seq3 : atgcgaaatcgcagtgcagaccctcgtctctactaattagttcgttggaaaacgtgagtccgggcattaggccacgctttttaagccaaaatatggaagc :  33300
Seq4 : atgcgaaatcgcagtgcagaccctcgtctctactaattagttcgttggaaaacgtgagtccgggcattaggccacgctttttaagccaaaatatggaagc :  33300

                 *     33320         *     33340         *     33360         *     33380         *     33400
Seq1 : gaatgatccagaaaagaagattccttctactgcagcaaaggcaataagtctctctccataaccggcgctgtcatgtatccacttttgagcccaatcggcc :  33400
Seq2 : gaatgatccagaaaagaagattccttctactgcagcaaaggcaataagtctctctccataaccggcgctgtcatgtatccacttttgagcccaatcggcc :  33400
Seq3 : gaatgatccagaaaagaagattccttctactgcagcaaaggcaataagtctctctccataaccggcgctgtcatgtatccacttttgagcccaatcggcc :  33400
Seq4 : gaatgatccagaaaagaagattccttctactgcagcaaaggcaataagtctctctccataaccggcgctgtcatgtatccacttttgagcccaatcggcc :  33400

                 *     33420         *     33440         *     33460         *     33480         *     33500
Seq1 : ttcttttttacacaaggcatcgtttctatggcattaaagagatagtttttttcattactatctttaacataagtatcgatcaaaagactatacatttccg :  33500
Seq2 : ttcttttttacacaaggcatcgtttctatggcattaaagagatagtttttttcattactatctttaacataagtatcgatcaaaagactatacatttccg :  33500
Seq3 : ttcttttttacacaaggcatcgtttctatggcattaaagagatagtttttttcattactatctttaacataagtatcgatcaaaagactatacatttccg :  33500
Seq4 : ttcttttttacacaaggcatcgtttctatggcattaaagagatagtttttttcattactatctttaacataagtatcgatcaaaagactatacatttccg :  33500

                 *     33520         *     33540         *     33560         *     33580         *     33600
Seq1 : aatgaatgttttcaatggccatctgaaatccgtagaaacatctagcctcggtaatctgtacttctgtacaaaatcgttccgccaaattttcattcactat :  33600
Seq2 : aatgaatgttttcaatggccatctgaaatccgtagaaacatctagcctcggtaatctgtacttctgtacaaaatcgttccgccaaattttcattcactat :  33600
Seq3 : aatgaatgttttcaatggccatctgaaatccgtagaaacatctagcctcggtaatctgtacttctgtacaaaatcgttccgccaaattttcattcactat :  33600
Seq4 : aatgaatgttttcaatggccatctgaaatccgtagaaacatctagcctcggtaatctgtacttctgtacaaaatcgttccgccaaattttcattcactat :  33600
```

```
                *     33620         *     33640         *     33660         *     33680         *     33700
Seq1 : tccgtcactggctgcaaaaaacgccaatacatgttttataaaatatttttcgtctggtgttagtttattccaatcattgatatctttagatatatctact :  33700
Seq2 : tccgtcactggctgcaaaaaacgccaatacatgttttataaaatatttttcgtctggtgttagtttattccaatcattgatatctttagatatatctact :  33700
Seq3 : tccgtcactggctgcaaaaaacgccaatacatgttttataaaatatttttcgtctggtgttagtttattccaatcattgatatctttagatatatctact :  33700
Seq4 : tccgtcactggctgcaaaaaacgccaatacatgttttataaaatatttttcgtctggtgttagtttattccaatcattgatatctttagatatatctact :  33700

                *     33720         *     33740         *     33760         *     33780         *     33800
Seq1 : tcttccactgtccaaaatgatgcctctgccttttatacatgttccagatgtcataatattggattgggaaaataacaaatctatttggatttggtgcaa :  33800
Seq2 : tcttccactgtccaaaatgatgcctctgccttttatacatgttccagatgtcataatattggattgggaaaataacaaatctatttggatttggtgcaa :  33800
Seq3 : tcttccactgtccaaaatgatgcctctgccttttatacatgttccagatgtcataatattggattgggaaaataacaaatctatttggatttggtgcaa :  33800
Seq4 : tcttccactgtccaaaatgatgcctctgccttttatacatgttccagatgtcataatattggattgggaaaataacaaatctatttggatttggtgcaa :  33800

                *     33820         *     33840         *     33860         *     33880         *     33900
Seq1 : ggatgggttccataactaaattaacaataacaataaatttttttcagttatctatatgcctgtacttggatcttttgtacatcgatatcgccgcaatca :  33900
Seq2 : ggatgggttccataactaaattaacaataacaataaatttttttcagttatctatatgcctgtacttggatcttttgtacatcgatatcgccgcaatca :  33900
Seq3 : ggatgggttccataactaaattaacaataacaataaatttttttcagttatctatatgcctgtacttggatcttttgtacatcgatatcgccgcaatca :  33900
Seq4 : ggatgggttccataactaaattaacaataacaataaatttttttcagttatctatatgcctgtacttggatcttttgtacatcgatatcgccgcaatca :  33900

                *     33920         *     33940         *     33960         *     33980         *     34000
Seq1 : ctacaataattacaagtattattgatagcattgttattagtactatcataattaaattatcgacattcatgggtgctgaataatcgttattatcatcatt :  34000
Seq2 : ctacaataattacaagtattattgatagcattgttattagtactatcataattaaattatcgacattcatgggtgctgaataatcgttattatcatcatt :  34000
Seq3 : ctacaataattacaagtattattgatagcattgttattagtactatcataattaaattatcgacattcatgggtgctgaataatcgttattatcatcatt :  34000
Seq4 : ctacaataattacaagtattattgatagcattgttattagtactatcataattaaattatcgacattcatgggtgctgaataatcgttattatcatcatt :  34000

                *     34020         *     34040         *     34060         *     34080         *     34100
Seq1 : atcattttgtaattgtgacatcatactagataaatcgtttgcgagattgttgtgggaagcgggcatggaggatgaattatcgttattattatttaacgcc :  34100
Seq2 : atcattttgtaattgtgacatcatactagataaatcgtttgcgagattgttgtgggaagcgggcatggaggatgaattatcgttattattatttaacgcc :  34100
Seq3 : atcattttgtaattgtgacatcatactagataaatcgtttgcgagattgttgtgggaagcgggcatggaggatgaattatcgttattattatttaacgcc :  34100
Seq4 : atcattttgtaattgtgacatcatactagataaatcgtttgcgagattgttgtgggaagcgggcatggaggatgaattatcgttattattatttaacgcc :  34100

                *     34120         *     34140         *     34160         *     34180         *     34200
Seq1 : tcccattcggattcacaaatgttacgcacattcaacattttatggaaactataattttgtgaaaacagataacaagaaaactcgtcatcgttcaaatttt :  34200
Seq2 : tcccattcggattcacaaatgttacgcacattcaacattttatggaaactataattttgtgaaaacagataacaagaaaactcgtcatcgttcaaatttt :  34200
Seq3 : tcccattcggattcacaaatgttacgcacattcaacattttatggaaactataattttgtgaaaacagataacaagaaaactcgtcatcgttcaaatttt :  34200
Seq4 : tcccattcggattcacaaatgttacgcacattcaacattttatggaaactataattttgtgaaaacagataacaagaaaactcgtcatcgttcaaatttt :  34200

                *     34220         *     34240         *     34260         *     34280         *     34300
Seq1 : taacgatagtaaaccgattaaacgtcgagctaatttctaacgctagcgactctgttggatatgggtttccagatatatatcttttcagttcccctacgta :  34300
Seq2 : taacgatagtaaaccgattaaacgtcgagctaatttctaacgctagcgactctgttggatatgggtttccagatatatatcttttcagttcccctacgta :  34300
Seq3 : taacgatagtaaaccgattaaacgtcgagctaatttctaacgctagcgactctgttggatatgggtttccagatatatatcttttcagttcccctacgta :  34300
Seq4 : taacgatagtaaaccgattaaacgtcgagctaatttctaacgctagcgactctgttggatatgggtttccagatatatatcttttcagttcccctacgta :  34300
```

```
                 *      34320         *      34340         *      34360         *      34380         *      34400
Seq1 : tctataatcatctgtaggaaatggaagatatttccatttatctactgttcctaatatcatatgtggtggtgtagtagaaccattaagcgcgaaagatgtt :  34400
Seq2 : tctataatcatctgtaggaaatggaagatatttccatttatctactgttcctaatatcatatgtggtggtgtagtagaaccattaagcgcgaaagatgtt :  34400
Seq3 : tctataatcatctgtaggaaatggaagatatttccatttatctactgttcctaatatcatatgtggtggtgtagtagaaccattaagcgcgaaagatgtt :  34400
Seq4 : tctataatcatctgtaggaaatggaagatatttccatttatctactgttcctaatatcatatgtggtggtgtagtagaaccattaagcgcgaaagatgtt :  34400

                 *      34420         *      34440         *      34460         *      34480         *      34500
Seq1 : atttcgcatcgtattttaacttcgcaataatttctggttagataacgcactctaccagtcaagtcaatgatattagcctttacagatatattcatagtag :  34500
Seq2 : atttcgcatcgtattttaacttcgcaataatttctggttagataacgcactctaccagtcaagtcaatgatattagcctttacagatatattcatagtag :  34500
Seq3 : atttcgcatcgtattttaacttcgcaataatttctggttagataacgcactctaccagtcaagtcaatgatattagcctttacagatatattcatagtag :  34500
Seq4 : atttcgcatcgtattttaacttcgcaataatttctggttagataacgcactctaccagtcaagtcaatgatattagcctttacagatatattcatagtag :  34500

                 *      34520         *      34540         *      34560         *      34580         *      34600
Seq1 : ttgtaacgatgactccatcttttagatgcgatactcctttgtatgtaccagaatcttcgtaccgcaaactcgatatatttaaacaagttaatgagatatt :  34600
Seq2 : ttgtaacgatgactccatcttttagatgcgatactcctttgtatgtaccagaatcttcgtaccgcaaactcgatatatttaaacaagttaatgagatatt :  34600
Seq3 : ttgtaacgatgactccatcttttagatgcgatactcctttgtatgtaccagaatcttcgtaccgcaaactcgatatatttaaacaagttaatgagatatt :  34600
Seq4 : ttgtaacgatgactccatcttttagatgcgatactcctttgtatgtaccagaatcttcgtaccgcaaactcgatatatttaaacaagttaatgagatatt :  34600

                 *      34620         *      34640         *      34660         *      34680         *      34700
Seq1 : aacgcgttttatgaatgatgatatataaccagaagttttatcctcggtggctagcgctataaccttatcattataataccaactagtgtgattaatatgt :  34700
Seq2 : aacgcgttttatgaatgatgatatataaccagaagttttatcctcggtggctagcgctataaccttatcattataataccaactagtgtgattaatatgt :  34700
Seq3 : aacgcgttttatgaatgatgatatataaccagaagttttatcctcggtggctagcgctataaccttatcattataataccaactagtgtgattaatatgt :  34700
Seq4 : aacgcgttttatgaatgatgatatataaccagaagttttatcctcggtggctagcgctataaccttatcattataataccaactagtgtgattaatatgt :  34700

                 *      34720         *      34740         *      34760         *      34780         *      34800
Seq1 : gacacgttagtgtgggtacaaatatgtacattatcgtctacgtcgtattcgatacatccgcatacagccaacaaatataaaatgacaaatactctaacgc :  34800
Seq2 : gacacgttagtgtgggtacaaatatgtacattatcgtctacgtcgtattcgatacatccgcatacagccaacaaatataaaatgacaaatactctaacgc :  34800
Seq3 : gacacgttagtgtgggtacaaatatgtacattatcgtctacgtcgtattcgatacatccgcatacagccaacaaatataaaatgacaaatactctaacgc :  34800
Seq4 : gacacgttagtgtgggtacaaatatgtacattatcgtctacgtcgtattcgatacatccgcatacagccaacaaatataaaatgacaaatactctaacgc :  34800

                 *      34820         *      34840         *      34860         *      34880         *      34900
Seq1 : cgttcgtacccatcttgatgcggtttaataaatgttttgatttcaatttattgtaaaaaaagattcggttttatactgttcgatattctcattgcttata :  34900
Seq2 : cgttcgtacccatcttgatgcggtttaataaatgttttgatttcaatttattgtaaaaaaagattcggttttatactgttcgatattctcattgcttata :  34900
Seq3 : cgttcgtacccatcttgatgcggtttaataaatgttttgatttcaatttattgtaaaaaaagattcggttttatactgttcgatattctcattgcttata :  34900
Seq4 : cgttcgtacccatcttgatgcggtttaataaatgttttgatttcaatttattgtaaaaaaagattcggttttatactgttcgatattctcattgcttata :  34900

                 *      34920         *      34940         *      34960         *      34980         *      35000
Seq1 : ttttcatctatcatctccacacagtcaaatccgtggttagcatgcacctcatcaaccggtaaaagactatcggactcttctatcattataactctagaat :  35000
Seq2 : ttttcatctatcatctccacacagtcaaatccgtggttagcatgcacctcatcaaccggtaaaagactatcggactcttctatcattataactctagaat :  35000
Seq3 : ttttcatctatcatctccacacagtcaaatccgtggttagcatgcacctcatcaaccggtaaaagactatcggactcttctatcattataactctagaat :  35000
Seq4 : ttttcatctatcatctccacacagtcaaatccgtggttagcatgcacctcatcaaccggtaaaagactatcggactcttctatcattataactctagaat :  35000
```

```
                  *     35020          *     35040          *     35060          *     35080          *     35100
Seq1 : atttaatttggtcattattaatcaagtcaattatcttatttttaacaaacgtgagtattttactcattttttataaaaacttttagaaatatacagactc :  35100
Seq2 : atttaatttggtcattattaatcaagtcaattatcttatttttaacaaacgtgagtattttactcattttttataaaaacttttagaaatatacagactc :  35100
Seq3 : atttaatttggtcattattaatcaagtcaattatcttatttttaacaaacgtgagtattttactcattttttataaaaacttttagaaatatacagactc :  35100
Seq4 : atttaatttggtcattattaatcaagtcaattatcttatttttaacaaacgtgagtattttactcattttttataaaaacttttagaaatatacagactc :  35100

                  *     35120          *     35140          *     35160          *     35180          *     35200
Seq1 : tatcgtgtgtctatatcttctttttatatccaatgtatttatgtctgattttcttcatttatcatatataatggtccaaattctacacgtgcttcggat :  35200
Seq2 : tatcgtgtgtctatatcttctttttatatccaatgtatttatgtctgattttcttcatttatcatatataatggtccaaattctacacgtgcttcggat :  35200
Seq3 : tatcgtgtgtctatatcttctttttatatccaatgtatttatgtctgattttcttcatttatcatatataatggtccaaattctacacgtgcttcggat :  35200
Seq4 : tatcgtgtgtctatatcttctttttatatccaatgtatttatgtctgattttcttcatttatcatatataatggtccaaattctacacgtgcttcggat :  35200

                  *     35220          *     35240          *     35260          *     35280          *     35300
Seq1 : tcatccagatcattaaggttcttataattgtaacatccttctcttccctcttctacatcttccttcttattcttattcttagcgtcacagaatctaccac :  35300
Seq2 : tcatccagatcattaaggttcttataattgtaacatccttctcttccctcttctacatcttccttcttattcttattcttagcgtcacagaatctaccac :  35300
Seq3 : tcatccagatcattaaggttcttataattgtaacatccttctcttccctcttctacatcttccttcttattcttattcttagcgtcacagaatctaccac :  35300
Seq4 : tcatccagatcattaaggttcttataattgtaacatccttctcttccctcttctacatcttccttcttattcttattcttagcgtcacagaatctaccac :  35300

                  *     35320          *     35340          *     35360          *     35380          *     35400
Seq1 : agcaggatcccatgacgagcgtcatattaaactaatccattttcaattataatatacgattagtaatgaccattaaaataaaaaatattcttcataaccg :  35400
Seq2 : agcaggatcccatgacgagcgtcatattaaactaatccattttcaattataatatacgattagtaatgaccattaaaataaaaaatattcttcataaccg :  35400
Seq3 : agcaggatcccatgacgagcgtcatattaaactaatccattttcaattataatatacgattagtaatgaccattaaaataaaaaatattcttcataaccg :  35400
Seq4 : agcaggatcccatgacgagcgtcatattaaactaatccattttcaattataatatacgattagtaatgaccattaaaataaaaaatattcttcataaccg :  35400

                  *     35420          *     35440          *     35460          *     35480          *     35500
Seq1 : gcaagaaagtgaaaagttcacattgaaactatgtcagtagtatacatcatgaaatgatgatatatatatactctattttggtggaggattatatgatata :  35500
Seq2 : gcaagaaagtgaaaagttcacattgaaactatgtcagtagtatacatcatgaaatgatgatatatatatactctattttggtggaggattatatgatata :  35500
Seq3 : gcaagaaagtgaaaagttcacattgaaactatgtcagtagtatacatcatgaaatgatgatatatatatactctattttggtggaggattatatgatata :  35500
Seq4 : gcaagaaagtgaaaagttcacattgaaactatgtcagtagtatacatcatgaaatgatgatatatatatactctattttggtggaggattatatgatata :  35500

                  *     35520          *     35540          *     35560          *     35580          *     35600
Seq1 : attcgtggataatcattcttaagacacatttcttcattcgtaaatcttttcacgttaaatgagtgtccatattttgcaatttcttcatatgatggcggtg :  35600
Seq2 : attcgtggataatcattcttaagacacatttcttcattcgtaaatcttttcacgttaaatgagtgtccatattttgcaatttcttcatatgatggcggtg :  35600
Seq3 : attcgtggataatcattcttaagacacatttcttcattcgtaaatcttttcacgttaaatgagtgtccatattttgcaatttcttcatatgatggcggtg :  35600
Seq4 : attcgtggataatcattcttaagacacatttcttcattcgtaaatcttttcacgttaaatgagtgtccatattttgcaatttcttcatatgatggcggtg :  35600

                  *     35620          *     35640          *     35660          *     35680          *     35700
Seq1 : tacgtggacgaagctgctcctgttcttgttgtagtcgccgactgtcgtgtttgcgtttagatccctccattatcgcgattgcgtagatggagtactatta :  35700
Seq2 : tacgtggacgaagctgctcctgttcttgttgtagtcgccgactgtcgtgtttgcgtttagatccctccattatcgcgattgcgtagatggagtactatta :  35700
Seq3 : tacgtggacgaagctgctcctgttcttgttgtagtcgccgactgtcgtgtttgcgtttagatccctccattatcgcgattgcgtagatggagtactatta :  35700
Seq4 : tacgtggacgaagctgctcctgttcttgttgtagtcgccgactgtcgtgtttgcgtttagatccctccattatcgcgattgcgtagatggagtactatta :  35700
```

```
                    *     35720         *     35740         *     35760         *     35780         *     35800
Seq1 : tataccttgtaattaaatttttttattaattaaacgtataaaaacgttccgtatctgtatttaagagccagatttcgtctaatagaacaaatagctacag :  35800
Seq2 : tataccttgtaattaaatttttttattaattaaacgtataaaaacgttccgtatctgtatttaagagccagatttcgtctaatagaacaaatagctacag :  35800
Seq3 : tataccttgtaattaaatttttttattaattaaacgtataaaaacgttccgtatctgtatttaagagccagatttcgtctaatagaacaaatagctacag :  35800
Seq4 : tataccttgtaattaaatttttttattaattaaacgtataaaaacgttccgtatctgtatttaagagccagatttcgtctaatagaacaaatagctacag :  35800

                    *     35820         *     35840         *     35860         *     35880         *     35900
Seq1 : taaaaataactagaataattgctacacccactagaaaccacggatcgtaatacggcaatcggttttcgataataggtggaacgtatatttatttaagga :  35900
Seq2 : taaaaataactagaataattgctacacccactagaaaccacggatcgtaatacggcaatcggttttcgataataggtggaacgtatatttatttaagga :  35900
Seq3 : taaaaataactagaataattgctacacccactagaaaccacggatcgtaatacggcaatcggttttcgataataggtggaacgtatatttatttaagga :  35900
Seq4 : taaaaataactagaataattgctacacccactagaaaccacggatcgtaatacggcaatcggttttcgataataggtggaacgtatatttatttaagga :  35900

                    *     35920         *     35940         *     35960         *     35980         *     36000
Seq1 : cttaacaattgtctgtaaaccacaatttgcttccgcggatcctgtattaactatctgtaaaagcatatgttgaccgggcggagccgaacattctccgata :  36000
Seq2 : cttaacaattgtctgtaaaccacaatttgcttccgcggatcctgtattaactatctgtaaaagcatatgttgaccgggcggagccgaacattctccgata :  36000
Seq3 : cttaacaattgtctgtaaaccacaatttgcttccgcggatcctgtattaactatctgtaaaagcatatgttgaccgggcggagccgaacattctccgata :  36000
Seq4 : cttaacaattgtctgtaaaccacaatttgcttccgcggatcctgtattaactatctgtaaaagcatatgttgaccgggcggagccgaacattctccgata :  36000

                    *     36020         *     36040         *     36060         *     36080         *     36100
Seq1 : tctaatttctgtatatctataatattattaacctccgcatacgcattacagttcttttctagcttggataccgcactaggtacatcgtctagatctattc :  36100
Seq2 : tctaatttctgtatatctataatattattaacctccgcatacgcattacagttcttttctagcttggataccgcactaggtacatcgtctagatctattc :  36100
Seq3 : tctaatttctgtatatctataatattattaacctccgcatacgcattacagttcttttctagcttggataccgcactaggtacatcgtctagatctattc :  36100
Seq4 : tctaatttctgtatatctataatattattaacctccgcatacgcattacagttcttttctagcttggataccgcactaggtacatcgtctagatctattc :  36100

                    *     36120         *     36140         *     36160         *     36180         *     36200
Seq1 : ctatttcttcagcgatagctcttctatccttttccggaagcaatgaaatcacttcaataaatgattcaaccatgagtgtgaaactaagtcgagaattact :  36200
Seq2 : ctatttcttcagcgatagctcttctatccttttccggaagcaatgaaatcacttcaataaatgattcaaccatgagtgtgaaactaagtcgagaattact :  36200
Seq3 : ctatttcttcagcgatagctcttctatccttttccggaagcaatgaaatcacttcaataaatgattcaaccatgagtgtgaaactaagtcgagaattact :  36200
Seq4 : ctatttcttcagcgatagctcttctatccttttccggaagcaatgaaatcacttcaataaatgattcaaccatgagtgtgaaactaagtcgagaattact :  36200

                    *     36220         *     36240         *     36260         *     36280         *     36300
Seq1 : catgcatttgttagttattcggagcgcgcaatttttaaactgtcctataacctctcctatatgaatagcacaagtgacattagtagggatagaatgttga :  36300
Seq2 : catgcatttgttagttattcggagcgcgcaatttttaaactgtcctataacctctcctatatgaatagcacaagtgacattagtagggatagaatgttga :  36300
Seq3 : catgcatttgttagttattcggagcgcgcaatttttaaactgtcctataacctctcctatatgaatagcacaagtgacattagtagggatagaatgttga :  36300
Seq4 : catgcatttgttagttattcggagcgcgcaatttttaaactgtcctataacctctcctatatgaatagcacaagtgacattagtagggatagaatgttga :  36300

                    *     36320         *     36340         *     36360         *     36380         *     36400
Seq1 : gctaattttgtaaataactatctataaaaagattatacaaagttttaaactctttagtttccgccatttatccagtctgagaaaatgtctctcataata :  36400
Seq2 : gctaattttgtaaataactatctataaaaagattatacaaagttttaaactctttagtttccgccatttatccagtctgagaaaatgtctctcataata :  36400
Seq3 : gctaattttgtaaataactatctataaaaagattatacaaagttttaaactctttagtttccgccatttatccagtctgagaaaatgtctctcataata :  36400
Seq4 : gctaattttgtaaataactatctataaaaagattatacaaagttttaaactctttagtttccgccatttatccagtctgagaaaatgtctctcataata :  36400
```

```
                 *     36420         *     36440         *     36460         *     36480         *     36500
Seq1 : aatttttccaagaaactaattgggtgaagaatggaaacctttaatctatatttatcacagtctgttttggtacacatgatgaattcttctaatgctgtac :  36500
Seq2 : aatttttccaagaaactaattgggtgaagaatggaaacctttaatctatatttatcacagtctgttttggtacacatgatgaattcttctaatgctgtac :  36500
Seq3 : aatttttccaagaaactaattgggtgaagaatggaaacctttaatctatatttatcacagtctgttttggtacacatgatgaattcttctaatgctgtac :  36500
Seq4 : aatttttccaagaaactaattgggtgaagaatggaaacctttaatctatatttatcacagtctgttttggtacacatgatgaattcttctaatgctgtac :  36500

                 *     36520         *     36540         *     36560         *     36580         *     36600
Seq1 : taaattcgatatctttttcgatttctggatatgtttttaataaagtatgaacaaagaaatggaaatcgtaataccagttatgttcaactttgaaattgtt :  36600
Seq2 : taaattcgatatctttttcgatttctggatatgtttttaataaagtatgaacaaagaaatggaaatcgtaataccagttatgttcaactttgaaattgtt :  36600
Seq3 : taaattcgatatctttttcgatttctggatatgtttttaataaagtatgaacaaagaaatggaaatcgtaataccagttatgttcaactttgaaattgtt :  36600
Seq4 : taaattcgatatctttttcgatttctggatatgtttttaataaagtatgaacaaagaaatggaaatcgtaataccagttatgttcaactttgaaattgtt :  36600

                 *     36620         *     36640         *     36660         *     36680         *     36700
Seq1 : ttttattttcttgttaatgattccagccacttgggaaaagtcaaagtcgtttaatgccgatttaatacgttcattaaaaacaaactttttatcctttaga :  36700
Seq2 : ttttattttcttgttaatgattccagccacttgggaaaagtcaaagtcgtttaatgccgatttaatacgttcattaaaaacaaactttttatcctttaga :  36700
Seq3 : ttttattttcttgttaatgattccagccacttgggaaaagtcaaagtcgtttaatgccgatttaatacgttcattaaaaacaaactttttatcctttaga :  36700
Seq4 : ttttattttcttgttaatgattccagccacttgggaaaagtcaaagtcgtttaatgccgatttaatacgttcattaaaaacaaactttttatcctttaga :  36700

                 *     36720         *     36740         *     36760         *     36780         *     36800
Seq1 : tgaattattattggttcattggaatcaaaaagtaagatattatcgggtttaagatctgcgtgtaaaaagttgtcgcaacagggtagttcgtagattttaa :  36800
Seq2 : tgaattattattggttcattggaatcaaaaagtaagatattatcgggtttaagatctgcgtgtaaaaagttgtcgcaacagggtagttcgtagattttaa :  36800
Seq3 : tgaattattattggttcattggaatcaaaaagtaagatattatcgggtttaagatctgcgtgtaaaaagttgtcgcaacagggtagttcgtagattttaa :  36800
Seq4 : tgaattattattggttcattggaatcaaaaagtaagatattatcgggtttaagatctgcgtgtaaaaagttgtcgcaacagggtagttcgtagattttaa :  36800

                 *     36820         *     36840         *     36860         *     36880         *     36900
Seq1 : tgtataacagagccatctgtaaaaagataaactttatgtattgtaccaaagatttaaatcctaatttgatagctaactcggtatctactttatctgccga :  36900
Seq2 : tgtataacagagccatctgtaaaaagataaactttatgtattgtaccaaagatttaaatcctaatttgatagctaactcggtatctactttatctgccga :  36900
Seq3 : tgtataacagagccatctgtaaaaagataaactttatgtattgtaccaaagatttaaatcctaatttgatagctaactcggtatctactttatctgccga :  36900
Seq4 : tgtataacagagccatctgtaaaaagataaactttatgtattgtaccaaagatttaaatcctaatttgatagctaactcggtatctactttatctgccga :  36900

                 *     36920         *     36940         *     36960         *     36980         *     37000
Seq1 : atacagtgctaggggaaaaattataatatttcctctttcgtattcgtagttagttctcttttcatgttcgaaaaagtgaaacatgcggttaaaatagttt :  37000
Seq2 : atacagtgctaggggaaaaattataatatttcctctttcgtattcgtagttagttctcttttcatgttcgaaaaagtgaaacatgcggttaaaatagttt :  37000
Seq3 : atacagtgctaggggaaaaattataatatttcctctttcgtattcgtagttagttctcttttcatgttcgaaaaagtgaaacatgcggttaaaatagttt :  37000
Seq4 : atacagtgctaggggaaaaattataatatttcctctttcgtattcgtagttagttctcttttcatgttcgaaaaagtgaaacatgcggttaaaatagttt :  37000

                 *     37020         *     37040         *     37060         *     37080         *     37100
Seq1 : ataacattaatattactgttaataactgccggataaaagtgggatagtaatttcacgaatttgatactgtcctttctctcgttaaacgcctttaaaaaaa :  37100
Seq2 : ataacattaatattactgttaataactgccggataaaagtgggatagtaatttcacgaatttgatactgtcctttctctcgttaaacgcctttaaaaaaa :  37100
Seq3 : ataacattaatattactgttaataactgccggataaaagtgggatagtaatttcacgaatttgatactgtcctttctctcgttaaacgcctttaaaaaaa :  37100
Seq4 : ataacattaatattactgttaataactgccggataaaagtgggatagtaatttcacgaatttgatactgtcctttctctcgttaaacgcctttaaaaaaa :  37100
```

```
                *      37120         *      37140         *      37160         *      37180         *      37200
Seq1 : ctttagaagaatatctcaatgagagttcctgaccatccatagtttgtatcaataatagcaacatatgaagaacccgtttatacagagtatgtaaaaatgt :  37200
Seq2 : ctttagaagaatatctcaatgagagttcctgaccatccatagtttgtatcaataatagcaacatatgaagaacccgtttatacagagtatgtaaaaatgt :  37200
Seq3 : ctttagaagaatatctcaatgagagttcctgaccatccatagtttgtatcaataatagcaacatatgaagaacccgtttatacagagtatgtaaaaatgt :  37200
Seq4 : ctttagaagaatatctcaatgagagttcctgaccatccatagtttgtatcaataatagcaacatatgaagaacccgtttatacagagtatgtaaaaatgt :  37200

                *      37220         *      37240         *      37260         *      37280         *      37300
Seq1 : taatttatagtttaatcccatggcccacgcacacacgattaattttttttcatctccctttagattgttgtatagaaatttgggtactgtgaactccgcc :  37300
Seq2 : taatttatagtttaatcccatggcccacgcacacacgattaattttttttcatctccctttagattgttgtatagaaatttgggtactgtgaactccgcc :  37300
Seq3 : taatttatagtttaatcccatggcccacgcacacacgattaattttttttcatctccctttagattgttgtatagaaatttgggtactgtgaactccgcc :  37300
Seq4 : taatttatagtttaatcccatggcccacgcacacacgattaattttttttcatctccctttagattgttgtatagaaatttgggtactgtgaactccgcc :  37300

                *      37320         *      37340         *      37360         *      37380         *      37400
Seq1 : gtagtttccatgggactatataattttgtggcctcgaatacaaattttactacatagttatctatcttaaagactataccatatcctcctgtagatatgt :  37400
Seq2 : gtagtttccatgggactatataattttgtggcctcgaatacaaattttactacatagttatctatcttaaagactataccatatcctcctgtagatatgt :  37400
Seq3 : gtagtttccatgggactatataattttgtggcctcgaatacaaattttactacatagttatctatcttaaagactataccatatcctcctgtagatatgt :  37400
Seq4 : gtagtttccatgggactatataattttgtggcctcgaatacaaattttactacatagttatctatcttaaagactataccatatcctcctgtagatatgt :  37400

                *      37420         *      37440         *      37460         *      37480         *      37500
Seq1 : gataaaaatcgtcgtttataggataaaatcgtttatccttttgttggaaaaaggatgaattaatgtaatcattctcttctatctttagtagtgtttcctt :  37500
Seq2 : gataaaaatcgtcgtttataggataaaatcgtttatccttttgttggaaaaaggatgaattaatgtaatcattctcttctatctttagtagtgtttcctt :  37500
Seq3 : gataaaaatcgtcgtttataggataaaatcgtttatccttttgttggaaaaaggatgaattaatgtaatcattctcttctatctttagtagtgtttcctt :  37500
Seq4 : gataaaaatcgtcgtttataggataaaatcgtttatccttttgttggaaaaaggatgaattaatgtaatcattctcttctatctttagtagtgtttcctt :  37500

                *      37520         *      37540         *      37560         *      37580         *      37600
Seq1 : attaaaattcttaaaataatttaacaatctaactgacggagcccaattttggtgtaaatctaattgggacattatgttgttaaaatacaaacagtctcct :  37600
Seq2 : attaaaattcttaaaataatttaacaatctaactgacggagcccaattttggtgtaaatctaattgggacattatgttgttaaaatacaaacagtctcct :  37600
Seq3 : attaaaattcttaaaataatttaacaatctaactgacggagcccaattttggtgtaaatctaattgggacattatgttgttaaaatacaaacagtctcct :  37600
Seq4 : attaaaattcttaaaataatttaacaatctaactgacggagcccaattttggtgtaaatctaattgggacattatgttgttaaaatacaaacagtctcct :  37600

                *      37620         *      37640         *      37660         *      37680         *      37700
Seq1 : aatataacagtatctgataatctatggggagacatccattgatattcaggggatgaatcattggcaacacccatttattgtacaaaaagccccaatttac :  37700
Seq2 : aatataacagtatctgataatctatggggagacatccattgatattcaggggatgaatcattggcaacacccatttattgtacaaaaagccccaatttac :  37700
Seq3 : aatataacagtatctgataatctatggggagacatccattgatattcaggggatgaatcattggcaacacccatttattgtacaaaaagccccaatttac :  37700
Seq4 : aatataacagtatctgataatctatggggagacatccattgatattcaggggatgaatcattggcaacacccatttattgtacaaaaagccccaatttac :  37700

                *      37720         *      37740         *      37760         *      37780         *      37800
Seq1 : aaacgaaagtccaggtttgatagagacaaacaattaactatttgtctctgtttttaatttctttggtaatgaaattattcacaatatcagtatcttctt :  37800
Seq2 : aaacgaaagtccaggtttgatagagacaaacaattaactatttgtctctgtttttaatttctttggtaatgaaattattcacaatatcagtatcttctt :  37800
Seq3 : aaacgaaagtccaggtttgatagagacaaacaattaactatttgtctctgtttttaatttctttggtaatgaaattattcacaatatcagtatcttctt :  37800
Seq4 : aaacgaaagtccaggtttgatagagacaaacaattaactatttgtctctgtttttaatttctttggtaatgaaattattcacaatatcagtatcttctt :  37800
```

```
               *     37820           *     37840           *     37860           *     37880           *     37900
Seq1 : tatctaccagagattttactaacttgataaccttggctgtctcattcaatagggtagtaatatttgtatgtgtgatattgatatctttttgaattgtttc :  37900
Seq2 : tatctaccagagattttactaacttgataaccttggctgtctcattcaatagggtagtaatatttgtatgtgtgatattgatatctttttgaattgtttc :  37900
Seq3 : tatctaccagagattttactaacttgataaccttggctgtctcattcaatagggtagtaatatttgtatgtgtgatattgatatctttttgaattgtttc :  37900
Seq4 : tatctaccagagattttactaacttgataaccttggctgtctcattcaatagggtagtaatatttgtatgtgtgatattgatatctttttgaattgtttc :  37900

               *     37920           *     37940           *     37960           *     37980           *     38000
Seq1 : ttttagaagtgattctttgatggtgccagaatacgaattacaataatgcagaaactcagttaacatgcaggaattatagtaagccaattccaattgttgc :  38000
Seq2 : ttttagaagtgattctttgatggtgccagaatacgaattacaataatgcagaaactcagttaacatgcaggaattatagtaagccaattccaattgttgc :  38000
Seq3 : ttttagaagtgattctttgatggtgccagaatacgaattacaataatgcagaaactcagttaacatgcaggaattatagtaagccaattccaattgttgc :  38000
Seq4 : ttttagaagtgattctttgatggtgccagaatacgaattacaataatgcagaaactcagttaacatgcaggaattatagtaagccaattccaattgttgc :  38000

               *     38020           *     38040           *     38060           *     38080           *     38100
Seq1 : ctgtattgtattagagtattaatatgcgcaatggtgtccttgcgtttctctgatagaatgcgagcagcgattttggcgttatcatttgacgatatttctg :  38100
Seq2 : ctgtattgtattagagtattaatatgcgcaatggtgtccttgcgtttctctgatagaatgcgagcagcgattttggcgttatcatttgacgatatttctg :  38100
Seq3 : ctgtattgtattagagtattaatatgcgcaatggtgtccttgcgtttctctgatagaatgcgagcagcgattttggcgttatcatttgacgatatttctg :  38100
Seq4 : ctgtattgtattagagtattaatatgcgcaatggtgtccttgcgtttctctgatagaatgcgagcagcgattttggcgttatcatttgacgatatttctg :  38100

               *     38120           *     38140           *     38160           *     38180           *     38200
Seq1 : gaatgacgaatcctgtttctactaactttttggtaggacaaagtgaaacaatcaagaagatagcttctcctcctatttgtggaagaaattgaactcctct :  38200
Seq2 : gaatgacgaatcctgtttctactaactttttggtaggacaaagtgaaacaatcaagaagatagcttctcctcctatttgtggaagaaattgaactcctct :  38200
Seq3 : gaatgacgaatcctgtttctactaactttttggtaggacaaagtgaaacaatcaagaagatagcttctcctcctatttgtggaagaaattgaactcctct :  38200
Seq4 : gaatgacgaatcctgtttctactaactttttggtaggacaaagtgaaacaatcaagaagatagcttctcctcctatttgtggaagaaattgaactcctct :  38200

               *     38220           *     38240           *     38260           *     38280           *     38300
Seq1 : agatgatctactgacgatagtatctccttgacagatattggaccgaattacagaagtacctggaatgtaaagccctgaaacccccctcatttttaagcag :  38300
Seq2 : agatgatctactgacgatagtatctccttgacagatattggaccgaattacagaagtacctggaatgtaaagccctgaaacccccctcatttttaagcag :  38300
Seq3 : agatgatctactgacgatagtatctccttgacagatattggaccgaattacagaagtacctggaatgtaaagccctgaaacccccctcatttttaagcag :  38300
Seq4 : agatgatctactgacgatagtatctccttgacagatattggaccgaattacagaagtacctggaatgtaaagccctgaaacccccctcatttttaagcag :  38300

               *     38320           *     38340           *     38360           *     38380           *     38400
Seq1 : attgttgccgtaaatcctgcactgtgaccaagatagagagctcctttggtgaatccatctctatgtttcagtttaaccaagaaacagtcagctggtctaa :  38400
Seq2 : attgttgccgtaaatcctgcactgtgaccaagatagagagctcctttggtgaatccatctctatgtttcagtttaaccaagaaacagtcagctggtctaa :  38400
Seq3 : attgttgccgtaaatcctgcactgtgaccaagatagagagctcctttggtgaatccatctctatgtttcagtttaaccaagaaacagtcagctggtctaa :  38400
Seq4 : attgttgccgtaaatcctgcactgtgaccaagatagagagctcctttggtgaatccatctctatgtttcagtttaaccaagaaacagtcagctggtctaa :  38400

               *     38420           *     38440           *     38460           *     38480           *     38500
Seq1 : aatttccatctctatctaatacagcatctaacttgatgtcaggaactatgaccggtttaatgttatatgtaacattgagtaaatccttaagttcataatc :  38500
Seq2 : aatttccatctctatctaatacagcatctaacttgatgtcaggaactatgaccggtttaatgttatatgtaacattgagtaaatccttaagttcataatc :  38500
Seq3 : aatttccatctctatctaatacagcatctaacttgatgtcaggaactatgaccggtttaatgttatatgtaacattgagtaaatccttaagttcataatc :  38500
Seq4 : aatttccatctctatctaatacagcatctaacttgatgtcaggaactatgaccggtttaatgttatatgtaacattgagtaaatccttaagttcataatc :  38500
```

```
                 *     38520          *     38540          *     38560          *     38580          *     38600
Seq1 : atcactgtcatcagttatgtacgatccaaacaatgtttctactggcatagtggatacgaagatgctatccatcagaatgtttccctgattagtattttct :  38600
Seq2 : atcactgtcatcagttatgtacgatccaaacaatgtttctactggcatagtggatacgaagatgctatccatcagaatgtttccctgattagtattttct :  38600
Seq3 : atcactgtcatcagttatgtacgatccaaacaatgtttctactggcatagtggatacgaagatgctatccatcagaatgtttccctgattagtattttct :  38600
Seq4 : atcactgtcatcagttatgtacgatccaaacaatgtttctactggcatagtggatacgaagatgctatccatcagaatgtttccctgattagtattttct :  38600

                 *     38620          *     38640          *     38660          *     38680          *     38700
Seq1 : atatagctattcttctttaaacgattttccaaatcagtaactatgttcatttttttaggagtaggacgcctagccagtatggaagaggattttctagatc :  38700
Seq2 : atatagctattcttctttaaacgattttccaaatcagtaactatgttcatttttttaggagtaggacgcctagccagtatggaagaggattttctagatc :  38700
Seq3 : atatagctattcttctttaaacgattttccaaatcagtaactatgttcatttttttaggagtaggacgcctagccagtatggaagaggattttctagatc :  38700
Seq4 : atatagctattcttctttaaacgattttccaaatcagtaactatgttcatttttttaggagtaggacgcctagccagtatggaagaggattttctagatc :  38700

                 *     38720          *     38740          *     38760          *     38780          *     38800
Seq1 : ctctcttcaacatctttgatctcgatggaatgcaaaaccccatagtgaaacaaccaacgataaaaataatattgtttttcacttttttataattttaccat :  38800
Seq2 : ctctcttcaacatctttgatctcgatggaatgcaaaaccccatagtgaaacaaccaacgataaaaataatattgtttttcacttttttataattttaccat :  38800
Seq3 : ctctcttcaacatctttgatctcgatggaatgcaaaaccccatagtgaaacaaccaacgataaaaataatattgtttttcacttttttataattttaccat :  38800
Seq4 : ctctcttcaacatctttgatctcgatggaatgcaaaaccccatagtgaaacaaccaacgataaaaataatattgtttttcacttttttataattttaccat :  38800

                 *     38820          *     38840          *     38860          *     38880          *     38900
Seq1 : ctgactcatggattcattaatatctttataagagctactaacgtataattctttataactgaactgagatatatacaccggatctatggtttccataatt :  38900
Seq2 : ctgactcatggattcattaatatctttataagagctactaacgtataattctttataactgaactgagatatatacaccggatctatggtttccataatt :  38900
Seq3 : ctgactcatggattcattaatatctttataagagctactaacgtataattctttataactgaactgagatatatacaccggatctatggtttccataatt :  38900
Seq4 : ctgactcatggattcattaatatctttataagagctactaacgtataattctttataactgaactgagatatatacaccggatctatggtttccataatt :  38900

                 *     38920          *     38940          *     38960          *     38980          *     39000
Seq1 : gagtaaatgaatgctcggcaataactaatggcaaatgtatagaacaacgaaattatactagagttgttaaagttaatattttctatgagctgttccaata :  39000
Seq2 : gagtaaatgaatgctcggcaataactaatggcaaatgtatagaacaacgaaattatactagagttgttaaagttaatattttctatgagctgttccaata :  39000
Seq3 : gagtaaatgaatgctcggcaataactaatggcaaatgtatagaacaacgaaattatactagagttgttaaagttaatattttctatgagctgttccaata :  39000
Seq4 : gagtaaatgaatgctcggcaataactaatggcaaatgtatagaacaacgaaattatactagagttgttaaagttaatattttctatgagctgttccaata :  39000

                 *     39020          *     39040          *     39060          *     39080          *     39100
Seq1 : aattatttgttgtaactgcgttcaagtcataaatcatcttgatactatccagtaaaccgtgtttaagttctggaatattatcatcccattgtaaagcccc :  39100
Seq2 : aattatttgttgtaactgcgttcaagtcataaatcatcttgatactatccagtaaaccgtgtttaagttctggaatattatcatcccattgtaaagcccc :  39100
Seq3 : aattatttgttgtaactgcgttcaagtcataaatcatcttgatactatccagtaaaccgtgtttaagttctggaatattatcatcccattgtaaagcccc :  39100
Seq4 : aattatttgttgtaactgcgttcaagtcataaatcatcttgatactatccagtaaaccgtgtttaagttctggaatattatcatcccattgtaaagcccc :  39100

                 *     39120          *     39140          *     39160          *     39180          *     39200
Seq1 : taattcgactatcgaatatcctgctctgatagcagtttcaatatcgacggacgtcaatactgtaataaaggtggtagtattgtcatcatcgtgataaact :  39200
Seq2 : taattcgactatcgaatatcctgctctgatagcagtttcaatatcgacggacgtcaatactgtaataaaggtggtagtattgtcatcatcgtgataaact :  39200
Seq3 : taattcgactatcgaatatcctgctctgatagcagtttcaatatcgacggacgtcaatactgtaataaaggtggtagtattgtcatcatcgtgataaact :  39200
Seq4 : taattcgactatcgaatatcctgctctgatagcagtttcaatatcgacggacgtcaatactgtaataaaggtggtagtattgtcatcatcgtgataaact :  39200
```

```
               *     39220         *     39240         *     39260         *     39280         *     39300
Seq1 : acgggaatatggtcgttagtaggtacggtgactttacacaacgcgatatataactttccttttgtaccatttttaacgtagttgggacgtcctgcagggt :  39300
Seq2 : acgggaatatggtcgttagtaggtacggtgactttacacaacgcgatatataactttccttttgtaccatttttaacgtagttgggacgtcctgcagggt :  39300
Seq3 : acgggaatatggtcgttagtaggtacggtgactttacacaacgcgatatataactttccttttgtaccatttttaacgtagttgggacgtcctgcagggt :  39300
Seq4 : acgggaatatggtcgttagtaggtacggtgactttacacaacgcgatatataactttccttttgtaccatttttaacgtagttgggacgtcctgcagggt :  39300

               *     39320         *     39340         *     39360         *     39380         *     39400
Seq1 : attgttttgaagaaatgatatcgagaacagatttgatacgatatttgttggattcctgattattcactataatataatctagacagatagatgattcgat :  39400
Seq2 : attgttttgaagaaatgatatcgagaacagatttgatacgatatttgttggattcctgattattcactataatataatctagacagatagatgattcgat :  39400
Seq3 : attgttttgaagaaatgatatcgagaacagatttgatacgatatttgttggattcctgattattcactataatataatctagacagatagatgattcgat :  39400
Seq4 : attgttttgaagaaatgatatcgagaacagatttgatacgatatttgttggattcctgattattcactataatataatctagacagatagatgattcgat :  39400

               *     39420         *     39440         *     39460         *     39480         *     39500
Seq1 : aaatagagaaggtatatcgttggtaggataatacatccccattccagtattctcggatactctattgatgacactagttaagaacatgtcttctattcta :  39500
Seq2 : aaatagagaaggtatatcgttggtaggataatacatccccattccagtattctcggatactctattgatgacactagttaagaacatgtcttctattcta :  39500
Seq3 : aaatagagaaggtatatcgttggtaggataatacatccccattccagtattctcggatactctattgatgacactagttaagaacatgtcttctattcta :  39500
Seq4 : aaatagagaaggtatatcgttggtaggataatacatccccattccagtattctcggatactctattgatgacactagttaagaacatgtcttctattcta :  39500

               *     39520         *     39540         *     39560         *     39580         *     39600
Seq1 : gaaaacgaaaacatcctacatggactcattaaaacttctaacgctcctgattgtgtctcgaatgcctcgtacaaggatttcaaggatgccatagattctt :  39600
Seq2 : gaaaacgaaaacatcctacatggactcattaaaacttctaacgctcctgattgtgtctcgaatgcctcgtacaaggatttcaaggatgccatagattctt :  39600
Seq3 : gaaaacgaaaacatcctacatggactcattaaaacttctaacgctcctgattgtgtctcgaatgcctcgtacaaggatttcaaggatgccatagattctt :  39600
Seq4 : gaaaacgaaaacatcctacatggactcattaaaacttctaacgctcctgattgtgtctcgaatgcctcgtacaaggatttcaaggatgccatagattctt :  39600

               *     39620         *     39640         *     39660         *     39680         *     39700
Seq1 : tgaccaacgatttagaattgcgtttagcatctgatttttttattaaatcgaatggtcggctctctggtttgctaccccaatgataacaatagtcttgtaa :  39700
Seq2 : tgaccaacgatttagaattgcgtttagcatctgatttttttattaaatcgaatggtcggctctctggtttgctaccccaatgataacaatagtcttgtaa :  39700
Seq3 : tgaccaacgatttagaattgcgtttagcatctgatttttttattaaatcgaatggtcggctctctggtttgctaccccaatgataacaatagtcttgtaa :  39700
Seq4 : tgaccaacgatttagaattgcgtttagcatctgatttttttattaaatcgaatggtcggctctctggtttgctaccccaatgataacaatagtcttgtaa :  39700

               *     39720         *     39740         *     39760         *     39780         *     39800
Seq1 : agataaaccgcaagaaaatttatacgcatccatccaaataaccctagcaccatcggatgatattaatgtattattatagattttccatccacaattattg :  39800
Seq2 : agataaaccgcaagaaaatttatacgcatccatccaaataaccctagcaccatcggatgatattaatgtattattatagattttccatccacaattattg :  39800
Seq3 : agataaaccgcaagaaaatttatacgcatccatccaaataaccctagcaccatcggatgatattaatgtattattatagattttccatccacaattattg :  39800
Seq4 : agataaaccgcaagaaaatttatacgcatccatccaaataaccctagcaccatcggatgatattaatgtattattatagattttccatccacaattattg :  39800

               *     39820         *     39840         *     39860         *     39880         *     39900
Seq1 : ggccagtatactgttagcaacggtatatcgaatagattactcatgtaacctactagaatgatagttcgtgtactagtcataatatctttaatccaatcta :  39900
Seq2 : ggccagtatactgttagcaacggtatatcgaatagattactcatgtaacctactagaatgatagttcgtgtactagtcataatatctttaatccaatcta :  39900
Seq3 : ggccagtatactgttagcaacggtatatcgaatagattactcatgtaacctactagaatgatagttcgtgtactagtcataatatctttaatccaatcta :  39900
Seq4 : ggccagtatactgttagcaacggtatatcgaatagattactcatgtaacctactagaatgatagttcgtgtactagtcataatatctttaatccaatcta :  39900
```

```
                 *     39920         *     39940         *     39960         *     39980         *     40000
Seq1 : agaaatttaaaattagattttttacactgttaaagttaacaaaggtattacccggatacgtggatatcatatatggcattggtccattatcagtaatagc :  40000
Seq2 : agaaatttaaaattagattttttacactgttaaagttaacaaaggtattacccggatacgtggatatcatatatggcattggtccattatcagtaatagc :  40000
Seq3 : agaaatttaaaattagattttttacactgttaaagttaacaaaggtattacccggatacgtggatatcatatatggcattggtccattatcagtaatagc :  40000
Seq4 : agaaatttaaaattagattttttacactgttaaagttaacaaaggtattacccggatacgtggatatcatatatggcattggtccattatcagtaatagc :  40000

                 *     40020         *     40040         *     40060         *     40080         *     40100
Seq1 : tccataaactgatacggcgatggtttttatatgtgtttgatctaacgaggaagaaattcgcgcccacaattcatctctagatatgtatttaatatcaaac :  40100
Seq2 : tccataaactgatacggcgatggtttttatatgtgtttgatctaacgaggaagaaattcgcgcccacaattcatctctagatatgtatttaatatcaaac :  40100
Seq3 : tccataaactgatacggcgatggtttttatatgtgtttgatctaacgaggaagaaattcgcgcccacaattcatctctagatatgtatttaatatcaaac :  40100
Seq4 : tccataaactgatacggcgatggtttttatatgtgtttgatctaacgaggaagaaattcgcgcccacaattcatctctagatatgtatttaatatcaaac :  40100

                 *     40120         *     40140         *     40160         *     40180         *     40200
Seq1 : ggtaacacatcaatttcgggacgcgtatatgtttctaaattttttaatccaaatataatgatgacctatatgccctattatcatactgtcaactatagtac :  40200
Seq2 : ggtaacacatcaatttcgggacgcgtatatgtttctaaattttttaatccaaatataatgatgacctatatgccctattatcatactgtcaactatagtac :  40200
Seq3 : ggtaacacatcaatttcgggacgcgtatatgtttctaaattttttaatccaaatataatgatgacctatatgccctattatcatactgtcaactatagtac :  40200
Seq4 : ggtaacacatcaatttcgggacgcgtatatgtttctaaattttttaatccaaatataatgatgacctatatgccctattatcatactgtcaactatagtac :  40200

                 *     40220         *     40240         *     40260         *     40280         *     40300
Seq1 : acctagagaacttacgatacatctgtttcctataatcgttaaattttacaaatctataacatgctaaaccttttgacgacagccattcattaatttctga :  40300
Seq2 : acctagagaacttacgatacatctgtttcctataatcgttaaattttacaaatctataacatgctaaaccttttgacgacagccattcattaatttctga :  40300
Seq3 : acctagagaacttacgatacatctgtttcctataatcgttaaattttacaaatctataacatgctaaaccttttgacgacagccattcattaatttctga :  40300
Seq4 : acctagagaacttacgatacatctgtttcctataatcgttaaattttacaaatctataacatgctaaaccttttgacgacagccattcattaatttctga :  40300

                 *     40320         *     40340         *     40360         *     40380         *     40400
Seq1 : tatggaatctgtattctcgataccgtattgttctaaagccagtgctatatctccctgttcgtgggaacgctttcgtataatatcgatcaacggataatct :  40400
Seq2 : tatggaatctgtattctcgataccgtattgttctaaagccagtgctatatctccctgttcgtgggaacgctttcgtataatatcgatcaacggataatct :  40400
Seq3 : tatggaatctgtattctcgataccgtattgttctaaagccagtgctatatctccctgttcgtgggaacgctttcgtataatatcgatcaacggataatct :  40400
Seq4 : tatggaatctgtattctcgataccgtattgttctaaagccagtgctatatctccctgttcgtgggaacgctttcgtataatatcgatcaacggataatct :  40400

                 *     40420         *     40440         *     40460         *     40480         *     40500
Seq1 : gaagtttttggagaataatatgactcatgatctatttcgtccataaacaatctagacataggaattggaggcgatgatcttaattttgtgcaatgagtcg :  40500
Seq2 : gaagtttttggagaataatatgactcatgatctatttcgtccataaacaatctagacataggaattggaggcgatgatcttaattttgtgcaatgagtcg :  40500
Seq3 : gaagtttttggagaataatatgactcatgatctatttcgtccataaacaatctagacataggaattggaggcgatgatcttaattttgtgcaatgagtcg :  40500
Seq4 : gaagtttttggagaataatatgactcatgatctatttcgtccataaacaatctagacataggaattggaggcgatgatcttaattttgtgcaatgagtcg :  40500

                 *     40520         *     40540         *     40560         *     40580         *     40600
Seq1 : tcaatcctataacttctaatcttgtaatattcatcatcgacataatactatctatgttatcatcgtatattagtataccacggccttcttcatttcgtgc :  40600
Seq2 : tcaatcctataacttctaatcttgtaatattcatcatcgacataatactatctatgttatcatcgtatattagtataccacggccttcttcatttcgtgc :  40600
Seq3 : tcaatcctataacttctaatcttgtaatattcatcatcgacataatactatctatgttatcatcgtatattagtataccacggccttcttcatttcgtgc :  40600
Seq4 : tcaatcctataacttctaatcttgtaatattcatcatcgacataatactatctatgttatcatcgtatattagtataccacggccttcttcatttcgtgc :  40600
```

```
                 *     40620         *     40640         *     40660         *     40680         *     40700
Seq1 : caaaataatatacagtcttaaataattacgcaatatctcaatagtttcataattgttagctgttttcatcaagatttgtaccctgtttaacatgatggcg :  40700
Seq2 : caaaataatatacagtcttaaataattacgcaatatctcaatagtttcataattgttagctgttttcatcaagatttgtaccctgtttaacatgatggcg :  40700
Seq3 : caaaataatatacagtcttaaataattacgcaatatctcaatagtttcataattgttagctgttttcatcaagatttgtaccctgtttaacatgatggcg :  40700
Seq4 : caaaataatatacagtcttaaataattacgcaatatctcaatagtttcataattgttagctgttttcatcaagatttgtaccctgtttaacatgatggcg :  40700

                 *     40720         *     40740         *     40760         *     40780         *     40800
Seq1 : ttctatacgtctctatttcttttttttaaatttttaacgatttactgtggctagatacccaatctctctcaaatattttttagcctcgcttacaagct :  40800
Seq2 : ttctatacgtctctatttcttttttttaaatttttaacgatttactgtggctagatacccaatctctctcaaatattttttagcctcgcttacaagct :  40800
Seq3 : ttctatacgtctctatttcttttttttaaatttttaacgatttactgtggctagatacccaatctctctcaaatattttttagcctcgcttacaagct :  40800
Seq4 : ttctatacgtctctatttcttttttttaaatttttaacgatttactgtggctagatacccaatctctctcaaatattttttagcctcgcttacaagct :  40800

                 *     40820         *     40840         *     40860         *     40880         *     40900
Seq1 : gtttatctatactattaaaactgacgaatccgtgattttggtaatgggttccgtcgaaatttgccgaagtgatatgaacatattcgtcgtcgactatcaa :  40900
Seq2 : gtttatctatactattaaaactgacgaatccgtgattttggtaatgggttccgtcgaaatttgccgaagtgatatgaacatattcgtcgtcgactatcaa :  40900
Seq3 : gtttatctatactattaaaactgacgaatccgtgattttggtaatgggttccgtcgaaatttgccgaagtgatatgaacatattcgtcgtcgactatcaa :  40900
Seq4 : gtttatctatactattaaaactgacgaatccgtgattttggtaatgggttccgtcgaaatttgccgaagtgatatgaacatattcgtcgtcgactatcaa :  40900

                 *     40920         *     40940         *     40960         *     40980         *     41000
Seq1 : caattttgtattattctgaatagtgaaaaccttcacagatagatcattttgaacacacaacgcgtctagacttctggcggttgccatagaatatacgtcg :  41000
Seq2 : caattttgtattattctgaatagtgaaaaccttcacagatagatcattttgaacacacaacgcgtctagacttctggcggttgccatagaatatacgtcg :  41000
Seq3 : caattttgtattattctgaatagtgaaaaccttcacagatagatcattttgaacacacaacgcgtctagacttctggcggttgccatagaatatacgtcg :  41000
Seq4 : caattttgtattattctgaatagtgaaaaccttcacagatagatcattttgaacacacaacgcgtctagacttctggcggttgccatagaatatacgtcg :  41000

                 *     41020         *     41040         *     41060         *     41080         *     41100
Seq1 : ttcttatcccaattaccaactagaagtctgatcttaactcctctattaatggctgcttctataatggagttgtaaatgtcgggccaatagtagctattac :  41100
Seq2 : ttcttatcccaattaccaactagaagtctgatcttaactcctctattaatggctgcttctataatggagttgtaaatgtcgggccaatagtagctattac :  41100
Seq3 : ttcttatcccaattaccaactagaagtctgatcttaactcctctattaatggctgcttctataatggagttgtaaatgtcgggccaatagtagctattac :  41100
Seq4 : ttcttatcccaattaccaactagaagtctgatcttaactcctctattaatggctgcttctataatggagttgtaaatgtcgggccaatagtagctattac :  41100

                 *     41120         *     41140         *     41160         *     41180         *     41200
Seq1 : cgtcgacacgtgtagtgggaactatggccaaatgttcaatatctatactagtcttagccgacttgagtttatcaataactacatcggtatctagatctct :  41200
Seq2 : cgtcgacacgtgtagtgggaactatggccaaatgttcaatatctatactagtcttagccgacttgagtttatcaataactacatcggtatctagatctct :  41200
Seq3 : cgtcgacacgtgtagtgggaactatggccaaatgttcaatatctatactagtcttagccgacttgagtttatcaataactacatcggtatctagatctct :  41200
Seq4 : cgtcgacacgtgtagtgggaactatggccaaatgttcaatatctatactagtcttagccgacttgagtttatcaataactacatcggtatctagatctct :  41200

                 *     41220         *     41240         *     41260         *     41280         *     41300
Seq1 : agaatatcccaataggtgttccggagaatcagtaaagaacactccacctataggattcttaatatgatacgcagtgctaactggcaaacaacaagccgca :  41300
Seq2 : agaatatcccaataggtgttccggagaatcagtaaagaacactccacctataggattcttaatatgatacgcagtgctaactggcaaacaacaagccgca :  41300
Seq3 : agaatatcccaataggtgttccggagaatcagtaaagaacactccacctataggattcttaatatgatacgcagtgctaactggcaaacaacaagccgca :  41300
Seq4 : agaatatcccaataggtgttccggagaatcagtaaagaacactccacctataggattcttaatatgatacgcagtgctaactggcaaacaacaagccgca :  41300
```

```
          *     41320         *     41340         *     41360         *     41380         *     41400
Seq1 : gagcataaattcaaccatgaattttttgcgctattaaaggctttaaaagtatcaaatcttctacgaagatctgtggccagcgggggataatcagaatata :  41400
Seq2 : gagcataaattcaaccatgaattttttgcgctattaaaggctttaaaagtatcaaatcttctacgaagatctgtggccagcgggggataatcagaatata :  41400
Seq3 : gagcataaattcaaccatgaattttttgcgctattaaaggctttaaaagtatcaaatcttctacgaagatctgtggccagcgggggataatcagaatata :  41400
Seq4 : gagcataaattcaaccatgaattttttgcgctattaaaggctttaaaagtatcaaatcttctacgaagatctgtggccagcgggggataatcagaatata :  41400

          *     41420         *     41440         *     41460         *     41480         *     41500
Seq1 : cacctaacgttttaatcgtatgtatagatcctccagtaaatgacgcgtttcctacataacatctttcatcatctgacacccaaaaacaaccgagtagtag :  41500
Seq2 : cacctaacgttttaatcgtatgtatagatcctccagtaaatgacgcgtttcctacataacatctttcatcatctgacacccaaaaacaaccgagtagtag :  41500
Seq3 : cacctaacgttttaatcgtatgtatagatcctccagtaaatgacgcgtttcctacataacatctttcatcatctgacacccaaaaacaaccgagtagtag :  41500
Seq4 : cacctaacgttttaatcgtatgtatagatcctccagtaaatgacgcgtttcctacataacatctttcatcatctgacacccaaaaacaaccgagtagtag :  41500

          *     41520         *     41540         *     41560         *     41580         *     41600
Seq1 : tcccacattattttttttatctatattaacggttataaaatttatatccgggcagtgactttgtagctctcccagatttcttttccctcgttcatctagc :  41600
Seq2 : tcccacattattttttttatctatattaacggttataaaatttatatccgggcagtgactttgtagctctcccagatttcttttccctcgttcatctagc :  41600
Seq3 : tcccacattattttttttatctatattaacggttataaaatttatatccgggcagtgactttgtagctctcccagatttcttttccctcgttcatctagc :  41600
Seq4 : tcccacattattttttttatctatattaacggttataaaatttatatccgggcagtgactttgtagctctcccagatttcttttccctcgttcatctagc :  41600

          *     41620         *     41640         *     41660         *     41680         *     41700
Seq1 : aaaactattatttaatccctttttcagatgcctcttttagtttatcaaaaaataagcgctcccctagtcgtactcagaggattacaacaaaaagatgcta :  41700
Seq2 : aaaactattatttaatccctttttcagatgcctcttttagtttatcaaaaaataagcgctcccctagtcgtactcagaggattacaacaaaaagatgcta :  41700
Seq3 : aaaactattatttaatccctttttcagatgcctcttttagtttatcaaaaaataagcgctcccctagtcgtactcagaggattacaacaaaaagatgcta :  41700
Seq4 : aaaactattatttaatccctttttcagatgcctcttttagtttatcaaaaaataagcgctcccctagtcgtactcagaggattacaacaaaaagatgcta :  41700

          *     41720         *     41740         *     41760         *     41780         *     41800
Seq1 : tgtatatatatttcttagctagagtgataatttcgttaaaacattcaaatgttgttaaatgatcggatctaaaatccatattttctggtagtgtttctac :  41800
Seq2 : tgtatatatatttcttagctagagtgataatttcgttaaaacattcaaatgttgttaaatgatcggatctaaaatccatattttctggtagtgtttctac :  41800
Seq3 : tgtatatatatttcttagctagagtgataatttcgttaaaacattcaaatgttgttaaatgatcggatctaaaatccatattttctggtagtgtttctac :  41800
Seq4 : tgtatatatatttcttagctagagtgataatttcgttaaaacattcaaatgttgttaaatgatcggatctaaaatccatattttctggtagtgtttctac :  41800

          *     41820         *     41840         *     41860         *     41880         *     41900
Seq1 : cagcctacatttgctcccgcaggtaccgatgcaaatggccacatttagttaacataaaaacttatacatcctgttctatcaacgattctagaatatcat :  41900
Seq2 : cagcctacatttgctcccgcaggtaccgatgcaaatggccacatttagttaacataaaaacttatacatcctgttctatcaacgattctagaatatcat :  41900
Seq3 : cagcctacatttgctcccgcaggtaccgatgcaaatggccacatttagttaacataaaaacttatacatcctgttctatcaacgattctagaatatcat :  41900
Seq4 : cagcctacatttgctcccgcaggtaccgatgcaaatggccacatttagttaacataaaaacttatacatcctgttctatcaacgattctagaatatcat :  41900

          *     41920         *     41940         *     41960         *     41980         *     42000
Seq1 : cggctatatcgctaaaattttcatcaaagtcgacatcacaacctaactcagtcaatatattaagaagttccatgatgtcatcttcgtctatttctatatc :  42000
Seq2 : cggctatatcgctaaaattttcatcaaagtcgacatcacaacctaactcagtcaatatattaagaagttccatgatgtcatcttcgtctatttctatatc :  42000
Seq3 : cggctatatcgctaaaattttcatcaaagtcgacatcacaacctaactcagtcaatatattaagaagttccatgatgtcatcttcgtctatttctatatc :  42000
Seq4 : cggctatatcgctaaaattttcatcaaagtcgacatcacaacctaactcagtcaatatattaagaagttccatgatgtcatcttcgtctatttctatatc :  42000
```

```
                 *     42020         *     42040         *     42060         *     42080         *     42100
Seq1 : cgtatccattgtagattgttgaccgattatcgagtttaaatcattactaatactcaatccttcagaatacaatctgtgtttcattgtaaatttataggcg :  42100
Seq2 : cgtatccattgtagattgttgaccgattatcgagtttaaatcattactaatactcaatccttcagaatacaatctgtgtttcattgtaaatttataggcg :  42100
Seq3 : cgtatccattgtagattgttgaccgattatcgagtttaaatcattactaatactcaatccttcagaatacaatctgtgtttcattgtaaatttataggcg :  42100
Seq4 : cgtatccattgtagattgttgaccgattatcgagtttaaatcattactaatactcaatccttcagaatacaatctgtgtttcattgtaaatttataggcg :  42100

                 *     42120         *     42140         *     42160         *     42180         *     42200
Seq1 : gtgtatttaagttggtagattttcaattatgtatcaatatagcaacagtagttcttgctcctccttgattctagcatcctcttcattatttcttctacg :  42200
Seq2 : gtgtatttaagttggtagattttcaattatgtatcaatatagcaacagtagttcttgctcctccttgattctagcatcctcttcattatttcttctacg :  42200
Seq3 : gtgtatttaagttggtagattttcaattatgtatcaatatagcaacagtagttcttgctcctccttgattctagcatcctcttcattatttcttctacg :  42200
Seq4 : gtgtatttaagttggtagattttcaattatgtatcaatatagcaacagtagttcttgctcctccttgattctagcatcctcttcattatttcttctacg :  42200

                 *     42220         *     42240         *     42260         *     42280         *     42300
Seq1 : tacataagcatgtccaatacgttagacaacacaccgacgatggcggccgccacagacacgaatatgactagaccgatgaccatttaaaacccctctcta :  42300
Seq2 : tacataagcatgtccaatacgttagacaacacaccgacgatggcggccgccacagacacgaatatgactagaccgatgaccatttaaaacccctctcta :  42300
Seq3 : tacataagcatgtccaatacgttagacaacacaccgacgatggcggccgccacagacacgaatatgactagaccgatgaccatttaaaacccctctcta :  42300
Seq4 : tacataagcatgtccaatacgttagacaacacaccgacgatggcggccgccacagacacgaatatgactagaccgatgaccatttaaaacccctctcta :  42300

                 *     42320         *     42340         *     42360         *     42380         *     42400
Seq1 : gctttcacttaaactgtatcgattattcttttagaacatgtataatataaaaacattattctatttcgaatttaggcttccaaaaatttttcatccgtaa :  42400
Seq2 : gctttcacttaaactgtatcgattattcttttagaacatgtataatataaaaacattattctatttcgaatttaggcttccaaaaatttttcatccgtaa :  42400
Seq3 : gctttcacttaaactgtatcgattattcttttagaacatgtataatataaaaacattattctatttcgaatttaggcttccaaaaatttttcatccgtaa :  42400
Seq4 : gctttcacttaaactgtatcgattattcttttagaacatgtataatataaaaacattattctatttcgaatttaggcttccaaaaatttttcatccgtaa :  42400

                 *     42420         *     42440         *     42460         *     42480         *     42500
Seq1 : accgataataatatatatatagacttgttaatagtcggaataaatagattaatgcttaaactatcatcatctccacgattagagatacaatatttacatttt :  42500
Seq2 : accgataataatatatatatagacttgttaatagtcggaataaatagattaatgcttaaactatcatcatctccacgattagagatacaatatttacatttt :  42500
Seq3 : accgataataatatatatatagacttgttaatagtcggaataaatagattaatgcttaaactatcatcatctccacgattagagatacaatatttacatttt :  42500
Seq4 : accgataataatatatatatagacttgttaatagtcggaataaatagattaatgcttaaactatcatcatctccacgattagagatacaatatttacatttt :  42500

                 *     42520         *     42540         *     42560         *     42580         *     42600
Seq1 : ttttgctgtttcgaaactttatcaatacacgttaatacaaacccaggaaggagatattgaaactgaggctgttgaaaatgaaacggtgaatacaataatt :  42600
Seq2 : ttttgctgtttcgaaactttatcaatacacgttaatacaaacccaggaaggagatattgaaactgaggctgttgaaaatgaaacggtgaatacaataatt :  42600
Seq3 : ttttgctgtttcgaaactttatcaatacacgttaatacaaacccaggaaggagatattgaaactgaggctgttgaaaatgaaacggtgaatacaataatt :  42600
Seq4 : ttttgctgtttcgaaactttatcaatacacgttaatacaaacccaggaaggagatattgaaactgaggctgttgaaaatgaaacggtgaatacaataatt :  42600

                 *     42620         *     42640         *     42660         *     42680         *     42700
Seq1 : cagataatgtaaaatcatgattccgtattctgatgatattagaactgctaatggatgtcgatggtatgtatctaggagtatctattttaacaaagcatcg :  42700
Seq2 : cagataatgtaaaatcatgattccgtattctgatgatattagaactgctaatggatgtcgatggtatgtatctaggagtatctattttaacaaagcatcg :  42700
Seq3 : cagataatgtaaaatcatgattccgtattctgatgatattagaactgctaatggatgtcgatggtatgtatctaggagtatctattttaacaaagcatcg :  42700
Seq4 : cagataatgtaaaatcatgattccgtattctgatgatattagaactgctaatggatgtcgatggtatgtatctaggagtatctattttaacaaagcatcg :  42700
```

```
                *     42720         *     42740         *     42760         *     42780         *     42800
Seq1 : atttgctaatatacaattatccttttgattaattgttattttattcatattcttaaaaggtttcatatttatcaattcttctacattaaaaatttccatt :  42800
Seq2 : atttgctaatatacaattatccttttgattaattgttattttattcatattcttaaaaggtttcatatttatcaattcttctacattaaaaatttccatt :  42800
Seq3 : atttgctaatatacaattatccttttgattaattgttattttattcatattcttaaaaggtttcatatttatcaattcttctacattaaaaatttccatt :  42800
Seq4 : atttgctaatatacaattatccttttgattaattgttattttattcatattcttaaaaggtttcatatttatcaattcttctacattaaaaatttccatt :  42800

                *     42820         *     42840         *     42860         *     42880         *     42900
Seq1 : tttaatttatgtagccccgcaatactcctcattacgtttcatttttgtctataatatccattttgttcatctcggtacatagattatccaattgagaag :  42900
Seq2 : tttaatttatgtagccccgcaatactcctcattacgtttcatttttgtctataatatccattttgttcatctcggtacatagattatccaattgagaag :  42900
Seq3 : tttaatttatgtagccccgcaatactcctcattacgtttcatttttgtctataatatccattttgttcatctcggtacatagattatccaattgagaag :  42900
Seq4 : tttaatttatgtagccccgcaatactcctcattacgtttcatttttgtctataatatccattttgttcatctcggtacatagattatccaattgagaag :  42900

                *     42920         *     42940         *     42960         *     42980         *     43000
Seq1 : cgcatttagtagttttgtacattttaagtttattgacgaatcgtcgaaaactagttatagttaacattttattatttgataccctgatattaatacccct :  43000
Seq2 : cgcatttagtagttttgtacattttaagtttattgacgaatcgtcgaaaactagttatagttaacattttattatttgataccctgatattaatacccct :  43000
Seq3 : cgcatttagtagttttgtacattttaagtttattgacgaatcgtcgaaaactagttatagttaacattttattatttgataccctgatattaatacccct :  43000
Seq4 : cgcatttagtagttttgtacattttaagtttattgacgaatcgtcgaaaactagttatagttaacattttattatttgataccctgatattaatacccct :  43000

                *     43020         *     43040         *     43060         *     43080         *     43100
Seq1 : gccgttactattatttataactgatgtaatccacgtaacattagaattaattatcgatagtaatgcatcgacgcttccaaaattgtctattataaactca :  43100
Seq2 : gccgttactattatttataactgatgtaatccacgtaacattagaattaattatcgatagtaatgcatcgacgcttccaaaattgtctattataaactca :  43100
Seq3 : gccgttactattatttataactgatgtaatccacgtaacattagaattaattatcgatagtaatgcatcgacgcttccaaaattgtctattataaactca :  43100
Seq4 : gccgttactattatttataactgatgtaatccacgtaacattagaattaattatcgatagtaatgcatcgacgcttccaaaattgtctattataaactca :  43100

                *     43120         *     43140         *     43160         *     43180         *     43200
Seq1 : ccgataatttttttattgcatgttttcatattcattaggattatcaaatctttaatcttactacgattgtatgcgttgatattgcaagacgtcattctaa :  43200
Seq2 : ccgataatttttttattgcatgttttcatattcattaggattatcaaatctttaatcttactacgattgtatgcgttgatattgcaagacgtcattctaa :  43200
Seq3 : ccgataatttttttattgcatgttttcatattcattaggattatcaaatctttaatcttactacgattgtatgcgttgatattgcaagacgtcattctaa :  43200
Seq4 : ccgataatttttttattgcatgttttcatattcattaggattatcaaatctttaatcttactacgattgtatgcgttgatattgcaagacgtcattctaa :  43200

                *     43220         *     43240         *     43260         *     43280         *     43300
Seq1 : aagacggaggatctccatcaaatgccagacaatcacgtacaaagtacatggaaataggttttgttctattgcgcatcatagatttatatagaacacccgt :  43300
Seq2 : aagacggaggatctccatcaaatgccagacaatcacgtacaaagtacatggaaataggttttgttctattgcgcatcatagatttatatagaacacccgt :  43300
Seq3 : aagacggaggatctccatcaaatgccagacaatcacgtacaaagtacatggaaataggttttgttctattgcgcatcatagatttatatagaacacccgt :  43300
Seq4 : aagacggaggatctccatcaaatgccagacaatcacgtacaaagtacatggaaataggttttgttctattgcgcatcatagatttatatagaacacccgt :  43300

                *     43320         *     43340         *     43360         *     43380         *     43400
Seq1 : agaaatactaatttgttttactctataaaatactaatgcatctatttcatcgttttgtataacgtctttccaagtgtcaaattccaaattttttttcattg :  43400
Seq2 : agaaatactaatttgttttactctataaaatactaatgcatctatttcatcgttttgtataacgtctttccaagtgtcaaattccaaattttttttcattg :  43400
Seq3 : agaaatactaatttgttttactctataaaatactaatgcatctatttcatcgttttgtataacgtctttccaagtgtcaaattccaaattttttttcattg :  43400
Seq4 : agaaatactaatttgttttactctataaaatactaatgcatctatttcatcgttttgtataacgtctttccaagtgtcaaattccaaattttttttcattg :  43400
```

```
              *     43420         *     43440         *     43460         *     43480         *     43500
Seq1 : atagtaccaaattcttctatctctttaactacttgcatagataggtaattacagtgatgcctacatgccgtttttgaaactgaatagatgcgtctagaa :  43500
Seq2 : atagtaccaaattcttctatctctttaactacttgcatagataggtaattacagtgatgcctacatgccgtttttgaaactgaatagatgcgtctagaa :  43500
Seq3 : atagtaccaaattcttctatctctttaactacttgcatagataggtaattacagtgatgcctacatgccgtttttgaaactgaatagatgcgtctagaa :  43500
Seq4 : atagtaccaaattcttctatctctttaactacttgcatagataggtaattacagtgatgcctacatgccgtttttgaaactgaatagatgcgtctagaa :  43500

              *     43520         *     43540         *     43560         *     43580         *     43600
Seq1 : gcgatgctacgctagtcacaatcaccactttcatatttagaatatatgtatgtaaaaatatagtagaatttcattttgtttttttctatgctataaatga :  43600
Seq2 : gcgatgctacgctagtcacaatcaccactttcatatttagaatatatgtatgtaaaaatatagtagaatttcattttgtttttttctatgctataaatga :  43600
Seq3 : gcgatgctacgctagtcacaatcaccactttcatatttagaatatatgtatgtaaaaatatagtagaatttcattttgtttttttctatgctataaatga :  43600
Seq4 : gcgatgctacgctagtcacaatcaccactttcatatttagaatatatgtatgtaaaaatatagtagaatttcattttgtttttttctatgctataaatga :  43600

              *     43620         *     43640         *     43660         *     43680         *     43700
Seq1 : attctcatttgcatctgctcatactccgttttatatcaataccaaagaaggaagatatctggttctaaaagccgttaaagtatgcgatgttagaactgt :  43700
Seq2 : attctcatttgcatctgctcatactccgttttatatcaataccaaagaaggaagatatctggttctaaaagccgttaaagtatgcgatgttagaactgt :  43700
Seq3 : attctcatttgcatctgctcatactccgttttatatcaataccaaagaaggaagatatctggttctaaaagccgttaaagtatgcgatgttagaactgt :  43700
Seq4 : attctcatttgcatctgctcatactccgttttatatcaataccaaagaaggaagatatctggttctaaaagccgttaaagtatgcgatgttagaactgt :  43700

              *     43720         *     43740         *     43760         *     43780         *     43800
Seq1 : agaatgcgaaggaagtaaagcttcctgcgtactcaaagtagataaaccctcatcgcccgcgtgtgagagaagaccttcgtccccgtccagatgcgagaga :  43800
Seq2 : agaatgcgaaggaagtaaagcttcctgcgtactcaaagtagataaaccctcatcgcccgcgtgtgagagaagaccttcgtccccgtccagatgcgagaga :  43800
Seq3 : agaatgcgaaggaagtaaagcttcctgcgtactcaaagtagataaaccctcatcgcccgcgtgtgagagaagaccttcgtccccgtccagatgcgagaga :  43800
Seq4 : agaatgcgaaggaagtaaagcttcctgcgtactcaaagtagataaaccctcatcgcccgcgtgtgagagaagaccttcgtccccgtccagatgcgagaga :  43800

              *     43820         *     43840         *     43860         *     43880         *     43900
Seq1 : atgaataaccctggaaaacaagttccgtttatgaggacggacatgctacaaaatatgttcgcggctaatcgcgacaacgtggcgtcgagacttttgaact :  43900
Seq2 : atgaataaccctggaaaacaagttccgtttatgaggacggacatgctacaaaatatgttcgcggctaatcgcgacaacgtggcgtcgagacttttgaact :  43900
Seq3 : atgaataaccctggaaaacaagttccgtttatgaggacggacatgctacaaaatatgttcgcggctaatcgcgacaacgtggcgtcgagacttttgaact :  43900
Seq4 : atgaataaccctggaaaacaagttccgtttatgaggacggacatgctacaaaatatgttcgcggctaatcgcgacaacgtggcgtcgagacttttgaact :  43900

              *     43920         *     43940         *     43960         *     43980         *     44000
Seq1 : aaaatacaattatatcctttttcgatattaataaatccgtgtcgtccaggtttttatctctttcagtatgtgaatagataggtattttatctctattcat :  44000
Seq2 : aaaatacaattatatcctttttcgatattaataaatccgtgtcgtccaggtttttatctctttcagtatgtgaatagataggtattttatctctattcat :  44000
Seq3 : aaaatacaattatatcctttttcgatattaataaatccgtgtcgtccaggtttttatctctttcagtatgtgaatagataggtattttatctctattcat :  44000
Seq4 : aaaatacaattatatcctttttcgatattaataaatccgtgtcgtccaggtttttatctctttcagtatgtgaatagataggtattttatctctattcat :  44000

              *     44020         *     44040         *     44060         *     44080         *     44100
Seq1 : catcgaatttaagagatccgataaacattgtttgtattctccagatgtcagcatctgatacaacaatatatgtgcacataaacctctggcacttatttca :  44100
Seq2 : catcgaatttaagagatccgataaacattgtttgtattctccagatgtcagcatctgatacaacaatatatgtgcacataaacctctggcacttatttca :  44100
Seq3 : catcgaatttaagagatccgataaacattgtttgtattctccagatgtcagcatctgatacaacaatatatgtgcacataaacctctggcacttatttca :  44100
Seq4 : catcgaatttaagagatccgataaacattgtttgtattctccagatgtcagcatctgatacaacaatatatgtgcacataaacctctggcacttatttca :  44100
```

```
               *     44120         *     44140         *     44160         *     44180         *     44200
Seq1 : tgtaccttccccttatcactaaggagaatagtatttgagaaatatgtatacatgatattatcatgaattagatatacagaatttgtaacactctcgaaat :  44200
Seq2 : tgtaccttccccttatcactaaggagaatagtatttgagaaatatgtatacatgatattatcatgaattagatatacagaatttgtaacactctcgaaat :  44200
Seq3 : tgtaccttccccttatcactaaggagaatagtatttgagaaatatgtatacatgatattatcatgaattagatatacagaatttgtaacactctcgaaat :  44200
Seq4 : tgtaccttccccttatcactaaggagaatagtatttgagaaatatgtatacatgatattatcatgaattagatatacagaatttgtaacactctcgaaat :  44200

               *     44220         *     44240         *     44260         *     44280         *     44300
Seq1 : cacacgatgtgtcggcgttaagatctaatatatcactcgataacacattttcatctagatacactagacatttttttaaagctaaaatagtctttagtagt :  44300
Seq2 : cacacgatgtgtcggcgttaagatctaatatatcactcgataacacattttcatctagatacactagacatttttttaaagctaaaatagtctttagtagt :  44300
Seq3 : cacacgatgtgtcggcgttaagatctaatatatcactcgataacacattttcatctagatacactagacatttttttaaagctaaaatagtctttagtagt :  44300
Seq4 : cacacgatgtgtcggcgttaagatctaatatatcactcgataacacattttcatctagatacactagacatttttttaaagctaaaatagtctttagtagt :  44300

               *     44320         *     44340         *     44360         *     44380         *     44400
Seq1 : gacagtaactatgcgattattttcatcgatgatacatttcatcggcatattattacgcttaccatcaaagactataccatgtgtatatctaacgtattct :  44400
Seq2 : gacagtaactatgcgattattttcatcgatgatacatttcatcggcatattattacgcttaccatcaaagactataccatgtgtatatctaacgtattct :  44400
Seq3 : gacagtaactatgcgattattttcatcgatgatacatttcatcggcatattattacgcttaccatcaaagactataccatgtgtatatctaacgtattct :  44400
Seq4 : gacagtaactatgcgattattttcatcgatgatacatttcatcggcatattattacgcttaccatcaaagactataccatgtgtatatctaacgtattct :  44400

               *     44420         *     44440         *     44460         *     44480         *     44500
Seq1 : agcatggttgccatacgcgcattaaacttttcaggatctttggatagatcttccaatctatctatttgagaaaacatttttatcatgttcaatagttgaa :  44500
Seq2 : agcatggttgccatacgcgcattaaacttttcaggatctttggatagatcttccaatctatctatttgagaaaacatttttatcatgttcaatagttgaa :  44500
Seq3 : agcatggttgccatacgcgcattaaacttttcaggatctttggatagatcttccaatctatctatttgagaaaacatttttatcatgttcaatagttgaa :  44500
Seq4 : agcatggttgccatacgcgcattaaacttttcaggatctttggatagatcttccaatctatctatttgagaaaacatttttatcatgttcaatagttgaa :  44500

               *     44520         *     44540         *     44560         *     44580         *     44600
Seq1 : acgtcggatccactatatagatattatctataaagattttaggaactacgttcatggtatcctggcgaatattaaaactatcaatgatatgattatcgtt :  44600
Seq2 : acgtcggatccactatatagatattatctataaagattttaggaactacgttcatggtatcctggcgaatattaaaactatcaatgatatgattatcgtt :  44600
Seq3 : acgtcggatccactatatagatattatctataaagattttaggaactacgttcatggtatcctggcgaatattaaaactatcaatgatatgattatcgtt :  44600
Seq4 : acgtcggatccactatatagatattatctataaagattttaggaactacgttcatggtatcctggcgaatattaaaactatcaatgatatgattatcgtt :  44600

               *     44620         *     44640         *     44660         *     44680         *     44700
Seq1 : ttcatcttttatcaccatatagtttctaagatatgggattttacttaatataatattatttcccgtgataaattttattagaaaggccaaatctataaga :  44700
Seq2 : ttcatcttttatcaccatatagtttctaagatatgggattttacttaatataatattatttcccgtgataaattttattagaaaggccaaatctataaga :  44700
Seq3 : ttcatcttttatcaccatatagtttctaagatatgggattttacttaatataatattatttcccgtgataaattttattagaaaggccaaatctataaga :  44700
Seq4 : ttcatcttttatcaccatatagtttctaagatatgggattttacttaatataatattatttcccgtgataaattttattagaaaggccaaatctataaga :  44700

               *     44720         *     44740         *     44760         *     44780         *     44800
Seq1 : aaagttctagaattagtctgaagaatatctatatcgccgtaccgtatatttggattaattagatatagagaatatgatccgtaacatatacaacttttat :  44800
Seq2 : aaagttctagaattagtctgaagaatatctatatcgccgtaccgtatatttggattaattagatatagagaatatgatccgtaacatatacaacttttat :  44800
Seq3 : aaagttctagaattagtctgaagaatatctatatcgccgtaccgtatatttggattaattagatatagagaatatgatccgtaacatatacaacttttat :  44800
Seq4 : aaagttctagaattagtctgaagaatatctatatcgccgtaccgtatatttggattaattagatatagagaatatgatccgtaacatatacaacttttat :  44800
```

```
              *     44820           *     44840           *     44860           *     44880           *     44900
Seq1 : tatggcgtctaagatattcttccatcaacttattaacattttttgactagggaagatacattatgacgtcccattacttttgccttgtctattactgcgac :  44900
Seq2 : tatggcgtctaagatattcttccatcaacttattaacattttttgactagggaagatacattatgacgtcccattacttttgccttgtctattactgcgac :  44900
Seq3 : tatggcgtctaagatattcttccatcaacttattaacattttttgactagggaagatacattatgacgtcccattacttttgccttgtctattactgcgac :  44900
Seq4 : tatggcgtctaagatattcttccatcaacttattaacattttttgactagggaagatacattatgacgtcccattacttttgccttgtctattactgcgac :  44900

              *     44920           *     44940           *     44960           *     44980           *     45000
Seq1 : gttcatagaatttagcatatctcttgccaattcttccattgatgttacattataagaaattttagatgaaattacatttggagctttaatagtaagaact :  45000
Seq2 : gttcatagaatttagcatatctcttgccaattcttccattgatgttacattataagaaattttagatgaaattacatttggagctttaatagtaagaact :  45000
Seq3 : gttcatagaatttagcatatctcttgccaattcttccattgatgttacattataagaaattttagatgaaattacatttggagctttaatagtaagaact :  45000
Seq4 : gttcatagaatttagcatatctcttgccaattcttccattgatgttacattataagaaattttagatgaaattacatttggagctttaatagtaagaact :  45000

              *     45020           *     45040           *     45060           *     45080           *     45100
Seq1 : cctaatatgtccgtgtatgtggtcactaatacagattgtagttctataatcgtaaataatttacctatattatatgtttgagtctgtttagaaaagtagc :  45100
Seq2 : cctaatatgtccgtgtatgtggtcactaatacagattgtagttctataatcgtaaataatttacctatattatatgtttgagtctgtttagaaaagtagc :  45100
Seq3 : cctaatatgtccgtgtatgtggtcactaatacagattgtagttctataatcgtaaataatttacctatattatatgtttgagtctgtttagaaaagtagc :  45100
Seq4 : cctaatatgtccgtgtatgtggtcactaatacagattgtagttctataatcgtaaataatttacctatattatatgtttgagtctgtttagaaaagtagc :  45100

              *     45120           *     45140           *     45160           *     45180           *     45200
Seq1 : taagtatacgatcttttatttctgatgcagatgtattaacatcggaaaaaaatctttttttattctttttttactaaagatacaaatatgtctttgttaaa :  45200
Seq2 : taagtatacgatcttttatttctgatgcagatgtattaacatcggaaaaaaatctttttttattctttttttactaaagatacaaatatgtctttgttaaa :  45200
Seq3 : taagtatacgatcttttatttctgatgcagatgtattaacatcggaaaaaaatctttttttattctttttttactaaagatacaaatatgtctttgttaaa :  45200
Seq4 : taagtatacgatcttttatttctgatgcagatgtattaacatcggaaaaaaatctttttttattctttttttactaaagatacaaatatgtctttgttaaa :  45200

              *     45220           *     45240           *     45260           *     45280           *     45300
Seq1 : aacagttatttttttgaatatttctagcttgtaattttaacatatgatattcgttcacactaggtactctgcctaaataggtttctataatctttaatgta :  45300
Seq2 : aacagttatttttttgaatatttctagcttgtaattttaacatatgatattcgttcacactaggtactctgcctaaataggtttctataatctttaatgta :  45300
Seq3 : aacagttatttttttgaatatttctagcttgtaattttaacatatgatattcgttcacactaggtactctgcctaaataggtttctataatctttaatgta :  45300
Seq4 : aacagttatttttttgaatatttctagcttgtaattttaacatatgatattcgttcacactaggtactctgcctaaataggtttctataatctttaatgta :  45300

              *     45320           *     45340           *     45360           *     45380           *     45400
Seq1 : atattaggaagagtattctgatcaggattcctattcattttgaggatttaaaactctgattattgtctaatatggtctctacgcaaactttttcacagag :  45400
Seq2 : atattaggaagagtattctgatcaggattcctattcattttgaggatttaaaactctgattattgtctaatatggtctctacgcaaactttttcacagag :  45400
Seq3 : atattaggaagagtattctgatcaggattcctattcattttgaggatttaaaactctgattattgtctaatatggtctctacgcaaactttttcacagag :  45400
Seq4 : atattaggaagagtattctgatcaggattcctattcattttgaggatttaaaactctgattattgtctaatatggtctctacgcaaactttttcacagag :  45400

              *     45420           *     45440           *     45460           *     45480           *     45500
Seq1 : cgatagagtttttgataactcgtttttcttaagaaatataaaactactgtctccagagctcgctctatctttttattttatttaattcgatacaaactcct :  45500
Seq2 : cgatagagtttttgataactcgtttttcttaagaaatataaaactactgtctccagagctcgctctatctttttattttatttaattcgatacaaactcct :  45500
Seq3 : cgatagagtttttgataactcgtttttcttaagaaatataaaactactgtctccagagctcgctctatctttttattttatttaattcgatacaaactcct :  45500
Seq4 : cgatagagtttttgataactcgtttttcttaagaaatataaaactactgtctccagagctcgctctatctttttattttatttaattcgatacaaactcct :  45500
```

```
                *     45520         *     45540         *     45560         *     45580         *     45600
Seq1 : gatactggttcagaaagtaattcattaattttcagtcctttatagaagatatttaatatagataatacaaaattttcagttcttgatatcgatctgattg :  45600
Seq2 : gatactggttcagaaagtaattcattaattttcagtcctttatagaagatatttaatatagataatacaaaattttcagttcttgatatcgatctgattg :  45600
Seq3 : gatactggttcagaaagtaattcattaattttcagtcctttatagaagatatttaatatagataatacaaaattttcagttcttgatatcgatctgattg :  45600
Seq4 : gatactggttcagaaagtaattcattaattttcagtcctttatagaagatatttaatatagataatacaaaattttcagttcttgatatcgatctgattg :  45600

                *     45620         *     45640         *     45660         *     45680         *     45700
Seq1 : atcctagaactagatatattaataacgtgctcattaggcagtttatggcagcttgataattagatatagtatattccagttcatatttattagataccgc :  45700
Seq2 : atcctagaactagatatattaataacgtgctcattaggcagtttatggcagcttgataattagatatagtatattccagttcatatttattagataccgc :  45700
Seq3 : atcctagaactagatatattaataacgtgctcattaggcagtttatggcagcttgataattagatatagtatattccagttcatatttattagataccgc :  45700
Seq4 : atcctagaactagatatattaataacgtgctcattaggcagtttatggcagcttgataattagatatagtatattccagttcatatttattagataccgc :  45700

                *     45720         *     45740         *     45760         *     45780         *     45800
Seq1 : attgcccagattttgatattctatgaattcctctgaaaataaatccaaaataactaaacattctatttttgtggattagtgtactctcttccctctatc :  45800
Seq2 : attgcccagattttgatattctatgaattcctctgaaaataaatccaaaataactaaacattctatttttgtggattagtgtactctcttccctctatc :  45800
Seq3 : attgcccagattttgatattctatgaattcctctgaaaataaatccaaaataactaaacattctatttttgtggattagtgtactctcttccctctatc :  45800
Seq4 : attgcccagattttgatattctatgaattcctctgaaaataaatccaaaataactaaacattctatttttgtggattagtgtactctcttccctctatc :  45800

                *     45820         *     45840         *     45860         *     45880         *     45900
Seq1 : atgttcactactggtgtccacgatgataaatatctagagggaatataatatagtccataggatgccaatctagcaatgtcgaataactgtaatttgattc :  45900
Seq2 : atgttcactactggtgtccacgatgataaatatctagagggaatataatatagtccataggatgccaatctagcaatgtcgaataactgtaatttgattc :  45900
Seq3 : atgttcactactggtgtccacgatgataaatatctagagggaatataatatagtccataggatgccaatctagcaatgtcgaataactgtaatttgattc :  45900
Seq4 : atgttcactactggtgtccacgatgataaatatctagagggaatataatatagtccataggatgccaatctagcaatgtcgaataactgtaatttgattc :  45900

                *     45920         *     45940         *     45960         *     45980         *     46000
Seq1 : ttcgttcttcattatgaattgattcttgaggtataaacctaacacaaattatattattagacttttcgtatgtaatgtctttcatgttataagtttttaa :  46000
Seq2 : ttcgttcttcattatgaattgattcttgaggtataaacctaacacaaattatattattagacttttcgtatgtaatgtctttcatgttataagtttttaa :  46000
Seq3 : ttcgttcttcattatgaattgattcttgaggtataaacctaacacaaattatattattagacttttcgtatgtaatgtctttcatgttataagtttttaa :  46000
Seq4 : ttcgttcttcattatgaattgattcttgaggtataaacctaacacaaattatattattagacttttcgtatgtaatgtctttcatgttataagtttttaa :  46000

                *     46020         *     46040         *     46060         *     46080         *     46100
Seq1 : tcctggaatagaatctattttaatgaggcttttaaacgcagagttctccaacgagtcaaagcataatactctgttgtttttcttatatacgatgttacga :  46100
Seq2 : tcctggaatagaatctattttaatgaggcttttaaacgcagagttctccaacgagtcaaagcataatactctgttgtttttcttatatacgatgttacga :  46100
Seq3 : tcctggaatagaatctattttaatgaggcttttaaacgcagagttctccaacgagtcaaagcataatactctgttgtttttcttatatacgatgttacga :  46100
Seq4 : tcctggaatagaatctattttaatgaggcttttaaacgcagagttctccaacgagtcaaagcataatactctgttgtttttcttatatacgatgttacga :  46100

                *     46120         *     46140         *     46160         *     46180         *     46200
Seq1 : ttttcttctttgaatggaataggtttttgaattagtttataattacaacataatagataaggaagtgtgcaaatagtacgcggaaaaaacataatagctc :  46200
Seq2 : ttttcttctttgaatggaataggtttttgaattagtttataattacaacataatagataaggaagtgtgcaaatagtacgcggaaaaaacataatagctc :  46200
Seq3 : ttttcttctttgaatggaataggtttttgaattagtttataattacaacataatagataaggaagtgtgcaaatagtacgcggaaaaaacataatagctc :  46200
Seq4 : ttttcttctttgaatggaataggtttttgaattagtttataattacaacataatagataaggaagtgtgcaaatagtacgcggaaaaaacataatagctc :  46200
```

```
                 *     46220         *     46240         *     46260         *     46280         *     46300
Seq1 : ccctgttttcatccatggttttaagtaaatgatcactggcttctttagtcaatggatattcgaacattaaccgtttcatcatcattggacagaatccata :  46300
Seq2 : ccctgttttcatccatggttttaagtaaatgatcactggcttctttagtcaatggatattcgaacattaaccgtttcatcatcattggacagaatccata :  46300
Seq3 : ccctgttttcatccatggttttaagtaaatgatcactggcttctttagtcaatggatattcgaacattaaccgtttcatcatcattggacagaatccata :  46300
Seq4 : ccctgttttcatccatggttttaagtaaatgatcactggcttctttagtcaatggatattcgaacattaaccgtttcatcatcattggacagaatccata :  46300

                 *     46320         *     46340         *     46360         *     46380         *     46400
Seq1 : tttcttaatgtaaagagtgatcaaatcattgtgtttattgtaccatcttgttgtaaatgtgtattcggttatcggatctgctcctttttctattaaagta :  46400
Seq2 : tttcttaatgtaaagagtgatcaaatcattgtgtttattgtaccatcttgttgtaaatgtgtattcggttatcggatctgctcctttttctattaaagta :  46400
Seq3 : tttcttaatgtaaagagtgatcaaatcattgtgtttattgtaccatcttgttgtaaatgtgtattcggttatcggatctgctcctttttctattaaagta :  46400
Seq4 : tttcttaatgtaaagagtgatcaaatcattgtgtttattgtaccatcttgttgtaaatgtgtattcggttatcggatctgctcctttttctattaaagta :  46400

                 *     46420         *     46440         *     46460         *     46480         *     46500
Seq1 : tcgatgtcgatctcgtctaagaattcaactatatcgacatatttcatttgtatacacataaccattactaacgtagaatgtataggaagagatgtaacgg :  46500
Seq2 : tcgatgtcgatctcgtctaagaattcaactatatcgacatatttcatttgtatacacataaccattactaacgtagaatgtataggaagagatgtaacgg :  46500
Seq3 : tcgatgtcgatctcgtctaagaattcaactatatcgacatatttcatttgtatacacataaccattactaacgtagaatgtataggaagagatgtaacgg :  46500
Seq4 : tcgatgtcgatctcgtctaagaattcaactatatcgacatatttcatttgtatacacataaccattactaacgtagaatgtataggaagagatgtaacgg :  46500

                 *     46520         *     46540         *     46560         *     46580         *     46600
Seq1 : gaacagggtttgttgattcgcaaactattctaatacataattcttctgttaatacgtcttgcacgtaatctattatagatgccaagatatctatataatt :  46600
Seq2 : gaacagggtttgttgattcgcaaactattctaatacataattcttctgttaatacgtcttgcacgtaatctattatagatgccaagatatctatataatt :  46600
Seq3 : gaacagggtttgttgattcgcaaactattctaatacataattcttctgttaatacgtcttgcacgtaatctattatagatgccaagatatctatataatt :  46600
Seq4 : gaacagggtttgttgattcgcaaactattctaatacataattcttctgttaatacgtcttgcacgtaatctattatagatgccaagatatctatataatt :  46600

                 *     46620         *     46640         *     46660         *     46680         *     46700
Seq1 : attttgtaagatgatgttaactatgtgatctatataagtagtgtaataattcatgtatttcgatatatgttccaactctgtctttgtgatgtctagtttc :  46700
Seq2 : attttgtaagatgatgttaactatgtgatctatataagtagtgtaataattcatgtatttcgatatatgttccaactctgtctttgtgatgtctagtttc :  46700
Seq3 : attttgtaagatgatgttaactatgtgatctatataagtagtgtaataattcatgtatttcgatatatgttccaactctgtctttgtgatgtctagtttc :  46700
Seq4 : attttgtaagatgatgttaactatgtgatctatataagtagtgtaataattcatgtatttcgatatatgttccaactctgtctttgtgatgtctagtttc :  46700

                 *     46720         *     46740         *     46760         *     46780         *     46800
Seq1 : gtaatatctatagcatcctcaaaaaatatattcgcatatattcccaagtcttcagttctatcttctaaaaaatcttcaacgtatggaatataataatcta :  46800
Seq2 : gtaatatctatagcatcctcaaaaaatatattcgcatatattcccaagtcttcagttctatcttctaaaaaatcttcaacgtatggaatataataatcta :  46800
Seq3 : gtaatatctatagcatcctcaaaaaatatattcgcatatattcccaagtcttcagttctatcttctaaaaaatcttcaacgtatggaatataataatcta :  46800
Seq4 : gtaatatctatagcatcctcaaaaaatatattcgcatatattcccaagtcttcagttctatcttctaaaaaatcttcaacgtatggaatataataatcta :  46800

                 *     46820         *     46840         *     46860         *     46880         *     46900
Seq1 : ttttacctcttctgatatcattaatgatatagtttttgacactatcttctgtcaattgattcttattcactatatctaagaaacggatagcgtccctagg :  46900
Seq2 : ttttacctcttctgatatcattaatgatatagtttttgacactatcttctgtcaattgattcttattcactatatctaagaaacggatagcgtccctagg :  46900
Seq3 : ttttacctcttctgatatcattaatgatatagtttttgacactatcttctgtcaattgattcttattcactatatctaagaaacggatagcgtccctagg :  46900
Seq4 : ttttacctcttctgatatcattaatgatatagtttttgacactatcttctgtcaattgattcttattcactatatctaagaaacggatagcgtccctagg :  46900
```

```
             *     46920         *     46940         *     46960         *     46980         *     47000
Seq1 : acgaactactgccattaatatctctattatagcttctggacataattcatctattataccagaattaatgggaactattccgtatctatctaacatagtt :  47000
Seq2 : acgaactactgccattaatatctctattatagcttctggacataattcatctattataccagaattaatgggaactattccgtatctatctaacatagtt :  47000
Seq3 : acgaactactgccattaatatctctattatagcttctggacataattcatctattataccagaattaatgggaactattccgtatctatctaacatagtt :  47000
Seq4 : acgaactactgccattaatatctctattatagcttctggacataattcatctattataccagaattaatgggaactattccgtatctatctaacatagtt :  47000

             *     47020         *     47040         *     47060         *     47080         *     47100
Seq1 : ttaagaaagtcagaatctaagacctgatgttcatatattggttcatacatgaaatgatctctattgatgatagtgactatttcattctctgaaaattggt :  47100
Seq2 : ttaagaaagtcagaatctaagacctgatgttcatatattggttcatacatgaaatgatctctattgatgatagtgactatttcattctctgaaaattggt :  47100
Seq3 : ttaagaaagtcagaatctaagacctgatgttcatatattggttcatacatgaaatgatctctattgatgatagtgactatttcattctctgaaaattggt :  47100
Seq4 : ttaagaaagtcagaatctaagacctgatgttcatatattggttcatacatgaaatgatctctattgatgatagtgactatttcattctctgaaaattggt :  47100

             *     47120         *     47140         *     47160         *     47180         *     47200
Seq1 : aactcattctatatatgctttccttgttgatgaaggatagaatatactcaatagaatttgtaccaacaaactgttctcttatgaatcgtatatcatcatc :  47200
Seq2 : aactcattctatatatgctttccttgttgatgaaggatagaatatactcaatagaatttgtaccaacaaactgttctcttatgaatcgtatatcatcatc :  47200
Seq3 : aactcattctatatatgctttccttgttgatgaaggatagaatatactcaatagaatttgtaccaacaaactgttctcttatgaatcgtatatcatcatc :  47200
Seq4 : aactcattctatatatgctttccttgttgatgaaggatagaatatactcaatagaatttgtaccaacaaactgttctcttatgaatcgtatatcatcatc :  47200

             *     47220         *     47240         *     47260         *     47280         *     47300
Seq1 : tgaaataatcatgtaaggcatacatttaacaattagagacttgtctcctgttatcaatatactattcttgtgataatttatgtgtgaggcaaatttgtcc :  47300
Seq2 : tgaaataatcatgtaaggcatacatttaacaattagagacttgtctcctgttatcaatatactattcttgtgataatttatgtgtgaggcaaatttgtcc :  47300
Seq3 : tgaaataatcatgtaaggcatacatttaacaattagagacttgtctcctgttatcaatatactattcttgtgataatttatgtgtgaggcaaatttgtcc :  47300
Seq4 : tgaaataatcatgtaaggcatacatttaacaattagagacttgtctcctgttatcaatatactattcttgtgataatttatgtgtgaggcaaatttgtcc :  47300

             *     47320         *     47340         *     47360         *     47380         *     47400
Seq1 : acgttctttaattttgttatagtagatatcaaatccaatggagctacagttcttggcttaaacagatatagtttttctggaacgaattctacaacattat :  47400
Seq2 : acgttctttaattttgttatagtagatatcaaatccaatggagctacagttcttggcttaaacagatatagtttttctggaacgaattctacaacattat :  47400
Seq3 : acgttctttaattttgttatagtagatatcaaatccaatggagctacagttcttggcttaaacagatatagtttttctggaacgaattctacaacattat :  47400
Seq4 : acgttctttaattttgttatagtagatatcaaatccaatggagctacagttcttggcttaaacagatatagtttttctggaacgaattctacaacattat :  47400

             *     47420         *     47440         *     47460         *     47480         *     47500
Seq1 : tataaaggactttgggtagataagtgggatgaaatcctattttaattaatgcgatagccttgtcctcgtgcagatatccaaacgcttttgtgatagtatg :  47500
Seq2 : tataaaggactttgggtagataagtgggatgaaatcctattttaattaatgcgatagccttgtcctcgtgcagatatccaaacgcttttgtgatagtatg :  47500
Seq3 : tataaaggactttgggtagataagtgggatgaaatcctattttaattaatgcgatagccttgtcctcgtgcagatatccaaacgcttttgtgatagtatg :  47500
Seq4 : tataaaggactttgggtagataagtgggatgaaatcctattttaattaatgcgatagccttgtcctcgtgcagatatccaaacgcttttgtgatagtatg :  47500

             *     47520         *     47540         *     47560         *     47580         *     47600
Seq1 : gcattcattgtctagaaacgctctacgaatatctgtgacagatatcatctttagagaatatactagtcgcgttaatagtactacaatttgtatttttaa :  47600
Seq2 : gcattcattgtctagaaacgctctacgaatatctgtgacagatatcatctttagagaatatactagtcgcgttaatagtactacaatttgtatttttaa :  47600
Seq3 : gcattcattgtctagaaacgctctacgaatatctgtgacagatatcatctttagagaatatactagtcgcgttaatagtactacaatttgtatttttaa :  47600
Seq4 : gcattcattgtctagaaacgctctacgaatatctgtgacagatatcatctttagagaatatactagtcgcgttaatagtactacaatttgtatttttaa :  47600
```

```
         *     47620         *     47640         *     47660         *     47680         *     47700
Seq1 : tctatctcaataaaaaaattaatatgtatgattcaatgtataactaaactactaactgttattgataactagaatcagaatctaatgatgacgtaaccaa :  47700
Seq2 : tctatctcaataaaaaaattaatatgtatgattcaatgtataactaaactactaactgttattgataactagaatcagaatctaatgatgacgtaaccaa :  47700
Seq3 : tctatctcaataaaaaaattaatatgtatgattcaatgtataactaaactactaactgttattgataactagaatcagaatctaatgatgacgtaaccaa :  47700
Seq4 : tctatctcaataaaaaaattaatatgtatgattcaatgtataactaaactactaactgttattgataactagaatcagaatctaatgatgacgtaaccaa :  47700

         *     47720         *     47740         *     47760         *     47780         *     47800
Seq1 : gaagtttatctactgccaatttagctgcattatttttagcatctcgtttagattttccatctgccttatcgaatactcttccgtcgatgtctacacaggc :  47800
Seq2 : gaagtttatctactgccaatttagctgcattatttttagcatctcgtttagattttccatctgccttatcgaatactcttccgtcgatgtctacacaggc :  47800
Seq3 : gaagtttatctactgccaatttagctgcattatttttagcatctcgtttagattttccatctgccttatcgaatactcttccgtcgatgtctacacaggc :  47800
Seq4 : gaagtttatctactgccaatttagctgcattatttttagcatctcgtttagattttccatctgccttatcgaatactcttccgtcgatgtctacacaggc :  47800

         *     47820         *     47840         *     47860         *     47880         *     47900
Seq1 : ataaaatgtaggagagttactaggccccactgattcaatacgaaaagaccaatctctcctagtaatttggcagtactcattaataacggtgacagggtta :  47900
Seq2 : ataaaatgtaggagagttactaggccccactgattcaatacgaaaagaccaatctctcctagtaatttggcagtactcattaataacggtgacagggtta :  47900
Seq3 : ataaaatgtaggagagttactaggccccactgattcaatacgaaaagaccaatctctcctagtaatttggcagtactcattaataacggtgacagggtta :  47900
Seq4 : ataaaatgtaggagagttactaggccccactgattcaatacgaaaagaccaatctctcctagtaatttggcagtactcattaataacggtgacagggtta :  47900

         *     47920         *     47940         *     47960         *     47980         *     48000
Seq1 : gcacctttccaatcaataattttttttagccggaataacatcatcaaaagacttatgatcctctctcattgatttttcgcgggatacatcatctattatga :  48000
Seq2 : gcacctttccaatcaataattttttttagccggaataacatcatcaaaagacttatgatcctctctcattgatttttcgcgggatacatcatctattatga :  48000
Seq3 : gcacctttccaatcaataattttttttagccggaataacatcatcaaaagacttatgatcctctctcattgatttttcgcgggatacatcatctattatga :  48000
Seq4 : gcacctttccaatcaataattttttttagccggaataacatcatcaaaagacttatgatcctctctcattgatttttcgcgggatacatcatctattatga :  48000

         *     48020         *     48040         *     48060         *     48080         *     48100
Seq1 : cgtcagccatagcatcagcatccggcttatccgcctccgttgtcataaaccaacgaggaggaatatcgtcggagctgtacaccatagcactacgttgaag :  48100
Seq2 : cgtcagccatagcatcagcatccggcttatccgcctccgttgtcataaaccaacgaggaggaatatcgtcggagctgtacaccatagcactacgttgaag :  48100
Seq3 : cgtcagccatagcatcagcatccggcttatccgcctccgttgtcataaaccaacgaggaggaatatcgtcggagctgtacaccatagcactacgttgaag :  48100
Seq4 : cgtcagccatagcatcagcatccggcttatccgcctccgttgtcataaaccaacgaggaggaatatcgtcggagctgtacaccatagcactacgttgaag :  48100

         *     48120         *     48140         *     48160         *     48180         *     48200
Seq1 : atcgtacagagctttattaacttctcgcttctccatattaagttgtctagttagttgtgcagcagtagctccttcgattccaatggttttaatagcctca :  48200
Seq2 : atcgtacagagctttattaacttctcgcttctccatattaagttgtctagttagttgtgcagcagtagctccttcgattccaatggttttaatagcctca :  48200
Seq3 : atcgtacagagctttattaacttctcgcttctccatattaagttgtctagttagttgtgcagcagtagctccttcgattccaatggttttaatagcctca :  48200
Seq4 : atcgtacagagctttattaacttctcgcttctccatattaagttgtctagttagttgtgcagcagtagctccttcgattccaatggttttaatagcctca :  48200

         *     48220         *     48240         *     48260         *     48280         *     48300
Seq1 : cacacaatctctgcgttagaacgctcgtcgatatagatttagacatttttagagagaactaacgcaatcagtaataaaactaatttattttatcatttt :  48300
Seq2 : cacacaatctctgcgttagaacgctcgtcgatatagatttagacatttttagagagaactaacgcaatcagtaataaaactaatttattttatcatttt :  48300
Seq3 : cacacaatctctgcgttagaacgctcgtcgatatagatttagacatttttagagagaactaacgcaatcagtaataaaactaatttattttatcatttt :  48300
Seq4 : cacacaatctctgcgttagaacgctcgtcgatatagatttagacatttttagagagaactaacgcaatcagtaataaaactaatttattttatcatttt :  48300
```

```
                 *     48320         *     48340         *     48360         *     48380         *     48400
Seq1 : ttttattcatcatcctctggtggttcgtcgtttctatcgaatgtagctctgattaacccgtcatctataggtgatgctggttctggagattctggaggag :  48400
Seq2 : ttttattcatcatcctctggtggttcgtcgtttctatcgaatgtagctctgattaacccgtcatctataggtgatgctggttctggagattctggaggag :  48400
Seq3 : ttttattcatcatcctctggtggttcgtcgtttctatcgaatgtagctctgattaacccgtcatctataggtgatgctggttctggagattctggaggag :  48400
Seq4 : ttttattcatcatcctctggtggttcgtcgtttctatcgaatgtagctctgattaacccgtcatctataggtgatgctggttctggagattctggaggag :  48400

                 *     48420         *     48440         *     48460         *     48480         *     48500
Seq1 : atggattattatctggaagaatctctgttatttccttgttttcatgtatcgattgcgttgtaacattaagattgcgaaatgctctaaatttgggaggctt :  48500
Seq2 : atggattattatctggaagaatctctgttatttccttgttttcatgtatcgattgcgttgtaacattaagattgcgaaatgctctaaatttgggaggctt :  48500
Seq3 : atggattattatctggaagaatctctgttatttccttgttttcatgtatcgattgcgttgtaacattaagattgcgaaatgctctaaatttgggaggctt :  48500
Seq4 : atggattattatctggaagaatctctgttatttccttgttttcatgtatcgattgcgttgtaacattaagattgcgaaatgctctaaatttgggaggctt :  48500

                 *     48520         *     48540         *     48560         *     48580         *     48600
Seq1 : aaagtgttgtttgcaatctctacacgcgtgtctaactagtggaggttcgtcagcggctctagtttgaatcatcatcggcgtagtattcctacttttacag :  48600
Seq2 : aaagtgttgtttgcaatctctacacgcgtgtctaactagtggaggttcgtcagcggctctagtttgaatcatcatcggcgtagtattcctacttttacag :  48600
Seq3 : aaagtgttgtttgcaatctctacacgcgtgtctaactagtggaggttcgtcagcggctctagtttgaatcatcatcggcgtagtattcctacttttacag :  48600
Seq4 : aaagtgttgtttgcaatctctacacgcgtgtctaactagtggaggttcgtcagcggctctagtttgaatcatcatcggcgtagtattcctacttttacag :  48600

                 *     48620         *     48640         *     48660         *     48680         *     48700
Seq1 : ttaggacacggtgtattgtatttctcgtcgagaacgttaaaataatcgttgtaactcacatcctttattttatctatattgtattctactcctttcttaa :  48700
Seq2 : ttaggacacggtgtattgtatttctcgtcgagaacgttaaaataatcgttgtaactcacatcctttattttatctatattgtattctactcctttcttaa :  48700
Seq3 : ttaggacacggtgtattgtatttctcgtcgagaacgttaaaataatcgttgtaactcacatcctttattttatctatattgtattctactcctttcttaa :  48700
Seq4 : ttaggacacggtgtattgtatttctcgtcgagaacgttaaaataatcgttgtaactcacatcctttattttatctatattgtattctactcctttcttaa :  48700

                 *     48720         *     48740         *     48760         *     48780         *     48800
Seq1 : tgcattttataccgaataagagatagcgaaggaattctttttcggtgccgctagtacccttaatcatatcacatagtgttttatattccaaatttgtggc :  48800
Seq2 : tgcattttataccgaataagagatagcgaaggaattctttttcggtgccgctagtacccttaatcatatcacatagtgttttatattccaaatttgtggc :  48800
Seq3 : tgcattttataccgaataagagatagcgaaggaattctttttcggtgccgctagtacccttaatcatatcacatagtgttttatattccaaatttgtggc :  48800
Seq4 : tgcattttataccgaataagagatagcgaaggaattctttttcggtgccgctagtacccttaatcatatcacatagtgttttatattccaaatttgtggc :  48800

                 *     48820         *     48840         *     48860         *     48880         *     48900
Seq1 : aatagacggtttatttctatacgatagtttgtttctggaatcctttgagtattctataccaatattattctttgattcgaatttagtttcttcgatatta :  48900
Seq2 : aatagacggtttatttctatacgatagtttgtttctggaatcctttgagtattctataccaatattattctttgattcgaatttagtttcttcgatatta :  48900
Seq3 : aatagacggtttatttctatacgatagtttgtttctggaatcctttgagtattctataccaatattattctttgattcgaatttagtttcttcgatatta :  48900
Seq4 : aatagacggtttatttctatacgatagtttgtttctggaatcctttgagtattctataccaatattattctttgattcgaatttagtttcttcgatatta :  48900

                 *     48920         *     48940         *     48960         *     48980         *     49000
Seq1 : gattttgtattacctatattcttgatgtagtactttgatgatttttccatggcccattctattaagtcttccaagttggcatcatccacatattgtgata :  49000
Seq2 : gattttgtattacctatattcttgatgtagtactttgatgatttttccatggcccattctattaagtcttccaagttggcatcatccacatattgtgata :  49000
Seq3 : gattttgtattacctatattcttgatgtagtactttgatgatttttccatggcccattctattaagtcttccaagttggcatcatccacatattgtgata :  49000
Seq4 : gattttgtattacctatattcttgatgtagtactttgatgatttttccatggcccattctattaagtcttccaagttggcatcatccacatattgtgata :  49000
```

* 49020 * 49040 * 49060 * 49080 * 49100
Seq1 : gtaattctcggatatcagtagcggttaccgccattgatgtttgttcattggatgagtaactactaatgtatacattttccatttataacacttatgtatt : 49100
Seq2 : gtaattctcggatatcagtagcggttaccgccattgatgtttgttcattggatgagtaactactaatgtatacattttccatttataacacttatgtatt : 49100
Seq3 : gtaattctcggatatcagtagcggttaccgccattgatgtttgttcattggatgagtaactactaatgtatacattttccatttataacacttatgtatt : 49100
Seq4 : gtaattctcggatatcagtagcggttaccgccattgatgtttgttcattggatgagtaactactaatgtatacattttccatttataacacttatgtatt : 49100

* 49120 * 49140 * 49160 * 49180 * 49200
Seq1 : aactttgttcatttatatttttcattattatgttgatattaacaaaagtgaatatatatatgttaataattgtattgtggttatacggctacaattttta : 49200
Seq2 : aactttgttcatttatatttttcattattatgttgatattaacaaaagtgaatatatatatgttaataattgtattgtggttatacggctacaattttta : 49200
Seq3 : aactttgttcatttatatttttcattattatgttgatattaacaaaagtgaatatatatatgttaataattgtattgtggttatacggctacaattttta : 49200
Seq4 : aactttgttcatttatatttttcattattatgttgatattaacaaaagtgaatatatatatgttaataattgtattgtggttatacggctacaattttta : 49200

* 49220 * 49240 * 49260 * 49280 * 49300
Seq1 : taattagtgaaagtcagtgtccgatgatcaatgacgatagctttactctgaaaagaaagtatcaaatcgatagtgcggagtcaacaataaaaatggataa : 49300
Seq2 : taattagtgaaagtcagtgtccgatgatcaatgacgatagctttactctgaaaagaaagtatcaaatcgatagtgcggagtcaacaataaaaatggataa : 49300
Seq3 : taattagtgaaagtcagtgtccgatgatcaatgacgatagctttactctgaaaagaaagtatcaaatcgatagtgcggagtcaacaataaaaatggataa : 49300
Seq4 : taattagtgaaagtcagtgtccgatgatcaatgacgatagctttactctgaaaagaaagtatcaaatcgatagtgcggagtcaacaataaaaatggataa : 49300

* 49320 * 49340 * 49360 * 49380 * 49400
Seq1 : gaagaggacaaagtttcagaatagagccaaaatggtaaaagaaataaatcagacaataagagcagcacaaactcattacgagacattgaaactaggatac : 49400
Seq2 : gaagaggacaaagtttcagaatagagccaaaatggtaaaagaaataaatcagacaataagagcagcacaaactcattacgagacattgaaactaggatac : 49400
Seq3 : gaagaggacaaagtttcagaatagagccaaaatggtaaaagaaataaatcagacaataagagcagcacaaactcattacgagacattgaaactaggatac : 49400
Seq4 : gaagaggacaaagtttcagaatagagccaaaatggtaaaagaaataaatcagacaataagagcagcacaaactcattacgagacattgaaactaggatac : 49400

* 49420 * 49440 * 49460 * 49480 * 49500
Seq1 : ataaaatttaagagaatgattaggactactactctagaagatatagcaccatctattccaaataatcagaaaacttataaactattctcggacatttcag : 49500
Seq2 : ataaaatttaagagaatgattaggactactactctagaagatatagcaccatctattccaaataatcagaaaacttataaactattctcggacatttcag : 49500
Seq3 : ataaaatttaagagaatgattaggactactactctagaagatatagcaccatctattccaaataatcagaaaacttataaactattctcggacatttcag : 49500
Seq4 : ataaaatttaagagaatgattaggactactactctagaagatatagcaccatctattccaaataatcagaaaacttataaactattctcggacatttcag : 49500

* 49520 * 49540 * 49560 * 49580 * 49600
Seq1 : ccatcggcaaagcatcacggaatccaagtaagatggtatatgctctgctgctttacatgtttcccaatttgtttggagatgatcatagattcattcgtta : 49600
Seq2 : ccatcggcaaagcatcacggaatccaagtaagatggtatatgctctgctgctttacatgtttcccaatttgtttggagatgatcatagattcattcgtta : 49600
Seq3 : ccatcggcaaagcatcacggaatccaagtaagatggtatatgctctgctgctttacatgtttcccaatttgtttggagatgatcatagattcattcgtta : 49600
Seq4 : ccatcggcaaagcatcacggaatccaagtaagatggtatatgctctgctgctttacatgtttcccaatttgtttggagatgatcatagattcattcgtta : 49600

* 49620 * 49640 * 49660 * 49680 * 49700
Seq1 : tagaatgcatccaatgagtaaaatcaaacacaagatcttctctcctttcaaacttaatcttattagaatattagtggaagaaagattctataataatgaa : 49700
Seq2 : tagaatgcatccaatgagtaaaatcaaacacaagatcttctctcctttcaaacttaatcttattagaatattagtggaagaaagattctataataatgaa : 49700
Seq3 : tagaatgcatccaatgagtaaaatcaaacacaagatcttctctcctttcaaacttaatcttattagaatattagtggaagaaagattctataataatgaa : 49700
Seq4 : tagaatgcatccaatgagtaaaatcaaacacaagatcttctctcctttcaaacttaatcttattagaatattagtggaagaaagattctataataatgaa : 49700

```
             *     49720         *     49740         *     49760         *     49780         *     49800
Seq1 : tgcagatctaataaatggagaataattggaacacaagttgataaaatgttgatagctgaatctgataaatatacaatagatgcaaggtataacctaaaac :  49800
Seq2 : tgcagatctaataaatggagaataattggaacacaagttgataaaatgttgatagctgaatctgataaatatacaatagatgcaaggtataacctaaaac :  49800
Seq3 : tgcagatctaataaatggagaataattggaacacaagttgataaaatgttgatagctgaatctgataaatatacaatagatgcaaggtataacctaaaac :  49800
Seq4 : tgcagatctaataaatggagaataattggaacacaagttgataaaatgttgatagctgaatctgataaatatacaatagatgcaaggtataacctaaaac :  49800

             *     49820         *     49840         *     49860         *     49880         *     49900
Seq1 : ccatgtatagaatcaagggaaaatctgaagaagataccctctttatcaaacagatggtagaacaatgtgtgacatcccaggaattggtggaaaaagtgtt :  49900
Seq2 : ccatgtatagaatcaagggaaaatctgaagaagataccctctttatcaaacagatggtagaacaatgtgtgacatcccaggaattggtggaaaaagtgtt :  49900
Seq3 : ccatgtatagaatcaagggaaaatctgaagaagataccctctttatcaaacagatggtagaacaatgtgtgacatcccaggaattggtggaaaaagtgtt :  49900
Seq4 : ccatgtatagaatcaagggaaaatctgaagaagataccctctttatcaaacagatggtagaacaatgtgtgacatcccaggaattggtggaaaaagtgtt :  49900

             *     49920         *     49940         *     49960         *     49980         *     50000
Seq1 : gaagatactgtttagagatttgttcaagagtggagaatacaaagcgtacagatacgatgatgatgtagaaaatggatttattggattggatacactaaaa :  50000
Seq2 : gaagatactgtttagagatttgttcaagagtggagaatacaaagcgtacagatacgatgatgatgtagaaaatggatttattggattggatacactaaaa :  50000
Seq3 : gaagatactgtttagagatttgttcaagagtggagaatacaaagcgtacagatacgatgatgatgtagaaaatggatttattggattggatacactaaaa :  50000
Seq4 : gaagatactgtttagagatttgttcaagagtggagaatacaaagcgtacagatacgatgatgatgtagaaaatggatttattggattggatacactaaaa :  50000

             *     50020         *     50040         *     50060         *     50080         *     50100
Seq1 : ttaaacattgttcatgatatagttgaaccatgtatgcctgttcgtaggccagtggctaagatactgtgtaaagaaatggtaaataaatactttgagaatc :  50100
Seq2 : ttaaacattgttcatgatatagttgaaccatgtatgcctgttcgtaggccagtggctaagatactgtgtaaagaaatggtaaataaatactttgagaatc :  50100
Seq3 : ttaaacattgttcatgatatagttgaaccatgtatgcctgttcgtaggccagtggctaagatactgtgtaaagaaatggtaaataaatactttgagaatc :  50100
Seq4 : ttaaacattgttcatgatatagttgaaccatgtatgcctgttcgtaggccagtggctaagatactgtgtaaagaaatggtaaataaatactttgagaatc :  50100

             *     50120         *     50140         *     50160         *     50180         *     50200
Seq1 : cgctacatattattggtaaaaatcttcaagagtgcattgactttgttagtgaataggcatttcatctttctccaatactaattcaaattgttaaattaat :  50200
Seq2 : cgctacatattattggtaaaaatcttcaagagtgcattgactttgttagtgaataggcatttcatctttctccaatactaattcaaattgttaaattaat :  50200
Seq3 : cgctacatattattggtaaaaatcttcaagagtgcattgactttgttagtgaataggcatttcatctttctccaatactaattcaaattgttaaattaat :  50200
Seq4 : cgctacatattattggtaaaaatcttcaagagtgcattgactttgttagtgaataggcatttcatctttctccaatactaattcaaattgttaaattaat :  50200

             *     50220         *     50240         *     50260         *     50280         *     50300
Seq1 : aatggatagtataaatagttattagtgataaaatagtaaaaataattattagaataagagtgtagtatcatagataactctcttctataaaaatggattt :  50300
Seq2 : aatggatagtataaatagttattagtgataaaatagtaaaaataattattagaataagagtgtagtatcatagataactctcttctataaaaatggattt :  50300
Seq3 : aatggatagtataaatagttattagtgataaaatagtaaaaataattattagaataagagtgtagtatcatagataactctcttctataaaaatggattt :  50300
Seq4 : aatggatagtataaatagttattagtgataaaatagtaaaaataattattagaataagagtgtagtatcatagataactctcttctataaaaatggattt :  50300

             *     50320         *     50340         *     50360         *     50380         *     50400
Seq1 : tattcgtagaaagtatcttatatacacagtagaaaataatatagattttttaaaggatgatacattaagtaaagtaaacaattttaccctcaatcatgta :  50400
Seq2 : tattcgtagaaagtatcttatatacacagtagaaaataatatagattttttaaaggatgatacattaagtaaagtaaacaattttaccctcaatcatgta :  50400
Seq3 : tattcgtagaaagtatcttatatacacagtagaaaataatatagattttttaaaggatgatacattaagtaaagtaaacaattttaccctcaatcatgta :  50400
Seq4 : tattcgtagaaagtatcttatatacacagtagaaaataatatagattttttaaaggatgatacattaagtaaagtaaacaattttaccctcaatcatgta :  50400
```

```
                 *     50420         *     50440         *     50460         *     50480         *     50500
Seq1 : ctagctctcaagtatctagttagcaattttcctcaacatgttattactaaggatgtattagctaataccaatttttttgttttcatacatatggtacgat :  50500
Seq2 : ctagctctcaagtatctagttagcaattttcctcaacatgttattactaaggatgtattagctaataccaatttttttgttttcatacatatggtacgat :  50500
Seq3 : ctagctctcaagtatctagttagcaattttcctcaacatgttattactaaggatgtattagctaataccaatttttttgttttcatacatatggtacgat :  50500
Seq4 : ctagctctcaagtatctagttagcaattttcctcaacatgttattactaaggatgtattagctaataccaatttttttgttttcatacatatggtacgat :  50500

                 *     50520         *     50540         *     50560         *     50580         *     50600
Seq1 : gttgtaaagtgtacgaagcggttttacgacacgcatttgatgcacccacgttgtacgttaaagcattgactaagaattatttatcgtttagtaacacaat :  50600
Seq2 : gttgtaaagtgtacgaagcggttttacgacacgcatttgatgcacccacgttgtacgttaaagcattgactaagaattatttatcgtttagtaacacaat :  50600
Seq3 : gttgtaaagtgtacgaagcggttttacgacacgcatttgatgcacccacgttgtacgttaaagcattgactaagaattatttatcgtttagtaacacaat :  50600
Seq4 : gttgtaaagtgtacgaagcggttttacgacacgcatttgatgcacccacgttgtacgttaaagcattgactaagaattatttatcgtttagtaacacaat :  50600

                 *     50620         *     50640         *     50660         *     50680         *     50700
Seq1 : acaatcgtacaaggaaaccgtgcataaactaacacaagatgaaaaattttagaggttgccaaatacatggacgaattaggagaacttataggcgtaaat :  50700
Seq2 : acaatcgtacaaggaaaccgtgcataaactaacacaagatgaaaaattttagaggttgccaaatacatggacgaattaggagaacttataggcgtaaat :  50700
Seq3 : acaatcgtacaaggaaaccgtgcataaactaacacaagatgaaaaattttagaggttgccaaatacatggacgaattaggagaacttataggcgtaaat :  50700
Seq4 : acaatcgtacaaggaaaccgtgcataaactaacacaagatgaaaaattttagaggttgccaaatacatggacgaattaggagaacttataggcgtaaat :  50700

                 *     50720         *     50740         *     50760         *     50780         *     50800
Seq1 : tatgacttagttcttaatccattatttcacggaggggaacccatcaaagatatggaaatcatttttttaaaactgtttaagaaaacagacttcaaagttg :  50800
Seq2 : tatgacttagttcttaatccattatttcacggaggggaacccatcaaagatatggaaatcatttttttaaaactgtttaagaaaacagacttcaaagttg :  50800
Seq3 : tatgacttagttcttaatccattatttcacggaggggaacccatcaaagatatggaaatcatttttttaaaactgtttaagaaaacagacttcaaagttg :  50800
Seq4 : tatgacttagttcttaatccattatttcacggaggggaacccatcaaagatatggaaatcatttttttaaaactgtttaagaaaacagacttcaaagttg :  50800

                 *     50820         *     50840         *     50860         *     50880         *     50900
Seq1 : ttaaaaaattaagtgttataagattacttatttggcttacctaagcaagaaagatacaggcatagagtttgcggataatgatagacaagatatatatac :  50900
Seq2 : ttaaaaaattaagtgttataagattacttatttggcttacctaagcaagaaagatacaggcatagagtttgcggataatgatagacaagatatatatac :  50900
Seq3 : ttaaaaaattaagtgttataagattacttatttggcttacctaagcaagaaagatacaggcatagagtttgcggataatgatagacaagatatatatac :  50900
Seq4 : ttaaaaaattaagtgttataagattacttatttggcttacctaagcaagaaagatacaggcatagagtttgcggataatgatagacaagatatatatac :  50900

                 *     50920         *     50940         *     50960         *     50980         *     51000
Seq1 : tctatttcaacaaactggtagaatcgtccatagcaatctaacagaaacgtttagagattatatctttcccggagataagactagctattgggtgtggtta :  51000
Seq2 : tctatttcaacaaactggtagaatcgtccatagcaatctaacagaaacgtttagagattatatctttcccggagataagactagctattgggtgtggtta :  51000
Seq3 : tctatttcaacaaactggtagaatcgtccatagcaatctaacagaaacgtttagagattatatctttcccggagataagactagctattgggtgtggtta :  51000
Seq4 : tctatttcaacaaactggtagaatcgtccatagcaatctaacagaaacgtttagagattatatctttcccggagataagactagctattgggtgtggtta :  51000

                 *     51020         *     51040         *     51060         *     51080         *     51100
Seq1 : aacgaaagtatagctaatgatgcggatattgttcttaatagacacgccattaccatgtatgataaaattcttagttatatatactctgagataaaacaag :  51100
Seq2 : aacgaaagtatagctaatgatgcggatattgttcttaatagacacgccattaccatgtatgataaaattcttagttatatatactctgagataaaacaag :  51100
Seq3 : aacgaaagtatagctaatgatgcggatattgttcttaatagacacgccattaccatgtatgataaaattcttagttatatatactctgagataaaacaag :  51100
Seq4 : aacgaaagtatagctaatgatgcggatattgttcttaatagacacgccattaccatgtatgataaaattcttagttatatatactctgagataaaacaag :  51100
```

```
                 *     51120         *     51140         *     51160         *     51180         *     51200
Seq1 : gacgcgttaataaaaacatgcttaagttagtttatatctttgagcctgaaaaagatatcagagaacttctgctagaaatcatatatgatattcctggaga :  51200
Seq2 : gacgcgttaataaaaacatgcttaagttagtttatatctttgagcctgaaaaagatatcagagaacttctgctagaaatcatatatgatattcctggaga :  51200
Seq3 : gacgcgttaataaaaacatgcttaagttagtttatatctttgagcctgaaaaagatatcagagaacttctgctagaaatcatatatgatattcctggaga :  51200
Seq4 : gacgcgttaataaaaacatgcttaagttagtttatatctttgagcctgaaaaagatatcagagaacttctgctagaaatcatatatgatattcctggaga :  51200

                 *     51220         *     51240         *     51260         *     51280         *     51300
Seq1 : tatcctatctattattgatgcaaaaaacgacgattggaaaaaatatttttattagtttttataaagctaattttattaacggtaatacatttattagtgat :  51300
Seq2 : tatcctatctattattgatgcaaaaaacgacgattggaaaaaatatttttattagtttttataaagctaattttattaacggtaatacatttattagtgat :  51300
Seq3 : tatcctatctattattgatgcaaaaaacgacgattggaaaaaatatttttattagtttttataaagctaattttattaacggtaatacatttattagtgat :  51300
Seq4 : tatcctatctattattgatgcaaaaaacgacgattggaaaaaatatttttattagtttttataaagctaattttattaacggtaatacatttattagtgat :  51300

                 *     51320         *     51340         *     51360         *     51380         *     51400
Seq1 : agaacgtttaacgaggacttattcagagttgttgttcaaatagatcccgaatatttcgataatgaacgaattatgtctttattctctacgagtgctgcgg :  51400
Seq2 : agaacgtttaacgaggacttattcagagttgttgttcaaatagatcccgaatatttcgataatgaacgaattatgtctttattctctacgagtgctgcgg :  51400
Seq3 : agaacgtttaacgaggacttattcagagttgttgttcaaatagatcccgaatatttcgataatgaacgaattatgtctttattctctacgagtgctgcgg :  51400
Seq4 : agaacgtttaacgaggacttattcagagttgttgttcaaatagatcccgaatatttcgataatgaacgaattatgtctttattctctacgagtgctgcgg :  51400

                 *     51420         *     51440         *     51460         *     51480         *     51500
Seq1 : acattaaacgatttgatgagttagatattaataacagttatatatctaatataatttatgaggtgaacgatatcacattagatacaatggatgatatgaa :  51500
Seq2 : acattaaacgatttgatgagttagatattaataacagttatatatctaatataatttatgaggtgaacgatatcacattagatacaatggatgatatgaa :  51500
Seq3 : acattaaacgatttgatgagttagatattaataacagttatatatctaatataatttatgaggtgaacgatatcacattagatacaatggatgatatgaa :  51500
Seq4 : acattaaacgatttgatgagttagatattaataacagttatatatctaatataatttatgaggtgaacgatatcacattagatacaatggatgatatgaa :  51500

                 *     51520         *     51540         *     51560         *     51580         *     51600
Seq1 : gaagtgtcaaatctttaacgaggatacgtcgtattatgttaaggaatacaatacatacctgtttttgcacgagtcggatcccatggtcatagagaacgga :  51600
Seq2 : gaagtgtcaaatctttaacgaggatacgtcgtattatgttaaggaatacaatacatacctgtttttgcacgagtcggatcccatggtcatagagaacgga :  51600
Seq3 : gaagtgtcaaatctttaacgaggatacgtcgtattatgttaaggaatacaatacatacctgtttttgcacgagtcggatcccatggtcatagagaacgga :  51600
Seq4 : gaagtgtcaaatctttaacgaggatacgtcgtattatgttaaggaatacaatacatacctgtttttgcacgagtcggatcccatggtcatagagaacgga :  51600

                 *     51620         *     51640         *     51660         *     51680         *     51700
Seq1 : atactaaagaaactgtcatctataaaatccaagagtagacggctgaacttgtttagcaaaaacattttaaaatattatttagacggacaattggctcgtc :  51700
Seq2 : atactaaagaaactgtcatctataaaatccaagagtagacggctgaacttgtttagcaaaaacattttaaaatattatttagacggacaattggctcgtc :  51700
Seq3 : atactaaagaaactgtcatctataaaatccaagagtagacggctgaacttgtttagcaaaaacattttaaaatattatttagacggacaattggctcgtc :  51700
Seq4 : atactaaagaaactgtcatctataaaatccaagagtagacggctgaacttgtttagcaaaaacattttaaaatattatttagacggacaattggctcgtc :  51700

                 *     51720         *     51740         *     51760         *     51780         *     51800
Seq1 : taggtcttgtgttagatgattataaaggagacttgttagttaaaatgataaaccatcttaagtctgtggaggatgtatccgcattcgttcgattttctac :  51800
Seq2 : taggtcttgtgttagatgattataaaggagacttgttagttaaaatgataaaccatcttaagtctgtggaggatgtatccgcattcgttcgattttctac :  51800
Seq3 : taggtcttgtgttagatgattataaaggagacttgttagttaaaatgataaaccatcttaagtctgtggaggatgtatccgcattcgttcgattttctac :  51800
Seq4 : taggtcttgtgttagatgattataaaggagacttgttagttaaaatgataaaccatcttaagtctgtggaggatgtatccgcattcgttcgattttctac :  51800
```

```
              *     51820         *     51840         *     51860         *     51880         *     51900
Seq1 : agataaaaaccctagtattcttccatcgctaatcaaaactattttagctagttataatatttccatcatcgtcttatttcaaaggtttttaagagataat :  51900
Seq2 : agataaaaaccctagtattcttccatcgctaatcaaaactattttagctagttataatatttccatcatcgtcttatttcaaaggtttttaagagataat :  51900
Seq3 : agataaaaaccctagtattcttccatcgctaatcaaaactattttagctagttataatatttccatcatcgtcttatttcaaaggtttttaagagataat :  51900
Seq4 : agataaaaaccctagtattcttccatcgctaatcaaaactattttagctagttataatatttccatcatcgtcttatttcaaaggtttttaagagataat :  51900

              *     51920         *     51940         *     51960         *     51980         *     52000
Seq1 : ctatatcatgtagaagaattcttggataaaagcatccatctaaccaagacggataagaaatatatacttcaattgataagacacggtagatcatagaaca :  52000
Seq2 : ctatatcatgtagaagaattcttggataaaagcatccatctaaccaagacggataagaaatatatacttcaattgataagacacggtagatcatagaaca :  52000
Seq3 : ctatatcatgtagaagaattcttggataaaagcatccatctaaccaagacggataagaaatatatacttcaattgataagacacggtagatcatagaaca :  52000
Seq4 : ctatatcatgtagaagaattcttggataaaagcatccatctaaccaagacggataagaaatatatacttcaattgataagacacggtagatcatagaaca :  52000

              *     52020         *     52040         *     52060         *     52080         *     52100
Seq1 : gaccaaatatattattaataatttgtatatacatagatataattatcacatattaaaaattcacacattttgataaatgggaactgctgcaacaattca :  52100
Seq2 : gaccaaatatattattaataatttgtatatacatagatataattatcacatattaaaaattcacacattttgataaatgggaactgctgcaacaattca :  52100
Seq3 : gaccaaatatattattaataatttgtatatacatagatataattatcacatattaaaaattcacacattttgataaatgggaactgctgcaacaattca :  52100
Seq4 : gaccaaatatattattaataatttgtatatacatagatataattatcacatattaaaaattcacacattttgataaatgggaactgctgcaacaattca :  52100

              *     52120         *     52140         *     52160         *     52180         *     52200
Seq1 : gactcccaccaaattaatgaataaagaaaatgcagaaatgattttggaaaaaattgttgatcatatagttatgtatattagtgacgaatcaagtgattca :  52200
Seq2 : gactcccaccaaattaatgaataaagaaaatgcagaaatgattttggaaaaaattgttgatcatatagttatgtatattagtgacgaatcaagtgattca :  52200
Seq3 : gactcccaccaaattaatgaataaagaaaatgcagaaatgattttggaaaaaattgttgatcatatagttatgtatattagtgacgaatcaagtgattca :  52200
Seq4 : gactcccaccaaattaatgaataaagaaaatgcagaaatgattttggaaaaaattgttgatcatatagttatgtatattagtgacgaatcaagtgattca :  52200

              *     52220         *     52240         *     52260         *     52280         *     52300
Seq1 : gaaaataatcctgaatatattgattttcgtaacagatacgaagactatagatctctcattataaaaagtgatcacgagtttgtaaagctatgtaaaaatc :  52300
Seq2 : gaaaataatcctgaatatattgattttcgtaacagatacgaagactatagatctctcattataaaaagtgatcacgagtttgtaaagctatgtaaaaatc :  52300
Seq3 : gaaaataatcctgaatatattgattttcgtaacagatacgaagactatagatctctcattataaaaagtgatcacgagtttgtaaagctatgtaaaaatc :  52300
Seq4 : gaaaataatcctgaatatattgattttcgtaacagatacgaagactatagatctctcattataaaaagtgatcacgagtttgtaaagctatgtaaaaatc :  52300

              *     52320         *     52340         *     52360         *     52380         *     52400
Seq1 : atgcagagaaaagttctccagaaacgcaacaaatgattatcaaacacatatacgaacaatatcttattccagtatctgaagtactattaaaacctataat :  52400
Seq2 : atgcagagaaaagttctccagaaacgcaacaaatgattatcaaacacatatacgaacaatatcttattccagtatctgaagtactattaaaacctataat :  52400
Seq3 : atgcagagaaaagttctccagaaacgcaacaaatgattatcaaacacatatacgaacaatatcttattccagtatctgaagtactattaaaacctataat :  52400
Seq4 : atgcagagaaaagttctccagaaacgcaacaaatgattatcaaacacatatacgaacaatatcttattccagtatctgaagtactattaaaacctataat :  52400

              *     52420         *     52440         *     52460         *     52480         *     52500
Seq1 : gtccatgggtgacataattacatataacggatgtaaagacaatgaatggatgctagaacaactctctaccctaaactttaacaatctccgcacatggaac :  52500
Seq2 : gtccatgggtgacataattacatataacggatgtaaagacaatgaatggatgctagaacaactctctaccctaaactttaacaatctccgcacatggaac :  52500
Seq3 : gtccatgggtgacataattacatataacggatgtaaagacaatgaatggatgctagaacaactctctaccctaaactttaacaatctccgcacatggaac :  52500
Seq4 : gtccatgggtgacataattacatataacggatgtaaagacaatgaatggatgctagaacaactctctaccctaaactttaacaatctccgcacatggaac :  52500
```

```
                  *     52520          *     52540          *     52560          *     52580          *     52600
Seq1 : tcatgtagcataggcaatgtaacgcgtctgttttatacatttttttagttatctgatgaaagataaactaaatatataagtataatcccattctaatactt :  52600
Seq2 : tcatgtagcataggcaatgtaacgcgtctgttttatacatttttttagttatctgatgaaagataaactaaatatataagtataatcccattctaatactt :  52600
Seq3 : tcatgtagcataggcaatgtaacgcgtctgttttatacatttttttagttatctgatgaaagataaactaaatatataagtataatcccattctaatactt :  52600
Seq4 : tcatgtagcataggcaatgtaacgcgtctgttttatacatttttttagttatctgatgaaagataaactaaatatataagtataatcccattctaatactt :  52600

                  *     52620          *     52640          *     52660          *     52680          *     52700
Seq1 : taacctgatgtattagcatcttattagaatattaacctaactaaaagacataacataaaaactcattacatagttgataaaaagcggtaggatataaata :  52700
Seq2 : taacctgatgtattagcatcttattagaatattaacctaactaaaagacataacataaaaactcattacatagttgataaaaagcggtaggatataaata :  52700
Seq3 : taacctgatgtattagcatcttattagaatattaacctaactaaaagacataacataaaaactcattacatagttgataaaaagcggtaggatataaata :  52700
Seq4 : taacctgatgtattagcatcttattagaatattaacctaactaaaagacataacataaaaactcattacatagttgataaaaagcggtaggatataaata :  52700

                  *     52720          *     52740          *     52760          *     52780          *     52800
Seq1 : ttatggctgccaccgttccgcgttttgacgacgtgtacaaaaatgcacaaagaagaattctagatcaagaaacatttttttagtagaggtctaagtagacc :  52800
Seq2 : ttatggctgccaccgttccgcgttttgacgacgtgtacaaaaatgcacaaagaagaattctagatcaagaaacatttttttagtagaggtctaagtagacc :  52800
Seq3 : ttatggctgccaccgttccgcgttttgacgacgtgtacaaaaatgcacaaagaagaattctagatcaagaaacatttttttagtagaggtctaagtagacc :  52800
Seq4 : ttatggctgccaccgttccgcgttttgacgacgtgtacaaaaatgcacaaagaagaattctagatcaagaaacatttttttagtagaggtctaagtagacc :  52800

                  *     52820          *     52840          *     52860          *     52880          *     52900
Seq1 : gttaatgaaaaacacatatctatttgataattacgcgtatggatggataccagaaactgcaatttggagtagtagatacgcaaacttagatgcaagtgac :  52900
Seq2 : gttaatgaaaaacacatatctatttgataattacgcgtatggatggataccagaaactgcaatttggagtagtagatacgcaaacttagatgcaagtgac :  52900
Seq3 : gttaatgaaaaacacatatctatttgataattacgcgtatggatggataccagaaactgcaatttggagtagtagatacgcaaacttagatgcaagtgac :  52900
Seq4 : gttaatgaaaaacacatatctatttgataattacgcgtatggatggataccagaaactgcaatttggagtagtagatacgcaaacttagatgcaagtgac :  52900

                  *     52920          *     52940          *     52960          *     52980          *     53000
Seq1 : tattatcccatttcgttgggattacttaaaaagttcgagtttctcatgtctctatataaaggtcctattccagtatacgaagaaaaagtaaatactgaat :  53000
Seq2 : tattatcccatttcgttgggattacttaaaaagttcgagtttctcatgtctctatataaaggtcctattccagtatacgaagaaaaagtaaatactgaat :  53000
Seq3 : tattatcccatttcgttgggattacttaaaaagttcgagtttctcatgtctctatataaaggtcctattccagtatacgaagaaaaagtaaatactgaat :  53000
Seq4 : tattatcccatttcgttgggattacttaaaaagttcgagtttctcatgtctctatataaaggtcctattccagtatacgaagaaaaagtaaatactgaat :  53000

                  *     53020          *     53040          *     53060          *     53080          *     53100
Seq1 : tcattgctaatggatcgttctctggtagatacgtatcatatcttcgaaagttttctgctcttccaacaaacgagtttattagtttttttgttactgacttc :  53100
Seq2 : tcattgctaatggatcgttctctggtagatacgtatcatatcttcgaaagttttctgctcttccaacaaacgagtttattagtttttttgttactgacttc :  53100
Seq3 : tcattgctaatggatcgttctctggtagatacgtatcatatcttcgaaagttttctgctcttccaacaaacgagtttattagtttttttgttactgacttc :  53100
Seq4 : tcattgctaatggatcgttctctggtagatacgtatcatatcttcgaaagttttctgctcttccaacaaacgagtttattagtttttttgttactgacttc :  53100

                  *     53120          *     53140          *     53160          *     53180          *     53200
Seq1 : cattccaatctataatatcttgttctggtttaaaaatactcagtttgatattactaaacacacattattcagatacgtctatacagataatgccaaacac :  53200
Seq2 : cattccaatctataatatcttgttctggtttaaaaatactcagtttgatattactaaacacacattattcagatacgtctatacagataatgccaaacac :  53200
Seq3 : cattccaatctataatatcttgttctggtttaaaaatactcagtttgatattactaaacacacattattcagatacgtctatacagataatgccaaacac :  53200
Seq4 : cattccaatctataatatcttgttctggtttaaaaatactcagtttgatattactaaacacacattattcagatacgtctatacagataatgccaaacac :  53200
```

```
             *     53220         *     53240         *     53260         *     53280         *     53300
Seq1 : ctggcgttggctaggtatatgcatcaaacaggagactataagcctttgtttagtcgtctcaaagagaattatatatttaccggtcccgttccaataggta :  53300
Seq2 : ctggcgttggctaggtatatgcatcaaacaggagactataagcctttgtttagtcgtctcaaagagaattatatatttaccggtcccgttccaataggta :  53300
Seq3 : ctggcgttggctaggtatatgcatcaaacaggagactataagcctttgtttagtcgtctcaaagagaattatatatttaccggtcccgttccaataggta :  53300
Seq4 : ctggcgttggctaggtatatgcatcaaacaggagactataagcctttgtttagtcgtctcaaagagaattatatatttaccggtcccgttccaataggta :  53300

             *     53320         *     53340         *     53360         *     53380         *     53400
Seq1 : tcaaagatataaatcaccctaatcttagtagagcaagaagtccatccgattatgagacattagctaatattagtactatattgtactttaccaagtatga :  53400
Seq2 : tcaaagatataaatcaccctaatcttagtagagcaagaagtccatccgattatgagacattagctaatattagtactatattgtactttaccaagtatga :  53400
Seq3 : tcaaagatataaatcaccctaatcttagtagagcaagaagtccatccgattatgagacattagctaatattagtactatattgtactttaccaagtatga :  53400
Seq4 : tcaaagatataaatcaccctaatcttagtagagcaagaagtccatccgattatgagacattagctaatattagtactatattgtactttaccaagtatga :  53400

             *     53420         *     53440         *     53460         *     53480         *     53500
Seq1 : tccggtattaatgtttttattgttttacgtacctgggtattcaattactacaaaaattactccagccgtagaatatctaatggataaactgaatctaaca :  53500
Seq2 : tccggtattaatgtttttattgttttacgtacctgggtattcaattactacaaaaattactccagccgtagaatatctaatggataaactgaatctaaca :  53500
Seq3 : tccggtattaatgtttttattgttttacgtacctgggtattcaattactacaaaaattactccagccgtagaatatctaatggataaactgaatctaaca :  53500
Seq4 : tccggtattaatgtttttattgttttacgtacctgggtattcaattactacaaaaattactccagccgtagaatatctaatggataaactgaatctaaca :  53500

             *     53520         *     53540         *     53560         *     53580         *     53600
Seq1 : aagagcgacgtacaactgttgtaaattattttatgcttcgtaaaatgtaggttttgaaccaaacattctttcaaagaatgagatgcataaaactttatta :  53600
Seq2 : aagagcgacgtacaactgttgtaaattattttatgcttcgtaaaatgtaggttttgaaccaaacattctttcaaagaatgagatgcataaaactttatta :  53600
Seq3 : aagagcgacgtacaactgttgtaaattattttatgcttcgtaaaatgtaggttttgaaccaaacattctttcaaagaatgagatgcataaaactttatta :  53600
Seq4 : aagagcgacgtacaactgttgtaaattattttatgcttcgtaaaatgtaggttttgaaccaaacattctttcaaagaatgagatgcataaaactttatta :  53600

             *     53620         *     53640         *     53660         *     53680         *     53700
Seq1 : tccaatagattgactatttcggacgtcaatcgtttaaagtaaacttcgtaaaatattctttgatcactgccgagtttaaaacttctatcgataattgttt :  53700
Seq2 : tccaatagattgactatttcggacgtcaatcgtttaaagtaaacttcgtaaaatattctttgatcactgccgagtttaaaacttctatcgataattgttt :  53700
Seq3 : tccaatagattgactatttcggacgtcaatcgtttaaagtaaacttcgtaaaatattctttgatcactgccgagtttaaaacttctatcgataattgttt :  53700
Seq4 : tccaatagattgactatttcggacgtcaatcgtttaaagtaaacttcgtaaaatattctttgatcactgccgagtttaaaacttctatcgataattgttt :  53700

             *     53720         *     53740         *     53760         *     53780         *     53800
Seq1 : catatgttttaatatttacaagtttttggtccatggtacattagccggacaaatatatgcaaaataatatcgttctccaagttctatagtttctggatt :  53800
Seq2 : catatgttttaatatttacaagtttttggtccatggtacattagccggacaaatatatgcaaaataatatcgttctccaagttctatagtttctggatt :  53800
Seq3 : catatgttttaatatttacaagtttttggtccatggtacattagccggacaaatatatgcaaaataatatcgttctccaagttctatagtttctggatt :  53800
Seq4 : catatgttttaatatttacaagtttttggtccatggtacattagccggacaaatatatgcaaaataatatcgttctccaagttctatagtttctggatt :  53800

             *     53820         *     53840         *     53860         *     53880         *     53900
Seq1 : atttttattatattcagtaaccaaatacatattagggttatctgcggatttataatttgagtgatgcattcgactcaacataaataattctagaggagac :  53900
Seq2 : atttttattatattcagtaaccaaatacatattagggttatctgcggatttataatttgagtgatgcattcgactcaacataaataattctagaggagac :  53900
Seq3 : atttttattatattcagtaaccaaatacatattagggttatctgcggatttataatttgagtgatgcattcgactcaacataaataattctagaggagac :  53900
Seq4 : atttttattatattcagtaaccaaatacatattagggttatctgcggatttataatttgagtgatgcattcgactcaacataaataattctagaggagac :  53900
```

```
                 *     53920         *     53940         *     53960         *     53980         *     54000
Seq1 : gatctactatcaaattcggatcgtaaatctgtttctaaagaacggagaatatctatacatacctgattagaattcatccgtccttcagacaacatctcag :  54000
Seq2 : gatctactatcaaattcggatcgtaaatctgtttctaaagaacggagaatatctatacatacctgattagaattcatccgtccttcagacaacatctcag :  54000
Seq3 : gatctactatcaaattcggatcgtaaatctgtttctaaagaacggagaatatctatacatacctgattagaattcatccgtccttcagacaacatctcag :  54000
Seq4 : gatctactatcaaattcggatcgtaaatctgtttctaaagaacggagaatatctatacatacctgattagaattcatccgtccttcagacaacatctcag :  54000

                 *     54020         *     54040         *     54060         *     54080         *     54100
Seq1 : acagtctggtcttgtatgtcttaatcatattcttatgaaacttggaaacatctcttctagtttcactagtacctttattaattctctcaggtacagattt :  54100
Seq2 : acagtctggtcttgtatgtcttaatcatattcttatgaaacttggaaacatctcttctagtttcactagtacctttattaattctctcaggtacagattt :  54100
Seq3 : acagtctggtcttgtatgtcttaatcatattcttatgaaacttggaaacatctcttctagtttcactagtacctttattaattctctcaggtacagattt :  54100
Seq4 : acagtctggtcttgtatgtcttaatcatattcttatgaaacttggaaacatctcttctagtttcactagtacctttattaattctctcaggtacagattt :  54100

                 *     54120         *     54140         *     54160         *     54180         *     54200
Seq1 : tgaattcgacgatgccgagtatttcatcgttgtatatttcttcttcgattgcataatcagattcttatataccgcctcaaactctatttaaaattatta :  54200
Seq2 : tgaattcgacgatgccgagtatttcatcgttgtatatttcttcttcgattgcataatcagattcttatataccgcctcaaactctatttaaaattatta :  54200
Seq3 : tgaattcgacgatgccgagtatttcatcgttgtatatttcttcttcgattgcataatcagattcttatataccgcctcaaactctatttaaaattatta :  54200
Seq4 : tgaattcgacgatgccgagtatttcatcgttgtatatttcttcttcgattgcataatcagattcttatataccgcctcaaactctatttaaaattatta :  54200

                 *     54220         *     54240         *     54260         *     54280         *     54300
Seq1 : aacaatactctattattaatcagtcgttctaactcctttgctatttctatggacttatctacatcttgactgtctatctctgtaaacacggagtcggtat :  54300
Seq2 : aacaatactctattattaatcagtcgttctaactcctttgctatttctatggacttatctacatcttgactgtctatctctgtaaacacggagtcggtat :  54300
Seq3 : aacaatactctattattaatcagtcgttctaactcctttgctatttctatggacttatctacatcttgactgtctatctctgtaaacacggagtcggtat :  54300
Seq4 : aacaatactctattattaatcagtcgttctaactcctttgctatttctatggacttatctacatcttgactgtctatctctgtaaacacggagtcggtat :  54300

                 *     54320         *     54340         *     54360         *     54380         *     54400
Seq1 : ctccatacacgctacgaaaacgaaatctgtaatctataggcaacgatgttttcacaatcggattaatatctctatcgtccatataaaatggattacttaa :  54400
Seq2 : ctccatacacgctacgaaaacgaaatctgtaatctataggcaacgatgttttcacaatcggattaatatctctatcgtccatataaaatggattacttaa :  54400
Seq3 : ctccatacacgctacgaaaacgaaatctgtaatctataggcaacgatgttttcacaatcggattaatatctctatcgtccatataaaatggattacttaa :  54400
Seq4 : ctccatacacgctacgaaaacgaaatctgtaatctataggcaacgatgttttcacaatcggattaatatctctatcgtccatataaaatggattacttaa :  54400

                 *     54420         *     54440         *     54460         *     54480         *     54500
Seq1 : tggattggcaaaccgtaacataccgttagataactctgctccatttagtaccgattctagatacaagatcattctacgtcctatggatgtgcaactctta :  54500
Seq2 : tggattggcaaaccgtaacataccgttagataactctgctccatttagtaccgattctagatacaagatcattctacgtcctatggatgtgcaactctta :  54500
Seq3 : tggattggcaaaccgtaacataccgttagataactctgctccatttagtaccgattctagatacaagatcattctacgtcctatggatgtgcaactctta :  54500
Seq4 : tggattggcaaaccgtaacataccgttagataactctgctccatttagtaccgattctagatacaagatcattctacgtcctatggatgtgcaactctta :  54500

                 *     54520         *     54540         *     54560         *     54580         *     54600
Seq1 : gccgaagcgtatgagtatagagcactatttctaaatcccatcagaccatatactgagttggctactatcttgtacgtatattgcatggaatcatagatgg :  54600
Seq2 : gccgaagcgtatgagtatagagcactatttctaaatcccatcagaccatatactgagttggctactatcttgtacgtatattgcatggaatcatagatgg :  54600
Seq3 : gccgaagcgtatgagtatagagcactatttctaaatcccatcagaccatatactgagttggctactatcttgtacgtatattgcatggaatcatagatgg :  54600
Seq4 : gccgaagcgtatgagtatagagcactatttctaaatcccatcagaccatatactgagttggctactatcttgtacgtatattgcatggaatcatagatgg :  54600
```

```
               *     54620          *     54640          *     54660          *     54680          *     54700
Seq1 : ccttttcagttgaactggtagcctgttttaacatctttttatatctggctctctctgccaaaaatgttcttaatagtctaggaatggttccttctatcga :  54700
Seq2 : ccttttcagttgaactggtagcctgttttaacatctttttatatctggctctctctgccaaaaatgttcttaatagtctaggaatggttccttctatcga :  54700
Seq3 : ccttttcagttgaactggtagcctgttttaacatctttttatatctggctctctctgccaaaaatgttcttaatagtctaggaatggttccttctatcga :  54700
Seq4 : ccttttcagttgaactggtagcctgttttaacatctttttatatctggctctctctgccaaaaatgttcttaatagtctaggaatggttccttctatcga :  54700

               *     54720          *     54740          *     54760          *     54780          *     54800
Seq1 : tctatcgaaaattgctatttcagagatgaggttcggtagtctaggttcacaatgaaccgtaatatatctaggaggtggatatttctgaagcaagagctga :  54800
Seq2 : tctatcgaaaattgctatttcagagatgaggttcggtagtctaggttcacaatgaaccgtaatatatctaggaggtggatatttctgaagcaagagctga :  54800
Seq3 : tctatcgaaaattgctatttcagagatgaggttcggtagtctaggttcacaatgaaccgtaatatatctaggaggtggatatttctgaagcaagagctga :  54800
Seq4 : tctatcgaaaattgctatttcagagatgaggttcggtagtctaggttcacaatgaaccgtaatatatctaggaggtggatatttctgaagcaagagctga :  54800

               *     54820          *     54840          *     54860          *     54880          *     54900
Seq1 : ttatttatttcttcttccaatctattggtactaacaacgacaccgactaatgtttccggagatagatttccaaagatacacacattaggatacagactgt :  54900
Seq2 : ttatttatttcttcttccaatctattggtactaacaacgacaccgactaatgtttccggagatagatttccaaagatacacacattaggatacagactgt :  54900
Seq3 : ttatttatttcttcttccaatctattggtactaacaacgacaccgactaatgtttccggagatagatttccaaagatacacacattaggatacagactgt :  54900
Seq4 : ttatttatttcttcttccaatctattggtactaacaacgacaccgactaatgtttccggagatagatttccaaagatacacacattaggatacagactgt :  54900

               *     54920          *     54940          *     54960          *     54980          *     55000
Seq1 : tataatcaaagattaatacattattactaaacatttttttgttttggagcaaataccttaccgccttcataaggaaacttttgttttgtttctgatctaac :  55000
Seq2 : tataatcaaagattaatacattattactaaacatttttttgttttggagcaaataccttaccgccttcataaggaaacttttgttttgtttctgatctaac :  55000
Seq3 : tataatcaaagattaatacattattactaaacatttttttgttttggagcaaataccttaccgccttcataaggaaacttttgttttgtttctgatctaac :  55000
Seq4 : tataatcaaagattaatacattattactaaacatttttttgttttggagcaaataccttaccgccttcataaggaaacttttgttttgtttctgatctaac :  55000

               *     55020          *     55040          *     55060          *     55080          *     55100
Seq1 : taagatagttttagtttccaacaatagctttaacagtggacccttgatgactgtactcgctctatattcgaataccatggattgaggaagcacatatgtt :  55100
Seq2 : taagatagttttagtttccaacaatagctttaacagtggacccttgatgactgtactcgctctatattcgaataccatggattgaggaagcacatatgtt :  55100
Seq3 : taagatagttttagtttccaacaatagctttaacagtggacccttgatgactgtactcgctctatattcgaataccatggattgaggaagcacatatgtt :  55100
Seq4 : taagatagttttagtttccaacaatagctttaacagtggacccttgatgactgtactcgctctatattcgaataccatggattgaggaagcacatatgtt :  55100

               *     55120          *     55140          *     55160          *     55180          *     55200
Seq1 : gacgcacccgcgtctgtttttgtttctactccataatactcccacaaatactgacacaaacaagcatcatgaatacagtatctagccatatctaaagcta :  55200
Seq2 : gacgcacccgcgtctgtttttgtttctactccataatactcccacaaatactgacacaaacaagcatcatgaatacagtatctagccatatctaaagcta :  55200
Seq3 : gacgcacccgcgtctgtttttgtttctactccataatactcccacaaatactgacacaaacaagcatcatgaatacagtatctagccatatctaaagcta :  55200
Seq4 : gacgcacccgcgtctgtttttgtttctactccataatactcccacaaatactgacacaaacaagcatcatgaatacagtatctagccatatctaaagcta :  55200

               *     55220          *     55240          *     55260          *     55280          *     55300
Seq1 : tgtttagattataatccttatacatctgagctaaatcaacgtcatcctttccgaaagataatttatatgtatcattaggtaaagtaggacataatagtac :  55300
Seq2 : tgtttagattataatccttatacatctgagctaaatcaacgtcatcctttccgaaagataatttatatgtatcattaggtaaagtaggacataatagtac :  55300
Seq3 : tgtttagattataatccttatacatctgagctaaatcaacgtcatcctttccgaaagataatttatatgtatcattaggtaaagtaggacataatagtac :  55300
Seq4 : tgtttagattataatccttatacatctgagctaaatcaacgtcatcctttccgaaagataatttatatgtatcattaggtaaagtaggacataatagtac :  55300
```

```
             *     55320          *     55340          *     55360          *     55380          *     55400
Seq1 : gactttaaatccattttcccaaatatctttacgaattactttacatataatatcctcatcaacagtcacataattacctgtggttaaaacctttgcaaat :  55400
Seq2 : gactttaaatccattttcccaaatatctttacgaattactttacatataatatcctcatcaacagtcacataattacctgtggttaaaacctttgcaaat :  55400
Seq3 : gactttaaatccattttcccaaatatctttacgaattactttacatataatatcctcatcaacagtcacataattacctgtggttaaaacctttgcaaat :  55400
Seq4 : gactttaaatccattttcccaaatatctttacgaattactttacatataatatcctcatcaacagtcacataattacctgtggttaaaacctttgcaaat :  55400

             *     55420          *     55440          *     55460          *     55480          *     55500
Seq1 : gcagcggctttgcctttcgcgtccgtagtatcgtcaccgatgaacgtcatttctctaactcctctatttaatactttacccatgcaactgaacgcgttct :  55500
Seq2 : gcagcggctttgcctttcgcgtccgtagtatcgtcaccgatgaacgtcatttctctaactcctctatttaatactttacccatgcaactgaacgcgttct :  55500
Seq3 : gcagcggctttgcctttcgcgtccgtagtatcgtcaccgatgaacgtcatttctctaactcctctatttaatactttacccatgcaactgaacgcgttct :  55500
Seq4 : gcagcggctttgcctttcgcgtccgtagtatcgtcaccgatgaacgtcatttctctaactcctctatttaatactttacccatgcaactgaacgcgttct :  55500

             *     55520          *     55540          *     55560          *     55580          *     55600
Seq1 : tggatatagaatccaatttgtacgaatccaatttttcagattttgaatgaatgaatatagatcgaaaaatatagttccattattgttattaacgtgaaa :  55600
Seq2 : tggatatagaatccaatttgtacgaatccaatttttcagattttgaatgaatgaatatagatcgaaaaatatagttccattattgttattaacgtgaaa :  55600
Seq3 : tggatatagaatccaatttgtacgaatccaatttttcagattttgaatgaatgaatatagatcgaaaaatatagttccattattgttattaacgtgaaa :  55600
Seq4 : tggatatagaatccaatttgtacgaatccaatttttcagattttgaatgaatgaatatagatcgaaaaatatagttccattattgttattaacgtgaaa :  55600

             *     55620          *     55640          *     55660          *     55680          *     55700
Seq1 : cgtagtattggccatgccgcctactcccttatgactagactgatttctctcataaatacagagatgtacagcttcctttttgtccggagatctaaagata :  55700
Seq2 : cgtagtattggccatgccgcctactcccttatgactagactgatttctctcataaatacagagatgtacagcttcctttttgtccggagatctaaagata :  55700
Seq3 : cgtagtattggccatgccgcctactcccttatgactagactgatttctctcataaatacagagatgtacagcttcctttttgtccggagatctaaagata :  55700
Seq4 : cgtagtattggccatgccgcctactcccttatgactagactgatttctctcataaatacagagatgtacagcttcctttttgtccggagatctaaagata :  55700

             *     55720          *     55740          *     55760          *     55780          *     55800
Seq1 : atcttctctcctgttaataactctagacgattagtaatatatctcagatcaaagttatgtccgttaaaggtaacgacatagtcgaacgttagttccaaca :  55800
Seq2 : atcttctctcctgttaataactctagacgattagtaatatatctcagatcaaagttatgtccgttaaaggtaacgacatagtcgaacgttagttccaaca :  55800
Seq3 : atcttctctcctgttaataactctagacgattagtaatatatctcagatcaaagttatgtccgttaaaggtaacgacatagtcgaacgttagttccaaca :  55800
Seq4 : atcttctctcctgttaataactctagacgattagtaatatatctcagatcaaagttatgtccgttaaaggtaacgacatagtcgaacgttagttccaaca :  55800

             *     55820          *     55840          *     55860          *     55880          *     55900
Seq1 : attgtttagctattcgtaacaaaactatttcagaacataaaactagttctcgttcgtaatccatttccattagtgactgtatcctcaaacatcctctatc :  55900
Seq2 : attgtttagctattcgtaacaaaactatttcagaacataaaactagttctcgttcgtaatccatttccattagtgactgtatcctcaaacatcctctatc :  55900
Seq3 : attgtttagctattcgtaacaaaactatttcagaacataaaactagttctcgttcgtaatccatttccattagtgactgtatcctcaaacatcctctatc :  55900
Seq4 : attgtttagctattcgtaacaaaactatttcagaacataaaactagttctcgttcgtaatccatttccattagtgactgtatcctcaaacatcctctatc :  55900

             *     55920          *     55940          *     55960          *     55980          *     56000
Seq1 : gacggcttcttgtatttcctgttccgttaacatctcttcattaatgagcgtaaacaataatcgtttaccacttaaatcgatataacagtaacttgtatgc :  56000
Seq2 : gacggcttcttgtatttcctgttccgttaacatctcttcattaatgagcgtaaacaataatcgtttaccacttaaatcgatataacagtaacttgtatgc :  56000
Seq3 : gacggcttcttgtatttcctgttccgttaacatctcttcattaatgagcgtaaacaataatcgtttaccacttaaatcgatataacagtaacttgtatgc :  56000
Seq4 : gacggcttcttgtatttcctgttccgttaacatctcttcattaatgagcgtaaacaataatcgtttaccacttaaatcgatataacagtaacttgtatgc :  56000
```

```
                 *      56020          *      56040          *      56060          *      56080          *      56100
Seq1 : gagattgggttaataaatacagaaggaaacttcttatcgaagtgacactctatatctagaaataagtacgatcttgggatatcgaatctaggtatttttt :  56100
Seq2 : gagattgggttaataaatacagaaggaaacttcttatcgaagtgacactctatatctagaaataagtacgatcttgggatatcgaatctaggtatttttt :  56100
Seq3 : gagattgggttaataaatacagaaggaaacttcttatcgaagtgacactctatatctagaaataagtacgatcttgggatatcgaatctaggtatttttt :  56100
Seq4 : gagattgggttaataaatacagaaggaaacttcttatcgaagtgacactctatatctagaaataagtacgatcttgggatatcgaatctaggtatttttt :  56100

                 *      56120          *      56140          *      56160          *      56180          *      56200
Seq1 : tagcgaaacagttacgtggatcgtcacaatgataacatccattgttaatctttgtcaaatattgctcgtccaacgagtaacatccgtctggagatatccc :  56200
Seq2 : tagcgaaacagttacgtggatcgtcacaatgataacatccattgttaatctttgtcaaatattgctcgtccaacgagtaacatccgtctggagatatccc :  56200
Seq3 : tagcgaaacagttacgtggatcgtcacaatgataacatccattgttaatctttgtcaaatattgctcgtccaacgagtaacatccgtctggagatatccc :  56200
Seq4 : tagcgaaacagttacgtggatcgtcacaatgataacatccattgttaatctttgtcaaatattgctcgtccaacgagtaacatccgtctggagatatccc :  56200

                 *      56220          *      56240          *      56260          *      56280          *      56300
Seq1 : gttagaaatataaaaccaactaatattgagaaattcatccatggtggcattttgtatgctgcgtttctttggctcttctatcaaccacatatctgcgacg :  56300
Seq2 : gttagaaatataaaaccaactaatattgagaaattcatccatggtggcattttgtatgctgcgtttctttggctcttctatcaaccacatatctgcgacg :  56300
Seq3 : gttagaaatataaaaccaactaatattgagaaattcatccatggtggcattttgtatgctgcgtttctttggctcttctatcaaccacatatctgcgacg :  56300
Seq4 : gttagaaatataaaaccaactaatattgagaaattcatccatggtggcattttgtatgctgcgtttctttggctcttctatcaaccacatatctgcgacg :  56300

                 *      56320          *      56340          *      56360          *      56380          *      56400
Seq1 : gagcattttctatctttaatatctagattataacttattgtctcgtcaatgtctatagttctcatctttcccaacggcctcgcattaaatggaggaggag :  56400
Seq2 : gagcattttctatctttaatatctagattataacttattgtctcgtcaatgtctatagttctcatctttcccaacggcctcgcattaaatggaggaggag :  56400
Seq3 : gagcattttctatctttaatatctagattataacttattgtctcgtcaatgtctatagttctcatctttcccaacggcctcgcattaaatggaggaggag :  56400
Seq4 : gagcattttctatctttaatatctagattataacttattgtctcgtcaatgtctatagttctcatctttcccaacggcctcgcattaaatggaggaggag :  56400

                 *      56420          *      56440          *      56460          *      56480          *      56500
Seq1 : acaatgactgatatatttcgtccgtcactacgtaataaaagtaatgaggaaatcgtataaatacggtctcaccatttcgacatctggatttcagatataa :  56500
Seq2 : acaatgactgatatatttcgtccgtcactacgtaataaaagtaatgaggaaatcgtataaatacggtctcaccatttcgacatctggatttcagatataa :  56500
Seq3 : acaatgactgatatatttcgtccgtcactacgtaataaaagtaatgaggaaatcgtataaatacggtctcaccatttcgacatctggatttcagatataa :  56500
Seq4 : acaatgactgatatatttcgtccgtcactacgtaataaaagtaatgaggaaatcgtataaatacggtctcaccatttcgacatctggatttcagatataa :  56500

                 *      56520          *      56540          *      56560          *      56580          *      56600
Seq1 : aaatctgttttcaccgtgactttcaaaccaattaatgcaccgaacatccatttatagaatttagaaatatattttcatttaaatgaatcccaaacattgg :  56600
Seq2 : aaatctgttttcaccgtgactttcaaaccaattaatgcaccgaacatccatttatagaatttagaaatatattttcatttaaatgaatcccaaacattgg :  56600
Seq3 : aaatctgttttcaccgtgactttcaaaccaattaatgcaccgaacatccatttatagaatttagaaatatattttcatttaaatgaatcccaaacattgg :  56600
Seq4 : aaatctgttttcaccgtgactttcaaaccaattaatgcaccgaacatccatttatagaatttagaaatatattttcatttaaatgaatcccaaacattgg :  56600

                 *      56620          *      56640          *      56660          *      56680          *      56700
Seq1 : ggaagagccgtatggaccattatttttatagtactttcgcaagcgggtttagacggcaacatagaagcgtgtaaacgaaaactatatactatagttagca :  56700
Seq2 : ggaagagccgtatggaccattatttttatagtactttcgcaagcgggtttagacggcaacatagaagcgtgtaaacgaaaactatatactatagttagca :  56700
Seq3 : ggaagagccgtatggaccattatttttatagtactttcgcaagcgggtttagacggcaacatagaagcgtgtaaacgaaaactatatactatagttagca :  56700
Seq4 : ggaagagccgtatggaccattatttttatagtactttcgcaagcgggtttagacggcaacatagaagcgtgtaaacgaaaactatatactatagttagca :  56700
```

* 56720 * 56740 * 56760 * 56780 * 56800
Seq1 : ctcttccatgtcctgcatgtagacggcacgcgactatcgctatagaggacaataatgtcatgtctagcgatgatctgaattatatttattattttttcat : 56800
Seq2 : ctcttccatgtcctgcatgtagacggcacgcgactatcgctatagaggacaataatgtcatgtctagcgatgatctgaattatatttattattttttcat : 56800
Seq3 : ctcttccatgtcctgcatgtagacggcacgcgactatcgctatagaggacaataatgtcatgtctagcgatgatctgaattatatttattattttttcat : 56800
Seq4 : ctcttccatgtcctgcatgtagacggcacgcgactatcgctatagaggacaataatgtcatgtctagcgatgatctgaattatatttattattttttcat : 56800

* 56820 * 56840 * 56860 * 56880 * 56900
Seq1 : cagattatttaacaatttggcatctgatcccaaatacgcgatcgatgtgacaaaggttaaccctttataaacttaacccattataaaacttatgattagt : 56900
Seq2 : cagattatttaacaatttggcatctgatcccaaatacgcgatcgatgtgacaaaggttaaccctttataaacttaacccattataaaacttatgattagt : 56900
Seq3 : cagattatttaacaatttggcatctgatcccaaatacgcgatcgatgtgacaaaggttaaccctttataaacttaacccattataaaacttatgattagt : 56900
Seq4 : cagattatttaacaatttggcatctgatcccaaatacgcgatcgatgtgacaaaggttaaccctttataaacttaacccattataaaacttatgattagt : 56900

* 56920 * 56940 * 56960 * 56980 * 57000
Seq1 : cacgactgaaataaccgcgtgattattttttggtataattctacacggcatggtttctgtgactatgaattcaacccccgttacattagtgaaatcttta : 57000
Seq2 : cacgactgaaataaccgcgtgattattttttggtataattctacacggcatggtttctgtgactatgaattcaacccccgttacattagtgaaatcttta : 57000
Seq3 : cacgactgaaataaccgcgtgattattttttggtataattctacacggcatggtttctgtgactatgaattcaacccccgttacattagtgaaatcttta : 57000
Seq4 : cacgactgaaataaccgcgtgattattttttggtataattctacacggcatggtttctgtgactatgaattcaacccccgttacattagtgaaatcttta : 57000

* 57020 * 57040 * 57060 * 57080 * 57100
Seq1 : acaaacagcaagggttcgtcaaagacataaaactcattgtttacaatcgaaatagacccccctatcacacttaaaataaaaaatatccttatcctttacca : 57100
Seq2 : acaaacagcaagggttcgtcaaagacataaaactcattgtttacaatcgaaatagacccccctatcacacttaaaataaaaaatatccttatcctttacca : 57100
Seq3 : acaaacagcaagggttcgtcaaagacataaaactcattgtttacaatcgaaatagacccccctatcacacttaaaataaaaaatatccttatcctttacca : 57100
Seq4 : acaaacagcaagggttcgtcaaagacataaaactcattgtttacaatcgaaatagacccccctatcacacttaaaataaaaaatatccttatcctttacca : 57100

* 57120 * 57140 * 57160 * 57180 * 57200
Seq1 : ccaaataaaattctgattggtcaatgtgaatgtattcacttaacagttccacaaatttatttattaactccgaggcacatacatcgtcggtattttttat : 57200
Seq2 : ccaaataaaattctgattggtcaatgtgaatgtattcacttaacagttccacaaatttatttattaactccgaggcacatacatcgtcggtattttttat : 57200
Seq3 : ccaaataaaattctgattggtcaatgtgaatgtattcacttaacagttccacaaatttatttattaactccgaggcacatacatcgtcggtattttttat : 57200
Seq4 : ccaaataaaattctgattggtcaatgtgaatgtattcacttaacagttccacaaatttatttattaactccgaggcacatacatcgtcggtattttttat : 57200

* 57220 * 57240 * 57260 * 57280 * 57300
Seq1 : ggcaaactttactcttccagcatccgtttctaaaaaaatattaacgagttccatttatatcatccaatattattgaaatgacgttgatggacaaatgata : 57300
Seq2 : ggcaaactttactcttccagcatccgtttctaaaaaaatattaacgagttccatttatatcatccaatattattgaaatgacgttgatggacaaatgata : 57300
Seq3 : ggcaaactttactcttccagcatccgtttctaaaaaaatattaacgagttccatttatatcatccaatattattgaaatgacgttgatggacaaatgata : 57300
Seq4 : ggcaaactttactcttccagcatccgtttctaaaaaaatattaacgagttccatttatatcatccaatattattgaaatgacgttgatggacaaatgata : 57300

* 57320 * 57340 * 57360 * 57380 * 57400
Seq1 : caaataagaaggtacggtacctttgtccaccatctcctccaattcatgctctatttgtcattaactttaatgtatgaaaacagtacgccacatgcttcc : 57400
Seq2 : caaataagaaggtacggtacctttgtccaccatctcctccaattcatgctctatttgtcattaactttaatgtatgaaaacagtacgccacatgcttcc : 57400
Seq3 : caaataagaaggtacggtacctttgtccaccatctcctccaattcatgctctatttgtcattaactttaatgtatgaaaacagtacgccacatgcttcc : 57400
Seq4 : caaataagaaggtacggtacctttgtccaccatctcctccaattcatgctctatttgtcattaactttaatgtatgaaaacagtacgccacatgcttcc : 57400

```
                  *     57420         *     57440         *     57460         *     57480         *     57500
Seq1 : atgacagtgtgtaacactttggatacaaaatgtttgacattagtataattgttcaagactgtcaatctataatagatagtagctataatatattctatga :  57500
Seq2 : atgacagtgtgtaacactttggatacaaaatgtttgacattagtataattgttcaagactgtcaatctataatagatagtagctataatatattctatga :  57500
Seq3 : atgacagtgtgtaacactttggatacaaaatgtttgacattagtataattgttcaagactgtcaatctataatagatagtagctataatatattctatga :  57500
Seq4 : atgacagtgtgtaacactttggatacaaaatgtttgacattagtataattgttcaagactgtcaatctataatagatagtagctataatatattctatga :  57500

                  *     57520         *     57540         *     57560         *     57580         *     57600
Seq1 : tggtattgaagaagatgacaaccttggcatattgatcatttaacacagacatggtatcaacagatagcttgaatgaaagagaatcagtaattggaataag :  57600
Seq2 : tggtattgaagaagatgacaaccttggcatattgatcatttaacacagacatggtatcaacagatagcttgaatgaaagagaatcagtaattggaataag :  57600
Seq3 : tggtattgaagaagatgacaaccttggcatattgatcatttaacacagacatggtatcaacagatagcttgaatgaaagagaatcagtaattggaataag :  57600
Seq4 : tggtattgaagaagatgacaaccttggcatattgatcatttaacacagacatggtatcaacagatagcttgaatgaaagagaatcagtaattggaataag :  57600

                  *     57620         *     57640         *     57660         *     57680         *     57700
Seq1 : cgtcttctcgatggagtgtccgtataccaacatgtctgatattttgatgtattccattaaattatttagttttttctttttattctcgttaaacagcatt :  57700
Seq2 : cgtcttctcgatggagtgtccgtataccaacatgtctgatattttgatgtattccattaaattatttagttttttctttttattctcgttaaacagcatt :  57700
Seq3 : cgtcttctcgatggagtgtccgtataccaacatgtctgatattttgatgtattccattaaattatttagttttttctttttattctcgttaaacagcatt :  57700
Seq4 : cgtcttctcgatggagtgtccgtataccaacatgtctgatattttgatgtattccattaaattatttagttttttctttttattctcgttaaacagcatt :  57700

                  *     57720         *     57740         *     57760         *     57780         *     57800
Seq1 : tctgtcaacggaccccaacatcgttgaccgattaagttttgattgatttttccgtgtaaggcgtatctagtcagatcgtatagcctatccaataatccat :  57800
Seq2 : tctgtcaacggaccccaacatcgttgaccgattaagttttgattgatttttccgtgtaaggcgtatctagtcagatcgtatagcctatccaataatccat :  57800
Seq3 : tctgtcaacggaccccaacatcgttgaccgattaagttttgattgatttttccgtgtaaggcgtatctagtcagatcgtatagcctatccaataatccat :  57800
Seq4 : tctgtcaacggaccccaacatcgttgaccgattaagttttgattgatttttccgtgtaaggcgtatctagtcagatcgtatagcctatccaataatccat :  57800

                  *     57820         *     57840         *     57860         *     57880         *     57900
Seq1 : cgtctgtgtgtagatcacatcgtacactttttaattctctatagaagagcgacagacatctggagcaattacagacagcaatttctttattctctacaga :  57900
Seq2 : cgtctgtgtgtagatcacatcgtacactttttaattctctatagaagagcgacagacatctggagcaattacagacagcaatttctttattctctacaga :  57900
Seq3 : cgtctgtgtgtagatcacatcgtacactttttaattctctatagaagagcgacagacatctggagcaattacagacagcaatttctttattctctacaga :  57900
Seq4 : cgtctgtgtgtagatcacatcgtacactttttaattctctatagaagagcgacagacatctggagcaattacagacagcaatttctttattctctacaga :  57900

                  *     57920         *     57940         *     57960         *     57980         *     58000
Seq1 : tgtaagatacttgaagacattcctatgatgatgcagaattttggataacacggtattgatggtatctgttaccataattcctttgatggctgatagtgtc :  58000
Seq2 : tgtaagatacttgaagacattcctatgatgatgcagaattttggataacacggtattgatggtatctgttaccataattcctttgatggctgatagtgtc :  58000
Seq3 : tgtaagatacttgaagacattcctatgatgatgcagaattttggataacacggtattgatggtatctgttaccataattcctttgatggctgatagtgtc :  58000
Seq4 : tgtaagatacttgaagacattcctatgatgatgcagaattttggataacacggtattgatggtatctgttaccataattcctttgatggctgatagtgtc :  58000

                  *     58020         *     58040         *     58060         *     58080         *     58100
Seq1 : agagcacaagatttccaatctttgacaatttttagcaccattatctttgttttgatatctatatcagacagcatggtgcgtctgacaacacaaggattaa :  58100
Seq2 : agagcacaagatttccaatctttgacaatttttagcaccattatctttgttttgatatctatatcagacagcatggtgcgtctgacaacacaaggattaa :  58100
Seq3 : agagcacaagatttccaatctttgacaatttttagcaccattatctttgttttgatatctatatcagacagcatggtgcgtctgacaacacaaggattaa :  58100
Seq4 : agagcacaagatttccaatctttgacaatttttagcaccattatctttgttttgatatctatatcagacagcatggtgcgtctgacaacacaaggattaa :  58100
```

```
                *     58120         *     58140         *     58160         *     58180         *     58200
Seq1 : gacggaaagatgaaatgattctctcaacatcttcaatggataccttgctattttttctggcattatctatatgtgcgagaatatcctctagagaatcagt :  58200
Seq2 : gacggaaagatgaaatgattctctcaacatcttcaatggataccttgctattttttctggcattatctatatgtgcgagaatatcctctagagaatcagt :  58200
Seq3 : gacggaaagatgaaatgattctctcaacatcttcaatggataccttgctattttttctggcattatctatatgtgcgagaatatcctctagagaatcagt :  58200
Seq4 : gacggaaagatgaaatgattctctcaacatcttcaatggataccttgctattttttctggcattatctatatgtgcgagaatatcctctagagaatcagt :  58200

                *     58220         *     58240         *     58260         *     58280         *     58300
Seq1 : atcctttttgatgatagtggatctcaatgacatgggacgtctaaaccttcttattctatcaccagattgcatggtgatttgtcttctttcttttatcata :  58300
Seq2 : atcctttttgatgatagtggatctcaatgacatgggacgtctaaaccttcttattctatcaccagattgcatggtgatttgtcttctttcttttatcata :  58300
Seq3 : atcctttttgatgatagtggatctcaatgacatgggacgtctaaaccttcttattctatcaccagattgcatggtgatttgtcttctttcttttatcata :  58300
Seq4 : atcctttttgatgatagtggatctcaatgacatgggacgtctaaaccttcttattctatcaccagattgcatggtgatttgtcttctttcttttatcata :  58300

                *     58320         *     58340         *     58360         *     58380         *     58400
Seq1 : atgtaatctctaaattcatcggcaaattgtctatatctaaaatcataatatgagatgtttacctctacaaatatctgttcgtccaatgttagagtattta :  58400
Seq2 : atgtaatctctaaattcatcggcaaattgtctatatctaaaatcataatatgagatgtttacctctacaaatatctgttcgtccaatgttagagtattta :  58400
Seq3 : atgtaatctctaaattcatcggcaaattgtctatatctaaaatcataatatgagatgtttacctctacaaatatctgttcgtccaatgttagagtattta :  58400
Seq4 : atgtaatctctaaattcatcggcaaattgtctatatctaaaatcataatatgagatgtttacctctacaaatatctgttcgtccaatgttagagtattta :  58400

                *     58420         *     58440         *     58460         *     58480         *     58500
Seq1 : catcagttttgtattccaaattaaacatggcaacggatttaattttatattcctctattaagtcctcgtcgataataacagaatgtagataatcatttaa :  58500
Seq2 : catcagttttgtattccaaattaaacatggcaacggatttaattttatattcctctattaagtcctcgtcgataataacagaatgtagataatcatttaa :  58500
Seq3 : catcagttttgtattccaaattaaacatggcaacggatttaattttatattcctctattaagtcctcgtcgataataacagaatgtagataatcatttaa :  58500
Seq4 : catcagttttgtattccaaattaaacatggcaacggatttaattttatattcctctattaagtcctcgtcgataataacagaatgtagataatcatttaa :  58500

                *     58520         *     58540         *     58560         *     58580         *     58600
Seq1 : tccatcgtacatggttggaagatgctcgttgacaaaatctttaattgtcttgatgaaggtgggactatatctaacatcttgattaataaaatttataaca :  58600
Seq2 : tccatcgtacatggttggaagatgctcgttgacaaaatctttaattgtcttgatgaaggtgggactatatctaacatcttgattaataaaatttataaca :  58600
Seq3 : tccatcgtacatggttggaagatgctcgttgacaaaatctttaattgtcttgatgaaggtgggactatatctaacatcttgattaataaaatttataaca :  58600
Seq4 : tccatcgtacatggttggaagatgctcgttgacaaaatctttaattgtcttgatgaaggtgggactatatctaacatcttgattaataaaatttataaca :  58600

                *     58620         *     58640         *     58660         *     58680         *     58700
Seq1 : ttgtccataggatactttgtaactagttttatacacatctcttcatcggtaagtttagacagaatatcgtgaacaggtggtatattatattcatcagata :  58700
Seq2 : ttgtccataggatactttgtaactagttttatacacatctcttcatcggtaagtttagacagaatatcgtgaacaggtggtatattatattcatcagata :  58700
Seq3 : ttgtccataggatactttgtaactagttttatacacatctcttcatcggtaagtttagacagaatatcgtgaacaggtggtatattatattcatcagata :  58700
Seq4 : ttgtccataggatactttgtaactagttttatacacatctcttcatcggtaagtttagacagaatatcgtgaacaggtggtatattatattcatcagata :  58700

                *     58720         *     58740         *     58760         *     58780         *     58800
Seq1 : tacgaagaacaatgtccaaatctatattgtttaatatattatatagatgtagtgtagctcctacaggaatatctttaactaagtcaatgatttcatcaac :  58800
Seq2 : tacgaagaacaatgtccaaatctatattgtttaatatattatatagatgtagtgtagctcctacaggaatatctttaactaagtcaatgatttcatcaac :  58800
Seq3 : tacgaagaacaatgtccaaatctatattgtttaatatattatatagatgtagtgtagctcctacaggaatatctttaactaagtcaatgatttcatcaac :  58800
Seq4 : tacgaagaacaatgtccaaatctatattgtttaatatattatatagatgtagtgtagctcctacaggaatatctttaactaagtcaatgatttcatcaac :  58800
```

```
                 *     58820         *     58840         *     58860         *     58880         *     58900
Seq1 : cgttagatctattttaaagttaatcatataggcattgatttttaaaaggtatgtagccttgactacattctcattaattaaccattccaagtcactgtgt :  58900
Seq2 : cgttagatctattttaaagttaatcatataggcattgatttttaaaaggtatgtagccttgactacattctcattaattaaccattccaagtcactgtgt :  58900
Seq3 : cgttagatctattttaaagttaatcatataggcattgatttttaaaaggtatgtagccttgactacattctcattaattaaccattccaagtcactgtgt :  58900
Seq4 : cgttagatctattttaaagttaatcatataggcattgatttttaaaaggtatgtagccttgactacattctcattaattaaccattccaagtcactgtgt :  58900

                 *     58920         *     58940         *     58960         *     58980         *     59000
Seq1 : gtaagaagattatattctatcataagcttgactacatttggtcccgataccattaaagaattcttatgatataaggaaacagattttaggtactcatcta :  59000
Seq2 : gtaagaagattatattctatcataagcttgactacatttggtcccgataccattaaagaattcttatgatataaggaaacagattttaggtactcatcta :  59000
Seq3 : gtaagaagattatattctatcataagcttgactacatttggtcccgataccattaaagaattcttatgatataaggaaacagattttaggtactcatcta :  59000
Seq4 : gtaagaagattatattctatcataagcttgactacatttggtcccgataccattaaagaattcttatgatataaggaaacagattttaggtactcatcta :  59000

                 *     59020         *     59040         *     59060         *     59080         *     59100
Seq1 : ctctacaagaattttggagagccttaacgatatcagtgacgtttattatttcaggaggaaaaaacctaacattgagaatatcggaattaatagcttccag :  59100
Seq2 : ctctacaagaattttggagagccttaacgatatcagtgacgtttattatttcaggaggaaaaaacctaacattgagaatatcggaattaatagcttccag :  59100
Seq3 : ctctacaagaattttggagagccttaacgatatcagtgacgtttattatttcaggaggaaaaaacctaacattgagaatatcggaattaatagcttccag :  59100
Seq4 : ctctacaagaattttggagagccttaacgatatcagtgacgtttattatttcaggaggaaaaaacctaacattgagaatatcggaattaatagcttccag :  59100

                 *     59120         *     59140         *     59160         *     59180         *     59200
Seq1 : atacagtgattttggcaatagtccgtgtaatccataatccagtaacacgagctggtgcttgctagacaccttttcaatgtttaatttttttgaaataagc :  59200
Seq2 : atacagtgattttggcaatagtccgtgtaatccataatccagtaacacgagctggtgcttgctagacaccttttcaatgtttaatttttttgaaataagc :  59200
Seq3 : atacagtgattttggcaatagtccgtgtaatccataatccagtaacacgagctggtgcttgctagacaccttttcaatgtttaatttttttgaaataagc :  59200
Seq4 : atacagtgattttggcaatagtccgtgtaatccataatccagtaacacgagctggtgcttgctagacaccttttcaatgtttaatttttttgaaataagc :  59200

                 *     59220         *     59240         *     59260         *     59280         *     59300
Seq1 : tttgataaagccttcctcgcaaattccggatacatgaacatgtcggcgacatgattaagtattgtttttcattatttttatattttctcaacaagttct :  59300
Seq2 : tttgataaagccttcctcgcaaattccggatacatgaacatgtcggcgacatgattaagtattgtttttcattatttttatattttctcaacaagttct :  59300
Seq3 : tttgataaagccttcctcgcaaattccggatacatgaacatgtcggcgacatgattaagtattgtttttcattatttttatattttctcaacaagttct :  59300
Seq4 : tttgataaagccttcctcgcaaattccggatacatgaacatgtcggcgacatgattaagtattgtttttcattatttttatattttctcaacaagttct :  59300

                 *     59320         *     59340         *     59360         *     59380         *     59400
Seq1 : caataccccaatagatgatagaatatcacccaatgcgtccatgttgtctatttccaacaggtcgctatatccaccaatagaagttttcccaaaaaagatt :  59400
Seq2 : caataccccaatagatgatagaatatcacccaatgcgtccatgttgtctatttccaacaggtcgctatatccaccaatagaagttttcccaaaaaagatt :  59400
Seq3 : caataccccaatagatgatagaatatcacccaatgcgtccatgttgtctatttccaacaggtcgctatatccaccaatagaagttttcccaaaaaagatt :  59400
Seq4 : caataccccaatagatgatagaatatcacccaatgcgtccatgttgtctatttccaacaggtcgctatatccaccaatagaagttttcccaaaaaagatt :  59400

                 *     59420         *     59440         *     59460         *     59480         *     59500
Seq1 : ctaggaacagttctaccaccagtaatttgttcaaaatagtcacgcaattcattttcgggtttaaattctttaatatcgacaatttcatacgctcctcttt :  59500
Seq2 : ctaggaacagttctaccaccagtaatttgttcaaaatagtcacgcaattcattttcgggtttaaattctttaatatcgacaatttcatacgctcctcttt :  59500
Seq3 : ctaggaacagttctaccaccagtaatttgttcaaaatagtcacgcaattcattttcgggtttaaattctttaatatcgacaatttcatacgctcctcttt :  59500
Seq4 : ctaggaacagttctaccaccagtaatttgttcaaaatagtcacgcaattcattttcgggtttaaattctttaatatcgacaatttcatacgctcctcttt :  59500
```

```
         *     59520         *     59540         *     59560         *     59580         *     59600
Seq1 : tgaaactaaacttatttagaatatccagtgcatttctacaaaaaggacatgtatacttgacaaaaattgtcactttgttattggccaacctttgttgtac :  59600
Seq2 : tgaaactaaacttatttagaatatccagtgcatttctacaaaaaggacatgtatacttgacaaaaattgtcactttgttattggccaacctttgttgtac :  59600
Seq3 : tgaaactaaacttatttagaatatccagtgcatttctacaaaaaggacatgtatacttgacaaaaattgtcactttgttattggccaacctttgttgtac :  59600
Seq4 : tgaaactaaacttatttagaatatccagtgcatttctacaaaaaggacatgtatacttgacaaaaattgtcactttgttattggccaacctttgttgtac :  59600

         *     59620         *     59640         *     59660         *     59680         *     59700
Seq1 : aaattcctcggccattttaatatttaagtgatataaaactatctcgacttatttaactctttagtcgagatatatggacgcagatagctatatgatagcc :  59700
Seq2 : aaattcctcggccattttaatatttaagtgatataaaactatctcgacttatttaactctttagtcgagatatatggacgcagatagctatatgatagcc :  59700
Seq3 : aaattcctcggccattttaatatttaagtgatataaaactatctcgacttatttaactctttagtcgagatatatggacgcagatagctatatgatagcc :  59700
Seq4 : aaattcctcggccattttaatatttaagtgatataaaactatctcgacttatttaactctttagtcgagatatatggacgcagatagctatatgatagcc :  59700

         *     59720         *     59740         *     59760         *     59780         *     59800
Seq1 : aactacagaaggcaaacgctataaaaaacataattacgacgagcatatttataaatatttttattcagcattacttgatatagtaatattaggcacagtc :  59800
Seq2 : aactacagaaggcaaacgctataaaaaacataattacgacgagcatatttataaatatttttattcagcattacttgatatagtaatattaggcacagtc :  59800
Seq3 : aactacagaaggcaaacgctataaaaaacataattacgacgagcatatttataaatatttttattcagcattacttgatatagtaatattaggcacagtc :  59800
Seq4 : aactacagaaggcaaacgctataaaaaacataattacgacgagcatatttataaatatttttattcagcattacttgatatagtaatattaggcacagtc :  59800

         *     59820         *     59840         *     59860         *     59880         *     59900
Seq1 : aaacattcaaccactctcgatacattaactctctcattttctttaacaaattctgcaatatcttcgtaaaaagattcttgaaactttttagaatatctat :  59900
Seq2 : aaacattcaaccactctcgatacattaactctctcattttctttaacaaattctgcaatatcttcgtaaaaagattcttgaaactttttagaatatctat :  59900
Seq3 : aaacattcaaccactctcgatacattaactctctcattttctttaacaaattctgcaatatcttcgtaaaaagattcttgaaactttttagaatatctat :  59900
Seq4 : aaacattcaaccactctcgatacattaactctctcattttctttaacaaattctgcaatatcttcgtaaaaagattcttgaaactttttagaatatctat :  59900

         *     59920         *     59940         *     59960         *     59980         *     60000
Seq1 : cgactctagatgaaatagcgttcgtcaacatactatgttttgtatacataaaggcgcccattttaacagtttctagtgacaaaatgctagcgatcctagg :  60000
Seq2 : cgactctagatgaaatagcgttcgtcaacatactatgttttgtatacataaaggcgcccattttaacagtttctagtgacaaaatgctagcgatcctagg :  60000
Seq3 : cgactctagatgaaatagcgttcgtcaacatactatgttttgtatacataaaggcgcccattttaacagtttctagtgacaaaatgctagcgatcctagg :  60000
Seq4 : cgactctagatgaaatagcgttcgtcaacatactatgttttgtatacataaaggcgcccattttaacagtttctagtgacaaaatgctagcgatcctagg :  60000

         *     60020         *     60040         *     60060         *     60080         *     60100
Seq1 : atcctttagaatcacatagattgacgattcgtctctcttagtaactctagtaaaataatcatacaatctagtacgcgaaataatattatccttgacttga :  60100
Seq2 : atcctttagaatcacatagattgacgattcgtctctcttagtaactctagtaaaataatcatacaatctagtacgcgaaataatattatccttgacttga :  60100
Seq3 : atcctttagaatcacatagattgacgattcgtctctcttagtaactctagtaaaataatcatacaatctagtacgcgaaataatattatccttgacttga :  60100
Seq4 : atcctttagaatcacatagattgacgattcgtctctcttagtaactctagtaaaataatcatacaatctagtacgcgaaataatattatccttgacttga :  60100

         *     60120         *     60140         *     60160         *     60180         *     60200
Seq1 : ggagatctaaacaatctagttttgagaacatcgataagttcatcgggaatgacatacatactatctttaatagaactcttttcatccagttgaatggatt :  60200
Seq2 : ggagatctaaacaatctagttttgagaacatcgataagttcatcgggaatgacatacatactatctttaatagaactcttttcatccagttgaatggatt :  60200
Seq3 : ggagatctaaacaatctagttttgagaacatcgataagttcatcgggaatgacatacatactatctttaatagaactcttttcatccagttgaatggatt :  60200
Seq4 : ggagatctaaacaatctagttttgagaacatcgataagttcatcgggaatgacatacatactatctttaatagaactcttttcatccagttgaatggatt :  60200
```

```
                *     60220         *     60240         *     60260         *     60280         *     60300
Seq1 : cgtccttaaccaactgattaatgagatcttctattttatcattttccagatgatatgtatgtccattaaagttaaattgtgtagcgcttctttttagtct :  60300
Seq2 : cgtccttaaccaactgattaatgagatcttctattttatcattttccagatgatatgtatgtccattaaagttaaattgtgtagcgcttctttttagtct :  60300
Seq3 : cgtccttaaccaactgattaatgagatcttctattttatcattttccagatgatatgtatgtccattaaagttaaattgtgtagcgcttctttttagtct :  60300
Seq4 : cgtccttaaccaactgattaatgagatcttctattttatcattttccagatgatatgtatgtccattaaagttaaattgtgtagcgcttctttttagtct :  60300

                *     60320         *     60340         *     60360         *     60380         *     60400
Seq1 : agcagccaatactttaacatcactaatatcgatatacaaaggagatgatttatctatggtattaagaattcgtttttcgacatctgtcaaaaccaattcc :  60400
Seq2 : agcagccaatactttaacatcactaatatcgatatacaaaggagatgatttatctatggtattaagaattcgtttttcgacatctgtcaaaaccaattcc :  60400
Seq3 : agcagccaatactttaacatcactaatatcgatatacaaaggagatgatttatctatggtattaagaattcgtttttcgacatctgtcaaaaccaattcc :  60400
Seq4 : agcagccaatactttaacatcactaatatcgatatacaaaggagatgatttatctatggtattaagaattcgtttttcgacatctgtcaaaaccaattcc :  60400

                *     60420         *     60440         *     60460         *     60480         *     60500
Seq1 : tttttgcctgtatcatccagttttccatcctttgtaaagaaattattttctactagactattaataagactgataaggattcctccataattgcacaatc :  60500
Seq2 : tttttgcctgtatcatccagttttccatcctttgtaaagaaattattttctactagactattaataagactgataaggattcctccataattgcacaatc :  60500
Seq3 : tttttgcctgtatcatccagttttccatcctttgtaaagaaattattttctactagactattaataagactgataaggattcctccataattgcacaatc :  60500
Seq4 : tttttgcctgtatcatccagttttccatcctttgtaaagaaattattttctactagactattaataagactgataaggattcctccataattgcacaatc :  60500

                *     60520         *     60540         *     60560         *     60580         *     60600
Seq1 : caaactttttcacaaaactagactttacaagatctacaggaatgcgtacttcaggtttcttagcttgtgattttttcttttgtggacattttcttgtgac :  60600
Seq2 : caaactttttcacaaaactagactttacaagatctacaggaatgcgtacttcaggtttcttagcttgtgattttttcttttgtggacattttcttgtgac :  60600
Seq3 : caaactttttcacaaaactagactttacaagatctacaggaatgcgtacttcaggtttcttagcttgtgattttttcttttgtggacattttcttgtgac :  60600
Seq4 : caaactttttcacaaaactagactttacaagatctacaggaatgcgtacttcaggtttcttagcttgtgattttttcttttgtggacattttcttgtgac :  60600

                *     60620         *     60640         *     60660         *     60680         *     60700
Seq1 : caactcatctaccatttcattgattttagcagtgaaataagctttcaatgcacgggcactgatactattgaaaacgagttgatcttcaaattccgccatt :  60700
Seq2 : caactcatctaccatttcattgattttagcagtgaaataagctttcaatgcacgggcactgatactattgaaaacgagttgatcttcaaattccgccatt :  60700
Seq3 : caactcatctaccatttcattgattttagcagtgaaataagctttcaatgcacgggcactgatactattgaaaacgagttgatcttcaaattccgccatt :  60700
Seq4 : caactcatctaccatttcattgattttagcagtgaaataagctttcaatgcacgggcactgatactattgaaaacgagttgatcttcaaattccgccatt :  60700

                *     60720         *     60740         *     60760         *     60780         *     60800
Seq1 : taagttcaccaaacaacttttaaatacaaatatatcaatagtagtagaataagaactataaaaaaaataataattaaccaataccaaccccaacaaccgg :  60800
Seq2 : taagttcaccaaacaacttttaaatacaaatatatcaatagtagtagaataagaactataaaaaaaataataattaaccaataccaaccccaacaaccgg :  60800
Seq3 : taagttcaccaaacaacttttaaatacaaatatatcaatagtagtagaataagaactataaaaaaaataataattaaccaataccaaccccaacaaccgg :  60800
Seq4 : taagttcaccaaacaacttttaaatacaaatatatcaatagtagtagaataagaactataaaaaaaataataattaaccaataccaaccccaacaaccgg :  60800

                *     60820         *     60840         *     60860         *     60880         *     60900
Seq1 : tattattagttgatgtgactgttttctcatcacttagaacagatttaacaatttctataaagtctgtcaaatcatcttccggagaccccataaatacacc :  60900
Seq2 : tattattagttgatgtgactgttttctcatcacttagaacagatttaacaatttctataaagtctgtcaaatcatcttccggagaccccataaatacacc :  60900
Seq3 : tattattagttgatgtgactgttttctcatcacttagaacagatttaacaatttctataaagtctgtcaaatcatcttccggagaccccataaatacacc :  60900
Seq4 : tattattagttgatgtgactgttttctcatcacttagaacagatttaacaatttctataaagtctgtcaaatcatcttccggagaccccataaatacacc :  60900
```

```
                 *     60920          *     60940          *     60960          *     60980          *     61000
Seq1 : aaatatagcggcgtacaacttatccatttatacattgaatattggcttttctttatcgctatcttcatcatattcatcatcaatatcaacaagtcccaga :  61000
Seq2 : aaatatagcggcgtacaacttatccatttatacattgaatattggcttttctttatcgctatcttcatcatattcatcatcaatatcaacaagtcccaga :  61000
Seq3 : aaatatagcggcgtacaacttatccatttatacattgaatattggcttttctttatcgctatcttcatcatattcatcatcaatatcaacaagtcccaga :  61000
Seq4 : aaatatagcggcgtacaacttatccatttatacattgaatattggcttttctttatcgctatcttcatcatattcatcatcaatatcaacaagtcccaga :  61000

                 *     61020          *     61040          *     61060          *     61080          *     61100
Seq1 : ttacgagccagatcttcttctacattttcagtcattgatacacgttcactatctccagagagtccgataacgttagccaccacttctctatcaatgatta :  61100
Seq2 : ttacgagccagatcttcttctacattttcagtcattgatacacgttcactatctccagagagtccgataacgttagccaccacttctctatcaatgatta :  61100
Seq3 : ttacgagccagatcttcttctacattttcagtcattgatacacgttcactatctccagagagtccgataacgttagccaccacttctctatcaatgatta :  61100
Seq4 : ttacgagccagatcttcttctacattttcagtcattgatacacgttcactatctccagagagtccgataacgttagccaccacttctctatcaatgatta :  61100

                 *     61120          *     61140          *     61160          *     61180          *     61200
Seq1 : gtttcttgagtgcgaatgtaatttttgtttccgttccggatctatagaaaactacaggtgtgataattgccttggccaattgtctttctcttttactgag :  61200
Seq2 : gtttcttgagtgcgaatgtaatttttgtttccgttccggatctatagaaaactacaggtgtgataattgccttggccaattgtctttctcttttactgag :  61200
Seq3 : gtttcttgagtgcgaatgtaatttttgtttccgttccggatctatagaaaactacaggtgtgataattgccttggccaattgtctttctcttttactgag :  61200
Seq4 : gtttcttgagtgcgaatgtaatttttgtttccgttccggatctatagaaaactacaggtgtgataattgccttggccaattgtctttctcttttactgag :  61200

                 *     61220          *     61240          *     61260          *     61280          *     61300
Seq1 : tgattctagttcaccttctatagatctgagaatggatgattctccagtcgaaacatattctaccatggctccgtttaatttgttgatgaagatggattca :  61300
Seq2 : tgattctagttcaccttctatagatctgagaatggatgattctccagtcgaaacatattctaccatggctccgtttaatttgttgatgaagatggattca :  61300
Seq3 : tgattctagttcaccttctatagatctgagaatggatgattctccagtcgaaacatattctaccatggctccgtttaatttgttgatgaagatggattca :  61300
Seq4 : tgattctagttcaccttctatagatctgagaatggatgattctccagtcgaaacatattctaccatggctccgtttaatttgttgatgaagatggattca :  61300

                 *     61320          *     61340          *     61360          *     61380          *     61400
Seq1 : tccttaaatgttttctctgtaatagtttccaccgaaagactatgcaaagaatttggaatgcgttccttgtgcttaatgtttccatagacggcttctagaa :  61400
Seq2 : tccttaaatgttttctctgtaatagtttccaccgaaagactatgcaaagaatttggaatgcgttccttgtgcttaatgtttccatagacggcttctagaa :  61400
Seq3 : tccttaaatgttttctctgtaatagtttccaccgaaagactatgcaaagaatttggaatgcgttccttgtgcttaatgtttccatagacggcttctagaa :  61400
Seq4 : tccttaaatgttttctctgtaatagtttccaccgaaagactatgcaaagaatttggaatgcgttccttgtgcttaatgtttccatagacggcttctagaa :  61400

                 *     61420          *     61440          *     61460          *     61480          *     61500
Seq1 : gttgatacaacataggactagccgcggtaactttatttttagaaagtatccatcgcttctatcttgtttagatttatttttataaagtttagtctctcc :  61500
Seq2 : gttgatacaacataggactagccgcggtaactttatttttagaaagtatccatcgcttctatcttgtttagatttatttttataaagtttagtctctcc :  61500
Seq3 : gttgatacaacataggactagccgcggtaactttatttttagaaagtatccatcgcttctatcttgtttagatttatttttataaagtttagtctctcc :  61500
Seq4 : gttgatacaacataggactagccgcggtaactttatttttagaaagtatccatcgcttctatcttgtttagatttatttttataaagtttagtctctcc :  61500

                 *     61520          *     61540          *     61560          *     61580          *     61600
Seq1 : ttccaacataataaaagtggaagtcatttgactagataaactatcagtaagttttatagagatagacgaacaattagcgtattgagaagcatttagtgta :  61600
Seq2 : ttccaacataataaaagtggaagtcatttgactagataaactatcagtaagttttatagagatagacgaacaattagcgtattgagaagcatttagtgta :  61600
Seq3 : ttccaacataataaaagtggaagtcatttgactagataaactatcagtaagttttatagagatagacgaacaattagcgtattgagaagcatttagtgta :  61600
Seq4 : ttccaacataataaaagtggaagtcatttgactagataaactatcagtaagttttatagagatagacgaacaattagcgtattgagaagcatttagtgta :  61600
```

```
                 *     61620         *     61640         *     61660         *     61680         *     61700
Seq1 : acgtattcgatacattttgcattagatttactaatcgattttgcatactctataacacccgcacaagtctgtagagaatcgctagatgcagtaggtcttg :  61700
Seq2 : acgtattcgatacattttgcattagatttactaatcgattttgcatactctataacacccgcacaagtctgtagagaatcgctagatgcagtaggtcttg :  61700
Seq3 : acgtattcgatacattttgcattagatttactaatcgattttgcatactctataacacccgcacaagtctgtagagaatcgctagatgcagtaggtcttg :  61700
Seq4 : acgtattcgatacattttgcattagatttactaatcgattttgcatactctataacacccgcacaagtctgtagagaatcgctagatgcagtaggtcttg :  61700

                 *     61720         *     61740         *     61760         *     61780         *     61800
Seq1 : gtgaagtttcaactctcttcttgattaccttactcatgattaaacctaaataattgtactttgtaatataatgatatatattttcactttatctcatttg :  61800
Seq2 : gtgaagtttcaactctcttcttgattaccttactcatgattaaacctaaataattgtactttgtaatataatgatatatattttcactttatctcatttg :  61800
Seq3 : gtgaagtttcaactctcttcttgattaccttactcatgattaaacctaaataattgtactttgtaatataatgatatatattttcactttatctcatttg :  61800
Seq4 : gtgaagtttcaactctcttcttgattaccttactcatgattaaacctaaataattgtactttgtaatataatgatatatattttcactttatctcatttg :  61800

                 *     61820         *     61840         *     61860         *     61880         *     61900
Seq1 : agaataaaaatgtttttgtttaaccactgcatgatgtacagatttcggaatcacaaaccaccggtggttttattttatccttgtccaatgtgaattgaat :  61900
Seq2 : agaataaaaatgtttttgtttaaccactgcatgatgtacagatttcggaatcacaaaccaccggtggttttattttatccttgtccaatgtgaattgaat :  61900
Seq3 : agaataaaaatgtttttgtttaaccactgcatgatgtacagatttcggaatcacaaaccaccggtggttttattttatccttgtccaatgtgaattgaat :  61900
Seq4 : agaataaaaatgtttttgtttaaccactgcatgatgtacagatttcggaatcacaaaccaccggtggttttattttatccttgtccaatgtgaattgaat :  61900

                 *     61920         *     61940         *     61960         *     61980         *     62000
Seq1 : gggagcggatgcgggtttcgtacgtagatagtacattcccgtttttagaccgagactccatccgtaaaaatgcatactcgttagtttggaataactcgga :  62000
Seq2 : gggagcggatgcgggtttcgtacgtagatagtacattcccgtttttagaccgagactccatccgtaaaaatgcatactcgttagtttggaataactcgga :  62000
Seq3 : gggagcggatgcgggtttcgtacgtagatagtacattcccgtttttagaccgagactccatccgtaaaaatgcatactcgttagtttggaataactcgga :  62000
Seq4 : gggagcggatgcgggtttcgtacgtagatagtacattcccgtttttagaccgagactccatccgtaaaaatgcatactcgttagtttggaataactcgga :  62000

                 *     62020         *     62040         *     62060         *     62080         *     62100
Seq1 : tctgctatatggatattcatagattgactttgatcgatgaaggctcccctgtctgcagccattttatgatcgtcttttgtggaatttcccaaatagttt :  62100
Seq2 : tctgctatatggatattcatagattgactttgatcgatgaaggctcccctgtctgcagccattttatgatcgtcttttgtggaatttcccaaatagttt :  62100
Seq3 : tctgctatatggatattcatagattgactttgatcgatgaaggctcccctgtctgcagccattttatgatcgtcttttgtggaatttcccaaatagttt :  62100
Seq4 : tctgctatatggatattcatagattgactttgatcgatgaaggctcccctgtctgcagccattttatgatcgtcttttgtggaatttcccaaatagttt :  62100

                 *     62120         *     62140         *     62160         *     62180         *     62200
Seq1 : tataaactcgcttaatatcttctggaaggtttgtattctgaatggatccaccatctgccataatcctattcttgatctcatcattccataatttctctc :  62200
Seq2 : tataaactcgcttaatatcttctggaaggtttgtattctgaatggatccaccatctgccataatcctattcttgatctcatcattccataatttctctc :  62200
Seq3 : tataaactcgcttaatatcttctggaaggtttgtattctgaatggatccaccatctgccataatcctattcttgatctcatcattccataatttctctc :  62200
Seq4 : tataaactcgcttaatatcttctggaaggtttgtattctgaatggatccaccatctgccataatcctattcttgatctcatcattccataatttctctc :  62200

                 *     62220         *     62240         *     62260         *     62280         *     62300
Seq1 : ggttaaaactctaaggagatgcggattaactacttgaaattctccagacaatactctccgagtgtaaatattactggtatacggttccaccgactcatta :  62300
Seq2 : ggttaaaactctaaggagatgcggattaactacttgaaattctccagacaatactctccgagtgtaaatattactggtatacggttccaccgactcatta :  62300
Seq3 : ggttaaaactctaaggagatgcggattaactacttgaaattctccagacaatactctccgagtgtaaatattactggtatacggttccaccgactcatta :  62300
Seq4 : ggttaaaactctaaggagatgcggattaactacttgaaattctccagacaatactctccgagtgtaaatattactggtatacggttccaccgactcatta :  62300
```

```
                 *     62320         *     62340         *     62360         *     62380         *     62400
Seq1 : tttcccaaaatttgagcagttgatgcagtcggcataggtgccaccaataaactatttctaagaccgtatgttctgattttatcttttagaggttcccaat :  62400
Seq2 : tttcccaaaatttgagcagttgatgcagtcggcataggtgccaccaataaactatttctaagaccgtatgttctgattttatcttttagaggttcccaat :  62400
Seq3 : tttcccaaaatttgagcagttgatgcagtcggcataggtgccaccaataaactatttctaagaccgtatgttctgattttatcttttagaggttcccaat :  62400
Seq4 : tttcccaaaatttgagcagttgatgcagtcggcataggtgccaccaataaactatttctaagaccgtatgttctgattttatcttttagaggttcccaat :  62400

                 *     62420         *     62440         *     62460         *     62480         *     62500
Seq1 : tccaaagatccgacggtacaacattccaaagatcatattgtagaataccgttactggcgtacgatcctacatatgtatcgtatggtccttccttctcagc :  62500
Seq2 : tccaaagatccgacggtacaacattccaaagatcatattgtagaataccgttactggcgtacgatcctacatatgtatcgtatggtccttccttctcagc :  62500
Seq3 : tccaaagatccgacggtacaacattccaaagatcatattgtagaataccgttactggcgtacgatcctacatatgtatcgtatggtccttccttctcagc :  62500
Seq4 : tccaaagatccgacggtacaacattccaaagatcatattgtagaataccgttactggcgtacgatcctacatatgtatcgtatggtccttccttctcagc :  62500

                 *     62520         *     62540         *     62560         *     62580         *     62600
Seq1 : tagttcacaactcgcctctaatgcaccgtaataaatggtttcgaagatcttcttatttagatcttgtgcttccaggctatcaaatggataatttaagaga :  62600
Seq2 : tagttcacaactcgcctctaatgcaccgtaataaatggtttcgaagatcttcttatttagatcttgtgcttccaggctatcaaatggataatttaagaga :  62600
Seq3 : tagttcacaactcgcctctaatgcaccgtaataaatggtttcgaagatcttcttatttagatcttgtgcttccaggctatcaaatggataatttaagaga :  62600
Seq4 : tagttcacaactcgcctctaatgcaccgtaataaatggtttcgaagatcttcttatttagatcttgtgcttccaggctatcaaatggataatttaagaga :  62600

                 *     62620         *     62640         *     62660         *     62680         *     62700
Seq1 : ataaacgcgtccgctaatccttgaacaccaataccgataggtctatgtctcttattagagatttcagcttctggaataggataataattaatatctataa :  62700
Seq2 : ataaacgcgtccgctaatccttgaacaccaataccgataggtctatgtctcttattagagatttcagcttctggaataggataataattaatatctataa :  62700
Seq3 : ataaacgcgtccgctaatccttgaacaccaataccgataggtctatgtctcttattagagatttcagcttctggaataggataataattaatatctataa :  62700
Seq4 : ataaacgcgtccgctaatccttgaacaccaataccgataggtctatgtctcttattagagatttcagcttctggaataggataataattaatatctataa :  62700

                 *     62720         *     62740         *     62760         *     62780         *     62800
Seq1 : ttttattgagatttctgacaattactttgaccacatccttcagtttgagaaaatcaaatcgcccatctattacaaacatgttcaaggcaacagatgccag :  62800
Seq2 : ttttattgagatttctgacaattactttgaccacatccttcagtttgagaaaatcaaatcgcccatctattacaaacatgttcaaggcaacagatgccag :  62800
Seq3 : ttttattgagatttctgacaattactttgaccacatccttcagtttgagaaaatcaaatcgcccatctattacaaacatgttcaaggcaacagatgccag :  62800
Seq4 : ttttattgagatttctgacaattactttgaccacatccttcagtttgagaaaatcaaatcgcccatctattacaaacatgttcaaggcaacagatgccag :  62800

                 *     62820         *     62840         *     62860         *     62880         *     62900
Seq1 : attacaaacggctacctcattagcatccgcatattgtattatctcagtgcaaagattactacacttgatagttcctaaattttgttgattactctttttg :  62900
Seq2 : attacaaacggctacctcattagcatccgcatattgtattatctcagtgcaaagattactacacttgatagttcctaaattttgttgattactctttttg :  62900
Seq3 : attacaaacggctacctcattagcatccgcatattgtattatctcagtgcaaagattactacacttgatagttcctaaattttgttgattactctttttg :  62900
Seq4 : attacaaacggctacctcattagcatccgcatattgtattatctcagtgcaaagattactacacttgatagttcctaaattttgttgattactctttttg :  62900

                 *     62920         *     62940         *     62960         *     62980         *     63000
Seq1 : ttacacgcatccttataaagaatgaatggagtaccagtttcaatctgagattctataatcgctttccagacgactcgagcctttattatagatttgtatc :  63000
Seq2 : ttacacgcatccttataaagaatgaatggagtaccagtttcaatctgagattctataatcgctttccagacgactcgagcctttattatagatttgtatc :  63000
Seq3 : ttacacgcatccttataaagaatgaatggagtaccagtttcaatctgagattctataatcgctttccagacgactcgagcctttattatagatttgtatc :  63000
Seq4 : ttacacgcatccttataaagaatgaatggagtaccagtttcaatctgagattctataatcgctttccagacgactcgagcctttattatagatttgtatc :  63000
```

```
               *     63020          *     63040          *     63060          *     63080          *     63100
Seq1 : tcctttctctttcgtatagtgtatacaatcgttcgaactcgtctccccaaacattgtccaatccaggacattcatccggacacatcaacgaccactctcc :  63100
Seq2 : tcctttctctttcgtatagtgtatacaatcgttcgaactcgtctccccaaacattgtccaatccaggacattcatccggacacatcaacgaccactctcc :  63100
Seq3 : tcctttctctttcgtatagtgtatacaatcgttcgaactcgtctccccaaacattgtccaatccaggacattcatccggacacatcaacgaccactctcc :  63100
Seq4 : tcctttctctttcgtatagtgtatacaatcgttcgaactcgtctccccaaacattgtccaatccaggacattcatccggacacatcaacgaccactctcc :  63100

               *     63120          *     63140          *     63160          *     63180          *     63200
Seq1 : gtcatccttcactcgtttcataaagagatcaggaatccaaagagctataaatagatctctggttctatgttcctcgtttcctgtattctttttaagatcg :  63200
Seq2 : gtcatccttcactcgtttcataaagagatcaggaatccaaagagctataaatagatctctggttctatgttcctcgtttcctgtattctttttaagatcg :  63200
Seq3 : gtcatccttcactcgtttcataaagagatcaggaatccaaagagctataaatagatctctggttctatgttcctcgtttcctgtattctttttaagatcg :  63200
Seq4 : gtcatccttcactcgtttcataaagagatcaggaatccaaagagctataaatagatctctggttctatgttcctcgtttcctgtattctttttaagatcg :  63200

               *     63220          *     63240          *     63260          *     63280          *     63300
Seq1 : aggaacgccataatatcagaatgccacggttccaagtatatggccataactccaggccgtttgtttcctccctgatctatgtatctagcggtgttattat :  63300
Seq2 : aggaacgccataatatcagaatgccacggttccaagtatatggccataactccaggccgtttgtttcctccctgatctatgtatctagcggtgttattat :  63300
Seq3 : aggaacgccataatatcagaatgccacggttccaagtatatggccataactccaggccgtttgtttcctccctgatctatgtatctagcggtgttattat :  63300
Seq4 : aggaacgccataatatcagaatgccacggttccaagtatatggccataactccaggccgtttgtttcctccctgatctatgtatctagcggtgttattat :  63300

               *     63320          *     63340          *     63360          *     63380          *     63400
Seq1 : aaactctcaacattggaataataccgtttgatataccattggtaccggagatatagcttccactggcacgaatattactaattgatagacctattccccc :  63400
Seq2 : aaactctcaacattggaataataccgtttgatataccattggtaccggagatatagcttccactggcacgaatattactaattgatagacctattccccc :  63400
Seq3 : aaactctcaacattggaataataccgtttgatataccattggtaccggagatatagcttccactggcacgaatattactaattgatagacctattccccc :  63400
Seq4 : aaactctcaacattggaataataccgtttgatataccattggtaccggagatatagcttccactggcacgaatattactaattgatagacctattccccc :  63400

               *     63420          *     63440          *     63460          *     63480          *     63500
Seq1 : tgccattttagagattaatgcgcatcgttttaacgtgtcatagataccctctatgctatcatcgatcatgttaagtagaaaacagctagacatttggtga :  63500
Seq2 : tgccattttagagattaatgcgcatcgttttaacgtgtcatagataccctctatgctatcatcgatcatgttaagtagaaaacagctagacatttggtga :  63500
Seq3 : tgccattttagagattaatgcgcatcgttttaacgtgtcatagataccctctatgctatcatcgatcatgttaagtagaaaacagctagacatttggtga :  63500
Seq4 : tgccattttagagattaatgcgcatcgttttaacgtgtcatagataccctctatgctatcatcgatcatgttaagtagaaaacagctagacatttggtga :  63500

               *     63520          *     63540          *     63560          *     63580          *     63600
Seq1 : cgactagttcccgcattaaataaggtaggagaagcgtgcgtaaaccattttcagaaagtagattgtacgtctcaatagctgagtctatatcccattgat :  63600
Seq2 : cgactagttcccgcattaaataaggtaggagaagcgtgcgtaaaccattttcagaaagtagattgtacgtctcaatagctgagtctatatcccattgat :  63600
Seq3 : cgactagttcccgcattaaataaggtaggagaagcgtgcgtaaaccattttcagaaagtagattgtacgtctcaatagctgagtctatatcccattgat :  63600
Seq4 : cgactagttcccgcattaaataaggtaggagaagcgtgcgtaaaccattttcagaaagtagattgtacgtctcaatagctgagtctatatcccattgat :  63600

               *     63620          *     63640          *     63660          *     63680          *     63700
Seq1 : gaattcctactgcgacacgcattaacatgtgctgaggtctttcaacgatcttgttgtttattttcaacaagtaggatttttccaaagttttaaaaccaaa :  63700
Seq2 : gaattcctactgcgacacgcattaacatgtgctgaggtctttcaacgatcttgttgtttattttcaacaagtaggatttttccaaagttttaaaaccaaa :  63700
Seq3 : gaattcctactgcgacacgcattaacatgtgctgaggtctttcaacgatcttgttgtttattttcaacaagtaggatttttccaaagttttaaaaccaaa :  63700
Seq4 : gaattcctactgcgacacgcattaacatgtgctgaggtctttcaacgatcttgttgtttattttcaacaagtaggatttttccaaagttttaaaaccaaa :  63700
```

```
               *     63720         *     63740         *     63760         *     63780         *     63800
Seq1 : atagttgtatgaaaagtctcgttcgtaaataataaccgagttgagtttatccttatatttgttaactatatccatggtgatacttgaaataatcggagaa :  63800
Seq2 : atagttgtatgaaaagtctcgttcgtaaataataaccgagttgagtttatccttatatttgttaactatatccatggtgatacttgaaataatcggagaa :  63800
Seq3 : atagttgtatgaaaagtctcgttcgtaaataataaccgagttgagtttatccttatatttgttaactatatccatggtgatacttgaaataatcggagaa :  63800
Seq4 : atagttgtatgaaaagtctcgttcgtaaataataaccgagttgagtttatccttatatttgttaactatatccatggtgatacttgaaataatcggagaa :  63800

               *     63820         *     63840         *     63860         *     63880         *     63900
Seq1 : tgtttcccatttttaggattaacatagttgaataaatcctccatcacttcactaaatagtttttttgtttccttgtgtagatttgatacggctattctgg :  63900
Seq2 : tgtttcccatttttaggattaacatagttgaataaatcctccatcacttcactaaatagtttttttgtttccttgtgtagatttgatacggctattctgg :  63900
Seq3 : tgtttcccatttttaggattaacatagttgaataaatcctccatcacttcactaaatagtttttttgtttccttgtgtagatttgatacggctattctgg :  63900
Seq4 : tgtttcccatttttaggattaacatagttgaataaatcctccatcacttcactaaatagtttttttgtttccttgtgtagatttgatacggctattctgg :  63900

               *     63920         *     63940         *     63960         *     63980         *     64000
Seq1 : cggctagaatggcataatccggatgttgtgtagtacaagtggctgctatttcggctgccagagtgtccaattctaccgttgttactccattatatattcc :  64000
Seq2 : cggctagaatggcataatccggatgttgtgtagtacaagtggctgctatttcggctgccagagtgtccaattctaccgttgttactccattatatattcc :  64000
Seq3 : cggctagaatggcataatccggatgttgtgtagtacaagtggctgctatttcggctgccagagtgtccaattctaccgttgttactccattatatattcc :  64000
Seq4 : cggctagaatggcataatccggatgttgtgtagtacaagtggctgctatttcggctgccagagtgtccaattctaccgttgttactccattatatattcc :  64000

               *     64020         *     64040         *     64060         *     64080         *     64100
Seq1 : ttgaataaccttcatagctatttaataggatctatatgatccgtgtttaagccataacataattttctaatacgagacgtgattttatcaaacatgaca :  64100
Seq2 : ttgaataaccttcatagctatttaataggatctatatgatccgtgtttaagccataacataattttctaatacgagacgtgattttatcaaacatgaca :  64100
Seq3 : ttgaataaccttcatagctatttaataggatctatatgatccgtgtttaagccataacataattttctaatacgagacgtgattttatcaaacatgaca :  64100
Seq4 : ttgaataaccttcatagctatttaataggatctatatgatccgtgtttaagccataacataattttctaatacgagacgtgattttatcaaacatgaca :  64100

               *     64120         *     64140         *     64160         *     64180         *     64200
Seq1 : ttttccttgtatccatttcgtttaatgacaaacatttttgttggtgtaataaaaaaattatttaacttttcattaatagggatttgacgtacgtagcgta :  64200
Seq2 : ttttccttgtatccatttcgtttaatgacaaacatttttgttggtgtaataaaaaaattatttaacttttcattaatagggatttgacgtacgtagcgta :  64200
Seq3 : ttttccttgtatccatttcgtttaatgacaaacatttttgttggtgtaataaaaaaattatttaacttttcattaatagggatttgacgtacgtagcgta :  64200
Seq4 : ttttccttgtatccatttcgtttaatgacaaacatttttgttggtgtaataaaaaaattatttaacttttcattaatagggatttgacgtacgtagcgta :  64200

               *     64220         *     64240         *     64260         *     64280         *     64300
Seq1 : caaaatgattgttcctggtatatagataaagagtcctatatatttgaaaatcgttacggctcgattaaactttaatgattgcatagtgaatatatcatta :  64300
Seq2 : caaaatgattgttcctggtatatagataaagagtcctatatatttgaaaatcgttacggctcgattaaactttaatgattgcatagtgaatatatcatta :  64300
Seq3 : caaaatgattgttcctggtatatagataaagagtcctatatatttgaaaatcgttacggctcgattaaactttaatgattgcatagtgaatatatcatta :  64300
Seq4 : caaaatgattgttcctggtatatagataaagagtcctatatatttgaaaatcgttacggctcgattaaactttaatgattgcatagtgaatatatcatta :  64300

               *     64320         *     64340         *     64360         *     64380         *     64400
Seq1 : ggatttaactccttgactatcagggcggcaccagaaattaccatcaaaagcattaatacagttatgcctatcgcagttagaacggttatagcatccacca :  64400
Seq2 : ggatttaactccttgactatcagggcggcaccagaaattaccatcaaaagcattaatacagttatgcctatcgcagttagaacggttatagcatccacca :  64400
Seq3 : ggatttaactccttgactatcagggcggcaccagaaattaccatcaaaagcattaatacagttatgcctatcgcagttagaacggttatagcatccacca :  64400
Seq4 : ggatttaactccttgactatcagggcggcaccagaaattaccatcaaaagcattaatacagttatgcctatcgcagttagaacggttatagcatccacca :  64400
```

```
                    *      64420          *      64440          *      64460          *      64480          *      64500
Seq1 : tttatatctaaaaattagatcaaagaatatgtgacaaagtcctagttgtatattgagaattgacaaaacaatgtttcttacatatttttttttattagt :  64500
Seq2 : tttatatctaaaaattagatcaaagaatatgtgacaaagtcctagttgtatattgagaattgacaaaacaatgtttcttacatatttttttttattagt :  64500
Seq3 : tttatatctaaaaattagatcaaagaatatgtgacaaagtcctagttgtatattgagaattgacaaaacaatgtttcttacatatttttttttattagt :  64500
Seq4 : tttatatctaaaaattagatcaaagaatatgtgacaaagtcctagttgtatattgagaattgacaaaacaatgtttcttacatatttttttttattagt :  64500

                    *      64520          *      64540          *      64560          *      64580          *      64600
Seq1 : aaccgacttaatagtaggaactggaaaactagacttgattattctataagtatagatacccttccaaataatattctctttgataaaagttccagaaaat :  64600
Seq2 : aaccgacttaatagtaggaactggaaaactagacttgattattctataagtatagatacccttccaaataatattctctttgataaaagttccagaaaat :  64600
Seq3 : aaccgacttaatagtaggaactggaaaactagacttgattattctataagtatagatacccttccaaataatattctctttgataaaagttccagaaaat :  64600
Seq4 : aaccgacttaatagtaggaactggaaaactagacttgattattctataagtatagatacccttccaaataatattctctttgataaaagttccagaaaat :  64600

                    *      64620          *      64640          *      64660          *      64680          *      64700
Seq1 : gtagaattttttaaaaagttatcttttgctattaccaagattgtgtttagacgcttattattaatatgagtgatgaaatccacaccgcctctagatatcg :  64700
Seq2 : gtagaattttttaaaaagttatcttttgctattaccaagattgtgtttagacgcttattattaatatgagtgatgaaatccacaccgcctctagatatcg :  64700
Seq3 : gtagaattttttaaaaagttatcttttgctattaccaagattgtgtttagacgcttattattaatatgagtgatgaaatccacaccgcctctagatatcg :  64700
Seq4 : gtagaattttttaaaaagttatcttttgctattaccaagattgtgtttagacgcttattattaatatgagtgatgaaatccacaccgcctctagatatcg :  64700

                    *      64720          *      64740          *      64760          *      64780          *      64800
Seq1 : cctttatttccacattagatggtaaatccaatagtgaaactatctttttaggaatgtatggactcgcgtttagaggagtgaacgtcttgggcgtcggaaa :  64800
Seq2 : cctttatttccacattagatggtaaatccaatagtgaaactatctttttaggaatgtatggactcgcgtttagaggagtgaacgtcttgggcgtcggaaa :  64800
Seq3 : cctttatttccacattagatggtaaatccaatagtgaaactatctttttaggaatgtatggactcgcgtttagaggagtgaacgtcttgggcgtcggaaa :  64800
Seq4 : cctttatttccacattagatggtaaatccaatagtgaaactatctttttaggaatgtatggactcgcgtttagaggagtgaacgtcttgggcgtcggaaa :  64800

                    *      64820          *      64840          *      64860          *      64880          *      64900
Seq1 : ggatgattcgtcaaacgaataaacaatttcacaaatggatgttaatgtattagtaggaaattttttgacgctagtggaattgaaaattctaatggatgat :  64900
Seq2 : ggatgattcgtcaaacgaataaacaatttcacaaatggatgttaatgtattagtaggaaattttttgacgctagtggaattgaaaattctaatggatgat :  64900
Seq3 : ggatgattcgtcaaacgaataaacaatttcacaaatggatgttaatgtattagtaggaaattttttgacgctagtggaattgaaaattctaatggatgat :  64900
Seq4 : ggatgattcgtcaaacgaataaacaatttcacaaatggatgttaatgtattagtaggaaattttttgacgctagtggaattgaaaattctaatggatgat :  64900

                    *      64920          *      64940          *      64960          *      64980          *      65000
Seq1 : gttctacctatttcatccgataacatgttaatttccgacaccaacggttttaatatttcgatgatatacggtagtctctctttcggacttatatagctta :  65000
Seq2 : gttctacctatttcatccgataacatgttaatttccgacaccaacggttttaatatttcgatgatatacggtagtctctctttcggacttatatagctta :  65000
Seq3 : gttctacctatttcatccgataacatgttaatttccgacaccaacggttttaatatttcgatgatatacggtagtctctctttcggacttatatagctta :  65000
Seq4 : gttctacctatttcatccgataacatgttaatttccgacaccaacggttttaatatttcgatgatatacggtagtctctctttcggacttatatagctta :  65000

                    *      65020          *      65040          *      65060          *      65080          *      65100
Seq1 : ttccacaatacgagtcattatatactccaaaaaacaaaataactagtataaaatctgtatcgaatgggaaaaacgaaattatcgacataggtatagaatc :  65100
Seq2 : ttccacaatacgagtcattatatactccaaaaaacaaaataactagtataaaatctgtatcgaatgggaaaaacgaaattatcgacataggtatagaatc :  65100
Seq3 : ttccacaatacgagtcattatatactccaaaaaacaaaataactagtataaaatctgtatcgaatgggaaaaacgaaattatcgacataggtatagaatc :  65100
Seq4 : ttccacaatacgagtcattatatactccaaaaaacaaaataactagtataaaatctgtatcgaatgggaaaaacgaaattatcgacataggtatagaatc :  65100
```

```
                 *     65120         *     65140         *     65160         *     65180         *     65200
Seq1 : cggaacattgaacgtattaatacttaattctttttctgtggtaagtaccgataggttattgacattgtatggttttaaatattctataacttgagacttg :  65200
Seq2 : cggaacattgaacgtattaatacttaattctttttctgtggtaagtaccgataggttattgacattgtatggttttaaatattctataacttgagacttg :  65200
Seq3 : cggaacattgaacgtattaatacttaattctttttctgtggtaagtaccgataggttattgacattgtatggttttaaatattctataacttgagacttg :  65200
Seq4 : cggaacattgaacgtattaatacttaattctttttctgtggtaagtaccgataggttattgacattgtatggttttaaatattctataacttgagacttg :  65200

                 *     65220         *     65240         *     65260         *     65280         *     65300
Seq1 : atagatattagtgatgaattgaaaattatttttatcaccacgtgtgtttcaggatcatcgtcgacgcccgtcaaccaaccgaacggagtaaaataaatat :  65300
Seq2 : atagatattagtgatgaattgaaaattatttttatcaccacgtgtgtttcaggatcatcgtcgacgcccgtcaaccaaccgaacggagtaaaataaatat :  65300
Seq3 : atagatattagtgatgaattgaaaattatttttatcaccacgtgtgtttcaggatcatcgtcgacgcccgtcaaccaaccgaacggagtaaaataaatat :  65300
Seq4 : atagatattagtgatgaattgaaaattatttttatcaccacgtgtgtttcaggatcatcgtcgacgcccgtcaaccaaccgaacggagtaaaataaatat :  65300

                 *     65320         *     65340         *     65360         *     65380         *     65400
Seq1 : cattaatatatgctctagatattagtatttttatcaatcctttgattatcatcttctcgtaggcgaatgattccatgatcaagagtgatttaagaacatc :  65400
Seq2 : cattaatatatgctctagatattagtatttttatcaatcctttgattatcatcttctcgtaggcgaatgattccatgatcaagagtgatttaagaacatc :  65400
Seq3 : cattaatatatgctctagatattagtatttttatcaatcctttgattatcatcttctcgtaggcgaatgattccatgatcaagagtgatttaagaacatc :  65400
Seq4 : cattaatatatgctctagatattagtatttttatcaatcctttgattatcatcttctcgtaggcgaatgattccatgatcaagagtgatttaagaacatc :  65400

                 *     65420         *     65440         *     65460         *     65480         *     65500
Seq1 : ctccggagtattaatgggcttagtaaacagtccatcgttgcaataataaaagttatccaagttaaaggatattatgcattcgtttaaagatatcacctca :  65500
Seq2 : ctccggagtattaatgggcttagtaaacagtccatcgttgcaataataaaagttatccaagttaaaggatattatgcattcgtttaaagatatcacctca :  65500
Seq3 : ctccggagtattaatgggcttagtaaacagtccatcgttgcaataataaaagttatccaagttaaaggatattatgcattcgtttaaagatatcacctca :  65500
Seq4 : ctccggagtattaatgggcttagtaaacagtccatcgttgcaataataaaagttatccaagttaaaggatattatgcattcgtttaaagatatcacctca :  65500

                 *     65520         *     65540         *     65560         *     65580         *     65600
Seq1 : tctgacggagacaattttttggtaggttttagagactttgaagctacttgtttaacaaagttattcatcgtcgtttactattctatttaattttgtagtt :  65600
Seq2 : tctgacggagacaattttttggtaggttttagagactttgaagctacttgtttaacaaagttattcatcgtcgtttactattctatttaattttgtagtt :  65600
Seq3 : tctgacggagacaattttttggtaggttttagagactttgaagctacttgtttaacaaagttattcatcgtcgtttactattctatttaattttgtagtt :  65600
Seq4 : tctgacggagacaattttttggtaggttttagagactttgaagctacttgtttaacaaagttattcatcgtcgtttactattctatttaattttgtagtt :  65600

                 *     65620         *     65640         *     65660         *     65680         *     65700
Seq1 : aatttatcacatatcacattaattgactttttggtccatttttccatacgtttatattcttttaatcctgcgttatccgtttccgttatatccagtgata :  65700
Seq2 : aatttatcacatatcacattaattgactttttggtccatttttccatacgtttatattcttttaatcctgcgttatccgtttccgttatatccagtgata :  65700
Seq3 : aatttatcacatatcacattaattgactttttggtccatttttccatacgtttatattcttttaatcctgcgttatccgtttccgttatatccagtgata :  65700
Seq4 : aatttatcacatatcacattaattgactttttggtccatttttccatacgtttatattcttttaatcctgcgttatccgtttccgttatatccagtgata :  65700

                 *     65720         *     65740         *     65760         *     65780         *     65800
Seq1 : gatcgtgcaggttaaatagaatgctcttaaataatgtcatttttttatccgctaaaaatttaaagaatgtataaacttttttcaaagatttaaaactttt :  65800
Seq2 : gatcgtgcaggttaaatagaatgctcttaaataatgtcatttttttatccgctaaaaatttaaagaatgtataaacttttttcaaagatttaaaactttt :  65800
Seq3 : gatcgtgcaggttaaatagaatgctcttaaataatgtcatttttttatccgctaaaaatttaaagaatgtataaacttttttcaaagatttaaaactttt :  65800
Seq4 : gatcgtgcaggttaaatagaatgctcttaaataatgtcatttttttatccgctaaaaatttaaagaatgtataaacttttttcaaagatttaaaactttt :  65800
```

```
              *      65820          *      65840          *      65860          *      65880          *      65900
Seq1 : aggtggtgtcctagtacacaatatcataaacaaactaataaacatcccgcattcagattccaacagctgattaacttccacattaatacagcctattttc :  65900
Seq2 : aggtggtgtcctagtacacaatatcataaacaaactaataaacatcccgcattcagattccaacagctgattaacttccacattaatacagcctattttc :  65900
Seq3 : aggtggtgtcctagtacacaatatcataaacaaactaataaacatcccgcattcagattccaacagctgattaacttccacattaatacagcctattttc :  65900
Seq4 : aggtggtgtcctagtacacaatatcataaacaaactaataaacatcccgcattcagattccaacagctgattaacttccacattaatacagcctattttc :  65900

              *      65920          *      65940          *      65960          *      65980          *      66000
Seq1 : gctccaaatgtacattcgaaaaatctgaataaaacatcaatgtcgcaatttgtattatccaatacagaatgtttgtgattcgtgttaaaaccatcggaga :  66000
Seq2 : gctccaaatgtacattcgaaaaatctgaataaaacatcaatgtcgcaatttgtattatccaatacagaatgtttgtgattcgtgttaaaaccatcggaga :  66000
Seq3 : gctccaaatgtacattcgaaaaatctgaataaaacatcaatgtcgcaatttgtattatccaatacagaatgtttgtgattcgtgttaaaaccatcggaga :  66000
Seq4 : gctccaaatgtacattcgaaaaatctgaataaaacatcaatgtcgcaatttgtattatccaatacagaatgtttgtgattcgtgttaaaaccatcggaga :  66000

              *      66020          *      66040          *      66060          *      66080          *      66100
Seq1 : aggaatagaaataaaaattattatagtggtggaattcagttggaatattgcctccggagtcataaaaggatactaaacattgtttttttatcataaattac :  66100
Seq2 : aggaatagaaataaaaattattatagtggtggaattcagttggaatattgcctccggagtcataaaaggatactaaacattgtttttttatcataaattac :  66100
Seq3 : aggaatagaaataaaaattattatagtggtggaattcagttggaatattgcctccggagtcataaaaggatactaaacattgtttttttatcataaattac :  66100
Seq4 : aggaatagaaataaaaattattatagtggtggaattcagttggaatattgcctccggagtcataaaaggatactaaacattgtttttttatcataaattac :  66100

              *      66120          *      66140          *      66160          *      66180          *      66200
Seq1 : acatttccaatgagacaaataacaaaatccaaacattacaaatctagaggtagaacttttaattttgtctttaagtatatacgataagatatgtttattc :  66200
Seq2 : acatttccaatgagacaaataacaaaatccaaacattacaaatctagaggtagaacttttaattttgtctttaagtatatacgataagatatgtttattc :  66200
Seq3 : acatttccaatgagacaaataacaaaatccaaacattacaaatctagaggtagaacttttaattttgtctttaagtatatacgataagatatgtttattc :  66200
Seq4 : acatttccaatgagacaaataacaaaatccaaacattacaaatctagaggtagaacttttaattttgtctttaagtatatacgataagatatgtttattc :  66200

              *      66220          *      66240          *      66260          *      66280          *      66300
Seq1 : ataaacgcgtcaaatttttcatgaatcgctaaggagtttaagaatctcatgtcaaattgtcctatataatccacttcggatccataagcaaactgagaga :  66300
Seq2 : ataaacgcgtcaaatttttcatgaatcgctaaggagtttaagaatctcatgtcaaattgtcctatataatccacttcggatccataagcaaactgagaga :  66300
Seq3 : ataaacgcgtcaaatttttcatgaatcgctaaggagtttaagaatctcatgtcaaattgtcctatataatccacttcggatccataagcaaactgagaga :  66300
Seq4 : ataaacgcgtcaaatttttcatgaatcgctaaggagtttaagaatctcatgtcaaattgtcctatataatccacttcggatccataagcaaactgagaga :  66300

              *      66320          *      66340          *      66360          *      66380          *      66400
Seq1 : ctaagttcttaatacttcgattgctcatccaggctcctctctcaggctctattttcatcttgacgacctttggattttcaccagtatgtattcctttacg :  66400
Seq2 : ctaagttcttaatacttcgattgctcatccaggctcctctctcaggctctattttcatcttgacgacctttggattttcaccagtatgtattcctttacg :  66400
Seq3 : ctaagttcttaatacttcgattgctcatccaggctcctctctcaggctctattttcatcttgacgacctttggattttcaccagtatgtattcctttacg :  66400
Seq4 : ctaagttcttaatacttcgattgctcatccaggctcctctctcaggctctattttcatcttgacgacctttggattttcaccagtatgtattcctttacg :  66400

              *      66420          *      66440          *      66460          *      66480          *      66500
Seq1 : tgataaatcatcgattttcaaatccatttgtgagaagtctatcgccttagatactttttcccgtagtcgaggtttaaagaaatacgctaacggtatacta :  66500
Seq2 : tgataaatcatcgattttcaaatccatttgtgagaagtctatcgccttagatactttttcccgtagtcgaggtttaaagaaatacgctaacggtatacta :  66500
Seq3 : tgataaatcatcgattttcaaatccatttgtgagaagtctatcgccttagatactttttcccgtagtcgaggtttaaagaaatacgctaacggtatacta :  66500
Seq4 : tgataaatcatcgattttcaaatccatttgtgagaagtctatcgccttagatactttttcccgtagtcgaggtttaaagaaatacgctaacggtatacta :  66500
```

```
                     *     66520         *     66540         *     66560         *     66580         *     66600
Seq1 : gtaggtaactcaaaaacatcatatatagaatggtaacgcgtctttaactcgtcggttaactctttcttttgatcgagttcgtcgctactattgggtctgc :  66600
Seq2 : gtaggtaactcaaaaacatcatatatagaatggtaacgcgtctttaactcgtcggttaactctttcttttgatcgagttcgtcgctactattgggtctgc :  66600
Seq3 : gtaggtaactcaaaaacatcatatatagaatggtaacgcgtctttaactcgtcggttaactctttcttttgatcgagttcgtcgctactattgggtctgc :  66600
Seq4 : gtaggtaactcaaaaacatcatatatagaatggtaacgcgtctttaactcgtcggttaactctttcttttgatcgagttcgtcgctactattgggtctgc :  66600

                     *     66620         *     66640         *     66660         *     66680         *     66700
Seq1 : tcaggtgccccgactctactagttccaacatcataccgataggaatacaagacactttgccggcggttgtagatttatcatatttttccactacatatcc :  66700
Seq2 : tcaggtgccccgactctactagttccaacatcataccgataggaatacaagacactttgccggcggttgtagatttatcatatttttccactacatatcc :  66700
Seq3 : tcaggtgccccgactctactagttccaacatcataccgataggaatacaagacactttgccggcggttgtagatttatcatatttttccactacatatcc :  66700
Seq4 : tcaggtgccccgactctactagttccaacatcataccgataggaatacaagacactttgccggcggttgtagatttatcatatttttccactacatatcc :  66700

                     *     66720         *     66740         *     66760         *     66780         *     66800
Seq1 : gttacaatttgttaaaaatttagatacatctatattgctacataatccagctagtgaatatatatgacataataaattggtaaatcctagttctggtatt :  66800
Seq2 : gttacaatttgttaaaaatttagatacatctatattgctacataatccagctagtgaatatatatgacataataaattggtaaatcctagttctggtatt :  66800
Seq3 : gttacaatttgttaaaaatttagatacatctatattgctacataatccagctagtgaatatatatgacataataaattggtaaatcctagttctggtatt :  66800
Seq4 : gttacaatttgttaaaaatttagatacatctatattgctacataatccagctagtgaatatatatgacataataaattggtaaatcctagttctggtatt :  66800

                     *     66820         *     66840         *     66860         *     66880         *     66900
Seq1 : ttactaattactaaatctgtatatctttccatttatcatggaaaagaatttaccagatatcttcttttttccaaactgcgttaatgtattctcttacaaa :  66900
Seq2 : ttactaattactaaatctgtatatctttccatttatcatggaaaagaatttaccagatatcttcttttttccaaactgcgttaatgtattctcttacaaa :  66900
Seq3 : ttactaattactaaatctgtatatctttccatttatcatggaaaagaatttaccagatatcttcttttttccaaactgcgttaatgtattctcttacaaa :  66900
Seq4 : ttactaattactaaatctgtatatctttccatttatcatggaaaagaatttaccagatatcttcttttttccaaactgcgttaatgtattctcttacaaa :  66900

                     *     66920         *     66940         *     66960         *     66980         *     67000
Seq1 : tattcacaagatgaattcagtaatatgagtaaaacggaacgtgatagtttctcattggccgtgtttccagttataaaacatagatggcataacgcacacg :  67000
Seq2 : tattcacaagatgaattcagtaatatgagtaaaacggaacgtgatagtttctcattggccgtgtttccagttataaaacatagatggcataacgcacacg :  67000
Seq3 : tattcacaagatgaattcagtaatatgagtaaaacggaacgtgatagtttctcattggccgtgtttccagttataaaacatagatggcataacgcacacg :  67000
Seq4 : tattcacaagatgaattcagtaatatgagtaaaacggaacgtgatagtttctcattggccgtgtttccagttataaaacatagatggcataacgcacacg :  67000

                     *     67020         *     67040         *     67060         *     67080         *     67100
Seq1 : ttgtaaaacataaaggaatatacaaagttagtacagaagcacgtggaaaaaaagtatctcctccatcactaggaaaacccgcacacataaacctaaccgc :  67100
Seq2 : ttgtaaaacataaaggaatatacaaagttagtacagaagcacgtggaaaaaaagtatctcctccatcactaggaaaacccgcacacataaacctaaccgc :  67100
Seq3 : ttgtaaaacataaaggaatatacaaagttagtacagaagcacgtggaaaaaaagtatctcctccatcactaggaaaacccgcacacataaacctaaccgc :  67100
Seq4 : ttgtaaaacataaaggaatatacaaagttagtacagaagcacgtggaaaaaaagtatctcctccatcactaggaaaacccgcacacataaacctaaccgc :  67100

                     *     67120         *     67140         *     67160         *     67180         *     67200
Seq1 : gaagcaatatatatacagtgaacacacaataagctttgaatgttatagttttctaaaatgtataacaaatacagaaatcaattcgttcgatgagtatata :  67200
Seq2 : gaagcaatatatatacagtgaacacacaataagctttgaatgttatagttttctaaaatgtataacaaatacagaaatcaattcgttcgatgagtatata :  67200
Seq3 : gaagcaatatatatacagtgaacacacaataagctttgaatgttatagttttctaaaatgtataacaaatacagaaatcaattcgttcgatgagtatata :  67200
Seq4 : gaagcaatatatatacagtgaacacacaataagctttgaatgttatagttttctaaaatgtataacaaatacagaaatcaattcgttcgatgagtatata :  67200
```

```
              *     67220          *     67240          *     67260          *     67280          *     67300
Seq1 : ttaagaggactattagaagctggtaatagtttacagatattttccaattccgtaggtaaacgaacagatactataggtgtactagggaataagtatccat :  67300
Seq2 : ttaagaggactattagaagctggtaatagtttacagatattttccaattccgtaggtaaacgaacagatactataggtgtactagggaataagtatccat :  67300
Seq3 : ttaagaggactattagaagctggtaatagtttacagatattttccaattccgtaggtaaacgaacagatactataggtgtactagggaataagtatccat :  67300
Seq4 : ttaagaggactattagaagctggtaatagtttacagatattttccaattccgtaggtaaacgaacagatactataggtgtactagggaataagtatccat :  67300

              *     67320          *     67340          *     67360          *     67380          *     67400
Seq1 : ttagcaaaattccattggcctcattaactcctaaagcacaacgagagatattttcagcgtggatttctcatagacctgtagttttaactggaggaactgg :  67400
Seq2 : ttagcaaaattccattggcctcattaactcctaaagcacaacgagagatattttcagcgtggatttctcatagacctgtagttttaactggaggaactgg :  67400
Seq3 : ttagcaaaattccattggcctcattaactcctaaagcacaacgagagatattttcagcgtggatttctcatagacctgtagttttaactggaggaactgg :  67400
Seq4 : ttagcaaaattccattggcctcattaactcctaaagcacaacgagagatattttcagcgtggatttctcatagacctgtagttttaactggaggaactgg :  67400

              *     67420          *     67440          *     67460          *     67480          *     67500
Seq1 : agtgggtaagacgtcacaggtacccaagttattgctttggtttaattatttatttggtggattctctactctagataaaatcactaactttcacgaaaga :  67500
Seq2 : agtgggtaagacgtcacaggtacccaagttattgctttggtttaattatttatttggtggattctctactctagataaaatcactaactttcacgaaaga :  67500
Seq3 : agtgggtaagacgtcacaggtacccaagttattgctttggtttaattatttatttggtggattctctactctagataaaatcactaactttcacgaaaga :  67500
Seq4 : agtgggtaagacgtcacaggtacccaagttattgctttggtttaattatttatttggtggattctctactctagataaaatcactaactttcacgaaaga :  67500

              *     67520          *     67540          *     67560          *     67580          *     67600
Seq1 : ccagtcattctatctcttcctaggatagctttagttagattgcatagcaataccattttaaaatcattgggatttaaggtactagatggatctcctattt :  67600
Seq2 : ccagtcattctatctcttcctaggatagctttagttagattgcatagcaataccattttaaaatcattgggatttaaggtactagatggatctcctattt :  67600
Seq3 : ccagtcattctatctcttcctaggatagctttagttagattgcatagcaataccattttaaaatcattgggatttaaggtactagatggatctcctattt :  67600
Seq4 : ccagtcattctatctcttcctaggatagctttagttagattgcatagcaataccattttaaaatcattgggatttaaggtactagatggatctcctattt :  67600

              *     67620          *     67640          *     67660          *     67680          *     67700
Seq1 : ctttacggtacggatctataccggaagaattaataaacaaacaaccaaaaaaatatggaattgtattttctacccataagttatctctaacaaaactatt :  67700
Seq2 : ctttacggtacggatctataccggaagaattaataaacaaacaaccaaaaaaatatggaattgtattttctacccataagttatctctaacaaaactatt :  67700
Seq3 : ctttacggtacggatctataccggaagaattaataaacaaacaaccaaaaaaatatggaattgtattttctacccataagttatctctaacaaaactatt :  67700
Seq4 : ctttacggtacggatctataccggaagaattaataaacaaacaaccaaaaaaatatggaattgtattttctacccataagttatctctaacaaaactatt :  67700

              *     67720          *     67740          *     67760          *     67780          *     67800
Seq1 : tagttatggcactcttattatagacgaagttcatgagcatgatcaaataggagatattattatagcagtagcgagaaagcatcatacgaaaatagattct :  67800
Seq2 : tagttatggcactcttattatagacgaagttcatgagcatgatcaaataggagatattattatagcagtagcgagaaagcatcatacgaaaatagattct :  67800
Seq3 : tagttatggcactcttattatagacgaagttcatgagcatgatcaaataggagatattattatagcagtagcgagaaagcatcatacgaaaatagattct :  67800
Seq4 : tagttatggcactcttattatagacgaagttcatgagcatgatcaaataggagatattattatagcagtagcgagaaagcatcatacgaaaatagattct :  67800

              *     67820          *     67840          *     67860          *     67880          *     67900
Seq1 : atgtttttaatgactgccacgttagaggatgaccgagaacggctaaaagtatttttacctaatcccgcatttatacatattcctggagatacactgttta :  67900
Seq2 : atgtttttaatgactgccacgttagaggatgaccgagaacggctaaaagtatttttacctaatcccgcatttatacatattcctggagatacactgttta :  67900
Seq3 : atgtttttaatgactgccacgttagaggatgaccgagaacggctaaaagtatttttacctaatcccgcatttatacatattcctggagatacactgttta :  67900
Seq4 : atgtttttaatgactgccacgttagaggatgaccgagaacggctaaaagtatttttacctaatcccgcatttatacatattcctggagatacactgttta :  67900
```

```
               *     67920         *     67940         *     67960         *     67980         *     68000
Seq1 : aaattagcgaggtatttattcataataagataaatccatcttccagaatggcatacatagaagaagaaaagagaaatttagttactgctatacagatgta :  68000
Seq2 : aaattagcgaggtatttattcataataagataaatccatcttccagaatggcatacatagaagaagaaaagagaaatttagttactgctatacagatgta :  68000
Seq3 : aaattagcgaggtatttattcataataagataaatccatcttccagaatggcatacatagaagaagaaaagagaaatttagttactgctatacagatgta :  68000
Seq4 : aaattagcgaggtatttattcataataagataaatccatcttccagaatggcatacatagaagaagaaaagagaaatttagttactgctatacagatgta :  68000

               *     68020         *     68040         *     68060         *     68080         *     68100
Seq1 : tactcctcctgatggatcatccggtatagtctttgtggcatccgttgcacagtgtcacgaatataaatcatatttagaaaaaagattaccgtatgatatg :  68100
Seq2 : tactcctcctgatggatcatccggtatagtctttgtggcatccgttgcacagtgtcacgaatataaatcatatttagaaaaaagattaccgtatgatatg :  68100
Seq3 : tactcctcctgatggatcatccggtatagtctttgtggcatccgttgcacagtgtcacgaatataaatcatatttagaaaaaagattaccgtatgatatg :  68100
Seq4 : tactcctcctgatggatcatccggtatagtctttgtggcatccgttgcacagtgtcacgaatataaatcatatttagaaaaaagattaccgtatgatatg :  68100

               *     68120         *     68140         *     68160         *     68180         *     68200
Seq1 : tatattattcatggtaaggtcttagatatagacgaaatattagaaaaagtgtattcatcacctaatgtatcgataattatttctactccttatttggaat :  68200
Seq2 : tatattattcatggtaaggtcttagatatagacgaaatattagaaaaagtgtattcatcacctaatgtatcgataattatttctactccttatttggaat :  68200
Seq3 : tatattattcatggtaaggtcttagatatagacgaaatattagaaaaagtgtattcatcacctaatgtatcgataattatttctactccttatttggaat :  68200
Seq4 : tatattattcatggtaaggtcttagatatagacgaaatattagaaaaagtgtattcatcacctaatgtatcgataattatttctactccttatttggaat :  68200

               *     68220         *     68240         *     68260         *     68280         *     68300
Seq1 : ccagcgttactatacgcaatgttacacacatttatgatatgggtaaagtttttgtccccgctccttttggaggatcgcaagaatttatttctaaatctat :  68300
Seq2 : ccagcgttactatacgcaatgttacacacatttatgatatgggtaaagtttttgtccccgctccttttggaggatcgcaagaatttatttctaaatctat :  68300
Seq3 : ccagcgttactatacgcaatgttacacacatttatgatatgggtaaagtttttgtccccgctccttttggaggatcgcaagaatttatttctaaatctat :  68300
Seq4 : ccagcgttactatacgcaatgttacacacatttatgatatgggtaaagtttttgtccccgctccttttggaggatcgcaagaatttatttctaaatctat :  68300

               *     68320         *     68340         *     68360         *     68380         *     68400
Seq1 : gagagatcaacgaaaaggaagagtaggaagagttaatcctggtacatacgtctatttctatgatctgtcttatatgaagtctatacagcgaatagattca :  68400
Seq2 : gagagatcaacgaaaaggaagagtaggaagagttaatcctggtacatacgtctatttctatgatctgtcttatatgaagtctatacagcgaatagattca :  68400
Seq3 : gagagatcaacgaaaaggaagagtaggaagagttaatcctggtacatacgtctatttctatgatctgtcttatatgaagtctatacagcgaatagattca :  68400
Seq4 : gagagatcaacgaaaaggaagagtaggaagagttaatcctggtacatacgtctatttctatgatctgtcttatatgaagtctatacagcgaatagattca :  68400

               *     68420         *     68440         *     68460         *     68480         *     68500
Seq1 : gaatttctacataattatatattgtacgctaataagtttaatctaacactccccgaagatttgtttataatccctacaaatttggatattctatggcgta :  68500
Seq2 : gaatttctacataattatatattgtacgctaataagtttaatctaacactccccgaagatttgtttataatccctacaaatttggatattctatggcgta :  68500
Seq3 : gaatttctacataattatatattgtacgctaataagtttaatctaacactccccgaagatttgtttataatccctacaaatttggatattctatggcgta :  68500
Seq4 : gaatttctacataattatatattgtacgctaataagtttaatctaacactccccgaagatttgtttataatccctacaaatttggatattctatggcgta :  68500

               *     68520         *     68540         *     68560         *     68580         *     68600
Seq1 : caaaggaatatatagactcgttcgatattagtacagaaacatggaataaattattatccaattattatatgaagatgatagagtatgctaaactttatgt :  68600
Seq2 : caaaggaatatatagactcgttcgatattagtacagaaacatggaataaattattatccaattattatatgaagatgatagagtatgctaaactttatgt :  68600
Seq3 : caaaggaatatatagactcgttcgatattagtacagaaacatggaataaattattatccaattattatatgaagatgatagagtatgctaaactttatgt :  68600
Seq4 : caaaggaatatatagactcgttcgatattagtacagaaacatggaataaattattatccaattattatatgaagatgatagagtatgctaaactttatgt :  68600
```

```
                *     68620         *     68640         *     68660         *     68680         *     68700
Seq1 : actaagtcctattctcgctgaggagttggataactttgagaggacgggagaattaactagtattgtacgagaagccattttatctctaaatttacgaatt :  68700
Seq2 : actaagtcctattctcgctgaggagttggataactttgagaggacgggagaattaactagtattgtacgagaagccattttatctctaaatttacgaatt :  68700
Seq3 : actaagtcctattctcgctgaggagttggataactttgagaggacgggagaattaactagtattgtacgagaagccattttatctctaaatttacgaatt :  68700
Seq4 : actaagtcctattctcgctgaggagttggataactttgagaggacgggagaattaactagtattgtacgagaagccattttatctctaaatttacgaatt :  68700

                *     68720         *     68740         *     68760         *     68780         *     68800
Seq1 : aagattttaaattttaaacataaagatgatgatacgtatatacacttttgtaaaatattattcggtgtctataacggaacaaacgctactatatattatc :  68800
Seq2 : aagattttaaattttaaacataaagatgatgatacgtatatacacttttgtaaaatattattcggtgtctataacggaacaaacgctactatatattatc :  68800
Seq3 : aagattttaaattttaaacataaagatgatgatacgtatatacacttttgtaaaatattattcggtgtctataacggaacaaacgctactatatattatc :  68800
Seq4 : aagattttaaattttaaacataaagatgatgatacgtatatacacttttgtaaaatattattcggtgtctataacggaacaaacgctactatatattatc :  68800

                *     68820         *     68840         *     68860         *     68880         *     68900
Seq1 : atagacctctaacgggatatatgaatatgatttcagatactatatttgttcctgtagataataactaaaaatcaaactctaatgaccacatcttttttta :  68900
Seq2 : atagacctctaacgggatatatgaatatgatttcagatactatatttgttcctgtagataataactaaaaatcaaactctaatgaccacatcttttttta :  68900
Seq3 : atagacctctaacgggatatatgaatatgatttcagatactatatttgttcctgtagataataactaaaaatcaaactctaatgaccacatcttttttta :  68900
Seq4 : atagacctctaacgggatatatgaatatgatttcagatactatatttgttcctgtagataataactaaaaatcaaactctaatgaccacatcttttttta :  68900

                *     68920         *     68940         *     68960         *     68980         *     69000
Seq1 : gagatgaaaaatttctacatctccttttgtagacacgactaaacattttgcaaaaaaaagtttattagtgtttagataatcgtatacttcatcagtgta :  69000
Seq2 : gagatgaaaaatttctacatctccttttgtagacacgactaaacattttgcaaaaaaaagtttattagtgtttagataatcgtatacttcatcagtgta :  69000
Seq3 : gagatgaaaaatttctacatctccttttgtagacacgactaaacattttgcaaaaaaaagtttattagtgtttagataatcgtatacttcatcagtgta :  69000
Seq4 : gagatgaaaaatttctacatctccttttgtagacacgactaaacattttgcaaaaaaaagtttattagtgtttagataatcgtatacttcatcagtgta :  69000

                *     69020         *     69040         *     69060         *     69080         *     69100
Seq1 : gatagtaaatgtgaacaaataaaaggtattcttactcaatagattggtaaattccatagaatatattaatcctttcttcttgagatcccacatcatttca :  69100
Seq2 : gatagtaaatgtgaacaaataaaaggtattcttactcaatagattggtaaattccatagaatatattaatcctttcttcttgagatcccacatcatttca :  69100
Seq3 : gatagtaaatgtgaacaaataaaaggtattcttactcaatagattggtaaattccatagaatatattaatcctttcttcttgagatcccacatcatttca :  69100
Seq4 : gatagtaaatgtgaacaaataaaaggtattcttactcaatagattggtaaattccatagaatatattaatcctttcttcttgagatcccacatcatttca :  69100

                *     69120         *     69140         *     69160         *     69180         *     69200
Seq1 : accagagacgttttatccaatgatttacctcgtactataccacatacaaaactagattttgcagtgacgtcgtacctggtattcctaccaaacaaaattt :  69200
Seq2 : accagagacgttttatccaatgatttacctcgtactataccacatacaaaactagattttgcagtgacgtcgtacctggtattcctaccaaacaaaattt :  69200
Seq3 : accagagacgttttatccaatgatttacctcgtactataccacatacaaaactagattttgcagtgacgtcgtacctggtattcctaccaaacaaaattt :  69200
Seq4 : accagagacgttttatccaatgatttacctcgtactataccacatacaaaactagattttgcagtgacgtcgtacctggtattcctaccaaacaaaattt :  69200

                *     69220         *     69240         *     69260         *     69280         *     69300
Seq1 : tacttttagttcttttagaaaattctaaggtagaatctctatttgccaatatgtcatctatggaattaccactagcaaaaaatgatagaaatatatattg :  69300
Seq2 : tacttttagttcttttagaaaattctaaggtagaatctctatttgccaatatgtcatctatggaattaccactagcaaaaaatgatagaaatatatattg :  69300
Seq3 : tacttttagttcttttagaaaattctaaggtagaatctctatttgccaatatgtcatctatggaattaccactagcaaaaaatgatagaaatatatattg :  69300
Seq4 : tacttttagttcttttagaaaattctaaggtagaatctctatttgccaatatgtcatctatggaattaccactagcaaaaaatgatagaaatatatattg :  69300
```

```
               *     69320          *     69340          *     69360          *     69380          *     69400
Seq1 : atacatcgcagctggttttgatctactatactttaaaaacgaatcagattccataattgcctgtatatcatcagctgaaaaactatgttttacacgtatt :  69400
Seq2 : atacatcgcagctggttttgatctactatactttaaaaacgaatcagattccataattgcctgtatatcatcagctgaaaaactatgttttacacgtatt :  69400
Seq3 : atacatcgcagctggttttgatctactatactttaaaaacgaatcagattccataattgcctgtatatcatcagctgaaaaactatgttttacacgtatt :  69400
Seq4 : atacatcgcagctggttttgatctactatactttaaaaacgaatcagattccataattgcctgtatatcatcagctgaaaaactatgttttacacgtatt :  69400

               *     69420          *     69440          *     69460          *     69480          *     69500
Seq1 : ccttcggcatttctttttaatgatatatcttgtttagacaatgataaagttatcatgtccatgagagacgcgtctccgtatcgtataaatatttcattag :  69500
Seq2 : ccttcggcatttctttttaatgatatatcttgtttagacaatgataaagttatcatgtccatgagagacgcgtctccgtatcgtataaatatttcattag :  69500
Seq3 : ccttcggcatttctttttaatgatatatcttgtttagacaatgataaagttatcatgtccatgagagacgcgtctccgtatcgtataaatatttcattag :  69500
Seq4 : ccttcggcatttctttttaatgatatatcttgtttagacaatgataaagttatcatgtccatgagagacgcgtctccgtatcgtataaatatttcattag :  69500

               *     69520          *     69540          *     69560          *     69580          *     69600
Seq1 : atgttagacgcttcattaggggtatacttctataaggtttcttaatcagtccatcattggttgcgtcaagaactactatcggatgttgttgggtatctct :  69600
Seq2 : atgttagacgcttcattaggggtatacttctataaggtttcttaatcagtccatcattggttgcgtcaagaactactatcggatgttgttgggtatctct :  69600
Seq3 : atgttagacgcttcattaggggtatacttctataaggtttcttaatcagtccatcattggttgcgtcaagaactactatcggatgttgttgggtatctct :  69600
Seq4 : atgttagacgcttcattaggggtatacttctataaggtttcttaatcagtccatcattggttgcgtcaagaactactatcggatgttgttgggtatctct :  69600

               *     69620          *     69640          *     69660          *     69680          *     69700
Seq1 : agtgttacacatggccttactaaagtttgggtaaataactatgatatctctattaattatagatgcatatatttcattcgtcaaggatattagtatcgac :  69700
Seq2 : agtgttacacatggccttactaaagtttgggtaaataactatgatatctctattaattatagatgcatatatttcattcgtcaaggatattagtatcgac :  69700
Seq3 : agtgttacacatggccttactaaagtttgggtaaataactatgatatctctattaattatagatgcatatatttcattcgtcaaggatattagtatcgac :  69700
Seq4 : agtgttacacatggccttactaaagtttgggtaaataactatgatatctctattaattatagatgcatatatttcattcgtcaaggatattagtatcgac :  69700

               *     69720          *     69740          *     69760          *     69780          *     69800
Seq1 : ttgctatcgtcattaatacgtgtaatgtaatcatataaatcatgcgatagccaaggaaaattcaaatagatgttcatcatataatcgtcgctataattca :  69800
Seq2 : ttgctatcgtcattaatacgtgtaatgtaatcatataaatcatgcgatagccaaggaaaattcaaatagatgttcatcatataatcgtcgctataattca :  69800
Seq3 : ttgctatcgtcattaatacgtgtaatgtaatcatataaatcatgcgatagccaaggaaaattcaaatagatgttcatcatataatcgtcgctataattca :  69800
Seq4 : ttgctatcgtcattaatacgtgtaatgtaatcatataaatcatgcgatagccaaggaaaattcaaatagatgttcatcatataatcgtcgctataattca :  69800

               *     69820          *     69840          *     69860          *     69880          *     69900
Seq1 : tattaatacgttgacattgactaatttgtaatatagcctcgccacgaagaaagctctcgtattcagtttcatcgataaaggataccgttaaatataactg :  69900
Seq2 : tattaatacgttgacattgactaatttgtaatatagcctcgccacgaagaaagctctcgtattcagtttcatcgataaaggataccgttaaatataactg :  69900
Seq3 : tattaatacgttgacattgactaatttgtaatatagcctcgccacgaagaaagctctcgtattcagtttcatcgataaaggataccgttaaatataactg :  69900
Seq4 : tattaatacgttgacattgactaatttgtaatatagcctcgccacgaagaaagctctcgtattcagtttcatcgataaaggataccgttaaatataactg :  69900

               *     69920          *     69940          *     69960          *     69980          *     70000
Seq1 : gttgccgatagtctcatagtctattaagtggtaagtttcgtacaaatacagaatccctaaaatattatctaatgttggattaatctttaccataactgta :  70000
Seq2 : gttgccgatagtctcatagtctattaagtggtaagtttcgtacaaatacagaatccctaaaatattatctaatgttggattaatctttaccataactgta :  70000
Seq3 : gttgccgatagtctcatagtctattaagtggtaagtttcgtacaaatacagaatccctaaaatattatctaatgttggattaatctttaccataactgta :  70000
Seq4 : gttgccgatagtctcatagtctattaagtggtaagtttcgtacaaatacagaatccctaaaatattatctaatgttggattaatctttaccataactgta :  70000
```

```
                 *      70020         *      70040         *      70060         *      70080         *      70100
Seq1 : taaaatggagacggagtcataactattttaccgtttgtacttactggaatagacgaaggaataatctccggacatgctggtaaagacccaaatgtctgtt :  70100
Seq2 : taaaatggagacggagtcataactattttaccgtttgtacttactggaatagacgaaggaataatctccggacatgctggtaaagacccaaatgtctgtt :  70100
Seq3 : taaaatggagacggagtcataactattttaccgtttgtacttactggaatagacgaaggaataatctccggacatgctggtaaagacccaaatgtctgtt :  70100
Seq4 : taaaatggagacggagtcataactattttaccgtttgtacttactggaatagacgaaggaataatctccggacatgctggtaaagacccaaatgtctgtt :  70100

                 *      70120         *      70140         *      70160         *      70180         *      70200
Seq1 : tgaagaaatccaatgttccaggtcctaatctcttaacaaaaattacgatattcgatcccgatatcctttgcattctatttaccagcatatcacgaactat :  70200
Seq2 : tgaagaaatccaatgttccaggtcctaatctcttaacaaaaattacgatattcgatcccgatatcctttgcattctatttaccagcatatcacgaactat :  70200
Seq3 : tgaagaaatccaatgttccaggtcctaatctcttaacaaaaattacgatattcgatcccgatatcctttgcattctatttaccagcatatcacgaactat :  70200
Seq4 : tgaagaaatccaatgttccaggtcctaatctcttaacaaaaattacgatattcgatcccgatatcctttgcattctatttaccagcatatcacgaactat :  70200

                 *      70220         *      70240         *      70260         *      70280         *      70300
Seq1 : attaagattatctatcatgtctattctcccaccgttatataaatcgcctccgctaagaaacgttagtatatccatacaatggaatacttcatttctaaaa :  70300
Seq2 : attaagattatctatcatgtctattctcccaccgttatataaatcgcctccgctaagaaacgttagtatatccatacaatggaatacttcatttctaaaa :  70300
Seq3 : attaagattatctatcatgtctattctcccaccgttatataaatcgcctccgctaagaaacgttagtatatccatacaatggaatacttcatttctaaaa :  70300
Seq4 : attaagattatctatcatgtctattctcccaccgttatataaatcgcctccgctaagaaacgttagtatatccatacaatggaatacttcatttctaaaa :  70300

                 *      70320         *      70340         *      70360         *      70380         *      70400
Seq1 : tagtattcgttttctaattctttaatgtgaaatcgtatactagaaagggaaaaattatctttgagttttccgttagaaaagaaccacgaaactaatgttc :  70400
Seq2 : tagtattcgttttctaattctttaatgtgaaatcgtatactagaaagggaaaaattatctttgagttttccgttagaaaagaaccacgaaactaatgttc :  70400
Seq3 : tagtattcgttttctaattctttaatgtgaaatcgtatactagaaagggaaaaattatctttgagttttccgttagaaaagaaccacgaaactaatgttc :  70400
Seq4 : tagtattcgttttctaattctttaatgtgaaatcgtatactagaaagggaaaaattatctttgagttttccgttagaaaagaaccacgaaactaatgttc :  70400

                 *      70420         *      70440         *      70460         *      70480         *      70500
Seq1 : tgattgcgtccgattccgttgctgaattaatggatttacaccaaaaactcatataacttctagatgtagaagcattcgctaaaaaattagtagaatcaaa :  70500
Seq2 : tgattgcgtccgattccgttgctgaattaatggatttacaccaaaaactcatataacttctagatgtagaagcattcgctaaaaaattagtagaatcaaa :  70500
Seq3 : tgattgcgtccgattccgttgctgaattaatggatttacaccaaaaactcatataacttctagatgtagaagcattcgctaaaaaattagtagaatcaaa :  70500
Seq4 : tgattgcgtccgattccgttgctgaattaatggatttacaccaaaaactcatataacttctagatgtagaagcattcgctaaaaaattagtagaatcaaa :  70500

                 *      70520         *      70540         *      70560         *      70580         *      70600
Seq1 : ggatataagtagatgttccaacaagtgagcaattcccaagatttcatctatatcattctcgaatccgaaattagaaattcccaagtagatatcctttttc :  70600
Seq2 : ggatataagtagatgttccaacaagtgagcaattcccaagatttcatctatatcattctcgaatccgaaattagaaattcccaagtagatatcctttttc :  70600
Seq3 : ggatataagtagatgttccaacaagtgagcaattcccaagatttcatctatatcattctcgaatccgaaattagaaattcccaagtagatatcctttttc :  70600
Seq4 : ggatataagtagatgttccaacaagtgagcaattcccaagatttcatctatatcattctcgaatccgaaattagaaattcccaagtagatatcctttttc :  70600

                 *      70620         *      70640         *      70660         *      70680         *      70700
Seq1 : atccgatcgttgatgaaaatacgaactttattcggtaagacaatcatttactaaggagtaaaataggaagtaatgttcgtatgtcgttatcatcgtataa :  70700
Seq2 : atccgatcgttgatgaaaatacgaactttattcggtaagacaatcatttactaaggagtaaaataggaagtaatgttcgtatgtcgttatcatcgtataa :  70700
Seq3 : atccgatcgttgatgaaaatacgaactttattcggtaagacaatcatttactaaggagtaaaataggaagtaatgttcgtatgtcgttatcatcgtataa :  70700
Seq4 : atccgatcgttgatgaaaatacgaactttattcggtaagacaatcatttactaaggagtaaaataggaagtaatgttcgtatgtcgttatcatcgtataa :  70700
```

```
               *     70720           *     70740           *     70760           *     70780           *     70800
Seq1 : attaaaggtgtgtttttaccattaagtgacattataattttaccaatattggaattataatataggtgtatttgcgcactcgcgacggttgatgcatcg :  70800
Seq2 : attaaaggtgtgtttttaccattaagtgacattataattttaccaatattggaattataatataggtgtatttgcgcactcgcgacggttgatgcatcg :  70800
Seq3 : attaaaggtgtgtttttaccattaagtgacattataattttaccaatattggaattataatataggtgtatttgcgcactcgcgacggttgatgcatcg :  70800
Seq4 : attaaaggtgtgtttttaccattaagtgacattataattttaccaatattggaattataatataggtgtatttgcgcactcgcgacggttgatgcatcg :  70800

               *     70820           *     70840           *     70860           *     70880           *     70900
Seq1 : gtaaatatagctgtatctaatgttctagtcggtatttcatcatttcgctgtctaataatagcgttttctctatctgtttccattacagctgcctgaagtt :  70900
Seq2 : gtaaatatagctgtatctaatgttctagtcggtatttcatcatttcgctgtctaataatagcgttttctctatctgtttccattacagctgcctgaagtt :  70900
Seq3 : gtaaatatagctgtatctaatgttctagtcggtatttcatcatttcgctgtctaataatagcgttttctctatctgtttccattacagctgcctgaagtt :  70900
Seq4 : gtaaatatagctgtatctaatgttctagtcggtatttcatcatttcgctgtctaataatagcgttttctctatctgtttccattacagctgcctgaagtt :  70900

               *     70920           *     70940           *     70960           *     70980           *     71000
Seq1 : tattggtcggataatatgtaaaataataagaaatacatacgaataacaaaaataaaataagatataataaagatgccatttagagatctaattttgttca :  71000
Seq2 : tattggtcggataatatgtaaaataataagaaatacatacgaataacaaaaataaaataagatataataaagatgccatttagagatctaattttgttca :  71000
Seq3 : tattggtcggataatatgtaaaataataagaaatacatacgaataacaaaaataaaataagatataataaagatgccatttagagatctaattttgttca :  71000
Seq4 : tattggtcggataatatgtaaaataataagaaatacatacgaataacaaaaataaaataagatataataaagatgccatttagagatctaattttgttca :  71000

               *     71020           *     71040           *     71060           *     71080           *     71100
Seq1 : acttgtccaaattcctacttacagaagatgaggaatcgttggagatagtgtcttccttatgtagaggatttgaaatatcttatgatgacttgataactta :  71100
Seq2 : acttgtccaaattcctacttacagaagatgaggaatcgttggagatagtgtcttccttatgtagaggatttgaaatatcttatgatgacttgataactta :  71100
Seq3 : acttgtccaaattcctacttacagaagatgaggaatcgttggagatagtgtcttccttatgtagaggatttgaaatatcttatgatgacttgataactta :  71100
Seq4 : acttgtccaaattcctacttacagaagatgaggaatcgttggagatagtgtcttccttatgtagaggatttgaaatatcttatgatgacttgataactta :  71100

               *     71120           *     71140           *     71160           *     71180           *     71200
Seq1 : ctttccagataggaaataccataaatatatttctaaagtatttgaacatgtagatttatcggaggaattaagtatggaattccatgatacaactttgcga :  71200
Seq2 : ctttccagataggaaataccataaatatatttctaaagtatttgaacatgtagatttatcggaggaattaagtatggaattccatgatacaactttgcga :  71200
Seq3 : ctttccagataggaaataccataaatatatttctaaagtatttgaacatgtagatttatcggaggaattaagtatggaattccatgatacaactttgcga :  71200
Seq4 : ctttccagataggaaataccataaatatatttctaaagtatttgaacatgtagatttatcggaggaattaagtatggaattccatgatacaactttgcga :  71200

               *     71220           *     71240           *     71260           *     71280           *     71300
Seq1 : gatttagtctatcttagattgtacaagtattccaagtgtatacggccgtgttataaattaggagataatctaaaaggcatagttgttataaaggacagga :  71300
Seq2 : gatttagtctatcttagattgtacaagtattccaagtgtatacggccgtgttataaattaggagataatctaaaaggcatagttgttataaaggacagga :  71300
Seq3 : gatttagtctatcttagattgtacaagtattccaagtgtatacggccgtgttataaattaggagataatctaaaaggcatagttgttataaaggacagga :  71300
Seq4 : gatttagtctatcttagattgtacaagtattccaagtgtatacggccgtgttataaattaggagataatctaaaaggcatagttgttataaaggacagga :  71300

               *     71320           *     71340           *     71360           *     71380           *     71400
Seq1 : atatttatattagggaagcaaatgatgacttgatagaatatctcctcaaggaatacactcctcagatttatacatattctaatgagcgcgtccccataac :  71400
Seq2 : atatttatattagggaagcaaatgatgacttgatagaatatctcctcaaggaatacactcctcagatttatacatattctaatgagcgcgtccccataac :  71400
Seq3 : atatttatattagggaagcaaatgatgacttgatagaatatctcctcaaggaatacactcctcagatttatacatattctaatgagcgcgtccccataac :  71400
Seq4 : atatttatattagggaagcaaatgatgacttgatagaatatctcctcaaggaatacactcctcagatttatacatattctaatgagcgcgtccccataac :  71400
```

```
               *     71420          *     71440          *     71460          *     71480          *     71500
Seq1 : tggttcaaaattaattctttgtggattttctcaagttacatttatggcgtatacaacgtcgcatataacaacaaataaaaaggtagatgttctcgtttcc :  71500
Seq2 : tggttcaaaattaattctttgtggattttctcaagttacatttatggcgtatacaacgtcgcatataacaacaaataaaaaggtagatgttctcgtttcc :  71500
Seq3 : tggttcaaaattaattctttgtggattttctcaagttacatttatggcgtatacaacgtcgcatataacaacaaataaaaaggtagatgttctcgtttcc :  71500
Seq4 : tggttcaaaattaattctttgtggattttctcaagttacatttatggcgtatacaacgtcgcatataacaacaaataaaaaggtagatgttctcgtttcc :  71500

               *     71520          *     71540          *     71560          *     71580          *     71600
Seq1 : aaaaaatgtatagatgaactagtcgatccaataaattatcaaatacttcaaaatttatttgataaaggaagcggaacaataaacaaaatactcaggaaga :  71600
Seq2 : aaaaaatgtatagatgaactagtcgatccaataaattatcaaatacttcaaaatttatttgataaaggaagcggaacaataaacaaaatactcaggaaga :  71600
Seq3 : aaaaaatgtatagatgaactagtcgatccaataaattatcaaatacttcaaaatttatttgataaaggaagcggaacaataaacaaaatactcaggaaga :  71600
Seq4 : aaaaaatgtatagatgaactagtcgatccaataaattatcaaatacttcaaaatttatttgataaaggaagcggaacaataaacaaaatactcaggaaga :  71600

               *     71620          *     71640          *     71660          *     71680          *     71700
Seq1 : tattttattcggtaacaggtggccaaactccataggtagctttttctatttcggattttagaatttccaaattcaccagcgatttatctgttttggtgaa :  71700
Seq2 : tattttattcggtaacaggtggccaaactccataggtagctttttctatttcggattttagaatttccaaattcaccagcgatttatctgttttggtgaa :  71700
Seq3 : tattttattcggtaacaggtggccaaactccataggtagctttttctatttcggattttagaatttccaaattcaccagcgatttatctgttttggtgaa :  71700
Seq4 : tattttattcggtaacaggtggccaaactccataggtagctttttctatttcggattttagaatttccaaattcaccagcgatttatctgttttggtgaa :  71700

               *     71720          *     71740          *     71760          *     71780          *     71800
Seq1 : atccaaggatttattaatgtccacaaatgccatttgttttgtctgtggattgtatttgaaaatggaaacgatgtagttagatagatgcgctgcgaagttt :  71800
Seq2 : atccaaggatttattaatgtccacaaatgccatttgttttgtctgtggattgtatttgaaaatggaaacgatgtagttagatagatgcgctgcgaagttt :  71800
Seq3 : atccaaggatttattaatgtccacaaatgccatttgttttgtctgtggattgtatttgaaaatggaaacgatgtagttagatagatgcgctgcgaagttt :  71800
Seq4 : atccaaggatttattaatgtccacaaatgccatttgttttgtctgtggattgtatttgaaaatggaaacgatgtagttagatagatgcgctgcgaagttt :  71800

               *     71820          *     71840          *     71860          *     71880          *     71900
Seq1 : cctattagggttccgcgcttcacgtcacccagcatacttgaatcaccatcctttaaaaaaaatgataagatatcaacatggagtatatcatactcggatt :  71900
Seq2 : cctattagggttccgcgcttcacgtcacccagcatacttgaatcaccatcctttaaaaaaaatgataagatatcaacatggagtatatcatactcggatt :  71900
Seq3 : cctattagggttccgcgcttcacgtcacccagcatacttgaatcaccatcctttaaaaaaaatgataagatatcaacatggagtatatcatactcggatt :  71900
Seq4 : cctattagggttccgcgcttcacgtcacccagcatacttgaatcaccatcctttaaaaaaaatgataagatatcaacatggagtatatcatactcggatt :  71900

               *     71920          *     71940          *     71960          *     71980          *     72000
Seq1 : ttaattcttctactgcatcactgacattttcacaaatactacaatacggtttaccgaaaataatcagtacgttcttcatttatgggtatcaaaaacttaa :  72000
Seq2 : ttaattcttctactgcatcactgacattttcacaaatactacaatacggtttaccgaaaataatcagtacgttcttcatttatgggtatcaaaaacttaa :  72000
Seq3 : ttaattcttctactgcatcactgacattttcacaaatactacaatacggtttaccgaaaataatcagtacgttcttcatttatgggtatcaaaaacttaa :  72000
Seq4 : ttaattcttctactgcatcactgacattttcacaaatactacaatacggtttaccgaaaataatcagtacgttcttcatttatgggtatcaaaaacttaa :  72000

               *     72020          *     72040          *     72060          *     72080          *     72100
Seq1 : aatcgttactgctggaaaataaatcactgacgatattagatgataatttatacaaagtatacaatggaatatttgtggatacaatgagtatttatatagc :  72100
Seq2 : aatcgttactgctggaaaataaatcactgacgatattagatgataatttatacaaagtatacaatggaatatttgtggatacaatgagtatttatatagc :  72100
Seq3 : aatcgttactgctggaaaataaatcactgacgatattagatgataatttatacaaagtatacaatggaatatttgtggatacaatgagtatttatatagc :  72100
Seq4 : aatcgttactgctggaaaataaatcactgacgatattagatgataatttatacaaagtatacaatggaatatttgtggatacaatgagtatttatatagc :  72100
```

```
               *     72120          *     72140          *     72160          *     72180          *     72200
Seq1 : cgtcgccaattgtgtcagaaacttagaagagttaactacggtattcataaaatacgtaaacggatgggtaaaaaagggagggcatgtaaccctttttatc :  72200
Seq2 : cgtcgccaattgtgtcagaaacttagaagagttaactacggtattcataaaatacgtaaacggatgggtaaaaaagggagggcatgtaaccctttttatc :  72200
Seq3 : cgtcgccaattgtgtcagaaacttagaagagttaactacggtattcataaaatacgtaaacggatgggtaaaaaagggagggcatgtaaccctttttatc :  72200
Seq4 : cgtcgccaattgtgtcagaaacttagaagagttaactacggtattcataaaatacgtaaacggatgggtaaaaaagggagggcatgtaaccctttttatc :  72200

               *     72220          *     72240          *     72260          *     72280          *     72300
Seq1 : gatagaggaagtataaaaattaaacaagacgttagagacaagagacgtaaatattctaaattaaccaaggacagaaaaatgctagaattagaaaagtgta :  72300
Seq2 : gatagaggaagtataaaaattaaacaagacgttagagacaagagacgtaaatattctaaattaaccaaggacagaaaaatgctagaattagaaaagtgta :  72300
Seq3 : gatagaggaagtataaaaattaaacaagacgttagagacaagagacgtaaatattctaaattaaccaaggacagaaaaatgctagaattagaaaagtgta :  72300
Seq4 : gatagaggaagtataaaaattaaacaagacgttagagacaagagacgtaaatattctaaattaaccaaggacagaaaaatgctagaattagaaaagtgta :  72300

               *     72320          *     72340          *     72360          *     72380          *     72400
Seq1 : catccgaaatacaaaatgttaccggatttatggaagaagaaataaaggcagaaatgcaattaaaaatcgataaactcacatttcaaatatatttatctga :  72400
Seq2 : catccgaaatacaaaatgttaccggatttatggaagaagaaataaaggcagaaatgcaattaaaaatcgataaactcacatttcaaatatatttatctga :  72400
Seq3 : catccgaaatacaaaatgttaccggatttatggaagaagaaataaaggcagaaatgcaattaaaaatcgataaactcacatttcaaatatatttatctga :  72400
Seq4 : catccgaaatacaaaatgttaccggatttatggaagaagaaataaaggcagaaatgcaattaaaaatcgataaactcacatttcaaatatatttatctga :  72400

               *     72420          *     72440          *     72460          *     72480          *     72500
Seq1 : ttctgataacataaaaatatcattgaatgagatactaacacatttcaacaataatgagaatgttacattattttattgtgatgaacgagacgcagaattc :  72500
Seq2 : ttctgataacataaaaatatcattgaatgagatactaacacatttcaacaataatgagaatgttacattattttattgtgatgaacgagacgcagaattc :  72500
Seq3 : ttctgataacataaaaatatcattgaatgagatactaacacatttcaacaataatgagaatgttacattattttattgtgatgaacgagacgcagaattc :  72500
Seq4 : ttctgataacataaaaatatcattgaatgagatactaacacatttcaacaataatgagaatgttacattattttattgtgatgaacgagacgcagaattc :  72500

               *     72520          *     72540          *     72560          *     72580          *     72600
Seq1 : gttatgtgtctcgaggctaaaacacatttctctaccacaggagaatggccgttgataataagtaccgatcaggatactatgctatttgcatctgctgata :  72600
Seq2 : gttatgtgtctcgaggctaaaacacatttctctaccacaggagaatggccgttgataataagtaccgatcaggatactatgctatttgcatctgctgata :  72600
Seq3 : gttatgtgtctcgaggctaaaacacatttctctaccacaggagaatggccgttgataataagtaccgatcaggatactatgctatttgcatctgctgata :  72600
Seq4 : gttatgtgtctcgaggctaaaacacatttctctaccacaggagaatggccgttgataataagtaccgatcaggatactatgctatttgcatctgctgata :  72600

               *     72620          *     72640          *     72660          *     72680          *     72700
Seq1 : atcatcctaagatgataaaaaacttaactcaactgtttaaatatgttccatctgcagaggataactatttagcaaaattaacggcgttagtgaatggatg :  72700
Seq2 : atcatcctaagatgataaaaaacttaactcaactgtttaaatatgttccatctgcagaggataactatttagcaaaattaacggcgttagtgaatggatg :  72700
Seq3 : atcatcctaagatgataaaaaacttaactcaactgtttaaatatgttccatctgcagaggataactatttagcaaaattaacggcgttagtgaatggatg :  72700
Seq4 : atcatcctaagatgataaaaaacttaactcaactgtttaaatatgttccatctgcagaggataactatttagcaaaattaacggcgttagtgaatggatg :  72700

               *     72720          *     72740          *     72760          *     72780          *     72800
Seq1 : tgatttctttcctggactctatggggcatctataacacccaacaacttaaacaaaatacaattgtttagtgattttacaatcgataatatagtcactagt :  72800
Seq2 : tgatttctttcctggactctatggggcatctataacacccaacaacttaaacaaaatacaattgtttagtgattttacaatcgataatatagtcactagt :  72800
Seq3 : tgatttctttcctggactctatggggcatctataacacccaacaacttaaacaaaatacaattgtttagtgattttacaatcgataatatagtcactagt :  72800
Seq4 : tgatttctttcctggactctatggggcatctataacacccaacaacttaaacaaaatacaattgtttagtgattttacaatcgataatatagtcactagt :  72800
```

```
               *     72820           *     72840           *     72860           *     72880           *     72900
Seq1 : ttggcaattaaaaattattatagaaagactaactctaccgtagacgtgcgtaatattgttacgtttataaacgattacgctaatttagacgatgtctact :  72900
Seq2 : ttggcaattaaaaattattatagaaagactaactctaccgtagacgtgcgtaatattgttacgtttataaacgattacgctaatttagacgatgtctact :  72900
Seq3 : ttggcaattaaaaattattatagaaagactaactctaccgtagacgtgcgtaatattgttacgtttataaacgattacgctaatttagacgatgtctact :  72900
Seq4 : ttggcaattaaaaattattatagaaagactaactctaccgtagacgtgcgtaatattgttacgtttataaacgattacgctaatttagacgatgtctact :  72900

               *     72920           *     72940           *     72960           *     72980           *     73000
Seq1 : cgtatattcctccttgtcaatgcactgttcaagaatttatattttccgcattagatgaaaaatggaatgaatttaaatcatcttatttagaaagcgtgcc :  73000
Seq2 : cgtatattcctccttgtcaatgcactgttcaagaatttatattttccgcattagatgaaaaatggaatgaatttaaatcatcttatttagaaagcgtgcc :  73000
Seq3 : cgtatattcctccttgtcaatgcactgttcaagaatttatattttccgcattagatgaaaaatggaatgaatttaaatcatcttatttagaaagcgtgcc :  73000
Seq4 : cgtatattcctccttgtcaatgcactgttcaagaatttatattttccgcattagatgaaaaatggaatgaatttaaatcatcttatttagaaagcgtgcc :  73000

               *     73020           *     73040           *     73060           *     73080           *     73100
Seq1 : gttaccctgccaattaatgtatgcgttagaaccacgcaaggagattgatgtttcagaagttaaaactttatcatcttatatagatttcgaaaatactaaa :  73100
Seq2 : gttaccctgccaattaatgtatgcgttagaaccacgcaaggagattgatgtttcagaagttaaaactttatcatcttatatagatttcgaaaatactaaa :  73100
Seq3 : gttaccctgccaattaatgtatgcgttagaaccacgcaaggagattgatgtttcagaagttaaaactttatcatcttatatagatttcgaaaatactaaa :  73100
Seq4 : gttaccctgccaattaatgtatgcgttagaaccacgcaaggagattgatgtttcagaagttaaaactttatcatcttatatagatttcgaaaatactaaa :  73100

               *     73120           *     73140           *     73160           *     73180           *     73200
Seq1 : tcagatatcgatgttataaaatctatatcctcgatcttcggatattctaacgaaaactgtaacacgatagtattcggcatctataaggataatttactac :  73200
Seq2 : tcagatatcgatgttataaaatctatatcctcgatcttcggatattctaacgaaaactgtaacacgatagtattcggcatctataaggataatttactac :  73200
Seq3 : tcagatatcgatgttataaaatctatatcctcgatcttcggatattctaacgaaaactgtaacacgatagtattcggcatctataaggataatttactac :  73200
Seq4 : tcagatatcgatgttataaaatctatatcctcgatcttcggatattctaacgaaaactgtaacacgatagtattcggcatctataaggataatttactac :  73200

               *     73220           *     73240           *     73260           *     73280           *     73300
Seq1 : tgagtataaatagttcattttactttaacgatagtctgttaataaccaatactaaaagtgataatataataaatataggttactagattaaaaatggtgt :  73300
Seq2 : tgagtataaatagttcattttactttaacgatagtctgttaataaccaatactaaaagtgataatataataaatataggttactagattaaaaatggtgt :  73300
Seq3 : tgagtataaatagttcattttactttaacgatagtctgttaataaccaatactaaaagtgataatataataaatataggttactagattaaaaatggtgt :  73300
Seq4 : tgagtataaatagttcattttactttaacgatagtctgttaataaccaatactaaaagtgataatataataaatataggttactagattaaaaatggtgt :  73300

               *     73320           *     73340           *     73360           *     73380           *     73400
Seq1 : tccaactcgtgtgctctacgtgcggcaaagatatttctcacgaacgatataaattgattatacgaaaaaaatcattaaaggatgtactcgtcagtgtaaa :  73400
Seq2 : tccaactcgtgtgctctacgtgcggcaaagatatttctcacgaacgatataaattgattatacgaaaaaaatcattaaaggatgtactcgtcagtgtaaa :  73400
Seq3 : tccaactcgtgtgctctacgtgcggcaaagatatttctcacgaacgatataaattgattatacgaaaaaaatcattaaaggatgtactcgtcagtgtaaa :  73400
Seq4 : tccaactcgtgtgctctacgtgcggcaaagatatttctcacgaacgatataaattgattatacgaaaaaaatcattaaaggatgtactcgtcagtgtaaa :  73400

               *     73420           *     73440           *     73460           *     73480           *     73500
Seq1 : gaacgaatgttgtaggttaaaattatctacacaaatagaacctcaacgtaacttaacagtgcaacctctattggatataaactaatatggatccggttaa :  73500
Seq2 : gaacgaatgttgtaggttaaaattatctacacaaatagaacctcaacgtaacttaacagtgcaacctctattggatataaactaatatggatccggttaa :  73500
Seq3 : gaacgaatgttgtaggttaaaattatctacacaaatagaacctcaacgtaacttaacagtgcaacctctattggatataaactaatatggatccggttaa :  73500
Seq4 : gaacgaatgttgtaggttaaaattatctacacaaatagaacctcaacgtaacttaacagtgcaacctctattggatataaactaatatggatccggttaa :  73500
```

```
                *     73520         *     73540         *     73560         *     73580         *     73600
Seq1 : ttttatcaagacatatgcgcctagaggttctattatttttattaattataccatgtcattaacaagtcatttgaatccatcgatagaaaaacatgtgggt :  73600
Seq2 : ttttatcaagacatatgcgcctagaggttctattatttttattaattataccatgtcattaacaagtcatttgaatccatcgatagaaaaacatgtgggt :  73600
Seq3 : ttttatcaagacatatgcgcctagaggttctattatttttattaattataccatgtcattaacaagtcatttgaatccatcgatagaaaaacatgtgggt :  73600
Seq4 : ttttatcaagacatatgcgcctagaggttctattatttttattaattataccatgtcattaacaagtcatttgaatccatcgatagaaaaacatgtgggt :  73600

                *     73620         *     73640         *     73660         *     73680         *     73700
Seq1 : atttattatggtacgttattatcggaacacttggtagttgaatcaacatatagaaaaggagttcgaatagtcccattggatagtttttttgaaggatatc :  73700
Seq2 : atttattatggtacgttattatcggaacacttggtagttgaatcaacatatagaaaaggagttcgaatagtcccattggatagtttttttgaaggatatc :  73700
Seq3 : atttattatggtacgttattatcggaacacttggtagttgaatcaacatatagaaaaggagttcgaatagtcccattggatagtttttttgaaggatatc :  73700
Seq4 : atttattatggtacgttattatcggaacacttggtagttgaatcaacatatagaaaaggagttcgaatagtcccattggatagtttttttgaaggatatc :  73700

                *     73720         *     73740         *     73760         *     73780         *     73800
Seq1 : ttagtgcaaaagtatacatgttagagaatattcaagttatgaaaatagcagctgatacgtcattaactttattgggtattccgtatggatttggtcatga :  73800
Seq2 : ttagtgcaaaagtatacatgttagagaatattcaagttatgaaaatagcagctgatacgtcattaactttattgggtattccgtatggatttggtcatga :  73800
Seq3 : ttagtgcaaaagtatacatgttagagaatattcaagttatgaaaatagcagctgatacgtcattaactttattgggtattccgtatggatttggtcatga :  73800
Seq4 : ttagtgcaaaagtatacatgttagagaatattcaagttatgaaaatagcagctgatacgtcattaactttattgggtattccgtatggatttggtcatga :  73800

                *     73820         *     73840         *     73860         *     73880         *     73900
Seq1 : tagaatgtattgttttaaattggtagctgactgttataaaaatgccggtattgatacatcgtctaaacgaatattaggtaaagatatttttctgagccaa :  73900
Seq2 : tagaatgtattgttttaaattggtagctgactgttataaaaatgccggtattgatacatcgtctaaacgaatattaggtaaagatatttttctgagccaa :  73900
Seq3 : tagaatgtattgttttaaattggtagctgactgttataaaaatgccggtattgatacatcgtctaaacgaatattaggtaaagatatttttctgagccaa :  73900
Seq4 : tagaatgtattgttttaaattggtagctgactgttataaaaatgccggtattgatacatcgtctaaacgaatattaggtaaagatatttttctgagccaa :  73900

                *     73920         *     73940         *     73960         *     73980         *     74000
Seq1 : aacttcacagacgataatagatggataaagatatatgattctaataatttaacattttggcaaattgattaccttaaagggtgagttaatatgcataact :  74000
Seq2 : aacttcacagacgataatagatggataaagatatatgattctaataatttaacattttggcaaattgattaccttaaagggtgagttaatatgcataact :  74000
Seq3 : aacttcacagacgataatagatggataaagatatatgattctaataatttaacattttggcaaattgattaccttaaagggtgagttaatatgcataact :  74000
Seq4 : aacttcacagacgataatagatggataaagatatatgattctaataatttaacattttggcaaattgattaccttaaagggtgagttaatatgcataact :  74000

                *     74020         *     74040         *     74060         *     74080         *     74100
Seq1 : actcctccgttgtttttttccctcgttctttttcttaacgttgtttgccatcactctcataatgtaaagatattctaaaatggtaaactttttgcatatcgg :  74100
Seq2 : actcctccgttgtttttttccctcgttctttttcttaacgttgtttgccatcactctcataatgtaaagatattctaaaatggtaaactttttgcatatcgg :  74100
Seq3 : actcctccgttgtttttttccctcgttctttttcttaacgttgtttgccatcactctcataatgtaaagatattctaaaatggtaaactttttgcatatcgg :  74100
Seq4 : actcctccgttgtttttttccctcgttctttttcttaacgttgtttgccatcactctcataatgtaaagatattctaaaatggtaaactttttgcatatcgg :  74100

                *     74120         *     74140         *     74160         *     74180         *     74200
Seq1 : acgcagaaattggtataaatgttgtaattgtattatttcccgtcaatggactagtcacagctccatcagttttatatcctttagagtatttctcactcgt :  74200
Seq2 : acgcagaaattggtataaatgttgtaattgtattatttcccgtcaatggactagtcacagctccatcagttttatatcctttagagtatttctcactcgt :  74200
Seq3 : acgcagaaattggtataaatgttgtaattgtattatttcccgtcaatggactagtcacagctccatcagttttatatcctttagagtatttctcactcgt :  74200
Seq4 : acgcagaaattggtataaatgttgtaattgtattatttcccgtcaatggactagtcacagctccatcagttttatatcctttagagtatttctcactcgt :  74200
```

```
                 *     74220          *     74240          *     74260          *     74280          *     74300
Seq1 : gtctaacattctagagcattccatgatctgtttatcgttgatattggccggaaagatagatttttatttttattatattactattggcaattgtagat :  74300
Seq2 : gtctaacattctagagcattccatgatctgtttatcgttgatattggccggaaagatagatttttatttttattatattactattggcaattgtagat :  74300
Seq3 : gtctaacattctagagcattccatgatctgtttatcgttgatattggccggaaagatagatttttatttttattatattactattggcaattgtagat :  74300
Seq4 : gtctaacattctagagcattccatgatctgtttatcgttgatattggccggaaagatagatttttatttttattatattactattggcaattgtagat :  74300

                 *     74320          *     74340          *     74360          *     74380          *     74400
Seq1 : ataacttctggtaaatatttttctacctttcaatctcttctattttcaagccggctatatattctgctatattgttgctagtatcaatacctttctgg :  74400
Seq2 : ataacttctggtaaatatttttctacctttcaatctcttctattttcaagccggctatatattctgctatattgttgctagtatcaatacctttctgg :  74400
Seq3 : ataacttctggtaaatatttttctacctttcaatctcttctattttcaagccggctatatattctgctatattgttgctagtatcaatacctttctgg :  74400
Seq4 : ataacttctggtaaatatttttctacctttcaatctcttctattttcaagccggctatatattctgctatattgttgctagtatcaatacctttctgg :  74400

                 *     74420          *     74440          *     74460          *     74480          *     74500
Seq1 : ctaagaagtcatatgtggtattcactatatcagttttaactggtagttccattagcctttccacttctgcagaataatcagaaattggttctttaccaga :  74500
Seq2 : ctaagaagtcatatgtggtattcactatatcagttttaactggtagttccattagcctttccacttctgcagaataatcagaaattggttctttaccaga :  74500
Seq3 : ctaagaagtcatatgtggtattcactatatcagttttaactggtagttccattagcctttccacttctgcagaataatcagaaattggttctttaccaga :  74500
Seq4 : ctaagaagtcatatgtggtattcactatatcagttttaactggtagttccattagcctttccacttctgcagaataatcagaaattggttctttaccaga :  74500

                 *     74520          *     74540          *     74560          *     74580          *     74600
Seq1 : aaatccagctactataataggctcaccgatgatcattggcaaaatcctatattgtaccagattaatgagagcatatttcatttccaataattctgctagt :  74600
Seq2 : aaatccagctactataataggctcaccgatgatcattggcaaaatcctatattgtaccagattaatgagagcatatttcatttccaataattctgctagt :  74600
Seq3 : aaatccagctactataataggctcaccgatgatcattggcaaaatcctatattgtaccagattaatgagagcatatttcatttccaataattctgctagt :  74600
Seq4 : aaatccagctactataataggctcaccgatgatcattggcaaaatcctatattgtaccagattaatgagagcatatttcatttccaataattctgctagt :  74600

                 *     74620          *     74640          *     74660          *     74680          *     74700
Seq1 : tcttgagacattgatttatttgatgaatctagttggttctctagatactctaccatttctgccgcatacaataacttgttagataaaatcagggttatca :  74700
Seq2 : tcttgagacattgatttatttgatgaatctagttggttctctagatactctaccatttctgccgcatacaataacttgttagataaaatcagggttatca :  74700
Seq3 : tcttgagacattgatttatttgatgaatctagttggttctctagatactctaccatttctgccgcatacaataacttgttagataaaatcagggttatca :  74700
Seq4 : tcttgagacattgatttatttgatgaatctagttggttctctagatactctaccatttctgccgcatacaataacttgttagataaaatcagggttatca :  74700

                 *     74720          *     74740          *     74760          *     74780          *     74800
Seq1 : aagtgtttagcgtggctagaatagtgggcttgcatgtattaaagaatgcggtagtatgagtaaaccgttttaacgaattatatagtctccagaaatctgt :  74800
Seq2 : aagtgtttagcgtggctagaatagtgggcttgcatgtattaaagaatgcggtagtatgagtaaaccgttttaacgaattatatagtctccagaaatctgt :  74800
Seq3 : aagtgtttagcgtggctagaatagtgggcttgcatgtattaaagaatgcggtagtatgagtaaaccgttttaacgaattatatagtctccagaaatctgt :  74800
Seq4 : aagtgtttagcgtggctagaatagtgggcttgcatgtattaaagaatgcggtagtatgagtaaaccgttttaacgaattatatagtctccagaaatctgt :  74800

                 *     74820          *     74840          *     74860          *     74880          *     74900
Seq1 : ggcgttacatacatgagccgaatgacatcgaagattgtccaatattttaatagctgctctttgtccattatttctatatttgactcgcaacaattgtag :  74900
Seq2 : ggcgttacatacatgagccgaatgacatcgaagattgtccaatattttaatagctgctctttgtccattatttctatatttgactcgcaacaattgtag :  74900
Seq3 : ggcgttacatacatgagccgaatgacatcgaagattgtccaatattttaatagctgctctttgtccattatttctatatttgactcgcaacaattgtag :  74900
Seq4 : ggcgttacatacatgagccgaatgacatcgaagattgtccaatattttaatagctgctctttgtccattatttctatatttgactcgcaacaattgtag :  74900
```

```
                *     74920         *     74940         *     74960         *     74980         *     75000
Seq1 : ataccattaatcaccgattcctttttcgatgccggacaatagcacaattgtttagctttggactctatgtattcagaattaatagatatatctcttaata :  75000
Seq2 : ataccattaatcaccgattcctttttcgatgccggacaatagcacaattgtttagctttggactctatgtattcagaattaatagatatatctcttaata :  75000
Seq3 : ataccattaatcaccgattcctttttcgatgccggacaatagcacaattgtttagctttggactctatgtattcagaattaatagatatatctcttaata :  75000
Seq4 : ataccattaatcaccgattcctttttcgatgccggacaatagcacaattgtttagctttggactctatgtattcagaattaatagatatatctcttaata :  75000

                *     75020         *     75040         *     75060         *     75080         *     75100
Seq1 : cagattgcactatacattttgaaactatgtcaaaaattgtagaacgacgctgttctgcagccatttaactttaaataatttacaaaaatttaaaatgagc :  75100
Seq2 : cagattgcactatacattttgaaactatgtcaaaaattgtagaacgacgctgttctgcagccatttaactttaaataatttacaaaaatttaaaatgagc :  75100
Seq3 : cagattgcactatacattttgaaactatgtcaaaaattgtagaacgacgctgttctgcagccatttaactttaaataatttacaaaaatttaaaatgagc :  75100
Seq4 : cagattgcactatacattttgaaactatgtcaaaaattgtagaacgacgctgttctgcagccatttaactttaaataatttacaaaaatttaaaatgagc :  75100

                *     75120         *     75140         *     75160         *     75180         *     75200
Seq1 : atccgtataaaaatcgataaactgcgccaaattgtggcatatttttcagagttcagtgaagaagtatctataaatgtagactcgacggatgagttaatgt :  75200
Seq2 : atccgtataaaaatcgataaactgcgccaaattgtggcatatttttcagagttcagtgaagaagtatctataaatgtagactcgacggatgagttaatgt :  75200
Seq3 : atccgtataaaaatcgataaactgcgccaaattgtggcatatttttcagagttcagtgaagaagtatctataaatgtagactcgacggatgagttaatgt :  75200
Seq4 : atccgtataaaaatcgataaactgcgccaaattgtggcatatttttcagagttcagtgaagaagtatctataaatgtagactcgacggatgagttaatgt :  75200

                *     75220         *     75240         *     75260         *     75280         *     75300
Seq1 : atatttttgccgccttgggcggatctgtaaacatttgggccattatacctctcagtgcatcagtgttctaccgcggagccgaaaatattgtgtttaatct :  75300
Seq2 : atatttttgccgccttgggcggatctgtaaacatttgggccattatacctctcagtgcatcagtgttctaccgcggagccgaaaatattgtgtttaatct :  75300
Seq3 : atatttttgccgccttgggcggatctgtaaacatttgggccattatacctctcagtgcatcagtgttctaccgcggagccgaaaatattgtgtttaatct :  75300
Seq4 : atatttttgccgccttgggcggatctgtaaacatttgggccattatacctctcagtgcatcagtgttctaccgcggagccgaaaatattgtgtttaatct :  75300

                *     75320         *     75340         *     75360         *     75380         *     75400
Seq1 : tcctgtgtccaaggtaaaatcgtgtttgtgtagttttcacaatgatgccatcatagatatagaacctgatctggaaaataatctagtaaaactttctagt :  75400
Seq2 : tcctgtgtccaaggtaaaatcgtgtttgtgtagttttcacaatgatgccatcatagatatagaacctgatctggaaaataatctagtaaaactttctagt :  75400
Seq3 : tcctgtgtccaaggtaaaatcgtgtttgtgtagttttcacaatgatgccatcatagatatagaacctgatctggaaaataatctagtaaaactttctagt :  75400
Seq4 : tcctgtgtccaaggtaaaatcgtgtttgtgtagttttcacaatgatgccatcatagatatagaacctgatctggaaaataatctagtaaaactttctagt :  75400

                *     75420         *     75440         *     75460         *     75480         *     75500
Seq1 : tatcatgtagtaagtgtcgattgtaataaggaactgatgcctattaggacagatactactatttgtctaagtatagatcaaaagaaatcttacgtgttta :  75500
Seq2 : tatcatgtagtaagtgtcgattgtaataaggaactgatgcctattaggacagatactactatttgtctaagtatagatcaaaagaaatcttacgtgttta :  75500
Seq3 : tatcatgtagtaagtgtcgattgtaataaggaactgatgcctattaggacagatactactatttgtctaagtatagatcaaaagaaatcttacgtgttta :  75500
Seq4 : tatcatgtagtaagtgtcgattgtaataaggaactgatgcctattaggacagatactactatttgtctaagtatagatcaaaagaaatcttacgtgttta :  75500

                *     75520         *     75540         *     75560         *     75580         *     75600
Seq1 : attttcacaagtatgaagaaaaatgttgtggtagaaccgtcattcatttagaatggttgttgggctttatcaagtgtattagtcagcatcagcatttggc :  75600
Seq2 : attttcacaagtatgaagaaaaatgttgtggtagaaccgtcattcatttagaatggttgttgggctttatcaagtgtattagtcagcatcagcatttggc :  75600
Seq3 : attttcacaagtatgaagaaaaatgttgtggtagaaccgtcattcatttagaatggttgttgggctttatcaagtgtattagtcagcatcagcatttggc :  75600
Seq4 : attttcacaagtatgaagaaaaatgttgtggtagaaccgtcattcatttagaatggttgttgggctttatcaagtgtattagtcagcatcagcatttggc :  75600
```

```
             *     75620         *     75640         *     75660         *     75680         *     75700
Seq1 : tattatgtttaaagatgacaatattattatgaagactcctggtaatactgatgcattttccagggaatattctatgactgaatgttctcaagaactacaa :  75700
Seq2 : tattatgtttaaagatgacaatattattatgaagactcctggtaatactgatgcattttccagggaatattctatgactgaatgttctcaagaactacaa :  75700
Seq3 : tattatgtttaaagatgacaatattattatgaagactcctggtaatactgatgcattttccagggaatattctatgactgaatgttctcaagaactacaa :  75700
Seq4 : tattatgtttaaagatgacaatattattatgaagactcctggtaatactgatgcattttccagggaatattctatgactgaatgttctcaagaactacaa :  75700

             *     75720         *     75740         *     75760         *     75780         *     75800
Seq1 : aagttttctttcaaaatagctatctcgtctctcaacaaactacgaggattcaaaaagagagtcaatgtttttgaaactagaatcgtaatggataatgacg :  75800
Seq2 : aagttttctttcaaaatagctatctcgtctctcaacaaactacgaggattcaaaaagagagtcaatgtttttgaaactagaatcgtaatggataatgacg :  75800
Seq3 : aagttttctttcaaaatagctatctcgtctctcaacaaactacgaggattcaaaaagagagtcaatgtttttgaaactagaatcgtaatggataatgacg :  75800
Seq4 : aagttttctttcaaaatagctatctcgtctctcaacaaactacgaggattcaaaaagagagtcaatgtttttgaaactagaatcgtaatggataatgacg :  75800

             *     75820         *     75840         *     75860         *     75880         *     75900
Seq1 : ataacatttaggaatgttgttttcggatagagttcaatcctttaagatcaacatctttatggcgtttttagattaatacttttcaatgagataaatatgg :  75900
Seq2 : ataacatttaggaatgttgttttcggatagagttcaatcctttaagatcaacatctttatggcgtttttagattaatacttttcaatgagataaatatgg :  75900
Seq3 : ataacatttaggaatgttgttttcggatagagttcaatcctttaagatcaacatctttatggcgtttttagattaatacttttcaatgagataaatatgg :  75900
Seq4 : ataacatttaggaatgttgttttcggatagagttcaatcctttaagatcaacatctttatggcgtttttagattaatacttttcaatgagataaatatgg :  75900

             *     75920         *     75940         *     75960         *     75980         *     76000
Seq1 : gtggcggagtaagtgttgagctccctaaacgggatccgcctccgggagtacccactgatgagatgttattaaacgtggataaaatgcatgacgtgatagc :  76000
Seq2 : gtggcggagtaagtgttgagctccctaaacgggatccgcctccgggagtacccactgatgagatgttattaaacgtggataaaatgcatgacgtgatagc :  76000
Seq3 : gtggcggagtaagtgttgagctccctaaacgggatccgcctccgggagtacccactgatgagatgttattaaacgtggataaaatgcatgacgtgatagc :  76000
Seq4 : gtggcggagtaagtgttgagctccctaaacgggatccgcctccgggagtacccactgatgagatgttattaaacgtggataaaatgcatgacgtgatagc :  76000

             *     76020         *     76040         *     76060         *     76080         *     76100
Seq1 : tcccgctaagcttttagaatatgtgcatataggaccactagcaaaagataaagaggataaagtaaagaaaagatatccagagtttagattagtcaacaca :  76100
Seq2 : tcccgctaagcttttagaatatgtgcatataggaccactagcaaaagataaagaggataaagtaaagaaaagatatccagagtttagattagtcaacaca :  76100
Seq3 : tcccgctaagcttttagaatatgtgcatataggaccactagcaaaagataaagaggataaagtaaagaaaagatatccagagtttagattagtcaacaca :  76100
Seq4 : tcccgctaagcttttagaatatgtgcatataggaccactagcaaaagataaagaggataaagtaaagaaaagatatccagagtttagattagtcaacaca :  76100

             *     76120         *     76140         *     76160         *     76180         *     76200
Seq1 : ggacccggtggtctttcggcattgttaagacaatcgtataatggaaccgcacccaattgctgtcgcacttttaatcgtactcattattggaagaaggatg :  76200
Seq2 : ggacccggtggtctttcggcattgttaagacaatcgtataatggaaccgcacccaattgctgtcgcacttttaatcgtactcattattggaagaaggatg :  76200
Seq3 : ggacccggtggtctttcggcattgttaagacaatcgtataatggaaccgcacccaattgctgtcgcacttttaatcgtactcattattggaagaaggatg :  76200
Seq4 : ggacccggtggtctttcggcattgttaagacaatcgtataatggaaccgcacccaattgctgtcgcacttttaatcgtactcattattggaagaaggatg :  76200

             *     76220         *     76240         *     76260         *     76280         *     76300
Seq1 : gaaagatatcagataagtatgaagagggtgcagtattagaatcgtgttggccagacgttcacgacaccggaaaatgcgatgttgatttattcgactggtg :  76300
Seq2 : gaaagatatcagataagtatgaagagggtgcagtattagaatcgtgttggccagacgttcacgacaccggaaaatgcgatgttgatttattcgactggtg :  76300
Seq3 : gaaagatatcagataagtatgaagagggtgcagtattagaatcgtgttggccagacgttcacgacaccggaaaatgcgatgttgatttattcgactggtg :  76300
Seq4 : gaaagatatcagataagtatgaagagggtgcagtattagaatcgtgttggccagacgttcacgacaccggaaaatgcgatgttgatttattcgactggtg :  76300
```

```
               *     76320         *     76340         *     76360         *     76380         *     76400
Seq1 : tcaggggatacgttcgatagaaacatatgccatcagtggatcggttcagcctttaataggagtaatagaactgtagagggtcaacaatcgttaataaat :  76400
Seq2 : tcaggggatacgttcgatagaaacatatgccatcagtggatcggttcagcctttaataggagtaatagaactgtagagggtcaacaatcgttaataaat :  76400
Seq3 : tcaggggatacgttcgatagaaacatatgccatcagtggatcggttcagcctttaataggagtaatagaactgtagagggtcaacaatcgttaataaat :  76400
Seq4 : tcaggggatacgttcgatagaaacatatgccatcagtggatcggttcagcctttaataggagtaatagaactgtagagggtcaacaatcgttaataaat :  76400

               *     76420         *     76440         *     76460         *     76480         *     76500
Seq1 : ctgtataataagatgcaaacattatgtagtaaagatgctagtgtaccaatatgtgaatcatttttgcatcatttacgcgcacacaatacagaagatagca :  76500
Seq2 : ctgtataataagatgcaaacattatgtagtaaagatgctagtgtaccaatatgtgaatcatttttgcatcatttacgcgcacacaatacagaagatagca :  76500
Seq3 : ctgtataataagatgcaaacattatgtagtaaagatgctagtgtaccaatatgtgaatcatttttgcatcatttacgcgcacacaatacagaagatagca :  76500
Seq4 : ctgtataataagatgcaaacattatgtagtaaagatgctagtgtaccaatatgtgaatcatttttgcatcatttacgcgcacacaatacagaagatagca :  76500

               *     76520         *     76540         *     76560         *     76580         *     76600
Seq1 : aagagatgatcgattatattctaagacaacagtctgcggactttaaacagaaatatatgagatgtagttatcccactagagataagttagaagagtcatt :  76600
Seq2 : aagagatgatcgattatattctaagacaacagtctgcggactttaaacagaaatatatgagatgtagttatcccactagagataagttagaagagtcatt :  76600
Seq3 : aagagatgatcgattatattctaagacaacagtctgcggactttaaacagaaatatatgagatgtagttatcccactagagataagttagaagagtcatt :  76600
Seq4 : aagagatgatcgattatattctaagacaacagtctgcggactttaaacagaaatatatgagatgtagttatcccactagagataagttagaagagtcatt :  76600

               *     76620         *     76640         *     76660         *     76680         *     76700
Seq1 : aaaatatgcggaacctcgagaatgttgggatccagagtgttcgaatgccaatgttaatttcttgctaacacgtaattataataatttaggactttgcaat :  76700
Seq2 : aaaatatgcggaacctcgagaatgttgggatccagagtgttcgaatgccaatgttaatttcttgctaacacgtaattataataatttaggactttgcaat :  76700
Seq3 : aaaatatgcggaacctcgagaatgttgggatccagagtgttcgaatgccaatgttaatttcttgctaacacgtaattataataatttaggactttgcaat :  76700
Seq4 : aaaatatgcggaacctcgagaatgttgggatccagagtgttcgaatgccaatgttaatttcttgctaacacgtaattataataatttaggactttgcaat :  76700

               *     76720         *     76740         *     76760         *     76780         *     76800
Seq1 : attgtacgatgtaatactagcgtgaacaacttacagatggataaaacttcctcattaagattgtcatgtggattaagcaatagtgatagattttctactg :  76800
Seq2 : attgtacgatgtaatactagcgtgaacaacttacagatggataaaacttcctcattaagattgtcatgtggattaagcaatagtgatagattttctactg :  76800
Seq3 : attgtacgatgtaatactagcgtgaacaacttacagatggataaaacttcctcattaagattgtcatgtggattaagcaatagtgatagattttctactg :  76800
Seq4 : attgtacgatgtaatactagcgtgaacaacttacagatggataaaacttcctcattaagattgtcatgtggattaagcaatagtgatagattttctactg :  76800

               *     76820         *     76840         *     76860         *     76880         *     76900
Seq1 : ttcccgtcaatagagcaaaagtagttcaacataatattaaacactcgttcgacctaaaattgcatttgatcagtttattatctctcttggtaatatggat :  76900
Seq2 : ttcccgtcaatagagcaaaagtagttcaacataatattaaacactcgttcgacctaaaattgcatttgatcagtttattatctctcttggtaatatggat :  76900
Seq3 : ttcccgtcaatagagcaaaagtagttcaacataatattaaacactcgttcgacctaaaattgcatttgatcagtttattatctctcttggtaatatggat :  76900
Seq4 : ttcccgtcaatagagcaaaagtagttcaacataatattaaacactcgttcgacctaaaattgcatttgatcagtttattatctctcttggtaatatggat :  76900

               *     76920         *     76940         *     76960         *     76980         *     77000
Seq1 : actaattgtagctatttaaatgggtgccgcggcaagcatacagacgacggtgaatacactcagcgaacgtatctcgtctaaattagaacaagaagcgaat :  77000
Seq2 : actaattgtagctatttaaatgggtgccgcggcaagcatacagacgacggtgaatacactcagcgaacgtatctcgtctaaattagaacaagaagcgaat :  77000
Seq3 : actaattgtagctatttaaatgggtgccgcggcaagcatacagacgacggtgaatacactcagcgaacgtatctcgtctaaattagaacaagaagcgaat :  77000
Seq4 : actaattgtagctatttaaatgggtgccgcggcaagcatacagacgacggtgaatacactcagcgaacgtatctcgtctaaattagaacaagaagcgaat :  77000
```

```
                 *     77020         *     77040         *     77060         *     77080         *     77100
Seq1 : gctagtgctcaaacaaaatgtgatatagaaatcggaaatttttatatccgacaaaaccatggatgtaacctcactgttaaaaatatgtgctctgcggacg :  77100
Seq2 : gctagtgctcaaacaaaatgtgatatagaaatcggaaatttttatatccgacaaaaccatggatgtaacctcactgttaaaaatatgtgctctgcggacg :  77100
Seq3 : gctagtgctcaaacaaaatgtgatatagaaatcggaaatttttatatccgacaaaaccatggatgtaacctcactgttaaaaatatgtgctctgcggacg :  77100
Seq4 : gctagtgctcaaacaaaatgtgatatagaaatcggaaatttttatatccgacaaaaccatggatgtaacctcactgttaaaaatatgtgctctgcggacg :  77100

                 *     77120         *     77140         *     77160         *     77180         *     77200
Seq1 : cggatgctcagttggatgctgtgttatcagccgctacagaaacatatagtggattaacaccggaacaaaaagcatacgtgccagctatgtttactgctgc :  77200
Seq2 : cggatgctcagttggatgctgtgttatcagccgctacagaaacatatagtggattaacaccggaacaaaaagcatacgtgccagctatgtttactgctgc :  77200
Seq3 : cggatgctcagttggatgctgtgttatcagccgctacagaaacatatagtggattaacaccggaacaaaaagcatacgtgccagctatgtttactgctgc :  77200
Seq4 : cggatgctcagttggatgctgtgttatcagccgctacagaaacatatagtggattaacaccggaacaaaaagcatacgtgccagctatgtttactgctgc :  77200

                 *     77220         *     77240         *     77260         *     77280         *     77300
Seq1 : gttaaacattcagacgagtgtaaacactgttgttagagattttgaaaattatgtgaaacagacttgtaattctagcgcggtcgtcgataacaaattaaag :  77300
Seq2 : gttaaacattcagacgagtgtaaacactgttgttagagattttgaaaattatgtgaaacagacttgtaattctagcgcggtcgtcgataacaaattaaag :  77300
Seq3 : gttaaacattcagacgagtgtaaacactgttgttagagattttgaaaattatgtgaaacagacttgtaattctagcgcggtcgtcgataacaaattaaag :  77300
Seq4 : gttaaacattcagacgagtgtaaacactgttgttagagattttgaaaattatgtgaaacagacttgtaattctagcgcggtcgtcgataacaaattaaag :  77300

                 *     77320         *     77340         *     77360         *     77380         *     77400
Seq1 : atacaaaacgtaatcatagatgaatgttacggagccccaggatctccaacaaatttggaatttattaatacaggatctagcaaaggaaattgtgccatta :  77400
Seq2 : atacaaaacgtaatcatagatgaatgttacggagccccaggatctccaacaaatttggaatttattaatacaggatctagcaaaggaaattgtgccatta :  77400
Seq3 : atacaaaacgtaatcatagatgaatgttacggagccccaggatctccaacaaatttggaatttattaatacaggatctagcaaaggaaattgtgccatta :  77400
Seq4 : atacaaaacgtaatcatagatgaatgttacggagccccaggatctccaacaaatttggaatttattaatacaggatctagcaaaggaaattgtgccatta :  77400

                 *     77420         *     77440         *     77460         *     77480         *     77500
Seq1 : aggcgttgatgcaattgacgactaaggccactactcaaatagcacctaaacaagttgctggtacaggagttcagttttatatgattgttatcggtgttat :  77500
Seq2 : aggcgttgatgcaattgacgactaaggccactactcaaatagcacctaaacaagttgctggtacaggagttcagttttatatgattgttatcggtgttat :  77500
Seq3 : aggcgttgatgcaattgacgactaaggccactactcaaatagcacctaaacaagttgctggtacaggagttcagttttatatgattgttatcggtgttat :  77500
Seq4 : aggcgttgatgcaattgacgactaaggccactactcaaatagcacctaaacaagttgctggtacaggagttcagttttatatgattgttatcggtgttat :  77500

                 *     77520         *     77540         *     77560         *     77580         *     77600
Seq1 : aatattggcagcgttgtttatgtactatgccaagcgtatgttgttcacatccaccaatgataaaatcaaacttattttagccaataaggaaaacgtccat :  77600
Seq2 : aatattggcagcgttgtttatgtactatgccaagcgtatgttgttcacatccaccaatgataaaatcaaacttattttagccaataaggaaaacgtccat :  77600
Seq3 : aatattggcagcgttgtttatgtactatgccaagcgtatgttgttcacatccaccaatgataaaatcaaacttattttagccaataaggaaaacgtccat :  77600
Seq4 : aatattggcagcgttgtttatgtactatgccaagcgtatgttgttcacatccaccaatgataaaatcaaacttattttagccaataaggaaaacgtccat :  77600

                 *     77620         *     77640         *     77660         *     77680         *     77700
Seq1 : tggactacttacatggacacattctttagaacttctccgatggttattgctaccacggatatgcaaaactgaaaatatattgataatattttaatagatt :  77700
Seq2 : tggactacttacatggacacattctttagaacttctccgatggttattgctaccacggatatgcaaaactgaaaatatattgataatattttaatagatt :  77700
Seq3 : tggactacttacatggacacattctttagaacttctccgatggttattgctaccacggatatgcaaaactgaaaatatattgataatattttaatagatt :  77700
Seq4 : tggactacttacatggacacattctttagaacttctccgatggttattgctaccacggatatgcaaaactgaaaatatattgataatattttaatagatt :  77700
```

```
               *     77720         *     77740         *     77760         *     77780         *     77800
Seq1 : aacatggaagttatcactgatcgtctagacgatatagtgaaacaaaatatagcggatgaaaaatttgtagattttgttatacacggtctagagcatcaat :  77800
Seq2 : aacatggaagttatcactgatcgtctagacgatatagtgaaacaaaatatagcggatgaaaaatttgtagattttgttatacacggtctagagcatcaat :  77800
Seq3 : aacatggaagttatcactgatcgtctagacgatatagtgaaacaaaatatagcggatgaaaaatttgtagattttgttatacacggtctagagcatcaat :  77800
Seq4 : aacatggaagttatcactgatcgtctagacgatatagtgaaacaaaatatagcggatgaaaaatttgtagattttgttatacacggtctagagcatcaat :  77800

               *     77820         *     77840         *     77860         *     77880         *     77900
Seq1 : gtcctgctatacttcgaccattaattaggttgtttattgatatactattatttgttatagtaatttatattttacggtacgtctagtaagtagaaatta :  77900
Seq2 : gtcctgctatacttcgaccattaattaggttgtttattgatatactattatttgttatagtaatttatattttacggtacgtctagtaagtagaaatta :  77900
Seq3 : gtcctgctatacttcgaccattaattaggttgtttattgatatactattatttgttatagtaatttatattttacggtacgtctagtaagtagaaatta :  77900
Seq4 : gtcctgctatacttcgaccattaattaggttgtttattgatatactattatttgttatagtaatttatattttacggtacgtctagtaagtagaaatta :  77900

               *     77920         *     77940         *     77960         *     77980         *     78000
Seq1 : tcaaatgttgttggcgttggtggcgctagtcatcacattaactatttttattactttatactataatagtactagactgacttctaacaaacatctcac :  78000
Seq2 : tcaaatgttgttggcgttggtggcgctagtcatcacattaactatttttattactttatactataatagtactagactgacttctaacaaacatctcac :  78000
Seq3 : tcaaatgttgttggcgttggtggcgctagtcatcacattaactatttttattactttatactataatagtactagactgacttctaacaaacatctcac :  78000
Seq4 : tcaaatgttgttggcgttggtggcgctagtcatcacattaactatttttattactttatactataatagtactagactgacttctaacaaacatctcac :  78000

               *     78020         *     78040         *     78060         *     78080         *     78100
Seq1 : ctgccataaataaatgcttgatattaaagtcttctatttctaacactattccatctgtggaaaataatactctgacattatcgctaattgacacatcggt :  78100
Seq2 : ctgccataaataaatgcttgatattaaagtcttctatttctaacactattccatctgtggaaaataatactctgacattatcgctaattgacacatcggt :  78100
Seq3 : ctgccataaataaatgcttgatattaaagtcttctatttctaacactattccatctgtggaaaataatactctgacattatcgctaattgacacatcggt :  78100
Seq4 : ctgccataaataaatgcttgatattaaagtcttctatttctaacactattccatctgtggaaaataatactctgacattatcgctaattgacacatcggt :  78100

               *     78120         *     78140         *     78160         *     78180         *     78200
Seq1 : gagtgatatgcctataaagtaataatcttctttgggcacatataccagtgtaccaggttctaacaacctatttactggtgctcctatagcatactttttc :  78200
Seq2 : gagtgatatgcctataaagtaataatcttctttgggcacatataccagtgtaccaggttctaacaacctatttactggtgctcctatagcatactttttc :  78200
Seq3 : gagtgatatgcctataaagtaataatcttctttgggcacatataccagtgtaccaggttctaacaacctatttactggtgctcctatagcatactttttc :  78200
Seq4 : gagtgatatgcctataaagtaataatcttctttgggcacatataccagtgtaccaggttctaacaacctatttactggtgctcctatagcatactttttc :  78200

               *     78220         *     78240         *     78260         *     78280         *     78300
Seq1 : tttaccttgagaatatccatcgtttgcttggtcaatagcgatatgtgatttttatcaaccactcgaaaaagtaattggagtgttcatatcctctacggg :  78300
Seq2 : tttaccttgagaatatccatcgtttgcttggtcaatagcgatatgtgatttttatcaaccactcgaaaaagtaattggagtgttcatatcctctacggg :  78300
Seq3 : tttaccttgagaatatccatcgtttgcttggtcaatagcgatatgtgatttttatcaaccactcgaaaaagtaattggagtgttcatatcctctacggg :  78300
Seq4 : tttaccttgagaatatccatcgtttgcttggtcaatagcgatatgtgatttttatcaaccactcgaaaaagtaattggagtgttcatatcctctacggg :  78300

               *     78320         *     78340         *     78360         *     78380         *     78400
Seq1 : ctattgtctcatggccgtgtatgaaatttaagtaacacgactgtggtagatttgttctatagagccggttgccgcaaatagatagaactaccaatatgtc :  78400
Seq2 : ctattgtctcatggccgtgtatgaaatttaagtaacacgactgtggtagatttgttctatagagccggttgccgcaaatagatagaactaccaatatgtc :  78400
Seq3 : ctattgtctcatggccgtgtatgaaatttaagtaacacgactgtggtagatttgttctatagagccggttgccgcaaatagatagaactaccaatatgtc :  78400
Seq4 : ctattgtctcatggccgtgtatgaaatttaagtaacacgactgtggtagatttgttctatagagccggttgccgcaaatagatagaactaccaatatgtc :  78400
```

```
             *     78420           *     78440           *     78460           *     78480           *     78500
Seq1 : tgtacaaatgttaaacattaattgattaacagaaaaaacaatgttcgttctgggaatagaaaccagatcaaaacaaaattcgttagaatatatgccacgt :  78500
Seq2 : tgtacaaatgttaaacattaattgattaacagaaaaaacaatgttcgttctgggaatagaaaccagatcaaaacaaaattcgttagaatatatgccacgt :  78500
Seq3 : tgtacaaatgttaaacattaattgattaacagaaaaaacaatgttcgttctgggaatagaaaccagatcaaaacaaaattcgttagaatatatgccacgt :  78500
Seq4 : tgtacaaatgttaaacattaattgattaacagaaaaaacaatgttcgttctgggaatagaaaccagatcaaaacaaaattcgttagaatatatgccacgt :  78500

             *     78520           *     78540           *     78560           *     78580           *     78600
Seq1 : ttatacattgaatataaaataactacagtttgaaaaataacagtatcatttaaacatttaacttgcggggttaatctcacaactttactgtttttgaact :  78600
Seq2 : ttatacattgaatataaaataactacagtttgaaaaataacagtatcatttaaacatttaacttgcggggttaatctcacaactttactgtttttgaact :  78600
Seq3 : ttatacattgaatataaaataactacagtttgaaaaataacagtatcatttaaacatttaacttgcggggttaatctcacaactttactgtttttgaact :  78600
Seq4 : ttatacattgaatataaaataactacagtttgaaaaataacagtatcatttaaacatttaacttgcggggttaatctcacaactttactgtttttgaact :  78600

             *     78620           *     78640           *     78660           *     78680           *     78700
Seq1 : gttcaaaatatagcatagatccgtgagaaatacgtttagccgcctttaatagaggaaatcccaccgcctttctggatctcaccaacgacgatagttctga :  78700
Seq2 : gttcaaaatatagcatagatccgtgagaaatacgtttagccgcctttaatagaggaaatcccaccgcctttctggatctcaccaacgacgatagttctga :  78700
Seq3 : gttcaaaatatagcatagatccgtgagaaatacgtttagccgcctttaatagaggaaatcccaccgcctttctggatctcaccaacgacgatagttctga :  78700
Seq4 : gttcaaaatatagcatagatccgtgagaaatacgtttagccgcctttaatagaggaaatcccaccgcctttctggatctcaccaacgacgatagttctga :  78700

             *     78720           *     78740           *     78760           *     78780           *     78800
Seq1 : ccagcaactcatttcttcatcatccacctgttttaacatataataggcaggagatagatatccgtcattgcaatattccttctcgtaggcacacaatcta :  78800
Seq2 : ccagcaactcatttcttcatcatccacctgttttaacatataataggcaggagatagatatccgtcattgcaatattccttctcgtaggcacacaatcta :  78800
Seq3 : ccagcaactcatttcttcatcatccacctgttttaacatataataggcaggagatagatatccgtcattgcaatattccttctcgtaggcacacaatcta :  78800
Seq4 : ccagcaactcatttcttcatcatccacctgttttaacatataataggcaggagatagatatccgtcattgcaatattccttctcgtaggcacacaatcta :  78800

             *     78820           *     78840           *     78860           *     78880           *     78900
Seq1 : atattgataaaatctccattctcttctctgcatttattatcttgtttcggtggctgattaggctgtagtcttggtttaggctttggtatatcgttgttga :  78900
Seq2 : atattgataaaatctccattctcttctctgcatttattatcttgtttcggtggctgattaggctgtagtcttggtttaggctttggtatatcgttgttga :  78900
Seq3 : atattgataaaatctccattctcttctctgcatttattatcttgtttcggtggctgattaggctgtagtcttggtttaggctttggtatatcgttgttga :  78900
Seq4 : atattgataaaatctccattctcttctctgcatttattatcttgtttcggtggctgattaggctgtagtcttggtttaggctttggtatatcgttgttga :  78900

             *     78920           *     78940           *     78960           *     78980           *     79000
Seq1 : atctatttggtcattaaatctttcatttcttcctggtatatttttatcacctcgtttggttggattttgtctatattatcgtttgtaacatcggtacg :  79000
Seq2 : atctatttggtcattaaatctttcatttcttcctggtatatttttatcacctcgtttggttggattttgtctatattatcgtttgtaacatcggtacg :  79000
Seq3 : atctatttggtcattaaatctttcatttcttcctggtatatttttatcacctcgtttggttggattttgtctatattatcgtttgtaacatcggtacg :  79000
Seq4 : atctatttggtcattaaatctttcatttcttcctggtatatttttatcacctcgtttggttggattttgtctatattatcgtttgtaacatcggtacg :  79000

             *     79020           *     79040           *     79060           *     79080           *     79100
Seq1 : ggtattcatttatcacaaaaaaaaacttctctaaatgagtctactgctagaaaacctcatcgaagaagataccatattttttgcaggaagtatatctgagt :  79100
Seq2 : ggtattcatttatcacaaaaaaaaacttctctaaatgagtctactgctagaaaacctcatcgaagaagataccatattttttgcaggaagtatatctgagt :  79100
Seq3 : ggtattcatttatcacaaaaaaaaacttctctaaatgagtctactgctagaaaacctcatcgaagaagataccatattttttgcaggaagtatatctgagt :  79100
Seq4 : ggtattcatttatcacaaaaaaaaacttctctaaatgagtctactgctagaaaacctcatcgaagaagataccatattttttgcaggaagtatatctgagt :  79100
```

```
                *     79120         *     79140         *     79160         *     79180         *     79200
Seq1 : atgatgatttacaaatggttattgccggcgcaaaatccaaatttccaagatctatgctttctatttttaatatagtacctagaacgatgtcaaaatatga :  79200
Seq2 : atgatgatttacaaatggttattgccggcgcaaaatccaaatttccaagatctatgctttctatttttaatatagtacctagaacgatgtcaaaatatga :  79200
Seq3 : atgatgatttacaaatggttattgccggcgcaaaatccaaatttccaagatctatgctttctatttttaatatagtacctagaacgatgtcaaaatatga :  79200
Seq4 : atgatgatttacaaatggttattgccggcgcaaaatccaaatttccaagatctatgctttctatttttaatatagtacctagaacgatgtcaaaatatga :  79200

                *     79220         *     79240         *     79260         *     79280         *     79300
Seq1 : gttggagttgattcataacgaaaatatcacaggagcaatgtttaccacaatgtataatataagaaacaatttgggtctaggagatgataaactaactatt :  79300
Seq2 : gttggagttgattcataacgaaaatatcacaggagcaatgtttaccacaatgtataatataagaaacaatttgggtctaggagatgataaactaactatt :  79300
Seq3 : gttggagttgattcataacgaaaatatcacaggagcaatgtttaccacaatgtataatataagaaacaatttgggtctaggagatgataaactaactatt :  79300
Seq4 : gttggagttgattcataacgaaaatatcacaggagcaatgtttaccacaatgtataatataagaaacaatttgggtctaggagatgataaactaactatt :  79300

                *     79320         *     79340         *     79360         *     79380         *     79400
Seq1 : gaagccattgaaaactatttcttggatcctaacaatgaagttatgcctcttattattaataatacggatatgactgccgtcattcctaaaaaaagtggta :  79400
Seq2 : gaagccattgaaaactatttcttggatcctaacaatgaagttatgcctcttattattaataatacggatatgactgccgtcattcctaaaaaaagtggta :  79400
Seq3 : gaagccattgaaaactatttcttggatcctaacaatgaagttatgcctcttattattaataatacggatatgactgccgtcattcctaaaaaaagtggta :  79400
Seq4 : gaagccattgaaaactatttcttggatcctaacaatgaagttatgcctcttattattaataatacggatatgactgccgtcattcctaaaaaaagtggta :  79400

                *     79420         *     79440         *     79460         *     79480         *     79500
Seq1 : ggagaaagaataagaacatggttatcttccgtcaaggatcatcacctatcttgtgtattttcgaaactcgtaaaaagattaatatttataaagaaaatat :  79500
Seq2 : ggagaaagaataagaacatggttatcttccgtcaaggatcatcacctatcttgtgtattttcgaaactcgtaaaaagattaatatttataaagaaaatat :  79500
Seq3 : ggagaaagaataagaacatggttatcttccgtcaaggatcatcacctatcttgtgtattttcgaaactcgtaaaaagattaatatttataaagaaaatat :  79500
Seq4 : ggagaaagaataagaacatggttatcttccgtcaaggatcatcacctatcttgtgtattttcgaaactcgtaaaaagattaatatttataaagaaaatat :  79500

                *     79520         *     79540         *     79560         *     79580         *     79600
Seq1 : ggaatccgcgtcgactgagtatacacctatcggagacaacaaggctttgatatctaaatatgcgggaattaatatcctaaatgtgtattctccttccaca :  79600
Seq2 : ggaatccgcgtcgactgagtatacacctatcggagacaacaaggctttgatatctaaatatgcgggaattaatatcctaaatgtgtattctccttccaca :  79600
Seq3 : ggaatccgcgtcgactgagtatacacctatcggagacaacaaggctttgatatctaaatatgcgggaattaatatcctaaatgtgtattctccttccaca :  79600
Seq4 : ggaatccgcgtcgactgagtatacacctatcggagacaacaaggctttgatatctaaatatgcgggaattaatatcctaaatgtgtattctccttccaca :  79600

                *     79620         *     79640         *     79660         *     79680         *     79700
Seq1 : tccataagattgaatgccatttacggattcaccaataaaaataaactagagaaacttagtactaataaggaactagaatcgtatagttctagccctcttc :  79700
Seq2 : tccataagattgaatgccatttacggattcaccaataaaaataaactagagaaacttagtactaataaggaactagaatcgtatagttctagccctcttc :  79700
Seq3 : tccataagattgaatgccatttacggattcaccaataaaaataaactagagaaacttagtactaataaggaactagaatcgtatagttctagccctcttc :  79700
Seq4 : tccataagattgaatgccatttacggattcaccaataaaaataaactagagaaacttagtactaataaggaactagaatcgtatagttctagccctcttc :  79700

                *     79720         *     79740         *     79760         *     79780         *     79800
Seq1 : aagaacccattaggttaaatgatttctgggactattggaatgtgttaaaaagaatattcctctaacagatattccgacaaaggattgattactataaat :  79800
Seq2 : aagaacccattaggttaaatgatttctgggactattggaatgtgttaaaaagaatattcctctaacagatattccgacaaaggattgattactataaat :  79800
Seq3 : aagaacccattaggttaaatgatttctgggactattggaatgtgttaaaaagaatattcctctaacagatattccgacaaaggattgattactataaat :  79800
Seq4 : aagaacccattaggttaaatgatttctgggactattggaatgtgttaaaaagaatattcctctaacagatattccgacaaaggattgattactataaat :  79800
```

```
                *     79820         *     79840         *     79860         *     79880         *     79900
Seq1 : ggagaatgttcctaatgtatactttaatcctgtgtttatagagcccacgtttaaacattctttattaagtgtttataaacacagattaatagttttattt :  79900
Seq2 : ggagaatgttcctaatgtatactttaatcctgtgtttatagagcccacgtttaaacattctttattaagtgtttataaacacagattaatagttttattt :  79900
Seq3 : ggagaatgttcctaatgtatactttaatcctgtgtttatagagcccacgtttaaacattctttattaagtgtttataaacacagattaatagttttattt :  79900
Seq4 : ggagaatgttcctaatgtatactttaatcctgtgtttatagagcccacgtttaaacattctttattaagtgtttataaacacagattaatagttttattt :  79900

                *     79920         *     79940         *     79960         *     79980         *     80000
Seq1 : gaagtattcgttgtattcattctaatatatgtattttttagatctgaattaaatatgttcttcatgcctaaacgaaaaatacccgatcctattgatagat :  80000
Seq2 : gaagtattcgttgtattcattctaatatatgtattttttagatctgaattaaatatgttcttcatgcctaaacgaaaaatacccgatcctattgatagat :  80000
Seq3 : gaagtattcgttgtattcattctaatatatgtattttttagatctgaattaaatatgttcttcatgcctaaacgaaaaatacccgatcctattgatagat :  80000
Seq4 : gaagtattcgttgtattcattctaatatatgtattttttagatctgaattaaatatgttcttcatgcctaaacgaaaaatacccgatcctattgatagat :  80000

                *     80020         *     80040         *     80060         *     80080         *     80100
Seq1 : tacgacgtgctaatctagcgtgtgaagacgataaattaatgatctatggattaccatggatgacaactcaaacatctgcgttatcaataaatagtaaacc :  80100
Seq2 : tacgacgtgctaatctagcgtgtgaagacgataaattaatgatctatggattaccatggatgacaactcaaacatctgcgttatcaataaatagtaaacc :  80100
Seq3 : tacgacgtgctaatctagcgtgtgaagacgataaattaatgatctatggattaccatggatgacaactcaaacatctgcgttatcaataaatagtaaacc :  80100
Seq4 : tacgacgtgctaatctagcgtgtgaagacgataaattaatgatctatggattaccatggatgacaactcaaacatctgcgttatcaataaatagtaaacc :  80100

                *     80120         *     80140         *     80160         *     80180         *     80200
Seq1 : gatagtgtataaagattgtgcaaagcttttgcgatcaataaatggatcacaaccagtatctcttaacgatgttcttcgcagatgatgattcattttttaa :  80200
Seq2 : gatagtgtataaagattgtgcaaagcttttgcgatcaataaatggatcacaaccagtatctcttaacgatgttcttcgcagatgatgattcattttttaa :  80200
Seq3 : gatagtgtataaagattgtgcaaagcttttgcgatcaataaatggatcacaaccagtatctcttaacgatgttcttcgcagatgatgattcattttttaa :  80200
Seq4 : gatagtgtataaagattgtgcaaagcttttgcgatcaataaatggatcacaaccagtatctcttaacgatgttcttcgcagatgatgattcattttttaa :  80200

                *     80220         *     80240         *     80260         *     80280         *     80300
Seq1 : gtatttggctagtcaagatgatgaatcttcattatctgatatattgcaaatcactcaatatctagactttctgttattattattgatccaatcaaaaaat :  80300
Seq2 : gtatttggctagtcaagatgatgaatcttcattatctgatatattgcaaatcactcaatatctagactttctgttattattattgatccaatcaaaaaat :  80300
Seq3 : gtatttggctagtcaagatgatgaatcttcattatctgatatattgcaaatcactcaatatctagactttctgttattattattgatccaatcaaaaaat :  80300
Seq4 : gtatttggctagtcaagatgatgaatcttcattatctgatatattgcaaatcactcaatatctagactttctgttattattattgatccaatcaaaaaat :  80300

                *     80320         *     80340         *     80360         *     80380         *     80400
Seq1 : aaattagaagccgtgggtcattgttatgaatctctttcagaggaatacagacaattgacaaaattcacagactttcaagattttaaaaaactgtttaaca :  80400
Seq2 : aaattagaagccgtgggtcattgttatgaatctctttcagaggaatacagacaattgacaaaattcacagactttcaagattttaaaaaactgtttaaca :  80400
Seq3 : aaattagaagccgtgggtcattgttatgaatctctttcagaggaatacagacaattgacaaaattcacagactttcaagattttaaaaaactgtttaaca :  80400
Seq4 : aaattagaagccgtgggtcattgttatgaatctctttcagaggaatacagacaattgacaaaattcacagactttcaagattttaaaaaactgtttaaca :  80400

                *     80420         *     80440         *     80460         *     80480         *     80500
Seq1 : aggtccctattgttacagatggaagggtcaaacttaataaaggatatttgttcgactttgtgattagtttgatgcgattcaaaaaagaatcctctctagc :  80500
Seq2 : aggtccctattgttacagatggaagggtcaaacttaataaaggatatttgttcgactttgtgattagtttgatgcgattcaaaaaagaatcctctctagc :  80500
Seq3 : aggtccctattgttacagatggaagggtcaaacttaataaaggatatttgttcgactttgtgattagtttgatgcgattcaaaaaagaatcctctctagc :  80500
Seq4 : aggtccctattgttacagatggaagggtcaaacttaataaaggatatttgttcgactttgtgattagtttgatgcgattcaaaaaagaatcctctctagc :  80500
```

```
                 *     80520          *     80540          *     80560          *     80580          *     80600
Seq1 : taccaccgcaatagatcctgttagatacatagatcctcgtcgcaatatcgcattttctaacgtgatggatatattaaagtcgaataaagtgaacaataat :  80600
Seq2 : taccaccgcaatagatcctgttagatacatagatcctcgtcgcaatatcgcattttctaacgtgatggatatattaaagtcgaataaagtgaacaataat :  80600
Seq3 : taccaccgcaatagatcctgttagatacatagatcctcgtcgcaatatcgcattttctaacgtgatggatatattaaagtcgaataaagtgaacaataat :  80600
Seq4 : taccaccgcaatagatcctgttagatacatagatcctcgtcgcaatatcgcattttctaacgtgatggatatattaaagtcgaataaagtgaacaataat :  80600

                 *     80620          *     80640          *     80660          *     80680          *     80700
Seq1 : taattctttattgtcatcatgaacggcggacatattcagttgataatcggccccatgttttcaggtaaaagtacagaattaattagacgagttagacgtt :  80700
Seq2 : taattctttattgtcatcatgaacggcggacatattcagttgataatcggccccatgttttcaggtaaaagtacagaattaattagacgagttagacgtt :  80700
Seq3 : taattctttattgtcatcatgaacggcggacatattcagttgataatcggccccatgttttcaggtaaaagtacagaattaattagacgagttagacgtt :  80700
Seq4 : taattctttattgtcatcatgaacggcggacatattcagttgataatcggccccatgttttcaggtaaaagtacagaattaattagacgagttagacgtt :  80700

                 *     80720          *     80740          *     80760          *     80780          *     80800
Seq1 : atcaaatagctcaatataaatgcgtgactataaaatattctaacgataatagatacggaacgggactatggacgcatgataagaataattttgaagcatt :  80800
Seq2 : atcaaatagctcaatataaatgcgtgactataaaatattctaacgataatagatacggaacgggactatggacgcatgataagaataattttgaagcatt :  80800
Seq3 : atcaaatagctcaatataaatgcgtgactataaaatattctaacgataatagatacggaacgggactatggacgcatgataagaataattttgaagcatt :  80800
Seq4 : atcaaatagctcaatataaatgcgtgactataaaatattctaacgataatagatacggaacgggactatggacgcatgataagaataattttgaagcatt :  80800

                 *     80820          *     80840          *     80860          *     80880          *     80900
Seq1 : ggaagcaactaaactatgtgatgtcttggaatcaattacagatttctccgtgataggtatcgatgaaggacagttctttccagacattgttgaattctgt :  80900
Seq2 : ggaagcaactaaactatgtgatgtcttggaatcaattacagatttctccgtgataggtatcgatgaaggacagttctttccagacattgttgaattctgt :  80900
Seq3 : ggaagcaactaaactatgtgatgtcttggaatcaattacagatttctccgtgataggtatcgatgaaggacagttctttccagacattgttgaattctgt :  80900
Seq4 : ggaagcaactaaactatgtgatgtcttggaatcaattacagatttctccgtgataggtatcgatgaaggacagttctttccagacattgttgaattctgt :  80900

                 *     80920          *     80940          *     80960          *     80980          *     81000
Seq1 : gagcgtatggcaaacgaaggaaaaatagttatagtagccgcactcgatgggacatttcaacgtaaaccgtttaataatattttgaatcttattccattat :  81000
Seq2 : gagcgtatggcaaacgaaggaaaaatagttatagtagccgcactcgatgggacatttcaacgtaaaccgtttaataatattttgaatcttattccattat :  81000
Seq3 : gagcgtatggcaaacgaaggaaaaatagttatagtagccgcactcgatgggacatttcaacgtaaaccgtttaataatattttgaatcttattccattat :  81000
Seq4 : gagcgtatggcaaacgaaggaaaaatagttatagtagccgcactcgatgggacatttcaacgtaaaccgtttaataatattttgaatcttattccattat :  81000

                 *     81020          *     81040          *     81060          *     81080          *     81100
Seq1 : ctgaaatggtggtaaaactaactgctgtgtgtatgaaatgctttaaggaggcttccttttctaaacgattgggtgaggaaaccgagatagaaataatagg :  81100
Seq2 : ctgaaatggtggtaaaactaactgctgtgtgtatgaaatgctttaaggaggcttccttttctaaacgattgggtgaggaaaccgagatagaaataatagg :  81100
Seq3 : ctgaaatggtggtaaaactaactgctgtgtgtatgaaatgctttaaggaggcttccttttctaaacgattgggtgaggaaaccgagatagaaataatagg :  81100
Seq4 : ctgaaatggtggtaaaactaactgctgtgtgtatgaaatgctttaaggaggcttccttttctaaacgattgggtgaggaaaccgagatagaaataatagg :  81100

                 *     81120          *     81140          *     81160          *     81180          *     81200
Seq1 : aggtaatgatatgtatcaatcggtgtgtagaaagtgttacatcgactcataatattatatttttatctaaaaaactaaaaataaacattgattaaattt :  81200
Seq2 : aggtaatgatatgtatcaatcggtgtgtagaaagtgttacatcgactcataatattatatttttatctaaaaaactaaaaataaacattgattaaattt :  81200
Seq3 : aggtaatgatatgtatcaatcggtgtgtagaaagtgttacatcgactcataatattatatttttatctaaaaaactaaaaataaacattgattaaattt :  81200
Seq4 : aggtaatgatatgtatcaatcggtgtgtagaaagtgttacatcgactcataatattatatttttatctaaaaaactaaaaataaacattgattaaattt :  81200
```

```
                 *     81220          *     81240          *     81260          *     81280          *     81300
Seq1 : taatataatacttaaaaatggatgttgtgtcgttagataaaccgtttatgtattttgaggaaattgataatgagttagattacgaaccagaaagtgcaaa :  81300
Seq2 : taatataatacttaaaaatggatgttgtgtcgttagataaaccgtttatgtattttgaggaaattgataatgagttagattacgaaccagaaagtgcaaa :  81300
Seq3 : taatataatacttaaaaatggatgttgtgtcgttagataaaccgtttatgtattttgaggaaattgataatgagttagattacgaaccagaaagtgcaaa :  81300
Seq4 : taatataatacttaaaaatggatgttgtgtcgttagataaaccgtttatgtattttgaggaaattgataatgagttagattacgaaccagaaagtgcaaa :  81300

                 *     81320          *     81340          *     81360          *     81380          *     81400
Seq1 : tgaggtcgcaaaaaaactgccgtatcaaggacagttaaaactattactaggagaattattttttcttagtaagttacagcgacacggtatattagatggt :  81400
Seq2 : tgaggtcgcaaaaaaactgccgtatcaaggacagttaaaactattactaggagaattattttttcttagtaagttacagcgacacggtatattagatggt :  81400
Seq3 : tgaggtcgcaaaaaaactgccgtatcaaggacagttaaaactattactaggagaattattttttcttagtaagttacagcgacacggtatattagatggt :  81400
Seq4 : tgaggtcgcaaaaaaactgccgtatcaaggacagttaaaactattactaggagaattattttttcttagtaagttacagcgacacggtatattagatggt :  81400

                 *     81420          *     81440          *     81460          *     81480          *     81500
Seq1 : gccaccgtagtgtatataggatctgctcccggtacacatatacgttatttgagagatcatttctataatttaggagtgatcatcaaatggatgctaattg :  81500
Seq2 : gccaccgtagtgtatataggatctgctcccggtacacatatacgttatttgagagatcatttctataatttaggagtgatcatcaaatggatgctaattg :  81500
Seq3 : gccaccgtagtgtatataggatctgctcccggtacacatatacgttatttgagagatcatttctataatttaggagtgatcatcaaatggatgctaattg :  81500
Seq4 : gccaccgtagtgtatataggatctgctcccggtacacatatacgttatttgagagatcatttctataatttaggagtgatcatcaaatggatgctaattg :  81500

                 *     81520          *     81540          *     81560          *     81580          *     81600
Seq1 : acggccgccatcatgatcctattttaaatggattgcgtgatgtgactctagtgactcggttcgttgatgaggaatatctacgatccatcaaaaaacaact :  81600
Seq2 : acggccgccatcatgatcctattttaaatggattgcgtgatgtgactctagtgactcggttcgttgatgaggaatatctacgatccatcaaaaaacaact :  81600
Seq3 : acggccgccatcatgatcctattttaaatggattgcgtgatgtgactctagtgactcggttcgttgatgaggaatatctacgatccatcaaaaaacaact :  81600
Seq4 : acggccgccatcatgatcctattttaaatggattgcgtgatgtgactctagtgactcggttcgttgatgaggaatatctacgatccatcaaaaaacaact :  81600

                 *     81620          *     81640          *     81660          *     81680          *     81700
Seq1 : gcatccttctaagattattttaatttctgatgtgagatccaaacgaggaggaaatgaacctagtacggcggatttactaagtaattacgctctacaaaat :  81700
Seq2 : gcatccttctaagattattttaatttctgatgtgagatccaaacgaggaggaaatgaacctagtacggcggatttactaagtaattacgctctacaaaat :  81700
Seq3 : gcatccttctaagattattttaatttctgatgtgagatccaaacgaggaggaaatgaacctagtacggcggatttactaagtaattacgctctacaaaat :  81700
Seq4 : gcatccttctaagattattttaatttctgatgtgagatccaaacgaggaggaaatgaacctagtacggcggatttactaagtaattacgctctacaaaat :  81700

                 *     81720          *     81740          *     81760          *     81780          *     81800
Seq1 : gtcatgattagtattttaaaccccgtggcgtctagtcttaaatggagatgcccgtttccagatcaatggatcaaggacttttatatcccacacggtaata :  81800
Seq2 : gtcatgattagtattttaaaccccgtggcgtctagtcttaaatggagatgcccgtttccagatcaatggatcaaggacttttatatcccacacggtaata :  81800
Seq3 : gtcatgattagtattttaaaccccgtggcgtctagtcttaaatggagatgcccgtttccagatcaatggatcaaggacttttatatcccacacggtaata :  81800
Seq4 : gtcatgattagtattttaaaccccgtggcgtctagtcttaaatggagatgcccgtttccagatcaatggatcaaggacttttatatcccacacggtaata :  81800

                 *     81820          *     81840          *     81860          *     81880          *     81900
Seq1 : aaatgttacaaccttttgctccttcatattcagctgaaatgagattattaagtatttataccggtgagaacatgagactgactcgagttaccaaatcaga :  81900
Seq2 : aaatgttacaaccttttgctccttcatattcagctgaaatgagattattaagtatttataccggtgagaacatgagactgactcgagttaccaaatcaga :  81900
Seq3 : aaatgttacaaccttttgctccttcatattcagctgaaatgagattattaagtatttataccggtgagaacatgagactgactcgagttaccaaatcaga :  81900
Seq4 : aaatgttacaaccttttgctccttcatattcagctgaaatgagattattaagtatttataccggtgagaacatgagactgactcgagttaccaaatcaga :  81900
```

```
             *     81920          *     81940          *     81960          *     81980          *     82000
Seq1 : cgctgtaaattatgaaaaaaagatgtactaccttaataagatcgtccgtaacaaagtagttgttaactttgattatcctaatcaggaatatgactatttt :  82000
Seq2 : cgctgtaaattatgaaaaaaagatgtactaccttaataagatcgtccgtaacaaagtagttgttaactttgattatcctaatcaggaatatgactatttt :  82000
Seq3 : cgctgtaaattatgaaaaaaagatgtactaccttaataagatcgtccgtaacaaagtagttgttaactttgattatcctaatcaggaatatgactatttt :  82000
Seq4 : cgctgtaaattatgaaaaaaagatgtactaccttaataagatcgtccgtaacaaagtagttgttaactttgattatcctaatcaggaatatgactatttt :  82000

             *     82020          *     82040          *     82060          *     82080          *     82100
Seq1 : cacatgtactttatgctgaggaccgtgtactgcaataaaacatttcctactactaaagcaaaggtactatttctacaacaatctatatttcgtttcttaa :  82100
Seq2 : cacatgtactttatgctgaggaccgtgtactgcaataaaacatttcctactactaaagcaaaggtactatttctacaacaatctatatttcgtttcttaa :  82100
Seq3 : cacatgtactttatgctgaggaccgtgtactgcaataaaacatttcctactactaaagcaaaggtactatttctacaacaatctatatttcgtttcttaa :  82100
Seq4 : cacatgtactttatgctgaggaccgtgtactgcaataaaacatttcctactactaaagcaaaggtactatttctacaacaatctatatttcgtttcttaa :  82100

             *     82120          *     82140          *     82160          *     82180          *     82200
Seq1 : atattccaacaacatcaactgaaaaagttagtcatgaaccaatacaacgtaaaatatctagcaaaaattctatgtctaaaaacagaaatagcaagagatc :  82200
Seq2 : atattccaacaacatcaactgaaaaagttagtcatgaaccaatacaacgtaaaatatctagcaaaaattctatgtctaaaaacagaaatagcaagagatc :  82200
Seq3 : atattccaacaacatcaactgaaaaagttagtcatgaaccaatacaacgtaaaatatctagcaaaaattctatgtctaaaaacagaaatagcaagagatc :  82200
Seq4 : atattccaacaacatcaactgaaaaagttagtcatgaaccaatacaacgtaaaatatctagcaaaaattctatgtctaaaaacagaaatagcaagagatc :  82200

             *     82220          *     82240          *     82260          *     82280          *     82300
Seq1 : cgtacgcagtaataaatagaaacgtactactgagatatactaccgatatagagtataatgatttagttactttaataaccgttagacataaaattgattc :  82300
Seq2 : cgtacgcagtaataaatagaaacgtactactgagatatactaccgatatagagtataatgatttagttactttaataaccgttagacataaaattgattc :  82300
Seq3 : cgtacgcagtaataaatagaaacgtactactgagatatactaccgatatagagtataatgatttagttactttaataaccgttagacataaaattgattc :  82300
Seq4 : cgtacgcagtaataaatagaaacgtactactgagatatactaccgatatagagtataatgatttagttactttaataaccgttagacataaaattgattc :  82300

             *     82320          *     82340          *     82360          *     82380          *     82400
Seq1 : tatgaaaactgtgtttcaggtatttaacgaatcatccataaattatactccggttgatgatgattatggagaaccaatcattataacatcgtatcttcaa :  82400
Seq2 : tatgaaaactgtgtttcaggtatttaacgaatcatccataaattatactccggttgatgatgattatggagaaccaatcattataacatcgtatcttcaa :  82400
Seq3 : tatgaaaactgtgtttcaggtatttaacgaatcatccataaattatactccggttgatgatgattatggagaaccaatcattataacatcgtatcttcaa :  82400
Seq4 : tatgaaaactgtgtttcaggtatttaacgaatcatccataaattatactccggttgatgatgattatggagaaccaatcattataacatcgtatcttcaa :  82400

             *     82420          *     82440          *     82460          *     82480          *     82500
Seq1 : aaaggtcataacaagtttcctgtaaatttctatacatagatgtggtaatatctgacttatttcctagctttgttagactagatactacagaaactaata :  82500
Seq2 : aaaggtcataacaagtttcctgtaaatttctatacatagatgtggtaatatctgacttatttcctagctttgttagactagatactacagaaactaata :  82500
Seq3 : aaaggtcataacaagtttcctgtaaatttctatacatagatgtggtaatatctgacttatttcctagctttgttagactagatactacagaaactaata :  82500
Seq4 : aaaggtcataacaagtttcctgtaaatttctatacatagatgtggtaatatctgacttatttcctagctttgttagactagatactacagaaactaata :  82500

             *     82520          *     82540          *     82560          *     82580          *     82600
Seq1 : tagttaatagtgtactacaaacaggcgatggtaaaaagactcttcgtcttcccaaaatgttagagacggaaatagttgtcaagattctctatcgccctaa :  82600
Seq2 : tagttaatagtgtactacaaacaggcgatggtaaaaagactcttcgtcttcccaaaatgttagagacggaaatagttgtcaagattctctatcgccctaa :  82600
Seq3 : tagttaatagtgtactacaaacaggcgatggtaaaaagactcttcgtcttcccaaaatgttagagacggaaatagttgtcaagattctctatcgccctaa :  82600
Seq4 : tagttaatagtgtactacaaacaggcgatggtaaaaagactcttcgtcttcccaaaatgttagagacggaaatagttgtcaagattctctatcgccctaa :  82600
```

```
                 *     82620         *     82640         *     82660         *     82680         *     82700
Seq1 : tataccattaaaaattgttagatttttccgcaataacatggtaactggagtagagatagccgatagatctgttatttcagtcgctgattaatcaattagt :  82700
Seq2 : tataccattaaaaattgttagatttttccgcaataacatggtaactggagtagagatagccgatagatctgttatttcagtcgctgattaatcaattagt :  82700
Seq3 : tataccattaaaaattgttagatttttccgcaataacatggtaactggagtagagatagccgatagatctgttatttcagtcgctgattaatcaattagt :  82700
Seq4 : tataccattaaaaattgttagatttttccgcaataacatggtaactggagtagagatagccgatagatctgttatttcagtcgctgattaatcaattagt :  82700

                 *     82720         *     82740         *     82760         *     82780         *     82800
Seq1 : agagatgagataagaacattataataatcaataatatatcttatatctcgtttagaaaaatgctaatattaaaatagctaacgctagtaatccaatcgga :  82800
Seq2 : agagatgagataagaacattataataatcaataatatatcttatatctcgtttagaaaaatgctaatattaaaatagctaacgctagtaatccaatcgga :  82800
Seq3 : agagatgagataagaacattataataatcaataatatatcttatatctcgtttagaaaaatgctaatattaaaatagctaacgctagtaatccaatcgga :  82800
Seq4 : agagatgagataagaacattataataatcaataatatatcttatatctcgtttagaaaaatgctaatattaaaatagctaacgctagtaatccaatcgga :  82800

                 *     82820         *     82840         *     82860         *     82880         *     82900
Seq1 : agccatttgatatctataatagggtatctaatttcctgattcagatagcggacagctatattctcggtagctactcgtttggaatcacaaacattattta :  82900
Seq2 : agccatttgatatctataatagggtatctaatttcctgattcagatagcggacagctatattctcggtagctactcgtttggaatcacaaacattattta :  82900
Seq3 : agccatttgatatctataatagggtatctaatttcctgattcagatagcggacagctatattctcggtagctactcgtttggaatcacaaacattattta :  82900
Seq4 : agccatttgatatctataatagggtatctaatttcctgattcagatagcggacagctatattctcggtagctactcgtttggaatcacaaacattattta :  82900

                 *     82920         *     82940         *     82960         *     82980         *     83000
Seq1 : catctaatttactatctgtaatggaaacgtttcccaatgaaatggtacaatccgatacattgcattttgttatatttttttttaaagaggctggtaacaa :  83000
Seq2 : catctaatttactatctgtaatggaaacgtttcccaatgaaatggtacaatccgatacattgcattttgttatatttttttttaaagaggctggtaacaa :  83000
Seq3 : catctaatttactatctgtaatggaaacgtttcccaatgaaatggtacaatccgatacattgcattttgttatatttttttttaaagaggctggtaacaa :  83000
Seq4 : catctaatttactatctgtaatggaaacgtttcccaatgaaatggtacaatccgatacattgcattttgttatatttttttttaaagaggctggtaacaa :  83000

                 *     83020         *     83040         *     83060         *     83080         *     83100
Seq1 : cgcatcgcttcgtttacatggctcgtaccaacaataatagggtaatcttgtatctattcctatccgtactatgcttttatcaggataaatacatttacat :  83100
Seq2 : cgcatcgcttcgtttacatggctcgtaccaacaataatagggtaatcttgtatctattcctatccgtactatgcttttatcaggataaatacatttacat :  83100
Seq3 : cgcatcgcttcgtttacatggctcgtaccaacaataatagggtaatcttgtatctattcctatccgtactatgcttttatcaggataaatacatttacat :  83100
Seq4 : cgcatcgcttcgtttacatggctcgtaccaacaataatagggtaatcttgtatctattcctatccgtactatgcttttatcaggataaatacatttacat :  83100

                 *     83120         *     83140         *     83160         *     83180         *     83200
Seq1 : cgtatatcgtctttgttagcatcacagaatgcataaatttgttcgtccgtcatgataaaaatttaaagtgtaaatataactattatttttatagttgtaa :  83200
Seq2 : cgtatatcgtctttgttagcatcacagaatgcataaatttgttcgtccgtcatgataaaaatttaaagtgtaaatataactattatttttatagttgtaa :  83200
Seq3 : cgtatatcgtctttgttagcatcacagaatgcataaatttgttcgtccgtcatgataaaaatttaaagtgtaaatataactattatttttatagttgtaa :  83200
Seq4 : cgtatatcgtctttgttagcatcacagaatgcataaatttgttcgtccgtcatgataaaaatttaaagtgtaaatataactattatttttatagttgtaa :  83200

                 *     83220         *     83240         *     83260         *     83280         *     83300
Seq1 : taaaaagggaaatttgattgtataccttcggttctttaaaagaaactgacttgataaaaatggctgtaatctctaaggttacgtatagtctatatgatca :  83300
Seq2 : taaaaagggaaatttgattgtataccttcggttctttaaaagaaactgacttgataaaaatggctgtaatctctaaggttacgtatagtctatatgatca :  83300
Seq3 : taaaaagggaaatttgattgtataccttcggttctttaaaagaaactgacttgataaaaatggctgtaatctctaaggttacgtatagtctatatgatca :  83300
Seq4 : taaaaagggaaatttgattgtataccttcggttctttaaaagaaactgacttgataaaaatggctgtaatctctaaggttacgtatagtctatatgatca :  83300
```

```
                 *      83320         *      83340         *      83360         *      83380         *      83400
Seq1 : aaaagagattaatgctacagatattatcattagtcatgttaaaaatgacgacgatatcggtaccgttaaagatggtagactaggtgctatggatggggca :  83400
Seq2 : aaaagagattaatgctacagatattatcattagtcatgttaaaaatgacgacgatatcggtaccgttaaagatggtagactaggtgctatggatggggca :  83400
Seq3 : aaaagagattaatgctacagatattatcattagtcatgttaaaaatgacgacgatatcggtaccgttaaagatggtagactaggtgctatggatggggca :  83400
Seq4 : aaaagagattaatgctacagatattatcattagtcatgttaaaaatgacgacgatatcggtaccgttaaagatggtagactaggtgctatggatggggca :  83400

                 *      83420         *      83440         *      83460         *      83480         *      83500
Seq1 : ttatgtaagacttgtgggaaaacggaattggaatgtttcggtcactggggtaaagtaagtatttataaaactcatatagttaagcctgaatttatttcag :  83500
Seq2 : ttatgtaagacttgtgggaaaacggaattggaatgtttcggtcactggggtaaagtaagtatttataaaactcatatagttaagcctgaatttatttcag :  83500
Seq3 : ttatgtaagacttgtgggaaaacggaattggaatgtttcggtcactggggtaaagtaagtatttataaaactcatatagttaagcctgaatttatttcag :  83500
Seq4 : ttatgtaagacttgtgggaaaacggaattggaatgtttcggtcactggggtaaagtaagtatttataaaactcatatagttaagcctgaatttatttcag :  83500

                 *      83520         *      83540         *      83560         *      83580         *      83600
Seq1 : aaattattcgtttactgaatcatatatgtattcactgcggattattgcgttcacgagaaccgtattccgacgatattaacctaaaagagttatcgggaca :  83600
Seq2 : aaattattcgtttactgaatcatatatgtattcactgcggattattgcgttcacgagaaccgtattccgacgatattaacctaaaagagttatcgggaca :  83600
Seq3 : aaattattcgtttactgaatcatatatgtattcactgcggattattgcgttcacgagaaccgtattccgacgatattaacctaaaagagttatcgggaca :  83600
Seq4 : aaattattcgtttactgaatcatatatgtattcactgcggattattgcgttcacgagaaccgtattccgacgatattaacctaaaagagttatcgggaca :  83600

                 *      83620         *      83640         *      83660         *      83680         *      83700
Seq1 : cgctcttaggagattaaaggataaaatattatccaagaaaaagtcatgttggaacagtgaatgtatgcaaccgtatcaaaaaattactttttcaaagaaa :  83700
Seq2 : cgctcttaggagattaaaggataaaatattatccaagaaaaagtcatgttggaacagtgaatgtatgcaaccgtatcaaaaaattactttttcaaagaaa :  83700
Seq3 : cgctcttaggagattaaaggataaaatattatccaagaaaaagtcatgttggaacagtgaatgtatgcaaccgtatcaaaaaattactttttcaaagaaa :  83700
Seq4 : cgctcttaggagattaaaggataaaatattatccaagaaaaagtcatgttggaacagtgaatgtatgcaaccgtatcaaaaaattactttttcaaagaaa :  83700

                 *      83720         *      83740         *      83760         *      83780         *      83800
Seq1 : aaggtttgtttcgtcaacaagttggatgatattaacgttcctaattctctcatctatcaaaagttaatttctattcatgaaaagttttggccattattag :  83800
Seq2 : aaggtttgtttcgtcaacaagttggatgatattaacgttcctaattctctcatctatcaaaagttaatttctattcatgaaaagttttggccattattag :  83800
Seq3 : aaggtttgtttcgtcaacaagttggatgatattaacgttcctaattctctcatctatcaaaagttaatttctattcatgaaaagttttggccattattag :  83800
Seq4 : aaggtttgtttcgtcaacaagttggatgatattaacgttcctaattctctcatctatcaaaagttaatttctattcatgaaaagttttggccattattag :  83800

                 *      83820         *      83840         *      83860         *      83880         *      83900
Seq1 : aaattcatcaatatccagctaacttattttatacagactactttcccatccctccgttgattattagaccggctattagtttttggatagatagtatacc :  83900
Seq2 : aaattcatcaatatccagctaacttattttatacagactactttcccatccctccgttgattattagaccggctattagtttttggatagatagtatacc :  83900
Seq3 : aaattcatcaatatccagctaacttattttatacagactactttcccatccctccgttgattattagaccggctattagtttttggatagatagtatacc :  83900
Seq4 : aaattcatcaatatccagctaacttattttatacagactactttcccatccctccgttgattattagaccggctattagtttttggatagatagtatacc :  83900

                 *      83920         *      83940         *      83960         *      83980         *      84000
Seq1 : caaagaaaccaatgaattaacttacttattaggtatgatcgttaagaattgtaacttgaatgctgatgaacaggttatccagaaggcggtaatagaatac :  84000
Seq2 : caaagaaaccaatgaattaacttacttattaggtatgatcgttaagaattgtaacttgaatgctgatgaacaggttatccagaaggcggtaatagaatac :  84000
Seq3 : caaagaaaccaatgaattaacttacttattaggtatgatcgttaagaattgtaacttgaatgctgatgaacaggttatccagaaggcggtaatagaatac :  84000
Seq4 : caaagaaaccaatgaattaacttacttattaggtatgatcgttaagaattgtaacttgaatgctgatgaacaggttatccagaaggcggtaatagaatac :  84000
```

```
               *     84020           *     84040           *     84060           *     84080           *     84100
Seq1 : gatgatattaaaattatttctaataacacttccagtatcaatttatcatatattacatccggcaaaaataatatgattagaagttatatcgtcgcccgac :  84100
Seq2 : gatgatattaaaattatttctaataacacttccagtatcaatttatcatatattacatccggcaaaaataatatgattagaagttatatcgtcgcccgac :  84100
Seq3 : gatgatattaaaattatttctaataacacttccagtatcaatttatcatatattacatccggcaaaaataatatgattagaagttatatcgtcgcccgac :  84100
Seq4 : gatgatattaaaattatttctaataacacttccagtatcaatttatcatatattacatccggcaaaaataatatgattagaagttatatcgtcgcccgac :  84100

               *     84120           *     84140           *     84160           *     84180           *     84200
Seq1 : gaaaagatcagacggctagatctgtaattggtcccagtacatctatcaccgttaatgaggtaggaatgcccgcatatattagaaatacacttacagaaaa :  84200
Seq2 : gaaaagatcagacggctagatctgtaattggtcccagtacatctatcaccgttaatgaggtaggaatgcccgcatatattagaaatacacttacagaaaa :  84200
Seq3 : gaaaagatcagacggctagatctgtaattggtcccagtacatctatcaccgttaatgaggtaggaatgcccgcatatattagaaatacacttacagaaaa :  84200
Seq4 : gaaaagatcagacggctagatctgtaattggtcccagtacatctatcaccgttaatgaggtaggaatgcccgcatatattagaaatacacttacagaaaa :  84200

               *     84220           *     84240           *     84260           *     84280           *     84300
Seq1 : gatatttgttaatgcctttacagtggataaagttaaacaactattagcatcaaaccaagttaaattttactttaataaacgattaaaccaattaacaaga :  84300
Seq2 : gatatttgttaatgcctttacagtggataaagttaaacaactattagcatcaaaccaagttaaattttactttaataaacgattaaaccaattaacaaga :  84300
Seq3 : gatatttgttaatgcctttacagtggataaagttaaacaactattagcatcaaaccaagttaaattttactttaataaacgattaaaccaattaacaaga :  84300
Seq4 : gatatttgttaatgcctttacagtggataaagttaaacaactattagcatcaaaccaagttaaattttactttaataaacgattaaaccaattaacaaga :  84300

               *     84320           *     84340           *     84360           *     84380           *     84400
Seq1 : atacgccaaggaaagtttatcaaaaataaaatacatttattgcctggtgattgggtagaagtagctgttcaagaatatacaagtattatttttggaagac :  84400
Seq2 : atacgccaaggaaagtttatcaaaaataaaatacatttattgcctggtgattgggtagaagtagctgttcaagaatatacaagtattatttttggaagac :  84400
Seq3 : atacgccaaggaaagtttatcaaaaataaaatacatttattgcctggtgattgggtagaagtagctgttcaagaatatacaagtattatttttggaagac :  84400
Seq4 : atacgccaaggaaagtttatcaaaaataaaatacatttattgcctggtgattgggtagaagtagctgttcaagaatatacaagtattatttttggaagac :  84400

               *     84420           *     84440           *     84460           *     84480           *     84500
Seq1 : agccgtctctacatagatacaacgtcatcgcttcatctatcagagctaccgaaggagatactatcaaaatatctcccggaattgccaactctcaaaatgc :  84500
Seq2 : agccgtctctacatagatacaacgtcatcgcttcatctatcagagctaccgaaggagatactatcaaaatatctcccggaattgccaactctcaaaatgc :  84500
Seq3 : agccgtctctacatagatacaacgtcatcgcttcatctatcagagctaccgaaggagatactatcaaaatatctcccggaattgccaactctcaaaatgc :  84500
Seq4 : agccgtctctacatagatacaacgtcatcgcttcatctatcagagctaccgaaggagatactatcaaaatatctcccggaattgccaactctcaaaatgc :  84500

               *     84520           *     84540           *     84560           *     84580           *     84600
Seq1 : tgatttcgacggagatgaagaatggatgatattagaacaaaatcctaaagctgtaattgaacaaagtattcttatgtatccgacgacgttactcaaacac :  84600
Seq2 : tgatttcgacggagatgaagaatggatgatattagaacaaaatcctaaagctgtaattgaacaaagtattcttatgtatccgacgacgttactcaaacac :  84600
Seq3 : tgatttcgacggagatgaagaatggatgatattagaacaaaatcctaaagctgtaattgaacaaagtattcttatgtatccgacgacgttactcaaacac :  84600
Seq4 : tgatttcgacggagatgaagaatggatgatattagaacaaaatcctaaagctgtaattgaacaaagtattcttatgtatccgacgacgttactcaaacac :  84600

               *     84620           *     84640           *     84660           *     84680           *     84700
Seq1 : gatattcatggagcccccgtttatggatctattcaagatgaaatcgtagcagcgtattcattgtttaggatacaagatctttgtttagatgaagtattga :  84700
Seq2 : gatattcatggagcccccgtttatggatctattcaagatgaaatcgtagcagcgtattcattgtttaggatacaagatctttgtttagatgaagtattga :  84700
Seq3 : gatattcatggagcccccgtttatggatctattcaagatgaaatcgtagcagcgtattcattgtttaggatacaagatctttgtttagatgaagtattga :  84700
Seq4 : gatattcatggagcccccgtttatggatctattcaagatgaaatcgtagcagcgtattcattgtttaggatacaagatctttgtttagatgaagtattga :  84700
```

```
                *     84720         *     84740         *     84760         *     84780         *     84800
Seq1 : acatcttggggaaatatggaagagagttcgatcctaaaggtaaatgtaaattcagcggtaaagatatctatacttacttgataggtgaaaagattaatta :  84800
Seq2 : acatcttggggaaatatggaagagagttcgatcctaaaggtaaatgtaaattcagcggtaaagatatctatacttacttgataggtgaaaagattaatta :  84800
Seq3 : acatcttggggaaatatggaagagagttcgatcctaaaggtaaatgtaaattcagcggtaaagatatctatacttacttgataggtgaaaagattaatta :  84800
Seq4 : acatcttggggaaatatggaagagagttcgatcctaaaggtaaatgtaaattcagcggtaaagatatctatacttacttgataggtgaaaagattaatta :  84800

                *     84820         *     84840         *     84860         *     84880         *     84900
Seq1 : tccgggtctcttaaaggatggtgaaattattgcaaacgacgtagatagtaattttgttgtggctatgaggcatctgtcattggctggactcttatccgat :  84900
Seq2 : tccgggtctcttaaaggatggtgaaattattgcaaacgacgtagatagtaattttgttgtggctatgaggcatctgtcattggctggactcttatccgat :  84900
Seq3 : tccgggtctcttaaaggatggtgaaattattgcaaacgacgtagatagtaattttgttgtggctatgaggcatctgtcattggctggactcttatccgat :  84900
Seq4 : tccgggtctcttaaaggatggtgaaattattgcaaacgacgtagatagtaattttgttgtggctatgaggcatctgtcattggctggactcttatccgat :  84900

                *     84920         *     84940         *     84960         *     84980         *     85000
Seq1 : cataagtcgaacgtggaaggtatcaactttattatcaagtcatcttatgtttttaagagatatctatctatttacggttttggggtgacattcaaagatc :  85000
Seq2 : cataagtcgaacgtggaaggtatcaactttattatcaagtcatcttatgtttttaagagatatctatctatttacggttttggggtgacattcaaagatc :  85000
Seq3 : cataagtcgaacgtggaaggtatcaactttattatcaagtcatcttatgtttttaagagatatctatctatttacggttttggggtgacattcaaagatc :  85000
Seq4 : cataagtcgaacgtggaaggtatcaactttattatcaagtcatcttatgtttttaagagatatctatctatttacggttttggggtgacattcaaagatc :  85000

                *     85020         *     85040         *     85060         *     85080         *     85100
Seq1 : tgagaccaaattcgacgttcactaataaattggaggccatcaacgtagaaaaaatagaacttatcaaagaagcatacgccaaatatctcaacgatgtaag :  85100
Seq2 : tgagaccaaattcgacgttcactaataaattggaggccatcaacgtagaaaaaatagaacttatcaaagaagcatacgccaaatatctcaacgatgtaag :  85100
Seq3 : tgagaccaaattcgacgttcactaataaattggaggccatcaacgtagaaaaaatagaacttatcaaagaagcatacgccaaatatctcaacgatgtaag :  85100
Seq4 : tgagaccaaattcgacgttcactaataaattggaggccatcaacgtagaaaaaatagaacttatcaaagaagcatacgccaaatatctcaacgatgtaag :  85100

                *     85120         *     85140         *     85160         *     85180         *     85200
Seq1 : agacgggaaaatagttccattatctaaagctttagaggcggactatgtggaatccatgttatccaacttgacaaatcttaatatccgagagatagaagaa :  85200
Seq2 : agacgggaaaatagttccattatctaaagctttagaggcggactatgtggaatccatgttatccaacttgacaaatcttaatatccgagagatagaagaa :  85200
Seq3 : agacgggaaaatagttccattatctaaagctttagaggcggactatgtggaatccatgttatccaacttgacaaatcttaatatccgagagatagaagaa :  85200
Seq4 : agacgggaaaatagttccattatctaaagctttagaggcggactatgtggaatccatgttatccaacttgacaaatcttaatatccgagagatagaagaa :  85200

                *     85220         *     85240         *     85260         *     85280         *     85300
Seq1 : catatgagacaaacgctgatagatgatccagataataacctcctgaaaatggccaaagcgggttataaagtaaatcctacagaactaatgtatattctag :  85300
Seq2 : catatgagacaaacgctgatagatgatccagataataacctcctgaaaatggccaaagcgggttataaagtaaatcctacagaactaatgtatattctag :  85300
Seq3 : catatgagacaaacgctgatagatgatccagataataacctcctgaaaatggccaaagcgggttataaagtaaatcctacagaactaatgtatattctag :  85300
Seq4 : catatgagacaaacgctgatagatgatccagataataacctcctgaaaatggccaaagcgggttataaagtaaatcctacagaactaatgtatattctag :  85300

                *     85320         *     85340         *     85360         *     85380         *     85400
Seq1 : gtacgtatggacaacaaaggattgatggtgaaccagcagagactcgagtattgggtagagtcttaccttactatcttccagactctaaggatccagaagg :  85400
Seq2 : gtacgtatggacaacaaaggattgatggtgaaccagcagagactcgagtattgggtagagtcttaccttactatcttccagactctaaggatccagaagg :  85400
Seq3 : gtacgtatggacaacaaaggattgatggtgaaccagcagagactcgagtattgggtagagtcttaccttactatcttccagactctaaggatccagaagg :  85400
Seq4 : gtacgtatggacaacaaaggattgatggtgaaccagcagagactcgagtattgggtagagtcttaccttactatcttccagactctaaggatccagaagg :  85400
```

```
             *     85420         *     85440         *     85460         *     85480         *     85500
Seq1 : aagaggttacattcttaattctttaacaaaaggattaacaggttctcaatattacttttcgatgctggttgcaagatctcaatctactgatatcgtctgt :  85500
Seq2 : aagaggttacattcttaattctttaacaaaaggattaacaggttctcaatattacttttcgatgctggttgcaagatctcaatctactgatatcgtctgt :  85500
Seq3 : aagaggttacattcttaattctttaacaaaaggattaacaggttctcaatattacttttcgatgctggttgcaagatctcaatctactgatatcgtctgt :  85500
Seq4 : aagaggttacattcttaattctttaacaaaaggattaacaggttctcaatattacttttcgatgctggttgcaagatctcaatctactgatatcgtctgt :  85500

             *     85520         *     85540         *     85560         *     85580         *     85600
Seq1 : gaaacatcacgtaccggaacactggctagaaaaatcattaaaaagatggaggatatggtggtcgacggatacggacaagtagttataggtaatacgctca :  85600
Seq2 : gaaacatcacgtaccggaacactggctagaaaaatcattaaaaagatggaggatatggtggtcgacggatacggacaagtagttataggtaatacgctca :  85600
Seq3 : gaaacatcacgtaccggaacactggctagaaaaatcattaaaaagatggaggatatggtggtcgacggatacggacaagtagttataggtaatacgctca :  85600
Seq4 : gaaacatcacgtaccggaacactggctagaaaaatcattaaaaagatggaggatatggtggtcgacggatacggacaagtagttataggtaatacgctca :  85600

             *     85620         *     85640         *     85660         *     85680         *     85700
Seq1 : tcaagtacgccgccaattataccaaaattctaggctcagtatgtaaacctgtagatcttatctatccagatgagtccatgacttggtatttggaaattag :  85700
Seq2 : tcaagtacgccgccaattataccaaaattctaggctcagtatgtaaacctgtagatcttatctatccagatgagtccatgacttggtatttggaaattag :  85700
Seq3 : tcaagtacgccgccaattataccaaaattctaggctcagtatgtaaacctgtagatcttatctatccagatgagtccatgacttggtatttggaaattag :  85700
Seq4 : tcaagtacgccgccaattataccaaaattctaggctcagtatgtaaacctgtagatcttatctatccagatgagtccatgacttggtatttggaaattag :  85700

             *     85720         *     85740         *     85760         *     85780         *     85800
Seq1 : tgctctgtggaataaaataaaacagggattcgtttactctcagaaacagaaacttgcaaagaagacattggcgccgtttaatttcctagtattcgtcaaa :  85800
Seq2 : tgctctgtggaataaaataaaacagggattcgtttactctcagaaacagaaacttgcaaagaagacattggcgccgtttaatttcctagtattcgtcaaa :  85800
Seq3 : tgctctgtggaataaaataaaacagggattcgtttactctcagaaacagaaacttgcaaagaagacattggcgccgtttaatttcctagtattcgtcaaa :  85800
Seq4 : tgctctgtggaataaaataaaacagggattcgtttactctcagaaacagaaacttgcaaagaagacattggcgccgtttaatttcctagtattcgtcaaa :  85800

             *     85820         *     85840         *     85860         *     85880         *     85900
Seq1 : cccaccactgaggataatgctattaaggttaaggatctgtacgatatgattcataacgtcattgatgatgtgagagagaaatacttctttacggtatcta :  85900
Seq2 : cccaccactgaggataatgctattaaggttaaggatctgtacgatatgattcataacgtcattgatgatgtgagagagaaatacttctttacggtatcta :  85900
Seq3 : cccaccactgaggataatgctattaaggttaaggatctgtacgatatgattcataacgtcattgatgatgtgagagagaaatacttctttacggtatcta :  85900
Seq4 : cccaccactgaggataatgctattaaggttaaggatctgtacgatatgattcataacgtcattgatgatgtgagagagaaatacttctttacggtatcta :  85900

             *     85920         *     85940         *     85960         *     85980         *     86000
Seq1 : atatagattttatggagtatatattcttgacgcatcttaatccttctagaattagaattacaaaagaaacggctatcactatctttgaaaagttctatga :  86000
Seq2 : atatagattttatggagtatatattcttgacgcatcttaatccttctagaattagaattacaaaagaaacggctatcactatctttgaaaagttctatga :  86000
Seq3 : atatagattttatggagtatatattcttgacgcatcttaatccttctagaattagaattacaaaagaaacggctatcactatctttgaaaagttctatga :  86000
Seq4 : atatagattttatggagtatatattcttgacgcatcttaatccttctagaattagaattacaaaagaaacggctatcactatctttgaaaagttctatga :  86000

             *     86020         *     86040         *     86060         *     86080         *     86100
Seq1 : aaaactcaattatactctaggtggtggaactcctattggaattatttctgcacaggtattgtctgagaagtttacacaacaagccctgtccagttttcac :  86100
Seq2 : aaaactcaattatactctaggtggtggaactcctattggaattatttctgcacaggtattgtctgagaagtttacacaacaagccctgtccagttttcac :  86100
Seq3 : aaaactcaattatactctaggtggtggaactcctattggaattatttctgcacaggtattgtctgagaagtttacacaacaagccctgtccagttttcac :  86100
Seq4 : aaaactcaattatactctaggtggtggaactcctattggaattatttctgcacaggtattgtctgagaagtttacacaacaagccctgtccagttttcac :  86100
```

```
               *     86120         *     86140         *     86160         *     86180         *     86200
Seq1 : actactgaaaaaagtggtgccgtcaaacaaaaacttggtttcaacgagtttaataacttgactaatttgagtaagaataagaccgaaattatcactctgg :  86200
Seq2 : actactgaaaaaagtggtgccgtcaaacaaaaacttggtttcaacgagtttaataacttgactaatttgagtaagaataagaccgaaattatcactctgg :  86200
Seq3 : actactgaaaaaagtggtgccgtcaaacaaaaacttggtttcaacgagtttaataacttgactaatttgagtaagaataagaccgaaattatcactctgg :  86200
Seq4 : actactgaaaaaagtggtgccgtcaaacaaaaacttggtttcaacgagtttaataacttgactaatttgagtaagaataagaccgaaattatcactctgg :  86200

               *     86220         *     86240         *     86260         *     86280         *     86300
Seq1 : tatccgatgatatctctaaacttcaatctgttaagattaatttcgaatttgtatgtttgggagaattaaatccagacatcactcttcgaaaagaaacaga :  86300
Seq2 : tatccgatgatatctctaaacttcaatctgttaagattaatttcgaatttgtatgtttgggagaattaaatccagacatcactcttcgaaaagaaacaga :  86300
Seq3 : tatccgatgatatctctaaacttcaatctgttaagattaatttcgaatttgtatgtttgggagaattaaatccagacatcactcttcgaaaagaaacaga :  86300
Seq4 : tatccgatgatatctctaaacttcaatctgttaagattaatttcgaatttgtatgtttgggagaattaaatccagacatcactcttcgaaaagaaacaga :  86300

               *     86320         *     86340         *     86360         *     86380         *     86400
Seq1 : taggtatgtagtagatataatagtcaatagattatacatcaagagagcagaaattaccgaattagtcgtcgaatatatgattgaacgattcatctccttt :  86400
Seq2 : taggtatgtagtagatataatagtcaatagattatacatcaagagagcagaaattaccgaattagtcgtcgaatatatgattgaacgattcatctccttt :  86400
Seq3 : taggtatgtagtagatataatagtcaatagattatacatcaagagagcagaaattaccgaattagtcgtcgaatatatgattgaacgattcatctccttt :  86400
Seq4 : taggtatgtagtagatataatagtcaatagattatacatcaagagagcagaaattaccgaattagtcgtcgaatatatgattgaacgattcatctccttt :  86400

               *     86420         *     86440         *     86460         *     86480         *     86500
Seq1 : agcgtcattgtaaaggaatggggtatggaaacattcattgaggacgaggataatattagatttactgtctatctaaatttcgttgaaccagaggaattga :  86500
Seq2 : agcgtcattgtaaaggaatggggtatggaaacattcattgaggacgaggataatattagatttactgtctatctaaatttcgttgaaccagaggaattga :  86500
Seq3 : agcgtcattgtaaaggaatggggtatggaaacattcattgaggacgaggataatattagatttactgtctatctaaatttcgttgaaccagaggaattga :  86500
Seq4 : agcgtcattgtaaaggaatggggtatggaaacattcattgaggacgaggataatattagatttactgtctatctaaatttcgttgaaccagaggaattga :  86500

               *     86520         *     86540         *     86560         *     86580         *     86600
Seq1 : atcttagtaagtttatgatggttcttccgggggcagccaacaagggaaagattagtaaattcaagattcctatctctgattatacgggttatgacgactt :  86600
Seq2 : atcttagtaagtttatgatggttcttccgggggcagccaacaagggaaagattagtaaattcaagattcctatctctgattatacgggttatgacgactt :  86600
Seq3 : atcttagtaagtttatgatggttcttccgggggcagccaacaagggaaagattagtaaattcaagattcctatctctgattatacgggttatgacgactt :  86600
Seq4 : atcttagtaagtttatgatggttcttccgggggcagccaacaagggaaagattagtaaattcaagattcctatctctgattatacgggttatgacgactt :  86600

               *     86620         *     86640         *     86660         *     86680         *     86700
Seq1 : caatcaaacaaaaaagctcaataagatgactgtagaactcatgaatctaaaagaattaggttctttcgatttggaaaacgtcaacgtgtatcctggagta :  86700
Seq2 : caatcaaacaaaaaagctcaataagatgactgtagaactcatgaatctaaaagaattaggttctttcgatttggaaaacgtcaacgtgtatcctggagta :  86700
Seq3 : caatcaaacaaaaaagctcaataagatgactgtagaactcatgaatctaaaagaattaggttctttcgatttggaaaacgtcaacgtgtatcctggagta :  86700
Seq4 : caatcaaacaaaaaagctcaataagatgactgtagaactcatgaatctaaaagaattaggttctttcgatttggaaaacgtcaacgtgtatcctggagta :  86700

               *     86720         *     86740         *     86760         *     86780         *     86800
Seq1 : tggaatacatacgatatcttcggtatcgaggccgctcgtgaatacttgtgcgaagccatgttaaacacctatggagaagggttcgattatctgtatcagc :  86800
Seq2 : tggaatacatacgatatcttcggtatcgaggccgctcgtgaatacttgtgcgaagccatgttaaacacctatggagaagggttcgattatctgtatcagc :  86800
Seq3 : tggaatacatacgatatcttcggtatcgaggccgctcgtgaatacttgtgcgaagccatgttaaacacctatggagaagggttcgattatctgtatcagc :  86800
Seq4 : tggaatacatacgatatcttcggtatcgaggccgctcgtgaatacttgtgcgaagccatgttaaacacctatggagaagggttcgattatctgtatcagc :  86800
```

```
                  *     86820         *     86840         *     86860         *     86880         *     86900
Seq1 : cttgtgatcttctcgctagtttactatgtgctagttacgaaccagaatcagtgaataaattcaagttcggcgcagctagtactcttaagagagctacgtt :  86900
Seq2 : cttgtgatcttctcgctagtttactatgtgctagttacgaaccagaatcagtgaataaattcaagttcggcgcagctagtactcttaagagagctacgtt :  86900
Seq3 : cttgtgatcttctcgctagtttactatgtgctagttacgaaccagaatcagtgaataaattcaagttcggcgcagctagtactcttaagagagctacgtt :  86900
Seq4 : cttgtgatcttctcgctagtttactatgtgctagttacgaaccagaatcagtgaataaattcaagttcggcgcagctagtactcttaagagagctacgtt :  86900

                  *     86920         *     86940         *     86960         *     86980         *     87000
Seq1 : cggagacaataaagcattgttaaacgcggctcttcataaaaagtcagaacctattaacgataatagtagctgccacttttttagcaaggtccctaatata :  87000
Seq2 : cggagacaataaagcattgttaaacgcggctcttcataaaaagtcagaacctattaacgataatagtagctgccacttttttagcaaggtccctaatata :  87000
Seq3 : cggagacaataaagcattgttaaacgcggctcttcataaaaagtcagaacctattaacgataatagtagctgccacttttttagcaaggtccctaatata :  87000
Seq4 : cggagacaataaagcattgttaaacgcggctcttcataaaaagtcagaacctattaacgataatagtagctgccacttttttagcaaggtccctaatata :  87000

                  *     87020         *     87040         *     87060         *     87080         *     87100
Seq1 : ggaactggatattacaaatactttatcgacttgggtcttctcatgagaatggaaaggaaactatctgataagatatcttctcaaaagatcaaggaaatgg :  87100
Seq2 : ggaactggatattacaaatactttatcgacttgggtcttctcatgagaatggaaaggaaactatctgataagatatcttctcaaaagatcaaggaaatgg :  87100
Seq3 : ggaactggatattacaaatactttatcgacttgggtcttctcatgagaatggaaaggaaactatctgataagatatcttctcaaaagatcaaggaaatgg :  87100
Seq4 : ggaactggatattacaaatactttatcgacttgggtcttctcatgagaatggaaaggaaactatctgataagatatcttctcaaaagatcaaggaaatgg :  87100

                  *     87120         *     87140         *     87160         *     87180         *     87200
Seq1 : aagaaacagaagacttttaattcttatcaataacatatttttctatgatctgtcttttaaacgatggattttccacaaatgcgcctctcaagtccctcat :  87200
Seq2 : aagaaacagaagacttttaattcttatcaataacatatttttctatgatctgtcttttaaacgatggattttccacaaatgcgcctctcaagtccctcat :  87200
Seq3 : aagaaacagaagacttttaattcttatcaataacatatttttctatgatctgtcttttaaacgatggattttccacaaatgcgcctctcaagtccctcat :  87200
Seq4 : aagaaacagaagacttttaattcttatcaataacatatttttctatgatctgtcttttaaacgatggattttccacaaatgcgcctctcaagtccctcat :  87200

                  *     87220         *     87240         *     87260         *     87280         *     87300
Seq1 : agaatgatacacgtataaaaaatatagcataggcaatgactccttatttttagacattagatatgccaaaatcatagccccgcttctatttactcccgca :  87300
Seq2 : agaatgatacacgtataaaaaatatagcataggcaatgactccttatttttagacattagatatgccaaaatcatagccccgcttctatttactcccgca :  87300
Seq3 : agaatgatacacgtataaaaaatatagcataggcaatgactccttatttttagacattagatatgccaaaatcatagccccgcttctatttactcccgca :  87300
Seq4 : agaatgatacacgtataaaaaatatagcataggcaatgactccttatttttagacattagatatgccaaaatcatagccccgcttctatttactcccgca :  87300

                  *     87320         *     87340         *     87360         *     87380         *     87400
Seq1 : gcacaatgaaccaacacgggctcgtttcgttgatcacatttagataaaaaggcggttacgtcgtcaaaatatttactaatatcggtagttgtatcatcta :  87400
Seq2 : gcacaatgaaccaacacgggctcgtttcgttgatcacatttagataaaaaggcggttacgtcgtcaaaatatttactaatatcggtagttgtatcatcta :  87400
Seq3 : gcacaatgaaccaacacgggctcgtttcgttgatcacatttagataaaaaggcggttacgtcgtcaaaatatttactaatatcggtagttgtatcatcta :  87400
Seq4 : gcacaatgaaccaacacgggctcgtttcgttgatcacatttagataaaaaggcggttacgtcgtcaaaatatttactaatatcggtagttgtatcatcta :  87400

                  *     87420         *     87440         *     87460         *     87480         *     87500
Seq1 : ccaacggtatatgaataatattaatattagagttaggtaatgtatatttatccatcgtcaaatttaaaacatatttgaacttaacttcagatgatggtgc :  87500
Seq2 : ccaacggtatatgaataatattaatattagagttaggtaatgtatatttatccatcgtcaaatttaaaacatatttgaacttaacttcagatgatggtgc :  87500
Seq3 : ccaacggtatatgaataatattaatattagagttaggtaatgtatatttatccatcgtcaaatttaaaacatatttgaacttaacttcagatgatggtgc :  87500
Seq4 : ccaacggtatatgaataatattaatattagagttaggtaatgtatatttatccatcgtcaaatttaaaacatatttgaacttaacttcagatgatggtgc :  87500
```

```
                 *     87520          *     87540          *     87560          *     87580          *     87600
Seq1 : atccatagcattttataatttcccaaatacacattattggttacccttgtcattatagtgggagatttggctctgtgcatatctccagttgaacgtagt :  87600
Seq2 : atccatagcattttataatttcccaaatacacattattggttacccttgtcattatagtgggagatttggctctgtgcatatctccagttgaacgtagt :  87600
Seq3 : atccatagcattttataatttcccaaatacacattattggttacccttgtcattatagtgggagatttggctctgtgcatatctccagttgaacgtagt :  87600
Seq4 : atccatagcattttataatttcccaaatacacattattggttacccttgtcattatagtgggagatttggctctgtgcatatctccagttgaacgtagt :  87600

                 *     87620          *     87640          *     87660          *     87680          *     87700
Seq1 : agtaagtatttatacaaacttttcttatccatttataacgtacaaatggataaaactactttatcggtaaacgcgtgtaatttagaatacgttagagaaa :  87700
Seq2 : agtaagtatttatacaaacttttcttatccatttataacgtacaaatggataaaactactttatcggtaaacgcgtgtaatttagaatacgttagagaaa :  87700
Seq3 : agtaagtatttatacaaacttttcttatccatttataacgtacaaatggataaaactactttatcggtaaacgcgtgtaatttagaatacgttagagaaa :  87700
Seq4 : agtaagtatttatacaaacttttcttatccatttataacgtacaaatggataaaactactttatcggtaaacgcgtgtaatttagaatacgttagagaaa :  87700

                 *     87720          *     87740          *     87760          *     87780          *     87800
Seq1 : aggctatagtaggcgtacaagcagccaaaacatcaacacttatattctttgttattatattggcaattagtgcgctattactctggtttcagacgtctga :  87800
Seq2 : aggctatagtaggcgtacaagcagccaaaacatcaacacttatattctttgttattatattggcaattagtgcgctattactctggtttcagacgtctga :  87800
Seq3 : aggctatagtaggcgtacaagcagccaaaacatcaacacttatattctttgttattatattggcaattagtgcgctattactctggtttcagacgtctga :  87800
Seq4 : aggctatagtaggcgtacaagcagccaaaacatcaacacttatattctttgttattatattggcaattagtgcgctattactctggtttcagacgtctga :  87800

                 *     87820          *     87840          *     87860          *     87880          *     87900
Seq1 : taatccagtctttaatgaattaacgagatatatgcgaattaaaaatacggttaacgattggaaatcattaacggatagcaaaacaaaattagaaagtgat :  87900
Seq2 : taatccagtctttaatgaattaacgagatatatgcgaattaaaaatacggttaacgattggaaatcattaacggatagcaaaacaaaattagaaagtgat :  87900
Seq3 : taatccagtctttaatgaattaacgagatatatgcgaattaaaaatacggttaacgattggaaatcattaacggatagcaaaacaaaattagaaagtgat :  87900
Seq4 : taatccagtctttaatgaattaacgagatatatgcgaattaaaaatacggttaacgattggaaatcattaacggatagcaaaacaaaattagaaagtgat :  87900

                 *     87920          *     87940          *     87960          *     87980          *     88000
Seq1 : agaggtagacttctagccgctggtaaggatgatatattcgaattcaaatgtgtggatttcggcgcctattttatagctatgcgattggataagaaaacat :  88000
Seq2 : agaggtagacttctagccgctggtaaggatgatatattcgaattcaaatgtgtggatttcggcgcctattttatagctatgcgattggataagaaaacat :  88000
Seq3 : agaggtagacttctagccgctggtaaggatgatatattcgaattcaaatgtgtggatttcggcgcctattttatagctatgcgattggataagaaaacat :  88000
Seq4 : agaggtagacttctagccgctggtaaggatgatatattcgaattcaaatgtgtggatttcggcgcctattttatagctatgcgattggataagaaaacat :  88000

                 *     88020          *     88040          *     88060          *     88080          *     88100
Seq1 : atctgccgcaagctattaggcgaggtactggagacgcgtggatggttaaaaaggcggcaaaggtcgatccatctgctcaacaattttgtcagtatttgat :  88100
Seq2 : atctgccgcaagctattaggcgaggtactggagacgcgtggatggttaaaaaggcggcaaaggtcgatccatctgctcaacaattttgtcagtatttgat :  88100
Seq3 : atctgccgcaagctattaggcgaggtactggagacgcgtggatggttaaaaaggcggcaaaggtcgatccatctgctcaacaattttgtcagtatttgat :  88100
Seq4 : atctgccgcaagctattaggcgaggtactggagacgcgtggatggttaaaaaggcggcaaaggtcgatccatctgctcaacaattttgtcagtatttgat :  88100

                 *     88120          *     88140          *     88160          *     88180          *     88200
Seq1 : aaaacacaagtctaataatgttattacttgtggtaatgagatgttaaatgaattaggttatagcggttattttatgtcaccgcattggtgttccgatttt :  88200
Seq2 : aaaacacaagtctaataatgttattacttgtggtaatgagatgttaaatgaattaggttatagcggttattttatgtcaccgcattggtgttccgatttt :  88200
Seq3 : aaaacacaagtctaataatgttattacttgtggtaatgagatgttaaatgaattaggttatagcggttattttatgtcaccgcattggtgttccgatttt :  88200
Seq4 : aaaacacaagtctaataatgttattacttgtggtaatgagatgttaaatgaattaggttatagcggttattttatgtcaccgcattggtgttccgatttt :  88200
```

```
                  *     88220         *     88240         *     88260         *     88280         *     88300
Seq1 : agtaatatggaatagtgttagataaatgcggtaacgaatgttcctgtaaggaaccataacagtttagatttaacgttaaagatgagcataaacataataa :  88300
Seq2 : agtaatatggaatagtgttagataaatgcggtaacgaatgttcctgtaaggaaccataacagtttagatttaacgttaaagatgagcataaacataataa :  88300
Seq3 : agtaatatggaatagtgttagataaatgcggtaacgaatgttcctgtaaggaaccataacagtttagatttaacgttaaagatgagcataaacataataa :  88300
Seq4 : agtaatatggaatagtgttagataaatgcggtaacgaatgttcctgtaaggaaccataacagtttagatttaacgttaaagatgagcataaacataataa :  88300

                  *     88320         *     88340         *     88360         *     88380         *     88400
Seq1 : acaaaattacaatcaaacctataacattaatatcaaacaatccaaaaaatgaaatcagtggagtagtaaacgcgtacataactcctggataacgtttagt :  88400
Seq2 : acaaaattacaatcaaacctataacattaatatcaaacaatccaaaaaatgaaatcagtggagtagtaaacgcgtacataactcctggataacgtttagt :  88400
Seq3 : acaaaattacaatcaaacctataacattaatatcaaacaatccaaaaaatgaaatcagtggagtagtaaacgcgtacataactcctggataacgtttagt :  88400
Seq4 : acaaaattacaatcaaacctataacattaatatcaaacaatccaaaaaatgaaatcagtggagtagtaaacgcgtacataactcctggataacgtttagt :  88400

                  *     88420         *     88440         *     88460         *     88480         *     88500
Seq1 : agctgccgttcctattctagaccaaaaattcggtttcatgttttcgaaacggtgttctgcaacaagtcggggatcgtgttctacatatttggcggcatta :  88500
Seq2 : agctgccgttcctattctagaccaaaaattcggtttcatgttttcgaaacggtgttctgcaacaagtcggggatcgtgttctacatatttggcggcatta :  88500
Seq3 : agctgccgttcctattctagaccaaaaattcggtttcatgttttcgaaacggtgttctgcaacaagtcggggatcgtgttctacatatttggcggcatta :  88500
Seq4 : agctgccgttcctattctagaccaaaaattcggtttcatgttttcgaaacggtgttctgcaacaagtcggggatcgtgttctacatatttggcggcatta :  88500

                  *     88520         *     88540         *     88560         *     88580         *     88600
Seq1 : tccagtatctgcctattgatcttcatttcgttttcaattctggctatttcaaaataaaatcccgatgatagacctccagactttataatttcatctacga :  88600
Seq2 : tccagtatctgcctattgatcttcatttcgttttcaattctggctatttcaaaataaaatcccgatgatagacctccagactttataatttcatctacga :  88600
Seq3 : tccagtatctgcctattgatcttcatttcgttttcaattctggctatttcaaaataaaatcccgatgatagacctccagactttataatttcatctacga :  88600
Seq4 : tccagtatctgcctattgatcttcatttcgttttcaattctggctatttcaaaataaaatcccgatgatagacctccagactttataatttcatctacga :  88600

                  *     88620         *     88640         *     88660         *     88680         *     88700
Seq1 : tgttcagcgccgtagtaactctaataatataggctgataagctaacatcataccctcctgtatatgtgaatatggcatgatttttgtccattacaagctc :  88700
Seq2 : tgttcagcgccgtagtaactctaataatataggctgataagctaacatcataccctcctgtatatgtgaatatggcatgatttttgtccattacaagctc :  88700
Seq3 : tgttcagcgccgtagtaactctaataatataggctgataagctaacatcataccctcctgtatatgtgaatatggcatgatttttgtccattacaagctc :  88700
Seq4 : tgttcagcgccgtagtaactctaataatataggctgataagctaacatcataccctcctgtatatgtgaatatggcatgatttttgtccattacaagctc :  88700

                  *     88720         *     88740         *     88760         *     88780         *     88800
Seq1 : ggttttaactttattgcctgtaataatttctctcatctgtaggatatctatttttttgtcatgcattgccttcaagacgggacgaagaaacgtaatatcc :  88800
Seq2 : ggttttaactttattgcctgtaataatttctctcatctgtaggatatctatttttttgtcatgcattgccttcaagacgggacgaagaaacgtaatatcc :  88800
Seq3 : ggttttaactttattgcctgtaataatttctctcatctgtaggatatctatttttttgtcatgcattgccttcaagacgggacgaagaaacgtaatatcc :  88800
Seq4 : ggttttaactttattgcctgtaataatttctctcatctgtaggatatctatttttttgtcatgcattgccttcaagacgggacgaagaaacgtaatatcc :  88800

                  *     88820         *     88840         *     88860         *     88880         *     88900
Seq1 : tcaataacgttatcgttttctacaataactacatattctacctttttattttctaactcggtaaaaaaattagaatcccatagggctaaatgtctagcga :  88900
Seq2 : tcaataacgttatcgttttctacaataactacatattctacctttttattttctaactcggtaaaaaaattagaatcccatagggctaaatgtctagcga :  88900
Seq3 : tcaataacgttatcgttttctacaataactacatattctacctttttattttctaactcggtaaaaaaattagaatcccatagggctaaatgtctagcga :  88900
Seq4 : tcaataacgttatcgttttctacaataactacatattctacctttttattttctaactcggtaaaaaaattagaatcccatagggctaaatgtctagcga :  88900
```

```
                 *     88920         *     88940         *     88960         *     88980         *     89000
Seq1 : tatttcttttcgtttcctctgtacacatagtgttacaaaaccctgaaaagaagtgagtatacttgtcatcatttctaatgtttcctccagtccactgtat :  89000
Seq2 : tatttcttttcgtttcctctgtacacatagtgttacaaaaccctgaaaagaagtgagtatacttgtcatcatttctaatgtttcctccagtccactgtat :  89000
Seq3 : tatttcttttcgtttcctctgtacacatagtgttacaaaaccctgaaaagaagtgagtatacttgtcatcatttctaatgtttcctccagtccactgtat :  89000
Seq4 : tatttcttttcgtttcctctgtacacatagtgttacaaaaccctgaaaagaagtgagtatacttgtcatcatttctaatgtttcctccagtccactgtat :  89000

                 *     89020         *     89040         *     89060         *     89080         *     89100
Seq1 : aaacgcataatccttgtaatgatctggatcatccttgactaccacaacatttcttttttctggcataacttcgttgtcctttacatcatcgaacttctga :  89100
Seq2 : aaacgcataatccttgtaatgatctggatcatccttgactaccacaacatttcttttttctggcataacttcgttgtcctttacatcatcgaacttctga :  89100
Seq3 : aaacgcataatccttgtaatgatctggatcatccttgactaccacaacatttcttttttctggcataacttcgttgtcctttacatcatcgaacttctga :  89100
Seq4 : aaacgcataatccttgtaatgatctggatcatccttgactaccacaacatttcttttttctggcataacttcgttgtcctttacatcatcgaacttctga :  89100

                 *     89120         *     89140         *     89160         *     89180         *     89200
Seq1 : tcattaatatgctcatgaacattaggaaatgtttctgatggaagtctatcaataactggcacaacaataacaggagttttcgccgccgccatttagttat :  89200
Seq2 : tcattaatatgctcatgaacattaggaaatgtttctgatggaagtctatcaataactggcacaacaataacaggagttttcgccgccgccatttagttat :  89200
Seq3 : tcattaatatgctcatgaacattaggaaatgtttctgatggaagtctatcaataactggcacaacaataacaggagttttcgccgccgccatttagttat :  89200
Seq4 : tcattaatatgctcatgaacattaggaaatgtttctgatggaagtctatcaataactggcacaacaataacaggagttttcgccgccgccatttagttat :  89200

                 *     89220         *     89240         *     89260         *     89280         *     89300
Seq1 : tgaaattaatcatatacaactctttaatacgagttatatttcgtctatccattgtttcacatttacatatttcgacaaaaagatataaaatgcgtattc :  89300
Seq2 : tgaaattaatcatatacaactctttaatacgagttatatttcgtctatccattgtttcacatttacatatttcgacaaaaagatataaaatgcgtattc :  89300
Seq3 : tgaaattaatcatatacaactctttaatacgagttatatttcgtctatccattgtttcacatttacatatttcgacaaaaagatataaaatgcgtattc :  89300
Seq4 : tgaaattaatcatatacaactctttaatacgagttatatttcgtctatccattgtttcacatttacatatttcgacaaaaagatataaaatgcgtattc :  89300

                 *     89320         *     89340         *     89360         *     89380         *     89400
Seq1 : caatgcttctctgtttaatgaattactaaaatatacaaacacgtcactgtctggcaataaatgatatcttagaatattgtaacaatttattttgtattgc :  89400
Seq2 : caatgcttctctgtttaatgaattactaaaatatacaaacacgtcactgtctggcaataaatgatatcttagaatattgtaacaatttattttgtattgc :  89400
Seq3 : caatgcttctctgtttaatgaattactaaaatatacaaacacgtcactgtctggcaataaatgatatcttagaatattgtaacaatttattttgtattgc :  89400
Seq4 : caatgcttctctgtttaatgaattactaaaatatacaaacacgtcactgtctggcaataaatgatatcttagaatattgtaacaatttattttgtattgc :  89400

                 *     89420         *     89440         *     89460         *     89480         *     89500
Seq1 : acatgttcgtgatctatgagttcttcttcgaatggcataggatctccgaatctgaaaacgtataaataggagttagaataataatatttgagagtattgg :  89500
Seq2 : acatgttcgtgatctatgagttcttcttcgaatggcataggatctccgaatctgaaaacgtataaataggagttagaataataatatttgagagtattgg :  89500
Seq3 : acatgttcgtgatctatgagttcttcttcgaatggcataggatctccgaatctgaaaacgtataaataggagttagaataataatatttgagagtattgg :  89500
Seq4 : acatgttcgtgatctatgagttcttcttcgaatggcataggatctccgaatctgaaaacgtataaataggagttagaataataatatttgagagtattgg :  89500

                 *     89520         *     89540         *     89560         *     89580         *     89600
Seq1 : taatatataaactctttagcggtataattagtttttttctctcaatttctattttagatgtgatggaaaaatgactaattttgtagcattagtatcatg :  89600
Seq2 : taatatataaactctttagcggtataattagtttttttctctcaatttctattttagatgtgatggaaaaatgactaattttgtagcattagtatcatg :  89600
Seq3 : taatatataaactctttagcggtataattagtttttttctctcaatttctattttagatgtgatggaaaaatgactaattttgtagcattagtatcatg :  89600
Seq4 : taatatataaactctttagcggtataattagtttttttctctcaatttctattttagatgtgatggaaaaatgactaattttgtagcattagtatcatg :  89600
```

```
                 *     89620         *     89640         *     89660         *     89680         *     89700
Seq1 : aactctaatcaaaatcttaatatcttcgtcacacgttagctctttgaagtttttaagagatgcatcagttggttcgaccgatggagtaggtgcaacaatt :  89700
Seq2 : aactctaatcaaaatcttaatatcttcgtcacacgttagctctttgaagtttttaagagatgcatcagttggttcgaccgatggagtaggtgcaacaatt :  89700
Seq3 : aactctaatcaaaatcttaatatcttcgtcacacgttagctctttgaagtttttaagagatgcatcagttggttcgaccgatggagtaggtgcaacaatt :  89700
Seq4 : aactctaatcaaaatcttaatatcttcgtcacacgttagctctttgaagtttttaagagatgcatcagttggttcgaccgatggagtaggtgcaacaatt :  89700

                 *     89720         *     89740         *     89760         *     89780         *     89800
Seq1 : ttttgttcgatgtatgtatgtactggagccattgttttaactataatggtgcttgtatcgaaaaactttaatgcagatagcggaagctcttcgccgcgac :  89800
Seq2 : ttttgttcgatgtatgtatgtactggagccattgttttaactataatggtgcttgtatcgaaaaactttaatgcagatagcggaagctcttcgccgcgac :  89800
Seq3 : ttttgttcgatgtatgtatgtactggagccattgttttaactataatggtgcttgtatcgaaaaactttaatgcagatagcggaagctcttcgccgcgac :  89800
Seq4 : ttttgttcgatgtatgtatgtactggagccattgttttaactataatggtgcttgtatcgaaaaactttaatgcagatagcggaagctcttcgccgcgac :  89800

                 *     89820         *     89840         *     89860         *     89880         *     89900
Seq1 : tttctacatcgtaattgggttctaacgccgatctctgaatggatactagttttctaagttctaatgtgattctctgaaaatgtaaatccaattcctccgg :  89900
Seq2 : tttctacatcgtaattgggttctaacgccgatctctgaatggatactagttttctaagttctaatgtgattctctgaaaatgtaaatccaattcctccgg :  89900
Seq3 : tttctacatcgtaattgggttctaacgccgatctctgaatggatactagttttctaagttctaatgtgattctctgaaaatgtaaatccaattcctccgg :  89900
Seq4 : tttctacatcgtaattgggttctaacgccgatctctgaatggatactagttttctaagttctaatgtgattctctgaaaatgtaaatccaattcctccgg :  89900

                 *     89920         *     89940         *     89960         *     89980         *     90000
Seq1 : cattatagatgtgtatacatcggtaaataaaactatagtatccaacgatcccttctcgcaaattctagtcttaaccaaaaaatcgtatataaccacggag :  90000
Seq2 : cattatagatgtgtatacatcggtaaataaaactatagtatccaacgatcccttctcgcaaattctagtcttaaccaaaaaatcgtatataaccacggag :  90000
Seq3 : cattatagatgtgtatacatcggtaaataaaactatagtatccaacgatcccttctcgcaaattctagtcttaaccaaaaaatcgtatataaccacggag :  90000
Seq4 : cattatagatgtgtatacatcggtaaataaaactatagtatccaacgatcccttctcgcaaattctagtcttaaccaaaaaatcgtatataaccacggag :  90000

                 *     90020         *     90040         *     90060         *     90080         *     90100
Seq1 : atggcgtatttaagagtggattcttctaccgttttgttcttggatgtcatataggaaactataaagtccgcactactgttaagaatgattactaacgcaa :  90100
Seq2 : atggcgtatttaagagtggattcttctaccgttttgttcttggatgtcatataggaaactataaagtccgcactactgttaagaatgattactaacgcaa :  90100
Seq3 : atggcgtatttaagagtggattcttctaccgttttgttcttggatgtcatataggaaactataaagtccgcactactgttaagaatgattactaacgcaa :  90100
Seq4 : atggcgtatttaagagtggattcttctaccgttttgttcttggatgtcatataggaaactataaagtccgcactactgttaagaatgattactaacgcaa :  90100

                 *     90120         *     90140         *     90160         *     90180         *     90200
Seq1 : ctatatagttcaaattaagcattttggaaacataaaataactctgtagacgatacttgactttcgaataagtttgcagacaaacgaagaaagaacagacc :  90200
Seq2 : ctatatagttcaaattaagcattttggaaacataaaataactctgtagacgatacttgactttcgaataagtttgcagacaaacgaagaaagaacagacc :  90200
Seq3 : ctatatagttcaaattaagcattttggaaacataaaataactctgtagacgatacttgactttcgaataagtttgcagacaaacgaagaaagaacagacc :  90200
Seq4 : ctatatagttcaaattaagcattttggaaacataaaataactctgtagacgatacttgactttcgaataagtttgcagacaaacgaagaaagaacagacc :  90200

                 *     90220         *     90240         *     90260         *     90280         *     90300
Seq1 : tctcttaatttcagaagaaaactttttttcgtattcctgacgtctagagtttatatcaataagaaagttaagaattagtcggttaatgttgtatttcatt :  90300
Seq2 : tctcttaatttcagaagaaaactttttttcgtattcctgacgtctagagtttatatcaataagaaagttaagaattagtcggttaatgttgtatttcatt :  90300
Seq3 : tctcttaatttcagaagaaaactttttttcgtattcctgacgtctagagtttatatcaataagaaagttaagaattagtcggttaatgttgtatttcatt :  90300
Seq4 : tctcttaatttcagaagaaaactttttttcgtattcctgacgtctagagtttatatcaataagaaagttaagaattagtcggttaatgttgtatttcatt :  90300
```

```
                 *     90320         *     90340         *     90360         *     90380         *     90400
Seq1 : acccaagtttgagatttcataatattatcaaaagacatgataatattaaagataaagcgctgactatgaacgaaatagctatatggttcgctcaaaaata :  90400
Seq2 : acccaagtttgagatttcataatattatcaaaagacatgataatattaaagataaagcgctgactatgaacgaaatagctatatggttcgctcaaaaata :  90400
Seq3 : acccaagtttgagatttcataatattatcaaaagacatgataatattaaagataaagcgctgactatgaacgaaatagctatatggttcgctcaaaaata :  90400
Seq4 : acccaagtttgagatttcataatattatcaaaagacatgataatattaaagataaagcgctgactatgaacgaaatagctatatggttcgctcaaaaata :  90400

                 *     90420         *     90440         *     90460         *     90480         *     90500
Seq1 : tagtcttgttaaacgtggaaacgataactgtatttttaatcacgtcagcggcatctaaattaaatataggtatatttattccacacactctacaatatgc :  90500
Seq2 : tagtcttgttaaacgtggaaacgataactgtatttttaatcacgtcagcggcatctaaattaaatataggtatatttattccacacactctacaatatgc :  90500
Seq3 : tagtcttgttaaacgtggaaacgataactgtatttttaatcacgtcagcggcatctaaattaaatataggtatatttattccacacactctacaatatgc :  90500
Seq4 : tagtcttgttaaacgtggaaacgataactgtatttttaatcacgtcagcggcatctaaattaaatataggtatatttattccacacactctacaatatgc :  90500

                 *     90520         *     90540         *     90560         *     90580         *     90600
Seq1 : cacaccatcttcataataaataaattcgttagcaaaattattaattttagtgaaatagttagcgtcaactttcatagcttccttcaatctaatttgatgc :  90600
Seq2 : cacaccatcttcataataaataaattcgttagcaaaattattaattttagtgaaatagttagcgtcaactttcatagcttccttcaatctaatttgatgc :  90600
Seq3 : cacaccatcttcataataaataaattcgttagcaaaattattaattttagtgaaatagttagcgtcaactttcatagcttccttcaatctaatttgatgc :  90600
Seq4 : cacaccatcttcataataaataaattcgttagcaaaattattaattttagtgaaatagttagcgtcaactttcatagcttccttcaatctaatttgatgc :  90600

                 *     90620         *     90640         *     90660         *     90680         *     90700
Seq1 : tcacacggtgcgaattccactctaacatccctttttccatgcctcaggttcatcgatctctataatatctagtttttttgcgtttcacaaacacaggctcgt :  90700
Seq2 : tcacacggtgcgaattccactctaacatccctttttccatgcctcaggttcatcgatctctataatatctagtttttttgcgtttcacaaacacaggctcgt :  90700
Seq3 : tcacacggtgcgaattccactctaacatccctttttccatgcctcaggttcatcgatctctataatatctagtttttttgcgtttcacaaacacaggctcgt :  90700
Seq4 : tcacacggtgcgaattccactctaacatccctttttccatgcctcaggttcatcgatctctataatatctagtttttttgcgtttcacaaacacaggctcgt :  90700

                 *     90720         *     90740         *     90760         *     90780         *     90800
Seq1 : ctctcgcgatgagatctgtatagtaactatgtaaatgataactagatagaaagatgtagctatatagatgacgatcctttaagagaggtataataacttt :  90800
Seq2 : ctctcgcgatgagatctgtatagtaactatgtaaatgataactagatagaaagatgtagctatatagatgacgatcctttaagagaggtataataacttt :  90800
Seq3 : ctctcgcgatgagatctgtatagtaactatgtaaatgataactagatagaaagatgtagctatatagatgacgatcctttaagagaggtataataacttt :  90800
Seq4 : ctctcgcgatgagatctgtatagtaactatgtaaatgataactagatagaaagatgtagctatatagatgacgatcctttaagagaggtataataacttt :  90800

                 *     90820         *     90840         *     90860         *     90880         *     90900
Seq1 : accccaatcagatagactgttgttatggtcttcggaaaaagaatttttataaatttttccagtattttccaaatatacgtacttaacatctaaaaaatcc :  90900
Seq2 : accccaatcagatagactgttgttatggtcttcggaaaaagaatttttataaatttttccagtattttccaaatatacgtacttaacatctaaaaaatcc :  90900
Seq3 : accccaatcagatagactgttgttatggtcttcggaaaaagaatttttataaatttttccagtattttccaaatatacgtacttaacatctaaaaaatcc :  90900
Seq4 : accccaatcagatagactgttgttatggtcttcggaaaaagaatttttataaatttttccagtattttccaaatatacgtacttaacatctaaaaaatcc :  90900

                 *     90920         *     90940         *     90960         *     90980         *     91000
Seq1 : ttaatgataataggaatggataatccgtctattttataaagaaatacatatcgcacattatacttttttttggaaatgggaataccgatgtgtctacata :  91000
Seq2 : ttaatgataataggaatggataatccgtctattttataaagaaatacatatcgcacattatacttttttttggaaatgggaataccgatgtgtctacata :  91000
Seq3 : ttaatgataataggaatggataatccgtctattttataaagaaatacatatcgcacattatacttttttttggaaatgggaataccgatgtgtctacata :  91000
Seq4 : ttaatgataataggaatggataatccgtctattttataaagaaatacatatcgcacattatacttttttttggaaatgggaataccgatgtgtctacata :  91000
```

```
                    *     91020         *     91040         *     91060         *     91080         *     91100
Seq1 : aatatgcaaagtctaaatattttttagagaatcttaattggtccaaattcttttccaagtacggtaatagatttttcatattgaacggtatcttcttaat :  91100
Seq2 : aatatgcaaagtctaaatattttttagagaatcttaattggtccaaattcttttccaagtacggtaatagatttttcatattgaacggtatcttcttaat :  91100
Seq3 : aatatgcaaagtctaaatattttttagagaatcttaattggtccaaattcttttccaagtacggtaatagatttttcatattgaacggtatcttcttaat :  91100
Seq4 : aatatgcaaagtctaaatattttttagagaatcttaattggtccaaattcttttccaagtacggtaatagatttttcatattgaacggtatcttcttaat :  91100

                    *     91120         *     91140         *     91160         *     91180         *     91200
Seq1 : ctctggttctagttccgcattaaatgatgaaactaagtcactatttttataactaacgattacatcacctctaacatcatcatttaccagaatactgatc :  91200
Seq2 : ctctggttctagttccgcattaaatgatgaaactaagtcactatttttataactaacgattacatcacctctaacatcatcatttaccagaatactgatc :  91200
Seq3 : ctctggttctagttccgcattaaatgatgaaactaagtcactatttttataactaacgattacatcacctctaacatcatcatttaccagaatactgatc :  91200
Seq4 : ctctggttctagttccgcattaaatgatgaaactaagtcactatttttataactaacgattacatcacctctaacatcatcatttaccagaatactgatc :  91200

                    *     91220         *     91240         *     91260         *     91280         *     91300
Seq1 : ttcttttgtcgtaaatacatgtctaatgtgttaaaaaaaagatcatacaagttatacgtcatttcatctgtggtattcttgtcattgaaggataaactcg :  91300
Seq2 : ttcttttgtcgtaaatacatgtctaatgtgttaaaaaaaagatcatacaagttatacgtcatttcatctgtggtattcttgtcattgaaggataaactcg :  91300
Seq3 : ttcttttgtcgtaaatacatgtctaatgtgttaaaaaaaagatcatacaagttatacgtcatttcatctgtggtattcttgtcattgaaggataaactcg :  91300
Seq4 : ttcttttgtcgtaaatacatgtctaatgtgttaaaaaaaagatcatacaagttatacgtcatttcatctgtggtattcttgtcattgaaggataaactcg :  91300

                    *     91320         *     91340         *     91360         *     91380         *     91400
Seq1 : tactaatctcttctttaacagcctgttcaaatttatatcctatatacgaaaaaatagcaaccagtgtttgatcatccgcgtcaatattctgttctatcgt :  91400
Seq2 : tactaatctcttctttaacagcctgttcaaatttatatcctatatacgaaaaaatagcaaccagtgtttgatcatccgcgtcaatattctgttctatcgt :  91400
Seq3 : tactaatctcttctttaacagcctgttcaaatttatatcctatatacgaaaaaatagcaaccagtgtttgatcatccgcgtcaatattctgttctatcgt :  91400
Seq4 : tactaatctcttctttaacagcctgttcaaatttatatcctatatacgaaaaaatagcaaccagtgtttgatcatccgcgtcaatattctgttctatcgt :  91400

                    *     91420         *     91440         *     91460         *     91480         *     91500
Seq1 : agtgtataacaatcgtatatcttcttctgtgatagtcgatacgttataaaggttgataacgaaaatatttttatttcgtgaaataaagtcatcgtaggat :  91500
Seq2 : agtgtataacaatcgtatatcttcttctgtgatagtcgatacgttataaaggttgataacgaaaatatttttatttcgtgaaataaagtcatcgtaggat :  91500
Seq3 : agtgtataacaatcgtatatcttcttctgtgatagtcgatacgttataaaggttgataacgaaaatatttttatttcgtgaaataaagtcatcgtaggat :  91500
Seq4 : agtgtataacaatcgtatatcttcttctgtgatagtcgatacgttataaaggttgataacgaaaatatttttatttcgtgaaataaagtcatcgtaggat :  91500

                    *     91520         *     91540         *     91560         *     91580         *     91600
Seq1 : tttggacttatattcgcgtctagtagatatgcttttatttttggaatgatctcaattagaatagtctctttagagtccatttaaagttacaaacaactag :  91600
Seq2 : tttggacttatattcgcgtctagtagatatgcttttatttttggaatgatctcaattagaatagtctctttagagtccatttaaagttacaaacaactag :  91600
Seq3 : tttggacttatattcgcgtctagtagatatgcttttatttttggaatgatctcaattagaatagtctctttagagtccatttaaagttacaaacaactag :  91600
Seq4 : tttggacttatattcgcgtctagtagatatgcttttatttttggaatgatctcaattagaatagtctctttagagtccatttaaagttacaaacaactag :  91600

                    *     91620         *     91640         *     91660         *     91680         *     91700
Seq1 : gaaattggtttatgatgtataatttttttagtttttatagattctttattctatacttaaaaaatgaaaataaatacaaaggttcttgagggttgtgtta :  91700
Seq2 : gaaattggtttatgatgtataatttttttagtttttatagattctttattctatacttaaaaaatgaaaataaatacaaaggttcttgagggttgtgtta :  91700
Seq3 : gaaattggtttatgatgtataatttttttagtttttatagattctttattctatacttaaaaaatgaaaataaatacaaaggttcttgagggttgtgtta :  91700
Seq4 : gaaattggtttatgatgtataatttttttagtttttatagattctttattctatacttaaaaaatgaaaataaatacaaaggttcttgagggttgtgtta :  91700
```

```
          *     91720          *     91740          *     91760          *     91780          *     91800
Seq1 : aattgaaagcgagaaataatcataaattatttcattatcgcgatatccgttaagtttgtatcgtaatggcgtggtcaattacaaataaagcggatactag :  91800
Seq2 : aattgaaagcgagaaataatcataaattatttcattatcgcgatatccgttaagtttgtatcgtaatggcgtggtcaattacaaataaagcggatactag :  91800
Seq3 : aattgaaagcgagaaataatcataaattatttcattatcgcgatatccgttaagtttgtatcgtaatggcgtggtcaattacaaataaagcggatactag :  91800
Seq4 : aattgaaagcgagaaataatcataaattatttcattatcgcgatatccgttaagtttgtatcgtaatggcgtggtcaattacaaataaagcggatactag :  91800

          *     91820          *     91840          *     91860          *     91880          *     91900
Seq1 : tagcttcacaaagatggctgaaatcagagctcatctaaaaaatagcgctgaaaataaagataaaaacgaggatattttcccggaagatgtaataattcca :  91900
Seq2 : tagcttcacaaagatggctgaaatcagagctcatctaaaaaatagcgctgaaaataaagataaaaacgaggatattttcccggaagatgtaataattcca :  91900
Seq3 : tagcttcacaaagatggctgaaatcagagctcatctaaaaaatagcgctgaaaataaagataaaaacgaggatattttcccggaagatgtaataattcca :  91900
Seq4 : tagcttcacaaagatggctgaaatcagagctcatctaaaaaatagcgctgaaaataaagataaaaacgaggatattttcccggaagatgtaataattcca :  91900

          *     91920          *     91940          *     91960          *     91980          *     92000
Seq1 : tctactaagcccaaaaccaaacgagccactactcctcgtaaaccagcggctactaaaagatcaaccaaaaaggaggaagtggaagaagaagtagttatag :  92000
Seq2 : tctactaagcccaaaaccaaacgagccactactcctcgtaaaccagcggctactaaaagatcaaccaaaaaggaggaagtggaagaagaagtagttatag :  92000
Seq3 : tctactaagcccaaaaccaaacgagccactactcctcgtaaaccagcggctactaaaagatcaaccaaaaaggaggaagtggaagaagaagtagttatag :  92000
Seq4 : tctactaagcccaaaaccaaacgagccactactcctcgtaaaccagcggctactaaaagatcaaccaaaaaggaggaagtggaagaagaagtagttatag :  92000

          *     92020          *     92040          *     92060          *     92080          *     92100
Seq1 : aggaatatcatcaaacaactgaaaaaaattctccatctcctggagtcagcgacattgtagaaagcgtggccgctgtagagctcgatgatagcgacgggga :  92100
Seq2 : aggaatatcatcaaacaactgaaaaaaattctccatctcctggagtcagcgacattgtagaaagcgtggccgctgtagagctcgatgatagcgacgggga :  92100
Seq3 : aggaatatcatcaaacaactgaaaaaaattctccatctcctggagtcagcgacattgtagaaagcgtggccgctgtagagctcgatgatagcgacgggga :  92100
Seq4 : aggaatatcatcaaacaactgaaaaaaattctccatctcctggagtcagcgacattgtagaaagcgtggccgctgtagagctcgatgatagcgacgggga :  92100

          *     92120          *     92140          *     92160          *     92180          *     92200
Seq1 : tgatgaacctatggtacaagttgaagctggtaaagtaaatcatagtgctagaagcgatctctctgacctaaaggtggctaccgacaatatcgttaaagat :  92200
Seq2 : tgatgaacctatggtacaagttgaagctggtaaagtaaatcatagtgctagaagcgatctctctgacctaaaggtggctaccgacaatatcgttaaagat :  92200
Seq3 : tgatgaacctatggtacaagttgaagctggtaaagtaaatcatagtgctagaagcgatctctctgacctaaaggtggctaccgacaatatcgttaaagat :  92200
Seq4 : tgatgaacctatggtacaagttgaagctggtaaagtaaatcatagtgctagaagcgatctctctgacctaaaggtggctaccgacaatatcgttaaagat :  92200

          *     92220          *     92240          *     92260          *     92280          *     92300
Seq1 : cttaagaaaattattactagaatctctgcagtatcgacggttctagaggatgttcaagcagctggtatctctagacaatttacttctatgactaaagcta :  92300
Seq2 : cttaagaaaattattactagaatctctgcagtatcgacggttctagaggatgttcaagcagctggtatctctagacaatttacttctatgactaaagcta :  92300
Seq3 : cttaagaaaattattactagaatctctgcagtatcgacggttctagaggatgttcaagcagctggtatctctagacaatttacttctatgactaaagcta :  92300
Seq4 : cttaagaaaattattactagaatctctgcagtatcgacggttctagaggatgttcaagcagctggtatctctagacaatttacttctatgactaaagcta :  92300

          *     92320          *     92340          *     92360          *     92380          *     92400
Seq1 : ttacaacactatctgatctagtcaccgagggaaaatctaaagttgttcgtaaaaaagttaaaacttgtaagaagtaaatgcgtgcactttttttataaaga :  92400
Seq2 : ttacaacactatctgatctagtcaccgagggaaaatctaaagttgttcgtaaaaaagttaaaacttgtaagaagtaaatgcgtgcactttttttataaaga :  92400
Seq3 : ttacaacactatctgatctagtcaccgagggaaaatctaaagttgttcgtaaaaaagttaaaacttgtaagaagtaaatgcgtgcactttttttataaaga :  92400
Seq4 : ttacaacactatctgatctagtcaccgagggaaaatctaaagttgttcgtaaaaaagttaaaacttgtaagaagtaaatgcgtgcactttttttataaaga :  92400
```

```
                *     92420         *     92440         *     92460         *     92480         *     92500
Seq1 : tggtaaactctttaccgataataattttttaaatcctgtatcagacgataatccagcgtatgaggttttgcaacatgttaaaattcctactcatttaaca :  92500
Seq2 : tggtaaactctttaccgataataattttttaaatcctgtatcagacgataatccagcgtatgaggttttgcaacatgttaaaattcctactcatttaaca :  92500
Seq3 : tggtaaactctttaccgataataattttttaaatcctgtatcagacgataatccagcgtatgaggttttgcaacatgttaaaattcctactcatttaaca :  92500
Seq4 : tggtaaactctttaccgataataattttttaaatcctgtatcagacgataatccagcgtatgaggttttgcaacatgttaaaattcctactcatttaaca :  92500

                *     92520         *     92540         *     92560         *     92580         *     92600
Seq1 : gatgtagtagtatatgaacaaacgtgggaggaggcgttaactagattaattttttgtgggaagtgattcaaaaggacgtagacaatacttttacggaaaaa :  92600
Seq2 : gatgtagtagtatatgaacaaacgtgggaggaggcgttaactagattaattttttgtgggaagtgattcaaaaggacgtagacaatacttttacggaaaaa :  92600
Seq3 : gatgtagtagtatatgaacaaacgtgggaggaggcgttaactagattaattttttgtgggaagtgattcaaaaggacgtagacaatacttttacggaaaaa :  92600
Seq4 : gatgtagtagtatatgaacaaacgtgggaggaggcgttaactagattaattttttgtgggaagtgattcaaaaggacgtagacaatacttttacggaaaaa :  92600

                *     92620         *     92640         *     92660         *     92680         *     92700
Seq1 : tgcatgtacagaatcgcaacgctaaaagagatcgtatttttgttagagtatataacgttatgaaacgaattaattgttttataaacaaaaatataaagaa :  92700
Seq2 : tgcatgtacagaatcgcaacgctaaaagagatcgtatttttgttagagtatataacgttatgaaacgaattaattgttttataaacaaaaatataaagaa :  92700
Seq3 : tgcatgtacagaatcgcaacgctaaaagagatcgtatttttgttagagtatataacgttatgaaacgaattaattgttttataaacaaaaatataaagaa :  92700
Seq4 : tgcatgtacagaatcgcaacgctaaaagagatcgtatttttgttagagtatataacgttatgaaacgaattaattgttttataaacaaaaatataaagaa :  92700

                *     92720         *     92740         *     92760         *     92780         *     92800
Seq1 : atcgtccacagattccaattatcagttggcggttttatgttaatggaaactatgtttttttattagatttggtaaaatgaaatatcttaaggagaatgaa :  92800
Seq2 : atcgtccacagattccaattatcagttggcggttttatgttaatggaaactatgtttttttattagatttggtaaaatgaaatatcttaaggagaatgaa :  92800
Seq3 : atcgtccacagattccaattatcagttggcggttttatgttaatggaaactatgtttttttattagatttggtaaaatgaaatatcttaaggagaatgaa :  92800
Seq4 : atcgtccacagattccaattatcagttggcggttttatgttaatggaaactatgtttttttattagatttggtaaaatgaaatatcttaaggagaatgaa :  92800

                *     92820         *     92840         *     92860         *     92880         *     92900
Seq1 : acagtagggttattaacactaaaaaataaacacatagaaataagtcccgatgaaatagttatcaagtttgtaggaaaggacaaagtttcacatgaatttg :  92900
Seq2 : acagtagggttattaacactaaaaaataaacacatagaaataagtcccgatgaaatagttatcaagtttgtaggaaaggacaaagtttcacatgaatttg :  92900
Seq3 : acagtagggttattaacactaaaaaataaacacatagaaataagtcccgatgaaatagttatcaagtttgtaggaaaggacaaagtttcacatgaatttg :  92900
Seq4 : acagtagggttattaacactaaaaaataaacacatagaaataagtcccgatgaaatagttatcaagtttgtaggaaaggacaaagtttcacatgaatttg :  92900

                *     92920         *     92940         *     92960         *     92980         *     93000
Seq1 : ttgttcataagtctaatagactatataagccgctattgaaactgacggatgattctagtcccgaagaatttctgttcaacaaactaagtgaacgaaaggt :  93000
Seq2 : ttgttcataagtctaatagactatataagccgctattgaaactgacggatgattctagtcccgaagaatttctgttcaacaaactaagtgaacgaaaggt :  93000
Seq3 : ttgttcataagtctaatagactatataagccgctattgaaactgacggatgattctagtcccgaagaatttctgttcaacaaactaagtgaacgaaaggt :  93000
Seq4 : ttgttcataagtctaatagactatataagccgctattgaaactgacggatgattctagtcccgaagaatttctgttcaacaaactaagtgaacgaaaggt :  93000

                *     93020         *     93040         *     93060         *     93080         *     93100
Seq1 : atatgaatgtatcaaacagtttggtattagaatcaaggatctccgaacgtatggagtcaattatacgttttatataatttttggacaaatgtaaagtcc :  93100
Seq2 : atatgaatgtatcaaacagtttggtattagaatcaaggatctccgaacgtatggagtcaattatacgttttatataatttttggacaaatgtaaagtcc :  93100
Seq3 : atatgaatgtatcaaacagtttggtattagaatcaaggatctccgaacgtatggagtcaattatacgttttatataatttttggacaaatgtaaagtcc :  93100
Seq4 : atatgaatgtatcaaacagtttggtattagaatcaaggatctccgaacgtatggagtcaattatacgttttatataatttttggacaaatgtaaagtcc :  93100
```

```
          *     93120         *     93140         *     93160         *     93180         *     93200
Seq1 : atatctcctcttccatcaccaaaaaagttaatagcgttaactatcaaacaaactgctgaagtggtaggtcatactccatcaatttcaaaaagagcttata :  93200
Seq2 : atatctcctcttccatcaccaaaaaagttaatagcgttaactatcaaacaaactgctgaagtggtaggtcatactccatcaatttcaaaaagagcttata :  93200
Seq3 : atatctcctcttccatcaccaaaaaagttaatagcgttaactatcaaacaaactgctgaagtggtaggtcatactccatcaatttcaaaaagagcttata :  93200
Seq4 : atatctcctcttccatcaccaaaaaagttaatagcgttaactatcaaacaaactgctgaagtggtaggtcatactccatcaatttcaaaaagagcttata :  93200

          *     93220         *     93240         *     93260         *     93280         *     93300
Seq1 : tggcaacgactattttagaaatggtaaaggataaaaatttttttagatgtagtatctaaaactacgttcgatgaattcctatctatagtcgtagatcacgt :  93300
Seq2 : tggcaacgactattttagaaatggtaaaggataaaaatttttttagatgtagtatctaaaactacgttcgatgaattcctatctatagtcgtagatcacgt :  93300
Seq3 : tggcaacgactattttagaaatggtaaaggataaaaatttttttagatgtagtatctaaaactacgttcgatgaattcctatctatagtcgtagatcacgt :  93300
Seq4 : tggcaacgactattttagaaatggtaaaggataaaaatttttttagatgtagtatctaaaactacgttcgatgaattcctatctatagtcgtagatcacgt :  93300

          *     93320         *     93340         *     93360         *     93380         *     93400
Seq1 : taaatcatctacggatggatgatatagatctttacacaaataattacaaaaccgataaatggaaatggataagcgtatgaaatctctcgcaatgaccgct :  93400
Seq2 : taaatcatctacggatggatgatatagatctttacacaaataattacaaaaccgataaatggaaatggataagcgtatgaaatctctcgcaatgaccgct :  93400
Seq3 : taaatcatctacggatggatgatatagatctttacacaaataattacaaaaccgataaatggaaatggataagcgtatgaaatctctcgcaatgaccgct :  93400
Seq4 : taaatcatctacggatggatgatatagatctttacacaaataattacaaaaccgataaatggaaatggataagcgtatgaaatctctcgcaatgaccgct :  93400

          *     93420         *     93440         *     93460         *     93480         *     93500
Seq1 : ttctttggggagctaagcacattagatattatggcattgataatgtctatatttaaacgccatccaaacaataccatttttttcagtggataaggatggtc :  93500
Seq2 : ttctttggggagctaagcacattagatattatggcattgataatgtctatatttaaacgccatccaaacaataccatttttttcagtggataaggatggtc :  93500
Seq3 : ttctttggggagctaagcacattagatattatggcattgataatgtctatatttaaacgccatccaaacaataccatttttttcagtggataaggatggtc :  93500
Seq4 : ttctttggggagctaagcacattagatattatggcattgataatgtctatatttaaacgccatccaaacaataccatttttttcagtggataaggatggtc :  93500

          *     93520         *     93540         *     93560         *     93580         *     93600
Seq1 : agtttatgattgatttcgaatacgataattataaggcttctcaatatttggatctgaccctcactccgatatctggagatgaatgcaagactcacgcatc :  93600
Seq2 : agtttatgattgatttcgaatacgataattataaggcttctcaatatttggatctgaccctcactccgatatctggagatgaatgcaagactcacgcatc :  93600
Seq3 : agtttatgattgatttcgaatacgataattataaggcttctcaatatttggatctgaccctcactccgatatctggagatgaatgcaagactcacgcatc :  93600
Seq4 : agtttatgattgatttcgaatacgataattataaggcttctcaatatttggatctgaccctcactccgatatctggagatgaatgcaagactcacgcatc :  93600

          *     93620         *     93640         *     93660         *     93680         *     93700
Seq1 : gagtatagccgaacaattggcgtgtgtggatattattaaagaggatattagcgaatatatcaaaactactccccgtcttaaacgatttataaaaaaatac :  93700
Seq2 : gagtatagccgaacaattggcgtgtgtggatattattaaagaggatattagcgaatatatcaaaactactccccgtcttaaacgatttataaaaaaatac :  93700
Seq3 : gagtatagccgaacaattggcgtgtgtggatattattaaagaggatattagcgaatatatcaaaactactccccgtcttaaacgatttataaaaaaatac :  93700
Seq4 : gagtatagccgaacaattggcgtgtgtggatattattaaagaggatattagcgaatatatcaaaactactccccgtcttaaacgatttataaaaaaatac :  93700

          *     93720         *     93740         *     93760         *     93780         *     93800
Seq1 : cgcaatagatcagatactcgcatcagtcgagatacagaaaagcttaaaatagctctagctaaaggcatagattacgaatatataaaagacgcttgttaat :  93800
Seq2 : cgcaatagatcagatactcgcatcagtcgagatacagaaaagcttaaaatagctctagctaaaggcatagattacgaatatataaaagacgcttgttaat :  93800
Seq3 : cgcaatagatcagatactcgcatcagtcgagatacagaaaagcttaaaatagctctagctaaaggcatagattacgaatatataaaagacgcttgttaat :  93800
Seq4 : cgcaatagatcagatactcgcatcagtcgagatacagaaaagcttaaaatagctctagctaaaggcatagattacgaatatataaaagacgcttgttaat :  93800
```

```
                 *     93820         *     93840         *     93860         *     93880         *     93900
Seq1 : aagtaaatgaaaaaaaactagtcgtttataataaaacacgatatggatgccaacgtagtatcatcttctactattgcgacgtatatagacgctttagcga :  93900
Seq2 : aagtaaatgaaaaaaaactagtcgtttataataaaacacgatatggatgccaacgtagtatcatcttctactattgcgacgtatatagacgctttagcga :  93900
Seq3 : aagtaaatgaaaaaaaactagtcgtttataataaaacacgatatggatgccaacgtagtatcatcttctactattgcgacgtatatagacgctttagcga :  93900
Seq4 : aagtaaatgaaaaaaaactagtcgtttataataaaacacgatatggatgccaacgtagtatcatcttctactattgcgacgtatatagacgctttagcga :  93900

                 *     93920         *     93940         *     93960         *     93980         *     94000
Seq1 : agaatgcttcggaattagaacagaggtctaccgcatacgaaataaataatgaattggaactagtatttattaagccgccattgattactttgacaaatgt :  94000
Seq2 : agaatgcttcggaattagaacagaggtctaccgcatacgaaataaataatgaattggaactagtatttattaagccgccattgattactttgacaaatgt :  94000
Seq3 : agaatgcttcggaattagaacagaggtctaccgcatacgaaataaataatgaattggaactagtatttattaagccgccattgattactttgacaaatgt :  94000
Seq4 : agaatgcttcggaattagaacagaggtctaccgcatacgaaataaataatgaattggaactagtatttattaagccgccattgattactttgacaaatgt :  94000

                 *     94020         *     94040         *     94060         *     94080         *     94100
Seq1 : agtgaatatctctacgattcaggaatcgtttattcgatttaccgttactaataaggaaggtgttaaaattagaactaagattccattatctaaggtacat :  94100
Seq2 : agtgaatatctctacgattcaggaatcgtttattcgatttaccgttactaataaggaaggtgttaaaattagaactaagattccattatctaaggtacat :  94100
Seq3 : agtgaatatctctacgattcaggaatcgtttattcgatttaccgttactaataaggaaggtgttaaaattagaactaagattccattatctaaggtacat :  94100
Seq4 : agtgaatatctctacgattcaggaatcgtttattcgatttaccgttactaataaggaaggtgttaaaattagaactaagattccattatctaaggtacat :  94100

                 *     94120         *     94140         *     94160         *     94180         *     94200
Seq1 : ggtctagatgtaaaaaatgtacagttagtagatgctatagataacatagtttgggaaaagaaatcattagtgacggaaaatcgtcttcacaaagaatgct :  94200
Seq2 : ggtctagatgtaaaaaatgtacagttagtagatgctatagataacatagtttgggaaaagaaatcattagtgacggaaaatcgtcttcacaaagaatgct :  94200
Seq3 : ggtctagatgtaaaaaatgtacagttagtagatgctatagataacatagtttgggaaaagaaatcattagtgacggaaaatcgtcttcacaaagaatgct :  94200
Seq4 : ggtctagatgtaaaaaatgtacagttagtagatgctatagataacatagtttgggaaaagaaatcattagtgacggaaaatcgtcttcacaaagaatgct :  94200

                 *     94220         *     94240         *     94260         *     94280         *     94300
Seq1 : tgttgagactatcgacagaggaacgtcatatattttggattacaagaaatatggatcctctatccgactagaattagtcaatcttattcaagcaaaaaac :  94300
Seq2 : tgttgagactatcgacagaggaacgtcatatattttggattacaagaaatatggatcctctatccgactagaattagtcaatcttattcaagcaaaaaac :  94300
Seq3 : tgttgagactatcgacagaggaacgtcatatattttggattacaagaaatatggatcctctatccgactagaattagtcaatcttattcaagcaaaaaac :  94300
Seq4 : tgttgagactatcgacagaggaacgtcatatattttggattacaagaaatatggatcctctatccgactagaattagtcaatcttattcaagcaaaaaac :  94300

                 *     94320         *     94340         *     94360         *     94380         *     94400
Seq1 : aaaaaactttacgatagactttaagctaaaatattttctaggatccggtgcccagtctaaaagttctttattacacgctattaatcatccaaagtcaagg :  94400
Seq2 : aaaaaactttacgatagactttaagctaaaatattttctaggatccggtgcccagtctaaaagttctttattacacgctattaatcatccaaagtcaagg :  94400
Seq3 : aaaaaactttacgatagactttaagctaaaatattttctaggatccggtgcccagtctaaaagttctttattacacgctattaatcatccaaagtcaagg :  94400
Seq4 : aaaaaactttacgatagactttaagctaaaatattttctaggatccggtgcccagtctaaaagttctttattacacgctattaatcatccaaagtcaagg :  94400

                 *     94420         *     94440         *     94460         *     94480         *     94500
Seq1 : cctaatacatctctggaaatagaatttacacctagagacaatgaaacagttccatatgatgaactaataaaggaattgacgactctctcgcgtcatatat :  94500
Seq2 : cctaatacatctctggaaatagaatttacacctagagacaatgaaacagttccatatgatgaactaataaaggaattgacgactctctcgcgtcatatat :  94500
Seq3 : cctaatacatctctggaaatagaatttacacctagagacaatgaaacagttccatatgatgaactaataaaggaattgacgactctctcgcgtcatatat :  94500
Seq4 : cctaatacatctctggaaatagaatttacacctagagacaatgaaacagttccatatgatgaactaataaaggaattgacgactctctcgcgtcatatat :  94500
```

```
                 *     94520         *     94540         *     94560         *     94580         *     94600
Seq1 : ttatggcttctccagagaatgtaattctttctccgcctattaacgcgcctataaaaacctttatgttgcctaaacaagatatagtaggtttggatctgga :  94600
Seq2 : ttatggcttctccagagaatgtaattctttctccgcctattaacgcgcctataaaaacctttatgttgcctaaacaagatatagtaggtttggatctgga :  94600
Seq3 : ttatggcttctccagagaatgtaattctttctccgcctattaacgcgcctataaaaacctttatgttgcctaaacaagatatagtaggtttggatctgga :  94600
Seq4 : ttatggcttctccagagaatgtaattctttctccgcctattaacgcgcctataaaaacctttatgttgcctaaacaagatatagtaggtttggatctgga :  94600

                 *     94620         *     94640         *     94660         *     94680         *     94700
Seq1 : aaatctatatgccgtaactaagactgacggcattcctataactatcagagttacatcaaacgggttgtattgttattttacacatcttggttatattatt :  94700
Seq2 : aaatctatatgccgtaactaagactgacggcattcctataactatcagagttacatcaaacgggttgtattgttattttacacatcttggttatattatt :  94700
Seq3 : aaatctatatgccgtaactaagactgacggcattcctataactatcagagttacatcaaacgggttgtattgttattttacacatcttggttatattatt :  94700
Seq4 : aaatctatatgccgtaactaagactgacggcattcctataactatcagagttacatcaaacgggttgtattgttattttacacatcttggttatattatt :  94700

                 *     94720         *     94740         *     94760         *     94780         *     94800
Seq1 : agatatcctgttaagagaataatagattccgaagtagtagtctttggtgaggcagttaaggataagaactggaccgtatatctcattaagctaatagagc :  94800
Seq2 : agatatcctgttaagagaataatagattccgaagtagtagtctttggtgaggcagttaaggataagaactggaccgtatatctcattaagctaatagagc :  94800
Seq3 : agatatcctgttaagagaataatagattccgaagtagtagtctttggtgaggcagttaaggataagaactggaccgtatatctcattaagctaatagagc :  94800
Seq4 : agatatcctgttaagagaataatagattccgaagtagtagtctttggtgaggcagttaaggataagaactggaccgtatatctcattaagctaatagagc :  94800

                 *     94820         *     94840         *     94860         *     94880         *     94900
Seq1 : ctgtgaatgcaatcaatgatagactagaagaaagtaagtatgttgaatctaaactagtggatatttgtgatcggatagtattcaagtcaaagaaatacga :  94900
Seq2 : ctgtgaatgcaatcaatgatagactagaagaaagtaagtatgttgaatctaaactagtggatatttgtgatcggatagtattcaagtcaaagaaatacga :  94900
Seq3 : ctgtgaatgcaatcaatgatagactagaagaaagtaagtatgttgaatctaaactagtggatatttgtgatcggatagtattcaagtcaaagaaatacga :  94900
Seq4 : ctgtgaatgcaatcaatgatagactagaagaaagtaagtatgttgaatctaaactagtggatatttgtgatcggatagtattcaagtcaaagaaatacga :  94900

                 *     94920         *     94940         *     94960         *     94980         *     95000
Seq1 : aggtccgtttactacaactagtgaagtcgtcgatatgttatctacatatttaccaaagcaaccagaaggtgttattctgttctattcaaagggacctaaa :  95000
Seq2 : aggtccgtttactacaactagtgaagtcgtcgatatgttatctacatatttaccaaagcaaccagaaggtgttattctgttctattcaaagggacctaaa :  95000
Seq3 : aggtccgtttactacaactagtgaagtcgtcgatatgttatctacatatttaccaaagcaaccagaaggtgttattctgttctattcaaagggacctaaa :  95000
Seq4 : aggtccgtttactacaactagtgaagtcgtcgatatgttatctacatatttaccaaagcaaccagaaggtgttattctgttctattcaaagggacctaaa :  95000

                 *     95020         *     95040         *     95060         *     95080         *     95100
Seq1 : tctaacattgattttaaaattaaaaaggaaaatactatagaccaaactgcaaatgtagtatttaggtacatgtccagtgaaccaattatctttggagagt :  95100
Seq2 : tctaacattgattttaaaattaaaaaggaaaatactatagaccaaactgcaaatgtagtatttaggtacatgtccagtgaaccaattatctttggagagt :  95100
Seq3 : tctaacattgattttaaaattaaaaaggaaaatactatagaccaaactgcaaatgtagtatttaggtacatgtccagtgaaccaattatctttggagagt :  95100
Seq4 : tctaacattgattttaaaattaaaaaggaaaatactatagaccaaactgcaaatgtagtatttaggtacatgtccagtgaaccaattatctttggagagt :  95100

                 *     95120         *     95140         *     95160         *     95180         *     95200
Seq1 : cgtctatctttgtagagtataagaaatttagcaacgataaaggctttcctaaagaatatggttctggtaagattgtgttatataacggcgttaattatct :  95200
Seq2 : cgtctatctttgtagagtataagaaatttagcaacgataaaggctttcctaaagaatatggttctggtaagattgtgttatataacggcgttaattatct :  95200
Seq3 : cgtctatctttgtagagtataagaaatttagcaacgataaaggctttcctaaagaatatggttctggtaagattgtgttatataacggcgttaattatct :  95200
Seq4 : cgtctatctttgtagagtataagaaatttagcaacgataaaggctttcctaaagaatatggttctggtaagattgtgttatataacggcgttaattatct :  95200
```

```
                *      95220         *      95240         *      95260         *      95280         *      95300
Seq1 : aaataatatctattgtttggaatatattaatacacataatgaagtgggtattaagtccgtggttgtacctattaagtttatagcagaattcttagttaat :  95300
Seq2 : aaataatatctattgtttggaatatattaatacacataatgaagtgggtattaagtccgtggttgtacctattaagtttatagcagaattcttagttaat :  95300
Seq3 : aaataatatctattgtttggaatatattaatacacataatgaagtgggtattaagtccgtggttgtacctattaagtttatagcagaattcttagttaat :  95300
Seq4 : aaataatatctattgtttggaatatattaatacacataatgaagtgggtattaagtccgtggttgtacctattaagtttatagcagaattcttagttaat :  95300

                *      95320         *      95340         *      95360         *      95380         *      95400
Seq1 : ggagaaatacttaaacctagaattgataaaaccatgaaatatattaactcagaagattattatggaaatcaacataatatcatagtcgaacatttaagag :  95400
Seq2 : ggagaaatacttaaacctagaattgataaaaccatgaaatatattaactcagaagattattatggaaatcaacataatatcatagtcgaacatttaagag :  95400
Seq3 : ggagaaatacttaaacctagaattgataaaaccatgaaatatattaactcagaagattattatggaaatcaacataatatcatagtcgaacatttaagag :  95400
Seq4 : ggagaaatacttaaacctagaattgataaaaccatgaaatatattaactcagaagattattatggaaatcaacataatatcatagtcgaacatttaagag :  95400

                *      95420         *      95440         *      95460         *      95480         *      95500
Seq1 : atcaaagcatcaaaataggagatatctttaacgaggataaactatcggatgtgggacatcaatacgccaataatgataaatttagattaaatccagaagt :  95500
Seq2 : atcaaagcatcaaaataggagatatctttaacgaggataaactatcggatgtgggacatcaatacgccaataatgataaatttagattaaatccagaagt :  95500
Seq3 : atcaaagcatcaaaataggagatatctttaacgaggataaactatcggatgtgggacatcaatacgccaataatgataaatttagattaaatccagaagt :  95500
Seq4 : atcaaagcatcaaaataggagatatctttaacgaggataaactatcggatgtgggacatcaatacgccaataatgataaatttagattaaatccagaagt :  95500

                *      95520         *      95540         *      95560         *      95580         *      95600
Seq1 : tagttattttacgaataaacgaactagaggaccgttgggaattttatcaaactacgtcaagactcttcttatttctatgtattgttccaaaacattttta :  95600
Seq2 : tagttattttacgaataaacgaactagaggaccgttgggaattttatcaaactacgtcaagactcttcttatttctatgtattgttccaaaacattttta :  95600
Seq3 : tagttattttacgaataaacgaactagaggaccgttgggaattttatcaaactacgtcaagactcttcttatttctatgtattgttccaaaacattttta :  95600
Seq4 : tagttattttacgaataaacgaactagaggaccgttgggaattttatcaaactacgtcaagactcttcttatttctatgtattgttccaaaacattttta :  95600

                *      95620         *      95640         *      95660         *      95680         *      95700
Seq1 : gacgattccaacaaacgaaaggtattggcgattgattttggaaacggtgctgacctggaaaaatacttttatggagagattgcgttattggtagcgacgg :  95700
Seq2 : gacgattccaacaaacgaaaggtattggcgattgattttggaaacggtgctgacctggaaaaatacttttatggagagattgcgttattggtagcgacgg :  95700
Seq3 : gacgattccaacaaacgaaaggtattggcgattgattttggaaacggtgctgacctggaaaaatacttttatggagagattgcgttattggtagcgacgg :  95700
Seq4 : gacgattccaacaaacgaaaggtattggcgattgattttggaaacggtgctgacctggaaaaatacttttatggagagattgcgttattggtagcgacgg :  95700

                *      95720         *      95740         *      95760         *      95780         *      95800
Seq1 : atccggatgctgatgctatagctagaggaaatgaaagatacaacaaattaaactctggaattaaaaccaagtactacaaatttgactacattcaggaaac :  95800
Seq2 : atccggatgctgatgctatagctagaggaaatgaaagatacaacaaattaaactctggaattaaaaccaagtactacaaatttgactacattcaggaaac :  95800
Seq3 : atccggatgctgatgctatagctagaggaaatgaaagatacaacaaattaaactctggaattaaaaccaagtactacaaatttgactacattcaggaaac :  95800
Seq4 : atccggatgctgatgctatagctagaggaaatgaaagatacaacaaattaaactctggaattaaaaccaagtactacaaatttgactacattcaggaaac :  95800

                *      95820         *      95840         *      95860         *      95880         *      95900
Seq1 : tattcgatccgatacatttgtctctagtgtcagagaagtattctattttggaaagtttaatatcatcgactggcagtttgctatccattattcttttcat :  95900
Seq2 : tattcgatccgatacatttgtctctagtgtcagagaagtattctattttggaaagtttaatatcatcgactggcagtttgctatccattattcttttcat :  95900
Seq3 : tattcgatccgatacatttgtctctagtgtcagagaagtattctattttggaaagtttaatatcatcgactggcagtttgctatccattattcttttcat :  95900
Seq4 : tattcgatccgatacatttgtctctagtgtcagagaagtattctattttggaaagtttaatatcatcgactggcagtttgctatccattattcttttcat :  95900
```

```
             *     95920         *     95940         *     95960         *     95980         *     96000
Seq1 : ccgagacattatgctaccgtcatgaataacttatccgaactaactgcttctggaggcaaggtattaatcactaccatggacggagacaaattatcaaaat :  96000
Seq2 : ccgagacattatgctaccgtcatgaataacttatccgaactaactgcttctggaggcaaggtattaatcactaccatggacggagacaaattatcaaaat :  96000
Seq3 : ccgagacattatgctaccgtcatgaataacttatccgaactaactgcttctggaggcaaggtattaatcactaccatggacggagacaaattatcaaaat :  96000
Seq4 : ccgagacattatgctaccgtcatgaataacttatccgaactaactgcttctggaggcaaggtattaatcactaccatggacggagacaaattatcaaaat :  96000

             *     96020         *     96040         *     96060         *     96080         *     96100
Seq1 : taacagataaaaagacttttataattcataagaatttacctagtagcgaaaactatatgtctgtagaaaaaatagctgatgatagaatagtggtatataa :  96100
Seq2 : taacagataaaaagacttttataattcataagaatttacctagtagcgaaaactatatgtctgtagaaaaaatagctgatgatagaatagtggtatataa :  96100
Seq3 : taacagataaaaagacttttataattcataagaatttacctagtagcgaaaactatatgtctgtagaaaaaatagctgatgatagaatagtggtatataa :  96100
Seq4 : taacagataaaaagacttttataattcataagaatttacctagtagcgaaaactatatgtctgtagaaaaaatagctgatgatagaatagtggtatataa :  96100

             *     96120         *     96140         *     96160         *     96180         *     96200
Seq1 : tccatcaacaatgtctactccaatgactgaatacattatcaaaaagaacgatatagtcagagtgtttaacgaatacggatttgttcttgtagataacgtt :  96200
Seq2 : tccatcaacaatgtctactccaatgactgaatacattatcaaaaagaacgatatagtcagagtgtttaacgaatacggatttgttcttgtagataacgtt :  96200
Seq3 : tccatcaacaatgtctactccaatgactgaatacattatcaaaaagaacgatatagtcagagtgtttaacgaatacggatttgttcttgtagataacgtt :  96200
Seq4 : tccatcaacaatgtctactccaatgactgaatacattatcaaaaagaacgatatagtcagagtgtttaacgaatacggatttgttcttgtagataacgtt :  96200

             *     96220         *     96240         *     96260         *     96280         *     96300
Seq1 : gatttcgctacaattatagaacgaagtaaaaagtttattaatggcgcatctacaatggaagatagaccatctacaagaaactttttcgaactaaatagag :  96300
Seq2 : gatttcgctacaattatagaacgaagtaaaaagtttattaatggcgcatctacaatggaagatagaccatctacaagaaactttttcgaactaaatagag :  96300
Seq3 : gatttcgctacaattatagaacgaagtaaaaagtttattaatggcgcatctacaatggaagatagaccatctacaagaaactttttcgaactaaatagag :  96300
Seq4 : gatttcgctacaattatagaacgaagtaaaaagtttattaatggcgcatctacaatggaagatagaccatctacaagaaactttttcgaactaaatagag :  96300

             *     96320         *     96340         *     96360         *     96380         *     96400
Seq1 : gagccattaaatgtgaaggtttagatgtcgaagacttacttagttactatgttgtttatgtcttttctaagcggtaaataataatatggtatgggttctg :  96400
Seq2 : gagccattaaatgtgaaggtttagatgtcgaagacttacttagttactatgttgtttatgtcttttctaagcggtaaataataatatggtatgggttctg :  96400
Seq3 : gagccattaaatgtgaaggtttagatgtcgaagacttacttagttactatgttgtttatgtcttttctaagcggtaaataataatatggtatgggttctg :  96400
Seq4 : gagccattaaatgtgaaggtttagatgtcgaagacttacttagttactatgttgtttatgtcttttctaagcggtaaataataatatggtatgggttctg :  96400

             *     96420         *     96440         *     96460         *     96480         *     96500
Seq1 : atctcccagttctaaatgcattaaataattccaatagagcgatttttgttcctataggaccttccaactgtggatactctgtattgttaatagatatatt :  96500
Seq2 : atctcccagttctaaatgcattaaataattccaatagagcgatttttgttcctataggaccttccaactgtggatactctgtattgttaatagatatatt :  96500
Seq3 : atctcccagttctaaatgcattaaataattccaatagagcgatttttgttcctataggaccttccaactgtggatactctgtattgttaatagatatatt :  96500
Seq4 : atctcccagttctaaatgcattaaataattccaatagagcgatttttgttcctataggaccttccaactgtggatactctgtattgttaatagatatatt :  96500

             *     96520         *     96540         *     96560         *     96580         *     96600
Seq1 : aatacttttgtcgggtaacagaggttctacgtcttttaaaaataaaagtttgataacatctggcctgttcataaataaaaacttggcgattctatatata :  96600
Seq2 : aatacttttgtcgggtaacagaggttctacgtcttttaaaaataaaagtttgataacatctggcctgttcataaataaaaacttggcgattctatatata :  96600
Seq3 : aatacttttgtcgggtaacagaggttctacgtcttttaaaaataaaagtttgataacatctggcctgttcataaataaaaacttggcgattctatatata :  96600
Seq4 : aatacttttgtcgggtaacagaggttctacgtcttttaaaaataaaagtttgataacatctggcctgttcataaataaaaacttggcgattctatatata :  96600
```

```
              *     96620         *     96640         *     96660         *     96680         *     96700
Seq1 : ctcttattatcaaatctagccattgtcttatagatgtgagctactgtaggtgtaccatttgattttctttctaatactatatatttctctcgaagaagtt :  96700
Seq2 : ctcttattatcaaatctagccattgtcttatagatgtgagctactgtaggtgtaccatttgattttctttctaatactatatatttctctcgaagaagtt :  96700
Seq3 : ctcttattatcaaatctagccattgtcttatagatgtgagctactgtaggtgtaccatttgattttctttctaatactatatatttctctcgaagaagtt :  96700
Seq4 : ctcttattatcaaatctagccattgtcttatagatgtgagctactgtaggtgtaccatttgattttctttctaatactatatatttctctcgaagaagtt :  96700

              *     96720         *     96740         *     96760         *     96780         *     96800
Seq1 : cttgcacatcatctgggaataaaatactactgttgagtaaatcagttattttttttatatcgatattgatggacatttttatagttaaggataataagta :  96800
Seq2 : cttgcacatcatctgggaataaaatactactgttgagtaaatcagttattttttttatatcgatattgatggacatttttatagttaaggataataagta :  96800
Seq3 : cttgcacatcatctgggaataaaatactactgttgagtaaatcagttattttttttatatcgatattgatggacatttttatagttaaggataataagta :  96800
Seq4 : cttgcacatcatctgggaataaaatactactgttgagtaaatcagttattttttttatatcgatattgatggacatttttatagttaaggataataagta :  96800

              *     96820         *     96840         *     96860         *     96880         *     96900
Seq1 : tcccaaagtagataacgacgataacgaagtatttatactttaggaaatcacaatgactttatcagattaaaattaacaaaattaaaggagcatgtattt :  96900
Seq2 : tcccaaagtagataacgacgataacgaagtatttatactttaggaaatcacaatgactttatcagattaaaattaacaaaattaaaggagcatgtattt :  96900
Seq3 : tcccaaagtagataacgacgataacgaagtatttatactttaggaaatcacaatgactttatcagattaaaattaacaaaattaaaggagcatgtattt :  96900
Seq4 : tcccaaagtagataacgacgataacgaagtatttatactttaggaaatcacaatgactttatcagattaaaattaacaaaattaaaggagcatgtattt :  96900

              *     96920         *     96940         *     96960         *     96980         *     97000
Seq1 : ttttctgaatatattgtgactccagatacatatggatctttatgcgtcgaattaaatgggtctagttttcagcacggcggtagatatatagaggtggagg :  97000
Seq2 : ttttctgaatatattgtgactccagatacatatggatctttatgcgtcgaattaaatgggtctagttttcagcacggcggtagatatatagaggtggagg :  97000
Seq3 : ttttctgaatatattgtgactccagatacatatggatctttatgcgtcgaattaaatgggtctagttttcagcacggcggtagatatatagaggtggagg :  97000
Seq4 : ttttctgaatatattgtgactccagatacatatggatctttatgcgtcgaattaaatgggtctagttttcagcacggcggtagatatatagaggtggagg :  97000

              *     97020         *     97040         *     97060         *     97080         *     97100
Seq1 : aatttatagatgctggaagacaagttagatggtgttctacatccaatcatatatctaaagatatacccgaagatatgcacactgataaatttgtcattta :  97100
Seq2 : aatttatagatgctggaagacaagttagatggtgttctacatccaatcatatatctaaagatatacccgaagatatgcacactgataaatttgtcattta :  97100
Seq3 : aatttatagatgctggaagacaagttagatggtgttctacatccaatcatatatctaaagatatacccgaagatatgcacactgataaatttgtcattta :  97100
Seq4 : aatttatagatgctggaagacaagttagatggtgttctacatccaatcatatatctaaagatatacccgaagatatgcacactgataaatttgtcattta :  97100

              *     97120         *     97140         *     97160         *     97180         *     97200
Seq1 : tgatatatacacttttgacgctttcaagaataaacgattggtattcgtacaggtacctccgtcgttaggagatgatagtcatttgactaatccgttattg :  97200
Seq2 : tgatatatacacttttgacgctttcaagaataaacgattggtattcgtacaggtacctccgtcgttaggagatgatagtcatttgactaatccgttattg :  97200
Seq3 : tgatatatacacttttgacgctttcaagaataaacgattggtattcgtacaggtacctccgtcgttaggagatgatagtcatttgactaatccgttattg :  97200
Seq4 : tgatatatacacttttgacgctttcaagaataaacgattggtattcgtacaggtacctccgtcgttaggagatgatagtcatttgactaatccgttattg :  97200

              *     97220         *     97240         *     97260         *     97280         *     97300
Seq1 : tctccgtattatcgtaattcagtagccagacaaatggtcaatgatatgattttaatcaagattcatttttaaaatatttattagaacatctgattagaa :  97300
Seq2 : tctccgtattatcgtaattcagtagccagacaaatggtcaatgatatgattttaatcaagattcatttttaaaatatttattagaacatctgattagaa :  97300
Seq3 : tctccgtattatcgtaattcagtagccagacaaatggtcaatgatatgattttaatcaagattcatttttaaaatatttattagaacatctgattagaa :  97300
Seq4 : tctccgtattatcgtaattcagtagccagacaaatggtcaatgatatgattttaatcaagattcatttttaaaatatttattagaacatctgattagaa :  97300
```

```
                  *     97320         *     97340         *     97360         *     97380         *     97400
Seq1 : gccactatagagtttctaaacatataacaatagttagatacaaggataccgaagaattaaatctaacgagaatatgttataatagagataagtttaaggc :  97400
Seq2 : gccactatagagtttctaaacatataacaatagttagatacaaggataccgaagaattaaatctaacgagaatatgttataatagagataagtttaaggc :  97400
Seq3 : gccactatagagtttctaaacatataacaatagttagatacaaggataccgaagaattaaatctaacgagaatatgttataatagagataagtttaaggc :  97400
Seq4 : gccactatagagtttctaaacatataacaatagttagatacaaggataccgaagaattaaatctaacgagaatatgttataatagagataagtttaaggc :  97400

                  *     97420         *     97440         *     97460         *     97480         *     97500
Seq1 : gtttgtattcgcttggtttaacggcgtttcggaaaatgaaaaggtactagatacgtataaaaaggtatctaatttgatataatgaattcagtgactgtat :  97500
Seq2 : gtttgtattcgcttggtttaacggcgtttcggaaaatgaaaaggtactagatacgtataaaaaggtatctaatttgatataatgaattcagtgactgtat :  97500
Seq3 : gtttgtattcgcttggtttaacggcgtttcggaaaatgaaaaggtactagatacgtataaaaaggtatctaatttgatataatgaattcagtgactgtat :  97500
Seq4 : gtttgtattcgcttggtttaacggcgtttcggaaaatgaaaaggtactagatacgtataaaaaggtatctaatttgatataatgaattcagtgactgtat :  97500

                  *     97520         *     97540         *     97560         *     97580         *     97600
Seq1 : cacacgcgccatatactattacttatcacgatgattgggaaccagtaatgagtcaattggtagagttttataacgaagtagccagttggctgctacgaga :  97600
Seq2 : cacacgcgccatatactattacttatcacgatgattgggaaccagtaatgagtcaattggtagagttttataacgaagtagccagttggctgctacgaga :  97600
Seq3 : cacacgcgccatatactattacttatcacgatgattgggaaccagtaatgagtcaattggtagagttttataacgaagtagccagttggctgctacgaga :  97600
Seq4 : cacacgcgccatatactattacttatcacgatgattgggaaccagtaatgagtcaattggtagagttttataacgaagtagccagttggctgctacgaga :  97600

                  *     97620         *     97640         *     97660         *     97680         *     97700
Seq1 : cgagacgtcgcctattcctgataagttctttatacagttgaaacaaccgcttagaaataaacgagtatgtgtgtgcggtatagatccgtatccgaaagat :  97700
Seq2 : cgagacgtcgcctattcctgataagttctttatacagttgaaacaaccgcttagaaataaacgagtatgtgtgtgcggtatagatccgtatccgaaagat :  97700
Seq3 : cgagacgtcgcctattcctgataagttctttatacagttgaaacaaccgcttagaaataaacgagtatgtgtgtgcggtatagatccgtatccgaaagat :  97700
Seq4 : cgagacgtcgcctattcctgataagttctttatacagttgaaacaaccgcttagaaataaacgagtatgtgtgtgcggtatagatccgtatccgaaagat :  97700

                  *     97720         *     97740         *     97760         *     97780         *     97800
Seq1 : ggaactggtgtaccgttcgaatcaccaaatttacaaaaaaatcaattaaggagatagcttcatctatatctagattaaccggagtaattgattataaag :  97800
Seq2 : ggaactggtgtaccgttcgaatcaccaaatttacaaaaaaatcaattaaggagatagcttcatctatatctagattaaccggagtaattgattataaag :  97800
Seq3 : ggaactggtgtaccgttcgaatcaccaaatttacaaaaaaatcaattaaggagatagcttcatctatatctagattaaccggagtaattgattataaag :  97800
Seq4 : ggaactggtgtaccgttcgaatcaccaaatttacaaaaaaatcaattaaggagatagcttcatctatatctagattaaccggagtaattgattataaag :  97800

                  *     97820         *     97840         *     97860         *     97880         *     97900
Seq1 : gttataaccttaatataatagacggggttataccctggaattattacttaagttgtaaattaggagaaacaaaaagtcacgcgatctactgggataagat :  97900
Seq2 : gttataaccttaatataatagacggggttataccctggaattattacttaagttgtaaattaggagaaacaaaaagtcacgcgatctactgggataagat :  97900
Seq3 : gttataaccttaatataatagacggggttataccctggaattattacttaagttgtaaattaggagaaacaaaaagtcacgcgatctactgggataagat :  97900
Seq4 : gttataaccttaatataatagacggggttataccctggaattattacttaagttgtaaattaggagaaacaaaaagtcacgcgatctactgggataagat :  97900

                  *     97920         *     97940         *     97960         *     97980         *     98000
Seq1 : ttccaagttactgctgcagcatataactaaacacgttagtgttctttattgtttgggtaaaacagatttctcgaatatacggccaagttagaatccccg :  98000
Seq2 : ttccaagttactgctgcagcatataactaaacacgttagtgttctttattgtttgggtaaaacagatttctcgaatatacggccaagttagaatccccg :  98000
Seq3 : ttccaagttactgctgcagcatataactaaacacgttagtgttctttattgtttgggtaaaacagatttctcgaatatacggccaagttagaatccccg :  98000
Seq4 : ttccaagttactgctgcagcatataactaaacacgttagtgttctttattgtttgggtaaaacagatttctcgaatatacggccaagttagaatccccg :  98000
```

```
                *      98020         *      98040         *      98060         *      98080         *      98100
Seq1 : gtaactaccatagtcggatatcatccagcggctagagaccgccaattcgagaaagatagatcatttgaaattatcaacgttttactggaattagacaaca :  98100
Seq2 : gtaactaccatagtcggatatcatccagcggctagagaccgccaattcgagaaagatagatcatttgaaattatcaacgttttactggaattagacaaca :  98100
Seq3 : gtaactaccatagtcggatatcatccagcggctagagaccgccaattcgagaaagatagatcatttgaaattatcaacgttttactggaattagacaaca :  98100
Seq4 : gtaactaccatagtcggatatcatccagcggctagagaccgccaattcgagaaagatagatcatttgaaattatcaacgttttactggaattagacaaca :  98100

                *      98120         *      98140         *      98160         *      98180         *      98200
Seq1 : aggcacctataaattgggctcaagggtttatttattaatgctttagtgaaattttaacttgtgttctaaatggatgcggctattagaggtaatgatgtta :  98200
Seq2 : aggcacctataaattgggctcaagggtttatttattaatgctttagtgaaattttaacttgtgttctaaatggatgcggctattagaggtaatgatgtta :  98200
Seq3 : aggcacctataaattgggctcaagggtttatttattaatgctttagtgaaattttaacttgtgttctaaatggatgcggctattagaggtaatgatgtta :  98200
Seq4 : aggcacctataaattgggctcaagggtttatttattaatgctttagtgaaattttaacttgtgttctaaatggatgcggctattagaggtaatgatgtta :  98200

                *      98220         *      98240         *      98260         *      98280         *      98300
Seq1 : tctttgttcttaagactataggtgtcccgtcagcgtgcagacaaaatgaagatccaagatttgtagaagcatttaaatgcgacgagttagaaagatatat :  98300
Seq2 : tctttgttcttaagactataggtgtcccgtcagcgtgcagacaaaatgaagatccaagatttgtagaagcatttaaatgcgacgagttagaaagatatat :  98300
Seq3 : tctttgttcttaagactataggtgtcccgtcagcgtgcagacaaaatgaagatccaagatttgtagaagcatttaaatgcgacgagttagaaagatatat :  98300
Seq4 : tctttgttcttaagactataggtgtcccgtcagcgtgcagacaaaatgaagatccaagatttgtagaagcatttaaatgcgacgagttagaaagatatat :  98300

                *      98320         *      98340         *      98360         *      98380         *      98400
Seq1 : tgagaataatccagaatgtacactattcgaaagtcttagggatgaggaagcatactctatagtcagaattttcatggatgtagatttagacgcgtgtcta :  98400
Seq2 : tgagaataatccagaatgtacactattcgaaagtcttagggatgaggaagcatactctatagtcagaattttcatggatgtagatttagacgcgtgtcta :  98400
Seq3 : tgagaataatccagaatgtacactattcgaaagtcttagggatgaggaagcatactctatagtcagaattttcatggatgtagatttagacgcgtgtcta :  98400
Seq4 : tgagaataatccagaatgtacactattcgaaagtcttagggatgaggaagcatactctatagtcagaattttcatggatgtagatttagacgcgtgtcta :  98400

                *      98420         *      98440         *      98460         *      98480         *      98500
Seq1 : gacgaaatagattatttaacggctattcaagattttattatcgaggtgtcaaactgtgtagctagattcgcgtttacagaatgcggcgccattcatgaaa :  98500
Seq2 : gacgaaatagattatttaacggctattcaagattttattatcgaggtgtcaaactgtgtagctagattcgcgtttacagaatgcggcgccattcatgaaa :  98500
Seq3 : gacgaaatagattatttaacggctattcaagattttattatcgaggtgtcaaactgtgtagctagattcgcgtttacagaatgcggcgccattcatgaaa :  98500
Seq4 : gacgaaatagattatttaacggctattcaagattttattatcgaggtgtcaaactgtgtagctagattcgcgtttacagaatgcggcgccattcatgaaa :  98500

                *      98520         *      98540         *      98560         *      98580         *      98600
Seq1 : atgtaataaaatccatgagatctaatttttcattgactaagtctacaaatagagataaaacaagttttcatattatctttttagacacgtataccactat :  98600
Seq2 : atgtaataaaatccatgagatctaatttttcattgactaagtctacaaatagagataaaacaagttttcatattatctttttagacacgtataccactat :  98600
Seq3 : atgtaataaaatccatgagatctaatttttcattgactaagtctacaaatagagataaaacaagttttcatattatctttttagacacgtataccactat :  98600
Seq4 : atgtaataaaatccatgagatctaatttttcattgactaagtctacaaatagagataaaacaagttttcatattatctttttagacacgtataccactat :  98600

                *      98620         *      98640         *      98660         *      98680         *      98700
Seq1 : ggatacattgatagctatgaaacgaacactattagaattaagtagatcatctgaaaatccactaacaagatcgatagacactgccgtatataggagaaaa :  98700
Seq2 : ggatacattgatagctatgaaacgaacactattagaattaagtagatcatctgaaaatccactaacaagatcgatagacactgccgtatataggagaaaa :  98700
Seq3 : ggatacattgatagctatgaaacgaacactattagaattaagtagatcatctgaaaatccactaacaagatcgatagacactgccgtatataggagaaaa :  98700
Seq4 : ggatacattgatagctatgaaacgaacactattagaattaagtagatcatctgaaaatccactaacaagatcgatagacactgccgtatataggagaaaa :  98700
```

```
             *     98720         *     98740         *     98760         *     98780         *     98800
Seq1 : acaactcttcgggttgtaggtactaggaaaaatccaaattgcgacactattcatgtaatgcaaccaccgcatgataatatagaagattacctattcactt :  98800
Seq2 : acaactcttcgggttgtaggtactaggaaaaatccaaattgcgacactattcatgtaatgcaaccaccgcatgataatatagaagattacctattcactt :  98800
Seq3 : acaactcttcgggttgtaggtactaggaaaaatccaaattgcgacactattcatgtaatgcaaccaccgcatgataatatagaagattacctattcactt :  98800
Seq4 : acaactcttcgggttgtaggtactaggaaaaatccaaattgcgacactattcatgtaatgcaaccaccgcatgataatatagaagattacctattcactt :  98800

             *     98820         *     98840         *     98860         *     98880         *     98900
Seq1 : acgtggatatgaacaacaatagttattacttttctctacaacaacgattggaggatttagttcctgataagttatgggaaccagggtttatttcattcga :  98900
Seq2 : acgtggatatgaacaacaatagttattacttttctctacaacaacgattggaggatttagttcctgataagttatgggaaccagggtttatttcattcga :  98900
Seq3 : acgtggatatgaacaacaatagttattacttttctctacaacaacgattggaggatttagttcctgataagttatgggaaccagggtttatttcattcga :  98900
Seq4 : acgtggatatgaacaacaatagttattacttttctctacaacaacgattggaggatttagttcctgataagttatgggaaccagggtttatttcattcga :  98900

             *     98920         *     98940         *     98960         *     98980         *     99000
Seq1 : agacgctataaaaagagtttcaaaaatattcattaattctataataaactttaatgatctcgatgaaaataattttacaacggtaccactggtcatagat :  99000
Seq2 : agacgctataaaaagagtttcaaaaatattcattaattctataataaactttaatgatctcgatgaaaataattttacaacggtaccactggtcatagat :  99000
Seq3 : agacgctataaaaagagtttcaaaaatattcattaattctataataaactttaatgatctcgatgaaaataattttacaacggtaccactggtcatagat :  99000
Seq4 : agacgctataaaaagagtttcaaaaatattcattaattctataataaactttaatgatctcgatgaaaataattttacaacggtaccactggtcatagat :  99000

             *     99020         *     99040         *     99060         *     99080         *     99100
Seq1 : tacgtaacaccttgtgcattatgtaaaaaacgatcgcataaacatccgcatcaactatcgttggaaaatggtgctattagaatttacaaaactggtaatc :  99100
Seq2 : tacgtaacaccttgtgcattatgtaaaaaacgatcgcataaacatccgcatcaactatcgttggaaaatggtgctattagaatttacaaaactggtaatc :  99100
Seq3 : tacgtaacaccttgtgcattatgtaaaaaacgatcgcataaacatccgcatcaactatcgttggaaaatggtgctattagaatttacaaaactggtaatc :  99100
Seq4 : tacgtaacaccttgtgcattatgtaaaaaacgatcgcataaacatccgcatcaactatcgttggaaaatggtgctattagaatttacaaaactggtaatc :  99100

             *     99120         *     99140         *     99160         *     99180         *     99200
Seq1 : cacatagttgtaaagttaaaattgttccgttggatggtaataaactgtttaatattgcacaaagaattttagacactaactctgttttattaaccgaacg :  99200
Seq2 : cacatagttgtaaagttaaaattgttccgttggatggtaataaactgtttaatattgcacaaagaattttagacactaactctgttttattaaccgaacg :  99200
Seq3 : cacatagttgtaaagttaaaattgttccgttggatggtaataaactgtttaatattgcacaaagaattttagacactaactctgttttattaaccgaacg :  99200
Seq4 : cacatagttgtaaagttaaaattgttccgttggatggtaataaactgtttaatattgcacaaagaattttagacactaactctgttttattaaccgaacg :  99200

             *     99220         *     99240         *     99260         *     99280         *     99300
Seq1 : aggagaccatatagtttggattaataattcatggaaatttaacagcgaagaacccttgataacaaaactaattttgtcaataagacatcaactacctaag :  99300
Seq2 : aggagaccatatagtttggattaataattcatggaaatttaacagcgaagaacccttgataacaaaactaattttgtcaataagacatcaactacctaag :  99300
Seq3 : aggagaccatatagtttggattaataattcatggaaatttaacagcgaagaacccttgataacaaaactaattttgtcaataagacatcaactacctaag :  99300
Seq4 : aggagaccatatagtttggattaataattcatggaaatttaacagcgaagaacccttgataacaaaactaattttgtcaataagacatcaactacctaag :  99300

             *     99320         *     99340         *     99360         *     99380         *     99400
Seq1 : gaatattcaagcgaattactctgtccaagaaaacgaaagactgtagaagctaacatacgagacatgttagtagattcagtagagaccgatacctatccgg :  99400
Seq2 : gaatattcaagcgaattactctgtccaagaaaacgaaagactgtagaagctaacatacgagacatgttagtagattcagtagagaccgatacctatccgg :  99400
Seq3 : gaatattcaagcgaattactctgtccaagaaaacgaaagactgtagaagctaacatacgagacatgttagtagattcagtagagaccgatacctatccgg :  99400
Seq4 : gaatattcaagcgaattactctgtccaagaaaacgaaagactgtagaagctaacatacgagacatgttagtagattcagtagagaccgatacctatccgg :  99400
```

```
               *     99420          *     99440          *     99460          *     99480          *     99500
Seq1 : ataaacttccgtttaaaaatggtgtattggacctggtagacggaatgttttactctggagatgatgctaaaaaatatacgtgtactgtatcaaccggatt :  99500
Seq2 : ataaacttccgtttaaaaatggtgtattggacctggtagacggaatgttttactctggagatgatgctaaaaaatatacgtgtactgtatcaaccggatt :  99500
Seq3 : ataaacttccgtttaaaaatggtgtattggacctggtagacggaatgttttactctggagatgatgctaaaaaatatacgtgtactgtatcaaccggatt :  99500
Seq4 : ataaacttccgtttaaaaatggtgtattggacctggtagacggaatgttttactctggagatgatgctaaaaaatatacgtgtactgtatcaaccggatt :  99500

               *     99520          *     99540          *     99560          *     99580          *     99600
Seq1 : taaatttgacgatacaaagttcgtcgaagacagtccagaaatggaagagttaatgaatatcattaacgatatccaaccattaacggatgaaaataagaaa :  99600
Seq2 : taaatttgacgatacaaagttcgtcgaagacagtccagaaatggaagagttaatgaatatcattaacgatatccaaccattaacggatgaaaataagaaa :  99600
Seq3 : taaatttgacgatacaaagttcgtcgaagacagtccagaaatggaagagttaatgaatatcattaacgatatccaaccattaacggatgaaaataagaaa :  99600
Seq4 : taaatttgacgatacaaagttcgtcgaagacagtccagaaatggaagagttaatgaatatcattaacgatatccaaccattaacggatgaaaataagaaa :  99600

               *     99620          *     99640          *     99660          *     99680          *     99700
Seq1 : aatagagagctatatgaaaaaacattatctagttgtttatgcggtgctaccaaaggatgtttaacattctttttggagaaactgcaactggaaagtcga :  99700
Seq2 : aatagagagctatatgaaaaaacattatctagttgtttatgcggtgctaccaaaggatgtttaacattctttttggagaaactgcaactggaaagtcga :  99700
Seq3 : aatagagagctatatgaaaaaacattatctagttgtttatgcggtgctaccaaaggatgtttaacattctttttggagaaactgcaactggaaagtcga :  99700
Seq4 : aatagagagctatatgaaaaaacattatctagttgtttatgcggtgctaccaaaggatgtttaacattctttttggagaaactgcaactggaaagtcga :  99700

               *     99720          *     99740          *     99760          *     99780          *     99800
Seq1 : caaccaaacgtttgttaaagtctgctatcggtgacctgtttgttgagacgggtcaaacaattttaacagatgtattggataaaggacctaatccatttat :  99800
Seq2 : caaccaaacgtttgttaaagtctgctatcggtgacctgtttgttgagacgggtcaaacaattttaacagatgtattggataaaggacctaatccatttat :  99800
Seq3 : caaccaaacgtttgttaaagtctgctatcggtgacctgtttgttgagacgggtcaaacaattttaacagatgtattggataaaggacctaatccatttat :  99800
Seq4 : caaccaaacgtttgttaaagtctgctatcggtgacctgtttgttgagacgggtcaaacaattttaacagatgtattggataaaggacctaatccatttat :  99800

               *     99820          *     99840          *     99860          *     99880          *     99900
Seq1 : cgctaacatgcatttgaaaagatctgtattctgtagcgaactacctgattttgcctgtagtggatcaaagaaaattagatctgacaatattaaaaagttg :  99900
Seq2 : cgctaacatgcatttgaaaagatctgtattctgtagcgaactacctgattttgcctgtagtggatcaaagaaaattagatctgacaatattaaaaagttg :  99900
Seq3 : cgctaacatgcatttgaaaagatctgtattctgtagcgaactacctgattttgcctgtagtggatcaaagaaaattagatctgacaatattaaaaagttg :  99900
Seq4 : cgctaacatgcatttgaaaagatctgtattctgtagcgaactacctgattttgcctgtagtggatcaaagaaaattagatctgacaatattaaaaagttg :  99900

               *     99920          *     99940          *     99960          *     99980          *    100000
Seq1 : acagaaccttgtgtcattggaagaccgtgtttctccaataaaattaataatagaaaccatgcgacaatcattatcgatactaattacaaacctgtttttg : 100000
Seq2 : acagaaccttgtgtcattggaagaccgtgtttctccaataaaattaataatagaaaccatgcgacaatcattatcgatactaattacaaacctgtttttg : 100000
Seq3 : acagaaccttgtgtcattggaagaccgtgtttctccaataaaattaataatagaaaccatgcgacaatcattatcgatactaattacaaacctgtttttg : 100000
Seq4 : acagaaccttgtgtcattggaagaccgtgtttctccaataaaattaataatagaaaccatgcgacaatcattatcgatactaattacaaacctgtttttg : 100000

               *    100020          *    100040          *    100060          *    100080          *    100100
Seq1 : ataggatagataacgcattaatgagaagaattgccgtcgtgcgattcagaacacacttttctcaaccttctggtagagaggctgctgaaaataatgacgc : 100100
Seq2 : ataggatagataacgcattaatgagaagaattgccgtcgtgcgattcagaacacacttttctcaaccttctggtagagaggctgctgaaaataatgacgc : 100100
Seq3 : ataggatagataacgcattaatgagaagaattgccgtcgtgcgattcagaacacacttttctcaaccttctggtagagaggctgctgaaaataatgacgc : 100100
Seq4 : ataggatagataacgcattaatgagaagaattgccgtcgtgcgattcagaacacacttttctcaaccttctggtagagaggctgctgaaaataatgacgc : 100100
```

```
              *    100120         *    100140         *    100160         *    100180         *    100200
Seq1 : gtacgataaagtcaaactattagacgaggggttagatggtaaaatacaaaataatagatatagattcgcatttctatacttgttggtgaaatggtacaga : 100200
Seq2 : gtacgataaagtcaaactattagacgaggggttagatggtaaaatacaaaataatagatatagattcgcatttctatacttgttggtgaaatggtacaga : 100200
Seq3 : gtacgataaagtcaaactattagacgaggggttagatggtaaaatacaaaataatagatatagattcgcatttctatacttgttggtgaaatggtacaga : 100200
Seq4 : gtacgataaagtcaaactattagacgaggggttagatggtaaaatacaaaataatagatatagattcgcatttctatacttgttggtgaaatggtacaga : 100200

              *    100220         *    100240         *    100260         *    100280         *    100300
Seq1 : aaatatcatgttcctattatgaaactatatcctacacccgaagagattcctgactttgcattctatctcaaaataggtactctgttagtatctagctctg : 100300
Seq2 : aaatatcatgttcctattatgaaactatatcctacacccgaagagattcctgactttgcattctatctcaaaataggtactctgttagtatctagctctg : 100300
Seq3 : aaatatcatgttcctattatgaaactatatcctacacccgaagagattcctgactttgcattctatctcaaaataggtactctgttagtatctagctctg : 100300
Seq4 : aaatatcatgttcctattatgaaactatatcctacacccgaagagattcctgactttgcattctatctcaaaataggtactctgttagtatctagctctg : 100300

              *    100320         *    100340         *    100360         *    100380         *    100400
Seq1 : taaagcatattccattaatgacggacctctccaaaaagggatatatattgtacgataatgtggtcactcttccgttgactactttccaacagaaaatatc : 100400
Seq2 : taaagcatattccattaatgacggacctctccaaaaagggatatatattgtacgataatgtggtcactcttccgttgactactttccaacagaaaatatc : 100400
Seq3 : taaagcatattccattaatgacggacctctccaaaaagggatatatattgtacgataatgtggtcactcttccgttgactactttccaacagaaaatatc : 100400
Seq4 : taaagcatattccattaatgacggacctctccaaaaagggatatatattgtacgataatgtggtcactcttccgttgactactttccaacagaaaatatc : 100400

              *    100420         *    100440         *    100460         *    100480         *    100500
Seq1 : caagtattttaattctagactatttggacacgatatagagagcttcatcaatagacataagaaatttgccaatgttagtgatgaatatctgcaatatata : 100500
Seq2 : caagtattttaattctagactatttggacacgatatagagagcttcatcaatagacataagaaatttgccaatgttagtgatgaatatctgcaatatata : 100500
Seq3 : caagtattttaattctagactatttggacacgatatagagagcttcatcaatagacataagaaatttgccaatgttagtgatgaatatctgcaatatata : 100500
Seq4 : caagtattttaattctagactatttggacacgatatagagagcttcatcaatagacataagaaatttgccaatgttagtgatgaatatctgcaatatata : 100500

              *    100520         *    100540         *    100560         *    100580         *    100600
Seq1 : ttcatagaggatatttcatctccgtaaatatatgctcatatatttatagaagatatcacatatctaaatgaataccggaatcatagatttatttgataat : 100600
Seq2 : ttcatagaggatatttcatctccgtaaatatatgctcatatatttatagaagatatcacatatctaaatgaataccggaatcatagatttatttgataat : 100600
Seq3 : ttcatagaggatatttcatctccgtaaatatatgctcatatatttatagaagatatcacatatctaaatgaataccggaatcatagatttatttgataat : 100600
Seq4 : ttcatagaggatatttcatctccgtaaatatatgctcatatatttatagaagatatcacatatctaaatgaataccggaatcatagatttatttgataat : 100600

              *    100620         *    100640         *    100660         *    100680         *    100700
Seq1 : catgttgatagtataccaactatattacctcatcagttagctactctagattatctagttagaactatcatagatgagaacagaagcgtgttattgttcc : 100700
Seq2 : catgttgatagtataccaactatattacctcatcagttagctactctagattatctagttagaactatcatagatgagaacagaagcgtgttattgttcc : 100700
Seq3 : catgttgatagtataccaactatattacctcatcagttagctactctagattatctagttagaactatcatagatgagaacagaagcgtgttattgttcc : 100700
Seq4 : catgttgatagtataccaactatattacctcatcagttagctactctagattatctagttagaactatcatagatgagaacagaagcgtgttattgttcc : 100700

              *    100720         *    100740         *    100760         *    100780         *    100800
Seq1 : atattatgggatcaggtaaaacaataatcgctttgttgttcgccttggtagcttccagatttaaaaaggtttacattctagtgcctaatatcaacatttt : 100800
Seq2 : atattatgggatcaggtaaaacaataatcgctttgttgttcgccttggtagcttccagatttaaaaaggtttacattctagtgcctaatatcaacatttt : 100800
Seq3 : atattatgggatcaggtaaaacaataatcgctttgttgttcgccttggtagcttccagatttaaaaaggtttacattctagtgcctaatatcaacatttt : 100800
Seq4 : atattatgggatcaggtaaaacaataatcgctttgttgttcgccttggtagcttccagatttaaaaaggtttacattctagtgcctaatatcaacatttt : 100800
```

```
               *    100820          *    100840          *    100860          *    100880          *    100900
Seq1 : gaaaattttaattataatatgggtgtagctatgaacttgtttaatgacgaattcatagctgagaatatctttattcattccacaacaagtttttattct : 100900
Seq2 : gaaaattttaattataatatgggtgtagctatgaacttgtttaatgacgaattcatagctgagaatatctttattcattccacaacaagtttttattct : 100900
Seq3 : gaaaattttaattataatatgggtgtagctatgaacttgtttaatgacgaattcatagctgagaatatctttattcattccacaacaagtttttattct : 100900
Seq4 : gaaaattttaattataatatgggtgtagctatgaacttgtttaatgacgaattcatagctgagaatatctttattcattccacaacaagtttttattct : 100900

               *    100920          *    100940          *    100960          *    100980          *    101000
Seq1 : cttaattataacgataacgtcattaattataacggattatctcgctacaataactctatttttatcgttgatgaggcacataatatctttgggaataata : 101000
Seq2 : cttaattataacgataacgtcattaattataacggattatctcgctacaataactctatttttatcgttgatgaggcacataatatctttgggaataata : 101000
Seq3 : cttaattataacgataacgtcattaattataacggattatctcgctacaataactctatttttatcgttgatgaggcacataatatctttgggaataata : 101000
Seq4 : cttaattataacgataacgtcattaattataacggattatctcgctacaataactctatttttatcgttgatgaggcacataatatctttgggaataata : 101000

               *    101020          *    101040          *    101060          *    101080          *    101100
Seq1 : ctggagaacttatgaccgtgataaaaaataaaaacaagattccttttctactattgtctggatctcccattactaacacacctaatactctgggtcatat : 101100
Seq2 : ctggagaacttatgaccgtgataaaaaataaaaacaagattccttttctactattgtctggatctcccattactaacacacctaatactctgggtcatat : 101100
Seq3 : ctggagaacttatgaccgtgataaaaaataaaaacaagattccttttctactattgtctggatctcccattactaacacacctaatactctgggtcatat : 101100
Seq4 : ctggagaacttatgaccgtgataaaaaataaaaacaagattccttttctactattgtctggatctcccattactaacacacctaatactctgggtcatat : 101100

               *    101120          *    101140          *    101160          *    101180          *    101200
Seq1 : tatagatttaatgtccgaagagacgatagattttggtgagattattagtcgtggtaagaaagtaattcagacacttcttaacgaacgcggtgtgaatgta : 101200
Seq2 : tatagatttaatgtccgaagagacgatagattttggtgagattattagtcgtggtaagaaagtaattcagacacttcttaacgaacgcggtgtgaatgta : 101200
Seq3 : tatagatttaatgtccgaagagacgatagattttggtgagattattagtcgtggtaagaaagtaattcagacacttcttaacgaacgcggtgtgaatgta : 101200
Seq4 : tatagatttaatgtccgaagagacgatagattttggtgagattattagtcgtggtaagaaagtaattcagacacttcttaacgaacgcggtgtgaatgta : 101200

               *    101220          *    101240          *    101260          *    101280          *    101300
Seq1 : cttaaggatttgcttaaaggaagaatatcatattacgaaatgcctgataaagatctaccaacgataagatatcacggacgtaagtttctagatactagag : 101300
Seq2 : cttaaggatttgcttaaaggaagaatatcatattacgaaatgcctgataaagatctaccaacgataagatatcacggacgtaagtttctagatactagag : 101300
Seq3 : cttaaggatttgcttaaaggaagaatatcatattacgaaatgcctgataaagatctaccaacgataagatatcacggacgtaagtttctagatactagag : 101300
Seq4 : cttaaggatttgcttaaaggaagaatatcatattacgaaatgcctgataaagatctaccaacgataagatatcacggacgtaagtttctagatactagag : 101300

               *    101320          *    101340          *    101360          *    101380          *    101400
Seq1 : tagtatattgtcacatgtctaaacttcaagagagagattatatgattactagacgacagctatgttatcatgaaatgtttgataaaaatatgtataacgt : 101400
Seq2 : tagtatattgtcacatgtctaaacttcaagagagagattatatgattactagacgacagctatgttatcatgaaatgtttgataaaaatatgtataacgt : 101400
Seq3 : tagtatattgtcacatgtctaaacttcaagagagagattatatgattactagacgacagctatgttatcatgaaatgtttgataaaaatatgtataacgt : 101400
Seq4 : tagtatattgtcacatgtctaaacttcaagagagagattatatgattactagacgacagctatgttatcatgaaatgtttgataaaaatatgtataacgt : 101400

               *    101420          *    101440          *    101460          *    101480          *    101500
Seq1 : gtcaatggcagtattgggacaacttaatctgatgaataatttagatactttatttcaggaacaggataaggaattgtacccaaatctgaaaataaataat : 101500
Seq2 : gtcaatggcagtattgggacaacttaatctgatgaataatttagatactttatttcaggaacaggataaggaattgtacccaaatctgaaaataaataat : 101500
Seq3 : gtcaatggcagtattgggacaacttaatctgatgaataatttagatactttatttcaggaacaggataaggaattgtacccaaatctgaaaataaataat : 101500
Seq4 : gtcaatggcagtattgggacaacttaatctgatgaataatttagatactttatttcaggaacaggataaggaattgtacccaaatctgaaaataaataat : 101500
```

```
                *    101520         *    101540         *    101560         *    101580         *    101600
Seq1 : ggcgtgttatacggagaagaattggtaacgttaaacattagttccaaatttaaatactttattaatcggatacagacactcaacggaaaacattttatat : 101600
Seq2 : ggcgtgttatacggagaagaattggtaacgttaaacattagttccaaatttaaatactttattaatcggatacagacactcaacggaaaacattttatat : 101600
Seq3 : ggcgtgttatacggagaagaattggtaacgttaaacattagttccaaatttaaatactttattaatcggatacagacactcaacggaaaacattttatat : 101600
Seq4 : ggcgtgttatacggagaagaattggtaacgttaaacattagttccaaatttaaatactttattaatcggatacagacactcaacggaaaacattttatat : 101600

                *    101620         *    101640         *    101660         *    101680         *    101700
Seq1 : acttttctaattctacatatggcggattggtaattaaatatatcatgctcagtaatggatattctgaatataatggttctcagggaactaatccacatat : 101700
Seq2 : acttttctaattctacatatggcggattggtaattaaatatatcatgctcagtaatggatattctgaatataatggttctcagggaactaatccacatat : 101700
Seq3 : acttttctaattctacatatggcggattggtaattaaatatatcatgctcagtaatggatattctgaatataatggttctcagggaactaatccacatat : 101700
Seq4 : acttttctaattctacatatggcggattggtaattaaatatatcatgctcagtaatggatattctgaatataatggttctcagggaactaatccacatat : 101700

                *    101720         *    101740         *    101760         *    101780         *    101800
Seq1 : gataaacggcaaaccaaaaacatttgctatcgttactagtaaaatgaaatcgtctttagaggatctattagatgtgtataattctcctgaaaacgatgat : 101800
Seq2 : gataaacggcaaaccaaaaacatttgctatcgttactagtaaaatgaaatcgtctttagaggatctattagatgtgtataattctcctgaaaacgatgat : 101800
Seq3 : gataaacggcaaaccaaaaacatttgctatcgttactagtaaaatgaaatcgtctttagaggatctattagatgtgtataattctcctgaaaacgatgat : 101800
Seq4 : gataaacggcaaaccaaaaacatttgctatcgttactagtaaaatgaaatcgtctttagaggatctattagatgtgtataattctcctgaaaacgatgat : 101800

                *    101820         *    101840         *    101860         *    101880         *    101900
Seq1 : ggtagtcaattgatgtttttgttttcgtcaaacattatgtccgaatcctatactctgaaagaggtaaggcatatttggtttatgactatcccagatactt : 101900
Seq2 : ggtagtcaattgatgtttttgttttcgtcaaacattatgtccgaatcctatactctgaaagaggtaaggcatatttggtttatgactatcccagatactt : 101900
Seq3 : ggtagtcaattgatgtttttgttttcgtcaaacattatgtccgaatcctatactctgaaagaggtaaggcatatttggtttatgactatcccagatactt : 101900
Seq4 : ggtagtcaattgatgtttttgttttcgtcaaacattatgtccgaatcctatactctgaaagaggtaaggcatatttggtttatgactatcccagatactt : 101900

                *    101920         *    101940         *    101960         *    101980         *    102000
Seq1 : tttctcaatacaaccaaattcttggacgatctattagaaaattctcttacgccgatatttctgaaccagttaatgtatatcttttagccgccgtatattc : 102000
Seq2 : tttctcaatacaaccaaattcttggacgatctattagaaaattctcttacgccgatatttctgaaccagttaatgtatatcttttagccgccgtatattc : 102000
Seq3 : tttctcaatacaaccaaattcttggacgatctattagaaaattctcttacgccgatatttctgaaccagttaatgtatatcttttagccgccgtatattc : 102000
Seq4 : tttctcaatacaaccaaattcttggacgatctattagaaaattctcttacgccgatatttctgaaccagttaatgtatatcttttagccgccgtatattc : 102000

                *    102020         *    102040         *    102060         *    102080         *    102100
Seq1 : cgatttcaatgacgaagtaacgtcattaaacgattacacacaggatgaattgattaatgttttaccatttgacatcaaaaagctgttatatctaaaattt : 102100
Seq2 : cgatttcaatgacgaagtaacgtcattaaacgattacacacaggatgaattgattaatgttttaccatttgacatcaaaaagctgttatatctaaaattt : 102100
Seq3 : cgatttcaatgacgaagtaacgtcattaaacgattacacacaggatgaattgattaatgttttaccatttgacatcaaaaagctgttatatctaaaattt : 102100
Seq4 : cgatttcaatgacgaagtaacgtcattaaacgattacacacaggatgaattgattaatgttttaccatttgacatcaaaaagctgttatatctaaaattt : 102100

                *    102120         *    102140         *    102160         *    102180         *    102200
Seq1 : aagactaaagaaacgaatagaatatactctattcttcaagagatgtctgaaacgtattctcttccaccacatccatcaattgtaaaagttttattggag : 102200
Seq2 : aagactaaagaaacgaatagaatatactctattcttcaagagatgtctgaaacgtattctcttccaccacatccatcaattgtaaaagttttattggag : 102200
Seq3 : aagactaaagaaacgaatagaatatactctattcttcaagagatgtctgaaacgtattctcttccaccacatccatcaattgtaaaagttttattggag : 102200
Seq4 : aagactaaagaaacgaatagaatatactctattcttcaagagatgtctgaaacgtattctcttccaccacatccatcaattgtaaaagttttattggag : 102200
```

```
            *    102220         *    102240         *    102260         *    102280         *    102300
Seq1 : aattggtcagacaatttttttataataattctcgtattaagtataacgactccaagttacttaaaatggttacatcagttataaaaaataaagaagacgc : 102300
Seq2 : aattggtcagacaatttttttataataattctcgtattaagtataacgactccaagttacttaaaatggttacatcagttataaaaaataaagaagacgc : 102300
Seq3 : aattggtcagacaatttttttataataattctcgtattaagtataacgactccaagttacttaaaatggttacatcagttataaaaaataaagaagacgc : 102300
Seq4 : aattggtcagacaatttttttataataattctcgtattaagtataacgactccaagttacttaaaatggttacatcagttataaaaaataaagaagacgc : 102300

            *    102320         *    102340         *    102360         *    102380         *    102400
Seq1 : taggaattacatagatgatattgtaaacggtcacttctttgtatcgaataaagtatttgataaatctcttttatacaaatacgaaaacgatattattaca : 102400
Seq2 : taggaattacatagatgatattgtaaacggtcacttctttgtatcgaataaagtatttgataaatctcttttatacaaatacgaaaacgatattattaca : 102400
Seq3 : taggaattacatagatgatattgtaaacggtcacttctttgtatcgaataaagtatttgataaatctcttttatacaaatacgaaaacgatattattaca : 102400
Seq4 : taggaattacatagatgatattgtaaacggtcacttctttgtatcgaataaagtatttgataaatctcttttatacaaatacgaaaacgatattattaca : 102400

            *    102420         *    102440         *    102460         *    102480         *    102500
Seq1 : gtaccgtttagactttcctacgaaccatttgtttggggagttaactttcgtaaagaatataacgtggtatcttctccataaaactgatgaaatatataaa : 102500
Seq2 : gtaccgtttagactttcctacgaaccatttgtttggggagttaactttcgtaaagaatataacgtggtatcttctccataaaactgatgaaatatataaa : 102500
Seq3 : gtaccgtttagactttcctacgaaccatttgtttggggagttaactttcgtaaagaatataacgtggtatcttctccataaaactgatgaaatatataaa : 102500
Seq4 : gtaccgtttagactttcctacgaaccatttgtttggggagttaactttcgtaaagaatataacgtggtatcttctccataaaactgatgaaatatataaa : 102500

            *    102520         *    102540         *    102560         *    102580         *    102600
Seq1 : gaaataaatgtcgagctttgttaccaatggataccttccagttacattggaaccacacgagctgacgttagacataaaaactaatattaggaatgccgta : 102600
Seq2 : gaaataaatgtcgagctttgttaccaatggataccttccagttacattggaaccacacgagctgacgttagacataaaaactaatattaggaatgccgta : 102600
Seq3 : gaaataaatgtcgagctttgttaccaatggataccttccagttacattggaaccacacgagctgacgttagacataaaaactaatattaggaatgccgta : 102600
Seq4 : gaaataaatgtcgagctttgttaccaatggataccttccagttacattggaaccacacgagctgacgttagacataaaaactaatattaggaatgccgta : 102600

            *    102620         *    102640         *    102660         *    102680         *    102700
Seq1 : tataagacgtatctccatagagaaattagtggtaaaatggccaagaaaatagaaattcgtgaagacgtggaattacctctcggcgaaatagttaataatt : 102700
Seq2 : tataagacgtatctccatagagaaattagtggtaaaatggccaagaaaatagaaattcgtgaagacgtggaattacctctcggcgaaatagttaataatt : 102700
Seq3 : tataagacgtatctccatagagaaattagtggtaaaatggccaagaaaatagaaattcgtgaagacgtggaattacctctcggcgaaatagttaataatt : 102700
Seq4 : tataagacgtatctccatagagaaattagtggtaaaatggccaagaaaatagaaattcgtgaagacgtggaattacctctcggcgaaatagttaataatt : 102700

            *    102720         *    102740         *    102760         *    102780         *    102800
Seq1 : ctgtagttataaacgttccgtgtgtaataacctacgcgtattatcacgttggggatatagtcagaggaacattaaacatcgaagatgaatcaaatgtaac : 102800
Seq2 : ctgtagttataaacgttccgtgtgtaataacctacgcgtattatcacgttggggatatagtcagaggaacattaaacatcgaagatgaatcaaatgtaac : 102800
Seq3 : ctgtagttataaacgttccgtgtgtaataacctacgcgtattatcacgttggggatatagtcagaggaacattaaacatcgaagatgaatcaaatgtaac : 102800
Seq4 : ctgtagttataaacgttccgtgtgtaataacctacgcgtattatcacgttggggatatagtcagaggaacattaaacatcgaagatgaatcaaatgtaac : 102800

            *    102820         *    102840         *    102860         *    102880         *    102900
Seq1 : tattcaatgtggagatttaatctgtaaactaagtagagattcgggtactgtatcatttagcgattcaaagtactgctttttttcgaaatggtaatgcgtat : 102900
Seq2 : tattcaatgtggagatttaatctgtaaactaagtagagattcgggtactgtatcatttagcgattcaaagtactgctttttttcgaaatggtaatgcgtat : 102900
Seq3 : tattcaatgtggagatttaatctgtaaactaagtagagattcgggtactgtatcatttagcgattcaaagtactgctttttttcgaaatggtaatgcgtat : 102900
Seq4 : tattcaatgtggagatttaatctgtaaactaagtagagattcgggtactgtatcatttagcgattcaaagtactgctttttttcgaaatggtaatgcgtat : 102900
```

```
                *    102920         *    102940         *    102960         *    102980         *    103000
Seq1 : gacaatggcagcgaagtcactgccgttctaatggaggctcaacaaggtatcgaatctagttttgtttttctcgcgaatatcgtcgactcataaaaaagag : 103000
Seq2 : gacaatggcagcgaagtcactgccgttctaatggaggctcaacaaggtatcgaatctagttttgtttttctcgcgaatatcgtcgactcataaaaaagag : 103000
Seq3 : gacaatggcagcgaagtcactgccgttctaatggaggctcaacaaggtatcgaatctagttttgtttttctcgcgaatatcgtcgactcataaaaaagag : 103000
Seq4 : gacaatggcagcgaagtcactgccgttctaatggaggctcaacaaggtatcgaatctagttttgtttttctcgcgaatatcgtcgactcataaaaaagag : 103000

                *    103020         *    103040         *    103060         *    103080         *    103100
Seq1 : aatagcggtaagtataaacacgaatactatggcaataattgcgaatgttttattctcttcgatatatttttgataatatgaaaaacatgtctctctcaaa : 103100
Seq2 : aatagcggtaagtataaacacgaatactatggcaataattgcgaatgttttattctcttcgatatatttttgataatatgaaaaacatgtctctctcaaa : 103100
Seq3 : aatagcggtaagtataaacacgaatactatggcaataattgcgaatgttttattctcttcgatatatttttgataatatgaaaaacatgtctctctcaaa : 103100
Seq4 : aatagcggtaagtataaacacgaatactatggcaataattgcgaatgttttattctcttcgatatatttttgataatatgaaaaacatgtctctctcaaa : 103100

                *    103120         *    103140         *    103160         *    103180         *    103200
Seq1 : tcggacaaccatctcataaaatagttctcgcgcgctggagaggtagttgctgctcgtataatctccccagaataatatacttgcgtgtcgtcgttcaatt : 103200
Seq2 : tcggacaaccatctcataaaatagttctcgcgcgctggagaggtagttgctgctcgtataatctccccagaataatatacttgcgtgtcgtcgttcaatt : 103200
Seq3 : tcggacaaccatctcataaaatagttctcgcgcgctggagaggtagttgctgctcgtataatctccccagaataatatacttgcgtgtcgtcgttcaatt : 103200
Seq4 : tcggacaaccatctcataaaatagttctcgcgcgctggagaggtagttgctgctcgtataatctccccagaataatatacttgcgtgtcgtcgttcaatt : 103200

                *    103220         *    103240         *    103260         *    103280         *    103300
Seq1 : tatacggatttctatagttctctgttatataatgcggttttccatcatgattagacgacgacaatagtgttctgaatttagatagttgatcagaatgaat : 103300
Seq2 : tatacggatttctatagttctctgttatataatgcggttttccatcatgattagacgacgacaatagtgttctgaatttagatagttgatcagaatgaat : 103300
Seq3 : tatacggatttctatagttctctgttatataatgcggttttccatcatgattagacgacgacaatagtgttctgaatttagatagttgatcagaatgaat : 103300
Seq4 : tatacggatttctatagttctctgttatataatgcggttttccatcatgattagacgacgacaatagtgttctgaatttagatagttgatcagaatgaat : 103300

                *    103320         *    103340         *    103360         *    103380         *    103400
Seq1 : gtttattggcgttggaaaaattatccatacagcgtctgcagagtggttgatagttgttcctagatatgtaaaataatccaacttactaggcagcaaattg : 103400
Seq2 : gtttattggcgttggaaaaattatccatacagcgtctgcagagtggttgatagttgttcctagatatgtaaaataatccaacttactaggcagcaaattg : 103400
Seq3 : gtttattggcgttggaaaaattatccatacagcgtctgcagagtggttgatagttgttcctagatatgtaaaataatccaacttactaggcagcaaattg : 103400
Seq4 : gtttattggcgttggaaaaattatccatacagcgtctgcagagtggttgatagttgttcctagatatgtaaaataatccaacttactaggcagcaaattg : 103400

                *    103420         *    103440         *    103460         *    103480         *    103500
Seq1 : tctagataaaatactgaatcaaacggtgcagacgtattggcggatctaatggaatccaattgattaactatcttttgaaaatatacatttttatgatcca : 103500
Seq2 : tctagataaaatactgaatcaaacggtgcagacgtattggcggatctaatggaatccaattgattaactatcttttgaaaatatacatttttatgatcca : 103500
Seq3 : tctagataaaatactgaatcaaacggtgcagacgtattggcggatctaatggaatccaattgattaactatcttttgaaaatatacatttttatgatcca : 103500
Seq4 : tctagataaaatactgaatcaaacggtgcagacgtattggcggatctaatggaatccaattgattaactatcttttgaaaatatacatttttatgatcca : 103500

                *    103520         *    103540         *    103560         *    103580         *    103600
Seq1 : atacttgtaagaatatagaaataatgataagtccatcatcgtgtttttttgcctcttcataagaactatatttttttcttattccaatgaacaagattaat : 103600
Seq2 : atacttgtaagaatatagaaataatgataagtccatcatcgtgtttttttgcctcttcataagaactatatttttttcttattccaatgaacaagattaat : 103600
Seq3 : atacttgtaagaatatagaaataatgataagtccatcatcgtgtttttttgcctcttcataagaactatatttttttcttattccaatgaacaagattaat : 103600
Seq4 : atacttgtaagaatatagaaataatgataagtccatcatcgtgtttttttgcctcttcataagaactatatttttttcttattccaatgaacaagattaat : 103600
```

```
             *    103620          *    103640          *    103660          *    103680          *    103700
Seq1 : ctctccagagtatttgtacacatctatcaagtgattggatccataatcgtcttcctttccccaatatatatgtagtgatgataacacatattcattgggg : 103700
Seq2 : ctctccagagtatttgtacacatctatcaagtgattggatccataatcgtcttcctttccccaatatatatgtagtgatgataacacatattcattgggg : 103700
Seq3 : ctctccagagtatttgtacacatctatcaagtgattggatccataatcgtcttcctttccccaatatatatgtagtgatgataacacatattcattgggg : 103700
Seq4 : ctctccagagtatttgtacacatctatcaagtgattggatccataatcgtcttcctttccccaatatatatgtagtgatgataacacatattcattgggg : 103700

             *    103720          *    103740          *    103760          *    103780          *    103800
Seq1 : agaaaccctccacttatatatcctcctttaaaattaatccttactagttttccagtgttctggatagtggttggtttcgactcattataatgtatgtcta : 103800
Seq2 : agaaaccctccacttatatatcctcctttaaaattaatccttactagttttccagtgttctggatagtggttggtttcgactcattataatgtatgtcta : 103800
Seq3 : agaaaccctccacttatatatcctcctttaaaattaatccttactagttttccagtgttctggatagtggttggtttcgactcattataatgtatgtcta : 103800
Seq4 : agaaaccctccacttatatatcctcctttaaaattaatccttactagttttccagtgttctggatagtggttggtttcgactcattataatgtatgtcta : 103800

             *    103820          *    103840          *    103860          *    103880          *    103900
Seq1 : acggcttcaatcgcgcgttagaaattgcttttttagtttctatattaataggagatagttgttgcggcatagtaaaaatgaaatgataactgtttaaaaa : 103900
Seq2 : acggcttcaatcgcgcgttagaaattgcttttttagtttctatattaataggagatagttgttgcggcatagtaaaaatgaaatgataactgtttaaaaa : 103900
Seq3 : acggcttcaatcgcgcgttagaaattgcttttttagtttctatattaataggagatagttgttgcggcatagtaaaaatgaaatgataactgtttaaaaa : 103900
Seq4 : acggcttcaatcgcgcgttagaaattgcttttttagtttctatattaataggagatagttgttgcggcatagtaaaaatgaaatgataactgtttaaaaa : 103900

             *    103920          *    103940          *    103960          *    103980          *    104000
Seq1 : tagctcttagtatgggaattacaatggatgaggaagtgatatttgaaactcctagagaattaatatctattaaacgaataaaagatattccaagatcaaa : 104000
Seq2 : tagctcttagtatgggaattacaatggatgaggaagtgatatttgaaactcctagagaattaatatctattaaacgaataaaagatattccaagatcaaa : 104000
Seq3 : tagctcttagtatgggaattacaatggatgaggaagtgatatttgaaactcctagagaattaatatctattaaacgaataaaagatattccaagatcaaa : 104000
Seq4 : tagctcttagtatgggaattacaatggatgaggaagtgatatttgaaactcctagagaattaatatctattaaacgaataaaagatattccaagatcaaa : 104000

             *    104020          *    104040          *    104060          *    104080          *    104100
Seq1 : agacacgcacgtgtttgctgcgtgtataacaagtgacggatatccgttaataggagctagaagaacttcattcgcgttccaggcgatattatctcaacaa : 104100
Seq2 : agacacgcacgtgtttgctgcgtgtataacaagtgacggatatccgttaataggagctagaagaacttcattcgcgttccaggcgatattatctcaacaa : 104100
Seq3 : agacacgcacgtgtttgctgcgtgtataacaagtgacggatatccgttaataggagctagaagaacttcattcgcgttccaggcgatattatctcaacaa : 104100
Seq4 : agacacgcacgtgtttgctgcgtgtataacaagtgacggatatccgttaataggagctagaagaacttcattcgcgttccaggcgatattatctcaacaa : 104100

             *    104120          *    104140          *    104160          *    104180          *    104200
Seq1 : aattcagattctatctttagagtatccactaaactattacggtttatgtactacaatgaactaagagaaatctttagacggttgagaaaaggttctatca : 104200
Seq2 : aattcagattctatctttagagtatccactaaactattacggtttatgtactacaatgaactaagagaaatctttagacggttgagaaaaggttctatca : 104200
Seq3 : aattcagattctatctttagagtatccactaaactattacggtttatgtactacaatgaactaagagaaatctttagacggttgagaaaaggttctatca : 104200
Seq4 : aattcagattctatctttagagtatccactaaactattacggtttatgtactacaatgaactaagagaaatctttagacggttgagaaaaggttctatca : 104200

             *    104220          *    104240          *    104260          *    104280          *    104300
Seq1 : acaatatcgatcctcactttgaagagttaatattattgggtggtaaactagataaaaaggaatctattaaagattgtttaagaagagaattaaaagagga : 104300
Seq2 : acaatatcgatcctcactttgaagagttaatattattgggtggtaaactagataaaaaggaatctattaaagattgtttaagaagagaattaaaagagga : 104300
Seq3 : acaatatcgatcctcactttgaagagttaatattattgggtggtaaactagataaaaaggaatctattaaagattgtttaagaagagaattaaaagagga : 104300
Seq4 : acaatatcgatcctcactttgaagagttaatattattgggtggtaaactagataaaaaggaatctattaaagattgtttaagaagagaattaaaagagga : 104300
```

```
              *    104320         *    104340         *    104360         *    104380         *    104400
Seq1 : aagtgatgaacgtataacagtaaaagaattcggaaatgtaattctaaaacttacaacgcgcgataaattatttaataaagtatatataggttattgcatg : 104400
Seq2 : aagtgatgaacgtataacagtaaaagaattcggaaatgtaattctaaaacttacaacgcgcgataaattatttaataaagtatatataggttattgcatg : 104400
Seq3 : aagtgatgaacgtataacagtaaaagaattcggaaatgtaattctaaaacttacaacgcgcgataaattatttaataaagtatatataggttattgcatg : 104400
Seq4 : aagtgatgaacgtataacagtaaaagaattcggaaatgtaattctaaaacttacaacgcgcgataaattatttaataaagtatatataggttattgcatg : 104400

              *    104420         *    104440         *    104460         *    104480         *    104500
Seq1 : gcgtgttttattaatcaatcgttggaggatttatcgcatactagtatttacaatgtagaaattagaaagattaaatcattaaatgattgtattaacgacg : 104500
Seq2 : gcgtgttttattaatcaatcgttggaggatttatcgcatactagtatttacaatgtagaaattagaaagattaaatcattaaatgattgtattaacgacg : 104500
Seq3 : gcgtgttttattaatcaatcgttggaggatttatcgcatactagtatttacaatgtagaaattagaaagattaaatcattaaatgattgtattaacgacg : 104500
Seq4 : gcgtgttttattaatcaatcgttggaggatttatcgcatactagtatttacaatgtagaaattagaaagattaaatcattaaatgattgtattaacgacg : 104500

              *    104520         *    104540         *    104560         *    104580         *    104600
Seq1 : ataaatacgaatatctgtcttatatttataatatgctagttaatagtaaatgaacttttacagatctagtataattagtcagattattaagtataataga : 104600
Seq2 : ataaatacgaatatctgtcttatatttataatatgctagttaatagtaaatgaacttttacagatctagtataattagtcagattattaagtataataga : 104600
Seq3 : ataaatacgaatatctgtcttatatttataatatgctagttaatagtaaatgaacttttacagatctagtataattagtcagattattaagtataataga : 104600
Seq4 : ataaatacgaatatctgtcttatatttataatatgctagttaatagtaaatgaacttttacagatctagtataattagtcagattattaagtataataga : 104600

              *    104620         *    104640         *    104660         *    104680         *    104700
Seq1 : cgactagctaagtctattatttgcgaggatgactctcaaattattacactcacggcattcgttaaccaatgcctatggtgtcataaacgagtatccgtgt : 104700
Seq2 : cgactagctaagtctattatttgcgaggatgactctcaaattattacactcacggcattcgttaaccaatgcctatggtgtcataaacgagtatccgtgt : 104700
Seq3 : cgactagctaagtctattatttgcgaggatgactctcaaattattacactcacggcattcgttaaccaatgcctatggtgtcataaacgagtatccgtgt : 104700
Seq4 : cgactagctaagtctattatttgcgaggatgactctcaaattattacactcacggcattcgttaaccaatgcctatggtgtcataaacgagtatccgtgt : 104700

              *    104720         *    104740         *    104760         *    104780         *    104800
Seq1 : ccgctattttattaactactgataacaaaatattagtatgtaacagacgagatagttttctctattctgaaataattagaactagaaacatgtttagaaa : 104800
Seq2 : ccgctattttattaactactgataacaaaatattagtatgtaacagacgagatagttttctctattctgaaataattagaactagaaacatgtttagaaa : 104800
Seq3 : ccgctattttattaactactgataacaaaatattagtatgtaacagacgagatagttttctctattctgaaataattagaactagaaacatgtttagaaa : 104800
Seq4 : ccgctattttattaactactgataacaaaatattagtatgtaacagacgagatagttttctctattctgaaataattagaactagaaacatgtttagaaa : 104800

              *    104820         *    104840         *    104860         *    104880         *    104900
Seq1 : gaaacgattatttctgaattattccaattatttgaacaaacaggaaagaagtatactatcgtcatttttttctctagatccagctactgctgataatgat : 104900
Seq2 : gaaacgattatttctgaattattccaattatttgaacaaacaggaaagaagtatactatcgtcatttttttctctagatccagctactgctgataatgat : 104900
Seq3 : gaaacgattatttctgaattattccaattatttgaacaaacaggaaagaagtatactatcgtcatttttttctctagatccagctactgctgataatgat : 104900
Seq4 : gaaacgattatttctgaattattccaattatttgaacaaacaggaaagaagtatactatcgtcatttttttctctagatccagctactgctgataatgat : 104900

              *    104920         *    104940         *    104960         *    104980         *    105000
Seq1 : agaatagacgctatttatccgggtggcatacccaaaaggggtgagaatgttccagagtgtttatccagggaaattaaagaagaagttaatatagacaatt : 105000
Seq2 : agaatagacgctatttatccgggtggcatacccaaaaggggtgagaatgttccagagtgtttatccagggaaattaaagaagaagttaatatagacaatt : 105000
Seq3 : agaatagacgctatttatccgggtggcatacccaaaaggggtgagaatgttccagagtgtttatccagggaaattaaagaagaagttaatatagacaatt : 105000
Seq4 : agaatagacgctatttatccgggtggcatacccaaaaggggtgagaatgttccagagtgtttatccagggaaattaaagaagaagttaatatagacaatt : 105000
```

```
                *    105020         *    105040         *    105060         *    105080         *    105100
Seq1 : cttttgtattcatagacactcggttttttattcatggcatcatagaagataccattattaataaattttttgaggtaatcttctttgtcggaagaatatc : 105100
Seq2 : cttttgtattcatagacactcggttttttattcatggcatcatagaagataccattattaataaattttttgaggtaatcttctttgtcggaagaatatc : 105100
Seq3 : cttttgtattcatagacactcggttttttattcatggcatcatagaagataccattattaataaattttttgaggtaatcttctttgtcggaagaatatc : 105100
Seq4 : cttttgtattcatagacactcggttttttattcatggcatcatagaagataccattattaataaattttttgaggtaatcttctttgtcggaagaatatc : 105100

                *    105120         *    105140         *    105160         *    105180         *    105200
Seq1 : tctaacgagtgatcaaatcattgatacatttaaaagtaatcatgaaatcaaggatctaatatttttagatccgaattcaggtaatggactccaatacgaa : 105200
Seq2 : tctaacgagtgatcaaatcattgatacatttaaaagtaatcatgaaatcaaggatctaatatttttagatccgaattcaggtaatggactccaatacgaa : 105200
Seq3 : tctaacgagtgatcaaatcattgatacatttaaaagtaatcatgaaatcaaggatctaatatttttagatccgaattcaggtaatggactccaatacgaa : 105200
Seq4 : tctaacgagtgatcaaatcattgatacatttaaaagtaatcatgaaatcaaggatctaatatttttagatccgaattcaggtaatggactccaatacgaa : 105200

                *    105220         *    105240         *    105260         *    105280         *    105300
Seq1 : attgcaaaatatgctctagatactgcaaaactcaaatgttatggccatagaggatgttattacgaatcattaaaaaaattaactgaggatgattgattag : 105300
Seq2 : attgcaaaatatgctctagatactgcaaaactcaaatgttatggccatagaggatgttattacgaatcattaaaaaaattaactgaggatgattgattag : 105300
Seq3 : attgcaaaatatgctctagatactgcaaaactcaaatgttatggccatagaggatgttattacgaatcattaaaaaaattaactgaggatgattgattag : 105300
Seq4 : attgcaaaatatgctctagatactgcaaaactcaaatgttatggccatagaggatgttattacgaatcattaaaaaaattaactgaggatgattgattag : 105300

                *    105320         *    105340         *    105360         *    105380         *    105400
Seq1 : aaaatataaattaatttaccatcgtgtatttttataacgggattgtccggcatatcatgtagatagttaccgtctacatcgtatactcgaccatctacgc : 105400
Seq2 : aaaatataaattaatttaccatcgtgtatttttataacgggattgtccggcatatcatgtagatagttaccgtctacatcgtatactcgaccatctacgc : 105400
Seq3 : aaaatataaattaatttaccatcgtgtatttttataacgggattgtccggcatatcatgtagatagttaccgtctacatcgtatactcgaccatctacgc : 105400
Seq4 : aaaatataaattaatttaccatcgtgtatttttataacgggattgtccggcatatcatgtagatagttaccgtctacatcgtatactcgaccatctacgc : 105400

                *    105420         *    105440         *    105460         *    105480         *    105500
Seq1 : ctttaaatcctctatttattgacattaatctattagaattggaataccaaatattagtaccctcaattagtttattggtaatatttttgttagacgatag : 105500
Seq2 : ctttaaatcctctatttattgacattaatctattagaattggaataccaaatattagtaccctcaattagtttattggtaatatttttgttagacgatag : 105500
Seq3 : ctttaaatcctctatttattgacattaatctattagaattggaataccaaatattagtaccctcaattagtttattggtaatatttttgttagacgatag : 105500
Seq4 : ctttaaatcctctatttattgacattaatctattagaattggaataccaaatattagtaccctcaattagtttattggtaatatttttgttagacgatag : 105500

                *    105520         *    105540         *    105560         *    105580         *    105600
Seq1 : atcgatggctcttgaaaccaaggttttccaaccggactcattgtcgatcggtgagaagtctttttcattagcatgaatccattctaatgatgtatgttta : 105600
Seq2 : atcgatggctcttgaaaccaaggttttccaaccggactcattgtcgatcggtgagaagtctttttcattagcatgaatccattctaatgatgtatgttta : 105600
Seq3 : atcgatggctcttgaaaccaaggttttccaaccggactcattgtcgatcggtgagaagtctttttcattagcatgaatccattctaatgatgtatgttta : 105600
Seq4 : atcgatggctcttgaaaccaaggttttccaaccggactcattgtcgatcggtgagaagtctttttcattagcatgaatccattctaatgatgtatgttta : 105600

                *    105620         *    105640         *    105660         *    105680         *    105700
Seq1 : aacactctaaacaattggacaaattcttttgatttgctttgaatgatttcaaataggtcttcgtctacagtaggcataccattagataatctagccatta : 105700
Seq2 : aacactctaaacaattggacaaattcttttgatttgctttgaatgatttcaaataggtcttcgtctacagtaggcataccattagataatctagccatta : 105700
Seq3 : aacactctaaacaattggacaaattcttttgatttgctttgaatgatttcaaataggtcttcgtctacagtaggcataccattagataatctagccatta : 105700
Seq4 : aacactctaaacaattggacaaattcttttgatttgctttgaatgatttcaaataggtcttcgtctacagtaggcataccattagataatctagccatta : 105700
```

```
                 *     105720         *     105740         *     105760         *     105780         *     105800
Seq1 : taaagtgcacgtttacatatctacgttctggaggagtaagaacgtgactattgagacgaatggctcttcctactatctgacgaagagacgcctcgttcca : 105800
Seq2 : taaagtgcacgtttacatatctacgttctggaggagtaagaacgtgactattgagacgaatggctcttcctactatctgacgaagagacgcctcgttcca : 105800
Seq3 : taaagtgcacgtttacatatctacgttctggaggagtaagaacgtgactattgagacgaatggctcttcctactatctgacgaagagacgcctcgttcca : 105800
Seq4 : taaagtgcacgtttacatatctacgttctggaggagtaagaacgtgactattgagacgaatggctcttcctactatctgacgaagagacgcctcgttcca : 105800

                 *     105820         *     105840         *     105860         *     105880         *     105900
Seq1 : tgtcatatctaaaatgaagatatcattgattgagaagaagctaataccctcgcctccactagaagagaatacgcatgttttaatgcattctccgttagtg : 105900
Seq2 : tgtcatatctaaaatgaagatatcattgattgagaagaagctaataccctcgcctccactagaagagaatacgcatgttttaatgcattctccgttagtg : 105900
Seq3 : tgtcatatctaaaatgaagatatcattgattgagaagaagctaataccctcgcctccactagaagagaatacgcatgttttaatgcattctccgttagtg : 105900
Seq4 : tgtcatatctaaaatgaagatatcattgattgagaagaagctaataccctcgcctccactagaagagaatacgcatgttttaatgcattctccgttagtg : 105900

                 *     105920         *     105940         *     105960         *     105980         *     106000
Seq1 : tttgattcttggttaaactcagccaccgccttgattctagtatcttttgttctagatgagaactctatattagagataccaaagactttgaaatatagta : 106000
Seq2 : tttgattcttggttaaactcagccaccgccttgattctagtatcttttgttctagatgagaactctatattagagataccaaagactttgaaatatagta : 106000
Seq3 : tttgattcttggttaaactcagccaccgccttgattctagtatcttttgttctagatgagaactctatattagagataccaaagactttgaaatatagta : 106000
Seq4 : tttgattcttggttaaactcagccaccgccttgattctagtatcttttgttctagatgagaactctatattagagataccaaagactttgaaatatagta : 106000

                 *     106020         *     106040         *     106060         *     106080         *     106100
Seq1 : ataagatttctattcctgactgattaacaaatggttcaaagactagacatttaccatgggatgctaatattcccaaacatacatctataaatttgacgct : 106100
Seq2 : ataagatttctattcctgactgattaacaaatggttcaaagactagacatttaccatgggatgctaatattcccaaacatacatctataaatttgacgct : 106100
Seq3 : ataagatttctattcctgactgattaacaaatggttcaaagactagacatttaccatgggatgctaatattcccaaacatacatctataaatttgacgct : 106100
Seq4 : ataagatttctattcctgactgattaacaaatggttcaaagactagacatttaccatgggatgctaatattcccaaacatacatctataaatttgacgct : 106100

                 *     106120         *     106140         *     106160         *     106180         *     106200
Seq1 : tttctcttttaattcagtaaatagagagatatcagccgcactagcatcccctcccaatagttctccccttttaaaggtatctaatgcagatttagaaaat : 106200
Seq2 : tttctcttttaattcagtaaatagagagatatcagccgcactagcatcccctcccaatagttctccccttttaaaggtatctaatgcagatttagaaaat : 106200
Seq3 : tttctcttttaattcagtaaatagagagatatcagccgcactagcatcccctcccaatagttctccccttttaaaggtatctaatgcagatttagaaaat : 106200
Seq4 : tttctcttttaattcagtaaatagagagatatcagccgcactagcatcccctcccaatagttctccccttttaaaggtatctaatgcagatttagaaaat : 106200

                 *     106220         *     106240         *     106260         *     106280         *     106300
Seq1 : tctctatctcttaatgaattttttaaaatcattatatagtgttgctatctcttgcgcgtattcgcccggatcacgattttgtctttcaggaaagctatcga : 106300
Seq2 : tctctatctcttaatgaattttttaaaatcattatatagtgttgctatctcttgcgcgtattcgcccggatcacgattttgtctttcaggaaagctatcga : 106300
Seq3 : tctctatctcttaatgaattttttaaaatcattatatagtgttgctatctcttgcgcgtattcgcccggatcacgattttgtctttcaggaaagctatcga : 106300
Seq4 : tctctatctcttaatgaattttttaaaatcattatatagtgttgctatctcttgcgcgtattcgcccggatcacgattttgtctttcaggaaagctatcga : 106300

                 *     106320         *     106340         *     106360         *     106380         *     106400
Seq1 : acgtaaacgtagtagccatacgtctcagaattctaaatgatgatatacctgtttttatttcagcgagtttagccttttgataaatttcttcttgcttttt : 106400
Seq2 : acgtaaacgtagtagccatacgtctcagaattctaaatgatgatatacctgtttttatttcagcgagtttagccttttgataaatttcttcttgcttttt : 106400
Seq3 : acgtaaacgtagtagccatacgtctcagaattctaaatgatgatatacctgtttttatttcagcgagtttagccttttgataaatttcttcttgcttttt : 106400
Seq4 : acgtaaacgtagtagccatacgtctcagaattctaaatgatgatatacctgtttttatttcagcgagtttagccttttgataaatttcttcttgcttttt : 106400
```

```
              *    106420         *    106440         *    106460         *    106480         *    106500
Seq1 : cgacatattaacgtatcgcattaatactgtttcttagcgaatgatgcagacccttctacgtcatcaaaaatagaaaactcgttattaactatgtacgaa : 106500
Seq2 : cgacatattaacgtatcgcattaatactgtttcttagcgaatgatgcagacccttctacgtcatcaaaaatagaaaactcgttattaactatgtacgaa : 106500
Seq3 : cgacatattaacgtatcgcattaatactgtttcttagcgaatgatgcagacccttctacgtcatcaaaaatagaaaactcgttattaactatgtacgaa : 106500
Seq4 : cgacatattaacgtatcgcattaatactgtttcttagcgaatgatgcagacccttctacgtcatcaaaaatagaaaactcgttattaactatgtacgaa : 106500

              *    106520         *    106540         *    106560         *    106580         *    106600
Seq1 : cataggcctcctagtttggagactaattctttttcatcaactagacgtttattctcaaatagcgattggtgttgtaaggatcctggtcgtagtaagttaa : 106600
Seq2 : cataggcctcctagtttggagactaattctttttcatcaactagacgtttattctcaaatagcgattggtgttgtaaggatcctggtcgtagtaagttaa : 106600
Seq3 : cataggcctcctagtttggagactaattctttttcatcaactagacgtttattctcaaatagcgattggtgttgtaaggatcctggtcgtagtaagttaa : 106600
Seq4 : cataggcctcctagtttggagactaattctttttcatcaactagacgtttattctcaaatagcgattggtgttgtaaggatcctggtcgtagtaagttaa : 106600

              *    106620         *    106640         *    106660         *    106680         *    106700
Seq1 : ccaacatggtgaattcttgcacactattgacgataggtgtagccgataaacaaatcatcttatggtttttaatgcgatggtcttagataaaaaattata : 106700
Seq2 : ccaacatggtgaattcttgcacactattgacgataggtgtagccgataaacaaatcatcttatggtttttaatgcgatggtcttagataaaaaattata : 106700
Seq3 : ccaacatggtgaattcttgcacactattgacgataggtgtagccgataaacaaatcatcttatggtttttaatgcgatggtcttagataaaaaattata : 106700
Seq4 : ccaacatggtgaattcttgcacactattgacgataggtgtagccgataaacaaatcatcttatggtttttaatgcgatggtcttagataaaaaattata : 106700

              *    106720         *    106740         *    106760         *    106780         *    106800
Seq1 : tactgaacgagtaggacggatcttaccatcttctttgattaatgatttagaaatgaagttatgacattcatcaataatgacgcatattctactcttggaa : 106800
Seq2 : tactgaacgagtaggacggatcttaccatcttctttgattaatgatttagaaatgaagttatgacattcatcaataatgacgcatattctactcttggaa : 106800
Seq3 : tactgaacgagtaggacggatcttaccatcttctttgattaatgatttagaaatgaagttatgacattcatcaataatgacgcatattctactcttggaa : 106800
Seq4 : tactgaacgagtaggacggatcttaccatcttctttgattaatgatttagaaatgaagttatgacattcatcaataatgacgcatattctactcttggaa : 106800

              *    106820         *    106840         *    106860         *    106880         *    106900
Seq1 : ttaatagttttgatattagtaaaaaatttatttctaaaattttgatcatcgtaattaataaaaatacaatccttcgttatctctggagcgtatctgagta : 106900
Seq2 : ttaatagttttgatattagtaaaaaatttatttctaaaattttgatcatcgtaattaataaaaatacaatccttcgttatctctggagcgtatctgagta : 106900
Seq3 : ttaatagttttgatattagtaaaaaatttatttctaaaattttgatcatcgtaattaataaaaatacaatccttcgttatctctggagcgtatctgagta : 106900
Seq4 : ttaatagttttgatattagtaaaaaatttatttctaaaattttgatcatcgtaattaataaaaatacaatccttcgttatctctggagcgtatctgagta : 106900

              *    106920         *    106940         *    106960         *    106980         *    107000
Seq1 : tagtgttcatccaaggatcttctatcaaagcctttttcaccaataagataatagcccaattcgtataaatatccttaagatgtttgagaatatatacagt : 107000
Seq2 : tagtgttcatccaaggatcttctatcaaagcctttttcaccaataagataatagcccaattcgtataaatatccttaagatgtttgagaatatatacagt : 107000
Seq3 : tagtgttcatccaaggatcttctatcaaagcctttttcaccaataagataatagcccaattcgtataaatatccttaagatgtttgagaatatatacagt : 107000
Seq4 : tagtgttcatccaaggatcttctatcaaagcctttttcaccaataagataatagcccaattcgtataaatatccttaagatgtttgagaatatatacagt : 107000

              *    107020         *    107040         *    107060         *    107080         *    107100
Seq1 : agtcattgttttaccgacacccgtttcatggaacaataaaagagaatgcatactgtctaatcctaagaaaactcttgctacaaaatgttgataatccttg : 107100
Seq2 : agtcattgttttaccgacacccgtttcatggaacaataaaagagaatgcatactgtctaatcctaagaaaactcttgctacaaaatgttgataatccttg : 107100
Seq3 : agtcattgttttaccgacacccgtttcatggaacaataaaagagaatgcatactgtctaatcctaagaaaactcttgctacaaaatgttgataatccttg : 107100
Seq4 : agtcattgttttaccgacacccgtttcatggaacaataaaagagaatgcatactgtctaatcctaagaaaactcttgctacaaaatgttgataatccttg : 107100
```

```
               *    107120          *    107140          *    107160          *    107180          *    107200
Seq1 : aggcgtactacgtccgaccccatcatttcaacaggcatattagtagttctgcgcaatgcataatcgatataggccgcgtgtgatttactcatttatgagt : 107200
Seq2 : aggcgtactacgtccgaccccatcatttcaacaggcatattagtagttctgcgcaatgcataatcgatataggccgcgtgtgatttactcatttatgagt : 107200
Seq3 : aggcgtactacgtccgaccccatcatttcaacaggcatattagtagttctgcgcaatgcataatcgatataggccgcgtgtgatttactcatttatgagt : 107200
Seq4 : aggcgtactacgtccgaccccatcatttcaacaggcatattagtagttctgcgcaatgcataatcgatataggccgcgtgtgatttactcatttatgagt : 107200

               *    107220          *    107240          *    107260          *    107280          *    107300
Seq1 : gataagtaataactatgttttaaaaatcacagcagtagtttaactagtcttctctgatgtttgttttcgatactttttgaatcagaagtcatactagaat : 107300
Seq2 : gataagtaataactatgttttaaaaatcacagcagtagtttaactagtcttctctgatgtttgttttcgatactttttgaatcagaagtcatactagaat : 107300
Seq3 : gataagtaataactatgttttaaaaatcacagcagtagtttaactagtcttctctgatgtttgttttcgatactttttgaatcagaagtcatactagaat : 107300
Seq4 : gataagtaataactatgttttaaaaatcacagcagtagtttaactagtcttctctgatgtttgttttcgatactttttgaatcagaagtcatactagaat : 107300

               *    107320          *    107340          *    107360          *    107380          *    107400
Seq1 : aaagcaacgagtgaacgtaatagagagcttcgtatactctattcgaaaactctaagaacttattaatgaattccgtatccactggattgtttaaaatact : 107400
Seq2 : aaagcaacgagtgaacgtaatagagagcttcgtatactctattcgaaaactctaagaacttattaatgaattccgtatccactggattgtttaaaatact : 107400
Seq3 : aaagcaacgagtgaacgtaatagagagcttcgtatactctattcgaaaactctaagaacttattaatgaattccgtatccactggattgtttaaaatact : 107400
Seq4 : aaagcaacgagtgaacgtaatagagagcttcgtatactctattcgaaaactctaagaacttattaatgaattccgtatccactggattgtttaaaatact : 107400

               *    107420          *    107440          *    107460          *    107480          *    107500
Seq1 : aaattgaacactgttcacatccttccaagaagaagacttagtgacggacttaacatgagacataaataaatccaaattttttttacaaacatcactagcc : 107500
Seq2 : aaattgaacactgttcacatccttccaagaagaagacttagtgacggacttaacatgagacataaataaatccaaattttttttacaaacatcactagcc : 107500
Seq3 : aaattgaacactgttcacatccttccaagaagaagacttagtgacggacttaacatgagacataaataaatccaaattttttttacaaacatcactagcc : 107500
Seq4 : aaattgaacactgttcacatccttccaagaagaagacttagtgacggacttaacatgagacataaataaatccaaattttttttacaaacatcactagcc : 107500

               *    107520          *    107540          *    107560          *    107580          *    107600
Seq1 : accataatggcgctatcttttaaccagctatcgcttacgcattttagcagtctaacatttttaaagagactacaatatattctcatagtatcgattacac : 107600
Seq2 : accataatggcgctatcttttaaccagctatcgcttacgcattttagcagtctaacatttttaaagagactacaatatattctcatagtatcgattacac : 107600
Seq3 : accataatggcgctatcttttaaccagctatcgcttacgcattttagcagtctaacatttttaaagagactacaatatattctcatagtatcgattacac : 107600
Seq4 : accataatggcgctatcttttaaccagctatcgcttacgcattttagcagtctaacatttttaaagagactacaatatattctcatagtatcgattacac : 107600

               *    107620          *    107640          *    107660          *    107680          *    107700
Seq1 : ctctaccgaataaagttggaagtttaataatacaatatttttcgtttacaaaatcaaataatggtcgaaacacgtcgaaggttaacatcttataatcgct : 107700
Seq2 : ctctaccgaataaagttggaagtttaataatacaatatttttcgtttacaaaatcaaataatggtcgaaacacgtcgaaggttaacatcttataatcgct : 107700
Seq3 : ctctaccgaataaagttggaagtttaataatacaatatttttcgtttacaaaatcaaataatggtcgaaacacgtcgaaggttaacatcttataatcgct : 107700
Seq4 : ctctaccgaataaagttggaagtttaataatacaatatttttcgtttacaaaatcaaataatggtcgaaacacgtcgaaggttaacatcttataatcgct : 107700

               *    107720          *    107740          *    107760          *    107780          *    107800
Seq1 : aatgtatagattgttttcagtgagatgattattagatttaatagcatctcgttcacgtttgaacagtttattgcgtgcgctgaggtcggcaactacggcg : 107800
Seq2 : aatgtatagattgttttcagtgagatgattattagatttaatagcatctcgttcacgtttgaacagtttattgcgtgcgctgaggtcggcaactacggcg : 107800
Seq3 : aatgtatagattgttttcagtgagatgattattagatttaatagcatctcgttcacgtttgaacagtttattgcgtgcgctgaggtcggcaactacggcg : 107800
Seq4 : aatgtatagattgttttcagtgagatgattattagatttaatagcatctcgttcacgtttgaacagtttattgcgtgcgctgaggtcggcaactacggcg : 107800
```

```
                 *    107820         *    107840         *    107860         *    107880         *    107900
Seq1 : tccgctttagtactcctcccataatactttacgctattaatctttaaaatttcatagactttatctagatcgctttctggtaacatgatatcatgtgtaa : 107900
Seq2 : tccgctttagtactcctcccataatactttacgctattaatctttaaaatttcatagactttatctagatcgctttctggtaacatgatatcatgtgtaa : 107900
Seq3 : tccgctttagtactcctcccataatactttacgctattaatctttaaaatttcatagactttatctagatcgctttctggtaacatgatatcatgtgtaa : 107900
Seq4 : tccgctttagtactcctcccataatactttacgctattaatctttaaaatttcatagactttatctagatcgctttctggtaacatgatatcatgtgtaa : 107900

                 *    107920         *    107940         *    107960         *    107980         *    108000
Seq1 : aaagttttaacatgtcggtcggcattctatttagatcattaactctagaaatctgaagaaagtaattagctccgtattccagactaggtaatgggctttt : 108000
Seq2 : aaagttttaacatgtcggtcggcattctatttagatcattaactctagaaatctgaagaaagtaattagctccgtattccagactaggtaatgggctttt : 108000
Seq3 : aaagttttaacatgtcggtcggcattctatttagatcattaactctagaaatctgaagaaagtaattagctccgtattccagactaggtaatgggctttt : 108000
Seq4 : aaagttttaacatgtcggtcggcattctatttagatcattaactctagaaatctgaagaaagtaattagctccgtattccagactaggtaatgggctttt : 108000

                 *    108020         *    108040         *    108060         *    108080         *    108100
Seq1 : acctagagacagattaagttctggcaatgtttcataaaatggaagaaggacatgcgttccctcccggatatttttacaatttcatccatttacaactct : 108100
Seq2 : acctagagacagattaagttctggcaatgtttcataaaatggaagaaggacatgcgttccctcccggatatttttacaatttcatccatttacaactct : 108100
Seq3 : acctagagacagattaagttctggcaatgtttcataaaatggaagaaggacatgcgttccctcccggatatttttacaatttcatccatttacaactct : 108100
Seq4 : acctagagacagattaagttctggcaatgtttcataaaatggaagaaggacatgcgttccctcccggatatttttacaatttcatccatttacaactct : 108100

                 *    108120         *    108140         *    108160         *    108180         *    108200
Seq1 : atagtttgttttcattattattagttattatctcccataatcttggtaatacttaccccttgatcgtaagataccttatacaggtcattacatacaacta : 108200
Seq2 : atagtttgttttcattattattagttattatctcccataatcttggtaatacttaccccttgatcgtaagataccttatacaggtcattacatacaacta : 108200
Seq3 : atagtttgttttcattattattagttattatctcccataatcttggtaatacttaccccttgatcgtaagataccttatacaggtcattacatacaacta : 108200
Seq4 : atagtttgttttcattattattagttattatctcccataatcttggtaatacttaccccttgatcgtaagataccttatacaggtcattacatacaacta : 108200

                 *    108220         *    108240         *    108260         *    108280         *    108300
Seq1 : ccaattgtttttgtacataatagattggatggttgacatccatggtggaataaactactcgaacagatagtttatctttccccctagatacattagccgt : 108300
Seq2 : ccaattgtttttgtacataatagattggatggttgacatccatggtggaataaactactcgaacagatagtttatctttccccctagatacattagccgt : 108300
Seq3 : ccaattgtttttgtacataatagattggatggttgacatccatggtggaataaactactcgaacagatagtttatctttccccctagatacattagccgt : 108300
Seq4 : ccaattgtttttgtacataatagattggatggttgacatccatggtggaataaactactcgaacagatagtttatctttccccctagatacattagccgt : 108300

                 *    108320         *    108340         *    108360         *    108380         *    108400
Seq1 : aatagttgtcggcctaaagaatatctttggtgtaaagttaaaagttagggttcttgttccattattgctttttgtcagtagttcattataaattctcgag : 108400
Seq2 : aatagttgtcggcctaaagaatatctttggtgtaaagttaaaagttagggttcttgttccattattgctttttgtcagtagttcattataaattctcgag : 108400
Seq3 : aatagttgtcggcctaaagaatatctttggtgtaaagttaaaagttagggttcttgttccattattgctttttgtcagtagttcattataaattctcgag : 108400
Seq4 : aatagttgtcggcctaaagaatatctttggtgtaaagttaaaagttagggttcttgttccattattgctttttgtcagtagttcattataaattctcgag : 108400

                 *    108420         *    108440         *    108460         *    108480         *    108500
Seq1 : atgggtccgttctctgaatatagaacatcatttccaaatctaacttctagtctagaaataatatcggtcttattcttaaaatctattcccttgatgaagg : 108500
Seq2 : atgggtccgttctctgaatatagaacatcatttccaaatctaacttctagtctagaaataatatcggtcttattcttaaaatctattcccttgatgaagg : 108500
Seq3 : atgggtccgttctctgaatatagaacatcatttccaaatctaacttctagtctagaaataatatcggtcttattcttaaaatctattcccttgatgaagg : 108500
Seq4 : atgggtccgttctctgaatatagaacatcatttccaaatctaacttctagtctagaaataatatcggtcttattcttaaaatctattcccttgatgaagg : 108500
```

```
                  *    108520         *    108540         *    108560         *    108580         *    108600
Seq1 : gatcgttaatgaacaaatccttggcctttgattcggctgatctattatctccgttatagacgttacgttgactagtccaaagacttacaggaatagatgt : 108600
Seq2 : gatcgttaatgaacaaatccttggcctttgattcggctgatctattatctccgttatagacgttacgttgactagtccaaagacttacaggaatagatgt : 108600
Seq3 : gatcgttaatgaacaaatccttggcctttgattcggctgatctattatctccgttatagacgttacgttgactagtccaaagacttacaggaatagatgt : 108600
Seq4 : gatcgttaatgaacaaatccttggcctttgattcggctgatctattatctccgttatagacgttacgttgactagtccaaagacttacaggaatagatgt : 108600

                  *    108620         *    108640         *    108660         *    108680         *    108700
Seq1 : atcgatgatgttgatactatgtgatatgtgagcaaagattgttctcttagtggcatcactatatgttccagtaatggcggaaaactttttagaaatgtta : 108700
Seq2 : atcgatgatgttgatactatgtgatatgtgagcaaagattgttctcttagtggcatcactatatgttccagtaatggcggaaaactttttagaaatgtta : 108700
Seq3 : atcgatgatgttgatactatgtgatatgtgagcaaagattgttctcttagtggcatcactatatgttccagtaatggcggaaaactttttagaaatgtta : 108700
Seq4 : atcgatgatgttgatactatgtgatatgtgagcaaagattgttctcttagtggcatcactatatgttccagtaatggcggaaaactttttagaaatgtta : 108700

                  *    108720         *    108740         *    108760         *    108780         *    108800
Seq1 : tatataaaagaattttttcgtgttccaaacattagcagattagtatgaagataaacactcatattatcaggaacattatcaatttttacatacacatcag : 108800
Seq2 : tatataaaagaattttttcgtgttccaaacattagcagattagtatgaagataaacactcatattatcaggaacattatcaatttttacatacacatcag : 108800
Seq3 : tatataaaagaattttttcgtgttccaaacattagcagattagtatgaagataaacactcatattatcaggaacattatcaatttttacatacacatcag : 108800
Seq4 : tatataaaagaattttttcgtgttccaaacattagcagattagtatgaagataaacactcatattatcaggaacattatcaatttttacatacacatcag : 108800

                  *    108820         *    108840         *    108860         *    108880         *    108900
Seq1 : catcttgaatagaaacgataccatcttctggaacctctacgatctcggcagactccggataaccagtcggtggaccatcgctaacaataactagatcatc : 108900
Seq2 : catcttgaatagaaacgataccatcttctggaacctctacgatctcggcagactccggataaccagtcggtggaccatcgctaacaataactagatcatc : 108900
Seq3 : catcttgaatagaaacgataccatcttctggaacctctacgatctcggcagactccggataaccagtcggtggaccatcgctaacaataactagatcatc : 108900
Seq4 : catcttgaatagaaacgataccatcttctggaacctctacgatctcggcagactccggataaccagtcggtggaccatcgctaacaataactagatcatc : 108900

                  *    108920         *    108940         *    108960         *    108980         *    109000
Seq1 : caacaatctactcacatatgcatctatataatctttttcatcttgtgagtaccctggatacgaaataaatttattatccgtatttccataataaggttta : 109000
Seq2 : caacaatctactcacatatgcatctatataatctttttcatcttgtgagtaccctggatacgaaataaatttattatccgtatttccataataaggttta : 109000
Seq3 : caacaatctactcacatatgcatctatataatctttttcatcttgtgagtaccctggatacgaaataaatttattatccgtatttccataataaggttta : 109000
Seq4 : caacaatctactcacatatgcatctatataatctttttcatcttgtgagtaccctggatacgaaataaatttattatccgtatttccataataaggttta : 109000

                  *    109020         *    109040         *    109060         *    109080         *    109100
Seq1 : gtataaacagagagcgatgttgccgcatgaacttcagttacagtcgccgttggttggtttatttgacctattactctcctaggtttctctataaatgatg : 109100
Seq2 : gtataaacagagagcgatgttgccgcatgaacttcagttacagtcgccgttggttggtttatttgacctattactctcctaggtttctctataaatgatg : 109100
Seq3 : gtataaacagagagcgatgttgccgcatgaacttcagttacagtcgccgttggttggtttatttgacctattactctcctaggtttctctataaatgatg : 109100
Seq4 : gtataaacagagagcgatgttgccgcatgaacttcagttacagtcgccgttggttggtttatttgacctattactctcctaggtttctctataaatgatg : 109100

                  *    109120         *    109140         *    109160         *    109180         *    109200
Seq1 : gtttaatttgtacattcttaaccatatatccaataaagctcaattcaggaacataaacaaattctttgttgaacgtttcaaagtcgaacgaagagtcacg : 109200
Seq2 : gtttaatttgtacattcttaaccatatatccaataaagctcaattcaggaacataaacaaattctttgttgaacgtttcaaagtcgaacgaagagtcacg : 109200
Seq3 : gtttaatttgtacattcttaaccatatatccaataaagctcaattcaggaacataaacaaattctttgttgaacgtttcaaagtcgaacgaagagtcacg : 109200
Seq4 : gtttaatttgtacattcttaaccatatatccaataaagctcaattcaggaacataaacaaattctttgttgaacgtttcaaagtcgaacgaagagtcacg : 109200
```

```
                   *    109220         *    109240         *    109260         *    109280         *    109300
Seq1 : aataacgatatcggatactggattgaaggttaccgttacggtaatttttgaatcggatagtttaagactgctgaatgtatcttccacatcaaacggagtt : 109300
Seq2 : aataacgatatcggatactggattgaaggttaccgttacggtaatttttgaatcggatagtttaagactgctgaatgtatcttccacatcaaacggagtt : 109300
Seq3 : aataacgatatcggatactggattgaaggttaccgttacggtaatttttgaatcggatagtttaagactgctgaatgtatcttccacatcaaacggagtt : 109300
Seq4 : aataacgatatcggatactggattgaaggttaccgttacggtaatttttgaatcggatagtttaagactgctgaatgtatcttccacatcaaacggagtt : 109300

                   *    109320         *    109340         *    109360         *    109380         *    109400
Seq1 : ttaatataaacgtatactgtagatggttctttaatagtgtcattaggagttaggccaatagaaatatcattaagttcactagaatatccagagtgtttca : 109400
Seq2 : ttaatataaacgtatactgtagatggttctttaatagtgtcattaggagttaggccaatagaaatatcattaagttcactagaatatccagagtgtttca : 109400
Seq3 : ttaatataaacgtatactgtagatggttctttaatagtgtcattaggagttaggccaatagaaatatcattaagttcactagaatatccagagtgtttca : 109400
Seq4 : ttaatataaacgtatactgtagatggttctttaatagtgtcattaggagttaggccaatagaaatatcattaagttcactagaatatccagagtgtttca : 109400

                   *    109420         *    109440         *    109460         *    109480         *    109500
Seq1 : aagcaattgtattattgatacaattattatataattcttcgccctcaatttcccaaataacaccgttacacgaagagatagatacgtgattaatacattt : 109500
Seq2 : aagcaattgtattattgatacaattattatataattcttcgccctcaatttcccaaataacaccgttacacgaagagatagatacgtgattaatacattt : 109500
Seq3 : aagcaattgtattattgatacaattattatataattcttcgccctcaatttcccaaataacaccgttacacgaagagatagatacgtgattaatacattt : 109500
Seq4 : aagcaattgtattattgatacaattattatataattcttcgccctcaatttcccaaataacaccgttacacgaagagatagatacgtgattaatacattt : 109500

                   *    109520         *    109540         *    109560         *    109580         *    109600
Seq1 : atatccaacatatggtacgtaaccgaatcttcccatacctttaacttctggaagttccaaactcagaaccaaatgattaagcgcagtaatatactgatcc : 109600
Seq2 : atatccaacatatggtacgtaaccgaatcttcccatacctttaacttctggaagttccaaactcagaaccaaatgattaagcgcagtaatatactgatcc : 109600
Seq3 : atatccaacatatggtacgtaaccgaatcttcccatacctttaacttctggaagttccaaactcagaaccaaatgattaagcgcagtaatatactgatcc : 109600
Seq4 : atatccaacatatggtacgtaaccgaatcttcccatacctttaacttctggaagttccaaactcagaaccaaatgattaagcgcagtaatatactgatcc : 109600

                   *    109620         *    109640         *    109660         *    109680         *    109700
Seq1 : ctaatttcgaagctagcgatagcctgattgtctggaccatcgtttgtcataactccggatagagaaatatattgcggcatatataaagttggaatttgac : 109700
Seq2 : ctaatttcgaagctagcgatagcctgattgtctggaccatcgtttgtcataactccggatagagaaatatattgcggcatatataaagttggaatttgac : 109700
Seq3 : ctaatttcgaagctagcgatagcctgattgtctggaccatcgtttgtcataactccggatagagaaatatattgcggcatatataaagttggaatttgac : 109700
Seq4 : ctaatttcgaagctagcgatagcctgattgtctggaccatcgtttgtcataactccggatagagaaatatattgcggcatatataaagttggaatttgac : 109700

                   *    109720         *    109740         *    109760         *    109780         *    109800
Seq1 : tatcgactgcgaagacattagaccgtttaatagagtcatccccaccgatcaaagaattaatgatagtattattcattttctatttaaaatggaaaaagct : 109800
Seq2 : tatcgactgcgaagacattagaccgtttaatagagtcatccccaccgatcaaagaattaatgatagtattattcattttctatttaaaatggaaaaagct : 109800
Seq3 : tatcgactgcgaagacattagaccgtttaatagagtcatccccaccgatcaaagaattaatgatagtattattcattttctatttaaaatggaaaaagct : 109800
Seq4 : tatcgactgcgaagacattagaccgtttaatagagtcatccccaccgatcaaagaattaatgatagtattattcattttctatttaaaatggaaaaagct : 109800

                   *    109820         *    109840         *    109860         *    109880         *    109900
Seq1 : tacaataaactccgtagagaaatatctataatttgtgagttttccttaaagtaacagcttccgtaaacgccgtctttatctcttagtaagtttattgtat : 109900
Seq2 : tacaataaactccgtagagaaatatctataatttgtgagttttccttaaagtaacagcttccgtaaacgccgtctttatctcttagtaagtttattgtat : 109900
Seq3 : tacaataaactccgtagagaaatatctataatttgtgagttttccttaaagtaacagcttccgtaaacgccgtctttatctcttagtaagtttattgtat : 109900
Seq4 : tacaataaactccgtagagaaatatctataatttgtgagttttccttaaagtaacagcttccgtaaacgccgtctttatctcttagtaagtttattgtat : 109900
```

```
               *    109920         *    109940         *    109960         *    109980         *    110000
Seq1 : ttataacctttttccttatcttcatagaatactaaaggcaacaaagaaatttttggttcttctctaagagctacgtgagacttaaccatagacgccaacga : 110000
Seq2 : ttataacctttttccttatcttcatagaatactaaaggcaacaaagaaatttttggttcttctctaagagctacgtgagacttaaccatagacgccaacga : 110000
Seq3 : ttataacctttttccttatcttcatagaatactaaaggcaacaaagaaatttttggttcttctctaagagctacgtgagacttaaccatagacgccaacga : 110000
Seq4 : ttataacctttttccttatcttcatagaatactaaaggcaacaaagaaatttttggttcttctctaagagctacgtgagacttaaccatagacgccaacga : 110000

               *    110020         *    110040         *    110060         *    110080         *    110100
Seq1 : atccctacatattttagaacagaaatacccaacttcaccacccttgaatgtctcaatactaataggtttaaaaaccaaatcttgattacaaaaccaacac : 110100
Seq2 : atccctacatattttagaacagaaatacccaacttcaccacccttgaatgtctcaatactaataggtttaaaaaccaaatcttgattacaaaaccaacac : 110100
Seq3 : atccctacatattttagaacagaaatacccaacttcaccacccttgaatgtctcaatactaataggtttaaaaaccaaatcttgattacaaaaccaacac : 110100
Seq4 : atccctacatattttagaacagaaatacccaacttcaccacccttgaatgtctcaatactaataggtttaaaaaccaaatcttgattacaaaaccaacac : 110100

               *    110120         *    110140         *    110160         *    110180         *    110200
Seq1 : ttatcaattacactatttgtcttaatagacacatctgccatagatttataatactttggtagtatacaagcgagtgcttcttctttagcgggcttaaaga : 110200
Seq2 : ttatcaattacactatttgtcttaatagacacatctgccatagatttataatactttggtagtatacaagcgagtgcttcttctttagcgggcttaaaga : 110200
Seq3 : ttatcaattacactatttgtcttaatagacacatctgccatagatttataatactttggtagtatacaagcgagtgcttcttctttagcgggcttaaaga : 110200
Seq4 : ttatcaattacactatttgtcttaatagacacatctgccatagatttataatactttggtagtatacaagcgagtgcttcttctttagcgggcttaaaga : 110200

               *    110220         *    110240         *    110260         *    110280         *    110300
Seq1 : ctgctttaggtgctgaaataaccacatctggaaggcttactcgcttagccatttaattacggaactatttttttatacttctaatgagcaagtagaaaac : 110300
Seq2 : ctgctttaggtgctgaaataaccacatctggaaggcttactcgcttagccatttaattacggaactatttttttatacttctaatgagcaagtagaaaac : 110300
Seq3 : ctgctttaggtgctgaaataaccacatctggaaggcttactcgcttagccatttaattacggaactatttttttatacttctaatgagcaagtagaaaac : 110300
Seq4 : ctgctttaggtgctgaaataaccacatctggaaggcttactcgcttagccatttaattacggaactatttttttatacttctaatgagcaagtagaaaac : 110300

               *    110320         *    110340         *    110360         *    110380         *    110400
Seq1 : ctctcatctacaaaaacatactcgtgtccataatcctctaccatagttacacgtttttttagatctcatatgtgctaaaaagttttcccatactaattggt : 110400
Seq2 : ctctcatctacaaaaacatactcgtgtccataatcctctaccatagttacacgtttttttagatctcatatgtgctaaaaagttttcccatactaattggt : 110400
Seq3 : ctctcatctacaaaaacatactcgtgtccataatcctctaccatagttacacgtttttttagatctcatatgtgctaaaaagttttcccatactaattggt : 110400
Seq4 : ctctcatctacaaaaacatactcgtgtccataatcctctaccatagttacacgtttttttagatctcatatgtgctaaaaagttttcccatactaattggt : 110400

               *    110420         *    110440         *    110460         *    110480         *    110500
Seq1 : tactattatttttcgtataattttaacagtttgaggttttagatttttagttacagaagtgatatcgaatattttatccaaaaagaatgaataattaat : 110500
Seq2 : tactattatttttcgtataattttaacagtttgaggttttagatttttagttacagaagtgatatcgaatattttatccaaaaagaatgaataattaat : 110500
Seq3 : tactattatttttcgtataattttaacagtttgaggttttagatttttagttacagaagtgatatcgaatattttatccaaaaagaatgaataattaat : 110500
Seq4 : tactattatttttcgtataattttaacagtttgaggttttagatttttagttacagaagtgatatcgaatattttatccaaaaagaatgaataattaat : 110500

               *    110520         *    110540         *    110560         *    110580         *    110600
Seq1 : tgtcttagaaggagtgttttcttggcaaaagaataccaagtgcttaaatatttctactacttcattaatcttttctgtactcagattcagtttctcatct : 110600
Seq2 : tgtcttagaaggagtgttttcttggcaaaagaataccaagtgcttaaatatttctactacttcattaatcttttctgtactcagattcagtttctcatct : 110600
Seq3 : tgtcttagaaggagtgttttcttggcaaaagaataccaagtgcttaaatatttctactacttcattaatcttttctgtactcagattcagtttctcatct : 110600
Seq4 : tgtcttagaaggagtgttttcttggcaaaagaataccaagtgcttaaatatttctactacttcattaatcttttctgtactcagattcagtttctcatct : 110600
```

```
                 *    110620          *    110640          *    110660          *    110680          *    110700
Seq1 : tttacttgattgattatttcaaagactaacttataatcctttttatttattctctcgttagccttaagaaaactagatacaaaatttgcatctacatcat : 110700
Seq2 : tttacttgattgattatttcaaagactaacttataatcctttttatttattctctcgttagccttaagaaaactagatacaaaatttgcatctacatcat : 110700
Seq3 : tttacttgattgattatttcaaagactaacttataatcctttttatttattctctcgttagccttaagaaaactagatacaaaatttgcatctacatcat : 110700
Seq4 : tttacttgattgattatttcaaagactaacttataatcctttttatttattctctcgttagccttaagaaaactagatacaaaatttgcatctacatcat : 110700

                 *    110720          *    110740          *    110760          *    110780          *    110800
Seq1 : ccgtggatatttgatttttttccatgatatccaagagttccgagataatttctccagaacattgatgagacaataatctccgcaatacatttctcaaatg : 110800
Seq2 : ccgtggatatttgatttttttccatgatatccaagagttccgagataatttctccagaacattgatgagacaataatctccgcaatacatttctcaaatg : 110800
Seq3 : ccgtggatatttgatttttttccatgatatccaagagttccgagataatttctccagaacattgatgagacaataatctccgcaatacatttctcaaatg : 110800
Seq4 : ccgtggatatttgatttttttccatgatatccaagagttccgagataatttctccagaacattgatgagacaataatctccgcaatacatttctcaaatg : 110800

                 *    110820          *    110840          *    110860          *    110880          *    110900
Seq1 : aataagtttattagacacatggaagtttgactttttttgtacctttgtacatttttgaaataccgactcgcaaaaaatacaatattcatatccttgttca : 110900
Seq2 : aataagtttattagacacatggaagtttgactttttttgtacctttgtacatttttgaaataccgactcgcaaaaaatacaatattcatatccttgttca : 110900
Seq3 : aataagtttattagacacatggaagtttgactttttttgtacctttgtacatttttgaaataccgactcgcaaaaaatacaatattcatatccttgttca : 110900
Seq4 : aataagtttattagacacatggaagtttgactttttttgtacctttgtacatttttgaaataccgactcgcaaaaaatacaatattcatatccttgttca : 110900

                 *    110920          *    110940          *    110960          *    110980          *    111000
Seq1 : gatactataccgttgtgtctacaaccgctacataatcgtagattcatgttaacactctacgtatctcgtcgtccaatattttatataaaaacattttatt : 111000
Seq2 : gatactataccgttgtgtctacaaccgctacataatcgtagattcatgttaacactctacgtatctcgtcgtccaatattttatataaaaacattttatt : 111000
Seq3 : gatactataccgttgtgtctacaaccgctacataatcgtagattcatgttaacactctacgtatctcgtcgtccaatattttatataaaaacattttatt : 111000
Seq4 : gatactataccgttgtgtctacaaccgctacataatcgtagattcatgttaacactctacgtatctcgtcgtccaatattttatataaaaacattttatt : 111000

                 *    111020          *    111040          *    111060          *    111080          *    111100
Seq1 : tctagacgttgccagaaaatcctgtaatatttttagttttttgggctgtgaataaagtatcgccctaatattgttaccgtcttccgccaatatagtagtt : 111100
Seq2 : tctagacgttgccagaaaatcctgtaatatttttagttttttgggctgtgaataaagtatcgccctaatattgttaccgtcttccgccaatatagtagtt : 111100
Seq3 : tctagacgttgccagaaaatcctgtaatatttttagttttttgggctgtgaataaagtatcgccctaatattgttaccgtcttccgccaatatagtagtt : 111100
Seq4 : tctagacgttgccagaaaatcctgtaatatttttagttttttgggctgtgaataaagtatcgccctaatattgttaccgtcttccgccaatatagtagtt : 111100

                 *    111120          *    111140          *    111160          *    111180          *    111200
Seq1 : aaattatccgcacatgcaaaagaacaccgcttaggcggattcagtacaatgttatatttttcgtaccaactcatttaaatatcataatctaaaatagttc : 111200
Seq2 : aaattatccgcacatgcaaaagaacaccgcttaggcggattcagtacaatgttatatttttcgtaccaactcatttaaatatcataatctaaaatagttc : 111200
Seq3 : aaattatccgcacatgcaaaagaacaccgcttaggcggattcagtacaatgttatatttttcgtaccaactcatttaaatatcataatctaaaatagttc : 111200
Seq4 : aaattatccgcacatgcaaaagaacaccgcttaggcggattcagtacaatgttatatttttcgtaccaactcatttaaatatcataatctaaaatagttc : 111200

                 *    111220          *    111240          *    111260          *    111280          *    111300
Seq1 : tgtaatatgtctagcgctaatatattgatcataatcctgtgcataaattaagatacaacaatgtctcgaaatcatcgacatggcttcttccatagttaga : 111300
Seq2 : tgtaatatgtctagcgctaatatattgatcataatcctgtgcataaattaagatacaacaatgtctcgaaatcatcgacatggcttcttccatagttaga : 111300
Seq3 : tgtaatatgtctagcgctaatatattgatcataatcctgtgcataaattaagatacaacaatgtctcgaaatcatcgacatggcttcttccatagttaga : 111300
Seq4 : tgtaatatgtctagcgctaatatattgatcataatcctgtgcataaattaagatacaacaatgtctcgaaatcatcgacatggcttcttccatagttaga : 111300
```

```
                 *     111320          *     111340          *     111360          *     111380          *     111400
Seq1 : agatcgtcgtcaaagttagcaacgtgattcatcaacatttgctgttttgaggcagcaaatactgaaccgtcgccattcaaccattcataaaaaccatcgt : 111400
Seq2 : agatcgtcgtcaaagttagcaacgtgattcatcaacatttgctgttttgaggcagcaaatactgaaccgtcgccattcaaccattcataaaaaccatcgt : 111400
Seq3 : agatcgtcgtcaaagttagcaacgtgattcatcaacatttgctgttttgaggcagcaaatactgaaccgtcgccattcaaccattcataaaaaccatcgt : 111400
Seq4 : agatcgtcgtcaaagttagcaacgtgattcatcaacatttgctgttttgaggcagcaaatactgaaccgtcgccattcaaccattcataaaaaccatcgt : 111400

                 *     111420          *     111440          *     111460          *     111480          *     111500
Seq1 : ctgaatccattgataatttcttgtactggtttttgagagctcgcatcaatctagcatttctagctcccggattgaaaacagaaagaggatcgtacatcca : 111500
Seq2 : ctgaatccattgataatttcttgtactggtttttgagagctcgcatcaatctagcatttctagctcccggattgaaaacagaaagaggatcgtacatcca : 111500
Seq3 : ctgaatccattgataatttcttgtactggtttttgagagctcgcatcaatctagcatttctagctcccggattgaaaacagaaagaggatcgtacatcca : 111500
Seq4 : ctgaatccattgataatttcttgtactggtttttgagagctcgcatcaatctagcatttctagctcccggattgaaaacagaaagaggatcgtacatcca : 111500

                 *     111520          *     111540          *     111560          *     111580          *     111600
Seq1 : gggtccatttctgtaaatagaatcgtataatgtcccttcaagaagatatcagacgatccacaatcaaagaattggtctccgagtttgtaacaaactgcg : 111600
Seq2 : gggtccatttctgtaaatagaatcgtataatgtcccttcaagaagatatcagacgatccacaatcaaagaattggtctccgagtttgtaacaaactgcg : 111600
Seq3 : gggtccatttctgtaaatagaatcgtataatgtcccttcaagaagatatcagacgatccacaatcaaagaattggtctccgagtttgtaacaaactgcg : 111600
Seq4 : gggtccatttctgtaaatagaatcgtataatgtcccttcaagaagatatcagacgatccacaatcaaagaattggtctccgagtttgtaacaaactgcg : 111600

                 *     111620          *     111640          *     111660          *     111680          *     111700
Seq1 : gactttaacctatacatgataccgtttagcatgatttctggtgatacgtcaatcggagtatcatctattagagatctaaagccggtgtaacattctccac : 111700
Seq2 : gactttaacctatacatgataccgtttagcatgatttctggtgatacgtcaatcggagtatcatctattagagatctaaagccggtgtaacattctccac : 111700
Seq3 : gactttaacctatacatgataccgtttagcatgatttctggtgatacgtcaatcggagtatcatctattagagatctaaagccggtgtaacattctccac : 111700
Seq4 : gactttaacctatacatgataccgtttagcatgatttctggtgatacgtcaatcggagtatcatctattagagatctaaagccggtgtaacattctccac : 111700

                 *     111720          *     111740          *     111760          *     111780          *     111800
Seq1 : caaacatattcttattctgacgtcgttctacataaaacatcattgctccattaacgataacaggggaatgaacagcactacccatcacattagttcccaa : 111800
Seq2 : caaacatattcttattctgacgtcgttctacataaaacatcattgctccattaacgataacaggggaatgaacagcactacccatcacattagttcccaa : 111800
Seq3 : caaacatattcttattctgacgtcgttctacataaaacatcattgctccattaacgataacaggggaatgaacagcactacccatcacattagttcccaa : 111800
Seq4 : caaacatattcttattctgacgtcgttctacataaaacatcattgctccattaacgataacaggggaatgaacagcactacccatcacattagttcccaa : 111800

                 *     111820          *     111840          *     111860          *     111880          *     111900
Seq1 : tggatcaatgtgtgtaactccagaacatcttccatatcctatgttaggaggagcgaacaccactcttccactattgccatcgaatgccatagaataaata : 111900
Seq2 : tggatcaatgtgtgtaactccagaacatcttccatatcctatgttaggaggagcgaacaccactcttccactattgccatcgaatgccatagaataaata : 111900
Seq3 : tggatcaatgtgtgtaactccagaacatcttccatatcctatgttaggaggagcgaacaccactcttccactattgccatcgaatgccatagaataaata : 111900
Seq4 : tggatcaatgtgtgtaactccagaacatcttccatatcctatgttaggaggagcgaacaccactcttccactattgccatcgaatgccatagaataaata : 111900

                 *     111920          *     111940          *     111960          *     111980          *     112000
Seq1 : tccttggaattgatagaaatcggactgtcggatgttgtgatcatcttcataggattaacaactatgtatggtgccgcctgaagtttcatatcgtaactga : 112000
Seq2 : tccttggaattgatagaaatcggactgtcggatgttgtgatcatcttcataggattaacaactatgtatggtgccgcctgaagtttcatatcgtaactga : 112000
Seq3 : tccttggaattgatagaaatcggactgtcggatgttgtgatcatcttcataggattaacaactatgtatggtgccgcctgaagtttcatatcgtaactga : 112000
Seq4 : tccttggaattgatagaaatcggactgtcggatgttgtgatcatcttcataggattaacaactatgtatggtgccgcctgaagtttcatatcgtaactga : 112000
```

```
                 *    112020          *    112040          *    112060          *    112080          *    112100
Seq1 : tgccgtttataggtctagccacagaaaccaacgtaggtctaaatccaactatagacaaaatagaagccaatatctgttcctcatctgtcataacttgaga : 112100
Seq2 : tgccgtttataggtctagccacagaaaccaacgtaggtctaaatccaactatagacaaaatagaagccaatatctgttcctcatctgtcataacttgaga : 112100
Seq3 : tgccgtttataggtctagccacagaaaccaacgtaggtctaaatccaactatagacaaaatagaagccaatatctgttcctcatctgtcataacttgaga : 112100
Seq4 : tgccgtttataggtctagccacagaaaccaacgtaggtctaaatccaactatagacaaaatagaagccaatatctgttcctcatctgtcataacttgaga : 112100

                 *    112120          *    112140          *    112160          *    112180          *    112200
Seq1 : gcatccagtatgaataatcttcattagatggggatctaccgcatcatcatcgttacaataaaaaattcccattctaatgttcataattgcttttctaatc : 112200
Seq2 : gcatccagtatgaataatcttcattagatggggatctaccgcatcatcatcgttacaataaaaaattcccattctaatgttcataattgcttttctaatc : 112200
Seq3 : gcatccagtatgaataatcttcattagatggggatctaccgcatcatcatcgttacaataaaaaattcccattctaatgttcataattgcttttctaatc : 112200
Seq4 : gcatccagtatgaataatcttcattagatggggatctaccgcatcatcatcgttacaataaaaaattcccattctaatgttcataattgcttttctaatc : 112200

                 *    112220          *    112240          *    112260          *    112280          *    112300
Seq1 : atggtatgcatgtttgctctctgaatctctgtggaaattagatctgatacacctgtaatcactatcggattatcctccgtaagacgattaaccaacaaca : 112300
Seq2 : atggtatgcatgtttgctctctgaatctctgtggaaattagatctgatacacctgtaatcactatcggattatcctccgtaagacgattaaccaacaaca : 112300
Seq3 : atggtatgcatgtttgctctctgaatctctgtggaaattagatctgatacacctgtaatcactatcggattatcctccgtaagacgattaaccaacaaca : 112300
Seq4 : atggtatgcatgtttgctctctgaatctctgtggaaattagatctgatacacctgtaatcactatcggattatcctccgtaagacgattaaccaacaaca : 112300

                 *    112320          *    112340          *    112360          *    112380          *    112400
Seq1 : tataattataagactttacttttctaaattcataaagttgctggattaggctataggtgtctccatgtacatacgcgttctcgagcgcaggaagtttaat : 112400
Seq2 : tataattataagactttacttttctaaattcataaagttgctggattaggctataggtgtctccatgtacatacgcgttctcgagcgcaggaagtttaat : 112400
Seq3 : tataattataagactttacttttctaaattcataaagttgctggattaggctataggtgtctccatgtacatacgcgttctcgagcgcaggaagtttaat : 112400
Seq4 : tataattataagactttacttttctaaattcataaagttgctggattaggctataggtgtctccatgtacatacgcgttctcgagcgcaggaagtttaat : 112400

                 *    112420          *    112440          *    112460          *    112480          *    112500
Seq1 : accgaatagtgccatcagaataggatgaatatagtaattagtttctggttttctataaataaaagacaaatcttgtgaactagacatatcggtaaaatgc : 112500
Seq2 : accgaatagtgccatcagaataggatgaatatagtaattagtttctggttttctataaataaaagacaaatcttgtgaactagacatatcggtaaaatgc : 112500
Seq3 : accgaatagtgccatcagaataggatgaatatagtaattagtttctggttttctataaataaaagacaaatcttgtgaactagacatatcggtaaaatgc : 112500
Seq4 : accgaatagtgccatcagaataggatgaatatagtaattagtttctggttttctataaataaaagacaaatcttgtgaactagacatatcggtaaaatgc : 112500

                 *    112520          *    112540          *    112560          *    112580          *    112600
Seq1 : atggattggaatcgtgtagtcgacagaagaatatgatgattagatggagagtatatttttatctaactctttgagttggtcaccgattctaggactagctc : 112600
Seq2 : atggattggaatcgtgtagtcgacagaagaatatgatgattagatggagagtatatttttatctaactctttgagttggtcaccgattctaggactagctc : 112600
Seq3 : atggattggaatcgtgtagtcgacagaagaatatgatgattagatggagagtatatttttatctaactctttgagttggtcaccgattctaggactagctc : 112600
Seq4 : atggattggaatcgtgtagtcgacagaagaatatgatgattagatggagagtatatttttatctaactctttgagttggtcaccgattctaggactagctc : 112600

                 *    112620          *    112640          *    112660          *    112680          *    112700
Seq1 : gagaatgaataagtactaaaggatgagtacatttcacagaaacactagcattgttcaatgtgctctttacatgggtaaggagttgaaatagctcgtttct : 112700
Seq2 : gagaatgaataagtactaaaggatgagtacatttcacagaaacactagcattgttcaatgtgctctttacatgggtaaggagttgaaatagctcgtttct : 112700
Seq3 : gagaatgaataagtactaaaggatgagtacatttcacagaaacactagcattgttcaatgtgctctttacatgggtaaggagttgaaatagctcgtttct : 112700
Seq4 : gagaatgaataagtactaaaggatgagtacatttcacagaaacactagcattgttcaatgtgctctttacatgggtaaggagttgaaatagctcgtttct : 112700
```

```
            *    112720         *    112740         *    112760         *    112780         *    112800
Seq1 : atttgttctgacaatatttagtttattcataatgttaagcatatcctgaatagtaaagttagatgtgtcatacttgttagtagttagatatttagcaatt : 112800
Seq2 : atttgttctgacaatatttagtttattcataatgttaagcatatcctgaatagtaaagttagatgtgtcatacttgttagtagttagatatttagcaatt : 112800
Seq3 : atttgttctgacaatatttagtttattcataatgttaagcatatcctgaatagtaaagttagatgtgtcatacttgttagtagttagatatttagcaatt : 112800
Seq4 : atttgttctgacaatatttagtttattcataatgttaagcatatcctgaatagtaaagttagatgtgtcatacttgttagtagttagatatttagcaatt : 112800

            *    112820         *    112840         *    112860         *    112880         *    112900
Seq1 : gcattcccatcatttctcaatctcgtactccaatcatgtgtagatgctacttcgtcgatggaaaccatacaatcctttttgataggctgttgagattgat : 112900
Seq2 : gcattcccatcatttctcaatctcgtactccaatcatgtgtagatgctacttcgtcgatggaaaccatacaatcctttttgataggctgttgagattgat : 112900
Seq3 : gcattcccatcatttctcaatctcgtactccaatcatgtgtagatgctacttcgtcgatggaaaccatacaatcctttttgataggctgttgagattgat : 112900
Seq4 : gcattcccatcatttctcaatctcgtactccaatcatgtgtagatgctacttcgtcgatggaaaccatacaatcctttttgataggctgttgagattgat : 112900

            *    112920         *    112940         *    112960         *    112980         *    113000
Seq1 : tatttcctgcacgtttaggtttggtacgttgatttctagcccctgcggatataaagtcatcgtctacaatttgggacaatgaattgcatacactacaaga : 113000
Seq2 : tatttcctgcacgtttaggtttggtacgttgatttctagcccctgcggatataaagtcatcgtctacaatttgggacaatgaattgcatacactacaaga : 113000
Seq3 : tatttcctgcacgtttaggtttggtacgttgatttctagcccctgcggatataaagtcatcgtctacaatttgggacaatgaattgcatacactacaaga : 113000
Seq4 : tatttcctgcacgtttaggtttggtacgttgatttctagcccctgcggatataaagtcatcgtctacaatttgggacaatgaattgcatacactacaaga : 113000

            *    113020         *    113040         *    113060         *    113080         *    113100
Seq1 : caaagatttatcagaagtgtgaatatgatcttcatctaccaaagaaagagtttgattagtataactagatttagtcctgcgttagatgttaaaaaaaca : 113100
Seq2 : caaagatttatcagaagtgtgaatatgatcttcatctaccaaagaaagagtttgattagtataactagatttagtcctgcgttagatgttaaaaaaaca : 113100
Seq3 : caaagatttatcagaagtgtgaatatgatcttcatctaccaaagaaagagtttgattagtataactagatttagtcctgcgttagatgttaaaaaaaca : 113100
Seq4 : caaagatttatcagaagtgtgaatatgatcttcatctaccaaagaaagagtttgattagtataactagatttagtcctgcgttagatgttaaaaaaaca : 113100

            *    113120         *    113140         *    113160         *    113180         *    113200
Seq1 : tcgctattgaccacggcttccattatttatattcgtagtttttactcgaaagcgtgattttaatatccaatcttattacttttggaatcgttcaaaacct : 113200
Seq2 : tcgctattgaccacggcttccattatttatattcgtagtttttactcgaaagcgtgattttaatatccaatcttattacttttggaatcgttcaaaacct : 113200
Seq3 : tcgctattgaccacggcttccattatttatattcgtagtttttactcgaaagcgtgattttaatatccaatcttattacttttggaatcgttcaaaacct : 113200
Seq4 : tcgctattgaccacggcttccattatttatattcgtagtttttactcgaaagcgtgattttaatatccaatcttattacttttggaatcgttcaaaacct : 113200

            *    113220         *    113240         *    113260         *    113280         *    113300
Seq1 : ttgactagttgtagaatttgatctattgccctacgcgtatactcccttgcatcatatacgttcgtcaccagatcgtttgtttcggcctgaagttggtgca : 113300
Seq2 : ttgactagttgtagaatttgatctattgccctacgcgtatactcccttgcatcatatacgttcgtcaccagatcgtttgtttcggcctgaagttggtgca : 113300
Seq3 : ttgactagttgtagaatttgatctattgccctacgcgtatactcccttgcatcatatacgttcgtcaccagatcgtttgtttcggcctgaagttggtgca : 113300
Seq4 : ttgactagttgtagaatttgatctattgccctacgcgtatactcccttgcatcatatacgttcgtcaccagatcgtttgtttcggcctgaagttggtgca : 113300

            *    113320         *    113340         *    113360         *    113380         *    113400
Seq1 : tatctctttcaacattcgacatgagatccttaagggccatatcgtctagatttgttgagatgctgctcctggatttggattttgttgtgctgttgtaca : 113400
Seq2 : tatctctttcaacattcgacatgagatccttaagggccatatcgtctagatttgttgagatgctgctcctggatttggattttgttgtgctgttgtaca : 113400
Seq3 : tatctctttcaacattcgacatgagatccttaagggccatatcgtctagatttgttgagatgctgctcctggatttggattttgttgtgctgttgtaca : 113400
Seq4 : tatctctttcaacattcgacatgagatccttaagggccatatcgtctagatttgttgagatgctgctcctggatttggattttgttgtgctgttgtaca : 113400
```

```
             *    113420         *    113440         *    113460         *    113480         *    113500
Seq1 : tactgtaccaccagtaggtgtaggagtacatacagtggccacaataggaggttgaggaggtgtaaccgttggagtagtacaagaaatatttccatccgat : 113500
Seq2 : tactgtaccaccagtaggtgtaggagtacatacagtggccacaataggaggttgaggaggtgtaaccgttggagtagtacaagaaatatttccatccgat : 113500
Seq3 : tactgtaccaccagtaggtgtaggagtacatacagtggccacaataggaggttgaggaggtgtaaccgttggagtagtacaagaaatatttccatccgat : 113500
Seq4 : tactgtaccaccagtaggtgtaggagtacatacagtggccacaataggaggttgaggaggtgtaaccgttggagtagtacaagaaatatttccatccgat : 113500

             *    113520         *    113540         *    113560         *    113580         *    113600
Seq1 : tgttgtgtacatgtagttgttggtaacgtctgagaaggttgggtagatggcggtgtcgtcgtcttttgatctttattaaatttagagataatatcctgaa : 113600
Seq2 : tgttgtgtacatgtagttgttggtaacgtctgagaaggttgggtagatggcggtgtcgtcgtcttttgatctttattaaatttagagataatatcctgaa : 113600
Seq3 : tgttgtgtacatgtagttgttggtaacgtctgagaaggttgggtagatggcggtgtcgtcgtcttttgatctttattaaatttagagataatatcctgaa : 113600
Seq4 : tgttgtgtacatgtagttgttggtaacgtctgagaaggttgggtagatggcggtgtcgtcgtcttttgatctttattaaatttagagataatatcctgaa : 113600

             *    113620         *    113640         *    113660         *    113680         *    113700
Seq1 : cagcattgctcggcgtcaacgctggaaggagtgaactcgccggcgcatcagtatcttcagacagccaatcaaaaagattagacatatcagatgatgtatt : 113700
Seq2 : cagcattgctcggcgtcaacgctggaaggagtgaactcgccggcgcatcagtatcttcagacagccaatcaaaaagattagacatatcagatgatgtatt : 113700
Seq3 : cagcattgctcggcgtcaacgctggaaggagtgaactcgccggcgcatcagtatcttcagacagccaatcaaaaagattagacatatcagatgatgtatt : 113700
Seq4 : cagcattgctcggcgtcaacgctggaaggagtgaactcgccggcgcatcagtatcttcagacagccaatcaaaaagattagacatatcagatgatgtatt : 113700

             *    113720         *    113740         *    113760         *    113780         *    113800
Seq1 : agtttgttgtcgtggttttggtgtaggaacagtactactaggtagaagaataggagccggtgtagctgttggaaccggctgtggagttatatgaatagtt : 113800
Seq2 : agtttgttgtcgtggttttggtgtaggaacagtactactaggtagaagaataggagccggtgtagctgttggaaccggctgtggagttatatgaatagtt : 113800
Seq3 : agtttgttgtcgtggttttggtgtaggaacagtactactaggtagaagaataggagccggtgtagctgttggaaccggctgtggagttatatgaatagtt : 113800
Seq4 : agtttgttgtcgtggttttggtgtaggaacagtactactaggtagaagaataggagccggtgtagctgttggaaccggctgtggagttatatgaatagtt : 113800

             *    113820         *    113840         *    113860         *    113880         *    113900
Seq1 : ggttgtagcggttggataggctgtctgctggcgaccgtcatattatctctagctagttgttctcgcaactgtctttgataatacgactcttgagacttta : 113900
Seq2 : ggttgtagcggttggataggctgtctgctggcgaccgtcatattatctctagctagttgttctcgcaactgtctttgataatacgactcttgagacttta : 113900
Seq3 : ggttgtagcggttggataggctgtctgctggcgaccgtcatattatctctagctagttgttctcgcaactgtctttgataatacgactcttgagacttta : 113900
Seq4 : ggttgtagcggttggataggctgtctgctggcgaccgtcatattatctctagctagttgttctcgcaactgtctttgataatacgactcttgagacttta : 113900

             *    113920         *    113940         *    113960         *    113980         *    114000
Seq1 : gtcctatttcaatcgcttcatccttttcgtatccggatccttttcttcagaataatagattgacgactttggtgtagaggattctgccagcccctgtga : 114000
Seq2 : gtcctatttcaatcgcttcatccttttcgtatccggatccttttcttcagaataatagattgacgactttggtgtagaggattctgccagcccctgtga : 114000
Seq3 : gtcctatttcaatcgcttcatccttttcgtatccggatccttttcttcagaataatagattgacgactttggtgtagaggattctgccagcccctgtga : 114000
Seq4 : gtcctatttcaatcgcttcatccttttcgtatccggatccttttcttcagaataatagattgacgactttggtgtagaggattctgccagcccctgtga : 114000

             *    114020         *    114040         *    114060         *    114080         *    114100
Seq1 : gaacttgttaaagaagtccatttaaggctttaaaattgaattgcgattataagattaaatggcagacacagacgatattatcgactatgaatccgatgat : 114100
Seq2 : gaacttgttaaagaagtccatttaaggctttaaaattgaattgcgattataagattaaatggcagacacagacgatattatcgactatgaatccgatgat : 114100
Seq3 : gaacttgttaaagaagtccatttaaggctttaaaattgaattgcgattataagattaaatggcagacacagacgatattatcgactatgaatccgatgat : 114100
Seq4 : gaacttgttaaagaagtccatttaaggctttaaaattgaattgcgattataagattaaatggcagacacagacgatattatcgactatgaatccgatgat : 114100
```

```
                *    114120         *    114140         *    114160         *    114180         *    114200
Seq1 : ctcaccgaatacgaggatgatgaagaagaggaagaagatggagagtcactagaaactagtgatatagatcccaaatcttcttataagattgtagaatcag : 114200
Seq2 : ctcaccgaatacgaggatgatgaagaagaggaagaagatggagagtcactagaaactagtgatatagatcccaaatcttcttataagattgtagaatcag : 114200
Seq3 : ctcaccgaatacgaggatgatgaagaagaggaagaagatggagagtcactagaaactagtgatatagatcccaaatcttcttataagattgtagaatcag : 114200
Seq4 : ctcaccgaatacgaggatgatgaagaagaggaagaagatggagagtcactagaaactagtgatatagatcccaaatcttcttataagattgtagaatcag : 114200

                *    114220         *    114240         *    114260         *    114280         *    114300
Seq1 : catccactcatatagaagatgcgcattccaatcttaaacatatagggaatcatatatctgctcttaaacgacgctatactagacgtataagtctatttga : 114300
Seq2 : catccactcatatagaagatgcgcattccaatcttaaacatatagggaatcatatatctgctcttaaacgacgctatactagacgtataagtctatttga : 114300
Seq3 : catccactcatatagaagatgcgcattccaatcttaaacatatagggaatcatatatctgctcttaaacgacgctatactagacgtataagtctatttga : 114300
Seq4 : catccactcatatagaagatgcgcattccaatcttaaacatatagggaatcatatatctgctcttaaacgacgctatactagacgtataagtctatttga : 114300

                *    114320         *    114340         *    114360         *    114380         *    114400
Seq1 : aatagcgggtataatagcagaaagctataacttgcttcaacgaggaagattacctctagtttcagaattttctgacgaaacgatgaagcaaaatatgcta : 114400
Seq2 : aatagcgggtataatagcagaaagctataacttgcttcaacgaggaagattacctctagtttcagaattttctgacgaaacgatgaagcaaaatatgcta : 114400
Seq3 : aatagcgggtataatagcagaaagctataacttgcttcaacgaggaagattacctctagtttcagaattttctgacgaaacgatgaagcaaaatatgcta : 114400
Seq4 : aatagcgggtataatagcagaaagctataacttgcttcaacgaggaagattacctctagtttcagaattttctgacgaaacgatgaagcaaaatatgcta : 114400

                *    114420         *    114440         *    114460         *    114480         *    114500
Seq1 : catgtaattatacaagagatagaggagggttcttgtcctatagtcatcgaaaagaacggagaattgttgtcggtaaacgattttgacaaagatggtctaa : 114500
Seq2 : catgtaattatacaagagatagaggagggttcttgtcctatagtcatcgaaaagaacggagaattgttgtcggtaaacgattttgacaaagatggtctaa : 114500
Seq3 : catgtaattatacaagagatagaggagggttcttgtcctatagtcatcgaaaagaacggagaattgttgtcggtaaacgattttgacaaagatggtctaa : 114500
Seq4 : catgtaattatacaagagatagaggagggttcttgtcctatagtcatcgaaaagaacggagaattgttgtcggtaaacgattttgacaaagatggtctaa : 114500

                *    114520         *    114540         *    114560         *    114580         *    114600
Seq1 : aattccatctagactatattatcaaaatttggaaacttcaaaaacgatattagaatttatacgaatatcgttctctaaatgtcacaatcaagtctcgcat : 114600
Seq2 : aattccatctagactatattatcaaaatttggaaacttcaaaaacgatattagaatttatacgaatatcgttctctaaatgtcacaatcaagtctcgcat : 114600
Seq3 : aattccatctagactatattatcaaaatttggaaacttcaaaaacgatattagaatttatacgaatatcgttctctaaatgtcacaatcaagtctcgcat : 114600
Seq4 : aattccatctagactatattatcaaaatttggaaacttcaaaaacgatattagaatttatacgaatatcgttctctaaatgtcacaatcaagtctcgcat : 114600

                *    114620         *    114640         *    114660         *    114680         *    114700
Seq1 : gttcagcaatttattgtcgtactttatatcgtgttcattaacgatatcttgcaaaatagtaatgattctatcttccttcgatagatattcttcagagatt : 114700
Seq2 : gttcagcaatttattgtcgtactttatatcgtgttcattaacgatatcttgcaaaatagtaatgattctatcttccttcgatagatattcttcagagatt : 114700
Seq3 : gttcagcaatttattgtcgtactttatatcgtgttcattaacgatatcttgcaaaatagtaatgattctatcttccttcgatagatattcttcagagatt : 114700
Seq4 : gttcagcaatttattgtcgtactttatatcgtgttcattaacgatatcttgcaaaatagtaatgattctatcttccttcgatagatattcttcagagatt : 114700

                *    114720         *    114740         *    114760         *    114780         *    114800
Seq1 : attgtcttatattttttcttgttatccgatatgaatttgataagactttgaacattattgatacccgtctgtttaatttttttctacagatattttagttt : 114800
Seq2 : attgtcttatattttttcttgttatccgatatgaatttgataagactttgaacattattgatacccgtctgtttaatttttttctacagatattttagttt : 114800
Seq3 : attgtcttatattttttcttgttatccgatatgaatttgataagactttgaacattattgatacccgtctgtttaatttttttctacagatattttagttt : 114800
Seq4 : attgtcttatattttttcttgttatccgatatgaatttgataagactttgaacattattgatacccgtctgtttaatttttttctacagatattttagttt : 114800
```

```
              *    114820          *    114840          *    114860          *    114880          *    114900
Seq1 : tggcagattctatcgtatctgtcaatagacatccaacatcgacattcgacgtcaattgtctataaatcaacgtataaattttagaaataacattagcgaa : 114900
Seq2 : tggcagattctatcgtatctgtcaatagacatccaacatcgacattcgacgtcaattgtctataaatcaacgtataaattttagaaataacattagcgaa : 114900
Seq3 : tggcagattctatcgtatctgtcaatagacatccaacatcgacattcgacgtcaattgtctataaatcaacgtataaattttagaaataacattagcgaa : 114900
Seq4 : tggcagattctatcgtatctgtcaatagacatccaacatcgacattcgacgtcaattgtctataaatcaacgtataaattttagaaataacattagcgaa : 114900

              *    114920          *    114940          *    114960          *    114980          *    115000
Seq1 : ttgttgtgcattgatgtcgttattctgaaacagtatgattttaggtagcattttcttaacaaagagaacgtatttattgttactcagttgaacagatgat : 115000
Seq2 : ttgttgtgcattgatgtcgttattctgaaacagtatgattttaggtagcattttcttaacaaagagaacgtatttattgttactcagttgaacagatgat : 115000
Seq3 : ttgttgtgcattgatgtcgttattctgaaacagtatgattttaggtagcattttcttaacaaagagaacgtatttattgttactcagttgaacagatgat : 115000
Seq4 : ttgttgtgcattgatgtcgttattctgaaacagtatgattttaggtagcattttcttaacaaagagaacgtatttattgttactcagttgaacagatgat : 115000

              *    115020          *    115040          *    115060          *    115080          *    115100
Seq1 : atatccagattactaacgcatctgattccgtataccaaactttcagaagaaatggtgtacaattgtttgtattcattcaatgtctcttttcagaaatta : 115100
Seq2 : atatccagattactaacgcatctgattccgtataccaaactttcagaagaaatggtgtacaattgtttgtattcattcaatgtctcttttcagaaatta : 115100
Seq3 : atatccagattactaacgcatctgattccgtataccaaactttcagaagaaatggtgtacaattgtttgtattcattcaatgtctcttttcagaaatta : 115100
Seq4 : atatccagattactaacgcatctgattccgtataccaaactttcagaagaaatggtgtacaattgtttgtattcattcaatgtctcttttcagaaatta : 115100

              *    115120          *    115140          *    115160          *    115180          *    115200
Seq1 : gtttagagtcgaatactgcaataattttcaagagatagttttcatcagataagattttatttagtgtagatatgataaaactattgttttgttggagaac : 115200
Seq2 : gtttagagtcgaatactgcaataattttcaagagatagttttcatcagataagattttatttagtgtagatatgataaaactattgttttgttggagaac : 115200
Seq3 : gtttagagtcgaatactgcaataattttcaagagatagttttcatcagataagattttatttagtgtagatatgataaaactattgttttgttggagaac : 115200
Seq4 : gtttagagtcgaatactgcaataattttcaagagatagttttcatcagataagattttatttagtgtagatatgataaaactattgttttgttggagaac : 115200

              *    115220          *    115240          *    115260          *    115280          *    115300
Seq1 : ttgatacgccgcgttctctgtagtcgacgctctcaaatgggaaacaatctccattatttttttggaatcggatactatatcttcggtatcttgacgcagt : 115300
Seq2 : ttgatacgccgcgttctctgtagtcgacgctctcaaatgggaaacaatctccattatttttttggaatcggatactatatcttcggtatcttgacgcagt : 115300
Seq3 : ttgatacgccgcgttctctgtagtcgacgctctcaaatgggaaacaatctccattatttttttggaatcggatactatatcttcggtatcttgacgcagt : 115300
Seq4 : ttgatacgccgcgttctctgtagtcgacgctctcaaatgggaaacaatctccattatttttttggaatcggatactatatcttcggtatcttgacgcagt : 115300

              *    115320          *    115340          *    115360          *    115380          *    115400
Seq1 : ctagtatacatagagttaagagagattagagtttgtacattaagcaacatgtctctaaatgtggctacaaacttttcctttttcacataatctagtttat : 115400
Seq2 : ctagtatacatagagttaagagagattagagtttgtacattaagcaacatgtctctaaatgtggctacaaacttttcctttttcacataatctagtttat : 115400
Seq3 : ctagtatacatagagttaagagagattagagtttgtacattaagcaacatgtctctaaatgtggctacaaacttttcctttttcacataatctagtttat : 115400
Seq4 : ctagtatacatagagttaagagagattagagtttgtacattaagcaacatgtctctaaatgtggctacaaacttttcctttttcacataatctagtttat : 115400

              *    115420          *    115440          *    115460          *    115480          *    115500
Seq1 : tatataccgatttcacaacggcaccagatttaaggaaccagaatgaaaaactctgataactacaatatttcatcatagttacgattttatcatcttctat : 115500
Seq2 : tatataccgatttcacaacggcaccagatttaaggaaccagaatgaaaaactctgataactacaatatttcatcatagttacgattttatcatcttctat : 115500
Seq3 : tatataccgatttcacaacggcaccagatttaaggaaccagaatgaaaaactctgataactacaatatttcatcatagttacgattttatcatcttctat : 115500
Seq4 : tatataccgatttcacaacggcaccagatttaaggaaccagaatgaaaaactctgataactacaatatttcatcatagttacgattttatcatcttctat : 115500
```

```
                *    115520         *    115540         *    115560         *    115580         *    115600
Seq1 : agttggtgtaatagcgcatacctttttctccaagactggaaccaacgtcataaaaatgtttaaatcaaaatccatatcaacatctgatgcgctaagacca : 115600
Seq2 : agttggtgtaatagcgcatacctttttctccaagactggaaccaacgtcataaaaatgtttaaatcaaaatccatatcaacatctgatgcgctaagacca : 115600
Seq3 : agttggtgtaatagcgcatacctttttctccaagactggaaccaacgtcataaaaatgtttaaatcaaaatccatatcaacatctgatgcgctaagacca : 115600
Seq4 : agttggtgtaatagcgcatacctttttctccaagactggaaccaacgtcataaaaatgtttaaatcaaaatccatatcaacatctgatgcgctaagacca : 115600

                *    115620         *    115640         *    115660         *    115680         *    115700
Seq1 : gtctcgcgttcaagattatctttactaatggtgacgaactcatcatatagaactctaagtttgtccattatttatttacagatttagttgtttaatttat : 115700
Seq2 : gtctcgcgttcaagattatctttactaatggtgacgaactcatcatatagaactctaagtttgtccattatttatttacagatttagttgtttaatttat : 115700
Seq3 : gtctcgcgttcaagattatctttactaatggtgacgaactcatcatatagaactctaagtttgtccattatttatttacagatttagttgtttaatttat : 115700
Seq4 : gtctcgcgttcaagattatctttactaatggtgacgaactcatcatatagaactctaagtttgtccattatttatttacagatttagttgtttaatttat : 115700

                *    115720         *    115740         *    115760         *    115780         *    115800
Seq1 : ttgtgctcttccagagttgggatagtatttttctaacgtcggtattatattattaggatctacgttcatatgtatcataatattaatcatccacgttttg : 115800
Seq2 : ttgtgctcttccagagttgggatagtatttttctaacgtcggtattatattattaggatctacgttcatatgtatcataatattaatcatccacgttttg : 115800
Seq3 : ttgtgctcttccagagttgggatagtatttttctaacgtcggtattatattattaggatctacgttcatatgtatcataatattaatcatccacgttttg : 115800
Seq4 : ttgtgctcttccagagttgggatagtatttttctaacgtcggtattatattattaggatctacgttcatatgtatcataatattaatcatccacgttttg : 115800

                *    115820         *    115840         *    115860         *    115880         *    115900
Seq1 : ataaatctatctttagcttctgaaataacgtatttaaacaaaggagaaaaatatttagctacggcatcagacgcaataacatttttttgtaaatgtaacgt : 115900
Seq2 : ataaatctatctttagcttctgaaataacgtatttaaacaaaggagaaaaatatttagctacggcatcagacgcaataacatttttttgtaaatgtaacgt : 115900
Seq3 : ataaatctatctttagcttctgaaataacgtatttaaacaaaggagaaaaatatttagctacggcatcagacgcaataacatttttttgtaaatgtaacgt : 115900
Seq4 : ataaatctatctttagcttctgaaataacgtatttaaacaaaggagaaaaatatttagctacggcatcagacgcaataacatttttttgtaaatgtaacgt : 115900

                *    115920         *    115940         *    115960         *    115980         *    116000
Seq1 : atttagacgacagatcttcgttaaaaagttttccatctatgtagaatccatcggttgttaacaccattcccgcgtcagattgaataggagtttgaatagt : 116000
Seq2 : atttagacgacagatcttcgttaaaaagttttccatctatgtagaatccatcggttgttaacaccattcccgcgtcagattgaataggagtttgaatagt : 116000
Seq3 : atttagacgacagatcttcgttaaaaagttttccatctatgtagaatccatcggttgttaacaccattcccgcgtcagattgaataggagtttgaatagt : 116000
Seq4 : atttagacgacagatcttcgttaaaaagttttccatctatgtagaatccatcggttgttaacaccattcccgcgtcagattgaataggagtttgaatagt : 116000

                *    116020         *    116040         *    116060         *    116080         *    116100
Seq1 : ttgttttggaaatagatccttcaataacttatagttgggtgggaaaaaaatcgattttatcactagactctttcttttttactatcattacctcatgaact : 116100
Seq2 : ttgttttggaaatagatccttcaataacttatagttgggtgggaaaaaaatcgattttatcactagactctttcttttttactatcattacctcatgaact : 116100
Seq3 : ttgttttggaaatagatccttcaataacttatagttgggtgggaaaaaaatcgattttatcactagactctttcttttttactatcattacctcatgaact : 116100
Seq4 : ttgttttggaaatagatccttcaataacttatagttgggtgggaaaaaaatcgattttatcactagactctttcttttttactatcattacctcatgaact : 116100

                *    116120         *    116140         *    116160         *    116180         *    116200
Seq1 : atttcttgaatgagtatatgtattttctttcctatatcggacgcgttcattggaaaatataccatgtcgttaactataagaatatttttatcctcgttta : 116200
Seq2 : atttcttgaatgagtatatgtattttctttcctatatcggacgcgttcattggaaaatataccatgtcgttaactataagaatatttttatcctcgttta : 116200
Seq3 : atttcttgaatgagtatatgtattttctttcctatatcggacgcgttcattggaaaatataccatgtcgttaactataagaatatttttatcctcgttta : 116200
Seq4 : atttcttgaatgagtatatgtattttctttcctatatcggacgcgttcattggaaaatataccatgtcgttaactataagaatatttttatcctcgttta : 116200
```

```
                *    116220         *    116240         *    116260         *    116280         *    116300
Seq1 : caaactgaataatatcagatgtagttcgtaaacgaactatatcatcaccagcacaacatctaactatatgatatccactagtttcctttagccgtttatt : 116300
Seq2 : caaactgaataatatcagatgtagttcgtaaacgaactatatcatcaccagcacaacatctaactatatgatatccactagtttcctttagccgtttatt : 116300
Seq3 : caaactgaataatatcagatgtagttcgtaaacgaactatatcatcaccagcacaacatctaactatatgatatccactagtttcctttagccgtttatt : 116300
Seq4 : caaactgaataatatcagatgtagttcgtaaacgaactatatcatcaccagcacaacatctaactatatgatatccactagtttcctttagccgtttatt : 116300

                *    116320         *    116340         *    116360         *    116380         *    116400
Seq1 : atcttgttccatattagcagtcattccatcatttaagaaggcgtcaaagataatagggagaaatgacattttggattctgttacgactttaccaaaatta : 116400
Seq2 : atcttgttccatattagcagtcattccatcatttaagaaggcgtcaaagataatagggagaaatgacattttggattctgttacgactttaccaaaatta : 116400
Seq3 : atcttgttccatattagcagtcattccatcatttaagaaggcgtcaaagataatagggagaaatgacattttggattctgttacgactttaccaaaatta : 116400
Seq4 : atcttgttccatattagcagtcattccatcatttaagaaggcgtcaaagataatagggagaaatgacattttggattctgttacgactttaccaaaatta : 116400

                *    116420         *    116440         *    116460         *    116480         *    116500
Seq1 : aggatatacggacttactatctttttctcaacgtcgatttgatgaacacacgatgaaaatgtgcttctatgagattgatcatgtagaaaacaacaaggga : 116500
Seq2 : aggatatacggacttactatctttttctcaacgtcgatttgatgaacacacgatgaaaatgtgcttctatgagattgatcatgtagaaaacaacaaggga : 116500
Seq3 : aggatatacggacttactatctttttctcaacgtcgatttgatgaacacacgatgaaaatgtgcttctatgagattgatcatgtagaaaacaacaaggga : 116500
Seq4 : aggatatacggacttactatctttttctcaacgtcgatttgatgaacacacgatgaaaatgtgcttctatgagattgatcatgtagaaaacaacaaggga : 116500

                *    116520         *    116540         *    116560         *    116580         *    116600
Seq1 : tacaatatttccgcatatcatgaaatatattaagaaatcccaccttattatatttccccaaaggatccatgcatgtaaacattatgccgttatcattaat : 116600
Seq2 : tacaatatttccgcatatcatgaaatatattaagaaatcccaccttattatatttccccaaaggatccatgcatgtaaacattatgccgttatcattaat : 116600
Seq3 : tacaatatttccgcatatcatgaaatatattaagaaatcccaccttattatatttccccaaaggatccatgcatgtaaacattatgccgttatcattaat : 116600
Seq4 : tacaatatttccgcatatcatgaaatatattaagaaatcccaccttattatatttccccaaaggatccatgcatgtaaacattatgccgttatcattaat : 116600

                *    116620         *    116640         *    116660         *    116680         *    116700
Seq1 : aaagacttctttctcatcggatttgtaaaagttgttactgatttttttcattccaggatctagataattaataatgatgggttttctattcttattcttt : 116700
Seq2 : aaagacttctttctcatcggatttgtaaaagttgttactgatttttttcattccaggatctagataattaataatgatgggttttctattcttattcttt : 116700
Seq3 : aaagacttctttctcatcggatttgtaaaagttgttactgatttttttcattccaggatctagataattaataatgatgggttttctattcttattcttt : 116700
Seq4 : aaagacttctttctcatcggatttgtaaaagttgttactgatttttttcattccaggatctagataattaataatgatgggttttctattcttattcttt : 116700

                *    116720         *    116740         *    116760         *    116780         *    116800
Seq1 : gtattttggcatatcctagaccagtaaacagtttccactttggtaaaatcagcagacttttgaacgctattaaacatggcattaatggcaataactaaaa : 116800
Seq2 : gtattttggcatatcctagaccagtaaacagtttccactttggtaaaatcagcagacttttgaacgctattaaacatggcattaatggcaataactaaaa : 116800
Seq3 : gtattttggcatatcctagaccagtaaacagtttccactttggtaaaatcagcagacttttgaacgctattaaacatggcattaatggcaataactaaaa : 116800
Seq4 : gtattttggcatatcctagaccagtaaacagtttccactttggtaaaatcagcagacttttgaacgctattaaacatggcattaatggcaataactaaaa : 116800

                *    116820         *    116840         *    116860         *    116880         *    116900
Seq1 : atgtaaaatattttttctatgttaggaatatggtttttcactttaatagatatatggtttttggccaaaatgatagatattttttttatccgaggatagtaa : 116900
Seq2 : atgtaaaatattttttctatgttaggaatatggtttttcactttaatagatatatggtttttggccaaaatgatagatattttttttatccgaggatagtaa : 116900
Seq3 : atgtaaaatattttttctatgttaggaatatggtttttcactttaatagatatatggtttttggccaaaatgatagatattttttttatccgaggatagtaa : 116900
Seq4 : atgtaaaatattttttctatgttaggaatatggtttttcactttaatagatatatggtttttggccaaaatgatagatattttttttatccgaggatagtaa : 116900
```

```
             *    116920         *    116940         *    116960         *    116980         *    117000
Seq1 : aatattattagtcgccgtctctataaaaatgaagctagtctcgatatccaattttattctagaattgataggagtcgccaaatgtaccttatacgttata : 117000
Seq2 : aatattattagtcgccgtctctataaaaatgaagctagtctcgatatccaattttattctagaattgataggagtcgccaaatgtaccttatacgttata : 117000
Seq3 : aatattattagtcgccgtctctataaaaatgaagctagtctcgatatccaattttattctagaattgataggagtcgccaaatgtaccttatacgttata : 117000
Seq4 : aatattattagtcgccgtctctataaaaatgaagctagtctcgatatccaattttattctagaattgataggagtcgccaaatgtaccttatacgttata : 117000

             *    117020         *    117040         *    117060         *    117080         *    117100
Seq1 : tctcccttgatgcgttccatttgtgtatctatatcggacacaagatctgtaaatagttttacgttattaatcatcacggtatcgccgtcgctagataacg : 117100
Seq2 : tctcccttgatgcgttccatttgtgtatctatatcggacacaagatctgtaaatagttttacgttattaatcatcacggtatcgccgtcgctagataacg : 117100
Seq3 : tctcccttgatgcgttccatttgtgtatctatatcggacacaagatctgtaaatagttttacgttattaatcatcacggtatcgccgtcgctagataacg : 117100
Seq4 : tctcccttgatgcgttccatttgtgtatctatatcggacacaagatctgtaaatagttttacgttattaatcatcacggtatcgccgtcgctagataacg : 117100

             *    117120         *    117140         *    117160         *    117180         *    117200
Seq1 : ctaatgtaccatccaagtcccaaatggagagatttaactgttcatcgtttagaataaaatgattaccggtcatattaataaagtgttcatcgtatctaga : 117200
Seq2 : ctaatgtaccatccaagtcccaaatggagagatttaactgttcatcgtttagaataaaatgattaccggtcatattaataaagtgttcatcgtatctaga : 117200
Seq3 : ctaatgtaccatccaagtcccaaatggagagatttaactgttcatcgtttagaataaaatgattaccggtcatattaataaagtgttcatcgtatctaga : 117200
Seq4 : ctaatgtaccatccaagtcccaaatggagagatttaactgttcatcgtttagaataaaatgattaccggtcatattaataaagtgttcatcgtatctaga : 117200

             *    117220         *    117240         *    117260         *    117280         *    117300
Seq1 : taacaacgacttataattaatgtccaagtcttgaactcgctgaatgatctttttttaacccagttagttttagattggtacgaaatatattgttaaacttt : 117300
Seq2 : taacaacgacttataattaatgtccaagtcttgaactcgctgaatgatctttttttaacccagttagttttagattggtacgaaatatattgttaaacttt : 117300
Seq3 : taacaacgacttataattaatgtccaagtcttgaactcgctgaatgatctttttttaacccagttagttttagattggtacgaaatatattgttaaacttt : 117300
Seq4 : taacaacgacttataattaatgtccaagtcttgaactcgctgaatgatctttttttaacccagttagttttagattggtacgaaatatattgttaaacttt : 117300

             *    117320         *    117340         *    117360         *    117380         *    117400
Seq1 : gattctacagtaatgtccaaatctagttgtggaaatacttccatcaacattgtttcaaacttgataatattattatctacatcttcatacgatccaaatt : 117400
Seq2 : gattctacagtaatgtccaaatctagttgtggaaatacttccatcaacattgtttcaaacttgataatattattatctacatcttcatacgatccaaatt : 117400
Seq3 : gattctacagtaatgtccaaatctagttgtggaaatacttccatcaacattgtttcaaacttgataatattattatctacatcttcatacgatccaaatt : 117400
Seq4 : gattctacagtaatgtccaaatctagttgtggaaatacttccatcaacattgtttcaaacttgataatattattatctacatcttcatacgatccaaatt : 117400

             *    117420         *    117440         *    117460         *    117480         *    117500
Seq1 : ccggaatagatgtatcacatgctcttgccacccagataaccaaaaagtcacacgctccaggatatacattgtataaaaagctatcgtttttttagtagtgt : 117500
Seq2 : ccggaatagatgtatcacatgctcttgccacccagataaccaaaaagtcacacgctccaggatatacattgtataaaaagctatcgtttttttagtagtgt : 117500
Seq3 : ccggaatagatgtatcacatgctcttgccacccagataaccaaaaagtcacacgctccaggatatacattgtataaaaagctatcgtttttttagtagtgt : 117500
Seq4 : ccggaatagatgtatcacatgctcttgccacccagataaccaaaaagtcacacgctccaggatatacattgtataaaaagctatcgtttttttagtagtgt : 117500

             *    117520         *    117540         *    117560         *    117580         *    117600
Seq1 : ttttttctgagtatatacgaagggattaaaaatagtattatcaacgtaactatattccaaattattcttatgagaatagataataatatcgtccttaata : 117600
Seq2 : ttttttctgagtatatacgaagggattaaaaatagtattatcaacgtaactatattccaaattattcttatgagaatagataataatatcgtccttaata : 117600
Seq3 : ttttttctgagtatatacgaagggattaaaaatagtattatcaacgtaactatattccaaattattcttatgagaatagataataatatcgtccttaata : 117600
Seq4 : ttttttctgagtatatacgaagggattaaaaatagtattatcaacgtaactatattccaaattattcttatgagaatagataataatatcgtccttaata : 117600
```

```
              *    117620         *    117640         *    117660         *    117680         *    117700
Seq1 : tctaacaaatttcctaaatatccctttaattgagtcattcgaagcgtcaatagaatatgtctcttaactatttccggctgttgtatatttaaatgacttc : 117700
Seq2 : tctaacaaatttcctaaatatccctttaattgagtcattcgaagcgtcaatagaatatgtctcttaactatttccggctgttgtatatttaaatgacttc : 117700
Seq3 : tctaacaaatttcctaaatatccctttaattgagtcattcgaagcgtcaatagaatatgtctcttaactatttccggctgttgtatatttaaatgacttc : 117700
Seq4 : tctaacaaatttcctaaatatccctttaattgagtcattcgaagcgtcaatagaatatgtctcttaactatttccggctgttgtatatttaaatgacttc : 117700

              *    117720         *    117740         *    117760         *    117780         *    117800
Seq1 : gtaaaaaataatatatatggcgacttctcatctatgtaatcatatggagtgagatatagggctcgttctacctcctgccccttacccacctgtaataccaa : 117800
Seq2 : gtaaaaaataatatatatggcgacttctcatctatgtaatcatatggagtgagatatagggctcgttctacctcctgccccttacccacctgtaataccaa : 117800
Seq3 : gtaaaaaataatatatatggcgacttctcatctatgtaatcatatggagtgagatatagggctcgttctacctcctgccccttacccacctgtaataccaa : 117800
Seq4 : gtaaaaaataatatatatggcgacttctcatctatgtaatcatatggagtgagatatagggctcgttctacctcctgccccttacccacctgtaataccaa : 117800

              *    117820         *    117840         *    117860         *    117880         *    117900
Seq1 : ttgcggacttactatatatcgcatatttatatcgtggggtaaagtgaaaatctactaccgatgatgtaagtcttacaatgttcgaaccagtaccagatct : 117900
Seq2 : ttgcggacttactatatatcgcatatttatatcgtggggtaaagtgaaaatctactaccgatgatgtaagtcttacaatgttcgaaccagtaccagatct : 117900
Seq3 : ttgcggacttactatatatcgcatatttatatcgtggggtaaagtgaaaatctactaccgatgatgtaagtcttacaatgttcgaaccagtaccagatct : 117900
Seq4 : ttgcggacttactatatatcgcatatttatatcgtggggtaaagtgaaaatctactaccgatgatgtaagtcttacaatgttcgaaccagtaccagatct : 117900

              *    117920         *    117940         *    117960         *    117980         *    118000
Seq1 : taatttggaggcctccgtagaactaggggaggtaaatatagatcaaacaacacctatgataaaggaaaatagcggttttatatcccgtagtagacgtcta : 118000
Seq2 : taatttggaggcctccgtagaactaggggaggtaaatatagatcaaacaacacctatgataaaggaaaatagcggttttatatcccgtagtagacgtcta : 118000
Seq3 : taatttggaggcctccgtagaactaggggaggtaaatatagatcaaacaacacctatgataaaggaaaatagcggttttatatcccgtagtagacgtcta : 118000
Seq4 : taatttggaggcctccgtagaactaggggaggtaaatatagatcaaacaacacctatgataaaggaaaatagcggttttatatcccgtagtagacgtcta : 118000

              *    118020         *    118040         *    118060         *    118080         *    118100
Seq1 : ttcgcccatagatctaaggatgatgagagaaaactagcactacgattctttttacaaagactttattttttagatcatagagagattcattatttgttca : 118100
Seq2 : ttcgcccatagatctaaggatgatgagagaaaactagcactacgattctttttacaaagactttattttttagatcatagagagattcattatttgttca : 118100
Seq3 : ttcgcccatagatctaaggatgatgagagaaaactagcactacgattctttttacaaagactttattttttagatcatagagagattcattatttgttca : 118100
Seq4 : ttcgcccatagatctaaggatgatgagagaaaactagcactacgattctttttacaaagactttattttttagatcatagagagattcattatttgttca : 118100

              *    118120         *    118140         *    118160         *    118180         *    118200
Seq1 : gatgcgttgacgctgtaaaagacgtcactattaccaaaaaaaataacattatcgtggcgccttatatagcacttttaactatcgcatcaaaaggatgcaa : 118200
Seq2 : gatgcgttgacgctgtaaaagacgtcactattaccaaaaaaaataacattatcgtggcgccttatatagcacttttaactatcgcatcaaaaggatgcaa : 118200
Seq3 : gatgcgttgacgctgtaaaagacgtcactattaccaaaaaaaataacattatcgtggcgccttatatagcacttttaactatcgcatcaaaaggatgcaa : 118200
Seq4 : gatgcgttgacgctgtaaaagacgtcactattaccaaaaaaaataacattatcgtggcgccttatatagcacttttaactatcgcatcaaaaggatgcaa : 118200

              *    118220         *    118240         *    118260         *    118280         *    118300
Seq1 : acttacagaaacaatgattgaagcatttttccagaactatataatgaacatagtaagaaatttaaattcaactctcaagtatccatcatccaagaaaaa : 118300
Seq2 : acttacagaaacaatgattgaagcatttttccagaactatataatgaacatagtaagaaatttaaattcaactctcaagtatccatcatccaagaaaaa : 118300
Seq3 : acttacagaaacaatgattgaagcatttttccagaactatataatgaacatagtaagaaatttaaattcaactctcaagtatccatcatccaagaaaaa : 118300
Seq4 : acttacagaaacaatgattgaagcatttttccagaactatataatgaacatagtaagaaatttaaattcaactctcaagtatccatcatccaagaaaaa : 118300
```

```
                *    118320          *    118340          *    118360          *    118380          *    118400
Seq1 : ctcggataccagtttggaaactatcacgtttatgattttgaaccgtattactctacagtagctctggctattcgagatgaacattcatctggcattttta : 118400
Seq2 : ctcggataccagtttggaaactatcacgtttatgattttgaaccgtattactctacagtagctctggctattcgagatgaacattcatctggcattttta : 118400
Seq3 : ctcggataccagtttggaaactatcacgtttatgattttgaaccgtattactctacagtagctctggctattcgagatgaacattcatctggcattttta : 118400
Seq4 : ctcggataccagtttggaaactatcacgtttatgattttgaaccgtattactctacagtagctctggctattcgagatgaacattcatctggcattttta : 118400

                *    118420          *    118440          *    118460          *    118480          *    118500
Seq1 : atatccgtcaagagagttatctggtaagttcattatctgaaataacatatagattttatctaattaatctaaaatctgatcttgttcaatggagtgctag : 118500
Seq2 : atatccgtcaagagagttatctggtaagttcattatctgaaataacatatagattttatctaattaatctaaaatctgatcttgttcaatggagtgctag : 118500
Seq3 : atatccgtcaagagagttatctggtaagttcattatctgaaataacatatagattttatctaattaatctaaaatctgatcttgttcaatggagtgctag : 118500
Seq4 : atatccgtcaagagagttatctggtaagttcattatctgaaataacatatagattttatctaattaatctaaaatctgatcttgttcaatggagtgctag : 118500

                *    118520          *    118540          *    118560          *    118580          *    118600
Seq1 : tacgggcgctgtaattaatcaaatggtaaatactgtattgattacagtgtatgaaaagttacaactggtcatagaaaatgattcacaatttacatgttca : 118600
Seq2 : tacgggcgctgtaattaatcaaatggtaaatactgtattgattacagtgtatgaaaagttacaactggtcatagaaaatgattcacaatttacatgttca : 118600
Seq3 : tacgggcgctgtaattaatcaaatggtaaatactgtattgattacagtgtatgaaaagttacaactggtcatagaaaatgattcacaatttacatgttca : 118600
Seq4 : tacgggcgctgtaattaatcaaatggtaaatactgtattgattacagtgtatgaaaagttacaactggtcatagaaaatgattcacaatttacatgttca : 118600

                *    118620          *    118640          *    118660          *    118680          *    118700
Seq1 : ttggctgtggaatcaaaacttccaataaaattacttaaagatagaaatgaattatttacaaaattcattaacgagttaaaaaagaccagttcattcaaga : 118700
Seq2 : ttggctgtggaatcaaaacttccaataaaattacttaaagatagaaatgaattatttacaaaattcattaacgagttaaaaaagaccagttcattcaaga : 118700
Seq3 : ttggctgtggaatcaaaacttccaataaaattacttaaagatagaaatgaattatttacaaaattcattaacgagttaaaaaagaccagttcattcaaga : 118700
Seq4 : ttggctgtggaatcaaaacttccaataaaattacttaaagatagaaatgaattatttacaaaattcattaacgagttaaaaaagaccagttcattcaaga : 118700

                *    118720          *    118740          *    118760          *    118780          *    118800
Seq1 : taagcaaacgcgataaggatacgctactaaaatattttacttaggactggagttagaatttatagacgactcatttcgtttatcattgttactattatta : 118800
Seq2 : taagcaaacgcgataaggatacgctactaaaatattttacttaggactggagttagaatttatagacgactcatttcgtttatcattgttactattatta : 118800
Seq3 : taagcaaacgcgataaggatacgctactaaaatattttacttaggactggagttagaatttatagacgactcatttcgtttatcattgttactattatta : 118800
Seq4 : taagcaaacgcgataaggatacgctactaaaatattttacttaggactggagttagaatttatagacgactcatttcgtttatcattgttactattatta : 118800

                *    118820          *    118840          *    118860          *    118880          *    118900
Seq1 : ctattactatcattattagtgttggcattattagtattcttcttgtcatcttgttcagaaatatacagcaatgctatacctaatactaaatacattatca : 118900
Seq2 : ctattactatcattattagtgttggcattattagtattcttcttgtcatcttgttcagaaatatacagcaatgctatacctaatactaaatacattatca : 118900
Seq3 : ctattactatcattattagtgttggcattattagtattcttcttgtcatcttgttcagaaatatacagcaatgctatacctaatactaaatacattatca : 118900
Seq4 : ctattactatcattattagtgttggcattattagtattcttcttgtcatcttgttcagaaatatacagcaatgctatacctaatactaaatacattatca : 118900

                *    118920          *    118940          *    118960          *    118980          *    119000
Seq1 : tgctcgcaatggctctaacaacaacgaaccaaaatgaatttggtcgtagcttttgttcacaaaaatacataaagaaatgtctacataaatctatggcgcc : 119000
Seq2 : tgctcgcaatggctctaacaacaacgaaccaaaatgaatttggtcgtagcttttgttcacaaaaatacataaagaaatgtctacataaatctatggcgcc : 119000
Seq3 : tgctcgcaatggctctaacaacaacgaaccaaaatgaatttggtcgtagcttttgttcacaaaaatacataaagaaatgtctacataaatctatggcgcc : 119000
Seq4 : tgctcgcaatggctctaacaacaacgaaccaaaatgaatttggtcgtagcttttgttcacaaaaatacataaagaaatgtctacataaatctatggcgcc : 119000
```

```
              *    119020         *    119040         *    119060         *    119080         *    119100
Seq1 : attggctacttgaaatagcgccagtcctcctacagattttaatatagctgtataacatgacatttattcatcatcaaaagagacagagtcaccatctgtc : 119100
Seq2 : attggctacttgaaatagcgccagtcctcctacagattttaatatagctgtataacatgacatttattcatcatcaaaagagacagagtcaccatctgtc : 119100
Seq3 : attggctacttgaaatagcgccagtcctcctacagattttaatatagctgtataacatgacatttattcatcatcaaaagagacagagtcaccatctgtc : 119100
Seq4 : attggctacttgaaatagcgccagtcctcctacagattttaatatagctgtataacatgacatttattcatcatcaaaagagacagagtcaccatctgtc : 119100

              *    119120         *    119140         *    119160         *    119180         *    119200
Seq1 : atatttagattttttttcatgtgttcaaagtatcctctactcatttcattataatagtttatcatacttagaattttaggacggatcaatgagtaagact : 119200
Seq2 : atatttagattttttttcatgtgttcaaagtatcctctactcatttcattataatagtttatcatacttagaattttaggacggatcaatgagtaagact : 119200
Seq3 : atatttagattttttttcatgtgttcaaagtatcctctactcatttcattataatagtttatcatacttagaattttaggacggatcaatgagtaagact : 119200
Seq4 : atatttagattttttttcatgtgttcaaagtatcctctactcatttcattataatagtttatcatacttagaattttaggacggatcaatgagtaagact : 119200

              *    119220         *    119240         *    119260         *    119280         *    119300
Seq1 : tgactagatcgtcagtagtaatttgtgcatcgtctattctgcatccgcttcgtcgaataatgtatagcatcgctttgagattctccatagctatcaagtc : 119300
Seq2 : tgactagatcgtcagtagtaatttgtgcatcgtctattctgcatccgcttcgtcgaataatgtatagcatcgctttgagattctccatagctatcaagtc : 119300
Seq3 : tgactagatcgtcagtagtaatttgtgcatcgtctattctgcatccgcttcgtcgaataatgtatagcatcgctttgagattctccatagctatcaagtc : 119300
Seq4 : tgactagatcgtcagtagtaatttgtgcatcgtctattctgcatccgcttcgtcgaataatgtatagcatcgctttgagattctccatagctatcaagtc : 119300

              *    119320         *    119340         *    119360         *    119380         *    119400
Seq1 : tttatacaatgacatggaaatatctgtgaatactttatacttctccaacatcgatgccttaacatcatcgcctactttagcattgaaaatacgttctatt : 119400
Seq2 : tttatacaatgacatggaaatatctgtgaatactttatacttctccaacatcgatgccttaacatcatcgcctactttagcattgaaaatacgttctatt : 119400
Seq3 : tttatacaatgacatggaaatatctgtgaatactttatacttctccaacatcgatgccttaacatcatcgcctactttagcattgaaaatacgttctatt : 119400
Seq4 : tttatacaatgacatggaaatatctgtgaatactttatacttctccaacatcgatgccttaacatcatcgcctactttagcattgaaaatacgttctatt : 119400

              *    119420         *    119440         *    119460         *    119480         *    119500
Seq1 : gtgtagatggatgtagcaagatttttaaacaacaatgccatcttacacgatgattgcctcaagtctccaatcttttgtttagaacgattagctacagagt : 119500
Seq2 : gtgtagatggatgtagcaagatttttaaacaacaatgccatcttacacgatgattgcctcaagtctccaatcttttgtttagaacgattagctacagagt : 119500
Seq3 : gtgtagatggatgtagcaagatttttaaacaacaatgccatcttacacgatgattgcctcaagtctccaatcttttgtttagaacgattagctacagagt : 119500
Seq4 : gtgtagatggatgtagcaagatttttaaacaacaatgccatcttacacgatgattgcctcaagtctccaatcttttgtttagaacgattagctacagagt : 119500

              *    119520         *    119540         *    119560         *    119580         *    119600
Seq1 : tcaacgcttggctgactagcatattattatctttagaaattgtattcttcaatgaggcgtttatcatatctgtgatttcgttagtcatattacagtctga : 119600
Seq2 : tcaacgcttggctgactagcatattattatctttagaaattgtattcttcaatgaggcgtttatcatatctgtgatttcgttagtcatattacagtctga : 119600
Seq3 : tcaacgcttggctgactagcatattattatctttagaaattgtattcttcaatgaggcgtttatcatatctgtgatttcgttagtcatattacagtctga : 119600
Seq4 : tcaacgcttggctgactagcatattattatctttagaaattgtattcttcaatgaggcgtttatcatatctgtgatttcgttagtcatattacagtctga : 119600

              *    119620         *    119640         *    119660         *    119680         *    119700
Seq1 : ctgggttgtaatgttatccaacatatcacctatggatacggtacacgtaccagcatttgtaataatcctatctaagatgttgtatggcattgcgcagaaa : 119700
Seq2 : ctgggttgtaatgttatccaacatatcacctatggatacggtacacgtaccagcatttgtaataatcctatctaagatgttgtatggcattgcgcagaaa : 119700
Seq3 : ctgggttgtaatgttatccaacatatcacctatggatacggtacacgtaccagcatttgtaataatcctatctaagatgttgtatggcattgcgcagaaa : 119700
Seq4 : ctgggttgtaatgttatccaacatatcacctatggatacggtacacgtaccagcatttgtaataatcctatctaagatgttgtatggcattgcgcagaaa : 119700
```

```
                *    119720         *    119740         *    119760         *    119780         *    119800
Seq1 : atatcttctcctgtaatatctccactctcgataaatctactcagattattcttaaatgccttattctctggagaaaagatatcagtgtccatcatttcat : 119800
Seq2 : atatcttctcctgtaatatctccactctcgataaatctactcagattattcttaaatgccttattctctggagaaaagatatcagtgtccatcatttcat : 119800
Seq3 : atatcttctcctgtaatatctccactctcgataaatctactcagattattcttaaatgccttattctctggagaaaagatatcagtgtccatcatttcat : 119800
Seq4 : atatcttctcctgtaatatctccactctcgataaatctactcagattattcttaaatgccttattctctggagaaaagatatcagtgtccatcatttcat : 119800

                *    119820         *    119840         *    119860         *    119880         *    119900
Seq1 : taatagtatacgcagaaaagataccacgagtatcaattctatccaagatacttatcggttccgagtcacagataatggtttcctctccttcgggagatcc : 119900
Seq2 : taatagtatacgcagaaaagataccacgagtatcaattctatccaagatacttatcggttccgagtcacagataatggtttcctctccttcgggagatcc : 119900
Seq3 : taatagtatacgcagaaaagataccacgagtatcaattctatccaagatacttatcggttccgagtcacagataatggtttcctctccttcgggagatcc : 119900
Seq4 : taatagtatacgcagaaaagataccacgagtatcaattctatccaagatacttatcggttccgagtcacagataatggtttcctctccttcgggagatcc : 119900

                *    119920         *    119940         *    119960         *    119980         *    120000
Seq1 : tgcatagaaatatctaggacaatagtttctatactgtctgtaactctgataatctctaaagtcactaactgataccatgaaattgagaagatcaaacgct : 120000
Seq2 : tgcatagaaatatctaggacaatagtttctatactgtctgtaactctgataatctctaaagtcactaactgataccatgaaattgagaagatcaaacgct : 120000
Seq3 : tgcatagaaatatctaggacaatagtttctatactgtctgtaactctgataatctctaaagtcactaactgataccatgaaattgagaagatcaaacgct : 120000
Seq4 : tgcatagaaatatctaggacaatagtttctatactgtctgtaactctgataatctctaaagtcactaactgataccatgaaattgagaagatcaaacgct : 120000

                *    120020         *    120040         *    120060         *    120080         *    120100
Seq1 : gaagtaatcaatttttctgcctcgtttttactacaactagttttcatcaatgtagcgacgatgtattgtttagttactcttggtctaatactgatgatag : 120100
Seq2 : gaagtaatcaatttttctgcctcgtttttactacaactagttttcatcaatgtagcgacgatgtattgtttagttactcttggtctaatactgatgatag : 120100
Seq3 : gaagtaatcaatttttctgcctcgtttttactacaactagttttcatcaatgtagcgacgatgtattgtttagttactcttggtctaatactgatgatag : 120100
Seq4 : gaagtaatcaatttttctgcctcgtttttactacaactagttttcatcaatgtagcgacgatgtattgtttagttactcttggtctaatactgatgatag : 120100

                *    120120         *    120140         *    120160         *    120180         *    120200
Seq1 : agatattattgcttcccataatggatcttctagtagtcaccttaaagcccattgatgcgaatagcagatagataaagtcttggtatgactcctttctaat : 120200
Seq2 : agatattattgcttcccataatggatcttctagtagtcaccttaaagcccattgatgcgaatagcagatagataaagtcttggtatgactcctttctaat : 120200
Seq3 : agatattattgcttcccataatggatcttctagtagtcaccttaaagcccattgatgcgaatagcagatagataaagtcttggtatgactcctttctaat : 120200
Seq4 : agatattattgcttcccataatggatcttctagtagtcaccttaaagcccattgatgcgaatagcagatagataaagtcttggtatgactcctttctaat : 120200

                *    120220         *    120240         *    120260         *    120280         *    120300
Seq1 : atagtacggactacctttgtcacccaactttatacccacataagccataacaacctctttaatagccgtttcatgaggtttatcagccatgagcctgagt : 120300
Seq2 : atagtacggactacctttgtcacccaactttatacccacataagccataacaacctctttaatagccgtttcatgaggtttatcagccatgagcctgagt : 120300
Seq3 : atagtacggactacctttgtcacccaactttatacccacataagccataacaacctctttaatagccgtttcatgaggtttatcagccatgagcctgagt : 120300
Seq4 : atagtacggactacctttgtcacccaactttatacccacataagccataacaacctctttaatagccgtttcatgaggtttatcagccatgagcctgagt : 120300

                *    120320         *    120340         *    120360         *    120380         *    120400
Seq1 : agttggaagaatctcatgaatcccgtctcagaaagtcctatatgcatgatagatttatctttcctgggaaactctcgtatagtcatagatgaaatactct : 120400
Seq2 : agttggaagaatctcatgaatcccgtctcagaaagtcctatatgcatgatagatttatctttcctgggaaactctcgtatagtcatagatgaaatactct : 120400
Seq3 : agttggaagaatctcatgaatcccgtctcagaaagtcctatatgcatgatagatttatctttcctgggaaactctcgtatagtcatagatgaaatactct : 120400
Seq4 : agttggaagaatctcatgaatcccgtctcagaaagtcctatatgcatgatagatttatctttcctgggaaactctcgtatagtcatagatgaaatactct : 120400
```

```
           *    120420          *    120440          *    120460          *    120480          *    120500
Seq1 : tcaaagtttctgaaataagattagtaacagtcttacctccgactactctgggtaacaaacatactctaataggtgttttctctgcggagataatatcaga : 120500
Seq2 : tcaaagtttctgaaataagattagtaacagtcttacctccgactactctgggtaacaaacatactctaataggtgttttctctgcggagataatatcaga : 120500
Seq3 : tcaaagtttctgaaataagattagtaacagtcttacctccgactactctgggtaacaaacatactctaataggtgttttctctgcggagataatatcaga : 120500
Seq4 : tcaaagtttctgaaataagattagtaacagtcttacctccgactactctgggtaacaaacatactctaataggtgttttctctgcggagataatatcaga : 120500

           *    120520          *    120540          *    120560          *    120580          *    120600
Seq1 : aaggatagagcaataagtagtattattgtgattataaagaccgaatacataacaggtagaatttataaacatcatgtcctgaaggtttttagacttgtat : 120600
Seq2 : aaggatagagcaataagtagtattattgtgattataaagaccgaatacataacaggtagaatttataaacatcatgtcctgaaggtttttagacttgtat : 120600
Seq3 : aaggatagagcaataagtagtattattgtgattataaagaccgaatacataacaggtagaatttataaacatcatgtcctgaaggtttttagacttgtat : 120600
Seq4 : aaggatagagcaataagtagtattattgtgattataaagaccgaatacataacaggtagaatttataaacatcatgtcctgaaggtttttagacttgtat : 120600

           *    120620          *    120640          *    120660          *    120680          *    120700
Seq1 : tcctcgtaatccataccgtcccaaaacatggatttggtaactttgatagccgtagatctttgttccttcgccaacaggttaaagaaattaataaagaatt : 120700
Seq2 : tcctcgtaatccataccgtcccaaaacatggatttggtaactttgatagccgtagatctttgttccttcgccaacaggttaaagaaattaataaagaatt : 120700
Seq3 : tcctcgtaatccataccgtcccaaaacatggatttggtaactttgatagccgtagatctttgttccttcgccaacaggttaaagaaattaataaagaatt : 120700
Seq4 : tcctcgtaatccataccgtcccaaaacatggatttggtaactttgatagccgtagatctttgttccttcgccaacaggttaaagaaattaataaagaatt : 120700

           *    120720          *    120740          *    120760          *    120780          *    120800
Seq1 : tgttgtttctatttatgtccacaaattgcacgtttggaagcgccacggttacattcactgcagcattttgaggatcgcgagtatgaagtacgatgttatt : 120800
Seq2 : tgttgtttctatttatgtccacaaattgcacgtttggaagcgccacggttacattcactgcagcattttgaggatcgcgagtatgaagtacgatgttatt : 120800
Seq3 : tgttgtttctatttatgtccacaaattgcacgtttggaagcgccacggttacattcactgcagcattttgaggatcgcgagtatgaagtacgatgttatt : 120800
Seq4 : tgttgtttctatttatgtccacaaattgcacgtttggaagcgccacggttacattcactgcagcattttgaggatcgcgagtatgaagtacgatgttatt : 120800

           *    120820          *    120840          *    120860          *    120880          *    120900
Seq1 : gtttactggtatatctggaaagaaatctaccagtctaggaataagagattgatatcgcatagaaatagtaaagtttataatctcatcatcgaagagcatt : 120900
Seq2 : gtttactggtatatctggaaagaaatctaccagtctaggaataagagattgatatcgcatagaaatagtaaagtttataatctcatcatcgaagagcatt : 120900
Seq3 : gtttactggtatatctggaaagaaatctaccagtctaggaataagagattgatatcgcatagaaatagtaaagtttataatctcatcatcgaagagcatt : 120900
Seq4 : gtttactggtatatctggaaagaaatctaccagtctaggaataagagattgatatcgcatagaaatagtaaagtttataatctcatcatcgaagagcatt : 120900

           *    120920          *    120940          *    120960          *    120980          *    121000
Seq1 : ttgttaccattgtaataaatatccactctgtcatatgtataaatgaagtactgttcaaacatgatgagatgtttatatgttggcatagtagtgagatcga : 121000
Seq2 : ttgttaccattgtaataaatatccactctgtcatatgtataaatgaagtactgttcaaacatgatgagatgtttatatgttggcatagtagtgagatcga : 121000
Seq3 : ttgttaccattgtaataaatatccactctgtcatatgtataaatgaagtactgttcaaacatgatgagatgtttatatgttggcatagtagtgagatcga : 121000
Seq4 : ttgttaccattgtaataaatatccactctgtcatatgtataaatgaagtactgttcaaacatgatgagatgtttatatgttggcatagtagtgagatcga : 121000

           *    121020          *    121040          *    121060          *    121080          *    121100
Seq1 : cgtttggtaatggcaatgtattaagattaactccataatgtctagcagcatctgcgatgttataagcgtcgtcaaagcggggtcgatcttgtattgttat : 121100
Seq2 : cgtttggtaatggcaatgtattaagattaactccataatgtctagcagcatctgcgatgttataagcgtcgtcaaagcggggtcgatcttgtattgttat : 121100
Seq3 : cgtttggtaatggcaatgtattaagattaactccataatgtctagcagcatctgcgatgttataagcgtcgtcaaagcggggtcgatcttgtattgttat : 121100
Seq4 : cgtttggtaatggcaatgtattaagattaactccataatgtctagcagcatctgcgatgttataagcgtcgtcaaagcggggtcgatcttgtattgttat : 121100
```

```
             *    121120         *    121140         *    121160         *    121180         *    121200
Seq1 : atattgtctaacacctataagattatcaaaatcttgtctgcttaatacaccgttaacaattttttgccttgaattcttttattggtgcattaataacatcc : 121200
Seq2 : atattgtctaacacctataagattatcaaaatcttgtctgcttaatacaccgttaacaattttttgccttgaattcttttattggtgcattaataacatcc : 121200
Seq3 : atattgtctaacacctataagattatcaaaatcttgtctgcttaatacaccgttaacaattttttgccttgaattcttttattggtgcattaataacatcc : 121200
Seq4 : atattgtctaacacctataagattatcaaaatcttgtctgcttaatacaccgttaacaattttttgccttgaattcttttattggtgcattaataacatcc : 121200

             *    121220         *    121240         *    121260         *    121280         *    121300
Seq1 : ttatagaggatgttaaacaaataagtgttatcaaagttaagatctggatatttcttttctgctagaacatccattgagtcggagccatctggtttaatat : 121300
Seq2 : ttatagaggatgttaaacaaataagtgttatcaaagttaagatctggatatttcttttctgctagaacatccattgagtcggagccatctggtttaatat : 121300
Seq3 : ttatagaggatgttaaacaaataagtgttatcaaagttaagatctggatatttcttttctgctagaacatccattgagtcggagccatctggtttaatat : 121300
Seq4 : ttatagaggatgttaaacaaataagtgttatcaaagttaagatctggatatttcttttctgctagaacatccattgagtcggagccatctggtttaatat : 121300

             *    121320         *    121340         *    121360         *    121380         *    121400
Seq1 : aaccaccgataaatctagctctgtattctgtatccgtcaatctaatattaagaaggtgttgagtgaaaggtggaagatcgtaaaagctgtgagtattaat : 121400
Seq2 : aaccaccgataaatctagctctgtattctgtatccgtcaatctaatattaagaaggtgttgagtgaaaggtggaagatcgtaaaagctgtgagtattaat : 121400
Seq3 : aaccaccgataaatctagctctgtattctgtatccgtcaatctaatattaagaaggtgttgagtgaaaggtggaagatcgtaaaagctgtgagtattaat : 121400
Seq4 : aaccaccgataaatctagctctgtattctgtatccgtcaatctaatattaagaaggtgttgagtgaaaggtggaagatcgtaaaagctgtgagtattaat : 121400

             *    121420         *    121440         *    121460         *    121480         *    121500
Seq1 : gataggattagtttccgaactaatgttaattggggtattaataatatctatatttccagcgttaagtgtaacattaaacagttttaattcacgtgacgtg : 121500
Seq2 : gataggattagtttccgaactaatgttaattggggtattaataatatctatatttccagcgttaagtgtaacattaaacagttttaattcacgtgacgtg : 121500
Seq3 : gataggattagtttccgaactaatgttaattggggtattaataatatctatatttccagcgttaagtgtaacattaaacagttttaattcacgtgacgtg : 121500
Seq4 : gataggattagtttccgaactaatgttaattggggtattaataatatctatatttccagcgttaagtgtaacattaaacagttttaattcacgtgacgtg : 121500

             *    121520         *    121540         *    121560         *    121580         *    121600
Seq1 : gtatcaattaaataattaatgcccaatttggatatagcagcctgaagctcatcttgtttagttacggatcctaatgagttattaagcaatatatcgaacg : 121600
Seq2 : gtatcaattaaataattaatgcccaatttggatatagcagcctgaagctcatcttgtttagttacggatcctaatgagttattaagcaatatatcgaacg : 121600
Seq3 : gtatcaattaaataattaatgcccaatttggatatagcagcctgaagctcatcttgtttagttacggatcctaatgagttattaagcaatatatcgaacg : 121600
Seq4 : gtatcaattaaataattaatgcccaatttggatatagcagcctgaagctcatcttgtttagttacggatcctaatgagttattaagcaatatatcgaacg : 121600

             *    121620         *    121640         *    121660         *    121680         *    121700
Seq1 : gatgaacgaaggttgttttaagttggtcacatactttgtaatctagacatagatgcggaagaacggtagaaactatacgaaataaatattcagagtcctc : 121700
Seq2 : gatgaacgaaggttgttttaagttggtcacatactttgtaatctagacatagatgcggaagaacggtagaaactatacgaaataaatattcagagtcctc : 121700
Seq3 : gatgaacgaaggttgttttaagttggtcacatactttgtaatctagacatagatgcggaagaacggtagaaactatacgaaataaatattcagagtcctc : 121700
Seq4 : gatgaacgaaggttgttttaagttggtcacatactttgtaatctagacatagatgcggaagaacggtagaaactatacgaaataaatattcagagtcctc : 121700

             *    121720         *    121740         *    121760         *    121780         *    121800
Seq1 : taattgatcaagagtaactattgacttaataggcatcatttatttagtattaaatgacgaccgtaccagtgacggatatacaaaacgatttaattacaga : 121800
Seq2 : taattgatcaagagtaactattgacttaataggcatcatttatttagtattaaatgacgaccgtaccagtgacggatatacaaaacgatttaattacaga : 121800
Seq3 : taattgatcaagagtaactattgacttaataggcatcatttatttagtattaaatgacgaccgtaccagtgacggatatacaaaacgatttaattacaga : 121800
Seq4 : taattgatcaagagtaactattgacttaataggcatcatttatttagtattaaatgacgaccgtaccagtgacggatatacaaaacgatttaattacaga : 121800
```

```
                 *    121820         *    121840         *    121860         *    121880         *    121900
Seq1 : gttttcagaagataattatccatctaacaaaaattatgaaataactcttcgtcaaatgtctattctaactcacgttaacaacgtggtagatagagaacat : 121900
Seq2 : gttttcagaagataattatccatctaacaaaaattatgaaataactcttcgtcaaatgtctattctaactcacgttaacaacgtggtagatagagaacat : 121900
Seq3 : gttttcagaagataattatccatctaacaaaaattatgaaataactcttcgtcaaatgtctattctaactcacgttaacaacgtggtagatagagaacat : 121900
Seq4 : gttttcagaagataattatccatctaacaaaaattatgaaataactcttcgtcaaatgtctattctaactcacgttaacaacgtggtagatagagaacat : 121900

                 *    121920         *    121940         *    121960         *    121980         *    122000
Seq1 : aatgccgccgtagtgtcatctccagaggaaatatcctcacaacttaatgaagatctatttccagatgatgattcaccggccactattatcgaacgagtac : 122000
Seq2 : aatgccgccgtagtgtcatctccagaggaaatatcctcacaacttaatgaagatctatttccagatgatgattcaccggccactattatcgaacgagtac : 122000
Seq3 : aatgccgccgtagtgtcatctccagaggaaatatcctcacaacttaatgaagatctatttccagatgatgattcaccggccactattatcgaacgagtac : 122000
Seq4 : aatgccgccgtagtgtcatctccagaggaaatatcctcacaacttaatgaagatctatttccagatgatgattcaccggccactattatcgaacgagtac : 122000

                 *    122020         *    122040         *    122060         *    122080         *    122100
Seq1 : aacctcatactactattattgacgatactccacctcctactttcgtagagagttattgatatcggaacaacgtcaacaacgagaaaaaaagatttaatat : 122100
Seq2 : aacctcatactactattattgacgatactccacctcctactttcgtagagagttattgatatcggaacaacgtcaacaacgagaaaaaaagatttaatat : 122100
Seq3 : aacctcatactactattattgacgatactccacctcctactttcgtagagagttattgatatcggaacaacgtcaacaacgagaaaaaaagatttaatat : 122100
Seq4 : aacctcatactactattattgacgatactccacctcctactttcgtagagagttattgatatcggaacaacgtcaacaacgagaaaaaaagatttaatat : 122100

                 *    122120         *    122140         *    122160         *    122180         *    122200
Seq1 : tacagtatcgaaaaatgctgaagcaataatggaatctagatctatgataacttctatgccaacacaaacaccatccttgggagtagtttatgataaagat : 122200
Seq2 : tacagtatcgaaaaatgctgaagcaataatggaatctagatctatgataacttctatgccaacacaaacaccatccttgggagtagtttatgataaagat : 122200
Seq3 : tacagtatcgaaaaatgctgaagcaataatggaatctagatctatgataacttctatgccaacacaaacaccatccttgggagtagtttatgataaagat : 122200
Seq4 : tacagtatcgaaaaatgctgaagcaataatggaatctagatctatgataacttctatgccaacacaaacaccatccttgggagtagtttatgataaagat : 122200

                 *    122220         *    122240         *    122260         *    122280         *    122300
Seq1 : aaaagaattcagatgttggaggatgaagtggttaatcttagaaatcaacgatctaatacaaaatcatctgataatttagataattttaccaaaatactat : 122300
Seq2 : aaaagaattcagatgttggaggatgaagtggttaatcttagaaatcaacgatctaatacaaaatcatctgataatttagataattttaccaaaatactat : 122300
Seq3 : aaaagaattcagatgttggaggatgaagtggttaatcttagaaatcaacgatctaatacaaaatcatctgataatttagataattttaccaaaatactat : 122300
Seq4 : aaaagaattcagatgttggaggatgaagtggttaatcttagaaatcaacgatctaatacaaaatcatctgataatttagataattttaccaaaatactat : 122300

                 *    122320         *    122340         *    122360         *    122380         *    122400
Seq1 : ttggtaagactccgtataaatcaacagaagttaataagcgtatagccatcgttaattatgcaaatttgaacgggtctcccttatcagtcgaggacttgga : 122400
Seq2 : ttggtaagactccgtataaatcaacagaagttaataagcgtatagccatcgttaattatgcaaatttgaacgggtctcccttatcagtcgaggacttgga : 122400
Seq3 : ttggtaagactccgtataaatcaacagaagttaataagcgtatagccatcgttaattatgcaaatttgaacgggtctcccttatcagtcgaggacttgga : 122400
Seq4 : ttggtaagactccgtataaatcaacagaagttaataagcgtatagccatcgttaattatgcaaatttgaacgggtctcccttatcagtcgaggacttgga : 122400

                 *    122420         *    122440         *    122460         *    122480         *    122500
Seq1 : tgtttgttcagaggatgaaatagatagaatctataaaacgattaaacaatatcacgaaagtagaaaacaaaaaattatcgtcactaacgtgattattatt : 122500
Seq2 : tgtttgttcagaggatgaaatagatagaatctataaaacgattaaacaatatcacgaaagtagaaaacaaaaaattatcgtcactaacgtgattattatt : 122500
Seq3 : tgtttgttcagaggatgaaatagatagaatctataaaacgattaaacaatatcacgaaagtagaaaacaaaaaattatcgtcactaacgtgattattatt : 122500
Seq4 : tgtttgttcagaggatgaaatagatagaatctataaaacgattaaacaatatcacgaaagtagaaaacaaaaaattatcgtcactaacgtgattattatt : 122500
```

```
              *    122520         *    122540         *    122560         *    122580         *    122600
Seq1 : gtcataaacattatcgagcaagcattgctaaaactcggatttgaagaaatcaaaggactgagtaccgatatcacttcagaaattatcgatgtggagatcg : 122600
Seq2 : gtcataaacattatcgagcaagcattgctaaaactcggatttgaagaaatcaaaggactgagtaccgatatcacttcagaaattatcgatgtggagatcg : 122600
Seq3 : gtcataaacattatcgagcaagcattgctaaaactcggatttgaagaaatcaaaggactgagtaccgatatcacttcagaaattatcgatgtggagatcg : 122600
Seq4 : gtcataaacattatcgagcaagcattgctaaaactcggatttgaagaaatcaaaggactgagtaccgatatcacttcagaaattatcgatgtggagatcg : 122600

              *    122620         *    122640         *    122660         *    122680         *    122700
Seq1 : gagatgactgcgatgctgtagcatcaaaactaggaatcggtaacagtccggttcttaatattgtattgtttatactcaagatattcgttaaacgaattaa : 122700
Seq2 : gagatgactgcgatgctgtagcatcaaaactaggaatcggtaacagtccggttcttaatattgtattgtttatactcaagatattcgttaaacgaattaa : 122700
Seq3 : gagatgactgcgatgctgtagcatcaaaactaggaatcggtaacagtccggttcttaatattgtattgtttatactcaagatattcgttaaacgaattaa : 122700
Seq4 : gagatgactgcgatgctgtagcatcaaaactaggaatcggtaacagtccggttcttaatattgtattgtttatactcaagatattcgttaaacgaattaa : 122700

              *    122720         *    122740         *    122760         *    122780         *    122800
Seq1 : aattatttaatttaatacattcccatatccagacaacaatcgtctggattaatctgttcctgtcgtctcataccggacgacatattaatctttttattag : 122800
Seq2 : aattatttaatttaatacattcccatatccagacaacaatcgtctggattaatctgttcctgtcgtctcataccggacgacatattaatctttttattag : 122800
Seq3 : aattatttaatttaatacattcccatatccagacaacaatcgtctggattaatctgttcctgtcgtctcataccggacgacatattaatctttttattag : 122800
Seq4 : aattatttaatttaatacattcccatatccagacaacaatcgtctggattaatctgttcctgtcgtctcataccggacgacatattaatctttttattag : 122800

              *    122820         *    122840         *    122860         *    122880         *    122900
Seq1 : taggcatctttttagatggtttctttttcccagcattaactgagtcgatacctagaagatcgtgattgatctctccgaccattccacgaacttctaattg : 122900
Seq2 : taggcatctttttagatggtttctttttcccagcattaactgagtcgatacctagaagatcgtgattgatctctccgaccattccacgaacttctaattg : 122900
Seq3 : taggcatctttttagatggtttctttttcccagcattaactgagtcgatacctagaagatcgtgattgatctctccgaccattccacgaacttctaattg : 122900
Seq4 : taggcatctttttagatggtttctttttcccagcattaactgagtcgatacctagaagatcgtgattgatctctccgaccattccacgaacttctaattg : 122900

              *    122920         *    122940         *    122960         *    122980         *    123000
Seq1 : gccgtctctaacggtaccataaactattttaccagcattagtaacagcttggacaatctgaccatccatcgcattgtacgatgtagtagtaactgttgtt : 123000
Seq2 : gccgtctctaacggtaccataaactattttaccagcattagtaacagcttggacaatctgaccatccatcgcattgtacgatgtagtagtaactgttgtt : 123000
Seq3 : gccgtctctaacggtaccataaactattttaccagcattagtaacagcttggacaatctgaccatccatcgcattgtacgatgtagtagtaactgttgtt : 123000
Seq4 : gccgtctctaacggtaccataaactattttaccagcattagtaacagcttggacaatctgaccatccatcgcattgtacgatgtagtagtaactgttgtt : 123000

              *    123020         *    123040         *    123060         *    123080         *    123100
Seq1 : ctacgtctaggagcaccagaagtatttttggagcccttggaggctgatgtagaagaagacgaggattttgattttggtttacatgtaatacattttgaac : 123100
Seq2 : ctacgtctaggagcaccagaagtatttttggagcccttggaggctgatgtagaagaagacgaggattttgattttggtttacatgtaatacattttgaac : 123100
Seq3 : ctacgtctaggagcaccagaagtatttttggagcccttggaggctgatgtagaagaagacgaggattttgattttggtttacatgtaatacattttgaac : 123100
Seq4 : ctacgtctaggagcaccagaagtatttttggagcccttggaggctgatgtagaagaagacgaggattttgattttggtttacatgtaatacattttgaac : 123100

              *    123120         *    123140         *    123160         *    123180         *    123200
Seq1 : tctttgattttgtatcacatgcgccggcagtcacatctgtttgagaattaagattattgttgcctcctttgacggctgcatctccaccgatttgcgctag : 123200
Seq2 : tctttgattttgtatcacatgcgccggcagtcacatctgtttgagaattaagattattgttgcctcctttgacggctgcatctccaccgatttgcgctag : 123200
Seq3 : tctttgattttgtatcacatgcgccggcagtcacatctgtttgagaattaagattattgttgcctcctttgacggctgcatctccaccgatttgcgctag : 123200
Seq4 : tctttgattttgtatcacatgcgccggcagtcacatctgtttgagaattaagattattgttgcctcctttgacggctgcatctccaccgatttgcgctag : 123200
```

```
         *    123220         *    123240         *    123260         *    123280         *    123300
Seq1 : tagattttaagctgtggtgtaatcttattaactgtttcgatataatcatcgtaactgcttctaacggctaaatttttttatccgccatttagaagcta : 123300
Seq2 : tagattttaagctgtggtgtaatcttattaactgtttcgatataatcatcgtaactgcttctaacggctaaatttttttatccgccatttagaagcta : 123300
Seq3 : tagattttaagctgtggtgtaatcttattaactgtttcgatataatcatcgtaactgcttctaacggctaaatttttttatccgccatttagaagcta : 123300
Seq4 : tagattttaagctgtggtgtaatcttattaactgtttcgatataatcatcgtaactgcttctaacggctaaatttttttatccgccatttagaagcta : 123300

         *    123320         *    123340         *    123360         *    123380         *    123400
Seq1 : aaaatattttatttatacagaagatttaactagattatacaatgaactaatatgatccttttccagattatttacaaacttggtatttttggttctgg : 123400
Seq2 : aaaatattttatttatacagaagatttaactagattatacaatgaactaatatgatccttttccagattatttacaaacttggtatttttggttctgg : 123400
Seq3 : aaaatattttatttatacagaagatttaactagattatacaatgaactaatatgatccttttccagattatttacaaacttggtatttttggttctgg : 123400
Seq4 : aaaatattttatttatacagaagatttaactagattatacaatgaactaatatgatccttttccagattatttacaaacttggtatttttggttctgg : 123400

         *    123420         *    123440         *    123460         *    123480         *    123500
Seq1 : aggaggcgaatttaaattcggacttggattcggattttgtgagttcttgatcttattatacatcgagtataggatggcgacggtaactgctacacaaata : 123500
Seq2 : aggaggcgaatttaaattcggacttggattcggattttgtgagttcttgatcttattatacatcgagtataggatggcgacggtaactgctacacaaata : 123500
Seq3 : aggaggcgaatttaaattcggacttggattcggattttgtgagttcttgatcttattatacatcgagtataggatggcgacggtaactgctacacaaata : 123500
Seq4 : aggaggcgaatttaaattcggacttggattcggattttgtgagttcttgatcttattatacatcgagtataggatggcgacggtaactgctacacaaata : 123500

         *    123520         *    123540         *    123560         *    123580         *    123600
Seq1 : ccgatcaacaaaagaataccaatcatttattgacaataacttcactattgatcaagtatgcaatatatcatcttttcactaaataagtagtaataatgat : 123600
Seq2 : ccgatcaacaaaagaataccaatcatttattgacaataacttcactattgatcaagtatgcaatatatcatcttttcactaaataagtagtaataatgat : 123600
Seq3 : ccgatcaacaaaagaataccaatcatttattgacaataacttcactattgatcaagtatgcaatatatcatcttttcactaaataagtagtaataatgat : 123600
Seq4 : ccgatcaacaaaagaataccaatcatttattgacaataacttcactattgatcaagtatgcaatatatcatcttttcactaaataagtagtaataatgat : 123600

         *    123620         *    123640         *    123660         *    123680         *    123700
Seq1 : tcaacaatgtcgagatatatggacgataataatttagttcatggaaatatcgctatgattggtatgaatgactccgctaactctgtggggcgcgcagtgc : 123700
Seq2 : tcaacaatgtcgagatatatggacgataataatttagttcatggaaatatcgctatgattggtatgaatgactccgctaactctgtggggcgcgcagtgc : 123700
Seq3 : tcaacaatgtcgagatatatggacgataataatttagttcatggaaatatcgctatgattggtatgaatgactccgctaactctgtggggcgcgcagtgc : 123700
Seq4 : tcaacaatgtcgagatatatggacgataataatttagttcatggaaatatcgctatgattggtatgaatgactccgctaactctgtggggcgcgcagtgc : 123700

         *    123720         *    123740         *    123760         *    123780         *    123800
Seq1 : tttccccacatagaataaattagcattccgactgtgataataataccaagtataaacgccataatactcaatactttccatgtacgagtgggactggtag : 123800
Seq2 : tttccccacatagaataaattagcattccgactgtgataataataccaagtataaacgccataatactcaatactttccatgtacgagtgggactggtag : 123800
Seq3 : tttccccacatagaataaattagcattccgactgtgataataataccaagtataaacgccataatactcaatactttccatgtacgagtgggactggtag : 123800
Seq4 : tttccccacatagaataaattagcattccgactgtgataataataccaagtataaacgccataatactcaatactttccatgtacgagtgggactggtag : 123800

         *    123820         *    123840         *    123860         *    123880         *    123900
Seq1 : acttactaaagtcaataaaggcgaagatacacgaaagaatcaaaagaatgattccagcgattagcacgccggaaaaataatttccaatcataagcatcat : 123900
Seq2 : acttactaaagtcaataaaggcgaagatacacgaaagaatcaaaagaatgattccagcgattagcacgccggaaaaataatttccaatcataagcatcat : 123900
Seq3 : acttactaaagtcaataaaggcgaagatacacgaaagaatcaaaagaatgattccagcgattagcacgccggaaaaataatttccaatcataagcatcat : 123900
Seq4 : acttactaaagtcaataaaggcgaagatacacgaaagaatcaaaagaatgattccagcgattagcacgccggaaaaataatttccaatcataagcatcat : 123900
```

```
               *    123920         *    123940         *    123960         *    123980         *    124000
Seq1 : gtccatttaactaataaaaattttaaatcgccgaatgaacaaagtggaatataaaccatataaaaacaatagtttgtactgcaaaaataatatctatttt : 124000
Seq2 : gtccatttaactaataaaaattttaaatcgccgaatgaacaaagtggaatataaaccatataaaaacaatagtttgtactgcaaaaataatatctatttt : 124000
Seq3 : gtccatttaactaataaaaattttaaatcgccgaatgaacaaagtggaatataaaccatataaaaacaatagtttgtactgcaaaaataatatctatttt : 124000
Seq4 : gtccatttaactaataaaaattttaaatcgccgaatgaacaaagtggaatataaaccatataaaaacaatagtttgtactgcaaaaataatatctatttt : 124000

               *    124020         *    124040         *    124060         *    124080         *    124100
Seq1 : tgttttcgaagatatggtaaaattaaatagtagtacacagcatgttataactaacagcagcaacggctcgtaattacttatcatttactagacgaaaagg : 124100
Seq2 : tgttttcgaagatatggtaaaattaaatagtagtacacagcatgttataactaacagcagcaacggctcgtaattacttatcatttactagacgaaaagg : 124100
Seq3 : tgttttcgaagatatggtaaaattaaatagtagtacacagcatgttataactaacagcagcaacggctcgtaattacttatcatttactagacgaaaagg : 124100
Seq4 : tgttttcgaagatatggtaaaattaaatagtagtacacagcatgttataactaacagcagcaacggctcgtaattacttatcatttactagacgaaaagg : 124100

               *    124120         *    124140         *    124160         *    124180         *    124200
Seq1 : tggtgggatattttcttgctcaaataatacgaatatatcacccatccattttatgcgatgtttatatactctaatctttaatagatctatagacgacggg : 124200
Seq2 : tggtgggatattttcttgctcaaataatacgaatatatcacccatccattttatgcgatgtttatatactctaatctttaatagatctatagacgacggg : 124200
Seq3 : tggtgggatattttcttgctcaaataatacgaatatatcacccatccattttatgcgatgtttatatactctaatctttaatagatctatagacgacggg : 124200
Seq4 : tggtgggatattttcttgctcaaataatacgaatatatcacccatccattttatgcgatgtttatatactctaatctttaatagatctatagacgacggg : 124200

               *    124220         *    124240         *    124260         *    124280         *    124300
Seq1 : tttaccaacaatatagattttatcgattcatctaatttaaacccttccttaaacgtgaatgatctattatctggcataacgatgaccctacctgatgaat : 124300
Seq2 : tttaccaacaatatagattttatcgattcatctaatttaaacccttccttaaacgtgaatgatctattatctggcataacgatgaccctacctgatgaat : 124300
Seq3 : tttaccaacaatatagattttatcgattcatctaatttaaacccttccttaaacgtgaatgatctattatctggcataacgatgaccctacctgatgaat : 124300
Seq4 : tttaccaacaatatagattttatcgattcatctaatttaaacccttccttaaacgtgaatgatctattatctggcataacgatgaccctacctgatgaat : 124300

               *    124320         *    124340         *    124360         *    124380         *    124400
Seq1 : cggacaatgtactgggccatgtagaataaattatcaacgaattatcgtctacgaacatttatatcatttgttttaattttaggacgcgaataaatagata : 124400
Seq2 : cggacaatgtactgggccatgtagaataaattatcaacgaattatcgtctacgaacatttatatcatttgttttaattttaggacgcgaataaatagata : 124400
Seq3 : cggacaatgtactgggccatgtagaataaattatcaacgaattatcgtctacgaacatttatatcatttgttttaattttaggacgcgaataaatagata : 124400
Seq4 : cggacaatgtactgggccatgtagaataaattatcaacgaattatcgtctacgaacatttatatcatttgttttaattttaggacgcgaataaatagata : 124400

               *    124420         *    124440         *    124460         *    124480         *    124500
Seq1 : taaaatagaaaataacagatattacaaccagtgttatggccgcgcccaaccaggtaggcagttttattttatcttttactacaggttctcctggatgtac : 124500
Seq2 : taaaatagaaaataacagatattacaaccagtgttatggccgcgcccaaccaggtaggcagttttattttatcttttactacaggttctcctggatgtac : 124500
Seq3 : taaaatagaaaataacagatattacaaccagtgttatggccgcgcccaaccaggtaggcagttttattttatcttttactacaggttctcctggatgtac : 124500
Seq4 : taaaatagaaaataacagatattacaaccagtgttatggccgcgcccaaccaggtaggcagttttattttatcttttactacaggttctcctggatgtac : 124500

               *    124520         *    124540         *    124560         *    124580         *    124600
Seq1 : gtcaccaacggcggacgtagttctagtacaattagacgtaagttccgcttgggaatttttttaacgctaaagagttaacgttaatcgtgcacccaacgtat : 124600
Seq2 : gtcaccaacggcggacgtagttctagtacaattagacgtaagttccgcttgggaatttttttaacgctaaagagttaacgttaatcgtgcacccaacgtat : 124600
Seq3 : gtcaccaacggcggacgtagttctagtacaattagacgtaagttccgcttgggaatttttttaacgctaaagagttaacgttaatcgtgcacccaacgtat : 124600
Seq4 : gtcaccaacggcggacgtagttctagtacaattagacgtaagttccgcttgggaatttttttaacgctaaagagttaacgttaatcgtgcacccaacgtat : 124600
```

```
           *    124620          *    124640          *    124660          *    124680          *    124700
Seq1 : ttacatctagttctttgaacatcttgattataatataaccattttctatctctagattcgtcggtgcactcatgtaaccaacataccctaggtcctaaat : 124700
Seq2 : ttacatctagttctttgaacatcttgattataatataaccattttctatctctagattcgtcggtgcactcatgtaaccaacataccctaggtcctaaat : 124700
Seq3 : ttacatctagttctttgaacatcttgattataatataaccattttctatctctagattcgtcggtgcactcatgtaaccaacataccctaggtcctaaat : 124700
Seq4 : ttacatctagttctttgaacatcttgattataatataaccattttctatctctagattcgtcggtgcactcatgtaaccaacataccctaggtcctaaat : 124700

           *    124720          *    124740          *    124760          *    124780          *    124800
Seq1 : atttatctccggaattagattttggataattcgcgcaccaacaatttctatttcctttatgatcgttacaaaagacgtataatgccgtatccccaaaagt : 124800
Seq2 : atttatctccggaattagattttggataattcgcgcaccaacaatttctatttcctttatgatcgttacaaaagacgtataatgccgtatccccaaaagt : 124800
Seq3 : atttatctccggaattagattttggataattcgcgcaccaacaatttctatttcctttatgatcgttacaaaagacgtataatgccgtatccccaaaagt : 124800
Seq4 : atttatctccggaattagattttggataattcgcgcaccaacaatttctatttcctttatgatcgttacaaaagacgtataatgccgtatccccaaaagt : 124800

           *    124820          *    124840          *    124860          *    124880          *    124900
Seq1 : aaaataatcaggacgaataattctaataaactcagaacaatatctcgcatccatatgtttggagcaaatatcggaataagtagacatagccggtttccgt : 124900
Seq2 : aaaataatcaggacgaataattctaataaactcagaacaatatctcgcatccatatgtttggagcaaatatcggaataagtagacatagccggtttccgt : 124900
Seq3 : aaaataatcaggacgaataattctaataaactcagaacaatatctcgcatccatatgtttggagcaaatatcggaataagtagacatagccggtttccgt : 124900
Seq4 : aaaataatcaggacgaataattctaataaactcagaacaatatctcgcatccatatgtttggagcaaatatcggaataagtagacatagccggtttccgt : 124900

           *    124920          *    124940          *    124960          *    124980          *    125000
Seq1 : tttgcacgtaaccattctaaacaattggggtttccaggatcgtttctacaaaatccagtcatgaaatcgtcacaatgttctgtcttgtaattattattaa : 125000
Seq2 : tttgcacgtaaccattctaaacaattggggtttccaggatcgtttctacaaaatccagtcatgaaatcgtcacaatgttctgtcttgtaattattattaa : 125000
Seq3 : tttgcacgtaaccattctaaacaattggggtttccaggatcgtttctacaaaatccagtcatgaaatcgtcacaatgttctgtcttgtaattattattaa : 125000
Seq4 : tttgcacgtaaccattctaaacaattggggtttccaggatcgtttctacaaaatccagtcatgaaatcgtcacaatgttctgtcttgtaattattattaa : 125000

           *    125020          *    125040          *    125060          *    125080          *    125100
Seq1 : atatttttggacagtgtttggtatttgtcttagaacaacatttgccacgctatcactatcgcccaggagataatcctttttttataaaatgacatcgttg : 125100
Seq2 : atatttttggacagtgtttggtatttgtcttagaacaacatttgccacgctatcactatcgcccaggagataatcctttttttataaaatgacatcgttg : 125100
Seq3 : atatttttggacagtgtttggtatttgtcttagaacaacatttgccacgctatcactatcgcccaggagataatcctttttttataaaatgacatcgttg : 125100
Seq4 : atatttttggacagtgtttggtatttgtcttagaacaacatttgccacgctatcactatcgcccaggagataatcctttttttataaaatgacatcgttg : 125100

           *    125120          *    125140          *    125160          *    125180          *    125200
Seq1 : cccggatgctatataatcagtagcgtgttttaaatccttaatatattcaggagttacctcgttctgataatagattaatgatccaggacgaaatttgaaa : 125200
Seq2 : cccggatgctatataatcagtagcgtgttttaaatccttaatatattcaggagttacctcgttctgataatagattaatgatccaggacgaaatttgaaa : 125200
Seq3 : cccggatgctatataatcagtagcgtgttttaaatccttaatatattcaggagttacctcgttctgataatagattaatgatccaggacgaaatttgaaa : 125200
Seq4 : cccggatgctatataatcagtagcgtgttttaaatccttaatatattcaggagttacctcgttctgataatagattaatgatccaggacgaaatttgaaa : 125200

           *    125220          *    125240          *    125260          *    125280          *    125300
Seq1 : gaactacatggttctccatgaattaatacatattgtttagcaaattcaggaactataaaactactacaatgatctatcgacataccatctatcaaacaaa : 125300
Seq2 : gaactacatggttctccatgaattaatacatattgtttagcaaattcaggaactataaaactactacaatgatctatcgacataccatctatcaaacaaa : 125300
Seq3 : gaactacatggttctccatgaattaatacatattgtttagcaaattcaggaactataaaactactacaatgatctatcgacataccatctatcaaacaaa : 125300
Seq4 : gaactacatggttctccatgaattaatacatattgtttagcaaattcaggaactataaaactactacaatgatctatcgacataccatctatcaaacaaa : 125300
```

```
               *    125320         *    125340         *    125360         *    125380         *    125400
Seq1 : acttgggtttaatttctcccggagatgtttcataatagtacgtataactttcttctgcaaacttaacagctctattatattcaggataattaaaacctaa : 125400
Seq2 : acttgggtttaatttctcccggagatgtttcataatagtacgtataactttcttctgcaaacttaacagctctattatattcaggataattaaaacctaa : 125400
Seq3 : acttgggtttaatttctcccggagatgtttcataatagtacgtataactttcttctgcaaacttaacagctctattatattcaggataattaaaacctaa : 125400
Seq4 : acttgggtttaatttctcccggagatgtttcataatagtacgtataactttcttctgcaaacttaacagctctattatattcaggataattaaaacctaa : 125400

               *    125420         *    125440         *    125460         *    125480         *    125500
Seq1 : ttccatatatttgtctcgtatatctgctattcctggtgctattttgattctattaagagtaacagctgcccccattcttaataatcgtcagtatttaaac : 125500
Seq2 : ttccatatatttgtctcgtatatctgctattcctggtgctattttgattctattaagagtaacagctgcccccattcttaataatcgtcagtatttaaac : 125500
Seq3 : ttccatatatttgtctcgtatatctgctattcctggtgctattttgattctattaagagtaacagctgcccccattcttaataatcgtcagtatttaaac : 125500
Seq4 : ttccatatatttgtctcgtatatctgctattcctggtgctattttgattctattaagagtaacagctgcccccattcttaataatcgtcagtatttaaac : 125500

               *    125520         *    125540         *    125560         *    125580         *    125600
Seq1 : tgttaaatgttggtatatcaacatctaccttatttcccgcagtataaggtttgttgcaggtatactgttcaggaatggttacatttatacttcttctata : 125600
Seq2 : tgttaaatgttggtatatcaacatctaccttatttcccgcagtataaggtttgttgcaggtatactgttcaggaatggttacatttatacttcttctata : 125600
Seq3 : tgttaaatgttggtatatcaacatctaccttatttcccgcagtataaggtttgttgcaggtatactgttcaggaatggttacatttatacttcttctata : 125600
Seq4 : tgttaaatgttggtatatcaacatctaccttatttcccgcagtataaggtttgttgcaggtatactgttcaggaatggttacatttatacttcttctata : 125600

               *    125620         *    125640         *    125660         *    125680         *    125700
Seq1 : gtcctgtctttcgatgttcatcacatatgcaaagaacagaataaacaaaataatgtaagaaataatattaaatatctgtgaattcgtaaatacattgatt : 125700
Seq2 : gtcctgtctttcgatgttcatcacatatgcaaagaacagaataaacaaaataatgtaagaaataatattaaatatctgtgaattcgtaaatacattgatt : 125700
Seq3 : gtcctgtctttcgatgttcatcacatatgcaaagaacagaataaacaaaataatgtaagaaataatattaaatatctgtgaattcgtaaatacattgatt : 125700
Seq4 : gtcctgtctttcgatgttcatcacatatgcaaagaacagaataaacaaaataatgtaagaaataatattaaatatctgtgaattcgtaaatacattgatt : 125700

               *    125720         *    125740         *    125760         *    125780         *    125800
Seq1 : gccataataattacagcagctacaatacacacaatagacattcccacagtgttgccattacctccacgatacatttgagttactaagcaataggtaataa : 125800
Seq2 : gccataataattacagcagctacaatacacacaatagacattcccacagtgttgccattacctccacgatacatttgagttactaagcaataggtaataa : 125800
Seq3 : gccataataattacagcagctacaatacacacaatagacattcccacagtgttgccattacctccacgatacatttgagttactaagcaataggtaataa : 125800
Seq4 : gccataataattacagcagctacaatacacacaatagacattcccacagtgttgccattacctccacgatacatttgagttactaagcaataggtaataa : 125800

               *    125820         *    125840         *    125860         *    125880         *    125900
Seq1 : ctaagctagtaagaggcaatagaaaagatgagataaatatcatcaatatagagattagaggagggctatatagagccaagacgaacaaaatcaaaccgag : 125900
Seq2 : ctaagctagtaagaggcaatagaaaagatgagataaatatcatcaatatagagattagaggagggctatatagagccaagacgaacaaaatcaaaccgag : 125900
Seq3 : ctaagctagtaagaggcaatagaaaagatgagataaatatcatcaatatagagattagaggagggctatatagagccaagacgaacaaaatcaaaccgag : 125900
Seq4 : ctaagctagtaagaggcaatagaaaagatgagataaatatcatcaatatagagattagaggagggctatatagagccaagacgaacaaaatcaaaccgag : 125900

               *    125920         *    125940         *    125960         *    125980         *    126000
Seq1 : taacgttctaacatcattatttttgaagattcccaaataatcattcattcctccataatcgttttgcatcatacctccatctttaggcataaacgattgc : 126000
Seq2 : taacgttctaacatcattatttttgaagattcccaaataatcattcattcctccataatcgttttgcatcatacctccatctttaggcataaacgattgc : 126000
Seq3 : taacgttctaacatcattatttttgaagattcccaaataatcattcattcctccataatcgttttgcatcatacctccatctttaggcataaacgattgc : 126000
Seq4 : taacgttctaacatcattatttttgaagattcccaaataatcattcattcctccataatcgttttgcatcatacctccatctttaggcataaacgattgc : 126000
```

```
             *    126020         *    126040         *    126060         *    126080         *    126100
Seq1 : tgctgttcctctgtaaataaatctttatcaagcactccagcacccgcagagaagtcgtcaagcatattgtaatatcttaaataactcatttatatattaa : 126100
Seq2 : tgctgttcctctgtaaataaatctttatcaagcactccagcacccgcagagaagtcgtcaagcatattgtaatatcttaaataactcatttatatattaa : 126100
Seq3 : tgctgttcctctgtaaataaatctttatcaagcactccagcacccgcagagaagtcgtcaagcatattgtaatatcttaaataactcatttatatattaa : 126100
Seq4 : tgctgttcctctgtaaataaatctttatcaagcactccagcacccgcagagaagtcgtcaagcatattgtaatatcttaaataactcatttatatattaa : 126100

             *    126120         *    126140         *    126160         *    126180         *    126200
Seq1 : aaaatgtcactattaaagatggagtataatctttatgccgaactaaaaaaaatgacttgtggtcaacccctaagtctttttaacgaagacggggatttcg : 126200
Seq2 : aaaatgtcactattaaagatggagtataatctttatgccgaactaaaaaaaatgacttgtggtcaacccctaagtctttttaacgaagacggggatttcg : 126200
Seq3 : aaaatgtcactattaaagatggagtataatctttatgccgaactaaaaaaaatgacttgtggtcaacccctaagtctttttaacgaagacggggatttcg : 126200
Seq4 : aaaatgtcactattaaagatggagtataatctttatgccgaactaaaaaaaatgacttgtggtcaacccctaagtctttttaacgaagacggggatttcg : 126200

             *    126220         *    126240         *    126260         *    126280         *    126300
Seq1 : tagaagttgaaccgggatcatcctttaagtttctgatacctaagggatttacgcctctccttccgtaaagacgagtctagtatttgaaacattaacaac : 126300
Seq2 : tagaagttgaaccgggatcatcctttaagtttctgatacctaagggatttacgcctctccttccgtaaagacgagtctagtatttgaaacattaacaac : 126300
Seq3 : tagaagttgaaccgggatcatcctttaagtttctgatacctaagggatttacgcctctccttccgtaaagacgagtctagtatttgaaacattaacaac : 126300
Seq4 : tagaagttgaaccgggatcatcctttaagtttctgatacctaagggatttacgcctctccttccgtaaagacgagtctagtatttgaaacattaacaac : 126300

             *    126320         *    126340         *    126360         *    126380         *    126400
Seq1 : gaccgataataaaatcactagtatcaatccaacaaatgcgccaaagttatatcctcttcaacgcaaagtcgtatctgaagtagtttctaatatgaggaaa : 126400
Seq2 : gaccgataataaaatcactagtatcaatccaacaaatgcgccaaagttatatcctcttcaacgcaaagtcgtatctgaagtagtttctaatatgaggaaa : 126400
Seq3 : gaccgataataaaatcactagtatcaatccaacaaatgcgccaaagttatatcctcttcaacgcaaagtcgtatctgaagtagtttctaatatgaggaaa : 126400
Seq4 : gaccgataataaaatcactagtatcaatccaacaaatgcgccaaagttatatcctcttcaacgcaaagtcgtatctgaagtagtttctaatatgaggaaa : 126400

             *    126420         *    126440         *    126460         *    126480         *    126500
Seq1 : atgatcgaatcaaaacgtcctctatacattactcttcacttggcgtgtggatttggtaagactattaccacgtgttatcttatggctacacacggtagaa : 126500
Seq2 : atgatcgaatcaaaacgtcctctatacattactcttcacttggcgtgtggatttggtaagactattaccacgtgttatcttatggctacacacggtagaa : 126500
Seq3 : atgatcgaatcaaaacgtcctctatacattactcttcacttggcgtgtggatttggtaagactattaccacgtgttatcttatggctacacacggtagaa : 126500
Seq4 : atgatcgaatcaaaacgtcctctatacattactcttcacttggcgtgtggatttggtaagactattaccacgtgttatcttatggctacacacggtagaa : 126500

             *    126520         *    126540         *    126560         *    126580         *    126600
Seq1 : aaaccgtcatttgcgtacccaataaaatgttaatacatcaatggaagacacaggtagaggcagtcggattggaacataagatatccatagatggagtaag : 126600
Seq2 : aaaccgtcatttgcgtacccaataaaatgttaatacatcaatggaagacacaggtagaggcagtcggattggaacataagatatccatagatggagtaag : 126600
Seq3 : aaaccgtcatttgcgtacccaataaaatgttaatacatcaatggaagacacaggtagaggcagtcggattggaacataagatatccatagatggagtaag : 126600
Seq4 : aaaccgtcatttgcgtacccaataaaatgttaatacatcaatggaagacacaggtagaggcagtcggattggaacataagatatccatagatggagtaag : 126600

             *    126620         *    126640         *    126660         *    126680         *    126700
Seq1 : tagtctattaaaggaactaaagactcaaagtccggatgtattaatagtagtcagtagacatctgacaaacgatgccttttgtaaatatatcaataagcat : 126700
Seq2 : tagtctattaaaggaactaaagactcaaagtccggatgtattaatagtagtcagtagacatctgacaaacgatgccttttgtaaatatatcaataagcat : 126700
Seq3 : tagtctattaaaggaactaaagactcaaagtccggatgtattaatagtagtcagtagacatctgacaaacgatgccttttgtaaatatatcaataagcat : 126700
Seq4 : tagtctattaaaggaactaaagactcaaagtccggatgtattaatagtagtcagtagacatctgacaaacgatgccttttgtaaatatatcaataagcat : 126700
```

```
                 *    126720         *    126740         *    126760         *    126780         *    126800
Seq1 : tatgatttgttcatcttggatgaatcacatacgtataatctgatgaacaatacagcagttacaagatttttagcgtattatcctccgatgatgtgttatt : 126800
Seq2 : tatgatttgttcatcttggatgaatcacatacgtataatctgatgaacaatacagcagttacaagatttttagcgtattatcctccgatgatgtgttatt : 126800
Seq3 : tatgatttgttcatcttggatgaatcacatacgtataatctgatgaacaatacagcagttacaagatttttagcgtattatcctccgatgatgtgttatt : 126800
Seq4 : tatgatttgttcatcttggatgaatcacatacgtataatctgatgaacaatacagcagttacaagatttttagcgtattatcctccgatgatgtgttatt : 126800

                 *    126820         *    126840         *    126860         *    126880         *    126900
Seq1 : ttttaactgctacacctagaccagctaaccgaatttattgtaacagtattattaatattgccaagttatccgatctaaaaaaaactatctatgcggtaga : 126900
Seq2 : ttttaactgctacacctagaccagctaaccgaatttattgtaacagtattattaatattgccaagttatccgatctaaaaaaaactatctatgcggtaga : 126900
Seq3 : ttttaactgctacacctagaccagctaaccgaatttattgtaacagtattattaatattgccaagttatccgatctaaaaaaaactatctatgcggtaga : 126900
Seq4 : ttttaactgctacacctagaccagctaaccgaatttattgtaacagtattattaatattgccaagttatccgatctaaaaaaaactatctatgcggtaga : 126900

                 *    126920         *    126940         *    126960         *    126980         *    127000
Seq1 : tagtttttttgagccatattccacagacaatattagacatatggtaaaacgactagatggaccatctaataaatatcatatatataccgagaagttatta : 127000
Seq2 : tagtttttttgagccatattccacagacaatattagacatatggtaaaacgactagatggaccatctaataaatatcatatatataccgagaagttatta : 127000
Seq3 : tagtttttttgagccatattccacagacaatattagacatatggtaaaacgactagatggaccatctaataaatatcatatatataccgagaagttatta : 127000
Seq4 : tagtttttttgagccatattccacagacaatattagacatatggtaaaacgactagatggaccatctaataaatatcatatatataccgagaagttatta : 127000

                 *    127020         *    127040         *    127060         *    127080         *    127100
Seq1 : tctgtagacgagcctagaaatcaacttattcttaataccctggtagaagaattcaagtcaggaactattaatcgcattttagttattactaaactacgtg : 127100
Seq2 : tctgtagacgagcctagaaatcaacttattcttaataccctggtagaagaattcaagtcaggaactattaatcgcattttagttattactaaactacgtg : 127100
Seq3 : tctgtagacgagcctagaaatcaacttattcttaataccctggtagaagaattcaagtcaggaactattaatcgcattttagttattactaaactacgtg : 127100
Seq4 : tctgtagacgagcctagaaatcaacttattcttaataccctggtagaagaattcaagtcaggaactattaatcgcattttagttattactaaactacgtg : 127100

                 *    127120         *    127140         *    127160         *    127180         *    127200
Seq1 : aacatatggtattattctacaaacgattattagatcttttcggaccagaggttgtatttataggagacgcccaaaatagacgtactccagatatggtcaa : 127200
Seq2 : aacatatggtattattctacaaacgattattagatcttttcggaccagaggttgtatttataggagacgcccaaaatagacgtactccagatatggtcaa : 127200
Seq3 : aacatatggtattattctacaaacgattattagatcttttcggaccagaggttgtatttataggagacgcccaaaatagacgtactccagatatggtcaa : 127200
Seq4 : aacatatggtattattctacaaacgattattagatcttttcggaccagaggttgtatttataggagacgcccaaaatagacgtactccagatatggtcaa : 127200

                 *    127220         *    127240         *    127260         *    127280         *    127300
Seq1 : atcaatcaaggaactaaatagatttatattcgtatccaccttattttattccggtactggtttagatattcctagtttggattcgttgttcatttgctcg : 127300
Seq2 : atcaatcaaggaactaaatagatttatattcgtatccaccttattttattccggtactggtttagatattcctagtttggattcgttgttcatttgctcg : 127300
Seq3 : atcaatcaaggaactaaatagatttatattcgtatccaccttattttattccggtactggtttagatattcctagtttggattcgttgttcatttgctcg : 127300
Seq4 : atcaatcaaggaactaaatagatttatattcgtatccaccttattttattccggtactggtttagatattcctagtttggattcgttgttcatttgctcg : 127300

                 *    127320         *    127340         *    127360         *    127380         *    127400
Seq1 : gcagtaatcaacaatatgcaaatagagcaattactagggagggtatgtcgagaaacagaactattagataggacggtatatgtatttcctaacacatcca : 127400
Seq2 : gcagtaatcaacaatatgcaaatagagcaattactagggagggtatgtcgagaaacagaactattagataggacggtatatgtatttcctaacacatcca : 127400
Seq3 : gcagtaatcaacaatatgcaaatagagcaattactagggagggtatgtcgagaaacagaactattagataggacggtatatgtatttcctaacacatcca : 127400
Seq4 : gcagtaatcaacaatatgcaaatagagcaattactagggagggtatgtcgagaaacagaactattagataggacggtatatgtatttcctaacacatcca : 127400
```

```
             *    127420          *    127440          *    127460          *    127480          *    127500
Seq1 : tcaaagaaataaagtacatgataggaaatttcatgcaacgaattattagtctgtctgtagataaactaggatttaaacaagaaagttatcggaaacatca : 127500
Seq2 : tcaaagaaataaagtacatgataggaaatttcatgcaacgaattattagtctgtctgtagataaactaggatttaaacaagaaagttatcggaaacatca : 127500
Seq3 : tcaaagaaataaagtacatgataggaaatttcatgcaacgaattattagtctgtctgtagataaactaggatttaaacaagaaagttatcggaaacatca : 127500
Seq4 : tcaaagaaataaagtacatgataggaaatttcatgcaacgaattattagtctgtctgtagataaactaggatttaaacaagaaagttatcggaaacatca : 127500

             *    127520          *    127540          *    127560          *    127580          *    127600
Seq1 : agaatccgatcccacttctgtatgtacaacatcctccagagaagaacgtgtattaaatagaatatttaactcgcaaaatcgttaagaagtttaagcgacg : 127600
Seq2 : agaatccgatcccacttctgtatgtacaacatcctccagagaagaacgtgtattaaatagaatatttaactcgcaaaatcgttaagaagtttaagcgacg : 127600
Seq3 : agaatccgatcccacttctgtatgtacaacatcctccagagaagaacgtgtattaaatagaatatttaactcgcaaaatcgttaagaagtttaagcgacg : 127600
Seq4 : agaatccgatcccacttctgtatgtacaacatcctccagagaagaacgtgtattaaatagaatatttaactcgcaaaatcgttaagaagtttaagcgacg : 127600

             *    127620          *    127640          *    127660          *    127680          *    127700
Seq1 : atccgcatgctgcgcaggccagtgtattacccctcatagtattaatataatccaatgatacttttgtgatgtcggaaatcttaaccaatttagactgaca : 127700
Seq2 : atccgcatgctgcgcaggccagtgtattacccctcatagtattaatataatccaatgatacttttgtgatgtcggaaatcttaaccaatttagactgaca : 127700
Seq3 : atccgcatgctgcgcaggccagtgtattacccctcatagtattaatataatccaatgatacttttgtgatgtcggaaatcttaaccaatttagactgaca : 127700
Seq4 : atccgcatgctgcgcaggccagtgtattacccctcatagtattaatataatccaatgatacttttgtgatgtcggaaatcttaaccaatttagactgaca : 127700

             *    127720          *    127740          *    127760          *    127780          *    127800
Seq1 : ggcagaacacgtcatgcaatcatcatcgtcatcgataactgtagtcttgggcttctttttgcggctcttcattccggaacgcacattggtgctatccatt : 127800
Seq2 : ggcagaacacgtcatgcaatcatcatcgtcatcgataactgtagtcttgggcttctttttgcggctcttcattccggaacgcacattggtgctatccatt : 127800
Seq3 : ggcagaacacgtcatgcaatcatcatcgtcatcgataactgtagtcttgggcttctttttgcggctcttcattccggaacgcacattggtgctatccatt : 127800
Seq4 : ggcagaacacgtcatgcaatcatcatcgtcatcgataactgtagtcttgggcttctttttgcggctcttcattccggaacgcacattggtgctatccatt : 127800

             *    127820          *    127840          *    127860          *    127880          *    127900
Seq1 : taggtagtaaaaaataagtcagaatatgccctataacacgatcgtgcaaaacctggtatatcgtctctatctttatcacaatatagtgtatcgacatctt : 127900
Seq2 : taggtagtaaaaaataagtcagaatatgccctataacacgatcgtgcaaaacctggtatatcgtctctatctttatcacaatatagtgtatcgacatctt : 127900
Seq3 : taggtagtaaaaaataagtcagaatatgccctataacacgatcgtgcaaaacctggtatatcgtctctatctttatcacaatatagtgtatcgacatctt : 127900
Seq4 : taggtagtaaaaaataagtcagaatatgccctataacacgatcgtgcaaaacctggtatatcgtctctatctttatcacaatatagtgtatcgacatctt : 127900

             *    127920          *    127940          *    127960          *    127980          *    128000
Seq1 : tattattattgacctcgtttatcttggaacatggaatgggaacatttttgttatcaacggccacctttgccttaattccagatgttgtaaaattataact : 128000
Seq2 : tattattattgacctcgtttatcttggaacatggaatgggaacatttttgttatcaacggccacctttgccttaattccagatgttgtaaaattataact : 128000
Seq3 : tattattattgacctcgtttatcttggaacatggaatgggaacatttttgttatcaacggccacctttgccttaattccagatgttgtaaaattataact : 128000
Seq4 : tattattattgacctcgtttatcttggaacatggaatgggaacatttttgttatcaacggccacctttgccttaattccagatgttgtaaaattataact : 128000

             *    128020          *    128040          *    128060          *    128080          *    128100
Seq1 : aaacagtctatcatcgacacaaatgaaattcttgtttagacgtttgtagtttacgtatgcggctcgttcgcgtctcatttttcagatattgcaggtact : 128100
Seq2 : aaacagtctatcatcgacacaaatgaaattcttgtttagacgtttgtagtttacgtatgcggctcgttcgcgtctcatttttcagatattgcaggtact : 128100
Seq3 : aaacagtctatcatcgacacaaatgaaattcttgtttagacgtttgtagtttacgtatgcggctcgttcgcgtctcatttttcagatattgcaggtact : 128100
Seq4 : aaacagtctatcatcgacacaaatgaaattcttgtttagacgtttgtagtttacgtatgcggctcgttcgcgtctcatttttcagatattgcaggtact : 128100
```

```
                *    128120          *    128140          *    128160          *    128180          *    128200
Seq1 : ataatattaaaaataagaatgaaataacataggattaaaaataaagttatcatgacttctagcgctgatttaactaacttaaaagaattacttagtctgt : 128200
Seq2 : ataatattaaaaataagaatgaaataacataggattaaaaataaagttatcatgacttctagcgctgatttaactaacttaaaagaattacttagtctgt : 128200
Seq3 : ataatattaaaaataagaatgaaataacataggattaaaaataaagttatcatgacttctagcgctgatttaactaacttaaaagaattacttagtctgt : 128200
Seq4 : ataatattaaaaataagaatgaaataacataggattaaaaataaagttatcatgacttctagcgctgatttaactaacttaaaagaattacttagtctgt : 128200

                *    128220          *    128240          *    128260          *    128280          *    128300
Seq1 : acaaaagtttgaaattttcagattctgcggctatagaaaagtataattctttggtagaatggggaacatctacttactggaaaataggcgtgcaaaaggt : 128300
Seq2 : acaaaagtttgaaattttcagattctgcggctatagaaaagtataattctttggtagaatggggaacatctacttactggaaaataggcgtgcaaaaggt : 128300
Seq3 : acaaaagtttgaaattttcagattctgcggctatagaaaagtataattctttggtagaatggggaacatctacttactggaaaataggcgtgcaaaaggt : 128300
Seq4 : acaaaagtttgaaattttcagattctgcggctatagaaaagtataattctttggtagaatggggaacatctacttactggaaaataggcgtgcaaaaggt : 128300

                *    128320          *    128340          *    128360          *    128380          *    128400
Seq1 : agctaatgtcgagacgtcaatatctgattattatgatgaggtaaaaaataaaccgtttaatattgatccgggctattacatttcttaccggtatatttt : 128400
Seq2 : agctaatgtcgagacgtcaatatctgattattatgatgaggtaaaaaataaaccgtttaatattgatccgggctattacatttcttaccggtatatttt : 128400
Seq3 : agctaatgtcgagacgtcaatatctgattattatgatgaggtaaaaaataaaccgtttaatattgatccgggctattacatttcttaccggtatatttt : 128400
Seq4 : agctaatgtcgagacgtcaatatctgattattatgatgaggtaaaaaataaaccgtttaatattgatccgggctattacatttcttaccggtatatttt : 128400

                *    128420          *    128440          *    128460          *    128480          *    128500
Seq1 : gggagcgtctttatttattcgaagggtaaaaatatggtagaacttggatctggaaactcttttcaaataccagatgatatgcgaagtgcgtgtaacaaag : 128500
Seq2 : gggagcgtctttatttattcgaagggtaaaaatatggtagaacttggatctggaaactcttttcaaataccagatgatatgcgaagtgcgtgtaacaaag : 128500
Seq3 : gggagcgtctttatttattcgaagggtaaaaatatggtagaacttggatctggaaactcttttcaaataccagatgatatgcgaagtgcgtgtaacaaag : 128500
Seq4 : gggagcgtctttatttattcgaagggtaaaaatatggtagaacttggatctggaaactcttttcaaataccagatgatatgcgaagtgcgtgtaacaaag : 128500

                *    128520          *    128540          *    128560          *    128580          *    128600
Seq1 : tattagacagcgataacggaatagactttctgagatttgttttgttaaacaatagatggataatggaagatgctatatcaaaatatcagtctccagttaa : 128600
Seq2 : tattagacagcgataacggaatagactttctgagatttgttttgttaaacaatagatggataatggaagatgctatatcaaaatatcagtctccagttaa : 128600
Seq3 : tattagacagcgataacggaatagactttctgagatttgttttgttaaacaatagatggataatggaagatgctatatcaaaatatcagtctccagttaa : 128600
Seq4 : tattagacagcgataacggaatagactttctgagatttgttttgttaaacaatagatggataatggaagatgctatatcaaaatatcagtctccagttaa : 128600

                *    128620          *    128640          *    128660          *    128680          *    128700
Seq1 : tatatttaaactagctagtgagtacggattaaacatacccaaatatttagaaattgaaatagaggaagacacattatttgacgacgagttatactctatt : 128700
Seq2 : tatatttaaactagctagtgagtacggattaaacatacccaaatatttagaaattgaaatagaggaagacacattatttgacgacgagttatactctatt : 128700
Seq3 : tatatttaaactagctagtgagtacggattaaacatacccaaatatttagaaattgaaatagaggaagacacattatttgacgacgagttatactctatt : 128700
Seq4 : tatatttaaactagctagtgagtacggattaaacatacccaaatatttagaaattgaaatagaggaagacacattatttgacgacgagttatactctatt : 128700

                *    128720          *    128740          *    128760          *    128780          *    128800
Seq1 : atagaacgctcttttgatgataaatttccaaaaatatccatatcgtatattaagttgggagaacttaggcggcaagttgtagactttttcaaattctcgt : 128800
Seq2 : atagaacgctcttttgatgataaatttccaaaaatatccatatcgtatattaagttgggagaacttaggcggcaagttgtagactttttcaaattctcgt : 128800
Seq3 : atagaacgctcttttgatgataaatttccaaaaatatccatatcgtatattaagttgggagaacttaggcggcaagttgtagactttttcaaattctcgt : 128800
Seq4 : atagaacgctcttttgatgataaatttccaaaaatatccatatcgtatattaagttgggagaacttaggcggcaagttgtagactttttcaaattctcgt : 128800
```

```
              *    128820         *    128840         *    128860         *    128880         *    128900
Seq1 : tcatgtatattgagtccatcaaggtagatcgtataggagataatatttttattcctagcgttataacaaaatcaggaaaaaagatattagtaaaagatgt : 128900
Seq2 : tcatgtatattgagtccatcaaggtagatcgtataggagataatatttttattcctagcgttataacaaaatcaggaaaaaagatattagtaaaagatgt : 128900
Seq3 : tcatgtatattgagtccatcaaggtagatcgtataggagataatatttttattcctagcgttataacaaaatcaggaaaaaagatattagtaaaagatgt : 128900
Seq4 : tcatgtatattgagtccatcaaggtagatcgtataggagataatatttttattcctagcgttataacaaaatcaggaaaaaagatattagtaaaagatgt : 128900

              *    128920         *    128940         *    128960         *    128980         *    129000
Seq1 : agaccatttaatacgatctaaggttagagaacatacatttgtaaaagtaaaaaagaaaaacacattttccattttatacgactatgatgggaacggaaca : 129000
Seq2 : agaccatttaatacgatctaaggttagagaacatacatttgtaaaagtaaaaaagaaaaacacattttccattttatacgactatgatgggaacggaaca : 129000
Seq3 : agaccatttaatacgatctaaggttagagaacatacatttgtaaaagtaaaaaagaaaaacacattttccattttatacgactatgatgggaacggaaca : 129000
Seq4 : agaccatttaatacgatctaaggttagagaacatacatttgtaaaagtaaaaaagaaaaacacattttccattttatacgactatgatgggaacggaaca : 129000

              *    129020         *    129040         *    129060         *    129080         *    129100
Seq1 : gaaactagaggagaagtaataaaacgaattatagacactataggacgagactattatgttaacggaaagtatttctctaaggttggtagtgcaggcttaa : 129100
Seq2 : gaaactagaggagaagtaataaaacgaattatagacactataggacgagactattatgttaacggaaagtatttctctaaggttggtagtgcaggcttaa : 129100
Seq3 : gaaactagaggagaagtaataaaacgaattatagacactataggacgagactattatgttaacggaaagtatttctctaaggttggtagtgcaggcttaa : 129100
Seq4 : gaaactagaggagaagtaataaaacgaattatagacactataggacgagactattatgttaacggaaagtatttctctaaggttggtagtgcaggcttaa : 129100

              *    129120         *    129140         *    129160         *    129180         *    129200
Seq1 : agcaattgactaataaattagatattaatgagtgcgcaactgtcgatgagttagttgatgagattaataaatccggaactgtaaaacgaaaaataaaaaa : 129200
Seq2 : agcaattgactaataaattagatattaatgagtgcgcaactgtcgatgagttagttgatgagattaataaatccggaactgtaaaacgaaaaataaaaaa : 129200
Seq3 : agcaattgactaataaattagatattaatgagtgcgcaactgtcgatgagttagttgatgagattaataaatccggaactgtaaaacgaaaaataaaaaa : 129200
Seq4 : agcaattgactaataaattagatattaatgagtgcgcaactgtcgatgagttagttgatgagattaataaatccggaactgtaaaacgaaaaataaaaaa : 129200

              *    129220         *    129240         *    129260         *    129280         *    129300
Seq1 : ccaatcagcatttgatttaagcagagaatgtttgggatatccagaagcagattttataacgttagttaataacatgcggttcaaaatagaaaattgtaag : 129300
Seq2 : ccaatcagcatttgatttaagcagagaatgtttgggatatccagaagcagattttataacgttagttaataacatgcggttcaaaatagaaaattgtaag : 129300
Seq3 : ccaatcagcatttgatttaagcagagaatgtttgggatatccagaagcagattttataacgttagttaataacatgcggttcaaaatagaaaattgtaag : 129300
Seq4 : ccaatcagcatttgatttaagcagagaatgtttgggatatccagaagcagattttataacgttagttaataacatgcggttcaaaatagaaaattgtaag : 129300

              *    129320         *    129340         *    129360         *    129380         *    129400
Seq1 : gttgtaaatttcaatattgaaaatactaattgtttaaataacccgagtattgaaactatatatggaaactttaaccagttcgtctcaatctttaatatcg : 129400
Seq2 : gttgtaaatttcaatattgaaaatactaattgtttaaataacccgagtattgaaactatatatggaaactttaaccagttcgtctcaatctttaatatcg : 129400
Seq3 : gttgtaaatttcaatattgaaaatactaattgtttaaataacccgagtattgaaactatatatggaaactttaaccagttcgtctcaatctttaatatcg : 129400
Seq4 : gttgtaaatttcaatattgaaaatactaattgtttaaataacccgagtattgaaactatatatggaaactttaaccagttcgtctcaatctttaatatcg : 129400

              *    129420         *    129440         *    129460         *    129480         *    129500
Seq1 : tcaccgatgtcaaaaaaagattattcgagtgaaataatatgcgcctttgatataggtgcaaaaaatcctgccagaactgttttagaagtcaaggataact : 129500
Seq2 : tcaccgatgtcaaaaaaagattattcgagtgaaataatatgcgcctttgatataggtgcaaaaaatcctgccagaactgttttagaagtcaaggataact : 129500
Seq3 : tcaccgatgtcaaaaaaagattattcgagtgaaataatatgcgcctttgatataggtgcaaaaaatcctgccagaactgttttagaagtcaaggataact : 129500
Seq4 : tcaccgatgtcaaaaaaagattattcgagtgaaataatatgcgcctttgatataggtgcaaaaaatcctgccagaactgttttagaagtcaaggataact : 129500
```

```
             *    129520         *    129540         *    129560         *    129580         *    129600
Seq1 : ccgttagggtattggatatatcaaaattagactggagttctgattgggaaaggcgcatagctaaagatttgtcacaatatgaatacactacagttcttct : 129600
Seq2 : ccgttagggtattggatatatcaaaattagactggagttctgattgggaaaggcgcatagctaaagatttgtcacaatatgaatacactacagttcttct : 129600
Seq3 : ccgttagggtattggatatatcaaaattagactggagttctgattgggaaaggcgcatagctaaagatttgtcacaatatgaatacactacagttcttct : 129600
Seq4 : ccgttagggtattggatatatcaaaattagactggagttctgattgggaaaggcgcatagctaaagatttgtcacaatatgaatacactacagttcttct : 129600

             *    129620         *    129640         *    129660         *    129680         *    129700
Seq1 : agaacgtcagcctagaaggtcgccgtatgttaaatttatctattttattaaaggctttttatatcatacatcggctgccaaagttatttgcgtctcgcct : 129700
Seq2 : agaacgtcagcctagaaggtcgccgtatgttaaatttatctattttattaaaggctttttatatcatacatcggctgccaaagttatttgcgtctcgcct : 129700
Seq3 : agaacgtcagcctagaaggtcgccgtatgttaaatttatctattttattaaaggctttttatatcatacatcggctgccaaagttatttgcgtctcgcct : 129700
Seq4 : agaacgtcagcctagaaggtcgccgtatgttaaatttatctattttattaaaggctttttatatcatacatcggctgccaaagttatttgcgtctcgcct : 129700

             *    129720         *    129740         *    129760         *    129780         *    129800
Seq1 : gtcatgtctggtaattcatatagagatcgaaaaaagagatcggtcgaagcatttcttgattggatggacacattcggattgcgagactccgttccggata : 129800
Seq2 : gtcatgtctggtaattcatatagagatcgaaaaaagagatcggtcgaagcatttcttgattggatggacacattcggattgcgagactccgttccggata : 129800
Seq3 : gtcatgtctggtaattcatatagagatcgaaaaaagagatcggtcgaagcatttcttgattggatggacacattcggattgcgagactccgttccggata : 129800
Seq4 : gtcatgtctggtaattcatatagagatcgaaaaaagagatcggtcgaagcatttcttgattggatggacacattcggattgcgagactccgttccggata : 129800

             *    129820         *    129840         *    129860         *    129880         *    129900
Seq1 : gacgcaaattagacgatgtagcggatagtttcaatttggctatgagatacgtattagataaatggaatactaattatacaccttataataggtgtaaatc : 129900
Seq2 : gacgcaaattagacgatgtagcggatagtttcaatttggctatgagatacgtattagataaatggaatactaattatacaccttataataggtgtaaatc : 129900
Seq3 : gacgcaaattagacgatgtagcggatagtttcaatttggctatgagatacgtattagataaatggaatactaattatacaccttataataggtgtaaatc : 129900
Seq4 : gacgcaaattagacgatgtagcggatagtttcaatttggctatgagatacgtattagataaatggaatactaattatacaccttataataggtgtaaatc : 129900

             *    129920         *    129940         *    129960         *    129980         *    130000
Seq1 : tagaaattacataaaaaaaatgtaataacgttagtaacgccattatggataatctatttacctttctacatgaaatagaagatagatatgccagaactat : 130000
Seq2 : tagaaattacataaaaaaaatgtaataacgttagtaacgccattatggataatctatttacctttctacatgaaatagaagatagatatgccagaactat : 130000
Seq3 : tagaaattacataaaaaaaatgtaataacgttagtaacgccattatggataatctatttacctttctacatgaaatagaagatagatatgccagaactat : 130000
Seq4 : tagaaattacataaaaaaaatgtaataacgttagtaacgccattatggataatctatttacctttctacatgaaatagaagatagatatgccagaactat : 130000

             *    130020         *    130040         *    130060         *    130080         *    130100
Seq1 : ttttaactttcatctaataagttgcgatgaaataggagatatatatggtcttatgaaagaacgcatttcctcagaggatatgtttgataatatagtatat : 130100
Seq2 : ttttaactttcatctaataagttgcgatgaaataggagatatatatggtcttatgaaagaacgcatttcctcagaggatatgtttgataatatagtatat : 130100
Seq3 : ttttaactttcatctaataagttgcgatgaaataggagatatatatggtcttatgaaagaacgcatttcctcagaggatatgtttgataatatagtatat : 130100
Seq4 : ttttaactttcatctaataagttgcgatgaaataggagatatatatggtcttatgaaagaacgcatttcctcagaggatatgtttgataatatagtatat : 130100

             *    130120         *    130140         *    130160         *    130180         *    130200
Seq1 : aataaagatatacatcctgccattaagaaactagtgtattgcgacatccaacttactaaacacattattaatcagaatacgtatccggtatttaacgatt : 130200
Seq2 : aataaagatatacatcctgccattaagaaactagtgtattgcgacatccaacttactaaacacattattaatcagaatacgtatccggtatttaacgatt : 130200
Seq3 : aataaagatatacatcctgccattaagaaactagtgtattgcgacatccaacttactaaacacattattaatcagaatacgtatccggtatttaacgatt : 130200
Seq4 : aataaagatatacatcctgccattaagaaactagtgtattgcgacatccaacttactaaacacattattaatcagaatacgtatccggtatttaacgatt : 130200
```

```
                 *    130220         *    130240         *    130260         *    130280         *    130300
Seq1 : cttcacaagtgaaatgttgtcattatttcgacataaactcagataatagcaatattagctctcgtacagtagagatatttgagagggaaaagtcatctct : 130300
Seq2 : cttcacaagtgaaatgttgtcattatttcgacataaactcagataatagcaatattagctctcgtacagtagagatatttgagagggaaaagtcatctct : 130300
Seq3 : cttcacaagtgaaatgttgtcattatttcgacataaactcagataatagcaatattagctctcgtacagtagagatatttgagagggaaaagtcatctct : 130300
Seq4 : cttcacaagtgaaatgttgtcattatttcgacataaactcagataatagcaatattagctctcgtacagtagagatatttgagagggaaaagtcatctct : 130300

                 *    130320         *    130340         *    130360         *    130380         *    130400
Seq1 : tgtatcatatattaaaactaccaataagaagagaaaggtcaattacggcgaaataaagaaaactgttcatggaggcactaatgcaaattacttttccggt : 130400
Seq2 : tgtatcatatattaaaactaccaataagaagagaaaggtcaattacggcgaaataaagaaaactgttcatggaggcactaatgcaaattacttttccggt : 130400
Seq3 : tgtatcatatattaaaactaccaataagaagagaaaggtcaattacggcgaaataaagaaaactgttcatggaggcactaatgcaaattacttttccggt : 130400
Seq4 : tgtatcatatattaaaactaccaataagaagagaaaggtcaattacggcgaaataaagaaaactgttcatggaggcactaatgcaaattacttttccggt : 130400

                 *    130420         *    130440         *    130460         *    130480         *    130500
Seq1 : aaaaagtctgacgagtatctgagtactacagttagatccaacattaatcaaccttggatcaaaaccatttctaagagaatgagagtagatatcattaatc : 130500
Seq2 : aaaaagtctgacgagtatctgagtactacagttagatccaacattaatcaaccttggatcaaaaccatttctaagagaatgagagtagatatcattaatc : 130500
Seq3 : aaaaagtctgacgagtatctgagtactacagttagatccaacattaatcaaccttggatcaaaaccatttctaagagaatgagagtagatatcattaatc : 130500
Seq4 : aaaaagtctgacgagtatctgagtactacagttagatccaacattaatcaaccttggatcaaaaccatttctaagagaatgagagtagatatcattaatc : 130500

                 *    130520         *    130540         *    130560         *    130580         *    130600
Seq1 : actctatagtaacgcgtggaaaaagctctatattacaaactatagaaattatttttactaatagaacatgtgtgaaaatattcaaggattctactatgca : 130600
Seq2 : actctatagtaacgcgtggaaaaagctctatattacaaactatagaaattatttttactaatagaacatgtgtgaaaatattcaaggattctactatgca : 130600
Seq3 : actctatagtaacgcgtggaaaaagctctatattacaaactatagaaattatttttactaatagaacatgtgtgaaaatattcaaggattctactatgca : 130600
Seq4 : actctatagtaacgcgtggaaaaagctctatattacaaactatagaaattatttttactaatagaacatgtgtgaaaatattcaaggattctactatgca : 130600

                 *    130620         *    130640         *    130660         *    130680         *    130700
Seq1 : cattattctatccaaggacaaggatgaaaaggggtgtatacacatgattgacaaattattctatgtctattataatttatttctgttgttcgaagatatc : 130700
Seq2 : cattattctatccaaggacaaggatgaaaaggggtgtatacacatgattgacaaattattctatgtctattataatttatttctgttgttcgaagatatc : 130700
Seq3 : cattattctatccaaggacaaggatgaaaaggggtgtatacacatgattgacaaattattctatgtctattataatttatttctgttgttcgaagatatc : 130700
Seq4 : cattattctatccaaggacaaggatgaaaaggggtgtatacacatgattgacaaattattctatgtctattataatttatttctgttgttcgaagatatc : 130700

                 *    130720         *    130740         *    130760         *    130780         *    130800
Seq1 : atccaaaacgagtactttaaagaagtagctaatgttgtaaaccacgtacttacggctacggcattagatgagaaattattcctaattaagaaaatggctg : 130800
Seq2 : atccaaaacgagtactttaaagaagtagctaatgttgtaaaccacgtacttacggctacggcattagatgagaaattattcctaattaagaaaatggctg : 130800
Seq3 : atccaaaacgagtactttaaagaagtagctaatgttgtaaaccacgtacttacggctacggcattagatgagaaattattcctaattaagaaaatggctg : 130800
Seq4 : atccaaaacgagtactttaaagaagtagctaatgttgtaaaccacgtacttacggctacggcattagatgagaaattattcctaattaagaaaatggctg : 130800

                 *    130820         *    130840         *    130860         *    130880         *    130900
Seq1 : aacacgatgtttatggagttagcaatttcaaaatagggatgtttaacctgacatttattaagtcgttggatcataccgttttcccctctctgttagatga : 130900
Seq2 : aacacgatgtttatggagttagcaatttcaaaatagggatgtttaacctgacatttattaagtcgttggatcataccgttttcccctctctgttagatga : 130900
Seq3 : aacacgatgtttatggagttagcaatttcaaaatagggatgtttaacctgacatttattaagtcgttggatcataccgttttcccctctctgttagatga : 130900
Seq4 : aacacgatgtttatggagttagcaatttcaaaatagggatgtttaacctgacatttattaagtcgttggatcataccgttttcccctctctgttagatga : 130900
```

```
                *    130920         *    130940         *    130960         *    130980         *    131000
Seq1 : ggatagcaaaataaagttttttaaggggaaaaagctcaatattgtagcattacgatctctggaggattgtataaattacgtgactaaatccgagaatatg : 131000
Seq2 : ggatagcaaaataaagttttttaaggggaaaaagctcaatattgtagcattacgatctctggaggattgtataaattacgtgactaaatccgagaatatg : 131000
Seq3 : ggatagcaaaataaagttttttaaggggaaaaagctcaatattgtagcattacgatctctggaggattgtataaattacgtgactaaatccgagaatatg : 131000
Seq4 : ggatagcaaaataaagttttttaaggggaaaaagctcaatattgtagcattacgatctctggaggattgtataaattacgtgactaaatccgagaatatg : 131000

                *    131020         *    131040         *    131060         *    131080         *    131100
Seq1 : atagaaatgatgaaggaaagatcgactattttaaatagcatagatatagaaacggaatcggtagatcgtctaaaagaattgcttctaaaatgaaaaaaaa : 131100
Seq2 : atagaaatgatgaaggaaagatcgactattttaaatagcatagatatagaaacggaatcggtagatcgtctaaaagaattgcttctaaaatgaaaaaaaa : 131100
Seq3 : atagaaatgatgaaggaaagatcgactattttaaatagcatagatatagaaacggaatcggtagatcgtctaaaagaattgcttctaaaatgaaaaaaaa : 131100
Seq4 : atagaaatgatgaaggaaagatcgactattttaaatagcatagatatagaaacggaatcggtagatcgtctaaaagaattgcttctaaaatgaaaaaaaa : 131100

                *    131120         *    131140         *    131160         *    131180         *    131200
Seq1 : cactgattcagaaatggatcaacgactagggtataagtttttggtgcctgatcctaaagccggagttttttatagaccgttacatttccaatatgtatcg : 131200
Seq2 : cactgattcagaaatggatcaacgactagggtataagtttttggtgcctgatcctaaagccggagttttttatagaccgttacatttccaatatgtatcg : 131200
Seq3 : cactgattcagaaatggatcaacgactagggtataagtttttggtgcctgatcctaaagccggagttttttatagaccgttacatttccaatatgtatcg : 131200
Seq4 : cactgattcagaaatggatcaacgactagggtataagtttttggtgcctgatcctaaagccggagttttttatagaccgttacatttccaatatgtatcg : 131200

                *    131220         *    131240         *    131260         *    131280         *    131300
Seq1 : tattctaattttatattgcatcgattgcatgaaatcttgaccgtcaagcggccactcttatcgtttaagaataatacagaacgaattatgatagaaatta : 131300
Seq2 : tattctaattttatattgcatcgattgcatgaaatcttgaccgtcaagcggccactcttatcgtttaagaataatacagaacgaattatgatagaaatta : 131300
Seq3 : tattctaattttatattgcatcgattgcatgaaatcttgaccgtcaagcggccactcttatcgtttaagaataatacagaacgaattatgatagaaatta : 131300
Seq4 : tattctaattttatattgcatcgattgcatgaaatcttgaccgtcaagcggccactcttatcgtttaagaataatacagaacgaattatgatagaaatta : 131300

                *    131320         *    131340         *    131360         *    131380         *    131400
Seq1 : gcaatgttaaagtgactcctccagattactcacctataatcgcgagtattaaaggtaagagttatgatgcattagccacgttcactgtaaatatctttaa : 131400
Seq2 : gcaatgttaaagtgactcctccagattactcacctataatcgcgagtattaaaggtaagagttatgatgcattagccacgttcactgtaaatatctttaa : 131400
Seq3 : gcaatgttaaagtgactcctccagattactcacctataatcgcgagtattaaaggtaagagttatgatgcattagccacgttcactgtaaatatctttaa : 131400
Seq4 : gcaatgttaaagtgactcctccagattactcacctataatcgcgagtattaaaggtaagagttatgatgcattagccacgttcactgtaaatatctttaa : 131400

                *    131420         *    131440         *    131460         *    131480         *    131500
Seq1 : agaggtaatgaccaaagagggtatatccatcactaaaataagtagttatgagggaaaagattctcatttgataaaaattccgctactaataggatacggg : 131500
Seq2 : agaggtaatgaccaaagagggtatatccatcactaaaataagtagttatgagggaaaagattctcatttgataaaaattccgctactaataggatacggg : 131500
Seq3 : agaggtaatgaccaaagagggtatatccatcactaaaataagtagttatgagggaaaagattctcatttgataaaaattccgctactaataggatacggg : 131500
Seq4 : agaggtaatgaccaaagagggtatatccatcactaaaataagtagttatgagggaaaagattctcatttgataaaaattccgctactaataggatacggg : 131500

                *    131520         *    131540         *    131560         *    131580         *    131600
Seq1 : aataaaaatccacttgatacagccaagtatcttgttcctaatgtcataggtggagtctttatcaataaacaatctgtcgaaaaagtaggaattaatctag : 131600
Seq2 : aataaaaatccacttgatacagccaagtatcttgttcctaatgtcataggtggagtctttatcaataaacaatctgtcgaaaaagtaggaattaatctag : 131600
Seq3 : aataaaaatccacttgatacagccaagtatcttgttcctaatgtcataggtggagtctttatcaataaacaatctgtcgaaaaagtaggaattaatctag : 131600
Seq4 : aataaaaatccacttgatacagccaagtatcttgttcctaatgtcataggtggagtctttatcaataaacaatctgtcgaaaaagtaggaattaatctag : 131600
```

```
         *    131620         *    131640         *    131660         *    131680         *    131700
Seq1 : tagaaaagattacaacatggccaaaatttagggttgttaagccaaactcattcactttctcgttttcctccgtatcccctcctaatgtattaccgacaag : 131700
Seq2 : tagaaaagattacaacatggccaaaatttagggttgttaagccaaactcattcactttctcgttttcctccgtatcccctcctaatgtattaccgacaag : 131700
Seq3 : tagaaaagattacaacatggccaaaatttagggttgttaagccaaactcattcactttctcgttttcctccgtatcccctcctaatgtattaccgacaag : 131700
Seq4 : tagaaaagattacaacatggccaaaatttagggttgttaagccaaactcattcactttctcgttttcctccgtatcccctcctaatgtattaccgacaag : 131700

         *    131720         *    131740         *    131760         *    131780         *    131800
Seq1 : atatcgccattacaagatatctctggatatatcacaattggaagcgttgaatatatcatcgacaaagacatttataacggtcaatattgttttgctgtct : 131800
Seq2 : atatcgccattacaagatatctctggatatatcacaattggaagcgttgaatatatcatcgacaaagacatttataacggtcaatattgttttgctgtct : 131800
Seq3 : atatcgccattacaagatatctctggatatatcacaattggaagcgttgaatatatcatcgacaaagacatttataacggtcaatattgttttgctgtct : 131800
Seq4 : atatcgccattacaagatatctctggatatatcacaattggaagcgttgaatatatcatcgacaaagacatttataacggtcaatattgttttgctgtct : 131800

         *    131820         *    131840         *    131860         *    131880         *    131900
Seq1 : caatatttatctagagtgagtctagaattcattagacgtagtttatcatacgatatgcctccagaagttgtctatctagtaaacgcgataatagatagtg : 131900
Seq2 : caatatttatctagagtgagtctagaattcattagacgtagtttatcatacgatatgcctccagaagttgtctatctagtaaacgcgataatagatagtg : 131900
Seq3 : caatatttatctagagtgagtctagaattcattagacgtagtttatcatacgatatgcctccagaagttgtctatctagtaaacgcgataatagatagtg : 131900
Seq4 : caatatttatctagagtgagtctagaattcattagacgtagtttatcatacgatatgcctccagaagttgtctatctagtaaacgcgataatagatagtg : 131900

         *    131920         *    131940         *    131960         *    131980         *    132000
Seq1 : ctaaacgaattactgaatctattactgactttaatattgatacatacattaatgacctggtggaagctgaacacattaaacaaaaatctcagttaacgat : 132000
Seq2 : ctaaacgaattactgaatctattactgactttaatattgatacatacattaatgacctggtggaagctgaacacattaaacaaaaatctcagttaacgat : 132000
Seq3 : ctaaacgaattactgaatctattactgactttaatattgatacatacattaatgacctggtggaagctgaacacattaaacaaaaatctcagttaacgat : 132000
Seq4 : ctaaacgaattactgaatctattactgactttaatattgatacatacattaatgacctggtggaagctgaacacattaaacaaaaatctcagttaacgat : 132000

         *    132020         *    132040         *    132060         *    132080         *    132100
Seq1 : caacgagttcaaatatgaaatgctgcataactttttacctcatatgaactatacacccgatcaactaaagggattttatatgatatctttactaagaaag : 132100
Seq2 : caacgagttcaaatatgaaatgctgcataactttttacctcatatgaactatacacccgatcaactaaagggattttatatgatatctttactaagaaag : 132100
Seq3 : caacgagttcaaatatgaaatgctgcataactttttacctcatatgaactatacacccgatcaactaaagggattttatatgatatctttactaagaaag : 132100
Seq4 : caacgagttcaaatatgaaatgctgcataactttttacctcatatgaactatacacccgatcaactaaagggattttatatgatatctttactaagaaag : 132100

         *    132120         *    132140         *    132160         *    132180         *    132200
Seq1 : tttctctactgtatctaccacacttctagatatccagatagagattcgatggtttgtcatcgcatcctaacgtacggcaaatattttgagacgttggcac : 132200
Seq2 : tttctctactgtatctaccacacttctagatatccagatagagattcgatggtttgtcatcgcatcctaacgtacggcaaatattttgagacgttggcac : 132200
Seq3 : tttctctactgtatctaccacacttctagatatccagatagagattcgatggtttgtcatcgcatcctaacgtacggcaaatattttgagacgttggcac : 132200
Seq4 : tttctctactgtatctaccacacttctagatatccagatagagattcgatggtttgtcatcgcatcctaacgtacggcaaatattttgagacgttggcac : 132200

         *    132220         *    132240         *    132260         *    132280         *    132300
Seq1 : atgatgaattagagaattacataggcaacatccgaaacgatatcatgaacaatcacaagaacagaggcacttacgcggtaaacattcatgtactaacaac : 132300
Seq2 : atgatgaattagagaattacataggcaacatccgaaacgatatcatgaacaatcacaagaacagaggcacttacgcggtaaacattcatgtactaacaac : 132300
Seq3 : atgatgaattagagaattacataggcaacatccgaaacgatatcatgaacaatcacaagaacagaggcacttacgcggtaaacattcatgtactaacaac : 132300
Seq4 : atgatgaattagagaattacataggcaacatccgaaacgatatcatgaacaatcacaagaacagaggcacttacgcggtaaacattcatgtactaacaac : 132300
```

```
                *     132320          *     132340          *     132360          *     132380          *     132400
Seq1 : tcccggacttaatcacgcgttttctagcttattgagtggaaagttcaaaaagtcagacggtagttatcgaacacatcctcactattcatggatgcagaat : 132400
Seq2 : tcccggacttaatcacgcgttttctagcttattgagtggaaagttcaaaaagtcagacggtagttatcgaacacatcctcactattcatggatgcagaat : 132400
Seq3 : tcccggacttaatcacgcgttttctagcttattgagtggaaagttcaaaaagtcagacggtagttatcgaacacatcctcactattcatggatgcagaat : 132400
Seq4 : tcccggacttaatcacgcgttttctagcttattgagtggaaagttcaaaaagtcagacggtagttatcgaacacatcctcactattcatggatgcagaat : 132400

                *     132420          *     132440          *     132460          *     132480          *     132500
Seq1 : atttctattcctaggagtgttggattttatccggatcaagtaaagatttcaaagatgttttctgtcagaaaataccatccaagtcaatatctttactttt : 132500
Seq2 : atttctattcctaggagtgttggattttatccggatcaagtaaagatttcaaagatgttttctgtcagaaaataccatccaagtcaatatctttactttt : 132500
Seq3 : atttctattcctaggagtgttggattttatccggatcaagtaaagatttcaaagatgttttctgtcagaaaataccatccaagtcaatatctttactttt : 132500
Seq4 : atttctattcctaggagtgttggattttatccggatcaagtaaagatttcaaagatgttttctgtcagaaaataccatccaagtcaatatctttactttt : 132500

                *     132520          *     132540          *     132560          *     132580          *     132600
Seq1 : gttcatcggacgttccggaaagaggtcctcaggtaggtttagtatctcaattgtctgtcttgagttccattacaaatatactaacgtctgagtatttgga : 132600
Seq2 : gttcatcggacgttccggaaagaggtcctcaggtaggtttagtatctcaattgtctgtcttgagttccattacaaatatactaacgtctgagtatttgga : 132600
Seq3 : gttcatcggacgttccggaaagaggtcctcaggtaggtttagtatctcaattgtctgtcttgagttccattacaaatatactaacgtctgagtatttgga : 132600
Seq4 : gttcatcggacgttccggaaagaggtcctcaggtaggtttagtatctcaattgtctgtcttgagttccattacaaatatactaacgtctgagtatttgga : 132600

                *     132620          *     132640          *     132660          *     132680          *     132700
Seq1 : tttggaaaagaaaatttgtgagtatatcagatcatattataaagatgatataagttactttgaaacaggatttccaatcactatagaaaatgctctagtc : 132700
Seq2 : tttggaaaagaaaatttgtgagtatatcagatcatattataaagatgatataagttactttgaaacaggatttccaatcactatagaaaatgctctagtc : 132700
Seq3 : tttggaaaagaaaatttgtgagtatatcagatcatattataaagatgatataagttactttgaaacaggatttccaatcactatagaaaatgctctagtc : 132700
Seq4 : tttggaaaagaaaatttgtgagtatatcagatcatattataaagatgatataagttactttgaaacaggatttccaatcactatagaaaatgctctagtc : 132700

                *     132720          *     132740          *     132760          *     132780          *     132800
Seq1 : gcatctcttaatccaaatatgatatgtgatttgtaactgactttagacgtagaaaacggatgggattcttcggtaacttggaggtaggtattactttag : 132800
Seq2 : gcatctcttaatccaaatatgatatgtgatttgtaactgactttagacgtagaaaacggatgggattcttcggtaacttggaggtaggtattactttag : 132800
Seq3 : gcatctcttaatccaaatatgatatgtgatttgtaactgactttagacgtagaaaacggatgggattcttcggtaacttggaggtaggtattactttag : 132800
Seq4 : gcatctcttaatccaaatatgatatgtgatttgtaactgactttagacgtagaaaacggatgggattcttcggtaacttggaggtaggtattactttag : 132800

                *     132820          *     132840          *     132860          *     132880          *     132900
Seq1 : ttagggatcacatgaatgaaattcgcattaatattggagcgggaagattagtcagaccattcttggttgtggataacggagagctcatgatggatgtgtg : 132900
Seq2 : ttagggatcacatgaatgaaattcgcattaatattggagcgggaagattagtcagaccattcttggttgtggataacggagagctcatgatggatgtgtg : 132900
Seq3 : ttagggatcacatgaatgaaattcgcattaatattggagcgggaagattagtcagaccattcttggttgtggataacggagagctcatgatggatgtgtg : 132900
Seq4 : ttagggatcacatgaatgaaattcgcattaatattggagcgggaagattagtcagaccattcttggttgtggataacggagagctcatgatggatgtgtg : 132900

                *     132920          *     132940          *     132960          *     132980          *     133000
Seq1 : tccggagttagaaagcagattagacgacatgacattctctgacattcagaaagagtttccgcatgtcatcgaaatggtagatatagaacaatttactttt : 133000
Seq2 : tccggagttagaaagcagattagacgacatgacattctctgacattcagaaagagtttccgcatgtcatcgaaatggtagatatagaacaatttactttt : 133000
Seq3 : tccggagttagaaagcagattagacgacatgacattctctgacattcagaaagagtttccgcatgtcatcgaaatggtagatatagaacaatttactttt : 133000
Seq4 : tccggagttagaaagcagattagacgacatgacattctctgacattcagaaagagtttccgcatgtcatcgaaatggtagatatagaacaatttactttt : 133000
```

```
                 *    133020         *    133040         *    133060         *    133080         *    133100
Seq1 : agtaacgtatgtgaatcggttcaaaaatttagaatgatgtcaaaggatgaaagaaagcaatacgatttatgtgactttcctgccgaatttagagatggat : 133100
Seq2 : agtaacgtatgtgaatcggttcaaaaatttagaatgatgtcaaaggatgaaagaaagcaatacgatttatgtgactttcctgccgaatttagagatggat : 133100
Seq3 : agtaacgtatgtgaatcggttcaaaaatttagaatgatgtcaaaggatgaaagaaagcaatacgatttatgtgactttcctgccgaatttagagatggat : 133100
Seq4 : agtaacgtatgtgaatcggttcaaaaatttagaatgatgtcaaaggatgaaagaaagcaatacgatttatgtgactttcctgccgaatttagagatggat : 133100

                 *    133120         *    133140         *    133160         *    133180         *    133200
Seq1 : atgtggcatcttcattagtgggaatcaatcacaattctggacccagagctattcttggatgtgctcaagctaaacaagctatctcttgtctgagttcgga : 133200
Seq2 : atgtggcatcttcattagtgggaatcaatcacaattctggacccagagctattcttggatgtgctcaagctaaacaagctatctcttgtctgagttcgga : 133200
Seq3 : atgtggcatcttcattagtgggaatcaatcacaattctggacccagagctattcttggatgtgctcaagctaaacaagctatctcttgtctgagttcgga : 133200
Seq4 : atgtggcatcttcattagtgggaatcaatcacaattctggacccagagctattcttggatgtgctcaagctaaacaagctatctcttgtctgagttcgga : 133200

                 *    133220         *    133240         *    133260         *    133280         *    133300
Seq1 : tatacgaaataaaatagacaatggaattcatttgatgtatccagagaggccaatcgtgattagtaaggctttagaaacttcaaagattgcggctaattgc : 133300
Seq2 : tatacgaaataaaatagacaatggaattcatttgatgtatccagagaggccaatcgtgattagtaaggctttagaaacttcaaagattgcggctaattgc : 133300
Seq3 : tatacgaaataaaatagacaatggaattcatttgatgtatccagagaggccaatcgtgattagtaaggctttagaaacttcaaagattgcggctaattgc : 133300
Seq4 : tatacgaaataaaatagacaatggaattcatttgatgtatccagagaggccaatcgtgattagtaaggctttagaaacttcaaagattgcggctaattgc : 133300

                 *    133320         *    133340         *    133360         *    133380         *    133400
Seq1 : ttcggccaacatgttactatagcattaatgtcgtacaaaggtatcaatcaagaggatggaattatcatcaaaaaacaatttattcagagaggcggtctcg : 133400
Seq2 : ttcggccaacatgttactatagcattaatgtcgtacaaaggtatcaatcaagaggatggaattatcatcaaaaaacaatttattcagagaggcggtctcg : 133400
Seq3 : ttcggccaacatgttactatagcattaatgtcgtacaaaggtatcaatcaagaggatggaattatcatcaaaaaacaatttattcagagaggcggtctcg : 133400
Seq4 : ttcggccaacatgttactatagcattaatgtcgtacaaaggtatcaatcaagaggatggaattatcatcaaaaaacaatttattcagagaggcggtctcg : 133400

                 *    133420         *    133440         *    133460         *    133480         *    133500
Seq1 : atatagttaccgcaaagaaacatcaagtagaaattccgttggaaaactttaataacaaagaaagagataggtctaacgcctattcaaaattagaaagtaa : 133500
Seq2 : atatagttaccgcaaagaaacatcaagtagaaattccgttggaaaactttaataacaaagaaagagataggtctaacgcctattcaaaattagaaagtaa : 133500
Seq3 : atatagttaccgcaaagaaacatcaagtagaaattccgttggaaaactttaataacaaagaaagagataggtctaacgcctattcaaaattagaaagtaa : 133500
Seq4 : atatagttaccgcaaagaaacatcaagtagaaattccgttggaaaactttaataacaaagaaagagataggtctaacgcctattcaaaattagaaagtaa : 133500

                 *    133520         *    133540         *    133560         *    133580         *    133600
Seq1 : tggattagttagactgaatgctttcttggaatccggagacgctatggcacgaaatatctcatcaagaactcttgaagatgattttgctagagataatcag : 133600
Seq2 : tggattagttagactgaatgctttcttggaatccggagacgctatggcacgaaatatctcatcaagaactcttgaagatgattttgctagagataatcag : 133600
Seq3 : tggattagttagactgaatgctttcttggaatccggagacgctatggcacgaaatatctcatcaagaactcttgaagatgattttgctagagataatcag : 133600
Seq4 : tggattagttagactgaatgctttcttggaatccggagacgctatggcacgaaatatctcatcaagaactcttgaagatgattttgctagagataatcag : 133600

                 *    133620         *    133640         *    133660         *    133680         *    133700
Seq1 : attagcttcgatgtttccgagaaatataccgatatgtacaaatctcgcgttgaacgagtacaagtagaacttactgacaaagttaaggtacgagtattaa : 133700
Seq2 : attagcttcgatgtttccgagaaatataccgatatgtacaaatctcgcgttgaacgagtacaagtagaacttactgacaaagttaaggtacgagtattaa : 133700
Seq3 : attagcttcgatgtttccgagaaatataccgatatgtacaaatctcgcgttgaacgagtacaagtagaacttactgacaaagttaaggtacgagtattaa : 133700
Seq4 : attagcttcgatgtttccgagaaatataccgatatgtacaaatctcgcgttgaacgagtacaagtagaacttactgacaaagttaaggtacgagtattaa : 133700
```

```
                 *    133720         *    133740         *    133760         *    133780         *    133800
Seq1 : ccatgaaagaaagaagacccattctaggagataaatttaccactagaacgagtcaaaagggaacagtcgcgtatgtcgcggatgaaacggaacttccata : 133800
Seq2 : ccatgaaagaaagaagacccattctaggagataaatttaccactagaacgagtcaaaagggaacagtcgcgtatgtcgcggatgaaacggaacttccata : 133800
Seq3 : ccatgaaagaaagaagacccattctaggagataaatttaccactagaacgagtcaaaagggaacagtcgcgtatgtcgcggatgaaacggaacttccata : 133800
Seq4 : ccatgaaagaaagaagacccattctaggagataaatttaccactagaacgagtcaaaagggaacagtcgcgtatgtcgcggatgaaacggaacttccata : 133800

                 *    133820         *    133840         *    133860         *    133880         *    133900
Seq1 : cgacgaaaatggtatcacaccagatgtcattattaattctacatccatcttctctagaaaaactatatctatgttgatagaggttattttaacagccgca : 133900
Seq2 : cgacgaaaatggtatcacaccagatgtcattattaattctacatccatcttctctagaaaaactatatctatgttgatagaggttattttaacagccgca : 133900
Seq3 : cgacgaaaatggtatcacaccagatgtcattattaattctacatccatcttctctagaaaaactatatctatgttgatagaggttattttaacagccgca : 133900
Seq4 : cgacgaaaatggtatcacaccagatgtcattattaattctacatccatcttctctagaaaaactatatctatgttgatagaggttattttaacagccgca : 133900

                 *    133920         *    133940         *    133960         *    133980         *    134000
Seq1 : tattctgctaagccgtacaacaataagggagaaaaccgacctgtctgttttcctagtagtaacgaaacatccatcgatacatatatgcaattcgctaaac : 134000
Seq2 : tattctgctaagccgtacaacaataagggagaaaaccgacctgtctgttttcctagtagtaacgaaacatccatcgatacatatatgcaattcgctaaac : 134000
Seq3 : tattctgctaagccgtacaacaataagggagaaaaccgacctgtctgttttcctagtagtaacgaaacatccatcgatacatatatgcaattcgctaaac : 134000
Seq4 : tattctgctaagccgtacaacaataagggagaaaaccgacctgtctgttttcctagtagtaacgaaacatccatcgatacatatatgcaattcgctaaac : 134000

                 *    134020         *    134040         *    134060         *    134080         *    134100
Seq1 : aatgttatgagcattcaaatccgaaattgtccgatgaagaattatcggataaaatcttttgtgaaaagattctctatgatcctgaaacggataagcctta : 134100
Seq2 : aatgttatgagcattcaaatccgaaattgtccgatgaagaattatcggataaaatcttttgtgaaaagattctctatgatcctgaaacggataagcctta : 134100
Seq3 : aatgttatgagcattcaaatccgaaattgtccgatgaagaattatcggataaaatcttttgtgaaaagattctctatgatcctgaaacggataagcctta : 134100
Seq4 : aatgttatgagcattcaaatccgaaattgtccgatgaagaattatcggataaaatcttttgtgaaaagattctctatgatcctgaaacggataagcctta : 134100

                 *    134120         *    134140         *    134160         *    134180         *    134200
Seq1 : tgcatccaaagtatttttttggaccaatttattacttgcgtctgaggcatttaactcaggacaaggcaaccgttagatgtagaggtaaaaagacgaagctc : 134200
Seq2 : tgcatccaaagtatttttttggaccaatttattacttgcgtctgaggcatttaactcaggacaaggcaaccgttagatgtagaggtaaaaagacgaagctc : 134200
Seq3 : tgcatccaaagtatttttttggaccaatttattacttgcgtctgaggcatttaactcaggacaaggcaaccgttagatgtagaggtaaaaagacgaagctc : 134200
Seq4 : tgcatccaaagtatttttttggaccaatttattacttgcgtctgaggcatttaactcaggacaaggcaaccgttagatgtagaggtaaaaagacgaagctc : 134200

                 *    134220         *    134240         *    134260         *    134280         *    134300
Seq1 : attagacaggcgaatgagggacgaaaacgtggaggaggtatcaagttcggagaaatggagagagactgtttaatagcgcatggcgcagccaatactatta : 134300
Seq2 : attagacaggcgaatgagggacgaaaacgtggaggaggtatcaagttcggagaaatggagagagactgtttaatagcgcatggcgcagccaatactatta : 134300
Seq3 : attagacaggcgaatgagggacgaaaacgtggaggaggtatcaagttcggagaaatggagagagactgtttaatagcgcatggcgcagccaatactatta : 134300
Seq4 : attagacaggcgaatgagggacgaaaacgtggaggaggtatcaagttcggagaaatggagagagactgtttaatagcgcatggcgcagccaatactatta : 134300

                 *    134320         *    134340         *    134360         *    134380         *    134400
Seq1 : cagaagttttaaaagactcagaagaggattatcaagatgtgtatgtttgtgaaaattgtggagacatagcagcacaaatcaagggtattaatacatgtct : 134400
Seq2 : cagaagttttaaaagactcagaagaggattatcaagatgtgtatgtttgtgaaaattgtggagacatagcagcacaaatcaagggtattaatacatgtct : 134400
Seq3 : cagaagttttaaaagactcagaagaggattatcaagatgtgtatgtttgtgaaaattgtggagacatagcagcacaaatcaagggtattaatacatgtct : 134400
Seq4 : cagaagttttaaaagactcagaagaggattatcaagatgtgtatgtttgtgaaaattgtggagacatagcagcacaaatcaagggtattaatacatgtct : 134400
```

```
              *    134420          *    134440          *    134460          *    134480          *    134500
Seq1 : tagatgttcaaaacttaatctctctcctctcttaacaaaaattgataccacgcacgtatctaaagtatttcttactcaaatgaacgccagaggcgtaaaa : 134500
Seq2 : tagatgttcaaaacttaatctctctcctctcttaacaaaaattgataccacgcacgtatctaaagtatttcttactcaaatgaacgccagaggcgtaaaa : 134500
Seq3 : tagatgttcaaaacttaatctctctcctctcttaacaaaaattgataccacgcacgtatctaaagtatttcttactcaaatgaacgccagaggcgtaaaa : 134500
Seq4 : tagatgttcaaaacttaatctctctcctctcttaacaaaaattgataccacgcacgtatctaaagtatttcttactcaaatgaacgccagaggcgtaaaa : 134500

              *    134520          *    134540          *    134560          *    134580          *    134600
Seq1 : gtcaaattagatttcgaacgaaggcctccttcgttttataaaccattagataaagttgatctcaagccgtcttttctggtgtaatattctagtttggtag : 134600
Seq2 : gtcaaattagatttcgaacgaaggcctccttcgttttataaaccattagataaagttgatctcaagccgtcttttctggtgtaatattctagtttggtag : 134600
Seq3 : gtcaaattagatttcgaacgaaggcctccttcgttttataaaccattagataaagttgatctcaagccgtcttttctggtgtaatattctagtttggtag : 134600
Seq4 : gtcaaattagatttcgaacgaaggcctccttcgttttataaaccattagataaagttgatctcaagccgtcttttctggtgtaatattctagtttggtag : 134600

              *    134620          *    134640          *    134660          *    134680          *    134700
Seq1 : tagatacatatcaatatcatcaaattcgagatccgaattataaaatgggcgtggattgttaactatagaatcggacgtctgatattcgaaaatctgtgga : 134700
Seq2 : tagatacatatcaatatcatcaaattcgagatccgaattataaaatgggcgtggattgttaactatagaatcggacgtctgatattcgaaaatctgtgga : 134700
Seq3 : tagatacatatcaatatcatcaaattcgagatccgaattataaaatgggcgtggattgttaactatagaatcggacgtctgatattcgaaaatctgtgga : 134700
Seq4 : tagatacatatcaatatcatcaaattcgagatccgaattataaaatgggcgtggattgttaactatagaatcggacgtctgatattcgaaaatctgtgga : 134700

              *    134720          *    134740          *    134760          *    134780          *    134800
Seq1 : gttttaggttttggtggaggtgtaactgctacttgggatactgaagtctgatattcagaaagctgggggatgttctggttcgacatccaccgatggtgtc : 134800
Seq2 : gttttaggttttggtggaggtgtaactgctacttgggatactgaagtctgatattcagaaagctgggggatgttctggttcgacatccaccgatggtgtc : 134800
Seq3 : gttttaggttttggtggaggtgtaactgctacttgggatactgaagtctgatattcagaaagctgggggatgttctggttcgacatccaccgatggtgtc : 134800
Seq4 : gttttaggttttggtggaggtgtaactgctacttgggatactgaagtctgatattcagaaagctgggggatgttctggttcgacatccaccgatggtgtc : 134800

              *    134820          *    134840          *    134860          *    134880          *    134900
Seq1 : acatcactaatctgttcggtaacgtctgtggatggaggtgctacttctacagaacctgtagcctcagttgtcaacggagatacatttttaatgcgaggaa : 134900
Seq2 : acatcactaatctgttcggtaacgtctgtggatggaggtgctacttctacagaacctgtagcctcagttgtcaacggagatacatttttaatgcgaggaa : 134900
Seq3 : acatcactaatctgttcggtaacgtctgtggatggaggtgctacttctacagaacctgtagcctcagttgtcaacggagatacatttttaatgcgaggaa : 134900
Seq4 : acatcactaatctgttcggtaacgtctgtggatggaggtgctacttctacagaacctgtagcctcagttgtcaacggagatacatttttaatgcgaggaa : 134900

              *    134920          *    134940          *    134960          *    134980          *    135000
Seq1 : atgtataatttggtaatggtttctcatgtggatctgaagaagaggtaagatatctactagaaagataccgatcacgttctagttctcttttgtagaactt : 135000
Seq2 : atgtataatttggtaatggtttctcatgtggatctgaagaagaggtaagatatctactagaaagataccgatcacgttctagttctcttttgtagaactt : 135000
Seq3 : atgtataatttggtaatggtttctcatgtggatctgaagaagaggtaagatatctactagaaagataccgatcacgttctagttctcttttgtagaactt : 135000
Seq4 : atgtataatttggtaatggtttctcatgtggatctgaagaagaggtaagatatctactagaaagataccgatcacgttctagttctcttttgtagaactt : 135000

              *    135020          *    135040          *    135060          *    135080          *    135100
Seq1 : aactttttctttctcagcatctagttgatattccaacctcttcacgttactacgttcagattccaattcacgttcgcatgggttacctccgcagttttta : 135100
Seq2 : aactttttctttctcagcatctagttgatattccaacctcttcacgttactacgttcagattccaattcacgttcgcatgggttacctccgcagttttta : 135100
Seq3 : aactttttctttctcagcatctagttgatattccaacctcttcacgttactacgttcagattccaattcacgttcgcatgggttacctccgcagttttta : 135100
Seq4 : aactttttctttctcagcatctagttgatattccaacctcttcacgttactacgttcagattccaattcacgttcgcatgggttacctccgcagttttta : 135100
```

```
              *    135120          *    135140          *    135160          *    135180          *    135200
Seq1 : cgagcgatttcacgttcagccttcatgcgtctctccttctctctatcgagtttatcagagcagtctttctgaaggcgatcgaactccataaatttctcca : 135200
Seq2 : cgagcgatttcacgttcagccttcatgcgtctctccttctctctatcgagtttatcagagcagtctttctgaaggcgatcgaactccataaatttctcca : 135200
Seq3 : cgagcgatttcacgttcagccttcatgcgtctctccttctctctatcgagtttatcagagcagtctttctgaaggcgatcgaactccataaatttctcca : 135200
Seq4 : cgagcgatttcacgttcagccttcatgcgtctctccttctctctatcgagtttatcagagcagtctttctgaaggcgatcgaactccataaatttctcca : 135200

              *    135220          *    135240          *    135260          *    135280          *    135300
Seq1 : acgctttgattgtttccatagatttccgaagttcagcttttaggactgtgattctttttctttcgaattcacagctggatgtgcaaccgtttccattacc : 135300
Seq2 : acgctttgattgtttccatagatttccgaagttcagcttttaggactgtgattctttttctttcgaattcacagctggatgtgcaaccgtttccattacc : 135300
Seq3 : acgctttgattgtttccatagatttccgaagttcagcttttaggactgtgattctttttctttcgaattcacagctggatgtgcaaccgtttccattacc : 135300
Seq4 : acgctttgattgtttccatagatttccgaagttcagcttttaggactgtgattctttttctttcgaattcacagctggatgtgcaaccgtttccattacc : 135300

              *    135320          *    135340          *    135360          *    135380          *    135400
Seq1 : gccatctctaagtttcttttctagatcggcaacatttcatccccatgccttttacattcctcgagtctactgtcgtcgaaatatcgttccagctcctttt : 135400
Seq2 : gccatctctaagtttcttttctagatcggcaacatttcatccccatgccttttacattcctcgagtctactgtcgtcgaaatatcgttccagctcctttt : 135400
Seq3 : gccatctctaagtttcttttctagatcggcaacatttcatccccatgccttttacattcctcgagtctactgtcgtcgaaatatcgttccagctcctttt : 135400
Seq4 : gccatctctaagtttcttttctagatcggcaacatttcatccccatgccttttacattcctcgagtctactgtcgtcgaaatatcgttccagctcctttt : 135400

              *    135420          *    135440          *    135460          *    135480          *    135500
Seq1 : cgacatcaataactttagcacgttgtctctcaagctctcttttgtagttatctgattccctggcacgtttaagatcttcatgcaattgagtcagctctta : 135500
Seq2 : cgacatcaataactttagcacgttgtctctcaagctctcttttgtagttatctgattccctggcacgtttaagatcttcatgcaattgagtcagctctta : 135500
Seq3 : cgacatcaataactttagcacgttgtctctcaagctctcttttgtagttatctgattccctggcacgtttaagatcttcatgcaattgagtcagctctta : 135500
Seq4 : cgacatcaataactttagcacgttgtctctcaagctctcttttgtagttatctgattccctggcacgtttaagatcttcatgcaattgagtcagctctta : 135500

              *    135520          *    135540          *    135560          *    135580          *    135600
Seq1 : acacaatctcttgcttcttcgtcatagtacttacaatcactatgggatccattgttaccacgtctacactcggcgagctcgcgtttaagagattcaattt : 135600
Seq2 : acacaatctcttgcttcttcgtcatagtacttacaatcactatgggatccattgttaccacgtctacactcggcgagctcgcgtttaagagattcaattt : 135600
Seq3 : acacaatctcttgcttcttcgtcatagtacttacaatcactatgggatccattgttaccacgtctacactcggcgagctcgcgtttaagagattcaattt : 135600
Seq4 : acacaatctcttgcttcttcgtcatagtacttacaatcactatgggatccattgttaccacgtctacactcggcgagctcgcgtttaagagattcaattt : 135600

              *    135620          *    135640          *    135660          *    135680          *    135700
Seq1 : cccgtttgtattggtccatgtttccattgctaccaccattagatttacaggctgctagttgtcgttcgagatcagaaatacgggtttcttggaattgat : 135700
Seq2 : cccgtttgtattggtccatgtttccattgctaccaccattagatttacaggctgctagttgtcgttcgagatcagaaatacgggtttcttggaattgat : 135700
Seq3 : cccgtttgtattggtccatgtttccattgctaccaccattagatttacaggctgctagttgtcgttcgagatcagaaatacgggtttcttggaattgat : 135700
Seq4 : cccgtttgtattggtccatgtttccattgctaccaccattagatttacaggctgctagttgtcgttcgagatcagaaatacgggtttcttggaattgat : 135700

              *    135720          *    135740          *    135760          *    135780          *    135800
Seq1 : ttcgtcgatgtacttggcatcgaaacacttattaagttctttttccaattctacgatttatttctttcgcgagtcaattccctcctgtagtaactatct : 135800
Seq2 : ttcgtcgatgtacttggcatcgaaacacttattaagttctttttccaattctacgatttatttctttcgcgagtcaattccctcctgtagtaactatct : 135800
Seq3 : ttcgtcgatgtacttggcatcgaaacacttattaagttctttttccaattctacgatttatttctttcgcgagtcaattccctcctgtagtaactatct : 135800
Seq4 : ttcgtcgatgtacttggcatcgaaacacttattaagttctttttccaattctacgatttatttctttcgcgagtcaattccctcctgtagtaactatct : 135800
```

```
               *    135820         *    135840         *    135860         *    135880         *    135900
Seq1 : gttttgtcagattcacgctctctacgtagactttcttgcaagttactaatttgttccctagcacgtccgagtttagttttatatgctgaatagagttctg : 135900
Seq2 : gttttgtcagattcacgctctctacgtagactttcttgcaagttactaatttgttccctagcacgtccgagtttagttttatatgctgaatagagttctg : 135900
Seq3 : gttttgtcagattcacgctctctacgtagactttcttgcaagttactaatttgttccctagcacgtccgagtttagttttatatgctgaatagagttctg : 135900
Seq4 : gttttgtcagattcacgctctctacgtagactttcttgcaagttactaatttgttccctagcacgtccgagtttagttttatatgctgaatagagttctg : 135900

               *    135920         *    135940         *    135960         *    135980         *    136000
Seq1 : attcatcctttgagcagatctctagcgatcgtttaagattcctgattctagtctttagcctatttacctcctcagaagatgttccgttaccgttgcgttt : 136000
Seq2 : attcatcctttgagcagatctctagcgatcgtttaagattcctgattctagtctttagcctatttacctcctcagaagatgttccgttaccgttgcgttt : 136000
Seq3 : attcatcctttgagcagatctctagcgatcgtttaagattcctgattctagtctttagcctatttacctcctcagaagatgttccgttaccgttgcgttt : 136000
Seq4 : attcatcctttgagcagatctctagcgatcgtttaagattcctgattctagtctttagcctatttacctcctcagaagatgttccgttaccgttgcgttt : 136000

               *    136020         *    136040         *    136060         *    136080         *    136100
Seq1 : acactcgttaagctgtctatcaagatccatgattctatctctaagacgttgcatctctctttccatatcagcattgctttcattattacgtctgcagtca : 136100
Seq2 : acactcgttaagctgtctatcaagatccatgattctatctctaagacgttgcatctctctttccatatcagcattgctttcattattacgtctgcagtca : 136100
Seq3 : acactcgttaagctgtctatcaagatccatgattctatctctaagacgttgcatctctctttccatatcagcattgctttcattattacgtctgcagtca : 136100
Seq4 : acactcgttaagctgtctatcaagatccatgattctatctctaagacgttgcatctctctttccatatcagcattgctttcattattacgtctgcagtca : 136100

               *    136120         *    136140         *    136160         *    136180         *    136200
Seq1 : ctcaactgtctttcaatatctgagattctatctctaagacgtcgcatctctctctgtttcggcattggtttcattattacgtctacagtcgttcaactgt : 136200
Seq2 : ctcaactgtctttcaatatctgagattctatctctaagacgtcgcatctctctctgtttcggcattggtttcattattacgtctacagtcgttcaactgt : 136200
Seq3 : ctcaactgtctttcaatatctgagattctatctctaagacgtcgcatctctctctgtttcggcattggtttcattattacgtctacagtcgttcaactgt : 136200
Seq4 : ctcaactgtctttcaatatctgagattctatctctaagacgtcgcatctctctctgtttcggcattggtttcattattacgtctacagtcgttcaactgt : 136200

               *    136220         *    136240         *    136260         *    136280         *    136300
Seq1 : ctttcaagatctgatattctagattggagtctgctaatctctgtagcattttcacggcattcactcagttgtctttcaagatctgagattttagattgga : 136300
Seq2 : ctttcaagatctgatattctagattggagtctgctaatctctgtagcattttcacggcattcactcagttgtctttcaagatctgagattttagattgga : 136300
Seq3 : ctttcaagatctgatattctagattggagtctgctaatctctgtagcattttcacggcattcactcagttgtctttcaagatctgagattttagattgga : 136300
Seq4 : ctttcaagatctgatattctagattggagtctgctaatctctgtagcattttcacggcattcactcagttgtctttcaagatctgagattttagattgga : 136300

               *    136320         *    136340         *    136360         *    136380         *    136400
Seq1 : gtctgctaatctctgtaagatttcctcctccgctctcgatgcagtcggtcaacttattctctagttctctaatacgcgaacgcagtgcatcaacttcttg : 136400
Seq2 : gtctgctaatctctgtaagatttcctcctccgctctcgatgcagtcggtcaacttattctctagttctctaatacgcgaacgcagtgcatcaacttcttg : 136400
Seq3 : gtctgctaatctctgtaagatttcctcctccgctctcgatgcagtcggtcaacttattctctagttctctaatacgcgaacgcagtgcatcaacttcttg : 136400
Seq4 : gtctgctaatctctgtaagatttcctcctccgctctcgatgcagtcggtcaacttattctctagttctctaatacgcgaacgcagtgcatcaacttcttg : 136400

               *    136420         *    136440         *    136460         *    136480         *    136500
Seq1 : cgtgtcttcctggttgcgtgtacattcatcgagtctagattcgagatctctaacgcgtcgtcgttcttcctcaagttctctgcgtactacagaaagcgtg : 136500
Seq2 : cgtgtcttcctggttgcgtgtacattcatcgagtctagattcgagatctctaacgcgtcgtcgttcttcctcaagttctctgcgtactacagaaagcgtg : 136500
Seq3 : cgtgtcttcctggttgcgtgtacattcatcgagtctagattcgagatctctaacgcgtcgtcgttcttcctcaagttctctgcgtactacagaaagcgtg : 136500
Seq4 : cgtgtcttcctggttgcgtgtacattcatcgagtctagattcgagatctctaacgcgtcgtcgttcttcctcaagttctctgcgtactacagaaagcgtg : 136500
```

```
                *    136520         *    136540         *    136560         *    136580         *    136600
Seq1 : tccctatcttgttgatatttagcaatttctgattctagagtactgattttgcttacgtagttactaatagttgtcttggccttatcaagatcctccttgt : 136600
Seq2 : tccctatcttgttgatatttagcaatttctgattctagagtactgattttgcttacgtagttactaatagttgtcttggccttatcaagatcctccttgt : 136600
Seq3 : tccctatcttgttgatatttagcaatttctgattctagagtactgattttgcttacgtagttactaatagttgtcttggccttatcaagatcctccttgt : 136600
Seq4 : tccctatcttgttgatatttagcaatttctgattctagagtactgattttgcttacgtagttactaatagttgtcttggccttatcaagatcctccttgt : 136600

                *    136620         *    136640         *    136660         *    136680         *    136700
Seq1 : atttgtcgcattccttgatatccctacgaagtctggacagttcccattcgacattacgacgtttatcgatttcagctcggagatcgtcatcgcgttgttt : 136700
Seq2 : atttgtcgcattccttgatatccctacgaagtctggacagttcccattcgacattacgacgtttatcgatttcagctcggagatcgtcatcgcgttgttt : 136700
Seq3 : atttgtcgcattccttgatatccctacgaagtctggacagttcccattcgacattacgacgtttatcgatttcagctcggagatcgtcatcgcgttgttt : 136700
Seq4 : atttgtcgcattccttgatatccctacgaagtctggacagttcccattcgacattacgacgtttatcgatttcagctcggagatcgtcatcgcgttgttt : 136700

                *    136720         *    136740         *    136760         *    136780         *    136800
Seq1 : tagccacatacgactgagttcaagttctcgttgacaagatccatctacttttccattcctaatagtatccagttccttttctagttctgaacgcatttct : 136800
Seq2 : tagccacatacgactgagttcaagttctcgttgacaagatccatctacttttccattcctaatagtatccagttccttttctagttctgaacgcatttct : 136800
Seq3 : tagccacatacgactgagttcaagttctcgttgacaagatccatctacttttccattcctaatagtatccagttccttttctagttctgaacgcatttct : 136800
Seq4 : tagccacatacgactgagttcaagttctcgttgacaagatccatctacttttccattcctaatagtatccagttccttttctagttctgaacgcatttct : 136800

                *    136820         *    136840         *    136860         *    136880         *    136900
Seq1 : cgttccctatcaagcgattctctcaattctcggatagtcttcttatcaatttctaataaatctgaaccatcatctgtcccattttgaatatccctgtgtt : 136900
Seq2 : cgttccctatcaagcgattctctcaattctcggatagtcttcttatcaatttctaataaatctgaaccatcatctgtcccattttgaatatccctgtgtt : 136900
Seq3 : cgttccctatcaagcgattctctcaattctcggatagtcttcttatcaatttctaataaatctgaaccatcatctgtcccattttgaatatccctgtgtt : 136900
Seq4 : cgttccctatcaagcgattctctcaattctcggatagtcttcttatcaatttctaataaatctgaaccatcatctgtcccattttgaatatccctgtgtt : 136900

                *    136920         *    136940         *    136960         *    136980         *    137000
Seq1 : ctttgatctcttttgtaagtcggtcgattctttcggttttataaacagaatccctttccaaagtcctaatcttactgagtttatcactaagttctgcatt : 137000
Seq2 : ctttgatctcttttgtaagtcggtcgattctttcggttttataaacagaatccctttccaaagtcctaatcttactgagtttatcactaagttctgcatt : 137000
Seq3 : ctttgatctcttttgtaagtcggtcgattctttcggttttataaacagaatccctttccaaagtcctaatcttactgagtttatcactaagttctgcatt : 137000
Seq4 : ctttgatctcttttgtaagtcggtcgattctttcggttttataaacagaatccctttccaaagtcctaatcttactgagtttatcactaagttctgcatt : 137000

                *    137020         *    137040         *    137060         *    137080         *    137100
Seq1 : caattcggtgagttttctcttggcttcttccaactctgttttaaactctccactatttccgcattcttcctcgcatttatctaaccattcaattagttta : 137100
Seq2 : caattcggtgagttttctcttggcttcttccaactctgttttaaactctccactatttccgcattcttcctcgcatttatctaaccattcaattagttta : 137100
Seq3 : caattcggtgagttttctcttggcttcttccaactctgttttaaactctccactatttccgcattcttcctcgcatttatctaaccattcaattagttta : 137100
Seq4 : caattcggtgagttttctcttggcttcttccaactctgttttaaactctccactatttccgcattcttcctcgcatttatctaaccattcaattagttta : 137100

                *    137120         *    137140         *    137160         *    137180         *    137200
Seq1 : ttaataactagttggtaatcagcgattcctatagccgttcttgtaattgtgggaacataattaggatcttctaatggattgtatggcttgatagcatcat : 137200
Seq2 : ttaataactagttggtaatcagcgattcctatagccgttcttgtaattgtgggaacataattaggatcttctaatggattgtatggcttgatagcatcat : 137200
Seq3 : ttaataactagttggtaatcagcgattcctatagccgttcttgtaattgtgggaacataattaggatcttctaatggattgtatggcttgatagcatcat : 137200
Seq4 : ttaataactagttggtaatcagcgattcctatagccgttcttgtaattgtgggaacataattaggatcttctaatggattgtatggcttgatagcatcat : 137200
```

```
             *    137220         *    137240         *    137260         *    137280         *    137300
Seq1 : ctttatcattattagggggatggacaaccttaattggttggtcctcatctcctccagtagcgtgtggttcttcaataccagtgttagtaataggcttagg : 137300
Seq2 : ctttatcattattagggggatggacaaccttaattggttggtcctcatctcctccagtagcgtgtggttcttcaataccagtgttagtaataggcttagg : 137300
Seq3 : ctttatcattattagggggatggacaaccttaattggttggtcctcatctcctccagtagcgtgtggttcttcaataccagtgttagtaataggcttagg : 137300
Seq4 : ctttatcattattagggggatggacaaccttaattggttggtcctcatctcctccagtagcgtgtggttcttcaataccagtgttagtaataggcttagg : 137300
```

```
             *    137320         *    137340         *    137360         *    137380         *    137400
Seq1 : caaatgcttgtcgtacgcgggcacttcctcatccatcaagtatttataatcgggttctacgtctgaatattcttttctaagagacgcgacttcgggagtt : 137400
Seq2 : caaatgcttgtcgtacgcgggcacttcctcatccatcaagtatttataatcgggttctacgtctgaatattcttttctaagagacgcgacttcgggagtt : 137400
Seq3 : caaatgcttgtcgtacgcgggcacttcctcatccatcaagtatttataatcgggttctacgtctgaatattcttttctaagagacgcgacttcgggagtt : 137400
Seq4 : caaatgcttgtcgtacgcgggcacttcctcatccatcaagtatttataatcgggttctacgtctgaatattcttttctaagagacgcgacttcgggagtt : 137400
```

```
             *    137420         *    137440         *    137460         *    137480         *    137500
Seq1 : agtagaagaactctgtttctgtatctatcaacgctggaatcaatactcaagttaaggatagcgaatacctcatcgtcatcatccgtatcttctgaaacac : 137500
Seq2 : agtagaagaactctgtttctgtatctatcaacgctggaatcaatactcaagttaaggatagcgaatacctcatcgtcatcatccgtatcttctgaaacac : 137500
Seq3 : agtagaagaactctgtttctgtatctatcaacgctggaatcaatactcaagttaaggatagcgaatacctcatcgtcatcatccgtatcttctgaaacac : 137500
Seq4 : agtagaagaactctgtttctgtatctatcaacgctggaatcaatactcaagttaaggatagcgaatacctcatcgtcatcatccgtatcttctgaaacac : 137500
```

```
             *    137520         *    137540         *    137560         *    137580         *    137600
Seq1 : catcatatgacatttcatgaagtctaacgtattgataaatagaatcagatttagtattaaacagatccttaacctttttagtaaacgcatatgtatattt : 137600
Seq2 : catcatatgacatttcatgaagtctaacgtattgataaatagaatcagatttagtattaaacagatccttaacctttttagtaaacgcatatgtatattt : 137600
Seq3 : catcatatgacatttcatgaagtctaacgtattgataaatagaatcagatttagtattaaacagatccttaacctttttagtaaacgcatatgtatattt : 137600
Seq4 : catcatatgacatttcatgaagtctaacgtattgataaatagaatcagatttagtattaaacagatccttaacctttttagtaaacgcatatgtatattt : 137600
```

```
             *    137620         *    137640         *    137660         *    137680         *    137700
Seq1 : tagatctccagatttcataatatgatcacatgccttaaatgtcagtgcttccatgatataatctggaacactaatgggtgacgaaaaagatacagcacca : 137700
Seq2 : tagatctccagatttcataatatgatcacatgccttaaatgtcagtgcttccatgatataatctggaacactaatgggtgacgaaaaagatacagcacca : 137700
Seq3 : tagatctccagatttcataatatgatcacatgccttaaatgtcagtgcttccatgatataatctggaacactaatgggtgacgaaaaagatacagcacca : 137700
Seq4 : tagatctccagatttcataatatgatcacatgccttaaatgtcagtgcttccatgatataatctggaacactaatgggtgacgaaaaagatacagcacca : 137700
```

```
             *    137720         *    137740         *    137760         *    137780         *    137800
Seq1 : tatgctacgttgataaataaatctgaaccactaagtagataatgattaatgttaaggaagaggaaatattcagtatatagatatgccttagcatcatatc : 137800
Seq2 : tatgctacgttgataaataaatctgaaccactaagtagataatgattaatgttaaggaagaggaaatattcagtatatagatatgccttagcatcatatc : 137800
Seq3 : tatgctacgttgataaataaatctgaaccactaagtagataatgattaatgttaaggaagaggaaatattcagtatatagatatgccttagcatcatatc : 137800
Seq4 : tatgctacgttgataaataaatctgaaccactaagtagataatgattaatgttaaggaagaggaaatattcagtatatagatatgccttagcatcatatc : 137800
```

```
             *    137820         *    137840         *    137860         *    137880         *    137900
Seq1 : ttgtactaaacacgctaaacagtttattgatgtgatcaatttccaacagaataattagagcagcgggaataccaacaaacatattaccacatccgtattt : 137900
Seq2 : ttgtactaaacacgctaaacagtttattgatgtgatcaatttccaacagaataattagagcagcgggaataccaacaaacatattaccacatccgtattt : 137900
Seq3 : ttgtactaaacacgctaaacagtttattgatgtgatcaatttccaacagaataattagagcagcgggaataccaacaaacatattaccacatccgtattt : 137900
Seq4 : ttgtactaaacacgctaaacagtttattgatgtgatcaatttccaacagaataattagagcagcgggaataccaacaaacatattaccacatccgtattt : 137900
```

```
               *    137920          *    137940          *    137960          *    137980          *    138000
Seq1 : tctatgaatatcacatatcatgttaaaaaatcttgatagaagagcgaatatctcgtctgacttaatgagtcgtagttcagcagcaacataagtcataact : 138000
Seq2 : tctatgaatatcacatatcatgttaaaaaatcttgatagaagagcgaatatctcgtctgacttaatgagtcgtagttcagcagcaacataagtcataact : 138000
Seq3 : tctatgaatatcacatatcatgttaaaaaatcttgatagaagagcgaatatctcgtctgacttaatgagtcgtagttcagcagcaacataagtcataact : 138000
Seq4 : tctatgaatatcacatatcatgttaaaaaatcttgatagaagagcgaatatctcgtctgacttaatgagtcgtagttcagcagcaacataagtcataact : 138000

               *    138020          *    138040          *    138060          *    138080          *    138100
Seq1 : gtaaatagaacatactttcctgtagtgttgattctagactccacatcaacaccattattaaaaatagttttatatacatctttaatctgctctccgttaa : 138100
Seq2 : gtaaatagaacatactttcctgtagtgttgattctagactccacatcaacaccattattaaaaatagttttatatacatctttaatctgctctccgttaa : 138100
Seq3 : gtaaatagaacatactttcctgtagtgttgattctagactccacatcaacaccattattaaaaatagttttatatacatctttaatctgctctccgttaa : 138100
Seq4 : gtaaatagaacatactttcctgtagtgttgattctagactccacatcaacaccattattaaaaatagttttatatacatctttaatctgctctccgttaa : 138100

               *    138120          *    138140          *    138160          *    138180          *    138200
Seq1 : tcgtcgaacgttctagtatacggaaacactttgatttcttatctgtagttaatgacttagtgatatcacgaagaatattacgaattacatttcttgtttt : 138200
Seq2 : tcgtcgaacgttctagtatacggaaacactttgatttcttatctgtagttaatgacttagtgatatcacgaagaatattacgaattacatttcttgtttt : 138200
Seq3 : tcgtcgaacgttctagtatacggaaacactttgatttcttatctgtagttaatgacttagtgatatcacgaagaatattacgaattacatttcttgtttt : 138200
Seq4 : tcgtcgaacgttctagtatacggaaacactttgatttcttatctgtagttaatgacttagtgatatcacgaagaatattacgaattacatttcttgtttt : 138200

               *    138220          *    138240          *    138260          *    138280          *    138300
Seq1 : tcttgagagacctgattcagaactcaactcatcgttccatagtttttttacctcagtggcgaaatctttggagtgcttggtacatttttcaataaggttc : 138300
Seq2 : tcttgagagacctgattcagaactcaactcatcgttccatagtttttttacctcagtggcgaaatctttggagtgcttggtacatttttcaataaggttc : 138300
Seq3 : tcttgagagacctgattcagaactcaactcatcgttccatagtttttttacctcagtggcgaaatctttggagtgcttggtacatttttcaataaggttc : 138300
Seq4 : tcttgagagacctgattcagaactcaactcatcgttccatagtttttttacctcagtggcgaaatctttggagtgcttggtacatttttcaataaggttc : 138300

               *    138320          *    138340          *    138360          *    138380          *    138400
Seq1 : gtgacctccatttattataaaaaattttattcaaaacttaactacactgcagttacacggcgatctttccgcccttcttggcctttatgaggatctctct : 138400
Seq2 : gtgacctccatttattataaaaaattttattcaaaacttaactacactgcagttacacggcgatctttccgcccttcttggcctttatgaggatctctct : 138400
Seq3 : gtgacctccatttattataaaaaattttattcaaaacttaactacactgcagttacacggcgatctttccgcccttcttggcctttatgaggatctctct : 138400
Seq4 : gtgacctccatttattataaaaaattttattcaaaacttaactacactgcagttacacggcgatctttccgcccttcttggcctttatgaggatctctct : 138400

               *    138420          *    138440          *    138460          *    138480          *    138500
Seq1 : gatttttcttgcgtcgagttttccggtaagacctttcggtacttcgtccacaaacacaactcctccgcgcaactttttcgcggttgttacttgactggcg : 138500
Seq2 : gatttttcttgcgtcgagttttccggtaagacctttcggtacttcgtccacaaacacaactcctccgcgcaactttttcgcggttgttacttgactggcg : 138500
Seq3 : gatttttcttgcgtcgagttttccggtaagacctttcggtacttcgtccacaaacacaactcctccgcgcaactttttcgcggttgttacttgactggcg : 138500
Seq4 : gatttttcttgcgtcgagttttccggtaagacctttcggtacttcgtccacaaacacaactcctccgcgcaactttttcgcggttgttacttgactggcg : 138500

               *    138520          *    138540          *    138560          *    138580          *    138600
Seq1 : acgtaatccacgatctctttttccgtcatcgtctttccgtgctccaaaacaacaacggcggcgggaagttcaccggcgtcatcgtcgggaagacctgcga : 138600
Seq2 : acgtaatccacgatctctttttccgtcatcgtctttccgtgctccaaaacaacaacggcggcgggaagttcaccggcgtcatcgtcgggaagacctgcga : 138600
Seq3 : acgtaatccacgatctctttttccgtcatcgtctttccgtgctccaaaacaacaacggcggcgggaagttcaccggcgtcatcgtcgggaagacctgcga : 138600
Seq4 : acgtaatccacgatctctttttccgtcatcgtctttccgtgctccaaaacaacaacggcggcgggaagttcaccggcgtcatcgtcgggaagacctgcga : 138600
```

```
                   *    138620          *    138640          *    138660          *    138680          *    138700
Seq1 : cacctgcgtcgaagatgttggggtgttggagcaagatggattccaattcagcgggagccacctgatagcctttgtacttaatcagagacttcaggcggtc : 138700
Seq2 : cacctgcgtcgaagatgttggggtgttggagcaagatggattccaattcagcgggagccacctgatagcctttgtacttaatcagagacttcaggcggtc : 138700
Seq3 : cacctgcgtcgaagatgttggggtgttggagcaagatggattccaattcagcgggagccacctgatagcctttgtacttaatcagagacttcaggcggtc : 138700
Seq4 : cacctgcgtcgaagatgttggggtgttggagcaagatggattccaattcagcgggagccacctgatagcctttgtacttaatcagagacttcaggcggtc : 138700

                   *    138720          *    138740          *    138760          *    138780          *    138800
Seq1 : aacgatgaagaagtgttcgtcttcgtcccagtaagctatgtctccagaatgtagccatccatccttgtcaatcaaggcgttggtcgcttccggattgttt : 138800
Seq2 : aacgatgaagaagtgttcgtcttcgtcccagtaagctatgtctccagaatgtagccatccatccttgtcaatcaaggcgttggtcgcttccggattgttt : 138800
Seq3 : aacgatgaagaagtgttcgtcttcgtcccagtaagctatgtctccagaatgtagccatccatccttgtcaatcaaggcgttggtcgcttccggattgttt : 138800
Seq4 : aacgatgaagaagtgttcgtcttcgtcccagtaagctatgtctccagaatgtagccatccatccttgtcaatcaaggcgttggtcgcttccggattgttt : 138800

                   *    138820          *    138840          *    138860          *    138880          *    138900
Seq1 : acataaccggacataatcataggacctctcacacacagttcgcctctttgattaacgcccagcgttttcccggtatccagatccacaaccttcgcttcaa : 138900
Seq2 : acataaccggacataatcataggacctctcacacacagttcgcctctttgattaacgcccagcgttttcccggtatccagatccacaaccttcgcttcaa : 138900
Seq3 : acataaccggacataatcataggacctctcacacacagttcgcctctttgattaacgcccagcgttttcccggtatccagatccacaaccttcgcttcaa : 138900
Seq4 : acataaccggacataatcataggacctctcacacacagttcgcctctttgattaacgcccagcgttttcccggtatccagatccacaaccttcgcttcaa : 138900

                   *    138920          *    138940          *    138960          *    138980          *    139000
Seq1 : aaaatggaacaactttaccgaccgcgcccggtttatcatccccctcgggtgtaatcagaatagctgatgtagtctcagtgagcccatatccttgcctgat : 139000
Seq2 : aaaatggaacaactttaccgaccgcgcccggtttatcatccccctcgggtgtaatcagaatagctgatgtagtctcagtgagcccatatccttgcctgat : 139000
Seq3 : aaaatggaacaactttaccgaccgcgcccggtttatcatccccctcgggtgtaatcagaatagctgatgtagtctcagtgagcccatatccttgcctgat : 139000
Seq4 : aaaatggaacaactttaccgaccgcgcccggtttatcatccccctcgggtgtaatcagaatagctgatgtagtctcagtgagcccatatccttgcctgat : 139000

                   *    139020          *    139040          *    139060          *    139080          *    139100
Seq1 : acctggcagatggaacctcttggcaaccgcttccccgacttccttagagaggggagcgccaccagaagcaatttcgtgtaaattagataaatcgtatttg : 139100
Seq2 : acctggcagatggaacctcttggcaaccgcttccccgacttccttagagaggggagcgccaccagaagcaatttcgtgtaaattagataaatcgtatttg : 139100
Seq3 : acctggcagatggaacctcttggcaaccgcttccccgacttccttagagaggggagcgccaccagaagcaatttcgtgtaaattagataaatcgtatttg : 139100
Seq4 : acctggcagatggaacctcttggcaaccgcttccccgacttccttagagaggggagcgccaccagaagcaatttcgtgtaaattagataaatcgtatttg : 139100

                   *    139120          *    139140          *    139160          *    139180          *    139200
Seq1 : tcaatcagagtgcttttggcgaagaaggagaataggttggtaccagcagcgcactttgaatcttgtaatcctgaaggctcctcagaaacagctcttctt : 139200
Seq2 : tcaatcagagtgcttttggcgaagaaggagaataggttggcaccagcagcgcactttgaatcttgtaatcctgaaggctcctcagaaacagctcttctt : 139200
Seq3 : tcaatcagagtgcttttggcgaagaaggagaataggttggtaccagcagcgcactttgaatcttgtaatcctgaaggctcctcagaaacagctcttctt : 139200
Seq4 : tcaatcagagtgcttttggcgaagaaggagaataggttggcaccagcagcgcactttgaatcttgtaatcctgaaggctcctcagaaacagctcttctt : 139200

                   *    139220          *    139240          *    139260          *    139280          *    139300
Seq1 : caaatctatacattaagacgactcgaaatccacatatcaaatatccgagtgtagtaaacattccaaaaccgtgatggaatggaacaacacttaaaatcgc : 139300
Seq2 : caaatctatacattaagacgactcgaaatccacatatcaaatatccgagtgtagtaaacattccaaaaccgtgatggaatggaacaacacttaaaatcgc : 139300
Seq3 : caaatctatacattaagacgactcgaaatccacatatcaaatatccgagtgtagtaaacattccaaaaccgtgatggaatggaacaacacttaaaatcgc : 139300
Seq4 : caaatctatacattaagacgactcgaaatccacatatcaaatatccgagtgtagtaaacattccaaaaccgtgatggaatggaacaacacttaaaatcgc : 139300
```

```
                 *    139320         *    139340         *    139360         *    139380         *    139400
Seq1 : agtatccggaatgatttgattgccaaaaataggatctctggcatgcgagaatctcacgcaggcagttctatgaggcagagcgacacctttaggcagacca : 139400
Seq2 : agtatccggaatgatttgattgccaaaaataggatctctggcatgcgagaatctcacgcaggcagttctatgaggcagagcgacacctttaggcagacca : 139400
Seq3 : agtatccggaatgatttgattgccaaaaataggatctctggcatgcgagaatctcacgcaggcagttctatgaggcagagcgacacctttaggcagacca : 139400
Seq4 : agtatccggaatgatttgattgccaaaaataggatctctggcatgcgagaatctcacgcaggcagttctatgaggcagagcgacacctttaggcagacca : 139400

                 *    139420         *    139440         *    139460         *    139480         *    139500
Seq1 : gtagatccagaggagttcatgatcagtgcaattgtcttgtccctatcgaaggactctggcacaaaatcgtattcattaaaaccgggaggtagatgagatg : 139500
Seq2 : gtagatccagaggagttcatgatcagtgcaattgtcttgtccctatcgaaggactctggcacaaaatcgtattcattaaaaccgggaggtagatgagatg : 139500
Seq3 : gtagatccagaggagttcatgatcagtgcaattgtcttgtccctatcgaaggactctggcacaaaatcgtattcattaaaaccgggaggtagatgagatg : 139500
Seq4 : gtagatccagaggagttcatgatcagtgcaattgtcttgtccctatcgaaggactctggcacaaaatcgtattcattaaaaccgggaggtagatgagatg : 139500

                 *    139520         *    139540         *    139560         *    139580         *    139600
Seq1 : tgacgaacgtgtacatcgactgaaatccctggtaatccgttttagaatccatgataataatttttggatgattgggagctttttttgcacgttcaaaat : 139600
Seq2 : tgacgaacgtgtacatcgactgaaatccctggtaatccgttttagaatccatgataataatttttggatgattgggagctttttttgcacgttcaaaat : 139600
Seq3 : tgacgaacgtgtacatcgactgaaatccctggtaatccgttttagaatccatgataataatttttggatgattgggagctttttttgcacgttcaaaat : 139600
Seq4 : tgacgaacgtgtacatcgactgaaatccctggtaatccgttttagaatccatgataataatttttggatgattgggagctttttttgcacgttcaaaat : 139600

                 *    139620         *    139640         *    139660         *    139680         *    139700
Seq1 : tttttgcaacccctttttggaaacgaacaccacggtaggctgcgaaatgcccatactgttgagcaattcacgttcattataaatgtcgttcgcgggcgca : 139700
Seq2 : tttttgcaacccctttttggaaacgaacaccacggtaggctgcgaaatgcccatactgttgagcaattcacgttcattataaatgtcgttcgcgggcgca : 139700
Seq3 : tttttgcaacccctttttggaaacgaacaccacggtaggctgcgaaatgcccatactgttgagcaattcacgttcattataaatgtcgttcgcgggcgca : 139700
Seq4 : tttttgcaacccctttttggaaacgaacaccacggtaggctgcgaaatgcccatactgttgagcaattcacgttcattataaatgtcgttcgcgggcgca : 139700

                 *    139720         *    139740         *    139760         *    139780         *    139800
Seq1 : actgcaactccgataaataacgcgcccaacaccggcataaagaattgaagagagttttcactgcatacgacgattctgtgatttgtattcagcccatatc : 139800
Seq2 : actgcaactccgataaataacgcgcccaacaccggcataaagaattgaagagagttttcactgcatacgacgattctgtgatttgtattcagcccatatc : 139800
Seq3 : actgcaactccgataaataacgcgcccaacaccggcataaagaattgaagagagttttcactgcatacgacgattctgtgatttgtattcagcccatatc : 139800
Seq4 : actgcaactccgataaataacgcgcccaacaccggcataaagaattgaagagagttttcactgcatacgacgattctgtgatttgtattcagcccatatc : 139800

                 *    139820         *    139840         *    139860         *    139880         *    139900
Seq1 : gtttcatagcttctgccaaccggacggacatttcgaagtactcagcgtaagtgatgtccacctcgatatgtgcatctgtaaaagcaattgttccaggaac : 139900
Seq2 : gtttcatagcttctgccaaccggacggacatttcgaagtactcagcgtaagtgatgtccacctcgatatgtgcatctgtaaaagcaattgttccaggaac : 139900
Seq3 : gtttcatagcttctgccaaccggacggacatttcgaagtactcagcgtaagtgatgtccacctcgatatgtgcatctgtaaaagcaattgttccaggaac : 139900
Seq4 : gtttcatagcttctgccaaccggacggacatttcgaagtactcagcgtaagtgatgtccacctcgatatgtgcatctgtaaaagcaattgttccaggaac : 139900

                 *    139920         *    139940         *    139960         *    139980         *    140000
Seq1 : cagggcgtatctcttcatagccttatgcagttgctctccagcggttccatcttccagcggatagaatggcgccgggcctttctttatgtttttggcgtct : 140000
Seq2 : cagggcgtatctcttcatagccttatgcagttgctctccagcggttccatcttccagcggatagaatggcgccgggcctttctttatgtttttggcgtct : 140000
Seq3 : cagggcgtatctcttcatagccttatgcagttgctctccagcggttccatcttccagcggatagaatggcgccgggcctttctttatgtttttggcgtct : 140000
Seq4 : cagggcgtatctcttcatagccttatgcagttgctctccagcggttccatcttccagcggatagaatggcgccgggcctttctttatgtttttggcgtct : 140000
```

```
                 *    140020          *    140040          *    140060          *    140080          *    140100
Seq1 : tccatcccgtgcaataaatagatctaatttttcaatttttagctaatttttcaatttttggggtcgacttattattatttttgacaccagaccaactggt : 140100
Seq2 : tccatcccgtgcaataaatagatctaatttttcaatttttagctaatttttcaatttttggggtcgacttattattatttttgacaccagaccaactggt : 140100
Seq3 : tccatcccgtgcaataaatagatctaatttttcaatttttagctaatttttcaatttttggggtcgacttattattatttttgacaccagaccaactggt : 140100
Seq4 : tccatcccgtgcaataaatagatctaatttttcaatttttagctaatttttcaatttttggggtcgacttattattatttttgacaccagaccaactggt : 140100

                 *    140120          *    140140          *    140160          *    140180          *    140200
Seq1 : aatggtagcgaccggcgctcagctggaattccgccgatactgacgggctccaggagtcgtcgccaccaatccccatatggaaaccgtcgatattcagcca : 140200
Seq2 : aatggtagcgaccggcgctcagctggaattccgccgatactgacgggctccaggagtcgtcgccaccaatccccatatggaaaccgtcgatattcagcca : 140200
Seq3 : aatggtagcgaccggcgctcagctggaattccgccgatactgacgggctccaggagtcgtcgccaccaatccccatatggaaaccgtcgatattcagcca : 140200
Seq4 : aatggtagcgaccggcgctcagctggaattccgccgatactgacgggctccaggagtcgtcgccaccaatccccatatggaaaccgtcgatattcagcca : 140200

                 *    140220          *    140240          *    140260          *    140280          *    140300
Seq1 : tgtgccttcttccgcgtgcagcagatggcgatggctggtttccatcagttgctgttgactgtagcggctgatgttgaactggaagtcgccgcgccactgg : 140300
Seq2 : tgtgccttcttccgcgtgcagcagatggcgatggctggtttccatcagttgctgttgactgtagcggctgatgttgaactggaagtcgccgcgccactgg : 140300
Seq3 : tgtgccttcttccgcgtgcagcagatggcgatggctggtttccatcagttgctgttgactgtagcggctgatgttgaactggaagtcgccgcgccactgg : 140300
Seq4 : tgtgccttcttccgcgtgcagcagatggcgatggctggtttccatcagttgctgttgactgtagcggctgatgttgaactggaagtcgccgcgccactgg : 140300

                 *    140320          *    140340          *    140360          *    140380          *    140400
Seq1 : tgtgggccataattcaattcgcgcgtcccgcagcgcagaccgttttcgctcgggaagacgtacggggtatacatgtctgacaatggcagatcccagcggt : 140400
Seq2 : tgtgggccataattcaattcgcgcgtcccgcagcgcagaccgttttcgctcgggaagacgtacggggtatacatgtctgacaatggcagatcccagcggt : 140400
Seq3 : tgtgggccataattcaattcgcgcgtcccgcagcgcagaccgttttcgctcgggaagacgtacggggtatacatgtctgacaatggcagatcccagcggt : 140400
Seq4 : tgtgggccataattcaattcgcgcgtcccgcagcgcagaccgttttcgctcgggaagacgtacggggtatacatgtctgacaatggcagatcccagcggt : 140400

                 *    140420          *    140440          *    140460          *    140480          *    140500
Seq1 : caaaacaggcggcagtaaggcggtcgggatagttttcttgcggccctaatccgagccagtttacccgctctgctacctgcgccagctggcagttcaggcc : 140500
Seq2 : caaaacaggcggcagtaaggcggtcgggatagttttcttgcggccctaatccgagccagtttacccgctctgctacctgcgccagctggcagttcaggcc : 140500
Seq3 : caaaacaggcggcagtaaggcggtcgggatagttttcttgcggccctaatccgagccagtttacccgctctgctacctgcgccagctggcagttcaggcc : 140500
Seq4 : caaaacaggcggcagtaaggcggtcgggatagttttcttgcggccctaatccgagccagtttacccgctctgctacctgcgccagctggcagttcaggcc : 140500

                 *    140520          *    140540          *    140560          *    140580          *    140600
Seq1 : aatccgcgccggatgcggtgtatcgctcgccacttcaacatcaacggtaatcgccatttgaccactaccatcaatccggtaggttttccggctgataaat : 140600
Seq2 : aatccgcgccggatgcggtgtatcgctcgccacttcaacatcaacggtaatcgccatttgaccactaccatcaatccggtaggttttccggctgataaat : 140600
Seq3 : aatccgcgccggatgcggtgtatcgctcgccacttcaacatcaacggtaatcgccatttgaccactaccatcaatccggtaggttttccggctgataaat : 140600
Seq4 : aatccgcgccggatgcggtgtatcgctcgccacttcaacatcaacggtaatcgccatttgaccactaccatcaatccggtaggttttccggctgataaat : 140600

                 *    140620          *    140640          *    140660          *    140680          *    140700
Seq1 : aaggttttcccctgatgctgccacgcgtgagcggtcgtaatcagcaccgcatcagcaagtgtatctgccgtgcactgcaacaacgctgcttcggcctggt : 140700
Seq2 : aaggttttcccctgatgctgccacgcgtgagcggtcgtaatcagcaccgcatcagcaagtgtatctgccgtgcactgcaacaacgctgcttcggcctggt : 140700
Seq3 : aaggttttcccctgatgctgccacgcgtgagcggtcgtaatcagcaccgcatcagcaagtgtatctgccgtgcactgcaacaacgctgcttcggcctggt : 140700
Seq4 : aaggttttcccctgatgctgccacgcgtgagcggtcgtaatcagcaccgcatcagcaagtgtatctgccgtgcactgcaacaacgctgcttcggcctggt : 140700
```

```
                *    140720         *    140740         *    140760         *    140780         *    140800
Seq1 : aatggcccgccgccttccagcgttcgacccaggcgttagggtcaatgcgggtcgcttcacttacgccaatgtcgttatccagcggtgcacgggtgaactg : 140800
Seq2 : aatggcccgccgccttccagcgttcgacccaggcgttagggtcaatgcgggtcgcttcacttacgccaatgtcgttatccagcggtgcacgggtgaactg : 140800
Seq3 : aatggcccgccgccttccagcgttcgacccaggcgttagggtcaatgcgggtcgcttcacttacgccaatgtcgttatccagcggtgcacgggtgaactg : 140800
Seq4 : aatggcccgccgccttccagcgttcgacccaggcgttagggtcaatgcgggtcgcttcacttacgccaatgtcgttatccagcggtgcacgggtgaactg : 140800

                *    140820         *    140840         *    140860         *    140880         *    140900
Seq1 : atcgcgcagcggcgtcagcagttgttttttatcgccaatccacatctgtgaaagaaagcctgactggcggttaaattgccaacgcttattacccagctcg : 140900
Seq2 : atcgcgcagcggcgtcagcagttgttttttatcgccaatccacatctgtgaaagaaagcctgactggcggttaaattgccaacgcttattacccagctcg : 140900
Seq3 : atcgcgcagcggcgtcagcagttgttttttatcgccaatccacatctgtgaaagaaagcctgactggcggttaaattgccaacgcttattacccagctcg : 140900
Seq4 : atcgcgcagcggcgtcagcagttgttttttatcgccaatccacatctgtgaaagaaagcctgactggcggttaaattgccaacgcttattacccagctcg : 140900

                *    140920         *    140940         *    140960         *    140980         *    141000
Seq1 : atgcaaaaatccatttcgctggtggtcagatgcgggatggcgtgggacgcggcggggagcgtcacactgaggttttccgccagacgccactgctgccagg : 141000
Seq2 : atgcaaaaatccatttcgctggtggtcagatgcgggatggcgtgggacgcggcggggagcgtcacactgaggttttccgccagacgccactgctgccagg : 141000
Seq3 : atgcaaaaatccatttcgctggtggtcagatgcgggatggcgtgggacgcggcggggagcgtcacactgaggttttccgccagacgccactgctgccagg : 141000
Seq4 : atgcaaaaatccatttcgctggtggtcagatgcgggatggcgtgggacgcggcggggagcgtcacactgaggttttccgccagacgccactgctgccagg : 141000

                *    141020         *    141040         *    141060         *    141080         *    141100
Seq1 : cgctgatgtgcccggcttctgaccatgcggtcgcgttcggttgcactacgcgtactgtgagccagagttgcccggcgctctccggctgcggtagttcagg : 141100
Seq2 : cgctgatgtgcccggcttctgaccatgcggtcgcgttcggttgcactacgcgtactgtgagccagagttgcccggcgctctccggctgcggtagttcagg : 141100
Seq3 : cgctgatgtgcccggcttctgaccatgcggtcgcgttcggttgcactacgcgtactgtgagccagagttgcccggcgctctccggctgcggtagttcagg : 141100
Seq4 : cgctgatgtgcccggcttctgaccatgcggtcgcgttcggttgcactacgcgtactgtgagccagagttgcccggcgctctccggctgcggtagttcagg : 141100

                *    141120         *    141140         *    141160         *    141180         *    141200
Seq1 : cagttcaatcaactgtttaccttgtggagcgacatccagaggcacttcaccgcttgccagcggcttaccatccagcgccaccatccagtgcaggagctcg : 141200
Seq2 : cagttcaatcaactgtttaccttgtggagcgacatccagaggcacttcaccgcttgccagcggcttaccatccagcgccaccatccagtgcaggagctcg : 141200
Seq3 : cagttcaatcaactgtttaccttgtggagcgacatccagaggcacttcaccgcttgccagcggcttaccatccagcgccaccatccagtgcaggagctcg : 141200
Seq4 : cagttcaatcaactgtttaccttgtggagcgacatccagaggcacttcaccgcttgccagcggcttaccatccagcgccaccatccagtgcaggagctcg : 141200

                *    141220         *    141240         *    141260         *    141280         *    141300
Seq1 : ttatcgctatgacggaacaggtattcgctggtcacttcgatggtttgcccggataaacggaactggaaaaactgctgctggtgttttgcttccgtcagcg : 141300
Seq2 : ttatcgctatgacggaacaggtattcgctggtcacttcgatggtttgcccggataaacggaactggaaaaactgctgctggtgttttgcttccgtcagcg : 141300
Seq3 : ttatcgctatgacggaacaggtattcgctggtcacttcgatggtttgcccggataaacggaactggaaaaactgctgctggtgttttgcttccgtcagcg : 141300
Seq4 : ttatcgctatgacggaacaggtattcgctggtcacttcgatggtttgcccggataaacggaactggaaaaactgctgctggtgttttgcttccgtcagcg : 141300

                *    141320         *    141340         *    141360         *    141380         *    141400
Seq1 : ctggatgcggcgtgcggtcggcaaagaccagaccgttcatacagaactggcgatcgttcggcgtatcgccaaaatcaccgccgtaagccgaccacgggtt : 141400
Seq2 : ctggatgcggcgtgcggtcggcaaagaccagaccgttcatacagaactggcgatcgttcggcgtatcgccaaaatcaccgccgtaagccgaccacgggtt : 141400
Seq3 : ctggatgcggcgtgcggtcggcaaagaccagaccgttcatacagaactggcgatcgttcggcgtatcgccaaaatcaccgccgtaagccgaccacgggtt : 141400
Seq4 : ctggatgcggcgtgcggtcggcaaagaccagaccgttcatacagaactggcgatcgttcggcgtatcgccaaaatcaccgccgtaagccgaccacgggtt : 141400
```

```
                 *    141420         *    141440         *    141460         *    141480         *    141500
Seq1 : gccgttttcatcatatttaatcagcgactgatccacccagtcccagacgaagccgccctgtaaacggggatactgacgaaacgcctgccagtatttagcg : 141500
Seq2 : gccgttttcatcatatttaatcagcgactgatccacccagtcccagacgaagccgccctgtaaacggggatactgacgaaacgcctgccagtatttagcg : 141500
Seq3 : gccgttttcatcatatttaatcagcgactgatccacccagtcccagacgaagccgccctgtaaacggggatactgacgaaacgcctgccagtatttagcg : 141500
Seq4 : gccgttttcatcatatttaatcagcgactgatccacccagtcccagacgaagccgccctgtaaacggggatactgacgaaacgcctgccagtatttagcg : 141500

                 *    141520         *    141540         *    141560         *    141580         *    141600
Seq1 : aaaccgccaagactgttacccatcgcgtgggcgtattcgcaaaggatcagcgggcgcgtctctccaggtagcgaaagccatttttttgatggaccatttcg : 141600
Seq2 : aaaccgccaagactgttacccatcgcgtgggcgtattcgcaaaggatcagcgggcgcgtctctccaggtagcgaaagccatttttttgatggaccatttcg : 141600
Seq3 : aaaccgccaagactgttacccatcgcgtgggcgtattcgcaaaggatcagcgggcgcgtctctccaggtagcgaaagccatttttttgatggaccatttcg : 141600
Seq4 : aaaccgccaagactgttacccatcgcgtgggcgtattcgcaaaggatcagcgggcgcgtctctccaggtagcgaaagccatttttttgatggaccatttcg : 141600

                 *    141620         *    141640         *    141660         *    141680         *    141700
Seq1 : gcacagccgggaagggctggtcttcatccacgcgcgcgtacatcgggcaaataatatcggtggccgtggtgtcggctccgccgccttcatactgcaccgg : 141700
Seq2 : gcacagccgggaagggctggtcttcatccacgcgcgcgtacatcgggcaaataatatcggtggccgtggtgtcggctccgccgccttcatactgcaccgg : 141700
Seq3 : gcacagccgggaagggctggtcttcatccacgcgcgcgtacatcgggcaaataatatcggtggccgtggtgtcggctccgccgccttcatactgcaccgg : 141700
Seq4 : gcacagccgggaagggctggtcttcatccacgcgcgcgtacatcgggcaaataatatcggtggccgtggtgtcggctccgccgccttcatactgcaccgg : 141700

                 *    141720         *    141740         *    141760         *    141780         *    141800
Seq1 : gcgggaaggatcgacagatttgatccagcgatacagcgcgtcgtgattagcgccgtggcctgattcattccccagcgaccagatgatcacactcgggtga : 141800
Seq2 : gcgggaaggatcgacagatttgatccagcgatacagcgcgtcgtgattagcgccgtggcctgattcattccccagcgaccagatgatcacactcgggtga : 141800
Seq3 : gcgggaaggatcgacagatttgatccagcgatacagcgcgtcgtgattagcgccgtggcctgattcattccccagcgaccagatgatcacactcgggtga : 141800
Seq4 : gcgggaaggatcgacagatttgatccagcgatacagcgcgtcgtgattagcgccgtggcctgattcattccccagcgaccagatgatcacactcgggtga : 141800

                 *    141820         *    141840         *    141860         *    141880         *    141900
Seq1 : ttacgatcgcgctgcaccattcgcgttacgcgttcgctcatcgccggtagccagcgcggatcatcggtcagacgattcattggcaccatgccgtgggttt : 141900
Seq2 : ttacgatcgcgctgcaccattcgcgttacgcgttcgctcatcgccggtagccagcgcggatcatcggtcagacgattcattggcaccatgccgtgggttt : 141900
Seq3 : ttacgatcgcgctgcaccattcgcgttacgcgttcgctcatcgccggtagccagcgcggatcatcggtcagacgattcattggcaccatgccgtgggttt : 141900
Seq4 : ttacgatcgcgctgcaccattcgcgttacgcgttcgctcatcgccggtagccagcgcggatcatcggtcagacgattcattggcaccatgccgtgggttt : 141900

                 *    141920         *    141940         *    141960         *    141980         *    142000
Seq1 : caatattggcttcatccaccacatacaggccgtagcggtcgcacagcgtgtaccacagcggatggttcggataatgcgaacagcgcacggcgttaaagtt : 142000
Seq2 : caatattggcttcatccaccacatacaggccgtagcggtcgcacagcgtgtaccacagcggatggttcggataatgcgaacagcgcacggcgttaaagtt : 142000
Seq3 : caatattggcttcatccaccacatacaggccgtagcggtcgcacagcgtgtaccacagcggatggttcggataatgcgaacagcgcacggcgttaaagtt : 142000
Seq4 : caatattggcttcatccaccacatacaggccgtagcggtcgcacagcgtgtaccacagcggatggttcggataatgcgaacagcgcacggcgttaaagtt : 142000

                 *    142020         *    142040         *    142060         *    142080         *    142100
Seq1 : gttctgcttcatcagcaggatatcctgcaccatcgtctgctcatccatgacctgaccatgcagaggatgatgctcgtgacggttaacgcctcgaatcagc : 142100
Seq2 : gttctgcttcatcagcaggatatcctgcaccatcgtctgctcatccatgacctgaccatgcagaggatgatgctcgtgacggttaacgcctcgaatcagc : 142100
Seq3 : gttctgcttcatcagcaggatatcctgcaccatcgtctgctcatccatgacctgaccatgcagaggatgatgctcgtgacggttaacgcctcgaatcagc : 142100
Seq4 : gttctgcttcatcagcaggatatcctgcaccatcgtctgctcatccatgacctgaccatgcagaggatgatgctcgtgacggttaacgcctcgaatcagc : 142100
```

```
                  *    142120         *    142140         *    142160         *    142180         *    142200
Seq1 : aacggcttgccgttcagcagcagcagaccattttcaatccgcacctcgcggaaaccgacatcgcaggcttctgcttcaatcagcgtgccgtcggcggtgt : 142200
Seq2 : aacggcttgccgttcagcagcagcagaccattttcaatccgcacctcgcggaaaccgacatcgcaggcttctgcttcaatcagcgtgccgtcggcggtgt : 142200
Seq3 : aacggcttgccgttcagcagcagcagaccattttcaatccgcacctcgcggaaaccgacatcgcaggcttctgcttcaatcagcgtgccgtcggcggtgt : 142200
Seq4 : aacggcttgccgttcagcagcagcagaccattttcaatccgcacctcgcggaaaccgacatcgcaggcttctgcttcaatcagcgtgccgtcggcggtgt : 142200

                  *    142220         *    142240         *    142260         *    142280         *    142300
Seq1 : gcagttcaaccaccgcacgatagagattcgggatttcggcgctccacagtttcgggttttcgacgttcagacgtagtgtgacgcgatcggcataaccacc : 142300
Seq2 : gcagttcaaccaccgcacgatagagattcgggatttcggcgctccacagtttcgggttttcgacgttcagacgtagtgtgacgcgatcggcataaccacc : 142300
Seq3 : gcagttcaaccaccgcacgatagagattcgggatttcggcgctccacagtttcgggttttcgacgttcagacgtagtgtgacgcgatcggcataaccacc : 142300
Seq4 : gcagttcaaccaccgcacgatagagattcgggatttcggcgctccacagtttcgggttttcgacgttcagacgtagtgtgacgcgatcggcataaccacc : 142300

                  *    142320         *    142340         *    142360         *    142380         *    142400
Seq1 : acgctcatcgataatttcaccgccgaaaggcgcggtgccgctggcgacctgcgtttcaccctgccataaagaaactgttacccgtaggtagtcacgcaac : 142400
Seq2 : acgctcatcgataatttcaccgccgaaaggcgcggtgccgctggcgacctgcgtttcaccctgccataaagaaactgttacccgtaggtagtcacgcaac : 142400
Seq3 : acgctcatcgataatttcaccgccgaaaggcgcggtgccgctggcgacctgcgtttcaccctgccataaagaaactgttacccgtaggtagtcacgcaac : 142400
Seq4 : acgctcatcgataatttcaccgccgaaaggcgcggtgccgctggcgacctgcgtttcaccctgccataaagaaactgttacccgtaggtagtcacgcaac : 142400

                  *    142420         *    142440         *    142460         *    142480         *    142500
Seq1 : tcgccgcacatctgaacttcagcctccagtacagcgcggctgaaatcatcattaaagcgagtggcaacatggaaatcgctgatttgtgtagtcggtttat : 142500
Seq2 : tcgccgcacatctgaacttcagcctccagtacagcgcggctgaaatcatcattaaagcgagtggcaacatggaaatcgctgatttgtgtagtcggtttat : 142500
Seq3 : tcgccgcacatctgaacttcagcctccagtacagcgcggctgaaatcatcattaaagcgagtggcaacatggaaatcgctgatttgtgtagtcggtttat : 142500
Seq4 : tcgccgcacatctgaacttcagcctccagtacagcgcggctgaaatcatcattaaagcgagtggcaacatggaaatcgctgatttgtgtagtcggtttat : 142500

                  *    142520         *    142540         *    142560         *    142580         *    142600
Seq1 : gcagcaacgagacgtcacggaaaatgccgctcatccgccacatatcctgatcttccagataactgccgtcactccagcgcagcaccatcaccgcgaggcg : 142600
Seq2 : gcagcaacgagacgtcacggaaaatgccgctcatccgccacatatcctgatcttccagataactgccgtcactccagcgcagcaccatcaccgcgaggcg : 142600
Seq3 : gcagcaacgagacgtcacggaaaatgccgctcatccgccacatatcctgatcttccagataactgccgtcactccagcgcagcaccatcaccgcgaggcg : 142600
Seq4 : gcagcaacgagacgtcacggaaaatgccgctcatccgccacatatcctgatcttccagataactgccgtcactccagcgcagcaccatcaccgcgaggcg : 142600

                  *    142620         *    142640         *    142660         *    142680         *    142700
Seq1 : gttttctccggcgcgtaaaaatgcgctcaggtcaaattcagacggcaaacgactgtcctggccgtaaccgacccagcgcccgttgcaccacagatgaaac : 142700
Seq2 : gttttctccggcgcgtaaaaatgcgctcaggtcaaattcagacggcaaacgactgtcctggccgtaaccgacccagcgcccgttgcaccacagatgaaac : 142700
Seq3 : gttttctccggcgcgtaaaaatgcgctcaggtcaaattcagacggcaaacgactgtcctggccgtaaccgacccagcgcccgttgcaccacagatgaaac : 142700
Seq4 : gttttctccggcgcgtaaaaatgcgctcaggtcaaattcagacggcaaacgactgtcctggccgtaaccgacccagcgcccgttgcaccacagatgaaac : 142700

                  *    142720         *    142740         *    142760         *    142780         *    142800
Seq1 : gccgagttaacgccatcaaaaataattcgcgtctggccttcctgtagccagctttcatcaacattaaatgtgagcgagtaacaacccgtcggattctccg : 142800
Seq2 : gccgagttaacgccatcaaaaataattcgcgtctggccttcctgtagccagctttcatcaacattaaatgtgagcgagtaacaacccgtcggattctccg : 142800
Seq3 : gccgagttaacgccatcaaaaataattcgcgtctggccttcctgtagccagctttcatcaacattaaatgtgagcgagtaacaacccgtcggattctccg : 142800
Seq4 : gccgagttaacgccatcaaaaataattcgcgtctggccttcctgtagccagctttcatcaacattaaatgtgagcgagtaacaacccgtcggattctccg : 142800
```

```
                *    142820          *    142840          *    142860          *    142880          *    142900
Seq1 : tgggaacaaacggcggattgaccgtaatgggataggtcacgttggtgtagatgggcgcatcgtaaccgtgcatctgccagtttgaggggacgacgacagt : 142900
Seq2 : tgggaacaaacggcggattgaccgtaatgggataggtcacgttggtgtagatgggcgcatcgtaaccgtgcatctgccagtttgaggggacgacgacagt : 142900
Seq3 : tgggaacaaacggcggattgaccgtaatgggataggtcacgttggtgtagatgggcgcatcgtaaccgtgcatctgccagtttgaggggacgacgacagt : 142900
Seq4 : tgggaacaaacggcggattgaccgtaatgggataggtcacgttggtgtagatgggcgcatcgtaaccgtgcatctgccagtttgaggggacgacgacagt : 142900

                *    142920          *    142940          *    142960          *    142980          *    143000
Seq1 : atcggcctcaggaagatcgcactccagccagctttccggcaccgcttctggtgccggaaaccaggcaaagcgccattcgccattcaggctgcgcaactgt : 143000
Seq2 : atcggcctcaggaagatcgcactccagccagctttccggcaccgcttctggtgccggaaaccaggcaaagcgccattcgccattcaggctgcgcaactgt : 143000
Seq3 : atcggcctcaggaagatcgcactccagccagctttccggcaccgcttctggtgccggaaaccaggcaaagcgccattcgccattcaggctgcgcaactgt : 143000
Seq4 : atcggcctcaggaagatcgcactccagccagctttccggcaccgcttctggtgccggaaaccaggcaaagcgccattcgccattcaggctgcgcaactgt : 143000

                *    143020          *    143040          *    143060          *    143080          *    143100
Seq1 : tgggaagggcgatcggtgcgggcctcttcgctattacgccagctggcgaaagggggatgtgctgcaaggcgattaagttgggtaacgccagggttttccc : 143100
Seq2 : tgggaagggcgatcggtgcgggcctcttcgctattacgccagctggcgaaagggggatgtgctgcaaggcgattaagttgggtaacgccagggttttccc : 143100
Seq3 : tgggaagggcgatcggtgcgggcctcttcgctattacgccagctggcgaaagggggatgtgctgcaaggcgattaagttgggtaacgccagggttttccc : 143100
Seq4 : tgggaagggcgatcggtgcgggcctcttcgctattacgccagctggcgaaagggggatgtgctgcaaggcgattaagttgggtaacgccagggttttccc : 143100

                *    143120          *    143140          *    143160          *    143180          *    143200
Seq1 : agtcacgacgttgtaaaacgacgggatccctcgaggaattcatttatagcatagaaaaaaacaaaatgaaattcgttataaaatcgtagatctcccatgt : 143200
Seq2 : agtcacgacgttgtaaaacgacgggatccctcgaggaattcatttatagcatagaaaaaaacaaaatgaaattcgttataaaatcgtagatctcccatgt : 143200
Seq3 : agtcacgacgttgtaaaacgacgggatccctcgaggaattcatttatagcatagaaaaaaacaaaatgaaattcgttataaaatcgtagatctcccatgt : 143200
Seq4 : agtcacgacgttgtaaaacgacgggatccctcgaggaattcatttatagcatagaaaaaaacaaaatgaaattcgttataaaatcgtagatctcccatgt : 143200

                *    143220          *    143240          *    143260          *    143280          *    143300
Seq1 : ggtggaatactaccatctatcgcatgtggatggacagtaggtaatggccatgggaacagtaatgtttgcatatttatctttcttgccagtattactgcat : 143300
Seq2 : ggtggaatactaccatctatcgcatgtggatggacagtaggtaatggccatgggaacagtaatgtttgcatatttatctttcttgccagtattactgcat : 143300
Seq3 : ggtggaatactaccatctatcgcatgtggatggacagtaggtaatggccatgggaacagtaatgtttgcatatttatctttcttgccagtattactgcat : 143300
Seq4 : ggtggaatactaccatctatcgcatgtggatggacagtaggtaatggccatgggaacagtaatgtttgcatatttatctttcttgccagtattactgcat : 143300

                *    143320          *    143340          *    143360          *    143380          *    143400
Seq1 : attgtcccaatgtttcgatgtgatgttctaacctatcaactgccgctgtatcacaacaatagtgtccgatgaaattaagattatgatccaatgtgtttaa : 143400
Seq2 : attgtcccaatgtttcgatgtgatgttctaacctatcaactgccgctgtatcacaacaatagtgtccgatgaaattaagattatgatccaatgtgtttaa : 143400
Seq3 : attgtcccaatgtttcgatgtgatgttctaacctatcaactgccgctgtatcacaacaatagtgtccgatgaaattaagattatgatccaatgtgtttaa : 143400
Seq4 : attgtcccaatgtttcgatgtgatgttctaacctatcaactgccgctgtatcacaacaatagtgtccgatgaaattaagattatgatccaatgtgtttaa : 143400

                *    143420          *    143440          *    143460          *    143480          *    143500
Seq1 : tatatgattatcaagtcttatacgatccgcgtctttttttgacaggatcaggttcttctacaggaagaagtttcggcctcttatgatattcatgtctggga : 143500
Seq2 : tatatgattatcaagtcttatacgatccgcgtctttttttgacaggatcaggttcttctacaggaagaagtttcggcctcttatgatattcatgtctggga : 143500
Seq3 : tatatgattatcaagtcttatacgatccgcgtctttttttgacaggatcaggttcttctacaggaagaagtttcggcctcttatgatattcatgtctggga : 143500
Seq4 : tatatgattatcaagtcttatacgatccgcgtctttttttgacaggatcaggttcttctacaggaagaagtttcggcctcttatgatattcatgtctggga : 143500
```

```
               *     143520          *     143540          *     143560          *     143580          *     143600
Seq1 : aacggtggtctagggtgaggctccggtatcggagtgggttttggattataatcatcatcgtctatgacatcatcttcgacttcgatatttattttgctat : 143600
Seq2 : aacggtggtctagggtgaggctccggtatcggagtgggttttggattataatcatcatcgtctatgacatcatcttcgacttcgatatttattttgctat : 143600
Seq3 : aacggtggtctagggtgaggctccggtatcggagtgggttttggattataatcatcatcgtctatgacatcatcttcgacttcgatatttattttgctat : 143600
Seq4 : aacggtggtctagggtgaggctccggtatcggagtgggttttggattataatcatcatcgtctatgacatcatcttcgacttcgatatttattttgctat : 143600

               *     143620          *     143640          *     143660          *     143680          *     143700
Seq1 : cttgatgatgtcctgtatcagttgcattttcagcactcgactgaatattagcgcattcattgtctattattaccatatttctaaacccaaaatgtatgtg : 143700
Seq2 : cttgatgatgtcctgtatcagttgcattttcagcactcgactgaatattagcgcattcattgtctattattaccatatttctaaacccaaaatgtatgtg : 143700
Seq3 : cttgatgatgtcctgtatcagttgcattttcagcactcgactgaatattagcgcattcattgtctattattaccatatttctaaacccaaaatgtatgtg : 143700
Seq4 : cttgatgatgtcctgtatcagttgcattttcagcactcgactgaatattagcgcattcattgtctattattaccatatttctaaacccaaaatgtatgtg : 143700

               *     143720          *     143740          *     143760          *     143780          *     143800
Seq1 : ttgaacatcagtactatcgttgatgagtcttatagcatgaattcgcttatcgttatcgggtttatcttctgtcaccttagcaattcctttttattaaac : 143800
Seq2 : ttgaacatcagtactatcgttgatgagtcttatagcatgaattcgcttatcgttatcgggtttatcttctgtcaccttagcaattcctttttattaaac : 143800
Seq3 : ttgaacatcagtactatcgttgatgagtcttatagcatgaattcgcttatcgttatcgggtttatcttctgtcaccttagcaattcctttttattaaac : 143800
Seq4 : ttgaacatcagtactatcgttgatgagtcttatagcacgaatatccttatcgttatcgggtttatcttctgtcaccttagcaattcctttttattaaac : 143800

               *     143820          *     143840          *     143860          *     143880          *     143900
Seq1 : tctacataatcatatccatttctattgtttgttctaatataaacgagtatagcatcattgctaaattttcaatagtatcgaaaacagaatatcctaaac : 143900
Seq2 : tctacataatcatatccatttctattgtttgttctaatataaacgagtatagcatcattgctaaattttcaatagtatcgaaaacagaatatcctaaac : 143900
Seq3 : tctacataatcatatccatttctattgtttgttctaatataaacgagtatagcatcattgctaaattttcaatagtatcgaaaacagaatatcctaaac : 143900
Seq4 : tctacataatcatatccatttctattgtttgttctaatataaacgagtatagcatcattgctaaattttcaatagtatcgaaaacagaatatcctaaac : 143900

               *     143920          *     143940          *     143960          *     143980          *     144000
Seq1 : catataatatatattcagggacactcaaactaaatgtccaggattctcctaaatacgtaaactttaatagtgcgaaatcattcaaaaatctaccacttat : 144000
Seq2 : catataatatatattcagggacactcaaactaaatgtccaggattctcctaaatacgtaaactttaatagtgcgaaatcattcaaaaatctaccacttat : 144000
Seq3 : catataatatatattcagggacactcaaactaaatgtccaggattctcctaaatacgtaaactttaatagtgcgaaatcattcaaaaatctaccacttat : 144000
Seq4 : catataatatatattcagggacactcaaactaaatgtccaggattctcctaaatacgtaaactttaatagtgcgaaatcattcaaaaatctaccacttat : 144000

               *     144020          *     144040          *     144060          *     144080          *     144100
Seq1 : agatagatagtacataaatgcgtatagtagtctacctatctctttattatgaaaaccggcattacgatcatatatgtcgtgatatacctgtgatccgttt : 144100
Seq2 : agatagatagtacataaatgcgtatagtagtctacctatctctttattatgaaaaccggcattacgatcatatatgtcgtgatatacctgtgatccgttt : 144100
Seq3 : agatagatagtacataaatgcgtatagtagtctacctatctctttattatgaaaaccggcattacgatcatatatgtcgtgatatacctgtgatccgttt : 144100
Seq4 : agatagatagtacataaatgcgtatagtagtctacctatctctttattatgaaaaccggcattacgatcatatatgtcgtgatatacctgtgatccgttt : 144100

               *     144120          *     144140          *     144160          *     144180          *     144200
Seq1 : acgttaaaccataaatacatgggtgatcctataaacatgaatttatttctaattctcagagctatagttaattgaccgtgtaatatttgcttacatgcat : 144200
Seq2 : acgttaaaccataaatacatgggtgatcctataaacatgaatttatttctaattctcagagctatagttaattgaccgtgtaatatttgcttacatgcat : 144200
Seq3 : acgttaaaccataaatacatgggtgatcctataaacatgaatttatttctaattctcagagctatagttaattgaccgtgtaatatttgcttacatgcat : 144200
Seq4 : acgttaaaccataaatacatgggtgatcctataaacatgaatttatttctaattctcagagctatagttaattgaccgtgtaatatttgcttacatgcat : 144200
```

```
                   *     144220         *     144240         *     144260         *     144280         *     144300
Seq1 : acttgatacgctcattaataaaattttttatcattgctcgttatctcagaatcgtatatataaggagtaccatcgtgattcttaccagatattatacaaaa : 144300
Seq2 : acttgatacgctcattaataaaattttttatcattgctcgttatctcagaatcgtatatataaggagtaccatcgtgattcttaccagatattatacaaaa : 144300
Seq3 : acttgatacgctcattaataaaattttttatcattgctcgttatctcagaatcgtatatataaggagtaccatcgtgattcttaccagatattatacaaaa : 144300
Seq4 : -cttgatacgctcattaataaaattttttatcattgctcgttatctcagaatcgtatatataaggagtaccatcgtgattcttaccagatattatacaaaa : 144299

                   *     144320         *     144340         *     144360         *     144380         *     144400
Seq1 : tactatatataaaatatattgaccaacgttagtaatcatataaatgtttaacgttttaaatttgtattcaatgatccattatcatacgctagcatggtc : 144400
Seq2 : tactatatataaaatatattgaccaacgttagtaatcatataaatgtttaacgttttaaatttgtattcaatgatccattatcatacgctagcatggtc : 144400
Seq3 : tactatatataaaatatattgaccaacgttagtaatcatataaatgtttaacgttttaaatttgtattcaatgatccattatcatacgctagcatggtc : 144400
Seq4 : tactatatataaaatatattgaccaacgttagtaatcatataaatgtttaacgttttaaatttgtattcaatgatccattatcatacgctagcatggtc : 144399

                   *     144420         *     144440         *     144460         *     144480         *     144500
Seq1 : ttatgatattcattctttaaaatataatattgtgttagccattgcattggggctcctaatggagatttttattctcatccattttaggataggctttca : 144500
Seq2 : ttatgatattcattctttaaaatataatattgtgttagccattgcattggggctcctaatggagatttttattctcatccattttaggataggctttca : 144500
Seq3 : ttacgatattcattctttaaaatataatattgtgttagccattgcatt-gggctcctaatggagatttttattctcatccattttaggataggctttca : 144499
Seq4 : ttatgatattcattctttaaaatataatattgtgttagccattgcattggggctcctaatggagatttttattctcatccattttaggataggctttca : 144499

                   *     144520         *     144540         *     144560         *     144580         *     144600
Seq1 : taaagtccctaataacttcgtgaataatgtttctatgtttctactgatgcatgtatttgcttcgattttttatcccatgtttcatctatcatagattt : 144600
Seq2 : taaagtccctaataacttcgcgaataatgtttctacgtttctactgatgcatgtatttgcttcga-tttttatcccatgtttcatctatcatagattt : 144599
Seq3 : taaagtccctaataacttcgcgaataatgtttctacgtttctactgatgcatgtatttgcttcgattttttatcccatgtttcatctatcatagattt : 144599
Seq4 : taaagtcatcaataacttcgtgaataatgtttctatgatctctactgatgcatgtatttgcttcgattttttatcccatgtttcatctatcatagattt : 144599

                   *     144620         *     144640         *     144660         *     144680         *     144700
Seq1 : aaacgcagtaatgctcgcaacattaacatcttgaaccgttggtacaattccgttccataaatttataatgttcgccatcttatcgtcgtcatccttgtaa : 144700
Seq2 : aaacgcagtaatgctcgcaacattaacatcttgaaccgttggtacaattccgttccataaatttataatgttcgccatcttatcgtcgtcatccttgtaa : 144699
Seq3 : aaacgcagtaatgctcgcaacattaacatcttgaaccgttggtacaattccgttccataaatttataatgttcgccatcttatcgtcgtcatccttgtaa : 144699
Seq4 : aaacgcagtaatgctcgcaacattaacatcttgaaccgttggtacaattccgttccataaatttataatgttcgccatcttatcgtcgtcatccttgtaa : 144699

                   *     144720         *     144740         *     144760         *     144780         *     144800
Seq1 : tccatttatatgccaaaaaaaaaaaaaaaaaagctgatccaattgtcgacagagttaagttactcatatgggcgccgtccagtctgaacatcaatctttt : 144800
Seq2 : tccatttatatgcc-aaaaaaaaaaaaaaaaagctgatccaattgtcgacagagttaagttactcatatgggcgccgtccagtctgaacatcaatctttt : 144798
Seq3 : tccatttatatgccaaaaaaaaaaaaaaaaaagctgatccaattgtcgacagagttaagttactcatatgggcgccgtccagtctgaacatcaatctttt : 144799
Seq4 : tccatttatatgcc-aaaaaaaaaaaaaaaaagctgatccaattgtcgacagagttaagttactcatatgggcgccgtccagtctgaacatcaatctttt : 144798

                   *     144820         *     144840         *     144860         *     144880         *     144900
Seq1 : tagccagagatatcatagccgctcttagagtttcagcgtgattttccaacctaaatagaacttcatcgttgcgtttacaacacttttctatttgttcaaa : 144900
Seq2 : tagccagagatatcatagccgctcttagagtttcagcgtgattttccaacctaaatagaacttcatcgttgcgtttacaacacttttctatttgttcaaa : 144898
Seq3 : tagccagagatatcatagccgctcttagagtttcagcgtgattttccaacctaaatagaacttcatcgttgcgtttacaacacttttctatttgttcaaa : 144899
Seq4 : tagccagagatatcatagccgctcttagagtttcagcgtgattttccaacctaaatagaacttcatcgttgcgtttacaacacttttctatttgttcaaa : 144898
```

```
            *    144920         *    144940         *    144960         *    144980         *    145000
Seq1 : ctttgttgttacattagtaatctttttttccaaattagttagccgttgtttgagagtttcctcattgtcgtcttcatcggctttaacaattgcttcgcgt : 145000
Seq2 : ctttgttgttacattagtaatctttttttccaaattagttagccgttgtttgagagtttcctcattgtcgtcttcatcggctttaacaattgcttcgcgt : 144998
Seq3 : ctttgttgttacattagtaatctttttttccaaattagttagccgttgtttgagagtttcctcattgtcgtcttcatcggctttaacaattgcttcgcgt : 144999
Seq4 : ctttgttgttacattagtaatctttttttccaaattagttagccgttgtttgagagtttcctcattgtcgtcttcatcggctttaacaattgcttcgcgt : 144998

            *    145020         *    145040         *    145060         *    145080         *    145100
Seq1 : ttagcctctggctttttagcagcctttgtagaaaaaaattcagttgctggaattgcaagatcgtcatctccggggaaaagagttccgtccatttaaagta : 145100
Seq2 : ttagcctctggctttttagcagcctttgtagaaaaaaattcagttgctggaattgcaagatcgtcatctccggggaaaagagttccgtccatttaaagta : 145098
Seq3 : ttagcctctggctttttagcagcctttgtagaaaaaaattcagttgctggaattgcaagatcgtcatctccggggaaaagagttccgtccatttaaagta : 145099
Seq4 : ttagcctctggctttttagcagcctttgtagaaaaaaattcagttgctggaattgcaagatcgtcatctccggggaaaagagttccgtccatttaaagta : 145098

            *    145120         *    145140         *    145160         *    145180         *    145200
Seq1 : cagattttagaaactgacactctgcgttatttatatttggtacaacacatggattataaatatcgatgttaataacatcagaaaatgtaaagtctataca : 145200
Seq2 : cagattttagaaactgacactctgcgttatttatatttggtacaacacatggattataaatatcgatgttaataacatcagaaaatgtaaagtctataca : 145198
Seq3 : cagattttagaaactgacactctgcgttatttatatttggtacaacacatggattataaatatcgatgttaataacatcagaaaatgtaaagtctataca : 145199
Seq4 : cagattttagaaactgacactctgcgttatttatatttggtacaacacatggattataaatatcgatgttaataacatcagaaaatgtaaagtctataca : 145198

            *    145220         *    145240         *    145260         *    145280         *    145300
Seq1 : ttgttgcatcgtgttaaattttctaatggatctagtattattgggtccaacttctgcctgaaatccaaatatggaagcggatacaaaaccgtttcctgga : 145300
Seq2 : ttgttgcatcgtgttaaattttctaatggatctagtattattgggtccaacttctgcctgaaatccaaatatggaagcggatacaaaaccgtttcctgga : 145298
Seq3 : ttgttgcatcgtgttaaattttctaatggatctagtattattgggtccaacttctgcctgaaatccaaatatggaagcggatacaaaaccgtttcctgga : 145299
Seq4 : ttgttgcatcgtgttaaattttctaatggatctagtattattgggtccaacttctgcctgaaatccaaatatggaagcggatacaaaaccgtttcctgga : 145298

            *    145320         *    145340         *    145360         *    145380         *    145400
Seq1 : taaaccacacatctccactttttgctttacatcagaaattgtgtcgttgacatcttgaactctcctatctaatgccggtgttccacctatagattttgaat : 145400
Seq2 : taaaccacacatctccactttttgctttacatcagaaattgtgtcgttgacatcttgaactctcctatctaatgccggtgttccacctatagattttgaat : 145398
Seq3 : taaaccacacatctccactttttgctttacatcagaaattgtgtcgttgacatcttgaactctcctatctaatgccggtgttccacctatagattttgaat : 145399
Seq4 : taaaccacacatctccactttttgctttacatcagaaattgtgtcgttgacatcttgaactctcctatctaatgccggtgttccacctatagattttgaat : 145398

            *    145420         *    145440         *    145460         *    145480         *    145500
Seq1 : attcgaatgctgcatgagtagcattaaattccttaatattgccataattttcatatattgagtaaccctggataaaaagtaaacacaccgcagccgtcgc : 145500
Seq2 : attcgaatgctgcatgagtagcattaaattccttaatattgccataattttcatatattgagtaaccctggataaaaagtaaacacaccgcagccgtcgc : 145498
Seq3 : attcgaatgctgcatgagtagcattaaattccttaatattgccataattttcatatattgagtaaccctggataaaaagtaaacacaccgcagccgtcgc : 145499
Seq4 : attcgaatgctgcatgagtagcattaaattccttaatattgccataattttcatatattgagtaaccctggataaaaagtaaacacaccgcagccgtcgc : 145498

            *    145520         *    145540         *    145560         *    145580         *    145600
Seq1 : taccacaataaaaaaaattgatagagagttcatttataatctattagaagctgacaaaatttttttacacgcatcagacaatgctttaataaatagttca : 145600
Seq2 : taccacaataaaaaaaattgatagagagttcatttataatctattagaagctgacaaaatttttttacacgcatcagacaatgctttaataaatagttca : 145598
Seq3 : taccacaataaaaaaaattgatagagagttcatttataatctattagaagctgacaaaatttttttacacgcatcagacaatgctttaataaatagttca : 145599
Seq4 : taccacaataaaaaaaattgatagagagttcatttataatctattagaagctgacaaaatttttttacacgcatcagacaatgctttaataaatagttca : 145598
```

```
                  *    145620         *    145640         *    145660         *    145680         *    145700
Seq1 : acatctacttttgtcatatcgaaccgatggtatgattctaacctagaattacatccgaaaaagttgactatgttcatagtcattaagtcattaacaaaca : 145700
Seq2 : acatctacttttgtcatatcgaaccgatggtatgattctaacctagaattacatccgaaaaagttgactatgttcatagtcattaagtcattaacaaaca : 145698
Seq3 : acatctacttttgtcatatcgaaccgatggtatgattctaacctagaattacatccgaaaaagttgactatgttcatagtcattaagtcattaacaaaca : 145699
Seq4 : acatctacttttgtcatatcgaaccgatggtatgattctaacctagaattacatccgaaaaagttgactatgttcatagtcattaagtcattaacaaaca : 145698

                  *    145720         *    145740         *    145760         *    145780         *    145800
Seq1 : acattccagactctggattataagacgatactgtttcgtcacaattacctaccttaatcatgtgattatgaatattggctattagagcaccttctaagaa : 145800
Seq2 : acattccagactctggattataagacgatactgtttcgtcacaattacctaccttaatcatgtgattatgaatattggctattagagcaccttctaagaa : 145798
Seq3 : acattccagactctggattataagacgatactgtttcgtcacaattacctaccttaatcatgtgattatgaatattggctattagagcaccttctaagaa : 145799
Seq4 : acattccagactctggattataagacgatactgtttcgtcacaattacctaccttaatcatgtgattatgaatattggctattagagcaccttctaagaa : 145798

                  *    145820         *    145840         *    145860         *    145880         *    145900
Seq1 : atctataatatctttgaaacacgatttaaaatcaaaccacgaatatacttctacgaagaaagttagtttacccataggagaaataactataaatggagat : 145900
Seq2 : atctataatatctttgaaacacgatttaaaatcaaaccacgaatatacttctacgaagaaagttagtttacccataggagaaataactataaatggagat : 145898
Seq3 : atctataatatctttgaaacacgatttaaaatcaaaccacgaatatacttctacgaagaaagttagtttacccataggagaaataactataaatggagat : 145899
Seq4 : atctataatatctttgaaacacgatttaaaatcaaaccacgaatatacttctacgaagaaagttagtttacccataggagaaataactataaatggagat : 145898

                  *    145920         *    145940         *    145960         *    145980         *    146000
Seq1 : ctaaatacaaaatccggatctatgatagttttaacattattatattctctattaaatacctccacatctaaaaatgttaattttgaaactatgtcttcgt : 146000
Seq2 : ctaaatacaaaatccggatctatgatagttttaacattattatattctctattaaatacctccacatctaaaaatgttaattttgaaactatgtcttcgt : 145998
Seq3 : ctaaatacaaaatccggatctatgatagttttaacattattatattctctattaaatacctccacatctaaaaatgttaattttgaaactatgtcttcgt : 145999
Seq4 : ctaaatacaaaatccggatctatgatagttttaacattattatattctctattaaatacctccacatctaaaaatgttaattttgaaactatgtcttcgt : 145998

                  *    146020         *    146040         *    146060         *    146080         *    146100
Seq1 : ttattaccgtacctgaactaaacgctataagctctattgtttgagaactctttaaacgatattcttgaaatacatgtaacaaagtttcctttaactcggt : 146100
Seq2 : ttattaccgtacctgaactaaacgctataagctctattgtttgagaactctttaaacgatattcttgaaatacatgtaacaaagtttcctttaactcggt : 146098
Seq3 : ttattaccgtacctgaactaaacgctataagctctattgtttgagaactctttaaacgatattcttgaaatacatgtaacaaagtttcctttaactcggt : 146099
Seq4 : ttattaccgtacctgaactaaacgctataagctctattgtttgagaactctttaaacgatattcttgaaatacatgtaacaaagtttcctttaactcggt : 146098

                  *    146120         *    146140         *    146160         *    146180         *    146200
Seq1 : cggtttatctaccatagttacagaatttgtatccttatctataatataataatcaaaatcgtataaagttatataattatcgcgttcagattgggatctt : 146200
Seq2 : cggtttatctaccatagttacagaatttgtatccttatctataatataataatcaaaatcgtataaagttatataattatcgcgttcagattgggatctt : 146198
Seq3 : cggtttatctaccatagttacagaatttgtatccttatctataatataataatcaaaatcgtataaagttatataattatcgcgttcagattgggatctt : 146199
Seq4 : cggtttatctaccatagttacagaatttgtatccttatctataatataataatcaaaatcgtataaagttatataattatcgcgttcagattgggatctt : 146198

                  *    146220         *    146240         *    146260         *    146280         *    146300
Seq1 : ttcaaatagactaaaaaccccatttctctagtaagtatcttatgtatatgtttgtaaaatatcttcatggtgggaatatgctctaccgcagttagccatt : 146300
Seq2 : ttcaaatagactaaaaaccccatttctctagtaagtatcttatgtatatgtttgtaaaatatcttcatggtgggaatatgctctaccgcagttagccatt : 146298
Seq3 : ttcaaatagactaaaaaccccatttctctagtaagtatcttatgtatatgtttgtaaaatatcttcatggtgggaatatgctctaccgcagttagccatt : 146299
Seq4 : ttcaaatagactaaaaaccccatttctctagtaagtatcttatgtatatgtttgtaaaatatcttcatggtgggaatatgctctaccgcagttagccatt : 146298
```

```
           *    146320          *    146340          *    146360          *    146380          *    146400
Seq1 : cctcattgacagcggtagatgtattagacaaaactattccaatgtttaacaagggccattttacgagattattaaatccttgtttgataaatgtagccaa : 146400
Seq2 : cctcattgacagcggtagatgtattagacaaaactattccaatgtttaacaagggccattttacgagattattaaatccttgtttgataaatgtagccaa : 146398
Seq3 : cctcattgacagcggtagatgtattagacaaaactattccaatgtttaacaagggccattttacgagattattaaatccttgtttgataaatgtagccaa : 146399
Seq4 : cctcattgacagcggtagatgtattagacaaaactattccaatgtttaacaagggccattttacgagattattaaatccttgtttgataaatgtagccaa : 146398

           *    146420          *    146440          *    146460          *    146480          *    146500
Seq1 : tgagggttcgagttcaacgacgattgaattctcttcccgcggatgctgcatgatgaacgacgggatgttgttcgattgatttggaattctttttcgactt : 146500
Seq2 : tgagggttcgagttcaacgacgattgaattctcttcccgcggatgctgcatgatgaacgacgggatgttgttcgattgatttggaattctttttcgactt : 146498
Seq3 : tgagggttcgagttcaacgacgattgaattctcttcccgcggatgctgcatgatgaacgacgggatgttgttcgattgatttggaattctttttcgactt : 146499
Seq4 : tgagggttcgagttcaacgacgattgaattctcttcccgcggatgctgcatgatgaacgacgggatgttgttcgattgatttggaattctttttcgactt : 146498

           *    146520          *    146540          *    146560          *    146580          *    146600
Seq1 : tttgtttatattaaatattttaaaatttatagcggatagcaattcatgtaccacggataatgtagacgcgtattgcgcatcgatatctttattattagat : 146600
Seq2 : tttgtttatattaaatattttaaaatttatagcggatagcaattcatgtaccacggataatgtagacgcgtattgcgcatcgatatctttattattagat : 146598
Seq3 : tttgtttatattaaatattttaaaatttatagcggatagcaattcatgtaccacggataatgtagacgcgtattgcgcatcgatatctttattattagat : 146599
Seq4 : tttgtttatattaaatattttaaaatttatagcggatagcaattcatgtaccacggataatgtagacgcgtattgcgcatcgatatctttattattagat : 146598

           *    146620          *    146640          *    146660          *    146680          *    146700
Seq1 : aaatttatcaataaatgtgagaagtttgcctcgttaaggtcttccatttaaatattatataaacatttgtgtttgtatcttattcgtcttttatggaata : 146700
Seq2 : aaatttatcaataaatgtgagaagtttgcctcgttaaggtcttccatttaaatattatataaacatttgtgtttgtatcttattcgtcttttatggaata : 146698
Seq3 : aaatttatcaataaatgtgagaagtttgcctcgttaaggtcttccatttaaatattatataaacatttgtgtttgtatcttattcgtcttttatggaata : 146699
Seq4 : aaatttatcaataaatgtgagaagtttgcctcgttaaggtcttccatttaaatattatataaacatttgtgtttgtatcttattcgtcttttatggaata : 146698

           *    146720          *    146740          *    146760          *    146780          *    146800
Seq1 : gtttttactagtaaagctgcaattacacactttgtccgtaaaacataaatataaacaccagcttttatcaatcgttccaaaaagtcgacggcggacatt : 146800
Seq2 : gtttttactagtaaagctgcaattacacactttgtccgtaaaacataaatataaacaccagcttttatcaatcgttccaaaaagtcgacggcggacatt : 146798
Seq3 : gtttttactagtaaagctgcaattacacactttgtccgtaaaacataaatataaacaccagcttttatcaatcgttccaaaaagtcgacggcggacatt : 146799
Seq4 : gtttttactagtaaagctgcaattacacactttgtccgtaaaacataaatataaacaccagcttttatcaatcgttccaaaaagtcgacggcggacatt : 146798

           *    146820          *    146840          *    146860          *    146880          *    146900
Seq1 : tttaacatggcatctattttaaatacacttaggtttttggaaaaaacatcattttataattgtaacgattcaataactaaagaaaagattaagattaaac : 146900
Seq2 : tttaacatggcatctattttaaatacacttaggtttttggaaaaaacatcattttataattgtaacgattcaataactaaagaaaagattaagattaaac : 146898
Seq3 : tttaacatggcatctattttaaatacacttaggtttttggaaaaaacatcattttataattgtaacgattcaataactaaagaaaagattaagattaaac : 146899
Seq4 : tttaacatggcatctattttaaatacacttaggtttttggaaaaaacatcattttataattgtaacgattcaataactaaagaaaagattaagattaaac : 146898

           *    146920          *    146940          *    146960          *    146980          *    147000
Seq1 : ataagggaatgtcatttgtattttataagccaaagcattctaccgttgttaaatacttgtctggaggaggtatatatcatgatgatttggttgtattggg : 147000
Seq2 : ataagggaatgtcatttgtattttataagccaaagcattctaccgttgttaaatacttgtctggaggaggtatatatcatgatgatttggttgtattggg : 146998
Seq3 : ataagggaatgtcatttgtattttataagccaaagcattctaccgttgttaaatacttgtctggaggaggtatatatcatgatgatttggttgtattggg : 146999
Seq4 : ataagggaatgtcatttgtattttataagccaaagcattctaccgttgttaaatacttgtctggaggaggtatatatcatgatgatttggttgtattggg : 146998
```

```
              *    147020          *    147040          *    147060          *    147080          *    147100
Seq1 : gaaggtaacaattaataatctaaagatgatgctattttacatggatttatcatatcatggagtgacaagtagtggagcaatttacaaattgggatcgtct : 147100
Seq2 : gaaggtaacaattaataatctaaagatgatgctattttacatggatttatcatatcatggagtgacaagtagtggagcaatttacaaattgggatcgtct : 147098
Seq3 : gaaggtaacaattaataatctaaagatgatgctattttacatggatttatcatatcatggagtgacaagtagtggagcaatttacaaattgggatcgtct : 147099
Seq4 : gaaggtaacaattaataatctaaagatgatgctattttacatggatttatcatatcatggagtgacaagtagtggagcaatttacaaattgggatcgtct : 147098

              *    147120          *    147140          *    147160          *    147180          *    147200
Seq1 : atcgatagactttctctaaataggactattgttacaaaagttaataattatgatgatacatttttttgacgacgatgattgatcgctattgcacaattttg : 147200
Seq2 : atcgatagactttctctaaataggactattgttacaaaagttaataattatgatgatacatttttttgacgacgatgattgatcgctattgcacaattttg : 147198
Seq3 : atcgatagactttctctaaataggactattgttacaaaagttaataattatgatgatacatttttttgacgacgatgattgatcgctattgcacaattttg : 147199
Seq4 : atcgatagactttctctaaataggactattgttacaaaagttaataattatgatgatacatttttttgacgacgatgattgatcgctattgcacaattttg : 147198

              *    147220          *    147240          *    147260          *    147280          *    147300
Seq1 : tttttttactttctaatatagcgtttagattctttttcatgtgcgaatattgatttactaaaatatcgatgtttaacttttgttctatgacgtccttatc : 147300
Seq2 : tttttttactttctaatatagcgtttagattctttttcatgtgcgaatattgatttactaaaatatcgatgtttaacttttgttctatgacgtccttatc : 147298
Seq3 : tttttttactttctaatatagcgtttagattctttttcatgtgcgaatattgatttactaaaatatcgatgtttaacttttgttctatgacgtccttatc : 147299
Seq4 : tttttttactttctaatatagcgtttagattctttttcatgtgcgaatattgatttactaaaatatcgatgtttaacttttgttctatgacgtccttatc : 147298

              *    147320          *    147340          *    147360          *    147380          *    147400
Seq1 : agcggtatcggtacatatacgtaattcaccttcacaaaatacggagtcttcgataataatagccaatcgattattggatctagctgtctgtatcatattc : 147400
Seq2 : agcggtatcggtacatatacgtaattcaccttcacaaaatacggagtcttcgataataatagccaatcgattattggatctagctgtctgtatcatattc : 147398
Seq3 : agcggtatcggtacatatacgtaattcaccttcacaaaatacggagtcttcgataataatagccaatcgattattggatctagctgtctgtatcatattc : 147399
Seq4 : agcggtatcggtacatatacgtaattcaccttcacaaaatacggagtcttcgataataatagccaatcgattattggatctagctgtctgtatcatattc : 147398

              *    147420          *    147440          *    147460          *    147480          *    147500
Seq1 : aacatgtttaatatatcctttcgtttcccctttacaggcatcgatcgtagcatattttccgcgtctgagatggaaatgttaaaactacaaaaatgcgtaa : 147500
Seq2 : aacatgtttaatatatcctttcgtttcccctttacaggcatcgatcgtagcatattttccgcgtctgagatggaaatgttaaaactacaaaaatgcgtaa : 147498
Seq3 : aacatgtttaatatatcctttcgtttcccctttacaggcatcgatcgtagcatattttccgcgtctgagatggaaatgttaaaactacaaaaatgcgtaa : 147499
Seq4 : aacatgtttaatatatcctttcgtttcccctttacaggcatcgatcgtagcatattttccgcgtctgagatggaaatgttaaaactacaaaaatgcgtaa : 147498

              *    147520          *    147540          *    147560          *    147580          *    147600
Seq1 : tgttagcccgtcctaatattggtacgtgtctataagtttggcatagtagaataatagacgtgtttaaatgccttccaaagtttaagaattctattagagt : 147600
Seq2 : tgttagcccgtcctaatattggtacgtgtctataagtttggcatagtagaataatagacgtgtttaaatgccttccaaagtttaagaattctattagagt : 147598
Seq3 : tgttagcccgtcctaatattggtacgtgtctataagtttggcatagtagaataatagacgtgtttaaatgccttccaaagtttaagaattctattagagt : 147599
Seq4 : tgttagcccgtcctaatattggtacgtgtctataagtttggcatagtagaataatagacgtgtttaaatgccttccaaagtttaagaattctattagagt : 147598

              *    147620          *    147640          *    147660          *    147680          *    147700
Seq1 : attgcattttgatagtttatcacctacatcatcaaaaataagtaaaaagtgtgctgatttttttatgattttgtgcgacagcaatacatttttctatgtta : 147700
Seq2 : attgcattttgatagtttatcacctacatcatcaaaaataagtaaaaagtgtgctgatttttttatgattttgtgcgacagcaatacatttttctatgtta : 147698
Seq3 : attgcattttgatagtttatcacctacatcatcaaaaataagtaaaaagtgtgctgatttttttatgattttgtgcgacagcaatacatttttctatgtta : 147699
Seq4 : attgcattttgatagtttatcacctacatcatcaaaaataagtaaaaagtgtgctgatttttttatgattttgtgcgacagcaatacatttttctatgtta : 147698
```

```
                 *    147720          *    147740          *    147760          *    147780          *    147800
Seq1 : cttttagttcgtatcagattatattctagagattcctgactactaacgaaattaatatgatttggccaaatgtatccatcataatctgggttataaacgg : 147800
Seq2 : cttttagttcgtatcagattatattctagagattcctgactactaacgaaattaatatgatttggccaaatgtatccatcataatctgggttataaacgg : 147798
Seq3 : cttttagttcgtatcagattatattctagagattcctgactactaacgaaattaatatgatttggccaaatgtatccatcataatctgggttataaacgg : 147799
Seq4 : cttttagttcgtatcagattatattctagagattcctgactactaacgaaattaatatgatttggccaaatgtatccatcataatctgggttataaacgg : 147798

                 *    147820          *    147840          *    147860          *    147880          *    147900
Seq1 : gtgtaaacaagaatatatgtttatatttttaactagtgtagaaaacagagatagtaaatagatagtttttccagatccagatcctcccgttaaaaccat : 147900
Seq2 : gtgtaaacaagaatatatgtttatatttttaactagtgtagaaaacagagatagtaaatagatagtttttccagatccagatcctcccgttaaaaccat : 147898
Seq3 : gtgtaaacaagaatatatgtttatatttttaactagtgtagaaaacagagatagtaaatagatagtttttccagatccagatcctcccgttaaaaccat : 147899
Seq4 : gtgtaaacaagaatatatgtttatatttttaactagtgtagaaaacagagatagtaaatagatagtttttccagatccagatcctcccgttaaaaccat : 147898

                 *    147920          *    147940          *    147960          *    147980          *    148000
Seq1 : tctaaacggcatttttaataaatttctcttgaaaattgtttttcttggaaacaattcataattatatttacagttactaaattaatttgataataaatc : 148000
Seq2 : tctaaacggcatttttaataaatttctcttgaaaattgtttttcttggaaacaattcataattatatttacagttactaaattaatttgataataaatc : 147998
Seq3 : tctaaacggcatttttaataaatttctcttgaaaattgtttttcttggaaacaattcataattatatttacagttactaaattaatttgataataaatc : 147999
Seq4 : tctaaacggcatttttaataaatttctcttgaaaattgtttttcttggaaacaattcataattatatttacagttactaaattaatttgataataaatc : 147998

                 *    148020          *    148040          *    148060          *    148080          *    148100
Seq1 : aaaatatggaaaactaaggtcgttagtagggaggagaacaaagaaggcacatcgtgacataaataacatttattatcatgatgacaccagaaaacgacga : 148100
Seq2 : aaaatatggaaaactaaggtcgttagtagggaggagaacaaagaaggcacatcgtgacataaataacatttattatcatgatgacaccagaaaacgacga : 148098
Seq3 : aaaatatggaaaactaaggtcgttagtagggaggagaacaaagaaggcacatcgtgacataaataacatttattatcatgatgacaccagaaaacgacga : 148099
Seq4 : aaaatatggaaaactaaggtcgttagtagggaggagaacaaagaaggcacatcgtgacataaataacatttattatcatgatgacaccagaaaacgacga : 148098

                 *    148120          *    148140          *    148160          *    148180          *    148200
Seq1 : agagcagacatctgtgttctccgctactgtttacggagacaaaattcaaggaaagaataaacgcaaacgcgtgattggtctatgtattagaatatctatg : 148200
Seq2 : agagcagacatctgtgttctccgctactgtttacggagacaaaattcaaggaaagaataaacgcaaacgcgtgattggtctatgtattagaatatctatg : 148198
Seq3 : agagcagacatctgtgttctccgctactgtttacggagacaaaattcaaggaaagaataaacgcaaacgcgtgattggtctatgtattagaatatctatg : 148199
Seq4 : agagcagacatctgtgttctccgctactgtttacggagacaaaattcaaggaaagaataaacgcaaacgcgtgattggtctatgtattagaatatctatg : 148198

                 *    148220          *    148240          *    148260          *    148280          *    148300
Seq1 : gttatttcactactatctatgattaccatgtccgcgtttctcatagtgcgcctaaatcaatgcatgtctgctaacgaggctgctattactgacgccgctg : 148300
Seq2 : gttatttcactactatctatgattaccatgtccgcgtttctcatagtgcgcctaaatcaatgcatgtctgctaacgaggctgctattactgacgccgctg : 148298
Seq3 : gttatttcactactatctatgattaccatgtccgcgtttctcatagtgcgcctaaatcaatgcatgtctgctaacgaggctgctattactgacgccgctg : 148299
Seq4 : gttatttcactactatctatgattaccatgtccgcgtttctcatagtgcgcctaaatcaatgcatgtctgctaacgaggctgctattactgacgccgctg : 148298

                 *    148320          *    148340          *    148360          *    148380          *    148400
Seq1 : ttgccgttgctgctgcatcatctactcatagaaaggttgcgtctagcactacacaatatgatcacaaagaaagctgtaatggtttatattaccagggttc : 148400
Seq2 : ttgccgttgctgctgcatcatctactcatagaaaggttgcgtctagcactacacaatatgatcacaaagaaagctgtaatggtttatattaccagggttc : 148398
Seq3 : ttgccgttgctgctgcatcatctactcatagaaaggttgcgtctagcactacacaatatgatcacaaagaaagctgtaatggtttatattaccagggttc : 148399
Seq4 : ttgccgttgctgctgcatcatctactcatagaaaggttgcgtctagcactacacaatatgatcacaaagaaagctgtaatggtttatattaccagggttc : 148398
```

```
                 *    148420          *    148440          *    148460          *    148480          *    148500
Seq1 : ttgttatatattacattcagactaccagttattctcggatgctaaagcaaattgcactgcggaatcatcaacactacccaataaatccgatgtcttgatt : 148500
Seq2 : ttgttatatattacattcagactaccagttattctcggatgctaaagcaaattgcactgcggaatcatcaacactacccaataaatccgatgtcttgatt : 148498
Seq3 : ttgttatatattacattcagactaccagttattctcggatgctaaagcaaattgcactgcggaatcatcaacactacccaataaatccgatgtcttgatt : 148499
Seq4 : ttgttatatattacattcagactaccagttattctcggatgctaaagcaaattgcactgcggaatcatcaacactacccaataaatccgatgtcttgatt : 148498

                 *    148520          *    148540          *    148560          *    148580          *    148600
Seq1 : acctggctcattgattatgttgaggatacatggggatctgatggtaatccaattacaaaaactacatccgattatcaagattctgatgtatcacaagaag : 148600
Seq2 : acctggctcattgattatgttgaggatacatggggatctgatggtaatccaattacaaaaactacatccgattatcaagattctgatgtatcacaagaag : 148598
Seq3 : acctggctcattgattatgttgaggatacatggggatctgatggtaatccaattacaaaaactacatccgattatcaagattctgatgtatcacaagaag : 148599
Seq4 : acctggctcattgattatgttgaggatacatggggatctgatggtaatccaattacaaaaactacatccgattatcaagattctgatgtatcacaagaag : 148598

                 *    148620          *    148640          *    148660          *    148680          *    148700
Seq1 : ttagaaagtattttgtgttaaaacaatgaactaatatttattttgtacattaataaatgaaatcgcttaatagacaaactgtaagtaggtttaagaag : 148700
Seq2 : ttagaaagtattttgtgttaaaacaatgaactaatatttattttgtacattaataaatgaaatcgcttaatagacaaactgtaagtaggtttaagaag : 148698
Seq3 : ttagaaagtattttgtgttaaaacaatgaactaatatttattttgtacattaataaatgaaatcgcttaatagacaaactgtaagtaggtttaagaag : 148699
Seq4 : ttagaaagtattttgtgttaaaacaatgaactaatatttattttgtacattaataaatgaaatcgcttaatagacaaactgtaagtaggtttaagaag : 148698

                 *    148720          *    148740          *    148760          *    148780          *    148800
Seq1 : ttgtcggtgccggccgctataatgatgatactctcaaccattattagtggcataggaacatttctgcattacaaagaagaactgatgcctagtgcttgcg : 148800
Seq2 : ttgtcggtgccggccgctataatgatgatactctcaaccattattagtggcataggaacatttctgcattacaaagaagaactgatgcctagtgcttgcg : 148798
Seq3 : ttgtcggtgccggccgctataatgatgatactctcaaccattattagtggcataggaacatttctgcattacaaagaagaactgatgcctagtgcttgcg : 148799
Seq4 : ttgtcggtgccggccgctataatgatgatactctcaaccattattagtggcataggaacatttctgcattacaaagaagaactgatgcctagtgcttgcg : 148798

                 *    148820          *    148840          *    148860          *    148880          *    148900
Seq1 : ccaatggatggatacaatacgataaacattgttatttagatactaacattaaaatgtctacagataatgcggtttatcagtgtcgtaaattacgagccag : 148900
Seq2 : ccaatggatggatacaatacgataaacattgttatttagatactaacattaaaatgtctacagataatgcggtttatcagtgtcgtaaattacgagccag : 148898
Seq3 : ccaatggatggatacaatacgataaacattgttatttagatactaacattaaaatgtctacagataatgcggtttatcagtgtcgtaaattacgagccag : 148899
Seq4 : ccaatggatggatacaatacgataaacattgttatttagatactaacattaaaatgtctacagataatgcggtttatcagtgtcgtaaattacgagccag : 148898

                 *    148920          *    148940          *    148960          *    148980          *    149000
Seq1 : attgcctagaccggatactagacatctgagagtattgtttagtatttttataaagattattgggtaagtttaaaaaagaccaatgataaatggttagat : 149000
Seq2 : attgcctagaccggatactagacatctgagagtattgtttagtatttttataaagattattgggtaagtttaaaaaagaccaatgataaatggttagat : 148998
Seq3 : attgcctagaccggatactagacatctgagagtattgtttagtatttttataaagattattgggtaagtttaaaaaagaccaatgataaatggttagat : 148999
Seq4 : attgcctagaccggatactagacatctgagagtattgtttagtatttttataaagattattgggtaagtttaaaaaagaccaatgataaatggttagat : 148998

                 *    149020          *    149040          *    149060          *    149080          *    149100
Seq1 : attaataatgataaagatatagatattagtaaattaacaaattttaaacaactaaacagtacgacggatgctgaagcgtgttatatatacaagtctggaa : 149100
Seq2 : attaataatgataaagatatagatattagtaaattaacaaattttaaacaactaaacagtacgacggatgctgaagcgtgttatatatacaagtctggaa : 149098
Seq3 : attaataatgataaagatatagatattagtaaattaacaaattttaaacaactaaacagtacgacggatgctgaagcgtgttatatatacaagtctggaa : 149099
Seq4 : attaataatgataaagatatagatattagtaaattaacaaattttaaacaactaaacagtacgacggatgctgaagcgtgttatatatacaagtctggaa : 149098
```

```
                   *    149120         *    149140         *    149160         *    149180         *    149200
Seq1 : aactggttaaaacagtatgtaaaagtactcaatctgtactatgtgttaaaaaattctacaagtgacaacaaaaaatgaattaataataagtcgttaacgt : 149200
Seq2 : aactggttaaaacagtatgtaaaagtactcaatctgtactatgtgttaaaaaattctacaagtgacaacaaaaaatgaattaataataagtcgttaacgt : 149198
Seq3 : aactggttaaaacagtatgtaaaagtactcaatctgtactatgtgttaaaaaattctacaagtgacaacaaaaaatgaattaataataagtcgttaacgt : 149199
Seq4 : aactggttaaaacagtatgtaaaagtactcaatctgtactatgtgttaaaaaattctacaagtgacaacaaaaaatgaattaataataagtcgttaacgt : 149198

                   *    149220         *    149240         *    149260         *    149280         *    149300
Seq1 : acgccgccatggacgccgcgtttgttattactccaatgggtgtgttgactataacagatacattgtatgatgatctcgatatctcaatcatggactttat : 149300
Seq2 : acgccgccatggacgccgcgtttgttattactccaatgggtgtgttgactataacagatacattgtatgatgatctcgatatctcaatcatggactttat : 149298
Seq3 : acgccgccatggacgccgcgtttgttattactccaatgggtgtgttgactataacagatacattgtatgatgatctcgatatctcaatcatggactttat : 149299
Seq4 : acgccgccatggacgccgcgtttgttattactccaatgggtgtgttgactataacagatacattgtatgatgatctcgatatctcaatcatggactttat : 149298

                   *    149320         *    149340         *    149360         *    149380         *    149400
Seq1 : aggaccatacattataggtaacataaaaactgtccaaatagatgtacgggatataaaatattccgacatgcaaaaatgctactttagctataagggtaaa : 149400
Seq2 : aggaccatacattataggtaacataaaaactgtccaaatagatgtacgggatataaaatattccgacatgcaaaaatgctactttagctataagggtaaa : 149398
Seq3 : aggaccatacattataggtaacataaaaactgtccaaatagatgtacgggatataaaatattccgacatgcaaaaatgctactttagctataagggtaaa : 149399
Seq4 : aggaccatacattataggtaacataaaaactgtccaaatagatgtacgggatataaaatattccgacatgcaaaaatgctactttagctataagggtaaa : 149398

                   *    149420         *    149440         *    149460         *    149480         *    149500
Seq1 : atagttcctcaggattctaatgatttggctagattcaacatttatagcatttgtgccgcatacagatcaaaaaataccatcatcatagcatgcgactatg : 149500
Seq2 : atagttcctcaggattctaatgatttggctagattcaacatttatagcatttgtgccgcatacagatcaaaaaataccatcatcatagcatgcgactatg : 149498
Seq3 : atagttcctcaggattctaatgatttggctagattcaacatttatagcatttgtgccgcatacagatcaaaaaataccatcatcatagcatgcgactatg : 149499
Seq4 : atagttcctcaggattctaatgatttggctagattcaacatttatagcatttgtgccgcatacagatcaaaaaataccatcatcatagcatgcgactatg : 149498

                   *    149520         *    149540         *    149560         *    149580         *    149600
Seq1 : atatcatgttagatatagaagataaacatcagccattttatctattcccatctattgatgtttttaacgctacaatcatagaagcgtataacctgtatac : 149600
Seq2 : atatcatgttagatatagaagataaacatcagccattttatctattcccatctattgatgtttttaacgctacaatcatagaagcgtataacctgtatac : 149598
Seq3 : atatcatgttagatatagaagataaacatcagccattttatctattcccatctattgatgtttttaacgctacaatcatagaagcgtataacctgtatac : 149599
Seq4 : atatcatgttagatatagaagataaacatcagccattttatctattcccatctattgatgtttttaacgctacaatcatagaagcgtataacctgtatac : 149598

                   *    149620         *    149640         *    149660         *    149680         *    149700
Seq1 : agctggagattatcatctaatcatcaatccttcagataatctgaaaatgaaattgttgtttaattcttcattctgcatatcagacggcaatggatggatc : 149700
Seq2 : agctggagattatcatctaatcatcaatccttcagataatctgaaaatgaaattgttgtttaattcttcattctgcatatcagacggcaatggatggatc : 149698
Seq3 : agctggagattatcatctaatcatcaatccttcagataatctgaaaatgaaattgttgtttaattcttcattctgcatatcagacggcaatggatggatc : 149699
Seq4 : agctggagattatcatctaatcatcaatccttcagataatctgaaaatgaaattgttgtttaattcttcattctgcatatcagacggcaatggatggatc : 149698

                   *    149720         *    149740         *    149760         *    149780         *    149800
Seq1 : ataattgatgggaaatgcaatagtaatttttttatcataaaagttgtaaagtaaataataaaacaataaatattgaactagtagtacgtatattgagcaat : 149800
Seq2 : ataattgatgggaaatgcaatagtaatttttttatcataaaagttgtaaagtaaataataaaacaataaatattgaactagtagtacgtatattgagcaat : 149798
Seq3 : ataattgatgggaaatgcaatagtaatttttttatcataaaagttgtaaagtaaataataaaacaataaatattgaactagtagtacgtatattgagcaat : 149799
Seq4 : ataattgatgggaaatgcaatagtaatttttttatcataaaagttgtaaagtaaataataaaacaataaatattgaactagtagtacgtatattgagcaat : 149798
```

```
                *    149820          *    149840          *    149860          *    149880          *    149900
Seq1 : cagaaatgatgctggtacctcttatcacggtgaccgtagttgcgggaacaatattagtatgttatatattatatatttgtaggaaaaagatacgtactgt : 149900
Seq2 : cagaaatgatgctggtacctcttatcacggtgaccgtagttgcgggaacaatattagtatgttatatattatatatttgtaggaaaaagatacgtactgt : 149898
Seq3 : cagaaatgatgctggtacctcttatcacggtgaccgtagttgcgggaacaatattagtatgttatatattatatatttgtaggaaaaagatacgtactgt : 149899
Seq4 : cagaaatgatgctggtacctcttatcacggtgaccgtagttgcgggaacaatattagtatgttatatattatatatttgtaggaaaaagatacgtactgt : 149898

                *    149920          *    149940          *    149960          *    149980          *    150000
Seq1 : ctataatgacaataaaattatcatgacaaaattaaaaaagataaagagttctaattccagcaaatctagtaaatcaactgatagcgaatcagactgggag : 150000
Seq2 : ctataatgacaataaaattatcatgacaaaattaaaaaagataaagagttctaattccagcaaatctagtaaatcaactgatagcgaatcagactgggag : 149998
Seq3 : ctataatgacaataaaattatcatgacaaaattaaaaaagataaagagttctaattccagcaaatctagtaaatcaactgatagcgaatcagactgggag : 149999
Seq4 : ctataatgacaataaaattatcatgacaaaattaaaaaagataaagagttctaattccagcaaatctagtaaatcaactgatagcgaatcagactgggag : 149998

                *    150020          *    150040          *    150060          *    150080          *    150100
Seq1 : gatcactgtagtgctatggaacaaaacaatgacgtagataatatttctaggaatgagatattggacgatgatagcttcgctggtagtttaatatgggata : 150100
Seq2 : gatcactgtagtgctatggaacaaaacaatgacgtagataatatttctaggaatgagatattggacgatgatagcttcgctggtagtttaatatgggata : 150098
Seq3 : gatcactgtagtgctatggaacaaaacaatgacgtagataatatttctaggaatgagatattggacgatgatagcttcgctggtagtttaatatgggata : 150099
Seq4 : gatcactgtagtgctatggaacaaaacaatgacgtagataatatttctaggaatgagatattggacgatgatagcttcgctggtagtttaatatgggata : 150098

                *    150120          *    150140          *    150160          *    150180          *    150200
Seq1 : acgaatccaatgtcatggcgcctagcacagaacacatttacgatagtgttgctggaagcacgctgctaataaataatgatcgtaatgaacagactattta : 150200
Seq2 : acgaatccaatgtcatggcgcctagcacagaacacatttacgatagtgttgctggaagcacgctgctaataaataatgatcgtaatgaacagactattta : 150198
Seq3 : acgaatccaatgtcatggcgcctagcacagaacacatttacgatagtgttgctggaagcacgctgctaataaataatgatcgtaatgaacagactattta : 150199
Seq4 : acgaatccaatgtcatggcgcctagcacagaacacatttacgatagtgttgctggaagcacgctgctaataaataatgatcgtaatgaacagactattta : 150198

                *    150220          *    150240          *    150260          *    150280          *    150300
Seq1 : tcagaacactacagtagtaattaatgagacggagactgttgaagtacttaatgaagataccaaacagaatcctaactattcatccaatcctttcgtaaat : 150300
Seq2 : tcagaacactacagtagtaattaatgagacggagactgttgaagtacttaatgaagataccaaacagaatcctaactattcatccaatcctttcgtaaat : 150298
Seq3 : tcagaacactacagtagtaattaatgagacggagactgttgaagtacttaatgaagataccaaacagaatcctaactattcatccaatcctttcgtaaat : 150299
Seq4 : tcagaacactacagtagtaattaatgagacggagactgttgaagtacttaatgaagataccaaacagaatcctaactattcatccaatcctttcgtaaat : 150298

                *    150320          *    150340          *    150360          *    150380          *    150400
Seq1 : tataataaaaccagtatttgtagcaagtcaaatccgttcattacagaactcaacaataaatttagtgagaataatccgtttagacgagcacatagcgatg : 150400
Seq2 : tataataaaaccagtatttgtagcaagtcaaatccgttcattacagaactcaacaataaatttagtgagaataatccgtttagacgagcacatagcgatg : 150398
Seq3 : tataataaaaccagtatttgtagcaagtcaaatccgttcattacagaactcaacaataaatttagtgagaataatccgtttagacgagcacatagcgatg : 150399
Seq4 : tataataaaaccagtatttgtagcaagtcaaatccgttcattacagaactcaacaataaatttagtgagaataatccgtttagacgagcacatagcgatg : 150398

                *    150420          *    150440          *    150460          *    150480          *    150500
Seq1 : attatcttaataagcaagaacaagatcatgaacacgatgatatagaatcatcggtcgtatcattggtgtgattagtttccttttataaaattgaagtaa : 150500
Seq2 : attatcttaataagcaagaacaagatcatgaacacgatgatatagaatcatcggtcgtatcattggtgtgattagtttccttttataaaattgaagtaa : 150498
Seq3 : attatcttaataagcaagaacaagatcatgaacacgatgatatagaatcatcggtcgtatcattggtgtgattagtttccttttataaaattgaagtaa : 150499
Seq4 : attatcttaataagcaagaacaagatcatgaacacgatgatatagaatcatcggtcgtatcattggtgtgattagtttccttttataaaattgaagtaa : 150498
```

```
                 *    150520         *    150540         *    150560         *    150580         *    150600
Seq1 : tatttagtattattgctgccgtcacgttgtacaaatggagatattccctgtattcggcatttctaaaattagcaattttattgctaataatgactgtaga : 150600
Seq2 : tatttagtattattgctgccgtcacgttgtacaaatggagatattccctgtattcggcatttctaaaattagcaattttattgctaataatgactgtaga : 150598
Seq3 : tatttagtattattgctgccgtcacgttgtacaaatggagatattccctgtattcggcatttctaaaattagcaattttattgctaataatgactgtaga : 150599
Seq4 : tatttagtattattgctgccgtcacgttgtacaaatggagatattccctgtattcggcatttctaaaattagcaattttattgctaataatgactgtaga : 150598

                 *    150620         *    150640         *    150660         *    150680         *    150700
Seq1 : tattatatagatacagaacatcaaaaaattatatctgatgagatcaatagacagatggatgaaacggtacttcttaccaacatcttaagcgtagaagttg : 150700
Seq2 : tattatatagatacagaacatcaaaaaattatatctgatgagatcaatagacagatggatgaaacggtacttcttaccaacatcttaagcgtagaagttg : 150698
Seq3 : tattatatagatacagaacatcaaaaaattatatctgatgagatcaatagacagatggatgaaacggtacttcttaccaacatcttaagcgtagaagttg : 150699
Seq4 : tattatatagatacagaacatcaaaaaattatatctgatgagatcaatagacagatggatgaaacggtacttcttaccaacatcttaagcgtagaagttg : 150698

                 *    150720         *    150740         *    150760         *    150780         *    150800
Seq1 : taaatgacaatgagatgtaccatcttattccccatagactatcgactattatactctgtattagttctgtcggaggatgtgttatctctatagataatga : 150800
Seq2 : taaatgacaatgagatgtaccatcttattccccatagactatcgactattatactctgtattagttctgtcggaggatgtgttatctctatagataatga : 150798
Seq3 : taaatgacaatgagatgtaccatcttattccccatagactatcgactattatactctgtattagttctgtcggaggatgtgttatctctatagataatga : 150799
Seq4 : taaatgacaatgagatgtaccatcttattccccatagactatcgactattatactctgtattagttctgtcggaggatgtgttatctctatagataatga : 150798

                 *    150820         *    150840         *    150860         *    150880         *    150900
Seq1 : catcaatgacaaaaatattctaacatttcccattgatcatgctgtaatcatatccccactgagtaaatgtgtcgtagttagcaagggtcctacaaccata : 150900
Seq2 : catcaatgacaaaaatattctaacatttcccattgatcatgctgtaatcatatccccactgagtaaatgtgtcgtagttagcaagggtcctacaaccata : 150898
Seq3 : catcaatgacaaaaatattctaacatttcccattgatcatgctgtaatcatatccccactgagtaaatgtgtcgtagttagcaagggtcctacaaccata : 150899
Seq4 : catcaatgacaaaaatattctaacatttcccattgatcatgctgtaatcatatccccactgagtaaatgtgtcgtagttagcaagggtcctacaaccata : 150898

                 *    150920         *    150940         *    150960         *    150980         *    151000
Seq1 : ttggttgttaaagcggatatacccagcaaacgattggtaacatcgtttacaaacgacatactgtatgtaaacaatctgtcactgattaattatttgccgt : 151000
Seq2 : ttggttgttaaagcggatatacccagcaaacgattggtaacatcgtttacaaacgacatactgtatgtaaacaatctgtcactgattaattatttgccgt : 150998
Seq3 : ttggttgttaaagcggatatacccagcaaacgattggtaacatcgtttacaaacgacatactgtatgtaaacaatctgtcactgattaattatttgccgt : 150999
Seq4 : ttggttgttaaagcggatatacccagcaaacgattggtaacatcgtttacaaacgacatactgtatgtaaacaatctgtcactgattaattatttgccgt : 150998

                 *    151020         *    151040         *    151060         *    151080         *    151100
Seq1 : tgtctgtattcattattagacgagtcaccgactatttggatagacacatatgcgatcagatatttgcgaataataagtggtattccattataaccatcga : 151100
Seq2 : tgtctgtattcattattagacgagtcaccgactatttggatagacacatatgcgatcagatatttgcgaataataagtggtattccattataaccatcga : 151098
Seq3 : tgtctgtattcattattagacgagtcaccgactatttggatagacacatatgcgatcagatatttgcgaataataagtggtattccattataaccatcga : 151099
Seq4 : tgtctgtattcattattagacgagtcaccgactatttggatagacacatatgcgatcagatatttgcgaataataagtggtattccattataaccatcga : 151098

                 *    151120         *    151140         *    151160         *    151180         *    151200
Seq1 : cgataagcaatatcctattccatcaaactgtataggtatgtcctctgccaagtacataaattctagcatcgagcaagatactttaatacatgtttgtaac : 151200
Seq2 : cgataagcaatatcctattccatcaaactgtataggtatgtcctctgccaagtacataaattctagcatcgagcaagatactttaatacatgtttgtaac : 151198
Seq3 : cgataagcaatatcctattccatcaaactgtataggtatgtcctctgccaagtacataaattctagcatcgagcaagatactttaatacatgtttgtaac : 151199
Seq4 : cgataagcaatatcctattccatcaaactgtataggtatgtcctctgccaagtacataaattctagcatcgagcaagatactttaatacatgtttgtaac : 151198
```

```
                 *    151220         *    151240         *    151260         *    151280         *    151300
Seq1 : ctcgagcatccattcgacttagtatacaaaaaaatgcagtcgtacaattctgtacctatcaaggaacaaatattgtacggtagaattgataatataaata : 151300
Seq2 : ctcgagcatccattcgacttagtatacaaaaaaatgcagtcgtacaattctgtacctatcaaggaacaaatattgtacggtagaattgataatataaata : 151298
Seq3 : ctcgagcatccattcgacttagtatacaaaaaaatgcagtcgtacaattctgtacctatcaaggaacaaatattgtacggtagaattgataatataaata : 151299
Seq4 : ctcgagcatccattcgacttagtatacaaaaaaatgcagtcgtacaattctgtacctatcaaggaacaaatattgtacggtagaattgataatataaata : 151298

                 *    151320         *    151340         *    151360         *    151380         *    151400
Seq1 : tgagcattagtatttctgtggattaatagatttctagtatggggatcattaatcatctctaatctctaaatacctcataaaacgaaaaaaaagctattat : 151400
Seq2 : tgagcattagtatttctgtggattaatagatttctagtatggggatcattaatcatctctaatctctaaatacctcataaaacgaaaaaaaagctattat : 151398
Seq3 : tgagcattagtatttctgtggattaatagatttctagtatggggatcattaatcatctctaatctctaaatacctcataaaacgaaaaaaaagctattat : 151399
Seq4 : tgagcattagtatttctgtggattaatagatttctagtatggggatcattaatcatctctaatctctaaatacctcataaaacgaaaaaaaagctattat : 151398

                 *    151420         *    151440         *    151460         *    151480         *    151500
Seq1 : caaatactgtacggaatggattcattctcttctctttttatgaaactctgttgtatatctactgataaaactggaagcaaaaaatctgataaaaagaata : 151500
Seq2 : caaatactgtacggaatggattcattctcttctctttttatgaaactctgttgtatatctactgataaaactggaagcaaaaaatctgataaaaagaata : 151498
Seq3 : caaatactgtacggaatggattcattctcttctctttttatgaaactctgttgtatatctactgataaaactggaagcaaaaaatctgataaaaagaata : 151499
Seq4 : caaatactgtacggaatggattcattctcttctctttttatgaaactctgttgtatatctactgataaaactggaagcaaaaaatctgataaaaagaata : 151498

                 *    151520         *    151540         *    151560         *    151580         *    151600
Seq1 : agaataagatcaaggattatatggaacacgattattataaaataacaatagttcctggttcctcttccacgtctactagctcgtggtattatacacatgc : 151600
Seq2 : agaataagatcaaggattatatggaacacgattattataaaataacaatagttcctggttcctcttccacgtctactagctcgtggtattatacacatgc : 151598
Seq3 : agaataagatcaaggattatatggaacacgattattataaaataacaatagttcctggttcctcttccacgtctactagctcgtggtattatacacatgc : 151599
Seq4 : agaataagatcaaggattatatggaacacgattattataaaataacaatagttcctggttcctcttccacgtctactagctcgtggtattatacacatgc : 151598

                 *    151620         *    151640         *    151660         *    151680         *    151700
Seq1 : ctagtaatagtctctttgcgttgacggaaagcagactagaaataacaggctaaaatgttcagacaccataatagttcccaacccagataataacagagta : 151700
Seq2 : ctagtaatagtctctttgcgttgacggaaagcagactagaaataacaggctaaaatgttcagacaccataatagttcccaacccagataataacagagta : 151698
Seq3 : ctagtaatagtctctttgcgttgacggaaagcagactagaaataacaggctaaaatgttcagacaccataatagttcccaacccagataataacagagta : 151699
Seq4 : ctagtaatagtctctttgcgttgacggaaagcagactagaaataacaggctaaaatgttcagacaccataatagttcccaacccagataataacagagta : 151698

                 *    151720         *    151740         *    151760         *    151780         *    151800
Seq1 : ccatcaacacattcctttaaactcaatcccaaacccaaaaccgttaaaatgtatccggccaattgatagtagataatgaggtgtacagcgcatgataatt : 151800
Seq2 : ccatcaacacattcctttaaactcaatcccaaacccaaaaccgttaaaatgtatccggccaattgatagtagataatgaggtgtacagcgcatgataatt : 151798
Seq3 : ccatcaacacattcctttaaactcaatcccaaacccaaaaccgttaaaatgtatccggccaattgatagtagataatgaggtgtacagcgcatgataatt : 151799
Seq4 : ccatcaacacattcctttaaactcaatcccaaacccaaaaccgttaaaatgtatccggccaattgatagtagataatgaggtgtacagcgcatgataatt : 151798

                 *    151820         *    151840         *    151860         *    151880         *    151900
Seq1 : tacacagtaaccaaaatgaaaatactttagtaattataagaaatatagatggtaacgtcatcatcaacaatccaataatatgccggagagtaaacattga : 151900
Seq2 : tacacagtaaccaaaatgaaaatactttagtaattataagaaatatagatggtaacgtcatcatcaacaatccaataatatgccggagagtaaacattga : 151898
Seq3 : tacacagtaaccaaaatgaaaatactttagtaattataagaaatatagatggtaacgtcatcatcaacaatccaataatatgccggagagtaaacattga : 151899
Seq4 : tacacagtaaccaaaatgaaaatactttagtaattataagaaatatagatggtaacgtcatcatcaacaatccaataatatgccggagagtaaacattga : 151898
```

```
             *    151920          *    151940          *    151960          *    151980          *    152000
Seq1 : cggataaaacaaaaatgctccgcataactctatcatggcaataacacaaccaaatacttgtaagattcctaaattagtagaaaatacaacggatatcgat : 152000
Seq2 : cggataaaacaaaaatgctccgcataactctatcatggcaataacacaaccaaatacttgtaagattcctaaattagtagaaaatacaacggatatcgat : 151998
Seq3 : cggataaaacaaaaatgctccgcataactctatcatggcaataacacaaccaaatacttgtaagattcctaaattagtagaaaatacaacggatatcgat : 151999
Seq4 : cggataaaacaaaaatgctccgcataactctatcatggcaataacacaaccaaatacttgtaagattcctaaattagtagaaaatacaacggatatcgat : 151998

             *    152020          *    152040          *    152060          *    152080          *    152100
Seq1 : gtataagtgatctcgagaaataataagaataaagtaatgcccgtaaagataaacatcaacattgtttggtaatcattaaaccaattagtatgaagttgaa : 152100
Seq2 : gtataagtgatctcgagaaataataagaataaagtaatgcccgtaaagataaacatcaacattgtttggtaatcattaaaccaattagtatgaagttgaa : 152098
Seq3 : gtataagtgatctcgagaaataataagaataaagtaatgcccgtaaagataaacatcaacattgtttggtaatcattaaaccaattagtatgaagttgaa : 152099
Seq4 : gtataagtgatctcgagaaataataagaataaagtaatgcccgtaaagataaacatcaacattgtttggtaatcattaaaccaattagtatgaagttgaa : 152098

             *    152120          *    152140          *    152160          *    152180          *    152200
Seq1 : ctaatttcacagtagattttattccagtgttatcctcgcatgtatacgtacctggtaagatatctttatattctataatcaatgagacatcactatccga : 152200
Seq2 : ctaatttcacagtagattttattccagtgttatcctcgcatgtatacgtacctggtaagatatctttatattctataatcaatgagacatcactatccga : 152198
Seq3 : ctaatttcacagtagattttattccagtgttatcctcgcatgtatacgtacctggtaagatatctttatattctataatcaatgagacatcactatccga : 152199
Seq4 : ctaatttcacagtagattttattccagtgttatcctcgcatgtatacgtacctggtaagatatctttatattctataatcaatgagacatcactatccga : 152198

             *    152220          *    152240          *    152260          *    152280          *    152300
Seq1 : taacgaatgaagtctagcactagtatgccatttacttaatatggtcgtcttggaagttttattataagttaaaatatcatggttgtccaatttccatcta : 152300
Seq2 : taacgaatgaagtctagcactagtatgccatttacttaatatggtcgtcttggaagttttattataagttaaaatatcatggttgtccaatttccatcta : 152298
Seq3 : taacgaatgaagtctagcactagtatgccatttacttaatatggtcgtcttggaagttttattataagttaaaatatcatggttgtccaatttccatcta : 152299
Seq4 : taacgaatgaagtctagcactagtatgccatttacttaatatggtcgtcttggaagttttattataagttaaaatatcatggttgtccaatttccatcta : 152298

             *    152320          *    152340          *    152360          *    152380          *    152400
Seq1 : atatactttgtcggattatctatagtacacggaataatgatggtattattacatgctgtatactctatagtctttgtagatgttataatcataaaagtac : 152400
Seq2 : atatactttgtcggattatctatagtacacggaataatgatggtattattacatgctgtatactctatagtctttgtagatgttataatcataaaagtac : 152398
Seq3 : atatactttgtcggattatctatagtacacggaataatgatggtattattacatgctgtatactctatagtctttgtagatgttataatcataaaagtac : 152399
Seq4 : atatactttgtcggattatctatagtacacggaataatgatggtattattacatgctgtatactctatagtctttgtagatgttataatcataaaagtac : 152398

             *    152420          *    152440          *    152460          *    152480          *    152500
Seq1 : agaggtatatcaacgatattctaactcttgacatttttttatttatttaaaatgatacctttgttatttattttattctattttgctaacggtatcgaatg : 152500
Seq2 : agaggtatatcaacgatattctaactcttgacatttttttatttatttaaaatgatacctttgttatttattttattctattttgctaacggtatcgaatg : 152498
Seq3 : agaggtatatcaacgatattctaactcttgacatttttttatttatttaaaatgatacctttgttatttattttattctattttgctaacggtatcgaatg : 152499
Seq4 : agaggtatatcaacgatattctaactcttgacatttttttatttatttaaaatgatacctttgttatttattttattctattttgctaacggtatcgaatg : 152498

             *    152520          *    152540          *    152560          *    152580          *    152600
Seq1 : gtataagtttgaaacgagtgaagaaataatttctacttacttattagacgacgtattatacacgggtgttaatggggcggtatacacattttcaaataat : 152600
Seq2 : gtataagtttgaaacgagtgaagaaataatttctacttacttattagacgacgtattatacacgggtgttaatggggcggtatacacattttcaaataat : 152598
Seq3 : gtataagtttgaaacgagtgaagaaataatttctacttacttattagacgacgtattatacacgggtgttaatggggcggtatacacattttcaaataat : 152599
Seq4 : gtataagtttgaaacgagtgaagaaataatttctacttacttattagacgacgtattatacacgggtgttaatggggcggtatacacattttcaaataat : 152598
```

```
              *    152620          *    152640          *    152660          *    152680          *    152700
Seq1 : aaactaaacaaaactggtttagctaatactaattatatcacaacatctataaaagtagaggatgcggataaggatacattagtatgcggaaccaataacg : 152700
Seq2 : aaactaaacaaaactggtttagctaatactaattatatcacaacatctataaaagtagaggatgcggataaggatacattagtatgcggaaccaataacg : 152698
Seq3 : aaactaaacaaaactggtttagctaatactaattatatcacaacatctataaaagtagaggatgcggataaggatacattagtatgcggaaccaataacg : 152699
Seq4 : aaactaaacaaaactggtttagctaatactaattatatcacaacatctataaaagtagaggatgcggataaggatacattagtatgcggaaccaataacg : 152698

              *    152720          *    152740          *    152760          *    152780          *    152800
Seq1 : gaaatcccaaatgttggaaaatagacggttcagacgacccaaaacatagaggtagaggatacgctccttatcaaaatagcaaagtaacgataatcagtca : 152800
Seq2 : gaaatcccaaatgttggaaaatagacggttcagacgacccaaaacatagaggtagaggatacgctccttatcaaaatagcaaagtaacgataatcagtca : 152798
Seq3 : gaaatcccaaatgttggaaaatagacggttcagacgacccaaaacatagaggtagaggatacgctccttatcaaaatagcaaagtaacgataatcagtca : 152799
Seq4 : gaaatcccaaatgttggaaaatagacggttcagacgacccaaaacatagaggtagaggatacgctccttatcaaaatagcaaagtaacgataatcagtca : 152798

              *    152820          *    152840          *    152860          *    152880          *    152900
Seq1 : caacggatgtgtactatctgacataaacatatcaaaagaaggaattaaacgatggagaagatttgacggaccatgtggttatgatttatacacggcggat : 152900
Seq2 : caacggatgtgtactatctgacataaacatatcaaaagaaggaattaaacgatggagaagatttgacggaccatgtggttatgatttatacacggcggat : 152898
Seq3 : caacggatgtgtactatctgacataaacatatcaaaagaaggaattaaacgatggagaagatttgacggaccatgtggttatgatttatacacggcggat : 152899
Seq4 : caacggatgtgtactatctgacataaacatatcaaaagaaggaattaaacgatggagaagatttgacggaccatgtggttatgatttatacacggcggat : 152898

              *    152920          *    152940          *    152960          *    152980          *    153000
Seq1 : aacgtaattccaaaagatggtttacgaggagcattcgtcgataaagatggtacttatgacaaagtttacattcttttcactgatactatcggctcaaaga : 153000
Seq2 : aacgtaattccaaaagatggtttacgaggagcattcgtcgataaagatggtacttatgacaaagtttacattcttttcactgatactatcggctcaaaga : 152998
Seq3 : aacgtaattccaaaagatggtttacgaggagcattcgtcgataaagatggtacttatgacaaagtttacattcttttcactgatactatcggctcaaaga : 152999
Seq4 : aacgtaattccaaaagatggtttacgaggagcattcgtcgataaagatggtacttatgacaaagtttacattcttttcactgatactatcggctcaaaga : 152998

              *    153020          *    153040          *    153060          *    153080          *    153100
Seq1 : gaattgtcaaaattccgtatatagcacaaatgtgcctaaacgacgaaggtggtccatcatcattgtctagtcatagatggtcgacgtttctcaaagtcga : 153100
Seq2 : gaattgtcaaaattccgtatatagcacaaatgtgcctaaacgacgaaggtggtccatcatcattgtctagtcatagatggtcgacgtttctcaaagtcga : 153098
Seq3 : gaattgtcaaaattccgtatatagcacaaatgtgcctaaacgacgaaggtggtccatcatcattgtctagtcatagatggtcgacgtttctcaaagtcga : 153099
Seq4 : gaattgtcaaaattccgtatatagcacaaatgtgcctaaacgacgaaggtggtccatcatcattgtctagtcatagatggtcgacgtttctcaaagtcga : 153098

              *    153120          *    153140          *    153160          *    153180          *    153200
Seq1 : attagaatgtgatatcgacggaagaagttatagacaaattattcattctagaactataaaaacagataatgatacgatactatatgtattcttcgatagt : 153200
Seq2 : attagaatgtgatatcgacggaagaagttatagacaaattattcattctagaactataaaaacagataatgatacgatactatatgtattcttcgatagt : 153198
Seq3 : attagaatgtgatatcgacggaagaagttatagacaaattattcattctagaactataaaaacagataatgatacgatactatatgtattcttcgatagt : 153199
Seq4 : attagaatgtgatatcgacggaagaagttatagacaaattattcattctagaactataaaaacagataatgatacgatactatatgtattcttcgatagt : 153198

              *    153220          *    153240          *    153260          *    153280          *    153300
Seq1 : ccgcattatgtacctattctatgaataccattaaacaatctttttctacgtcaaaattggaaggatatacaaagcaattgccgtctccagctcctggtat : 153300
Seq2 : ccgcattatgtacctattctatgaataccattaaacaatctttttctacgtcaaaattggaaggatatacaaagcaattgccgtctccagctcctggtat : 153298
Seq3 : ccgcattatgtacctattctatgaataccattaaacaatctttttctacgtcaaaattggaaggatatacaaagcaattgccgtctccagctcctggtat : 153299
Seq4 : ccgcattatgtacctattctatgaataccattaaacaatctttttctacgtcaaaattggaaggatatacaaagcaattgccgtctccagctcctggtat : 153298
```

```
             *    153320         *    153340         *    153360         *    153380         *    153400
Seq1 : atgtctaccagctggaaaagttgttccacataccacgtttgaagtcatagaaaaatataatgtactagatgatattataaagcctttatctaaccaacct : 153400
Seq2 : atgtctaccagctggaaaagttgttccacataccacgtttgaagtcatagaaaaatataatgtactagatgatattataaagcctttatctaaccaacct : 153398
Seq3 : atgtctaccagctggaaaagttgttccacataccacgtttgaagtcatagaaaaatataatgtactagatgatattataaagcctttatctaaccaacct : 153399
Seq4 : atgtctaccagctggaaaagttgttccacataccacgtttgaagtcatagaaaaatataatgtactagatgatattataaagcctttatctaaccaacct : 153398

             *    153420         *    153440         *    153460         *    153480         *    153500
Seq1 : atcttcgaaggaccgtctggtgttaaatggttcgatataaaggagaaggaaaatgaacatcgggaatatagaatatacttcataaaagaaaattctatat : 153500
Seq2 : atcttcgaaggaccgtctggtgttaaatggttcgatataaaggagaaggaaaatgaacatcgggaatatagaatatacttcataaaagaaaattctatat : 153498
Seq3 : atcttcgaaggaccgtctggtgttaaatggttcgatataaaggagaaggaaaatgaacatcgggaatatagaatatacttcataaaagaaaattctatat : 153499
Seq4 : atcttcgaaggaccgtctggtgttaaatggttcgatataaaggagaaggaaaatgaacatcgggaatatagaatatacttcataaaagaaaattctatat : 153498

             *    153520         *    153540         *    153560         *    153580         *    153600
Seq1 : attcgttcgatacaaaatctaaacaaactcgtagctcgcaagtcgatgcgcgactattttcagtaatggtaacttcgaaaccgttatttatagcagatat : 153600
Seq2 : attcgttcgatacaaaatctaaacaaactcgtagctcgcaagtcgatgcgcgactattttcagtaatggtaacttcgaaaccgttatttatagcagatat : 153598
Seq3 : attcgttcgatacaaaatctaaacaaactcgtagctcgcaagtcgatgcgcgactattttcagtaatggtaacttcgaaaccgttatttatagcagatat : 153599
Seq4 : attcgttcgatacaaaatctaaacaaactcgtagctcgcaagtcgatgcgcgactattttcagtaatggtaacttcgaaaccgttatttatagcagatat : 153598

             *    153620         *    153640         *    153660         *    153680         *    153700
Seq1 : agggataggagtaggaatgccacaaatgaaaaaaatacttaaaatgtaatcttaatcgagtacaccacacgacaatgaacaaacataagacagattatgc : 153700
Seq2 : agggataggagtaggaatgccacaaatgaaaaaaatacttaaaatgtaatcttaatcgagtacaccacacgacaatgaacaaacataagacagattatgc : 153698
Seq3 : agggataggagtaggaatgccacaaatgaaaaaaatacttaaaatgtaatcttaatcgagtacaccacacgacaatgaacaaacataagacagattatgc : 153699
Seq4 : agggataggagtaggaatgccacaaatgaaaaaaatacttaaaatgtaatcttaatcgagtacaccacacgacaatgaacaaacataagacagattatgc : 153698

             *    153720         *    153740         *    153760         *    153780         *    153800
Seq1 : tggttatgcttgctgcgtaatatgcggtctaattgttggaattatttttacagcgacactattaaaagttgtagaacgtaaattagttcatacaccatca : 153800
Seq2 : tggttatgcttgctgcgtaatatgcggtctaattgttggaattatttttacagcgacactattaaaagttgtagaacgtaaattagttcatacaccatca : 153798
Seq3 : tggttatgcttgctgcgtaatatgcggtctaattgttggaattatttttacagcgacactattaaaagttgtagaacgtaaattagttcatacaccatca : 153799
Seq4 : tggttatgcttgctgcgtaatatgcggtctaattgttggaattatttttacagcgacactattaaaagttgtagaacgtaaattagttcatacaccatca : 153798

             *    153820         *    153840         *    153860         *    153880         *    153900
Seq1 : atagataaaacgataaaagatgcatatattagagaagattgtcctactgactggataagctataataataaatgtatccatttatctactgatcgaaaaa : 153900
Seq2 : atagataaaacgataaaagatgcatatattagagaagattgtcctactgactggataagctataataataaatgtatccatttatctactgatcgaaaaa : 153898
Seq3 : atagataaaacgataaaagatgcatatattagagaagattgtcctactgactggataagctataataataaatgtatccatttatctactgatcgaaaaa : 153899
Seq4 : atagataaaacgataaaagatgcatatattagagaagattgtcctactgactggataagctataataataaatgtatccatttatctactgatcgaaaaa : 153898

             *    153920         *    153940         *    153960         *    153980         *    154000
Seq1 : cctgggaggaaggacgtaatgcatgcaaagctctaaatccaaattcggatctaattaagatagagactccaaacgagttaagttttttaagaagcattag : 154000
Seq2 : cctgggaggaaggacgtaatgcatgcaaagctctaaatccaaattcggatctaattaagatagagactccaaacgagttaagttttttaagaagcattag : 153998
Seq3 : cctgggaggaaggacgtaatgcatgcaaagctctaaatccaaattcggatctaattaagatagagactccaaacgagttaagttttttaagaagcattag : 153999
Seq4 : cctgggaggaaggacgtaatgcatgcaaagctctaaatccaaattcggatctaattaagatagagactccaaacgagttaagttttttaagaagcattag : 153998
```

```
                *    154020         *    154040         *    154060         *    154080         *    154100
Seq1 : acgcggatattgggtaggagaatccgaaatattaaaccagacaaccccatataattttatagctaagaatgccacgaagaatggaactaaaaaacggaaa : 154100
Seq2 : acgcggatattgggtaggagaatccgaaatattaaaccagacaaccccatataattttatagctaagaatgccacgaagaatggaactaaaaaacggaaa : 154098
Seq3 : acgcggatattgggtaggagaatccgaaatattaaaccagacaaccccatataattttatagctaagaatgccacgaagaatggaactaaaaaacggaaa : 154099
Seq4 : acgcggatattgggtaggagaatccgaaatattaaaccagacaaccccatataattttatagctaagaatgccacgaagaatggaactaaaaaacggaaa : 154098

                *    154120         *    154140         *    154160         *    154180         *    154200
Seq1 : tatatttgtagcacaacgaatactcccaaactgcattcgtgttacactatataacaattacactacatttttatcataccactacttcggttagatgttt : 154200
Seq2 : tatatttgtagcacaacgaatactcccaaactgcattcgtgttacactatataacaattacactacatttttatcataccactacttcggttagatgttt : 154198
Seq3 : tatatttgtagcacaacgaatactcccaaactgcattcgtgttacactatataacaattacactacatttttatcataccactacttcggttagatgttt : 154199
Seq4 : tatatttgtagcacaacgaatactcccaaactgcattcgtgttacactatataacaattacactacatttttatcataccactacttcggttagatgttt : 154198

                *    154220         *    154240         *    154260         *    154280         *    154300
Seq1 : tagaaaaaaataaatatcgccgtaccgttcttgtttttataaaaataacaattaacaattatcaaatttttttctttaatattttacgtggttgaccattc : 154300
Seq2 : tagaaaaaaataaatatcgccgtaccgttcttgtttttataaaaataacaattaacaattatcaaatttttttctttaatattttacgtggttgaccattc : 154298
Seq3 : tagaaaaaaataaatatcgccgtaccgttcttgtttttataaaaataacaattaacaattatcaaatttttttctttaatattttacgtggttgaccattc : 154299
Seq4 : tagaaaaaaataaatatcgccgtaccgttcttgtttttataaaaataacaattaacaattatcaaatttttttctttaatattttacgtggttgaccattc : 154298

                *    154320         *    154340         *    154360         *    154380         *    154400
Seq1 : ttggtggtaaaataatctcttagtgttggaatggaatgctgtttaatgtttccacactcatcgtatattttgacgtatgtagtcacatcgtttacgcaat : 154400
Seq2 : ttggtggtaaaataatctcttagtgttggaatggaatgctgtttaatgtttccacactcatcgtatattttgacgtatgtagtcacatcgtttacgcaat : 154398
Seq3 : ttggtggtaaaataatctcttagtgttggaatggaatgctgtttaatgtttccacactcatcgtatattttgacgtatgtagtcacatcgtttacgcaat : 154399
Seq4 : ttggtggtaaaataatctcttagtgttggaatggaatgctgtttaatgtttccacactcatcgtatattttgacgtatgtagtcacatcgtttacgcaat : 154398

                *    154420         *    154440         *    154460         *    154480         *    154500
Seq1 : agtcagactgtagttctatcatgcttcctacattagaaggaggaacagttttaaagtctcttggttttaatctattaccgttagttttcatgaaatcctt : 154500
Seq2 : agtcagactgtagttctatcatgcttcctacattagaaggaggaacagttttaaagtctcttggttttaatctattaccgttagttttcatgaaatcctt : 154498
Seq3 : agtcagactgtagttctatcatgcttcctacattagaaggaggaacagttttaaagtctcttggttttaatctattaccgttagttttcatgaaatcctt : 154499
Seq4 : agtcagactgtagttctatcatgcttcctacattagaaggaggaacagttttaaagtctcttggttttaatctattaccgttagttttcatgaaatcctt : 154498

                *    154520         *    154540         *    154560         *    154580         *    154600
Seq1 : tgttttatccacttcacattttaaataaatgtccactatacattcttctgttaattttactagatcgtcatgggtcatagaatttataggttccgtagtc : 154600
Seq2 : tgttttatccacttcacattttaaataaatgtccactatacattcttctgttaattttactagatcgtcatgggtcatagaatttataggttccgtagtc : 154598
Seq3 : tgttttatccacttcacattttaaataaatgtccactatacattcttctgttaattttactagatcgtcatgggtcatagaatttataggttccgtagtc : 154599
Seq4 : tgttttatccacttcacattttaaataaatgtccactatacattcttctgttaattttactagatcgtcatgggtcatagaatttataggttccgtagtc : 154598

                *    154620         *    154640         *    154660         *    154680         *    154700
Seq1 : catggatccaaactagcaaacttcgcgtatacggtatcgcgattagtgtatacaccaactgtatgaaaattaagaaaacagtttaatagatcaacagaaa : 154700
Seq2 : catggatccaaactagcaaacttcgcgtatacggtatcgcgattagtgtatacaccaactgtatgaaaattaagaaaacagtttaatagatcaacagaaa : 154698
Seq3 : catggatccaaactagcaaacttcgcgtatacggtatcgcgattagtgtatacaccaactgtatgaaaattaagaaaacagtttaatagatcaacagaaa : 154699
Seq4 : catggatccaaactagcaaacttcgcgtatacggtatcgcgattagtgtatacaccaactgtatgaaaattaagaaaacagtttaatagatcaacagaaa : 154698
```

```
             *    154720          *    154740          *    154760          *    154780          *    154800
Seq1 : tatttaatcctccgtttgatacagatgcaccatatttatggattttggattcacacgttgtttgtctgaggggttcgtctagcgttgcttctacataaac : 154800
Seq2 : tatttaatcctccgtttgatacagatgcaccatatttatggattttggattcacacgttgtttgtctgaggggttcgtctagcgttgcttctacataaac : 154798
Seq3 : tatttaatcctccgtttgatacagatgcaccatatttatggattttggattcacacgttgtttgtctgaggggttcgtctagcgttgcttctacataaac : 154799
Seq4 : tatttaatcctccgtttgatacagatgcaccatatttatggattttggattcacacgttgtttgtctgaggggttcgtctagcgttgcttctacataaac : 154798

             *    154820          *    154840          *    154860          *    154880          *    154900
Seq1 : ttctattcccatatattctttattgtcagaatcgcataccgatttatcatcatacactgtttgaaaactaaatggtatacacatcaaaataacaaatact : 154900
Seq2 : ttctattcccatatattctttattgtcagaatcgcataccgatttatcatcatacactgtttgaaaactaaatggtatacacatcaaaataacaaatact : 154898
Seq3 : ttctattcccatatattctttattgtcagaatcgcataccgatttatcatcatacactgtttgaaaactaaatggtatacacatcaaaataacaaatact : 154899
Seq4 : ttctattcccatatattctttattgtcagaatcgcataccgatttatcatcatacactgtttgaaaactaaatggtatacacatcaaaataacaaatact : 154898

             *    154920          *    154940          *    154960          *    154980          *    155000
Seq1 : aacgagtacattctgcaatattgttatcgtaattggaaaaatagtgttcgagtgagttggattatgtgagtattggattgtatatttatttatatttt : 155000
Seq2 : aacgagtacattctgcaatattgttatcgtaattggaaaaatagtgttcgagtgagttggattatgtgagtattggattgtatatttatttatatttt : 154998
Seq3 : aacgagtacattctgcaatattgttatcgtaattggaaaaatagtgttcgagtgagttggattatgtgagtattggattgtatatttatttatatttt : 154999
Seq4 : aacgagtacattctgcaatattgttatcgtaattggaaaaatagtgttcgagtgagttggattatgtgagtattggattgtatatttatttatatttt : 154998

             *    155020          *    155040          *    155060          *    155080          *    155100
Seq1 : gtaataagaataaaatgctaatgtcaagtttattccaatagatgtcttattaaaaacatatataataaataacaatggctgaatggcataaaattatcga : 155100
Seq2 : gtaataagaataaaatgctaatgtcaagtttattccaatagatgtcttattaaaaacatatataataaataacaatggctgaatggcataaaattatcga : 155098
Seq3 : gtaataagaataaaatgctaatgtcaagtttattccaatagatgtcttattaaaaacatatataataaataacaatggctgaatggcataaaattatcga : 155099
Seq4 : gtaataagaataaaatgctaatgtcaagtttattccaatagatgtcttattaaaaacatatataataaataacaatggctgaatggcataaaattatcga : 155098

             *    155120          *    155140          *    155160          *    155180          *    155200
Seq1 : ggatatctcaaaaaataataagttcgaggatgccgccatcgttgattacaagactacaaagaatgttctagctgctattcctaacagaacatttgccaag : 155200
Seq2 : ggatatctcaaaaaataataagttcgaggatgccgccatcgttgattacaagactacaaagaatgttctagctgctattcctaacagaacatttgccaag : 155198
Seq3 : ggatatctcaaaaaataataagttcgaggatgccgccatcgttgattacaagactacaaagaatgttctagctgctattcctaacagaacatttgccaag : 155199
Seq4 : ggatatctcaaaaaataataagttcgaggatgccgccatcgttgattacaagactacaaagaatgttctagctgctattcctaacagaacatttgccaag : 155198

             *    155220          *    155240          *    155260          *    155280          *    155300
Seq1 : attaatccgggtgaaattattcctctcatcactaatcgtaatattctaaaacctcttattggtcagaaatattgtattgtatatactaactctctaatgg : 155300
Seq2 : attaatccgggtgaaattattcctctcatcactaatcgtaatattctaaaacctcttattggtcagaaatattgtattgtatatactaactctctaatgg : 155298
Seq3 : attaatccgggtgaaattattcctctcatcactaatcgtaatattctaaaacctcttattggtcagaaatattgtattgtatatactaactctctaatgg : 155299
Seq4 : attaatccgggtgaaattattcctctcatcactaatcgtaatattctaaaacctcttattggtcagaaatattgtattgtatatactaactctctaatgg : 155298

             *    155320          *    155340          *    155360          *    155380          *    155400
Seq1 : atgagaacacgtatgctatggagttgcttactgggtacgcccctgtatctccgatcgttatagcgagaactcataccgcacttatatttttgatgggtaa : 155400
Seq2 : atgagaacacgtatgctatggagttgcttactgggtacgcccctgtatctccgatcgttatagcgagaactcataccgcacttatatttttgatgggtaa : 155398
Seq3 : atgagaacacgtatgctatggagttgcttactgggtacgcccctgtatctccgatcgttatagcgagaactcataccgcacttatatttttgatgggtaa : 155399
Seq4 : atgagaacacgtatgctatggagttgcttactgggtacgcccctgtatctccgatcgttatagcgagaactcataccgcacttatatttttgatgggtaa : 155398
```

```
          *    155420         *    155440         *    155460         *    155480         *    155500
Seq1 : gccaacaacatccagacgtgacgtgtatagaacgtgtagagatcacgctacccgtgtacgcgcaactggtaattaaaataaaaagtaatattcatatgta : 155500
Seq2 : gccaacaacatccagacgtgacgtgtatagaacgtgtagagatcacgctacccgtgtacgcgcaactggtaattaaaataaaaagtaatattcatatgta : 155498
Seq3 : gccaacaacatccagacgtgacgtgtatagaacgtgtagagatcacgctacccgtgtacgcgcaactggtaattaaaataaaaagtaatattcatatgta : 155499
Seq4 : gccaacaacatccagacgtgacgtgtatagaacgtgtagagatcacgctacccgtgtacgcgcaactggtaattaaaataaaaagtaatattcatatgta : 155498

          *    155520         *    155540         *    155560         *    155580         *    155600
Seq1 : gtgtcaattttaaatgatgatgatgaaatggataatatccatattgacgatgtcaataatgccggtattggcatacagctcatcgatttttagatttcat : 155600
Seq2 : gtgtcaattttaaatgatgatgatgaaatggataatatccatattgacgatgtcaataatgccggtattggcatacagctcatcgatttttagatttcat : 155598
Seq3 : gtgtcaattttaaatgatgatgatgaaatggataatatccatattgacgatgtcaataatgccggtattggcatacagctcatcgatttttagatttcat : 155599
Seq4 : gtgtcaattttaaatgatgatgatgaaatggataatatccatattgacgatgtcaataatgccggtattggcatacagctcatcgatttttagatttcat : 155598

          *    155620         *    155640         *    155660         *    155680         *    155700
Seq1 : tcagaggatgtggaattatgttatgggcatttgtattttgataggatctataatgtagtaaatataaaatataatccgcatattccatatagatataatt : 155700
Seq2 : tcagaggatgtggaattatgttatgggcatttgtattttgataggatctataatgtagtaaatataaaatataatccgcatattccatatagatataatt : 155698
Seq3 : tcagaggatgtggaattatgttatgggcatttgtattttgataggatctataatgtagtaaatataaaatataatccgcatattccatatagatataatt : 155699
Seq4 : tcagaggatgtggaattatgttatgggcatttgtattttgataggatctataatgtagtaaatataaaatataatccgcatattccatatagatataatt : 155698

          *    155720         *    155740         *    155760         *    155780         *    155800
Seq1 : ttattaatcgcacgttaaccgtagatgaactagacgataatgtcttttttacacatggttattttttaaaacacaaatatggttcacttaatcctagttt : 155800
Seq2 : ttattaatcgcacgttaaccgtagatgaactagacgataatgtcttttttacacatggttattttttaaaacacaaatatggttcacttaatcctagttt : 155798
Seq3 : ttattaatcgcacgttaaccgtagatgaactagacgataatgtcttttttacacatggttattttttaaaacacaaatatggttcacttaatcctagttt : 155799
Seq4 : ttattaatcgcacgttaaccgtagatgaactagacgataatgtcttttttacacatggttattttttaaaacacaaatatggttcacttaatcctagttt : 155798

          *    155820         *    155840         *    155860         *    155880         *    155900
Seq1 : gattgtctcattatcaggaaacttaaaatataatgatatacaatgctcagtaaatgtatcgtgtctcattaaaaatttggcaacgagtacatctactata : 155900
Seq2 : gattgtctcattatcaggaaacttaaaatataatgatatacaatgctcagtaaatgtatcgtgtctcattaaaaatttggcaacgagtacatctactata : 155898
Seq3 : gattgtctcattatcaggaaacttaaaatataatgatatacaatgctcagtaaatgtatcgtgtctcattaaaaatttggcaacgagtacatctactata : 155899
Seq4 : gattgtctcattatcaggaaacttaaaatataatgatatacaatgctcagtaaatgtatcgtgtctcattaaaaatttggcaacgagtacatctactata : 155898

          *    155920         *    155940         *    155960         *    155980         *    156000
Seq1 : ttaacatctaaacataagacttattctctacatcggtccacgtgtattactataataggatacgattctattatatggtataaagatataaatgacaagt : 156000
Seq2 : ttaacatctaaacataagacttattctctacatcggtccacgtgtattactataataggatacgattctattatatggtataaagatataaatgacaagt : 155998
Seq3 : ttaacatctaaacataagacttattctctacatcggtccacgtgtattactataataggatacgattctattatatggtataaagatataaatgacaagt : 155999
Seq4 : ttaacatctaaacataagacttattctctacatcggtccacgtgtattactataataggatacgattctattatatggtataaagatataaatgacaagt : 155998

          *    156020         *    156040         *    156060         *    156080         *    156100
Seq1 : ataatggcatctatgattttactgcaatatgtatgctaatagcgtctacattgatagtgaccatatacgtgtttaaaaaaataaaaatgaactcttaatt : 156100
Seq2 : ataatggcatctatgattttactgcaatatgtatgctaatagcgtctacattgatagtgaccatatacgtgtttaaaaaaataaaaatgaactcttaatt : 156098
Seq3 : ataatggcatctatgattttactgcaatatgtatgctaatagcgtctacattgatagtgaccatatacgtgtttaaaaaaataaaaatgaactcttaatt : 156099
Seq4 : ataatggcatctatgattttactgcaatatgtatgctaatagcgtctacattgatagtgaccatatacgtgtttaaaaaaataaaaatgaactcttaatt : 156098
```

```
              *    156120          *    156140          *    156160          *    156180          *    156200
Seq1 : atgctatgctattagaaatggataaaatcaaaattacggttgattcaaaaattggtaatgttgttaccatatcgtataacttggaaaagataactattga : 156200
Seq2 : atgctatgctattagaaatggataaaatcaaaattacggttgattcaaaaattggtaatgttgttaccatatcgtataacttggaaaagataactattga : 156198
Seq3 : atgctatgctattagaaatggataaaatcaaaattacggttgattcaaaaattggtaatgttgttaccatatcgtataacttggaaaagataactattga : 156199
Seq4 : atgctatgctattagaaatggataaaatcaaaattacggttgattcaaaaattggtaatgttgttaccatatcgtataacttggaaaagataactattga : 156198

              *    156220          *    156240          *    156260          *    156280          *    156300
Seq1 : tgtcacacctaaaaagaaaaaagaaaaggatgtattattagcgcaatcagttgctgtcgaagaggcaaaagatgtcaaggtagaagaaaaaaatattatc : 156300
Seq2 : tgtcacacctaaaaagaaaaaagaaaaggatgtattattagcgcaatcagttgctgtcgaagaggcaaaagatgtcaaggtagaagaaaaaaatattatc : 156298
Seq3 : tgtcacacctaaaaagaaaaaagaaaaggatgtattattagcgcaatcagttgctgtcgaagaggcaaaagatgtcaaggtagaagaaaaaaatattatc : 156299
Seq4 : tgtcacacctaaaaagaaaaaagaaaaggatgtattattagcgcaatcagttgctgtcgaagaggcaaaagatgtcaaggtagaagaaaaaaatattatc : 156298

              *    156320          *    156340          *    156360          *    156380          *    156400
Seq1 : gatattgaagatgacgatgatatggatgtagaaagcgcataatacgatctataaaaataagtatataaatacttttatttactgtactcttactgtgta : 156400
Seq2 : gatattgaagatgacgatgatatggatgtagaaagcgcataatacgatctataaaaataagtatataaatacttttatttactgtactcttactgtgta : 156398
Seq3 : gatattgaagatgacgatgatatggatgtagaaagcgcataatacgatctataaaaataagtatataaatacttttatttactgtactcttactgtgta : 156399
Seq4 : gatattgaagatgacgatgatatggatgtagaaagcgcataatacgatctataaaaataagtatataaatacttttatttactgtactcttactgtgta : 156398

              *    156420          *    156440          *    156460          *    156480          *    156500
Seq1 : gtggtgataccctactcgattattttttaaaaaaatacttattctgattcttctagccatttccgtgttcgttcgaatgccacatcgacgttaaagata : 156500
Seq2 : gtggtgataccctactcgattattttttaaaaaaatacttattctgattcttctagccatttccgtgttcgttcgaatgccacatcgacgttaaagata : 156498
Seq3 : gtggtgataccctactcgattattttttaaaaaaatacttattctgattcttctagccatttccgtgttcgttcgaatgccacatcgacgttaaagata : 156499
Seq4 : gtggtgataccctactcgattattttttaaaaaaatacttattctgattcttctagccatttccgtgttcgttcgaatgccacatcgacgttaaagata : 156498

              *    156520          *    156540          *    156560          *    156580          *    156600
Seq1 : ggggagtagttgaaatctagttctgcattgttggtacgcacctcaaatgtagtgttggatatcttcaacgtatagttgttgagtagtgatggttttctaa : 156600
Seq2 : ggggagtagttgaaatctagttctgcattgttggtacgcacctcaaatgtagtgttggatatcttcaacgtatagttgttgagtagtgatggttttctaa : 156598
Seq3 : ggggagtagttgaaatctagttctgcattgttggtacgcacctcaaatgtagtgttggatatcttcaacgtatagttgttgagtagtgatggttttctaa : 156599
Seq4 : ggggagtagttgaaatctagttctgcattgttggtacgcacctcaaatgtagtgttggatatcttcaacgtatagttgttgagtagtgatggttttctaa : 156598

              *    156620          *    156640          *    156660          *    156680          *    156700
Seq1 : atagaattctcttcatatcattcttgcacgcgtacatttttagcatccatcttggaattctagatccttgttctattcccaatggtttcatcaatagaag : 156700
Seq2 : atagaattctcttcatatcattcttgcacgcgtacatttttagcatccatcttggaattctagatccttgttctattcccaatggtttcatcaatagaag : 156698
Seq3 : atagaattctcttcatatcattcttgcacgcgtacatttttagcatccatcttggaattctagatccttgttctattcccaatggtttcatcaatagaag : 156699
Seq4 : atagaattctcttcatatcattcttgcacgcgtacatttttagcatccatcttggaattctagatccttgttctattcccaatggtttcatcaatagaag : 156698

              *    156720          *    156740          *    156760          *    156780          *    156800
Seq1 : attaaacatatcgtacgaacacgatggagagtaatcgtagcaaaagtaagcatttcctttaatctcagatcccggatactggatatattttgcagccaac : 156800
Seq2 : attaaacatatcgtacgaacacgatggagagtaatcgtagcaaaagtaagcatttcctttaatctcagatcccggatactggatatattttgcagccaac : 156798
Seq3 : attaaacatatcgtacgaacacgatggagagtaatcgtagcaaaagtaagcatttcctttaatctcagatcccggatactggatatattttgcagccaac : 156799
Seq4 : attaaacatatcgtacgaacacgatggagagtaatcgtagcaaaagtaagcatttcctttaatctcagatcccggatactggatatattttgcagccaac : 156798
```

```
              *    156820         *    156840         *    156860         *    156880         *    156900
Seq1 : acgtgcatccatgcaacatttcctacatatacccggctatgcaccgcgtcatcatcgactgtacgatacataatgttaccgtgttgcttacattgctcgt : 156900
Seq2 : acgtgcatccatgcaacatttcctacatatacccggctatgcaccgcgtcatcatcgactgtacgatacataatgttaccgtgttgcttacattgctcgt : 156898
Seq3 : acgtgcatccatgcaacatttcctacatatacccggctatgcaccgcgtcatcatcgactgtacgatacataatgttaccgtgttgcttacattgctcgt : 156899
Seq4 : acgtgcatccatgcaacatttcctacatatacccggctatgcaccgcgtcatcatcgactgtacgatacataatgttaccgtgttgcttacattgctcgt : 156898

              *    156920         *    156940         *    156960         *    156980         *    157000
Seq1 : aaaagactttcgtcaatttgtctccttctccgtaaattccagtgggtcttaggcaacaagtatacaattttgctccattcatgattacggaattattggc : 157000
Seq2 : aaaagactttcgtcaatttgtctccttctccgtaaattccagtgggtcttaggcaacaagtatacaattttgctccattcatgattacggaattattggc : 156998
Seq3 : aaaagactttcgtcaatttgtctccttctccgtaaattccagtgggtcttaggcaacaagtatacaattttgctccattcatgattacggaattattggc : 156999
Seq4 : aaaagactttcgtcaatttgtctccttctccgtaaattccagtgggtcttaggcaacaagtatacaattttgctccattcatgattacggaattattggc : 156998

              *    157020         *    157040         *    157060         *    157080         *    157100
Seq1 : tttcataaccagttgctcggccatacgtttactttttgcgtatacatgtcctggtgatatatcataaagggtatgctcatggccgatgaatggatcaccg : 157100
Seq2 : tttcataaccagttgctcggccatacgtttactttttgcgtatacatgtcctggtgatatatcataaagggtatgctcatggccgatgaatggatcaccg : 157098
Seq3 : tttcataaccagttgctcggccatacgtttactttttgcgtatacatgtcctggtgatatatcataaagggtatgctcatggccgatgaatggatcaccg : 157099
Seq4 : tttcataaccagttgctcggccatacgtttactttttgcgtatacatgtcctggtgatatatcataaagggtatgctcatggccgatgaatggatcaccg : 157098

              *    157120         *    157140         *    157160         *    157180         *    157200
Seq1 : tgtttatttggtcctattgcttccatgctactagtatagatcaaatacttgattcctaggtccacacaagctgccaatatagtctgtgttccataatagt : 157200
Seq2 : tgtttatttggtcctattgcttccatgctactagtatagatcaaatacttgattcctaggtccacacaagctgccaatatagtctgtgttccataatagt : 157198
Seq3 : tgtttatttggtcctattgcttccatgctactagtatagatcaaatacttgattcctaggtccacacaagctgccaatatagtctgtgttccataatagt : 157199
Seq4 : tgtttatttggtcctattgcttccatgctactagtatagatcaaatacttgattcctaggtccacacaagctgccaatatagtctgtgttccataatagt : 157198

              *    157220         *    157240         *    157260         *    157280         *    157300
Seq1 : ttactttcatgatttcattatcggtgtattttccaaatacatccactagagcagccgtatgaataatcagatttaccccatctagcgcttctctcacctt : 157300
Seq2 : ttactttcatgatttcattatcggtgtattttccaaatacatccactagagcagccgtatgaataatcagatttaccccatctagcgcttctctcacctt : 157298
Seq3 : ttactttcatgatttcattatcggtgtattttccaaatacatccactagagcagccgtatgaataatcagatttaccccatctagcgcttctctcacctt : 157299
Seq4 : ttactttcatgatttcattatcggtgtattttccaaatacatccactagagcagccgtatgaataatcagatttaccccatctagcgcttctctcacctt : 157298

              *    157320         *    157340         *    157360         *    157380         *    157400
Seq1 : atcaaagtcgtttatatcacattgtatatagtttataaccttaactttcgaggttattggttgtggatcttctacaatatctatgactctgatttcttga : 157400
Seq2 : atcaaagtcgtttatatcacattgtatatagtttataaccttaactttcgaggttattggttgtggatcttctacaatatctatgactctgatttcttga : 157398
Seq3 : atcaaagtcgtttatatcacattgtatatagtttataaccttaactttcgaggttattggttgtggatcttctacaatatctatgactctgatttcttga : 157399
Seq4 : atcaaagtcgtttatatcacattgtatatagtttataaccttaactttcgaggttattggttgtggatcttctacaatatctatgactctgatttcttga : 157398

              *    157420         *    157440         *    157460         *    157480         *    157500
Seq1 : acatcatctgcactaattaacagttttactatatacctgcctagaaatccggcaccaccagtaaccgcgtacacggccattgctgccactcataatatca : 157500
Seq2 : acatcatctgcactaattaacagttttactatatacctgcctagaaatccggcaccaccagtaaccgcgtacacggccattgctgccactcataatatca : 157498
Seq3 : acatcatctgcactaattaacagttttactatatacctgcctagaaatccggcaccaccagtaaccgcgtacacggccattgctgccactcataatatca : 157499
Seq4 : acatcatctgcactaattaacagttttactatatacctgcctagaaatccggcaccaccagtaaccgcgtacacggccattgctgccactcataatatca : 157498
```

```
                *    157520          *    157540          *    157560          *    157580          *    157600
Seq1 : gactacttattctattttactaaataatggctgtttgtataatagaccacgataatatcagaggagttatttactttgaaccagtccatggaaaagataa : 157600
Seq2 : gactacttattctattttactaaataatggctgtttgtataatagaccacgataatatcagaggagttatttactttgaaccagtccatggaaaagataa : 157598
Seq3 : gactacttattctattttactaaataatggctgtttgtataatagaccacgataatatcagaggagttatttactttgaaccagtccatggaaaagataa : 157599
Seq4 : gactacttattctattttactaaataatggctgtttgtataatagaccacgataatatcagaggagttatttactttgaaccagtccatggaaaagataa : 157598

                *    157620          *    157640          *    157660          *    157680          *    157700
Seq1 : agttttaggatcagttattggattaaaatccggaacgtatagtttgataattcatcgttacggagatattagtcaaggatgtgattccataggcagtcca : 157700
Seq2 : agttttaggatcagttattggattaaaatccggaacgtatagtttgataattcatcgttacggagatattagtcaaggatgtgattccataggcagtcca : 157698
Seq3 : agttttaggatcagttattggattaaaatccggaacgtatagtttgataattcatcgttacggagatattagtcaaggatgtgattccataggcagtcca : 157699
Seq4 : agttttaggatcagttattggattaaaatccggaacgtatagtttgataattcatcgttacggagatattagtcaaggatgtgattccataggcagtcca : 157698

                *    157720          *    157740          *    157760          *    157780          *    157800
Seq1 : gaaatatttatcggtaacatctttgtaaacagatatggtgtagcatatgtttatttagatacagatgtaaatatatctacaattattggaaaggcgttat : 157800
Seq2 : gaaatatttatcggtaacatctttgtaaacagatatggtgtagcatatgtttatttagatacagatgtaaatatatctacaattattggaaaggcgttat : 157798
Seq3 : gaaatatttatcggtaacatctttgtaaacagatatggtgtagcatatgtttatttagatacagatgtaaatatatctacaattattggaaaggcgttat : 157799
Seq4 : gaaatatttatcggtaacatctttgtaaacagatatggtgtagcatatgtttatttagatacagatgtaaatatatctacaattattggaaaggcgttat : 157798

                *    157820          *    157840          *    157860          *    157880          *    157900
Seq1 : ctatttcaaaaaatgatcagagattagcgtgtggagttattggtatttcttacataaatgaaaagataatacattttcttacaattaacgagaatggcgt : 157900
Seq2 : ctatttcaaaaaatgatcagagattagcgtgtggagttattggtatttcttacataaatgaaaagataatacattttcttacaattaacgagaatggcgt : 157898
Seq3 : ctatttcaaaaaatgatcagagattagcgtgtggagttattggtatttcttacataaatgaaaagataatacattttcttacaattaacgagaatggcgt : 157899
Seq4 : ctatttcaaaaaatgatcagagattagcgtgtggagttattggtatttcttacataaatgaaaagataatacattttcttacaattaacgagaatggcgt : 157898

                *    157920          *    157940          *    157960          *    157980          *    158000
Seq1 : ttgatatatcagttaatgcgtctaaaacaataaatgcattagtttacttttctactcagcaaaataaattagtcatacgtaatgaagttaatgatacaca : 158000
Seq2 : ttgatatatcagttaatgcgtctaaaacaataaatgcattagtttacttttctactcagcaaaataaattagtcatacgtaatgaagttaatgatacaca : 157998
Seq3 : ttgatatatcagttaatgcgtctaaaacaataaatgcattagtttacttttctactcagcaaaataaattagtcatacgtaatgaagttaatgatacaca : 157999
Seq4 : ttgatatatcagttaatgcgtctaaaacaataaatgcattagtttacttttctactcagcaaaataaattagtcatacgtaatgaagttaatgatacaca : 157998

                *    158020          *    158040          *    158060          *    158080          *    158100
Seq1 : ctacactgtcgaatttgatagggacaaagtagttgacacgtttatttcatataatagacataatgacaccatagagataagaggggtgcttccagaggaa : 158100
Seq2 : ctacactgtcgaatttgatagggacaaagtagttgacacgtttatttcatataatagacataatgacaccatagagataagaggggtgcttccagaggaa : 158098
Seq3 : ctacactgtcgaatttgatagggacaaagtagttgacacgtttatttcatataatagacataatgacaccatagagataagaggggtgcttccagaggaa : 158099
Seq4 : ctacactgtcgaatttgatagggacaaagtagttgacacgtttatttcatataatagacataatgacaccatagagataagaggggtgcttccagaggaa : 158098

                *    158120          *    158140          *    158160          *    158180          *    158200
Seq1 : actaatattggttgcgcggttaatacgccggttagtatgacttacttgtataataagtatagttttaaactgattttagcagaatatataagacacagaa : 158200
Seq2 : actaatattggttgcgcggttaatacgccggttagtatgacttacttgtataataagtatagttttaaactgattttagcagaatatataagacacagaa : 158198
Seq3 : actaatattggttgcgcggttaatacgccggttagtatgacttacttgtataataagtatagttttaaactgattttagcagaatatataagacacagaa : 158199
Seq4 : actaatattggttgcgcggttaatacgccggttagtatgacttacttgtataataagtatagttttaaactgattttagcagaatatataagacacagaa : 158198
```

```
                 *    158220         *    158240         *    158260         *    158280         *    158300
Seq1 : atactatatccggcaatatttattcggcattgatgacactagatgatttggctattaaacagtatggagacattgatctattatttaatgagaaacttaa : 158300
Seq2 : atactatatccggcaatatttattcggcattgatgacactagatgatttggctattaaacagtatggagacattgatctattatttaatgagaaacttaa : 158298
Seq3 : atactatatccggcaatatttattcggcattgatgacactagatgatttggctattaaacagtatggagacattgatctattatttaatgagaaacttaa : 158299
Seq4 : atactatatccggcaatatttattcggcattgatgacactagatgatttggctattaaacagtatggagacattgatctattatttaatgagaaacttaa : 158298

                 *    158320         *    158340         *    158360         *    158380         *    158400
Seq1 : agtagactccgattcgggactatttgactttgtcaactttgtaaaggatatgatatgttgtgattctagaatagtagtagctctatctagtctagtatct : 158400
Seq2 : agtagactccgattcgggactatttgactttgtcaactttgtaaaggatatgatatgttgtgattctagaatagtagtagctctatctagtctagtatct : 158398
Seq3 : agtagactccgattcgggactatttgactttgtcaactttgtaaaggatatgatatgttgtgattctagaatagtagtagctctatctagtctagtatct : 158399
Seq4 : agtagactccgattcgggactatttgactttgtcaactttgtaaaggatatgatatgttgtgattctagaatagtagtagctctatctagtctagtatct : 158398

                 *    158420         *    158440         *    158460         *    158480         *    158500
Seq1 : aaacattgggaattgacaaataaaaagtataggtgtatggcattagccgaacatatatctgatagtattccaatatctgagctatctagactacgataca : 158500
Seq2 : aaacattgggaattgacaaataaaaagtataggtgtatggcattagccgaacatatatctgatagtattccaatatctgagctatctagactacgataca : 158498
Seq3 : aaacattgggaattgacaaataaaaagtataggtgtatggcattagccgaacatatatctgatagtattccaatatctgagctatctagactacgataca : 158499
Seq4 : aaacattgggaattgacaaataaaaagtataggtgtatggcattagccgaacatatatctgatagtattccaatatctgagctatctagactacgataca : 158498

                 *    158520         *    158540         *    158560         *    158580         *    158600
Seq1 : atctatgtaagtatctacgcggacacactgagagcatagaggataaatttgattattttgaagacgatgattcgtctacatgttctgccgtaaccgacag : 158600
Seq2 : atctatgtaagtatctacgcggacacactgagagcatagaggataaatttgattattttgaagacgatgattcgtctacatgttctgccgtaaccgacag : 158598
Seq3 : atctatgtaagtatctacgcggacacactgagagcatagaggataaatttgattattttgaagacgatgattcgtctacatgttctgccgtaaccgacag : 158599
Seq4 : atctatgtaagtatctacgcggacacactgagagcatagaggataaatttgattattttgaagacgatgattcgtctacatgttctgccgtaaccgacag : 158598

                 *    158620         *    158640         *    158660         *    158680         *    158700
Seq1 : ggaaacggatgtataattttttttatagcgtgaaggatatgataaaaaatataattgttgtatttatcccattccaatcaccttatatgattctgtaaca : 158700
Seq2 : ggaaacggatgtataattttttttatagcgtgaaggatatgataaaaaatataattgttgtatttatcccattccaatcaccttatatgattctgtaaca : 158698
Seq3 : ggaaacggatgtataattttttttatagcgtgaaggatatgataaaaaatataattgttgtatttatcccattccaatcaccttatatgattctgtaaca : 158699
Seq4 : ggaaacggatgtataattttttttatagcgtgaaggatatgataaaaaatataattgttgtatttatcccattccaatcaccttatatgattctgtaaca : 158698

                 *    158720         *    158740         *    158760         *    158780         *    158800
Seq1 : caatgaaggagtctcatagatgtatagaggtcagatactggtttgataaactgtttattccacatgagtatgtttgactttatggttagacccgcatact : 158800
Seq2 : caatgaaggagtctcatagatgtatagaggtcagatactggtttgataaactgtttattccacatgagtatgtttgactttatggttagacccgcatact : 158798
Seq3 : caatgaaggagtctcatagatgtatagaggtcagatactggtttgataaactgtttattccacatgagtatgtttgactttatggttagacccgcatact : 158799
Seq4 : caatgaaggagtctcatagatgtatagaggtcagatactggtttgataaactgtttattccacatgagtatgtttgactttatggttagacccgcatact : 158798

                 *    158820         *    158840         *    158860         *    158880         *    158900
Seq1 : ttaacaaatcactgaaaattggagttaggtattgacctctcagaatcagttgccgttctggaacattaaatgtatttttttatgatatactccaacgcatt : 158900
Seq2 : ttaacaaatcactgaaaattggagttaggtattgacctctcagaatcagttgccgttctggaacattaaatgtatttttttatgatatactccaacgcatt : 158898
Seq3 : ttaacaaatcactgaaaattggagttaggtattgacctctcagaatcagttgccgttctggaacattaaatgtatttttttatgatatactccaacgcatt : 158899
Seq4 : ttaacaaatcactgaaaattggagttaggtattgacctctcagaatcagttgccgttctggaacattaaatgtatttttttatgatatactccaacgcatt : 158898
```

```
            *    158920         *    158940         *    158960         *    158980         *    159000
Seq1 : tatgtgggcatacaacaagtcattactaatggagtattccaagagttttagttgtctagtatttaacaagagaagagatttcaacagactgtttatgaac : 159000
Seq2 : tatgtgggcatacaacaagtcattactaatggagtattccaagagttttagttgtctagtatttaacaagagaagagatttcaacagactgtttatgaac : 158998
Seq3 : tatgtgggcatacaacaagtcattactaatggagtattccaagagttttagttgtctagtatttaacaagagaagagatttcaacagactgtttatgaac : 158999
Seq4 : tatgtgggcatacaacaagtcattactaatggagtattccaagagttttagttgtctagtatttaacaagagaagagatttcaacagactgtttatgaac : 158998

            *    159020         *    159040         *    159060         *    159080         *    159100
Seq1 : tcgaatgccgcctcattgtcgcttatattgatgatgtcgaattctcccaatatcatcaccgatgagtagctcatcttgttatcgggatccaagttttcta : 159100
Seq2 : tcgaatgccgcctcattgtcgcttatattgatgatgtcgaattctcccaatatcatcaccgatgagtagctcatcttgttatcgggatccaagttttcta : 159098
Seq3 : tcgaatgccgcctcattgtcgcttatattgatgatgtcgaattctcccaatatcatcaccgatgagtagctcatcttgttatcgggatccaagttttcta : 159099
Seq4 : tcgaatgccgcctcattgtcgcttatattgatgatgtcgaattctcccaatatcatcaccgatgagtagctcatcttgttatcgggatccaagttttcta : 159098

            *    159120         *    159140         *    159160         *    159180         *    159200
Seq1 : aagatgtcattaaaccctcgatcatgaatggatttatcatcatcgtttttatgttggacatgagcttagtccgtttgtccacatctatagacgacgattt : 159200
Seq2 : aagatgtcattaaaccctcgatcatgaatggatttatcatcatcgtttttatgttggacatgagcttagtccgtttgtccacatctatagacgacgattt : 159198
Seq3 : aagatgtcattaaaccctcgatcatgaatggatttatcatcatcgtttttatgttggacatgagcttagtccgtttgtccacatctatagacgacgattt : 159199
Seq4 : aagatgtcattaaaccctcgatcatgaatggatttatcatcatcgtttttatgttggacatgagcttagtccgtttgtccacatctatagacgacgattt : 159198

            *    159220         *    159240         *    159260         *    159280         *    159300
Seq1 : ctgaattattttatatatccctctctttaactccaggaacttgtcaggatggtctactttaatatgttctcgtctaagagatgaaaatctttggatggtt : 159300
Seq2 : ctgaattattttatatatccctctctttaactccaggaacttgtcaggatggtctactttaatatgttctcgtctaagagatgaaaatctttggatggtt : 159298
Seq3 : ctgaattattttatatatccctctctttaactccaggaacttgtcaggatggtctactttaatatgttctcgtctaagagatgaaaatctttggatggtt : 159299
Seq4 : ctgaattattttatatatccctctctttaactccaggaacttgtcaggatggtctactttaatatgttctcgtctaagagatgaaaatctttggatggtt : 159298

            *    159320         *    159340         *    159360         *    159380         *    159400
Seq1 : gcacgcgactttctctaaaggatgacgttgcccaagatcctctcttaaatgaatccatcttatccttggacaagatggacagtctattttccttagatg : 159400
Seq2 : gcacgcgactttctctaaaggatgacgttgcccaagatcctctcttaaatgaatccatcttatccttggacaagatggacagtctattttccttagatg : 159398
Seq3 : gcacgcgactttctctaaaggatgacgttgcccaagatcctctcttaaatgaatccatcttatccttggacaagatggacagtctattttccttagatg : 159399
Seq4 : gcacgcgactttctctaaaggatgacgttgcccaagatcctctcttaaatgaatccatcttatccttggacaagatggacagtctattttccttagatg : 159398

            *    159420         *    159440         *    159460         *    159480         *    159500
Seq1 : gtttaatatttttgttacccatgatctataaaggtagacctaatcgtctcggatgacctatatatttattttcagttttattatacgcataaattgtaaa : 159500
Seq2 : gtttaatatttttgttacccatgatctataaaggtagacctaatcgtctcggatgacctatatatttattttcagttttattatacgcataaattgtaaa : 159498
Seq3 : gtttaatatttttgttacccatgatctataaaggtagacctaatcgtctcggatgacctatatatttattttcagttttattatacgcataaattgtaaa : 159499
Seq4 : gtttaatatttttgttacccatgatctataaaggtagacctaatcgtctcggatgacctatatatttattttcagttttattatacgcataaattgtaaa : 159498

            *    159520         *    159540         *    159560         *    159580         *    159600
Seq1 : aaatatgttaggtttacaaaaatgtctcgtggggcattaatcgtttttgaaggattggacaaatctggaaaaacaacacaatgtatgaacatcatggaat : 159600
Seq2 : aaatatgttaggtttacaaaaatgtctcgtggggcattaatcgtttttgaaggattggacaaatctggaaaaacaacacaatgtatgaacatcatggaat : 159598
Seq3 : aaatatgttaggtttacaaaaatgtctcgtggggcattaatcgtttttgaaggattggacaaatctggaaaaacaacacaatgtatgaacatcatggaat : 159599
Seq4 : aaatatgttaggtttacaaaaatgtctcgtggggcattaatcgtttttgaaggattggacaaatctggaaaaacaacacaatgtatgaacatcatggaat : 159598
```

```
                 *    159620          *    159640          *    159660          *    159680          *    159700
Seq1 : ctataccggcaaacacgataaaatatcttaactttcctcagagatccactgtcactggaaagatgatagatgactatctaactcgtaaaaaaacctataa : 159700
Seq2 : ctataccggcaaacacgataaaatatcttaactttcctcagagatccactgtcactggaaagatgatagatgactatctaactcgtaaaaaaacctataa : 159698
Seq3 : ctataccggcaaacacgataaaatatcttaactttcctcagagatccactgtcactggaaagatgatagatgactatctaactcgtaaaaaaacctataa : 159699
Seq4 : ctataccggcaaacacgataaaatatcttaactttcctcagagatccactgtcactggaaagatgatagatgactatctaactcgtaaaaaaacctataa : 159698

                 *    159720          *    159740          *    159760          *    159780          *    159800
Seq1 : tgatcatatagttaatctattattttgtgcaaatagatgggagtttgcatcttttatacaagaacaactagaacagggaattactttaatagttgataga : 159800
Seq2 : tgatcatatagttaatctattattttgtgcaaatagatgggagtttgcatcttttatacaagaacaactagaacagggaattactttaatagttgataga : 159798
Seq3 : tgatcatatagttaatctattattttgtgcaaatagatgggagtttgcatcttttatacaagaacaactagaacagggaattactttaatagttgataga : 159799
Seq4 : tgatcatatagttaatctattattttgtgcaaatagatgggagtttgcatcttttatacaagaacaactagaacagggaattactttaatagttgataga : 159798

                 *    159820          *    159840          *    159860          *    159880          *    159900
Seq1 : tacgcgttctctggagtagcgtatgccgccgctaaaggcgcgtcaatgactctcagtaagagttatgaatctggattgcctaaacccgacttagttatat : 159900
Seq2 : tacgcgttctctggagtagcgtatgccgccgctaaaggcgcgtcaatgactctcagtaagagttatgaatctggattgcctaaacccgacttagttatat : 159898
Seq3 : tacgcgttctctggagtagcgtatgccgccgctaaaggcgcgtcaatgactctcagtaagagttatgaatctggattgcctaaacccgacttagttatat : 159899
Seq4 : tacgcgttctctggagtagcgtatgccgccgctaaaggcgcgtcaatgactctcagtaagagttatgaatctggattgcctaaacccgacttagttatat : 159898

                 *    159920          *    159940          *    159960          *    159980          *    160000
Seq1 : tcttggaatctggtagcaaagaaattaatagaaacgtcggcgaggaaatttatgaagatgttacattccaacaaaaggtattacaagaatataaaaaaat : 160000
Seq2 : tcttggaatctggtagcaaagaaattaatagaaacgtcggcgaggaaatttatgaagatgttacattccaacaaaaggtattacaagaatataaaaaaat : 159998
Seq3 : tcttggaatctggtagcaaagaaattaatagaaacgtcggcgaggaaatttatgaagatgttacattccaacaaaaggtattacaagaatataaaaaaat : 159999
Seq4 : tcttggaatctggtagcaaagaaattaatagaaacgtcggcgaggaaatttatgaagatgttacattccaacaaaaggtattacaagaatataaaaaaat : 159998

                 *    160020          *    160040          *    160060          *    160080          *    160100
Seq1 : gattgaagaaggagatattcattggcaaattatttcttctgaattcgaggaagatgtaaagaaggagttgattaagaatatagttatagaggctatacac : 160100
Seq2 : gattgaagaaggagatattcattggcaaattatttcttctgaattcgaggaagatgtaaagaaggagttgattaagaatatagttatagaggctatacac : 160098
Seq3 : gattgaagaaggagatattcattggcaaattatttcttctgaattcgaggaagatgtaaagaaggagttgattaagaatatagttatagaggctatacac : 160099
Seq4 : gattgaagaaggagatattcattggcaaattatttcttctgaattcgaggaagatgtaaagaaggagttgattaagaatatagttatagaggctatacac : 160098

                 *    160120          *    160140          *    160160          *    160180          *    160200
Seq1 : acggttactggaccagtggggcaactgtggatgtaatagtgaaattacattttttataaatagatgttagtacagtgttataaatggatgaagcatatta : 160200
Seq2 : acggttactggaccagtggggcaactgtggatgtaatagtgaaattacattttttataaatagatgttagtacagtgttataaatggatgaagcatatta : 160198
Seq3 : acggttactggaccagtggggcaactgtggatgtaatagtgaaattacattttttataaatagatgttagtacagtgttataaatggatgaagcatatta : 160199
Seq4 : acggttactggaccagtggggcaactgtggatgtaatagtgaaattacattttttataaatagatgttagtacagtgttataaatggatgaagcatatta : 160198

                 *    160220          *    160240          *    160260          *    160280          *    160300
Seq1 : ctctggcaacttggaatcagtactcggatacgtgtccgatatgcataccgaactcgcatcaatatctcaattagttattgccaagatagaaactatagat : 160300
Seq2 : ctctggcaacttggaatcagtactcggatacgtgtccgatatgcataccgaactcgcatcaatatctcaattagttattgccaagatagaaactatagat : 160298
Seq3 : ctctggcaacttggaatcagtactcggatacgtgtccgatatgcataccgaactcgcatcaatatctcaattagttattgccaagatagaaactatagat : 160299
Seq4 : ctctggcaacttggaatcagtactcggatacgtgtccgatatgcataccgaactcgcatcaatatctcaattagttattgccaagatagaaactatagat : 160298
```

```
              *    160320         *    160340         *    160360         *    160380         *    160400
Seq1 : aatgatatattaaacaaggacattgtaaattttatcatgtgtagatcaaacttggataatccatttatctctttcctagatactgtatatactattatag : 160400
Seq2 : aatgatatattaaacaaggacattgtaaattttatcatgtgtagatcaaacttggataatccatttatctctttcctagatactgtatatactattatag : 160398
Seq3 : aatgatatattaaacaaggacattgtaaattttatcatgtgtagatcaaacttggataatccatttatctctttcctagatactgtatatactattatag : 160399
Seq4 : aatgatatattaaacaaggacattgtaaattttatcatgtgtagatcaaacttggataatccatttatctctttcctagatactgtatatactattatag : 160398

              *    160420         *    160440         *    160460         *    160480         *    160500
Seq1 : atcaagagaactatcagaccgaattgattaattcattagacgacaatgaaattatcgattgtatagttaataagtttatgagcttttataaggataacct : 160500
Seq2 : atcaagagaactatcagaccgaattgattaattcattagacgacaatgaaattatcgattgtatagttaataagtttatgagcttttataaggataacct : 160498
Seq3 : atcaagagaactatcagaccgaattgattaattcattagacgacaatgaaattatcgattgtatagttaataagtttatgagcttttataaggataacct : 160499
Seq4 : atcaagagaactatcagaccgaattgattaattcattagacgacaatgaaattatcgattgtatagttaataagtttatgagcttttataaggataacct : 160498

              *    160520         *    160540         *    160560         *    160580         *    160600
Seq1 : agaaaatatagtagatgctatcattactctaaaatatataatgaataatccagattttaaaactacgtatgccgaagtactcggttccagaatagccgat : 160600
Seq2 : agaaaatatagtagatgctatcattactctaaaatatataatgaataatccagattttaaaactacgtatgccgaagtactcggttccagaatagccgat : 160598
Seq3 : agaaaatatagtagatgctatcattactctaaaatatataatgaataatccagattttaaaactacgtatgccgaagtactcggttccagaatagccgat : 160599
Seq4 : agaaaatatagtagatgctatcattactctaaaatatataatgaataatccagattttaaaactacgtatgccgaagtactcggttccagaatagccgat : 160598

              *    160620         *    160640         *    160660         *    160680         *    160700
Seq1 : atagatattaaacaagtgatacgtgagaatatactacaattgtctaatgatatccgcgaacgatatttgtgaaaaatattaaaaaaaatactttttttta : 160700
Seq2 : atagatattaaacaagtgatacgtgagaatatactacaattgtctaatgatatccgcgaacgatatttgtgaaaaatattaaaaaaaatactttttttta : 160698
Seq3 : atagatattaaacaagtgatacgtgagaatatactacaattgtctaatgatatccgcgaacgatatttgtgaaaaatattaaaaaaaatactttttttta : 160699
Seq4 : atagatattaaacaagtgatacgtgagaatatactacaattgtctaatgatatccgcgaacgatatttgtgaaaaatattaaaaaaaatactttttttta : 160698

              *    160720         *    160740         *    160760         *    160780         *    160800
Seq1 : ttaaatgacgtcgcttcgcgaatttagaaaattatgctgtgatatatatcacgcatcaggatataaagaaaaatctaaattaattagagactttataaca : 160800
Seq2 : ttaaatgacgtcgcttcgcgaatttagaaaattatgctgtgatatatatcacgcatcaggatataaagaaaaatctaaattaattagagactttataaca : 160798
Seq3 : ttaaatgacgtcgcttcgcgaatttagaaaattatgctgtgatatatatcacgcatcaggatataaagaaaaatctaaattaattagagactttataaca : 160799
Seq4 : ttaaatgacgtcgcttcgcgaatttagaaaattatgctgtgatatatatcacgcatcaggatataaagaaaaatctaaattaattagagactttataaca : 160798

              *    160820         *    160840         *    160860         *    160880         *    160900
Seq1 : gatagggatgataaatatttgatcattaagctattgcttcccggattagacgatagaatttataacatgaacgataaacaaattataaaattatatagta : 160900
Seq2 : gatagggatgataaatatttgatcattaagctattgcttcccggattagacgatagaatttataacatgaacgataaacaaattataaaattatatagta : 160898
Seq3 : gatagggatgataaatatttgatcattaagctattgcttcccggattagacgatagaatttataacatgaacgataaacaaattataaaattatatagta : 160899
Seq4 : gatagggatgataaatatttgatcattaagctattgcttcccggattagacgatagaatttataacatgaacgataaacaaattataaaattatatagta : 160898

              *    160920         *    160940         *    160960         *    160980         *    161000
Seq1 : taatatttaaacaatctcaggaagatatgctacaagatttaggatacggatatataggagacactattaggactttcttcaaagagaacacagaaatccg : 161000
Seq2 : taatatttaaacaatctcaggaagatatgctacaagatttaggatacggatatataggagacactattaggactttcttcaaagagaacacagaaatccg : 160998
Seq3 : taatatttaaacaatctcaggaagatatgctacaagatttaggatacggatatataggagacactattaggactttcttcaaagagaacacagaaatccg : 160999
Seq4 : taatatttaaacaatctcaggaagatatgctacaagatttaggatacggatatataggagacactattaggactttcttcaaagagaacacagaaatccg : 160998
```

```
                 *    161020         *    161040         *    161060         *    161080         *    161100
Seq1 : tccacgagataaaagcattttaactttagaagacgtggatagtttcttaactacgttatcatccgtaactaaagaatcgcatcaaataaaattattgact : 161100
Seq2 : tccacgagataaaagcattttaactttagaagacgtggatagtttcttaactacgttatcatccgtaactaaagaatcgcatcaaataaaattattgact : 161098
Seq3 : tccacgagataaaagcattttaactttagaagacgtggatagtttcttaactacgttatcatccgtaactaaagaatcgcatcaaataaaattattgact : 161099
Seq4 : tccacgagataaaagcattttaactttagaagacgtggatagtttcttaactacgttatcatccgtaactaaagaatcgcatcaaataaaattattgact : 161098

                 *    161120         *    161140         *    161160         *    161180         *    161200
Seq1 : gatatcgcatccgtttgtacatgtaatgatttaaaatgtgtagtcatgcttattgataaagatctaaaaattaaagcgggtcctcggtacgtacttaacg : 161200
Seq2 : gatatcgcatccgtttgtacatgtaatgatttaaaatgtgtagtcatgcttattgataaagatctaaaaattaaagcgggtcctcggtacgtacttaacg : 161198
Seq3 : gatatcgcatccgtttgtacatgtaatgatttaaaatgtgtagtcatgcttattgataaagatctaaaaattaaagcgggtcctcggtacgtacttaacg : 161199
Seq4 : gatatcgcatccgtttgtacatgtaatgatttaaaatgtgtagtcatgcttattgataaagatctaaaaattaaagcgggtcctcggtacgtacttaacg : 161198

                 *    161220         *    161240         *    161260         *    161280         *    161300
Seq1 : ctattagtcctaatgcctatgatgtgtttagaaaatctaataacttgaaagagataatagaaaatgcatctaaacaaaatctagactctatatctatttc : 161300
Seq2 : ctattagtcctaatgcctatgatgtgtttagaaaatctaataacttgaaagagataatagaaaatgcatctaaacaaaatctagactctatatctatttc : 161298
Seq3 : ctattagtcctaatgcctatgatgtgtttagaaaatctaataacttgaaagagataatagaaaatgcatctaaacaaaatctagactctatatctatttc : 161299
Seq4 : ctattagtcctaatgcctatgatgtgtttagaaaatctaataacttgaaagagataatagaaaatgcatctaaacaaaatctagactctatatctatttc : 161298

                 *    161320         *    161340         *    161360         *    161380         *    161400
Seq1 : tgttatgactccaattaatcccatgttagcggaatcgtgtgattctgtcaataaggcgtttaaaaaatttccatcaggaatgtttgcggaagtcaaatac : 161400
Seq2 : tgttatgactccaattaatcccatgttagcggaatcgtgtgattctgtcaataaggcgtttaaaaaatttccatcaggaatgtttgcggaagtcaaatac : 161398
Seq3 : tgttatgactccaattaatcccatgttagcggaatcgtgtgattctgtcaataaggcgtttaaaaaatttccatcaggaatgtttgcggaagtcaaatac : 161399
Seq4 : tgttatgactccaattaatcccatgttagcggaatcgtgtgattctgtcaataaggcgtttaaaaaatttccatcaggaatgtttgcggaagtcaaatac : 161398

                 *    161420         *    161440         *    161460         *    161480         *    161500
Seq1 : gatggtgaaagagtacaagttcataaaaataataacgagtttgccttctttagtagaaacatgaaaccagtactctctcataaagtggattatctcaaag : 161500
Seq2 : gatggtgaaagagtacaagttcataaaaataataacgagtttgccttctttagtagaaacatgaaaccagtactctctcataaagtggattatctcaaag : 161498
Seq3 : gatggtgaaagagtacaagttcataaaaataataacgagtttgccttctttagtagaaacatgaaaccagtactctctcataaagtggattatctcaaag : 161499
Seq4 : gatggtgaaagagtacaagttcataaaaataataacgagtttgccttctttagtagaaacatgaaaccagtactctctcataaagtggattatctcaaag : 161498

                 *    161520         *    161540         *    161560         *    161580         *    161600
Seq1 : aatacataccgaaagcatttaaaaaaagctacgtctatcgtattggattctgaaattgttcttgtagacgaacataatgtaccgctcccgtttggaagttt : 161600
Seq2 : aatacataccgaaagcatttaaaaaaagctacgtctatcgtattggattctgaaattgttcttgtagacgaacataatgtaccgctcccgtttggaagttt : 161598
Seq3 : aatacataccgaaagcatttaaaaaaagctacgtctatcgtattggattctgaaattgttcttgtagacgaacataatgtaccgctcccgtttggaagttt : 161599
Seq4 : aatacataccgaaagcatttaaaaaaagctacgtctatcgtattggattctgaaattgttcttgtagacgaacataatgtaccgctcccgtttggaagttt : 161598

                 *    161620         *    161640         *    161660         *    161680         *    161700
Seq1 : aggaatacacaaaaagaaagaatataaaaactctaacatgtgtttgttcgtgtttgactgtttgtactttgatggattcgatatgacggacattccattg : 161700
Seq2 : aggaatacacaaaaagaaagaatataaaaactctaacatgtgtttgttcgtgtttgactgtttgtactttgatggattcgatatgacggacattccattg : 161698
Seq3 : aggaatacacaaaaagaaagaatataaaaactctaacatgtgtttgttcgtgtttgactgtttgtactttgatggattcgatatgacggacattccattg : 161699
Seq4 : aggaatacacaaaaagaaagaatataaaaactctaacatgtgtttgttcgtgtttgactgtttgtactttgatggattcgatatgacggacattccattg : 161698
```

```
              *    161720          *    161740          *    161760          *    161780          *    161800
Seq1 : tacgaacgaagatcttttctcaaagatgttatggttgaaatacccaatagaatagtattctcagagttgacgaatattagtaacgagtctcagttaactg : 161800
Seq2 : tacgaacgaagatcttttctcaaagatgttatggttgaaatacccaatagaatagtattctcagagttgacgaatattagtaacgagtctcagttaactg : 161798
Seq3 : tacgaacgaagatcttttctcaaagatgttatggttgaaatacccaatagaatagtattctcagagttgacgaatattagtaacgagtctcagttaactg : 161799
Seq4 : tacgaacgaagatcttttctcaaagatgttatggttgaaatacccaatagaatagtattctcagagttgacgaatattagtaacgagtctcagttaactg : 161798

              *    161820          *    161840          *    161860          *    161880          *    161900
Seq1 : acgtattggatgatgcactaacgagaaaattagaaggattggtcttaaaagatattaatggagtatacgaaccgggaaagagaagatggttaaaaataaa : 161900
Seq2 : acgtattggatgatgcactaacgagaaaattagaaggattggtcttaaaagatattaatggagtatacgaaccgggaaagagaagatggttaaaaataaa : 161898
Seq3 : acgtattggatgatgcactaacgagaaaattagaaggattggtcttaaaagatattaatggagtatacgaaccgggaaagagaagatggttaaaaataaa : 161899
Seq4 : acgtattggatgatgcactaacgagaaaattagaaggattggtcttaaaagatattaatggagtatacgaaccgggaaagagaagatggttaaaaataaa : 161898

              *    161920          *    161940          *    161960          *    161980          *    162000
Seq1 : gcgagactatttgaacgagggttccatggcagattctgccgatttagtagtactaggtgcttactatggtaaaggagcaaagggtggtatcatggcagtc : 162000
Seq2 : gcgagactatttgaacgagggttccatggcagattctgccgatttagtagtactaggtgcttactatggtaaaggagcaaagggtggtatcatggcagtc : 161998
Seq3 : gcgagactatttgaacgagggttccatggcagattctgccgatttagtagtactaggtgcttactatggtaaaggagcaaagggtggtatcatggcagtc : 161999
Seq4 : gcgagactatttgaacgagggttccatggcagattctgccgatttagtagtactaggtgcttactatggtaaaggagcaaagggtggtatcatggcagtc : 161998

              *    162020          *    162040          *    162060          *    162080          *    162100
Seq1 : tttctaatgggttgttacgacgatgaatccggtaaatggaagacggttaccaagtgttcaggacacgatgataatacgttaagggttttgcaagaccaat : 162100
Seq2 : tttctaatgggttgttacgacgatgaatccggtaaatggaagacggttaccaagtgttcaggacacgatgataatacgttaagggttttgcaagaccaat : 162098
Seq3 : tttctaatgggttgttacgacgatgaatccggtaaatggaagacggttaccaagtgttcaggacacgatgataatacgttaagggttttgcaagaccaat : 162099
Seq4 : tttctaatgggttgttacgacgatgaatccggtaaatggaagacggttaccaagtgttcaggacacgatgataatacgttaagggttttgcaagaccaat : 162098

              *    162120          *    162140          *    162160          *    162180          *    162200
Seq1 : taacgatggttaaaattaacaaggatcccaaaaaaattccagagtggttggtagttaataaaatctatattcccgattttgtagtagaggatccgaaaca : 162200
Seq2 : taacgatggttaaaattaacaaggatcccaaaaaaattccagagtggttggtagttaataaaatctatattcccgattttgtagtagaggatccgaaaca : 162198
Seq3 : taacgatggttaaaattaacaaggatcccaaaaaaattccagagtggttggtagttaataaaatctatattcccgattttgtagtagaggatccgaaaca : 162199
Seq4 : taacgatggttaaaattaacaaggatcccaaaaaaattccagagtggttggtagttaataaaatctatattcccgattttgtagtagaggatccgaaaca : 162198

              *    162220          *    162240          *    162260          *    162280          *    162300
Seq1 : atctcagatatgggaaatttcaggagcagagtttacatcttccaagtcccataccgcaaatggaatatccattagatttcctagatttactaggattaga : 162300
Seq2 : atctcagatatgggaaatttcaggagcagagtttacatcttccaagtcccataccgcaaatggaatatccattagatttcctagatttactaggattaga : 162298
Seq3 : atctcagatatgggaaatttcaggagcagagtttacatcttccaagtcccataccgcaaatggaatatccattagatttcctagatttactaggattaga : 162299
Seq4 : atctcagatatgggaaatttcaggagcagagtttacatcttccaagtcccataccgcaaatggaatatccattagatttcctagatttactaggattaga : 162298

              *    162320          *    162340          *    162360          *    162380          *    162400
Seq1 : gaagataaaacgtggaaagaatctactcatctaaacgatttagtaaacttgactaaatcttaatagttacatacaaactgaaaattaaaataacactatt : 162400
Seq2 : gaagataaaacgtggaaagaatctactcatctaaacgatttagtaaacttgactaaatcttaatagttacatacaaactgaaaattaaaataacactatt : 162398
Seq3 : gaagataaaacgtggaaagaatctactcatctaaacgatttagtaaacttgactaaatcttaatagttacatacaaactgaaaattaaaataacactatt : 162399
Seq4 : gaagataaaacgtggaaagaatctactcatctaaacgatttagtaaacttgactaaatcttaatagttacatacaaactgaaaattaaaataacactatt : 162398
```

```
             *    162420         *    162440         *    162460         *    162480         *    162500
Seq1 : tagttggtggtcgccatggatggtgttattgtatactgtctaaacgcgttagtaaaacatggcgaggaaataaatcatataaaaaatgatttcatgatta : 162500
Seq2 : tagttggtggtcgccatggatggtgttattgtatactgtctaaacgcgttagtaaaacatggcgaggaaataaatcatataaaaaatgatttcatgatta : 162498
Seq3 : tagttggtggtcgccatggatggtgttattgtatactgtctaaacgcgttagtaaaacatggcgaggaaataaatcatataaaaaatgatttcatgatta : 162499
Seq4 : tagttggtggtcgccatggatggtgttattgtatactgtctaaacgcgttagtaaaacatggcgaggaaataaatcatataaaaaatgatttcatgatta : 162498

             *    162520         *    162540         *    162560         *    162580         *    162600
Seq1 : aaccatgttgtgaaagagtttgtgaaaaagtcaagaacgttcacattggcggacaatctaaaaacaatacagtgattgcagatttgccatatatggataa : 162600
Seq2 : aaccatgttgtgaaagagtttgtgaaaaagtcaagaacgttcacattggcggacaatctaaaaacaatacagtgattgcagatttgccatatatggataa : 162598
Seq3 : aaccatgttgtgaaagagtttgtgaaaaagtcaagaacgttcacattggcggacaatctaaaaacaatacagtgattgcagatttgccatatatggataa : 162599
Seq4 : aaccatgttgtgaaagagtttgtgaaaaagtcaagaacgttcacattggcggacaatctaaaaacaatacagtgattgcagatttgccatatatggataa : 162598

             *    162620         *    162640         *    162660         *    162680         *    162700
Seq1 : tgcggtatccgatgtatgcaattcactgtataaaaagaatgtatcaagaatatccagatttgctaatttgataaagatagatgacgatgacaagactcct : 162700
Seq2 : tgcggtatccgatgtatgcaattcactgtataaaaagaatgtatcaagaatatccagatttgctaatttgataaagatagatgacgatgacaagactcct : 162698
Seq3 : tgcggtatccgatgtatgcaattcactgtataaaaagaatgtatcaagaatatccagatttgctaatttgataaagatagatgacgatgacaagactcct : 162699
Seq4 : tgcggtatccgatgtatgcaattcactgtataaaaagaatgtatcaagaatatccagatttgctaatttgataaagatagatgacgatgacaagactcct : 162698

             *    162720         *    162740         *    162760         *    162780         *    162800
Seq1 : actggtgtatataattattttaaacctaaagatgttattcctgttatcatatctataggaaaggataaagatgtctgtgaactattaatctcatcagaca : 162800
Seq2 : actggtgtatataattattttaaacctaaagatgttattcctgttatcatatctataggaaaggataaagatgtctgtgaactattaatctcatcagaca : 162798
Seq3 : actggtgtatataattattttaaacctaaagatgttattcctgttatcatatctataggaaaggataaagatgtctgtgaactattaatctcatcagaca : 162799
Seq4 : actggtgtatataattattttaaacctaaagatgttattcctgttatcatatctataggaaaggataaagatgtctgtgaactattaatctcatcagaca : 162798

             *    162820         *    162840         *    162860         *    162880         *    162900
Seq1 : tatcgtgtgcatgcgtggagttaaattcatatcacgtagccattcttcccatggatgtttcctttttttaccaaaggaaatgcatcattgattattctcct : 162900
Seq2 : tatcgtgtgcatgcgtggagttaaattcatatcacgtagccattcttcccatggatgtttcctttttttaccaaaggaaatgcatcattgattattctcct : 162898
Seq3 : tatcgtgtgcatgcgtggagttaaattcatatcacgtagccattcttcccatggatgtttcctttttttaccaaaggaaatgcatcattgattattctcct : 162899
Seq4 : tatcgtgtgcatgcgtggagttaaattcatatcacgtagccattcttcccatggatgtttcctttttttaccaaaggaaatgcatcattgattattctcct : 162898

             *    162920         *    162940         *    162960         *    162980         *    163000
Seq1 : gtttgatttctctatcgatgcagcacctctcttaagaagtgtaaccgataataatgttattatatctagacaccagcgtctacatgacgagcttccgagt : 163000
Seq2 : gtttgatttctctatcgatgcagcacctctcttaagaagtgtaaccgataataatgttattatatctagacaccagcgtctacatgacgagcttccgagt : 162998
Seq3 : gtttgatttctctatcgatgcagcacctctcttaagaagtgtaaccgataataatgttattatatctagacaccagcgtctacatgacgagcttccgagt : 162999
Seq4 : gtttgatttctctatcgatgcagcacctctcttaagaagtgtaaccgataataatgttattatatctagacaccagcgtctacatgacgagcttccgagt : 162998

             *    163020         *    163040         *    163060         *    163080         *    163100
Seq1 : tccaattggttcaagttttacataagtataaagtccgactattgttctatattatatatggttgttgatggatctgtgatgcatgcgatagctgataata : 163100
Seq2 : tccaattggttcaagttttacataagtataaagtccgactattgttctatattatatatggttgttgatggatctgtgatgcatgcgatagctgataata : 163098
Seq3 : tccaattggttcaagttttacataagtataaagtccgactattgttctatattatatatggttgttgatggatctgtgatgcatgcgatagctgataata : 163099
Seq4 : tccaattggttcaagttttacataagtataaagtccgactattgttctatattatatatggttgttgatggatctgtgatgcatgcgatagctgataata : 163098
```

```
                *    163120         *    163140         *    163160         *    163180         *    163200
Seq1 : gaactcacgcaattattagcaaaaatatattagacaatactacgattaacgatgagtgtagatgctgttattttgaaccacagattaggattcttgatag : 163200
Seq2 : gaactcacgcaattattagcaaaaatatattagacaatactacgattaacgatgagtgtagatgctgttattttgaaccacagattaggattcttgatag : 163198
Seq3 : gaactcacgcaattattagcaaaaatatattagacaatactacgattaacgatgagtgtagatgctgttattttgaaccacagattaggattcttgatag : 163199
Seq4 : gaactcacgcaattattagcaaaaatatattagacaatactacgattaacgatgagtgtagatgctgttattttgaaccacagattaggattcttgatag : 163198

                *    163220         *    163240         *    163260         *    163280         *    163300
Seq1 : agatgagatgctcaatggatcatcgtgtgatatgaacagacattgtattatgatgaatttacctgatgtaggcaaatttggatctagtatgttggggaaa : 163300
Seq2 : agatgagatgctcaatggatcatcgtgtgatatgaacagacattgtattatgatgaatttacctgatgtaggcaaatttggatctagtatgttggggaaa : 163298
Seq3 : agatgagatgctcaatggatcatcgtgtgatatgaacagacattgtattatgatgaatttacctgatgtaggcaaatttggatctagtatgttggggaaa : 163299
Seq4 : agatgagatgctcaatggatcatcgtgtgatatgaacagacattgtattatgatgaatttacctgatgtaggcaaatttggatctagtatgttggggaaa : 163298

                *    163320         *    163340         *    163360         *    163380         *    163400
Seq1 : tatgaacctgacatgattaagattgctctttcggtggctggtaatttaataagaaatcgagactacattcccgggagacgaggatatagctactacgttt : 163400
Seq2 : tatgaacctgacatgattaagattgctctttcggtggctggtaatttaataagaaatcgagactacattcccgggagacgaggatatagctactacgttt : 163398
Seq3 : tatgaacctgacatgattaagattgctctttcggtggctggtaatttaataagaaatcgagactacattcccgggagacgaggatatagctactacgttt : 163399
Seq4 : tatgaacctgacatgattaagattgctctttcggtggctggtaatttaataagaaatcgagactacattcccgggagacgaggatatagctactacgttt : 163398

                *    163420         *    163440         *    163460         *    163480         *    163500
Seq1 : acggtatagcctctagataatttttttaagcacgaaataaaaaacataatttaaaccaatctatttcatactattttgtgtgatcaccatggacataaa : 163500
Seq2 : acggtatagcctctagataatttttttaagcacgaaataaaaaacataatttaaaccaatctatttcatactattttgtgtgatcaccatggacataaa : 163498
Seq3 : acggtatagcctctagataatttttttaagcacgaaataaaaaacataatttaaaccaatctatttcatactattttgtgtgatcaccatggacataaa : 163499
Seq4 : acggtatagcctctagataatttttttaagcacgaaataaaaaacataatttaaaccaatctatttcatactattttgtgtgatcaccatggacataaa : 163498

                *    163520         *    163540         *    163560         *    163580         *    163600
Seq1 : gatagatattagtatttctggtgataaatttacggtgactactaggagggaaaatgaagaaagaaaaaaatatctacctctccaaaaagaaaaaactact : 163600
Seq2 : gatagatattagtatttctggtgataaatttacggtgactactaggagggaaaatgaagaaagaaaaaaatatctacctctccaaaaagaaaaaactact : 163598
Seq3 : gatagatattagtatttctggtgataaatttacggtgactactaggagggaaaatgaagaaagaaaaaaatatctacctctccaaaaagaaaaaactact : 163599
Seq4 : gatagatattagtatttctggtgataaatttacggtgactactaggagggaaaatgaagaaagaaaaaaatatctacctctccaaaaagaaaaaactact : 163598

                *    163620         *    163640         *    163660         *    163680         *    163700
Seq1 : gatgttatcaaacctgattatcttgagtacgatgacttgttagatagagatgagatgtttactattctagaggaatattttatgtacagaggtctattag : 163700
Seq2 : gatgttatcaaacctgattatcttgagtacgatgacttgttagatagagatgagatgtttactattctagaggaatattttatgtacagaggtctattag : 163698
Seq3 : gatgttatcaaacctgattatcttgagtacgatgacttgttagatagagatgagatgtttactattctagaggaatattttatgtacagaggtctattag : 163699
Seq4 : gatgttatcaaacctgattatcttgagtacgatgacttgttagatagagatgagatgtttactattctagaggaatattttatgtacagaggtctattag : 163698

                *    163720         *    163740         *    163760         *    163780         *    163800
Seq1 : gcctcagaataaaatatggacgactctttaacgaaattaaaaaattcgacaatgatgcggaagaacaattcggtactatagaagaactcaagcagaaact : 163800
Seq2 : gcctcagaataaaatatggacgactctttaacgaaattaaaaaattcgacaatgatgcggaagaacaattcggtactatagaagaactcaagcagaaact : 163798
Seq3 : gcctcagaataaaatatggacgactctttaacgaaattaaaaaattcgacaatgatgcggaagaacaattcggtactatagaagaactcaagcagaaact : 163799
Seq4 : gcctcagaataaaatatggacgactctttaacgaaattaaaaaattcgacaatgatgcggaagaacaattcggtactatagaagaactcaagcagaaact : 163798
```

```
             *    163820          *    163840          *    163860          *    163880          *    163900
Seq1 : tagattaaattctgaagagggagcagataactttatagattatataaaggtacaaaaacaggatatcgtcaaacttactgtatacgattgcatatctatg : 163900
Seq2 : tagattaaattctgaagagggagcagataactttatagattatataaaggtacaaaaacaggatatcgtcaaacttactgtatacgattgcatatctatg : 163898
Seq3 : tagattaaattctgaagagggagcagataactttatagattatataaaggtacaaaaacaggatatcgtcaaacttactgtatacgattgcatatctatg : 163899
Seq4 : tagattaaattctgaagagggagcagataactttatagattatataaaggtacaaaaacaggatatcgtcaaacttactgtatacgattgcatatctatg : 163898

             *    163920          *    163940          *    163960          *    163980          *    164000
Seq1 : ataggattgtgtgcatgcgtggtagatgtttggagaaatgagaaactgttttctagatggaaatattgtttacgagctattaaactgtttattaatgatc : 164000
Seq2 : ataggattgtgtgcatgcgtggtagatgtttggagaaatgagaaactgttttctagatggaaatattgtttacgagctattaaactgtttattaatgatc : 163998
Seq3 : ataggattgtgtgcatgcgtggtagatgtttggagaaatgagaaactgttttctagatggaaatattgtttacgagctattaaactgtttattaatgatc : 163999
Seq4 : ataggattgtgtgcatgcgtggtagatgtttggagaaatgagaaactgttttctagatggaaatattgtttacgagctattaaactgtttattaatgatc : 163998

             *    164020          *    164040          *    164060          *    164080          *    164100
Seq1 : acatgcttgataagataaaatctatactgcagaatagactagtatatgtggaaatgtcatagaaagttaaaagttaatgagagcaaaaatatataaggtt : 164100
Seq2 : acatgcttgataagataaaatctatactgcagaatagactagtatatgtggaaatgtcatagaaagttaaaagttaatgagagcaaaaatatataaggtt : 164098
Seq3 : acatgcttgataagataaaatctatactgcagaatagactagtatatgtggaaatgtcatagaaagttaaaagttaatgagagcaaaaatatataaggtt : 164099
Seq4 : acatgcttgataagataaaatctatactgcagaatagactagtatatgtggaaatgtcatagaaagttaaaagttaatgagagcaaaaatatataaggtt : 164098

             *    164120          *    164140          *    164160          *    164180          *    164200
Seq1 : gtattccatatttgttattttttctgtaatagttagaaaaatacattcgatggtctatctaccagattattatgtgttataaggtactttttctcataat : 164200
Seq2 : gtattccatatttgttattttttctgtaatagttagaaaaatacattcgatggtctatctaccagattattatgtgttataaggtactttttctcataat : 164198
Seq3 : gtattccatatttgttattttttctgtaatagttagaaaaatacattcgatggtctatctaccagattattatgtgttataaggtactttttctcataat : 164199
Seq4 : gtattccatatttgttattttttctgtaatagttagaaaaatacattcgatggtctatctaccagattattatgtgttataaggtactttttctcataat : 164198

             *    164220          *    164240          *    164260          *    164280          *    164300
Seq1 : aaactagagtatgagtaagatagtgtttttcaaaacatataaatctaaaattgatggatgagatatacagctattaatttcgaaaatatattttaatctg : 164300
Seq2 : aaactagagtatgagtaagatagtgtttttcaaaacatataaatctaaaattgatggatgagatatacagctattaatttcgaaaatatattttaatctg : 164298
Seq3 : aaactagagtatgagtaagatagtgtttttcaaaacatataaatctaaaattgatggatgagatatacagctattaatttcgaaaatatattttaatctg : 164299
Seq4 : aaactagagtatgagtaagatagtgtttttcaaaacatataaatctaaaattgatggatgagatatacagctattaatttcgaaaatatattttaatctg : 164298

             *    164320          *    164340          *    164360          *    164380          *    164400
Seq1 : ataactttaaacatggattttgatggtggtttaacgttttaaaaaaaagattttgttattgtagtatatgataatattaaaagatggatataaagaattt : 164400
Seq2 : ataactttaaacatggattttgatggtggtttaacgttttaaaaaaaagattttgttattgtagtatatgataatattaaaagatggatataaagaattt : 164398
Seq3 : ataactttaaacatggattttgatggtggtttaacgttttaaaaaaaagattttgttattgtagtatatgataatattaaaagatggatataaagaattt : 164399
Seq4 : ataactttaaacatggattttgatggtggtttaacgttttaaaaaaaagattttgttattgtagtatatgataatattaaaagatggatataaagaattt : 164398

             *    164420          *    164440          *    164460          *    164480          *    164500
Seq1 : gctgactgcatgtactattttttacattactacattggctacggcagatatacctactccgccaccaacgggtcatgtgacaagggagaatatcttgata : 164500
Seq2 : gctgactgcatgtactattttttacattactacattggctacggcagatatacctactccgccaccaacgggtcatgtgacaagggagaatatcttgata : 164498
Seq3 : gctgactgcatgtactattttttacattactacattggctacggcagatatacctactccgccaccaacgggtcatgtgacaagggagaatatcttgata : 164499
Seq4 : gctgactgcatgtactattttttacattactacattggctacggcagatatacctactccgccaccaacgggtcatgtgacaagggagaatatcttgata : 164498
```

```
           *    164520         *    164540         *    164560         *    164580         *    164600
Seq1 : agaggcataatcaatgttgtaatcggtgtccacctggagaatttgccaaggttagatgtaatggtaacgataacacaaaatgtgaacgctgcccacctca : 164600
Seq2 : agaggcataatcaatgttgtaatcggtgtccacctggagaatttgccaaggttagatgtaatggtaacgataacacaaaatgtgaacgctgcccacctca : 164598
Seq3 : agaggcataatcaatgttgtaatcggtgtccacctggagaatttgccaaggttagatgtaatggtaacgataacacaaaatgtgaacgctgcccacctca : 164599
Seq4 : agaggcataatcaatgttgtaatcggtgtccacctggagaatttgccaaggttagatgtaatggtaacgataacacaaaatgtgaacgctgcccacctca : 164598

           *    164620         *    164640         *    164660         *    164680         *    164700
Seq1 : tacatataccacaatcccaattattctaatggatgtcatcaatgtagaaaatgcccaaccggatcatttgataaggtaaagtgtaccggaacacagaaca : 164700
Seq2 : tacatataccacaatcccaattattctaatggatgtcatcaatgtagaaaatgcccaaccggatcatttgataaggtaaagtgtaccggaacacagaaca : 164698
Seq3 : tacatataccacaatcccaattattctaatggatgtcatcaatgtagaaaatgcccaaccggatcatttgataaggtaaagtgtaccggaacacagaaca : 164699
Seq4 : tacatataccacaatcccaattattctaatggatgtcatcaatgtagaaaatgcccaaccggatcatttgataaggtaaagtgtaccggaacacagaaca : 164698

           *    164720         *    164740         *    164760         *    164780         *    164800
Seq1 : gtaaatgttcgtgtcttcctggttggtattgtgctactgattcttcacagactgaagattgttgaaattgtgtaccaaaaaggagatgtccatgcggata : 164800
Seq2 : gtaaatgttcgtgtcttcctggttggtattgtgctactgattcttcacagactgaagattgttgaaattgtgtaccaaaaaggagatgtccatgcggata : 164798
Seq3 : gtaaatgttcgtgtcttcctggttggtattgtgctactgattcttcacagactgaagattgttgaaattgtgtaccaaaaaggagatgtccatgcggata : 164799
Seq4 : gtaaatgttcgtgtcttcctggttggtattgtgctactgattcttcacagactgaagattgttgaaattgtgtaccaaaaaggagatgtccatgcggata : 164798

           *    164820         *    164840         *    164860         *    164880         *    164900
Seq1 : ctttggtggaatagatgaacaaggaaatcctatttgtaaatcgtgttgtgttggtgaatattgcgactacctacgtaattatagacttgatccatttcct : 164900
Seq2 : ctttggtggaatagatgaacaaggaaatcctatttgtaaatcgtgttgtgttggtgaatattgcgactacctacgtaattatagacttgatccatttcct : 164898
Seq3 : ctttggtggaatagatgaacaaggaaatcctatttgtaaatcgtgttgtgttggtgaatattgcgactacctacgtaattatagacttgatccatttcct : 164899
Seq4 : ctttggtggaatagatgaacaaggaaatcctatttgtaaatcgtgttgtgttggtgaatattgcgactacctacgtaattatagacttgatccatttcct : 164898

           *    164920         *    164940         *    164960         *    164980         *    165000
Seq1 : ccatgcaaactatctaaatgtaattaattatgattttgatgataatgttaccatacattatatcgctacttggttagtgtattattcagtatgaagacct : 165000
Seq2 : ccatgcaaactatctaaatgtaattaattatgattttgatgataatgttaccatacattatatcgctacttggttagtgtattattcagtatgaagacct : 164998
Seq3 : ccatgcaaactatctaaatgtaattaattatgattttgatgataatgttaccatacattatatcgctacttggttagtgtattattcagtatgaagacct : 164999
Seq4 : ccatgcaaactatctaaatgtaattaattatgattttgatgataatgttaccatacattatatcgctacttggttagtgtattattcagtatgaagacct : 164998

           *    165020         *    165040         *    165060         *    165080         *    165100
Seq1 : attaataattacttatcttttgacgatcttgttataattataatataaaaatacttatggcatagtaactcataattgctgacgcgataaattcgtaata : 165100
Seq2 : attaataattacttatcttttgacgatcttgttataattataatataaaaatacttatggcatagtaactcataattgctgacgcgataaattcgtaata : 165098
Seq3 : attaataattacttatcttttgacgatcttgttataattataatataaaaatacttatggcatagtaactcataattgctgacgcgataaattcgtaata : 165099
Seq4 : attaataattacttatcttttgacgatcttgttataattataatataaaaatacttatggcatagtaactcataattgctgacgcgataaattcgtaata : 165098

           *    165120         *    165140         *    165160         *    165180         *    165200
Seq1 : atctgttttgttcaaatttttataaggaatctacaggcataaaaataaaaatataatttataatatactcttacagcgcgccatcatgaataacagcagt : 165200
Seq2 : atctgttttgttcaaatttttataaggaatctacaggcataaaaataaaaatataatttataatatactcttacagcgcgccatcatgaataacagcagt : 165198
Seq3 : atctgttttgttcaaatttttataaggaatctacaggcataaaaataaaaatataatttataatatactcttacagcgcgccatcatgaataacagcagt : 165199
Seq4 : atctgttttgttcaaatttttataaggaatctacaggcataaaaataaaaatataatttataatatactcttacagcgcgccatcatgaataacagcagt : 165198
```

```
                 *    165220         *    165240         *    165260         *    165280         *    165300
Seq1 : gaattgattgctgttattaatggatttagaaatagtggacgattttgtgatattagtatagttattaatgatgaaaggataaacgctcataaactcatcc : 165300
Seq2 : gaattgattgctgttattaatggatttagaaatagtggacgattttgtgatattagtatagttattaatgatgaaaggataaacgctcataaactcatcc : 165298
Seq3 : gaattgattgctgttattaatggatttagaaatagtggacgattttgtgatattagtatagttattaatgatgaaaggataaacgctcataaactcatcc : 165299
Seq4 : gaattgattgctgttattaatggatttagaaatagtggacgattttgtgatattagtatagttattaatgatgaaaggataaacgctcataaactcatcc : 165298

                 *    165320         *    165340         *    165360         *    165380         *    165400
Seq1 : tatctggagcctccgaatatttttccattctgttttccaataattttatcgattctaatgaatacgaagttaatctaagtcatttagattatcaaagcgt : 165400
Seq2 : tatctggagcctccgaatatttttccattctgttttccaataattttatcgattctaatgaatacgaagttaatctaagtcatttagattatcaaagcgt : 165398
Seq3 : tatctggagcctccgaatatttttccattctgttttccaataattttatcgattctaatgaatacgaagttaatctaagtcatttagattatcaaagcgt : 165399
Seq4 : tatctggagcctccgaatatttttccattctgttttccaataattttatcgattctaatgaatacgaagttaatctaagtcatttagattatcaaagcgt : 165398

                 *    165420         *    165440         *    165460         *    165480         *    165500
Seq1 : taacgatttgatcgattacatttatgggatacctttgagcctaactaacgataacgtgaaatatattctttcaaccgctgatttttttacaaattggatct : 165500
Seq2 : taacgatttgatcgattacatttatgggatacctttgagcctaactaacgataacgtgaaatatattctttcaaccgctgatttttttacaaattggatct : 165498
Seq3 : taacgatttgatcgattacatttatgggatacctttgagcctaactaacgataacgtgaaatatattctttcaaccgctgatttttttacaaattggatct : 165499
Seq4 : taacgatttgatcgattacatttatgggatacctttgagcctaactaacgataacgtgaaatatattctttcaaccgctgatttttttacaaattggatct : 165498

                 *    165520         *    165540         *    165560         *    165580         *    165600
Seq1 : gctattacggagtgtgaaaattacatacttaaaaatctttgttctaaaaactgtatcgatttctacatatacgctgataaatataataacaagaaaatag : 165600
Seq2 : gctattacggagtgtgaaaattacatacttaaaaatctttgttctaaaaactgtatcgatttctacatatacgctgataaatataataacaagaaaatag : 165598
Seq3 : gctattacggagtgtgaaaattacatacttaaaaatctttgttctaaaaactgtatcgatttctacatatacgctgataaatataataacaagaaaatag : 165599
Seq4 : gctattacggagtgtgaaaattacatacttaaaaatctttgttctaaaaactgtatcgatttctacatatacgctgataaatataataacaagaaaatag : 165598

                 *    165620         *    165640         *    165660         *    165680         *    165700
Seq1 : aatcagcgtcgtttaacacaatattacaaaatattttgagactcatcaacgatgaaaactttaaatacttaacagaggaatcaatgataaaaattttaag : 165700
Seq2 : aatcagcgtcgtttaacacaatattacaaaatattttgagactcatcaacgatgaaaactttaaatacttaacagaggaatcaatgataaaaattttaag : 165698
Seq3 : aatcagcgtcgtttaacacaatattacaaaatattttgagactcatcaacgatgaaaactttaaatacttaacagaggaatcaatgataaaaattttaag : 165699
Seq4 : aatcagcgtcgtttaacacaatattacaaaatattttgagactcatcaacgatgaaaactttaaatacttaacagaggaatcaatgataaaaattttaag : 165698

                 *    165720         *    165740         *    165760         *    165780         *    165800
Seq1 : cgatgatatgttaaatataaaaaatgaggattttgcaccactaattctcattaaatggttagagagtactcaacaatcatgcaccgtcgagttacttaga : 165800
Seq2 : cgatgatatgttaaatataaaaaatgaggattttgcaccactaattctcattaaatggttagagagtactcaacaatcatgcaccgtcgagttacttaga : 165798
Seq3 : cgatgatatgttaaatataaaaaatgaggattttgcaccactaattctcattaaatggttagagagtactcaacaatcatgcaccgtcgagttacttaga : 165799
Seq4 : cgatgatatgttaaatataaaaaatgaggattttgcaccactaattctcattaaatggttagagagtactcaacaatcatgcaccgtcgagttacttaga : 165798

                 *    165820         *    165840         *    165860         *    165880         *    165900
Seq1 : tgcctcagaatatcattgctttccccacaagttataaaatcactttatagtcatcaactggttagttcaatctacgaatgtataacattcttaaacaata : 165900
Seq2 : tgcctcagaatatcattgctttccccacaagttataaaatcactttatagtcatcaactggttagttcaatctacgaatgtataacattcttaaacaata : 165898
Seq3 : tgcctcagaatatcattgctttccccacaagttataaaatcactttatagtcatcaactggttagttcaatctacgaatgtataacattcttaaacaata : 165899
Seq4 : tgcctcagaatatcattgctttccccacaagttataaaatcactttatagtcatcaactggttagttcaatctacgaatgtataacattcttaaacaata : 165898
```

```
                *    165920         *    165940         *    165960         *    165980         *    166000
Seq1 : tagcattcttggatgaatcatttcctagataccatagcatcgagttgatatctatcggtataagtaattcgcatgataagatttccataaactgctacaa : 166000
Seq2 : tagcattcttggatgaatcatttcctagataccatagcatcgagttgatatctatcggtataagtaattcgcatgataagatttccataaactgctacaa : 165998
Seq3 : tagcattcttggatgaatcatttcctagataccatagcatcgagttgatatctatcggtataagtaattcgcatgataagatttccataaactgctacaa : 165999
Seq4 : tagcattcttggatgaatcatttcctagataccatagcatcgagttgatatctatcggtataagtaattcgcatgataagatttccataaactgctacaa : 165998

                *    166020         *    166040         *    166060         *    166080         *    166100
Seq1 : tcataaaaaaaatacatgggaaatgatatcttcacgtagatataggtgtagtttcgcagtggccgtcctggataatattatctatatgatgggtggatat : 166100
Seq2 : tcataaaaaaaatacatgggaaatgatatcttcacgtagatataggtgtagtttcgcagtggccgtcctggataatattatctatatgatgggtggatat : 166098
Seq3 : tcataaaaaaaatacatgggaaatgatatcttcacgtagatataggtgtagtttcgcagtggccgtcctggataatattatctatatgatgggtggatat : 166099
Seq4 : tcataaaaaaaatacatgggaaatgatatcttcacgtagatataggtgtagtttcgcagtggccgtcctggataatattatctatatgatgggtggatat : 166098

                *    166120         *    166140         *    166160         *    166180         *    166200
Seq1 : gatcagtccccgtatagaagttcaaaggttatagcgtacaatacatgtacaaattcttggatatatgatataccagagctaaaatatcctcgttctaatt : 166200
Seq2 : gatcagtccccgtatagaagttcaaaggttatagcgtacaatacatgtacaaattcttggatatatgatataccagagctaaaatatcctcgttctaatt : 166198
Seq3 : gatcagtccccgtatagaagttcaaaggttatagcgtacaatacatgtacaaattcttggatatatgatataccagagctaaaatatcctcgttctaatt : 166199
Seq4 : gatcagtccccgtatagaagttcaaaggttatagcgtacaatacatgtacaaattcttggatatatgatataccagagctaaaatatcctcgttctaatt : 166198

                *    166220         *    166240         *    166260         *    166280         *    166300
Seq1 : gtgggggactggctgatgacgaatacatttattgtataggcggcatacgcgatcaggattcatcgttgacatctagtattgataaatggaagccatcaaa : 166300
Seq2 : gtgggggactggctgatgacgaatacatttattgtataggcggcatacgcgatcaggattcatcgttgacatctagtattgataaatggaagccatcaaa : 166298
Seq3 : gtgggggactggctgatgacgaatacatttattgtataggcggcatacgcgatcaggattcatcgttgacatctagtattgataaatggaagccatcaaa : 166299
Seq4 : gtgggggactggctgatgacgaatacatttattgtataggcggcatacgcgatcaggattcatcgttgacatctagtattgataaatggaagccatcaaa : 166298

                *    166320         *    166340         *    166360         *    166380         *    166400
Seq1 : accatattggcagaagtatgctaaaatgcgcgaaccaaaatgtgatatgggggttgcgatgttaaacggattaatatatgtcatgggtggaatcgttaaa : 166400
Seq2 : accatattggcagaagtatgctaaaatgcgcgaaccaaaatgtgatatgggggttgcgatgttaaacggattaatatatgtcatgggtggaatcgttaaa : 166398
Seq3 : accatattggcagaagtatgctaaaatgcgcgaaccaaaatgtgatatgggggttgcgatgttaaacggattaatatatgtcatgggtggaatcgttaaa : 166399
Seq4 : accatattggcagaagtatgctaaaatgcgcgaaccaaaatgtgatatgggggttgcgatgttaaacggattaatatatgtcatgggtggaatcgttaaa : 166398

                *    166420         *    166440         *    166460         *    166480         *    166500
Seq1 : ggtgacacgtgtaccgacgcactagagagtttatcagaagatggatggatgaagcatcaacgtcttccaataaaaatgtccaatatgtcgacgattgttc : 166500
Seq2 : ggtgacacgtgtaccgacgcactagagagtttatcagaagatggatggatgaagcatcaacgtcttccaataaaaatgtccaatatgtcgacgattgttc : 166498
Seq3 : ggtgacacgtgtaccgacgcactagagagtttatcagaagatggatggatgaagcatcaacgtcttccaataaaaatgtccaatatgtcgacgattgttc : 166499
Seq4 : ggtgacacgtgtaccgacgcactagagagtttatcagaagatggatggatgaagcatcaacgtcttccaataaaaatgtccaatatgtcgacgattgttc : 166498

                *    166520         *    166540         *    166560         *    166580         *    166600
Seq1 : atgatggcaagatttatatatctggaggttacaacaatagtagtgtagttaatgtaatatcgaatctagtccttagctataatccgatatatgatgaatg : 166600
Seq2 : atgatggcaagatttatatatctggaggttacaacaatagtagtgtagttaatgtaatatcgaatctagtccttagctataatccgatatatgatgaatg : 166598
Seq3 : atgatggcaagatttatatatctggaggttacaacaatagtagtgtagttaatgtaatatcgaatctagtccttagctataatccgatatatgatgaatg : 166599
Seq4 : atgatggcaagatttatatatctggaggttacaacaatagtagtgtagttaatgtaatatcgaatctagtccttagctataatccgatatatgatgaatg : 166598
```

```
                *    166620         *    166640         *    166660         *    166680         *    166700
Seq1 : gaccaaattatcatcattaaacattcctagaattaatcccgctctatggtcagcgcataataaattatatgtaggaggaggaatatctgatgatgttcga : 166700
Seq2 : gaccaaattatcatcattaaacattcctagaattaatcccgctctatggtcagcgcataataaattatatgtaggaggaggaatatctgatgatgttcga : 166698
Seq3 : gaccaaattatcatcattaaacattcctagaattaatcccgctctatggtcagcgcataataaattatatgtaggaggaggaatatctgatgatgttcga : 166699
Seq4 : gaccaaattatcatcattaaacattcctagaattaatcccgctctatggtcagcgcataataaattatatgtaggaggaggaatatctgatgatgttcga : 166698

                *    166720         *    166740         *    166760         *    166780         *    166800
Seq1 : actaatacatctgaaacatacgataaagaaaaagattgttggacattggataatggtcacgtgttaccacgcaattatataatgtataaatgcgaaccga : 166800
Seq2 : actaatacatctgaaacatacgataaagaaaaagattgttggacattggataatggtcacgtgttaccacgcaattatataatgtataaatgcgaaccga : 166798
Seq3 : actaatacatctgaaacatacgataaagaaaaagattgttggacattggataatggtcacgtgttaccacgcaattatataatgtataaatgcgaaccga : 166799
Seq4 : actaatacatctgaaacatacgataaagaaaaagattgttggacattggataatggtcacgtgttaccacgcaattatataatgtataaatgcgaaccga : 166798

                *    166820         *    166840         *    166860         *    166880         *    166900
Seq1 : ttaaacataaatatccattggaaaaaacacagtacacgaatgattttctaaagtatttggaaagttttataggtagttgatagaacaaaatacataattt : 166900
Seq2 : ttaaacataaatatccattggaaaaaacacagtacacgaatgattttctaaagtatttggaaagttttataggtagttgatagaacaaaatacataattt : 166898
Seq3 : ttaaacataaatatccattggaaaaaacacagtacacgaatgattttctaaagtatttggaaagttttataggtagttgatagaacaaaatacataattt : 166899
Seq4 : ttaaacataaatatccattggaaaaaacacagtacacgaatgattttctaaagtatttggaaagttttataggtagttgatagaacaaaatacataattt : 166898

                *    166920         *    166940         *    166960         *    166980         *    167000
Seq1 : tgtaaaaataaatcactttttatactaatatgacacgattaccaatacttttgttactaatatcattagtatacgctacaccttttcctcagacatctaa : 167000
Seq2 : tgtaaaaataaatcactttttatactaatatgacacgattaccaatacttttgttactaatatcattagtatacgctacaccttttcctcagacatctaa : 166998
Seq3 : tgtaaaaataaatcactttttatactaatatgacacgattaccaatacttttgttactaatatcattagtatacgctacaccttttcctcagacatctaa : 166999
Seq4 : tgtaaaaataaatcactttttatactaatatgacacgattaccaatacttttgttactaatatcattagtatacgctacaccttttcctcagacatctaa : 166998

                *    167020         *    167040         *    167060         *    167080         *    167100
Seq1 : aaaaataggtgatgatgcaactctatcatgtaatcgaaataatacaaatgactacgttgttatgagtgcttggtataaggagcccaattccattattctt : 167100
Seq2 : aaaaataggtgatgatgcaactctatcatgtaatcgaaataatacaaatgactacgttgttatgagtgcttggtataaggagcccaattccattattctt : 167098
Seq3 : aaaaataggtgatgatgcaactctatcatgtaatcgaaataatacaaatgactacgttgttatgagtgcttggtataaggagcccaattccattattctt : 167099
Seq4 : aaaaataggtgatgatgcaactctatcatgtaatcgaaataatacaaatgactacgttgttatgagtgcttggtataaggagcccaattccattattctt : 167098

                *    167120         *    167140         *    167160         *    167180         *    167200
Seq1 : ttagctgctaaaagcgacgtcttgtattttgataattataccaaggataaaatatcttacgactctccatacgatgatctagttacaactatcacaatta : 167200
Seq2 : ttagctgctaaaagcgacgtcttgtattttgataattataccaaggataaaatatcttacgactctccatacgatgatctagttacaactatcacaatta : 167198
Seq3 : ttagctgctaaaagcgacgtcttgtattttgataattataccaaggataaaatatcttacgactctccatacgatgatctagttacaactatcacaatta : 167199
Seq4 : ttagctgctaaaagcgacgtcttgtattttgataattataccaaggataaaatatcttacgactctccatacgatgatctagttacaactatcacaatta : 167198

                *    167220         *    167240         *    167260         *    167280         *    167300
Seq1 : aatcattgactgctagagatgccggtacttatgtatgtgcattctttatgacatcaactacaaatgacactgataaagtagattatgaagaatactccac : 167300
Seq2 : aatcattgactgctagagatgccggtacttatgtatgtgcattctttatgacatcaactacaaatgacactgataaagtagattatgaagaatactccac : 167298
Seq3 : aatcattgactgctagagatgccggtacttatgtatgtgcattctttatgacatcaactacaaatgacactgataaagtagattatgaagaatactccac : 167299
Seq4 : aatcattgactgctagagatgccggtacttatgtatgtgcattctttatgacatcaactacaaatgacactgataaagtagattatgaagaatactccac : 167298
```

```
                  *    167320         *    167340         *    167360         *    167380         *    167400
Seq1 : agagttgattgtaaatacagatagtgaatcgactatagacataatactatctggatctacacattcaccggaaactagttctaagaaacctgattatata : 167400
Seq2 : agagttgattgtaaatacagatagtgaatcgactatagacataatactatctggatctacacattcaccggaaactagttctaagaaacctgattatata : 167398
Seq3 : agagttgattgtaaatacagatagtgaatcgactatagacataatactatctggatctacacattcaccggaaactagttctaagaaacctgattatata : 167399
Seq4 : agagttgattgtaaatacagatagtgaatcgactatagacataatactatctggatctacacattcaccggaaactagttctaagaaacctgattatata : 167398

                  *    167420         *    167440         *    167460         *    167480         *    167500
Seq1 : gataattctaattgctcgtcggtattcgaaatcgcgactccggaaccaattactgataatgtagaagatcatacagacaccgtcacatacactagtgata : 167500
Seq2 : gataattctaattgctcgtcggtattcgaaatcgcgactccggaaccaattactgataatgtagaagatcatacagacaccgtcacatacactagtgata : 167498
Seq3 : gataattctaattgctcgtcggtattcgaaatcgcgactccggaaccaattactgataatgtagaagatcatacagacaccgtcacatacactagtgata : 167499
Seq4 : gataattctaattgctcgtcggtattcgaaatcgcgactccggaaccaattactgataatgtagaagatcatacagacaccgtcacatacactagtgata : 167498

                  *    167520         *    167540         *    167560         *    167580         *    167600
Seq1 : gcattaatacagtaagtgcatcatctggagaatccacaacagacgagactccggaaccaattactgataaagaagatcatacagttacagacactgtctc : 167600
Seq2 : gcattaatacagtaagtgcatcatctggagaatccacaacagacgagactccggaaccaattactgataaagaagatcatacagttacagacactgtctc : 167598
Seq3 : gcattaatacagtaagtgcatcatctggagaatccacaacagacgagactccggaaccaattactgataaagaagatcatacagttacagacactgtctc : 167599
Seq4 : gcattaatacagtaagtgcatcatctggagaatccacaacagacgagactccggaaccaattactgataaagaagatcatacagttacagacactgtctc : 167598

                  *    167620         *    167640         *    167660         *    167680         *    167700
Seq1 : atacactacagtaagtacatcatctggaattgtcactactaaatcaaccaccgatgatgcggatctttatgatacgtacaatgataatgatacagtacca : 167700
Seq2 : atacactacagtaagtacatcatctggaattgtcactactaaatcaaccaccgatgatgcggatctttatgatacgtacaatgataatgatacagtacca : 167698
Seq3 : atacactacagtaagtacatcatctggaattgtcactactaaatcaaccaccgatgatgcggatctttatgatacgtacaatgataatgatacagtacca : 167699
Seq4 : atacactacagtaagtacatcatctggaattgtcactactaaatcaaccaccgatgatgcggatctttatgatacgtacaatgataatgatacagtacca : 167698

                  *    167720         *    167740         *    167760         *    167780         *    167800
Seq1 : ccaactactgtaggcggtagtacaacctctattagcaattataaaaccaaggactttgtagaaatatttggtattaccgcattaattatattgtcggccg : 167800
Seq2 : ccaactactgtaggcggtagtacaacctctattagcaattataaaaccaaggactttgtagaaatatttggtattaccgcattaattatattgtcggccg : 167798
Seq3 : ccaactactgtaggcggtagtacaacctctattagcaattataaaaccaaggactttgtagaaatatttggtattaccgcattaattatattgtcggccg : 167799
Seq4 : ccaactactgtaggcggtagtacaacctctattagcaattataaaaccaaggactttgtagaaatatttggtattaccgcattaattatattgtcggccg : 167798

                  *    167820         *    167840         *    167860         *    167880         *    167900
Seq1 : tggcaattttctgtattacatattatatatataataaacgttcacgtaaatacaaaacagagaacaaagtctagatttttgacttacataaatgtctggg : 167900
Seq2 : tggcaattttctgtattacatattatatatataataaacgttcacgtaaatacaaaacagagaacaaagtctagatttttgacttacataaatgtctggg : 167898
Seq3 : tggcaattttctgtattacatattatatatataataaacgttcacgtaaatacaaaacagagaacaaagtctagatttttgacttacataaatgtctggg : 167899
Seq4 : tggcaattttctgtattacatattatatatataataaacgttcacgtaaatacaaaacagagaacaaagtctagatttttgacttacataaatgtctggg : 167898

                  *    167920         *    167940         *    167960         *    167980         *    168000
Seq1 : atagtaaaatctatcatattgagcggaccatctggtttaggaaagacagccatagccaaaagactatgggaatatatttggatttgtggtgtcccatacc : 168000
Seq2 : atagtaaaatctatcatattgagcggaccatctggtttaggaaagacagccatagccaaaagactatgggaatatatttggatttgtggtgtcccatacc : 167998
Seq3 : atagtaaaatctatcatattgagcggaccatctggtttaggaaagacagccatagccaaaagactatgggaatatatttggatttgtggtgtcccatacc : 167999
Seq4 : atagtaaaatctatcatattgagcggaccatctggtttaggaaagacagccatagccaaaagactatgggaatatatttggatttgtggtgtcccatacc : 167998
```

```
                 *    168020          *    168040          *    168060          *    168080          *    168100
Seq1 : actagatttcctcgtcctatggaacgagaaggtgttgattaccattacgttaacagagaggccatctggaagggaatagccgccggaaactttctagaac : 168100
Seq2 : actagatttcctcgtcctatggaacgagaaggtgttgattaccattacgttaacagagaggccatctggaagggaatagccgccggaaactttctagaac : 168098
Seq3 : actagatttcctcgtcctatggaacgagaaggtgttgattaccattacgttaacagagaggccatctggaagggaatagccgccggaaactttctagaac : 168099
Seq4 : actagatttcctcgtcctatggaacgagaaggtgttgattaccattacgttaacagagaggccatctggaagggaatagccgccggaaactttctagaac : 168098

                 *    168120          *    168140          *    168160          *    168180          *    168200
Seq1 : atactgagtttttaggaaatatttacggaacttctaaaacagctgtgaatacagcggctattaataatcgtatttgtgtgatggatctaaacatcgacgg : 168200
Seq2 : atactgagtttttaggaaatatttacggaacttctaaaacagctgtgaatacagcggctattaataatcgtatttgtgtgatggatctaaacatcgacgg : 168198
Seq3 : atactgagtttttaggaaatatttacggaacttctaaaacagctgtgaatacagcggctattaataatcgtatttgtgtgatggatctaaacatcgacgg : 168199
Seq4 : atactgagtttttaggaaatatttacggaacttctaaaacagctgtgaatacagcggctattaataatcgtatttgtgtgatggatctaaacatcgacgg : 168198

                 *    168220          *    168240          *    168260          *    168280          *    168300
Seq1 : tgttagaagtcttaaaaatacgtacctaatgccttactcggtgtatataagacctacctctcttaaaatggttgagaccaagcttcgttgtagaaacact : 168300
Seq2 : tgttagaagtcttaaaaatacgtacctaatgccttactcggtgtatataagacctacctctcttaaaatggttgagaccaagcttcgttgtagaaacact : 168298
Seq3 : tgttagaagtcttaaaaatacgtacctaatgccttactcggtgtatataagacctacctctcttaaaatggttgagaccaagcttcgttgtagaaacact : 168299
Seq4 : tgttagaagtcttaaaaatacgtacctaatgccttactcggtgtatataagacctacctctcttaaaatggttgagaccaagcttcgttgtagaaacact : 168298

                 *    168320          *    168340          *    168360          *    168380          *    168400
Seq1 : gaagctaacgatgagattcatcgtcgcgtgatattggcaaaaacggatatggatgaggccaacgaagcaggtctattcgacactattatcattgaagatg : 168400
Seq2 : gaagctaacgatgagattcatcgtcgcgtgatattggcaaaaacggatatggatgaggccaacgaagcaggtctattcgacactattatcattgaagatg : 168398
Seq3 : gaagctaacgatgagattcatcgtcgcgtgatattggcaaaaacggatatggatgaggccaacgaagcaggtctattcgacactattatcattgaagatg : 168399
Seq4 : gaagctaacgatgagattcatcgtcgcgtgatattggcaaaaacggatatggatgaggccaacgaagcaggtctattcgacactattatcattgaagatg : 168398

                 *    168420          *    168440          *    168460          *    168480          *    168500
Seq1 : atgtgaatttagcatatagtaagttaattcagatactacaggaccgtattagaatgtattttaacactaattagagacttaagacttaaaacttgataat : 168500
Seq2 : atgtgaatttagcatatagtaagttaattcagatactacaggaccgtattagaatgtattttaacactaattagagacttaagacttaaaacttgataat : 168498
Seq3 : atgtgaatttagcatatagtaagttaattcagatactacaggaccgtattagaatgtattttaacactaattagagacttaagacttaaaacttgataat : 168499
Seq4 : atgtgaatttagcatatagtaagttaattcagatactacaggaccgtattagaatgtattttaacactaattagagacttaagacttaaaacttgataat : 168498

                 *    168520          *    168540          *    168560          *    168580          *    168600
Seq1 : taataatataactcgtttttatatgtggctatttcaacgtctaatgtattagttaaatattaaaacttaccacgtaaaacttaaaatttaaaatgatatt : 168600
Seq2 : taataatataactcgtttttatatgtggctatttcaacgtctaatgtattagttaaatattaaaacttaccacgtaaaacttaaaatttaaaatgatatt : 168598
Seq3 : taataatataactcgtttttatatgtggctatttcaacgtctaatgtattagttaaatattaaaacttaccacgtaaaacttaaaatttaaaatgatatt : 168599
Seq4 : taataatataactcgtttttatatgtggctatttcaacgtctaatgtattagttaaatattaaaacttaccacgtaaaacttaaaatttaaaatgatatt : 168598

                 *    168620          *    168640          *    168660          *    168680          *    168700
Seq1 : tcattgacagatagatcacacattatgaactttcaaggacttgtgttaactgacaattgcaaaaatcaatgggtcgttggaccattaataggaaaaggtg : 168700
Seq2 : tcattgacagatagatcacacattatgaactttcaaggacttgtgttaactgacaattgcaaaaatcaatgggtcgttggaccattaataggaaaaggtg : 168698
Seq3 : tcattgacagatagatcacacattatgaactttcaaggacttgtgttaactgacaattgcaaaaatcaatgggtcgttggaccattaataggaaaaggtg : 168699
Seq4 : tcattgacagatagatcacacattatgaactttcaaggacttgtgttaactgacaattgcaaaaatcaatgggtcgttggaccattaataggaaaaggtg : 168698
```

```
                  *    168720         *    168740         *    168760         *    168780         *    168800
Seq1 : gattcggtagtatttatactactaatgacaataattatgtagtaaaaatagagcccaaagctaacggatcattatttaccgaacaggcattttatactag : 168800
Seq2 : gattcggtagtatttatactactaatgacaataattatgtagtaaaaatagagcccaaagctaacggatcattatttaccgaacaggcattttatactag : 168798
Seq3 : gattcggtagtatttatactactaatgacaataattatgtagtaaaaatagagcccaaagctaacggatcattatttaccgaacaggcattttatactag : 168799
Seq4 : gattcggtagtatttatactactaatgacaataattatgtagtaaaaatagagcccaaagctaacggatcattatttaccgaacaggcattttatactag : 168798

                  *    168820         *    168840         *    168860         *    168880         *    168900
Seq1 : agtacttaaaccatccgttatcgaagaatggaaaaaatctcacaatataaagcacgtaggtcttatcacgtgcaaggcatttggtctatacaaatccatt : 168900
Seq2 : agtacttaaaccatccgttatcgaagaatggaaaaaatctcacaatataaagcacgtaggtcttatcacgtgcaaggcatttggtctatacaaatccatt : 168898
Seq3 : agtacttaaaccatccgttatcgaagaatggaaaaaatctcacaatataaagcacgtaggtcttatcacgtgcaaggcatttggtctatacaaatccatt : 168899
Seq4 : agtacttaaaccatccgttatcgaagaatggaaaaaatctcacaatataaagcacgtaggtcttatcacgtgcaaggcatttggtctatacaaatccatt : 168898

                  *    168920         *    168940         *    168960         *    168980         *    169000
Seq1 : aatgtggaatatcgattcttggtaattaatagattaggtgcagatctagatgcggtgatcagagccaataataatagattaccaaaaaggtcggtgatgt : 169000
Seq2 : aatgtggaatatcgattcttggtaattaatagattaggtgcagatctagatgcggtgatcagagccaataataatagattaccaaaaaggtcggtgatgt : 168998
Seq3 : aatgtggaatatcgattcttggtaattaatagattaggtgcagatctagatgcggtgatcagagccaataataatagattaccaaaaaggtcggtgatgt : 168999
Seq4 : aatgtggaatatcgattcttggtaattaatagattaggtgcagatctagatgcggtgatcagagccaataataatagattaccaaaaaggtcggtgatgt : 168998

                  *    169020         *    169040         *    169060         *    169080         *    169100
Seq1 : tgatcggaatcgaaatcttaaataccatacaatttatgcacgagcaaggatattctcacggagatattaaagcgagtaatatagtcttggatcaaataga : 169100
Seq2 : tgatcggaatcgaaatcttaaataccatacaatttatgcacgagcaaggatattctcacggagatattaaagcgagtaatatagtcttggatcaaataga : 169098
Seq3 : tgatcggaatcgaaatcttaaataccatacaatttatgcacgagcaaggatattctcacggagatattaaagcgagtaatatagtcttggatcaaataga : 169099
Seq4 : tgatcggaatcgaaatcttaaataccatacaatttatgcacgagcaaggatattctcacggagatattaaagcgagtaatatagtcttggatcaaataga : 169098

                  *    169120         *    169140         *    169160         *    169180         *    169200
Seq1 : taagaataaattatatctagtggattacggattggtttctaaattcatgtctaatggagaacatgttccatttataagaaatccaaataaaatggataac : 169200
Seq2 : taagaataaattatatctagtggattacggattggtttctaaattcatgtctaatggagaacatgttccatttataagaaatccaaataaaatggataac : 169198
Seq3 : taagaataaattatatctagtggattacggattggtttctaaattcatgtctaatggagaacatgttccatttataagaaatccaaataaaatggataac : 169199
Seq4 : taagaataaattatatctagtggattacggattggtttctaaattcatgtctaatggagaacatgttccatttataagaaatccaaataaaatggataac : 169198

                  *    169220         *    169240         *    169260         *    169280         *    169300
Seq1 : ggtactctagaatttacacctatagattcgcataaaggatacgttgtatctagacgtggagatctagaaacacttggatattgtatgattagatggttgg : 169300
Seq2 : ggtactctagaatttacacctatagattcgcataaaggatacgttgtatctagacgtggagatctagaaacacttggatattgtatgattagatggttgg : 169298
Seq3 : ggtactctagaatttacacctatagattcgcataaaggatacgttgtatctagacgtggagatctagaaacacttggatattgtatgattagatggttgg : 169299
Seq4 : ggtactctagaatttacacctatagattcgcataaaggatacgttgtatctagacgtggagatctagaaacacttggatattgtatgattagatggttgg : 169298

                  *    169320         *    169340         *    169360         *    169380         *    169400
Seq1 : gaggtatcttgccatggactaagatatctgaaacaaagaattgtgcattagtaagtgccacaaaacagaaatatgttaacaatactgcgactttgttaat : 169400
Seq2 : gaggtatcttgccatggactaagatatctgaaacaaagaattgtgcattagtaagtgccacaaaacagaaatatgttaacaatactgcgactttgttaat : 169398
Seq3 : gaggtatcttgccatggactaagatatctgaaacaaagaattgtgcattagtaagtgccacaaaacagaaatatgttaacaatactgcgactttgttaat : 169399
Seq4 : gaggtatcttgccatggactaagatatctgaaacaaagaattgtgcattagtaagtgccacaaaacagaaatatgttaacaatactgcgactttgttaat : 169398
```

```
                *    169420         *    169440         *    169460         *    169480         *    169500
Seq1 : gaccagtttgcaatatgcacctagagaattgctgcaatatattaccatggtaaactctttgacatattttgaggaacccaattatgacgagtttcggcac : 169500
Seq2 : gaccagtttgcaatatgcacctagagaattgctgcaatatattaccatggtaaactctttgacatattttgaggaacccaattatgacgagtttcggcac : 169498
Seq3 : gaccagtttgcaatatgcacctagagaattgctgcaatatattaccatggtaaactctttgacatattttgaggaacccaattatgacgagtttcggcac : 169499
Seq4 : gaccagtttgcaatatgcacctagagaattgctgcaatatattaccatggtaaactctttgacatattttgaggaacccaattatgacgagtttcggcac : 169498

                *    169520         *    169540         *    169560         *    169580         *    169600
Seq1 : atattaatgcagggtgtatattattaagtgtggtgtttggtcgatgtaaaatttttgtcgataaaaattaaaaaataacttaatttattattgatctcgt : 169600
Seq2 : atattaatgcagggtgtatattattaagtgtggtgtttggtcgatgtaaaatttttgtcgataaaaattaaaaaataacttaatttattattgatctcgt : 169598
Seq3 : atattaatgcagggtgtatattattaagtgtggtgtttggtcgatgtaaaatttttgtcgataaaaattaaaaaataacttaatttattattgatctcgt : 169599
Seq4 : atattaatgcagggtgtatattattaagtgtggtgtttggtcgatgtaaaatttttgtcgataaaaattaaaaaataacttaatttattattgatctcgt : 169598

                *    169620         *    169640         *    169660         *    169680         *    169700
Seq1 : gtgtacaaccgaaatcatggcgatgttttacgcacacgctctcggtgggtacgacgagaatcttcatgcctttcctggaatatcatcgactgttgccaat : 169700
Seq2 : gtgtacaaccgaaatcatggcgatgttttacgcacacgctctcggtgggtacgacgagaatcttcatgcctttcctggaatatcatcgactgttgccaat : 169698
Seq3 : gtgtacaaccgaaatcatggcgatgttttacgcacacgctctcggtgggtacgacgagaatcttcatgcctttcctggaatatcatcgactgttgccaat : 169699
Seq4 : gtgtacaaccgaaatcatggcgatgttttacgcacacgctctcggtgggtacgacgagaatcttcatgcctttcctggaatatcatcgactgttgccaat : 169698

                *    169720         *    169740         *    169760         *    169780         *    169800
Seq1 : gatgtcaggaaatattctgttgtgtcagtttataataacaagtatgacattgtaaaagacaaatatatgtggtgttacagtcaggtgaacaagagatata : 169800
Seq2 : gatgtcaggaaatattctgttgtgtcagtttataataacaagtatgacattgtaaaagacaaatatatgtggtgttacagtcaggtgaacaagagatata : 169798
Seq3 : gatgtcaggaaatattctgttgtgtcagtttataataacaagtatgacattgtaaaagacaaatatatgtggtgttacagtcaggtgaacaagagatata : 169799
Seq4 : gatgtcaggaaatattctgttgtgtcagtttataataacaagtatgacattgtaaaagacaaatatatgtggtgttacagtcaggtgaacaagagatata : 169798

                *    169820         *    169840         *    169860         *    169880         *    169900
Seq1 : ttggagcactgctgcctatgtttgagtgcaatgaatatctacaaattggagatccgatccatgatcaagaaggaaatcaaatctctatcatcacatatcg : 169900
Seq2 : ttggagcactgctgcctatgtttgagtgcaatgaatatctacaaattggagatccgatccatgatcaagaaggaaatcaaatctctatcatcacatatcg : 169898
Seq3 : ttggagcactgctgcctatgtttgagtgcaatgaatatctacaaattggagatccgatccatgatcaagaaggaaatcaaatctctatcatcacatatcg : 169899
Seq4 : ttggagcactgctgcctatgtttgagtgcaatgaatatctacaaattggagatccgatccatgatcaagaaggaaatcaaatctctatcatcacatatcg : 169898

                *    169920         *    169940         *    169960         *    169980         *    170000
Seq1 : ccacaaaaactactatgctctaagcggaatcgggtacgagagtctagacttgtgtttggaaggagtagggattcatcatcacgtacttgaaacaggaaac : 170000
Seq2 : ccacaaaaactactatgctctaagcggaatcgggtacgagagtctagacttgtgtttggaaggagtagggattcatcatcacgtacttgaaacaggaaac : 169998
Seq3 : ccacaaaaactactatgctctaagcggaatcgggtacgagagtctagacttgtgtttggaaggagtagggattcatcatcacgtacttgaaacaggaaac : 169999
Seq4 : ccacaaaaactactatgctctaagcggaatcgggtacgagagtctagacttgtgtttggaaggagtagggattcatcatcacgtacttgaaacaggaaac : 169998

                *    170020         *    170040         *    170060         *    170080         *    170100
Seq1 : gctgtatatggaaaagttcaacatgattattctactatcaaagagaaggccaaagaaatgaatgcacttagtccaggacctatcattgattaccacgtct : 170100
Seq2 : gctgtatatggaaaagttcaacatgattattctactatcaaagagaaggccaaagaaatgaatgcacttagtccaggacctatcattgattaccacgtct : 170098
Seq3 : gctgtatatggaaaagttcaacatgattattctactatcaaagagaaggccaaagaaatgaatgcacttagtccaggacctatcattgattaccacgtct : 170099
Seq4 : gctgtatatggaaaagttcaacatgattattctactatcaaagagaaggccaaagaaatgaatgcacttagtccaggacctatcattgattaccacgtct : 170098
```

```
                *    170120          *    170140          *    170160          *    170180          *    170200
Seq1 : ggataggagattgtatctgtcaagttactgctgtggacgtacatggaaaggaaattatgagaatgagattcaaaaagggtgcggtgcttccgatcccaaa : 170200
Seq2 : ggataggagattgtatctgtcaagttactgctgtggacgtacatggaaaggaaattatgagaatgagattcaaaaagggtgcggtgcttccgatcccaaa : 170198
Seq3 : ggataggagattgtatctgtcaagttactgctgtggacgtacatggaaaggaaattatgagaatgagattcaaaaagggtgcggtgcttccgatcccaaa : 170199
Seq4 : ggataggagattgtatctgtcaagttactgctgtggacgtacatggaaaggaaattatgagaatgagattcaaaaagggtgcggtgcttccgatcccaaa : 170198

                *    170220          *    170240          *    170260          *    170280          *    170300
Seq1 : tctggtaaaagttaaacttggggagaatgatacagaaaatctttcttctactatatcggcggcaccatcgaggtaaccacctctctggaagacagcgtga : 170300
Seq2 : tctggtaaaagttaaacttggggagaatgatacagaaaatctttcttctactatatcggcggcaccatcgaggtaaccacctctctggaagacagcgtga : 170298
Seq3 : tctggtaaaagttaaacttggggagaatgatacagaaaatctttcttctactatatcggcggcaccatcgaggtaaccacctctctggaagacagcgtga : 170299
Seq4 : tctggtaaaagttaaacttggggagaatgatacagaaaatctttcttctactatatcggcggcaccatcgaggtaaccacctctctggaagacagcgtga : 170298

                *    170320          *    170340          *    170360          *    170380          *    170400
Seq1 : ataatgtactcatgaaacgtttggaaactatacgccatatgtggtctgtcgtatatgatcattttgatattgtgaatggtaaagaatgctgttatgtgca : 170400
Seq2 : ataatgtactcatgaaacgtttggaaactatacgccatatgtggtctgtcgtatatgatcattttgatattgtgaatggtaaagaatgctgttatgtgca : 170398
Seq3 : ataatgtactcatgaaaagtttggaaactatacgccatatgtggtctgtcgtatatgatcattttgatattgtgaatggtaaagaatgctgttatgtgca : 170399
Seq4 : ataatgtactcatgaaacgtttggaaactatacgccatatgtggtctgtcgtatatgatcattttgatattgtgaatggtaaagaatgctgttatgtgca : 170398

                *    170420          *    170440          *    170460          *    170480          *    170500
Seq1 : tacgcatttgtctaatcaaaatcttataccgagtactgtaaaaacaaatttgtacatgaagactatgggatcatgcattcaaatggattccatggaagct : 170500
Seq2 : tacgcatttgtctaatcaaaatcttataccgagtactgtaaaaacaaatttgtacatgaagactatgggatcatgcattcaaatggattccatggaagct : 170498
Seq3 : tacgcatttgtctaatcaaaatcttataccgagtactgtaaaaacaaatttgtacatgaagactatgggatcatgcattcaaatggattccatggaagct : 170499
Seq4 : tacgcatttgtctaatcaaaatcttataccgagtactgtaaaaacaaatttgtacatgaagactatgggatcatgcattcaaatggattccatggaagct : 170498

                *    170520          *    170540          *    170560          *    170580          *    170600
Seq1 : ctagagtatcttagcgaactgaaggaatcaggtggatggagtcccagaccagaaatgcaggaatttgaatatccagatggagtggaagacactgaatcaa : 170600
Seq2 : ctagagtatcttagcgaactgaaggaatcaggtggatggagtcccagaccagaaatgcaggaatttgaatatccagatggagtggaagacactgaatcaa : 170598
Seq3 : ctagagtatcttagcgaactgaaggaatcaggtggatggagtcccagaccagaaatgcaggaatttgaatatccagatggagtggaagacactgaatcaa : 170599
Seq4 : ctagagtatcttagcgaactgaaggaatcaggtggatggagtcccagaccagaaatgcaggaatttgaatatccagatggagtggaagacactgaatcaa : 170598

                *    170620          *    170640          *    170660          *    170680          *    170700
Seq1 : ttgagagattggtagaggagttcttcaatagatcagaacttcaggctggtgaatcagtcaaatttggtaattctattaatgttaaacatacatctgtttc : 170700
Seq2 : ttgagagattggtagaggagttcttcaatagatcagaacttcaggctggtgaatcagtcaaatttggtaattctattaatgttaaacatacatctgtttc : 170698
Seq3 : ttgagagattggtagaggagttcttcaatagatcagaacttcaggctggtgaatcagtcaaatttggtaattctattaatgttaaacatacatctgtttc : 170699
Seq4 : ttgagagattggtagaggagttcttcaatagatcagaacttcaggctggtgaatcagtcaaatttggtaattctattaatgttaaacatacatctgtttc : 170698

                *    170720          *    170740          *    170760          *    170780          *    170800
Seq1 : agctaagcaactaagaacacgtatacggcagcagcttcctttatactctcatcttttaccaacacaaagggtggatatttgttcattggagttgataata : 170800
Seq2 : agctaagcaactaagaacacgtatacggcagcagcttcctttatactctcatcttttaccaacacaaagggtggatatttgttcattggagttgataata : 170798
Seq3 : agctaagcaactaagaacacgtatacggcagcagcttcctttatactctcatcttttaccaacacaaagggtggatatttgttcattggagttgataata : 170799
Seq4 : agctaagcaactaagaacacgtatacggcagcagcttcctttatactctcatcttttaccaacacaaagggtggatatttgttcattggagttgataata : 170798
```

```
                  *    170820         *    170840         *    170860         *    170880         *    170900
Seq1 : atacacacaaagtaattggattcacggtgggtcatgactacctcagactggtagagaatgatatagaaaagcatatcaaaagacttcgtgttgtgcattt : 170900
Seq2 : atacacacaaagtaattggattcacggtgggtcatgactacctcagactggtagagaatgatatagaaaagcatatcaaaagacttcgtgttgtgcattt : 170898
Seq3 : atacacacaaagtaattggattcacggtgggtcatgactacctcagactggtagagaatgatatagaaaagcatatcaaaagacttcgtgttgtgcattt : 170899
Seq4 : atacacacaaagtaattggattcacggtgggtcatgactacctcagactggtagagaatgatatagaaaagcatatcaaaagacttcgtgttgtgcattt : 170898

                  *    170920         *    170940         *    170960         *    170980         *    171000
Seq1 : ctgtgagaagaaagaggacatcaagtacacgtgtcgattcatcaaggtatataaacctggggatgaggctacctcgacatacgtgtgcgctatcaaagtg : 171000
Seq2 : ctgtgagaagaaagaggacatcaagtacacgtgtcgattcatcaaggtatataaacctggggatgaggctacctcgacatacgtgtgcgctatcaaagtg : 170998
Seq3 : ctgtgagaagaaagaggacatcaagtacacgtgtcgattcatcaaggtatataaacctggggatgaggctacctcgacatacgtgtgcgctatcaaagtg : 170999
Seq4 : ctgtgagaagaaagaggacatcaagtacacgtgtcgattcatcaaggtatataaacctggggatgaggctacctcgacatacgtgtgcgctatcaaagtg : 170998

                  *    171020         *    171040         *    171060         *    171080         *    171100
Seq1 : gaaagatgctgttgtgctgtgtttgcagattggccagaatcatggtatatggatactaatggtatcaagaagtattctccagatgaatgggtgtcacata : 171100
Seq2 : gaaagatgctgttgtgctgtgtttgcagattggccagaatcatggtatatggatactaatggtatcaagaagtattctccagatgaatgggtgtcacata : 171098
Seq3 : gaaagatgctgttgtgctgtgtttgcagattggccagaatcatggtatatggatactaatggtatcaagaagtattctccagatgaatgggtgtcacata : 171099
Seq4 : gaaagatgctgttgtgctgtgtttgcagattggccagaatcatggtatatggatactaatggtatcaagaagtattctccagatgaatgggtgtcacata : 171098

                  *    171120         *    171140         *    171160         *    171180         *    171200
Seq1 : taaaattttaattaatgtaatagagaacaaataataaggttgtaatatcatatagacaataactaacaattaattagtaactgttatctcttttttaact : 171200
Seq2 : taaaattttaattaatgtaatagagaacaaataataaggttgtaatatcatatagacaataactaacaattaattagtaactgttatctcttttttaact : 171198
Seq3 : taaaattttaattaatgtaatagagaacaaataataaggttgtaatatcatatagacaataactaacaattaattagtaactgttatctcttttttaact : 171199
Seq4 : taaaattttaattaatgtaatagagaacaaataataaggttgtaatatcatatagacaataactaacaattaattagtaactgttatctcttttttaact : 171198

                  *    171220         *    171240         *    171260         *    171280         *    171300
Seq1 : aaccaactaactatatacctattaatacatcgtaattatagttcttaacatctattaatcattaattcgcttctttaattttttataaactaacattgtt : 171300
Seq2 : aaccaactaactatatacctattaatacatcgtaattatagttcttaacatctattaatcattaattcgcttctttaattttttataaactaacattgtt : 171298
Seq3 : aaccaactaactatatacctattaatacatcgtaattatagttcttaacatctattaatcattaattcgcttctttaattttttataaactaacattgtt : 171299
Seq4 : aaccaactaactatatacctattaatacatcgtaattatagttcttaacatctattaatcattaattcgcttctttaattttttataaactaacattgtt : 171298

                  *    171320         *    171340         *    171360         *    171380         *    171400
Seq1 : aattgaaaagggataacatgttacagaatataaattatatatggatttttttaaaaaggaaatacttgactggagtatatatttatctcttcattatata : 171400
Seq2 : aattgaaaagggataacatgttacagaatataaattatatatggatttttttaaaaaggaaatacttgactggagtatatatttatctcttcattatata : 171398
Seq3 : aattgaaaagggataacatgttacagaatataaattatatatggatttttttaaaaaggaaatacttgactggagtatatatttatctcttcattatata : 171399
Seq4 : aattgaaaagggataacatgttacagaatataaattatatatggatttttttaaaaaggaaatacttgactggagtatatatttatctcttcattatata : 171398

                  *    171420         *    171440         *    171460         *    171480         *    171500
Seq1 : gcacgcgtgttttccaatttttccacatcccatataatacaggattataatctcgttcgaacatacgagaaagtggataaaacaatagttgattttttat : 171500
Seq2 : gcacgcgtgttttccaatttttccacatcccatataatacaggattataatctcgttcgaacatacgagaaagtggataaaacaatagttgattttttat : 171498
Seq3 : gcacgcgtgttttccaatttttccacatcccatataatacaggattataatctcgttcgaacatacgagaaagtggataaaacaatagttgattttttat : 171499
Seq4 : gcacgcgtgttttccaatttttccacatcccatataatacaggattataatctcgttcgaacatacgagaaagtggataaaacaatagttgattttttat : 171498
```

```
               *    171520          *    171540          *    171560          *    171580          *    171600
Seq1 : ctaggttgccaaatttattccatatttagaatatggggaaaatattctacatatttattctatggatgatgctaatacgaatattataattttttttct : 171600
Seq2 : ctaggttgccaaatttattccatatttagaatatggggaaaatattctacatatttattctatggatgatgctaatacgaatattataattttttttct : 171598
Seq3 : ctaggttgccaaatttattccatatttagaatatggggaaaatattctacatatttattctatggatgatgctaatacgaatattataattttttttct : 171599
Seq4 : ctaggttgccaaatttattccatatttagaatatggggaaaatattctacatatttattctatggatgatgctaatacgaatattataattttttttct : 171598

               *    171620          *    171640          *    171660          *    171680          *    171700
Seq1 : agatagagtattaaatattaataagaacgggtcatttatacacaatctcaggttatcatcatccattaatataaaagaatatgtatatcaattagttaat : 171700
Seq2 : agatagagtattaaatattaataagaacgggtcatttatacacaatctcaggttatcatcatccattaatataaaagaatatgtatatcaattagttaat : 171698
Seq3 : agatagagtattaaatattaataagaacgggtcatttatacacaatctcaggttatcatcatccattaatataaaagaatatgtatatcaattagttaat : 171699
Seq4 : agatagagtattaaatattaataagaacgggtcatttatacacaatctcaggttatcatcatccattaatataaaagaatatgtatatcaattagttaat : 171698

               *    171720          *    171740          *    171760          *    171780          *    171800
Seq1 : aatgatcatccagataataggataagactaatgcttgaaaatggacgtagaacaagacatttttttgtcctatatatcagatacagttaatatctatatat : 171800
Seq2 : aatgatcatccagataataggataagactaatgcttgaaaatggacgtagaacaagacatttttttgtcctatatatcagatacagttaatatctatatat : 171798
Seq3 : aatgatcatccagataataggataagactaatgcttgaaaatggacgtagaacaagacatttttttgtcctatatatcagatacagttaatatctatatat : 171799
Seq4 : aatgatcatccagataataggataagactaatgcttgaaaatggacgtagaacaagacatttttttgtcctatatatcagatacagttaatatctatatat : 171798

               *    171820          *    171840          *    171860          *    171880          *    171900
Seq1 : gtattttaataaatcatggatttatatagatgccgaagacagttacggttgtacattattacatagatgtatatatcactataagaaatcagaatcaga : 171900
Seq2 : gtattttaataaatcatggatttatatagatgccgaagacagttacggttgtacattattacatagatgtatatatcactataagaaatcagaatcaga : 171898
Seq3 : gtattttaataaatcatggatttatatagatgccgaagacagttacggttgtacattattacatagatgtatatatcactataagaaatcagaatcaga : 171899
Seq4 : gtattttaataaatcatggatttatatagatgccgaagacagttacggttgtacattattacatagatgtatatatcactataagaaatcagaatcaga : 171898

               *    171920          *    171940          *    171960          *    171980          *    172000
Seq1 : atcatacaatgaattaattaagatattgttaaataatggatcagatgtagataaaaaagatacgtacggaaacacaccttttatcctattatgtaaacac : 172000
Seq2 : atcatacaatgaattaattaagatattgttaaataatggatcagatgtagataaaaaagatacgtacggaaacacaccttttatcctattatgtaaacac : 171998
Seq3 : atcatacaatgaattaattaagatattgttaaataatggatcagatgtagataaaaaagatacgtacggaaacacaccttttatcctattatgtaaacac : 171999
Seq4 : atcatacaatgaattaattaagatattgttaaataatggatcagatgtagataaaaaagatacgtacggaaacacaccttttatcctattatgtaaacac : 171998

               *    172020          *    172040          *    172060          *    172080          *    172100
Seq1 : gatatcaacaacgtggaattgtttgagatatgtttagagaatgctaatatagactctgtagactttaatagatatacacctcttcattatgtctcatgtc : 172100
Seq2 : gatatcaacaacgtggaattgtttgagatatgtttagagaatgctaatatagactctgtagactttaatagatatacacctcttcattatgtctcatgtc : 172098
Seq3 : gatatcaacaacgtggaattgtttgagatatgtttagagaatgctaatatagactctgtagactttaatagatatacacctcttcattatgtctcatgtc : 172099
Seq4 : gatatcaacaacgtggaattgtttgagatatgtttagagaatgctaatatagactctgtagactttaatagatatacacctcttcattatgtctcatgtc : 172098

               *    172120          *    172140          *    172160          *    172180          *    172200
Seq1 : gtaataaatatgatttttgtaaagttattaatttctaaaggagcaaatgttaatgcgcgtaatagattcggaactactccatttttattgtggaattataca : 172200
Seq2 : gtaataaatatgatttttgtaaagttattaatttctaaaggagcaaatgttaatgcgcgtaatagattcggaactactccatttttattgtggaattataca : 172198
Seq3 : gtaataaatatgatttttgtaaagttattaatttctaaaggagcaaatgttaatgcgcgtaatagattcggaactactccatttttattgtggaattataca : 172199
Seq4 : gtaataaatatgatttttgtaaagttattaatttctaaaggagcaaatgttaatgcgcgtaatagattcggaactactccatttttattgtggaattataca : 172198
```

```
              *    172220         *    172240         *    172260         *    172280         *    172300
Seq1 : cggtatctcgcttataaaactatatttggaatcagacacagagttagaaatagataatgaacatatagttcgtcatttaataatttttgatgctgttgaa : 172300
Seq2 : cggtatctcgcttataaaactatatttggaatcagacacagagttagaaatagataatgaacatatagttcgtcatttaataatttttgatgctgttgaa : 172298
Seq3 : cggtatctcgcttataaaactatatttggaatcagacacagagttagaaatagataatgaacatatagttcgtcatttaataatttttgatgctgttgaa : 172299
Seq4 : cggtatctcgcttataaaactatatttggaatcagacacagagttagaaatagataatgaacatatagttcgtcatttaataatttttgatgctgttgaa : 172298

              *    172320         *    172340         *    172360         *    172380         *    172400
Seq1 : tctttagattatctattatccagaggagttattgatattaactatcgtactatatacaacgaaacatctatttacgacgctgtcagttataatgcgtata : 172400
Seq2 : tctttagattatctattatccagaggagttattgatattaactatcgtactatatacaacgaaacatctatttacgacgctgtcagttataatgcgtata : 172398
Seq3 : tctttagattatctattatccagaggagttattgatattaactatcgtactatatacaacgaaacatctatttacgacgctgtcagttataatgcgtata : 172399
Seq4 : tctttagattatctattatccagaggagttattgatattaactatcgtactatatacaacgaaacatctatttacgacgctgtcagttataatgcgtata : 172398

              *    172420         *    172440         *    172460         *    172480         *    172500
Seq1 : atacgttggtctatctattaaacagaaatggtgattttgagacgattactactagtggatgtacatgtatttcggaagcagtcgcaaacaacaacaaaat : 172500
Seq2 : atacgttggtctatctattaaacagaaatggtgattttgagacgattactactagtggatgtacatgtatttcggaagcagtcgcaaacaacaacaaaat : 172498
Seq3 : atacgttggtctatctattaaacagaaatggtgattttgagacgattactactagtggatgtacatgtatttcggaagcagtcgcaaacaacaacaaaat : 172499
Seq4 : atacgttggtctatctattaaacagaaatggtgattttgagacgattactactagtggatgtacatgtatttcggaagcagtcgcaaacaacaacaaaat : 172498

              *    172520         *    172540         *    172560         *    172580         *    172600
Seq1 : aataatggaagtactattgtctaaacgaccatctttgaaaattatgatacagtctatgatagcaattactaaacataaacagcataatgcagatttattg : 172600
Seq2 : aataatggaagtactattgtctaaacgaccatctttgaaaattatgatacagtctatgatagcaattactaaacataaacagcataatgcagatttattg : 172598
Seq3 : aataatggaagtactattgtctaaacgaccatctttgaaaattatgatacagtctatgatagcaattactaaacataaacagcataatgcagatttattg : 172599
Seq4 : aataatggaagtactattgtctaaacgaccatctttgaaaattatgatacagtctatgatagcaattactaaacataaacagcataatgcagatttattg : 172598

              *    172620         *    172640         *    172660         *    172680         *    172700
Seq1 : aaaatgtgtataaaatatactgcgtgtatgaccgattatgatactcttatagatgtacagtcgctacagcaatataaatggtatattttaaaatgtttcg : 172700
Seq2 : aaaatgtgtataaaatatactgcgtgtatgaccgattatgatactcttatagatgtacagtcgctacagcaatataaatggtatattttaaaatgtttcg : 172698
Seq3 : aaaatgtgtataaaatatactgcgtgtatgaccgattatgatactcttatagatgtacagtcgctacagcaatataaatggtatattttaaaatgtttcg : 172699
Seq4 : aaaatgtgtataaaatatactgcgtgtatgaccgattatgatactcttatagatgtacagtcgctacagcaatataaatggtatattttaaaatgtttcg : 172698

              *    172720         *    172740         *    172760         *    172780         *    172800
Seq1 : atgaaatagatatcatgaagagatgttatataaaaaataaaactgtattccaattagtttttttgtatcaaagacattaatactttaatgagatacggtaa : 172800
Seq2 : atgaaatagatatcatgaagagatgttatataaaaaataaaactgtattccaattagtttttttgtatcaaagacattaatactttaatgagatacggtaa : 172798
Seq3 : atgaaatagatatcatgaagagatgttatataaaaaataaaactgtattccaattagtttttttgtatcaaagacattaatactttaatgagatacggtaa : 172799
Seq4 : atgaaatagatatcatgaagagatgttatataaaaaataaaactgtattccaattagtttttttgtatcaaagacattaatactttaatgagatacggtaa : 172798

              *    172820         *    172840         *    172860         *    172880         *    172900
Seq1 : acatccttctttcgtgaaatgcactagtctcgacgtatacggaagtcgtgtacgtaatatcatagcatctattagatatcgtcagagattaattagtcta : 172900
Seq2 : acatccttctttcgtgaaatgcactagtctcgacgtatacggaagtcgtgtacgtaatatcatagcatctattagatatcgtcagagattaattagtcta : 172898
Seq3 : acatccttctttcgtgaaatgcactagtctcgacgtatacggaagtcgtgtacgtaatatcatagcatctattagatatcgtcagagattaattagtcta : 172899
Seq4 : acatccttctttcgtgaaatgcactagtctcgacgtatacggaagtcgtgtacgtaatatcatagcatctattagatatcgtcagagattaattagtcta : 172898
```

```
           *    172920         *    172940         *    172960         *    172980         *    173000
Seq1 : ttatccaagaagctggatcctggagataaatggtcgtgttttcctaacgaaataaaatataaaatattggaaaactttaacgataacgaactatccacat : 173000
Seq2 : ttatccaagaagctggatcctggagataaatggtcgtgttttcctaacgaaataaaatataaaatattggaaaactttaacgataacgaactatccacat : 172998
Seq3 : ttatccaagaagctggatcctggagataaatggtcgtgttttcctaacgaaataaaatataaaatattggaaaactttaacgataacgaactatccacat : 172999
Seq4 : ttatccaagaagctggatcctggagataaatggtcgtgttttcctaacgaaataaaatataaaatattggaaaactttaacgataacgaactatccacat : 172998

           *    173020         *    173040         *    173060         *    173080         *    173100
Seq1 : atctaaaaatcttataaacactattaaaatataaaatctaagtaggataaaatcacactacatcattgtttccttttagtgctcgacagtgtatactatt : 173100
Seq2 : atctaaaaatcttataaacactattaaaatataaaatctaagtaggataaaatcacactacatcattgtttccttttagtgctcgacagtgtatactatt : 173098
Seq3 : atctaaaaatcttataaacactattaaaatataaaatctaagtaggataaaatcacactacatcattgtttccttttagtgctcgacagtgtatactatt : 173099
Seq4 : atctaaaaatcttataaacactattaaaatataaaatctaagtaggataaaatcacactacatcattgtttccttttagtgctcgacagtgtatactatt : 173098

           *    173120         *    173140         *    173160         *    173180         *    173200
Seq1 : tttaacactcataaataaaaatgaaaacgatttccgttgttacgttgttatgcgtactacctgctgttgtttattcaacatgtactgtacccactatgaa : 173200
Seq2 : tttaacactcataaataaaaatgaaaacgatttccgttgttacgttgttatgcgtactacctgctgttgtttattcaacatgtactgtacccactatgaa : 173198
Seq3 : tttaacactcataaataaaaatgaaaacgatttccgttgttacgttgttatgcgtactacctgctgttgtttattcaacatgtactgtacccactatgaa : 173199
Seq4 : tttaacactcataaataaaaatgaaaacgatttccgttgttacgttgttatgcgtactacctgctgttgtttattcaacatgtactgtacccactatgaa : 173198

           *    173220         *    173240         *    173260         *    173280         *    173300
Seq1 : taacgctaaattaacgtctaccgaaacatcgtttaatgataaacagaaagttacgtttacatgtgatcagggatatcattcttcggatccaaatgctgtc : 173300
Seq2 : taacgctaaattaacgtctaccgaaacatcgtttaatgataaacagaaagttacgtttacatgtgatcagggatatcattcttcggatccaaatgctgtc : 173298
Seq3 : taacgctaaattaacgtctaccgaaacatcgtttaatgataaacagaaagttacgtttacatgtgatcagggatatcattcttcggatccaaatgctgtc : 173299
Seq4 : taacgctaaattaacgtctaccgaaacatcgtttaatgataaacagaaagttacgtttacatgtgatcagggatatcattcttcggatccaaatgctgtc : 173298

           *    173320         *    173340         *    173360         *    173380         *    173400
Seq1 : tgcgaaacagataaatggaaatacgaaaatccatgcaaaaaaatgtgcacagtttctgattacatctctgaattatataataaaccgctatacgaagtga : 173400
Seq2 : tgcgaaacagataaatggaaatacgaaaatccatgcaaaaaaatgtgcacagtttctgattacatctctgaattatataataaaccgctatacgaagtga : 173398
Seq3 : tgcgaaacagataaatggaaatacgaaaatccatgcaaaaaaatgtgcacagtttctgattacatctctgaattatataataaaccgctatacgaagtga : 173399
Seq4 : tgcgaaacagataaatggaaatacgaaaatccatgcaaaaaaatgtgcacagtttctgattacatctctgaattatataataaaccgctatacgaagtga : 173398

           *    173420         *    173440         *    173460         *    173480         *    173500
Seq1 : attccaccatgacactaagttgcaacggcgaaacaaaatatttcgttgcgaagaaaaaaaatggaaatacttcttggaatgatactgttacgtgtcctaa : 173500
Seq2 : attccaccatgacactaagttgcaacggcgaaacaaaatatttcgttgcgaagaaaaaaaatggaaatacttcttggaatgatactgttacgtgtcctaa : 173498
Seq3 : attccaccatgacactaagttgcaacggcgaaacaaaatatttcgttgcgaagaaaaaaaatggaaatacttcttggaatgatactgttacgtgtcctaa : 173499
Seq4 : attccaccatgacactaagttgcaacggcgaaacaaaatatttcgttgcgaagaaaaaaaatggaaatacttcttggaatgatactgttacgtgtcctaa : 173498

           *    173520         *    173540         *    173560         *    173580         *    173600
Seq1 : tgcggaatgtcaacctcttcaattagaacacggatcgtgtcaaccagttaaagaaaaatactcatttggggaatatatgactatcaactgtgatgttgga : 173600
Seq2 : tgcggaatgtcaacctcttcaattagaacacggatcgtgtcaaccagttaaagaaaaatactcatttggggaatatatgactatcaactgtgatgttgga : 173598
Seq3 : tgcggaatgtcaacctcttcaattagaacacggatcgtgtcaaccagttaaagaaaaatactcatttggggaatatatgactatcaactgtgatgttgga : 173599
Seq4 : tgcggaatgtcaacctcttcaattagaacacggatcgtgtcaaccagttaaagaaaaatactcatttggggaatatatgactatcaactgtgatgttgga : 173598
```

```
                 *    173620         *    173640         *    173660         *    173680         *    173700
Seq1 : tatgaggttattggtgcttcgtacataagttgtacagctaattcttggaatgttattccatcatgtcaacaaaaatgtgatatgccgtctctatctaatg : 173700
Seq2 : tatgaggttattggtgcttcgtacataagttgtacagctaattcttggaatgttattccatcatgtcaacaaaaatgtgatatgccgtctctatctaatg : 173698
Seq3 : tatgaggttattggtgcttcgtacataagttgtacagctaattcttggaatgttattccatcatgtcaacaaaaatgtgatatgccgtctctatctaatg : 173699
Seq4 : tatgaggttattggtgcttcgtacataagttgtacagctaattcttggaatgttattccatcatgtcaacaaaaatgtgatatgccgtctctatctaatg : 173698

                 *    173720         *    173740         *    173760         *    173780         *    173800
Seq1 : gattaatttccggatctacattttctatcggtggcgttatacatcttagttgtaaaagtggttttacactaacggggtctccatcatccacatgtatcga : 173800
Seq2 : gattaatttccggatctacattttctatcggtggcgttatacatcttagttgtaaaagtggttttacactaacggggtctccatcatccacatgtatcga : 173798
Seq3 : gattaatttccggatctacattttctatcggtggcgttatacatcttagttgtaaaagtggttttacactaacggggtctccatcatccacatgtatcga : 173799
Seq4 : gattaatttccggatctacattttctatcggtggcgttatacatcttagttgtaaaagtggttttacactaacggggtctccatcatccacatgtatcga : 173798

                 *    173820         *    173840         *    173860         *    173880         *    173900
Seq1 : cggtaaatggaatcccgtactcccaatatgtgtacgaactaacgaagaatttgatccagtggatgatggtcccgacgatgagacagatttgagcaaactc : 173900
Seq2 : cggtaaatggaatcccgtactcccaatatgtgtacgaactaacgaagaatttgatccagtggatgatggtcccgacgatgagacagatttgagcaaactc : 173898
Seq3 : cggtaaatggaatcccgtactcccaatatgtgtacgaactaacgaagaatttgatccagtggatgatggtcccgacgatgagacagatttgagcaaactc : 173899
Seq4 : cggtaaatggaatcccgtactcccaatatgtgtacgaactaacgaagaatttgatccagtggatgatggtcccgacgatgagacagatttgagcaaactc : 173898

                 *    173920         *    173940         *    173960         *    173980         *    174000
Seq1 : tcgaaagacgttgtacaatatgaacaagaaatagaatcgttagaagcaacttatcatataatcatagtggcgttaacaattatgggcgtcatatttttaa : 174000
Seq2 : tcgaaagacgttgtacaatatgaacaagaaatagaatcgttagaagcaacttatcatataatcatagtggcgttaacaattatgggcgtcatatttttaa : 173998
Seq3 : tcgaaagacgttgtacaatatgaacaagaaatagaatcgttagaagcaacttatcatataatcatagtggcgttaacaattatgggcgtcatatttttaa : 173999
Seq4 : tcgaaagacgttgtacaatatgaacaagaaatagaatcgttagaagcaacttatcatataatcatagtggcgttaacaattatgggcgtcatatttttaa : 173998

                 *    174020         *    174040         *    174060         *    174080         *    174100
Seq1 : tctccgttatagtattagtttgttcctgtgacaaaaataatgaccaatataagttccataaattgctaccgtaaatataaatccgttaaaataatgaata : 174100
Seq2 : tctccgttatagtattagtttgttcctgtgacaaaaataatgaccaatataagttccataaattgctaccgtaaatataaatccgttaaaataatgaata : 174098
Seq3 : tctccgttatagtattagtttgttcctgtgacaaaaataatgaccaatataagttccataaattgctaccgtaaatataaatccgttaaaataatgaata : 174099
Seq4 : tctccgttatagtattagtttgttcctgtgacaaaaataatgaccaatataagttccataaattgctaccgtaaatataaatccgttaaaataatgaata : 174098

                 *    174120         *    174140         *    174160         *    174180         *    174200
Seq1 : attaataacgaacaagtatcaaaagattaaagaattatagctagaatcaattgagatgtcttcttcagtggatgttgatatctacgatgccgttagagca : 174200
Seq2 : attaataacgaacaagtatcaaaagattaaagaattatagctagaatcaattgagatgtcttcttcagtggatgttgatatctacgatgccgttagagca : 174198
Seq3 : attaataacgaacaagtatcaaaagattaaagaattatagctagaatcaattgagatgtcttcttcagtggatgttgatatctacgatgccgttagagca : 174199
Seq4 : attaataacgaacaagtatcaaaagattaaagaattatagctagaatcaattgagatgtcttcttcagtggatgttgatatctacgatgccgttagagca : 174198

                 *    174220         *    174240         *    174260         *    174280         *    174300
Seq1 : tttttactcaggcactattataacaagagatttattgtgtatggaagaagtaacgccatattacataatatatacaggctatttacaagatgcgccgtta : 174300
Seq2 : tttttactcaggcactattataacaagagatttattgtgtatggaagaagtaacgccatattacataatatatacaggctatttacaagatgcgccgtta : 174298
Seq3 : tttttactcaggcactattataacaagagatttattgtgtatggaagaagtaacgccatattacataatatatacaggctatttacaagatgcgccgtta : 174299
Seq4 : tttttactcaggcactattataacaagagatttattgtgtatggaagaagtaacgccatattacataatatatacaggctatttacaagatgcgccgtta : 174298
```

```
              *    174320         *    174340         *    174360         *    174380         *    174400
Seq1 : taccgttcgatgatatagtacgtactatgccaaatgaatcacgtgttaaacaatgggtgatggatacacttaatggtataatgatgaatgaacgcgatgt : 174400
Seq2 : taccgttcgatgatatagtacgtactatgccaaatgaatcacgtgttaaacaatgggtgatggatacacttaatggtataatgatgaatgaacgcgatgt : 174398
Seq3 : taccgttcgatgatatagtacgtactatgccaaatgaatcacgtgttaaacaatgggtgatggatacacttaatggtataatgatgaatgaacgcgatgt : 174399
Seq4 : taccgttcgatgatatagtacgtactatgccaaatgaatcacgtgttaaacaatgggtgatggatacacttaatggtataatgatgaatgaacgcgatgt : 174398

              *    174420         *    174440         *    174460         *    174480         *    174500
Seq1 : ttctgtaagcgttggcaccggaatactattcatggaaatgtttttcgattacaataaaaatagtatcaacaatcaactaatgtatgatataattaatagc : 174500
Seq2 : ttctgtaagcgttggcaccggaatactattcatggaaatgtttttcgattacaataaaaatagtatcaacaatcaactaatgtatgatataattaatagc : 174498
Seq3 : ttctgtaagcgttggcaccggaatactattcatggaaatgtttttcgattacaataaaaatagtatcaacaatcaactaatgtatgatataattaatagc : 174499
Seq4 : ttctgtaagcgttggcaccggaatactattcatggaaatgtttttcgattacaataaaaatagtatcaacaatcaactaatgtatgatataattaatagc : 174498

              *    174520         *    174540         *    174560         *    174580         *    174600
Seq1 : gtatctataattctagctaatgagagatatagaagcgcttttaacgacgatggtatatacatccgtagaaatatgattaacaagttgtacggatacgcat : 174600
Seq2 : gtatctataattctagctaatgagagatatagaagcgcttttaacgacgatggtatatacatccgtagaaatatgattaacaagttgtacggatacgcat : 174598
Seq3 : gtatctataattctagctaatgagagatatagaagcgcttttaacgacgatggtatatacatccgtagaaatatgattaacaagttgtacggatacgcat : 174599
Seq4 : gtatctataattctagctaatgagagatatagaagcgcttttaacgacgatggtatatacatccgtagaaatatgattaacaagttgtacggatacgcat : 174598

              *    174620         *    174640         *    174660         *    174680         *    174700
Seq1 : ctctaactactattggcacgatcgctggaggtgtttgttattatctgttgatgcatctagttagtttgtataaataattatttcaatatactagttaaaa : 174700
Seq2 : ctctaactactattggcacgatcgctggaggtgtttgttattatctgttgatgcatctagttagtttgtataaataattatttcaatatactagttaaaa : 174698
Seq3 : ctctaactactattggcacgatcgctggaggtgtttgttattatctgttgatgcatctagttagtttgtataaataattatttcaatatactagttaaaa : 174699
Seq4 : ctctaactactattggcacgatcgctggaggtgtttgttattatctgttgatgcatctagttagtttgtataaataattatttcaatatactagttaaaa : 174698

              *    174720         *    174740         *    174760         *    174780         *    174800
Seq1 : ttttaagattttaaatgtataaaaaactaataacgtttttatttgtaataggtgcattagcatcctattcgaataatgagtacactccgtttaataaact : 174800
Seq2 : ttttaagattttaaatgtataaaaaactaataacgtttttatttgtaataggtgcattagcatcctattcgaataatgagtacactccgtttaataaact : 174798
Seq3 : ttttaagattttaaatgtataaaaaactaataacgtttttatttgtaataggtgcattagcatcctattcgaataatgagtacactccgtttaataaact : 174799
Seq4 : ttttaagattttaaatgtataaaaaactaataacgtttttatttgtaataggtgcattagcatcctattcgaataatgagtacactccgtttaataaact : 174798

              *    174820         *    174840         *    174860         *    174880         *    174900
Seq1 : gagtgtaaaactctatatagatggagtagataatatagaaaattcatatactgatgataataatgaattggtgttaaattttaaagagtacacaatttct : 174900
Seq2 : gagtgtaaaactctatatagatggagtagataatatagaaaattcatatactgatgataataatgaattggtgttaaattttaaagagtacacaatttct : 174898
Seq3 : gagtgtaaaactctatatagatggagtagataatatagaaaattcatatactgatgataataatgaattggtgttaaattttaaagagtacacaatttct : 174899
Seq4 : gagtgtaaaactctatatagatggagtagataatatagaaaattcatatactgatgataataatgaattggtgttaaattttaaagagtacacaatttct : 174898

              *    174920         *    174940         *    174960         *    174980         *    175000
Seq1 : attattacagagtcatgcgacgtcggatttgattccatagatatagatgttataaacgactataaaattattgatatgtataccattgactcgtctacta : 175000
Seq2 : attattacagagtcatgcgacgtcggatttgattccatagatatagatgttataaacgactataaaattattgatatgtataccattgactcgtctacta : 174998
Seq3 : attattacagagtcatgcgacgtcggatttgattccatagatatagatgttataaacgactataaaattattgatatgtataccattgactcgtctacta : 174999
Seq4 : attattacagagtcatgcgacgtcggatttgattccatagatatagatgttataaacgactataaaattattgatatgtataccattgactcgtctacta : 174998
```

```
                *    175020         *    175040         *    175060         *    175080         *    175100
Seq1 : ttcaacgcagaggtcacacgtgtagaatatctaccaaattatcatgccattatgataagtacccttatattcacaaatatgatggtgatgagcgacaata : 175100
Seq2 : ttcaacgcagaggtcacacgtgtagaatatctaccaaattatcatgccattatgataagtacccttatattcacaaatatgatggtgatgagcgacaata : 175098
Seq3 : ttcaacgcagaggtcacacgtgtagaatatctaccaaattatcatgccattatgataagtacccttatattcacaaatatgatggtgatgagcgacaata : 175099
Seq4 : ttcaacgcagaggtcacacgtgtagaatatctaccaaattatcatgccattatgataagtacccttatattcacaaatatgatggtgatgagcgacaata : 175098

                *    175120         *    175140         *    175160         *    175180         *    175200
Seq1 : ttctattactgcagagggaaaatgctataaaggaataaaatatgaaataagtatgatcaacgatgatactctattgagaaaacatactcttaaaattgga : 175200
Seq2 : ttctattactgcagagggaaaatgctataaaggaataaaatatgaaataagtatgatcaacgatgatactctattgagaaaacatactcttaaaattgga : 175198
Seq3 : ttctattactgcagagggaaaatgctataaaggaataaaatatgaaataagtatgatcaacgatgatactctattgagaaaacatactcttaaaattgga : 175199
Seq4 : ttctattactgcagagggaaaatgctataaaggaataaaatatgaaataagtatgatcaacgatgatactctattgagaaaacatactcttaaaattgga : 175198

                *    175220         *    175240         *    175260         *    175280         *    175300
Seq1 : tctacttatatatttgatcgtcatggacatagtaatacatattattcaaaatatgatttttaaaaatttaaaatatattatcacttcagtgacagtagtc : 175300
Seq2 : tctacttatatatttgatcgtcatggacatagtaatacatattattcaaaatatgatttttaaaaatttaaaatatattatcacttcagtgacagtagtc : 175298
Seq3 : tctacttatatatttgatcgtcatggacatagtaatacatattattcaaaatatgatttttaaaaatttaaaatatattatcacttcagtgacagtagtc : 175299
Seq4 : tctacttatatatttgatcgtcatggacatagtaatacatattattcaaaatatgatttttaaaaatttaaaatatattatcacttcagtgacagtagtc : 175298

                *    175320         *    175340         *    175360         *    175380         *    175400
Seq1 : aaataacaaacaacaccatgagatatattataattctcgcagttttgttcattaatagtatacacgctaaaataactagttataagtttgaatccgtcaa : 175400
Seq2 : aaataacaaacaacaccatgagatatattataattctcgcagttttgttcattaatagtatacacgctaaaataactagttataagtttgaatccgtcaa : 175398
Seq3 : aaataacaaacaacaccatgagatatattataattctcgcagttttgttcattaatagtatacacgctaaaataactagttataagtttgaatccgtcaa : 175399
Seq4 : aaataacaaacaacaccatgagatatattataattctcgcagttttgttcattaatagtatacacgctaaaataactagttataagtttgaatccgtcaa : 175398

                *    175420         *    175440         *    175460         *    175480         *    175500
Seq1 : ttttgattccaaaattgaatggactggggatggtctatacaatatatcccttaaaaattatggcatcaagacgtggcaaacaatgtatacaaatgtacca : 175500
Seq2 : ttttgattccaaaattgaatggactggggatggtctatacaatatatcccttaaaaattatggcatcaagacgtggcaaacaatgtatacaaatgtacca : 175498
Seq3 : ttttgattccaaaattgaatggactggggatggtctatacaatatatcccttaaaaattatggcatcaagacgtggcaaacaatgtatacaaatgtacca : 175499
Seq4 : ttttgattccaaaattgaatggactggggatggtctatacaatatatcccttaaaaattatggcatcaagacgtggcaaacaatgtatacaaatgtacca : 175498

                *    175520         *    175540         *    175560         *    175580         *    175600
Seq1 : gaaggaacatacgacatatccgcatttccaaagaatgatttcgtatctttctgggttaaatttgaacaaggcgattataaagtggaagagtattgtacgg : 175600
Seq2 : gaaggaacatacgacatatccgcatttccaaagaatgatttcgtatctttctgggttaaatttgaacaaggcgattataaagtggaagagtattgtacgg : 175598
Seq3 : gaaggaacatacgacatatccgcatttccaaagaatgatttcgtatctttctgggttaaatttgaacaaggcgattataaagtggaagagtattgtacgg : 175599
Seq4 : gaaggaacatacgacatatccgcatttccaaagaatgatttcgtatctttctgggttaaatttgaacaaggcgattataaagtggaagagtattgtacgg : 175598

                *    175620         *    175640         *    175660         *    175680         *    175700
Seq1 : gactatgcgtcgaagtaaaaattggaccaccgactgtaacattgactgaatacgacgaccatatcaatttgtacatcgagcatccgtatgctactagagg : 175700
Seq2 : gactatgcgtcgaagtaaaaattggaccaccgactgtaacattgactgaatacgacgaccatatcaatttgtacatcgagcatccgtatgctactagagg : 175698
Seq3 : gactatgcgtcgaagtaaaaattggaccaccgactgtaacattgactgaatacgacgaccatatcaatttgtacatcgagcatccgtatgctactagagg : 175699
Seq4 : gactatgcgtcgaagtaaaaattggaccaccgactgtaacattgactgaatacgacgaccatatcaatttgtacatcgagcatccgtatgctactagagg : 175698
```

```
                *    175720         *    175740         *    175760         *    175780         *    175800
Seq1 : tagcaaaaagattcctatttacaaacgcggtgacatgtgtgatatctacttgttgtatacggctaacttcacattcggagattctaaagaaccagtacca : 175800
Seq2 : tagcaaaaagattcctatttacaaacgcggtgacatgtgtgatatctacttgttgtatacggctaacttcacattcggagattctaaagaaccagtacca : 175798
Seq3 : tagcaaaaagattcctatttacaaacgcggtgacatgtgtgatatctacttgttgtatacggctaacttcacattcggagattctaaagaaccagtacca : 175799
Seq4 : tagcaaaaagattcctatttacaaacgcggtgacatgtgtgatatctacttgttgtatacggctaacttcacattcggagattctaaagaaccagtacca : 175798

                *    175820         *    175840         *    175860         *    175880         *    175900
Seq1 : tatgatatcgatgactacgattgcacgtctacaggttgcagcatagactttgtcacaacagaaaaagtgtgcgtgacagcacagggagccacagaagggt : 175900
Seq2 : tatgatatcgatgactacgattgcacgtctacaggttgcagcatagactttgtcacaacagaaaaagtgtgcgtgacagcacagggagccacagaagggt : 175898
Seq3 : tatgatatcgatgactacgattgcacgtctacaggttgcagcatagactttgtcacaacagaaaaagtgtgcgtgacagcacagggagccacagaagggt : 175899
Seq4 : tatgatatcgatgactacgattgcacgtctacaggttgcagcatagactttgtcacaacagaaaaagtgtgcgtgacagcacagggagccacagaagggt : 175898

                *    175920         *    175940         *    175960         *    175980         *    176000
Seq1 : ttctcgaaaaaattactccatggagttcgaaagtatgtctgacacctaaaaagagtgtatatacatgcgcaattagatccaaagaagatgttcccaattt : 176000
Seq2 : ttctcgaaaaaattactccatggagttcgaaagtatgtctgacacctaaaaagagtgtatatacatgcgcaattagatccaaagaagatgttcccaattt : 175998
Seq3 : ttctcgaaaaaattactccatggagttcgaaagtatgtctgacacctaaaaagagtgtatatacatgcgcaattagatccaaagaagatgttcccaattt : 175999
Seq4 : ttctcgaaaaaattactccatggagttcgaaagtatgtctgacacctaaaaagagtgtatatacatgcgcaattagatccaaagaagatgttcccaattt : 175998

                *    176020         *    176040         *    176060         *    176080         *    176100
Seq1 : caaggacaaaatggccagagttatcaagagaaaatttaataaacagtctcaatcttatttaactaaatttctcggtagcacatcaaatgatgttaccact : 176100
Seq2 : caaggacaaaatggccagagttatcaagagaaaatttaataaacagtctcaatcttatttaactaaatttctcggtagcacatcaaatgatgttaccact : 176098
Seq3 : caaggacaaaatggccagagttatcaagagaaaatttaataaacagtctcaatcttatttaactaaatttctcggtagcacatcaaatgatgttaccact : 176099
Seq4 : caaggacaaaatggccagagttatcaagagaaaatttaataaacagtctcaatcttatttaactaaatttctcggtagcacatcaaatgatgttaccact : 176098

                *    176120         *    176140         *    176160         *    176180         *    176200
Seq1 : tttcttagcatgcttaacttgactaaatattcataactaatttttattaatgatacaaaaacgaaataaaactgcatattatacactggttaacgccctt : 176200
Seq2 : tttcttagcatgcttaacttgactaaatattcataactaatttttattaatgatacaaaaacgaaataaaactgcatattatacactggttaacgccctt : 176198
Seq3 : tttcttagcatgcttaacttgactaaatattcataactaatttttattaatgatacaaaaacgaaataaaactgcatattatacactggttaacgccctt : 176199
Seq4 : tttcttagcatgcttaacttgactaaatattcataactaatttttattaatgatacaaaaacgaaataaaactgcatattatacactggttaacgccctt : 176198

                *    176220         *    176240         *    176260         *    176280         *    176300
Seq1 : ataggctctaaccattttcaagatgaggtccctgattatagtccttctgttcccctctatcatctactccatgtctattagacaatgtgagaagactgaa : 176300
Seq2 : ataggctctaaccattttcaagatgaggtccctgattatagtccttctgttcccctctatcatctactccatgtctattagacaatgtgagaagactgaa : 176298
Seq3 : ataggctctaaccattttcaagatgaggtccctgattatagtccttctgttcccctctatcatctactccatgtctattagacaatgtgagaagactgaa : 176299
Seq4 : ataggctctaaccattttcaagatgaggtccctgattatagtccttctgttcccctctatcatctactccatgtctattagacaatgtgagaagactgaa : 176298

                *    176320         *    176340         *    176360         *    176380         *    176400
Seq1 : gaggaaacatggggattgaaaatagggttgtgtataattgccaaagatttctatcccgaaagaactgattgcagtgttcatctcccaactgcaagtgaag : 176400
Seq2 : gaggaaacatggggattgaaaatagggttgtgtataattgccaaagatttctatcccgaaagaactgattgcagtgttcatctcccaactgcaagtgaag : 176398
Seq3 : gaggaaacatggggattgaaaatagggttgtgtataattgccaaagatttctatcccgaaagaactgattgcagtgttcatctcccaactgcaagtgaag : 176399
Seq4 : gaggaaacatggggattgaaaatagggttgtgtataattgccaaagatttctatcccgaaagaactgattgcagtgttcatctcccaactgcaagtgaag : 176398
```

```
                 *    176420          *    176440          *    176460          *    176480          *    176500
Seq1 : gattgataactgaaggcaatggattcagggatatacgaaacaccgataaattataaaaaaagcaatgtgtccgctgtttccgttaataatactattttcg : 176500
Seq2 : gattgataactgaaggcaatggattcagggatatacgaaacaccgataaattataaaaaaagcaatgtgtccgctgtttccgttaataatactattttcg : 176498
Seq3 : gattgataactgaaggcaatggattcagggatatacgaaacaccgataaattataaaaaaagcaatgtgtccgctgtttccgttaataatactattttcg : 176499
Seq4 : gattgataactgaaggcaatggattcagggatatacgaaacaccgataaattataaaaaaagcaatgtgtccgctgtttccgttaataatactattttcg : 176498

                 *    176520          *    176540          *    176560          *    176580          *    176600
Seq1 : taactggcggattattcataaataactctaatagcacgatcgtggttaacaatatggaaaaacttgacatttataaagacaaacaatggtcgattataga : 176600
Seq2 : taactggcggattattcataaataactctaatagcacgatcgtggttaacaatatggaaaaacttgacatttataaagacaaacaatggtcgattataga : 176598
Seq3 : taactggcggattattcataaataactctaatagcacgatcgtggttaacaatatggaaaaacttgacatttataaagacaaacaatggtcgattataga : 176599
Seq4 : taactggcggattattcataaataactctaatagcacgatcgtggttaacaatatggaaaaacttgacatttataaagacaaacaatggtcgattataga : 176598

                 *    176620          *    176640          *    176660          *    176680          *    176700
Seq1 : aatgcctatggctagggtatatcacggcatcgactcgacatttggaatgttatattttgccggaggtctatccgttaccgaacaatatggtaatttagag : 176700
Seq2 : aatgcctatggctagggtatatcacggcatcgactcgacatttggaatgttatattttgccggaggtctatccgttaccgaacaatatggtaatttagag : 176698
Seq3 : aatgcctatggctagggtatatcacggcatcgactcgacatttggaatgttatattttgccggaggtctatccgttaccgaacaatatggtaatttagag : 176699
Seq4 : aatgcctatggctagggtatatcacggcatcgactcgacatttggaatgttatattttgccggaggtctatccgttaccgaacaatatggtaatttagag : 176698

                 *    176720          *    176740          *    176760          *    176780          *    176800
Seq1 : aaaaacaacgagatatcttgttacaatcctagaacgaataagtggtttgatatttcatatactatttataagatatccatatcatcattgtgtaaactaa : 176800
Seq2 : aaaaacaacgagatatcttgttacaatcctagaacgaataagtggtttgatatttcatatactatttataagatatccatatcatcattgtgtaaactaa : 176798
Seq3 : aaaaacaacgagatatcttgttacaatcctagaacgaataagtggtttgatatttcatatactatttataagatatccatatcatcattgtgtaaactaa : 176799
Seq4 : aaaaacaacgagatatcttgttacaatcctagaacgaataagtggtttgatatttcatatactatttataagatatccatatcatcattgtgtaaactaa : 176798

                 *    176820          *    176840          *    176860          *    176880          *    176900
Seq1 : ataacgtcttctatgtatttagtaaggacattggatatgtggaaaagtatgatggtgcatggaagttagtacatgatcgtctccccgctataaaggcatt : 176900
Seq2 : ataacgtcttctatgtatttagtaaggacattggatatgtggaaaagtatgatggtgcatggaagttagtacatgatcgtctccccgctataaaggcatt : 176898
Seq3 : ataacgtcttctatgtatttagtaaggacattggatatgtggaaaagtatgatggtgcatggaagttagtacatgatcgtctccccgctataaaggcatt : 176899
Seq4 : ataacgtcttctatgtatttagtaaggacattggatatgtggaaaagtatgatggtgcatggaagttagtacatgatcgtctccccgctataaaggcatt : 176898

                 *    176920          *    176940          *    176960          *    176980          *    177000
Seq1 : atcaacttctccttattgattgaaaatgaaaatataaatagtttttatgtatagcagtattaccctatagttttattgcttactactaacatggatacag : 177000
Seq2 : atcaacttctccttattgattgaaaatgaaaatataaatagtttttatgtatagcagtattaccctatagttttattgcttactactaacatggatacag : 176998
Seq3 : atcaacttctccttattgattgaaaatgaaaatataaatagtttttatgtatagcagtattaccctatagttttattgcttactactaacatggatacag : 176999
Seq4 : atcaacttctccttattgattgaaaatgaaaatataaatagtttttatgtatagcagtattaccctatagttttattgcttactactaacatggatacag : 176998

                 *    177020          *    177040          *    177060          *    177080          *    177100
Seq1 : atgttacaaatgtagaagatatcataaatgaaatagatagagagaaagaagaaatactaaaaaatgtagaaattgaaaataataaaaacattaacaagaa : 177100
Seq2 : atgttacaaatgtagaagatatcataaatgaaatagatagagagaaagaagaaatactaaaaaatgtagaaattgaaaataataaaaacattaacaagaa : 177098
Seq3 : atgttacaaatgtagaagatatcataaatgaaatagatagagagaaagaagaaatactaaaaaatgtagaaattgaaaataataaaaacattaacaagaa : 177099
Seq4 : atgttacaaatgtagaagatatcataaatgaaatagatagagagaaagaagaaatactaaaaaatgtagaaattgaaaataataaaaacattaacaagaa : 177098
```

```
                *    177120         *    177140         *    177160         *    177180         *    177200
Seq1 : tcatccaagtggatatattagagaagcactcgttattaataccagtagtaatagtgattccattgataaagaagttatagaatgtatctgtcacgatgta : 177200
Seq2 : tcatccaagtggatatattagagaagcactcgttattaataccagtagtaatagtgattccattgataaagaagttatagaatgtatctgtcacgatgta : 177198
Seq3 : tcatccaagtggatatattagagaagcactcgttattaataccagtagtaatagtgattccattgataaagaagttatagaatgtatctgtcacgatgta : 177199
Seq4 : tcatccaagtggatatattagagaagcactcgttattaataccagtagtaatagtgattccattgataaagaagttatagaatgtatctgtcacgatgta : 177198

                *    177220         *    177240         *    177260         *    177280         *    177300
Seq1 : ggaatatagatcatatctactaattttataatcgatacaaaacataaaaaacaactcgttattacatagcaggcatggaatccttcaagtattgttttg : 177300
Seq2 : ggaatatagatcatatctactaattttataatcgatacaaaacataaaaaacaactcgttattacatagcaggcatggaatccttcaagtattgttttg : 177298
Seq3 : ggaatatagatcatatctactaattttataatcgatacaaaacataaaaaacaactcgttattacatagcaggcatggaatccttcaagtattgttttg : 177299
Seq4 : ggaatatagatcatatctactaattttataatcgatacaaaacataaaaaacaactcgttattacatagcaggcatggaatccttcaagtattgttttg : 177298

                *    177320         *    177340         *    177360         *    177380         *    177400
Seq1 : ataacgatggcaagaaatggattatcggaaatactttatattctggtaattcaatactatataaggtcagaaaaaatttcactagttcgttctacaatta : 177400
Seq2 : ataacgatggcaagaaatggattatcggaaatactttatattctggtaattcaatactatataaggtcagaaaaaatttcactagttcgttctacaatta : 177398
Seq3 : ataacgatggcaagaaatggattatcggaaatactttatattctggtaattcaatactatataaggtcagaaaaaatttcactagttcgttctacaatta : 177399
Seq4 : ataacgatggcaagaaatggattatcggaaatactttatattctggtaattcaatactatataaggtcagaaaaaatttcactagttcgttctacaatta : 177398

                *    177420         *    177440         *    177460         *    177480         *    177500
Seq1 : cgtaatgaagatagatcacaaatcacacaagccattgttgtctgaaatacgattctatatatctgtattggatcctttgactatcgacaactggacacgg : 177500
Seq2 : cgtaatgaagatagatcacaaatcacacaagccattgttgtctgaaatacgattctatatatctgtattggatcctttgactatcgacaactggacacgg : 177498
Seq3 : cgtaatgaagatagatcacaaatcacacaagccattgttgtctgaaatacgattctatatatctgtattggatcctttgactatcgacaactggacacgg : 177499
Seq4 : cgtaatgaagatagatcacaaatcacacaagccattgttgtctgaaatacgattctatatatctgtattggatcctttgactatcgacaactggacacgg : 177498

                *    177520         *    177540         *    177560         *    177580         *    177600
Seq1 : gaacgtggtataaagtatttggctattccagatctgtatggaattggagaaaccgatgattatatgttcttcgttataaagaatttgggaagagtattcg : 177600
Seq2 : gaacgtggtataaagtatttggctattccagatctgtatggaattggagaaaccgatgattatatgttcttcgttataaagaatttgggaagagtattcg : 177598
Seq3 : gaacgtggtataaagtatttggctattccagatctgtatggaattggagaaaccgatgattatatgttcttcgttataaagaatttgggaagagtattcg : 177599
Seq4 : gaacgtggtataaagtatttggctattccagatctgtatggaattggagaaaccgatgattatatgttcttcgttataaagaatttgggaagagtattcg : 177598

                *    177620         *    177640         *    177660         *    177680         *    177700
Seq1 : ccccaaaggatactgaatcagtcttcgaagcatgcgtcactatgataaacacgttagagtttatacactctcaaggatttacccatggaaaaatagaacc : 177700
Seq2 : ccccaaaggatactgaatcagtcttcgaagcatgcgtcactatgataaacacgttagagtttatacactctcaaggatttacccatggaaaaatagaacc : 177698
Seq3 : ccccaaaggatactgaatcagtcttcgaagcatgcgtcactatgataaacacgttagagtttatacactctcaaggatttacccatggaaaaatagaacc : 177699
Seq4 : ccccaaaggatactgaatcagtcttcgaagcatgcgtcactatgataaacacgttagagtttatacactctcaaggatttacccatggaaaaatagaacc : 177698

                *    177720         *    177740         *    177760         *    177780         *    177800
Seq1 : gaggaatatactgattagaaataaacgtctttcactaattgactattctagaactaacaaactatacaagagtggaaactcacatatagattacaacgag : 177800
Seq2 : gaggaatatactgattagaaataaacgtctttcactaattgactattctagaactaacaaactatacaagagtggaaactcacatatagattacaacgag : 177798
Seq3 : gaggaatatactgattagaaataaacgtctttcactaattgactattctagaactaacaaactatacaagagtggaaactcacatatagattacaacgag : 177799
Seq4 : gaggaatatactgattagaaataaacgtctttcactaattgactattctagaactaacaaactatacaagagtggaaactcacatatagattacaacgag : 177798
```

```
             *    177820          *    177840          *    177860          *    177880          *    177900
Seq1 : gacatgataacttcaggaaatatcaattatatgtgtgtagacaatcatcttggagcaacagtttcaagacgaggagatttagaaatgttgggatattgca : 177900
Seq2 : gacatgataacttcaggaaatatcaattatatgtgtgtagacaatcatcttggagcaacagtttcaagacgaggagatttagaaatgttgggatattgca : 177898
Seq3 : gacatgataacttcaggaaatatcaattatatgtgtgtagacaatcatcttggagcaacagtttcaagacgaggagatttagaaatgttgggatattgca : 177899
Seq4 : gacatgataacttcaggaaatatcaattatatgtgtgtagacaatcatcttggagcaacagtttcaagacgaggagatttagaaatgttgggatattgca : 177898

             *    177920          *    177940          *    177960          *    177980          *    178000
Seq1 : tgatagaatggttcggtggcaaacttccatggaaaaacgaaagtagtataaaagtaataaaacaaaaaaaagaatataaaaaatttatagctactttctt : 178000
Seq2 : tgatagaatggttcggtggcaaacttccatggaaaaacgaaagtagtataaaagtaataaaacaaaaaaaagaatataaaaaatttatagctactttctt : 177998
Seq3 : tgatagaatggttcggtggcaaacttccatggaaaaacgaaagtagtataaaagtaataaaacaaaaaaaagaatataaaaaatttatagctactttctt : 177999
Seq4 : tgatagaatggttcggtggcaaacttccatggaaaaacgaaagtagtataaaagtaataaaacaaaaaaaagaatataaaaaatttatagctactttctt : 177998

             *    178020          *    178040          *    178060          *    178080          *    178100
Seq1 : tgaggactgttttcctgaaggaaatgaacctctggaattagttagatatatagaattagtatacacgttagattattctcaaactcctaattatgacaga : 178100
Seq2 : tgaggactgttttcctgaaggaaatgaacctctggaattagttagatatatagaattagtatacacgttagattattctcaaactcctaattatgacaga : 178098
Seq3 : tgaggactgttttcctgaaggaaatgaacctctggaattagttagatatatagaattagtatacacgttagattattctcaaactcctaattatgacaga : 178099
Seq4 : tgaggactgttttcctgaaggaaatgaacctctggaattagttagatatatagaattagtatacacgttagattattctcaaactcctaattatgacaga : 178098

             *    178120          *    178140          *    178160          *    178180          *    178200
Seq1 : ctacgtaaactgtttatacaagattgaaattatattctttttttttatagagtgtggtagtgttacggatatttaatattagactatctctatcgcgctac : 178200
Seq2 : ctacgtaaactgtttatacaagattgaaattatattctttttttttatagagtgtggtagtgttacggatatttaatattagactatctctatcgcgctac : 178198
Seq3 : ctacgtaaactgtttatacaagattgaaattatattctttttttttatagagtgtggtagtgttacggatatttaatattagactatctctatcgcgctac : 178199
Seq4 : ctacgtaaactgtttatacaagattgaaattatattctttttttttatagagtgtggtagtgttacggatatttaatattagactatctctatcgcgctac : 178198

             *    178220          *    178240          *    178260          *    178280          *    178300
Seq1 : acgaccaatatcgattactatggatatcttcagggaaatcgcatcttctatgaaaggagagaatgtattcatttctccagcgtcaatctcgtcagtattg : 178300
Seq2 : acgaccaatatcgattactatggatatcttcagggaaatcgcatcttctatgaaaggagagaatgtattcatttctccagcgtcaatctcgtcagtattg : 178298
Seq3 : acgaccaatatcgattactatggatatcttcagggaaatcgcatcttctatgaaaggagagaatgtattcatttctccagcgtcaatctcgtcagtattg : 178299
Seq4 : acgaccaatatcgattactatggatatcttcagggaaatcgcatcttctatgaaaggagagaatgtattcatttctccagcgtcaatctcgtcagtattg : 178298

             *    178320          *    178340          *    178360          *    178380          *    178400
Seq1 : acaatactgtattatggagctaatggatccactgctgaacagctatcaaaatatgtagaaaaggaggagaacatggataaggttagcgctcaaaatatct : 178400
Seq2 : acaatactgtattatggagctaatggatccactgctgaacagctatcaaaatatgtagaaaaggaggagaacatggataaggttagcgctcaaaatatct : 178398
Seq3 : acaatactgtattatggagctaatggatccactgctgaacagctatcaaaatatgtagaaaaggaggagaacatggataaggttagcgctcaaaatatct : 178399
Seq4 : acaatactgtattatggagctaatggatccactgctgaacagctatcaaaatatgtagaaaaggaggagaacatggataaggttagcgctcaaaatatct : 178398

             *    178420          *    178440          *    178460          *    178480          *    178500
Seq1 : cattcaaatccataaataaagtatatgggcgatattctgccgtgtttaaagattccttttttgagaaaaattggcgataagtttcaaactgttgacttcac : 178500
Seq2 : cattcaaatccataaataaagtatatgggcgatattctgccgtgtttaaagattccttttttgagaaaaattggcgataagtttcaaactgttgacttcac : 178498
Seq3 : cattcaaatccataaataaagtatatgggcgatattctgccgtgtttaaagattccttttttgagaaaaattggcgataagtttcaaactgttgacttcac : 178499
Seq4 : cattcaaatccataaataaagtatatgggcgatattctgccgtgtttaaagattccttttttgagaaaaattggcgataagtttcaaactgttgacttcac : 178498
```

```
            *    178520          *    178540          *    178560          *    178580          *    178600
Seq1 : tgattgtcgcactatagatgcaatcaacaagtgtgtagatatctttactgaggggaaaatcaatccactattggatgaaccattgtctcctgatacctgt : 178600
Seq2 : tgattgtcgcactatagatgcaatcaacaagtgtgtagatatctttactgaggggaaaatcaatccactattggatgaaccattgtctcctgatacctgt : 178598
Seq3 : tgattgtcgcactatagatgcaatcaacaagtgtgtagatatctttactgaggggaaaatcaatccactattggatgaaccattgtctcctgatacctgt : 178599
Seq4 : tgattgtcgcactatagatgcaatcaacaagtgtgtagatatctttactgaggggaaaatcaatccactattggatgaaccattgtctcctgatacctgt : 178598

            *    178620          *    178640          *    178660          *    178680          *    178700
Seq1 : ctcctagcaattagtgccgtatactttaaagcaaaatggttgacgccattcgaaaaggaatttaccagtgattatcccttttacgtatctccgacggaaa : 178700
Seq2 : ctcctagcaattagtgccgtatactttaaagcaaaatggttgacgccattcgaaaaggaatttaccagtgattatcccttttacgtatctccgacggaaa : 178698
Seq3 : ctcctagcaattagtgccgtatactttaaagcaaaatggttgacgccattcgaaaaggaatttaccagtgattatcccttttacgtatctccgacggaaa : 178699
Seq4 : ctcctagcaattagtgccgtatactttaaagcaaaatggttgacgccattcgaaaaggaatttaccagtgattatcccttttacgtatctccgacggaaa : 178698

            *    178720          *    178740          *    178760          *    178780          *    178800
Seq1 : tggtagatgtaagtatgatgtctatgtacggcaaggcatttaatcacgcatctgtaaaggaatcattcggcaacttttcaatcatagaactgccatatgt : 178800
Seq2 : tggtagatgtaagtatgatgtctatgtacggcaaggcatttaatcacgcatctgtaaaggaatcattcggcaacttttcaatcatagaactgccatatgt : 178798
Seq3 : tggtagatgtaagtatgatgtctatgtacggcaaggcatttaatcacgcatctgtaaaggaatcattcggcaacttttcaatcatagaactgccatatgt : 178799
Seq4 : tggtagatgtaagtatgatgtctatgtacggcaaggcatttaatcacgcatctgtaaaggaatcattcggcaacttttcaatcatagaactgccatatgt : 178798

            *    178820          *    178840          *    178860          *    178880          *    178900
Seq1 : tggagatactagtatgatggtcattcttccagacaagattgatggattagaatccatagaacaaaatctaacagatacaaattttaagaaatggtgtaac : 178900
Seq2 : tggagatactagtatgatggtcattcttccagacaagattgatggattagaatccatagaacaaaatctaacagatacaaattttaagaaatggtgtaac : 178898
Seq3 : tggagatactagtatgatggtcattcttccagacaagattgatggattagaatccatagaacaaaatctaacagatacaaattttaagaaatggtgtaac : 178899
Seq4 : tggagatactagtatgatggtcattcttccagacaagattgatggattagaatccatagaacaaaatctaacagatacaaattttaagaaatggtgtaac : 178898

            *    178920          *    178940          *    178960          *    178980          *    179000
Seq1 : tctctggaagctacgtttatcgatgttcacattcccaagtttaaggtaacaggctcgtataatctggtggatactctagtaaagtcaggactgacagagg : 179000
Seq2 : tctctggaagctacgtttatcgatgttcacattcccaagtttaaggtaacaggctcgtataatctggtggatactctagtaaagtcaggactgacagagg : 178998
Seq3 : tctctggaagctacgtttatcgatgttcacattcccaagtttaaggtaacaggctcgtataatctggtggatactctagtaaagtcaggactgacagagg : 178999
Seq4 : tctctggaagctacgtttatcgatgttcacattcccaagtttaaggtaacaggctcgtataatctggtggatactctagtaaagtcaggactgacagagg : 178998

            *    179020          *    179040          *    179060          *    179080          *    179100
Seq1 : tgttcggttcaactggagattatagcaatatgtgtaattcagatgtgagtgtcgacgctatgatccacaaaacgtatatagatgtcaatgaagagtatac : 179100
Seq2 : tgttcggttcaactggagattatagcaatatgtgtaattcagatgtgagtgtcgacgctatgatccacaaaacgtatatagatgtcaatgaagagtatac : 179098
Seq3 : tgttcggttcaactggagattatagcaatatgtgtaattcagatgtgagtgtcgacgctatgatccacaaaacgtatatagatgtcaatgaagagtatac : 179099
Seq4 : tgttcggttcaactggagattatagcaatatgtgtaattcagatgtgagtgtcgacgctatgatccacaaaacgtatatagatgtcaatgaagagtatac : 179098

            *    179120          *    179140          *    179160          *    179180          *    179200
Seq1 : agaagcagctgcagcaacttgtgcactggtgtcagactgtgcatcaacaattacaaatgagttctgtgtagatcatccgttcatctatgtgattaggcat : 179200
Seq2 : agaagcagctgcagcaacttgtgcactggtgtcagactgtgcatcaacaattacaaatgagttctgtgtagatcatccgttcatctatgtgattaggcat : 179198
Seq3 : agaagcagctgcagcaacttgtgcactggtgtcagactgtgcatcaacaattacaaatgagttctgtgtagatcatccgttcatctatgtgattaggcat : 179199
Seq4 : agaagcagctgcagcaacttgtgcactggtgtcagactgtgcatcaacaattacaaatgagttctgtgtagatcatccgttcatctatgtgattaggcat : 179198
```

```
                 *    179220          *    179240          *    179260          *    179280          *    179300
Seq1 : gttgatggaaaaattcttttctttggtagatattgctctccgacaactaattgttaaccattttttttaaaaaatagaaaaaacatgtggtattagtgca : 179300
Seq2 : gttgatggaaaaattcttttctttggtagatattgctctccgacaactaattgttaaccattttttttaaaaaatagaaaaaacatgtggtattagtgca : 179298
Seq3 : gttgatggaaaaattcttttctttggtagatattgctctccgacaactaattgttaaccattttttttaaaaaatagaaaaaacatgtggtattagtgca : 179299
Seq4 : gttgatggaaaaattcttttctttggtagatattgctctccgacaactaattgttaaccattttttttaaaaaatagaaaaaacatgtggtattagtgca : 179298

                 *    179320          *    179340          *    179360          *    179380          *    179400
Seq1 : ggtcgttattcttccaattgcaattggtaagatgacggccaactttagtacccacgtcttttcaccacagcactgtggatgtgacagactgaccagtatt : 179400
Seq2 : ggtcgttattcttccaattgcaattggtaagatgacggccaactttagtacccacgtcttttcaccacagcactgtggatgtgacagactgaccagtatt : 179398
Seq3 : ggtcgttattcttccaattgcaattggtaagatgacggccaactttagtacccacgtcttttcaccacagcactgtggatgtgacagactgaccagtatt : 179399
Seq4 : ggtcgttattcttccaattgcaattggtaagatgacggccaactttagtacccacgtcttttcaccacagcactgtggatgtgacagactgaccagtatt : 179398

                 *    179420          *    179440          *    179460          *    179480          *    179500
Seq1 : gatgacgtcagacaatgtttgactgaatatatttattggtcgtcctatgcataccgcaacaggcaatgcgctggacaattgtattccacactcctctctt : 179500
Seq2 : gatgacgtcagacaatgtttgactgaatatatttattggtcgtcctatgcataccgcaacaggcaatgcgctggacaattgtattccacactcctctctt : 179498
Seq3 : gatgacgtcagacaatgtttgactgaatatatttattggtcgtcctatgcataccgcaacaggcaatgcgctggacaattgtattccacactcctctctt : 179499
Seq4 : gatgacgtcagacaatgtttgactgaatatatttattggtcgtcctatgcataccgcaacaggcaatgcgctggacaattgtattccacactcctctctt : 179498

                 *    179520          *    179540          *    179560          *    179580          *    179600
Seq1 : ttagagatgatgcggaattagtgttcatcgacattcgcgagctggtaaaaaatatgccgtgggatgatgtcaaagattgtgcagaaatcatccgttgtta : 179600
Seq2 : ttagagatgatgcggaattagtgttcatcgacattcgcgagctggtaaaaaatatgccgtgggatgatgtcaaagattgtgcagaaatcatccgttgtta : 179598
Seq3 : ttagagatgatgcggaattagtgttcatcgacattcgcgagctggtaaaaaatatgccgtgggatgatgtcaaagattgtgcagaaatcatccgttgtta : 179599
Seq4 : ttagagatgatgcggaattagtgttcatcgacattcgcgagctggtaaaaaatatgccgtgggatgatgtcaaagattgtgcagaaatcatccgttgtta : 179598

                 *    179620          *    179640          *    179660          *    179680          *    179700
Seq1 : tataccggatgagcaaaaaaccatcagagagatttcggccatcatcggactttgtgcatatgctgctacttactggggaggtgaagaccatcccactagt : 179700
Seq2 : tataccggatgagcaaaaaaccatcagagagatttcggccatcatcggactttgtgcatatgctgctacttactggggaggtgaagaccatcccactagt : 179698
Seq3 : tataccggatgagcaaaaaaccatcagagagatttcggccatcatcggactttgtgcatatgctgctacttactggggaggtgaagaccatcccactagt : 179699
Seq4 : tataccggatgagcaaaaaaccatcagagagatttcggccatcatcggactttgtgcatatgctgctacttactggggaggtgaagaccatcccactagt : 179698

                 *    179720          *    179740          *    179760          *    179780          *    179800
Seq1 : aacagtctgaacgcattgtttgtgatgcttgagatgctcaattacgtggattataacatcatattccggcgtatgaattgatgagttgtacatcttgaca : 179800
Seq2 : aacagtctgaacgcattgtttgtgatgcttgagatgctcaattacgtggattataacatcatattccggcgtatgaattgatgagttgtacatcttgaca : 179798
Seq3 : aacagtctgaacgcattgtttgtgatgcttgagatgctcaattacgtggattataacatcatattccggcgtatgaattgatgagttgtacatcttgaca : 179799
Seq4 : aacagtctgaacgcattgtttgtgatgcttgagatgctcaattacgtggattataacatcatattccggcgtatgaattgatgagttgtacatcttgaca : 179798

                 *    179820          *    179840          *    179860          *    179880          *    179900
Seq1 : ttttcttctttcttctcttctccctttcccagaaacaaactttttttacccactataaaataaaatgagtatactacctgttatatttctttctatattt : 179900
Seq2 : ttttcttctttcttctcttctccctttcccagaaacaaactttttttacccactataaaataaaatgagtatactacctgttatatttctttctatattt : 179898
Seq3 : ttttcttctttcttctcttctccctttcccagaaacaaactttttttacccactataaaataaaatgagtatactacctgttatatttctttctatattt : 179899
Seq4 : ttttcttctttcttctcttctccctttcccagaaacaaactttttttacccactataaaataaaatgagtatactacctgttatatttctttctatattt : 179898
```

```
                *    179920         *    179940         *    179960         *    179980         *    180000
Seq1 : ttttattcttcattcgttcagacttttaacgcgcctgaatgtatcgacaaagggcaatattttgcatcattcatggagttagaaaacgagccagtaatct : 180000
Seq2 : ttttattcttcattcgttcagacttttaacgcgcctgaatgtatcgacaaagggcaatattttgcatcattcatggagttagaaaacgagccagtaatct : 179998
Seq3 : ttttattcttcattcgttcagacttttaacgcgcctgaatgtatcgacaaagggcaatattttgcatcattcatggagttagaaaacgagccagtaatct : 179999
Seq4 : ttttattcttcattcgttcagacttttaacgcgcctgaatgtatcgacaaagggcaatattttgcatcattcatggagttagaaaacgagccagtaatct : 179998

                *    180020         *    180040         *    180060         *    180080         *    180100
Seq1 : taccatgtcctcaaataaatacgctatcatccggatataatatattagatattttatgggaaaaacgaggagcggataatgatagaattataccgataga : 180100
Seq2 : taccatgtcctcaaataaatacgctatcatccggatataatatattagatattttatgggaaaaacgaggagcggataatgatagaattataccgataga : 180098
Seq3 : taccatgtcctcaaataaatacgctatcatccggatataatatattagatattttatgggaaaaacgaggagcggataatgatagaattataccgataga : 180099
Seq4 : taccatgtcctcaaataaatacgctatcatccggatataatatattagatattttatgggaaaaacgaggagcggataatgatagaattataccgataga : 180098

                *    180120         *    180140         *    180160         *    180180         *    180200
Seq1 : taatggtagcaatatgctaattctgaacccgacacaatcagactctggtatttatatatgcattaccacgaacgaaacctactgtgacatgatgtcgtta : 180200
Seq2 : taatggtagcaatatgctaattctgaacccgacacaatcagactctggtatttatatatgcattaccacgaacgaaacctactgtgacatgatgtcgtta : 180198
Seq3 : taatggtagcaatatgctaattctgaacccgacacaatcagactctggtatttatatatgcattaccacgaacgaaacctactgtgacatgatgtcgtta : 180199
Seq4 : taatggtagcaatatgctaattctgaacccgacacaatcagactctggtatttatatatgcattaccacgaacgaaacctactgtgacatgatgtcgtta : 180198

                *    180220         *    180240         *    180260         *    180280         *    180300
Seq1 : aatttgacaatcgtgtctgtctcagaatcaaatatagatcttatctcgtatccacaaatagtaaatgagagatctactggcgaaatggtatgtcccaata : 180300
Seq2 : aatttgacaatcgtgtctgtctcagaatcaaatatagatcttatctcgtatccacaaatagtaaatgagagatctactggcgaaatggtatgtcccaata : 180298
Seq3 : aatttgacaatcgtgtctgtctcagaatcaaatatagatcttatctcgtatccacaaatagtaaatgagagatctactggcgaaatggtatgtcccaata : 180299
Seq4 : aatttgacaatcgtgtctgtctcagaatcaaatatagatcttatctcgtatccacaaatagtaaatgagagatctactggcgaaatggtatgtcccaata : 180298

                *    180320         *    180340         *    180360         *    180380         *    180400
Seq1 : ttaatgcatttattgctagtaacgtaaacgcagatattatatggagcgggcatcgacgccttagaaataagagacttaaacaacggacacctggaattat : 180400
Seq2 : ttaatgcatttattgctagtaacgtaaacgcagatattatatggagcgggcatcgacgccttagaaataagagacttaaacaacggacacctggaattat : 180398
Seq3 : ttaatgcatttattgctagtaacgtaaacgcagatattatatggagcgggcatcgacgccttagaaataagagacttaaacaacggacacctggaattat : 180399
Seq4 : ttaatgcatttattgctagtaacgtaaacgcagatattatatggagcgggcatcgacgccttagaaataagagacttaaacaacggacacctggaattat : 180398

                *    180420         *    180440         *    180460         *    180480         *    180500
Seq1 : taccatagaagatgttagaaaaaatgatgctggttattatacatgtgttttagaatatatatacggtggcaaaacatataacgtaaccagaattgtaaaa : 180500
Seq2 : taccatagaagatgttagaaaaaatgatgctggttattatacatgtgttttagaatatatatacggtggcaaaacatataacgtaaccagaattgtaaaa : 180498
Seq3 : taccatagaagatgttagaaaaaatgatgctggttattatacatgtgttttagaatatatatacggtggcaaaacatataacgtaaccagaattgtaaaa : 180499
Seq4 : taccatagaagatgttagaaaaaatgatgctggttattatacatgtgttttagaatatatatacggtggcaaaacatataacgtaaccagaattgtaaaa : 180498

                *    180520         *    180540         *    180560         *    180580         *    180600
Seq1 : ttagaggtacgggataaaataataccttctactatgcaattaccagatggcattgtaacttcaataggtagtaatttgactattgcatgcagagtatcgt : 180600
Seq2 : ttagaggtacgggataaaataataccttctactatgcaattaccagatggcattgtaacttcaataggtagtaatttgactattgcatgcagagtatcgt : 180598
Seq3 : ttagaggtacgggataaaataataccttctactatgcaattaccagatggcattgtaacttcaataggtagtaatttgactattgcatgcagagtatcgt : 180599
Seq4 : ttagaggtacgggataaaataataccttctactatgcaattaccagatggcattgtaacttcaataggtagtaatttgactattgcatgcagagtatcgt : 180598
```

```
                  *    180620         *    180640         *    180660         *    180680         *    180700
Seq1 : tgagacctcccacaacggatgcagacgtcttttggataagtaatggtatgtattacgaagaagatgatggggacggaaacggtagaataagtgtagcaaa : 180700
Seq2 : tgagacctcccacaacggatgcagacgtcttttggataagtaatggtatgtattacgaagaagatgatggggacggaaacggtagaataagtgtagcaaa : 180698
Seq3 : tgagacctcccacaacggatgcagacgtcttttggataagtaatggtatgtattacgaagaagatgatggggacggaaacggtagaataagtgtagcaaa : 180699
Seq4 : tgagacctcccacaacggatgcagacgtcttttggataagtaatggtatgtattacgaagaagatgatggggacggaaacggtagaataagtgtagcaaa : 180698

                  *    180720         *    180740         *    180760         *    180780         *    180800
Seq1 : taaaatctatatgaccgataagagacgtgttattacatcccggttaaacattaatcctgtcaaggaagaagatgctacaacgtttacgtgtatggcgttt : 180800
Seq2 : taaaatctatatgaccgataagagacgtgttattacatcccggttaaacattaatcctgtcaaggaagaagatgctacaacgtttacgtgtatggcgttt : 180798
Seq3 : taaaatctatatgaccgataagagacgtgttattacatcccggttaaacattaatcctgtcaaggaagaagatgctacaacgtttacgtgtatggcgttt : 180799
Seq4 : taaaatctatatgaccgataagagacgtgttattacatcccggttaaacattaatcctgtcaaggaagaagatgctacaacgtttacgtgtatggcgttt : 180798

                  *    180820         *    180840         *    180860         *    180880         *    180900
Seq1 : actattcctagcatcagtaaaacagttactgttagtataacgtgaatgtatgttgttacatttccatgtcaattgagtttataagaattttttatacatta : 180900
Seq2 : actattcctagcatcagtaaaacagttactgttagtataacgtgaatgtatgttgttacatttccatgtcaattgagtttataagaattttttatacatta : 180898
Seq3 : actattcctagcatcagtaaaacagttactgttagtataacgtgaatgtatgttgttacatttccatgtcaattgagtttataagaattttttatacatta : 180899
Seq4 : actattcctagcatcagtaaaacagttactgttagtataacgtgaatgtatgttgttacatttccatgtcaattgagtttataagaattttttatacatta : 180898

                  *    180920         *    180940         *    180960         *    180980         *    181000
Seq1 : tcttccaacaagcaattgacgaacgtattgctatgattaactcccacgatactatgcatattattaatcattaacttgcagactatacctagagctattt : 181000
Seq2 : tcttccaacaagcaattgacgaacgtattgctatgattaactcccacgatactatgcatattattaatcattaacttgcagactatacctagagctattt : 180998
Seq3 : tcttccaacaagcaattgacgaacgtattgctatgattaactcccacgatactatgcatattattaatcattaacttgcagactatacctagagctattt : 180999
Seq4 : tcttccaacaagcaattgacgaacgtattgctatgattaactcccacgatactatgcatattattaatcattaacttgcagactatacctagagctattt : 180998

                  *    181020         *    181040         *    181060         *    181080         *    181100
Seq1 : tgacatactcgtgttcttgtgtaattgcagtatctatattattaaagtacgtaaatctagctatagttttattatttaattttagataatataccgtctc : 181100
Seq2 : tgacatactcgtgttcttgtgtaattgcagtatctatattattaaagtacgtaaatctagctatagttttattatttaattttagataatataccgtctc : 181098
Seq3 : tgacatactcgtgttcttgtgtaattgcagtatctatattattaaagtacgtaaatctagctatagttttattatttaattttagataatataccgtctc : 181099
Seq4 : tgacatactcgtgttcttgtgtaattgcagtatctatattattaaagtacgtaaatctagctatagttttattatttaattttagataatataccgtctc : 181098

                  *    181120         *    181140         *    181160         *    181180         *    181200
Seq1 : cttatttttaaaaattgccacatcctttattaaatcatgaatgggaatttctatgtcatagttaatatattgtgaacaacaagagcagatatctatagga : 181200
Seq2 : cttatttttaaaaattgccacatcctttattaaatcatgaatgggaatttctatgtcatagttaatatattgtgaacaacaagagcagatatctatagga : 181198
Seq3 : cttatttttaaaaattgccacatcctttattaaatcatgaatgggaatttctatgtcatagttaatatattgtgaacaacaagagcagatatctatagga : 181199
Seq4 : cttatttttaaaaattgccacatcctttattaaatcatgaatgggaatttctatgtcatagttaatatattgtgaacaacaagagcagatatctatagga : 181198

                  *    181220         *    181240         *    181260         *    181280         *    181300
Seq1 : aagggtggaatgcgatacattgatctatgtagttttaaaacacacgcaaactttgaagaatttatataaatcattccatcgatacatccttctatgttga : 181300
Seq2 : aagggtggaatgcgatacattgatctatgtagttttaaaacacacgcaaactttgaagaatttatataaatcattccatcgatacatccttctatgttga : 181298
Seq3 : aagggtggaatgcgatacattgatctatgtagttttaaaacacacgcaaactttgaagaatttatataaatcattccatcgatacatccttctatgttga : 181299
Seq4 : aagggtggaatgcgatacattgatctatgtagttttaaaacacacgcaaactttgaagaatttatataaatcattccatcgatacatccttctatgttga : 181298
```

```
             *    181320         *    181340         *    181360         *    181380         *    181400
Seq1 : gatgtatatatccaggaattcgtttattaatatcgggaaatgtataaactaaaacattgcccggaagcggagcttctaccggagttatatcagtttttaa : 181400
Seq2 : gatgtatatatccaggaattcgtttattaatatcgggaaatgtataaactaaaacattgcccggaagcggagcttctaccggagttatatcagtttttaa : 181398
Seq3 : gatgtatatatccaggaattcgtttattaatatcgggaaatgtataaactaaaacattgcccggaagcggagcttctaccggagttatatcagtttttaa : 181399
Seq4 : gatgtatatatccaggaattcgtttattaatatcgggaaatgtataaactaaaacattgcccggaagcggagcttctaccggagttatatcagtttttaa : 181398

             *    181420         *    181440         *    181460         *    181480         *    181500
Seq1 : cttacaaaatgtaaccaatacctttgcatgacttgttttgttcggcaacgttagtttaaacttgacgaatggattaattacaatagcatgatccgcgcat : 181500
Seq2 : cttacaaaatgtaaccaatacctttgcatgacttgttttgttcggcaacgttagtttaaacttgacgaatggattaattacaatagcatgatccgcgcat : 181498
Seq3 : cttacaaaatgtaaccaatacctttgcatgacttgttttgttcggcaacgttagtttaaacttgacgaatggattaattacaatagcatgatccgcgcat : 181499
Seq4 : cttacaaaatgtaaccaatacctttgcatgacttgttttgttcggcaacgttagtttaaacttgacgaatggattaattacaatagcatgatccgcgcat : 181498

             *    181520         *    181540         *    181560         *    181580         *    181600
Seq1 : ctattaagtttttttactttaacgcccttgtatgtttttacagagactttatctaaatttctagtgcttgtatgtgttataaatataacgggatatagaa : 181600
Seq2 : ctattaagtttttttactttaacgcccttgtatgtttttacagagactttatctaaatttctagtgcttgtatgtgttataaatataacgggatatagaa : 181598
Seq3 : ctattaagtttttttactttaacgcccttgtatgtttttacagagactttatctaaatttctagtgcttgtatgtgttataaatataacgggatatagaa : 181599
Seq4 : ctattaagtttttttactttaacgcccttgtatgtttttacagagactttatctaaatttctagtgcttgtatgtgttataaatataacgggatatagaa : 181598

             *    181620         *    181640         *    181660         *    181680         *    181700
Seq1 : ctgaatcacctaccttagatacccaattacattttatcagatccagataataaacaaatttttgtcgccctaactaattctatattgttatatattttaca : 181700
Seq2 : ctgaatcacctaccttagatacccaattacattttatcagatccagataataaacaaatttttgtcgccctaactaattctatattgttatatattttaca : 181698
Seq3 : ctgaatcacctaccttagatacccaattacattttatcagatccagataataaacaaatttttgtcgccctaactaattctatattgttatatattttaca : 181699
Seq4 : ctgaatcacctaccttagatacccaattacattttatcagatccagataataaacaaatttttgtcgccctaactaattctatattgttatatattttaca : 181698

             *    181720         *    181740         *    181760         *    181780         *    181800
Seq1 : attggttatgatatcatgtaataacttggaatctaacgcacatcgtcgtacgtttatacaattgtgatttagtgtagtatatctacacatgtatttttcc : 181800
Seq2 : attggttatgatatcatgtaataacttggaatctaacgcacatcgtcgtacgtttatacaattgtgatttagtgtagtatatctacacatgtatttttcc : 181798
Seq3 : attggttatgatatcatgtaataacttggaatctaacgcacatcgtcgtacgtttatacaattgtgatttagtgtagtatatctacacatgtatttttcc : 181799
Seq4 : attggttatgatatcatgtaataacttggaatctaacgcacatcgtcgtacgtttatacaattgtgatttagtgtagtatatctacacatgtatttttcc : 181798

             *    181820         *    181840         *    181860         *    181880         *    181900
Seq1 : gcgctatagtattctggactagtgataaaactatcgttatatctgtcttcaatgaactcatcgagatattgctctctgtcatattcatacacctgcataa : 181900
Seq2 : gcgctatagtattctggactagtgataaaactatcgttatatctgtcttcaatgaactcatcgagatattgctctctgtcatattcatacacctgcataa : 181898
Seq3 : gcgctatagtattctggactagtgataaaactatcgttatatctgtcttcaatgaactcatcgagatattgctctctgtcatattcatacacctgcataa : 181899
Seq4 : gcgctatagtattctggactagtgataaaactatcgttatatctgtcttcaatgaactcatcgagatattgctctctgtcatattcatacacctgcataa : 181898

             *    181920         *    181940         *    181960         *    181980         *    182000
Seq1 : actttctagacatcttacaatccgtgttatttaggatcatatttacatatttacgggtatatcaaagatgttagattagttaatgggaatcgtctataa : 182000
Seq2 : actttctagacatcttacaatccgtgttatttaggatcatatttacatatttacgggtatatcaaagatgttagattagttaatgggaatcgtctataa : 181998
Seq3 : actttctagacatcttacaatccgtgttatttaggatcatatttacatatttacgggtatatcaaagatgttagattagttaatgggaatcgtctataa : 181999
Seq4 : actttctagacatcttacaatccgtgttatttaggatcatatttacatatttacgggtatatcaaagatgttagattagttaatgggaatcgtctataa : 181998
```

```
                 *    182020         *    182040         *    182060         *    182080         *    182100
Seq1 : taatgaatattaaacaattatatgaggacttttaccacaaagcatcataaaaatgagtcgtcgtctgatttatgttttaaatatcaaccgcgaatcaact : 182100
Seq2 : taatgaatattaaacaattatatgaggacttttaccacaaagcatcataaaaatgagtcgtcgtctgatttatgttttaaatatcaaccgcgaatcaact : 182098
Seq3 : taatgaatattaaacaattatatgaggacttttaccacaaagcatcataaaaatgagtcgtcgtctgatttatgttttaaatatcaaccgcgaatcaact : 182099
Seq4 : taatgaatattaaacaattatatgaggacttttaccacaaagcatcataaaaatgagtcgtcgtctgatttatgttttaaatatcaaccgcgaatcaact : 182098

                 *    182120         *    182140         *    182160         *    182180         *    182200
Seq1 : cataaaatacaagagaatgaaatatatacatattttagtcattgcaatatagaccatacttctacagaacttgattttgtagttaaaaactatgatctaa : 182200
Seq2 : cataaaatacaagagaatgaaatatatacatattttagtcattgcaatatagaccatacttctacagaacttgattttgtagttaaaaactatgatctaa : 182198
Seq3 : cataaaatacaagagaatgaaatatatacatattttagtcattgcaatatagaccatacttctacagaacttgattttgtagttaaaaactatgatctaa : 182199
Seq4 : cataaaatacaagagaatgaaatatatacatattttagtcattgcaatatagaccatacttctacagaacttgattttgtagttaaaaactatgatctaa : 182198

                 *    182220         *    182240         *    182260         *    182280         *    182300
Seq1 : acagacgacaacctgtaactgggtatactgcactacactgctatttgtataataattactttacaaacgatgtactgaagatattattaaatcatggagt : 182300
Seq2 : acagacgacaacctgtaactgggtatactgcactacactgctatttgtataataattactttacaaacgatgtactgaagatattattaaatcatggagt : 182298
Seq3 : acagacgacaacctgtaactgggtatactgcactacactgctatttgtataataattactttacaaacgatgtactgaagatattattaaatcatggagt : 182299
Seq4 : acagacgacaacctgtaactgggtatactgcactacactgctatttgtataataattactttacaaacgatgtactgaagatattattaaatcatggagt : 182298

                 *    182320         *    182340         *    182360         *    182380         *    182400
Seq1 : ggatgtaacgatgaaaaccagtagcggacgtatgcctgtttatatattgcttactagatgttgcaatatttcacatgatgtagtgatagatatgatagac : 182400
Seq2 : ggatgtaacgatgaaaaccagtagcggacgtatgcctgtttatatattgcttactagatgttgcaatatttcacatgatgtagtgatagatatgatagac : 182398
Seq3 : ggatgtaacgatgaaaaccagtagcggacgtatgcctgtttatatattgcttactagatgttgcaatatttcacatgatgtagtgatagatatgatagac : 182399
Seq4 : ggatgtaacgatgaaaaccagtagcggacgtatgcctgtttatatattgcttactagatgttgcaatatttcacatgatgtagtgatagatatgatagac : 182398

                 *    182420         *    182440         *    182460         *    182480         *    182500
Seq1 : aaagataaaaaccacttattacatagagactattccaacctattactagagtatataaaatctcgttacatgttattaaaggaagaggatatcgatgaga : 182500
Seq2 : aaagataaaaaccacttattacatagagactattccaacctattactagagtatataaaatctcgttacatgttattaaaggaagaggatatcgatgaga : 182498
Seq3 : aaagataaaaaccacttattacatagagactattccaacctattactagagtatataaaatctcgttacatgttattaaaggaagaggatatcgatgaga : 182499
Seq4 : aaagataaaaaccacttattacatagagactattccaacctattactagagtatataaaatctcgttacatgttattaaaggaagaggatatcgatgaga : 182498

                 *    182520         *    182540         *    182560         *    182580         *    182600
Seq1 : acatagtatccactttattagataagggaatcgatcctaactttaaacaagacggatatacagcgttacattattattatttgtgtctcgcacacgttta : 182600
Seq2 : acatagtatccactttattagataagggaatcgatcctaactttaaacaagacggatatacagcgttacattattattatttgtgtctcgcacacgttta : 182598
Seq3 : acatagtatccactttattagataagggaatcgatcctaactttaaacaagacggatatacagcgttacattattattatttgtgtctcgcacacgttta : 182599
Seq4 : acatagtatccactttattagataagggaatcgatcctaactttaaacaagacggatatacagcgttacattattattatttgtgtctcgcacacgttta : 182598

                 *    182620         *    182640         *    182660         *    182680         *    182700
Seq1 : taaaccaggtgagtgtagaaaaccgataacgataaaaaaggccaagcgaattatttctttgtttatacaacatggagctaatctaaacgcgttagataat : 182700
Seq2 : taaaccaggtgagtgtagaaaaccgataacgataaaaaaggccaagcgaattatttctttgtttatacaacatggagctaatctaaacgcgttagataat : 182698
Seq3 : taaaccaggtgagtgtagaaaaccgataacgataaaaaaggccaagcgaattatttctttgtttatacaacatggagctaatctaaacgcgttagataat : 182699
Seq4 : taaaccaggtgagtgtagaaaaccgataacgataaaaaaggccaagcgaattatttctttgtttatacaacatggagctaatctaaacgcgttagataat : 182698
```

```
                 *    182720          *    182740          *    182760          *    182780          *    182800
Seq1 : tgtggtaatacaccattccatttgtatcttagtattgaaatgtgtaataatattcatatgactaaaatgctgttgacttttaatccgaatttcgaaatat : 182800
Seq2 : tgtggtaatacaccattccatttgtatcttagtattgaaatgtgtaataatattcatatgactaaaatgctgttgacttttaatccgaatttcgaaatat : 182798
Seq3 : tgtggtaatacaccattccatttgtatcttagtattgaaatgtgtaataatattcatatgactaaaatgctgttgacttttaatccgaatttcgaaatat : 182799
Seq4 : tgtggtaatacaccattccatttgtatcttagtattgaaatgtgtaataatattcatatgactaaaatgctgttgacttttaatccgaatttcgaaatat : 182798

                 *    182820          *    182840          *    182860          *    182880          *    182900
Seq1 : gtaataatcatggattaacgcctatactatgttatataacttccgactacatacaacacgatattcttgttatgttaatacatcactatgaaacaaatgt : 182900
Seq2 : gtaataatcatggattaacgcctatactatgttatataacttccgactacatacaacacgatattcttgttatgttaatacatcactatgaaacaaatgt : 182898
Seq3 : gtaataatcatggattaacgcctatactatgttatataacttccgactacatacaacacgatattcttgttatgttaatacatcactatgaaacaaatgt : 182899
Seq4 : gtaataatcatggattaacgcctatactatgttatataacttccgactacatacaacacgatattcttgttatgttaatacatcactatgaaacaaatgt : 182898

                 *    182920          *    182940          *    182960          *    182980          *    183000
Seq1 : tggagaaatgccgatagatgagcgtcgtataatcgtattcgagtttatcaaaacatattctacacgtcctgcagattcgataacttatttgatgaatagg : 183000
Seq2 : tggagaaatgccgatagatgagcgtcgtataatcgtattcgagtttatcaaaacatattctacacgtcctgcagattcgataacttatttgatgaatagg : 182998
Seq3 : tggagaaatgccgatagatgagcgtcgtataatcgtattcgagtttatcaaaacatattctacacgtcctgcagattcgataacttatttgatgaatagg : 182999
Seq4 : tggagaaatgccgatagatgagcgtcgtataatcgtattcgagtttatcaaaacatattctacacgtcctgcagattcgataacttatttgatgaatagg : 182998

                 *    183020          *    183040          *    183060          *    183080          *    183100
Seq1 : tttaaaaatatagatatttatacccgctatgaaggaaagacattattacacgtagcatgtgaatataataatacacacgtaatagattatcttatacgta : 183100
Seq2 : tttaaaaatatagatatttatacccgctatgaaggaaagacattattacacgtagcatgtgaatataataatacacacgtaatagattatcttatacgta : 183098
Seq3 : tttaaaaatatagatatttatacccgctatgaaggaaagacattattacacgtagcatgtgaatataataatacacacgtaatagattatcttatacgta : 183099
Seq4 : tttaaaaatatagatatttatacccgctatgaaggaaagacattattacacgtagcatgtgaatataataatacacacgtaatagattatcttatacgta : 183098

                 *    183120          *    183140          *    183160          *    183180          *    183200
Seq1 : tcaacggagatataaatgcgttaaccgacaataacaaacacgctacacaactcattatagataacaaagaaaattccccatataccattaattgtttact : 183200
Seq2 : tcaacggagatataaatgcgttaaccgacaataacaaacacgctacacaactcattatagataacaaagaaaattccccatataccattaattgtttact : 183198
Seq3 : tcaacggagatataaatgcgttaaccgacaataacaaacacgctacacaactcattatagataacaaagaaaattccccatataccattaattgtttact : 183199
Seq4 : tcaacggagatataaatgcgttaaccgacaataacaaacacgctacacaactcattatagataacaaagaaaattccccatataccattaattgtttact : 183198

                 *    183220          *    183240          *    183260          *    183280          *    183300
Seq1 : gtatatacttagatatattgtagataagaatgtgataagatcgttggtggatcaacttccatctctacctatcttcgatataaaatcatttgagaaattc : 183300
Seq2 : gtatatacttagatatattgtagataagaatgtgataagatcgttggtggatcaacttccatctctacctatcttcgatataaaatcatttgagaaattc : 183298
Seq3 : gtatatacttagatatattgtagataagaatgtgataagatcgttggtggatcaacttccatctctacctatcttcgatataaaatcatttgagaaattc : 183299
Seq4 : gtatatacttagatatattgtagataagaatgtgataagatcgttggtggatcaacttccatctctacctatcttcgatataaaatcatttgagaaattc : 183298

                 *    183320          *    183340          *    183360          *    183380          *    183400
Seq1 : atatcctactgtatactttagatgacacattttacaatagacacgttaggaatcgcgattctaaaacgtatcgatacgcattttcaaaatacatgtcgt : 183400
Seq2 : atatcctactgtatactttagatgacacattttacaatagacacgttaggaatcgcgattctaaaacgtatcgatacgcattttcaaaatacatgtcgt : 183398
Seq3 : atatcctactgtatactttagatgacacattttacaatagacacgttaggaatcgcgattctaaaacgtatcgatacgcattttcaaaatacatgtcgt : 183399
Seq4 : atatcctactgtatactttagatgacacattttacaatagacacgttaggaatcgcgattctaaaacgtatcgatacgcattttcaaaatacatgtcgt : 183398
```

```
             *    183420          *    183440          *    183460          *    183480          *    183500
Seq1 : ttgataaatacgatggtataataactaaatgtcataaagaaacaatattgctcaaactatccactgttctagacactacactatatgcagttttaagatg : 183500
Seq2 : ttgataaatacgatggtataataactaaatgtcataaagaaacaatattgctcaaactatccactgttctagacactacactatatgcagttttaagatg : 183498
Seq3 : ttgataaatacgatggtataataactaaatgtcataaagaaacaatattgctcaaactatccactgttctagacactacactatatgcagttttaagatg : 183499
Seq4 : ttgataaatacgatggtataataactaaatgtcataaagaaacaatattgctcaaactatccactgttctagacactacactatatgcagttttaagatg : 183498

             *    183520          *    183540          *    183560          *    183580          *    183600
Seq1 : ccataattcgaaaaagttaagaagatacctcaccgagttaaaaaaatataataacgataagtcctttaaaatatattctaatattatgaatgagagatac : 183600
Seq2 : ccataattcgaaaaagttaagaagatacctcaccgagttaaaaaaatataataacgataagtcctttaaaatatattctaatattatgaatgagagatac : 183598
Seq3 : ccataattcgaaaaagttaagaagatacctcaccgagttaaaaaaatataataacgataagtcctttaaaatatattctaatattatgaatgagagatac : 183599
Seq4 : ccataattcgaaaaagttaagaagatacctcaccgagttaaaaaaatataataacgataagtcctttaaaatatattctaatattatgaatgagagatac : 183598

             *    183620          *    183640          *    183660          *    183680          *    183700
Seq1 : cttaatgtatattataaagatatgtacgtgtcaaaggtatatgataaactatttcctgttttcacagataaaaattgtctactaacattactaccttcag : 183700
Seq2 : cttaatgtatattataaagatatgtacgtgtcaaaggtatatgataaactatttcctgttttcacagataaaaattgtctactaacattactaccttcag : 183698
Seq3 : cttaatgtatattataaagatatgtacgtgtcaaaggtatatgataaactatttcctgttttcacagataaaaattgtctactaacattactaccttcag : 183699
Seq4 : cttaatgtatattataaagatatgtacgtgtcaaaggtatatgataaactatttcctgttttcacagataaaaattgtctactaacattactaccttcag : 183698

             *    183720          *    183740          *    183760          *    183780          *    183800
Seq1 : aaattatatacgaaatattatacatgctgacaattaacgatctttataatatatcgtatccacctaccaaagtatagttgtatttttctcatgcgatgtg : 183800
Seq2 : aaattatatacgaaatattatacatgctgacaattaacgatctttataatatatcgtatccacctaccaaagtatagttgtatttttctcatgcgatgtg : 183798
Seq3 : aaattatatacgaaatattatacatgctgacaattaacgatctttataatatatcgtatccacctaccaaagtatagttgtatttttctcatgcgatgtg : 183799
Seq4 : aaattatatacgaaatattatacatgctgacaattaacgatctttataatatatcgtatccacctaccaaagtatagttgtatttttctcatgcgatgtg : 183798

             *    183820          *    183840          *    183860          *    183880          *    183900
Seq1 : tgtaaaaaaactgatattatataaatattttagtgccgtataataaagatgacgatgaaaatgatggtacatatatatttcgtatcattattgttattgc : 183900
Seq2 : tgtaaaaaaactgatattatataaatattttagtgccgtataataaagatgacgatgaaaatgatggtacatatatatttcgtatcattattgttattgc : 183898
Seq3 : tgtaaaaaaactgatattatataaatattttagtgccgtataataaagatgacgatgaaaatgatggtacatatatatttcgtatcattattgttattgc : 183899
Seq4 : tgtaaaaaaactgatattatataaatattttagtgccgtataataaagatgacgatgaaaatgatggtacatatatatttcgtatcattattgttattgc : 183898

             *    183920          *    183940          *    183960          *    183980          *    184000
Seq1 : tattccacagttacgccatagacatcgaaaatgaaatcacagaattcttcaataaaatgagagatactctaccagctaaagactctaaatggttgaatcc : 184000
Seq2 : tattccacagttacgccatagacatcgaaaatgaaatcacagaattcttcaataaaatgagagatactctaccagctaaagactctaaatggttgaatcc : 183998
Seq3 : tattccacagttacgccatagacatcgaaaatgaaatcacagaattcttcaataaaatgagagatactctaccagctaaagactctaaatggttgaatcc : 183999
Seq4 : tattccacagttacgccatagacatcgaaaatgaaatcacagaattcttcaataaaatgagagatactctaccagctaaagactctaaatggttgaatcc : 183998

             *    184020          *    184040          *    184060          *    184080          *    184100
Seq1 : agcatgtatgttcggaggcacaatgaatgatatagccgctctaggagagccattcagcgcaaagtgtcctcctattgaagacagtcttttatcgcacaga : 184100
Seq2 : agcatgtatgttcggaggcacaatgaatgatatagccgctctaggagagccattcagcgcaaagtgtcctcctattgaagacagtcttttatcgcacaga : 184098
Seq3 : agcatgtatgttcggaggcacaatgaatgatatagccgctctaggagagccattcagcgcaaagtgtcctcctattgaagacagtcttttatcgcacaga : 184099
Seq4 : agcatgtatgttcggaggcacaatgaatgatatagccgctctaggagagccattcagcgcaaagtgtcctcctattgaagacagtcttttatcgcacaga : 184098
```

```
          *    184120          *    184140          *    184160          *    184180          *    184200
Seq1 : tataaagactatgtggttaaatgggaaaggctagaaaaaaatagacggcgacaggtttctaataaacgtgttaaacatggtgatttatggatagccaact : 184200
Seq2 : tataaagactatgtggttaaatgggaaaggctagaaaaaaatagacggcgacaggtttctaataaacgtgttaaacatggtgatttatggatagccaact : 184198
Seq3 : tataaagactatgtggttaaatgggaaaggctagaaaaaaatagacggcgacaggtttctaataaacgtgttaaacatggtgatttatggatagccaact : 184199
Seq4 : tataaagactatgtggttaaatgggaaaggctagaaaaaaatagacggcgacaggtttctaataaacgtgttaaacatggtgatttatggatagccaact : 184198

          *    184220          *    184240          *    184260          *    184280          *    184300
Seq1 : atacatctaaattcagtaaccgtaggtatttgtgcaccgtaactacaaagaatggtgactgtgttcagggtatagttagatctcatattagaaaacctcc : 184300
Seq2 : atacatctaaattcagtaaccgtaggtatttgtgcaccgtaactacaaagaatggtgactgtgttcagggtatagttagatctcatattagaaaacctcc : 184298
Seq3 : atacatctaaattcagtaaccgtaggtatttgtgcaccgtaactacaaagaatggtgactgtgttcagggtatagttagatctcatattagaaaacctcc : 184299
Seq4 : atacatctaaattcagtaaccgtaggtatttgtgcaccgtaactacaaagaatggtgactgtgttcagggtatagttagatctcatattagaaaacctcc : 184298

          *    184320          *    184340          *    184360          *    184380          *    184400
Seq1 : ttcatgcattccaaaaacatatgaactaggtactcatgataagtatggcatagacttatactgtggaattctttacgcaaaacattataataatataact : 184400
Seq2 : ttcatgcattccaaaaacatatgaactaggtactcatgataagtatggcatagacttatactgtggaattctttacgcaaaacattataataatataact : 184398
Seq3 : ttcatgcattccaaaaacatatgaactaggtactcatgataagtatggcatagacttatactgtggaattctttacgcaaaacattataataatataact : 184399
Seq4 : ttcatgcattccaaaaacatatgaactaggtactcatgataagtatggcatagacttatactgtggaattctttacgcaaaacattataataatataact : 184398

          *    184420          *    184440          *    184460          *    184480          *    184500
Seq1 : tggtataaagataataaggaaattaatatcgacgacattaagtattcacaaacgggaaaggaattaattattcataatccagagttagaagatagcggaa : 184500
Seq2 : tggtataaagataataaggaaattaatatcgacgacattaagtattcacaaacgggaaaggaattaattattcataatccagagttagaagatagcggaa : 184498
Seq3 : tggtataaagataataaggaaattaatatcgacgacattaagtattcacaaacgggaaaggaattaattattcataatccagagttagaagatagcggaa : 184499
Seq4 : tggtataaagataataaggaaattaatatcgacgacattaagtattcacaaacgggaaaggaattaattattcataatccagagttagaagatagcggaa : 184498

          *    184520          *    184540          *    184560          *    184580          *    184600
Seq1 : gatacgactgttacgttcattacgacgacgttagaatcaagaatgatatcgtagtatcaagatgtaaaatacttacggttataccgtcacaagaccacag : 184600
Seq2 : gatacgactgttacgttcattacgacgacgttagaatcaagaatgatatcgtagtatcaagatgtaaaatacttacggttataccgtcacaagaccacag : 184598
Seq3 : gatacgactgttacgttcattacgacgacgttagaatcaagaatgatatcgtagtatcaagatgtaaaatacttacggttataccgtcacaagaccacag : 184599
Seq4 : gatacgactgttacgttcattacgacgacgttagaatcaagaatgatatcgtagtatcaagatgtaaaatacttacggttataccgtcacaagaccacag : 184598

          *    184620          *    184640          *    184660          *    184680          *    184700
Seq1 : gtttaaactaatactagatccaaaaatcaacgtaacgataggagaacctgccaatataacatgcactgctgtgtcaacgtcattattgattgacgatgta : 184700
Seq2 : gtttaaactaatactagatccaaaaatcaacgtaacgataggagaacctgccaatataacatgcactgctgtgtcaacgtcattattgattgacgatgta : 184698
Seq3 : gtttaaactaatactagatccaaaaatcaacgtaacgataggagaacctgccaatataacatgcactgctgtgtcaacgtcattattgattgacgatgta : 184699
Seq4 : gtttaaactaatactagatccaaaaatcaacgtaacgataggagaacctgccaatataacatgcactgctgtgtcaacgtcattattgattgacgatgta : 184698

          *    184720          *    184740          *    184760          *    184780          *    184800
Seq1 : ctgattgaatgggaaaatccatccggatggcttataggattcgatttgatgtatactctgttttaactagtagaggcggtattaccgaggcgaccttgt : 184800
Seq2 : ctgattgaatgggaaaatccatccggatggcttataggattcgatttgatgtatactctgttttaactagtagaggcggtattaccgaggcgaccttgt : 184798
Seq3 : ctgattgaatgggaaaatccatccggatggcttataggattcgatttgatgtatactctgttttaactagtagaggcggtattaccgaggcgaccttgt : 184799
Seq4 : ctgattgaatgggaaaatccatccggatggcttataggattcgatttgatgtatactctgttttaactagtagaggcggtattaccgaggcgaccttgt : 184798
```

```
            *    184820          *    184840          *    184860          *    184880          *    184900
Seq1 : actttgaaaatgttactgaagaatatataggtaatacatataaatgtcgtggacacaactattattttgaaaaaacccttacaactacagtagtattgga : 184900
Seq2 : actttgaaaatgttactgaagaatatataggtaatacatataaatgtcgtggacacaactattattttgaaaaaacccttacaactacagtagtattgga : 184898
Seq3 : actttgaaaatgttactgaagaatatataggtaatacatataaatgtcgtggacacaactattattttgaaaaaacccttacaactacagtagtattgga : 184899
Seq4 : actttgaaaatgttactgaagaatatataggtaatacatataaatgtcgtggacacaactattattttgaaaaaacccttacaactacagtagtattgga : 184898

            *    184920          *    184940          *    184960          *    184980          *    185000
Seq1 : gtaaatatacaatgcattttatatacattactgaataattattattattatttatatcgtatttgtgctataacgcgactatctaggtatttgtatctc : 185000
Seq2 : gtaaatatacaatgcattttatatacattactgaataattattattattatttatatcgtatttgtgctataacgcgactatctaggtatttgtatctc : 184998
Seq3 : gtaaatatacaatgcattttatatacattactgaataattattattattatttatatcgtatttgtgctataacgcgactatctaggtatttgtatctc : 184999
Seq4 : gtaaatatacaatgcattttatatacattactgaataattattattattatttatatcgtatttgtgctataacgcgactatctaggtatttgtatctc : 184998

            *    185020          *    185040          *    185060          *    185080          *    185100
Seq1 : accgatagagaacatataaatgtagactctattaaacagttgtgtaaaatatcagatcctaatgcatgttatagatgtggatgtacggctttacatgagt : 185100
Seq2 : accgatagagaacatataaatgtagactctattaaacagttgtgtaaaatatcagatcctaatgcatgttatagatgtggatgtacggctttacatgagt : 185098
Seq3 : accgatagagaacatataaatgtagactctattaaacagttgtgtaaaatatcagatcctaatgcatgttatagatgtggatgtacggctttacatgagt : 185099
Seq4 : accgatagagaacatataaatgtagactctattaaacagttgtgtaaaatatcagatcctaatgcatgttatagatgtggatgtacggctttacatgagt : 185098

            *    185120          *    185140          *    185160          *    185180          *    185200
Seq1 : acttttataattatagatcagtcaacggaaaatacaagtatagatacaacggttactatcaatattatttatctagcgattatgaaaattataatgaata : 185200
Seq2 : acttttataattatagatcagtcaacggaaaatacaagtatagatacaacggttactatcaatattatttatctagcgattatgaaaattataatgaata : 185198
Seq3 : acttttataattatagatcagtcaacggaaaatacaagtatagatacaacggttactatcaatattatttatctagcgattatgaaaattataatgaata : 185199
Seq4 : acttttataattatagatcagtcaacggaaaatacaagtatagatacaacggttactatcaatattatttatctagcgattatgaaaattataatgaata : 185198

            *    185220          *    185240          *    185260          *    185280          *    185300
Seq1 : ttattatgatgattatgatagaactggtatgaacagtgagagtgataatatatcaatcaaaacagaatatgaattctatgatgaaacacaagatcaaagt : 185300
Seq2 : ttattatgatgattatgatagaactggtatgaacagtgagagtgataatatatcaatcaaaacagaatatgaattctatgatgaaacacaagatcaaagt : 185298
Seq3 : ttattatgatgattatgatagaactggtatgaacagtgagagtgataatatatcaatcaaaacagaatatgaattctatgatgaaacacaagatcaaagt : 185299
Seq4 : ttattatgatgattatgatagaactggtatgaacagtgagagtgataatatatcaatcaaaacagaatatgaattctatgatgaaacacaagatcaaagt : 185298

            *    185320          *    185340          *    185360          *    185380          *    185400
Seq1 : acacaactagtaggttacgacattaaactcaaaaccaatgaggatgattttatggctatgatagatcagtgggtgtccatgattatatagatgaatcaat : 185400
Seq2 : acacaactagtaggttacgacattaaactcaaaaccaatgaggatgattttatggctatgatagatcagtgggtgtccatgattatatagatgaatcaat : 185398
Seq3 : acacaactagtaggttacgacattaaactcaaaaccaatgaggatgattttatggctatgatagatcagtgggtgtccatgattatatagatgaatcaat : 185399
Seq4 : acacaactagtaggttacgacattaaactcaaaaccaatgaggatgattttatggctatgatagatcagtgggtgtccatgattatatagatgaatcaat : 185398

            *    185420          *    185440          *    185460          *    185480          *    185500
Seq1 : taataaagtagtatatggaagagagtctcacgtaagatggcgggatatatggcaagaacataatgatggcgtatacagtataggaaaggagtgcatagat : 185500
Seq2 : taataaagtagtatatggaagagagtctcacgtaagatggcgggatatatggcaagaacataatgatggcgtatacagtataggaaaggagtgcatagat : 185498
Seq3 : taataaagtagtatatggaagagagtctcacgtaagatggcgggatatatggcaagaacataatgatggcgtatacagtataggaaaggagtgcatagat : 185499
Seq4 : taataaagtagtatatggaagagagtctcacgtaagatggcgggatatatggcaagaacataatgatggcgtatacagtataggaaaggagtgcatagat : 185498
```

```
                 *    185520          *    185540          *    185560          *    185580          *    185600
Seq1 : aatatatacgaagacaaccataccgtagacgaattctacaagatagacagcgtatcagatgtagatgacgcggaacacatatctccgataactaatgatg : 185600
Seq2 : aatatatacgaagacaaccataccgtagacgaattctacaagatagacagcgtatcagatgtagatgacgcggaacacatatctccgataactaatgatg : 185598
Seq3 : aatatatacgaagacaaccataccgtagacgaattctacaagatagacagcgtatcagatgtagatgacgcggaacacatatctccgataactaatgatg : 185599
Seq4 : aatatatacgaagacaaccataccgtagacgaattctacaagatagacagcgtatcagatgtagatgacgcggaacacatatctccgataactaatgatg : 185598

                 *    185620          *    185640          *    185660          *    185680          *    185700
Seq1 : tatctacacaaacatgggaaaagaaatcagagttagatagatacatggaaatgtatcctcgtcatagatatagtaagcattctgtctttaagggattttc : 185700
Seq2 : tatctacacaaacatgggaaaagaaatcagagttagatagatacatggaaatgtatcctcgtcatagatatagtaagcattctgtctttaagggattttc : 185698
Seq3 : tatctacacaaacatgggaaaagaaatcagagttagatagatacatggaaatgtatcctcgtcatagatatagtaagcattctgtctttaagggattttc : 185699
Seq4 : tatctacacaaacatgggaaaagaaatcagagttagatagatacatggaaatgtatcctcgtcatagatatagtaagcattctgtctttaagggattttc : 185698

                 *    185720          *    185740          *    185760          *    185780          *    185800
Seq1 : tgacaaagttagaaaaaatgatttagacatgaatgtggtaaaagaattactttctaacggtgcatctctaacaattaaggatagcagtaataaggatcca : 185800
Seq2 : tgacaaagttagaaaaaatgatttagacatgaatgtggtaaaagaattactttctaacggtgcatctctaacaattaaggatagcagtaataaggatcca : 185798
Seq3 : tgacaaagttagaaaaaatgatttagacatgaatgtggtaaaagaattactttctaacggtgcatctctaacaattaaggatagcagtaataaggatcca : 185799
Seq4 : tgacaaagttagaaaaaatgatttagacatgaatgtggtaaaagaattactttctaacggtgcatctctaacaattaaggatagcagtaataaggatcca : 185798

                 *    185820          *    185840          *    185860          *    185880          *    185900
Seq1 : ataaccgtttattttcgaagaacgataatgaatttagaaatgattgatgaacgaaagtatatagtacactcctatctaaaaaattataaaaatttcgatt : 185900
Seq2 : ataaccgtttattttcgaagaacgataatgaatttagaaatgattgatgaacgaaagtatatagtacactcctatctaaaaaattataaaaatttcgatt : 185898
Seq3 : ataaccgtttattttcgaagaacgataatgaatttagaaatgattgatgaacgaaagtatatagtacactcctatctaaaaaattataaaaatttcgatt : 185899
Seq4 : ataaccgtttattttcgaagaacgataatgaatttagaaatgattgatgaacgaaagtatatagtacactcctatctaaaaaattataaaaatttcgatt : 185898

                 *    185920          *    185940          *    185960          *    185980          *    186000
Seq1 : atccattttcaggaagttagttttgactaataaacattgtctcaacaattattataatataagcgacagcaaatatggaacaccgctacatatattggc : 186000
Seq2 : atccattttcaggaagttagttttgactaataaacattgtctcaacaattattataatataagcgacagcaaatatggaacaccgctacatatattggc : 185998
Seq3 : atccattttcaggaagttagttttgactaataaacattgtctcaacaattattataatataagcgacagcaaatatggaacaccgctacatatattggc : 185999
Seq4 : atccattttcaggaagttagttttgactaataaacattgtctcaacaattattataatataagcgacagcaaatatggaacaccgctacatatattggc : 185998

                 *    186020          *    186040          *    186060          *    186080          *    186100
Seq1 : gtctaataaaaaattaataactcctaattacatgaagttattagtgtataacggaaatgatataaacgcacgaggtgaagatacacaaatgcgaactcca : 186100
Seq2 : gtctaataaaaaattaataactcctaattacatgaagttattagtgtataacggaaatgatataaacgcacgaggtgaagatacacaaatgcgaactcca : 186098
Seq3 : gtctaataaaaaattaataactcctaattacatgaagttattagtgtataacggaaatgatataaacgcacgaggtgaagatacacaaatgcgaactcca : 186099
Seq4 : gtctaataaaaaattaataactcctaattacatgaagttattagtgtataacggaaatgatataaacgcacgaggtgaagatacacaaatgcgaactcca : 186098

                 *    186120          *    186140          *    186160          *    186180          *    186200
Seq1 : ttacacaaatatttgtgtaaatttgtatatcataatattgaatatggtatccgatactataatgaaaagattatagacgcatttatagagttaggagccg : 186200
Seq2 : ttacacaaatatttgtgtaaatttgtatatcataatattgaatatggtatccgatactataatgaaaagattatagacgcatttatagagttaggagccg : 186198
Seq3 : ttacacaaatatttgtgtaaatttgtatatcataatattgaatatggtatccgatactataatgaaaagattatagacgcatttatagagttaggagccg : 186199
Seq4 : ttacacaaatatttgtgtaaatttgtatatcataatattgaatatggtatccgatactataatgaaaagattatagacgcatttatagagttaggagccg : 186198
```

```
               *    186220         *    186240         *    186260         *    186280         *    186300
Seq1 : atctaactattccaaatgacgatggaatgataccagtagtttactgtatacactcaaatgccgaatatggttataacaatattactaacataaagataat : 186300
Seq2 : atctaactattccaaatgacgatggaatgataccagtagtttactgtatacactcaaatgccgaatatggttataacaatattactaacataaagataat : 186298
Seq3 : atctaactattccaaatgacgatggaatgataccagtagtttactgtatacactcaaatgccgaatatggttataacaatattactaacataaagataat : 186299
Seq4 : atctaactattccaaatgacgatggaatgataccagtagtttactgtatacactcaaatgccgaatatggttataacaatattactaacataaagataat : 186298

               *    186320         *    186340         *    186360         *    186380         *    186400
Seq1 : acgtaaactacttaatcttagtagacgtgcgtcacataatctatttagagatcgagtcatgcacgattatataagtaatacatatattgatcttgagtgt : 186400
Seq2 : acgtaaactacttaatcttagtagacgtgcgtcacataatctatttagagatcgagtcatgcacgattatataagtaatacatatattgatcttgagtgt : 186398
Seq3 : acgtaaactacttaatcttagtagacgtgcgtcacataatctatttagagatcgagtcatgcacgattatataagtaatacatatattgatcttgagtgt : 186399
Seq4 : acgtaaactacttaatcttagtagacgtgcgtcacataatctatttagagatcgagtcatgcacgattatataagtaatacatatattgatcttgagtgt : 186398

               *    186420         *    186440         *    186460         *    186480         *    186500
Seq1 : ttagatattattagatcgttggatggattcgatatcaatggttactttgaaggacgtacaccacttcattgcgctatacaacataacttcactcagattg : 186500
Seq2 : ttagatattattagatcgttggatggattcgatatcaatggttactttgaaggacgtacaccacttcattgcgctatacaacataacttcactcagattg : 186498
Seq3 : ttagatattattagatcgttggatggattcgatatcaatggttactttgaaggacgtacaccacttcattgcgctatacaacataacttcactcagattg : 186499
Seq4 : ttagatattattagatcgttggatggattcgatatcaatggttactttgaaggacgtacaccacttcattgcgctatacaacataacttcactcagattg : 186498

               *    186520         *    186540         *    186560         *    186580         *    186600
Seq1 : ctaagtacttattagatcgaggagctgatatagtcgtacccaacacattgattatacatcagtacatacagtaaatagcatagatatggaggaggataca : 186600
Seq2 : ctaagtacttattagatcgaggagctgatatagtcgtacccaacacattgattatacatcagtacatacagtaaatagcatagatatggaggaggataca : 186598
Seq3 : ctaagtacttattagatcgaggagctgatatagtcgtacccaacacattgattatacatcagtacatacagtaaatagcatagatatggaggaggataca : 186599
Seq4 : ctaagtacttattagatcgaggagctgatatagtcgtacccaacacattgattatacatcagtacatacagtaaatagcatagatatggaggaggataca : 186598

               *    186620         *    186640         *    186660         *    186680         *    186700
Seq1 : aatatttcaaataaagttataaggtacaacactgtcaataatatatgggaaacattacctaacttctggactggaactataaatccaggcgtggtctcgc : 186700
Seq2 : aatatttcaaataaagttataaggtacaacactgtcaataatatatgggaaacattacctaacttctggactggaactataaatccaggcgtggtctcgc : 186698
Seq3 : aatatttcaaataaagttataaggtacaacactgtcaataatatatgggaaacattacctaacttctggactggaactataaatccaggcgtggtctcgc : 186699
Seq4 : aatatttcaaataaagttataaggtacaacactgtcaataatatatgggaaacattacctaacttctggactggaactataaatccaggcgtggtctcgc : 186698

               *    186720         *    186740         *    186760         *    186780         *    186800
Seq1 : ataaagatgatatatatgttgtatgcgacatcaaagatgaaaaaaatgttaaaacttgtatatttagatataacacgaatacgtataacggatgggaatt : 186800
Seq2 : ataaagatgatatatatgttgtatgcgacatcaaagatgaaaaaaatgttaaaacttgtatatttagatataacacgaatacgtataacggatgggaatt : 186798
Seq3 : ataaagatgatatatatgttgtatgcgacatcaaagatgaaaaaaatgttaaaacttgtatatttagatataacacgaatacgtataacggatgggaatt : 186799
Seq4 : ataaagatgatatatatgttgtatgcgacatcaaagatgaaaaaaatgttaaaacttgtatatttagatataacacgaatacgtataacggatgggaatt : 186798

               *    186820         *    186840         *    186860         *    186880         *    186900
Seq1 : ggtcacgacgacagaaagcagattatcagctctgcatactattctttataacaataccataatgatgttacattgttatgaatcgtatatgttacaagat : 186900
Seq2 : ggtcacgacgacagaaagcagattatcagctctgcatactattctttataacaataccataatgatgttacattgttatgaatcgtatatgttacaagat : 186898
Seq3 : ggtcacgacgacagaaagcagattatcagctctgcatactattctttataacaataccataatgatgttacattgttatgaatcgtatatgttacaagat : 186899
Seq4 : ggtcacgacgacagaaagcagattatcagctctgcatactattctttataacaataccataatgatgttacattgttatgaatcgtatatgttacaagat : 186898
```

```
             *    186920          *    186940          *    186960          *    186980          *    187000
Seq1 : acatttaatgtgtacactcgcgaatggaatcatatgtgtcatcaacattcgaatagttatatcatgtacaatatactacccatctactaaatataataga : 187000
Seq2 : acatttaatgtgtacactcgcgaatggaatcatatgtgtcatcaacattcgaatagttatatcatgtacaatatactacccatctactaaatataataga : 186998
Seq3 : acatttaatgtgtacactcgcgaatggaatcatatgtgtcatcaacattcgaatagttatatcatgtacaatatactacccatctactaaatataataga : 186999
Seq4 : acatttaatgtgtacactcgcgaatggaatcatatgtgtcatcaacattcgaatagttatatcatgtacaatatactacccatctactaaatataataga : 186998

             *    187020          *    187040          *    187060          *    187080          *    187100
Seq1 : ataaaataaatgagtatgatcattttagataacgattgattttatcattaccgcttcattcttatattctttgcttacggaacctatatttagaaacatc : 187100
Seq2 : ataaaataaatgagtatgatcattttagataacgattgattttatcattaccgcttcattcttatattctttgcttacggaacctatatttagaaacatc : 187098
Seq3 : ataaaataaatgagtatgatcattttagataacgattgattttatcattaccgcttcattcttatattctttgcttacggaacctatatttagaaacatc : 187099
Seq4 : ataaaataaatgagtatgatcattttagataacgattgattttatcattaccgcttcattcttatattctttgcttacggaacctatatttagaaacatc : 187098

             *    187120          *    187140          *    187160          *    187180          *    187200
Seq1 : tactaacgattttttatgcttgcattattaatggtatgtaatatgattgattgtgtacgcaataccaatttgttaagtatgaatacggggtacaaacata : 187200
Seq2 : tactaacgattttttatgcttgcattattaatggtatgtaatatgattgattgtgtacgcaataccaatttgttaagtatgaatacggggtacaaacata : 187198
Seq3 : tactaacgattttttatgcttgcattattaatggtatgtaatatgattgattgtgtacgcaataccaatttgttaagtatgaatacggggtacaaacata : 187199
Seq4 : tactaacgattttttatgcttgcattattaatggtatgtaatatgattgattgtgtacgcaataccaatttgttaagtatgaatacggggtacaaacata : 187198

             *    187220          *    187240          *    187260          *    187280          *    187300
Seq1 : aactgaagtttaacattatttatttatgatatatatcgttattgtttggtctataccatggatatctttaaagaactaatcttaaaacacacggatgaaa : 187300
Seq2 : aactgaagtttaacattatttatttatgatatatatcgttattgtttggtctataccatggatatctttaaagaactaatcttaaaacacacggatgaaa : 187298
Seq3 : aactgaagtttaacattatttatttatgatatatatcgttattgtttggtctataccatggatatctttaaagaactaatcttaaaacacacggatgaaa : 187299
Seq4 : aactgaagtttaacattatttatttatgatatatatcgttattgtttggtctataccatggatatctttaaagaactaatcttaaaacacacggatgaaa : 187298

             *    187320          *    187340          *    187360          *    187380          *    187400
Seq1 : atgttttgatttctccagtttctattttatctactttatctattctaaatcatggagcagctggttctacagctgaacaactatcaaaatatatagagaa : 187400
Seq2 : atgttttgatttctccagtttctattttatctactttatctattctaaatcatggagcagctggttctacagctgaacaactatcaaaatatatagagaa : 187398
Seq3 : atgttttgatttctccagtttctattttatctactttatctattctaaatcatggagcagctggttctacagctgaacaactatcaaaatatatagagaa : 187399
Seq4 : atgttttgatttctccagtttctattttatctactttatctattctaaatcatggagcagctggttctacagctgaacaactatcaaaatatatagagaa : 187398

             *    187420          *    187440          *    187460          *    187480          *    187500
Seq1 : tatgaatgagaatacacccgatgacaataatgacatggacgtagatattccgtattgtgcgacactagctaccgcaaataaaatatacggtagcgatagt : 187500
Seq2 : tatgaatgagaatacacccgatgacaataatgacatggacgtagatattccgtattgtgcgacactagctaccgcaaataaaatatacggtagcgatagt : 187498
Seq3 : tatgaatgagaatacacccgatgacaataatgacatggacgtagatattccgtattgtgcgacactagctaccgcaaataaaatatacggtagcgatagt : 187499
Seq4 : tatgaatgagaatacacccgatgacaataatgacatggacgtagatattccgtattgtgcgacactagctaccgcaaataaaatatacggtagcgatagt : 187498

             *    187520          *    187540          *    187560          *    187580          *    187600
Seq1 : atcgagttccacgcctccttcctacaaaaaataaaagacgattttcaaactgtaaactttaataatgctaaccaaacaaaggaactaatcaacgaatggg : 187600
Seq2 : atcgagttccacgcctccttcctacaaaaaataaaagacgattttcaaactgtaaactttaataatgctaaccaaacaaaggaactaatcaacgaatggg : 187598
Seq3 : atcgagttccacgcctccttcctacaaaaaataaaagacgattttcaaactgtaaactttaataatgctaaccaaacaaaggaactaatcaacgaatggg : 187599
Seq4 : atcgagttccacgcctccttcctacaaaaaataaaagacgattttcaaactgtaaactttaataatgctaaccaaacaaaggaactaatcaacgaatggg : 187598
```

```
                *    187620         *    187640         *    187660         *    187680         *    187700
Seq1 : ttaagacaatgacaaatggtaaaattaattccttattgactagtccgctatccattaatactcgtatgacagttgttagcgccgtccattttaaagcaat : 187700
Seq2 : ttaagacaatgacaaatggtaaaattaattccttattgactagtccgctatccattaatactcgtatgacagttgttagcgccgtccattttaaagcaat : 187698
Seq3 : ttaagacaatgacaaatggtaaaattaattccttattgactagtccgctatccattaatactcgtatgacagttgttagcgccgtccattttaaagcaat : 187699
Seq4 : ttaagacaatgacaaatggtaaaattaattccttattgactagtccgctatccattaatactcgtatgacagttgttagcgccgtccattttaaagcaat : 187698

                *    187720         *    187740         *    187760         *    187780         *    187800
Seq1 : gtggaaatatccatttctaaacatcttacatatacagacaagttttatatttctaagaatatagttaccagtgttgatatgatggtgagcactgagaat : 187800
Seq2 : gtggaaatatccatttctaaacatcttacatatacagacaagttttatatttctaagaatatagttaccagtgttgatatgatggtgagcactgagaat : 187798
Seq3 : gtggaaatatccatttctaaacatcttacatatacagacaagttttatatttctaagaatatagttaccagtgttgatatgatggtgagcactgagaat : 187799
Seq4 : gtggaaatatccatttctaaacatcttacatatacagacaagttttatatttctaagaatatagttaccagtgttgatatgatggtgagcactgagaat : 187798

                *    187820         *    187840         *    187860         *    187880         *    187900
Seq1 : aacttgcaatatgtacatattaatgaattattcggaggattctctattatcgatattccatacgagggaaactctagtatggtaattatactaccggacg : 187900
Seq2 : aacttgcaatatgtacatattaatgaattattcggaggattctctattatcgatattccatacgagggaaactctagtatggtaattatactaccggacg : 187898
Seq3 : aacttgcaatatgtacatattaatgaattattcggaggattctctattatcgatattccatacgagggaaactctagtatggtaattatactaccggacg : 187899
Seq4 : aacttgcaatatgtacatattaatgaattattcggaggattctctattatcgatattccatacgagggaaactctagtatggtaattatactaccggacg : 187898

                *    187920         *    187940         *    187960         *    187980         *    188000
Seq1 : acatagaaggtatatataacatagaaaaaaatataacagatgaaaaatttaaaaaatggtgtggtatgttatctactaaaagtatagacttgtatatgcc : 188000
Seq2 : acatagaaggtatatataacatagaaaaaaatataacagatgaaaaatttaaaaaatggtgtggtatgttatctactaaaagtatagacttgtatatgcc : 187998
Seq3 : acatagaaggtatatataacatagaaaaaaatataacagatgaaaaatttaaaaaatggtgtggtatgttatctactaaaagtatagacttgtatatgcc : 187999
Seq4 : acatagaaggtatatataacatagaaaaaaatataacagatgaaaaatttaaaaaatggtgtggtatgttatctactaaaagtatagacttgtatatgcc : 187998

                *    188020         *    188040         *    188060         *    188080         *    188100
Seq1 : aaagtttaaagtggaaatgacagaaccgtataatctggtaccgattttagaaaatttaggacttactaatatattcggatattatgcagattttagcaag : 188100
Seq2 : aaagtttaaagtggaaatgacagaaccgtataatctggtaccgattttagaaaatttaggacttactaatatattcggatattatgcagattttagcaag : 188098
Seq3 : aaagtttaaagtggaaatgacagaaccgtataatctggtaccgattttagaaaatttaggacttactaatatattcggatattatgcagattttagcaag : 188099
Seq4 : aaagtttaaagtggaaatgacagaaccgtataatctggtaccgattttagaaaatttaggacttactaatatattcggatattatgcagattttagcaag : 188098

                *    188120         *    188140         *    188160         *    188180         *    188200
Seq1 : atgtgtaatgaaactatcactgtagaaaaatttctacatacgacgtttatagatgttaatgaggagtatacagaagcatcggccgttacaggagtattta : 188200
Seq2 : atgtgtaatgaaactatcactgtagaaaaatttctacatacgacgtttatagatgttaatgaggagtatacagaagcatcggccgttacaggagtattta : 188198
Seq3 : atgtgtaatgaaactatcactgtagaaaaatttctacatacgacgtttatagatgttaatgaggagtatacagaagcatcggccgttacaggagtattta : 188199
Seq4 : atgtgtaatgaaactatcactgtagaaaaatttctacatacgacgtttatagatgttaatgaggagtatacagaagcatcggccgttacaggagtattta : 188198

                *    188220         *    188240         *    188260         *    188280         *    188300
Seq1 : tgactaacttttcgatggtatatcgtacgaaggtctacataaaccatccattcatgtacatgattaaagacaacacaggacgtatactttttatagggaa : 188300
Seq2 : tgactaacttttcgatggtatatcgtacgaaggtctacataaaccatccattcatgtacatgattaaagacaacacaggacgtatactttttatagggaa : 188298
Seq3 : tgactaacttttcgatggtatatcgtacgaaggtctacataaaccatccattcatgtacatgattaaagacaacacaggacgtatactttttatagggaa : 188299
Seq4 : tgactaacttttcgatggtatatcgtacgaaggtctacataaaccatccattcatgtacatgattaaagacaacacaggacgtatactttttatagggaa : 188298
```

```
                 *    188320         *    188340         *    188360         *    188380         *    188400
Seq1 : atactgctatccgcaataaatataaacaaatagacttttatcacgtttatctatgtctaaatattacaaatagtaatagtataaactaaagctgataata : 188400
Seq2 : atactgctatccgcaataaatataaacaaatagacttttatcacgtttatctatgtctaaatattacaaatagtaatagtataaactaaagctgataata : 188398
Seq3 : atactgctatccgcaataaatataaacaaatagacttttatcacgtttatctatgtctaaatattacaaatagtaatagtataaactaaagctgataata : 188399
Seq4 : atactgctatccgcaataaatataaacaaatagacttttatcacgtttatctatgtctaaatattacaaatagtaatagtataaactaaagctgataata : 188398

                 *    188420         *    188440         *    188460         *    188480         *    188500
Seq1 : cttaaaaaaataataatatcatttacaattaatagtataaactaaaaattaaacaaatcgttattataagtaatatcaaaatgatgatatacggattaat : 188500
Seq2 : cttaaaaaaataataatatcatttacaattaatagtataaactaaaaattaaacaaatcgttattataagtaatatcaaaatgatgatatacggattaat : 188498
Seq3 : cttaaaaaaataataatatcatttacaattaatagtataaactaaaaattaaacaaatcgttattataagtaatatcaaaatgatgatatacggattaat : 188499
Seq4 : cttaaaaaaataataatatcatttacaattaatagtataaactaaaaattaaacaaatcgttattataagtaatatcaaaatgatgatatacggattaat : 188498

                 *    188520         *    188540         *    188560         *    188580         *    188600
Seq1 : agcgtgtcttatattcgtgacttcatccatcgctagtccactttatattcccgttattccacccatttcggaagataaatcgttcaatagtgtagaggta : 188600
Seq2 : agcgtgtcttatattcgtgacttcatccatcgctagtccactttatattcccgttattccacccatttcggaagataaatcgttcaatagtgtagaggta : 188598
Seq3 : agcgtgtcttatattcgtgacttcatccatcgctagtccactttatattcccgttattccacccatttcggaagataaatcgttcaatagtgtagaggta : 188599
Seq4 : agcgtgtcttatattcgtgacttcatccatcgctagtccactttatattcccgttattccacccatttcggaagataaatcgttcaatagtgtagaggta : 188598

                 *    188620         *    188640         *    188660         *    188680         *    188700
Seq1 : ttagtttccttgtttagagatgaccaaaaagactatacggtaacttctcagttcaataactacactatcgataccaaagactggactatcggcgtactat : 188700
Seq2 : ttagtttccttgtttagagatgaccaaaaagactatacggtaacttctcagttcaataactacactatcgataccaaagactggactatcggcgtactat : 188698
Seq3 : ttagtttccttgtttagagatgaccaaaaagactatacggtaacttctcagttcaataactacactatcgataccaaagactggactatcggcgtactat : 188699
Seq4 : ttagtttccttgtttagagatgaccaaaaagactatacggtaacttctcagttcaataactacactatcgataccaaagactggactatcggcgtactat : 188698

                 *    188720         *    188740         *    188760         *    188780         *    188800
Seq1 : ccacacctgatggtttggatataccattgactaatataacttattggtcacggtttactataggtcgtgcattgttcaaatcagagtctgaggatatttt : 188800
Seq2 : ccacacctgatggtttggatataccattgactaatataacttattggtcacggtttactataggtcgtgcattgttcaaatcagagtctgaggatatttt : 188798
Seq3 : ccacacctgatggtttggatataccattgactaatataacttattggtcacggtttactataggtcgtgcattgttcaaatcagagtctgaggatatttt : 188799
Seq4 : ccacacctgatggtttggatataccattgactaatataacttattggtcacggtttactataggtcgtgcattgttcaaatcagagtctgaggatatttt : 188798

                 *    188820         *    188840         *    188860         *    188880         *    188900
Seq1 : ccaaaagaaaatgagtattctaggtgtttctatagaatgtaagaagtcgtcgacattacttactttttttgaccgtgcgtaaaatgactcgagtatttaat : 188900
Seq2 : ccaaaagaaaatgagtattctaggtgtttctatagaatgtaagaagtcgtcgacattacttactttttttgaccgtgcgtaaaatgactcgagtatttaat : 188898
Seq3 : ccaaaagaaaatgagtattctaggtgtttctatagaatgtaagaagtcgtcgacattacttactttttttgaccgtgcgtaaaatgactcgagtatttaat : 188899
Seq4 : ccaaaagaaaatgagtattctaggtgtttctatagaatgtaagaagtcgtcgacattacttactttttttgaccgtgcgtaaaatgactcgagtatttaat : 188898

                 *    188920         *    188940         *    188960         *    188980         *    189000
Seq1 : aaatttccagatatggcttattatcgaggagactgtttaaaagccgtttatgtaacaatgacttataaaaatactaaaactggagagactgattacacgt : 189000
Seq2 : aaatttccagatatggcttattatcgaggagactgtttaaaagccgtttatgtaacaatgacttataaaaatactaaaactggagagactgattacacgt : 188998
Seq3 : aaatttccagatatggcttattatcgaggagactgtttaaaagccgtttatgtaacaatgacttataaaaatactaaaactggagagactgattacacgt : 188999
Seq4 : aaatttccagatatggcttattatcgaggagactgtttaaaagccgtttatgtaacaatgacttataaaaatactaaaactggagagactgattacacgt : 188998
```

```
          *    189020         *    189040         *    189060         *    189080         *    189100
Seq1 : acctctctaatggggggttgcctgcatactatcgtaatggggtcgatggttgattattgattagtatattccttattctttttattcacacaaaaagaac : 189100
Seq2 : acctctctaatggggggttgcctgcatactatcgtaatggggtcgatggttgattattgattagtatattccttattctttttattcacacaaaaagaac : 189098
Seq3 : acctctctaatggggggttgcctgcatactatcgtaatggggtcgatggttgattattgattagtatattccttattctttttattcacacaaaaagaac : 189099
Seq4 : acctctctaatggggggttgcctgcatactatcgtaatggggtcgatggttgattattgattagtatattccttattctttttattcacacaaaaagaac : 189098

          *    189120         *    189140         *    189160         *    189180         *    189200
Seq1 : attttataaacatgaaaccactgtctaaatgtaattatgatcttgatttatagatgaagatcagcctttagaggattttaaccagtatgtttaatatga : 189200
Seq2 : attttataaacatgaaaccactgtctaaatgtaattatgatcttgatttatagatgaagatcagcctttagaggattttaaccagtatgtttaatatga : 189198
Seq3 : attttataaacatgaaaccactgtctaaatgtaattatgatcttgatttatagatgaagatcagcctttagaggattttaaccagtatgtttaatatga : 189199
Seq4 : attttataaacatgaaaccactgtctaaatgtaattatgatcttgatttatagatgaagatcagcctttagaggattttaaccagtatgtttaatatga : 189198

          *    189220         *    189240         *    189260         *    189280         *    189300
Seq1 : aaaaaataaacataacatatttgagattaagcgctattgtgcaagattatattagaatcaaattaatctttcatacgagaaaaataacgacatacgtcg : 189300
Seq2 : aaaaaataaacataacatatttgagattaagcgctattgtgcaagattatattagaatcaaattaatctttcatacgagaaaaataacgacatacgtcg : 189298
Seq3 : aaaaaataaacataacatatttgagattaagcgctattgtgcaagattatattagaatcaaattaatctttcatacgagaaaaataacgacatacgtcg : 189299
Seq4 : aaaaaataaacataacatatttgagattaagcgctattgtgcaagattatattagaatcaaattaatctttcatacgagaaaaataacgacatacgtcg : 189298

          *    189320         *    189340         *    189360         *    189380         *    189400
Seq1 : tcaacaaattaaacttttttatttattagttaactagcttatagaacttgctcattgttatgtttctaaaacgggtacgacatataggacaattatccgac : 189400
Seq2 : tcaacaaattaaacttttttatttattagttaactagcttatagaacttgctcattgttatgtttctaaaacgggtacgacatataggacaattatccgac : 189398
Seq3 : tcaacaaattaaacttttttatttattagttaactagcttatagaacttgctcattgttatgtttctaaaacgggtacgacatataggacaattatccgac : 189399
Seq4 : tcaacaaattaaacttttttatttattagttaactagcttatagaacttgctcattgttatgtttctaaaacgggtacgacatataggacaattatccgac : 189398

          *    189420         *    189440         *    189460         *    189480         *    189500
Seq1 : gcaccggtttctcttcgtgttttatgccatatattgatgcatgttatgcaaaatatatgagtacacgaatccaataaaccaaagtatctatcgttttgag : 189500
Seq2 : gcaccggtttctcttcgtgttttatgccatatattgatgcatgttatgcaaaatatatgagtacacgaatccaataaaccaaagtatctatcgttttgag : 189498
Seq3 : gcaccggtttctcttcgtgttttatgccatatattgatgcatgttatgcaaaatatatgagtacacgaatccaataaaccaaagtatctatcgttttgag : 189499
Seq4 : gcaccggtttctcttcgtgttttatgccatatattgatgcatgttatgcaaaatatatgagtacacgaatccaataaaccaaagtatctatcgttttgag : 189498

          *    189520         *    189540         *    189560         *    189580         *    189600
Seq1 : taaacaacttcatagcaaattccacattctttttctttacttactctatacacgtcctcgtatttatccagtattttgatgatatccaactcagaaatgg : 189600
Seq2 : taaacaacttcatagcaaattccacattctttttctttacttactctatacacgtcctcgtatttatccagtattttgatgatatccaactcagaaatgg : 189598
Seq3 : taaacaacttcatagcaaattccacattctttttctttacttactctatacacgtcctcgtatttatccagtattttgatgatatccaactcagaaatgg : 189599
Seq4 : taaacaacttcatagcaaattccacattctttttctttacttactctatacacgtcctcgtatttatccagtattttgatgatatccaactcagaaatgg : 189598

          *    189620         *    189640         *    189660         *    189680         *    189700
Seq1 : ttgttgtattattgggtgtataggtattattagctatgtaccaatttaccaaccctcttaatattgattgaacaatcacatcggttatccaatcaataac : 189700
Seq2 : ttgttgtattattgggtgtataggtattattagctatgtaccaatttaccaaccctcttaatattgattgaacaatcacatcggttatccaatcaataac : 189698
Seq3 : ttgttgtattattgggtgtataggtattattagctatgtaccaatttaccaaccctcttaatattgattgaacaatcacatcggttatccaatcaataac : 189699
Seq4 : ttgttgtattattgggtgtataggtattattagctatgtaccaatttaccaaccctcttaatattgattgaacaatcacatcggttatccaatcaataac : 189698
```

```
                     *    189720         *    189740         *    189760         *    189780         *    189800
Seq1 : cacattaataactaaattgtagtgtatatatagaccatatatgtttctatttttttgacagttacgtatagtttcagtaagttttgattgttgtattcct : 189800
Seq2 : cacattaataactaaattgtagtgtatatatagaccatatatgtttctatttttttgacagttacgtatagtttcagtaagttttgattgttgtattcct : 189798
Seq3 : cacattaataactaaattgtagtgtatatatagaccatatatgtttctatttttttgacagttacgtatagtttcagtaagttttgattgttgtattcct : 189799
Seq4 : cacattaataactaaattgtagtgtatatatagaccatatatgtttctatttttttgacagttacgtatagtttcagtaagttttgattgttgtattcct : 189798

                     *    189820         *    189840         *    189860         *    189880         *    189900
Seq1 : gtatctctagataagttagtcatatagtcccttccggcgatacgtttttttccaagcccgaaattgattagccaaatgtgtatttatttttgtgatattga : 189900
Seq2 : gtatctctagataagttagtcatatagtcccttccggcgatacgtttttttccaagcccgaaattgattagccaaatgtgtatttatttttgtgatattga : 189898
Seq3 : gtatctctagataagttagtcatatagtcccttccggcgatacgtttttttccaagcccgaaattgattagccaaatgtgtatttatttttgtgatattga : 189899
Seq4 : gtatctctagataagttagtcatatagtcccttccggcgatacgtttttttccaagcccgaaattgattagccaaatgtgtatttatttttgtgatattga : 189898

                     *    189920         *    189940         *    189960         *    189980         *    190000
Seq1 : tataatatgtaatgttattaatatttcggataatgcatactgttagtcttatatcatttggttcatctatgtattgtaatattgttacatgatctataga : 190000
Seq2 : tataatatgtaatgttattaatatttcggataatgcatactgttagtcttatatcatttggttcatctatgtattgtaatattgttacatgatctataga : 189998
Seq3 : tataatatgtaatgttattaatatttcggataatgcatactgttagtcttatatcatttggttcatctatgtattgtaatattgttacatgatctataga : 189999
Seq4 : tataatatgtaatgttattaatatttcggataatgcatactgttagtcttatatcatttggttcatctatgtattgtaatattgttacatgatctataga : 189998

                     *    190020         *    190040         *    190060         *    190080         *    190100
Seq1 : tgatgtattgattttggcaggatcgaattccatatccgcgactaaacagtgaaaaaaatgtaaatacttttttaaattttaaattagtaaaacttttttt : 190100
Seq2 : tgatgtattgattttggcaggatcgaattccatatccgcgactaaacagtgaaaaaaatgtaaatacttttttaaattttaaattagtaaaacttttttt : 190098
Seq3 : tgatgtattgattttggcaggatcgaattccatatccgcgactaaacagtgaaaaaaatgtaaatacttttttaaattttaaattagtaaaacttttttt : 190099
Seq4 : tgatgtattgattttggcaggatcgaattccatatccgcgactaaacagtgaaaaaaatgtaaatacttttttaaattttaaattagtaaaacttttttt : 190098

                     *    190120         *    190140         *    190160         *    190180         *    190200
Seq1 : atttttatgattccaaaaatactgaatacaaagtcctaaattataaatatggagatcatactaccacaacttattattatgcatactcagccggtgtaa : 190200
Seq2 : atttttatgattccaaaaatactgaatacaaagtcctaaattataaatatggagatcatactaccacaacttattattatgcatactcagccggtgtaa : 190198
Seq3 : atttttatgattccaaaaatactgaatacaaagtcctaaattataaatatggagatcatactaccacaacttattattatgcatactcagccggtgtaa : 190199
Seq4 : atttttatgattccaaaaatactgaatacaaagtcctaaattataaatatggagatcatactaccacaacttattattatgcatactcagccggtgtaa : 190198

                     *    190220         *    190240         *    190260         *    190280         *    190300
Seq1 : tagatagatatatatataattctattacaccggcagacaattaccgatcggtatttgtcgttaccaacataccgtataatatgtaatatacaattccataac : 190300
Seq2 : tagatagatatatatataattctattacaccggcagacaattaccgatcggtatttgtcgttaccaacataccgtataatatgtaatatacaattccataac : 190298
Seq3 : tagatagatatatatataattctattacaccggcagacaattaccgatcggtatttgtcgttaccaacataccgtataatatgtaatatacaattccataac : 190299
Seq4 : tagatagatatatatataattctattacaccggcagacaattaccgatcggtatttgtcgttaccaacataccgtataatatgtaatatacaattccataac : 190298

                     *    190320         *    190340         *    190360         *    190380         *    190400
Seq1 : ccattgacagttgttatacatcaaaattgcaattcttttgattacgatgttataagaatgtagttaattgatgtatgatgttaatgtgtcctctttcctc : 190400
Seq2 : ccattgacagttgttatacatcaaaattgcaattcttttgattacgatgttataagaatgtagttaattgatgtatgatgttaatgtgtcctctttcctc : 190398
Seq3 : ccattgacagttgttatacatcaaaattgcaattcttttgattacgatgttataagaatgtagttaattgatgtatgatgttaatgtgtcctctttcctc : 190399
Seq4 : ccattgacagttgttatacatcaaaattgcaattcttttgattacgatgttataagaatgtagttaattgatgtatgatgttaatgtgtcctctttcctc : 190398
```

```
              *    190420         *    190440         *    190460         *    190480         *    190500
Seq1 : ttataacatcgtaatcaaaaacttttttataatatatacctaataatgtgtcttaatagttctcgtgattcgtcaaacaatcattcttataaaatataat : 190500
Seq2 : ttataacatcgtaatcaaaaacttttttataatatatacctaataatgtgtcttaatagttctcgtgattcgtcaaacaatcattcttataaaatataat : 190498
Seq3 : ttataacatcgtaatcaaaaacttttttataatatatacctaataatgtgtcttaatagttctcgtgattcgtcaaacaatcattcttataaaatataat : 190499
Seq4 : ttataacatcgtaatcaaaaacttttttataatatatacctaataatgtgtcttaatagttctcgtgattcgtcaaacaatcattcttataaaatataat : 190498

              *    190520         *    190540         *    190560         *    190580         *    190600
Seq1 : aaagcaacgtaaaacacataaaaataagcgtaactaataagacaatggatatttacgacgataaaggtctacagactattaaactgtttaataatgaatt : 190600
Seq2 : aaagcaacgtaaaacacataaaaataagcgtaactaataagacaatggatatttacgacgataaaggtctacagactattaaactgtttaataatgaatt : 190598
Seq3 : aaagcaacgtaaaacacataaaaataagcgtaactaataagacaatggatatttacgacgataaaggtctacagactattaaactgtttaataatgaatt : 190599
Seq4 : aaagcaacgtaaaacacataaaaataagcgtaactaataagacaatggatatttacgacgataaaggtctacagactattaaactgtttaataatgaatt : 190598

              *    190620         *    190640         *    190660         *    190680         *    190700
Seq1 : tgattgtataaggaatgacatcagagaattatttaaacatgtaactgattccgatagtatacaacttccgatggaagacaattctgatattatagaaaat : 190700
Seq2 : tgattgtataaggaatgacatcagagaattatttaaacatgtaactgattccgatagtatacaacttccgatggaagacaattctgatattatagaaaat : 190698
Seq3 : tgattgtataaggaatgacatcagagaattatttaaacatgtaactgattccgatagtatacaacttccgatggaagacaattctgatattatagaaaat : 190699
Seq4 : tgattgtataaggaatgacatcagagaattatttaaacatgtaactgattccgatagtatacaacttccgatggaagacaattctgatattatagaaaat : 190698

              *    190720         *    190740         *    190760         *    190780         *    190800
Seq1 : atcagaaaaatactatatagacgattaaaaaatgtagaatgtgttgacatcgatagtacaataacttttatgaaatacgatccaaatgatgataataagc : 190800
Seq2 : atcagaaaaatactatatagacgattaaaaaatgtagaatgtgttgacatcgatagtacaataacttttatgaaatacgatccaaatgatgataataagc : 190798
Seq3 : atcagaaaaatactatatagacgattaaaaaatgtagaatgtgttgacatcgatagtacaataacttttatgaaatacgatccaaatgatgataataagc : 190799
Seq4 : atcagaaaaatactatatagacgattaaaaaatgtagaatgtgttgacatcgatagtacaataacttttatgaaatacgatccaaatgatgataataagc : 190798

              *    190820         *    190840         *    190860         *    190880         *    190900
Seq1 : gtacgtgttctaattgggtacccttaactaataactatatggaatattgtctagtaatatatttggaaacaccgatatgtggaggcaaaataaaattata : 190900
Seq2 : gtacgtgttctaattgggtacccttaactaataactatatggaatattgtctagtaatatatttggaaacaccgatatgtggaggcaaaataaaattata : 190898
Seq3 : gtacgtgttctaattgggtacccttaactaataactatatggaatattgtctagtaatatatttggaaacaccgatatgtggaggcaaaataaaattata : 190899
Seq4 : gtacgtgttctaattgggtacccttaactaataactatatggaatattgtctagtaatatatttggaaacaccgatatgtggaggcaaaataaaattata : 190898

              *    190920         *    190940         *    190960         *    190980         *    191000
Seq1 : ccaccctacaggaaatataaagtcggataaggatattatgtttgcaaagactctagactttaaatcaaagaaagtgttaactggacgtaaaacaattgcc : 191000
Seq2 : ccaccctacaggaaatataaagtcggataaggatattatgtttgcaaagactctagactttaaatcaaagaaagtgttaactggacgtaaaacaattgcc : 190998
Seq3 : ccaccctacaggaaatataaagtcggataaggatattatgtttgcaaagactctagactttaaatcaaagaaagtgttaactggacgtaaaacaattgcc : 190999
Seq4 : ccaccctacaggaaatataaagtcggataaggatattatgtttgcaaagactctagactttaaatcaaagaaagtgttaactggacgtaaaacaattgcc : 190998

              *    191020         *    191040         *    191060         *    191080         *    191100
Seq1 : gttctagacatatccgtttcatataatagatcaatgactactattcactacaacgacgacgttgatatagatatacatactgataaaaatggaaaagagt : 191100
Seq2 : gttctagacatatccgtttcatataatagatcaatgactactattcactacaacgacgacgttgatatagatatacatactgataaaaatggaaaagagt : 191098
Seq3 : gttctagacatatccgtttcatataatagatcaatgactactattcactacaacgacgacgttgatatagatatacatactgataaaaatggaaaagagt : 191099
Seq4 : gttctagacatatccgtttcatataatagatcaatgactactattcactacaacgacgacgttgatatagatatacatactgataaaaatggaaaagagt : 191098
```

```
                 *    191120          *    191140          *    191160          *    191180          *    191200
Seq1 : tatgttattgttatataacaatagatgatcattacttggttgatgtggaaactataggagttatagtcaatagatctggaaaatgtctgttagtaaataa : 191200
Seq2 : tatgttattgttatataacaatagatgatcattacttggttgatgtggaaactataggagttatagtcaatagatctggaaaatgtctgttagtaaataa : 191198
Seq3 : tatgttattgttatataacaatagatgatcattacttggttgatgtggaaactataggagttatagtcaatagatctggaaaatgtctgttagtaaataa : 191199
Seq4 : tatgttattgttatataacaatagatgatcattacttggttgatgtggaaactataggagttatagtcaatagatctggaaaatgtctgttagtaaataa : 191198

                 *    191220          *    191240          *    191260          *    191280          *    191300
Seq1 : ccatctaggtataggtatcgttaaagataaacgtataagcgatagttttggagatgtatgtatggatacaatatttgacttttctgaagcacgagagtta : 191300
Seq2 : ccatctaggtataggtatcgttaaagataaacgtataagcgatagttttggagatgtatgtatggatacaatatttgacttttctgaagcacgagagtta : 191298
Seq3 : ccatctaggtataggtatcgttaaagataaacgtataagcgatagttttggagatgtatgtatggatacaatatttgacttttctgaagcacgagagtta : 191299
Seq4 : ccatctaggtataggtatcgttaaagataaacgtataagcgatagttttggagatgtatgtatggatacaatatttgacttttctgaagcacgagagtta : 191298

                 *    191320          *    191340          *    191360          *    191380          *    191400
Seq1 : ttttcattaactaatgatgataacaggaatatagcatgggacactgataaactagacgatgatacagatatatggactcccgtcacagaagatgattaca : 191400
Seq2 : ttttcattaactaatgatgataacaggaatatagcatgggacactgataaactagacgatgatacagatatatggactcccgtcacagaagatgattaca : 191398
Seq3 : ttttcattaactaatgatgataacaggaatatagcatgggacactgataaactagacgatgatacagatatatggactcccgtcacagaagatgattaca : 191399
Seq4 : ttttcattaactaatgatgataacaggaatatagcatgggacactgataaactagacgatgatacagatatatggactcccgtcacagaagatgattaca : 191398

                 *    191420          *    191440          *    191460          *    191480          *    191500
Seq1 : aatttctttctagactagtattgtatgcaaaatctcaatcggatactgtattcgactattatgttcttactggtgatacggaaccacccactgtattcat : 191500
Seq2 : aatttctttctagactagtattgtatgcaaaatctcaatcggatactgtattcgactattatgttcttactggtgatacggaaccacccactgtattcat : 191498
Seq3 : aatttctttctagactagtattgtatgcaaaatctcaatcggatactgtattcgactattatgttcttactggtgatacggaaccacccactgtattcat : 191499
Seq4 : aatttctttctagactagtattgtatgcaaaatctcaatcggatactgtattcgactattatgttcttactggtgatacggaaccacccactgtattcat : 191498

                 *    191520          *    191540          *    191560          *    191580          *    191600
Seq1 : tttcaaggtaactagattttactttaatatgccgaaataaaaaatttttgtataatatctagaggtagaggtattgtttagataaatacaaataacatag : 191600
Seq2 : tttcaaggtaactagattttactttaatatgccgaaataaaaaatttttgtataatatctagaggtagaggtattgtttagataaatacaaataacatag : 191598
Seq3 : tttcaaggtaactagattttactttaatatgccgaaataaaaaatttttgtataatatctagaggtagaggtattgtttagataaatacaaataacatag : 191599
Seq4 : tttcaaggtaactagattttactttaatatgccgaaataaaaaatttttgtataatatctagaggtagaggtattgtttagataaatacaaataacatag : 191598

                 *    191620          *    191640          *    191660          *    191680          *    191700
Seq1 : atacatcgcatacttagcattttataaatatacataagacatacactttatacatttttttttgtaaaaatactcataaaaaaaatttataaaaattatgg : 191700
Seq2 : atacatcgcatacttagcattttataaatatacataagacatacactttatacatttttttttgtaaaaatactcataaaaaaaatttataaaaattatgg : 191698
Seq3 : atacatcgcatacttagcattttataaatatacataagacatacactttatacatttttttttgtaaaaatactcataaaaaaaatttataaaaattatgg : 191699
Seq4 : atacatcgcatacttagcattttataaatatacataagacatacactttatacatttttttttgtaaaaatactcataaaaaaaatttataaaaattatgg : 191698

                 *    191720          *    191740          *    191760          *    191780          *    191800
Seq1 : cacaaccatatcttgtataggtagtttagttcgtcgagtgaacctataaacagataatagacaacacgtaataataataatgcctactaatacaagcata : 191800
Seq2 : cacaaccatatcttgtataggtagtttagttcgtcgagtgaacctataaacagataatagacaacacgtaataataataatgcctactaatacaagcata : 191798
Seq3 : cacaaccatatcttgtataggtagtttagttcgtcgagtgaacctataaacagataatagacaacacgtaataataataatgcctactaatacaagcata : 191799
Seq4 : cacaaccatatcttgtataggtagtttagttcgtcgagtgaacctataaacagataatagacaacacgtaataataataatgcctactaatacaagcata : 191798
```

```
                 *    191820          *    191840          *    191860          *    191880          *    191900
Seq1 : ataccgggagatgggatatatgacgttgtagtgtttgggttttctgaacgttgatagtctactaatactacatgctgacatctaatgcctgtataaccat : 191900
Seq2 : ataccgggagatgggatatatgacgttgtagtgtttgggttttctgaacgttgatagtctactaatactacatgctgacatctaatgcctgtataaccat : 191898
Seq3 : ataccgggagatgggatatatgacgttgtagtgtttgggttttctgaacgttgatagtctactaatactacatgctgacatctaatgcctgtataaccat : 191899
Seq4 : ataccgggagatgggatatatgacgttgtagtgtttgggttttctgaacgttgatagtctactaatactacatgctgacatctaatgcctgtataaccat : 191898

                 *    191920          *    191940          *    191960          *    191980          *    192000
Seq1 : gagagcatctacaatacataccgtcaatatctctagcgtggatacagtcaccgtgtaaacaatatccatctccctctggaccgcataatctgatagctgg : 192000
Seq2 : gagagcatctacaatacataccgtcaatatctctagcgtggatacagtcaccgtgtaaacaatatccatctccctctggaccgcataatctgatagctgg : 191998
Seq3 : gagagcatctacaatacataccgtcaatatctctagcgtggatacagtcaccgtgtaaacaatatccatctccctctggaccgcataatctgatagctgg : 191999
Seq4 : gagagcatctacaatacataccgtcaatatctctagcgtggatacagtcaccgtgtaaacaatatccatctccctctggaccgcataatctgatagctgg : 191998

                 *    192020          *    192040          *    192060          *    192080          *    192100
Seq1 : aatatctgttgtagcgtttgtaatttctggcgatgtcgtttcgatagcgttaccactatcggcgaatgatctgattatcatagcagcgaacaacaacatc : 192100
Seq2 : aatatctgttgtagcgtttgtaatttctggcgatgtcgtttcgatagcgttaccactatcggcgaatgatctgattatcatagcagcgaacaacaacatc : 192098
Seq3 : aatatctgttgtagcgtttgtaatttctggcgatgtcgtttcgatagcgttaccactatcggcgaatgatctgattatcatagcagcgaacaacaacatc : 192099
Seq4 : aatatctgttgtagcgtttgtaatttctggcgatgtcgtttcgatagcgttaccactatcggcgaatgatctgattatcatagcagcgaacaacaacatc : 192098

                 *    192120          *    192140          *    192160          *    192180          *    192200
Seq1 : agatatttcatcgacattttgatggattttgtgtttatgctgtttctcagtgtgtgtttatgacaagattgggaattttatattattaattcagtaata : 192200
Seq2 : agatatttcatcgacattttgatggattttgtgtttatgctgtttctcagtgtgtgtttatgacaagattgggaattttatattattaattcagtaata : 192198
Seq3 : agatatttcatcgacattttgatggattttgtgtttatgctgtttctcagtgtgtgtttatgacaagattgggaattttatattattaattcagtaata : 192199
Seq4 : agatatttcatcgacattttgatggattttgtgtttatgctgtttctcagtgtgtgtttatgacaagattgggaattttatattattaattcagtaata : 192198

                 *    192220          *    192240          *    192260          *    192280          *    192300
Seq1 : taaactaataatatattgttaattgtgtaaataatataaaaataacaatacaatattgaatgtgttgctgttaaaaatgatcataaacacggagtttatt : 192300
Seq2 : taaactaataatatattgttaattgtgtaaataatataaaaataacaatacaatattgaatgtgttgctgttaaaaatgatcataaacacggagtttatt : 192298
Seq3 : taaactaataatatattgttaattgtgtaaataatataaaaataacaatacaatattgaatgtgttgctgttaaaaatgatcataaacacggagtttatt : 192299
Seq4 : taaactaataatatattgttaattgtgtaaataatataaaaataacaatacaatattgaatgtgttgctgttaaaaatgatcataaacacggagtttatt : 192298

                 *    192320          *    192340          *    192360          *    192380          *    192400
Seq1 : ttatatgtctcgcataaacattactaaaaaaatatattgttctgtttttctttcacatctttaattatgaaaaagtaaatcattatgagatggacgagat : 192400
Seq2 : ttatatgtctcgcataaacattactaaaaaaatatattgttctgtttttctttcacatctttaattatgaaaaagtaaatcattatgagatggacgagat : 192398
Seq3 : ttatatgtctcgcataaacattactaaaaaaatatattgttctgtttttctttcacatctttaattatgaaaaagtaaatcattatgagatggacgagat : 192399
Seq4 : ttatatgtctcgcataaacattactaaaaaaatatattgttctgtttttctttcacatctttaattatgaaaaagtaaatcattatgagatggacgagat : 192398

                 *    192420          *    192440          *    192460          *    192480          *    192500
Seq1 : tgtacgcatcgttcgcgacagtatgtggtacatacctaacgtatttatggacgacggtaagaatgaaggtcacgtttctgtcaacaatgtctgtcatatg : 192500
Seq2 : tgtacgcatcgttcgcgacagtatgtggtacatacctaacgtatttatggacgacggtaagaatgaaggtcacgtttctgtcaacaatgtctgtcatatg : 192498
Seq3 : tgtacgcatcgttcgcgacagtatgtggtacatacctaacgtatttatggacgacggtaagaatgaaggtcacgtttctgtcaacaatgtctgtcatatg : 192499
Seq4 : tgtacgcatcgttcgcgacagtatgtggtacatacctaacgtatttatggacgacggtaagaatgaaggtcacgtttctgtcaacaatgtctgtcatatg : 192498
```

```
                *    192520         *    192540         *    192560         *    192580         *    192600
Seq1 : tatttcacgttctttgatgtggatacatcgtctcatctgtttaagctagttattaaacactgcgatctgaataaacgaggtaactctccattacattgct : 192600
Seq2 : tatttcacgttctttgatgtggatacatcgtctcatctgtttaagctagttattaaacactgcgatctgaataaacgaggtaactctccattacattgct : 192598
Seq3 : tatttcacgttctttgatgtggatacatcgtctcatctgtttaagctagttattaaacactgcgatctgaataaacgaggtaactctccattacattgct : 192599
Seq4 : tatttcacgttctttgatgtggatacatcgtctcatctgtttaagctagttattaaacactgcgatctgaataaacgaggtaactctccattacattgct : 192598

                *    192620         *    192640         *    192660         *    192680         *    192700
Seq1 : atacgatgaatacacgatttaatccatctgtattaaagatattgttacaccacggcatgcgtaactttgatagcaaggatgaaaaaggacaccactatca : 192700
Seq2 : atacgatgaatacacgatttaatccatctgtattaaagatattgttacaccacggcatgcgtaactttgatagcaaggatgaaaaaggacaccactatca : 192698
Seq3 : atacgatgaatacacgatttaatccatctgtattaaagatattgttacaccacggcatgcgtaactttgatagcaaggatgaaaaaggacaccactatca : 192699
Seq4 : atacgatgaatacacgatttaatccatctgtattaaagatattgttacaccacggcatgcgtaactttgatagcaaggatgaaaaaggacaccactatca : 192698

                *    192720         *    192740         *    192760         *    192780         *    192800
Seq1 : atcgataacaagatctttgatatactaacggacaccattgatgactttagtaaatcatccgatctattgctgtgttatcttagatataaattcaatggga : 192800
Seq2 : atcgataacaagatctttgatatactaacggacaccattgatgactttagtaaatcatccgatctattgctgtgttatcttagatataaattcaatggga : 192798
Seq3 : atcgataacaagatctttgatatactaacggacaccattgatgactttagtaaatcatccgatctattgctgtgttatcttagatataaattcaatggga : 192799
Seq4 : atcgataacaagatctttgatatactaacggacaccattgatgactttagtaaatcatccgatctattgctgtgttatcttagatataaattcaatggga : 192798

                *    192820         *    192840         *    192860         *    192880         *    192900
Seq1 : gcttaaactattacgttctgtacaaaggatccgacgaggatgaactcacttctcttcattactactgtaaacacatatccacgttctacaaaagcaatta : 192900
Seq2 : gcttaaactattacgttctgtacaaaggatccgacgaggatgaactcacttctcttcattactactgtaaacacatatccacgttctacaaaagcaatta : 192898
Seq3 : gcttaaactattacgttctgtacaaaggatccgacgaggatgaactcacttctcttcattactactgtaaacacatatccacgttctacaaaagcaatta : 192899
Seq4 : gcttaaactattacgttctgtacaaaggatccgacgaggatgaactcacttctcttcattactactgtaaacacatatccacgttctacaaaagcaatta : 192898

                *    192920         *    192940         *    192960         *    192980         *    193000
Seq1 : ttacaagttaagtcacactaagatgcgagccgagaagcgattcatctacgcgataatagattatggagcaaacattaacgcggttacacacttaccttca : 193000
Seq2 : ttacaagttaagtcacactaagatgcgagccgagaagcgattcatctacgcgataatagattatggagcaaacattaacgcggttacacacttaccttca : 192998
Seq3 : ttacaagttaagtcacactaagatgcgagccgagaagcgattcatctacgcgataatagattatggagcaaacattaacgcggttacacacttaccttca : 192999
Seq4 : ttacaagttaagtcacactaagatgcgagccgagaagcgattcatctacgcgataatagattatggagcaaacattaacgcggttacacacttaccttca : 192998

                *    193020         *    193040         *    193060         *    193080         *    193100
Seq1 : acagtataccaaacatagtcctcgtgtggtgtatgctcttttatctcgaggagccgatacgaggatacgtaataatcttgattgtacacccatcatggaa : 193100
Seq2 : acagtataccaaacatagtcctcgtgtggtgtatgctcttttatctcgaggagccgatacgaggatacgtaataatcttgattgtacacccatcatggaa : 193098
Seq3 : acagtataccaaacatagtcctcgtgtggtgtatgctcttttatctcgaggagccgatacgaggatacgtaataatcttgattgtacacccatcatggaa : 193099
Seq4 : acagtataccaaacatagtcctcgtgtggtgtatgctcttttatctcgaggagccgatacgaggatacgtaataatcttgattgtacacccatcatggaa : 193098

                *    193120         *    193140         *    193160         *    193180         *    193200
Seq1 : cgattgtgcaacaggtcatattctcataatgttactcaattggcacgaacaaaaggaagaaggacaacatctactttatctattcataaaacataatcaa : 193200
Seq2 : cgattgtgcaacaggtcatattctcataatgttactcaattggcacgaacaaaaggaagaaggacaacatctactttatctattcataaaacataatcaa : 193198
Seq3 : cgattgtgcaacaggtcatattctcataatgttactcaattggcacgaacaaaaggaagaaggacaacatctactttatctattcataaaacataatcaa : 193199
Seq4 : cgattgtgcaacaggtcatattctcataatgttactcaattggcacgaacaaaaggaagaaggacaacatctactttatctattcataaaacataatcaa : 193198
```

```
               *    193220         *    193240         *    193260         *    193280         *    193300
Seq1 : ggatacactctcaatatactacggtatctactagataggttcgacattcagaaagacgaatactataataccgcctttcaaaattgtaacaacaatgttg : 193300
Seq2 : ggatacactctcaatatactacggtatctactagataggttcgacattcagaaagacgaatactataataccgcctttcaaaattgtaacaacaatgttg : 193298
Seq3 : ggatacactctcaatatactacggtatctactagataggttcgacattcagaaagacgaatactataataccgcctttcaaaattgtaacaacaatgttg : 193299
Seq4 : ggatacactctcaatatactacggtatctactagataggttcgacattcagaaagacgaatactataataccgcctttcaaaattgtaacaacaatgttg : 193298

               *    193320         *    193340         *    193360         *    193380         *    193400
Seq1 : cctcatacatcggatacgacatcaaccttccgactaaagacggtattcgacttggtgtttgaaaacagaaacatcatatacaaggcggatgttgtgaatg : 193400
Seq2 : cctcatacatcggatacgacatcaaccttccgactaaagacggtattcgacttggtgtttgaaaacagaaacatcatatacaaggcggatgttgtgaatg : 193398
Seq3 : cctcatacatcggatacgacatcaaccttccgactaaagacggtattcgacttggtgtttgaaaacagaaacatcatatacaaggcggatgttgtgaatg : 193399
Seq4 : cctcatacatcggatacgacatcaaccttccgactaaagacggtattcgacttggtgtttgaaaacagaaacatcatatacaaggcggatgttgtgaatg : 193398

               *    193420         *    193440         *    193460         *    193480         *    193500
Seq1 : acatcatccaccacagactgaaagtatctctacctatgattaaatcgttgttctacaagatgtctctccctacgacgattactacgtaaagaagataata : 193500
Seq2 : acatcatccaccacagactgaaagtatctctacctatgattaaatcgttgttctacaagatgtctctccctacgacgattactacgtaaagaagataata : 193498
Seq3 : acatcatccaccacagactgaaagtatctctacctatgattaaatcgttgttctacaagatgtctctccctacgacgattactacgtaaagaagataata : 193499
Seq4 : acatcatccaccacagactgaaagtatctctacctatgattaaatcgttgttctacaagatgtctctccctacgacgattactacgtaaagaagataata : 193498

               *    193520         *    193540         *    193560         *    193580         *    193600
Seq1 : gcctactgcctattaagggacgagtcattcgcggaactacatagtaaattctgtttaaacgaggactataaaagtgtatttatgaaaaatatatcattcg : 193600
Seq2 : gcctactgcctattaagggacgagtcattcgcggaactacatagtaaattctgtttaaacgaggactataaaagtgtatttatgaaaaatatatcattcg : 193598
Seq3 : gcctactgcctattaagggacgagtcattcgcggaactacatagtaaattctgtttaaacgaggactataaaagtgtatttatgaaaaatatatcattcg : 193599
Seq4 : gcctactgcctattaagggacgagtcattcgcggaactacatagtaaattctgtttaaacgaggactataaaagtgtatttatgaaaaatatatcattcg : 193598

               *    193620         *    193640         *    193660         *    193680         *    193700
Seq1 : ataagatagattccatcatcgtgacataagtcgccttaaagagattcgaatctccgacaccgacctgtatacggtatcacagctatcttaaagccataca : 193700
Seq2 : ataagatagattccatcatcgtgacataagtcgccttaaagagattcgaatctccgacaccgacctgtatacggtatcacagctatcttaaagccataca : 193698
Seq3 : ataagatagattccatcatcgtgacataagtcgccttaaagagattcgaatctccgacaccgacctgtatacggtatcacagctatcttaaagccataca : 193699
Seq4 : ataagatagattccatcatcgtgacataagtcgccttaaagagattcgaatctccgacaccgacctgtatacggtatcacagctatcttaaagccataca : 193698

               *    193720         *    193740         *    193760         *    193780         *    193800
Seq1 : ttcagacagtcacatttcatttcccatgtacgacgatctcatagaacagtgccatctatcgatggagcgtaaaagtaaactcgtcgacaaagcactcaat : 193800
Seq2 : ttcagacagtcacatttcatttcccatgtacgacgatctcatagaacagtgccatctatcgatggagcgtaaaagtaaactcgtcgacaaagcactcaat : 193798
Seq3 : ttcagacagtcacatttcatttcccatgtacgacgatctcatagaacagtgccatctatcgatggagcgtaaaagtaaactcgtcgacaaagcactcaat : 193799
Seq4 : ttcagacagtcacatttcatttcccatgtacgacgatctcatagaacagtgccatctatcgatggagcgtaaaagtaaactcgtcgacaaagcactcaat : 193798

               *    193820         *    193840         *    193860         *    193880         *    193900
Seq1 : aaattagagtctaccatcggtcaatctagactatcgtatttgcctccggaaattatgcgcaatatcatctaaacagtatgttgtacgaaaagaaccatta : 193900
Seq2 : aaattagagtctaccatcggtcaatctagactatcgtatttgcctccggaaattatgcgcaatatcatctaaacagtatgttgtacgaaaagaaccatta : 193898
Seq3 : aaattagagtctaccatcggtcaatctagactatcgtatttgcctccggaaattatgcgcaatatcatctaaacagtatgttgtacgaaaagaaccatta : 193899
Seq4 : aaattagagtctaccatcggtcaatctagactatcgtatttgcctccggaaattatgcgcaatatcatctaaacagtatgttgtacgaaaagaaccatta : 193898
```

```
               *    193920         *    193940         *    193960         *    193980         *    194000
Seq1 : caaatattatccatgatagaaagaaaatatctatatgattggagaagtaggaaacaggaacaagacgacgattactacattattaaatcatgaagtccgt : 194000
Seq2 : caaatattatccatgatagaaagaaaatatctatatgattggagaagtaggaaacaggaacaagacgacgattactacattattaaatcatgaagtccgt : 193998
Seq3 : caaatattatccatgatagaaagaaaatatctatatgattggagaagtaggaaacaggaacaagacgacgattactacattattaaatcatgaagtccgt : 193999
Seq4 : caaatattatccatgatagaaagaaaatatctatatgattggagaagtaggaaacaggaacaagacgacgattactacattattaaatcatgaagtccgt : 193998

               *    194020         *    194040         *    194060         *    194080         *    194100
Seq1 : attatactcgtatatattgtttctctcatgtataataataaacggaagagatatagcaccgcatgcaccatccgatggaaagtgtaaagacaacgaatac : 194100
Seq2 : attatactcgtatatattgtttctctcatgtataataataaacggaagagatatagcaccgcatgcaccatccgatggaaagtgtaaagacaacgaatac : 194098
Seq3 : attatactcgtatatattgtttctctcatgtataataataaacggaagagatatagcaccgcatgcaccatccgatggaaagtgtaaagacaacgaatac : 194099
Seq4 : attatactcgtatatattgtttctctcatgtataataataaacggaagagatatagcaccgcatgcaccatccgatggaaagtgtaaagacaacgaatac : 194098

               *    194120         *    194140         *    194160         *    194180         *    194200
Seq1 : aaacgccataatttgtgtccgggaacatacgcttccagattatgcgatagcaagactaacacacaatgtacgccgtgtggttcgggtaccttcacatctc : 194200
Seq2 : aaacgccataatttgtgtccgggaacatacgcttccagattatgcgatagcaagactaacacacaatgtacgccgtgtggttcgggtaccttcacatctc : 194198
Seq3 : aaacgccataatttgtgtccgggaacatacgcttccagattatgcgatagcaagactaacacacaatgtacgccgtgtggttcgggtaccttcacatctc : 194199
Seq4 : aaacgccataatttgtgtccgggaacatacgcttccagattatgcgatagcaagactaacacacaatgtacgccgtgtggttcgggtaccttcacatctc : 194198

               *    194220         *    194240         *    194260         *    194280         *    194300
Seq1 : gcaataatcatttacccgcttgtctaagttgtaacggaagacgcgaccgtgtaacacgactcacaatagaatctgtgaatgctctcccggatattattgt : 194300
Seq2 : gcaataatcatttacccgcttgtctaagttgtaacggaagacgcgaccgtgtaacacgactcacaatagaatctgtgaatgctctcccggatattattgt : 194298
Seq3 : gcaataatcatttacccgcttgtctaagttgtaacggaagacgcgaccgtgtaacacgactcacaatagaatctgtgaatgctctcccggatattattgt : 194299
Seq4 : gcaataatcatttacccgcttgtctaagttgtaacggaagacgcgaccgtgtaacacgactcacaatagaatctgtgaatgctctcccggatattattgt : 194298

               *    194320         *    194340         *    194360         *    194380         *    194400
Seq1 : cttctcaaaggatcatccggatgcaaggcatgtgtttcccaaacaaaatgtggaataggatacggagtatccggagacgtcatctgttctccgtgtggtc : 194400
Seq2 : cttctcaaaggatcatccggatgcaaggcatgtgtttcccaaacaaaatgtggaataggatacggagtatccggagacgtcatctgttctccgtgtggtc : 194398
Seq3 : cttctcaaaggatcatccggatgcaaggcatgtgtttcccaaacaaaatgtggaataggatacggagtatccggagacgtcatctgttctccgtgtggtc : 194399
Seq4 : cttctcaaaggatcatccggatgcaaggcatgtgtttcccaaacaaaatgtggaataggatacggagtatccggagacgtcatctgttctccgtgtggtc : 194398

               *    194420         *    194440         *    194460         *    194480         *    194500
Seq1 : tcggaacatattctcacaccgtctcttccgcagataaatgcgaacccgtacccagtaatacctttaactatatcgatgtggaaattaatctgtatccagt : 194500
Seq2 : tcggaacatattctcacaccgtctcttccgcagataaatgcgaacccgtacccagtaatacctttaactatatcgatgtggaaattaatctgtatccagt : 194498
Seq3 : tcggaacatattctcacaccgtctcttccgcagataaatgcgaacccgtacccagtaatacctttaactatatcgatgtggaaattaatctgtatccagt : 194499
Seq4 : tcggaacatattctcacaccgtctcttccgcagataaatgcgaacccgtacccagtaatacctttaactatatcgatgtggaaattaatctgtatccagt : 194498

               *    194520         *    194540         *    194560         *    194580         *    194600
Seq1 : taacgaccactaccggtctcagcgaatccatctcaacgtcggaactaactattactatgaatcataaagactgtaatcccgtatttcgtgaggaatactt : 194600
Seq2 : taacgaccactaccggtctcagcgaatccatctcaacgtcggaactaactattactatgaatcataaagactgtaatcccgtatttcgtgaggaatactt : 194598
Seq3 : taacgaccactaccggtctcagcgaatccatctcaacgtcggaactaactattactatgaatcataaagactgtaatcccgtatttcgtgaggaatactt : 194599
Seq4 : taacgaccactaccggtctcagcgaatccatctcaacgtcggaactaactattactatgaatcataaagactgtaatcccgtatttcgtgaggaatactt : 194598
```

```
                 *    194620         *    194640         *    194660         *    194680         *    194700
Seq1 : ctccgtccttaataaggtagcaacttcaggtttctttacaggagaaaggtgtgcactctgaatttcgagattaaatgcaataacaaaaattttcctcca : 194700
Seq2 : ctccgtccttaataaggtagcaacttcaggtttctttacaggagaaaggtgtgcactctgaatttcgagattaaatgcaataacaaaaattttcctcca : 194698
Seq3 : ctccgtccttaataaggtagcaacttcaggtttctttacaggagaaaggtgtgcactctgaatttcgagattaaatgcaataacaaaaattttcctcca : 194699
Seq4 : ctccgtccttaataaggtagcaacttcaggtttctttacaggagaaaggtgtgcactctgaatttcgagattaaatgcaataacaaaaattttcctcca : 194698

                 *    194720         *    194740         *    194760         *    194780         *    194800
Seq1 : aacagttaacgaaagcaaagaatgatgacggtatcatgccgcattcggagactgtctatctagcgtcgacatctatatactatatagtaataccaatact : 194800
Seq2 : aacagttaacgaaagcaaagaatgatgacggtatcatgccgcattcggagactgtctatctagcgtcgacatctatatactatatagtaataccaatact : 194798
Seq3 : aacagttaacgaaagcaaagaatgatgacggtatcatgccgcattcggagactgtctatctagcgtcgacatctatatactatatagtaataccaatact : 194799
Seq4 : aacagttaacgaaagcaaagaatgatgacggtatcatgccgcattcggagactgtctatctagcgtcgacatctatatactatatagtaataccaatact : 194798

                 *    194820         *    194840         *    194860         *    194880         *    194900
Seq1 : caagactacgaaactgatacaatctcttatcatgtgggtaatgttctcgatgtcgatagccatatgcccggtagttgcgatatacataaactgatcacta : 194900
Seq2 : caagactacgaaactgatacaatctcttatcatgtgggtaatgttctcgatgtcgatagccatatgcccggtagttgcgatatacataaactgatcacta : 194898
Seq3 : caagactacgaaactgatacaatctcttatcatgtgggtaatgttctcgatgtcgatagccatatgcccggtagttgcgatatacataaactgatcacta : 194899
Seq4 : caagactacgaaactgatacaatctcttatcatgtgggtaatgttctcgatgtcgatagccatatgcccggtagttgcgatatacataaactgatcacta : 194898

                 *    194920         *    194940         *    194960         *    194980         *    195000
Seq1 : attccaaacccacccgcttttatagtaagtttttcacccataaataataaatacaataattaatttctcgtaaaagtagaaaatatattctaatttatt : 195000
Seq2 : attccaaacccacccgcttttatagtaagtttttcacccataaataataaatacaataattaatttctcgtaaaagtagaaaatatattctaatttatt : 194998
Seq3 : attccaaacccacccgcttttatagtaagtttttcacccataaataataaatacaataattaatttctcgtaaaagtagaaaatatattctaatttatt : 194999
Seq4 : attccaaacccacccgcttttatagtaagtttttcacccataaataataaatacaataattaatttctcgtaaaagtagaaaatatattctaatttatt : 194998

                 *    195020         *    195040         *    195060         *    195080         *    195100
Seq1 : gcacggtaaggaagtagaatcataaagaacagtactcaatcaatagcaatcatgaaacaatatatcgtcctggcatgcatgtgcctgccagtcttcagca : 195100
Seq2 : gcacggtaaggaagtagaatcataaagaacagtactcaatcaatagcaatcatgaaacaatatatcgtcctggcatgcatgtgcctgccagtcttcagca : 195098
Seq3 : gcacggtaaggaagtagaatcataaagaacagtactcaatcaatagcaatcatgaaacaatatatcgtcctggcatgcatgtgcctgccagtcttcagca : 195099
Seq4 : gcacggtaaggaagtagaatcataaagaacagtactcaatcaatagcaatcatgaaacaatatatcgtcctggcatgcatgtgcctgccagtcttcagca : 195098

                 *    195120         *    195140         *    195160         *    195180         *    195200
Seq1 : atcatcctcatcctcctcctcgtgtacggaagaagaaaacaaacatcatatgggaatcgatgttattatcaaagtcacaaagcaagaccaaacaccgacc : 195200
Seq2 : atcatcctcatcctcctcctcgtgtacggaagaagaaaacaaacatcatatgggaatcgatgttattatcaaagtcacaaagcaagaccaaacaccgacc : 195198
Seq3 : atcatcctcatcctcctcctcgtgtacggaagaagaaaacaaacatcatatgggaatcgatgttattatcaaagtcacaaagcaagaccaaacaccgacc : 195199
Seq4 : atcatcctcatcctcctcctcgtgtacggaagaagaaaacaaacatcatatgggaatcgatgttattatcaaagtcacaaagcaagaccaaacaccgacc : 195198

                 *    195220         *    195240         *    195260         *    195280         *    195300
Seq1 : aatgataagatttgccaatccgtaacggaaattacagagtccgagtcagatccagatcccgaggtggaatcagaagatgattccacatcagtcgaggatg : 195300
Seq2 : aatgataagatttgccaatccgtaacggaaattacagagtccgagtcagatccagatcccgaggtggaatcagaagatgattccacatcagtcgaggatg : 195298
Seq3 : aatgataagatttgccaatccgtaacggaaattacagagtccgagtcagatccagatcccgaggtggaatcagaagatgattccacatcagtcgaggatg : 195299
Seq4 : aatgataagatttgccaatccgtaacggaaattacagagtccgagtcagatccagatcccgaggtggaatcagaagatgattccacatcagtcgaggatg : 195298
```

```
                 *    195320          *    195340          *    195360          *    195380          *    195400
Seq1 : tagatcctcctaccacttattactccatcatcggtggaggtctgagaatgaactttggattcaccaaatgtcctcagattaaatccatctcagaatccgc : 195400
Seq2 : tagatcctcctaccacttattactccatcatcggtggaggtctgagaatgaactttggattcaccaaatgtcctcagattaaatccatctcagaatccgc : 195398
Seq3 : tagatcctcctaccacttattactccatcatcggtggaggtctgagaatgaactttggattcaccaaatgtcctcagattaaatccatctcagaatccgc : 195399
Seq4 : tagatcctcctaccacttattactccatcatcggtggaggtctgagaatgaactttggattcaccaaatgtcctcagattaaatccatctcagaatccgc : 195398

                 *    195420          *    195440          *    195460          *    195480          *    195500
Seq1 : tgatggaaacacagtgaatgctagattgtccagcgtgtccccaggacaaggtaaggactctcccgcgatcactcatgaagaagctctggctatgatcaaa : 195500
Seq2 : tgatggaaacacagtgaatgctagattgtccagcgtgtccccaggacaaggtaaggactctcccgcgatcactcatgaagaagctctggctatgatcaaa : 195498
Seq3 : tgatggaaacacagtgaatgctagattgtccagcgtgtccccaggacaaggtaaggactctcccgcgatcactcatgaagaagctctggctatgatcaaa : 195499
Seq4 : tgatggaaacacagtgaatgctagattgtccagcgtgtccccaggacaaggtaaggactctcccgcgatcactcatgaagaagctctggctatgatcaaa : 195498

                 *    195520          *    195540          *    195560          *    195580          *    195600
Seq1 : gactgtgaggtgtctatcgacatcagatgtagcgaagaagagaaagacagcgacatcaagacccatccagtactcgggtctaacatctctcataagaaag : 195600
Seq2 : gactgtgaggtgtctatcgacatcagatgtagcgaagaagagaaagacagcgacatcaagacccatccagtactcgggtctaacatctctcataagaaag : 195598
Seq3 : gactgtgaggtgtctatcgacatcagatgtagcgaagaagagaaagacagcgacatcaagacccatccagtactcgggtctaacatctctcataagaaag : 195599
Seq4 : gactgtgaggtgtctatcgacatcagatgtagcgaagaagagaaagacagcgacatcaagacccatccagtactcgggtctaacatctctcataagaaag : 195598

                 *    195620          *    195640          *    195660          *    195680          *    195700
Seq1 : tgagttacgaagatatcatcggttcaacgatcgtcgatacaaaatgtgtcaagaatctagagtttagcgttcgtatcggagacatgtgcaaggaatcatc : 195700
Seq2 : tgagttacgaagatatcatcggttcaacgatcgtcgatacaaaatgtgtcaagaatctagagtttagcgttcgtatcggagacatgtgcaaggaatcatc : 195698
Seq3 : tgagttacgaagatatcatcggttcaacgatcgtcgatacaaaatgtgtcaagaatctagagtttagcgttcgtatcggagacatgtgcaaggaatcatc : 195699
Seq4 : tgagttacgaagatatcatcggttcaacgatcgtcgatacaaaatgtgtcaagaatctagagtttagcgttcgtatcggagacatgtgcaaggaatcatc : 195698

                 *    195720          *    195740          *    195760          *    195780          *    195800
Seq1 : tgaacttgaggtcaaggatggatttaagtatgtcgacggatcggcatctgaaggtgcaaccgatgatacttcactcatcgattcaacaaaactcaaagcg : 195800
Seq2 : tgaacttgaggtcaaggatggatttaagtatgtcgacggatcggcatctgaaggtgcaaccgatgatacttcactcatcgattcaacaaaactcaaagcg : 195798
Seq3 : tgaacttgaggtcaaggatggatttaagtatgtcgacggatcggcatctgaaggtgcaaccgatgatacttcactcatcgattcaacaaaactcaaagcg : 195799
Seq4 : tgaacttgaggtcaaggatggatttaagtatgtcgacggatcggcatctgaaggtgcaaccgatgatacttcactcatcgattcaacaaaactcaaagcg : 195798

                 *    195820          *    195840          *    195860          *    195880          *    195900
Seq1 : tgtgtctgaatcgataactctattcatctgaaattggatgagtagggttaatcgaacgattcaggcacaccacgaattaaaaaagtgtaccggacactat : 195900
Seq2 : tgtgtctgaatcgataactctattcatctgaaattggatgagtagggttaatcgaacgattcaggcacaccacgaattaaaaaagtgtaccggacactat : 195898
Seq3 : tgtgtctgaatcgataactctattcatctgaaattggatgagtagggttaatcgaacgattcaggcacaccacgaattaaaaaagtgtaccggacactat : 195899
Seq4 : tgtgtctgaatcgataactctattcatctgaaattggatgagtagggttaatcgaacgattcaggcacaccacgaattaaaaaagtgtaccggacactat : 195898

                 *    195920          *    195940          *    195960          *    195980          *    196000
Seq1 : attccggtttgcaaaacaaaaatgttcttaactacattcacaaaaagttacctctcgcgacttcttctttttctgtctcaatagtgtgatacgattatga : 196000
Seq2 : attccggtttgcaaaacaaaaatgttcttaactacattcacaaaaagttacctctcgcgacttcttctttttctgtctcaatagtgtgatacgattatga : 195998
Seq3 : attccggtttgcaaaacaaaaatgttcttaactacattcacaaaaagttacctctcgcgacttcttctttttctgtctcaatagtgtgatacgattatga : 195999
Seq4 : attccggtttgcaaaacaaaaatgttcttaactacattcacaaaaagttacctctcgcgacttcttctttttctgtctcaatagtgtgatacgattatga : 195998
```

```
          *    196020          *    196040          *    196060          *    196080          *    196100
Seq1 : cactattcctattcctattcctatttcctttcagggtatcacaaaaatattaaacctctttctgatggtctcataaaaaaagttttacaaaaatattttt : 196100
Seq2 : cactattcctattcctattcctatttcctttcagggtatcacaaaaatattaaacctctttctgatggtctcataaaaaaagttttacaaaaatattttt : 196098
Seq3 : cactattcctattcctattcctatttcctttcagggtatcacaaaaatattaaacctctttctgatggtctcataaaaaaagttttacaaaaatattttt : 196099
Seq4 : cactattcctattcctattcctatttcctttcagggtatcacaaaaatattaaacctctttctgatggtctcataaaaaaagttttacaaaaatattttt : 196098

          *    196120          *    196140          *    196160          *    196180          *    196200
Seq1 : attctctttctctctttgatggtctcataaaaaaagttttacaaaaatatttttattctctttctctctttgatggtctcataaaaaaagttttacaaaa : 196200
Seq2 : attctctttctctctttgatggtctcataaaaaaagttttacaaaaatatttttattctctttctctctttgatggtctcataaaaaaagttttacaaaa : 196198
Seq3 : attctctttctctctttgatggtctcataaaaaaagttttacaaaaatatttttattctctttctctctttgatggtctcataaaaaaagttttacaaaa : 196199
Seq4 : attctctttctctctttgatggtctcataaaaaaagttttacaaaaatatttttattctctttctctctttgatggtctcataaaaaaagttttacaaaa : 196198

          *    196220          *    196240          *    196260          *    196280          *    196300
Seq1 : atatttttattctctttctctctttgatggtctcataaaaaaagttttacaaaaatatttttattctctttctctctttgatggtctcataaaaaaagtt : 196300
Seq2 : atatttttattctctttctctctttgatggtctcataaaaaaagttttacaaaaatatttttattctctttctctctttgatggtctcataaaaaaagtt : 196298
Seq3 : atatttttattctctttctctctttgatggtctcataaaaaaagttttacaaaaatatttttattctctttctctctttgatggtctcataaaaaaagtt : 196299
Seq4 : atatttttattctctttctctctttgatggtctcataaaaaaagttttacaaaaatatttttattctctttctctctttgatggtctcataaaaaaagtt : 196298

          *    196320          *    196340          *    196360          *    196380          *    196400
Seq1 : ttacaaaaatatttttattctctttctctctttgatggtctcataaaaaaagttttacaaaaatatttttattctctttctctctttgatggtctcataa : 196400
Seq2 : ttacaaaaatatttttattctctttctctctttgatggtctcataaaaaaagttttacaaaaatatttttattctctttctctctttgatggtctcataa : 196398
Seq3 : ttacaaaaatatttttattctctttctctctttgatggtctcataaaaaaagttttacaaaaatatttttattctctttctctctttgatggtctcataa : 196399
Seq4 : ttacaaaaatatttttattctctttctctctttgatggtctcataaaaaaagttttacaaaaatatttttattctctttctctctttgatggtctcataa : 196398

          *    196420          *    196440          *    196460          *    196480          *    196500
Seq1 : aaaaagttttacaaaaatatttttattctctttctctctttgatggtctcataaaaaaagttttacaaaaatatttttattctctttctctctttgatgg : 196500
Seq2 : aaaaagttttacaaaaatatttttattctctttctctctttgatggtctcataaaaaaagttttacaaaaatatttttattctctttctctctttgatgg : 196498
Seq3 : aaaaagttttacaaaaatatttttattctctttctctctttgatggtctcataaaaaaagttttacaaaaatatttttattctctttctctctttgatgg : 196499
Seq4 : aaaaagttttacaaaaatatttttattctctttctctctttgatggtctcataaaaaaagttttacaaaaatatttttattctctttctctctttgatgg : 196498

          *    196520          *    196540          *    196560          *    196580          *    196600
Seq1 : tctcataaaaaatattaaacctctttctgatggtgtcactaaaatatttttattctcattctcattttctctttctctcttcaatggagtcataaaatat : 196600
Seq2 : tctcataaaaaatattaaacctctttctgatggtgtcactaaaatatttttattctcattctcattttctctttctctcttcaatggagtcataaaatat : 196598
Seq3 : tctcataaaaaatattaaacctctttctgatggtgtcactaaaatatttttattctcattctcattttctctttctctcttcaatggagtcataaaatat : 196599
Seq4 : tctcataaaaaatattaaacctctttctgatggtgtcactaaaatatttttattctcattctcattttctctttctctcttcaatggagtcataaaatat : 196598

          *    196620          *    196640          *    196660          *    196680          *    196700
Seq1 : ttttattctctttctctcttcgatggtctcacaaaaatattaaacctctttctgatggtgtcactaaaatatttttattctcattctcattttctctttc : 196700
Seq2 : ttttattctctttctctcttcgatggtctcacaaaaatattaaacctctttctgatggtgtcactaaaatatttttattctcattctcattttctctttc : 196698
Seq3 : ttttattctctttctctcttcgatggtctcacaaaaatattaaacctctttctgatggtgtcactaaaatatttttattctcattctcattttctctttc : 196699
Seq4 : ttttattctctttctctcttcgatggtctcacaaaaatattaaacctctttctgatggtgtcactaaaatatttttattctcattctcattttctctttc : 196698
```

```
                 *    196720         *    196740         *    196760         *    196780         *    196800
Seq1 : tctcttcaatggagtcataaaatatttttattctctttctctcttcgatggtctcacaaaaatattaaacctctttctgatggagtcgtaaaaaagtttt : 196800
Seq2 : tctcttcaatggagtcataaaatatttttattctctttctctcttcgatggtctcacaaaaatattaaacctctttctgatggagtcgtaaaaaagtttt : 196798
Seq3 : tctcttcaatggagtcataaaatatttttattctctttctctcttcgatggtctcacaaaaatattaaacctctttctgatggagtcgtaaaaaagtttt : 196799
Seq4 : tctcttcaatggagtcataaaatatttttattctctttctctcttcgatggtctcacaaaaatattaaacctctttctgatggagtcgtaaaaaagtttt : 196798

                 *    196820         *    196840         *    196860         *    196880         *    196900
Seq1 : atctctttctctcttcgatggtctcactaaaatattaaacctctttctgatggagtcgtaaaaaagttttatctctttctctcttcgatggtctcacaaa : 196900
Seq2 : atctctttctctcttcgatggtctcactaaaatattaaacctctttctgatggagtcgtaaaaaagttttatctctttctctcttcgatggtctcacaaa : 196898
Seq3 : atctctttctctcttcgatggtctcacaaaaatattaaacctctttctgatggagtcgtaaaaaagttttatctctttctctcttcgatggtctcacaaa : 196899
Seq4 : atctctttctctcttcgatggtctcacaaaaatattaaacctctttctgatggagtcgtaaaaaagttttatctctttctctcttcgatggtctcacaaa : 196898

                 *    196920         *    196940         *    196960         *    196980         *    197000
Seq1 : aatattaaacctctttctgatggagtcgtaaaaaagttttatctctttctctcttcgatggtctcacaaaaatattaaacctctttctgatggagtcgta : 197000
Seq2 : aatattaaacctctttctgatggagtcgtaaaaaagttttatctctttctctcttcgatggtctcacaaaaatattaaacctctttctgatggagtcgta : 196998
Seq3 : aatattaaacctctttctgatggagtcgtaaaaaagttttatctctttctctcttcgatggtctcacaaaaatattaaacctctttctgatggagtcgta : 196999
Seq4 : aatattaaacctctttctgatggagtcgtaaaaaagttttatctctttctctcttcgatggtctcacaaaaatattaaacctctttctgatggagtcgta : 196998

                 *    197020         *    197040         *    197060         *    197080         *    197100
Seq1 : aaaaagttttatctctttctctcttcgatggtctcacaaaaatattaaacctctttctgatggagtcgtaaaaaagttttatctctttctctcttcgatg : 197100
Seq2 : aaaaagttttatctctttctctcttcgatggtctcacaaaaatattaaacctctttctgatggagtcgtaaaaaagttttatctctttctctcttcgatg : 197098
Seq3 : aaaaagttttatctctttctctcttcgatggtctcacaaaaatattaaacctctttctgatggagtcgtaaaaaagttttatctctttctctcttcgatg : 197099
Seq4 : aaaaagttttatctctttctctcttcgatggtctcacaaaaatattaaacctctttctgatggagtcgtaaaaaagttttatctctttctctcttcgatg : 197098

                 *    197120         *    197140         *    197160         *    197180         *    197200
Seq1 : gtctcacaaaaatattaaacctctttctgatggagtcgtaaaaaagttttatctctttctctcttcgatggtctcacaaaaatattaaacctctttctga : 197200
Seq2 : gtctcacaaaaatattaaacctctttctgatggagtcgtaaaaaagttttatctctttctctcttcgatggtctcacaaaaatattaaacctctttctga : 197198
Seq3 : gtctcacaaaaatattaaacctctttctgatggagtcgtaaaaaagttttatctctttctctcttcgatggtctcacaaaaatattaaacctctttctga : 197199
Seq4 : gtctcacaaaaatattaaacctctttctgatggagtcgtaaaaaagttttatctctttctctcttcgatggtctcacaaaaatattaaacctctttctga : 197198

                 *    197220         *    197240         *    197260         *    197280         *    197300
Seq1 : tggagtcgtaaaaaagttttatctctttctctcttcgatggtctcacaaaaatattaaacctctttctgatggagtcgtaaaaaagttttatctctttct : 197300
Seq2 : tggagtcgtaaaaaagttttatctctttctctcttcgatggtctcacaaaaatattaaacctctttctgatggagtcgtaaaaaagttttatctctttct : 197298
Seq3 : tggagtcgtaaaaaagttttatctctttctctcttcgatggtctcacaaaaatattaaacctctttctgatggagtcgtaaaaaagttttatctctttct : 197299
Seq4 : tggagtcgtaaaaaagttttatctctttctctcttcgatggtctcacaaaaatattaaacctctttctgatggagtcgtaaaaaagttttatctctttct : 197298

                 *    197320         *    197340         *    197360         *    197380         *    197400
Seq1 : ctcttcgatggtctcacaaaaatattaaacctctttctgatggagtcgtaaaaaagttttatctctttctctcttcgatggtctcacaaaaatattaaac : 197400
Seq2 : ctcttcgatggtctcacaaaaatattaaacctctttctgatggagtcgtaaaaaagttttatctctttctctcttcgatggtctcacaaaaatattaaac : 197398
Seq3 : ctcttcgatggtctcacaaaaatattaaacctctttctgatggagtcgtaaaaaagttttatctctttctctcttcgatggtctcacaaaaatattaaac : 197399
Seq4 : ctcttcgatggtctcacaaaaatattaaacctctttctgatggagtcgtaaaaaagttttatctctttctctcttcgatggtctcacaaaaatattaaac : 197398
```

```
             *    197420          *    197440          *    197460          *    197480          *    197500
Seq1 : ctctttctgatggagtcgtaaaaaagttttatctctttctctcttcgatggtctcacaaaaatattaaacctctttctgatggagtcgtaaaaaagtttt : 197500
Seq2 : ctctttctgatggagtcgtaaaaaagttttatctctttctctcttcgatggtctcacaaaaatattaaacctctttctgatggagtcgtaaaaaagtttt : 197498
Seq3 : ctctttctgatggagtcgtaaaaaagttttatctctttctctcttcgatggtctcacaaaaatattaaacctctttctgatggagtcgtaaaaaagtttt : 197499
Seq4 : ctctttctgatggagtcgtaaaaaagttttatctctttctctcttcgatggtctcacaaaaatattaaacctctttctgatggagtcgtaaaaaagtttt : 197498

             *    197520          *    197540          *    197560          *    197580          *    197600
Seq1 : atctctttctctcttcgatggtctcacaaaaatattaaacctctttctgatggagtcgtaaaaaagttttatctctttctctcttcgatggtctcacaaa : 197600
Seq2 : atctctttctctcttcgatggtctcacaaaaatattaaacctctttctgatggagtcgtaaaaaagttttatctctttctctcttcgatggtctcacaaa : 197598
Seq3 : atctctttctctcttcgatggtctcacaaaaatattaaacctctttctgatggagtcgtaaaaaagttttatctctttctctcttcgatggtctcacaaa : 197599
Seq4 : atctctttctctcttcgatggtctcacaaaaatattaaacctctttctgatggagtcgtaaaaaagttttatctctttctctcttcgatggtctcacaaa : 197598

             *    197620          *    197640          *    197660          *    197680          *    197700
Seq1 : aatattaaacctctttctgatggagtcgtaaaaaagttttatctctttctctcttcgatggtctcacaaaaatattaaacctctttctgatggagtcgta : 197700
Seq2 : aatattaaacctctttctgatggagtcgtaaaaaagttttatctctttctctcttcgatggtctcacaaaaatattaaacctctttctgatggagtcgta : 197698
Seq3 : aatattaaacctctttctgatggagtcgtaaaaaagttttatctctttctctcttcgatggtctcacaaaaatattaaacctctttctgatggagtcgta : 197699
Seq4 : aatattaaacctctttctgatggagtcgtaaaaaagttttatctctttctctcttcgatggtctcacaaaaatattaaacctctttctgatggagtcgta : 197698

             *    197720          *    197740          *    197760          *    197780          *    197800
Seq1 : aaaaagttttatctctttctctcttcgatggtctcacaaaaatattaaacctctttctgatggagtcgtaaaaaagttttatctctttctctcttcgatg : 197800
Seq2 : aaaaagttttatctctttctctcttcgatggtctcacaaaaatattaaacctctttctgatggagtcgtaaaaaagttttatctctttctctcttcgatg : 197798
Seq3 : aaaaagttttatctctttctctcttcgatggtctcacaaaaatattaaacctctttctgatggagtcgtaaaaaagttttatctctttctctcttcgatg : 197799
Seq4 : aaaaagttttatctctttctctcttcgatggtctcacaaaaatattaaacctctttctgatggagtcgtaaaaaagttttatctctttctctcttcgatg : 197798

             *    197820          *    197840          *    197860          *    197880          *    197900
Seq1 : gtctcacaaaaatattaaacctctttctgatggagtcgtaaaaaagttttatctctttctctcttcgatggtctcacaaaaatattaaacctctttctga : 197900
Seq2 : gtctcacaaaaatattaaacctctttctgatggagtcgtaaaaaagttttatctctttctctcttcgatggtctcacaaaaatattaaacctctttctga : 197898
Seq3 : gtctcacaaaaatattaaacctctttctgatggagtcgtaaaaaagttttatctctttctctcttcgatggtctcacaaaaatattaaacctctttctga : 197899
Seq4 : gtctcacaaaaatattaaacctctttctgatggagtcgtaaaaaagttttatctctttctctcttcgatggtctcacaaaaatattaaacctctttctga : 197898

             *    197920          *    197940          *    197960          *    197980          *    198000
Seq1 : tggagtcgtaaaaaagttttatctctttctctcttcgatggtctcacaaaaatattaaacctctttctgatggagtcgtaaaaaagttttatctctttct : 198000
Seq2 : tggagtcgtaaaaaagttttatctctttctctcttcgatggtctcacaaaaatattaaacctctttctgatggagtcgtaaaaaagttttatctctttct : 197998
Seq3 : tggagtcgtaaaaaagttttatctctttctctcttcgatggtctcacaaaaatattaaacctctttctgatggagtcgtaaaaaagttttatctctttct : 197999
Seq4 : tggagtcgtaaaaaagttttatctctttctctcttcgatggtctcacaaaaatattaaacctctttctgatggagtcgtaaaaaagttttatctctttct : 197998

             *    198020          *    198040          *    198060          *    198080          *    198100
Seq1 : ctcttcgatggtctcactaaaatattttttattctctttctgatgcatcaactatttcttaaacaataacgtccaacaacatatactcgtcgagcttatc : 198100
Seq2 : ctcttcgatggtctcactaaaatattttttattctctttctgatgcatcaactatttcttaaacaataacgtccaacaacatatactcgtcgagcttatc : 198098
Seq3 : ctcttcgatggtctcactaaaatattttttattctctttctgatgcatcaactatttcttaaacaataacgtccaacaacatatactcgtcgagcttatc : 198099
Seq4 : ctcttcgatggtctcactaaaatattttttattctctttctgatgcatcaactatttcttaaacaataacgtccaacaacatatactcgtcgagcttatc : 198098
```

```
                 *    198120          *    198140          *    198160          *    198180          *    198200
Seq1 : aacatcccctatgcccatctaggttaccagacaattgtatatcataaaataatgtttataatttacacgttaaaatcatataataaaacgtagatcgtat : 198200
Seq2 : aacatcccctatgcccatctaggttaccagacaattgtatatcataaaataatgtttataatttacacgttaaaatcatataataaaacgtagatcgtat : 198198
Seq3 : aacatcccctatgcccatctaggttaccagacaattgtatatcataaaataatgtttataatttacacgttaaaatcatataataaaacgtagatcgtat : 198199
Seq4 : aacatcccctatgcccatctaggttaccagacaattgtatatcataaaataatgtttataatttacacgttaaaatcatataataaaacgtagatcgtat : 198198

                 *    198220          *    198240          *    198260          *    198280          *    198300
Seq1 : aatattttttggtatataaatgatctagtaaaatccatgtaggggatactgctcacattttttctttggtacaaaatttcacacaagtttttatacagac : 198300
Seq2 : aatattttttggtatataaatgatctagtaaaatccatgtaggggatactgctcacattttttctttggtacaaaatttcacacaagtttttatacagac : 198298
Seq3 : aatattttttggtatataaatgatctagtaaaatccatgtaggggatactgctcacattttttctttggtacaaaatttcacacaagtttttatacagac : 198299
Seq4 : aatattttttggtatataaatgatctagtaaaatccatgtaggggatactgctcacattttttctttggtacaaaatttcacacaagtttttatacagac : 198298

                 *    198320          *    198340          *    198360          *    198380          *    198400
Seq1 : aaattcttgtccatatattttaaaacattgacttttgtactaagaaaaatatctagactaactatctctttctctttctctcttcgatggtctcacaaaa : 198400
Seq2 : aaattcttgtccatatattttaaaacattgacttttgtactaagaaaaatatctagactaactatctctttctctttctctcttcgatggtctcacaaaa : 198398
Seq3 : aaattcttgtccatatattttaaaacattgacttttgtactaagaaaaatatctagactaactatctctttctctttctctcttcgatggtctcacaaaa : 198399
Seq4 : aaattcttgtccatatattttaaaacattgacttttgtactaagaaaaatatctagactaactatctctttctctttctctcttcgatggtctcacaaaa : 198398

                 *    198420          *    198440          *    198460          *    198480          *    198500
Seq1 : atattaaacctctttctgatggagtcgtaaaaaagttttatctctttctctcttcgatggtctcacaaaaatattaaacctctttctgatggagtcgtaa : 198500
Seq2 : atattaaacctctttctgatggagtcgtaaaaaagttttatctctttctctcttcgatggtctcacaaaaatattaaacctctttctgatggagtcgtaa : 198498
Seq3 : atattaaacctctttctgatggagtcgtaaaaaagttttatctctttctctcttcgatggtctcacaaaaatattaaacctctttctgatggagtcgtaa : 198499
Seq4 : atattaaacctctttctgatggagtcgtaaaaaagttttatctctttctctcttcgatggtctcacaaaaatattaaacctctttctgatggagtcgtaa : 198498

                 *    198520          *    198540          *    198560          *    198580          *    198600
Seq1 : aaaagttttatctctttctctcttcgatggtctcacaaaaatattaaacctctttctgatggagtcgtaaaaaagttttatctctttctctcttcgatgg : 198600
Seq2 : aaaagttttatctctttctctcttcgatggtctcacaaaaatattaaacctctttctgatggagtcgtaaaaaagttttatctctttctctcttcgatgg : 198598
Seq3 : aaaagttttatctctttctctcttcgatggtctcacaaaaatattaaacctctttctgatggagtcgtaaaaaagttttatctctttctctcttcgatgg : 198599
Seq4 : aaaagttttatctctttctctcttcgatggtctcacaaaaatattaaacctctttctgatggagtcgtaaaaaagttttatctctttctctcttcgatgg : 198598

                 *    198620          *    198640          *    198660          *    198680          *    198700
Seq1 : tctcacaaaaatattaaacctctttctgatggagtcgtaaaaaagttttatctctttctctcttcgatggtctcacaaaaatattaaacctctttctgat : 198700
Seq2 : tctcacaaaaatattaaacctctttctgatggagtcgtaaaaaagttttatctctttctctcttcgatggtctcacaaaaatattaaacctctttctgat : 198698
Seq3 : tctcacaaaaatattaaacctctttctgatggagtcgtaaaaaagttttatctctttctctcttcgatggtctcacaaaaatattaaacctctttctgat : 198699
Seq4 : tctcacaaaaatattaaacctctttctgatggagtcgtaaaaaagttttatctctttctctcttcgatggtctcacaaaaatattaaacctctttctgat : 198698

                 *    198720          *    198740          *    198760          *    198780          *    198800
Seq1 : ggagtcgtaaaaaagttttatctctttctctcttcgatggtctcacaaaaatattaaacctctttctgatggagtcgtaaaaaagttttatctctttctc : 198800
Seq2 : ggagtcgtaaaaaagttttatctctttctctcttcgatggtctcacaaaaatattaaacctctttctgatggagtcgtaaaaaagttttatctctttctc : 198798
Seq3 : ggagtcgtaaaaaagttttatctctttctctcttcgatggtctcacaaaaatattaaacctctttctgatggagtcgtaaaaaagttttatctctttctc : 198799
Seq4 : ggagtcgtaaaaaagttttatctctttctctcttcgatggtctcacaaaaatattaaacctctttctgatggagtcgtaaaaaagttttatctctttctc : 198798
```

```
             *    198820          *    198840          *    198860          *    198880          *    198900
Seq1 : cttcgatggtctcacaaaaatattaaacctctttctgatggagtcgtaaaaaagttttatctctttctctcttcgatggtctcacaaaaatattaaacct : 198900
Seq2 : cttcgatggtctcacaaaaatattaaacctctttctgatggagtcgtaaaaaagttttatctctttctctcttcgatggtctcacaaaaatattaaacct : 198898
Seq3 : cttcgatggtctcacaaaaatattaaacctctttctgatggagtcgtaaaaaagttttatctctttctctcttcgatggtctcacaaaaatattaaacct : 198899
Seq4 : cttcgatggtctcacaaaaatattaaacctctttctgatggagtcgtaaaaaagttttatctctttctctcttcgatggtctcacaaaaatattaaacct : 198898

             *    198920          *    198940          *    198960          *    198980          *    199000
Seq1 : ctttctgatggagtcgtaaaaaagttttatctctttctctcttcgatggtctcacaaaaatattaaacctctttctgatggagtcgtaaaaaagttttat : 199000
Seq2 : ctttctgatggagtcgtaaaaaagttttatctctttctctcttcgatggtctcacaaaaatattaaacctctttctgatggagtcgtaaaaaagttttat : 198998
Seq3 : ctttctgatggagtcgtaaaaaagttttatctctttctctcttcgatggtctcacaaaaatattaaacctctttctgatggagtcgtaaaaaagttttat : 198999
Seq4 : ctttctgatggagtcgtaaaaaagttttatctctttctctcttcgatggtctcacaaaaatattaaacctctttctgatggagtcgtaaaaaagttttat : 198998

             *    199020          *    199040          *    199060          *    199080          *    199100
Seq1 : ctctttctccttcgatggtctcacaaaaatattaaacctctttctgatggagtcgtaaaaaagttttatctctttctccttcgatggtctcacaaaaata : 199100
Seq2 : ctctttctccttcgatggtctcacaaaaatattaaacctctttctgatggagtcgtaaaaaagttttatctctttctccttcgatggtctcacaaaaata : 199098
Seq3 : ctctttctccttcgatggtctcacaaaaatattaaacctctttctgatggagtcgtaaaaaagttttatctctttctccttcgatggtctcacaaaaata : 199099
Seq4 : ctctttctccttcgatggtctcacaaaaatattaaacctctttctgatggagtcgtaaaaaagttttatctctttctccttcgatggtctcacaaaaata : 199098

             *    199120          *    199140          *    199160          *    199180          *    199200
Seq1 : ttaaacctctttctgatggagtcgtaaaaaagttttatctctttctctcttcgatggtctcacaaaaatattaaacctctttctgatggagtcgtaaaaa : 199200
Seq2 : ttaaacctctttctgatggagtcgtaaaaaagttttatctctttctctcttcgatggtctcacaaaaatattaaacctctttctgatggagtcgtaaaaa : 199198
Seq3 : ttaaacctctttctgatggagtcgtaaaaaagttttatctctttctctcttcgatggtctcacaaaaatattaaacctctttctgatggagtcgtaaaaa : 199199
Seq4 : ttaaacctctttctgatggagtcgtaaaaaagttttatctctttctctcttcgatggtctcacaaaaatattaaacctctttctgatggagtcgtaaaaa : 199198

             *    199220          *    199240          *    199260          *    199280          *    199300
Seq1 : agttttatctctttctctcttcgatggtctcacaaaaatattaaacctctttctgatggtctctataaagcgatcgatctttcttacactctagagtttc : 199300
Seq2 : agttttatctctttctctcttcgatggtctcacaaaaatattaaacctctttctgatggtctctataaagcgatcgatctttcttacactctagagtttc : 199298
Seq3 : agttttatctctttctctcttcgatggtctcacaaaaatattaaacctctttctgatggtctctataaagcgatcgatctttcttacactctagagtttc : 199299
Seq4 : agttttatctctttctctcttcgatggtctcacaaaaatattaaacctctttctgatggtctctataaagcgatcgatctttcttacactctagagtttc : 199298

             *    199320          *    199340          *
Seq1 : ctacagtcatgggtcacacatttttttctagacactaaataaaattagtaaaat : 199354
Seq2 : ctacagtcatgggtcacacatttttttctagacactaaataaaattagtaaaat : 199352
Seq3 : ctacagtcatgggtcacacatttttttctagacactaaataaaattagtaaaat : 199353
Seq4 : ctacagtcatgggtcacacatttttttctagacactaaataaaattagtaaaat : 199352
```
